# Levels and trends in fertility rates among adolescents aged 10–14 and 15–19 in 201 countries and areas from 1950 to 2023

**DOI:** 10.1136/bmjgh-2025-021301

**Published:** 2026-07-29

**Authors:** Fengqing Chao, Vladimíra Kantorová, Giulia Gonnella, Bruno Schoumaker, David A Sánchez-Páez, Patrick Gerland

**Affiliations:** 1 Computational Social Science Division, School of Humanities and Social Science, The Chinese University of Hong Kong, Shenzhen, Shenzhen, Guangdong, China; 2 Department of Economic and Social Affairs, United Nations Population Division, New York, New York, USA; 3 Centre for Demographic Research, Iacchos Institute, UCLouvain, Louvain-la-Neuve, Belgium; 4 Department of Economic Theory, Universidad de Valladolid, Valladolid, Spain

**Keywords:** Mathematical modelling, Gender-Based Violence, Global Health, Child health, Medical demography

## Abstract

**Introduction:**

Reducing adolescent childbearing is essential for improving sexual and reproductive health and the social and economic well-being of adolescents. Adolescent birth rate is an indicator to monitor progress towards Sustainable Development Goal (SDG) Target 3.7 by 2030, ensuring universal access to sexual and reproductive healthcare services. However, the availability and reliability of data on adolescent fertility rates are low.

**Methods:**

We produce annual estimates of age-specific fertility rates (ASFR) for girls aged 10–14 and 15–19 from 1950 to 2023 for the world, regions and 201 countries and territories. Based on a comprehensive ASFR database, we estimate the under-20 ASFR with a Bayesian hierarchical time series model, using covariates such as total fertility rates and female educational attainment and adjusting for biases and under-reporting. We calculate the number of births to adolescents by applying rates to the female population in the respective age groups.

**Results:**

Between 1990 and 2023, global ASFR for girls aged 10–14 decreased by 76%, from 4.5 (95% uncertainty interval (UI) (4.1 to 5.2)) to 1.1 (95% UI 0.9 to 1.4) births per 1000, while ASFR for girls aged 15–19 fell by 48%, from 74.7 (95% UI 72.5 to 77.1) to 39.0 (95% UI 36.8 to 41.4) births per 1000. In 2023, births to mothers aged 10–14 years were estimated at 347 (95% UI 285 to 454) thousand, and for those aged 15–19 years at 12.2 (95% UI 11.5 to 12.9) million. The number of births among adolescents aged 15–19 in sub-Saharan Africa increased during 1990–2023 due to population growth and slower fertility declines.

**Conclusion:**

Despite significant global reduction in adolescent childbearing, regional and national disparities persist. Targeted interventions addressing child marriage, educational inequities and lack of access to reproductive health services will be critical for achieving related SDG targets and advancing gender equality and women’s empowerment.

**WHAT IS ALREADY KNOWN ON THIS TOPIC**

- Adolescent childbearing has declined, but girls in some regions are still at heightened risk.
- Most of the global evidence is based on data for age group 15–19. Previous global estimates of adolescent birth rates for ages 10–14 relied on indirect modelling processes and were based on limited data sources, lacking direct birth history observations from surveys.

**WHAT THIS STUDY ADDS**

- We provided estimates of the age-specific fertility rates (ASFR) and births by mothers aged 10–14 and 15–19 for all countries from 1950 to 2023.
- Our estimates were based on Bayesian hierarchical time series models that were fitted to an extensive database of national ASFRs up to year 2023 compiled for this study, including estimates for age group 10–14 from birth histories from surveys. The estimates with uncertainty assessments that take into account the data availability and quality provide insights into disparities in the ASFRs as well as trends over time.

**HOW THIS STUDY MIGHT AFFECT RESEARCH, PRACTICE OR POLICY**

- Global adolescent fertility rates have declined significantly since 1990, yet regional disparities persist; in sub-Saharan Africa, after relatively slow declines compared with other regions, fertility among girls aged 10–14 and 15–19 remains high.
- The findings underscore the urgent need for targeted interventions addressing child marriage, lack of access to sexual and reproductive healthcare services and information, educational barriers and socio-economic inequalities.
- Strengthened data collection systems are essential to improve monitoring and ensure progress toward Sustainable Development Goal Target 3.7. The evidence provided by this study supports policymaking and programmatic efforts to reduce adolescent fertility and promote gender equality.

## Introduction

 Early childbearing leads to significant adverse consequences for the health and well-being of both the young mothers and their children. Pregnancy complications and unsafe abortions are among the leading causes of death among girls aged 10–19 years. Adolescent mothers face higher risks of eclampsia, puerperal endometritis and systemic infections compared with women aged 20–24 years, and babies of adolescent mothers face higher risks of low birth weight, preterm birth and severe neonatal conditions.^1 2^ From a socio-economic standpoint, early childbearing often results in girls abandoning education and later in life having fewer opportunities for decent work, experiencing lower lifetime earnings and facing increased risks of intergenerational poverty.^3–6^ Reducing adolescent childbearing is therefore crucial to reducing maternal mortality and morbidity, relieving public health burden, ensuring that all women and girls are empowered to achieve their life goals, reducing poverty and contributing to economic development at a societal level. In this context, Target 3.7 of the 2030 Agenda for Sustainable Development aims to ‘ensure universal access to sexual and reproductive health-care services, including for family planning, information and education’ by 2030. To monitor progress towards this target, Sustainable Development Goal (SDG) indicator 3.7.2 measures the ‘Adolescent birth rate’ among girls and young women aged 10–14 years and 15–19 years, respectively.^7 8^ The adolescent birth rate for ages 15–19 was previously used to track progress towards the Millennium Development Goals between 2005 and 2015.

Monitoring trends and understanding the underlying factors of adolescent childbearing are essential to safeguarding girls’ human rights, improving access to sexual and reproductive healthcare services and achieving gender equality and women’s empowerment. However, monitoring is challenging due to the scarcity of high-quality data in many countries. While the contribution of childbearing below age 15 to the overall fertility rates and the total number of births is minimal, it is a particularly grave violation of the human rights of girls, and therefore, it is crucial to have comprehensive and comparable estimates over time and across countries. Vital statistics from registration systems for births by age of mother are particularly deficient in countries where early childbearing is the most common, and the reliability of adolescent fertility rates is often compromised due to the under-reporting of births among the youngest mothers or the misreporting of their age.^9^ Additionally, while some data exist for births by age of mother below age 15 from countries with complete vital registration systems, these data have been neglected in the past fertility analyses that generally considered the reproductive age of women starting at age 15. The estimates of childbearing among girls below age 15 for countries without high-quality vital registration systems have only recently been established based on the analysis of birth histories from surveys.^10 11^

Previous estimates of childbearing among girls below age 15 have either relied on data in a limited number of countries,^12^ or used indirect methods for estimation with minimal use of newly available empirical data that may lead to unrealistic estimates of early adolescent fertility. The most recent global study on adolescent fertility that included the age group 10–14 was conducted by the Global Burden of Diseases Study (GBD) 2021 Fertility and Forecasting Collaborators.^13^ However, the GBD estimation did not consider temporal correlation and relied on a complicated six-step modelling process (see online supplemental appendix pp 7). Estimates for specific countries and limited periods give a partial picture of levels and trends of early adolescent fertility, which may vary widely across countries and over time. Meanwhile, the estimates based on multisteps of modelling may heavily rely on expert opinions of model set-ups and can override the levels and trends suggested by the empirical data.

This study provides annual estimates and uncertainty intervals (UIs) of adolescent birth rates for the 10–14 and 15–19 age groups from 1950 to 2023 for 201 countries and territories with a total population of at least 90 000 inhabitants in 2024, along with 22 subregional, seven regional and global estimates. We compiled an extensive and comprehensive database with data for age-specific fertility rates for 236 countries and territories, which is used in the overall model implementation and the calculation of estimates for global and regional aggregates. For the age group 10–14, we included all newly available observations calculated from birth histories from household surveys using the methods developed by Schoumaker and Sánchez-Páez.^11^ We developed a three-step Bayesian estimation process that integrates diverse data sources while accounting for potential biases. These data and methods enable a systematic analysis of fertility among girls and adolescents aged 10–14 and 15–19, filling gaps in missing data and reconciling discrepancies across data sources and estimation methods and provide an assessment of UIs. This approach has improved international comparability of trends in adolescent childbearing across all countries since 1950. The methods, the full database with the data on age-specific fertility rates from various sources, including metadata, and the results of this study have all been incorporated into the 2024 revision of the United Nations (UN) World Population Prospects (WPP),^14^ which includes global, regional and national estimates of ASFRs for the 10–14 and 15–19 age groups. These estimates served as inputs for the cohort-component method of the UN WPP population estimation since 1950 based on all components of demographic change by age and sex.

## Data and methods

We summarised here the data and methods used. A more detailed description of the model specifications, statistical computation and model validation is available in the online supplemental appendix (DOI: 10.6084 /m9.figshare.31524454).

### Database construction

The age-specific fertility rates (ASFR) database for this study was compiled by the UN Department of Economic and Social Affairs, Population Division, encompassing a total of 36 702 observations in 236 countries or areas exceeding 1000 inhabitants by 1 July 2024, where 15 685 are for ages 10–14 and 21 017 for ages 15–19, respectively. All available nationally representative data on fertility were systematically assessed for quality.^15^ The empirical databases and final estimates undergo biannual updates and are openly accessible to the public.

Data were sourced from vital registration systems, household surveys and population censuses (reference years: 1950–2023). We extracted the fertility rates for 10–14 and 15–19 age groups, with results reported for these groups. Detailed data sources by country and age group and the extraction process are documented in the online supplemental appendix pp 1–7, tables 17 and 18. The majority of the available data come from household surveys (49.7% of data points for age group 10–14 and 38% for age group 15–19), in particular the Demographic Health Surveys (67.1% of the total survey data for age group 10–14 and 58.7% for age group 15–19) and the Multiple Indicators Cluster Surveys (17.6% for age group 10–14 and 17.3% for age group 15–19), followed by the World Fertility Surveys and the Reproductive Health Surveys. The second primary data source is vital registration, with a total of 154 countries with registration data for age group 10–14 (32.8% of the available data for the age group) and 169 for age group 15–19 (36.7%).

Compared with all previous studies, we leveraged to the fullest extent the information on full birth histories from surveys to calculate birth rates among girls in the age group 10–14. The information from full birth histories, for which women of reproductive age (usually defined as 15–49 years) are asked to reconstruct the full list of live births they had, including information on the dates of birth, permits calculation of age-specific fertility rates for a series of successive 3-year or 5-year periods preceding each survey.^10 16^ This makes it possible to compute fertility rates in the age group 10–14 up to 35 years before each survey containing full birth histories (see online supplemental appendix pp 4 for further details). This source of information was particularly important for assessing fertility rates for ages 10–14, as it provided estimates for countries and periods that were not otherwise covered by vital registration or censuses.

### Estimating ASFR for age groups 10–14 and 15–19

A Bayesian estimation model integrated ASFR empirical data with national-level total fertility rate (TFR) estimates published by the UN WPP 2024 to derive robust fertility estimates.^14^ The TFR was estimated externally and independent of the ASFR estimation. ASFR was not part of the TFR estimation model. TFR is estimated on the more robust and comprehensive set of empirical data, considering the biases and uncertainty associated with empirical estimates from different types of data sources, direct and indirect estimation methods and other factors that contribute to systematic biases and non-sampling errors from different types of data sources.^17^ This approach contributes additional information especially for the periods with missing data on ASFRs, because some of the indirect methods for the calculation of TFR only provide an estimate of TFR and not ASFRs. The estimation models for the ASFR for age groups 10–14 and 15–19 are summarised below. Full model specifications, implementation, validation and sensitivity analyses details are presented in the online supplemental appendix (pp 7–21).

First, we apply country-specific regression functions to adjust data biases in age groups 10–14 and 15–19. Data source type, collection method and recall lag are covariates to estimate and predict biases for all observations. Second, we estimated the ASFR for the age group 15–19. We modelled the ASFR for the age group 15–19 in a specific country and a particular year on the logit scale. The sole purpose of modelling ASFR on the logit scale is to constrain the ASFR between 0 and 1. The logit-transformed ASFR from a specific country-year was modelled by incorporating country-specific effects of female educational attainment,^13^ regional TFR effects,^14^ country-specific temporal effects and country-specific offsets. Third, the ASFR for the 10–14 age group was derived by modelling the ratio of the logit-scaled ASFR for 10–14 to the logit-scaled ASFR for 15–19, accounting for correlations between these age groups. Supported by the empirical evidence of our extensive database (online supplemental appendix pp 9–10), this approach allowed the limited data available for the 10–14 age group to benefit from the greater availability for the 15–19 age group. Our sensitivity analyses show that having this model assumption greatly improved Bayesian model fitting and prediction power (online supplemental appendix pp 18–20). The uncertainties from ASFR observations, including sampling and non-sampling variances, were addressed within the Bayesian framework, with less informative observations appropriately downweighted. The posterior samples reflect the data uncertainty.

Model performance was validated using out-of-sample testing (online supplemental appendix pp 17–18). All computations were conducted using the R-package INLA^18 19^ and R,^20^ with analysis scripts available on request.

### Post-modelling processing

We used the inverse-logit transformation on the model estimates of the logit ASFR to obtain the original scale of ASFR results. These actual ASFR values can then be interpreted as number of births per year per 1000 women from a certain age group.

We adjusted the median estimates and the 95% UIs of ASFR for each country-year for age groups 10–14 and 15–19 (online supplemental appendix pp 16). We matched the sum of births across all age groups of mothers (based on the ASFR from all age groups) to the total number of births (based on the TFR). The TFR trajectories from the UN WPP 2024 revision were used to produce the final UIs of the estimated ASFRs for all country-years (online supplemental appendix pp 15–16).

### Calculating number of adolescent births and aggregates

The number of births by age of the mother is calculated by multiplying the age-specific fertility rates derived as described above by the number of women in the age group estimated in WPP. Aggregated ASFRs were derived by dividing the number of births in the age group by the population of women in this age group in a region.

## Results

We report ASFR for the age groups 10–14 and 15–19, with median estimates and 95% UIs for 201 countries or areas, 22 subregions, seven regions and globally for 1990, 2015 and 2023. Year 2015 was chosen to signify the adoption of the 2030 Agenda for Sustainable Development to understand whether the trends have changed before and after the SDGs. Year 2023 was the most recent year of the estimates published in the UN WPP 2024 revision. Annual results since 1950 are presented in the online supplemental appendix (tables 13–16 and pp 111–514).

### ASFR estimates on global, regional and national levels

Table 1 presents the fertility rates for the age groups 10–14 and 15–19 in 1990, 2015 and 2023 for the world and seven regions (refer to online supplemental appendix table 12 for the regional classification). In both age groups (table 1), global fertility rates declined significantly between 1990 and 2023. For girls aged 10–14, fertility fell by 76% from 4.5 (95% UI 4.1 to 5.2) births per 1000 girls in 1990 to 1.1 (95% UI 0.9 to 1.4) per 1000 in 2023, with a significant total decline of −3.4 (95% UI −4.1 to −2.9) per 1000. While for those aged 15–19, there was a 48% reduction from 74.7 (95% UI 72.5 to 77.1) births per 1000 girls and young women in 1990 to 39.0 (95% UI 36.8 to 41.4) per 1000 in 2023 with a significant total decline of −35.7 (95% UI −38.9 to −32.4) per 1000.

**Table 1:** Estimates and 95% uncertainty intervals for age-specific fertility rate per 1000 female population for age groups 10–14 and 15–19, for the world and regions, in 1990, 2015, and 2023, the total change and the annual rate of reduction for periods 1990–2015, 2015–2023 and 1990–2023.

Indicator	ASFR			Total change			Annual rate of reduction (in %)		
Year	1990	2015	2023	1990–2015	2015–2023	1990–2023	1990–2015	2015–2023	1990–2023
	ASFR 10–14 (births per 1000 girls aged 10–14)								
World	4.5 (4.1 to 5.2)	1.5 (1.3 to 1.6)	1.1 (0.9 to 1.4)	−3.0 (−3.8 to −2.6)*	−0.4 (−0.7 to −0.1)*	−3.4 (−4.2 to −2.9)*	4.46 (3.86 to 5.16)*	4.14 (0.65 to 7.04)*	4.39 (3.52 to 5.15)*
Sub-Saharan Africa	11.6 (10.8 to 12.5)	4.3 (3.8 to 4.9)	3.2 (2.5 to 4.2)	−7.3 (−8.3 to −6.4)*	−1.1 (−2.0 to 0.0)*	−8.4 (−9.6 to −7.2)*	3.98 (3.41 to 4.55)*	3.73 (0.09 to 6.96)*	3.92 (3.08 to 4.65)*
Latin America and the Caribbean	3.0 (2.5 to 3.9)	2.7 (2.3 to 3.3)	1.7 (1.3 to 2.4)	−0.3 (−1.3 to 0.5)	−1.0 (−1.7 to −0.2)*	−1.3 (−2.3 to −0.4)*	0.42 (−0.63 to 1.61)	5.88 (1.10 to 10.30)*	1.74 (0.52 to 2.94)*
Oceania (excluding Australia and New Zealand)	4.8 (3.0 to 7.6)	1.8 (1.0 to 3.6)	1.4 (0.5 to 4.4)	−3.0 (−6.0 to −0.5)*	−0.4 (−2.4 to 2.7)	−3.4 (−6.4 to 0.1)	3.90 (0.61 to 7.03)*	3.47 (−13.43 to 19.60)	3.79 (−0.07 to 7.50)
Northern Africa and Western Asia	2.6 (2.1 to 3.7)	0.9 (0.5 to 1.4)	0.6 (0.3 to 1.4)	−1.7 (−2.8 to −1.0)*	−0.3 (−0.9 to 0.6)	−2.0 (−3.1 to −1.1)*	4.35 (2.18 to 6.63)*	4.39 (−7.54 to 15.75)	4.36 (1.68 to 6.88)*
Central and Southern Asia	8.5 (7.2 to 10.1)	0.8 (0.6 to 1.1)	0.4 (0.2 to 1.1)	−7.7 (−9.4 to −6.4)*	−0.4 (−0.8 to 0.4)	−8.1 (−9.7 to −6.6)*	9.57 (8.11 to 10.93)*	9.12 (−4.85 to 19.42)	9.46 (6.18 to 11.77)*
Eastern and South-Eastern Asia	0.8 (0.6 to 2.9)	0.2 (0.1 to 0.5)	0.1 (0.1 to 0.4)	−0.6 (−2.6 to −0.3)*	−0.1 (−0.4 to 0.3)	−0.6 (−2.7 to −0.3)*	5.50 (1.85 to 10.81)*	5.04 (−11.37 to 18.34)	5.39 (1.60 to 9.63)*
Europe, Northern America, Australia and New Zealand	0.5 (0.4 to 0.7)	0.2 (0.2 to 0.3)	0.1 (0.1 to 0.2)	−0.3 (−0.5 to −0.2)*	−0.1 (−0.2 to 0.0)	−0.4 (−0.6 to −0.3)*	3.72 (2.36 to 4.99)*	6.26 (−0.95 to 12.33)	4.33 (2.52 to 5.82)*
	ASFR 15–19 (births per 1000 girls aged 15–19)								
World	74.7 (72.5 to 77.1)	45.9 (44.9 to 46.9)	39.0 (36.8 to 41.4)	−28.8 (−31.3 to −26.3)*	−6.9 (−9.3 to −4.3)*	−35.7 (−38.9 to −32.4)*	1.95 (1.80 to 2.10)*	2.04 (1.23 to 2.80)*	1.97 (1.77 to 2.17)*
Sub-Saharan Africa	139.5 (136.3 to 142.9)	110.5 (106.8 to 114.6)	94.4 (86.3 to 103.4)	−29.0 (−34.1 to −23.9)*	−16.1 (−25.1 to −6.3)*	−45.1 (−54.0 to −35.4)*	0.93 (0.76 to 1.10)*	1.97 (0.74 to 3.17)*	1.18 (0.90 to 1.47)*
Latin America and the Caribbean	89.1 (82.2 to 97.4)	67.2 (64.8 to 69.7)	51.2 (45.8 to 57.5)	−21.9 (−30.5 to −14.7)*	−16.0 (−21.8 to −9.4)*	−37.9 (−47.5 to −28.5)*	1.13 (0.78 to 1.51)*	3.40 (1.90 to 4.86)*	1.68 (1.24 to 2.11)*
Oceania (excluding Australia and New Zealand)	78.9 (70.7 to 89.0)	56.6 (50.2 to 64.0)	49.9 (35.1 to 72.0)	−22.3 (−34.2 to −11.3)*	−6.7 (−23.5 to 16.5)	−29.0 (−47.1 to −5.4)*	1.33 (0.68 to 1.99)*	1.57 (−3.27 to 6.25)	1.39 (0.22 to 2.54)*
Northern Africa and Western Asia	66.3 (63.8 to 68.9)	45.2 (41.1 to 49.9)	36.3 (30.7 to 43.0)	−21.1 (−26.0 to −15.7)*	−8.9 (−16.1 to −0.9)*	−30.0 (−36.2 to −22.7)*	1.53 (1.10 to 1.94)*	2.75 (0.27 to 5.12)*	1.82 (1.29 to 2.35)*
Central and Southern Asia	131.4 (123.1 to 140.3)	32.1 (30.5 to 33.8)	26.2 (22.6 to 30.5)	−99.3 (−108.4 to −90.9)*	−5.9 (−9.8 to −1.3)*	−105.2 (−114.6 to −95.9)*	5.64 (5.31 to 5.98)*	2.54 (0.50 to 4.46)*	4.89 (4.39 to 5.36)*
Eastern and South-Eastern Asia	30.5 (29.1 to 32.1)	21.7 (20.2 to 23.5)	14.3 (12.4 to 16.5)	−8.8 (−11.0 to −6.6)*	−7.4 (−10.0 to −4.7)*	−16.2 (−18.6 to −13.6)*	1.36 (1.00 to 1.72)*	5.22 (3.17 to 7.20)*	2.30 (1.84 to 2.74)*
Europe, Northern America, Australia and New Zealand	39.0 (37.9 to 40.1)	16.6 (16.1 to 17.2)	9.9 (9.0 to 10.9)	−22.4 (−23.6 to −21.2)*	−6.8 (−7.8 to −5.7)*	−29.1 (−30.6 to −27.7)*	3.41 (3.23 to 3.58)*	6.51 (5.29 to 7.72)*	4.16 (3.86 to 4.45)*

* Change is significantly different from zero. Total change: difference between values at period end and period start. Annual rate of reduction: ln(ASFR_t2_/ASFR_t1_)/(t1 – t2), where t1 and t2 refer to different years with t1 smaller than t2.

ASFR, age-specific fertility rate.

All regions experienced declines in adolescent birth rates, but progress was uneven. All seven regions were estimated to have significant reductions in fertility from 1990 to 2023 in both age groups, except for under-15 fertility in Oceania (excluding Australia and New Zealand). Sub-Saharan Africa achieved the largest regional absolute reduction among girls aged 10–14 with decreases of −8.4 (95% UI −9.6 to −7.2) births per 1000, and experienced the second largest regional absolute decline for age group 15–19 at −45.1 (95% UI −54.0 to −35.4) births per 1000, however making slow progress toward reducing the burden of early childbearing on the relative scale. The adolescent birth rates in Sub-Saharan Africa remained the highest globally for both age groups in 2023 (3.2 (95% UI 2.5 to 4.2) for ages 10–14 and 94.4 (95% UI 86.3 to 103.4) for ages 15–19). Central and Southern Asia achieved the most substantial relative declines, with fertility for girls aged 10–14 decreasing by 95% and for those aged 15–19 by 80%. In 1990, the region had the second-highest fertility rates globally, at 8.5 (95% UI 7.2 to 10.1) and 131.4 (95% UI 123.1 to 140.3) per 1000 girls aged 10–14 and 15–19, respectively. By 2023, rates had fallen to 0.4 (95% UI 0.2 to 1.1) and 26.2 (95% UI 22.6 to 30.5) per 1000.

Global declines in adolescent birth rates measured by the annual reduction rate (ARR; a positive ARR is equivalent to a decrease in the ASFR during the period) of 4.39% (95% UI 3.52% to 5.15%) and 1.97% (95% UI 1.77% to 2.17%) for age groups 10–14 and 15–19, respectively, indicate that fertility declined more than two times as fast for the younger girls than the older adolescents. While ARRs were similar for both periods 1990–2015 and 2015–2023 at the global level, the declines differed by region and country for each period (online supplemental appendix tables 13–14 for subregional and national ARRs). The ARRs for age group 10–14 were significant in all subregions of sub-Saharan Africa in 1990–2015. For age group 15–19, most subregions with ARR significantly different from zero had a faster decline in the most recent period 2015–2023, rather than in the earlier years (1990–2015).

Due to the slower decline in most subregions with the highest adolescent birth rates, the regional disparities increased over time. In 2023, the birth rate for girls under age 15 in sub-Saharan Africa was 3.2 (95% UI 2.5 to 4.2) per 1000, more than 30 times larger compared with 0.1 (95% UI 0.1 to 0.2) per 1000 in Europe, Northern America, Australia and New Zealand. Birth rates for girls below age 15 were also higher than the global level in both Latin America and the Caribbean (LAC) (1.7 (95% UI 1.3 to 2.4) births per 1000 girls aged 10–14 years) and in Oceania (excluding Australia and New Zealand) (1.4 (95% UI 0.5 to 4.4) births per 1000 girls aged 10–14). The relative difference between the regions with the highest birth rates (sub-Saharan Africa) and the lowest birth rate (Europe, Northern America, Australia and New Zealand) increased in the recent period: it was 23 times in 1990, 22 times in 2015 and increased to 32 times in 2023. The corresponding relative difference between the highest and the lowest regional fertility rates for ages 15–19 is narrower but increased over time: around 4 times, 7 times and 10 times in 1990, 2015 and 2023, respectively.

**Figure 1:**
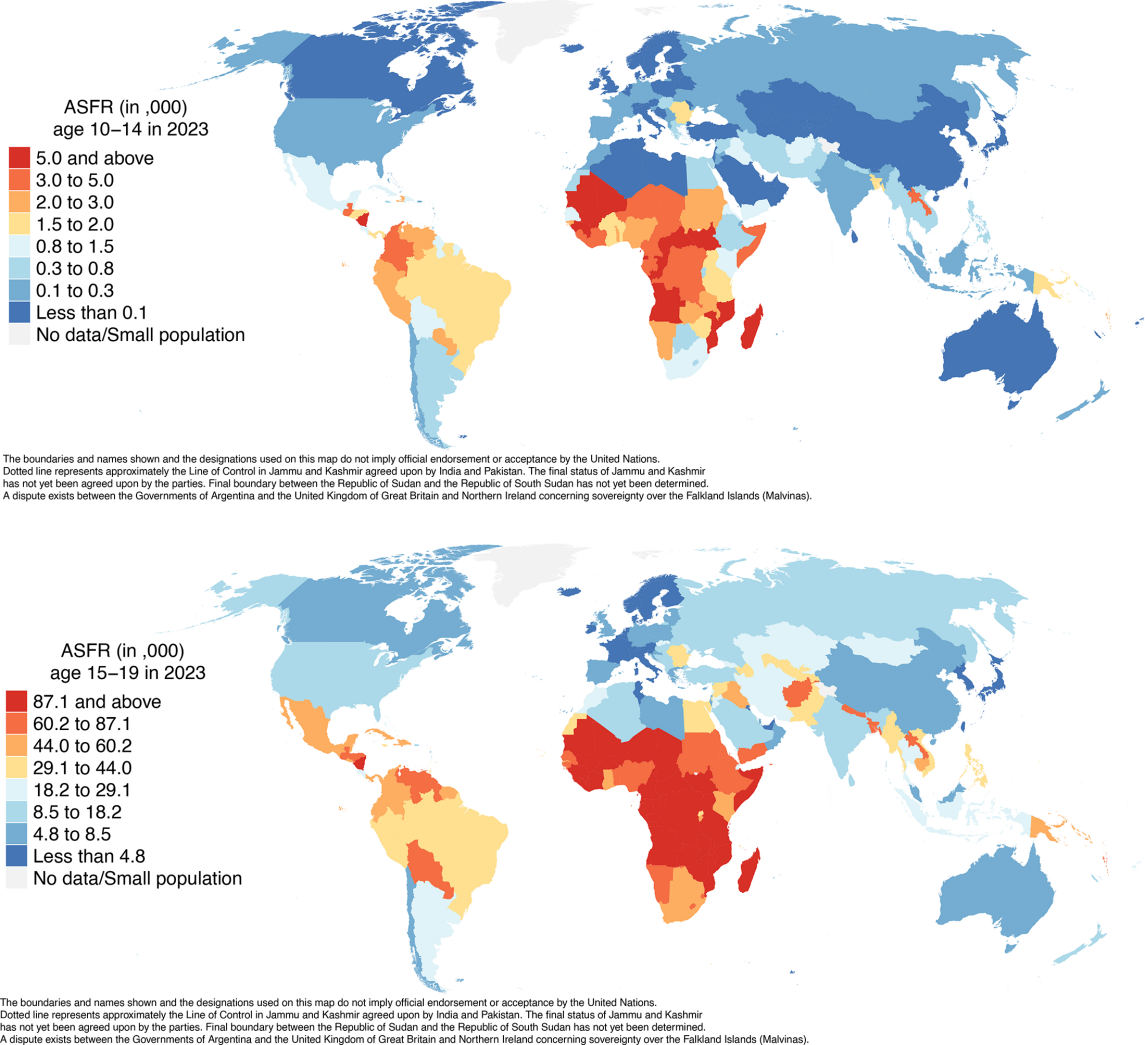
ASFR aged 10–14 (top) and 15–19 (bottom) in 2023. Median estimates are shown in the plot. Countries/areas in grey: estimates are not reported due to small populations or lack of data. ASFR, age-specific fertility rate.

**Figure 2:**
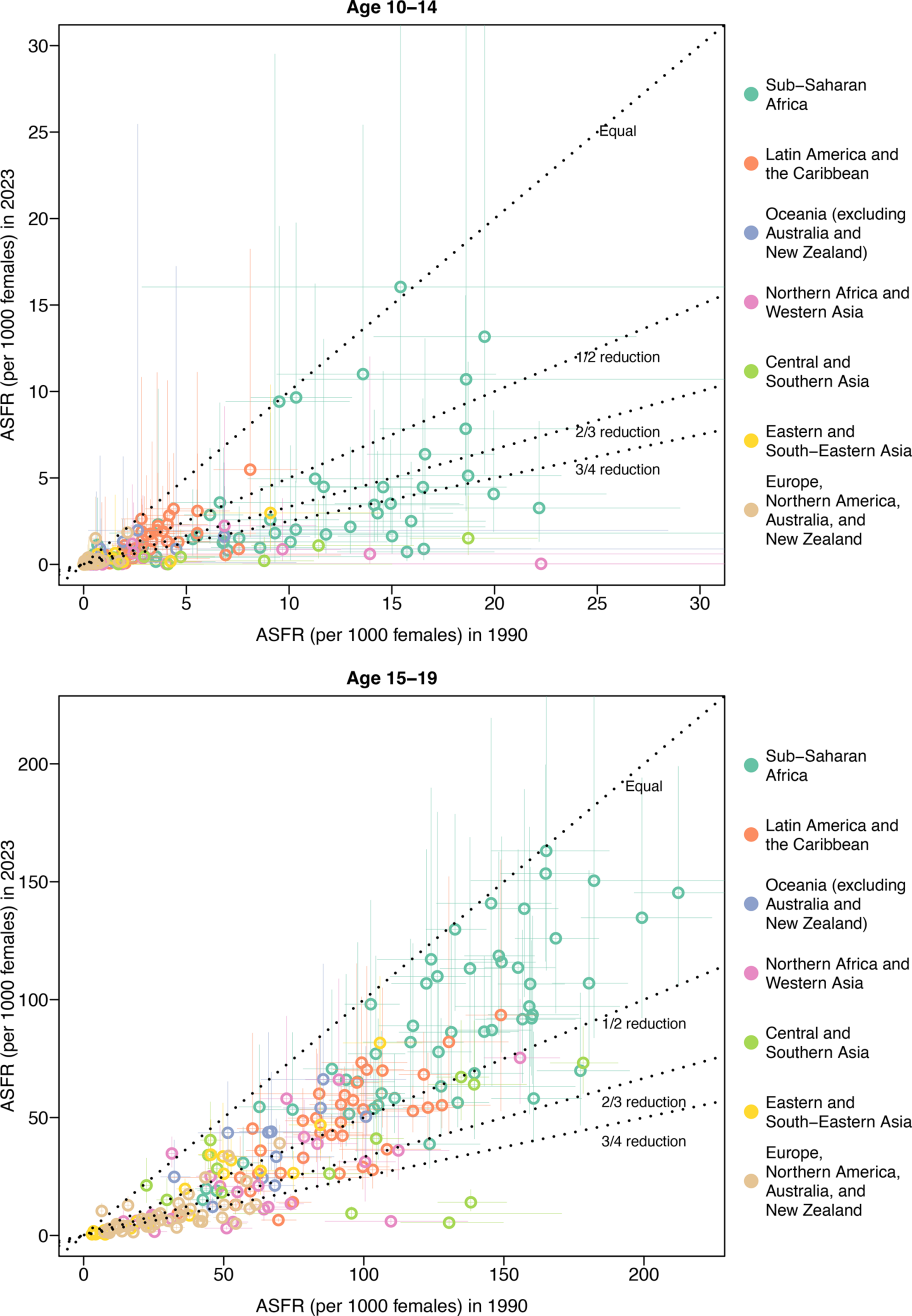
ASFR in 1990 and 2023 for age groups 10–14 (top) and 15–19 (bottom). Estimated rates are shown with 95% UI (vertical lines are 95% UI in 2023, and horizontal lines 95% UI in 1990), by country and SDG region (SDG region groupings can be found in the online supplemental appendix table 10, pp 25–26). ASFR, age-specific fertility rate; SDG, Sustainable Development Goal; UI, uncertainty interval.

In 2023, four countries (Angola, Equatorial Guinea, Mauritania and South Sudan), all in sub-Saharan Africa, had fertility rates greater than 10 births per 1000 girls aged 10–14 (figure 1). The national fertility rates for girls under 15 decreased by at least two-thirds in 115 (95% UI 102 to 122) countries; among them, 94 (95% UI 84 to 104) decreased by more than three-quarters (figure 2). The country-level ARRs are significantly above zero in 88 countries over 1990–2023, 85 between 1990 and 2015 and 18 over 2015 and 2023 (online supplemental appendix tables 13 and 14). For girls and young women aged 15–19, 72 (95% UI 67 to 79) saw their fertility in ages 15–19 decline by more than two-thirds between 1990 and 2023; of those, 44 (95% UI 41 to 51) had more than a 75% reduction since 1990.

### Births by mothers under-20

**Figure 3:**
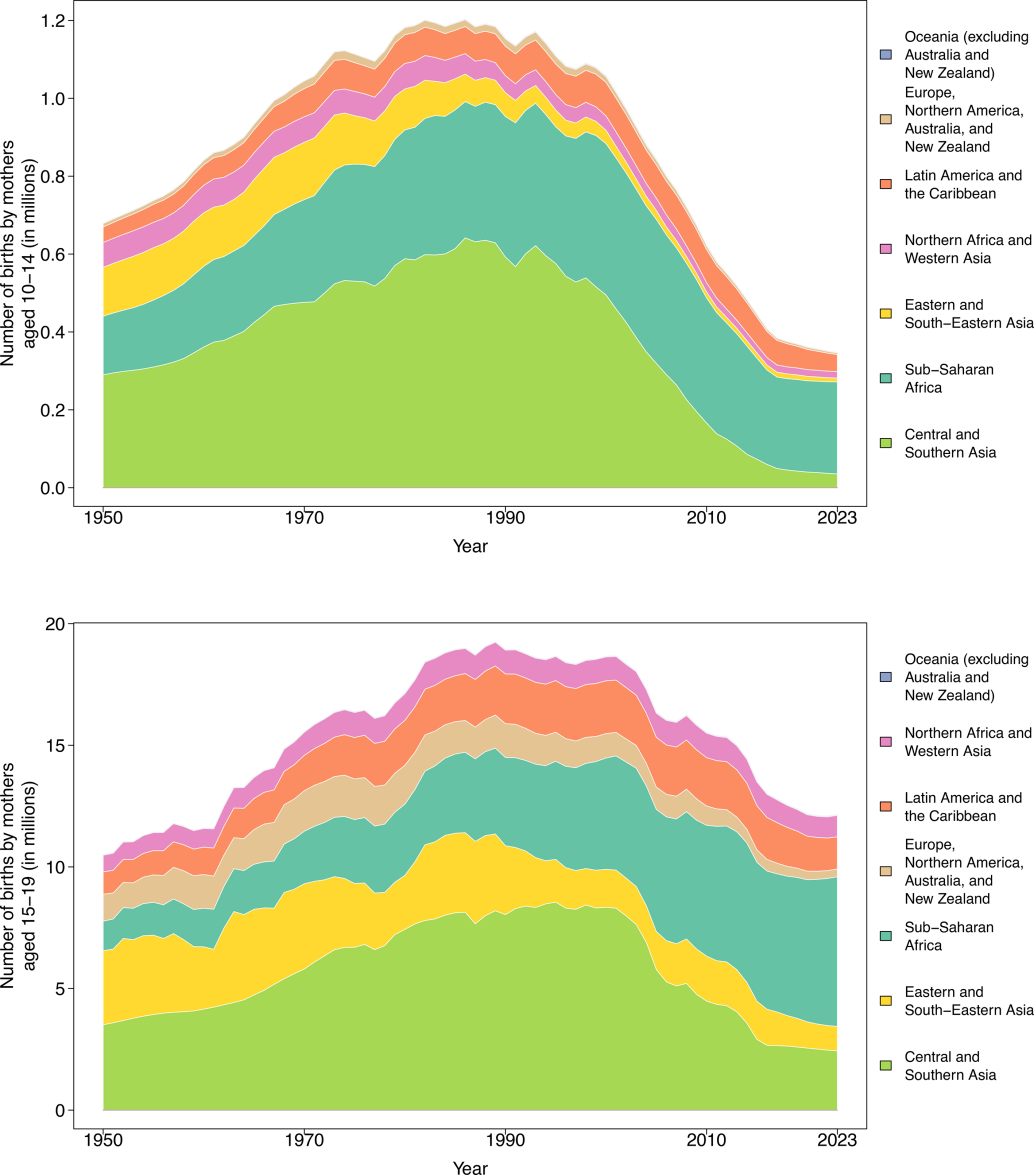
Number of births (in thousands) by mothers aged 10–14 (top) and 15–19 (bottom) from 1950 to 2023, by SDG regions. SDG, Sustainable Development Goal.

In 2023, estimated global births to girls aged 10–14 totalled 347 (95% UI 285 to 454) thousand, down from 1150 (95% UI 1050 to 1340) thousand in 1990 (table 2, figure 3). For girls and young women aged 15–19, births decreased from 18.9 (95% UI 18.4 to 19.5) million in 1990 to 12.2 (95% 11.5 to 12.9) million in 2023. The number of births decreased in all regions in both age groups, except the number of births to girls and young women aged 15–19 that rose in sub-Saharan Africa (3.6 (95% UI 3.6 to 3.7) million births in 1990 to 6.2 (95% UI 5.6 to 6.7) million in 2023) and in Oceania (excluding Australia and New Zealand – 25 (22 to 28)^21 22^ thousand births in 1990 and 32 (29 to 37)^23 24^ thousand in 2023), driven by population growth and slow declines in birth rates in this age group. In 1990, Central and Southern Asia had the highest percentage of the world’s adolescent births among all regions, with 592 (95% UI 500 to 704) thousand births age 10–14 (accounting for 51.3% (95% UI 44.1% to 56.2%) of total births) and 8.0 (95% UI 7.5 to 8.6) million (42.4% (95% UI 40.8% to 44.1%)) for age group 15–19, while in 2023, most of the births to adolescents occurred in sub-Saharan Africa, accounting for 68.1% (95% UI 53.9% to 77.6%) and 50.6% (95% UI 47.7% to 53.5%) of total births for age 10–14 and 15–19, respectively.

**Table 2:** Estimates and 95% uncertainty intervals for the number of births (in thousands) for mothers from age groups 10–14, 15–19, for the world and regions, in 1990, 2015 and 2023.

Age group	10–14 years			15–19 years		
Year	1990	2015	2023	1990	2015	2023
Number of births (in thousands)						
World	1150 (1050 to 1340)	446 (407 to 499)	347 (285 to 454)	18 900 (18 400 to 19 500)	13 500 (13 200 to 13 800)	12 200 (11 500 to 12 900)
Sub-Saharan Africa	361 (336 to 389)	260 (231 to 294)	236 (189 to 309)	3630 (3550 to 3720)	5710 (5520 to 5920)	6150 (5620 to 6740)
Latin America and the Caribbean	76 (63 to 97)	73 (62 to 86)	44 (32 to 62)	2040 (1890 to 2240)	1810 (1750 to 1880)	1320 (1180 to 1480)
Oceania (excluding Australia and New Zealand)	2 (1 to 3)	1 (1 to 2)	1 (0 to 3)	25 (22 to 28)	32 (29 to 37)	32 (23 to 47)
Northern Africa and Western Asia	44 (36 to 62)	20 (13 to 33)	17 (8 to 39)	979 (942 to 1020)	974 (884 to 1080)	883 (748 to 1050)
Central and Southern Asia	592 (500 to 704)	73 (54 to 100)	35 (17 to 104)	8040 (7530 to 8590)	2910 (2760 to 3070)	2440 (2100 to 2840)
Eastern and South-Eastern Asia	61 (46 to 231)	13 (9 to 35)	10 (5 to 34)	2830 (2700 to 2980)	1570 (1460 to 1700)	1000 (874 to 1160)
Europe, Northern America, Australia and New Zealand	19 (15 to 24)	7 (5 to 8)	4 (3 to 7)	1390 (1350 to 1430)	515 (499 to 533)	319 (292 to 350)
Proportion of births among total births						
World	100%	100%	100%	100%	100%	100%
Sub-Saharan Africa	31.2% (26.6% to 34.7%)	58.2% (52.6% to 63.0%)	68.1% (53.9% to 77.6%)	19.2% (18.5% to 19.9%)	42.2% (41.1% to 43.4%)	50.6% (47.7% to 53.5%)
Latin America and the Caribbean	6.6% (5.2% to 8.4%)	16.3% (13.6% to 19.3%)	12.6% (8.7% to 17.9%)	10.8% (10.0% to 11.7%)	13.4% (12.9% to 13.9%)	10.9% (9.7% to 12.2%)
Oceania (excluding Australia and New Zealand)	0.1% (0.1% to 0.2%)	0.2% (0.1% to 0.5%)	0.3% (0.1% to 0.9%)	0.1% (0.1% to 0.1%)	0.2% (0.2% to 0.3%)	0.3% (0.2%; to 0.4%)
Northern Africa and Western Asia	3.8% (3.0% to 5.3%)	4.4% (2.8% to 7.1%)	4.8% (2.2% to 10.8%)	5.2% (4.9% to 5.4%)	7.2% (6.6% to 7.9%)	7.3% (6.2% to 8.6%)
Central and Southern Asia	51.3% (44.1% to 56.2%)	16.4% (12.3% to 21.4%)	10.2% (4.9% to 25.4%)	42.4% (40.8% to 44.1%)	21.5% (20.5% to 22.5%)	20.1% (17.6% to 22.8%)
Eastern and South-Eastern Asia	5.3% (4.0% to 17.2%)	3.0% (2.1% to 7.5%)	2.8% (1.5% to 9.2%)	15.0% (14.2% to 15.7%)	11.6% (10.9% to 12.5%)	8.3% (7.2% to 9.6%)
Europe, Northern America, Australia and New Zealand	1.6% (1.2% to 2.1%)	1.5% (1.2% to 1.9%)	1.2% (0.7% to 2.2%)	7.3% (7.1% to 7.7%)	3.8% (3.7% to 4.0%)	2.6% (2.4% to 2.9%)

The proportion of births among total births in an age group for the world and each region. Regional births and proportions may not sum up to the global births or 100%, respectively, due to rounding.

The births by under-15 girls were also concentrated in a small number of countries—36% of all births worldwide born by under-15 girls in 2023 occurred in just five countries (in descending order of median estimates): Nigeria, Democratic Republic of the Congo, Angola, Madagascar and Mozambique (online supplemental appendix table 15).

## Discussion

Our study provides annual estimates and uncertainty assessments of adolescent birth rates (ABR) for girls aged 10–14 and 15–19 across 201 countries or territories from 1950 to 2023. To our knowledge, we compiled the most comprehensive publicly available global database for adolescent fertility, integrating diverse data sources and systematically adjusting for biases. Using a Bayesian statistical model, the ABR estimates for age groups 10–14 and 15–19 are more data-driven than estimates from the GBD^13^ and previous revisions of World Population Prospects estimates.^25^ With the hierarchical structure in the Bayesian estimation model, we allow data sharing across all countries to better inform the levels and trends of ABR in country-periods with limited or no data. Doing so provides a clearer understanding of early childbearing trends, highlighting disparities and progress across different contexts and periods. After considering the female population sizes, we presented the distribution of births to girls aged 15–19 and under 15 across countries over time.

Despite advances in data collection and estimation methods, substantial data gaps remain and data availability, timeliness and coverage remain a challenge for many countries, as discussed in the Methods section. These data gaps and discrepancies observed in specific data sources, can impact fertility estimates and our statistical model reflects them in the results and uncertainty ranges. However, some of the discrepancies require additional in-depth country-specific research, for example, different data sources for the adolescent birth rate in India in 2019 suggest very different values (online supplemental appendix pp 398): 43 births per 1000 girls and young women aged 15–19 years^21^ and 12 births per 1000 girls and young women aged 15–19 years.^26^ Various data sources all point to large decreases in ABR since 1990, yet at a different pace. Since India has the largest population of adolescents globally, its estimate greatly impacts the evaluation of progress made towards reducing adolescent childbearing, not only in the country but also in the region and globally. Addressing these gaps requires enhanced data collection systems, including evaluating the reporting for completeness of vital registration and detailed disaggregation by socioeconomic and geographical factors to identify vulnerable populations. Improving the reliability, coverage, timeliness and accessibility of demographic data should be a central focus of efforts to strengthen statistical systems for monitoring progress towards reducing adolescent childbearing.

Our methodology demonstrates several improvements over previous studies. First, the database is much more extensive than the GBD database; it is 3.6 times the size of the GBD database. Specifically, the GBD study does not use retrospective birth history data for ages 10–14. Second, unlike GBD estimates, which rely on a six-step modelling process, this study relies on a neater three-step procedure and produces plausible results with good validation results. Third, our Bayesian models use the TFR as an external covariate to enhance the ASFR model results. This differs from the GBD 2021 study, where the TFR is calculated by summing up the ASFR. Generally, more observations are available for TFR, including estimates from indirect methods from censuses and surveys that cannot be calculated for ASFRs. Hence, in the WPP approach, the TFR is modelled separately from the ASFR^17^ and the ASFR across age groups are adjusted to be consistent with TFR estimates. Lastly, the regular biennial updates of the WPP will provide updated estimates of ABR 10–14 and 15–19 based on the most recent data at every revision.

Our study is subject to limitations regarding data and model assumptions. First is the reliance on backwards projections for countries with limited historical data. We used total fertility and educational attainment as covariates in the model to assist backwards projection from the earliest available observation to 1950. In our database, 53% and 62% of countries have their earliest data prior to 1960 for ages 10–14 and 15–19, respectively. The average backwards projection period is 14 years among countries and territories with data for ages 10–14, whereas the period exceeds 20 years for 29% of these locations (online supplemental appendix pp 4). Only 21% and 9% countries for ages 10–14 and 15–19 requires a backward extrapolation for 25 years and above to 1950. Therefore, estimates in the early period were more dependent on these auxiliary covariates. This is a less relevant limitation for the most recent periods. While the country figures provide estimates with underlying data for 1950–2023 (online supplemental appendix pp 111–514), the main results are discussed from 1990 to 2023. Second, the covariate choice is universal across all country-years. This might not fully capture the underlying adolescent fertility pattern for a certain country-period. However, it would be extremely challenging to obtain variables available for all country-years with plausible quality. Third, the uncertainty bound with respect to the estimated ASFR level can be wide due to either lack of informative data in the early period or close-to-zero estimates in the recent period (online supplemental appendix pp 19, 21), which is inevitable for all modelling studies. That said, we provide uncertainty so that users are informed about the levels and associated uncertainty when interpreting the results. Finally, while our model already includes the bias specific to the time elapsed between the event and interview date (ie, the recall lag), no adjustment was applied to the empirical data and further exploration in our model is needed to incorporate this potential bias.

Our findings show significant global reductions in adolescent fertility rates since 1990. The global progress in reducing adolescent childbearing since 2015, after adopting the SDG, has remained similar to the period between 1990 and 2015 as measured by annual rates of change. While in the period 1990–2015, most of the global progress was driven by the declines in ABR in Southern Asia, since 2015, LAC, the region with previously stagnant ABR, and Europe, Northern America and Australia/New Zealand, the region with already low ABR, have seen an acceleration of the decline, showing that decline in ABR can accelerate in the regions with previously high and slow decline, and further rapid decline can happen also in countries with already low levels of ABR.

The greatest absolute and relative decreases were observed in Central and Southern Asia; within the region, the largest decline was observed in the region’s largest country. In India, ABR is estimated to have declined between 1990 and 2023 from 8.8 (95% UI 6.9 to 11.2) per 1000 to 0.2 (95% UI 0.0 to 1.6) per 1000 for ages 10–14 and from 138.2 (95% UI 126.4 to 151.0) per 1000 in 1990 to 14.1 (95% UI 10.3 to 19.5) per 1000 for ages 15–19. These reductions are partially attributable to the decline in the incidence of child marriage from 26.1% of women married by age 15 and 54.2% married by age 18–4.8% married by age 15 and 23.3% married by age 18 between the early 1990s and the early 2020s.^21 27^ Also, education expanded greatly among young adolescents in India. In the early 1990s, only 55% of girls aged 11–14 were attending school; by 2020, this percentage reached 91%. India implemented major adolescent health policies, namely the Adolescent Reproductive and Sexual Health Strategy in 2005, Rashtriya Kishor Swasthya Karyakram in 2014 and the School Health Programme in 2020, providing a comprehensive framework for sexual and reproductive health services, and expanding the scope of adolescent health programming, and addressing various health aspects, leading to higher use of modern contraception among adolescents, decline in the incidence of violence and increase in positive health behaviours, such as condom use.^28^ Challenges remain in limited access to health services, particularly in rural areas, low health insurance coverage and persistent inequalities.

Compared with other regions, sub-Saharan Africa experienced slower declines in adolescent fertility rates and remains the region with the highest fertility rates for both age groups 10–14 and 15–19. Higher levels of poverty, lower access to education, limited availability of sexual and reproductive healthcare services and the persistence of early and child marriage contribute to the region’s slow progress in reducing adolescent fertility.^22 29 30^ At the same time, sexual and reproductive healthcare services are often unavailable, unaffordable or not designed to meet the needs of young people, especially if they are unmarried. Cultural stigma around adolescent sexuality and contraceptive use, and misconceptions about contraception further discourage access and use.^23 31^ While the adolescent birth rates declined slowly from 1990 to 2023, the number of births to adolescent mothers increased in sub-Saharan Africa, driven by growth in the numbers of adolescents in the population resulting from prevailing high TFR. Therefore, the births to adolescent mothers are now concentrated to this region, accounting for 68% of global births in the age group below 15% and 51% in the age group 15–19. The global efforts to reduce adolescent childbearing should increasingly concentrate on this region where girls are still subject to high risk of early childbearing and the number of adolescent girls is projected still to increase in coming decades.^14^

After no or only small reductions from 1990 to 2015, several countries in LAC—such as Argentina, Chile, Costa Rica and Uruguay—experienced a sharp decrease in adolescent birth rates after 2015. Chile registered the highest reductions with ARRs equal to 36.18% (95% UI 26.80% to 46.15%) for age group 10–14 and 22.80% (95% UI 20.79% to 24.10%) for age group 15–19 during 2015–2023. The recent declines resulted from effective national public policies backed by the 2013 Montevideo Consensus on Population and Development,^32–34^ which called for reducing adolescent pregnancy through comprehensive programmes that ensure access to high-quality, confidential and free sexual and reproductive health services for adolescents. In Argentina, the National Plan for the Prevention of Unintended Adolescent Pregnancy (ENIA) included comprehensive sexuality education, access to contraceptive methods, strengthening adolescent-friendly health services and training of health and education professionals.^35^ Chile implemented the National Sexual and Reproductive Health Strategy for Adolescents, focused on family planning, healthcare rights, abortion and health system innovation to safeguard adolescents’ access to confidential sexual and reproductive healthcare services.^36^ In Uruguay, the National and Intersectoral Strategy for the Prevention of Unintended Pregnancy in Adolescents has contributed to reducing adolescent pregnancies through intersectoral coordination, educational campaigns and access to health services.^37^ Of the 33 countries in LAC that committed to the Montevideo Consensus, 22 have reported progress regularly.^24^

Countries with the highest rates of child marriage are also those with high rates of early childbearing. Globally, most births to adolescent mothers occur within marriage.^11 38 39^ Still, there are large regional differences, and early childbearing outside marriage remains rare in Central and Southern Asia, parts of South-Eastern Asia, and Northern Africa and Western Asia.^39^ Over the past three decades, the incidence of child marriage has declined globally, driven predominantly by a decline in India, as well as in populous countries where the practice has historically been common, such as Bangladesh and Ethiopia. Levels of child marriage are highest in Western and Central Africa, where progress has stalled.^40^ WHO’s guidelines for preventing child marriage recommend implementing interventions to empower girls, engaging communities, providing incentives for education and delaying marriage, removing barriers to schooling, and boosting girls’ economic skills.^41^ Future work should investigate the impact of policy and programmatic interventions (such as raising legal age at marriage and implementing it, increasing secondary education participation of girls) on pregnancy and births rates among the youngest adolescents.

In many contexts, adolescents face significant challenges related to their sexual and reproductive health and rights.^42^ Early sexual activity, marriage and pregnancy often occur in environments where gender-based violence and violations of sexual and reproductive health rights are prevalent.^43^ Adolescents, and particularly those unmarried, who are sexually active and do not desire to become pregnant soon, are known to have lower levels of contraceptive use compared with older women. This is particularly true in sub-Saharan Africa, where only about half of adolescent females aged 15–19 who want to avoid pregnancy used modern contraceptive methods—the lowest share of any region.^25 44^ The region’s large and growing share of the population of girls at risk of early pregnancy will require substantial investments into family planning programmes, especially in poor and rural areas where adolescent girls and young women are less likely to use modern contraception.^45^ Increasing access to, uptake of, and continued use of contraception among adolescents requires the implementation of gender-transformative behaviour-change interventions with adolescents to strengthen their ability to make decisions about their contraceptive use to shift gender norms to support contraceptive decision-making; and to improve the quality of health services.^41^ It is estimated that annually some 5.7 million adolescent pregnancies end in abortions, the majority of which occur in unsafe conditions^46^ and successful policies also require interventions to reduce unsafe abortion.

Especially in regions with high rates of adolescent pregnancies and childbearing, efforts to address the specific needs and rights of girls who are pregnant or are mothers should include age-appropriate antenatal, birth and postnatal care that mitigates common health risks that affect pregnant and parenting adolescents and addresses adolescent-specific challenges and barriers to care.^47 48^ Concurrently, they should receive support for further education and acquiring employment skills.

In conclusion, our study provides insights into global, regional and national levels and trends in adolescent fertility trends, offering robust evidence to inform policy and programmatic interventions. While substantial progress has been made since 1990, achieving the 2030 SDG will require sustained efforts to address regional disparities, pursue data collection efforts, enhance data quality and promote gender equality worldwide.

## Supplementary material

10.1136/bmjgh-2025-021301 online supplemental file 1

## Data Availability

Input database is available on the UN WPP website https://population.un.org/wpp/data-sources. All other data relevant to the study are included in the article or uploaded as supplementary information.
