# Supplementary material for: Levels and trends in fertility rates among adolescents aged 10–14 and 15–19 in 201 countries and areas from 1950 to 2023
Source: BMJ Glob Health. 2026 Jul 29;11(7):e021301. doi: 10.1136/bmjgh-2025-021301 (PMC13475349; doi:10.1136/bmjgh-2025-021301)
Supplement: Supplementary file 1 [file bmjgh-11-7-s001.pdf]

Web Appendix for
Levels and trends in fertility rates among adolescents aged 10–14 and 15–19 in 201 countries or areas from 1950 to 2023

Fengqing Chao*[1], Vladimíra Kantorová[2], Giulia Gonnella[2], Bruno Schoumaker[3], David A. Sánchez-Páez[4], and Patrick Gerland[2]

[1]Computational Social Science Division, School of Humanities and Social Science, The Chinese University of Hong Kong, Shenzhen, Guangdong, 518172, P.R. China

[2]Department of Economic and Social Affairs, United Nations Population Division, New York, NY, USA

[3]Centre for Demographic Research, Iacchos Institute, UCLouvain, Belgium

[4]Department of Economic Theory, University of Valladolid, Spain

July 14, 2026

*Corresponding author (FC); Email: chaofengqing@cuhk.edu.cn
Please cite this web appendix using its DOI: 10.6084/m9.figshare.31524454
The views expressed herein are those of the authors and do not necessarily reflect the views of the United Nations.

Contents

1 Data **1**
- 1.1 Data sources . . . 1
- 1.2 Data availability . . . 1
- 1.3 Data from birth histories . . . 4
- 1.4 Most recent data available . . . 4
- 1.5 Data type . . . 4

2 Data pre-processing **5**
- 2.1 Extraction by data source type . . . 5
- 2.2 Adjustments for the incompleteness of birth registration . . . 6
- 2.3 Exclusion criteria . . . 7

3 Methods for age-specific fertility rate 10–19 **7**
- 3.1 Data model for age group 15–19 . . . 7
- 3.2 Data model for age group 10–14 . . . 9
- 3.3 Process model for ASFR 15–19 . . . 9
- 3.4 Process model for ASFR 10–14 . . . 12
- 3.5 Model summary . . . 13
- 3.6 Computation of final results . . . 15
  - 3.6.1 Rounding . . . 15
  - 3.6.2 Constructing uncertainty intervals and point estimates . . . 15
  - 3.6.3 Age-specific births to be consistent with total births . . . 16
  - 3.6.4 Annual rate of reduction . . . 16
  - 3.6.5 Computing aggregated results . . . 17

4 Validation exercise and results **17**

5 Sensitivity analyses **18**
- 5.1 Time series model setup . . . 18
- 5.2 ASFR 10–14 model setup . . . 19

6 Uncertainty assessments of model results **19**

7 Sub-regional results **21**

8 Country-level results **21**

9 Comparison of the results with GBD 2021 estimates **23**

10 GATHER Compliance **28**

11 Supplementary tables **31**

12 Supplementary figures **111**

List of Tables

1 Distribution of fertility observations by data source for girls aged 10–14 and 15–19 2
2 Data availability on fertility rates for age groups 10–14 and 15–19 by the type of survey 2
3 Number of observations of fertility rates for age groups 10–14 and 15–19 by age group and recall period 4
4 Number of countries with the latest available ASFR data in age groups 10–14 and 15–19, by 5-year periods preceding the survey 5
5 Distribution of fertility observations by data type for mothers aged 10–14 and 15–19 5
6 Notation summary 14
7 Validation results for left-out observations by age group. 18
8 Summary of differences in ASFR estimates in observation years 2000, 2005, and 2010 based on training dataset and full dataset. 18
9 Model comparison for time series setup 19
10 Model comparison for time series setup. 19
11 Checklist of information that should be included in new reports of global health estimates 28
12 Classification of countries/areas by SGD region and subregion 31
13 Estimates and 95% uncertainty intervals for ASFR 10–14 in 1990, 2015, and 2023, for the world, SDG regions, subregions, and all countries 33
14 Estimates and 95% uncertainty intervals for ASFR 15–19 in 1990, 2015, and 2023, for the world, SDG regions, subregions, and all countries 39
15 Estimates and uncertainty intervals for births (in thousands) by mothers aged 10–14 and the percentage of births in each location among all births of 10–14 for 1990, 2015, and 2023 for the world, SDG regions, sub-regions, and all countries 45
16 Estimates and uncertainty intervals for births (in thousands) by mothers aged 15–19 and the percentage of births in each location among all births of 15–19 for 1990, 2015, and 2023 for the world, SDG regions, sub-regions, and all countries 50
17 Age-specific fertility rate data sources for age group 10–14, by country 55
18 Age-specific fertility rate data sources for age group 15–19, by country 75

List of Figures

1 Overview of the earliest data across countries by age group 2
2 Number of observations for age-specific fertility rates 10–14 by country 3
3 Number of observations for age-specific fertility rates 15–19 by country 3
4 Data and model process overview 8
5 Scatter plots between ASFR 10–14 and 15–19 observations, by data type and by region 10
6 Illustration of $\lambda_{c,t}$ as input data for model 10–14 for one country 11
7 Regional relation between TFR and ASFR 15–19 12
8 ASFR 10–14 model fit comparison during 1950–2023 20
9 Coefficient of variance for ASFR 10–14 model results 22
10 Coefficient of variance for ASFR 15–19 model results 23
11 ARR by mothers aged 10–14 and 15–19, in 22 sub-regions 24
12 ASFR aged 10–14 in 2023 on global, regional, and national levels 25
13 ASFR aged 15–19 in 2023 on global, regional, and national levels 26
14 Distribution of regional absolute and percentage differences between the WPP 2024[1] and the GBD 2021 estimates, for years 1990 and 2021 27
15 ASFR 10–14 estimates and uncertainty intervals during 1950–2023, by country 111
16 ASFR 15–19 estimates and uncertainty intervals during 1950–2023, by country 313

List of Abbreviations

AR1	first-order Autoregressive
ARR	Annual Rate of Reduction
ASFR	Age-Specific Fertility Rate
BRC	Birth-reporting Completeness
CI	Credible Interval
CPO	Conditional Predictive Ordinates
CPS	Contraceptive Prevalence Surveys
CRVS	Civil Registration and Vital Statistics
CV	Coefficient of Variance
DHS	Demographic and Health Survey
DIC	Deviance Information Criterion
GATHER	Guidelines for Accurate and Transparent Health Estimates Reporting
GBD	Global Burden of Disease
IHME	Institute for Health Metrics and Evaluation
INLA	Integrated Nested Laplace Approximation
IPUMS	Integrated Public Use Microdata Series
IQR	Interquartile Range
MA	Moving Average
MICS	Multiple Indicator Cluster Surveys
OECD	Organisation for Economic Co-operation and Development
PAPCHILD	Pan-Arab Project for Child Development
PAPFAM	Pan-Arab Project for Family Health
PC	Penalized Complexity
PI	Prediction Interval
PMA	Performance Monitoring and Accountability
RHS	Reproductive Health Surveys
RW1	first-order Random Walk
RW2	second-order Random Walk
SDG	Sustainable Development Goals
SRS	Sample Registration System
TFR	Total Fertility Rate
UNFPA	United Nations Fund for Population Activities
UNICEF	United Nations Children's Emergency Fund
VR	Vital Registration
WAIC	Watanabe-Akaike Information Criterion
WFS	World Fertility Survey
WHS	World Health Surveys
WHO	World Health Organization
WPP	World Population Prospects

1 Data

1.1 Data sources

Empirical fertility data would ideally be collected and published as counts of live births by age of the mother through a system of civil registration and vital statistics (CRVS). However, not all countries produce or make available vital registration (VR) data that are reliable with national coverage and a high level of completeness. For those countries with lacking or deficient VR data, empirical fertility data can be complemented using estimates coming from household surveys that collect information on the complete childbearing history of the women interviewed. Such surveys are, for example, the Demographic and Health Survey (DHS), the Multiple Indicators Cluster Survey (MICS), and other international survey programs[1]. Other countries, instead, rely on their own national demographic surveys or sample vital registration systems. Many population censuses also collect fertility data by asking women about the number of children ever born and about the births that occurred in the 12 (or 24) months prior to the census date.

The estimates of age-specific fertility rates for girls aged 10–14 and 15–19 presented in this paper are based on a set of empirical data points, which have been collected and stored in the internal database of the United Nations Population Division (DemoData SQL)[2]. From the data available in the database, we selected those series that met the highest standards. In particular, we included only VR data for location-years for which birth registration completeness was higher than 60% [6]. In case of more than one series of estimates or VR that were available in a specific country-year, we kept only one series of estimates and one series of VR to avoid duplication.

1.2 Data availability

This study only reports the model estimates of age-specific fertility rates for mothers aged 10–14 and 15–19 among 201 countries (referring to populations that are considered as “countries” or “areas” in the UN classification; hence, the expressions “country” and “country/areas” are interchangeable in this appendix) with at least 90,000 inhabitants by mid-2024. The input database is based on a bigger set of countries with 1,000 and above inhabitants by mid-2024.

Table 1 presents the observation distribution by data source for age groups 10–14 and 15–19. The sub-sources within the survey main source type by age group are further listed in Table 2. The data coverage for the majority of countries/areas are good, dating back in the 1960s. Figure 1 summarizes the earliest data available across countries by age group.

At the end of the selection process, we had 15,685 empirical data points available for ages 10–14 (Table 1), covering 224 out of the 236 countries or areas with 1,000 inhabitants or more by mid-2024 (Figure 2). Most of these data were obtained from surveys (49.7%) and register (32.8%), and 17.4% were estimates (which are originated from VR data). Only 14 observations came from censuses (0.1%), which is considerably lower than for the age group 15–19 both in the absolute number and in percentage. The main reason for the difference is that women younger than 15 years are usually not requested to answer questions regarding recent births, and birth data are not tabulated for ages 10–14.

For the age group 15–19, we had a total of 21,017 empirical data points available (Table 1), covering all countries or areas with populations of at least 1,000 inhabitants by mid-2024 (Figure 3). The data were predominantly obtained from surveys (38.0%) and register (36.7%). One-fifth of the data points (20.3%) were estimates. Unlike the age group 10–14, where census data were rare, the age group 15–19 had a substantial number of observations from censuses, with 1,056 observations accounting for 5% of the total.

[1] Fertility estimates from some other international survey programs were considered, for example the Performance Monitoring and Accountability (PMA) surveys. Other international survey programs that provided fertility estimates in decades prior to 2010 included the World Fertility Survey (WFS), the Contraceptive Prevalence Surveys (CPS), the Reproductive Health Surveys (RHS), the Pan-Arab Project for Family Health (PAPFAM), and the Pan-Arab Project for Child Development (PAPCHILD).

[2] The data on number of births and fertility rates are obtained from the United Nations Statistics Division (Demographic Yearbook), national statistical offices and regional ones (e.g., Eurostat, OECD), United Nations Regional Commissions, other United Nations entities (e.g., UNFPA, UNICEF, WHO, World Bank), and complemented using international databases (the Human Fertility Database [2] and Human Fertility Collection [3], the International Data Base [4], the Global Burden of Disease project [5]), and public use microdata archives (e.g., DHS, MICS, IPUMS-International).

Data Source	Age group 10–14			Age group 15–19		
	# Countries	# obs.	Proportion of obs. (%)	# Countries	# obs.	Proportion of obs. (%)
Census	14	15	0.1	126	1,056	5.0
Survey	109	7,799	49.7	136	7,990	38.0
Estimate	4	2,727	17.4	96	4,262	20.3
Register	154	5,144	32.8	169	7,709	36.7
No data	12	0	0	0	0	0
**Total**	224	15,685	100	236	21,017	100
**Reference year range**	**1950–2023**			**1950–2023**		

Table 1: **Distribution of fertility observations by data source for girls aged 10–14 and 15–19.** Obs.: observation(s).

Survey	Age group 10–14			Age group 15–19		
	# Countries	# obs.	Proportion of obs. (%)	# Countries	# obs.	Proportion of obs. (%)
DHS	82	5,234	67.1	90	4,687	58.7
MICS	52	1,373	17.6	64	1,380	17.3
WFS	29	372	4.8	37	428	5.4
RHS	7	283	3.6	14	257	3.2
PAPFAM/PAPCHILD	10	267	3.4	14	242	3.0
MIS	19	125	1.6	19	128	1.6
Other	10	126	1.6	93	852	10.7
Panel	1	19	0.2	1	16	0.2
**Total**	109	7,799	100	136	7,990	100

Table 2: **Data availability on fertility rates for age groups 10–14 and 15–19 by the type of survey.** DHS: Demographic and Health Survey; MICS: Multiple Cluster Indicator Survey; WFS: World Fertility Survey; RHS: Reproductive Health Survey; PAPFAM: the Pan-Arab Project for Family Health; PAPCHILD: the Pan-Arab Project for Child Development; MIS: Malaria Indicator Survey. Obs.: observation(s).

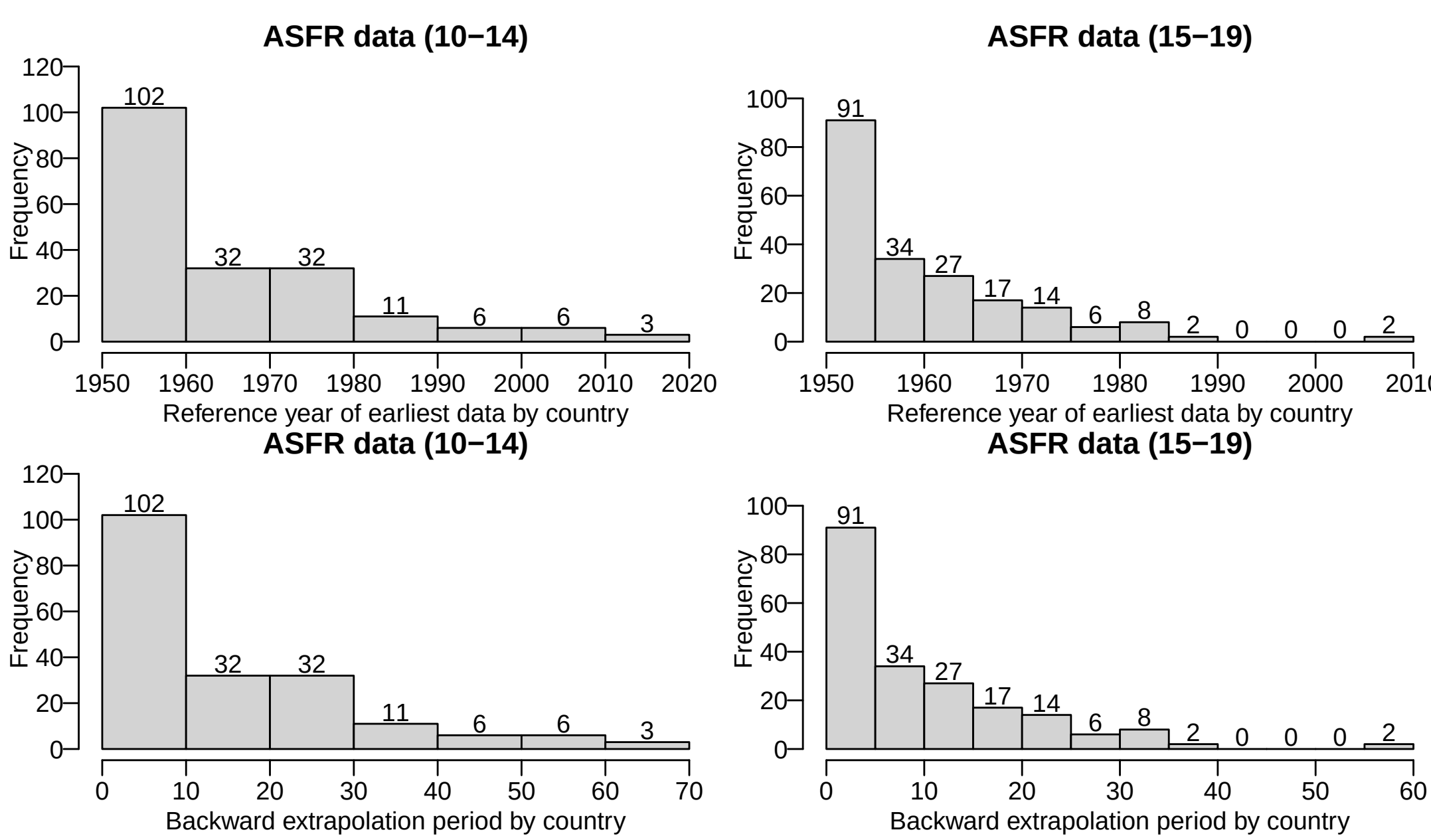

Figure 1: **Overview of the earliest data across countries by age group.** Top row: reference year of the earliest observation across countries. Bottom row: backward extrapolation period, computed as the difference between reference year of the earliest observation per country and 1950.

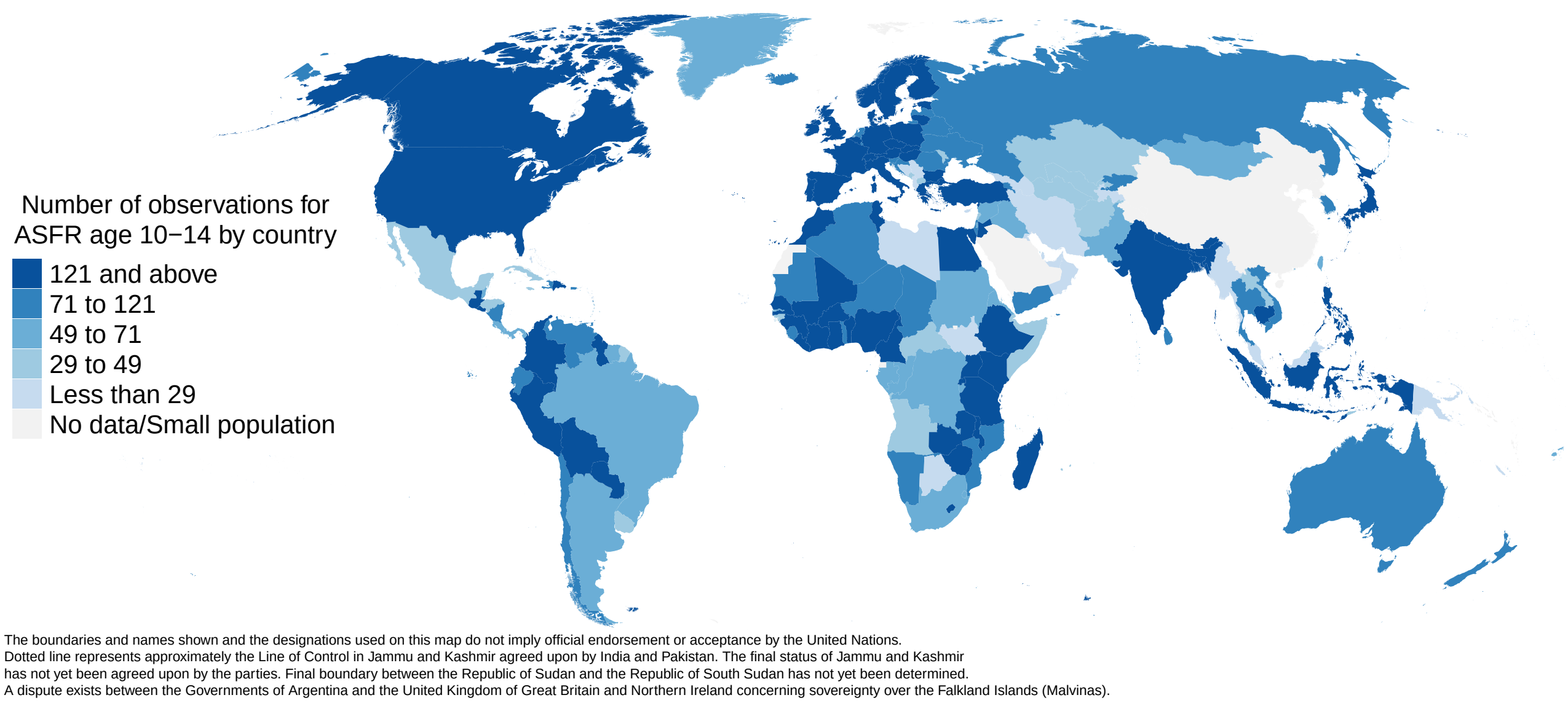

Figure 2: **Number of observations for age-specific fertility rates 10–14 by country.** Countries/areas are coloured by quintiles. Countries/areas in grey: not reporting due to small population (below 90,000 by mid-2024) or no data. Disclaimer: The boundaries and names shown and the designations used on this map do not imply official endorsement or acceptance by the United Nations. Dotted line represents approximately the Line of Control in Jammu and Kashmir agreed upon by India and Pakistan. The final status of Jammu and Kashmir has not yet been agreed upon by the parties. Final boundary between the Republic of Sudan and the Republic of South Sudan has not yet been determined. A dispute exists between the Governments of Argentina and the United Kingdom of Great Britain and Northern Ireland concerning sovereignty over the Falkland Islands (Malvinas).

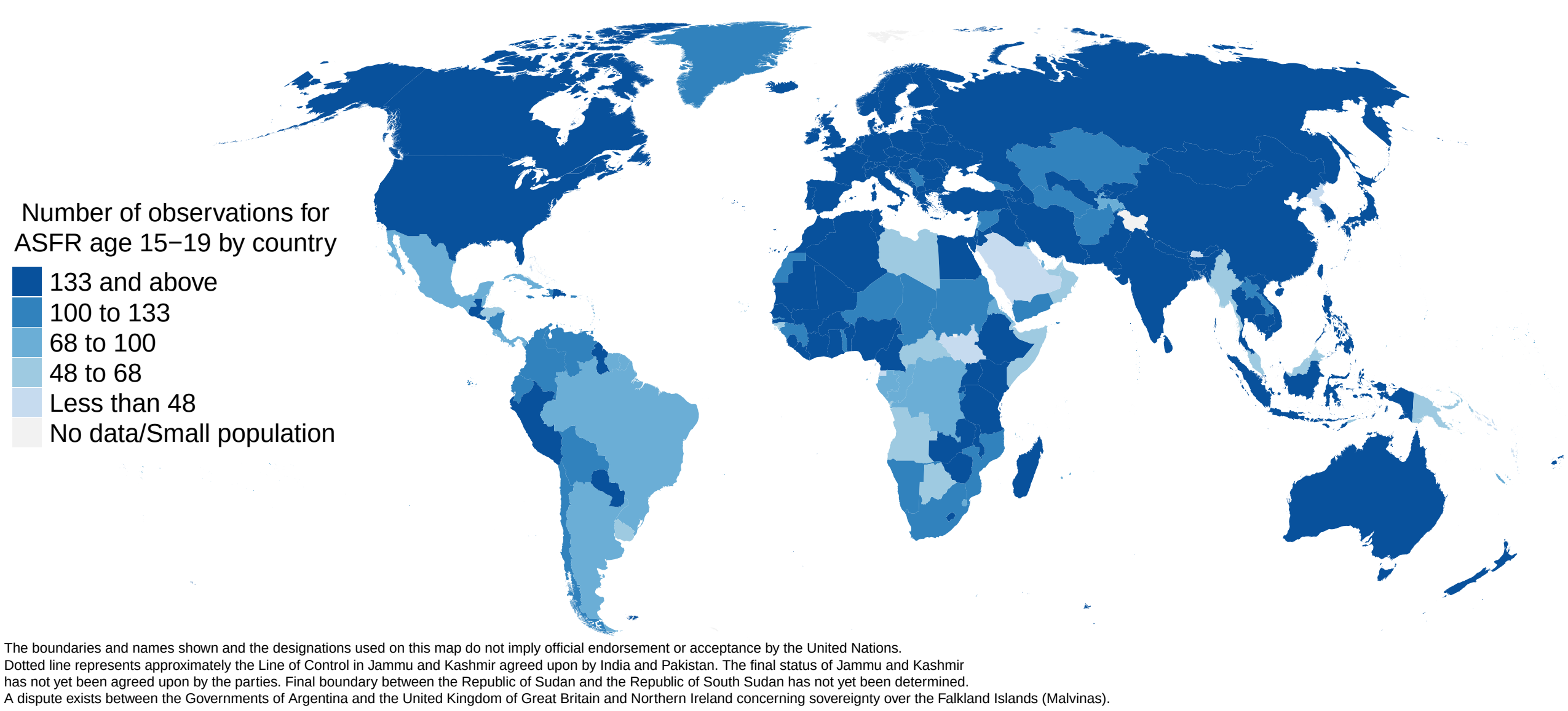

Figure 3: **Number of observations for age-specific fertility rates 15–19 by country.** Countries/areas are coloured by quintiles. Countries/areas in grey: not reporting due to small population (below 90,000 by mid-2024) or no data. Disclaimer: The boundaries and names shown and the designations used on this map do not imply official endorsement or acceptance by the United Nations. Dotted line represents approximately the Line of Control in Jammu and Kashmir agreed upon by India and Pakistan. The final status of Jammu and Kashmir has not yet been agreed upon by the parties. Final boundary between the Republic of Sudan and the Republic of South Sudan has not yet been determined. A dispute exists between the Governments of Argentina and the United Kingdom of Great Britain and Northern Ireland concerning sovereignty over the Falkland Islands (Malvinas).

1.3 Data from birth histories

Among the data coming from surveys (Table 2), for the age group 10–14, more than 5,200 observations came from DHS (67.1% of all survey data) and more than 1,300 observations from MICS (17.6%). For the age group 15–19, DHS also provided the largest proportion of survey data, with 4,687 observations, accounting for 58.7%, while MICS contributed 1,380 observations (17.3%).

In these surveys, women of reproductive age (usually defined as 15 to 49 years) are asked to reconstruct the full list of live births (or pregnancies) they had, including information on the dates of birth. This information on full birth histories permits calculation of age-specific fertility rates (ASFRs) by 3-year or 5-year periods prior to each survey ([7], [8]). Although only women 15–49 are usually eligible in these surveys, all their births were recorded. This makes it possible to compute fertility rates in the age group 10–14 up to 35 years before each survey containing full birth histories. For most time periods, the 10–14 fertility rates were computed in the same way as for the other age groups, by dividing the number of woman-years by the exposure. For the most recent period (3 or 5 years before the survey), the 10–14 fertility rate was computed using the "Lexis approach" proposed by Pullum and colleagues [7].[3] Table 3 shows the availability of such data for age groups 10–14 and 15–19. This source of information was particularly important for the assessment of fertility rates for ages 10–14 as it provided estimates for periods of time that were not otherwise covered by VR or censuses.

	**Number of years preceding the survey**						
**Age group**	**0–4**	**5–9**	**10–14**	**15–19**	**20–24**	**25–29**	**30–34**
10–14	470	427	421	414	405	385	371
15–19	473	429	429	426	430	400	0

Table 3: **Number of observations of fertility rates for age groups 10–14 and 15–19 by age group and recall period.** Recall period: 5-year intervals preceding surveys. Note: Observations that were selected from DemoData SQL database (`https://population.un.org/DemoData/web/` for the analysis of ASFRs as of March 2022.

1.4 Most recent data available

Regarding the recency of the empirical evidence, a large majority of the countries or areas with data available had observations for 2015 or later. For 44 countries, the latest data collection for the age group 10–14 occurred in 2014 or earlier (Table 4). Of these, for 19 countries, the most recent data were collected between 2010 and 2014, and for 18 countries, between 2000 and 2009. Among the countries with 90,000 inhabitants or more by mid-2024, 12 had no empirical data available on fertility rates for girls aged 10–14. For ages 15–19, only 25 countries had the most recent data available in 2014 or earlier.

1.5 Data type

We categorised all data sources into five types for data from age groups 10–14 and 15–19 (Table 5). This grouping of data types is used in the model setup where we model the non-sampling errors by data type (detailed in Section 3.1). Specifically, the groupings of data series into five different data source types are:

- Direct estimates of DHS: standard DHS with full birth histories;
- Direct estimates of other DHS-type surveys: Interim DHS, Special DHS, DHS AIDS Indicator Survey, DHS Malaria Indicators Survey, National DHS, World Fertility Survey, and surveys with similar sampling design and based on full birth histories;
- Others direct estimates: census, Reproductive Health Survey (RHS), Pan-Arab Programme on Family Health (PAPFAM), National MICS, panel data, and national reports, based on full birth histories;

[3] The rate in the age-group 10–14 for the three years before the survey is computed as a weighted average of the single-year rates for ages 12, 13, and 14. The method relies on the assumption that no births occurred before age 12. For the 5 years preceding the survey, the rate is computed as a weighted average of the single-year rates for ages 10, 11, 12, 13, and 14.

Reference year	Age group 10–14	15–19
2014 and earlier	44	25
2015	6	7
2016	13	9
2017	11	9
2018	24	19
2019	21	21
2020	17	23
2021	67	69
2022	19	49
2023	2	5
**Total**	224	236

Table 4: **Number of countries with the latest available ASFR data in age groups 10–14 and 15–19, by 5-year periods preceding the survey.** Note: Observations that were selected from DemoData SQL database (`https://population.un.org/DemoData/web/` for the analysis of ASFRs as of July 2024.

- Indirect estimates: national reports, based on summary birth histories and other model-based and undefined estimates;
- VR or Sample Registration System (SRS).

Data Type	Age group 10–14			Age group 15–19		
	# Countries	# obs.	Proportion of obs. (%)	# Countries	# obs.	Proportion of obs. (%)
DHS Direct	81	4,919	31.4	87	4,335	20.6
Other DHS Direct	88	2,799	17.9	106	2,747	13.1
Others Direct	19	92	0.6	135	802	3.8
Indirect	2	4	0.03	100	1,162	5.5
VR/SRS	157	7,871	50.2	180	11,951	56.9
No data	12	0	0.0	0	0	0.0
**Total**	236	15,685	100	236	20,997	100
**Reference year range**	**1950–2023**			**1950–2023**		

Table 5: **Distribution of fertility observations by data type for mothers aged 10–14 and 15–19.** DHS: Demographic and Health Survey. SRS: Sample Registration System. VR: Vital Registration. Data type groupings are explained in Section 1.1. Obs.: observation(s).

2 Data pre-processing

The database we use for model fitting (summarized in Section 1) is based on multiple data quality assessments and pre-processing steps which are detailed in this section.

2.1 Extraction by data source type

Vital registration data The data on the number of births and fertility rates are obtained from the United Nations Statistics Division (Demographic Yearbook), national statistical offices and regional ones (e.g., Eurostat, OECD), United Nations Regional Commissions, other United Nations entities (e.g., UNFPA, UNICEF, WHO, World Bank), and complemented using international databases (the Human Fertility Database [2] and Human Fertility Collection [3]).

Birth and sibling histories in surveys The International Data Base [4], the Global Burden of Disease project [5], and public use microdata archives (e.g., DHS, MICS, IPUMS-International).

Censuses Reports on recent household births were used to compute age-specific estimates. Data from the summary birth histories in censuses were also used for children under five.

2.2 Adjustments for the incompleteness of birth registration

In the first preprocessing step, we computed the stochastic error for VR data. We compiled the number of births by women in a specific age group as the product of the observed fertility rates and the number of women in that age group. The resulting number of births, however, is smaller than the actual one as births are subject to under-reporting. We use the reported birth-reporting completeness (BRC) for country c in year t, denoted as $z_{c,t}$. We assume the reported BRC $z_{c,t}$ to be uniformly distributed. The g-th simulated BRC $z_{c,t}^{(g)}$ is obtained by:

$$z_{c,t}^{(g)} \sim U(z_{c,t} - \delta(z)_{c,t}, z_{c,t} + \delta(z)_{c,t}), \text{ for } g \in \{1, \cdots, G\},$$

where $\delta(z)_{c,t}$ is the standard error of reported BRC for country c in year t. It is assumed to increase linearly from 0.05 to 0.25 when the reported BRC $z_{c,t}$ is within the interval [60%, 95%]. When $z_{c,t}$ further increases to 100%, $\delta(z)_{c,t}$ is assumed to further decline linearly to zero. $\delta(z)_{c,t}$ is imputed as:

$$\begin{aligned}\delta(z)_{c,t} &= 0.25 - \frac{0.25-0.05}{0.95-0.6}(z_{c,t} - 0.6), \text{ if } 60\% \leq z_{c,t} < 95\%,\\ \delta(z)_{c,t} &= 0.05 - \frac{0.05}{1-0.95}(z_{c,t} - 0.95), \text{ if } z_{c,t} \geq 95\%.\end{aligned}$$

It is worth noting that the above assumptions made in simulating BRC are largely based on expert opinions. When additional information is available on how $z_{c,t}$ distributes, the simulation steps can be updated accordingly.

The g-th simulated number of under-reported births $B_{c,t}^{\text{under}(g)}$ is calculated as the product of the number of births reported $B_{c,t}^{\text{report}(g)}$ and the difference between 1 and the g-th simulated BRC $z_{c,t}^{(g)}$:

$$B_{c,t}^{\text{under}(g)} = B_{c,t}^{\text{report}(g)}\left(1 - z_{c,t}^{(g)}\right), \text{ for } g \in \{1, \cdots, G\}.$$

The g-th simulated total number of births $B_{c,t}^{(g)}$ is the sum of reported births and g-th simulated under-reported births. Uncertainty in the estimates is included by assuming that the simulated number of births has a Poisson distribution:

$$B_{c,t}^{(g)} \sim \text{Poisson}\left(B_{c,t}^{\text{report}(g)} + B_{c,t}^{\text{under}(g)}\right), \text{ for } g \in \{1, \cdots, G\}.$$

Compute stochastic error At this point, the g-th simulated number of total births $B_{c,t}^{(g)}$ is divided by the number of women in the specific age group a, denoted as $N_{a,c,t}^{\text{female}}$ to obtain the g-th simulated ASFR of the age group, $F_{a,c,t}^{(g)}$:

$$F_{a,c,t}^{(g)} = B_{c,t}^{(g)} / N_{a,c,t}^{\text{female}}, \text{ for } g \in \{1, \cdots, G\}.$$

The stochastic error is the standard deviation of the simulated $F_{a,c,t}^{(g)}$:

$$\sigma_{c,t} = \sqrt{\frac{\sum_{g=1}^{G}(F_{a,c,t}^{(g)} - \bar{F}_{a,c,t})^2}{G-1}}, \text{ where } \bar{F}_{a,c,t} = \frac{\sum_{g=1}^{G} F_{a,c,t}^{(g)}}{G}.$$

Compute sampling error The second preprocessing step entailed the compilation of sampling errors for non-VR data. In this case, we calculated the sampling errors from microdata sets whenever available. If the sampling errors were missing, we imputed them as the median of the sampling errors within each combination of age group and the period before the survey ("recall lag") for all available observations with sampling errors from microdata sets. To calculate the sampling errors, we generated simulated ASFRs from a normal distribution, with the mean at the observed rates and the standard deviation at the sampling error calculated from the microdata. We finally obtained the sampling error for non-VR data as the computed standard deviation of the simulated fertility rates on the logit scale.

2.3 Exclusion criteria

Before fitting the observations to the Bayesian model, the country analysts reviewed the data sources and data points available. Some of the data points were excluded due to concerns about data quality and completeness. We used the following exclusion criteria to ensure data quality used in our model:

- Exclude ASFR data with outlyingly high values using age-specific cutoffs. Specifically, we only keep ASFR data:
  - Below 0.03 for age group 10–14;
  - Below 0.25 for age group 15–19;
- For VR data, exclude those with low completeness of birth reporting below 60% [6];
- For survey data, exclude data points beyond 35 years before the survey.

3 Methods for age-specific fertility rate 10–19

We provide a full model specification in this web appendix. The modeling procedures to estimate the ASFR for girls aged 10–14 are explained in Section 3.2 and Section 3.4. The modeling procedures to estimate the ASFR for girls and adolescent females aged 15–19 are described in Section 3.1 and Section 3.3. For simplicity, we omit the index for age group, and all the notations in Section 3.1–Section 3.4 are age-specific.

The outcome of interest is $1/(1+\exp\{-\Psi_{c,t}\})$, ASFR in the country c in year t, where $\Psi_{c,t}$ is the logit of the ASFR. For the country and year indexes, $c \in \{1,\cdots,k\}$ with $k = 201$ referring to the total number of countries, and $t \in \{1,\cdots,j\}$ with $j = 74$ indicating the individual years from 1950 ($t = 1$) to the year 2023 ($t = j$). Throughout the report, $\mathscr{N}(\mu,\sigma^2)$ refers to a normal distribution with mean μ and variance σ^2 and $\mathscr{U}(a,b)$ refers to a continuous uniform distribution with lower and upper bounds at a and b respectively.

The complete data process and model overview are in the flowchart Figure 4. In particular, Blocks I and II of the flowchart indicates a three-step modeling procedure. This is much improved and straightforward than the Global Burden of Diseases Study (GBD) 2021 ASFR estimation process which involved a six-step modeling process:

1. estimate ASFR for ages 20–24;
2. deriving ASFR for ages 15–19 based on the 20–24 group;
3. adjusting data sources using outputs from previous steps;
4. re-estimating ASFR for ages 20–24 based on adjusted data;
5. recalculating ASFR for ages 15–19; and
6. estimating ASFR for ages 10–14 based on the revised ASFR for ages 15–19

Additionally, the age groups 15–19 and 20–24 have similar data availability and quality across countries and time periods; therefore, there is no strong reasoning based on data availability and quality for inclusion of the steps related to the relationship of 15–19 and 20–24.

The detailed explanations of the model and the posterior distribution are in the rest of this section.

3.1 Data model for age group 15–19

Let z_i to be the ith observed ASFR and $y_i = \text{logit}(z_i)$. We have the model:

$$y_i = \Psi_{c[i],t[i]} + \varepsilon_i, \text{ for } i \in \{1,\cdots,n\}, \tag{1}$$

$$\varepsilon_i|\omega_{s[i]} \sim \mathscr{N}(0, u_i^2 + \omega_{s[i]}^2), \text{ independent for } i \in \{1,\cdots,n\}, \tag{2}$$

$$\tau_{\omega,s} = \omega_s^{-2} \overset{\text{i.i.d.}}{\sim} \mathscr{PC}(w, 0.01), \text{ for } s \in \{1,\cdots,m\}. \tag{3}$$

For a certain age group, the observations of ASFR are indexed by $i \in \{1,\cdots,n\}$. y_i denotes the ith logit of observed ASFR in country $c[i]$ in year $t[i]$ and is modeled on the logit scale. $1/(1+\exp\{-\Psi_{c,t}\})$ is the outcome of interest, the true ASFR in country c in year t. ε_i is the error term for y_i.

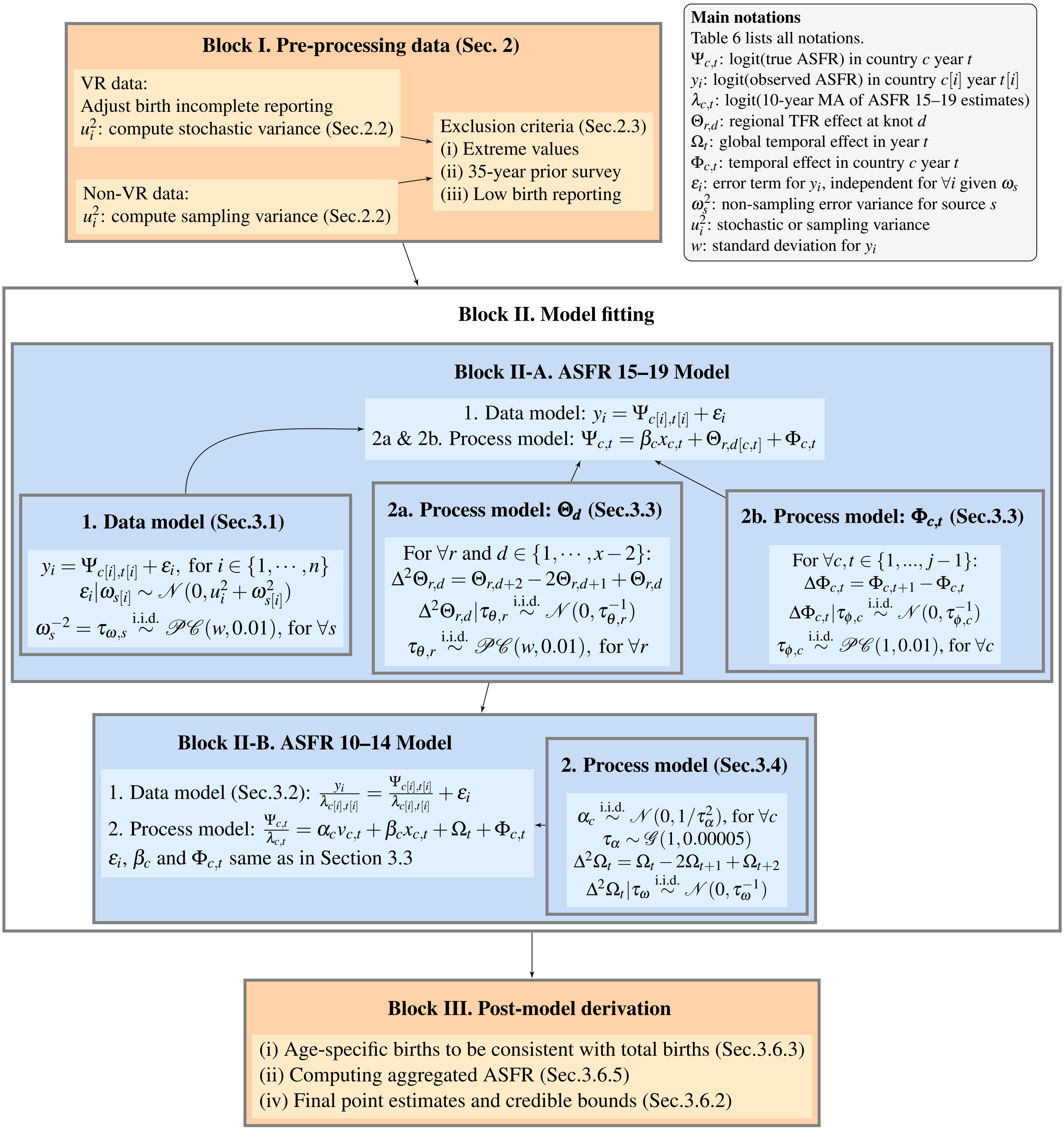

Figure 4: **Data and model process overview.** This flow chart summarizes the major steps of pre-precessing data (Block I), the Bayesian modelling steps (Block II), where $\Psi_{c,t}$ refers to the true age-specific fertility rate on the logit-scale, and the post-model derivation (Block III).

The error term ε_i follows a normal distribution as shown in Equation 2. The variance is the sum of two parts: (i) known stochastic/sampling error variance u_i^2, (ii) unknown non-sampling error variance $\omega_{s[i]}^2$ that differs by data source type s. The sampling variance is pre-calculated using the Jackknife resampling method by the R-package **demogsurv**[4] to account for the uncertainty due to the sample design of the surveys. We assume the non-sampling error for VR/SRS data is zero (i.e., $s = m$ refers to data source type VR/SRS). For data from non-VR/SRS source types, we estimate non-sampling error variances that vary across data source types ($s \in \{1, \cdots, m\}$ refer to the data in m source types). w is the standard deviation of y_i. We assign Penalized Complexity (PC) priors for the precision parameter $\tau_{\omega,s}$ [9].

The Bayesian estimates are pooled towards informative observations and are less influenced by weakly informative observations. Retrospective estimates based on surveys and recent household deaths could be affected by differential underreporting of births in boys or girls. However, comparisons of birth histories in surveys with prospective demographic surveillance did not find that data quality markers were associated with the sex of the child [10].

PC priors are default priors assigned to precision parameters used in the R-package `INLA` [11], which we used for Bayesian inference (refer to Section 3.5, paragraph Statistical computation). The main advantages of PC priors are that they are invariant to reparameterizations and have excellent robustness properties according to Simpson and colleagues [9].

3.2 Data model for age group 10–14

Instead of modeling y_i directly, we model the ratio of logit observed ASFR 10–14 to logit of ASFR median estimates 15–19. Let $\lambda_{c[i],t[i]}$ be the model estimate of ASFR from the age group 15–19 on the logit scale (obtained in Section 3.3). The data model for estimating ASFR from the age group 10–14 is:

$$\frac{y_i}{\lambda_{c[i],t[i]}} = \frac{\Psi_{c[i],t[i]}}{\lambda_{c[i],t[i]}} + \varepsilon_i, \text{ for } i \in \{1, \cdots, n\}. \tag{4}$$

Modeling $\frac{y_i}{\lambda_{c[i],t[i]}}$ instead of y_i assists backward projection to 1950 in countries without data in the early period. By doing so, we assume the following:

- Country-specific pattern of ASFR 10–14 is similar to ASFR 15–19 when no data is available for ASFR 10–14 to inform the model, following the assumption made in previous study [12]. Figure 5 shows a positive correlation between ASFR 10–14 and 15–19 across data types and across regions.
- 10-year moving average (MA): to smooth out the year-by-year natural fluctuation while maintaining the levels and trends of the time series. Figure 6 compares between annual time series and 10-year MA for one example country.

ε_i has the same model specifications as described in Section 3.1

3.3 Process model for ASFR 15–19

The process model for $\Psi_{c,t}$, the ASFR for age group 15–19 on logit scale, is defined as:

$$\Psi_{c,t} = \beta_c x_{c,t} + \Theta_{r,d[c,t]} + \Phi_{c,t}, \tag{5}$$

where

- $x_{c,t}$: proportion of female education attainment for country c in year t on log scale;
- β_c: country-specific coefficient for the female education attainment effect;
- $\Theta_{r,d[c,t]}$: regional effect from the total fertility rate (TFR) for country c in year t (more explanations in the next paragraph);
- $\Phi_{c,t}$: year-by-year fluctuation around the global relation, capturing the intra-country temporal correlation between country-specific observations.

[4] Available at: `https://github.com/mrc-ide/demogsurv`. Accessed on July 27th, 2020.

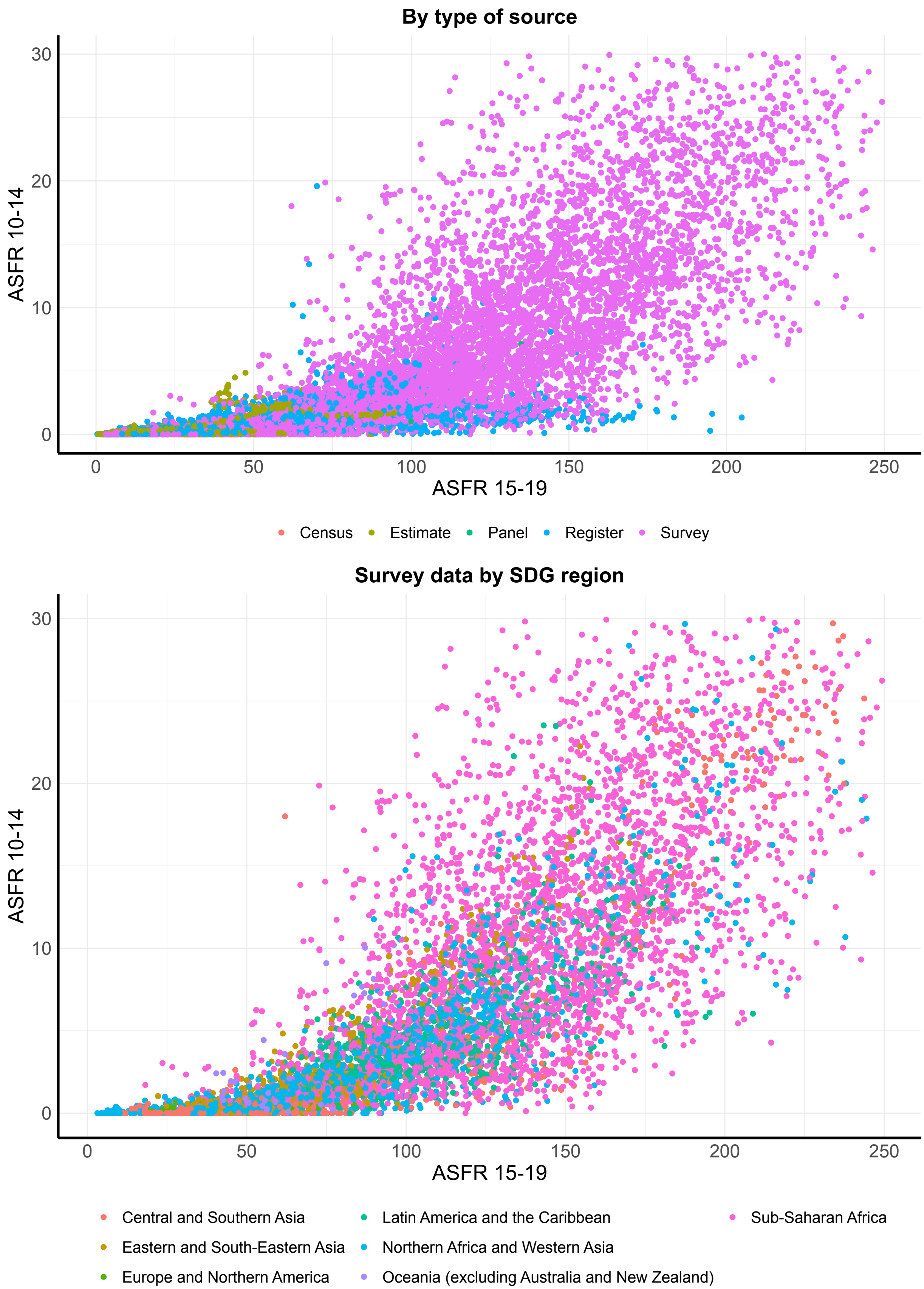

Figure 5: **Scatter plots between ASFR 10–14 and 15–19 observations, by data type (top) and by region (bottom).**

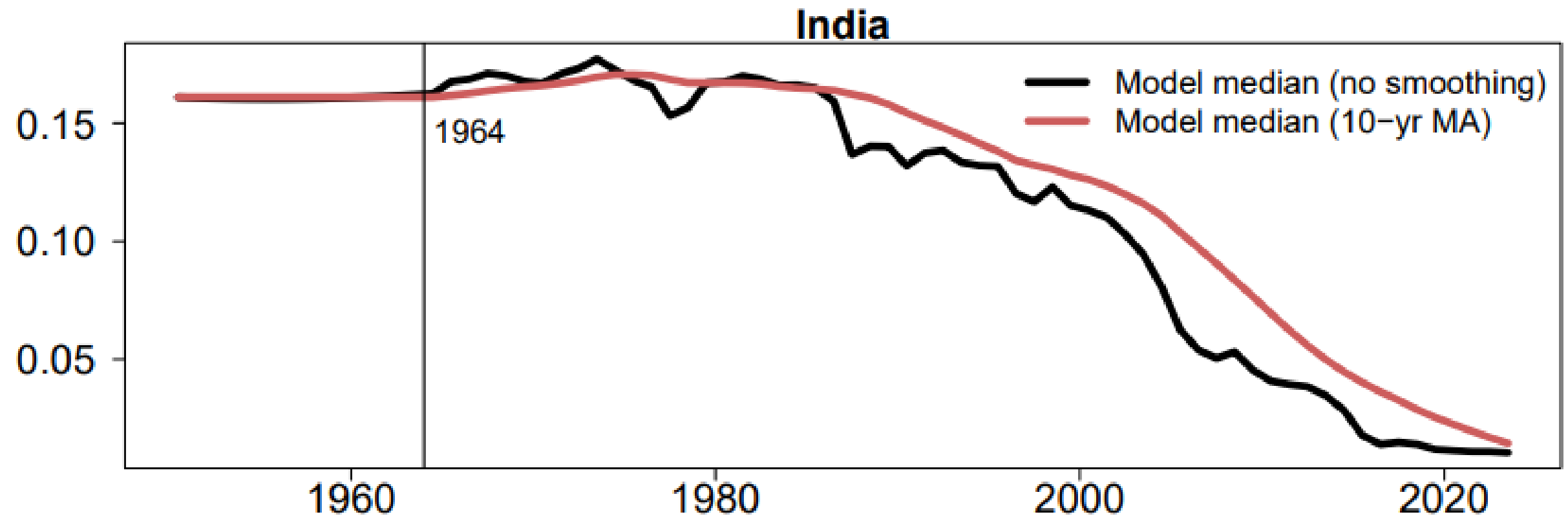

Figure 6: **Illustration of $\lambda_{c,t}$ as input data for model 10–14 for one country.** Black curve: median estimates of ASFR 15–19 without smoothing. Red curve: 10-year moving average of the median estimates of ASFR 15–19 as input data for modeling ASFR 10–14, corresponding to $\lambda_{c,t}$ in Equation 4.

β_c is the country-level fixed effect of female education attainment. It follows a hierarchical normal distribution with mean at zero and a global precision parameter:

$$\beta_c|\tau_\beta \overset{\text{i.i.d.}}{\sim} \mathscr{N}(0, 1/\tau_\beta), \text{ for } c \in \{1, \cdots, k\}. \tag{6}$$

We assign non-informative priors to the global precision parameter τ_β:

$$\tau_\beta \sim \mathscr{G}(1, 0.00005). \tag{7}$$

$\Theta_{r,d[c,t]}$ models the regional non-linear relation between $\Psi_{c,t}$ and the TFR in country c, year t. Specifically, let $v_{c,t}$ denote the log of TFR for country c year t, taken from the UN WPP 2024 estimates [1]. We define a grid of values κ_d for $d \in \{1, \cdots, x\}$. $x = 150$ is the number of grid points where $\Theta_{r,d}$ is evaluated. Let $\kappa_1 = \log(0.7)$, the minimum $v_{c,t}$, corresponding to 0.7 birth per 1,000 population on the log scale. We set $\kappa_x = \log(8.1)$ as the 99.5th percentile of $v_{c,t}$ across all country-years with data. We match each $v_{c,t}$ to the κ_d with the smallest absolute difference from $v_{c,t}$, denoting the dth index for country-year c, t as $d[c,t]$. To model the relation between $\Theta_{r,d}$ and κ_d, we use a second-order random walk (RW2) process. We assume $\Theta_{r,d}$ is constant outside the range of κ_1 and κ_x across all c and t. In particular,

$$\Delta^2\Theta_{r,d} = \Theta_{r,d+2} - 2\Theta_{r,d+1} + \Theta_{r,d}, \text{ for } r \in \{1, \cdots, p\} \text{ and } d \in \{1, \cdots, x-2\} \tag{8}$$

$$\Delta^2\Theta_{r,d}|\tau_{\theta,r} \overset{\text{i.i.d.}}{\sim} \mathscr{N}(0, \tau_{\theta,r}^{-1}), \text{ for } r \in \{1, \cdots, p\} \text{ and } d \in \{1, \cdots, x-2\}. \tag{9}$$

We assign a Penalized Complex (PC) prior to the regional precision parameter $\tau_{\theta,r}$:

$$\tau_{\theta,r} \overset{\text{i.i.d.}}{\sim} \mathscr{PC}(w, 0.01), \text{ for } r \in \{1, \cdots, p\}. \tag{10}$$

where w is the standard deviation of y_i. The PC prior is a vague prior. The paper by Simpson et al. [9] documented the PC prior specification in detail. Figure 7 illustrates the RW2 model results for $\Theta_{r,d}$, the regional TFR effects on ASFR, for age group 15–19.

$\Phi_{c,t}$ models the country-specific temporal effects. We use a first-order random walk (RW1) model for $\Phi_{c,t}$ and assign a PC prior to country-specific precision parameter $\tau_{\phi,c}$:

$$\Delta\Phi_{c,t} = \Phi_{c,t+1} - \Phi_{c,t}, \text{ for } c \in \{1, \cdots, k\}, t \in \{1, ..., j-1\} \tag{11}$$

$$\Delta\Phi_{c,t}|\tau_{\phi,c} \overset{\text{i.i.d.}}{\sim} \mathscr{N}(0, \tau_{\phi,c}^{-1}), \text{ for } c \in \{1, \cdots, k\}, t \in \{1, ..., j-1\}, \tag{12}$$

$$\tau_{\phi,c} \overset{\text{i.i.d.}}{\sim} \mathscr{PC}(1, 0.01), \text{ for } c \in \{1, \cdots, k\}. \tag{13}$$

The sum-to-zero constraint is imposed on $\Phi_{c,t}$ so that the precision matrix is full-rank:

$$\sum_{t=1}^{j} \Phi_{c,t} = 0, \text{ for } c \in \{1, \cdots, k\}. \tag{14}$$

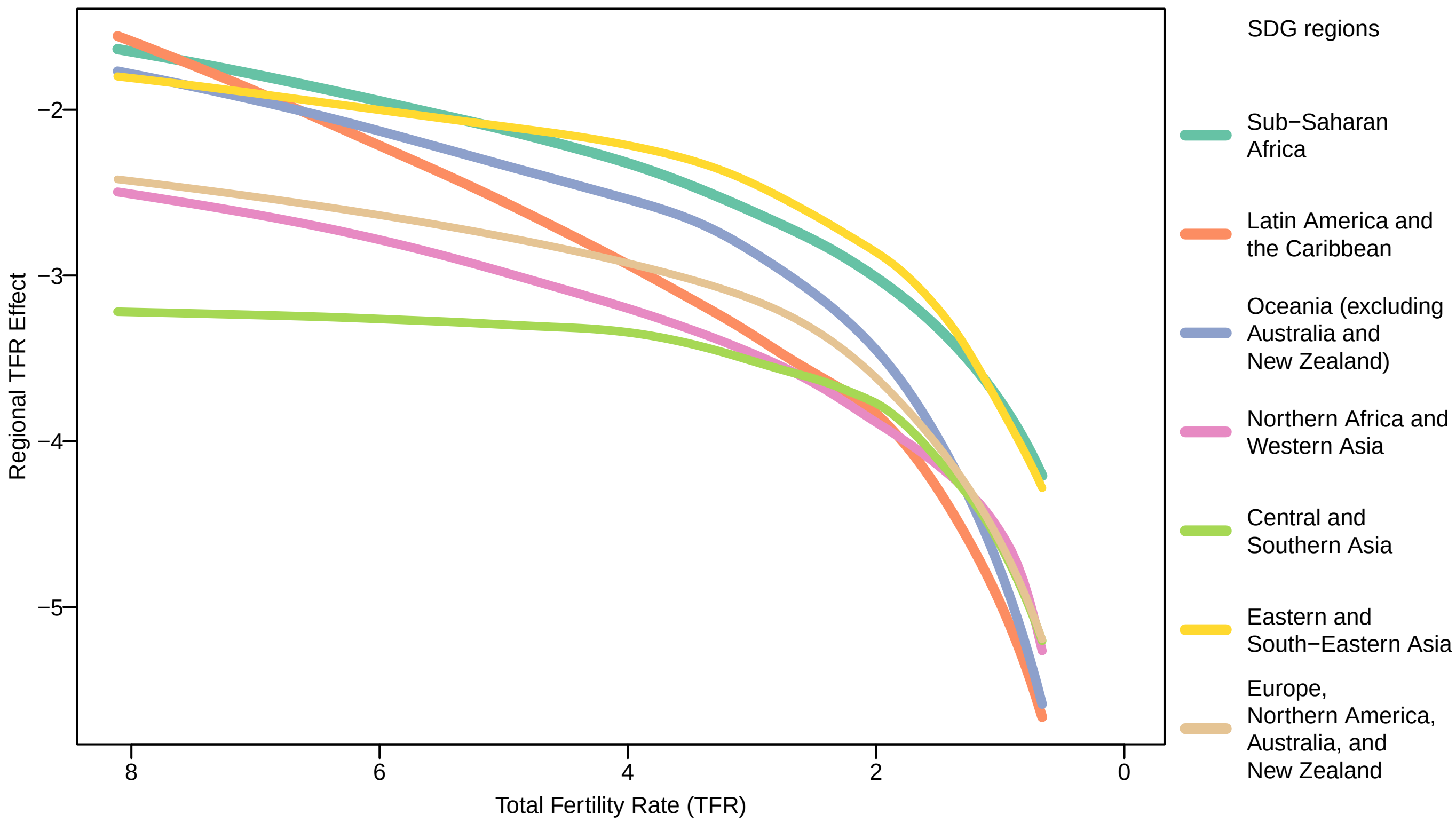

Figure 7: **Regional relation between TFR and ASFR 15–19.** The model median estimates for the TFR effect from different regions are differentiated by colors. TFR: total fertility rate. ASFR: age-specific fertility rate.

3.4 Process model for ASFR 10–14

The process model for logit of ASFR for age group $a =$10–14, denoted as $\Psi_{c,t}$, is defined as:

$$\frac{\Psi_{c,t}}{\lambda_{c,t}} = \alpha_c v_{c,t} + \beta_c x_{c,t} + \Omega_t + \Phi_{c,t}, \tag{15}$$

where

- $\lambda_{c,t}$: logit of the 10-year moving average (MA) of ASFR 15–19 medians obtained in Section 3.3 (more explanations below);
- $v_{c,t}$: TFR for country c in year t on log scale;
- α_c: country-specific coefficient for the TFR effect;
- $x_{c,t}$: proportion of female education attainment for country c in year t on log scale;
- β_c: country-specific coefficient for the female education attainment effect;
- Ω_t: global time trend in year t;
- $\Phi_{c,t}$: year-by-year fluctuation around the global relation, capturing the intra-country temporal correlation between country-specific observations.

The country-specific parameters β_c and $\Phi_{c,t}$ are modeled the same as in Section 3.3. The country-specific regression coefficients α_c follow a hierarchical distribution with a non-informative prior assigned to its global precision parameter τ_α:

$$\alpha_c|\tau_\alpha \stackrel{\text{i.i.d.}}{\sim} \mathcal{N}(0, 1/\tau_\alpha^2), \text{ for } c \in \{1, \cdots, k\}, \tag{16}$$

$$\tau_\alpha \sim \mathcal{G}(1, 0.00005). \tag{17}$$

Ω_t models the global non-linear relation between ASFR and time with a random walk model of order 2 (RW2):

$$\Delta^2\Omega_t = \Omega_t - 2\Omega_{t+1} + \Omega_{t+2}, \tag{18}$$
$$\Delta^2\Omega_t|\tau_\omega \overset{\text{i.i.d.}}{\sim} \mathcal{N}(0, \tau_\omega^{-1}), \text{ for } t \in \{1, \cdots, j-2\}, \tag{19}$$
$$\tau_\omega \sim \mathscr{PC}(1, 0.01). \tag{20}$$

3.5 Model summary

Notations Table 6 summarizes the notation and indexes used in Section 3.1, 3.2, 3.3, and 3.4.

Data model 15–19

$$\begin{aligned} y_i &= \Psi_{c[i],t[i]} + \varepsilon_i, \text{ for } i \in \{1, \cdots, n\}, \\ \varepsilon_i|\omega_{s[i]} &\sim \mathcal{N}(0, u_i^2 + \omega_{s[i]}^2), \text{ independent for } i \in \{1, \cdots, n\}. \end{aligned}$$

Process model 15–19

$$\begin{aligned} \Psi_{c,t} &= \beta_c x_{c,t} + \Theta_{r,d[c,t]} + \Phi_{c,t}, \text{ for } c \in \{1, \cdots, k\}, t \in \{1, \cdots, j\}, \\ \beta_c|\tau_\beta &\overset{\text{i.i.d.}}{\sim} \mathcal{N}(0, 1/\tau_\beta), \text{ for } c \in \{1, \cdots, k\}, \\ \Delta^2\Theta_{r,d} &= \Theta_{r,d+2} - 2\Theta_{r,d+1} + \Theta_{r,d}, \text{ for } r \in \{1, \cdots, p\} \text{ and } d \in \{1, \cdots, x-2\}, \\ \Delta^2\Theta_{r,d}|\tau_{\theta,r} &\overset{\text{i.i.d.}}{\sim} \mathcal{N}(0, \tau_{\theta,r}^{-1}), \text{ for } r \in \{1, \cdots, p\} \text{ and } d \in \{1, \cdots, x-2\}, \\ \Delta\Phi_{c,t} &= \Phi_{c,t+1} - \Phi_{c,t}, \text{ for } c \in \{1, \cdots, k\}, t \in \{1, \cdots, j-1\}, \\ \Delta\Phi_{c,t}|\tau_{\phi,c} &\overset{\text{i.i.d.}}{\sim} \mathcal{N}(0, \tau_{\phi,c}^{-1}), \text{ for } c \in \{1, \cdots, k\}, t \in \{1, \cdots, j-1\}. \end{aligned}$$

Prior distributions 15–19 Non-informative priors are assigned to hyper-parameters:

$$\begin{aligned} \tau_{\omega,s} = \omega_s^{-2} &\overset{\text{i.i.d.}}{\sim} \mathscr{PC}(w, 0.01), \text{ for } s \in \{1, \cdots, m\}, \\ \tau_\beta &\sim \mathscr{G}(1, 0.00005), \\ \tau_{\theta,r} &\overset{\text{i.i.d.}}{\sim} \mathscr{PC}(w, 0.01), \text{ for } r \in \{1, \cdots, p\}, \\ \tau_{\phi,c} &\overset{\text{i.i.d.}}{\sim} \mathscr{PC}(1, 0.01), \text{ for } c \in \{1, \cdots, k\}. \end{aligned}$$

Data model 10–14

$$\begin{aligned} \frac{y_i}{\lambda_{c[i],t[i]}} &= \frac{\Psi_{c[i],t[i]}}{\lambda_{c[i],t[i]}} + \varepsilon_i, \text{ for } i \in \{1, \cdots, n\}, \\ \varepsilon_i|\omega_{s[i]} &\sim \mathcal{N}(0, u_i^2 + \omega_{s[i]}^2), \text{ independent for } i \in \{1, \cdots, n\}, \\ \tau_{\omega,s} = \omega_s^{-2} &\overset{\text{i.i.d.}}{\sim} \mathscr{PC}(w, 0.01), \text{ for } s \in \{1, \cdots, m\}. \end{aligned}$$

Symbol	Description
	Indexes
i	Indicator for observations across all country-years, $i \in \{1, \cdots, n\}$.
r	Indicator for region, $r \in \{1, \cdots, p\}$, where $p = 7$.
c	Indicator for country, $c \in \{1, \cdots, k\}$, where $k = 201$. $c[i]$ refers to the country index of observation i.
t	Indicator for year, $t \in \{1, \cdots, j\}$, where $j = 74$. $t = 1$ corresponds to the year 1950 and $t = j$ to the year 2023. $t[i]$ refers to the year index of observation i.
s	Indicator for data source type, $s \in \{1, \cdots, m\}$, where $m = 5$. Refer to Table 17 and 18 for data sources.
d	Indicator for the d-th unique value of the total fertility rate (TFR) κ_d and corresponding regional TFR effect $\Theta_{r,d[c,t]}$. $d[c,t]$ refers to the index based on TFR in country-year c, t. More explanations are in the row of κ_d.
	Known quantities
z_i	ith observed age-specific fertility rate (ASFR) for country $c[i]$ in year $t[i]$ with data source type $s[i]$.
y_i	$y_i = \text{logit}(z_i)$.
u_i^2	The ith sampling error variance for y_i.
w	The standard deviation of y_i.
$Q_{c,t}$	The TFR median estimate for country c in year t from the UN WPP [1].
$v_{c,t}$	$v_{c,t} = \log(Q_{c,t})$
$x_{c,t}$	The proportion of female education attainment for country c in year t on log scale [13].
κ_d	Grid of $v_{c,t}$ where $\Theta_{r,d}$ is evaluated for $d \in \{1, \cdots, x\}$, and $x = 150$. $\kappa_1 = \log(0.7/1000)$, i.e. log of 0.7 birth per 1000 population. $\kappa_x = \log(8.1/1000)$ is the 99.5th percentile of $v_{c,t}$ across all country-years with data.
$\lambda_{c,t}$	logit of the 10-year moving average (MA) of ASFR 15–19 medians obtained in Section 3.3
	Unknown parameters
$\Psi_{c,t}$	The true logit of ASFR for country c, year t.
Ω_t	global time trend in year t.
$\Theta_{r,d[c,t]}$	The regional TFR effect evaluated at κ_d for country c, year t.
$\Phi_{c,t}$	The year-by-year within country discrepancy between $\Psi_{c,t}$ and $\Theta_{r,d}$.
α_c	country-specific coefficient for the TFR effect.
β_c	country-specific coefficient for the female education attainment effect.
$\tau_{\omega,s}$	The precision parameter for non-sampling error variance with data source type s.
$\tau_{\theta,r}$	The precision parameter for the second-order increment $\Delta^2\Theta_{r,d}$.
$\tau_{\phi,c}$	The country-specific precision parameter for the first-order increment $\Delta\Phi_{c,t}$ for country c.

Table 6: **Notation summary.**

Process model 10–14

$$
\begin{aligned}
\frac{\Psi_{c,t}}{\lambda_{c,t}} &= \alpha_c v_{c,t} + \beta_c x_{c,t} + \Omega_t + \Phi_{c,t}, \text{ for } c \in \{1,\cdots,k\}, t \in \{1,\cdots,j\},\\
\Delta^2\Omega_t &= \Omega_t - 2\Omega_{t+1} + \Omega_{t+2}, \text{ for } t \in \{1,\cdots,j-2\},\\
\Delta^2\Omega_t|\tau_\omega &\overset{\text{i.i.d.}}{\sim} \mathcal{N}(0,\tau_\omega^{-1}), \text{ for } t \in \{1,\cdots,j-2\},\\
\Delta\Phi_{c,t} &= \Phi_{c,t+1} - \Phi_{c,t}, \text{ for } c \in \{1,\cdots,k\}, t \in \{1,...,j-1\},\\
\Delta\Phi_{c,t}|\tau_{\phi,c} &\overset{\text{i.i.d.}}{\sim} \mathcal{N}(0,\tau_{\phi,c}^{-1}), \text{ for } c \in \{1,\cdots,k\}, t \in \{1,...,j-1\},\\
\alpha_c|\tau_\alpha &\overset{\text{i.i.d.}}{\sim} \mathcal{N}(0,1/\tau_\alpha^2), \text{ for } c \in \{1,\cdots,k\},\\
\beta_c|\tau_\beta &\overset{\text{i.i.d.}}{\sim} \mathcal{N}(0,1/\tau_\beta), \text{ for } c \in \{1,\cdots,k\}.
\end{aligned}
$$

Prior distributions 10–14 Non-informative priors are assigned to hyper-parameters:

$$
\begin{aligned}
\tau_{\phi,c} &\overset{\text{i.i.d.}}{\sim} \mathscr{PC}(1,0.01), \text{ for } c \in \{1,\cdots,k\},\\
\tau_\omega &\sim \mathscr{PC}(1,0.01),\\
\tau_\alpha &\sim \mathscr{G}(1,0.00005),\\
\tau_\beta &\sim \mathscr{G}(1,0.00005).
\end{aligned}
$$

Statistical computation We use the Integrated Nested Laplace Approximation (INLA) for Bayesian inference [14]. We use the `R`-package `R-INLA` [11] to implement the INLA estimation procedure. The statistical source code is available upon request.

3.6 Computation of final results

Throughout the project, we presented our results in the format of "point estimate [lower bound; upper bound]". E.g. the ASFR and births are presented as $Q^{\text{P.E.}}_{a,c,t}[Q^{\text{L}}_{a,c,t};Q^{\text{U}}_{a,c,t}]$ and $B^{\text{P.E.}}_{a,c,t}[B^{\text{L}}_{a,c,t};B^{\text{U}}_{a,c,t}]$ respectively. This section will explain how we derived each component in the result.

3.6.1 Rounding

We kept three significant figures for all reported estimates and uncertainty intervals. We round the number of births by mothers from different age groups for each country-year to its nearest integer.

3.6.2 Constructing uncertainty intervals and point estimates

The uncertainties from TFR were incorporated into the final uncertainties of the ASFR. For the g-th trajectory of the ASFR for age 15–19 on the logit scale $\Psi^{(g)}_{c,t}$, with $g \in \{1,...,G\}$, it is obtained as:

$$
\Psi^{(g)}_{c,t} = \beta^{(g)}_c x_{c,t} + \Theta^{(g)}_{r,d[c,t]} + \Phi^{(g)}_{c,t},
$$

where $\beta^{(g)}_c$ and $\Phi^{(g)}_{c,t}$ are the g-th trajectories of the model parameters β_c and $\Phi_{c,t}$, obtained directly from the Bayesian model itself as posterior samples. $\Theta^{(g)}_{r,d[c,t]}$ is the g-th trajectory of the TFR regional effect based on the g-th trajectory of the TFR $Q^{(g)}_{c,t}$. The TFR trajectories are from the UN WPP [1].

For the g-th trajectory of the ASFR for age 10–14 on the logit scale $\Psi^{(g)}_{c,t}$, with $g \in \{1,...,G\}$, it is obtained as:

$$
\Psi^{(g)}_{c,t} = \left(\alpha^{(g)}_c v^{(g)}_{c,t} + \beta^{(g)}_c x_{c,t} + \Omega^{(g)}_t + \Phi^{(g)}_{c,t}\right)\lambda^{(g)}_{c,t},
$$

where $\alpha_c^{(g)}$, $\beta_c^{(g)}$, $\Omega_t^{(g)}$ and $\Phi_{c,t}^{(g)}$ are the g-th trajectories of the model parameters α_c, β_c, Ω_t, and $\Phi_{c,t}$, obtained directly from the Bayesian model itself as posterior samples. $v_{c,t}^{(g)}$ is the g-th trajectory of the TFR on the log scale $\log(Q_{c,t}^{(g)})$. The TFR trajectories are from the UN WPP [1]. Whereas $\lambda_{c,t}^{(g)}$ is the g-th trajectory of the 10-year moving avarage of logit ASFR 15–19 obtained above.

All the indicators in this section are country-year-specific. Hence, we omit the indexes c and t for country and year for simplicity. The 95% uncertainty intervals for $\Psi(\text{full})_a$, $Q(\text{full})_a$, and $B(\text{full})_a$, denoted as $[*^L; *^U]$, are the 2.5th and 97.5th percentiles of the corresponding posterior samples:

$$\begin{aligned} *^L &= \text{percentile}_{2.5\%}\left\{{}^{(1)}*, \ldots, {}^{(G)}*\right\}, \\ *^U &= \text{percentile}_{97.5\%}\left\{{}^{(1)}*, \ldots, {}^{(G)}*\right\}. \end{aligned}$$

where $*$ can be substituted with $\Psi(\text{full})_a$, $Q(\text{full})_a$, and $B(\text{full})_a$.

The point estimates $\exp\{\Psi(\text{full})\}_a^{\text{P.E.}}$ are combined with the point estimates of total fertility rate $Q(\text{full})_a^{\text{P.E.}}$ and the number of total births $B(\text{full})_a^{\text{P.E.}}$ (both were obtained from the UN WPP 2024 results [1], not part of the modelling process) to derive the point estimates of age-specific births $B(\text{full})_a^{\text{P.E.}}$.

To assess the uncertainty with respect to the point estimates, we calculated the coefficient of variance (CV). For the CV of the Bayesian model result from age group a in country c and year t, we have:

$$\text{CV}_{a,c,t} = \frac{Q_{a,c,t}^{\text{U}} - Q_{a,c,t}^{\text{L}}}{4Q_{a,c,t}^{\text{P.E.}}},$$

where four standard errors is assumed to fall between the lower bound $Q_{a,c,t}^{\text{L}}$ and upper bound $Q_{a,c,t}^{\text{U}}$ of the point estimate $Q_{a,c,t}^{\text{P.E.}}$.

3.6.3 Age-specific births to be consistent with total births

Due to rounding (Section 3.6.1) and the different data availability and quality between the ASFR and the TFR, the sum of the births by mothers from different age groups for a particular country-year $\sum_a B(\text{full})_a^{\text{P.E.}}$ may be slightly different than the total births for the same country-year $B(\text{full})^{\text{P.E.}}$, where total births computation is not part of the modelling process. When the inconsistency occurs for a certain country-year, we:

- compute the ratio of $B(\text{full})^{\text{P.E.}}$ to $\sum_a B(\text{full})_a^{\text{P.E.}}$
- apply the ratio to the trajectories of $B(\text{full})_a^{(\text{g})}$ for $g \in \{1, \ldots, G\}$.

This adjustment is typically small but ensures that $\sum_a B(\text{full})_a^{\text{P.E.}} = B(\text{full})^{\text{P.E.}}$ for all country-years. We adjust both the point estimates and the uncertainty intervals.

3.6.4 Annual rate of reduction

The annual rate of reduction (ARR) for country c during the period of $[t_1, t_2]$ is defined as

$$\text{ARR}_{c,[t_1,t_2]} = \frac{\log(\Psi_{t_2}/\Psi_{t_1})}{t_1 - t_2}, \text{ with } t_1 < t_2.$$

Ψ_{t_1} and Ψ_{t_2} refer to the model estimates for the ASFR in years t_1 and t_2 respectively.

For country-years with ASFR in ages 10–14 and 15–19 below 0.01 per 1000 females, we use 0.01 per 1,000 for that country-year when computing the ARR. If the maximum values of the posterior samples of ASFR in both years t_1 and t_2 are below 0.01 per 1,000, i.e. $\Psi_{t_1} < 10^{-5}$ and $\Psi_{t_2} < 10^{-5}$, then we assume the changes are largely stochastic rather than actual changes and we consider $\text{ARR}_{c,[t_1,t_2]} = 0$ for that period.

If the median ASFRs in both years t_1 and t_2 are below 0.1 per 1,000, i.e. $\Psi_{t_1} < 10^{-4}$ and $\Psi_{t_2} < 10^{-4}$, then we do not report the ARR results for that location-period and assume most changes are stochastic.

3.6.5 Computing aggregated results

The 201 countries or areas with total population larger than 90,000 by mid-2024 (according to the UN WPP 2024 population estimates [1]) were aggregated following the regional grouping in Table 12. The number of births by age group of the mother from region r for the a-th age group for year t, denoted by $BR_{a,r,t}$, was computed as:

$$BR_{a,r,t} = \sum_{c \in \{\text{region}[c]=r\}} B(\text{full})_{a,c,t}, \text{ for } r \in \{1, \cdots, p\}.$$

The ASFR from region r for the a-th age group for year t, denoted by $QR_{a,r,t}$, was computed as:

$$QR_{a,r,t} = QR(\text{full})_{r,t} \cdot \frac{BR_{a,r,t} \cdot 5}{BR(\text{full})_{r,t}}, \text{ for } r \in \{1, \cdots, p\},$$

where

$$BR(\text{full})_{r,t} = \sum_{c \in \{\text{region}[c]=r\}} B(\text{full})_{c,t}.$$

$QR(\text{full})_{r,t}$ is the aggregated TFR from region r in year t from the UN WPP 2024 results [1].

4 Validation exercise and results

We validate the model performance by leaving out data collected after a particular year, referred to as the "survey year", rather than randomly leaving out data. This validation approach has been used in various previous global health and population studies [15, 16, 17, 18, 19, 20, 21, 22, 23]. The left-out observations are roughly 20% of the total observations (due to the varying number of observations collected in each survey year, the left-out observations can be slightly above or below 20%). We call the left-out observations "testing dataset" and the remaining observations "training dataset".

For each left-out observation in the testing dataset, we generate a posterior predictive distribution based on the Bayesian model fittings for the training dataset. We compute the error as the difference between the left-out observation and the median of the posterior predictive distribution. We report the median errors of all the left-out observations and the median of the absolute errors. We also report the coverage of the 90% prediction intervals (PIs). The lower and upper bounds of the 90% PI for each left-out observation is the 5th and 95th percentiles of the posterior predictive distribution. To summarise the coverage, we compute the proportion of left-out observations that fall outside the 90% PIs in Table 7. Median errors are close to zero for left-out observations in both age groups. While the errors can be negative or positive and are usually distributed around zero (if the model is well calibrated, as shown in Table 7), the absolute errors can only be positive because it is the absolute values of the errors. Hence, the median absolute errors in all age groups are expected to be higher than the median errors. Coverage of 90% prediction intervals is slightly higher than expected at 96.2% for the age group 10–14 and 91.3% for the age group 15–19.

We also validate the model performance by comparing the model estimates based on the full and training datasets. We compute the error for each country-year as the difference between the median estimates based on the full dataset and those based on the training dataset. We report the median of these errors and absolute errors for all the country-years. We check the percentage of the country-years where the median estimates based on the full dataset fall outside the credible bounds of the estimates based on the training dataset. Table 8 shows the results for the comparison between estimates obtained based on the full dataset and estimates based on the training set. Median errors and median absolute errors were close to zero, and the proportion of updated estimates that fell outside the uncertainty intervals constructed based on the training set was mostly within the expected level (less than 5%). The exception is the median estimates based on the full run tend to be higher than the 90% credible interval (CI) based on the training database. This implies that the recent data show that the ASFR from age 15–19 declined faster than the historical experience based on the model assumptions.

Age group	10–14	15–19
Median error	0.000	-0.001
Median absolute error	0.001	0.013
% of left-out observations below 90% prediction interval	4.4	6.0
% of left-out observations above 90% prediction interval	1.4	2.7
**Expected proportions (%)**	**5.0**	**5.0**
# left out observations	3,385	3,795
% of left out to total observations	15.4	17.7
Survey year left out	2013	2012

Table 7: **Validation results for left-out observations by age group.** Errors are defined as the difference between a left-out observation and the posterior median of its predictive distribution. Survey year left out: left out all observations that were collected in or after the year.

Age group	10–14			15–19		
**Year**	**2000**	**2005**	**2010**	**2000**	**2005**	**2010**
Median error	0.000	0.000	0.000	0.000	0.000	0.000
Median absolute error	0.000	0.000	0.000	0.002	0.002	0.002
Below 90% uncertainty interval (%)	3.0	5.1	3.0	2.1	3.8	3.0
Above 90% uncertainty interval (%)	3.0	3.8	5.1	8.0	7.2	7.2
**Expected proportions (%)**	**≤5**	**≤5.0**	**≤5.0**	**≤5.0**	**≤5.0**	**≤5.0**

Table 8: **Summary of differences in ASFR estimates in observation years 2000, 2005, and 2010 based on the training and full data sets.** Errors are the differences between estimates based on the full dataset and the training set. The proportions refer to the proportions (%) of countries where the median ASFR estimates based on the full data set fall below or above their corresponding 90% uncertainty intervals based on the training dataset. The results are broken down by age groups and observation years.

5 Sensitivity analyses

In addition to the validation exercises (Section 4), we conducted sensitivity analyses in this section to test model assumptions. Essentially, two widely accepted criteria of Bayesian model assessment and comparison were used, namely Deviance Information Criterion (DIC) and Watanabe-Akaike Information Criterion (WAIC). In addition, we checked the cross-validation and predictive measures of fit using the Conditional Predictive Ordinates (CPO) to strengthen the model assessment and comparison.

5.1 Time series model setup

As presented in Section 3.3, an RW1 model is used to estimate the country-specific temporal effects $\Phi_{c,t}$. We tested another model with all other model assumptions remains unchanged, the only change is to use an autoregressive of order 1 (AR1) model for $\Phi_{c,t}$. That is,

$$\begin{aligned}\Phi_{c,t} &= \rho\Phi_{c,t-1} + \varepsilon_{c,t}, \text{ with } |\rho| < 1, \text{ for } c \in \{1, \cdots, k\}, t \in \{2, \cdots, j\} \\ \Phi_{c,t} &\sim \mathcal{N}(0, \frac{\sigma_\varepsilon^2}{1-\rho^2}), \text{ for } t = 1, \\ \varepsilon_{c,t}|\sigma_\varepsilon &\overset{\text{i.i.d.}}{\sim} \mathcal{N}(0, \sigma_\varepsilon^2).\end{aligned}$$

with weakly informative priors assigned to $1/\sigma_\varepsilon^2$ and ρ.

We compared the DIC, WAIC, and CPO for the two models in Table 9. A model with smaller DIC or WAIC should be preferred to one with larger DIC or WAIC. Whereas for CPO, a larger value indicates a model with better fit than one with a smaller CPO. Both DIC and CPO suggest the original model with RW1 time series setup for $\Phi_{c,t}$

is better than the alternative model with AR1 setup. Meanwhile, the CPO values from both models are very similar to each other.

Model	DIC	WAIC	CPO
Original (RW1)	-33,408	-32,173	129,62
Alternative (AR1)	-33,401	-32,204	129,53

Table 9: **Model comparison for time series setup.** CPO= $\sum_{i=1}^{n} \log(\text{cpo}_i)$.

5.2 ASFR 10–14 model setup

As explained in Section 3.4, the ASFR 10–14 estimation made use of the pattern of the ASFR 15–19 to assist in providing more plausible results.

To test this model assumption, we conducted model diagnostics and checks between two models:

- Original model for ASFR 10–14: as described in Section 3.4.
- Alternative model for ASFR 10–14: the same model as for ASFR 15–19 without using ASFR pattern from the neighbouring age group as described in Section 3.3.

We compared the DIC, WAIC, and the CPO for the two models in Table 10. All three criteria suggest the original model has better fits than the alternative model.

Model 10–14	DIC	WAIC	CPO
Original	-6,511	-4,045	919
Alternative	20,324	23,052	-13,175

Table 10: **Model comparison for time series setup.** CPO= $\sum_{i=1}^{n} \log(\text{cpo}_i)$. Original model for ASFR 10–14: as described in Section 3.4. Alternative model for ASFR 10–14: the same model as for ASFR 15–19 without using ASFR pattern from the neighbouring age group as described in Section 3.3.

In addition to the better model fit for the original model than the alternative model, there are two other reasons we chose the original model against the alternative model:

1. The original model provides narrower uncertainty bounds for country-periods without data than the alternative model: Figure 8 top.
2. The original model provides more realistic backward projections in countries known to have high teenage fertility rate than the alternative model: Figure 8 bottom.

6 Uncertainty assessments of model results

The uncertainty bound with respect to the estimated ASFR level can be wide due to: (i) lack of data in the early period, or (ii) close-to-zero estimates in the recent period. The coefficient of variance (CV) across all country-years are presented in Figure 9 for ages 10–14 and in Figure 10 for ages 15–19.

The CVs for ages 15–19 in all country-years of the model results are good as shown in Figure 10. The maximum CV is below 0.6.

The CVs for ages 10–14 have a wider spectrum than the older age group as shown in Figure 9. The countries are identified according to the country-specific mean CVs, denoted as $\overline{\text{CV}}_{a,c} = \sum_{t=1950}^{2023} \text{CV}_{a,c,t}/(2023-1950+1)$. The main reasons of causing the high CVs are:

i. No data;
ii. Limited data;
iii. Close-to-zero point estimates of ASFR;
iv. ASFR observations are weakly informative with large sampling errors;

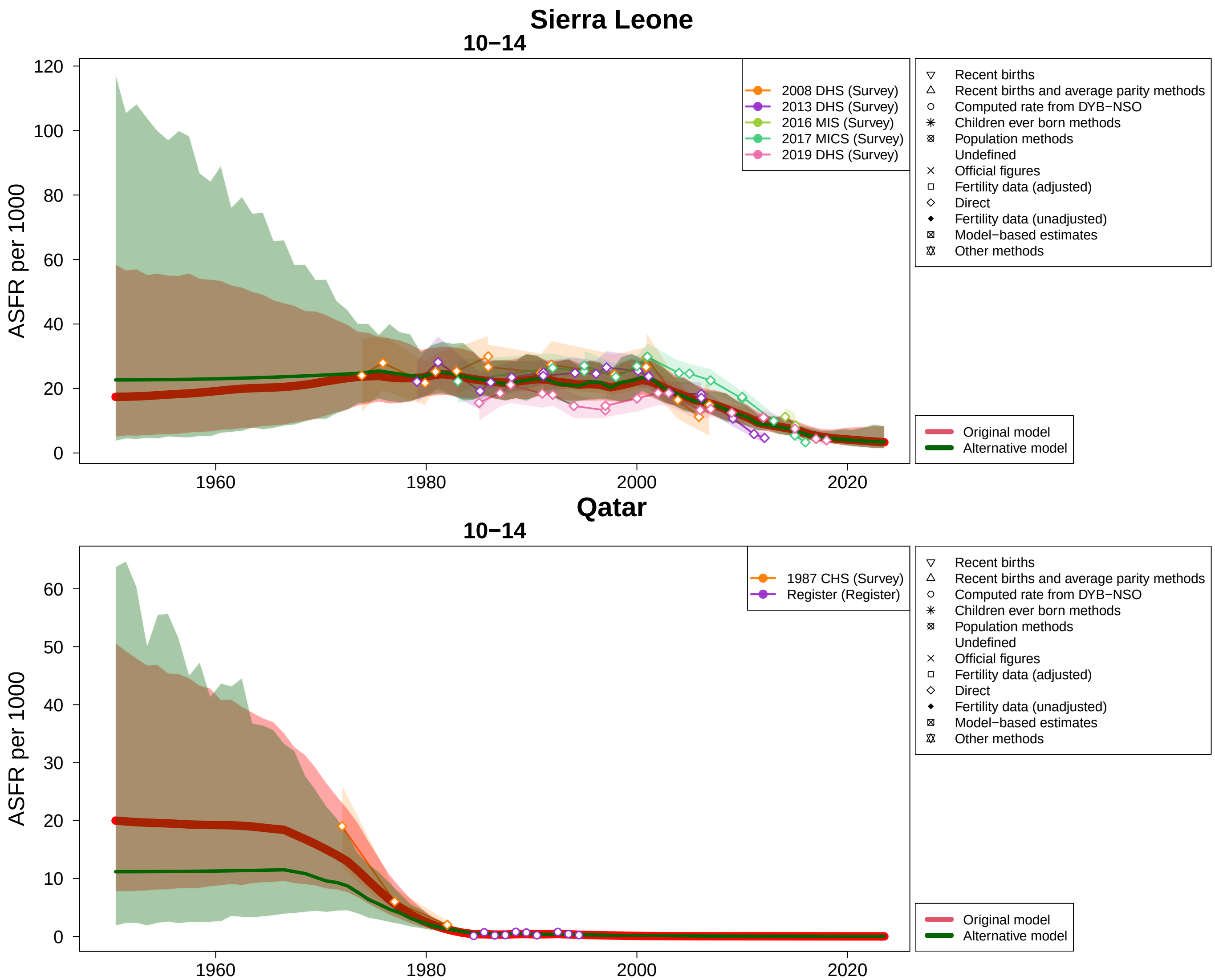

Figure 8: **ASFR 10–14 model fit comparison during 1950–2023.** Top: original model can provide narrower uncertainty bounds for country-periods without data. Bottom: original model provides more realistic backward projections in countries known to have high teenage fertility rate. Red curves and shades: median estimates and 95% uncertainty intervals from the original model (Section 3.4). Green curves and shades: median estimates and 95% uncertainty intervals from the alternative model (the same model as for ASFR 15–19 without using ASFR pattern from the neighbouring age group as described in Section 3.3). Dots with connection lines: input data series differentiated by colors. Shades around the data series: sampling errors (for non-VR data) or stochastic errors (for VR data).

The countries/areas (reason ID(s) of causing the high CVs are in the brackets after each country/area name) are:

- $\overline{\text{CV}}_{a,c} \in [20, +\infty]$: Dem. People's Republic of Korea (i, iii);
- $\overline{\text{CV}}_{a,c} \in [2, 20]$: Bhutan (i); China (i, iii); Cyprus (ii, iii); Georgia (ii, iii); Kuwait (ii, iii); Libya (iii); Mayotte (i); Saudi Arabia (i); Solomon Islands (i); United Arab Emirates (ii, iii); Vanuatu (i); Western Sahara (i);
- $\overline{\text{CV}}_{a,c} \in [1.5, 2]$: China, Macao SAR (iii); Curaçao (ii); Malaysia (ii, iii); Qatar (ii, iii); Serbia (ii, iii); Tonga (ii, iii);
- $\overline{\text{CV}}_{a,c} \in [1, 1.5]$: Aruba (ii); Azerbaijan (iii); Bahamas (ii); Bosnia and Herzegovina (ii, iii); Cabo Verde (ii); Equatorial Guinea (i); French Polynesia (iv); Lebanon (iv); North Macedonia (iii); Republic of Korea (iii); Republic of Moldova (iii); Slovenia (iii); Tajikistan (iii); Turkmenistan (iii);

The ASFR 10–14 observations, model estimates and uncertainty intervals during 1950–2023, by country are in Figure 15. One may refer to Figure 15 for the detailed information for each identified country model results.

7 Sub-regional results

We used the annual rate of reduction (ARR) to measure the instantaneous reduction over a period of time. While ARRs were similar for both periods 1990–2015 and 2015–2023 at the global level, the declines differed by sub-region for each period (Figure 11). The ARRs for age group 10–14 were significant in all sub-regions of Sub-Saharan Africa in 1990–2015. Particularly, Western Africa had significant reductions in both periods, with ARR increasing from 4.20% [3.28; 5.11] in 1990–2015 to 6.84% [0.62; 11.88] in 2015–2023, showing an acceleration of the reduction. Overall, sub-regions in other parts of the world experienced larger, when significant, reductions compared to Sub-Saharan African sub-regions.

For age group 15–19, most sub-regions with ARR significantly different from zero had a faster decline in the most recent period 2015–2023, rather than in the earlier years (1990–2015). The only exception is Southern Asia, where countries like India and Bangladesh experienced high rates of reduction in the earlier period (8.01% [7.58; 8.65] and 3.17% [2.31; 3.19], respectively, between 1990 and 2015), but had no significant reductions since 2015. Since 2015, the highest rates of reduction were registered in the regions with low adolescent birth rates, with Eastern Asia, all European sub-regions, Northern America, Australia and New Zealand having an ARR larger than 5% over the period 2015–2023, suggesting that adolescent birth rates can continue to decline to very low levels. Additionally, South America experienced a steep increase in the rate of reduction from 1.17% [0.78; 1.58] in 1990–2015 to 4.31% [2.93; 5.73] in 2015–2023. In countries like Argentina and Uruguay, the ARR was not significant in the period 1990–2015 and then reached 10.89% [8.44; 13.62] and 9.50% [6.22; 12.81] in 2015–2023, respectively. In Chile, ARR increased from 2.38% [1.76; 2.77] in 1990–2015 to 22.80% [20.79; 24.10] in 2015–2023. The sub-regions in Sub-Saharan Africa, where adolescent fertility reaches the highest values, did experience either non-significant reductions (Eastern Africa, Middle Africa) or low reduction rates (Southern Africa, Western Africa) compared to other regions. Melanesia, Micronesia and Polynesia did not have significant reductions in 2015–2023, while they experienced a reduction of less than 2% in 1990–2015.

8 Country-level results

This section provides an overview of the national ASFR distribution and spread relative to their regional ASFR and global ASFR for age groups 10–14 and 15–19. The interpretation of country-level outlying estimates should be taken with caution, because they suffer from lower data availability for the extreme cases.

The fertility rates for girls aged 10–14 and 15–19 varied substantially across countries in 2023, even after significant fertility declines globally and in all regions (Figure 12 and Figure 13). In Sub-Saharan Africa, the region with the highest fertility among girls under 15, four countries (Equatorial Guinea, Mauritania, Angola, and South Sudan) have estimated fertility rates of more than 10 births per 1,000 girls under 15 in 2023. In Eastern and South-Eastern Asia, Lao People's Democratic Republic exceeded 3.0 [0.9; 10.5] births per 1,000, despite lower regional rates overall. In Europe, Northern America, Australia, and New Zealand, where fertility rates for this age group are generally lowest, Bulgaria and Romania reported over 1 birth per 1,000 girls under 15, based on the complete data from the

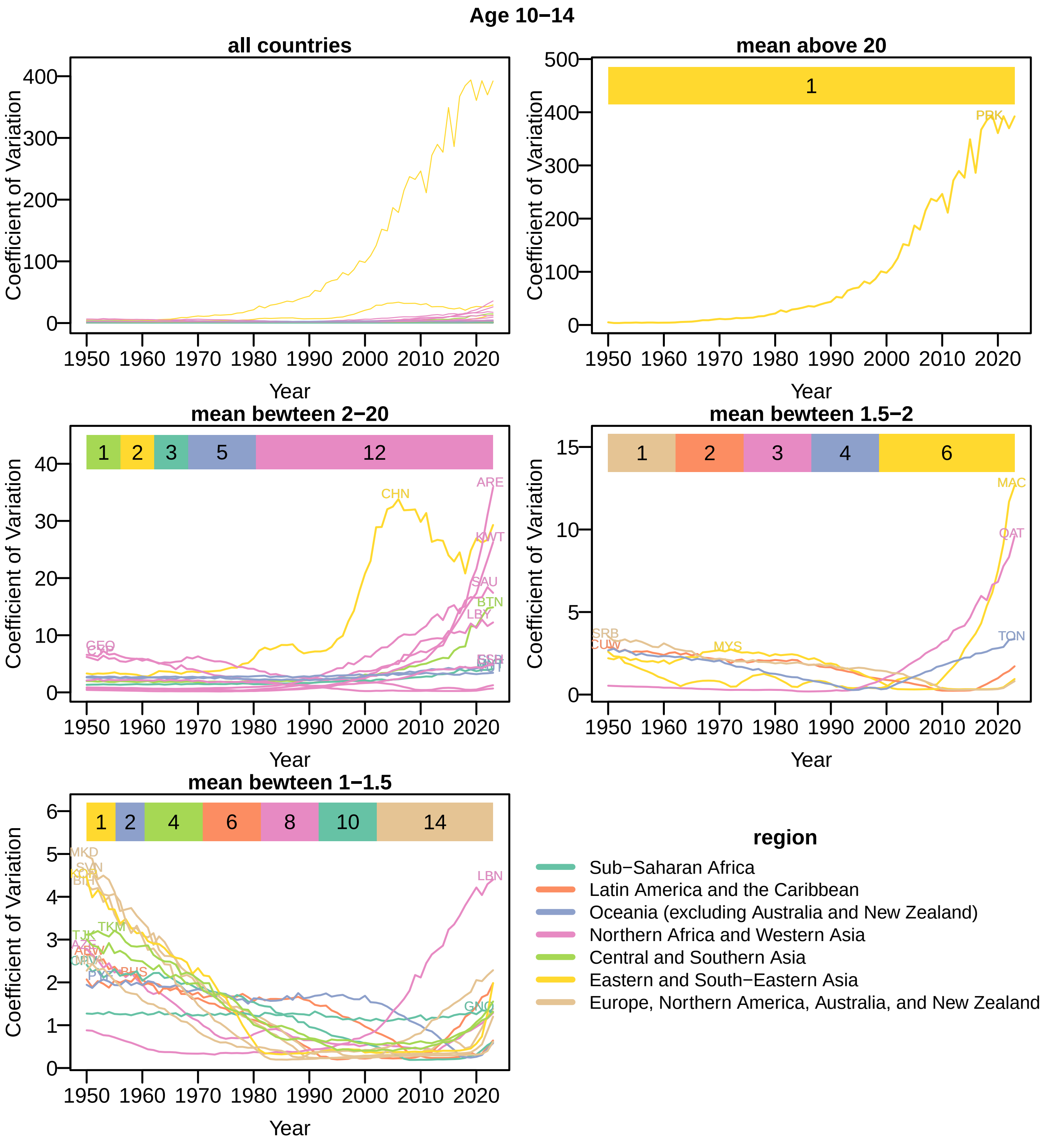

Figure 9: **Coefficient of variance (CV) for ASFR 10–14 model results, by country during 1950–2023.** Top left: CV by country over time for all countries. The remaining subfigures shows CV over time from countries with mean CV (average over time for each country) within intervals [20, +∞], [2, 20], [1.5, 2], and [1, 1.5]. Country time series are colored by region. The bar on top of each subfigure shows number of countries by region with mean CV falling into a certain interval. Country ISOs are listed next to each time series. Interval [20, +∞]: PRK–Dem. People's Republic of Korea. Interval [2, 20]: BTN–Bhutan; CHN–China; CYP–Cyprus; GEO–Georgia; KWT–Kuwait; LBY–Libya; MYT–Mayotte; SAU–Saudi Arabia; SLB–Solomon Islands; ARE–United Arab Emirates; VUT–Vanuatu; ESH–Western Sahara. Interval [1.5, 2]: MAC–China, Macao SAR; CUW–Curaçao; MYS–Malaysia; QAT–Qatar; SRB–Serbia; TON–Tonga. Interval [1, 1.5]: ABW–Aruba; AZE–Azerbaijan; BHS–Bahamas; BIH–Bosnia and Herzegovina; CPV–Cabo Verde; GNQ–Equatorial Guinea; PYF–French Polynesia; LBN–Lebanon; MKD–North Macedonia; KOR–Republic of Korea; MDA–Republic of Moldova; SVN–Slovenia; TJK–Tajikistan; TKM–Turkmenistan.

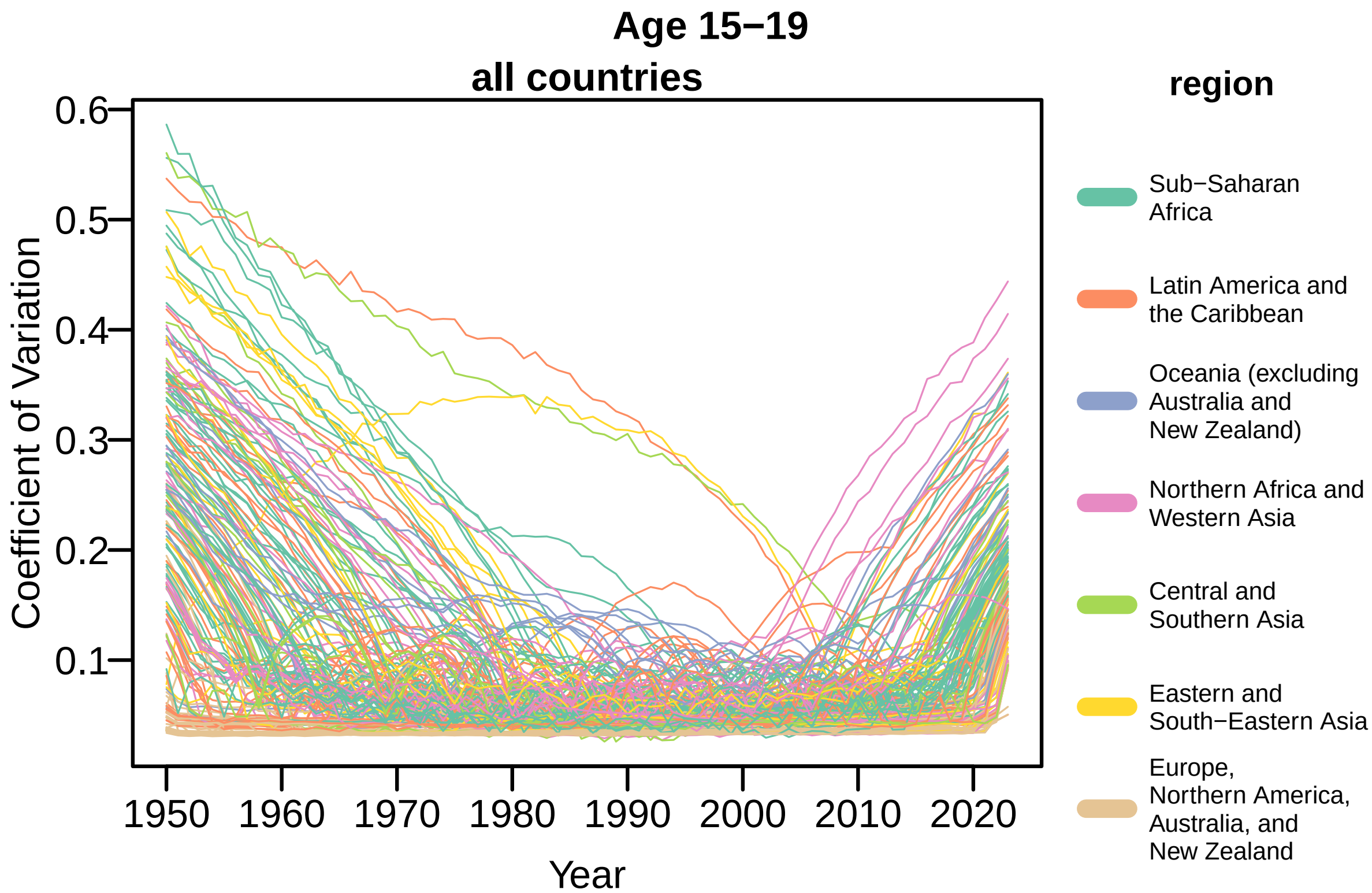

Figure 10: **Coefficient of variance for ASFR 15–19 model results, by country during 1950–2023.** Country time series are colored by region.

vital registration in the two countries. The national ASFR for the age group 15–19 follows a similar pattern as the age group 10–14, whereas the highest national fertility rates worldwide are concentrated in Sub-Saharan Africa and Latin America and the Caribbean, the variability within each region is less than that of the 10–14 age group. Only five countries are identified as outliers compared to other national fertility rates within a region. The fertility rate for ages 15–19 in the Lao People's Democratic Republic is more than five times the regional level at 81.7 [59.9; 110.0] births per 1,000.

9 Comparison of the results with GBD 2021 estimates

Figure 14 illustrates the absolute and percentage differences between the estimates compiled for WPP 2024 Revision and the ones published by the Institute for Health Metrics and Evaluation (IHME) in the Global Burden of Disease Study 2021 [24]. Here, the absolute difference is computed as GBD estimate minus the Bayesian estimates (i.e. the absolute difference can be negative).

In 1990, GBD fertility estimates for age group 10–14 are lower than WPP in 78% of countries, with the largest absolute and relative differences observed in Sub-Saharan Africa. WPP estimates show a sharp decline after 1990, with rates falling below 1 child per 1,000 girls aged 10-14 in more than half of the countries, leading to smaller absolute differences in 2021.

Country-specific examples further illustrate these discrepancies (Table 13). In Liberia, WPP estimated that fertility had declined from 16.5 children per 1,000 girls aged 10-14 in 1990 to 4.7 in 2021. These estimates were informed by full birth history series coming from DHS and MICS surveys carried out between 2007 and 2022. In contrast, GBD estimated a decline from 1.8 children per 1,000 girls in 1990 (-88.9% compared to WPP) to 1.12 in 2021 (-75.1%), showing the crucial importance of data from birth histories for age group 10-14. Similarly, in Bangladesh, WPP estimated that fertility in this age group fell from 18.7 children per 1,000 girls in 1990 to 1.9 in 2021. GBD, however, estimated a decline from 2.1 to 1.1, leading to differences of -16.6% in 1990 and -40% in 2021 compared to WPP.

For girls aged 15–19, absolute differences between WPP and GBD estimates are larger than for girls aged 10–14,

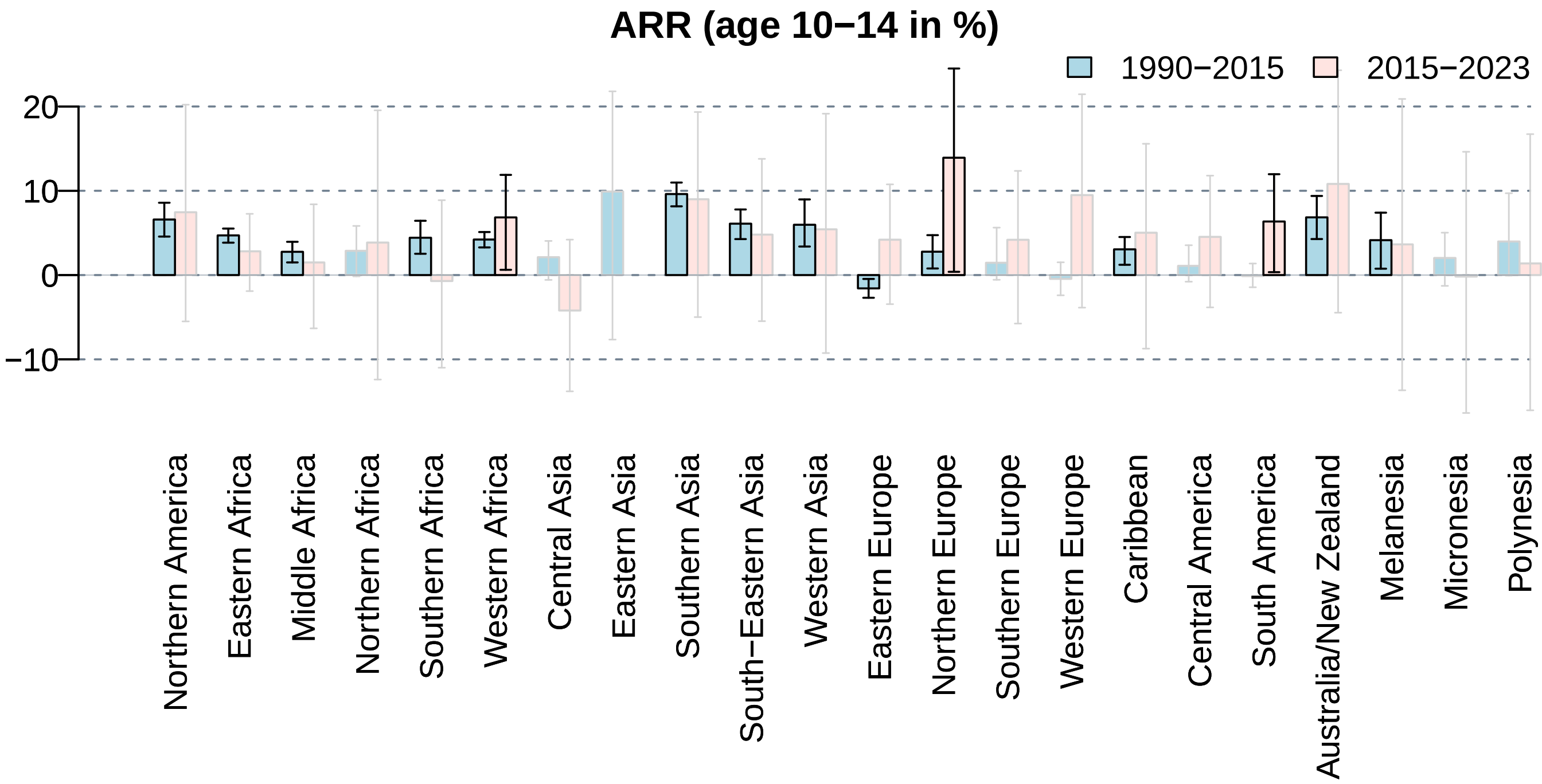

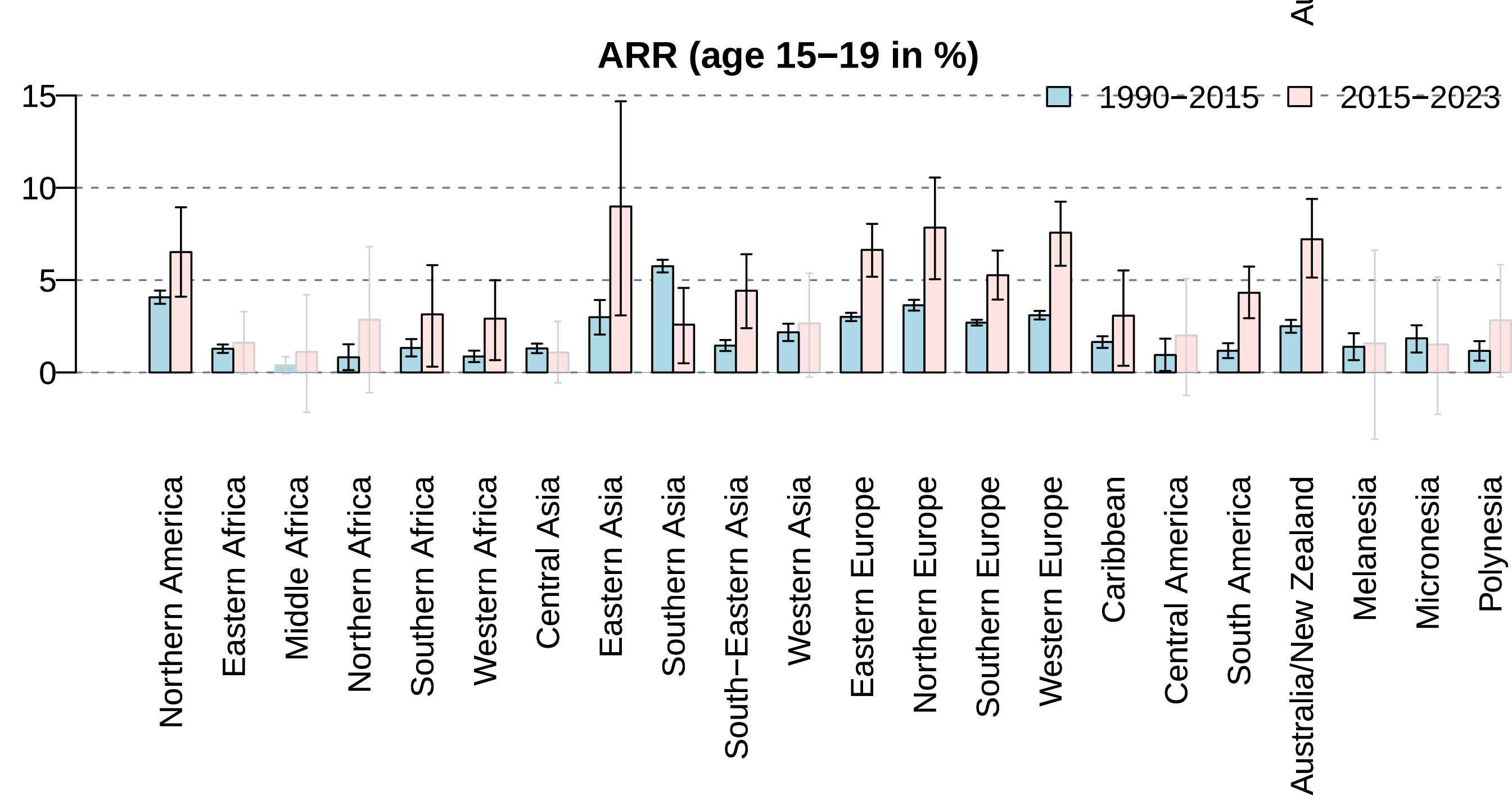

Figure 11: **ARR by mothers aged 10–14 (top) and 15–19 (bottom), in 22 sub-regions.** ARRs significantly different from zero are shown in black bars; otherwise, they are in grey. We do not report ARR if the age-specific fertility rates in both years are below 0.01 per 1,000. ARR: annual rates of reduction.

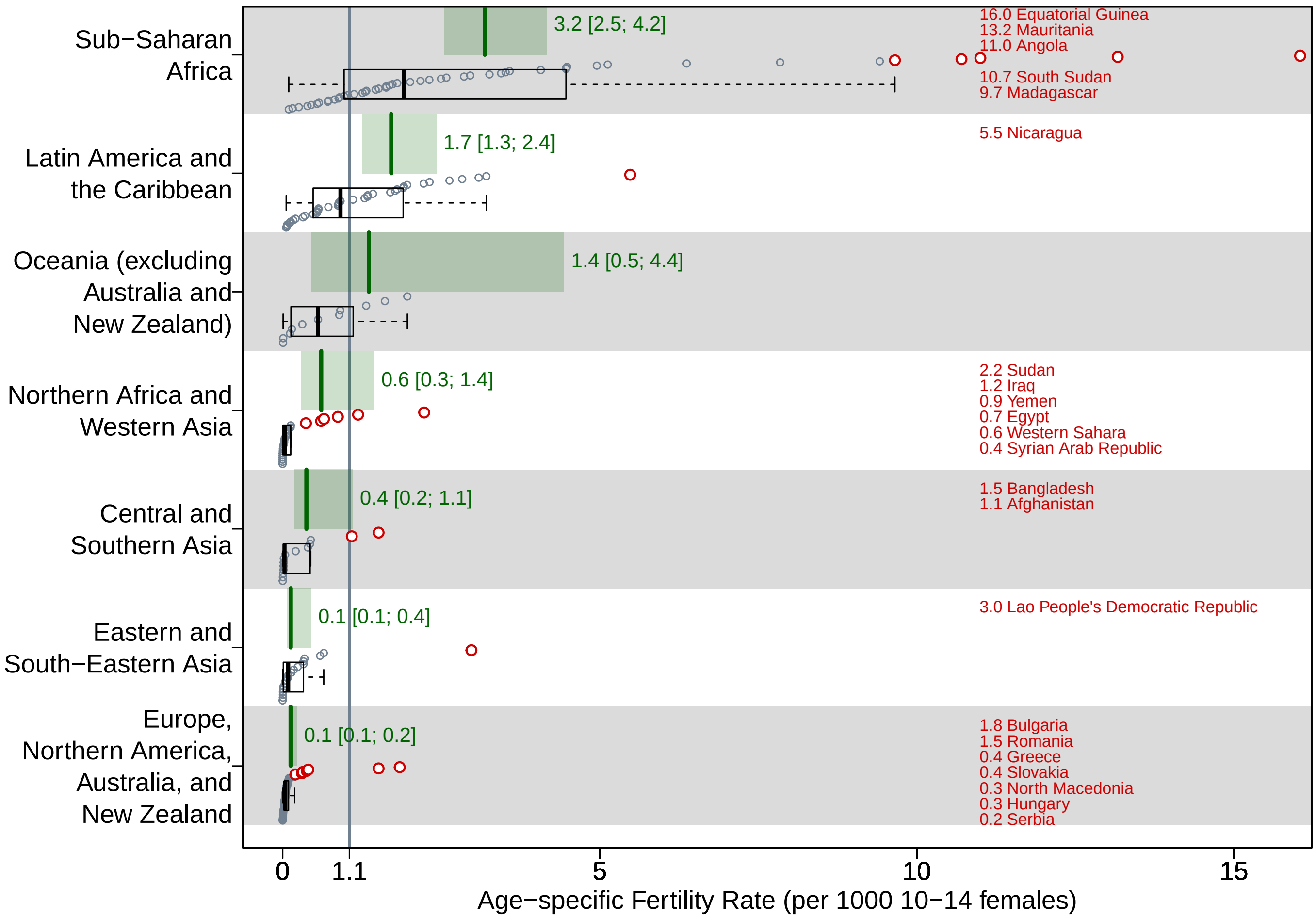

Figure 12: **ASFR aged 10–14 in 2023 on global, regional, and national levels.** Global ASFR: grey vertical line. Regional ASFR: median estimates are in dark green line segments with 95% uncertainty intervals as shaded areas and printed values in green. Box plots summarize the distributions of national ASFR within each region. National ASFRs are shown (dots), with those outside the range of [25th percentile−1.5IQR; 75th percentile+1.5IQR] highlighted in red. The countries with outlying national ASFR are listed in the legend with the median estimates reported (ordered by median estimate).

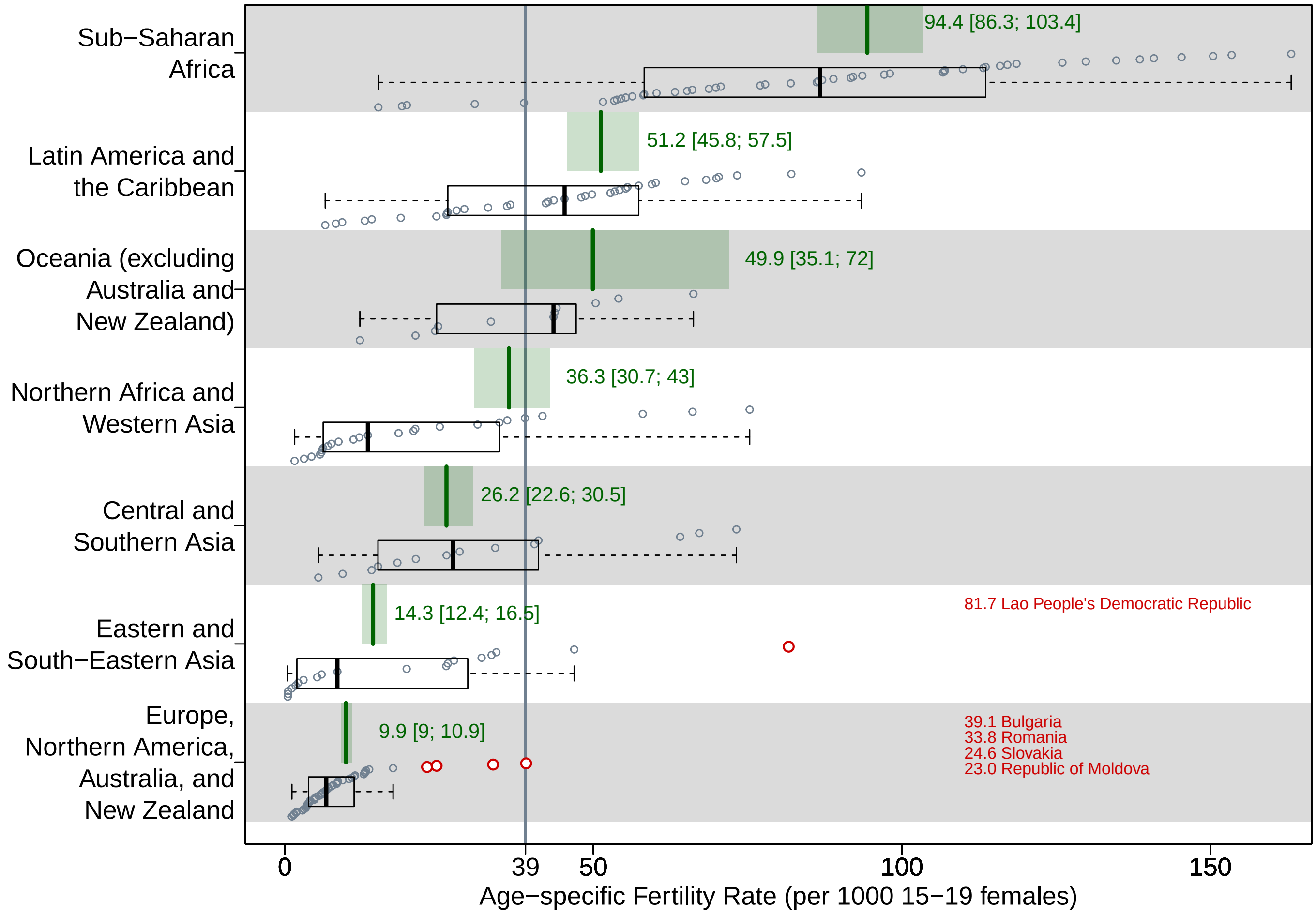

Figure 13: **ASFR aged 15–19 in 2023 on global, regional, and national levels.** Global ASFR: grey vertical line. Regional ASFR: median estimates are in dark green line segments with 95% uncertainty intervals as shaded areas and printed values in green. Box plots summarize the distributions of national ASFR within each region. National ASFRs are shown (dots), with those outside the range of [25th percentile−1.5IQR; 75th percentile+1.5IQR] highlighted in red. The countries with outlying national ASFR are listed in the legend with the median estimates reported (ordered by median estimate).

which aligns with generally higher fertility levels in this age group. However, percentage differences are narrower. In 1990, the largest discrepancies were concentrated in Central and Southern Asia. By 2021, the largest absolute differences were found in Sub-Saharan Africa, and the largest relative differences in Northern Africa and Western Asia, a result of WPP estimates being based on more recent data.

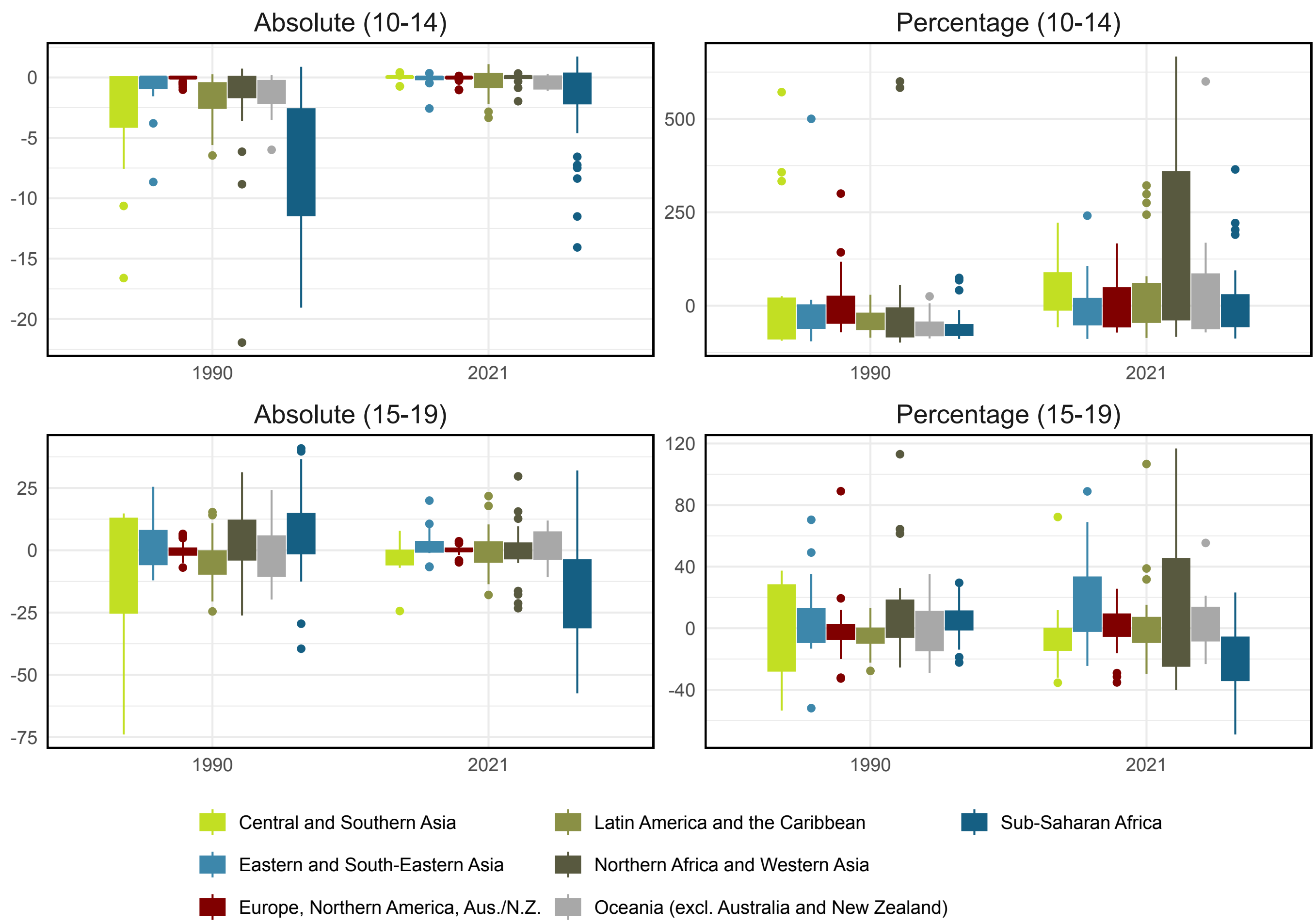

Figure 14: **Distribution of regional absolute and percentage differences between the WPP 2024[1] and the GBD 2021 estimates, for years 1990 and 2021.** Top row: results for ages 10–14. Bottom row: results for ages 15–19. Left column: absolute differences between the WPP 2024 and the GBD 2021 estimates. Right column: percentage differences between the WPP 2024 and the GBD 2021 estimates. Results from different regions are in different colors.

10 GATHER Compliance

Guidelines for Accurate and Transparent Health Estimates Reporting (GATHER) are reported in Table 11.

Table 11: **Checklist of information that should be included in new reports of global health estimates.** M: main manuscript. A: Appendix. Section titles after "M" are stated for the main manuscript. Page numbers after "A" are shown for the appendix.

Item #	Checklist item	Reported on page # or section #
**Objectives and funding**		
1	Define the indicator(s), populations (including age, sex, and geographic entities), and time period(s) for which estimates were made.	M-Introduction, A1–5
2	List the funding sources for the work.	M-Abstract
**Data inputs**		
For all data inputs from multiple sources that are synthesized as part of the study:		
3	Describe how the data were identified and how the data were accessed.	M-Database construction, A1–5
4	Specify the inclusion and exclusion criteria. Identify all ad-hoc exclusions.	M-Database construction, A5–7
5	Provide information on all included data sources and their main characteristics. For each data source used, report reference information or contact name/institution, the population represented, data collection method, year(s) of data collection, sex and age range, diagnostic criteria or measurement method, and sample size, as relevant.	A1–5 & A55–111
6	Identify and describe any categories of input data that have potentially important biases (e.g., based on characteristics listed in item 5).	A5–7
For data inputs that contribute to the analysis but were not synthesized as part of the study:		
7	Describe and give sources for any other data inputs.	M-Database construction, A1–6
For all data inputs:		
8	Provide all data inputs in a file format from which data can be efficiently extracted (e.g., a spreadsheet rather than a PDF), including all relevant meta-data listed in item 5. For any data inputs that cannot be shared because of ethical or legal reasons, such as third-party ownership, provide a contact name or the name of the institution that retains the right to the data.	M-Database construction
**Data analysis**		
9	Provide a conceptual overview of the data analysis method. A diagram may be helpful.	M-Database construction, A8
10	Provide a detailed description of all analysis steps, including mathematical formulae. This description should cover, as relevant, data cleaning, data pre-processing, data adjustments and weighting of data sources, and mathematical or statistical model(s).	M-Statistical analysis, A5–17
11	Describe how candidate models were evaluated and how the final model(s) were selected.	M-Statistical analysis, A17
12	Provide the results of an evaluation of model performance, if done, as well as the results of any relevant sensitivity analysis.	A17–21

Continued on next page

Table 11 – continued from previous page

Item #	Checklist item	Reported on page # or section #
13	Describe methods for calculating the uncertainty of the estimates. State which sources of uncertainty were, and were not, accounted for in the uncertainty analysis.	A7–17
14	State how analytic or statistical source code used to generate estimates can be accessed.	M-Statistical analysis
**Results and Discussion**		
15	Provide published estimates in a file format from which data can be efficiently extracted.	A33–55 & 111
16	Report a quantitative measure of the uncertainty of the estimates (e.g., uncertainty intervals).	A21–23
17	Interpret results in light of existing evidence. If updating a previous set of estimates, describe the reasons for changes in estimates.	M-Results
18	Discuss limitations of the estimates. Include a discussion of any modelling assumptions or data limitations that affect the interpretation of the estimates.	M-Discussion

References

1 UN, DESA. World Population Prospects: The 2024 Revision; 2024. Available at https://population.un.org/wpp/. Accessed 16 Feb 2026.

2 Max Planck Institute for Demographic Research (Germany) and Vienna Institute of Demography (Austria). Human Fertility Database. 2021. Available at www.humanfertility.org.

3 Max Planck Institute for Demographic Research (Germany) and Vienna Institute of Demography (Austria). Human Fertility Collection. 2020. Available at www.fertilitydata.org. Accessed 16 Feb 2026.

4 U S Bureau of the Census. International Data Base. 2020. Available at https://www.census.gov/data-tools/demo/idb/.

5 Institute for Health Metrics and Evaluation. Global Burden of Disease Study 2019 (GBD 2019). 2020. Available at www.healthdata.org/gbd.

6 Preston SH. The Use of Direct and Indirect Techniques for Estimating the Completeness of Death Registration Systems. In: Databases for Mortality Measurements. UN; 1984. p. 66-76.

7 Pullum TW, Croft T, MacQuarrie KLD. Methods to estimate under-15 fertility using demographic and health surveys data. Rockville, Maryland, USA: ICF. 2018.

8 Schoumaker B. Estimation of adolescent fertility (women aged 10-14) from international surveys prepared as harmonized data sets for the Population Division database (DemoData). New York, USA. 2019.

9 Simpson D, Rue H, Riebler A, Martins TG, Sørbye SH. Penalising model component complexity: A principled, practical approach to constructing priors. Statistical Science. 2017;32(1):1-28.

10 Nareeba T, Dzabeng F, Alam N, Biks GA, Thysen SM, Akuze J, et al. Neonatal and child mortality data in retrospective population-based surveys compared with prospective demographic surveillance: EN-INDEPTH study. Population Health Metrics. 2021;19:1-17.

11 Rue H, Martino S, Lindgren F, Simpson D, Riebler A. R-INLA: Approximate Bayesian Inference Using Integrated Nested Laplace Approximations. Trondheim, Norway. 2013. Available at: http://www.r-inla.org.

12 Wang H, Abbas KM, Abbasifard M, Abbasi-Kangevari M, Abbastabar H, Abd-Allah F, et al. Global age-sex-specific fertility, mortality, healthy life expectancy (HALE), and population estimates in 204 countries and territories, 1950–2019: a comprehensive demographic analysis for the Global Burden of Disease Study 2019. The Lancet. 2020;396(10258):1160-203.

13 Bhattacharjee NV, Schumacher AE, Aali A, Abate YH, Abbasgholizadeh R, Abbasian M, et al. Global fertility in 204 countries and territories, 1950–2021, with forecasts to 2100: a comprehensive demographic analysis for the Global Burden of Disease Study 2021. The Lancet. 2024;403(10440):2057-99.

14 Rue H, Martino S, Chopin N. Approximate Bayesian Inference for Latent Gaussian Models Using Integrated Nested Laplace Approximations (with discussion). Journal of the Royal Statistical Society B. 2009;71:319-92.

15 Alkema L, Chao F, You D, Pedersen J, Sawyer CC. National, regional, and global sex ratios of infant, child, and under-5 mortality and identification of countries with outlying ratios: a systematic assessment. The Lancet Global Health. 2014;2(9):e521-30.

16 Chao F, You D, Pedersen J, Hug L, Alkema L. National and regional under-5 mortality rate by economic status for low-income and middle-income countries: a systematic assessment. The Lancet Global Health. 2018;6(5):e535-47.

17 Chao F, You D, Pedersen J, Hug L, Alkema L. Web appendix National and regional under-5 mortality rate by economic status for low-income and middle-income countries: a systematic assessment. 2018. DOI: 10.6084/m9.figshare.12442244. Available at `https://ars.els-cdn.com/content/image/1-s2.0-S2214109X18300597-mmc1.pdf`.

18 Chao F, Gerland P, Cook AR, Alkema L. Systematic assessment of the sex ratio at birth for all countries and estimation of national imbalances and regional reference levels. Proceedings of the National Academy of Sciences. 2019;116(19):9303-11.

19 Chao F, Gerland P, Cook AR, Alkema L. Web Appendix Systematic assessment of the sex ratio at birth for all countries and estimation of national imbalances and regional reference levels. 2019. DOI: 10.6084/m9.figshare.12442373. Available at `https://www.pnas.org/content/pnas/suppl/2019/04/10/1812593116.DCSupplemental/pnas.1812593116.sapp.pdf`.

20 Chao F, Yadav AK. Levels and trends in the sex ratio at birth and missing female births for 29 states and union territories in India 1990–2016: A Bayesian modeling study. Foundations of Data Science. 2019;1(2):177-96.

21 Chao F, Gerland P, Cook AR, Alkema L. Global estimation and scenario-based projections of sex ratio at birth and missing female births using a Bayesian hierarchical time series mixture model. Ann Appl Statist. 2021;15(3):1499-528.

22 Chao F, Masquelier B, You D, Hug L, Liu Y, Sharrow D, et al. Sex differences in mortality among children, adolescents, and young people aged 0–24 years: a systematic assessment of national, regional, and global trends from 1990 to 2021. The Lancet Global Health. 2023;11(10):e1519-30.

23 Chao F, Masquelier B, You D, Hug L, Liu Y, Sharrow D, et al. Web Appendix for Sex differences in mortality among children, adolescents, and youth aged 0–24: A systematic assessment of national, regional, and global trends from 1990 to 2021. 2023. DOI: 10.6084/m9.figshare.23659998. Available at `https://www.thelancet.com/cms/10.1016/S2214-109X(23)00376-5/attachment/adc20287-2d64-4e50-ab58-40009007fed5/mmc1.pdf`.

24 Bhattacharjee NV, Schumacher AE, Aali A, et al. Global fertility in 204 countries and territories, 1950–2021, with forecasts to 2100: a comprehensive demographic analysis for the Global Burden of Disease Study 2021. The Lancet. 2024;403(10440):2057-99.

11 Supplementary tables

- Table 12: Classification of countries/areas by SGD region and subregion (page 31–33).
- Table 13: Estimates and 95% uncertainty intervals for ASFR 10–14 in 1990, 2015, and 2023, for the world, SDG regions, subregions, and all countries (page 33–39).
- Table 14: Estimates and 95% uncertainty intervals for ASFR 15–19 in 1990, 2015, and 2023, for the world, SDG regions, subregions, and all countries (page 39–45).
- Table 15: Estimates and uncertainty intervals for births (in thousands) by mothers aged 10–14 and the percentage of births in each location among all births of 10–14 for 1990, 2015, and 2023 for the world, SDG regions, subregions, and all countries (page 45–50).
- Table 16: Estimates and uncertainty intervals for births (in thousands) by mothers aged 15–19 and the percentage of births in each location among all births of 15–19 for 1990, 2015, and 2023 for the world, SDG regions, subregions, and all countries (page 50–55).
- Table 17: Age-specific fertility rate data sources for age group 10–14, by country (page 55–75).
- Table 18: Age-specific fertility rate data sources for age group 15–19, by country (page 75–111).

Table 12: **Classification of countries/areas by SGD region and subregion.** Countries are in alphabetical order in each subregion. Numbers in brackets after each region and subregion name indicate the number of countries from each (sub)region. Only countries/areas with population size above 90,000 by mid-2024 are shown.

SDG Region	Subregion	Country/Area
Sub-Saharan Africa [50]	Middle Africa [9]	Angola; Cameroon; Central African Republic; Chad; Republic of the Congo; Democratic Republic of the Congo; Equatorial Guinea; Gabon; Sao Tome and Principe
	Western Africa [16]	Benin; Burkina Faso; Cabo Verde; Côte d'Ivoire; Gambia; Ghana; Guinea; Guinea-Bissau; Liberia; Mali; Mauritania; Niger; Nigeria; Senegal; Sierra Leone; Togo
	Southern Africa [5]	Botswana; Eswatini; Lesotho; Namibia; South Africa
	Eastern Africa [20]	Burundi; Comoros; Djibouti; Eritrea; Ethiopia; Kenya; Madagascar; Malawi; Mauritius; Mayotte; Mozambique; Réunion; Rwanda; Seychelles; Somalia; South Sudan; Uganda; United Republic of Tanzania; Zambia; Zimbabwe
Latin America and the Caribbean [37]	Caribbean [16]	Antigua and Barbuda; Aruba; Bahamas; Barbados; Cuba; Curaçao; Dominican Republic; Grenada; Guadeloupe; Haiti; Jamaica; Martinique; Puerto Rico; Saint Lucia; Saint Vincent and the Grenadines; Trinidad and Tobago
	South America [13]	Argentina; Bolivia (Plurinational State of); Brazil; Chile; Colombia; Ecuador; French Guiana; Guyana; Paraguay; Peru; Suriname; Uruguay; Venezuela (Bolivarian Republic of)
	Central America [8]	Belize; Costa Rica; El Salvador; Guatemala; Honduras; Mexico; Nicaragua; Panama
Oceania (excluding Australia and New Zealand) [11]	Polynesia [3]	French Polynesia; Samoa; Tonga
	Melanesia [5]	Fiji; New Caledonia; Papua New Guinea; Solomon Islands; Vanuatu
	Micronesia [3]	Guam; Kiribati; Micronesia (Fed. States of)

Continued on next page

Table 12 – continued from previous page

SDG Region	Subregion	Country/Area
Northern Africa and Western Asia [25]	Northern Africa [7]	Algeria; Egypt; Libya; Morocco; Sudan; Tunisia; Western Sahara
	Western Asia [18]	Armenia; Azerbaijan; Bahrain; Cyprus; Georgia; Iraq; Israel; Jordan; Kuwait; Lebanon; Oman; Qatar; Saudi Arabia; State of Palestine; Syrian Arab Republic; Türkiye; United Arab Emirates; Yemen
Central and Southern Asia [14]	Southern Asia [9]	Afghanistan; Bangladesh; Bhutan; India; Iran (Islamic Republic of); Maldives; Nepal; Pakistan; Sri Lanka
	Central Asia [5]	Kazakhstan; Kyrgyz Republic; Tajikistan; Turkmenistan; Uzbekistan
Eastern and South-Eastern Asia [19]	South-Eastern Asia [11]	Brunei Darussalam; Cambodia; Indonesia; Lao People's Democratic Republic; Malaysia; Myanmar; Philippines; Singapore; Thailand; Timor-Leste; Viet Nam
	Eastern Asia [8]	China; China, Hong Kong SAR; Macao, SAR of China; China, Taiwan Province of China; Dem. People's Republic of Korea; Japan; Mongolia; Republic of Korea
Europe, Northern America, Australia, and New Zealand [45]	Southern Europe [13]	Albania; Bosnia and Herzegovina; Croatia; Greece; Italy; Kosovo (under UNSC res. 1244); Malta; Montenegro; North Macedonia; Portugal; Serbia; Slovenia; Spain
	Australia/New Zealand [2]	Australia; New Zealand
	Western Europe [7]	Austria; Belgium; France; Germany; Luxembourg; Netherlands; Switzerland
	Eastern Europe [10]	Belarus; Bulgaria; Czechia; Hungary; Poland; Republic of Moldova; Romania; Russian Federation; Slovakia; Ukraine
	Northern America [2]	Canada; United States of America
	Northern Europe [11]	Denmark; Estonia; Finland; Iceland; Ireland; Jersey; Latvia; Lithuania; Norway; Sweden; United Kingdom

Table 13: **Estimates and 95% uncertainty intervals for ASFR 10–14 in 1990, 2015, and 2023, the total changes between 1990–2015, between 2015–2013, and between 1990–2023, and the annual rate of reduction (ARR) between 1990–2015, between 2015–2013, and between 1990–2023, for the world, seven SDG regions, 22 subregions, and 201 countries*.** †: Change or ARR is significantly different from zero. *We report results from countries/areas with more than 90,000 population size by mid-2024.

Age-specific fertility rate 10–14										
		Estimate (per 1,000)			Total Change (per 1,000)			Annual Rate of Reduction (per 100)		
**Location Name**	**Location Type**	**1990**	**2015**	**2023**	**1990–2015**	**2015–2023**	**1990–2023**	**1990–2015**	**2015–2023**	**1990–2023**
World	World	4.5 [4.1; 5.2]	1.5 [1.3; 1.6]	1.1 [0.9; 1.4]	-3.0 [-3.8; -2.6]†	-0.4 [-0.7; -0.1]†	-3.4 [-4.2; -2.9]†	4.46 [3.86; 5.16]†	4.14 [0.65; 7.04]†	4.39 [3.52; 5.15]†
Sub-Saharan Africa	SDG region	11.6 [10.8; 12.5]	4.3 [3.8; 4.9]	3.2 [2.5; 4.2]	-7.3 [-8.3; -6.4]†	-1.1 [-2.0; 0.0]†	-8.4 [-9.6; -7.2]†	3.98 [3.41; 4.55]†	3.73 [0.09; 6.96]†	3.92 [3.08; 4.65]†
Latin America and the Caribbean	SDG region	3.0 [2.5; 3.9]	2.7 [2.3; 3.3]	1.7 [1.3; 2.4]	-0.3 [-1.3; 0.5]	-1.0 [-1.7; -0.2]†	-1.3 [-2.3; -0.4]†	0.42 [-0.63; 1.61]	5.88 [1.10; 10.30]†	1.74 [0.52; 2.94]†
Oceania (excluding Australia and New Zealand)	SDG region	4.8 [3.0; 7.6]	1.8 [1.0; 3.6]	1.4 [0.5; 4.4]	-3.0 [-6.0; -0.5]†	-0.4 [-2.4; 2.7]	-3.4 [-6.4; 0.1]	3.90 [0.61; 7.03]†	3.47 [-13.43; 19.60]	3.79 [-0.07; 7.50]
Northern Africa and Western Asia	SDG region	2.6 [2.1; 3.7]	0.9 [0.5; 1.4]	0.6 [0.3; 1.4]	-1.7 [-2.8; -1.0]†	-0.3 [-0.9; 0.6]	-2.0 [-3.1; -1.1]†	4.35 [2.18; 6.63]†	4.39 [-7.54; 15.75]	4.36 [1.68; 6.88]†
Central and Southern Asia	SDG region	8.5 [7.2; 10.1]	0.8 [0.6; 1.1]	0.4 [0.2; 1.1]	-7.7 [-9.4; -6.4]†	-0.4 [-0.8; 0.4]	-8.1 [-9.7; -6.6]†	9.57 [8.11; 10.93]†	9.12 [-4.85; 19.42]	9.46 [6.18; 11.77]†
Eastern and South-Eastern Asia	SDG region	0.8 [0.6; 2.9]	0.2 [0.1; 0.5]	0.1 [0.1; 0.4]	-0.6 [-2.6; -0.3]†	-0.1 [-0.4; 0.3]	-0.6 [-2.7; -0.3]†	5.50 [1.85; 10.81]†	5.04 [-11.37; 18.34]	5.39 [1.60; 9.63]†
Europe, Northern America, Australia, and New Zealand	SDG region	0.5 [0.4; 0.7]	0.2 [0.2; 0.3]	0.1 [0.1; 0.2]	-0.3 [-0.5; -0.2]†	-0.1 [-0.2; 0.0]	-0.4 [-0.6; -0.3]†	3.72 [2.36; 4.99]†	6.26 [-0.95; 12.33]	4.33 [2.52; 5.82]†
Northern America	Subregion	1.6 [1.1; 2.2]	0.3 [0.2; 0.4]	0.2 [0.1; 0.5]	-1.3 [-1.9; -0.8]†	-0.1 [-0.3; 0.2]	-1.4 [-2.0; -0.9]†	6.59 [4.57; 8.58]†	7.45 [-5.50; 20.21]	6.80 [3.67; 9.74]†
Eastern Africa	Subregion	10.4 [9.4; 11.5]	3.2 [2.7; 3.9]	2.6 [1.9; 3.6]	-7.2 [-8.4; -6.0]†	-0.6 [-1.6; 0.5]	-7.8 [-9.1; -6.4]†	4.71 [3.84; 5.52]†	2.82 [-1.90; 7.26]	4.25 [3.18; 5.23]†
Middle Africa	Subregion	13.1 [11.2; 15.5]	6.6 [5.1; 8.7]	5.8 [3.6; 10.4]	-6.5 [-9.3; -3.7]†	-0.7 [-3.8; 4.0]	-7.3 [-10.5; -2.3]†	2.76 [1.50; 3.95]†	1.50 [-6.32; 8.40]	2.45 [0.62; 3.99]†
Northern Africa	Subregion	2.2 [1.7; 3.0]	1.1 [0.5; 2.2]	0.8 [0.3; 2.5]	-1.1 [-2.1; 0.1]	-0.3 [-1.5; 1.5]	-1.4 [-2.4; 0.3]	2.88 [-0.15; 5.83]	3.86 [-12.39; 19.54]	3.11 [-0.44; 6.47]
Southern Africa	Subregion	3.0 [2.2; 4.4]	1.0 [0.7; 1.4]	1.1 [0.5; 2.2]	-2.0 [-3.4; -1.1]†	0.1 [-0.6; 1.3]	-2.0 [-3.4; -0.6]†	4.43 [2.53; 6.44]†	-0.71 [-10.99; 8.88]	3.18 [0.73; 5.51]†
Western Africa	Subregion	14.2 [12.6; 16.1]	5.0 [4.1; 6.1]	2.9 [2.0; 4.6]	-9.3 [-11.3; -7.3]†	-2.1 [-3.5; -0.2]†	-11.3 [-13.5; -9.1]†	4.22 [3.28; 5.11]†	6.84 [0.62; 11.89]†	4.85 [3.43; 5.99]†
Central Asia	Subregion	0.0 [0.0; 0.0]	0.0 [0.0; 0.0]	0.0 [0.0; 0.0]	0.0 [0.0; 0.0]	0.0 [0.0; 0.0]	0.0 [0.0; 0.0]	2.12 [-0.57; 4.05]	-4.21 [-13.79; 4.20]	0.59 [-2.24; 2.69]
Eastern Asia	Subregion	0.1 [0.0; 3.3]	0.0 [0.0; 0.6]	0.0 [0.0; 0.5]	-0.1 [-3.1; 0.4]	0.0 [-0.6; 0.5]	-0.1 [-3.2; 0.3]	9.91 [-7.65; 21.80]	0.00 [-44.51; 46.46]	7.50 [-6.19; 16.69]
Southern Asia	Subregion	8.9 [7.5; 10.5]	0.8 [0.6; 1.1]	0.4 [0.2; 1.2]	-8.1 [-9.7; -6.6]†	-0.4 [-0.8; 0.4]	-8.5 [-10.1; -6.9]†	9.61 [8.16; 10.98]†	9.00 [-4.99; 19.34]	9.46 [6.18; 11.78]†
South-Eastern Asia	Subregion	2.2 [1.8; 2.8]	0.5 [0.3; 0.7]	0.3 [0.2; 0.7]	-1.7 [-2.3; -1.2]†	-0.2 [-0.4; 0.2]	-1.9 [-2.4; -1.3]†	6.10 [4.27; 7.78]†	4.80 [-5.47; 13.79]	5.78 [3.47; 7.67]†
Western Asia	Subregion	2.9 [2.3; 5.0]	0.7 [0.4; 1.2]	0.4 [0.2; 1.2]	-2.3 [-4.4; -1.5]†	-0.2 [-0.8; 0.6]	-2.5 [-4.6; -1.5]†	5.97 [3.39; 8.97]†	5.43 [-9.25; 19.15]	5.84 [2.70; 9.04]†
Eastern Europe	Subregion	0.2 [0.2; 0.2]	0.3 [0.2; 0.4]	0.2 [0.1; 0.4]	0.1 [0.0; 0.2]†	-0.1 [-0.2; 0.1]	0.0 [-0.1; 0.2]	-1.58 [-2.69; -0.46]†	4.20 [-3.45; 10.76]	-0.18 [-1.95; 1.35]
Northern Europe	Subregion	0.1 [0.1; 0.2]	0.1 [0.0; 0.1]	0.0 [0.0; 0.1]	-0.1 [-0.1; 0.0]†	0.0 [-0.1; 0.0]†	-0.1 [-0.2; -0.1]†	2.77 [0.78; 4.74]†	13.92 [0.39; 24.51]†	5.48 [2.18; 8.01]†
Southern Europe	Subregion	0.2 [0.1; 0.4]	0.1 [0.1; 0.1]	0.1 [0.0; 0.2]	0.0 [-0.3; 0.0]	0.0 [-0.1; 0.1]	-0.1 [-0.4; 0.0]	1.46 [-0.56; 5.63]	4.18 [-5.75; 12.36]	2.12 [-0.40; 5.63]
Western Europe	Subregion	0.1 [0.1; 0.1]	0.1 [0.1; 0.1]	0.0 [0.0; 0.1]	0.0 [0.0; 0.1]	0.0 [-0.1; 0.0]	0.0 [-0.1; 0.0]	-0.45 [-2.40; 1.51]	9.49 [-3.86; 21.45]	1.96 [-1.19; 4.90]
Caribbean	Subregion	4.4 [3.7; 5.3]	2.0 [1.5; 3.2]	1.4 [0.7; 4.1]	-2.3 [-3.4; -1.1]†	-0.7 [-2.0; 2.0]	-3.0 [-4.2; -0.3]†	3.05 [1.22; 4.51]†	5.02 [-8.73; 15.57]	3.53 [0.19; 5.79]†
Central America	Subregion	2.7 [1.9; 4.8]	2.1 [1.6; 2.7]	1.4 [0.9; 2.7]	-0.6 [-2.8; 0.4]	-0.6 [-1.5; 0.7]	-1.3 [-3.3; 0.2]	1.09 [-0.78; 3.54]	4.53 [-3.83; 11.80]	1.92 [-0.29; 4.18]
South America	Subregion	3.0 [2.4; 4.1]	3.1 [2.6; 3.9]	1.9 [1.3; 2.9]	0.1 [-1.1; 1.1]	-1.3 [-2.3; -0.1]†	-1.2 [-2.4; 0.1]	-0.12 [-1.44; 1.37]	6.35 [0.34; 11.97]†	1.45 [-0.08; 2.88]
Australia/New Zealand	Subregion	0.6 [0.4; 1.0]	0.1 [0.1; 0.2]	0.0 [0.0; 0.2]	-0.5 [-0.9; -0.3]†	-0.1 [-0.1; 0.0]	-0.6 [-0.9; -0.3]†	6.84 [4.27; 9.39]†	10.81 [-4.46; 24.30]	7.81 [4.22; 11.05]†
Melanesia	Subregion	5.4 [3.4; 8.9]	1.9 [1.0; 3.9]	1.4 [0.4; 4.8]	-3.5 [-7.1; -0.7]†	-0.5 [-2.7; 3.0]	-4.0 [-7.6; -0.1]†	4.14 [0.76; 7.40]†	3.64 [-13.67; 20.89]	4.02 [0.06; 7.97]†
Micronesia	Subregion	1.2 [0.8; 1.7]	0.7 [0.4; 1.5]	0.7 [0.3; 2.1]	-0.5 [-1.1; 0.4]	0.0 [-0.9; 1.5]	-0.5 [-1.2; 1.0]	2.03 [-1.28; 5.03]	-0.18 [-16.36; 14.63]	1.49 [-1.96; 4.52]
Polynesia	Subregion	1.0 [0.5; 3.5]	0.4 [0.2; 0.8]	0.3 [0.1; 1.1]	-0.6 [-3.1; 0.0]	0.0 [-0.5; 0.8]	-0.7 [-3.1; 0.3]	3.98 [-0.07; 9.71]	1.38 [-16.04; 16.72]	3.35 [-0.95; 8.07]
Afghanistan	Country	11.4 [8.2; 16.1]	1.2 [0.6; 2.2]	1.1 [0.3; 3.7]	-10.2 [-14.9; -6.8]†	-0.1 [-1.3; 2.5]	-10.3 [-15.0; -6.4]†	9.10 [6.15; 12.00]†	0.90 [-15.76; 17.37]	7.11 [3.34; 10.77]†
Albania	Country	0.0 [0.0; 0.3]	0.2 [0.1; 0.4]	0.2 [0.1; 0.6]	0.2 [0.0; 0.4]	0.0 [-0.3; 0.4]	0.2 [-0.1; 0.6]	-7.18 [-13.73; 0.76]	0.68 [-14.33; 19.82]	-5.28 [-10.84; 2.16]
Algeria	Country	0.3 [0.1; 0.5]	0.0 [0.0; 0.1]	0.0 [0.0; 0.2]	-0.2 [-0.4; -0.1]†	0.0 [-0.1; 0.2]	-0.2 [-0.4; 0.0]†	11.35 [4.75; 14.75]†	-1.79 [-32.13; 20.67]	8.17 [0.78; 11.24]†
Angola	Country	13.6 [9.4; 20.1]	10.9 [6.8; 17.6]	11.0 [4.6; 25.4]	-2.7 [-10.4; 5.1]	0.1 [-9.2; 15.0]	-2.6 [-12.0; 12.5]	0.84 [-1.54; 3.28]	0.03 [-11.94; 12.17]	0.64 [-2.12; 3.50]
Antigua and Barbuda	Country	2.4 [1.0; 6.1]	0.6 [0.3; 1.3]	0.3 [0.1; 1.2]	-1.8 [-5.5; -0.1]†	-0.3 [-1.0; 0.6]	-2.1 [-5.8; -0.5]†	5.82 [0.52; 10.38]†	7.28 [-10.87; 25.24]	6.18 [1.49; 10.52]†
Argentina	Country	2.2 [1.6; 3.1]	1.7 [1.2; 2.5]	0.6 [0.2; 1.4]	-0.5 [-1.5; 0.5]	-1.1 [-2.0; -0.2]†	-1.6 [-2.6; -0.6]†	1.13 [-1.00; 3.02]	13.74 [1.55; 25.84]†	4.18 [1.20; 6.95]†
Armenia	Country	0.0 [0.0; 0.0]	0.0 [0.0; 0.0]	0.0 [0.0; 0.0]	0.0 [0.0; 0.0]†	0.0 [0.0; 0.0]	0.0 [0.0; 0.0]	1.72 [0.00; 4.12]	0.00 [-18.19; 0.00]	1.30 [-3.35; 2.98]
Aruba	Country	0.5 [0.2; 1.3]	0.4 [0.3; 0.6]	0.2 [0.1; 0.6]	-0.1 [-0.9; 0.3]	-0.2 [-0.5; 0.2]	-0.3 [-1.0; 0.2]	1.47 [-2.59; 5.04]	8.84 [-5.78; 23.51]	3.26 [-1.17; 7.13]
Australia	Country	0.6 [0.4; 1.0]	0.1 [0.1; 0.2]	0.0 [0.0; 0.2]	-0.5 [-0.9; -0.2]†	-0.1 [-0.2; 0.1]	-0.6 [-1.0; -0.3]†	6.86 [3.80; 10.12]†	11.47 [-7.63; 29.13]	7.98 [3.61; 12.39]†
Austria	Country	0.1 [0.1; 0.2]	0.1 [0.0; 0.1]	0.0 [0.0; 0.1]	0.0 [-0.1; 0.0]	-0.1 [-0.1; 0.0]	-0.1 [-0.2; 0.0]†	1.85 [-0.79; 4.89]	19.24 [-1.46; 29.82]	6.07 [1.26; 8.47]†

†: Change is significantly different from zero.
Total Change (Year 1–Year 2): ASFR (Year 2) - ASFR (Year 1).
Annual Rate of Reduction (Year 1–Year 2): $\log(\text{ASFR}_{\text{Year 2}}/\text{ASFR}_{\text{Year 1}})/(\text{Year 1} - \text{Year 2})$, where Year 1 is smaller than Year 2.

Continued on next page

Table 13 – continued from previous page

Age-specific fertility rate 10–14										
		Estimate (per 1,000)			Total Change (per 1,000)			Annual Rate of Reduction (per 100)		
**Location Name**	**Location Type**	**1990**	**2015**	**2023**	**1990–2015**	**2015–2023**	**1990–2023**	**1990–2015**	**2015–2023**	**1990–2023**
Azerbaijan	Country	0.0 [0.0; 0.0]	0.0 [0.0; 0.1]	0.1 [0.0; 0.3]	0.0 [0.0; 0.1]	0.0 [-0.1; 0.3]	0.0 [0.0; 0.3]	-5.56 [-9.04; 0.64]	-3.59 [-29.05; 19.24]	-5.08 [-10.00; 0.78]
Bahamas	Country	0.8 [0.1; 5.0]	0.3 [0.1; 0.8]	0.1 [0.0; 1.1]	-0.5 [-4.7; 0.4]	-0.1 [-0.7; 0.8]	-0.6 [-4.9; 0.4]	4.47 [-4.30; 13.45]	8.89 [-22.29; 37.59]	5.54 [-2.83; 13.67]
Bahrain	Country	0.4 [0.2; 0.7]	0.1 [0.0; 0.5]	0.1 [0.0; 0.8]	-0.3 [-0.6; 0.1]	0.0 [-0.4; 0.7]	-0.3 [-0.6; 0.4]	6.09 [-1.18; 13.66]	4.87 [-35.16; 39.11]	5.79 [-2.24; 11.82]
Bangladesh	Country	18.7 [15.9; 22.1]	4.5 [3.1; 6.5]	1.5 [0.6; 4.0]	-14.2 [-17.9; -10.7]†	-3.0 [-5.2; -0.1]†	-17.2 [-20.7; -13.5]†	5.93 [4.10; 7.39]†	13.91 [0.42; 26.81]†	7.86 [4.64; 10.72]†
Barbados	Country	0.9 [0.6; 1.4]	0.5 [0.1; 2.0]	0.6 [0.1; 3.6]	-0.4 [-1.0; 1.2]	0.1 [-1.4; 3.1]	-0.3 [-1.1; 2.7]	2.56 [-3.45; 8.94]	-2.02 [-31.92; 27.87]	1.45 [-4.53; 7.34]
Belarus	Country	0.0 [0.0; 0.1]	0.1 [0.0; 0.1]	0.0 [0.0; 0.1]	0.0 [0.0; 0.1]	0.0 [-0.1; 0.1]	0.0 [0.0; 0.1]	-1.93 [-4.85; 0.66]	12.72 [-8.79; 26.85]	1.62 [-3.92; 5.00]
Belgium	Country	0.1 [0.0; 0.1]	0.1 [0.0; 0.1]	0.0 [0.0; 0.3]	0.0 [-0.1; 0.1]	0.0 [-0.1; 0.2]	0.0 [-0.1; 0.2]	0.26 [-2.89; 3.12]	7.27 [-17.42; 27.54]	1.96 [-4.02; 6.70]
Belize	Country	2.0 [1.4; 2.9]	1.1 [0.7; 1.6]	1.1 [0.6; 2.2]	-0.9 [-1.9; -0.1]†	0.0 [-0.7; 1.2]	-0.9 [-1.9; 0.3]	2.64 [0.30; 4.70]†	0.01 [-10.27; 9.34]	2.01 [-0.51; 4.16]
Benin	Country	11.8 [9.3; 15.1]	2.6 [1.7; 4.1]	1.7 [0.6; 4.6]	-9.2 [-12.6; -6.2]†	-0.9 [-2.8; 2.0]	-10.1 [-13.5; -6.3]†	5.97 [3.95; 7.97]†	5.27 [-7.92; 19.32]	5.80 [2.79; 8.92]†
Bhutan	Country	1.7 [0.2; 15.0]	0.2 [0.0; 3.7]	0.0 [0.0; 1.1]	-1.5 [-14.5; 2.0]	-0.1 [-3.6; 0.9]	-1.7 [-15.0; 0.1]	13.69 [-5.75; 24.32]	25.57 [-36.41; 67.54]	16.57 [-0.74; 20.90]
Bolivia (Plurinational State of)	Country	3.3 [2.5; 4.5]	1.6 [0.5; 5.6]	1.3 [0.2; 7.1]	-1.7 [-3.4; 2.3]	-0.3 [-4.1; 5.6]	-2.0 [-3.6; 3.8]	2.93 [-2.21; 8.01]	2.57 [-23.88; 28.58]	2.84 [-2.45; 7.96]
Bosnia and Herzegovina	Country	0.1 [0.1; 0.2]	0.0 [0.0; 0.1]	0.0 [0.0; 0.2]	-0.1 [-0.2; 0.0]	0.0 [-0.1; 0.2]	-0.1 [-0.2; 0.1]	3.20 [-2.12; 8.16]	3.46 [-22.69; 26.26]	3.26 [-2.59; 7.83]
Botswana	Country	3.5 [0.9; 13.9]	0.5 [0.3; 0.8]	0.5 [0.1; 1.4]	-3.0 [-13.4; -0.3]†	-0.1 [-0.5; 0.9]	-3.1 [-13.3; -0.3]†	7.89 [1.80; 13.42]†	1.70 [-13.25; 16.30]	6.39 [0.98; 11.71]†
Brazil	Country	2.8 [1.6; 4.9]	3.3 [2.3; 4.8]	1.9 [0.9; 3.8]	0.5 [-1.8; 2.4]	-1.4 [-3.2; 0.7]	-0.9 [-3.1; 1.3]	-0.37 [-3.26; 2.01]	6.68 [-3.00; 16.81]	1.34 [-1.50; 3.88]
Brunei Darussalam	Country	0.5 [0.3; 0.8]	0.2 [0.1; 0.5]	0.2 [0.1; 0.9]	-0.2 [-0.5; 0.1]	0.0 [-0.3; 0.7]	-0.2 [-0.6; 0.4]	2.78 [-0.65; 5.80]	-0.26 [-18.43; 19.17]	2.05 [-2.29; 6.59]
Bulgaria	Country	2.1 [1.6; 2.8]	2.4 [1.7; 3.5]	1.8 [0.9; 3.8]	0.3 [-0.7; 1.5]	-0.6 [-2.0; 1.5]	-0.3 [-1.5; 1.8]	-0.65 [-2.31; 1.31]	3.80 [-6.68; 13.81]	0.43 [-1.97; 2.89]
Burkina Faso	Country	7.6 [5.9; 9.6]	4.2 [2.8; 6.2]	1.5 [0.7; 3.3]	-3.4 [-5.8; -0.8]†	-2.7 [-4.8; -0.4]†	-6.1 [-8.3; -3.7]†	2.42 [0.49; 4.18]†	12.84 [1.53; 23.68]†	4.94 [2.37; 7.34]†
Burundi	Country	2.9 [1.7; 4.7]	0.8 [0.4; 1.6]	0.5 [0.1; 2.4]	-2.1 [-4.0; -0.6]†	-0.2 [-1.2; 1.7]	-2.3 [-4.2; -0.2]†	5.17 [1.50; 8.87]†	4.71 [-16.91; 25.05]	5.06 [0.20; 9.77]†
Cabo Verde	Country	2.4 [0.6; 9.6]	0.9 [0.6; 1.3]	0.4 [0.1; 1.1]	-1.5 [-8.7; 0.3]	-0.5 [-1.0; 0.3]	-2.0 [-9.1; 0.0]†	4.29 [-1.73; 9.96]	9.84 [-3.79; 23.68]	5.63 [0.23; 10.81]†
Cambodia	Country	1.4 [0.9; 2.2]	0.4 [0.2; 0.8]	0.3 [0.1; 1.2]	-1.0 [-1.8; -0.4]†	0.0 [-0.5; 0.9]	-1.1 [-1.9; -0.1]†	5.80 [1.85; 9.09]†	0.85 [-18.17; 19.37]	4.60 [0.25; 8.44]†
Cameroon	Country	14.6 [11.9; 17.8]	5.3 [3.5; 8.0]	4.5 [1.8; 11.2]	-9.3 [-13.0; -5.6]†	-0.8 [-4.6; 5.8]	-10.1 [-14.3; -3.0]†	4.02 [2.22; 5.91]†	2.86 [-10.63; 14.36]	3.74 [0.74; 6.41]†
Canada	Country	0.3 [0.2; 0.5]	0.1 [0.1; 0.1]	0.0 [0.0; 0.2]	-0.3 [-0.5; -0.1]†	0.0 [-0.1; 0.1]	-0.3 [-0.5; -0.1]†	5.55 [2.89; 8.30]†	9.55 [-10.49; 26.90]	6.52 [1.82; 10.74]†
Central African Republic	Country	9.5 [6.9; 12.9]	10.2 [6.5; 15.6]	9.4 [4.5; 19.6]	0.7 [-4.4; 6.7]	-0.8 [-8.1; 10.0]	-0.1 [-6.0; 10.6]	-1.10 [-2.42; 1.92]	0.11 [-9.81; 11.93]	-0.80 [-2.41; 2.42]
Chad	Country	20.0 [15.6; 25.4]	9.1 [6.4; 12.9]	4.1 [1.9; 8.9]	-10.8 [-17.2; -5.1]†	-5.0 [-9.4; 0.4]	-15.9 [-21.9; -9.4]†	3.03 [1.43; 4.87]†	10.09 [-0.66; 20.81]	4.74 [2.30; 7.29]†
Chile	Country	1.3 [0.9; 1.8]	1.3 [0.8; 1.9]	0.1 [0.0; 0.1]	0.0 [-0.7; 0.7]	-1.2 [-1.8; -0.8]†	-1.2 [-1.8; -0.8]†	0.18 [-2.11; 2.22]	36.18 [26.80; 46.15]†	8.91 [6.64; 11.18]†
China	Country	0.1 [0.0; 4.0]	0.0 [0.0; 0.7]	0.0 [0.0; 0.7]	-0.1 [-3.8; 0.5]	0.0 [-0.7; 0.7]	-0.1 [-3.9; 0.5]	10.04 [-9.10; 22.61]	0.00 [-48.57; 47.62]	7.61 [-7.63; 17.27]
China, Hong Kong SAR	Country	0.1 [0.0; 0.1]	0.0 [0.0; 0.1]	0.0 [0.0; 0.1]	0.0 [-0.1; 0.0]	0.0 [-0.1; 0.0]	-0.1 [-0.1; 0.0]†	2.89 [-1.88; 6.84]	14.91 [-7.05; 25.88]	5.81 [0.31; 7.68]†
Macao, SAR of China	Country	0.1 [0.0; 0.3]	0.0 [0.0; 0.4]	0.0 [0.0; 0.3]	-0.1 [-0.2; 0.3]	0.0 [-0.4; 0.2]	-0.1 [-0.3; 0.2]	5.56 [-6.42; 11.74]	10.91 [-33.58; 43.28]	6.86 [-3.84; 9.82]
China, Taiwan Province of China	Country	0.5 [0.3; 0.8]	0.1 [0.0; 0.2]	0.0 [0.0; 0.4]	-0.4 [-0.8; -0.2]†	0.0 [-0.1; 0.3]	-0.4 [-0.8; -0.1]†	7.63 [3.29; 10.73]†	5.41 [-20.10; 30.80]	7.09 [0.48; 12.35]†
Colombia	Country	4.4 [3.0; 6.4]	3.8 [2.7; 5.4]	3.2 [1.6; 6.4]	-0.5 [-2.8; 1.6]	-0.6 [-2.9; 2.7]	-1.2 [-3.8; 2.2]	0.67 [-1.56; 2.59]	4.31 [-7.45; 11.93]	1.55 [-1.45; 3.38]
Comoros	Country	10.1 [6.9; 14.8]	1.8 [0.6; 5.8]	1.3 [0.2; 6.9]	-8.2 [-12.9; -3.3]†	-0.5 [-4.5; 4.9]	-8.8 [-13.5; -2.4]†	6.98 [1.96; 11.50]†	3.87 [-20.47; 29.82]	6.23 [1.04; 11.37]†
Republic of the Congo	Country	11.3 [8.0; 16.0]	5.7 [2.7; 11.8]	5.0 [1.5; 16.2]	-5.6 [-11.1; 1.2]	-0.7 [-7.8; 10.4]	-6.3 [-12.4; 5.1]	2.58 [-0.45; 5.90]	1.78 [-15.55; 19.26]	2.38 [-1.21; 6.20]
Costa Rica	Country	2.5 [1.7; 3.6]	2.4 [1.7; 3.6]	0.9 [0.4; 2.0]	0.0 [-1.4; 1.3]	-1.5 [-2.7; -0.2]†	-1.6 [-2.8; -0.3]†	0.48 [-1.99; 2.12]	12.05 [1.66; 22.96]†	3.28 [0.48; 5.56]†
Côte d'Ivoire	Country	14.3 [11.4; 18.0]	5.5 [3.7; 8.2]	3.0 [1.4; 6.5]	-8.8 [-13.0; -4.9]†	-2.5 [-5.7; 1.5]	-11.3 [-15.4; -6.8]†	3.78 [2.00; 5.70]†	8.06 [-3.52; 18.86]	4.82 [2.24; 7.28]†
Croatia	Country	0.1 [0.1; 0.2]	0.1 [0.0; 0.1]	0.0 [0.0; 0.1]	0.0 [-0.1; 0.0]	0.0 [-0.1; 0.0]	-0.1 [-0.1; 0.0]†	1.85 [-1.21; 4.65]	10.71 [-1.94; 22.86]	4.00 [0.76; 6.88]†
Cuba	Country	4.1 [2.6; 6.1]	1.3 [0.9; 2.0]	1.3 [0.6; 3.2]	-2.7 [-4.8; -1.1]†	0.0 [-1.0; 1.9]	-2.7 [-4.9; -0.4]†	4.24 [2.08; 6.77]†	-1.06 [-12.05; 11.85]	2.96 [0.38; 6.28]†
Curaçao	Country	0.8 [0.1; 5.8]	0.6 [0.4; 1.0]	0.2 [0.0; 1.2]	-0.3 [-5.0; 0.6]	-0.4 [-0.8; 0.6]	-0.7 [-5.5; 0.5]	1.51 [-6.42; 9.28]	14.88 [-9.79; 41.62]	4.75 [-3.28; 13.07]
Cyprus	Country	0.6 [0.0; 5.7]	0.1 [0.0; 0.2]	0.1 [0.0; 0.7]	-0.5 [-5.6; 0.1]	0.1 [-0.1; 0.6]	-0.4 [-5.6; 0.3]	9.12 [-2.37; 19.53]	-9.69 [-34.72; 17.22]	4.56 [-4.30; 13.47]
Czechia	Country	0.1 [0.0; 0.1]	0.1 [0.0; 0.1]	0.1 [0.0; 0.3]	0.0 [0.0; 0.1]	0.0 [-0.1; 0.2]	0.0 [0.0; 0.2]	-0.91 [-3.06; 2.04]	-1.41 [-19.99; 15.42]	-1.03 [-5.10; 3.13]
Dem. People's Republic of Korea	Country	0.0 [0.0; 0.2]	0.0 [0.0; 0.0]	0.0 [0.0; 0.0]	0.0 [-0.2; 0.0]†	0.0 [0.0; 0.0]	0.0 [-0.2; 0.0]†	0.00 [0.00; 11.81]	0.00 [0.00; 0.00]	0.00 [0.00; 8.95]
Democratic Republic of the Congo	Country	11.7 [8.5; 16.0]	4.8 [2.7; 8.4]	4.5 [1.6; 13.0]	-6.9 [-11.7; -2.2]†	-0.3 [-4.9; 8.3]	-7.2 [-12.4; 1.8]	3.58 [1.00; 6.22]†	1.44 [-14.32; 15.35]	3.06 [-0.48; 6.17]
Denmark	Country	0.0 [0.0; 0.0]	0.0 [0.0; 0.0]	0.0 [0.0; 0.0]	0.0 [0.0; 0.0]	0.0 [0.0; 0.0]	0.0 [0.0; 0.0]	1.10 [-2.23; 4.27]	4.48 [-13.37; 12.16]	1.92 [-2.08; 3.62]
Djibouti	Country	3.5 [1.9; 6.4]	0.2 [0.0; 1.2]	0.2 [0.0; 1.9]	-3.3 [-6.2; -1.5]†	0.0 [-1.0; 1.6]	-3.4 [-6.2; -1.1]†	11.44 [4.05; 19.13]†	3.44 [-34.94; 40.19]	9.50 [1.65; 16.99]†
Dominican Republic	Country	7.6 [5.7; 10.1]	2.8 [2.0; 3.8]	0.9 [0.5; 1.7]	-4.8 [-7.4; -2.6]†	-1.9 [-3.0; -0.8]†	-6.7 [-9.2; -4.6]†	4.16 [2.31; 5.74]†	13.87 [4.83; 23.52]†	6.51 [4.29; 8.69]†
Ecuador	Country	4.2 [2.7; 6.4]	4.4 [3.2; 6.0]	2.8 [1.3; 5.9]	0.2 [-2.3; 2.4]	-1.6 [-3.8; 1.7]	-1.3 [-4.1; 2.0]	-0.32 [-2.36; 1.93]	8.33 [-4.52; 15.84]	1.78 [-1.38; 3.77]

†: Change is significantly different from zero.
Total Change (Year 1–Year 2): ASFR (Year 2) - ASFR (Year 1).
Annual Rate of Reduction (Year 1–Year 2): $\log(\text{ASFR}_{\text{Year 2}}/\text{ASFR}_{\text{Year 1}})/(\text{Year 1} - \text{Year 2})$, where Year 1 is smaller than Year 2.

Continued on next page

Table 13 – continued from previous page

Age-specific fertility rate 10–14										
		Estimate (per 1,000)			Total Change (per 1,000)			Annual Rate of Reduction (per 100)		
**Location Name**	**Location Type**	**1990**	**2015**	**2023**	**1990–2015**	**2015–2023**	**1990–2023**	**1990–2015**	**2015–2023**	**1990–2023**
Egypt	Country	2.4<br>[1.8; 3.4]	0.7<br>[0.2; 2.4]	0.7<br>[0.1; 3.6]	-1.7<br>[-2.8; 0.1]	-0.1<br>[-1.7; 2.9]	-1.8<br>[-2.9; 1.2]	4.89<br>[-0.13; 9.46]	1.11<br>[-24.12; 26.49]	3.97<br>[-1.23; 9.02]
El Salvador	Country	3.0<br>[2.4; 3.9]	2.9<br>[2.0; 4.2]	1.7<br>[0.5; 5.1]	-0.1<br>[-1.4; 1.4]	-1.2<br>[-3.0; 2.3]	-1.4<br>[-2.9; 2.2]	0.64<br>[-1.69; 1.99]	5.83<br>[-7.96; 21.49]	1.90<br>[-1.73; 5.35]
Equatorial Guinea	Country	15.4<br>[2.8; 83.0]	18.0<br>[3.4; 91.4]	16.0<br>[2.6; 89.0]	2.6<br>[-61.0; 73.6]	-2.0<br>[-73.7; 66.3]	0.6<br>[-64.7; 70.5]	-0.78<br>[-9.97; 9.01]	2.00<br>[-28.50; 31.12]	-0.11<br>[-7.59; 7.09]
Eritrea	Country	6.8<br>[4.6; 10.2]	1.3<br>[0.4; 4.8]	1.3<br>[0.3; 6.7]	-5.5<br>[-9.0; -1.5]†	0.0<br>[-3.6; 5.4]	-5.5<br>[-9.1; 0.1]	6.41<br>[1.17; 11.93]†	0.84<br>[-25.23; 26.71]	5.06<br>[-0.08; 10.18]
Estonia	Country	0.1<br>[0.0; 0.1]	0.1<br>[0.0; 0.1]	0.0<br>[0.0; 0.1]	0.0<br>[-0.1; 0.1]	0.0<br>[-0.1; 0.1]	0.0<br>[-0.1; 0.1]	-0.06<br>[-2.48; 2.72]	11.04<br>[-8.68; 25.91]	2.63<br>[-1.97; 6.32]
Eswatini	Country	8.6<br>[6.2; 11.8]	1.1<br>[0.4; 3.1]	1.0<br>[0.2; 4.2]	-7.4<br>[-10.7; -4.5]†	-0.2<br>[-2.3; 3.2]	-7.6<br>[-10.9; -3.8]†	8.26<br>[3.87; 12.19]†	2.02<br>[-20.11; 23.85]	6.75<br>[2.10; 11.24]†
Ethiopia	Country	15.7<br>[12.3; 20.0]	1.9<br>[1.1; 3.3]	0.7<br>[0.2; 2.6]	-13.9<br>[-18.1; -10.2]†	-1.1<br>[-2.6; 0.8]	-15.0<br>[-19.3; -11.3]†	8.59<br>[6.09; 10.97]†	12.12<br>[-5.83; 28.79]	9.44<br>[5.45; 13.16]†
Fiji	Country	0.5<br>[0.2; 1.1]	0.0<br>[0.0; 0.1]	0.0<br>[0.0; 0.0]	-0.5<br>[-1.0; -0.2]†	0.0<br>[-0.1; 0.0]	-0.5<br>[-1.0; -0.2]†	11.82<br>[6.95; 16.23]†	12.34<br>[-10.15; 21.67]	11.95<br>[6.51; 13.89]†
Finland	Country	0.0<br>[0.0; 0.0]	0.0<br>[0.0; 0.0]	0.0<br>[0.0; 0.0]	0.0<br>[0.0; 0.0]	0.0<br>[0.0; 0.0]	0.0<br>[0.0; 0.0]	0.18<br>[-2.91; 3.42]	11.06<br>[-7.88; 17.77]	2.82<br>[-1.54; 4.35]
France	Country	0.1<br>[0.0; 0.1]	0.1<br>[0.1; 0.2]	0.1<br>[0.0; 0.2]	0.1<br>[0.0; 0.1]	-0.1<br>[-0.1; 0.1]	0.0<br>[-0.1; 0.2]	-2.49<br>[-5.40; 0.29]	8.72<br>[-11.17; 27.06]	0.23<br>[-4.53; 4.70]
French Guiana	Country	4.6<br>[2.2; 9.7]	2.4<br>[1.7; 3.6]	1.3<br>[0.5; 2.9]	-2.2<br>[-7.2; 0.6]	-1.2<br>[-2.6; 0.7]	-3.3<br>[-8.3; -0.4]†	2.58<br>[-0.88; 5.87]	8.18<br>[-3.38; 19.80]	3.94<br>[0.47; 7.22]†
French Polynesia	Country	1.5<br>[0.2; 10.4]	0.3<br>[0.1; 0.8]	0.1<br>[0.0; 0.3]	-1.2<br>[-10.0; 0.2]	-0.2<br>[-0.7; 0.1]	-1.4<br>[-10.2; -0.1]†	6.28<br>[-2.55; 14.72]	13.51<br>[-3.94; 29.68]	8.03<br>[1.00; 14.46]†
Gabon	Country	14.1<br>[10.7; 18.5]	4.3<br>[2.5; 7.5]	3.4<br>[1.3; 8.9]	-9.8<br>[-14.4; -5.2]†	-0.9<br>[-4.6; 4.9]	-10.7<br>[-15.5; -4.3]†	4.80<br>[2.28; 7.16]†	2.60<br>[-10.82; 16.45]	4.27<br>[1.23; 7.28]†
Gambia	Country	16.6<br>[12.6; 21.8]	2.2<br>[1.2; 4.0]	0.9<br>[0.3; 3.0]	-14.3<br>[-19.6; -9.9]†	-1.3<br>[-3.3; 1.0]	-15.7<br>[-20.8; -11.2]†	8.11<br>[5.42; 10.65]†	11.23<br>[-5.58; 28.45]	8.87<br>[5.08; 12.72]†
Georgia	Country	0.3<br>[0.1; 1.2]	0.2<br>[0.1; 0.3]	0.1<br>[0.0; 0.2]	0.0<br>[-1.0; 0.2]	-0.2<br>[-0.3; 0.0]†	-0.2<br>[-1.1; 0.0]	0.83<br>[-5.58; 7.15]	16.45<br>[1.48; 32.37]†	4.61<br>[-1.08; 10.35]
Germany	Country	0.1<br>[0.1; 0.2]	0.1<br>[0.1; 0.2]	0.1<br>[0.0; 0.2]	0.0<br>[-0.1; 0.1]	-0.1<br>[-0.2; 0.1]	-0.1<br>[-0.2; 0.1]	0.50<br>[-2.86; 3.38]	9.25<br>[-9.66; 28.04]	2.62<br>[-2.08; 7.08]
Ghana	Country	6.9<br>[5.2; 9.1]	1.8<br>[1.1; 3.0]	1.7<br>[0.6; 4.5]	-5.1<br>[-7.5; -2.9]†	-0.1<br>[-1.7; 2.8]	-5.2<br>[-7.6; -2.0]†	5.51<br>[2.97; 7.80]†	0.49<br>[-13.38; 14.56]	4.30<br>[1.22; 7.28]†
Greece	Country	0.2<br>[0.2; 0.4]	0.3<br>[0.2; 0.6]	0.4<br>[0.1; 1.6]	0.1<br>[-0.1; 0.3]	0.1<br>[-0.3; 1.3]	0.2<br>[-0.2; 1.3]	-0.76<br>[-3.76; 2.29]	-3.24<br>[-22.82; 16.30]	-1.36<br>[-5.85; 3.00]
Grenada	Country	6.9<br>[3.8; 12.5]	0.7<br>[0.4; 1.2]	0.5<br>[0.1; 2.2]	-6.2<br>[-11.8; -3.0]†	-0.1<br>[-0.8; 1.4]	-6.4<br>[-11.9; -2.9]†	9.28<br>[5.98; 12.44]†	2.83<br>[-15.88; 21.74]	7.71<br>[3.16; 12.30]†
Guadeloupe	Country	0.7<br>[0.3; 1.6]	0.2<br>[0.0; 1.0]	0.1<br>[0.0; 0.5]	-0.5<br>[-1.4; 0.4]	-0.1<br>[-0.9; 0.3]	-0.6<br>[-1.5; -0.1]†	4.23<br>[-2.52; 11.52]	8.87<br>[-17.87; 35.93]	5.36<br>[0.43; 10.94]†
Guam	Country	0.8<br>[0.3; 1.7]	0.2<br>[0.1; 0.3]	0.1<br>[0.0; 0.6]	-0.6<br>[-1.5; -0.1]†	-0.1<br>[-0.2; 0.4]	-0.6<br>[-1.5; 0.0]†	5.24<br>[1.36; 8.93]†	3.63<br>[-13.17; 21.85]	4.85<br>[0.19; 9.79]†
Guatemala	Country	5.5<br>[4.3; 7.1]	4.0<br>[2.9; 5.6]	3.1<br>[1.5; 6.4]	-1.5<br>[-3.5; 0.6]	-0.9<br>[-3.2; 2.4]	-2.4<br>[-4.7; 1.0]	0.93<br>[-0.44; 2.98]	2.96<br>[-6.69; 13.37]	1.42<br>[-0.56; 4.08]
Guinea	Country	18.7<br>[15.2; 23.2]	7.9<br>[5.6; 11.1]	5.1<br>[2.2; 11.7]	-10.8<br>[-15.9; -6.1]†	-2.8<br>[-7.1; 4.2]	-13.6<br>[-18.8; -6.1]†	3.48<br>[1.87; 5.07]†	5.33<br>[-5.71; 16.40]	3.93<br>[1.31; 6.51]†
Guinea-Bissau	Country	13.0<br>[8.4; 19.5]	3.6<br>[1.9; 6.6]	2.2<br>[0.6; 6.8]	-9.4<br>[-16.1; -4.0]†	-1.4<br>[-4.8; 3.4]	-10.8<br>[-17.4; -4.4]†	5.08<br>[2.16; 8.20]†	6.27<br>[-9.57; 23.11]	5.37<br>[1.65; 9.37]†
Guyana	Country	3.2<br>[2.0; 5.2]	1.1<br>[0.5; 2.4]	0.9<br>[0.2; 3.1]	-2.1<br>[-4.1; -0.4]†	-0.2<br>[-1.7; 1.9]	-2.4<br>[-4.4; 0.0]	4.45<br>[0.71; 7.82]†	2.28<br>[-14.82; 22.75]	3.93<br>[-0.01; 8.31]
Haiti	Country	4.1<br>[2.8; 6.0]	2.8<br>[1.3; 6.2]	2.3<br>[0.5; 10.6]	-1.3<br>[-3.7; 2.4]	-0.5<br>[-4.3; 7.6]	-1.8<br>[-4.5; 6.5]	6.00<br>[-2.05; 4.94]	3.70<br>[-18.54; 23.59]	5.45<br>[-2.92; 6.53]
Honduras	Country	5.5<br>[3.6; 8.4]	2.4<br>[0.5; 10.5]	1.8<br>[0.3; 11.1]	-3.1<br>[-6.6; 5.0]	-0.6<br>[-8.7; 8.9]	-3.7<br>[-7.0; 5.5]	3.61<br>[-2.78; 9.41]	3.39<br>[-26.09; 33.11]	3.55<br>[-2.19; 9.00]
Hungary	Country	0.7<br>[0.5; 1.0]	0.4<br>[0.3; 0.7]	0.3<br>[0.1; 0.9]	-0.3<br>[-0.6; 0.0]	-0.1<br>[-0.4; 0.5]	-0.4<br>[-0.8; 0.2]	1.94<br>[-0.12; 4.56]	4.27<br>[-11.19; 18.75]	2.50<br>[-0.84; 6.03]
Iceland	Country	0.2<br>[0.1; 0.4]	0.1<br>[0.1; 0.3]	0.0<br>[0.0; 0.3]	-0.1<br>[-0.3; 0.1]	-0.1<br>[-0.2; 0.2]	-0.2<br>[-0.3; 0.1]	2.54<br>[-1.61; 6.40]	13.41<br>[-15.49; 36.82]	5.18<br>[-1.43; 10.08]
India	Country	8.8<br>[6.9; 11.2]	0.3<br>[0.2; 0.7]	0.2<br>[0.0; 1.6]	-8.5<br>[-10.9; -6.5]†	-0.1<br>[-0.5; 1.2]	-8.6<br>[-11.0; -6.3]†	13.30<br>[9.86; 16.30]†	28.25<br>[-21.40; 33.08]	16.93<br>[5.19; 17.55]†
Indonesia	Country	4.2<br>[3.2; 5.5]	0.4<br>[0.2; 0.8]	0.2<br>[0.0; 1.0]	-3.9<br>[-5.1; -2.8]†	-0.2<br>[-0.7; 0.6]	-4.1<br>[-5.3; -2.8]†	9.71<br>[6.37; 12.94]†	9.47<br>[-13.94; 33.69]	9.65<br>[4.24; 14.99]†
Iran (Islamic Republic of)	Country	1.5<br>[0.8; 2.5]	0.6<br>[0.3; 1.0]	0.4<br>[0.1; 1.1]	-0.9<br>[-2.0; -0.2]†	-0.2<br>[-0.7; 0.6]	-1.1<br>[-2.2; -0.2]†	4.27<br>[0.68; 7.09]†	4.02<br>[-10.25; 19.45]	4.21<br>[0.53; 7.59]†
Iraq	Country	2.4<br>[1.4; 4.0]	2.3<br>[1.1; 4.7]	1.2<br>[0.3; 4.6]	0.0<br>[-2.0; 2.5]	-1.1<br>[-3.6; 2.5]	-1.2<br>[-3.0; 2.3]	0.06<br>[-3.34; 3.55]	7.87<br>[-11.42; 27.15]	1.96<br>[-2.31; 6.49]
Ireland	Country	0.1<br>[0.0; 0.1]	0.0<br>[0.0; 0.1]	0.0<br>[0.0; 0.0]	0.0<br>[-0.1; 0.0]	0.0<br>[0.0; 0.0]	-0.1<br>[-0.1; 0.0]†	2.77<br>[-0.05; 5.71]	14.94<br>[-3.83; 20.30]	5.72<br>[1.29; 7.00]†
Israel	Country	0.1<br>[0.1; 0.1]	0.0<br>[0.0; 0.0]	0.0<br>[0.0; 0.0]	-0.1<br>[-0.1; -0.1]†	0.0<br>[0.0; 0.0]	-0.1<br>[-0.1; -0.1]†	8.54<br>[6.85; 10.41]†	0.00<br>[0.00; 0.00]	6.47<br>[5.14; 7.88]†
Italy	Country	0.0<br>[0.0; 0.0]	0.0<br>[0.0; 0.0]	0.0<br>[0.0; 0.0]	0.0<br>[0.0; 0.0]	0.0<br>[0.0; 0.0]	0.0<br>[0.0; 0.0]	-0.55<br>[-2.80; 1.62]	1.72<br>[-9.48; 9.33]	0.00<br>[-2.64; 1.49]
Jamaica	Country	2.9<br>[1.9; 4.6]	0.8<br>[0.2; 3.7]	0.5<br>[0.1; 3.9]	-2.1<br>[-3.9; 0.9]	-0.4<br>[-3.1; 3.0]	-2.5<br>[-4.2; 1.1]	5.08<br>[-1.23; 11.08]	7.53<br>[-24.31; 38.38]	5.68<br>[-1.06; 11.77]
Japan	Country	0.0<br>[0.0; 0.0]	0.0<br>[0.0; 0.0]	0.0<br>[0.0; 0.1]	0.0<br>[0.0; 0.0]	0.0<br>[0.0; 0.1]	0.0<br>[0.0; 0.1]	-2.69<br>[-5.01; 0.87]	4.12<br>[-17.81; 14.67]	-1.04<br>[-5.84; 1.56]
Jersey	Country	0.2<br>[0.1; 0.3]	0.1<br>[0.1; 0.1]	0.0<br>[0.0; 0.1]	-0.1<br>[-0.2; 0.0]†	-0.1<br>[-0.1; 0.0]	-0.2<br>[-0.3; -0.1]†	3.17<br>[0.65; 5.65]†	13.53<br>[-3.53; 28.72]	5.68<br>[1.62; 9.36]†
Jordan	Country	0.3<br>[0.2; 0.5]	0.1<br>[0.1; 0.4]	0.1<br>[0.0; 0.5]	-0.2<br>[-0.3; 0.1]	-0.1<br>[-0.3; 0.3]	-0.2<br>[-0.4; 0.2]	3.09<br>[-1.28; 7.04]	8.94<br>[-18.14; 33.63]	4.51<br>[-1.82; 9.97]
Kazakhstan	Country	0.0<br>[0.0; 0.0]	0.0<br>[0.0; 0.0]	0.0<br>[0.0; 0.1]	0.0<br>[0.0; 0.0]	0.0<br>[0.0; 0.1]	0.0<br>[0.0; 0.1]	-2.86<br>[-5.40; 1.29]	5.23<br>[-15.62; 16.59]	-0.90<br>[-5.65; 2.64]
Kenya	Country	7.0<br>[5.5; 9.0]	1.9<br>[1.3; 3.0]	0.8<br>[0.4; 1.6]	-5.1<br>[-7.1; -3.3]†	-1.1<br>[-2.2; -0.1]†	-6.2<br>[-8.1; -4.6]†	5.14<br>[3.24; 7.05]†	11.31<br>[1.16; 20.30]†	6.64<br>[4.37; 8.57]†
Kiribati	Country	1.9<br>[0.9; 4.0]	1.3<br>[0.5; 3.6]	1.3<br>[0.3; 6.2]	-0.6<br>[-2.8; 1.9]	0.0<br>[-2.5; 4.8]	-0.6<br>[-2.9; 4.5]	1.84<br>[-3.41; 6.50]	-0.56<br>[-22.91; 23.56]	1.26<br>[-4.11; 6.45]
Kosovo (under UNSC res. 1244)	Country	0.0<br>[0.0; 0.1]	0.0<br>[0.0; 0.1]	0.0<br>[0.0; 0.1]	0.0<br>[0.0; 0.0]	0.0<br>[0.0; 0.1]	0.0<br>[0.0; 0.1]	2.36<br>[-2.28; 5.67]	-3.86<br>[-26.80; 15.67]	0.85<br>[-4.90; 4.59]
Kuwait	Country	0.0<br>[0.0; 0.0]	0.0<br>[0.0; 0.0]	0.0<br>[0.0; 0.0]	0.0<br>[0.0; 0.0]†	0.0<br>[0.0; 0.0]	0.0<br>[0.0; 0.0]†	0.00<br>[0.00; 0.00]	0.00<br>[0.00; 0.00]	0.00<br>[0.00; 0.00]

†: Change is significantly different from zero.
Total Change (Year 1–Year 2): ASFR (Year 2) - ASFR (Year 1).
Annual Rate of Reduction (Year 1–Year 2): $\log(\text{ASFR}_{\text{Year 2}}/\text{ASFR}_{\text{Year 1}})/(\text{Year 1} - \text{Year 2})$, where Year 1 is smaller than Year 2.

Continued on next page

Table 13 – continued from previous page

Age-specific fertility rate 10–14										
		Estimate (per 1,000)			Total Change (per 1,000)			Annual Rate of Reduction (per 100)		
**Location Name**	**Location Type**	**1990**	**2015**	**2023**	**1990–2015**	**2015–2023**	**1990–2023**	**1990–2015**	**2015–2023**	**1990–2023**
Kyrgyz Republic	Country	0.0 [0.0; 0.0]	0.0 [0.0; 0.0]	0.0 [0.0; 0.0]	0.0 [0.0; 0.0]†	0.0 [0.0; 0.0]	0.0 [0.0; 0.0]	3.03 [0.01; 5.74]†	-1.42 [-16.48; 1.61]	1.95 [-2.23; 4.06]
Lao People's Democratic Republic	Country	9.1 [5.7; 14.4]	3.1 [1.6; 6.2]	3.0 [0.9; 10.4]	-6.0 [-11.6; -1.4]†	-0.1 [-3.9; 7.2]	-6.1 [-11.9; 1.5]	4.49 [0.92; 7.63]†	0.23 [-17.30; 18.51]	3.46 [-0.54; 7.21]
Latvia	Country	0.1 [0.0; 0.3]	0.1 [0.1; 0.2]	0.0 [0.0; 0.2]	0.0 [-0.1; 0.1]	-0.1 [-0.1; 0.1]	0.0 [-0.2; 0.1]	-0.98 [-5.59; 3.67]	12.10 [-6.25; 28.57]	2.19 [-3.06; 6.99]
Lebanon	Country	0.4 [0.2; 0.8]	0.0 [0.0; 0.0]	0.0 [0.0; 0.1]	-0.4 [-0.8; -0.2]†	0.0 [0.0; 0.1]	-0.4 [-0.8; -0.1]†	14.53 [8.47; 17.36]†	0.00 [-26.50; 16.19]	11.01 [3.99; 13.05]†
Lesotho	Country	1.9 [1.2; 3.0]	0.8 [0.4; 1.7]	0.7 [0.2; 2.6]	-1.1 [-2.3; 0.0]	-0.1 [-1.1; 1.9]	-1.2 [-2.4; 0.8]	3.60 [-0.07; 7.10]	1.20 [-18.06; 20.09]	3.02 [-1.28; 7.26]
Liberia	Country	16.5 [13.3; 20.6]	5.4 [3.7; 7.9]	4.5 [2.1; 9.6]	-11.1 [-15.5; -7.0]†	-0.9 [-4.4; 4.3]	-12.0 [-16.9; -6.0]†	4.48 [2.75; 6.19]†	2.62 [-8.22; 12.69]	4.03 [1.51; 6.27]†
Libya	Country	0.0 [0.0; 0.1]	0.0 [0.0; 0.0]	0.0 [0.0; 0.1]	0.0 [-0.1; 0.0]†	0.0 [0.0; 0.1]†	0.0 [-0.1; 0.0]	4.23 [0.00; 8.57]	0.00 [-20.94; 0.00]	3.20 [-2.25; 6.37]
Lithuania	Country	0.1 [0.1; 0.2]	0.1 [0.1; 0.2]	0.0 [0.0; 0.1]	0.0 [-0.1; 0.1]	-0.1 [-0.2; 0.0]†	-0.1 [-0.2; 0.0]†	0.47 [-2.44; 2.92]	19.74 [2.49; 35.56]†	5.14 [0.86; 8.78]†
Luxembourg	Country	0.1 [0.0; 0.1]	0.1 [0.1; 0.2]	0.1 [0.0; 0.5]	0.1 [0.0; 0.2]	-0.1 [-0.2; 0.4]	0.0 [-0.1; 0.5]	-2.84 [-6.88; 0.66]	7.46 [-20.54; 32.47]	-0.34 [-7.22; 5.41]
Madagascar	Country	10.3 [8.1; 13.1]	11.6 [8.1; 16.2]	9.7 [4.7; 19.8]	1.2 [-3.2; 6.4]	-1.9 [-8.7; 8.4]	-0.7 [-6.4; 9.6]	-0.38 [-2.14; 1.21]	2.34 [-7.39; 12.04]	0.28 [-2.05; 2.52]
Malawi	Country	10.3 [8.4; 12.8]	2.6 [1.8; 4.0]	2.0 [0.8; 4.8]	-7.7 [-10.3; -5.3]†	-0.6 [-2.5; 2.2]	-8.3 [-11.1; -4.9]†	5.43 [3.59; 7.32]†	3.98 [-8.62; 15.88]	5.08 [2.21; 7.81]†
Malaysia	Country	0.3 [0.0; 2.2]	0.1 [0.1; 0.3]	0.1 [0.0; 0.3]	-0.1 [-2.0; 0.2]	-0.1 [-0.2; 0.2]	-0.2 [-2.1; 0.2]	2.75 [-5.91; 10.92]	7.59 [-11.46; 26.20]	3.92 [-3.63; 11.33]
Maldives	Country	4.1 [2.8; 5.8]	0.0 [0.0; 0.0]	0.0 [0.0; 0.1]	-4.1 [-5.8; -2.8]†	0.0 [0.0; 0.1]	-4.1 [-5.8; -2.8]†	22.46 [18.04; 24.93]†	0.43 [-25.84; 15.24]	17.12 [10.64; 19.07]†
Mali	Country	16.6 [13.8; 20.0]	9.1 [6.6; 12.8]	6.4 [3.1; 13.1]	-7.5 [-11.6; -2.7]†	-2.8 [-7.7; 4.4]	-10.2 [-15.0; -2.9]†	2.32 [0.81; 3.88]†	4.69 [-5.37; 14.12]	2.90 [0.61; 5.12]†
Malta	Country	0.3 [0.1; 2.0]	0.3 [0.1; 0.5]	0.1 [0.0; 0.4]	-0.1 [-1.8; 0.3]	-0.2 [-0.4; 0.1]	-0.3 [-1.9; 0.1]	1.31 [-6.26; 8.70]	13.55 [-5.82; 35.14]	4.28 [-2.69; 11.35]
Martinique	Country	0.5 [0.3; 0.8]	0.1 [0.0; 0.5]	0.1 [0.0; 0.3]	-0.4 [-0.7; 0.1]	-0.1 [-0.4; 0.2]	-0.4 [-0.8; -0.1]†	5.59 [-0.47; 11.71]	8.88 [-19.33; 35.24]	6.39 [1.14; 11.54]†
Mauritania	Country	19.5 [14.1; 26.9]	13.0 [8.3; 20.3]	13.2 [5.2; 33.3]	-6.5 [-15.5; 2.5]	0.1 [-10.7; 21.0]	-6.3 [-17.3; 14.4]	2.37 [-0.59; 3.85]	5.54 [-13.37; 12.75]	3.14 [-1.79; 4.14]
Mauritius	Country	0.5 [0.3; 0.9]	0.6 [0.3; 1.0]	0.6 [0.2; 1.5]	0.1 [-0.4; 0.5]	0.0 [-0.5; 0.9]	0.1 [-0.4; 1.0]	-0.51 [-3.84; 2.42]	0.91 [-13.55; 13.32]	-0.16 [-3.85; 2.79]
Mayotte	Country	9.3 [1.3; 63.9]	2.3 [0.2; 29.9]	1.8 [0.1; 29.5]	-7.0 [-59.1; 21.7]	-0.5 [-26.8; 26.3]	-7.5 [-59.8; 20.8]	5.55 [-7.56; 18.49]	3.39 [-46.09; 51.33]	5.03 [-5.48; 15.02]
Mexico	Country	1.9 [0.8; 4.7]	1.4 [1.0; 2.0]	0.9 [0.3; 2.6]	-0.5 [-3.4; 0.8]	-0.5 [-1.4; 1.2]	-1.0 [-3.7; 1.0]	1.22 [-2.55; 5.20]	5.71 [-8.03; 20.23]	2.31 [-1.91; 6.61]
Micronesia (Fed. States of)	Country	0.8 [0.3; 2.5]	1.0 [0.2; 4.5]	0.9 [0.1; 6.3]	0.2 [-1.6; 3.7]	-0.1 [-3.6; 5.0]	0.1 [-1.6; 5.5]	-0.87 [-8.28; 6.46]	1.86 [-30.05; 32.11]	-0.21 [-7.13; 6.37]
Mongolia	Country	0.3 [0.1; 0.7]	0.0 [0.0; 0.0]	0.1 [0.0; 0.2]	-0.3 [-0.7; -0.1]†	0.1 [0.0; 0.2]†	-0.2 [-0.6; 0.0]†	12.56 [7.11; 16.28]†	-24.81 [-36.29; -5.90]†	3.50 [0.13; 7.51]†
Montenegro	Country	0.1 [0.0; 0.4]	0.1 [0.1; 0.3]	0.1 [0.0; 0.4]	0.0 [-0.3; 0.2]	0.0 [-0.2; 0.2]	0.0 [-0.3; 0.2]	-0.05 [-5.62; 5.17]	5.10 [-12.65; 22.98]	1.20 [-4.41; 6.39]
Morocco	Country	0.7 [0.4; 1.2]	0.1 [0.0; 0.8]	0.1 [0.0; 1.3]	-0.6 [-1.1; 0.1]	0.0 [-0.6; 1.2]	-0.6 [-1.1; 0.6]	6.76 [-0.68; 14.55]	0.66 [-35.80; 37.08]	5.28 [-1.94; 12.33]
Mozambique	Country	18.6 [14.4; 24.0]	8.2 [5.3; 12.6]	7.8 [4.0; 15.6]	-10.4 [-16.5; -4.3]†	-0.3 [-6.2; 7.9]	-10.8 [-17.4; -2.2]†	3.41 [1.25; 5.34]†	0.30 [-9.52; 10.64]	2.65 [0.40; 4.77]†
Myanmar	Country	1.5 [0.8; 3.1]	0.6 [0.2; 2.1]	0.6 [0.1; 4.0]	-0.9 [-2.5; 0.8]	0.0 [-1.6; 3.3]	-0.9 [-2.6; 2.6]	3.60 [-2.22; 9.05]	-0.23 [-27.55; 28.32]	2.67 [-3.33; 8.67]
Namibia	Country	3.6 [2.5; 5.2]	2.5 [1.0; 6.5]	2.3 [0.5; 10.1]	-1.1 [-3.4; 3.0]	-0.2 [-4.5; 7.8]	-1.3 [-3.8; 6.3]	1.58 [-2.64; 5.65]	0.03 [-21.42; 22.59]	1.21 [-3.19; 5.91]
Nepal	Country	3.0 [2.2; 4.0]	1.3 [0.8; 2.1]	0.4 [0.2; 1.3]	-1.7 [-2.8; -0.6]†	-0.8 [-1.7; 0.1]	-2.5 [-3.6; -1.4]†	3.66 [1.05; 5.58]†	13.05 [-1.46; 27.89]	5.94 [2.40; 9.07]†
Netherlands	Country	0.1 [0.0; 0.1]	0.0 [0.0; 0.1]	0.0 [0.0; 0.1]	0.0 [-0.1; 0.0]	0.0 [0.0; 0.0]	0.0 [-0.1; 0.0]	3.04 [-1.14; 7.18]	11.19 [-12.76; 20.32]	5.02 [-0.80; 7.04]
New Caledonia	Country	0.4 [0.2; 1.1]	0.0 [0.0; 0.4]	0.0 [0.0; 0.2]	-0.4 [-1.0; 0.0]†	0.0 [-0.3; 0.2]	-0.4 [-1.1; -0.1]†	7.40 [0.25; 17.07]†	7.54 [-26.13; 42.18]	7.44 [2.44; 13.90]†
New Zealand	Country	0.8 [0.5; 1.2]	0.2 [0.1; 0.3]	0.1 [0.0; 0.2]	-0.6 [-1.1; -0.3]†	-0.1 [-0.2; 0.1]	-0.7 [-1.1; -0.4]†	6.30 [3.41; 9.20]†	9.26 [-5.60; 24.23]	7.02 [3.44; 10.65]†
Nicaragua	Country	8.1 [6.3; 10.4]	6.0 [2.5; 13.5]	5.5 [1.6; 18.2]	-2.1 [-6.2; 5.5]	-0.5 [-9.1; 12.8]	-2.6 [-7.2; 9.9]	1.92 [-2.15; 4.69]	0.69 [-17.49; 19.89]	1.62 [-2.55; 5.01]
Niger	Country	14.9 [12.2; 18.3]	7.0 [4.2; 11.5]	3.5 [1.7; 7.2]	-8.0 [-12.4; -2.6]†	-3.5 [-8.3; 1.2]	-11.4 [-15.2; -6.9]†	2.99 [0.84; 5.24]†	8.82 [-2.64; 19.70]	4.40 [2.13; 6.69]†
Nigeria	Country	15.9 [13.0; 19.6]	4.9 [3.4; 7.0]	2.5 [1.1; 5.9]	-11.1 [-14.9; -7.5]†	-2.4 [-4.9; 1.3]	-13.4 [-17.2; -9.0]†	4.75 [3.10; 6.36]†	8.48 [-3.34; 19.59]	5.65 [2.96; 8.17]†
North Macedonia	Country	0.4 [0.3; 0.6]	0.4 [0.2; 0.7]	0.3 [0.1; 0.9]	0.0 [-0.3; 0.3]	-0.1 [-0.4; 0.5]	-0.1 [-0.4; 0.5]	0.26 [-2.80; 3.15]	1.94 [-11.50; 16.77]	0.67 [-2.54; 4.15]
Norway	Country	0.0 [0.0; 0.1]	0.0 [0.0; 0.0]	0.0 [0.0; 0.0]	0.0 [0.0; 0.0]†	0.0 [0.0; 0.0]	0.0 [-0.1; 0.0]†	4.30 [0.62; 6.50]†	1.37 [-9.39; 11.46]	3.59 [0.62; 5.12]†
Oman	Country	22.3 [7.7; 62.1]	0.0 [0.0; 0.1]	0.0 [0.0; 0.1]	-22.2 [-62.0; -7.7]†	0.0 [-0.1; 0.1]	-22.2 [-62.0; -7.6]†	25.93 [18.76; 32.12]†	-0.26 [-22.49; 24.65]	19.58 [14.40; 24.61]†
Pakistan	Country	4.7 [3.5; 6.4]	0.6 [0.3; 1.1]	0.4 [0.1; 2.0]	-4.2 [-5.8; -2.8]†	-0.1 [-0.8; 1.5]	-4.3 [-5.9; -2.4]†	8.62 [5.30; 11.52]†	3.36 [-17.61; 23.72]	7.34 [2.51; 11.73]†
Panama	Country	3.2 [2.4; 4.4]	3.5 [2.5; 4.9]	1.8 [0.8; 3.8]	0.2 [-1.4; 1.9]	-1.7 [-3.4; 0.4]	-1.5 [-3.1; 0.7]	-0.28 [-2.15; 1.61]	8.37 [-1.65; 18.77]	1.82 [-0.66; 4.26]
Papua New Guinea	Country	6.8 [4.2; 11.0]	2.2 [1.1; 4.4]	1.6 [0.4; 5.9]	-4.6 [-8.9; -1.1]†	-0.6 [-3.2; 3.7]	-5.2 [-9.6; -0.4]†	4.55 [1.05; 7.79]†	3.94 [-14.33; 22.50]	4.40 [0.20; 8.67]†
Paraguay	Country	2.8 [1.8; 4.3]	2.6 [1.1; 6.1]	2.6 [0.7; 10.8]	-0.3 [-2.4; 3.4]	0.1 [-4.1; 8.2]	-0.2 [-2.7; 7.9]	0.67 [-3.49; 4.24]	-0.83 [-20.98; 20.34]	0.31 [-4.18; 4.42]
Peru	Country	3.6 [2.2; 5.8]	2.5 [0.9; 7.1]	2.0 [0.4; 11.1]	-1.0 [-3.8; 3.8]	-0.6 [-5.5; 8.2]	-1.6 [-4.4; 7.3]	2.69 [-3.26; 5.91]	6.46 [-21.87; 28.11]	3.61 [-3.51; 7.19]
Philippines	Country	0.3 [0.2; 0.4]	0.4 [0.3; 0.6]	0.3 [0.1; 0.8]	0.1 [0.0; 0.4]	-0.1 [-0.4; 0.4]	0.0 [-0.2; 0.6]	-1.43 [-3.61; 0.45]	3.37 [-9.53; 15.95]	-0.27 [-3.43; 2.62]
Poland	Country	0.1 [0.1; 0.1]	0.1 [0.0; 0.1]	0.0 [0.0; 0.1]	0.0 [-0.1; 0.1]	0.0 [-0.1; 0.0]	-0.1 [-0.1; 0.0]	0.34 [-2.43; 2.73]	12.57 [-5.32; 27.64]	3.31 [-1.12; 6.62]
Portugal	Country	0.3 [0.2; 0.5]	0.2 [0.1; 0.4]	0.1 [0.0; 0.4]	-0.1 [-0.3; 0.1]	-0.1 [-0.3; 0.2]	-0.2 [-0.4; 0.1]	1.49 [-0.94; 4.25]	10.16 [-8.25; 28.28]	3.59 [-0.58; 8.10]

†: Change is significantly different from zero.

Total Change (Year 1–Year 2): ASFR (Year 2) - ASFR (Year 1).

Annual Rate of Reduction (Year 1–Year 2): $\log(\text{ASFR}_{\text{Year 2}}/\text{ASFR}_{\text{Year 1}})/(\text{Year 1} - \text{Year 2})$, where Year 1 is smaller than Year 2.

Continued on next page

Table 13 – continued from previous page

Age-specific fertility rate 10–14										
		Estimate (per 1,000)			Total Change (per 1,000)			Annual Rate of Reduction (per 100)		
**Location Name**	**Location Type**	**1990**	**2015**	**2023**	**1990–2015**	**2015–2023**	**1990–2023**	**1990–2015**	**2015–2023**	**1990–2023**
Puerto Rico	Country	2.0 [1.4; 2.9]	0.4 [0.3; 0.6]	0.1 [0.0; 0.2]	-1.6 [-2.5; -1.0]†	-0.3 [-0.6; -0.2]†	-2.0 [-2.8; -1.3]†	6.53 [4.12; 8.61]†	22.09 [9.43; 34.02]†	10.30 [7.12; 12.99]†
Qatar	Country	0.3 [0.2; 0.5]	0.0 [0.0; 0.2]	0.0 [0.0; 0.2]	-0.3 [-0.5; -0.1]†	0.0 [-0.2; 0.1]	-0.3 [-0.5; -0.1]†	13.44 [2.00; 15.38]†	2.73 [-31.25; 34.89]	10.84 [2.19; 11.74]†
Republic of Korea	Country	0.0 [0.0; 0.0]	0.0 [0.0; 0.0]	0.0 [0.0; 0.0]	0.0 [0.0; 0.0]†	0.0 [0.0; 0.0]	0.0 [0.0; 0.0]	-1.99 [-5.55; 0.00]	6.22 [-11.38; 16.63]	0.00 [-4.41; 0.00]
Republic of Moldova	Country	0.2 [0.1; 0.3]	0.0 [0.0; 0.0]	0.0 [0.0; 0.0]	-0.2 [-0.3; -0.1]†	0.0 [0.0; 0.0]†	-0.2 [-0.3; -0.1]†	11.49 [8.89; 14.02]†	0.00 [0.00; 0.00]	8.71 [6.65; 10.62]†
Réunion	Country	0.4 [0.2; 0.8]	0.4 [0.1; 1.2]	0.1 [0.0; 0.3]	0.0 [-0.5; 0.8]	-0.3 [-1.0; 0.1]	-0.3 [-0.7; 0.0]†	0.48 [-4.86; 5.46]	16.86 [-4.12; 37.11]	4.45 [0.19; 8.47]†
Romania	Country	0.6 [0.4; 0.8]	1.4 [0.9; 2.2]	1.5 [0.6; 3.9]	0.8 [0.2; 1.6]†	0.1 [-1.1; 2.6]	0.9 [-0.1; 3.3]	-3.61 [-5.88; -1.07]†	-0.44 [-14.63; 12.59]	-2.84 [-6.02; 0.26]
Russian Federation	Country	0.1 [0.0; 0.1]	0.1 [0.1; 0.2]	0.1 [0.0; 0.2]	0.1 [0.0; 0.2]†	-0.1 [-0.2; 0.1]	0.0 [-0.1; 0.1]	-3.15 [-5.66; -0.37]†	8.65 [-5.09; 21.90]	-0.29 [-3.43; 3.02]
Rwanda	Country	1.2 [0.7; 1.9]	0.3 [0.1; 0.6]	0.3 [0.1; 1.2]	-0.9 [-1.6; -0.4]†	0.0 [-0.4; 0.9]	-0.9 [-1.6; 0.1]	5.98 [2.25; 9.58]†	1.27 [-21.25; 22.01]	4.84 [-0.35; 9.54]
Saint Lucia	Country	2.0 [1.4; 2.7]	0.9 [0.4; 1.9]	0.7 [0.2; 2.6]	-1.1 [-2.0; 0.1]	-0.2 [-1.3; 1.8]	-1.3 [-2.2; 0.6]	3.49 [-0.14; 6.48]	3.00 [-15.95; 21.24]	3.37 [-0.88; 7.10]
Saint Vincent and the Grenadines	Country	2.9 [1.9; 4.4]	1.7 [1.1; 2.6]	1.4 [0.6; 3.5]	-1.1 [-2.8; 0.2]	-0.3 [-1.6; 1.8]	-1.4 [-3.2; 0.7]	2.25 [-0.44; 4.50]	1.75 [-10.02; 14.75]	2.13 [-0.81; 5.12]
Samoa	Country	0.8 [0.3; 2.2]	0.4 [0.2; 1.3]	0.6 [0.1; 2.6]	-0.4 [-1.8; 0.6]	0.1 [-0.8; 2.2]	-0.3 [-1.7; 1.8]	2.47 [-3.03; 8.18]	-3.20 [-26.65; 20.34]	1.09 [-4.21; 6.78]
Sao Tome and Principe	Country	2.7 [1.7; 4.3]	1.5 [0.8; 3.1]	0.9 [0.3; 2.8]	-1.2 [-3.0; 0.7]	-0.6 [-2.3; 1.4]	-1.8 [-3.5; 0.4]	3.84 [-1.06; 5.73]	7.43 [-10.32; 23.95]	4.71 [-0.52; 7.24]
Saudi Arabia	Country	4.0 [0.5; 38.8]	0.0 [0.0; 2.3]	0.0 [0.0; 1.7]	-4.0 [-38.7; -0.1]†	0.0 [-2.1; 1.6]	-4.0 [-38.5; -0.1]†	18.57 [0.34; 30.28]†	5.99 [-53.26; 61.13]	15.52 [0.74; 23.27]†
Senegal	Country	6.8 [5.4; 8.5]	1.7 [1.1; 2.5]	1.3 [0.4; 3.8]	-5.1 [-6.9; -3.5]†	-0.4 [-1.6; 2.1]	-5.5 [-7.4; -2.6]†	5.62 [3.77; 7.45]†	3.84 [-11.05; 17.91]	5.18 [1.61; 8.52]†
Serbia	Country	1.0 [0.1; 6.7]	0.3 [0.1; 0.5]	0.2 [0.1; 0.7]	-0.7 [-6.5; 0.2]	-0.1 [-0.3; 0.5]	-0.8 [-6.5; 0.2]	5.27 [-2.85; 13.48]	3.18 [-14.42; 20.06]	4.76 [-2.43; 11.61]
Seychelles	Country	0.7 [0.4; 1.1]	1.1 [0.7; 1.8]	1.1 [0.3; 3.9]	0.4 [-0.2; 1.2]	0.0 [-1.1; 2.8]	0.5 [-0.5; 3.2]	-1.98 [-4.82; 0.72]	-0.26 [-16.75; 16.20]	-1.57 [-5.69; 2.50]
Sierra Leone	Country	22.2 [16.8; 29.0]	6.6 [4.7; 9.4]	3.3 [1.3; 8.3]	-15.6 [-22.8; -9.7]†	-3.3 [-6.7; 2.0]	-18.9 [-26.0; -11.6]†	4.89 [3.14; 6.60]†	8.82 [-3.67; 21.00]	5.85 [2.83; 8.72]†
Singapore	Country	0.1 [0.1; 0.3]	0.1 [0.0; 0.2]	0.0 [0.0; 0.2]	0.0 [-0.2; 0.1]	0.0 [-0.2; 0.1]	-0.1 [-0.2; 0.0]	1.95 [-2.71; 5.69]	10.15 [-10.71; 29.74]	3.94 [-1.10; 8.22]
Slovakia	Country	0.2 [0.1; 0.3]	0.4 [0.2; 0.6]	0.4 [0.1; 1.1]	0.2 [0.0; 0.4]	0.0 [-0.4; 0.8]	0.2 [-0.1; 0.9]	-2.43 [-5.01; 0.07]	-0.11 [-15.12; 14.33]	-1.87 [-5.39; 1.55]
Slovenia	Country	0.1 [0.0; 0.1]	0.0 [0.0; 0.1]	0.1 [0.0; 0.3]	0.0 [-0.1; 0.0]	0.0 [-0.1; 0.2]	0.0 [-0.1; 0.2]	1.10 [-1.93; 4.12]	-1.59 [-23.79; 19.85]	0.45 [-4.65; 5.41]
Solomon Islands	Country	4.5 [0.5; 37.0]	1.1 [0.1; 19.9]	0.9 [0.0; 17.2]	-3.4 [-34.1; 14.3]	-0.2 [-18.2; 15.1]	-3.6 [-34.2; 12.1]	5.52 [-8.34; 19.76]	2.47 [-49.15; 53.25]	4.78 [-6.33; 15.83]
Somalia	Country	6.6 [4.2; 10.3]	3.8 [2.1; 6.9]	3.6 [1.3; 9.4]	-2.8 [-6.9; 1.1]	-0.2 [-4.1; 5.8]	-3.0 [-7.3; 3.0]	2.27 [-0.77; 5.10]	0.44 [-13.43; 15.77]	1.83 [-1.29; 5.17]
South Africa	Country	2.9 [1.9; 4.4]	0.9 [0.6; 1.4]	1.0 [0.4; 2.4]	-1.9 [-3.5; -0.8]†	0.1 [-0.7; 1.5]	-1.9 [-3.5; -0.1]†	4.49 [2.13; 6.78]†	-0.53 [-13.10; 10.77]	3.27 [0.19; 5.98]†
South Sudan	Country	18.6 [10.5; 33.3]	11.3 [4.5; 28.8]	10.7 [3.1; 35.7]	-7.3 [-23.4; 12.1]	-0.6 [-19.4; 24.7]	-7.9 [-24.0; 18.0]	1.48 [-2.41; 6.42]	1.06 [-18.31; 20.00]	1.38 [-2.38; 5.87]
Spain	Country	0.1 [0.1; 0.2]	0.1 [0.1; 0.2]	0.1 [0.0; 0.2]	0.0 [-0.1; 0.1]	-0.1 [-0.2; 0.1]	-0.1 [-0.2; 0.1]	-0.03 [-2.61; 3.15]	11.07 [-8.79; 28.32]	2.66 [-1.89; 7.09]
Sri Lanka	Country	0.1 [0.1; 0.2]	0.1 [0.0; 0.1]	0.0 [0.0; 0.3]	-0.1 [-0.2; 0.0]	0.0 [-0.1; 0.2]	-0.1 [-0.2; 0.2]	3.04 [-0.70; 6.56]	4.66 [-21.41; 25.55]	3.43 [-3.03; 8.17]
State of Palestine	Country	0.9 [0.6; 1.5]	0.0 [0.0; 0.0]	0.0 [0.0; 0.0]	-0.9 [-1.5; -0.6]†	0.0 [0.0; 0.0]	-0.9 [-1.5; -0.5]†	18.31 [16.05; 20.10]†	0.00 [0.00; 0.00]	13.87 [12.08; 15.22]†
Sudan	Country	6.9 [4.1; 11.3]	3.2 [1.4; 7.8]	2.2 [0.6; 9.1]	-3.6 [-8.4; 1.8]	-1.0 [-5.7; 6.0]	-4.6 [-9.4; 2.6]	3.07 [-1.14; 6.99]	4.78 [-15.53; 25.29]	3.48 [-1.11; 7.80]
Suriname	Country	3.9 [2.7; 5.4]	2.6 [1.7; 3.9]	1.9 [0.8; 4.5]	-1.3 [-3.1; 0.5]	-0.7 [-2.4; 2.0]	-1.9 [-3.9; 0.8]	4.00 [-0.59; 3.78]	1.04 [-8.35; 15.64]	3.28 [-0.66; 4.97]
Sweden	Country	0.0 [0.0; 0.1]	0.0 [0.0; 0.0]	0.0 [0.0; 0.0]	0.0 [0.0; 0.0]	0.0 [0.0; 0.0]	0.0 [-0.1; 0.0]	1.36 [-1.72; 4.87]	7.75 [-10.53; 17.28]	2.91 [-1.31; 5.21]
Switzerland	Country	0.0 [0.0; 0.0]	0.0 [0.0; 0.0]	0.0 [0.0; 0.0]	0.0 [0.0; 0.0]	0.0 [0.0; 0.0]	0.0 [0.0; 0.0]	-1.32 [-4.60; 2.26]	8.32 [-7.17; 16.06]	1.01 [-2.72; 2.77]
Syrian Arab Republic	Country	2.9 [1.7; 4.9]	0.4 [0.1; 2.8]	0.4 [0.0; 4.0]	-2.5 [-4.5; 0.1]	0.0 [-2.3; 3.4]	-2.5 [-4.5; 1.3]	7.83 [-0.21; 16.06]	2.40 [-37.57; 40.22]	6.52 [-1.29; 13.87]
Tajikistan	Country	0.0 [0.0; 0.0]	0.0 [0.0; 0.0]	0.0 [0.0; 0.0]	0.0 [0.0; 0.0]†	0.0 [0.0; 0.0]	0.0 [0.0; 0.0]	1.54 [0.00; 5.80]	0.00 [-12.49; 0.00]	1.17 [-2.36; 4.27]
Thailand	Country	0.7 [0.4; 1.0]	1.2 [0.4; 3.4]	0.6 [0.2; 1.6]	0.5 [-0.3; 2.7]	-0.6 [-2.8; 0.6]	-0.1 [-0.6; 0.9]	-2.30 [-6.80; 2.13]	8.74 [-9.17; 26.98]	0.37 [-2.78; 3.66]
Timor-Leste	Country	1.9 [1.2; 3.0]	0.4 [0.1; 1.0]	0.1 [0.0; 0.9]	-1.5 [-2.7; -0.6]†	-0.2 [-0.8; 0.5]	-1.8 [-2.9; -0.8]†	9.70 [2.38; 10.73]†	12.82 [-13.21; 38.22]	10.46 [2.01; 13.72]†
Togo	Country	9.1 [6.7; 12.4]	2.5 [1.3; 4.9]	2.6 [0.7; 8.8]	-6.5 [-10.2; -3.2]†	0.0 [-2.9; 6.2]	-6.5 [-10.3; 0.0]†	5.06 [2.15; 7.92]†	-0.05 [-17.50; 17.04]	3.82 [0.00; 7.70]†
Tonga	Country	0.6 [0.2; 1.8]	0.3 [0.0; 2.4]	0.3 [0.0; 4.2]	-0.4 [-1.5; 1.7]	0.1 [-2.0; 3.8]	-0.3 [-1.4; 3.4]	3.67 [-6.09; 13.47]	-2.67 [-43.87; 40.59]	2.13 [-6.28; 10.58]
Trinidad and Tobago	Country	1.0 [0.7; 1.5]	0.5 [0.1; 2.1]	0.5 [0.1; 3.9]	-0.6 [-1.2; 1.1]	0.1 [-1.6; 3.3]	-0.5 [-1.2; 2.8]	3.37 [-2.95; 8.98]	-1.46 [-31.74; 28.93]	2.20 [-4.00; 8.02]
Tunisia	Country	0.0 [0.0; 0.0]	0.0 [0.0; 0.0]	0.0 [0.0; 0.0]	0.0 [0.0; 0.0]†	0.0 [0.0; 0.0]	0.0 [0.0; 0.0]†	3.03 [0.00; 5.72]	0.00 [-4.67; 0.00]	2.29 [0.00; 4.33]
Türkiye	Country	1.9 [1.2; 2.9]	0.1 [0.1; 0.1]	0.0 [0.0; 0.1]	-1.8 [-2.8; -1.1]†	-0.1 [-0.1; 0.0]†	-1.8 [-2.9; -1.2]†	12.21 [9.48; 14.54]†	15.45 [0.06; 29.62]†	12.99 [9.18; 16.18]†
Turkmenistan	Country	0.1 [0.0; 0.2]	0.0 [0.0; 0.0]	0.0 [0.0; 0.0]	-0.1 [-0.2; 0.0]†	0.0 [0.0; 0.0]	-0.1 [-0.2; 0.0]†	8.06 [3.33; 12.45]†	0.00 [-3.12; 0.00]	6.11 [2.37; 9.43]†
Uganda	Country	15.0 [12.4; 18.3]	2.2 [1.4; 3.4]	1.6 [0.6; 4.4]	-12.8 [-16.2; -9.7]†	-0.6 [-2.2; 2.2]	-13.4 [-16.8; -9.5]†	7.53 [5.68; 9.56]†	4.18 [-9.34; 17.20]	6.72 [3.62; 9.77]†
Ukraine	Country	0.1 [0.0; 0.1]	0.2 [0.1; 0.3]	0.1 [0.0; 0.5]	0.1 [0.0; 0.2]†	-0.1 [-0.2; 0.3]	0.0 [-0.1; 0.4]	-3.70 [-6.15; -0.94]†	7.80 [-14.63; 28.77]	-0.91 [-6.14; 4.35]
United Arab Emirates	Country	0.6 [0.2; 2.3]	0.0 [0.0; 0.2]	0.0 [0.0; 0.0]	-0.6 [-2.3; -0.1]†	0.0 [-0.2; 0.0]†	-0.6 [-2.3; -0.2]†	16.77 [4.87; 21.43]†	0.00 [0.00; 34.12]	12.71 [8.49; 16.46]†
United Kingdom	Country	0.2 [0.1; 0.3]	0.1 [0.1; 0.1]	0.0 [0.0; 0.1]	-0.1 [-0.2; 0.0]†	-0.1 [-0.1; 0.0]	-0.2 [-0.2; -0.1]†	3.09 [0.63; 5.51]†	14.72 [-1.71; 28.98]	5.91 [1.93; 9.32]†

†: Change is significantly different from zero.

Total Change (Year 1–Year 2): ASFR (Year 2) - ASFR (Year 1).

Annual Rate of Reduction (Year 1–Year 2): $\log(\text{ASFR}_{\text{Year 2}}/\text{ASFR}_{\text{Year 1}})/(\text{Year 1} - \text{Year 2})$, where Year 1 is smaller than Year 2.

Continued on next page

Table 13 – continued from previous page

Age-specific fertility rate 10–14										
		**Estimate (per 1,000)**			**Total Change (per 1,000)**			**Annual Rate of Reduction (per 100)**		
**Location Name**	**Location Type**	**1990**	**2015**	**2023**	**1990–2015**	**2015–2023**	**1990–2023**	**1990–2015**	**2015–2023**	**1990–2023**
United Republic of Tanzania	Country	5.5<br>[4.2; 7.2]	1.6<br>[0.9; 2.9]	1.6<br>[0.6; 4.3]	-3.9<br>[-5.7; -2.1]†	0.0<br>[-1.6; 2.8]	-3.9<br>[-5.8; -1.0]†	4.89<br>[2.38; 7.51]†	-3.82<br>[-14.18; 14.06]	2.78<br>[0.64; 6.86]†
United States of America	Country	1.7<br>[1.2; 2.4]	0.3<br>[0.2; 0.5]	0.2<br>[0.1; 0.5]	-1.4<br>[-2.1; -0.9]†	-0.1<br>[-0.3; 0.2]	-1.5<br>[-2.2; -1.0]†	6.86<br>[4.63; 8.73]†	7.57<br>[-5.96; 20.68]	7.03<br>[3.63; 9.93]†
Uruguay	Country	1.3<br>[0.7; 2.4]	1.1<br>[0.8; 1.7]	0.3<br>[0.1; 0.8]	-0.2<br>[-1.4; 0.7]	-0.8<br>[-1.4; -0.2]†	-1.0<br>[-2.1; -0.2]†	0.51<br>[-2.52; 3.63]	15.48<br>[3.18; 28.74]†	4.14<br>[0.90; 7.77]†
Uzbekistan	Country	0.0<br>[0.0; 0.0]	0.0<br>[0.0; 0.0]	0.0<br>[0.0; 0.0]	0.0<br>[0.0; 0.0]	0.0<br>[0.0; 0.0]	0.0<br>[0.0; 0.0]	0.00<br>[-1.98; 2.64]	-6.08<br>[-17.37; 3.43]	-1.48<br>[-4.04; 1.40]
Vanuatu	Country	2.6<br>[0.2; 28.4]	1.8<br>[0.1; 24.3]	2.0<br>[0.1; 25.5]	-0.8<br>[-25.5; 20.0]	0.1<br>[-20.2; 22.3]	-0.7<br>[-25.0; 21.1]	1.24<br>[-12.95; 15.29]	-0.97<br>[-46.73; 47.87]	0.71<br>[-10.05; 12.02]
Venezuela (Bolivarian Republic of)	Country	3.6<br>[2.3; 5.7]	4.5<br>[3.2; 6.3]	2.2<br>[0.9; 5.3]	0.9<br>[-1.6; 3.1]	-2.3<br>[-4.5; 1.1]	-1.4<br>[-3.9; 1.9]	-0.77<br>[-3.17; 1.39]	9.00<br>[-2.96; 20.74]	1.60<br>[-1.53; 4.46]
Viet Nam	Country	0.3<br>[0.1; 0.5]	0.2<br>[0.1; 0.6]	0.3<br>[0.1; 1.4]	0.0<br>[-0.3; 0.4]	0.1<br>[-0.4; 1.1]	0.1<br>[-0.3; 1.1]	0.46<br>[-4.19; 4.84]	-4.85<br>[-26.26; 18.57]	-0.82<br>[-5.50; 4.15]
Western Sahara	Country	13.9<br>[2.8; 65.1]	0.7<br>[0.0; 11.1]	0.6<br>[0.0; 12.0]	-13.2<br>[-63.7; 1.2]	-0.1<br>[-9.6; 10.7]	-13.3<br>[-63.6; 1.6]	12.73<br>[-0.72; 25.13]	1.40<br>[-48.80; 51.93]	9.98<br>[-0.74; 19.48]
Yemen	Country	9.7<br>[7.5; 12.5]	0.9<br>[0.4; 2.4]	0.9<br>[0.2; 3.8]	-8.8<br>[-11.7; -6.1]†	0.0<br>[-1.7; 3.0]	-8.8<br>[-11.6; -5.2]†	9.54<br>[5.36; 13.30]†	0.71<br>[-21.30; 22.72]	7.40<br>[2.71; 11.80]†
Zambia	Country	6.2<br>[4.9; 7.8]	3.2<br>[2.1; 4.8]	2.9<br>[1.2; 6.7]	-2.9<br>[-4.8; -0.9]†	-0.3<br>[-2.7; 3.6]	-3.3<br>[-5.6; 0.7]	2.53<br>[0.71; 4.44]†	1.68<br>[-10.34; 13.19]	2.32<br>[-0.33; 5.01]
Zimbabwe	Country	5.3<br>[4.0; 7.1]	1.5<br>[0.9; 2.6]	1.5<br>[0.5; 4.3]	-3.8<br>[-5.7; -2.1]†	0.0<br>[-1.6; 2.9]	-3.8<br>[-5.8; -0.8]†	5.07<br>[2.48; 7.55]†	0.04<br>[-15.25; 15.58]	3.85<br>[0.55; 7.26]†

†: Change is significantly different from zero.

Total Change (Year 1–Year 2): ASFR (Year 2) - ASFR (Year 1).

Annual Rate of Reduction (Year 1–Year 2): $\log(\text{ASFR}_{\text{Year 2}}/\text{ASFR}_{\text{Year 1}})/(\text{Year 1} - \text{Year 2})$, where Year 1 is smaller than Year 2.

Table 14: **Estimates and 95% uncertainty intervals for ASFR 15–19 in 1990, 2015, and 2023, the total changes between 1990–2015, between 2015–2013, and between 1990–2023, and the annual rate of reduction (ARR) between 1990–2015, between 2015–2013, and between 1990–2023, for the world, seven SDG regions, 22 subregions, and 201 countries*.** †: Change or ARR is significantly different from zero. *We report results from countries/areas with more than 90,000 population size by mid-2024.

		Age-specific fertility rate 15–19								
		Estimate (per 1,000)			Total Change (per 1,000)			Annual Rate of Reduction (per 100)		
Location Name	Location Type	1990	2015	2023	1990–2015	2015–2023	1990–2023	1990–2015	2015–2023	1990–2023
World	World	74.7 [72.5; 77.1]	45.9 [44.9; 46.9]	39.0 [36.8; 41.4]	-28.8 [-31.3; -26.3]†	-6.9 [-9.3; -4.3]†	-35.7 [-38.9; -32.4]†	1.95 [1.80; 2.10]†	2.04 [1.23; 2.80]†	1.97 [1.77; 2.17]†
Sub-Saharan Africa	SDG region	139.5 [136.3; 142.9]	110.5 [106.8; 114.6]	94.4 [86.3; 103.4]	-29.0 [-34.1; -23.9]†	-16.1 [-25.1; -6.3]†	-45.1 [-54.0; -35.4]†	0.93 [0.76; 1.10]†	1.97 [0.74; 3.17]†	1.18 [0.90; 1.47]†
Latin America and the Caribbean	SDG region	89.1 [82.2; 97.4]	67.2 [64.8; 69.7]	51.2 [45.8; 57.5]	-21.9 [-30.5; -14.7]†	-16.0 [-21.8; -9.4]†	-37.9 [-47.5; -28.5]†	1.13 [0.78; 1.51]†	3.40 [1.90; 4.86]†	1.68 [1.24; 2.11]†
Oceania (excluding Australia and New Zealand)	SDG region	78.9 [70.7; 89.0]	56.6 [50.2; 64.0]	49.9 [35.1; 72.0]	-22.3 [-34.2; -11.3]†	-6.7 [-23.5; 16.5]	-29.0 [-47.1; -5.4]†	1.33 [0.68; 1.99]†	1.57 [-3.27; 6.25]	1.39 [0.22; 2.54]†
Northern Africa and Western Asia	SDG region	66.3 [63.8; 68.9]	45.2 [41.1; 49.9]	36.3 [30.7; 43.0]	-21.1 [-26.0; -15.7]†	-8.9 [-16.1; -0.9]†	-30.0 [-36.2; -22.7]†	1.53 [1.10; 1.94]†	2.75 [0.27; 5.12]†	1.82 [1.29; 2.35]†
Central and Southern Asia	SDG region	131.4 [123.1; 140.3]	32.1 [30.5; 33.8]	26.2 [22.6; 30.5]	-99.3 [-108.4; -90.9]†	-5.9 [-9.8; -1.3]†	-105.2 [-114.6; -95.9]†	5.64 [5.31; 5.98]†	2.54 [0.50; 4.46]†	4.89 [4.39; 5.36]†
Eastern and South-Eastern Asia	SDG region	30.5 [29.1; 32.1]	21.7 [20.2; 23.5]	14.3 [12.4; 16.5]	-8.8 [-11.0; -6.6]†	-7.4 [-10.0; -4.7]†	-16.2 [-18.6; -13.6]†	1.36 [1.00; 1.72]†	5.22 [3.17; 7.20]†	2.30 [1.84; 2.74]†
Europe, Northern America, Australia, and New Zealand	SDG region	39.0 [37.9; 40.1]	16.6 [16.1; 17.2]	9.9 [9.0; 10.9]	-22.4 [-23.6; -21.2]†	-6.8 [-7.8; -5.7]†	-29.1 [-30.6; -27.7]†	3.41 [3.23; 3.58]†	6.51 [5.29; 7.72]†	4.16 [3.86; 4.45]†
Northern America	Subregion	57.9 [54.5; 61.7]	20.9 [19.6; 22.3]	12.4 [10.3; 14.9]	-36.9 [-41.0; -33.3]†	-8.5 [-11.0; -5.7]†	-45.4 [-49.7; -41.3]†	4.07 [3.71; 4.43]†	6.52 [4.10; 8.94]†	4.66 [4.09; 5.24]†
Eastern Africa	Subregion	145.0 [140.3; 150.0]	105.3 [100.6; 110.5]	92.6 [81.6; 104.9]	-39.8 [-46.7; -33.1]†	-12.7 [-24.9; 0.7]	-52.4 [-64.6; -39.3]†	1.28 [1.06; 1.51]†	1.60 [-0.08; 3.29]	1.36 [0.97; 1.76]†
Middle Africa	Subregion	141.4 [133.8; 149.7]	128.4 [116.7; 141.5]	117.5 [93.4; 149.4]	-13.0 [-27.9; 2.4]	-10.9 [-38.1; 23.2]	-24.0 [-49.6; 8.3]	0.39 [-0.07; 0.85]	1.11 [-2.16; 4.21]	0.56 [-0.17; 1.28]
Northern Africa	Subregion	58.9 [55.4; 62.7]	47.9 [40.8; 56.2]	38.2 [29.0; 49.8]	-10.9 [-19.1; -1.8]†	-9.8 [-22.1; 4.0]	-20.7 [-30.5; -8.3]†	0.82 [0.13; 1.52]†	2.85 [-1.10; 6.81]	1.31 [0.47; 2.16]†
Southern Africa	Subregion	95.7 [87.3; 105.3]	68.7 [63.9; 73.8]	53.4 [43.8; 66.0]	-27.0 [-37.8; -17.2]†	-15.3 [-26.4; -1.7]†	-42.3 [-56.1; -27.5]†	1.33 [0.86; 1.81]†	3.14 [0.31; 5.81]†	1.77 [1.09; 2.44]†
Western Africa	Subregion	142.9 [136.7; 149.3]	115.2 [108.5; 122.6]	91.2 [78.1; 108.1]	-27.7 [-37.4; -18.1]†	-23.9 [-38.9; -5.8]†	-51.6 [-66.3; -34.0]†	0.86 [0.56; 1.18]†	2.91 [0.67; 4.99]†	1.36 [0.84; 1.85]†
Central Asia	Subregion	44.6 [42.6; 46.8]	32.3 [30.8; 33.8]	29.6 [26.1; 33.4]	-12.4 [-15.0; -9.9]†	-2.7 [-6.5; 1.4]	-15.1 [-19.1; -10.7]†	1.30 [1.04; 1.56]†	1.08 [-0.56; 2.76]	1.25 [0.85; 1.64]†
Eastern Asia	Subregion	20.8 [19.1; 22.6]	9.8 [7.9; 12.3]	4.8 [3.2; 7.4]	-10.9 [-13.6; -8.0]†	-5.0 [-7.9; -1.9]†	-16.0 [-18.4; -13.0]†	2.99 [2.05; 3.92]†	8.98 [3.09; 14.68]†	4.44 [3.14; 5.69]†
Southern Asia	Subregion	135.1 [126.4; 144.4]	32.1 [30.4; 33.9]	26.1 [22.4; 30.6]	-103.0 [-112.4; -94.2]†	-6.0 [-10.0; -1.2]†	-109.0 [-118.7; -99.3]†	5.75 [5.42; 6.10]†	2.59 [0.49; 4.58]†	4.98 [4.47; 5.48]†
South-Eastern Asia	Subregion	59.9 [57.3; 62.5]	41.6 [39.2; 44.2]	29.2 [25.3; 33.9]	-18.2 [-21.9; -14.7]†	-12.4 [-17.2; -7.0]†	-30.7 [-35.3; -25.4]†	1.45 [1.16; 1.75]†	4.42 [2.40; 6.40]†	2.17 [1.70; 2.63]†
Western Asia	Subregion	73.6 [70.0; 77.4]	42.8 [38.6; 47.5]	34.6 [28.5; 42.6]	-30.9 [-36.5; -25.0]†	-8.2 [-15.8; 0.8]	-39.1 [-46.3; -30.4]†	2.17 [1.70; 2.64]†	2.66 [-0.25; 5.38]	2.29 [1.64; 2.90]†
Eastern Europe	Subregion	50.7 [49.0; 52.6]	23.9 [22.9; 25.0]	14.1 [12.7; 15.7]	-26.8 [-28.9; -24.8]†	-9.9 [-11.6; -8.0]†	-36.7 [-38.9; -34.4]†	3.01 [2.78; 3.23]†	6.64 [5.18; 8.04]†	3.89 [3.55; 4.22]†
Northern Europe	Subregion	29.5 [28.1; 30.9]	11.9 [11.2; 12.5]	6.3 [5.1; 7.8]	-17.6 [-19.2; -16.1]†	-5.5 [-6.9; -3.9]†	-23.1 [-25.0; -21.1]†	3.64 [3.35; 3.93]†	7.84 [5.05; 10.55]†	4.66 [4.01; 5.32]†
Southern Europe	Subregion	15.9 [15.5; 16.3]	8.1 [7.9; 8.4]	5.3 [4.8; 5.9]	-7.8 [-8.3; -7.3]†	-2.8 [-3.4; -2.2]†	-10.6 [-11.2; -9.9]†	2.70 [2.54; 2.85]†	5.26 [3.94; 6.60]†	3.32 [3.00; 3.63]†
Western Europe	Subregion	15.9 [15.3; 16.6]	7.3 [7.0; 7.6]	4.0 [3.5; 4.6]	-8.6 [-9.3; -7.9]†	-3.3 [-3.9; -2.7]†	-11.9 [-12.7; -11.0]†	3.10 [2.87; 3.33]†	7.57 [5.78; 9.25]†	4.18 [3.75; 4.59]†
Caribbean	Subregion	88.5 [84.4; 92.7]	58.6 [55.1; 62.5]	45.9 [38.1; 56.4]	-29.9 [-35.5; -24.2]†	-12.8 [-21.5; -1.6]†	-42.7 [-51.5; -31.2]†	1.65 [1.33; 1.96]†	3.07 [0.36; 5.52]†	1.99 [1.34; 2.57]†
Central America	Subregion	93.4 [76.0; 115.8]	73.8 [69.1; 79.0]	62.8 [49.5; 81.0]	-19.7 [-42.1; -1.6]†	-10.9 [-25.1; 7.5]	-30.6 [-56.7; -5.4]†	0.95 [0.08; 1.83]†	2.01 [-1.25; 5.08]	1.20 [0.20; 2.18]†
South America	Subregion	87.3 [80.0; 95.9]	65.1 [62.2; 68.1]	46.1 [41.6; 51.1]	-22.2 [-31.0; -14.3]†	-19.0 [-24.5; -13.3]†	-41.2 [-50.8; -32.2]†	1.17 [0.78; 1.58]†	4.31 [2.94; 5.73]†	1.93 [1.53; 2.36]†
Australia/New Zealand	Subregion	24.7 [23.4; 26.1]	13.2 [12.3; 14.1]	7.4 [6.4; 8.6]	-11.5 [-13.1; -9.9]†	-5.8 [-7.2; -4.3]†	-17.3 [-19.0; -15.5]†	2.50 [2.15; 2.84]†	7.21 [5.14; 9.40]†	3.64 [3.17; 4.16]†
Melanesia	Subregion	82.0 [72.3; 93.8]	58.0 [50.8; 66.2]	51.1 [35.1; 75.3]	-24.0 [-37.9; -11.5]†	-6.9 [-25.1; 18.7]	-30.9 [-51.1; -4.7]†	1.39 [0.67; 2.12]†	1.58 [-3.62; 6.62]	1.43 [0.18; 2.67]†
Micronesia	Subregion	77.0 [69.7; 85.3]	48.5 [42.1; 57.4]	43.0 [33.6; 56.0]	-28.5 [-39.2; -17.3]†	-5.5 [-18.1; 9.3]	-34.0 [-46.7; -19.1]†	1.85 [1.08; 2.55]†	1.51 [-2.27; 5.17]	1.77 [0.91; 2.56]†
Polynesia	Subregion	52.6 [48.3; 57.4]	39.3 [35.5; 43.6]	31.4 [25.2; 39.3]	-13.3 [-19.3; -7.3]†	-8.0 [-15.6; 0.8]	-21.3 [-29.1; -12.3]†	1.17 [0.63; 1.70]†	2.82 [-0.25; 5.83]	1.57 [0.84; 2.28]†
Afghanistan	Country	139.4 [123.0; 158.1]	81.0 [66.3; 98.5]	64.1 [44.8; 91.1]	-58.3 [-82.3; -34.7]†	-17.0 [-42.7; 13.1]	-75.3 [-102.7; -44.0]†	2.58 [1.24; 3.12]†	3.02 [-2.10; 7.90]	2.68 [1.24; 3.51]†
Albania	Country	15.8 [14.8; 17.0]	20.7 [18.9; 22.6]	12.8 [9.9; 16.6]	4.8 [2.8; 7.0]†	-7.9 [-11.3; -3.8]†	-3.0 [-6.2; 1.0]	-0.96 [-1.50; -0.63]†	5.75 [2.65; 9.42]†	0.67 [-0.18; 1.45]
Algeria	Country	24.4 [22.5; 26.4]	10.3 [9.6; 11.1]	8.7 [6.1; 12.3]	-14.1 [-16.2; -12.0]†	-1.6 [-4.2; 2.0]	-15.7 [-19.0; -11.7]†	3.93 [3.01; 3.88]†	0.91 [-2.21; 6.47]	3.19 [2.06; 4.20]†
Angola	Country	145.4 [125.9; 167.0]	150.2 [121.6; 182.9]	140.8 [87.2; 219.4]	4.8 [-31.7; 42.5]	-9.4 [-71.4; 73.5]	-4.6 [-62.8; 75.9]	-0.58 [-1.09; 0.89]	0.65 [-5.30; 7.14]	-0.28 [-1.30; 1.58]
Antigua and Barbuda	Country	78.4 [69.3; 88.4]	35.9 [31.6; 40.7]	32.9 [23.9; 45.5]	-42.4 [-53.4; -32.0]†	-3.0 [-13.2; 10.2]	-45.4 [-58.9; -29.6]†	3.07 [2.42; 3.82]†	0.50 [-3.30; 5.42]	2.44 [1.56; 3.68]†
Argentina	Country	70.3 [63.7; 77.4]	63.9 [58.7; 69.3]	26.4 [21.9; 32.0]	-6.4 [-15.0; 2.2]	-37.5 [-44.5; -30.0]†	-43.9 [-52.3; -35.3]†	0.58 [-0.13; 0.89]	10.89 [8.44; 13.62]†	3.08 [2.31; 3.60]†
Armenia	Country	73.9 [69.7; 78.5]	25.1 [22.9; 27.5]	13.4 [10.4; 17.4]	-48.8 [-53.9; -43.8]†	-11.7 [-15.6; -7.1]†	-60.5 [-66.0; -54.6]†	4.46 [3.89; 4.77]†	6.57 [4.36; 11.18]†	4.97 [4.35; 5.94]†
Aruba	Country	59.5 [52.4; 67.3]	32.3 [28.5; 36.8]	18.8 [13.9; 25.3]	-27.2 [-35.7; -18.9]†	-13.6 [-20.4; -6.0]†	-40.7 [-49.9; -31.4]†	3.17 [1.72; 3.13]†	6.99 [2.77; 10.98]†	4.10 [2.53; 4.48]†
Australia	Country	22.4 [20.9; 24.0]	12.0 [11.1; 13.1]	6.7 [5.5; 8.1]	-10.4 [-12.2; -8.5]†	-5.3 [-6.9; -3.6]†	-15.7 [-17.7; -13.7]†	2.43 [2.04; 2.92]†	6.72 [4.70; 9.99]†	3.47 [3.05; 4.29]†
Austria	Country	22.5 [21.1; 24.1]	7.8 [7.3; 8.4]	3.8 [3.0; 5.0]	-14.7 [-16.3; -13.1]†	-4.0 [-5.1; -2.8]†	-18.7 [-20.5; -16.9]†	4.15 [3.82; 4.62]†	8.35 [5.57; 12.27]†	5.17 [4.55; 6.18]†

†: Change is significantly different from zero.
Total Change (Year 1–Year 2): ASFR (Year 2) - ASFR (Year 1).
Annual Rate of Reduction (Year 1–Year 2): $\log(\text{ASFR}_{\text{Year 2}}/\text{ASFR}_{\text{Year 1}})/(\text{Year 1} - \text{Year 2})$, where Year 1 is smaller than Year 2.

Continued on next page

Table 14 – continued from previous page

Age-specific fertility rate 15–19										
		Estimate (per 1,000)			Total Change (per 1,000)			Annual Rate of Reduction (per 100)		
**Location Name**	**Location Type**	**1990**	**2015**	**2023**	**1990–2015**	**2015–2023**	**1990–2023**	**1990–2015**	**2015–2023**	**1990–2023**
Azerbaijan	Country	31.5 [29.3; 33.8]	62.7 [57.5; 68.4]	34.8 [28.9; 41.9]	31.2 [25.4; 37.3]†	-27.9 [-36.0; -19.4]†	3.3 [-3.0; 10.7]	-2.64 [-3.20; -2.30]†	5.64 [4.84; 9.92]†	-0.63 [-0.90; 0.30]
Bahamas	Country	55.9 [29.8; 102.0]	30.8 [22.7; 42.3]	24.6 [14.0; 42.0]	-25.0 [-71.1; 3.6]	-6.3 [-22.0; 12.6]	-31.3 [-77.4; 0.0]†	2.90 [-0.41; 5.06]	2.31 [-4.80; 10.96]	2.76 [0.00; 4.97]†
Bahrain	Country	24.6 [22.3; 27.2]	15.1 [13.6; 16.6]	7.5 [5.5; 10.4]	-9.5 [-12.4; -6.7]†	-7.6 [-10.1; -4.5]†	-17.1 [-20.4; -13.6]†	1.89 [1.40; 2.51]†	8.24 [4.55; 12.65]†	3.43 [2.58; 4.58]†
Bangladesh	Country	178.3 [166.5; 190.8]	89.3 [82.0; 97.5]	73.2 [60.2; 88.7]	-89.0 [-103.4; -74.5]†	-16.1 [-31.5; 1.3]	-105.1 [-123.2; -85.7]†	3.17 [2.31; 3.19]†	2.71 [-0.20; 5.23]	3.06 [2.07; 3.33]†
Barbados	Country	60.3 [54.8; 66.1]	45.5 [28.1; 71.1]	45.3 [23.4; 85.8]	-14.7 [-33.2; 11.4]	-0.2 [-35.1; 43.1]	-14.9 [-37.4; 25.6]	1.12 [-0.71; 3.09]	0.01 [-9.96; 10.03]	0.85 [-1.07; 2.87]
Belarus	Country	42.4 [39.6; 45.2]	18.3 [17.1; 19.7]	8.6 [6.0; 12.6]	-24.1 [-27.1; -21.0]†	-9.7 [-12.6; -5.7]†	-33.8 [-37.9; -29.0]†	3.46 [2.97; 3.73]†	8.10 [4.66; 14.08]†	4.58 [3.66; 5.98]†
Belgium	Country	12.0 [11.2; 12.8]	6.7 [6.2; 7.2]	3.7 [2.8; 4.8]	-5.3 [-6.3; -4.4]†	-3.0 [-4.0; -1.8]†	-8.3 [-9.5; -7.0]†	2.19 [1.97; 2.76]†	7.05 [3.99; 10.75]†	3.37 [2.76; 4.40]†
Belize	Country	127.9 [112.3; 145.2]	70.2 [64.1; 76.9]	55.2 [45.6; 66.8]	-57.7 [-76.0; -40.6]†	-15.0 [-26.5; -1.9]†	-72.7 [-92.9; -53.0]†	2.51 [1.76; 3.01]†	2.69 [0.36; 5.61]†	2.56 [1.83; 3.24]†
Benin	Country	126.7 [115.6; 138.4]	96.3 [85.4; 109.0]	77.8 [55.1; 108.0]	-30.3 [-46.4; -12.8]†	-18.5 [-44.3; 12.7]	-48.8 [-74.4; -17.5]†	0.98 [0.46; 1.70]†	3.11 [-1.61; 7.25]	1.50 [0.46; 2.54]†
Bhutan	Country	95.6 [53.4; 170.6]	16.5 [12.4; 21.7]	9.4 [7.2; 12.2]	-79.1 [-154.0; -36.4]†	-7.1 [-12.9; -2.2]†	-86.2 [-160.9; -44.0]†	7.39 [4.46; 9.63]†	7.04 [2.24; 11.88]†	7.31 [5.09; 9.03]†
Bolivia (Plurinational State of)	Country	97.5 [89.9; 105.5]	72.7 [49.1; 105.5]	64.8 [35.6; 115.3]	-24.8 [-50.0; 8.4]	-7.9 [-51.3; 47.5]	-32.7 [-63.1; 18.5]	1.55 [-0.34; 2.77]	1.37 [-7.13; 10.25]	1.50 [-0.53; 3.09]
Bosnia and Herzegovina	Country	38.1 [35.6; 40.7]	10.8 [9.1; 12.7]	11.2 [8.0; 15.7]	-27.3 [-30.4; -24.1]†	0.4 [-3.4; 5.2]	-26.9 [-31.1; -21.7]†	5.09 [4.32; 5.77]†	1.20 [-5.24; 4.22]	4.15 [2.66; 4.76]†
Botswana	Country	103.6 [82.2; 128.5]	57.2 [51.0; 64.0]	53.8 [38.4; 74.3]	-46.4 [-72.2; -23.6]†	-3.4 [-20.5; 18.9]	-49.8 [-79.7; -19.3]†	2.63 [1.34; 3.38]†	0.66 [-3.73; 5.22]	2.16 [0.74; 3.22]†
Brazil	Country	88.5 [74.8; 104.6]	61.8 [56.9; 67.1]	42.7 [35.7; 51.4]	-26.7 [-43.7; -12.1]†	-19.1 [-28.1; -9.4]†	-45.8 [-63.5; -29.3]†	1.56 [0.70; 2.18]†	5.27 [2.11; 7.16]†	2.46 [1.43; 2.96]†
Brunei Darussalam	Country	37.8 [34.0; 42.1]	11.9 [10.6; 13.4]	8.5 [6.4; 11.2]	-25.9 [-30.3; -21.8]†	-3.4 [-5.9; -0.4]†	-29.3 [-34.1; -24.6]†	4.61 [3.99; 5.25]†	3.22 [0.49; 8.02]†	4.27 [3.63; 5.44]†
Bulgaria	Country	70.0 [65.6; 74.5]	39.1 [36.5; 41.8]	39.1 [32.4; 46.8]	-30.9 [-36.0; -25.7]†	0.0 [-7.2; 8.4]	-30.9 [-38.9; -21.8]†	2.41 [1.95; 2.70]†	-0.19 [-2.48; 2.51]	1.78 [1.18; 2.36]†
Burkina Faso	Country	145.7 [132.4; 159.8]	119.4 [105.8; 134.1]	87.1 [63.3; 117.8]	-26.3 [-46.0; -6.7]†	-32.3 [-60.0; 0.9]	-58.6 [-87.0; -25.6]†	0.99 [0.21; 1.41]†	3.93 [-0.09; 8.10]	1.70 [0.61; 2.58]†
Burundi	Country	74.6 [64.4; 86.2]	62.7 [53.2; 73.4]	53.4 [32.4; 86.1]	-11.8 [-26.8; 2.9]	-9.4 [-33.6; 24.9]	-21.2 [-45.3; 13.8]	0.71 [-0.17; 1.56]	1.65 [-4.36; 8.69]	0.93 [-0.53; 2.60]
Cabo Verde	Country	123.5 [107.0; 141.5]	58.0 [53.1; 63.2]	38.8 [28.4; 53.0]	-65.5 [-84.3; -47.8]†	-19.2 [-31.0; -4.4]†	-84.7 [-106.4; -62.7]†	3.26 [2.35; 3.68]†	4.84 [1.02; 9.05]†	3.64 [2.47; 4.54]†
Cambodia	Country	84.5 [73.3; 96.4]	53.6 [45.1; 63.3]	46.9 [34.1; 64.7]	-30.9 [-45.4; -16.1]†	-6.7 [-23.3; 13.0]	-37.6 [-54.8; -16.2]†	1.75 [0.94; 2.67]†	1.03 [-2.93; 6.22]	1.58 [0.70; 2.82]†
Cameroon	Country	159.4 [147.9; 171.7]	119.7 [103.3; 139.1]	106.7 [71.7; 156.3]	-39.7 [-60.3; -17.5]†	-13.1 [-53.1; 39.7]	-52.7 [-90.3; -2.8]†	1.00 [0.48; 1.80]†	1.35 [-3.71; 6.70]	1.09 [0.06; 2.45]†
Canada	Country	25.4 [23.7; 27.1]	9.7 [9.1; 10.4]	4.8 [3.7; 6.2]	-15.6 [-17.5; -13.8]†	-4.9 [-6.2; -3.3]†	-20.6 [-22.6; -18.4]†	3.93 [3.45; 4.21]†	8.03 [5.42; 12.07]†	4.92 [4.22; 5.81]†
Central African Republic	Country	165.1 [145.6; 187.7]	164.1 [138.7; 193.6]	163.1 [112.4; 229.9]	-1.0 [-34.5; 34.6]	-1.0 [-58.6; 69.9]	-2.0 [-56.9; 65.9]	-0.55 [-0.81; 0.86]	0.19 [-4.70; 5.05]	-0.37 [-1.03; 1.20]
Chad	Country	199.3 [177.5; 224.3]	162.1 [138.4; 188.2]	134.7 [92.3; 194.1]	-37.2 [-71.2; -3.2]†	-27.4 [-78.4; 37.2]	-64.5 [-114.4; -1.5]†	0.58 [0.07; 1.60]†	2.58 [-2.71; 7.55]	1.07 [0.03; 2.40]†
Chile	Country	69.5 [63.5; 75.9]	39.4 [36.2; 42.9]	6.5 [5.9; 7.2]	-30.1 [-37.3; -23.0]†	-32.9 [-36.5; -29.6]†	-63.0 [-69.4; -56.9]†	2.38 [1.76; 2.77]†	22.80 [20.79; 24.10]†	7.33 [6.76; 7.57]†
China	Country	23.2 [21.3; 25.4]	10.9 [8.7; 13.7]	5.2 [3.4; 8.1]	-12.3 [-15.5; -8.9]†	-5.7 [-9.1; -2.2]†	-18.0 [-20.9; -14.6]†	3.50 [2.05; 4.01]†	7.57 [3.16; 15.29]†	4.48 [3.20; 5.87]†
China, Hong Kong SAR	Country	6.1 [5.6; 6.5]	3.3 [3.0; 3.6]	1.1 [0.9; 1.4]	-2.8 [-3.3; -2.2]†	-2.2 [-2.6; -1.8]†	-4.9 [-5.4; -4.4]†	2.93 [1.94; 2.92]†	11.42 [10.46; 16.55]†	4.99 [4.41; 5.82]†
Macao, SAR of China	Country	7.7 [6.7; 8.8]	2.3 [1.9; 2.7]	0.5 [0.4; 0.6]	-5.4 [-6.5; -4.4]†	-1.8 [-2.2; -1.4]†	-7.2 [-8.3; -6.3]†	4.75 [4.02; 5.71]†	19.37 [16.20; 23.46]†	8.30 [7.66; 9.34]†
China, Taiwan Province of China	Country	16.8 [15.7; 18.0]	4.6 [4.3; 4.9]	3.0 [2.5; 3.7]	-12.2 [-13.4; -11.1]†	-1.6 [-2.2; -0.8]†	-13.8 [-15.1; -12.5]†	5.45 [4.80; 5.57]†	4.64 [2.61; 7.89]†	5.26 [4.56; 5.84]†
Colombia	Country	93.2 [83.6; 103.5]	69.8 [64.1; 75.8]	59.5 [49.3; 71.6]	-23.4 [-35.6; -12.4]†	-10.3 [-22.0; 3.1]	-33.7 [-48.3; -19.0]†	1.54 [0.63; 1.72]†	3.80 [-0.58; 4.53]	2.09 [0.73; 2.00]†
Comoros	Country	105.1 [92.3; 119.3]	64.4 [47.0; 86.4]	55.2 [32.3; 91.9]	-40.7 [-63.7; -14.9]†	-9.2 [-41.2; 32.2]	-49.9 [-77.2; -10.4]†	1.86 [0.66; 3.35]†	1.84 [-5.68; 9.61]	1.85 [0.33; 3.62]†
Republic of the Congo	Country	126.3 [109.4; 145.4]	120.7 [90.4; 158.9]	109.9 [64.7; 179.5]	-5.6 [-41.8; 36.2]	-10.8 [-71.6; 67.0]	-16.4 [-65.1; 55.4]	0.33 [-1.08; 1.45]	1.01 [-6.15; 8.61]	0.49 [-1.14; 2.06]
Costa Rica	Country	91.3 [83.6; 99.6]	56.6 [52.0; 61.3]	26.3 [21.7; 31.8]	-34.7 [-44.0; -25.8]†	-30.3 [-37.1; -23.2]†	-65.1 [-74.5; -55.5]†	1.90 [1.44; 2.39]†	9.56 [6.98; 12.29]†	3.75 [3.14; 4.41]†
Côte d'Ivoire	Country	159.9 [143.8; 177.5]	116.2 [102.0; 132.3]	92.1 [67.9; 125.1]	-43.7 [-66.4; -21.1]†	-24.1 [-53.1; 10.5]	-67.8 [-98.6; -32.2]†	1.32 [0.60; 1.95]†	3.13 [-1.13; 7.01]	1.76 [0.71; 2.67]†
Croatia	Country	27.6 [25.8; 29.4]	9.8 [9.1; 10.6]	6.6 [5.8; 7.4]	-17.7 [-19.7; -15.8]†	-3.3 [-4.3; -2.2]†	-21.0 [-23.0; -19.0]†	4.23 [3.73; 4.52]†	4.51 [3.30; 6.70]†	4.30 [3.94; 4.74]†
Cuba	Country	78.2 [72.0; 85.0]	50.1 [46.1; 54.5]	48.7 [38.3; 62.3]	-28.1 [-36.0; -20.4]†	-1.4 [-13.0; 12.6]	-29.6 [-42.0; -14.7]†	1.71 [1.31; 2.24]†	0.34 [-2.85; 3.61]	1.38 [0.66; 2.22]†
Curaçao	Country	54.2 [43.7; 66.9]	29.5 [26.1; 33.4]	13.0 [9.0; 18.8]	-24.6 [-37.8; -13.5]†	-16.6 [-22.1; -10.1]†	-41.2 [-54.6; -29.3]†	2.94 [1.45; 3.39]†	8.70 [5.47; 15.10]†	4.34 [3.05; 5.64]†
Cyprus	Country	31.3 [29.2; 33.6]	5.5 [4.9; 6.2]	7.0 [5.3; 9.1]	-25.8 [-28.1; -23.6]†	1.5 [-0.3; 3.7]	-24.4 [-27.2; -21.3]†	6.98 [6.39; 7.51]†	-3.62 [-6.66; 0.69]	4.41 [3.70; 5.41]†
Czechia	Country	41.1 [38.5; 43.9]	11.9 [11.1; 12.7]	6.0 [4.9; 7.2]	-29.3 [-32.1; -26.5]†	-5.9 [-7.2; -4.4]†	-35.2 [-38.1; -32.2]†	5.39 [4.60; 5.36]†	7.68 [6.08; 11.14]†	5.95 [5.23; 6.47]†
Dem. People's Republic of Korea	Country	3.0 [1.7; 5.3]	0.6 [0.4; 1.0]	0.5 [0.3; 1.0]	-2.4 [-4.7; -1.0]†	-0.1 [-0.5; 0.5]	-2.5 [-4.8; -1.1]†	6.43 [3.46; 9.47]†	2.16 [-8.30; 12.18]	5.40 [2.70; 7.96]†
Democratic Republic of the Congo	Country	122.3 [109.9; 136.4]	115.8 [97.0; 138.4]	106.9 [68.0; 163.7]	-6.5 [-30.7; 19.8]	-9.0 [-54.8; 50.4]	-15.4 [-56.9; 40.8]	0.22 [-0.63; 1.08]	0.93 [-4.77; 7.18]	0.39 [-0.90; 1.82]
Denmark	Country	9.2 [8.6; 9.9]	3.5 [3.2; 3.8]	1.1 [0.9; 1.4]	-5.7 [-6.5; -5.0]†	-2.4 [-2.7; -2.0]†	-8.1 [-8.8; -7.4]†	3.95 [3.40; 4.28]†	13.68 [11.43; 16.80]†	6.31 [5.71; 6.99]†
Djibouti	Country	47.5 [40.6; 55.5]	21.5 [14.1; 32.2]	19.0 [10.0; 35.9]	-26.0 [-36.7; -13.2]†	-2.5 [-16.6; 16.0]	-28.5 [-40.8; -10.6]†	3.86 [1.43; 4.92]†	2.15 [-7.93; 11.08]	3.45 [0.79; 4.78]†
Dominican Republic	Country	117.5 [107.0; 128.6]	83.2 [75.9; 91.1]	52.8 [43.8; 63.5]	-34.4 [-47.5; -21.5]†	-30.4 [-42.3; -17.6]†	-64.7 [-79.5; -49.7]†	2.16 [0.87; 1.89]†	6.66 [3.11; 8.27]†	3.25 [1.78; 3.05]†
Ecuador	Country	91.9 [80.6; 105.1]	79.4 [72.4; 86.8]	55.5 [43.4; 70.7]	-12.5 [-27.8; 1.2]	-23.8 [-37.8; -7.3]†	-36.4 [-53.8; -17.7]†	0.53 [-0.06; 1.25]	5.79 [1.26; 7.72]†	1.81 [0.71; 2.38]†

†: Change is significantly different from zero.

Total Change (Year 1–Year 2): ASFR (Year 2) - ASFR (Year 1).

Annual Rate of Reduction (Year 1–Year 2): $\log(\text{ASFR}_{\text{Year 2}}/\text{ASFR}_{\text{Year 1}})/(\text{Year 1} - \text{Year 2})$, where Year 1 is smaller than Year 2.

Continued on next page

Table 14 – continued from previous page

Age-specific fertility rate 15–19										
		Estimate (per 1,000)			Total Change (per 1,000)			Annual Rate of Reduction (per 100)		
**Location Name**	**Location Type**	**1990**	**2015**	**2023**	**1990–2015**	**2015–2023**	**1990–2023**	**1990–2015**	**2015–2023**	**1990–2023**
Egypt	Country	78.7 [71.8; 86.0]	57.1 [45.4; 71.6]	41.8 [29.7; 57.5]	-21.6 [-35.5; -5.5]†	-15.3 [-33.6; 4.2]	-36.9 [-51.5; -20.0]†	1.00 [0.31; 2.26]†	3.83 [-0.98; 9.03]	1.69 [0.92; 3.01]†
El Salvador	Country	122.9 [114.0; 132.3]	69.3 [63.7; 75.2]	54.2 [37.6; 77.4]	-53.6 [-64.6; -43.0]†	-15.1 [-32.8; 8.3]	-68.7 [-88.6; -44.2]†	2.24 [1.85; 2.75]†	3.79 [-1.46; 7.78]	2.62 [1.37; 3.62]†
Equatorial Guinea	Country	182.2 [138.2; 238.5]	169.7 [117.2; 239.3]	150.4 [83.8; 249.1]	-12.5 [-91.0; 68.6]	-19.2 [-115.2; 92.7]	-31.7 [-118.4; 78.7]	0.36 [-1.48; 2.15]	1.34 [-6.37; 9.97]	0.60 [-1.22; 2.49]
Eritrea	Country	97.8 [85.7; 111.0]	72.7 [44.8; 115.1]	65.2 [32.6; 124.3]	-25.2 [-56.3; 19.9]	-7.5 [-61.6; 58.1]	-32.6 [-67.6; 27.1]	1.38 [-0.78; 3.17]	1.36 [-8.59; 11.59]	1.37 [-0.75; 3.33]
Estonia	Country	54.3 [50.8; 58.2]	12.8 [11.7; 13.9]	5.0 [3.9; 6.6]	-41.5 [-45.4; -37.8]†	-7.8 [-9.4; -6.0]†	-49.3 [-53.3; -45.5]†	5.89 [5.35; 6.20]†	10.61 [8.21; 15.20]†	7.03 [6.37; 8.05]†
Eswatini	Country	139.5 [123.9; 157.1]	77.0 [56.5; 103.6]	68.7 [40.9; 115.3]	-62.5 [-89.8; -30.8]†	-8.3 [-47.2; 42.8]	-70.8 [-104.3; -21.5]†	2.34 [1.08; 3.69]†	1.30 [-6.17; 8.97]	2.09 [0.51; 3.76]†
Ethiopia	Country	177.3 [161.8; 194.9]	79.4 [69.8; 90.4]	69.9 [46.7; 102.9]	-97.9 [-117.7; -79.3]†	-9.6 [-35.1; 25.6]	-107.5 [-137.3; -69.7]†	3.56 [2.60; 3.84]†	1.68 [-3.67; 6.84]	3.11 [1.60; 4.07]†
Fiji	Country	68.2 [61.7; 75.5]	23.7 [21.5; 26.2]	21.2 [14.2; 31.5]	-44.5 [-52.1; -37.4]†	-2.6 [-10.0; 8.0]	-47.1 [-57.0; -35.2]†	4.55 [3.66; 4.80]†	1.24 [-3.66; 6.62]	3.75 [2.32; 4.80]†
Finland	Country	12.3 [11.4; 13.2]	6.4 [5.9; 6.9]	3.1 [2.6; 3.7]	-5.9 [-6.9; -4.9]†	-3.3 [-4.0; -2.5]†	-9.2 [-10.3; -8.1]†	2.70 [2.21; 3.04]†	8.59 [6.50; 11.67]†	4.13 [3.57; 4.78]†
France	Country	12.9 [12.0; 13.7]	8.2 [7.6; 8.8]	3.5 [3.2; 3.9]	-4.7 [-5.7; -3.6]†	-4.7 [-5.3; -4.0]†	-9.4 [-10.3; -8.4]†	1.59 [1.43; 2.18]†	10.75 [9.05; 12.07]†	3.81 [3.57; 4.30]†
French Guiana	Country	99.8 [77.1; 128.8]	72.8 [66.6; 79.7]	53.4 [41.3; 67.8]	-26.9 [-56.4; -3.5]†	-19.4 [-33.2; -4.2]†	-46.3 [-77.5; -19.9]†	1.09 [0.18; 2.34]†	3.90 [0.76; 7.25]†	1.77 [0.84; 2.97]†
French Polynesia	Country	63.8 [58.1; 70.3]	37.0 [31.6; 43.1]	24.3 [18.8; 31.7]	-26.9 [-35.3; -18.5]†	-12.6 [-20.7; -3.6]†	-39.5 [-48.2; -30.3]†	2.17 [1.46; 2.91]†	4.91 [1.39; 9.00]†	2.83 [2.08; 3.77]†
Gabon	Country	156.6 [139.8; 175.5]	101.7 [85.9; 120.7]	91.7 [64.7; 129.5]	-54.9 [-79.4; -29.1]†	-10.0 [-42.9; 30.8]	-64.9 [-99.2; -25.4]†	2.03 [0.88; 2.55]†	0.81 [-3.51; 6.13]	1.73 [0.56; 2.77]†
Gambia	Country	160.6 [145.5; 177.8]	76.9 [64.2; 92.4]	58.1 [39.4; 86.4]	-83.8 [-105.2; -61.9]†	-18.8 [-42.7; 12.5]	-102.5 [-128.8; -70.9]†	2.82 [2.12; 3.78]†	3.59 [-2.01; 8.83]	3.00 [1.85; 4.32]†
Georgia	Country	62.3 [58.4; 66.6]	48.1 [44.2; 52.3]	21.1 [17.6; 25.7]	-14.2 [-20.1; -8.3]†	-27.0 [-32.5; -21.0]†	-41.2 [-46.8; -35.4]†	0.95 [0.60; 1.46]†	9.26 [7.67; 12.81]†	2.96 [2.67; 3.88]†
Germany	Country	21.2 [19.9; 22.7]	8.1 [7.6; 8.7]	5.5 [4.2; 7.0]	-13.1 [-14.7; -11.6]†	-2.6 [-4.0; -1.0]†	-15.8 [-17.7; -13.7]†	3.51 [3.46; 4.22]†	4.43 [1.72; 8.29]†	3.73 [3.33; 4.91]†
Ghana	Country	111.0 [100.9; 121.2]	72.5 [64.5; 81.1]	58.2 [43.5; 77.0]	-38.5 [-51.8; -25.6]†	-14.2 [-32.0; 6.0]	-52.8 [-70.7; -31.4]†	1.76 [1.13; 2.30]†	1.99 [-1.04; 6.68]	1.82 [1.05; 2.87]†
Greece	Country	20.8 [19.3; 22.4]	8.4 [7.7; 9.2]	7.0 [5.5; 9.1]	-12.4 [-14.2; -10.7]†	-1.4 [-3.2; 0.8]	-13.8 [-16.0; -11.3]†	3.62 [3.14; 4.10]†	1.93 [-1.20; 5.70]	3.21 [2.48; 4.09]†
Grenada	Country	99.7 [90.5; 109.1]	35.8 [30.5; 42.1]	29.1 [19.6; 43.1]	-63.8 [-74.6; -52.8]†	-6.7 [-18.1; 7.9]	-70.6 [-83.8; -53.6]†	4.19 [3.35; 4.83]†	2.10 [-2.67; 7.88]	3.68 [2.47; 4.94]†
Guadeloupe	Country	34.8 [29.1; 41.7]	15.1 [13.5; 16.8]	8.3 [6.3; 10.8]	-19.7 [-26.8; -13.8]†	-6.8 [-9.5; -3.8]†	-26.5 [-33.8; -20.1]†	3.37 [2.52; 4.20]†	7.06 [3.81; 11.18]†	4.26 [3.35; 5.37]†
Guam	Country	68.7 [53.3; 89.4]	41.0 [36.9; 45.7]	33.4 [22.6; 49.5]	-27.8 [-48.6; -11.8]†	-7.6 [-19.4; 9.1]	-35.4 [-58.7; -13.1]†	2.07 [0.98; 3.20]†	2.51 [-2.53; 7.64]	2.17 [0.78; 3.60]†
Guatemala	Country	121.4 [112.5; 131.0]	85.8 [78.5; 93.8]	68.3 [53.3; 86.3]	-35.6 [-47.6; -23.8]†	-17.5 [-34.4; 1.8]	-53.1 [-71.1; -33.4]†	1.61 [0.93; 1.85]†	3.26 [-0.26; 6.17]	2.01 [1.00; 2.54]†
Guinea	Country	148.2 [135.9; 161.0]	129.7 [115.8; 144.8]	118.6 [85.1; 162.6]	-18.5 [-37.4; 0.8]	-11.1 [-48.9; 34.5]	-29.6 [-65.4; 16.7]	0.79 [-0.02; 1.10]	1.56 [-3.07; 5.58]	0.98 [-0.33; 1.72]
Guinea-Bissau	Country	116.7 [99.8; 135.6]	98.4 [81.5; 118.6]	82.0 [52.9; 125.7]	-18.3 [-43.9; 8.2]	-16.4 [-51.8; 30.4]	-34.7 [-69.6; 12.3]	0.80 [-0.31; 1.65]	2.59 [-3.54; 8.21]	1.24 [-0.33; 2.48]
Guyana	Country	106.7 [92.4; 123.0]	80.1 [65.8; 96.5]	69.9 [45.8; 105.1]	-26.6 [-47.9; -4.2]†	-10.2 [-39.4; 27.5]	-36.7 [-66.2; 1.8]	1.69 [0.18; 2.11]†	1.77 [-3.92; 7.44]	1.71 [-0.05; 2.63]
Haiti	Country	83.0 [72.9; 94.8]	57.3 [48.5; 67.3]	49.8 [31.2; 79.1]	-25.7 [-40.4; -11.7]†	-7.5 [-29.3; 22.5]	-33.3 [-55.7; -1.7]†	2.08 [0.66; 2.35]†	2.02 [-4.33; 8.13]	2.06 [0.06; 3.04]†
Honduras	Country	130.4 [111.8; 151.6]	88.1 [55.5; 135.7]	82.1 [43.2; 152.2]	-42.3 [-81.3; 9.4]	-6.0 [-68.2; 70.0]	-48.3 [-92.2; 24.7]	1.92 [-0.30; 3.53]	1.12 [-8.55; 10.61]	1.72 [-0.56; 3.39]
Hungary	Country	37.5 [35.0; 40.1]	23.7 [22.1; 25.3]	17.5 [13.5; 22.5]	-13.8 [-16.8; -10.8]†	-6.1 [-10.4; -0.9]†	-19.9 [-24.7; -14.6]†	2.19 [1.46; 2.22]†	3.41 [0.52; 7.07]†	2.48 [1.54; 3.11]†
Iceland	Country	33.2 [30.5; 36.1]	7.5 [6.7; 8.4]	3.4 [2.5; 4.5]	-25.7 [-28.6; -22.8]†	-4.1 [-5.4; -2.8]†	-29.8 [-32.8; -26.9]†	6.04 [5.38; 6.51]†	9.45 [6.24; 14.05]†	6.87 [6.04; 7.87]†
India	Country	138.2 [126.4; 151.0]	18.2 [16.4; 20.1]	14.1 [10.3; 19.5]	-120.0 [-132.7; -108.1]†	-4.1 [-8.4; 1.4]	-124.1 [-137.1; -111.2]†	8.01 [7.58; 8.65]†	6.73 [-0.96; 7.37]	7.70 [5.93; 7.90]†
Indonesia	Country	74.8 [69.7; 80.5]	37.9 [33.4; 43.1]	26.4 [19.5; 35.7]	-37.0 [-44.2; -29.4]†	-11.4 [-20.2; -1.3]†	-48.4 [-57.3; -38.2]†	2.74 [2.11; 3.31]†	5.01 [0.47; 8.60]†	3.29 [2.23; 4.10]†
Iran (Islamic Republic of)	Country	87.7 [75.2; 102.3]	37.4 [32.9; 42.6]	26.2 [20.6; 33.6]	-50.3 [-65.3; -37.0]†	-11.2 [-18.9; -2.8]†	-61.5 [-77.4; -46.8]†	3.17 [2.63; 4.20]†	4.57 [0.99; 7.88]†	3.51 [2.77; 4.53]†
Iraq	Country	72.4 [63.0; 83.2]	76.4 [62.6; 91.6]	58.0 [35.8; 93.0]	4.0 [-13.4; 22.1]	-18.4 [-45.5; 18.3]	-14.4 [-39.7; 21.6]	-0.15 [-1.15; 0.75]	3.77 [-2.89; 9.89]	0.80 [-0.82; 2.23]
Ireland	Country	16.0 [14.9; 17.2]	8.0 [7.5; 8.6]	4.1 [3.2; 5.3]	-8.0 [-9.3; -6.7]†	-3.9 [-5.0; -2.6]†	-11.9 [-13.5; -10.3]†	2.78 [2.36; 3.18]†	8.14 [5.06; 11.73]†	4.08 [3.33; 4.96]†
Israel	Country	18.9 [17.7; 20.2]	9.7 [8.9; 10.6]	6.2 [4.8; 8.0]	-9.2 [-10.8; -7.7]†	-3.5 [-5.1; -1.5]†	-12.7 [-14.7; -10.6]†	2.84 [2.23; 3.13]†	5.57 [2.19; 8.91]†	3.50 [2.58; 4.16]†
Italy	Country	8.9 [8.4; 9.6]	5.1 [4.8; 5.5]	2.9 [2.2; 3.7]	-3.8 [-4.5; -3.1]†	-2.3 [-3.0; -1.4]†	-6.1 [-7.0; -5.0]†	2.12 [1.84; 2.61]†	7.45 [4.02; 10.68]†	3.41 [2.66; 4.26]†
Jamaica	Country	108.3 [91.3; 127.5]	45.2 [41.5; 49.4]	36.5 [24.9; 53.3]	-63.1 [-82.0; -45.7]†	-8.6 [-21.0; 8.5]	-71.7 [-93.4; -48.9]†	3.54 [2.73; 4.20]†	3.04 [-2.13; 7.50]	3.42 [2.07; 4.52]†
Japan	Country	3.6 [3.3; 3.8]	4.0 [3.8; 4.3]	1.7 [1.4; 2.1]	0.5 [0.1; 0.9]†	-2.3 [-2.7; -1.8]†	-1.8 [-2.2; -1.4]†	-0.47 [-0.90; -0.12]†	10.65 [7.95; 13.11]†	2.22 [1.54; 2.81]†
Jersey	Country	26.3 [23.5; 29.4]	12.4 [10.8; 14.2]	8.5 [6.2; 11.7]	-13.9 [-17.2; -10.5]†	-3.9 [-6.8; -0.4]†	-17.7 [-21.6; -13.5]†	2.80 [2.29; 3.70]†	4.62 [0.38; 8.90]†	3.24 [2.38; 4.40]†
Jordan	Country	54.9 [50.9; 59.1]	30.4 [26.0; 35.6]	18.4 [13.4; 24.8]	-24.5 [-30.5; -17.9]†	-12.0 [-19.3; -4.1]†	-36.5 [-43.2; -29.0]†	2.85 [1.67; 3.04]†	5.55 [1.99; 10.82]†	3.50 [2.38; 4.30]†
Kazakhstan	Country	49.3 [45.1; 53.6]	32.0 [29.5; 34.8]	18.2 [15.2; 22.1]	-17.3 [-22.3; -12.5]†	-13.8 [-18.0; -9.1]†	-31.1 [-36.4; -25.5]†	1.95 [1.26; 2.19]†	5.61 [4.39; 9.56]†	2.84 [2.38; 3.63]†
Kenya	Country	133.5 [123.2; 144.7]	81.9 [71.9; 92.5]	56.3 [45.9; 69.2]	-51.7 [-66.8; -36.8]†	-25.6 [-40.9; -9.1]†	-77.2 [-92.5; -60.7]†	2.03 [1.37; 2.57]†	3.74 [1.61; 7.68]†	2.45 [1.94; 3.28]†
Kiribati	Country	67.0 [55.6; 81.1]	49.5 [39.6; 61.6]	44.0 [27.7; 69.3]	-17.5 [-34.6; -1.0]†	-5.5 [-26.0; 21.4]	-23.0 [-44.5; 4.5]	0.91 [0.07; 2.35]†	0.90 [-4.85; 7.76]	0.90 [-0.21; 2.79]
Kosovo (under UNSC res. 1244)	Country	34.6 [31.7; 37.7]	9.8 [8.9; 10.8]	7.8 [5.6; 11.2]	-24.8 [-28.0; -21.7]†	-2.0 [-4.5; 1.4]	-26.8 [-30.6; -22.4]†	5.07 [4.52; 5.55]†	2.98 [-1.73; 7.27]	4.57 [3.40; 5.57]†
Kuwait	Country	25.3 [21.6; 29.7]	7.4 [6.7; 8.2]	1.6 [1.3; 2.0]	-17.9 [-22.3; -14.1]†	-5.8 [-6.6; -5.1]†	-23.7 [-28.1; -20.0]†	4.30 [4.18; 5.65]†	18.79 [16.40; 22.23]†	7.82 [7.57; 9.22]†

†: Change is significantly different from zero.
Total Change (Year 1–Year 2): ASFR (Year 2) - ASFR (Year 1).
Annual Rate of Reduction (Year 1–Year 2): $\log(\text{ASFR}_{\text{Year 2}}/\text{ASFR}_{\text{Year 1}})/(\text{Year 1} - \text{Year 2})$, where Year 1 is smaller than Year 2.

Continued on next page

Table 14 – continued from previous page

Age-specific fertility rate 15–19										
		Estimate (per 1,000)			Total Change (per 1,000)			Annual Rate of Reduction (per 100)		
**Location Name**	**Location Type**	**1990**	**2015**	**2023**	**1990–2015**	**2015–2023**	**1990–2023**	**1990–2015**	**2015–2023**	**1990–2023**
Kyrgyz Republic	Country	47.7 [44.2; 51.3]	42.8 [39.3; 46.8]	28.3 [23.5; 34.3]	-4.8 [-9.9; 0.2]	-14.5 [-20.7; -7.5]†	-19.4 [-25.6; -12.4]†	0.82 [-0.02; 0.88]	3.94 [2.53; 7.70]†	1.58 [0.95; 2.19]†
Lao People's Democratic Republic	Country	105.8 [92.6; 120.9]	84.8 [70.6; 101.4]	81.7 [59.9; 109.9]	-21.0 [-41.8; -0.1]†	-3.1 [-30.6; 28.1]	-24.1 [-51.1; 6.6]	1.00 [0.00; 1.78]†	0.50 [-3.91; 5.02]	0.88 [-0.19; 1.82]
Latvia	Country	49.5 [46.3; 52.8]	17.6 [16.4; 19.0]	7.6 [6.3; 9.2]	-31.8 [-35.3; -28.3]†	-10.1 [-12.0; -8.1]†	-41.9 [-45.4; -38.3]†	4.12 [3.72; 4.52]†	9.53 [7.96; 13.13]†	5.43 [5.05; 6.29]†
Lebanon	Country	48.7 [40.6; 57.8]	22.7 [12.1; 41.7]	20.8 [9.7; 46.8]	-26.0 [-40.5; -5.8]†	-1.9 [-24.1; 25.5]	-27.8 [-42.8; -1.0]†	3.86 [0.53; 5.66]†	0.59 [-11.37; 13.45]	3.06 [0.06; 4.98]†
Lesotho	Country	88.6 [78.4; 99.9]	83.9 [70.3; 99.9]	70.6 [47.6; 104.2]	-4.7 [-21.6; 13.5]	-13.3 [-41.9; 21.9]	-18.0 [-43.8; 16.2]	0.56 [-0.59; 1.05]	2.23 [-3.11; 7.65]	0.97 [-0.52; 1.97]
Liberia	Country	168.5 [154.0; 183.9]	140.3 [124.5; 158.3]	126.0 [94.2; 167.6]	-28.2 [-50.7; -5.0]†	-14.3 [-50.6; 29.6]	-42.5 [-77.9; 1.1]	0.91 [0.13; 1.33]†	1.67 [-2.48; 5.31]	1.09 [-0.02; 1.80]
Libya	Country	14.2 [11.3; 17.6]	7.4 [5.3; 10.5]	5.9 [3.3; 10.6]	-6.8 [-10.8; -2.6]†	-1.5 [-5.6; 3.6]	-8.3 [-12.7; -2.8]†	2.83 [0.97; 4.22]†	2.59 [-5.56; 11.30]	2.77 [0.76; 4.54]†
Lithuania	Country	42.3 [39.5; 45.3]	14.5 [13.5; 15.7]	5.9 [4.8; 7.2]	-27.7 [-30.9; -24.8]†	-8.7 [-10.2; -7.0]†	-36.4 [-39.6; -33.3]†	4.31 [3.88; 4.67]†	10.74 [8.61; 14.04]†	5.87 [5.33; 6.64]†
Luxembourg	Country	14.3 [12.9; 15.7]	5.3 [4.7; 6.1]	4.0 [3.0; 5.5]	-8.9 [-10.5; -7.4]†	-1.3 [-2.6; 0.3]	-10.3 [-12.0; -8.3]†	3.68 [3.26; 4.58]†	4.30 [-0.75; 7.72]	3.83 [2.86; 4.81]†
Madagascar	Country	132.6 [122.2; 143.7]	135.6 [118.2; 155.1]	129.8 [95.3; 178.7]	3.0 [-17.5; 24.8]	-5.8 [-44.9; 46.0]	-2.8 [-38.6; 47.2]	-0.18 [-0.72; 0.55]	0.66 [-3.77; 4.74]	0.02 [-0.93; 1.01]
Malawi	Country	155.1 [143.1; 168.3]	128.2 [112.3; 145.7]	113.6 [75.5; 163.7]	-26.9 [-47.6; -5.8]†	-14.6 [-58.1; 37.9]	-41.5 [-82.1; 10.0]	0.87 [0.16; 1.39]†	1.23 [-3.35; 6.98]	0.96 [-0.19; 2.20]
Malaysia	Country	19.0 [16.6; 21.6]	11.8 [10.8; 12.8]	6.0 [4.9; 7.2]	-7.2 [-10.0; -4.6]†	-5.8 [-7.3; -4.2]†	-13.0 [-15.8; -10.3]†	2.00 [1.27; 2.52]†	8.15 [5.81; 11.21]†	3.50 [2.79; 4.24]†
Maldives	Country	130.4 [113.1; 149.9]	11.7 [10.4; 13.2]	5.4 [3.7; 8.0]	-118.7 [-138.1; -101.3]†	-6.3 [-8.5; -3.4]†	-125.0 [-144.4; -107.5]†	10.34 [8.89; 10.35]†	8.69 [4.55; 14.79]†	9.94 [8.39; 10.87]†
Mali	Country	157.3 [146.1; 169.4]	159.1 [139.2; 180.8]	138.6 [101.2; 189.2]	1.8 [-21.6; 26.3]	-20.6 [-64.8; 32.7]	-18.7 [-58.3; 31.4]	0.09 [-0.64; 0.57]	1.95 [-2.51; 6.01]	0.54 [-0.56; 1.36]
Malta	Country	6.5 [5.9; 7.1]	12.7 [11.2; 14.4]	10.4 [7.8; 13.7]	6.2 [4.6; 8.0]†	-2.3 [-5.4; 1.3]	3.9 [1.3; 7.3]†	-2.39 [-3.33; -2.09]†	2.06 [-1.24; 6.37]	-1.31 [-2.32; -0.54]†
Martinique	Country	33.6 [30.4; 37.0]	14.0 [12.4; 15.8]	9.3 [7.0; 12.4]	-19.6 [-23.3; -16.0]†	-4.7 [-7.6; -1.3]†	-24.3 [-28.3; -19.9]†	3.20 [2.88; 4.10]†	5.00 [1.24; 8.89]†	3.64 [2.98; 4.79]†
Mauritania	Country	117.5 [105.3; 131.1]	99.0 [84.1; 116.0]	88.9 [62.9; 123.8]	-18.6 [-38.4; 2.6]	-10.1 [-41.6; 26.4]	-28.6 [-58.3; 8.1]	0.79 [-0.10; 1.46]	1.56 [-3.19; 6.14]	0.98 [-0.21; 1.95]
Mauritius	Country	43.8 [39.8; 48.1]	23.3 [21.2; 25.6]	19.8 [16.3; 24.0]	-20.5 [-25.3; -15.9]†	-3.5 [-7.8; 1.2]	-24.0 [-29.5; -18.2]†	2.64 [1.98; 3.05]†	1.84 [-0.67; 4.81]	2.45 [1.76; 3.06]†
Mayotte	Country	127.6 [90.6; 175.5]	69.0 [62.1; 76.2]	63.2 [42.7; 92.1]	-58.6 [-106.9; -20.7]†	-5.7 [-27.9; 23.2]	-64.4 [-116.1; -16.7]†	2.37 [1.04; 3.82]†	0.57 [-3.71; 6.23]	1.94 [0.56; 3.69]†
Mexico	Country	84.1 [60.7; 113.0]	69.3 [63.9; 75.4]	60.1 [41.7; 85.1]	-14.7 [-43.7; 9.1]	-9.2 [-28.4; 16.7]	-24.0 [-58.5; 10.0]	0.93 [-0.55; 1.97]	2.34 [-2.74; 6.47]	1.27 [-0.42; 2.46]
Micronesia (Fed. States of)	Country	65.9 [50.4; 85.7]	47.6 [29.5; 76.0]	43.7 [22.1; 86.1]	-18.3 [-45.8; 13.8]	-3.9 [-39.6; 41.6]	-22.2 [-52.5; 21.8]	1.37 [-0.84; 3.50]	1.30 [-9.39; 11.36]	1.35 [-0.90; 3.46]
Mongolia	Country	36.2 [33.2; 39.3]	30.3 [27.8; 33.1]	19.7 [16.2; 24.1]	-5.9 [-10.0; -1.8]†	-10.5 [-14.9; -5.6]†	-16.4 [-21.2; -11.4]†	1.09 [0.21; 1.20]†	4.87 [2.67; 7.93]†	2.01 [1.20; 2.48]†
Montenegro	Country	27.4 [25.5; 29.4]	11.2 [10.0; 12.6]	9.4 [7.5; 11.7]	-16.2 [-18.6; -13.8]†	-1.9 [-4.2; 0.7]	-18.0 [-20.8; -15.0]†	3.65 [3.01; 4.12]†	2.59 [-0.75; 5.33]	3.40 [2.56; 3.95]†
Morocco	Country	45.3 [40.2; 51.0]	28.8 [18.0; 45.5]	25.1 [13.0; 48.7]	-16.5 [-28.7; 1.2]	-3.7 [-24.7; 22.6]	-20.2 [-33.8; 3.5]	2.05 [-0.11; 3.77]	1.41 [-8.57; 11.96]	1.90 [-0.23; 3.79]
Mozambique	Country	164.9 [148.8; 182.0]	168.9 [144.6; 194.9]	153.4 [116.2; 199.6]	4.0 [-26.3; 34.6]	-15.5 [-60.5; 35.1]	-11.5 [-52.3; 36.8]	-0.22 [-0.80; 0.65]	1.39 [-2.53; 4.96]	0.17 [-0.63; 1.11]
Myanmar	Country	49.7 [42.6; 58.3]	35.2 [27.6; 44.9]	33.5 [19.8; 56.2]	-14.6 [-26.0; -2.1]†	-1.7 [-18.9; 21.5]	-16.2 [-32.6; 7.1]	1.53 [0.20; 2.53]†	0.75 [-6.37; 7.80]	1.34 [-0.43; 2.85]
Namibia	Country	93.7 [85.1; 103.1]	75.2 [56.1; 99.3]	66.0 [37.8; 110.8]	-18.5 [-39.4; 7.2]	-9.2 [-46.6; 39.1]	-27.7 [-57.0; 17.5]	0.61 [-0.30; 2.11]	1.47 [-5.83; 9.35]	0.82 [-0.53; 2.75]
Nepal	Country	134.7 [125.9; 144.7]	81.6 [72.5; 91.8]	67.2 [49.0; 91.2]	-53.1 [-66.8; -39.6]†	-14.5 [-35.0; 11.1]	-67.6 [-88.2; -41.7]†	1.93 [1.47; 2.58]†	2.92 [-1.61; 6.59]	2.17 [1.15; 3.08]†
Netherlands	Country	8.8 [8.2; 9.4]	3.2 [3.0; 3.4]	1.9 [1.4; 2.4]	-5.6 [-6.3; -5.0]†	-1.3 [-1.8; -0.7]†	-7.0 [-7.7; -6.1]†	3.75 [3.69; 4.48]†	7.10 [3.31; 9.97]†	4.56 [3.86; 5.51]†
New Caledonia	Country	46.0 [41.8; 50.9]	18.0 [15.9; 20.4]	12.2 [9.3; 16.0]	-28.0 [-33.3; -23.1]†	-5.9 [-9.7; -1.4]†	-33.9 [-39.7; -28.2]†	3.85 [3.12; 4.41]†	5.16 [1.06; 8.59]†	4.16 [3.16; 4.91]†
New Zealand	Country	35.6 [33.2; 38.0]	18.8 [17.2; 20.5]	10.9 [9.0; 13.1]	-16.8 [-19.7; -13.9]†	-7.9 [-10.4; -5.2]†	-24.7 [-27.8; -21.4]†	2.50 [2.12; 3.01]†	6.48 [4.22; 9.45]†	3.46 [2.97; 4.20]†
Nicaragua	Country	148.9 [136.4; 162.1]	102.1 [70.2; 144.2]	93.5 [52.8; 159.3]	-46.8 [-81.1; -2.8]†	-8.6 [-66.7; 64.3]	-55.5 [-99.6; 11.8]	1.52 [0.08; 3.02]†	0.92 [-6.92; 9.50]	1.38 [-0.24; 3.19]
Niger	Country	212.3 [196.5; 229.3]	178.4 [152.9; 206.8]	145.3 [105.7; 198.9]	-33.9 [-64.6; -1.8]†	-33.1 [-81.9; 25.1]	-66.9 [-110.3; -12.1]†	0.88 [0.04; 1.38]†	2.67 [-1.75; 6.92]	1.32 [0.18; 2.14]†
Nigeria	Country	143.0 [131.6; 155.3]	114.9 [102.7; 128.9]	86.4 [63.3; 118.4]	-28.1 [-45.7; -10.0]†	-28.4 [-55.4; 4.8]	-56.6 [-83.5; -23.4]†	1.00 [0.30; 1.43]†	3.45 [-0.56; 7.73]	1.59 [0.56; 2.53]†
North Macedonia	Country	50.1 [46.9; 53.5]	19.2 [17.5; 21.0]	13.0 [10.7; 15.8]	-31.0 [-34.9; -27.2]†	-6.2 [-9.1; -2.8]†	-37.2 [-41.4; -32.9]†	3.75 [3.39; 4.31]†	4.73 [2.05; 7.54]†	3.99 [3.46; 4.72]†
Norway	Country	17.7 [16.6; 19.0]	4.6 [4.2; 4.9]	1.4 [1.2; 1.7]	-13.2 [-14.5; -11.9]†	-3.2 [-3.6; -2.7]†	-16.3 [-17.6; -15.1]†	5.35 [5.00; 5.82]†	14.28 [12.12; 17.41]†	7.51 [7.06; 8.33]†
Oman	Country	109.7 [86.2; 137.1]	14.9 [13.7; 16.4]	6.0 [4.9; 7.3]	-94.7 [-122.0; -71.1]†	-9.0 [-10.8; -7.2]†	-103.7 [-130.9; -79.8]†	8.99 [6.95; 8.92]†	10.23 [8.75; 14.16]†	9.29 [7.85; 9.72]†
Pakistan	Country	104.4 [95.2; 114.2]	48.7 [42.0; 56.5]	41.1 [29.3; 57.8]	-55.7 [-67.3; -43.6]†	-7.6 [-21.9; 10.3]	-63.3 [-78.4; -44.6]†	3.56 [2.35; 3.73]†	1.75 [-2.48; 6.80]	3.12 [1.76; 3.87]†
Panama	Country	96.2 [87.6; 105.3]	86.6 [79.6; 94.1]	57.3 [45.2; 72.4]	-9.5 [-21.1; 1.8]	-29.3 [-43.8; -13.0]†	-38.8 [-54.2; -21.6]†	0.48 [-0.08; 0.91]	5.64 [2.06; 8.34]†	1.73 [0.81; 2.34]†
Papua New Guinea	Country	84.6 [71.3; 100.4]	61.7 [53.3; 71.7]	54.1 [34.9; 83.5]	-22.8 [-40.9; -6.5]†	-7.7 [-29.7; 23.8]	-30.5 [-55.9; 1.9]	0.59 [0.36; 2.17]†	1.86 [-4.15; 7.54]	0.90 [-0.06; 2.83]
Paraguay	Country	101.1 [88.9; 114.3]	76.9 [61.8; 94.8]	70.3 [42.5; 114.2]	-24.2 [-45.0; -2.3]†	-6.6 [-39.9; 38.1]	-30.7 [-61.8; 14.4]	1.42 [0.10; 2.13]†	1.22 [-5.46; 7.87]	1.37 [-0.40; 2.67]
Peru	Country	84.5 [71.2; 99.8]	55.1 [46.4; 65.0]	43.6 [31.8; 58.9]	-29.4 [-46.3; -12.9]†	-11.5 [-27.0; 6.3]	-41.0 [-60.3; -20.3]†	1.75 [0.75; 2.64]†	3.73 [-1.45; 7.39]	2.23 [0.94; 3.09]†
Philippines	Country	52.6 [49.0; 56.6]	53.6 [49.2; 58.3]	31.9 [24.7; 40.9]	1.0 [-5.0; 7.0]	-21.7 [-30.2; -11.8]†	-20.7 [-29.3; -11.1]†	0.43 [-0.52; 0.38]	6.60 [3.18; 9.84]†	1.93 [0.74; 2.33]†
Poland	Country	30.2 [28.2; 32.3]	12.7 [11.9; 13.6]	6.2 [5.1; 7.5]	-17.5 [-19.7; -15.2]†	-6.5 [-7.9; -5.0]†	-24.0 [-26.3; -21.7]†	3.82 [3.06; 3.83]†	7.84 [6.51; 11.57]†	4.79 [4.20; 5.41]†
Portugal	Country	23.7 [22.1; 25.3]	8.3 [7.7; 8.9]	7.0 [5.5; 8.9]	-15.4 [-17.1; -13.7]†	-1.3 [-2.9; 0.7]	-16.7 [-18.8; -14.2]†	4.19 [3.81; 4.59]†	2.55 [-1.05; 5.25]	3.79 [2.92; 4.46]†

†: Change is significantly different from zero.
Total Change (Year 1–Year 2): ASFR (Year 2) - ASFR (Year 1).
Annual Rate of Reduction (Year 1–Year 2): $\log(\text{ASFR}_{\text{Year 2}}/\text{ASFR}_{\text{Year 1}})/(\text{Year 1} - \text{Year 2})$, where Year 1 is smaller than Year 2.

Continued on next page

Table 14 – continued from previous page

Age-specific fertility rate 15–19										
		Estimate (per 1,000)			Total Change (per 1,000)			Annual Rate of Reduction (per 100)		
**Location Name**	**Location Type**	**1990**	**2015**	**2023**	**1990–2015**	**2015–2023**	**1990–2023**	**1990–2015**	**2015–2023**	**1990–2023**
Puerto Rico	Country	74.7 [68.8; 81.3]	34.7 [31.8; 37.8]	14.1 [11.6; 17.0]	-40.0 [-47.1; -33.3]†	-20.6 [-24.6; -16.5]†	-60.6 [-67.6; -54.0]†	3.15 [2.59; 3.55]†	11.57 [8.66; 13.90]†	5.19 [4.42; 5.68]†
Qatar	Country	53.3 [48.4; 58.7]	10.6 [9.5; 11.8]	5.7 [4.4; 7.5]	-42.7 [-48.1; -37.6]†	-4.9 [-6.7; -2.8]†	-47.6 [-53.3; -42.4]†	6.60 [5.88; 7.03]†	7.37 [4.07; 11.28]†	6.78 [5.90; 7.65]†
Republic of Korea	Country	4.3 [4.0; 4.6]	1.6 [1.5; 1.8]	0.5 [0.4; 0.7]	-2.6 [-3.0; -2.3]†	-1.1 [-1.3; -0.9]†	-3.7 [-4.1; -3.4]†	4.27 [3.42; 4.23]†	16.74 [11.13; 16.69]†	7.29 [5.60; 6.95]†
Republic of Moldova	Country	58.1 [53.6; 62.7]	35.7 [32.8; 38.8]	23.0 [19.0; 27.8]	-22.4 [-27.9; -17.0]†	-12.7 [-17.7; -7.0]†	-35.0 [-41.3; -28.5]†	2.39 [1.49; 2.41]†	4.44 [2.88; 8.08]†	2.89 [2.19; 3.42]†
Réunion	Country	42.5 [38.6; 46.6]	24.4 [22.3; 26.8]	15.2 [11.7; 19.6]	-18.1 [-22.9; -13.4]†	-9.3 [-13.5; -4.2]†	-27.4 [-32.8; -21.5]†	2.33 [1.66; 2.76]†	5.97 [2.52; 9.46]†	3.21 [2.29; 3.97]†
Romania	Country	51.7 [48.5; 55.3]	36.5 [33.5; 39.8]	33.8 [26.3; 43.5]	-15.2 [-19.8; -10.5]†	-2.7 [-10.8; 7.4]	-18.0 [-26.2; -7.6]†	1.44 [0.96; 1.82]†	0.77 [-2.35; 4.27]	1.28 [0.50; 2.08]†
Russian Federation	Country	54.5 [51.1; 58.1]	24.7 [22.7; 26.9]	13.0 [10.7; 15.6]	-29.8 [-33.9; -25.8]†	-11.8 [-14.9; -8.4]†	-41.6 [-45.9; -37.2]†	3.24 [2.74; 3.59]†	7.51 [5.48; 10.61]†	4.28 [3.76; 4.95]†
Rwanda	Country	56.9 [51.3; 63.2]	38.6 [33.8; 44.1]	30.8 [20.9; 46.0]	-18.4 [-26.5; -10.5]†	-7.8 [-19.4; 8.2]	-26.1 [-38.1; -10.2]†	1.53 [0.88; 2.26]†	2.99 [-2.49; 7.97]	1.89 [0.62; 3.09]†
Saint Lucia	Country	103.2 [94.2; 112.8]	36.9 [30.5; 44.2]	27.8 [20.0; 39.0]	-66.2 [-77.7; -54.6]†	-9.1 [-19.6; 3.5]	-75.3 [-87.9; -61.5]†	4.11 [3.30; 4.96]†	4.02 [-1.25; 8.13]	4.08 [2.92; 5.00]†
Saint Vincent and the Grenadines	Country	92.4 [79.6; 107.1]	61.4 [55.3; 68.3]	42.3 [32.3; 55.1]	-31.0 [-46.6; -16.4]†	-19.1 [-31.4; -5.1]†	-50.1 [-67.8; -32.4]†	1.56 [0.90; 2.33]†	4.86 [1.13; 8.27]†	2.36 [1.46; 3.32]†
Samoa	Country	51.5 [40.8; 64.8]	50.4 [41.4; 61.3]	43.5 [28.3; 65.3]	-1.0 [-17.1; 14.4]	-6.9 [-25.7; 16.5]	-7.9 [-28.0; 16.3]	-0.21 [-1.13; 1.28]	1.47 [-3.83; 7.67]	0.20 [-0.94; 1.98]
Sao Tome and Principe	Country	131.2 [112.6; 152.2]	101.9 [85.4; 120.6]	86.2 [56.4; 128.2]	-29.3 [-55.6; -2.7]†	-15.7 [-50.8; 28.3]	-45.0 [-80.9; 0.3]	0.88 [0.09; 1.90]†	2.17 [-3.33; 7.85]	1.19 [-0.01; 2.63]
Saudi Arabia	Country	64.3 [54.7; 75.9]	15.2 [9.6; 24.2]	11.1 [6.3; 20.0]	-49.2 [-62.1; -35.7]†	-4.1 [-14.2; 6.6]	-53.2 [-65.7; -40.1]†	6.25 [3.79; 7.76]†	3.12 [-5.78; 13.28]	5.49 [3.44; 7.14]†
Senegal	Country	106.3 [98.6; 114.6]	73.6 [66.2; 81.6]	60.2 [41.4; 86.7]	-32.8 [-43.6; -21.7]†	-13.3 [-34.4; 14.2]	-46.1 [-67.0; -18.7]†	1.59 [0.96; 1.98]†	2.50 [-2.24; 7.42]	1.81 [0.59; 2.87]†
Serbia	Country	33.9 [31.1; 36.9]	16.9 [15.6; 18.6]	13.7 [10.5; 17.5]	-17.0 [-20.2; -13.8]†	-3.3 [-6.8; 0.8]	-20.2 [-24.6; -15.4]†	3.91 [2.29; 3.26]†	2.38 [-0.61; 6.16]	3.54 [1.96; 3.58]†
Seychelles	Country	62.8 [55.5; 70.5]	57.7 [51.5; 64.8]	54.5 [36.1; 80.9]	-5.1 [-15.1; 5.0]	-3.1 [-22.9; 23.5]	-8.2 [-28.6; 19.1]	0.34 [-0.34; 1.00]	0.26 [-4.40; 6.00]	0.32 [-0.82; 1.72]
Sierra Leone	Country	160.2 [144.4; 176.7]	116.3 [104.4; 129.7]	93.6 [64.3; 135.5]	-43.9 [-64.3; -23.4]†	-22.7 [-55.3; 20.6]	-66.6 [-101.3; -22.2]†	1.01 [0.68; 1.87]†	2.75 [-2.13; 7.64]	1.43 [0.47; 2.84]†
Singapore	Country	7.4 [6.7; 8.1]	2.6 [2.3; 2.8]	2.2 [1.8; 2.7]	-4.8 [-5.6; -4.1]†	-0.4 [-0.9; 0.2]	-5.2 [-6.0; -4.4]†	4.18 [3.64; 4.78]†	3.49 [-0.81; 5.11]	4.01 [3.02; 4.41]†
Slovakia	Country	43.4 [40.7; 46.4]	24.3 [22.2; 26.5]	24.6 [19.3; 31.5]	-19.1 [-22.6; -15.6]†	0.3 [-5.3; 7.5]	-18.8 [-25.0; -11.5]†	2.59 [1.90; 2.75]†	-0.44 [-3.42; 3.01]	1.86 [0.95; 2.49]†
Slovenia	Country	24.0 [22.3; 25.7]	4.6 [4.2; 5.0]	3.5 [2.7; 4.6]	-19.4 [-21.2; -17.7]†	-1.1 [-2.0; 0.1]	-20.5 [-22.4; -18.4]†	6.79 [6.15; 7.07]†	2.80 [-0.33; 7.01]	5.82 [4.97; 6.70]†
Solomon Islands	Country	100.8 [83.4; 122.3]	59.5 [41.9; 82.6]	50.4 [31.1; 80.2]	-41.3 [-69.1; -12.0]†	-9.1 [-39.1; 24.5]	-50.4 [-79.6; -15.9]†	2.19 [0.56; 3.72]†	2.75 [-4.95; 9.49]	2.33 [0.56; 3.71]†
Somalia	Country	124.0 [104.8; 146.3]	132.2 [95.5; 179.8]	117.1 [68.7; 189.8]	8.2 [-34.1; 60.0]	-15.1 [-82.5; 64.3]	-6.9 [-60.8; 69.2]	-0.08 [-1.68; 1.19]	1.16 [-5.81; 9.27]	0.22 [-1.41; 1.90]
South Africa	Country	94.8 [85.0; 105.6]	68.0 [62.6; 73.6]	51.6 [40.5; 65.9]	-26.8 [-38.8; -15.4]†	-16.4 [-29.0; -1.0]†	-43.2 [-58.7; -25.9]†	1.64 [0.79; 1.87]†	3.02 [0.19; 6.66]†	1.97 [1.03; 2.65]†
South Sudan	Country	159.1 [132.7; 191.4]	106.8 [67.9; 161.2]	97.1 [51.4; 173.2]	-52.3 [-102.1; 7.8]	-9.7 [-81.2; 75.5]	-61.9 [-119.5; 16.6]	-1.39 [-0.20; 3.54]	0.94 [-7.98; 10.77]	-0.82 [-0.32; 3.55]
Spain	Country	11.7 [11.0; 12.6]	7.7 [7.2; 8.2]	4.8 [3.8; 6.1]	-4.1 [-5.0; -3.1]†	-2.9 [-4.1; -1.5]†	-6.9 [-8.3; -5.4]†	1.70 [1.32; 2.09]†	6.05 [2.69; 9.09]†	2.75 [1.95; 3.47]†
Sri Lanka	Country	29.7 [26.0; 34.0]	17.0 [15.5; 18.8]	15.1 [9.6; 24.2]	-12.7 [-17.2; -8.5]†	-2.0 [-7.7; 7.4]	-14.6 [-21.5; -4.9]†	2.35 [1.55; 2.88]†	1.61 [-4.57; 7.24]	2.17 [0.57; 3.46]†
State of Palestine	Country	112.3 [96.9; 130.5]	47.8 [39.2; 57.7]	36.0 [23.7; 55.2]	-64.6 [-84.4; -45.9]†	-11.7 [-27.5; 8.7]	-76.3 [-98.3; -52.4]†	3.82 [2.44; 4.39]†	3.24 [-2.29; 9.29]	3.68 [2.12; 4.81]†
Sudan	Country	91.1 [79.1; 105.5]	79.7 [59.7; 106.3]	66.1 [38.8; 108.9]	-11.4 [-37.0; 18.4]	-13.6 [-53.6; 33.1]	-25.0 [-56.5; 19.5]	-0.46 [-0.78; 1.88]	2.69 [-4.80; 10.09]	0.30 [-0.62; 2.64]
Suriname	Country	91.8 [83.6; 100.9]	59.9 [54.7; 65.3]	48.0 [37.5; 61.4]	-31.9 [-42.2; -22.1]†	-11.8 [-23.7; 2.4]	-43.8 [-57.9; -28.1]†	1.31 [1.20; 2.23]†	2.69 [-0.50; 6.02]	1.65 [1.17; 2.77]†
Sweden	Country	13.7 [12.7; 14.7]	4.7 [4.4; 5.1]	1.8 [1.5; 2.2]	-9.0 [-10.0; -8.0]†	-2.9 [-3.4; -2.4]†	-11.9 [-12.9; -10.9]†	4.39 [3.86; 4.68]†	11.53 [9.56; 14.54]†	6.12 [5.56; 6.75]†
Switzerland	Country	7.4 [6.9; 7.9]	2.8 [2.6; 3.0]	1.5 [1.2; 1.8]	-4.6 [-5.2; -4.1]†	-1.3 [-1.6; -0.9]†	-5.9 [-6.5; -5.3]†	3.83 [3.50; 4.36]†	8.17 [5.15; 10.36]†	4.88 [4.23; 5.46]†
Syrian Arab Republic	Country	83.5 [70.9; 97.7]	42.7 [25.4; 71.0]	38.9 [19.4; 77.0]	-40.8 [-63.6; -9.9]†	-3.8 [-37.5; 37.8]	-44.6 [-69.2; -4.3]†	2.82 [0.56; 4.88]†	1.88 [-9.71; 11.85]	2.59 [0.17; 4.48]†
Tajikistan	Country	45.2 [41.7; 49.2]	49.4 [45.2; 54.1]	40.4 [28.8; 56.6]	4.2 [-1.7; 10.0]	-9.0 [-21.4; 7.4]	-4.8 [-17.1; 11.7]	-0.34 [-0.84; 0.15]	3.00 [-1.76; 6.85]	0.47 [-0.71; 1.41]
Thailand	Country	50.0 [45.3; 55.0]	48.7 [45.0; 52.7]	26.1 [20.7; 32.8]	-1.3 [-7.4; 4.8]	-22.5 [-29.2; -14.8]†	-23.9 [-31.3; -15.7]†	0.36 [-0.40; 0.60]	8.52 [4.71; 10.79]†	2.34 [1.21; 2.74]†
Timor-Leste	Country	63.2 [55.5; 71.7]	36.8 [30.7; 43.7]	27.4 [17.3; 43.0]	-26.4 [-36.5; -16.1]†	-9.4 [-22.0; 7.9]	-35.8 [-49.3; -17.7]†	2.49 [1.31; 3.03]†	6.41 [-2.55; 9.82]	3.44 [1.09; 3.99]†
Togo	Country	104.3 [94.4; 114.8]	84.6 [72.5; 98.4]	77.0 [49.6; 118.6]	-19.7 [-35.5; -2.6]†	-7.5 [-39.6; 36.2]	-27.2 [-57.1; 16.1]	1.18 [0.11; 1.55]†	1.13 [-4.51; 7.23]	1.17 [-0.43; 2.29]
Tonga	Country	32.3 [27.5; 38.1]	25.6 [19.1; 34.3]	24.8 [15.1; 40.5]	-6.8 [-15.7; 3.2]	-0.7 [-13.5; 15.7]	-7.5 [-18.9; 8.5]	0.59 [-0.41; 2.29]	1.05 [-6.61; 7.59]	0.70 [-0.75; 2.41]
Trinidad and Tobago	Country	63.1 [58.0; 68.8]	39.0 [25.8; 59.1]	36.0 [19.4; 64.8]	-24.1 [-38.7; -3.6]†	-3.0 [-29.2; 28.9]	-27.1 [-44.8; 1.9]	1.93 [0.23; 3.63]†	1.05 [-8.08; 10.56]	1.72 [-0.09; 3.59]
Tunisia	Country	18.2 [16.0; 20.7]	8.8 [8.1; 9.6]	4.3 [3.3; 5.6]	-9.4 [-12.1; -7.1]†	-4.5 [-5.8; -3.0]†	-13.9 [-16.6; -11.4]†	3.11 [2.30; 3.56]†	8.86 [5.50; 12.24]†	4.50 [3.52; 5.22]†
Türkiye	Country	65.9 [58.9; 73.6]	25.6 [24.0; 27.4]	12.1 [10.0; 14.5]	-40.3 [-48.1; -33.1]†	-13.6 [-16.4; -10.7]†	-53.9 [-61.9; -46.3]†	3.90 [3.25; 4.30]†	9.41 [6.98; 11.96]†	5.23 [4.48; 5.82]†
Turkmenistan	Country	22.4 [20.5; 24.6]	28.7 [23.7; 35.0]	21.2 [13.9; 32.8]	6.3 [0.7; 12.9]†	-7.5 [-17.2; 5.1]	-1.2 [-8.9; 10.6]	-0.79 [-1.87; -0.11]†	2.55 [-2.19; 9.74]	0.02 [-1.20; 1.48]
Uganda	Country	180.4 [167.2; 194.2]	126.1 [113.6; 140.1]	107.0 [72.0; 154.8]	-54.3 [-73.8; -35.7]†	-19.2 [-56.4; 30.5]	-73.4 [-111.0; -24.2]†	1.49 [0.93; 1.95]†	2.39 [-2.72; 7.10]	1.71 [0.44; 2.80]†
Ukraine	Country	59.3 [55.4; 63.3]	26.2 [24.5; 28.0]	11.4 [8.2; 15.6]	-33.1 [-37.5; -28.8]†	-14.8 [-18.4; -10.4]†	-47.9 [-53.0; -42.3]†	3.32 [2.89; 3.64]†	9.55 [6.46; 14.51]†	4.83 [4.02; 5.99]†
United Arab Emirates	Country	51.0 [43.2; 60.2]	5.4 [5.0; 6.0]	3.1 [2.5; 3.8]	-45.6 [-54.8; -37.7]†	-2.3 [-3.1; -1.5]†	-47.9 [-57.1; -39.9]†	9.15 [8.19; 9.75]†	7.30 [4.14; 9.77]†	8.70 [7.68; 9.29]†
United Kingdom	Country	34.4 [32.2; 36.8]	14.7 [13.8; 15.8]	8.4 [6.5; 10.7]	-19.7 [-22.3; -17.3]†	-6.4 [-8.5; -3.8]†	-26.0 [-29.1; -22.9]†	3.25 [3.02; 3.77]†	6.32 [3.76; 10.35]†	3.99 [3.51; 5.08]†

†: Change is significantly different from zero.
Total Change (Year 1–Year 2): ASFR (Year 2) - ASFR (Year 1).
Annual Rate of Reduction (Year 1–Year 2): $\log(\text{ASFR}_{\text{Year 2}}/\text{ASFR}_{\text{Year 1}})/(\text{Year 1} - \text{Year 2})$, where Year 1 is smaller than Year 2.

Continued on next page

Table 14 – continued from previous page

Age-specific fertility rate 15–19										
		Estimate (per 1,000)			Total Change (per 1,000)			Annual Rate of Reduction (per 100)		
**Location Name**	**Location Type**	**1990**	**2015**	**2023**	**1990–2015**	**2015–2023**	**1990–2023**	**1990–2015**	**2015–2023**	**1990–2023**
United Republic of Tanzania	Country	137.9 [127.2; 149.0]	122.8 [107.3; 139.6]	113.2 [73.4; 168.9]	-15.1 [-33.7; 4.6]	-9.5 [-53.4; 49.2]	-24.7 [-65.3; 32.9]	0.56 [-0.14; 1.08]	0.75 [-4.34; 6.61]	0.61 [-0.66; 1.90]
United States of America	Country	61.5 [57.7; 65.7]	22.0 [20.6; 23.6]	13.1 [10.9; 15.8]	-39.5 [-44.0; -35.4]†	-8.9 [-11.6; -5.8]†	-48.4 [-53.1; -43.7]†	4.01 [3.73; 4.49]†	6.33 [3.98; 8.95]†	4.57 [4.08; 5.28]†
Uruguay	Country	62.3 [51.8; 75.0]	55.9 [51.4; 60.7]	26.2 [20.3; 33.5]	-6.4 [-19.7; 5.0]	-29.7 [-37.3; -21.0]†	-36.1 [-50.1; -23.5]†	0.54 [-0.36; 1.25]	9.50 [6.22; 12.81]†	2.71 [1.72; 3.57]†
Uzbekistan	Country	44.5 [41.0; 48.1]	25.3 [23.3; 27.6]	34.1 [28.1; 41.2]	-19.2 [-23.4; -14.9]†	8.8 [2.5; 16.3]†	-10.4 [-17.3; -2.5]†	2.67 [1.77; 2.73]†	-5.32 [-6.37; -1.14]†	0.73 [0.18; 1.42]†
Vanuatu	Country	85.5 [63.3; 115.0]	74.4 [52.4; 104.6]	66.2 [37.7; 115.2]	-11.1 [-46.5; 23.7]	-8.2 [-50.6; 45.1]	-19.3 [-60.8; 31.9]	0.64 [-1.15; 2.39]	0.94 [-6.61; 9.70]	0.71 [-1.08; 2.70]
Venezuela (Bolivarian Republic of)	Country	99.2 [85.0; 115.5]	88.5 [81.2; 96.3]	73.3 [53.5; 101.2]	-10.7 [-28.4; 5.3]	-15.2 [-36.4; 13.6]	-25.9 [-51.5; 4.9]	0.27 [-0.24; 1.16]	2.65 [-1.80; 6.37]	0.85 [-0.15; 1.95]
Viet Nam	Country	45.4 [40.9; 50.2]	41.7 [34.3; 50.7]	34.3 [23.8; 49.2]	-3.7 [-12.8; 6.1]	-7.4 [-20.9; 9.3]	-11.1 [-22.9; 4.4]	0.15 [-0.53; 1.23]	2.09 [-2.67; 7.55]	0.62 [-0.29; 2.02]
Western Sahara	Country	100.5 [90.7; 111.4]	34.9 [19.1; 64.0]	31.2 [14.5; 66.2]	-65.6 [-85.0; -34.7]†	-3.7 [-36.7; 35.0]	-69.2 [-89.4; -33.3]†	4.82 [1.76; 6.64]†	1.18 [-10.59; 13.38]	3.94 [1.23; 5.88]†
Yemen	Country	155.8 [142.9; 170.0]	72.7 [54.6; 94.4]	75.3 [55.9; 100.2]	-83.1 [-105.9; -58.3]†	2.7 [-26.0; 33.6]	-80.4 [-104.7; -52.5]†	3.16 [1.97; 4.20]†	-0.70 [-5.46; 4.38]	2.22 [1.31; 3.14]†
Zambia	Country	149.2 [137.6; 161.3]	128.1 [109.9; 148.3]	115.9 [77.7; 169.1]	-21.0 [-42.6; 2.4]	-12.2 [-56.0; 40.8]	-33.3 [-73.0; 19.5]	0.53 [-0.07; 1.29]	1.30 [-3.61; 6.54]	0.72 [-0.38; 1.99]
Zimbabwe	Country	102.5 [93.2; 112.8]	102.7 [87.5; 119.6]	98.1 [66.7; 142.2]	0.2 [-18.3; 19.6]	-4.6 [-40.9; 42.5]	-4.4 [-36.7; 40.9]	0.02 [-0.72; 0.74]	1.04 [-4.58; 5.81]	0.27 [-1.05; 1.31]

†: Change is significantly different from zero.
Total Change (Year 1–Year 2): ASFR (Year 2) - ASFR (Year 1).
Annual Rate of Reduction (Year 1–Year 2): $\log(\text{ASFR}_{\text{Year 2}}/\text{ASFR}_{\text{Year 1}})/(\text{Year 1} - \text{Year 2})$, where Year 1 is smaller than Year 2.

Table 15: **Estimates and uncertainty intervals for births (in thousands) by mothers aged 10–14 and the percentage of births in each location among all births of 10–14 for 1990, 2015, and 2023 for the world, seven SDG regions, 22 sub-regions, and 201 countries/areas*.** *We report results from countries/areas with more than 90,000 population size by mid-2024. Percentages may not sum up to 1 due to rounding.

		Age-specific births 10–14					
		Number (in thousands)			Proportion (in %)		
**Location Name**	**Location Type**	**1990**	**2015**	**2023**	**1990**	**2015**	**2023**
World	World	1,150 [1,050; 1,340]	446 [407; 499]	347 [285; 454]	100	100	100
Sub-Saharan Africa	SDG region	361 [336; 389]	260 [231; 294]	236 [189; 309]	31.2 [26.6; 34.7]	58.2 [52.6; 63.0]	68.1 [53.9; 77.6]
Latin America and the Caribbean	SDG region	76 [63; 97]	73 [62; 86]	44 [32; 62]	6.6 [5.2; 8.4]	16.3 [13.6; 19.3]	12.6 [8.7; 17.9]
Oceania (excluding Australia and New Zealand)	SDG region	2 [1; 3]	1 [1; 2]	1 [0; 0]	0.1 [0.1; 0.2]	0.2 [0.1; 0.5]	0.3 [0.1; 0.9]
Northern Africa and Western Asia	SDG region	44 [36; 62]	20 [13; 33]	17 [8; 39]	3.8 [3.0; 5.3]	4.4 [2.8; 7.1]	4.8 [2.2; 10.8]
Central and Southern Asia	SDG region	592 [500; 704]	73 [54; 100]	35 [17; 104]	51.3 [44.1; 56.2]	16.4 [12.3; 21.4]	10.2 [4.9; 25.4]
Eastern and South-Eastern Asia	SDG region	61 [46; 231]	13 [9; 35]	10 [5; 34]	5.3 [4.0; 17.2]	3.0 [2.1; 7.5]	2.8 [1.5; 9.2]
Europe, Northern America, Australia, and New Zealand	SDG region	19 [15; 24]	7 [5; 8]	4 [3; 7]	1.6 [1.2; 2.1]	1.5 [1.2; 1.9]	1.2 [0.7; 2.2]
Northern America	Subregion	15 [10; 20]	3 [2; 5]	2 [1; 5]	1.3 [0.9; 1.8]	0.8 [0.5; 1.1]	0.6 [0.2; 1.5]
Eastern Africa	Subregion	131 [119; 146]	82 [69; 99]	77 [57; 108]	11.4 [9.6; 13.0]	18.3 [15.3; 21.8]	22.2 [15.5; 30.3]
Middle Africa	Subregion	56 [48; 66]	65 [50; 85]	76 [47; 136]	4.9 [3.9; 5.9]	14.6 [11.4; 18.6]	22.0 [13.8; 35.1]
Northern Africa	Subregion	19 [14; 26]	12 [6; 24]	11 [4; 34]	1.6 [1.2; 2.2]	2.7 [1.4; 5.3]	3.1 [1.1; 9.2]
Southern Africa	Subregion	7 [5; 11]	3 [2; 4]	3 [2; 7]	0.6 [0.4; 0.9]	0.6 [0.4; 0.9]	1.0 [0.5; 2.1]
Western Africa	Subregion	166 [148; 188]	110 [92; 135]	80 [56; 127]	14.4 [11.8; 16.5]	24.7 [20.7; 29.3]	23.0 [15.6; 33.8]
Central Asia	Subregion	0.04 [0.03; 0.08]	0.03 [0.02; 0.04]	0.05 [0.03; 0.11]	0.0 [0.0; 0.0]	0.0 [0.0; 0.0]	0.0 [0.0; 0.0]
Eastern Asia	Subregion	7 [1; 183]	0.4 [0.1; 25.5]	0.3 [0.1; 23]	0.6 [0.1; 13.5]	0.1 [0.0; 5.3]	0.1 [0.0; 6.0]
Southern Asia	Subregion	592 [500; 704]	73 [54; 100]	35 [17; 104]	51.3 [44.1; 56.2]	16.4 [12.3; 21.4]	10.2 [4.9; 25.4]
South-Eastern Asia	Subregion	55 [44; 68]	13 [9; 19]	9 [5; 20]	4.7 [3.6; 5.9]	2.9 [2.0; 4.4]	2.7 [1.4; 5.6]
Western Asia	Subregion	25 [19; 42]	8 [5; 14]	6 [2; 16]	2.1 [1.6; 3.6]	1.7 [1.0; 3.1]	1.7 [0.7; 4.6]
Eastern Europe	Subregion	2 [2; 3]	2 [2; 3]	2 [1; 3]	0.2 [0.2; 0.2]	0.4 [0.3; 0.6]	0.5 [0.3; 0.9]
Northern Europe	Subregion	0.4 [0.3; 0.5]	0.2 [0.1; 0.3]	0.07 [0.03; 0.2]	0.0 [0.0; 0.0]	0.0 [0.0; 0.1]	0.0 [0.0; 0.1]
Southern Europe	Subregion	0.8 [0.5; 2.3]	0.4 [0.3; 0.5]	0.3 [0.2; 0.6]	0.1 [0.0; 0.2]	0.1 [0.1; 0.1]	0.1 [0.0; 0.2]
Western Europe	Subregion	0.4 [0.3; 0.6]	0.5 [0.3; 0.7]	0.2 [0.1; 0.6]	0.0 [0.0; 0.1]	0.1 [0.1; 0.2]	0.1 [0.0; 0.2]
Caribbean	Subregion	7 [6; 9]	4 [3; 6]	2 [1; 7]	0.6 [0.5; 0.8]	0.8 [0.6; 1.3]	0.7 [0.3; 2.0]
Central America	Subregion	19 [13; 33]	17 [13; 22]	12 [7; 21]	1.6 [1.1; 2.9]	3.7 [2.9; 5.0]	3.3 [1.9; 6.2]
South America	Subregion	49 [39; 67]	52 [43; 65]	30 [21; 47]	4.3 [3.2; 5.7]	11.7 [9.4; 14.6]	8.6 [5.5; 13.5]
Australia/New Zealand	Subregion	0.5 [0.3; 0.7]	0.1 [0.1; 0.2]	0.05 [0.02; 0.15]	0.0 [0.0; 0.1]	0.0 [0.0; 0.0]	0.0 [0.0; 0.0]
Melanesia	Subregion	2 [1; 3]	1 [1; 2]	0.9 [0.3; 3.1]	0.1 [0.1; 0.2]	0.2 [0.1; 0.5]	0.3 [0.1; 0.9]
Micronesia	Subregion	0.03 [0.02; 0.04]	0.02 [0.01; 0.04]	0.02 [0.01; 0.05]	0.0 [0.0; 0.0]	0.0 [0.0; 0.0]	0.0 [0.0; 0.0]
Polynesia	Subregion	0.03 [0.02; 0.11]	0.01 [0.01; 0.03]	0.01 [0; 0.04]	0.0 [0.0; 0.0]	0.0 [0.0; 0.0]	0.0 [0.0; 0.0]
Afghanistan	Country	9 [6; 12]	3 [1; 5]	3 [1; 10]	0.8 [0.5; 1.1]	0.6 [0.3; 1.1]	0.8 [0.3; 2.7]
Albania	Country	0.006 [0.001; 0.045]	0.02 [0.01; 0.04]	0.02 [0; 0.05]	0.0 [0.0; 0.0]	0.0 [0.0; 0.0]	0.0 [0.0; 0.0]
Algeria	Country	0.4 [0.2; 0.7]	0.02 [0.01; 0.1]	0.04 [0; 0.39]	0.0 [0.0; 0.1]	0.0 [0.0; 0.0]	0.0 [0.0; 0.1]
Angola	Country	9 [6; 13]	18 [12; 30]	26 [11; 60]	0.8 [0.5; 1.2]	4.1 [2.6; 6.5]	7.4 [3.1; 16.3]
Antigua and Barbuda	Country	0.007 [0.003; 0.018]	0.001 [0.001; 0.004]	0.001 [0; 0.004]	0.0 [0.0; 0.0]	0.0 [0.0; 0.0]	0.0 [0.0; 0.0]
Argentina	Country	3 [2; 5]	3 [2; 4]	1 [0; 2]	0.3 [0.2; 0.4]	0.7 [0.4; 0.9]	0.3 [0.1; 0.7]
Armenia	Country	0.002 [0.001; 0.004]	0 [0; 0]	0 [0; 0]	0.0 [0.0; 0.0]	0.0 [0.0; 0.0]	0.0 [0.0; 0.0]
Aruba	Country	0.001 [0; 0.003]	0.001 [0.001; 0.002]	0 [0; 0]	0.0 [0.0; 0.0]	0.0 [0.0; 0.0]	0.0 [0.0; 0.0]
Australia	Country	0.4 [0.2; 0.6]	0.07 [0.04; 0.13]	0.03 [0.01; 0.14]	0.0 [0.0; 0.1]	0.0 [0.0; 0.0]	0.0 [0.0; 0.0]
Austria	Country	0.03 [0.02; 0.04]	0.02 [0.01; 0.03]	0.003 [0.001; 0.015]	0.0 [0.0; 0.0]	0.0 [0.0; 0.0]	0.0 [0.0; 0.0]
Azerbaijan	Country	0.004 [0.001; 0.012]	0.01 [0; 0.03]	0.02 [0; 0.13]	0.0 [0.0; 0.0]	0.0 [0.0; 0.0]	0.0 [0.0; 0.0]
Bahamas	Country	0.009 [0.002; 0.064]	0.004 [0.001; 0.011]	0.001 [0; 0.014]	0.0 [0.0; 0.0]	0.0 [0.0; 0.0]	0.0 [0.0; 0.0]
Bahrain	Country	0.009 [0.005; 0.015]	0.003 [0.001; 0.021]	0.003 [0; 0.039]	0.0 [0.0; 0.0]	0.0 [0.0; 0.0]	0.0 [0.0; 0.0]

Continued on next page

Table 15 – continued from previous page

Location Name	Location Type	Age-specific births 10–14 Number (in thousands) 1990	2015	2023	Proportion (in %) 1990	2015	2023
Bangladesh	Country	130 [110; 153]	39 [27; 57]	12 [4; 31]	11.2 [9.0; 13.3]	8.8 [6.0; 12.3]	3.4 [1.2; 8.8]
Barbados	Country	0.009 [0.006; 0.015]	0.004 [0.001; 0.019]	0.005 [0.001; 0.031]	0.0 [0.0; 0.0]	0.0 [0.0; 0.0]	0.0 [0.0; 0.0]
Belarus	Country	0.01 [0.01; 0.02]	0.01 [0.01; 0.02]	0.006 [0.001; 0.033]	0.0 [0.0; 0.0]	0.0 [0.0; 0.0]	0.0 [0.0; 0.0]
Belgium	Country	0.02 [0.01; 0.04]	0.02 [0.01; 0.04]	0.01 [0; 0.09]	0.0 [0.0; 0.0]	0.0 [0.0; 0.0]	0.0 [0.0; 0.0]
Belize	Country	0.02 [0.02; 0.03]	0.02 [0.01; 0.03]	0.02 [0.01; 0.04]	0.0 [0.0; 0.0]	0.0 [0.0; 0.0]	0.0 [0.0; 0.0]
Benin	Country	4 [3; 5]	2 [1; 3]	1 [1; 4]	0.3 [0.2; 0.4]	0.4 [0.3; 0.6]	0.4 [0.2; 1.2]
Bhutan	Country	0.06 [0.01; 0.53]	0.005 [0; 0.129]	0.001 [0; 0.035]	0.0 [0.0; 0.0]	0.0 [0.0; 0.0]	0.0 [0.0; 0.0]
Bolivia (Plurinational State of)	Country	1 [1; 2]	0.9 [0.3; 3.2]	0.8 [0.1; 4.2]	0.1 [0.1; 0.2]	0.2 [0.1; 0.7]	0.2 [0.0; 1.2]
Bosnia and Herzegovina	Country	0.02 [0.01; 0.04]	0.004 [0.001; 0.013]	0.003 [0; 0.016]	0.0 [0.0; 0.0]	0.0 [0.0; 0.0]	0.0 [0.0; 0.0]
Botswana	Country	0.3 [0.1; 1.2]	0.06 [0.04; 0.09]	0.06 [0.02; 0.17]	0.0 [0.0; 0.1]	0.0 [0.0; 0.0]	0.0 [0.0; 0.0]
Brazil	Country	23 [14; 41]	26 [18; 38]	13 [7; 27]	2.0 [1.1; 3.5]	5.8 [4.0; 8.3]	3.9 [1.9; 7.7]
Brunei Darussalam	Country	0.005 [0.003; 0.009]	0.004 [0.002; 0.008]	0.004 [0.001; 0.014]	0.0 [0.0; 0.0]	0.0 [0.0; 0.0]	0.0 [0.0; 0.0]
Bulgaria	Country	0.7 [0.5; 0.9]	0.4 [0.3; 0.5]	0.3 [0.1; 0.6]	0.1 [0.0; 0.1]	0.1 [0.1; 0.1]	0.1 [0.0; 0.2]
Burkina Faso	Country	4 [3; 5]	5 [3; 7]	2 [1; 5]	0.4 [0.3; 0.5]	1.1 [0.7; 1.6]	0.7 [0.3; 1.4]
Burundi	Country	1 [1; 2]	0.5 [0.2; 1.1]	0.5 [0.1; 2.4]	0.1 [0.1; 0.1]	0.1 [0.1; 0.2]	0.2 [0.0; 0.7]
Cabo Verde	Country	0.06 [0.01; 0.23]	0.02 [0.01; 0.03]	0.009 [0.003; 0.026]	0.0 [0.0; 0.0]	0.0 [0.0; 0.0]	0.0 [0.0; 0.0]
Cambodia	Country	0.4 [0.3; 0.6]	0.3 [0.1; 0.6]	0.3 [0.1; 1]	0.0 [0.0; 0.1]	0.1 [0.0; 0.1]	0.1 [0.0; 0.3]
Cameroon	Country	10 [9; 13]	7 [5; 11]	8 [3; 19]	0.9 [0.7; 1.1]	1.6 [1.1; 2.5]	2.2 [0.9; 5.5]
Canada	Country	0.3 [0.2; 0.5]	0.08 [0.05; 0.13]	0.04 [0.01; 0.18]	0.0 [0.0; 0.0]	0.0 [0.0; 0.0]	0.0 [0.0; 0.1]
Central African Republic	Country	2 [1; 2]	3 [2; 5]	4 [2; 8]	0.1 [0.1; 0.2]	0.7 [0.5; 1.1]	1.0 [0.5; 2.2]
Chad	Country	8 [6; 10]	8 [6; 12]	5 [2; 11]	0.7 [0.5; 0.8]	1.9 [1.3; 2.7]	1.5 [0.6; 3.2]
Chile	Country	0.8 [0.6; 1.1]	0.7 [0.5; 1.1]	0.04 [0.02; 0.08]	0.1 [0.0; 0.1]	0.2 [0.1; 0.3]	0.0 [0.0; 0.0]
China	Country	6 [0; 191]	0.3 [0; 25.6]	0.2 [0; 28.4]	0.5 [0.0; 14.1]	0.1 [0.0; 5.3]	0.1 [0.0; 7.4]
China, Hong Kong SAR	Country	0.01 [0.01; 0.03]	0.005 [0.002; 0.011]	0.001 [0; 0.008]	0.0 [0.0; 0.0]	0.0 [0.0; 0.0]	0.0 [0.0; 0.0]
Macao, SAR of China	Country	0.001 [0; 0.003]	0 [0; 0]	0 [0; 0]	0.0 [0.0; 0.0]	0.0 [0.0; 0.0]	0.0 [0.0; 0.0]
China, Taiwan Province of China	Country	0.5 [0.3; 0.8]	0.05 [0.02; 0.11]	0.02 [0; 0.18]	0.0 [0.0; 0.1]	0.0 [0.0; 0.0]	0.0 [0.0; 0.1]
Colombia	Country	8 [5; 11]	8 [5; 11]	6 [3; 12]	0.7 [0.4; 1.0]	1.7 [1.2; 2.4]	1.7 [0.8; 3.4]
Comoros	Country	0.3 [0.2; 0.4]	0.08 [0.03; 0.24]	0.06 [0.01; 0.33]	0.0 [0.0; 0.0]	0.0 [0.0; 0.1]	0.0 [0.0; 0.1]
Republic of the Congo	Country	2 [1; 2]	2 [1; 3]	2 [1; 6]	0.1 [0.1; 0.2]	0.4 [0.2; 0.8]	0.6 [0.2; 1.8]
Costa Rica	Country	0.4 [0.3; 0.6]	0.4 [0.3; 0.6]	0.2 [0.1; 0.3]	0.0 [0.0; 0.1]	0.1 [0.1; 0.1]	0.0 [0.0; 0.1]
Côte d'Ivoire	Country	11 [9; 14]	8 [5; 11]	6 [3; 12]	1.0 [0.7; 1.3]	1.7 [1.1; 2.6]	1.6 [0.7; 3.5]
Croatia	Country	0.02 [0.01; 0.03]	0.006 [0.004; 0.012]	0.003 [0.001; 0.007]	0.0 [0.0; 0.0]	0.0 [0.0; 0.0]	0.0 [0.0; 0.0]
Cuba	Country	2 [1; 2]	0.4 [0.3; 0.6]	0.4 [0.2; 1]	0.1 [0.1; 0.2]	0.1 [0.1; 0.1]	0.1 [0.0; 0.3]
Curaçao	Country	0.005 [0.001; 0.038]	0.003 [0.002; 0.005]	0.001 [0; 0.006]	0.0 [0.0; 0.0]	0.0 [0.0; 0.0]	0.0 [0.0; 0.0]
Cyprus	Country	0.02 [0; 0.16]	0.002 [0.001; 0.006]	0.004 [0.001; 0.022]	0.0 [0.0; 0.0]	0.0 [0.0; 0.0]	0.0 [0.0; 0.0]
Czechia	Country	0.02 [0.02; 0.03]	0.01 [0.01; 0.02]	0.02 [0.01; 0.08]	0.0 [0.0; 0.0]	0.0 [0.0; 0.0]	0.0 [0.0; 0.0]
Dem. People's Republic of Korea	Country	0.001 [0; 0.163]	0 [0; 0]	0 [0; 0]	0.0 [0.0; 0.0]	0.0 [0.0; 0.0]	0.0 [0.0; 0.0]
Democratic Republic of the Congo	Country	25 [18; 34]	24 [13; 42]	30 [11; 87]	2.1 [1.5; 3.0]	5.4 [3.0; 9.2]	8.7 [3.1; 22.6]
Denmark	Country	0.003 [0.002; 0.005]	0.003 [0.001; 0.005]	0.001 [0; 0.006]	0.0 [0.0; 0.0]	0.0 [0.0; 0.0]	0.0 [0.0; 0.0]
Djibouti	Country	0.1 [0.1; 0.2]	0.01 [0; 0.07]	0.009 [0.001; 0.105]	0.0 [0.0; 0.0]	0.0 [0.0; 0.0]	0.0 [0.0; 0.0]
Dominican Republic	Country	3 [2; 4]	1 [1; 2]	0.4 [0.2; 0.9]	0.3 [0.2; 0.4]	0.3 [0.2; 0.4]	0.1 [0.1; 0.3]
Ecuador	Country	3 [2; 4]	3 [2; 5]	2 [1; 5]	0.2 [0.1; 0.3]	0.8 [0.5; 1.0]	0.6 [0.3; 1.4]
Egypt	Country	8 [6; 11]	4 [1; 11]	4 [1; 21]	0.7 [0.5; 1.0]	0.8 [0.3; 2.5]	1.1 [0.2; 5.8]
El Salvador	Country	1 [1; 1]	0.9 [0.6; 1.3]	0.5 [0.1; 1.4]	0.1 [0.1; 0.1]	0.2 [0.1; 0.3]	0.1 [0.0; 0.4]
Equatorial Guinea	Country	0.4 [0.1; 2]	1 [0; 7]	2 [0; 9]	0.0 [0.0; 0.2]	0.3 [0.1; 1.4]	0.5 [0.1; 2.5]
Eritrea	Country	0.9 [0.6; 1.3]	0.3 [0.1; 1]	0.3 [0.1; 1.5]	0.1 [0.0; 0.1]	0.1 [0.0; 0.2]	0.1 [0.0; 0.4]
Estonia	Country	0.004 [0.003; 0.006]	0.002 [0.001; 0.004]	0.001 [0; 0.005]	0.0 [0.0; 0.0]	0.0 [0.0; 0.0]	0.0 [0.0; 0.0]

Continued on next page

Table 15 – continued from previous page

Location Name	Location Type	Age-specific births 10–14 Number (in thousands) 1990	2015	2023	Proportion (in %) 1990	2015	2023
Eswatini	Country	0.5 [0.4; 0.7]	0.08 [0.03; 0.21]	0.06 [0.02; 0.28]	0.0 [0.0; 0.1]	0.0 [0.0; 0.0]	0.0 [0.0; 0.1]
Ethiopia	Country	46 [36; 58]	13 [7; 22]	5 [2; 19]	3.9 [3.0; 5.0]	2.8 [1.6; 4.9]	1.5 [0.4; 5.4]
Fiji	Country	0.02 [0.01; 0.05]	0.001 [0; 0.003]	0 [0; 0]	0.0 [0.0; 0.0]	0.0 [0.0; 0.0]	0.0 [0.0; 0.0]
Finland	Country	0.004 [0.002; 0.007]	0.003 [0.002; 0.006]	0.001 [0; 0.006]	0.0 [0.0; 0.0]	0.0 [0.0; 0.0]	0.0 [0.0; 0.0]
France	Country	0.1 [0.1; 0.2]	0.2 [0.1; 0.4]	0.1 [0; 0.5]	0.0 [0.0; 0.0]	0.0 [0.0; 0.1]	0.0 [0.0; 0.1]
French Guiana	Country	0.02 [0.01; 0.05]	0.04 [0.02; 0.05]	0.02 [0.01; 0.05]	0.0 [0.0; 0.0]	0.0 [0.0; 0.0]	0.0 [0.0; 0.0]
French Polynesia	Country	0.02 [0; 0.11]	0.004 [0.001; 0.01]	0.001 [0; 0.003]	0.0 [0.0; 0.0]	0.0 [0.0; 0.0]	0.0 [0.0; 0.0]
Gabon	Country	0.8 [0.6; 1.1]	0.5 [0.3; 0.8]	0.5 [0.2; 1.2]	0.1 [0.1; 0.1]	0.1 [0.1; 0.2]	0.1 [0.0; 0.4]
Gambia	Country	1 [1; 1]	0.3 [0.2; 0.5]	0.2 [0; 0.5]	0.1 [0.1; 0.1]	0.1 [0.0; 0.1]	0.0 [0.0; 0.1]
Georgia	Country	0.05 [0.01; 0.25]	0.02 [0.01; 0.03]	0.007 [0.002; 0.022]	0.0 [0.0; 0.0]	0.0 [0.0; 0.0]	0.0 [0.0; 0.0]
Germany	Country	0.2 [0.1; 0.4]	0.2 [0.1; 0.4]	0.1 [0; 0.4]	0.0 [0.0; 0.0]	0.0 [0.0; 0.1]	0.0 [0.0; 0.1]
Ghana	Country	7 [5; 9]	3 [2; 5]	3 [1; 9]	0.6 [0.4; 0.8]	0.6 [0.4; 1.1]	0.9 [0.3; 2.5]
Greece	Country	0.09 [0.06; 0.13]	0.08 [0.04; 0.15]	0.1 [0; 0.4]	0.0 [0.0; 0.0]	0.0 [0.0; 0.0]	0.0 [0.0; 0.1]
Grenada	Country	0.03 [0.02; 0.06]	0.003 [0.002; 0.005]	0.003 [0.001; 0.009]	0.0 [0.0; 0.0]	0.0 [0.0; 0.0]	0.0 [0.0; 0.0]
Guadeloupe	Country	0.01 [0.01; 0.03]	0.003 [0.001; 0.015]	0 [0; 0]	0.0 [0.0; 0.0]	0.0 [0.0; 0.0]	0.0 [0.0; 0.0]
Guam	Country	0.004 [0.002; 0.01]	0.001 [0.001; 0.002]	0.001 [0; 0.004]	0.0 [0.0; 0.0]	0.0 [0.0; 0.0]	0.0 [0.0; 0.0]
Guatemala	Country	3 [2; 4]	4 [3; 5]	3 [1; 6]	0.3 [0.2; 0.4]	0.9 [0.6; 1.2]	0.9 [0.4; 1.8]
Guinea	Country	7 [6; 9]	6 [4; 8]	4 [2; 10]	0.6 [0.5; 0.8]	1.3 [0.9; 1.8]	1.3 [0.5; 3.0]
Guinea-Bissau	Country	0.8 [0.5; 1.2]	0.4 [0.2; 0.7]	0.3 [0.1; 0.9]	0.1 [0.0; 0.1]	0.1 [0.0; 0.2]	0.1 [0.0; 0.3]
Guyana	Country	0.1 [0.1; 0.2]	0.04 [0.02; 0.09]	0.03 [0.01; 0.12]	0.0 [0.0; 0.0]	0.0 [0.0; 0.0]	0.0 [0.0; 0.0]
Haiti	Country	2 [1; 2]	2 [1; 4]	1 [0; 6]	0.1 [0.1; 0.2]	0.4 [0.2; 0.8]	0.4 [0.1; 1.8]
Honduras	Country	2 [1; 3]	1 [0; 6]	0.9 [0.2; 5.8]	0.2 [0.1; 0.2]	0.3 [0.1; 1.2]	0.3 [0.0; 1.7]
Hungary	Country	0.3 [0.2; 0.4]	0.1 [0.1; 0.2]	0.07 [0.02; 0.2]	0.0 [0.0; 0.0]	0.0 [0.0; 0.0]	0.0 [0.0; 0.1]
Iceland	Country	0.002 [0.001; 0.004]	0.001 [0.001; 0.003]	0.001 [0; 0.004]	0.0 [0.0; 0.0]	0.0 [0.0; 0.0]	0.0 [0.0; 0.0]
India	Country	414 [327; 528]	20 [9; 45]	12 [2; 95]	35.9 [29.2; 42.0]	4.6 [2.2; 9.5]	3.6 [0.5; 23.2]
Indonesia	Country	44 [34; 57]	4 [2; 9]	2 [0; 12]	3.8 [2.8; 5.0]	0.9 [0.4; 2.0]	0.6 [0.1; 3.4]
Iran (Islamic Republic of)	Country	5 [3; 9]	2 [1; 3]	1 [0; 4]	0.4 [0.2; 0.7]	0.4 [0.2; 0.6]	0.4 [0.1; 1.1]
Iraq	Country	3 [2; 5]	5 [2; 10]	3 [1; 12]	0.2 [0.1; 0.4]	1.1 [0.5; 2.2]	0.9 [0.2; 3.7]
Ireland	Country	0.01 [0.01; 0.02]	0.005 [0.003; 0.008]	0.002 [0; 0.007]	0.0 [0.0; 0.0]	0.0 [0.0; 0.0]	0.0 [0.0; 0.0]
Israel	Country	0.02 [0.01; 0.03]	0.001 [0.001; 0.003]	0 [0; 0]	0.0 [0.0; 0.0]	0.0 [0.0; 0.0]	0.0 [0.0; 0.0]
Italy	Country	0.02 [0.01; 0.03]	0.02 [0.01; 0.03]	0.007 [0.001; 0.034]	0.0 [0.0; 0.0]	0.0 [0.0; 0.0]	0.0 [0.0; 0.0]
Jamaica	Country	0.4 [0.2; 0.6]	0.1 [0; 0.4]	0.05 [0.01; 0.39]	0.0 [0.0; 0.1]	0.0 [0.0; 0.1]	0.0 [0.0; 0.1]
Japan	Country	0.04 [0.02; 0.08]	0.06 [0.03; 0.1]	0.04 [0.01; 0.2]	0.0 [0.0; 0.0]	0.0 [0.0; 0.0]	0.0 [0.0; 0.1]
Jersey	Country	0 [0; 0]	0 [0; 0]	0 [0; 0]	0.0 [0.0; 0.0]	0.0 [0.0; 0.0]	0.0 [0.0; 0.0]
Jordan	Country	0.07 [0.04; 0.1]	0.07 [0.03; 0.18]	0.04 [0.01; 0.3]	0.0 [0.0; 0.0]	0.0 [0.0; 0.0]	0.0 [0.0; 0.1]
Kazakhstan	Country	0.01 [0.01; 0.03]	0.02 [0.01; 0.03]	0.02 [0; 0.08]	0.0 [0.0; 0.0]	0.0 [0.0; 0.0]	0.0 [0.0; 0.0]
Kenya	Country	11 [9; 14]	6 [4; 9]	3 [1; 5]	0.9 [0.7; 1.2]	1.3 [0.8; 2.0]	0.8 [0.4; 1.6]
Kiribati	Country	0.008 [0.004; 0.016]	0.007 [0.003; 0.021]	0.01 [0; 0.05]	0.0 [0.0; 0.0]	0.0 [0.0; 0.0]	0.0 [0.0; 0.0]
Kosovo (under UNSC res. 1244)	Country	0.004 [0.002; 0.006]	0.002 [0.001; 0.004]	0.002 [0; 0.01]	0.0 [0.0; 0.0]	0.0 [0.0; 0.0]	0.0 [0.0; 0.0]
Kuwait	Country	0 [0; 0]	0 [0; 0]	0 [0; 0]	0.0 [0.0; 0.0]	0.0 [0.0; 0.0]	0.0 [0.0; 0.0]
Kyrgyz Republic	Country	0.004 [0.002; 0.01]	0.002 [0.001; 0.003]	0.004 [0.001; 0.013]	0.0 [0.0; 0.0]	0.0 [0.0; 0.0]	0.0 [0.0; 0.0]
Lao People's Democratic Republic	Country	2 [1; 4]	1 [1; 2]	1 [0; 4]	0.2 [0.1; 0.3]	0.3 [0.1; 0.5]	0.3 [0.1; 1.1]
Latvia	Country	0.008 [0.003; 0.022]	0.005 [0.003; 0.009]	0.002 [0.001; 0.008]	0.0 [0.0; 0.0]	0.0 [0.0; 0.0]	0.0 [0.0; 0.0]
Lebanon	Country	0.07 [0.03; 0.15]	0.001 [0; 0.011]	0.001 [0; 0.025]	0.0 [0.0; 0.0]	0.0 [0.0; 0.0]	0.0 [0.0; 0.0]
Lesotho	Country	0.2 [0.1; 0.3]	0.09 [0.04; 0.2]	0.09 [0.03; 0.34]	0.0 [0.0; 0.0]	0.0 [0.0; 0.0]	0.0 [0.0; 0.1]
Liberia	Country	3 [2; 3]	2 [1; 2]	2 [1; 3]	0.2 [0.2; 0.3]	0.3 [0.2; 0.5]	0.4 [0.2; 1.0]
Libya	Country	0.007 [0.003; 0.025]	0 [0; 0]	0 [0; 0]	0.0 [0.0; 0.0]	0.0 [0.0; 0.0]	0.0 [0.0; 0.0]

Continued on next page

Table 15 – continued from previous page

Location Name	Location Type	Age-specific births 10–14 Number (in thousands) 1990	2015	2023	Proportion (in %) 1990	2015	2023
Lithuania	Country	0.02 [0.01; 0.03]	0.009 [0.006; 0.016]	0.002 [0.001; 0.007]	0.0 [0.0; 0.0]	0.0 [0.0; 0.0]	0.0 [0.0; 0.0]
Luxembourg	Country	0.001 [0; 0.001]	0.002 [0.001; 0.003]	0.001 [0; 0.009]	0.0 [0.0; 0.0]	0.0 [0.0; 0.0]	0.0 [0.0; 0.0]
Madagascar	Country	8 [6; 10]	18 [13; 25]	18 [9; 36]	0.7 [0.5; 0.9]	4.1 [2.8; 5.7]	5.1 [2.5; 10.3]
Malawi	Country	6 [5; 7]	3 [2; 4]	3 [1; 7]	0.5 [0.4; 0.6]	0.7 [0.4; 1.0]	0.8 [0.3; 1.9]
Malaysia	Country	0.3 [0; 2.1]	0.2 [0.1; 0.4]	0.1 [0; 0.4]	0.0 [0.0; 0.2]	0.0 [0.0; 0.1]	0.0 [0.0; 0.1]
Maldives	Country	0.05 [0.04; 0.08]	0 [0; 0]	0 [0; 0]	0.0 [0.0; 0.0]	0.0 [0.0; 0.0]	0.0 [0.0; 0.0]
Mali	Country	10 [8; 12]	11 [8; 15]	10 [5; 21]	0.8 [0.7; 1.0]	2.4 [1.7; 3.4]	2.9 [1.4; 6.0]
Malta	Country	0.005 [0.001; 0.029]	0.003 [0.001; 0.005]	0.001 [0; 0.004]	0.0 [0.0; 0.0]	0.0 [0.0; 0.0]	0.0 [0.0; 0.0]
Martinique	Country	0.006 [0.004; 0.012]	0.001 [0; 0.006]	0.001 [0; 0.003]	0.0 [0.0; 0.0]	0.0 [0.0; 0.0]	0.0 [0.0; 0.0]
Mauritania	Country	2 [2; 3]	3 [2; 5]	4 [2; 11]	0.2 [0.1; 0.3]	0.7 [0.4; 1.1]	1.2 [0.5; 3.0]
Mauritius	Country	0.03 [0.02; 0.05]	0.03 [0.02; 0.04]	0.02 [0.01; 0.05]	0.0 [0.0; 0.0]	0.0 [0.0; 0.0]	0.0 [0.0; 0.0]
Mayotte	Country	0.05 [0.01; 0.33]	0.04 [0; 0.5]	0.04 [0; 0.63]	0.0 [0.0; 0.0]	0.0 [0.0; 0.1]	0.0 [0.0; 0.2]
Mexico	Country	10 [4; 24]	8 [5; 11]	5 [2; 14]	0.8 [0.3; 2.1]	1.7 [1.2; 2.5]	1.4 [0.4; 4.0]
Micronesia (Fed. States of)	Country	0.005 [0.002; 0.017]	0.006 [0.001; 0.028]	0.005 [0.001; 0.037]	0.0 [0.0; 0.0]	0.0 [0.0; 0.0]	0.0 [0.0; 0.0]
Mongolia	Country	0.04 [0.02; 0.09]	0.001 [0.001; 0.004]	0.02 [0.01; 0.04]	0.0 [0.0; 0.0]	0.0 [0.0; 0.0]	0.0 [0.0; 0.0]
Montenegro	Country	0.004 [0.001; 0.012]	0.003 [0.002; 0.005]	0.002 [0.001; 0.007]	0.0 [0.0; 0.0]	0.0 [0.0; 0.0]	0.0 [0.0; 0.0]
Morocco	Country	1 [1; 2]	0.2 [0; 1.2]	0.2 [0; 2.2]	0.1 [0.1; 0.2]	0.0 [0.0; 0.3]	0.1 [0.0; 0.6]
Mozambique	Country	15 [12; 20]	14 [9; 21]	17 [9; 34]	1.3 [1.0; 1.8]	3.1 [2.0; 4.8]	5.0 [2.4; 9.7]
Myanmar	Country	3 [2; 7]	1 [0; 5]	1 [0; 9]	0.3 [0.1; 0.6]	0.3 [0.1; 1.1]	0.4 [0.1; 2.5]
Namibia	Country	0.3 [0.2; 0.4]	0.3 [0.1; 0.8]	0.4 [0.1; 1.6]	0.0 [0.0; 0.0]	0.1 [0.0; 0.2]	0.1 [0.0; 0.5]
Nepal	Country	3 [2; 5]	2 [1; 3]	0.6 [0.2; 1.8]	0.3 [0.2; 0.4]	0.5 [0.3; 0.7]	0.2 [0.1; 0.5]
Netherlands	Country	0.02 [0.01; 0.05]	0.01 [0.01; 0.03]	0.003 [0; 0.029]	0.0 [0.0; 0.0]	0.0 [0.0; 0.0]	0.0 [0.0; 0.0]
New Caledonia	Country	0.004 [0.002; 0.011]	0 [0; 0]	0 [0; 0]	0.0 [0.0; 0.0]	0.0 [0.0; 0.0]	0.0 [0.0; 0.0]
New Zealand	Country	0.1 [0.1; 0.2]	0.02 [0.01; 0.04]	0.01 [0; 0.04]	0.0 [0.0; 0.0]	0.0 [0.0; 0.0]	0.0 [0.0; 0.0]
Nicaragua	Country	2 [2; 3]	2 [1; 4]	2 [1; 6]	0.2 [0.1; 0.3]	0.4 [0.2; 1.0]	0.5 [0.1; 1.7]
Niger	Country	7 [6; 9]	9 [5; 15]	6 [3; 13]	0.6 [0.5; 0.8]	2.0 [1.2; 3.4]	1.8 [0.8; 3.7]
Nigeria	Country	97 [80; 119]	56 [39; 81]	36 [16; 85]	8.4 [6.5; 10.3]	12.6 [9.0; 17.4]	10.3 [4.6; 22.4]
North Macedonia	Country	0.04 [0.02; 0.06]	0.02 [0.01; 0.04]	0.02 [0.01; 0.05]	0.0 [0.0; 0.0]	0.0 [0.0; 0.0]	0.0 [0.0; 0.0]
Norway	Country	0.004 [0.003; 0.007]	0.002 [0.001; 0.004]	0.001 [0; 0.004]	0.0 [0.0; 0.0]	0.0 [0.0; 0.0]	0.0 [0.0; 0.0]
Oman	Country	2 [1; 6]	0.004 [0.001; 0.017]	0.006 [0.002; 0.026]	0.2 [0.1; 0.5]	0.0 [0.0; 0.0]	0.0 [0.0; 0.0]
Pakistan	Country	31 [23; 42]	7 [3; 14]	6 [1; 29]	2.7 [1.9; 3.7]	1.6 [0.8; 3.1]	1.8 [0.4; 8.0]
Panama	Country	0.4 [0.3; 0.6]	0.6 [0.4; 0.8]	0.3 [0.2; 0.7]	0.0 [0.0; 0.1]	0.1 [0.1; 0.2]	0.1 [0.0; 0.2]
Papua New Guinea	Country	1 [1; 2]	1 [1; 2]	0.9 [0.2; 3.1]	0.1 [0.1; 0.2]	0.2 [0.1; 0.5]	0.2 [0.1; 0.9]
Paraguay	Country	0.6 [0.4; 1]	0.7 [0.3; 1.8]	0.8 [0.2; 3.3]	0.1 [0.0; 0.1]	0.2 [0.1; 0.4]	0.2 [0.1; 0.9]
Peru	Country	5 [3; 8]	3 [1; 10]	3 [0; 15]	0.4 [0.2; 0.7]	0.8 [0.3; 2.2]	0.8 [0.1; 4.3]
Philippines	Country	1 [1; 1]	2 [2; 3]	2 [1; 5]	0.1 [0.1; 0.1]	0.5 [0.3; 0.8]	0.6 [0.2; 1.4]
Poland	Country	0.1 [0.1; 0.2]	0.07 [0.04; 0.11]	0.03 [0.01; 0.11]	0.0 [0.0; 0.0]	0.0 [0.0; 0.0]	0.0 [0.0; 0.0]
Portugal	Country	0.1 [0.1; 0.2]	0.06 [0.04; 0.1]	0.02 [0.01; 0.09]	0.0 [0.0; 0.0]	0.0 [0.0; 0.0]	0.0 [0.0; 0.0]
Puerto Rico	Country	0.3 [0.2; 0.5]	0.04 [0.03; 0.07]	0.005 [0.002; 0.014]	0.0 [0.0; 0.0]	0.0 [0.0; 0.0]	0.0 [0.0; 0.0]
Qatar	Country	0.005 [0.003; 0.007]	0.001 [0; 0.009]	0 [0; 0]	0.0 [0.0; 0.0]	0.0 [0.0; 0.0]	0.0 [0.0; 0.0]
Republic of Korea	Country	0.008 [0.004; 0.015]	0.02 [0.01; 0.05]	0.006 [0.001; 0.048]	0.0 [0.0; 0.0]	0.0 [0.0; 0.0]	0.0 [0.0; 0.0]
Republic of Moldova	Country	0.03 [0.02; 0.06]	0 [0; 0]	0 [0; 0]	0.0 [0.0; 0.0]	0.0 [0.0; 0.0]	0.0 [0.0; 0.0]
Réunion	Country	0.01 [0.01; 0.03]	0.01 [0; 0.04]	0.003 [0.001; 0.011]	0.0 [0.0; 0.0]	0.0 [0.0; 0.0]	0.0 [0.0; 0.0]
Romania	Country	0.6 [0.4; 0.8]	0.7 [0.4; 1.1]	0.8 [0.3; 2]	0.0 [0.0; 0.1]	0.2 [0.1; 0.3]	0.2 [0.1; 0.6]
Russian Federation	Country	0.4 [0.2; 0.6]	0.5 [0.3; 0.8]	0.3 [0.1; 0.9]	0.0 [0.0; 0.0]	0.1 [0.1; 0.2]	0.1 [0.0; 0.3]
Rwanda	Country	0.6 [0.4; 0.9]	0.2 [0.1; 0.4]	0.2 [0; 1]	0.0 [0.0; 0.1]	0.0 [0.0; 0.1]	0.1 [0.0; 0.3]
Saint Lucia	Country	0.02 [0.01; 0.02]	0.005 [0.003; 0.013]	0.004 [0.001; 0.015]	0.0 [0.0; 0.0]	0.0 [0.0; 0.0]	0.0 [0.0; 0.0]

Continued on next page

Table 15 – continued from previous page

		Age-specific births 10–14					
		Number (in thousands)			Proportion (in %)		
**Location Name**	**Location Type**	**1990**	**2015**	**2023**	**1990**	**2015**	**2023**
Saint Vincent and the Grenadines	Country	0.02 [0.01; 0.03]	0.007 [0.005; 0.011]	0.005 [0.002; 0.013]	0.0 [0.0; 0.0]	0.0 [0.0; 0.0]	0.0 [0.0; 0.0]
Samoa	Country	0.008 [0.003; 0.023]	0.005 [0.002; 0.014]	0.008 [0.002; 0.035]	0.0 [0.0; 0.0]	0.0 [0.0; 0.0]	0.0 [0.0; 0.0]
Sao Tome and Principe	Country	0.02 [0.01; 0.03]	0.02 [0.01; 0.04]	0.01 [0; 0.04]	0.0 [0.0; 0.0]	0.0 [0.0; 0.0]	0.0 [0.0; 0.0]
Saudi Arabia	Country	2 [0; 22]	0.04 [0; 2.41]	0.03 [0; 2.18]	0.2 [0.0; 1.8]	0.0 [0.0; 0.5]	0.0 [0.0; 0.6]
Senegal	Country	3 [3; 4]	1 [1; 2]	1 [0; 4]	0.3 [0.2; 0.4]	0.3 [0.2; 0.5]	0.4 [0.1; 1.2]
Serbia	Country	0.3 [0; 1.8]	0.04 [0.03; 0.08]	0.03 [0.01; 0.12]	0.0 [0.0; 0.2]	0.0 [0.0; 0.0]	0.0 [0.0; 0.0]
Seychelles	Country	0.003 [0.002; 0.004]	0.004 [0.002; 0.006]	0.004 [0.001; 0.016]	0.0 [0.0; 0.0]	0.0 [0.0; 0.0]	0.0 [0.0; 0.0]
Sierra Leone	Country	5 [4; 7]	3 [2; 4]	2 [1; 4]	0.5 [0.3; 0.6]	0.6 [0.5; 0.9]	0.5 [0.2; 1.2]
Singapore	Country	0.01 [0.01; 0.02]	0.01 [0; 0.02]	0.004 [0.001; 0.018]	0.0 [0.0; 0.0]	0.0 [0.0; 0.0]	0.0 [0.0; 0.0]
Slovakia	Country	0.05 [0.03; 0.07]	0.05 [0.03; 0.08]	0.06 [0.02; 0.17]	0.0 [0.0; 0.0]	0.0 [0.0; 0.0]	0.0 [0.0; 0.0]
Slovenia	Country	0.004 [0.003; 0.007]	0.002 [0.001; 0.004]	0.003 [0.001; 0.016]	0.0 [0.0; 0.0]	0.0 [0.0; 0.0]	0.0 [0.0; 0.0]
Solomon Islands	Country	0.09 [0.01; 0.78]	0.04 [0; 0.73]	0.04 [0; 0.78]	0.0 [0.0; 0.1]	0.0 [0.0; 0.2]	0.0 [0.0; 0.2]
Somalia	Country	3 [2; 4]	3 [2; 6]	4 [2; 11]	0.2 [0.2; 0.4]	0.8 [0.4; 1.4]	1.2 [0.4; 3.1]
South Africa	Country	6 [4; 9]	2 [2; 3]	3 [1; 6]	0.5 [0.3; 0.8]	0.5 [0.3; 0.8]	0.8 [0.3; 1.9]
South Sudan	Country	6 [3; 10]	8 [3; 19]	9 [2; 28]	0.5 [0.3; 0.9]	1.7 [0.7; 4.3]	2.4 [0.7; 7.9]
Spain	Country	0.2 [0.1; 0.3]	0.1 [0.1; 0.2]	0.07 [0.02; 0.28]	0.0 [0.0; 0.0]	0.0 [0.0; 0.1]	0.0 [0.0; 0.1]
Sri Lanka	Country	0.1 [0.1; 0.2]	0.05 [0.02; 0.1]	0.04 [0; 0.25]	0.0 [0.0; 0.0]	0.0 [0.0; 0.0]	0.0 [0.0; 0.1]
State of Palestine	Country	0.1 [0.1; 0.2]	0 [0; 0]	0.001 [0; 0.003]	0.0 [0.0; 0.0]	0.0 [0.0; 0.0]	0.0 [0.0; 0.0]
Sudan	Country	9 [6; 15]	8 [4; 20]	7 [2; 28]	0.8 [0.5; 1.3]	1.9 [0.8; 4.4]	1.9 [0.5; 7.5]
Suriname	Country	0.08 [0.06; 0.12]	0.07 [0.05; 0.11]	0.05 [0.02; 0.12]	0.0 [0.0; 0.0]	0.0 [0.0; 0.0]	0.0 [0.0; 0.0]
Sweden	Country	0.009 [0.005; 0.015]	0.006 [0.003; 0.012]	0.004 [0.001; 0.015]	0.0 [0.0; 0.0]	0.0 [0.0; 0.0]	0.0 [0.0; 0.0]
Switzerland	Country	0.003 [0.001; 0.005]	0.004 [0.002; 0.007]	0.001 [0; 0.007]	0.0 [0.0; 0.0]	0.0 [0.0; 0.0]	0.0 [0.0; 0.0]
Syrian Arab Republic	Country	2 [1; 4]	0.5 [0.1; 3.4]	0.5 [0; 5.5]	0.2 [0.1; 0.4]	0.1 [0.0; 0.7]	0.1 [0.0; 1.6]
Tajikistan	Country	0.004 [0.001; 0.013]	0.001 [0; 0.004]	0.003 [0.001; 0.016]	0.0 [0.0; 0.0]	0.0 [0.0; 0.0]	0.0 [0.0; 0.0]
Thailand	Country	2 [1; 3]	3 [1; 8]	1 [0; 3]	0.2 [0.1; 0.2]	0.6 [0.2; 1.7]	0.3 [0.1; 0.9]
Timor-Leste	Country	0.07 [0.04; 0.11]	0.03 [0.01; 0.07]	0.01 [0; 0.07]	0.0 [0.0; 0.0]	0.0 [0.0; 0.0]	0.0 [0.0; 0.0]
Togo	Country	2 [2; 3]	1 [1; 2]	1 [0; 5]	0.2 [0.1; 0.3]	0.3 [0.1; 0.5]	0.4 [0.1; 1.4]
Tonga	Country	0.004 [0.001; 0.011]	0.002 [0; 0.015]	0.001 [0; 0.026]	0.0 [0.0; 0.0]	0.0 [0.0; 0.0]	0.0 [0.0; 0.0]
Trinidad and Tobago	Country	0.07 [0.05; 0.1]	0.02 [0.01; 0.1]	0.03 [0; 0.18]	0.0 [0.0; 0.0]	0.0 [0.0; 0.0]	0.0 [0.0; 0.1]
Tunisia	Country	0.01 [0; 0.02]	0 [0; 0]	0.001 [0; 0.007]	0.0 [0.0; 0.0]	0.0 [0.0; 0.0]	0.0 [0.0; 0.0]
Türkiye	Country	6 [4; 9]	0.3 [0.2; 0.5]	0.08 [0.03; 0.28]	0.5 [0.3; 0.8]	0.1 [0.0; 0.1]	0.0 [0.0; 0.1]
Turkmenistan	Country	0.02 [0; 0.05]	0 [0; 0]	0.001 [0; 0.004]	0.0 [0.0; 0.0]	0.0 [0.0; 0.0]	0.0 [0.0; 0.0]
Uganda	Country	17 [14; 21]	6 [4; 9]	5 [2; 14]	1.5 [1.1; 1.8]	1.3 [0.8; 2.0]	1.5 [0.5; 4.1]
Ukraine	Country	0.1 [0.1; 0.2]	0.2 [0.1; 0.3]	0.1 [0; 0.5]	0.0 [0.0; 0.0]	0.0 [0.0; 0.1]	0.0 [0.0; 0.2]
United Arab Emirates	Country	0.04 [0.01; 0.16]	0.001 [0; 0.034]	0 [0; 0]	0.0 [0.0; 0.0]	0.0 [0.0; 0.0]	0.0 [0.0; 0.0]
United Kingdom	Country	0.3 [0.2; 0.5]	0.2 [0.1; 0.2]	0.06 [0.01; 0.19]	0.0 [0.0; 0.0]	0.0 [0.0; 0.1]	0.0 [0.0; 0.1]
United Republic of Tanzania	Country	9 [7; 12]	5 [3; 10]	7 [2; 17]	0.8 [0.6; 1.1]	1.2 [0.7; 2.1]	1.9 [0.7; 5.1]
United States of America	Country	14 [10; 20]	3 [2; 5]	2 [1; 5]	1.2 [0.8; 1.7]	0.7 [0.5; 1.1]	0.5 [0.2; 1.5]
Uruguay	Country	0.2 [0.1; 0.3]	0.1 [0.1; 0.2]	0.04 [0.01; 0.09]	0.0 [0.0; 0.0]	0.0 [0.0; 0.0]	0.0 [0.0; 0.0]
Uzbekistan	Country	0.01 [0; 0.02]	0.007 [0.002; 0.021]	0.02 [0.01; 0.06]	0.0 [0.0; 0.0]	0.0 [0.0; 0.0]	0.0 [0.0; 0.0]
Vanuatu	Country	0.02 [0; 0.21]	0.02 [0; 0.33]	0.04 [0; 0.46]	0.0 [0.0; 0.0]	0.0 [0.0; 0.1]	0.0 [0.0; 0.1]
Venezuela (Bolivarian Republic of)	Country	4 [3; 7]	6 [5; 9]	3 [1; 7]	0.4 [0.2; 0.6]	1.4 [1.0; 2.0]	0.9 [0.3; 2.1]
Viet Nam	Country	1 [0; 0]	0.8 [0.3; 2.1]	1 [0; 6]	0.1 [0.0; 0.2]	0.2 [0.1; 0.5]	0.4 [0.1; 1.6]
Western Sahara	Country	0.1 [0; 0.6]	0.01 [0; 0.19]	0.01 [0; 0.26]	0.0 [0.0; 0.1]	0.0 [0.0; 0.0]	0.0 [0.0; 0.1]
Yemen	Country	9 [7; 11]	2 [1; 4]	2 [0; 8]	0.7 [0.6; 1.0]	0.4 [0.1; 1.0]	0.6 [0.1; 2.4]
Zambia	Country	3 [3; 4]	3 [2; 5]	4 [2; 9]	0.3 [0.2; 0.4]	0.8 [0.5; 1.2]	1.1 [0.4; 2.6]
Zimbabwe	Country	4 [3; 5]	1 [1; 2]	2 [1; 5]	0.3 [0.2; 0.5]	0.3 [0.2; 0.6]	0.5 [0.2; 1.4]

Table 16: **Estimates and uncertainty intervals for births (in thousands) by mothers aged 15–19 and the percentage of births in each location among all births of 15–19 for 1990, 2015, and 2023 for the world, seven SDG regions, 22 sub-regions, and 201 countries/areas*.** *We report results from countries/areas with more than 90,000 population size by mid-2024. Percentages may not sum up to 1 due to rounding.

		Age-specific births 15–19					
		Number (in thousands)			Proportion (in %)		
**Location Name**	**Location Type**	**1990**	**2015**	**2023**	**1990**	**2015**	**2023**
World	World	18,900 [18,400; 19,500]	13,500 [13,200; 13,800]	12,200 [11,500; 12,900]	100	100	100
Sub-Saharan Africa	SDG region	3,630 [3,550; 3,720]	5,710 [5,520; 5,920]	6,150 [5,620; 6,740]	19.2 [18.5; 19.9]	42.2 [41.1; 43.4]	50.6 [47.7; 53.5]
Latin America and the Caribbean	SDG region	2,040 [1,890; 2,240]	1,810 [1,750; 1,880]	1,320 [1,180; 1,480]	10.8 [10.0; 11.7]	13.4 [12.9; 13.9]	10.9 [9.7; 12.2]
Oceania (excluding Australia and New Zealand)	SDG region	25 [22; 28]	32 [29; 37]	32 [23; 47]	0.1 [0.1; 0.1]	0.2 [0.2; 0.3]	0.3 [0.2; 0.4]
Northern Africa and Western Asia	SDG region	979 [942; 1,020]	974 [884; 1,080]	883 [748; 1,050]	5.2 [4.9; 5.4]	7.2 [6.6; 7.9]	7.3 [6.2; 8.6]
Central and Southern Asia	SDG region	8,040 [7,530; 8,590]	2,910 [2,760; 3,070]	2,440 [2,100; 2,840]	42.4 [40.8; 44.1]	21.5 [20.5; 22.5]	20.1 [17.6; 22.8]
Eastern and South-Eastern Asia	SDG region	2,830 [2,700; 2,980]	1,570 [1,460; 1,700]	1,000 [874; 1,160]	15.0 [14.2; 15.7]	11.6 [10.9; 12.5]	8.3 [7.2; 9.6]
Europe, Northern America, Australia, and New Zealand	SDG region	1,390 [1,350; 1,430]	515 [499; 533]	319 [292; 350]	7.3 [7.1; 7.7]	3.8 [3.7; 4.0]	2.6 [2.4; 2.9]
Northern America	Subregion	540 [508; 575]	235 [221; 251]	149 [124; 178]	2.9 [2.7; 3.1]	1.7 [1.6; 1.9]	1.2 [1.0; 1.5]
Eastern Africa	Subregion	1,530 [1,480; 1,580]	2,300 [2,200; 2,410]	2,520 [2,220; 2,860]	8.1 [7.7; 8.4]	17.0 [16.3; 17.7]	20.8 [18.5; 23.2]
Middle Africa	Subregion	517 [489; 547]	1,070 [971; 1,180]	1,270 [1,010; 1,620]	2.7 [2.6; 2.9]	7.9 [7.2; 8.6]	10.5 [8.5; 13.1]
Northern Africa	Subregion	431 [406; 459]	494 [421; 580]	452 [344; 589]	2.3 [2.1; 2.4]	3.7 [3.1; 4.3]	3.7 [2.8; 4.8]
Southern Africa	Subregion	215 [196; 237]	202 [188; 216]	162 [133; 200]	1.1 [1.0; 1.3]	1.5 [1.4; 1.6]	1.3 [1.1; 1.7]
Western Africa	Subregion	1,370 [1,310; 1,430]	2,140 [2,020; 2,280]	2,190 [1,880; 2,600]	7.2 [6.9; 7.6]	15.8 [15.0; 16.7]	18.0 [15.7; 20.9]
Central Asia	Subregion	111 [106; 116]	95 [91; 100]	89 [79; 101]	0.6 [0.6; 0.6]	0.7 [0.7; 0.7]	0.7 [0.6; 0.8]
Eastern Asia	Subregion	1,440 [1,330; 1,570]	446 [359; 555]	206 [138; 316]	7.6 [7.0; 8.3]	3.3 [2.7; 4.1]	1.7 [1.1; 2.6]
Southern Asia	Subregion	7,930 [7,420; 8,480]	2,810 [2,660; 2,970]	2,350 [2,020; 2,750]	41.9 [40.2; 43.6]	20.8 [19.8; 21.8]	19.3 [16.9; 22.1]
South-Eastern Asia	Subregion	1,390 [1,330; 1,450]	1,120 [1,060; 1,200]	799 [692; 927]	7.3 [7.0; 7.7]	8.3 [7.8; 8.8]	6.6 [5.7; 7.6]
Western Asia	Subregion	548 [521; 576]	480 [433; 533]	431 [356; 532]	2.9 [2.7; 3.1]	3.5 [3.2; 3.9]	3.6 [2.9; 4.4]
Eastern Europe	Subregion	559 [540; 579]	166 [159; 174]	103 [93; 115]	3.0 [2.8; 3.1]	1.2 [1.2; 1.3]	0.8 [0.8; 1.0]
Northern Europe	Subregion	94 [89; 98]	34 [33; 36]	19 [16; 24]	0.5 [0.5; 0.5]	0.3 [0.2; 0.3]	0.2 [0.1; 0.2]
Southern Europe	Subregion	89 [87; 92]	30 [29; 31]	20 [18; 22]	0.5 [0.5; 0.5]	0.2 [0.2; 0.2]	0.2 [0.1; 0.2]
Western Europe	Subregion	89 [86; 93]	38 [36; 40]	20 [18; 23]	0.5 [0.4; 0.5]	0.3 [0.3; 0.3]	0.2 [0.1; 0.2]
Caribbean	Subregion	154 [147; 161]	105 [98; 112]	77 [64; 94]	0.8 [0.8; 0.9]	0.8 [0.7; 0.8]	0.6 [0.5; 0.8]
Central America	Subregion	608 [494; 754]	585 [547; 626]	498 [392; 642]	3.2 [2.6; 4.0]	4.3 [4.0; 4.6]	4.1 [3.2; 5.2]
South America	Subregion	1,280 [1,180; 1,410]	1,120 [1,070; 1,180]	749 [675; 830]	6.8 [6.2; 7.4]	8.3 [7.9; 8.7]	6.2 [5.5; 6.9]
Australia/New Zealand	Subregion	20 [19; 21]	11 [11; 12]	7 [6; 8]	0.1 [0.1; 0.1]	0.1 [0.1; 0.1]	0.1 [0.0; 0.1]
Melanesia	Subregion	22 [19; 25]	30 [26; 34]	30 [21; 45]	0.1 [0.1; 0.1]	0.2 [0.2; 0.3]	0.3 [0.2; 0.4]
Micronesia	Subregion	2 [1; 2]	1 [1; 1]	1 [0; 0]	0.0 [0.0; 0.0]	0.0 [0.0; 0.0]	0.0 [0.0; 0.0]
Polynesia	Subregion	2 [1; 2]	1 [1; 1]	1 [0; 0]	0.0 [0.0; 0.0]	0.0 [0.0; 0.0]	0.0 [0.0; 0.0]
Afghanistan	Country	91 [80; 103]	159 [130; 193]	146 [102; 208]	0.5 [0.4; 0.5]	1.2 [1.0; 1.4]	1.2 [0.8; 1.7]
Albania	Country	3 [2; 3]	3 [2; 3]	1 [1; 1]	0.0 [0.0; 0.0]	0.0 [0.0; 0.0]	0.0 [0.0; 0.0]
Algeria	Country	33 [30; 36]	15 [14; 16]	15 [10; 21]	0.2 [0.2; 0.2]	0.1 [0.1; 0.1]	0.1 [0.1; 0.2]
Angola	Country	82 [71; 94]	209 [169; 255]	271 [168; 422]	0.4 [0.4; 0.5]	1.5 [1.3; 1.9]	2.2 [1.4; 3.5]
Antigua and Barbuda	Country	0.2 [0.2; 0.2]	0.1 [0.1; 0.1]	0.1 [0.1; 0.1]	0.0 [0.0; 0.0]	0.0 [0.0; 0.0]	0.0 [0.0; 0.0]
Argentina	Country	98 [89; 108]	109 [100; 119]	45 [38; 55]	0.5 [0.5; 0.6]	0.8 [0.7; 0.9]	0.4 [0.3; 0.5]
Armenia	Country	11 [10; 12]	2 [2; 2]	1 [1; 2]	0.1 [0.1; 0.1]	0.0 [0.0; 0.0]	0.0 [0.0; 0.0]
Aruba	Country	0.1 [0.1; 0.1]	0.1 [0.1; 0.1]	0.06 [0.05; 0.09]	0.0 [0.0; 0.0]	0.0 [0.0; 0.0]	0.0 [0.0; 0.0]
Australia	Country	15 [14; 16]	9 [8; 9]	5 [4; 6]	0.1 [0.1; 0.1]	0.1 [0.1; 0.1]	0.0 [0.0; 0.1]
Austria	Country	6 [5; 6]	2 [2; 2]	0.8 [0.6; 1.1]	0.0 [0.0; 0.0]	0.0 [0.0; 0.0]	0.0 [0.0; 0.0]
Azerbaijan	Country	11 [10; 11]	20 [18; 22]	12 [10; 15]	0.1 [0.1; 0.1]	0.1 [0.1; 0.2]	0.1 [0.1; 0.1]
Bahamas	Country	0.8 [0.4; 1.4]	0.5 [0.3; 0.6]	0.3 [0.2; 0.6]	0.0 [0.0; 0.0]	0.0 [0.0; 0.0]	0.0 [0.0; 0.0]
Bahrain	Country	0.4 [0.4; 0.5]	0.5 [0.5; 0.6]	0.3 [0.2; 0.4]	0.0 [0.0; 0.0]	0.0 [0.0; 0.0]	0.0 [0.0; 0.0]

Continued on next page

Table 16 – continued from previous page

Location Name	Location Type	Age-specific births 15–19: Number (in thousands) 1990	2015	2023	Proportion (in %) 1990	2015	2023
Bangladesh	Country	1,020 [957; 1,100]	740 [680; 808]	631 [519; 764]	5.4 [5.0; 5.8]	5.5 [5.0; 6.0]	5.2 [4.2; 6.3]
Barbados	Country	0.7 [0.6; 0.8]	0.4 [0.3; 0.7]	0.4 [0.2; 0.8]	0.0 [0.0; 0.0]	0.0 [0.0; 0.0]	0.0 [0.0; 0.0]
Belarus	Country	15 [14; 16]	4 [4; 4]	2 [1; 3]	0.1 [0.1; 0.1]	0.0 [0.0; 0.0]	0.0 [0.0; 0.0]
Belgium	Country	4 [4; 4]	2 [2; 2]	1 [1; 2]	0.0 [0.0; 0.0]	0.0 [0.0; 0.0]	0.0 [0.0; 0.0]
Belize	Country	1 [1; 1]	1 [1; 1]	1 [1; 1]	0.0 [0.0; 0.0]	0.0 [0.0; 0.0]	0.0 [0.0; 0.0]
Benin	Country	35 [32; 38]	57 [50; 64]	57 [41; 80]	0.2 [0.2; 0.2]	0.4 [0.4; 0.5]	0.5 [0.3; 0.7]
Bhutan	Country	3 [2; 5]	0.6 [0.4; 0.7]	0.3 [0.2; 0.4]	0.0 [0.0; 0.0]	0.0 [0.0; 0.0]	0.0 [0.0; 0.0]
Bolivia (Plurinational State of)	Country	36 [33; 39]	40 [27; 58]	37 [21; 66]	0.2 [0.2; 0.2]	0.3 [0.2; 0.4]	0.3 [0.2; 0.6]
Bosnia and Herzegovina	Country	7 [7; 8]	1 [1; 1]	0.9 [0.6; 1.3]	0.0 [0.0; 0.0]	0.0 [0.0; 0.0]	0.0 [0.0; 0.0]
Botswana	Country	7 [6; 9]	6 [6; 7]	7 [5; 9]	0.0 [0.0; 0.0]	0.0 [0.0; 0.1]	0.1 [0.0; 0.1]
Brazil	Country	660 [558; 780]	515 [474; 559]	316 [264; 381]	3.5 [3.0; 4.1]	3.8 [3.5; 4.1]	2.6 [2.2; 3.1]
Brunei Darussalam	Country	0.4 [0.4; 0.5]	0.2 [0.2; 0.2]	0.1 [0.1; 0.2]	0.0 [0.0; 0.0]	0.0 [0.0; 0.0]	0.0 [0.0; 0.0]
Bulgaria	Country	22 [21; 24]	6 [6; 6]	6 [5; 8]	0.1 [0.1; 0.1]	0.0 [0.0; 0.0]	0.1 [0.0; 0.1]
Burkina Faso	Country	68 [62; 74]	117 [104; 132]	112 [81; 151]	0.4 [0.3; 0.4]	0.9 [0.8; 1.0]	0.9 [0.7; 1.2]
Burundi	Country	22 [19; 25]	33 [28; 39]	41 [25; 66]	0.1 [0.1; 0.1]	0.2 [0.2; 0.3]	0.3 [0.2; 0.5]
Cabo Verde	Country	3 [2; 3]	1 [1; 2]	0.9 [0.6; 1.2]	0.0 [0.0; 0.0]	0.0 [0.0; 0.0]	0.0 [0.0; 0.0]
Cambodia	Country	32 [28; 37]	39 [33; 46]	37 [27; 51]	0.2 [0.1; 0.2]	0.3 [0.2; 0.3]	0.3 [0.2; 0.4]
Cameroon	Country	95 [88; 102]	142 [123; 165]	162 [109; 238]	0.5 [0.5; 0.5]	1.1 [0.9; 1.2]	1.3 [0.9; 1.9]
Canada	Country	24 [22; 25]	10 [9; 11]	5 [4; 6]	0.1 [0.1; 0.1]	0.1 [0.1; 0.1]	0.0 [0.0; 0.1]
Central African Republic	Country	23 [21; 27]	46 [39; 54]	53 [36; 74]	0.1 [0.1; 0.1]	0.3 [0.3; 0.4]	0.4 [0.3; 0.6]
Chad	Country	65 [58; 73]	124 [106; 144]	138 [95; 199]	0.3 [0.3; 0.4]	0.9 [0.8; 1.1]	1.1 [0.8; 1.6]
Chile	Country	43 [40; 47]	26 [24; 28]	4 [3; 4]	0.2 [0.2; 0.3]	0.2 [0.2; 0.2]	0.0 [0.0; 0.0]
China	Country	1,390 [1,280; 1,520]	423 [336; 533]	196 [127; 303]	7.4 [6.8; 8.0]	3.1 [2.5; 3.9]	1.6 [1.0; 2.5]
China, Hong Kong SAR	Country	1 [1; 1]	0.6 [0.5; 0.7]	0.2 [0.1; 0.2]	0.0 [0.0; 0.0]	0.0 [0.0; 0.0]	0.0 [0.0; 0.0]
Macao, SAR of China	Country	0.1 [0.1; 0.1]	0.04 [0.03; 0.05]	0.007 [0.005; 0.008]	0.0 [0.0; 0.0]	0.0 [0.0; 0.0]	0.0 [0.0; 0.0]
China, Taiwan Province of China	Country	15 [14; 16]	3 [3; 3]	1 [1; 2]	0.1 [0.1; 0.1]	0.0 [0.0; 0.0]	0.0 [0.0; 0.0]
Colombia	Country	155 [139; 172]	145 [133; 157]	117 [97; 141]	0.8 [0.7; 0.9]	1.1 [1.0; 1.2]	1.0 [0.8; 1.2]
Comoros	Country	2 [2; 3]	3 [2; 3]	2 [1; 4]	0.0 [0.0; 0.0]	0.0 [0.0; 0.0]	0.0 [0.0; 0.0]
Republic of the Congo	Country	16 [14; 19]	29 [22; 38]	35 [21; 58]	0.1 [0.1; 0.1]	0.2 [0.2; 0.3]	0.3 [0.2; 0.5]
Costa Rica	Country	13 [12; 14]	11 [10; 12]	5 [4; 6]	0.1 [0.1; 0.1]	0.1 [0.1; 0.1]	0.0 [0.0; 0.0]
Côte d'Ivoire	Country	105 [94; 117]	138 [121; 157]	146 [107; 198]	0.6 [0.5; 0.6]	1.0 [0.9; 1.2]	1.2 [0.9; 1.6]
Croatia	Country	5 [4; 5]	1 [1; 1]	0.6 [0.6; 0.7]	0.0 [0.0; 0.0]	0.0 [0.0; 0.0]	0.0 [0.0; 0.0]
Cuba	Country	42 [38; 45]	17 [16; 19]	14 [11; 18]	0.2 [0.2; 0.2]	0.1 [0.1; 0.1]	0.1 [0.1; 0.1]
Curaçao	Country	0.3 [0.3; 0.4]	0.2 [0.1; 0.2]	0.07 [0.05; 0.1]	0.0 [0.0; 0.0]	0.0 [0.0; 0.0]	0.0 [0.0; 0.0]
Cyprus	Country	0.9 [0.9; 1]	0.2 [0.2; 0.2]	0.2 [0.2; 0.3]	0.0 [0.0; 0.0]	0.0 [0.0; 0.0]	0.0 [0.0; 0.0]
Czechia	Country	17 [16; 18]	3 [2; 3]	2 [1; 2]	0.1 [0.1; 0.1]	0.0 [0.0; 0.0]	0.0 [0.0; 0.0]
Dem. People's Republic of Korea	Country	3 [2; 6]	0.6 [0.4; 0.9]	0.4 [0.2; 0.8]	0.0 [0.0; 0.0]	0.0 [0.0; 0.0]	0.0 [0.0; 0.0]
Democratic Republic of the Congo	Country	223 [200; 249]	496 [416; 593]	588 [374; 900]	1.2 [1.1; 1.3]	3.7 [3.1; 4.4]	4.8 [3.1; 7.2]
Denmark	Country	2 [2; 2]	0.6 [0.6; 0.7]	0.2 [0.2; 0.2]	0.0 [0.0; 0.0]	0.0 [0.0; 0.0]	0.0 [0.0; 0.0]
Djibouti	Country	1 [1; 2]	1 [1; 2]	1 [1; 2]	0.0 [0.0; 0.0]	0.0 [0.0; 0.0]	0.0 [0.0; 0.0]
Dominican Republic	Country	47 [43; 51]	41 [37; 44]	25 [21; 30]	0.2 [0.2; 0.3]	0.3 [0.3; 0.3]	0.2 [0.2; 0.3]
Ecuador	Country	52 [46; 59]	60 [55; 66]	43 [33; 54]	0.3 [0.2; 0.3]	0.4 [0.4; 0.5]	0.4 [0.3; 0.5]
Egypt	Country	220 [201; 241]	254 [202; 319]	214 [152; 295]	1.2 [1.1; 1.3]	1.9 [1.5; 2.4]	1.8 [1.3; 2.4]
El Salvador	Country	36 [34; 39]	24 [22; 26]	15 [10; 21]	0.2 [0.2; 0.2]	0.2 [0.2; 0.2]	0.1 [0.1; 0.2]
Equatorial Guinea	Country	4 [3; 5]	10 [7; 14]	13 [7; 21]	0.0 [0.0; 0.0]	0.1 [0.1; 0.1]	0.1 [0.1; 0.2]
Eritrea	Country	10 [9; 12]	13 [8; 21]	14 [7; 26]	0.1 [0.0; 0.1]	0.1 [0.1; 0.2]	0.1 [0.1; 0.2]
Estonia	Country	3 [3; 3]	0.4 [0.3; 0.4]	0.2 [0.1; 0.2]	0.0 [0.0; 0.0]	0.0 [0.0; 0.0]	0.0 [0.0; 0.0]

Continued on next page

Table 16 – continued from previous page

Location Name	Location Type	Age-specific births 15–19 Number (in thousands) 1990	2015	2023	Proportion (in %) 1990	2015	2023
Eswatini	Country	7 [6; 7]	5 [4; 7]	4 [3; 7]	0.0 [0.0; 0.0]	0.0 [0.0; 0.0]	0.0 [0.0; 0.1]
Ethiopia	Country	430 [392; 472]	470 [413; 534]	497 [333; 732]	2.3 [2.1; 2.5]	3.5 [3.1; 3.9]	4.1 [2.8; 5.9]
Fiji	Country	3 [2; 3]	0.9 [0.9; 1]	0.9 [0.6; 1.3]	0.0 [0.0; 0.0]	0.0 [0.0; 0.0]	0.0 [0.0; 0.0]
Finland	Country	2 [2; 2]	0.9 [0.9; 1]	0.5 [0.4; 0.6]	0.0 [0.0; 0.0]	0.0 [0.0; 0.0]	0.0 [0.0; 0.0]
France	Country	27 [25; 29]	16 [15; 17]	7 [6; 8]	0.1 [0.1; 0.2]	0.1 [0.1; 0.1]	0.1 [0.1; 0.1]
French Guiana	Country	0.5 [0.4; 0.6]	0.9 [0.8; 1]	0.8 [0.6; 1]	0.0 [0.0; 0.0]	0.0 [0.0; 0.0]	0.0 [0.0; 0.0]
French Polynesia	Country	0.6 [0.6; 0.7]	0.4 [0.4; 0.5]	0.3 [0.2; 0.3]	0.0 [0.0; 0.0]	0.0 [0.0; 0.0]	0.0 [0.0; 0.0]
Gabon	Country	7 [7; 8]	10 [8; 11]	10 [7; 15]	0.0 [0.0; 0.0]	0.1 [0.1; 0.1]	0.1 [0.1; 0.1]
Gambia	Country	8 [7; 9]	9 [8; 11]	9 [6; 13]	0.0 [0.0; 0.0]	0.1 [0.1; 0.1]	0.1 [0.0; 0.1]
Georgia	Country	13 [12; 14]	5 [5; 6]	2 [2; 3]	0.1 [0.1; 0.1]	0.0 [0.0; 0.0]	0.0 [0.0; 0.0]
Germany	Country	46 [43; 49]	16 [15; 17]	10 [8; 13]	0.2 [0.2; 0.3]	0.1 [0.1; 0.1]	0.1 [0.1; 0.1]
Ghana	Country	92 [83; 100]	102 [91; 114]	99 [74; 131]	0.5 [0.4; 0.5]	0.8 [0.7; 0.8]	0.8 [0.6; 1.1]
Greece	Country	8 [7; 8]	2 [2; 2]	2 [1; 2]	0.0 [0.0; 0.0]	0.0 [0.0; 0.0]	0.0 [0.0; 0.0]
Grenada	Country	0.5 [0.4; 0.5]	0.2 [0.1; 0.2]	0.1 [0.1; 0.2]	0.0 [0.0; 0.0]	0.0 [0.0; 0.0]	0.0 [0.0; 0.0]
Guadeloupe	Country	0.7 [0.6; 0.8]	0.2 [0.2; 0.2]	0.1 [0.1; 0.1]	0.0 [0.0; 0.0]	0.0 [0.0; 0.0]	0.0 [0.0; 0.0]
Guam	Country	0.4 [0.3; 0.5]	0.3 [0.2; 0.3]	0.2 [0.1; 0.3]	0.0 [0.0; 0.0]	0.0 [0.0; 0.0]	0.0 [0.0; 0.0]
Guatemala	Country	58 [53; 62]	77 [71; 85]	64 [50; 81]	0.3 [0.3; 0.3]	0.6 [0.5; 0.6]	0.5 [0.4; 0.7]
Guinea	Country	48 [44; 52]	85 [76; 95]	91 [66; 125]	0.3 [0.2; 0.3]	0.6 [0.6; 0.7]	0.8 [0.5; 1.0]
Guinea-Bissau	Country	6 [5; 7]	9 [8; 11]	10 [6; 15]	0.0 [0.0; 0.0]	0.1 [0.1; 0.1]	0.1 [0.1; 0.1]
Guyana	Country	5 [4; 5]	3 [3; 4]	2 [2; 4]	0.0 [0.0; 0.0]	0.0 [0.0; 0.0]	0.0 [0.0; 0.0]
Haiti	Country	30 [26; 34]	32 [27; 38]	29 [18; 46]	0.2 [0.1; 0.2]	0.2 [0.2; 0.3]	0.2 [0.1; 0.4]
Honduras	Country	35 [30; 41]	44 [28; 68]	43 [22; 79]	0.2 [0.2; 0.2]	0.3 [0.2; 0.5]	0.4 [0.2; 0.7]
Hungary	Country	14 [13; 15]	6 [6; 6]	4 [3; 5]	0.1 [0.1; 0.1]	0.0 [0.0; 0.0]	0.0 [0.0; 0.0]
Iceland	Country	0.3 [0.3; 0.4]	0.08 [0.07; 0.09]	0.04 [0.03; 0.05]	0.0 [0.0; 0.0]	0.0 [0.0; 0.0]	0.0 [0.0; 0.0]
India	Country	5,840 [5,340; 6,380]	1,100 [998; 1,220]	859 [631; 1,190]	30.9 [28.9; 32.8]	8.2 [7.4; 9.0]	7.1 [5.2; 9.5]
Indonesia	Country	731 [681; 786]	413 [364; 470]	297 [219; 402]	3.9 [3.6; 4.2]	3.1 [2.7; 3.5]	2.4 [1.8; 3.3]
Iran (Islamic Republic of)	Country	228 [196; 266]	108 [95; 123]	79 [62; 101]	1.2 [1.0; 1.4]	0.8 [0.7; 0.9]	0.6 [0.5; 0.8]
Iraq	Country	73 [64; 84]	149 [122; 179]	132 [81; 212]	0.4 [0.3; 0.4]	1.1 [0.9; 1.3]	1.1 [0.7; 1.8]
Ireland	Country	3 [2; 3]	1 [1; 1]	0.7 [0.5; 0.9]	0.0 [0.0; 0.0]	0.0 [0.0; 0.0]	0.0 [0.0; 0.0]
Israel	Country	4 [4; 4]	3 [3; 3]	2 [2; 3]	0.0 [0.0; 0.0]	0.0 [0.0; 0.0]	0.0 [0.0; 0.0]
Italy	Country	19 [18; 20]	7 [7; 8]	4 [3; 5]	0.1 [0.1; 0.1]	0.1 [0.0; 0.1]	0.0 [0.0; 0.0]
Jamaica	Country	14 [12; 16]	6 [5; 7]	4 [3; 6]	0.1 [0.1; 0.1]	0.0 [0.0; 0.0]	0.0 [0.0; 0.0]
Japan	Country	18 [17; 19]	12 [11; 13]	5 [4; 6]	0.1 [0.1; 0.1]	0.1 [0.1; 0.1]	0.0 [0.0; 0.0]
Jersey	Country	0.06 [0.06; 0.07]	0.03 [0.03; 0.04]	0.02 [0.02; 0.03]	0.0 [0.0; 0.0]	0.0 [0.0; 0.0]	0.0 [0.0; 0.0]
Jordan	Country	11 [10; 11]	14 [12; 16]	10 [7; 14]	0.1 [0.1; 0.1]	0.1 [0.1; 0.1]	0.1 [0.1; 0.1]
Kazakhstan	Country	38 [34; 41]	19 [18; 21]	13 [11; 16]	0.2 [0.2; 0.2]	0.1 [0.1; 0.2]	0.1 [0.1; 0.1]
Kenya	Country	176 [162; 191]	208 [183; 235]	179 [146; 220]	0.9 [0.9; 1.0]	1.5 [1.4; 1.7]	1.5 [1.2; 1.8]
Kiribati	Country	0.2 [0.2; 0.3]	0.3 [0.2; 0.4]	0.3 [0.2; 0.4]	0.0 [0.0; 0.0]	0.0 [0.0; 0.0]	0.0 [0.0; 0.0]
Kosovo (under UNSC res. 1244)	Country	4 [3; 4]	0.8 [0.8; 0.9]	0.6 [0.4; 0.8]	0.0 [0.0; 0.0]	0.0 [0.0; 0.0]	0.0 [0.0; 0.0]
Kuwait	Country	2 [2; 3]	0.8 [0.7; 0.8]	0.2 [0.2; 0.3]	0.0 [0.0; 0.0]	0.0 [0.0; 0.0]	0.0 [0.0; 0.0]
Kyrgyz Republic	Country	11 [10; 11]	11 [10; 12]	8 [7; 10]	0.1 [0.1; 0.1]	0.1 [0.1; 0.1]	0.1 [0.1; 0.1]
Lao People's Democratic Republic	Country	24 [21; 27]	30 [25; 36]	29 [22; 40]	0.1 [0.1; 0.1]	0.2 [0.2; 0.3]	0.2 [0.2; 0.3]
Latvia	Country	4 [4; 5]	0.7 [0.7; 0.8]	0.4 [0.3; 0.4]	0.0 [0.0; 0.0]	0.0 [0.0; 0.0]	0.0 [0.0; 0.0]
Lebanon	Country	9 [7; 10]	6 [3; 11]	5 [2; 12]	0.0 [0.0; 0.1]	0.0 [0.0; 0.1]	0.0 [0.0; 0.1]
Lesotho	Country	8 [7; 9]	9 [8; 11]	8 [6; 12]	0.0 [0.0; 0.0]	0.1 [0.1; 0.1]	0.1 [0.0; 0.1]
Liberia	Country	22 [20; 24]	34 [30; 38]	39 [29; 52]	0.1 [0.1; 0.1]	0.2 [0.2; 0.3]	0.3 [0.2; 0.4]
Libya	Country	4 [3; 4]	2 [1; 3]	2 [1; 3]	0.0 [0.0; 0.0]	0.0 [0.0; 0.0]	0.0 [0.0; 0.0]

Continued on next page

Table 16 – continued from previous page

Location Name	Location Type	Age-specific births 15–19 Number (in thousands) 1990	2015	2023	Proportion (in %) 1990	2015	2023
Lithuania	Country	6 [5; 6]	1 [1; 1]	0.4 [0.3; 0.5]	0.0 [0.0; 0.0]	0.0 [0.0; 0.0]	0.0 [0.0; 0.0]
Luxembourg	Country	0.2 [0.1; 0.2]	0.08 [0.07; 0.1]	0.07 [0.05; 0.09]	0.0 [0.0; 0.0]	0.0 [0.0; 0.0]	0.0 [0.0; 0.0]
Madagascar	Country	87 [80; 95]	193 [168; 221]	214 [157; 295]	0.5 [0.4; 0.5]	1.4 [1.2; 1.6]	1.8 [1.3; 2.4]
Malawi	Country	78 [72; 84]	121 [106; 138]	143 [95; 206]	0.4 [0.4; 0.4]	0.9 [0.8; 1.0]	1.2 [0.8; 1.7]
Malaysia	Country	17 [15; 19]	16 [15; 17]	8 [7; 10]	0.1 [0.1; 0.1]	0.1 [0.1; 0.1]	0.1 [0.1; 0.1]
Maldives	Country	1 [1; 2]	0.2 [0.2; 0.2]	0.08 [0.05; 0.11]	0.0 [0.0; 0.0]	0.0 [0.0; 0.0]	0.0 [0.0; 0.0]
Mali	Country	75 [70; 81]	151 [132; 172]	183 [134; 250]	0.4 [0.4; 0.4]	1.1 [1.0; 1.3]	1.5 [1.1; 2.1]
Malta	Country	0.08 [0.08; 0.09]	0.1 [0.1; 0.2]	0.1 [0.1; 0.1]	0.0 [0.0; 0.0]	0.0 [0.0; 0.0]	0.0 [0.0; 0.0]
Martinique	Country	0.6 [0.5; 0.6]	0.2 [0.2; 0.2]	0.09 [0.07; 0.12]	0.0 [0.0; 0.0]	0.0 [0.0; 0.0]	0.0 [0.0; 0.0]
Mauritania	Country	12 [11; 14]	21 [18; 24]	24 [17; 34]	0.1 [0.1; 0.1]	0.2 [0.1; 0.2]	0.2 [0.1; 0.3]
Mauritius	Country	2 [2; 2]	1 [1; 1]	0.9 [0.7; 1.1]	0.0 [0.0; 0.0]	0.0 [0.0; 0.0]	0.0 [0.0; 0.0]
Mayotte	Country	0.6 [0.4; 0.8]	0.9 [0.8; 1]	1 [1; 2]	0.0 [0.0; 0.0]	0.0 [0.0; 0.0]	0.0 [0.0; 0.0]
Mexico	Country	415 [300; 558]	380 [350; 413]	331 [230; 468]	2.2 [1.6; 2.9]	2.8 [2.6; 3.1]	2.7 [1.9; 3.8]
Micronesia (Fed. States of)	Country	0.4 [0.3; 0.5]	0.3 [0.2; 0.5]	0.3 [0.1; 0.5]	0.0 [0.0; 0.0]	0.0 [0.0; 0.0]	0.0 [0.0; 0.0]
Mongolia	Country	4 [4; 5]	4 [3; 4]	2 [2; 3]	0.0 [0.0; 0.0]	0.0 [0.0; 0.0]	0.0 [0.0; 0.0]
Montenegro	Country	0.7 [0.7; 0.8]	0.2 [0.2; 0.3]	0.2 [0.1; 0.2]	0.0 [0.0; 0.0]	0.0 [0.0; 0.0]	0.0 [0.0; 0.0]
Morocco	Country	61 [54; 68]	43 [27; 67]	38 [20; 75]	0.3 [0.3; 0.4]	0.3 [0.2; 0.5]	0.3 [0.2; 0.6]
Mozambique	Country	113 [102; 125]	251 [215; 289]	279 [212; 363]	0.6 [0.5; 0.7]	1.9 [1.6; 2.1]	2.3 [1.7; 3.0]
Myanmar	Country	103 [88; 121]	79 [62; 101]	73 [43; 122]	0.5 [0.5; 0.6]	0.6 [0.5; 0.7]	0.6 [0.4; 1.0]
Namibia	Country	8 [7; 8]	9 [7; 12]	9 [5; 15]	0.0 [0.0; 0.0]	0.1 [0.1; 0.1]	0.1 [0.0; 0.1]
Nepal	Country	130 [122; 140]	135 [120; 152]	101 [74; 137]	0.7 [0.6; 0.7]	1.0 [0.9; 1.1]	0.8 [0.6; 1.1]
Netherlands	Country	5 [4; 5]	2 [1; 2]	0.9 [0.7; 1.2]	0.0 [0.0; 0.0]	0.0 [0.0; 0.0]	0.0 [0.0; 0.0]
New Caledonia	Country	0.4 [0.4; 0.5]	0.2 [0.2; 0.2]	0.1 [0.1; 0.2]	0.0 [0.0; 0.0]	0.0 [0.0; 0.0]	0.0 [0.0; 0.0]
New Zealand	Country	5 [5; 5]	3 [3; 3]	2 [1; 2]	0.0 [0.0; 0.0]	0.0 [0.0; 0.0]	0.0 [0.0; 0.0]
Nicaragua	Country	36 [33; 40]	32 [22; 46]	30 [17; 51]	0.2 [0.2; 0.2]	0.2 [0.2; 0.3]	0.2 [0.1; 0.4]
Niger	Country	89 [82; 96]	184 [158; 213]	211 [153; 288]	0.5 [0.4; 0.5]	1.4 [1.2; 1.6]	1.7 [1.3; 2.4]
Nigeria	Country	704 [648; 765]	1,100 [984; 1,240]	1,070 [786; 1,470]	3.7 [3.4; 4.0]	8.1 [7.3; 9.1]	8.8 [6.6; 11.7]
North Macedonia	Country	5 [4; 5]	1 [1; 1]	0.7 [0.5; 0.8]	0.0 [0.0; 0.0]	0.0 [0.0; 0.0]	0.0 [0.0; 0.0]
Norway	Country	3 [3; 3]	0.7 [0.7; 0.8]	0.2 [0.2; 0.3]	0.0 [0.0; 0.0]	0.0 [0.0; 0.0]	0.0 [0.0; 0.0]
Oman	Country	8 [6; 10]	2 [2; 2]	0.8 [0.7; 1]	0.0 [0.0; 0.1]	0.0 [0.0; 0.0]	0.0 [0.0; 0.0]
Pakistan	Country	582 [530; 636]	552 [476; 640]	518 [370; 729]	3.1 [2.8; 3.4]	4.1 [3.5; 4.7]	4.3 [3.1; 5.9]
Panama	Country	13 [11; 14]	14 [13; 16]	10 [8; 13]	0.1 [0.1; 0.1]	0.1 [0.1; 0.1]	0.1 [0.1; 0.1]
Papua New Guinea	Country	16 [14; 19]	26 [22; 30]	26 [17; 41]	0.1 [0.1; 0.1]	0.2 [0.2; 0.2]	0.2 [0.1; 0.3]
Paraguay	Country	20 [18; 23]	24 [19; 29]	20 [12; 33]	0.1 [0.1; 0.1]	0.2 [0.1; 0.2]	0.2 [0.1; 0.3]
Peru	Country	102 [86; 120]	73 [62; 87]	62 [45; 84]	0.5 [0.5; 0.6]	0.5 [0.5; 0.6]	0.5 [0.4; 0.7]
Philippines	Country	174 [162; 187]	280 [257; 305]	176 [136; 226]	0.9 [0.8; 1.0]	2.1 [1.9; 2.3]	1.4 [1.1; 1.9]
Poland	Country	42 [40; 45]	13 [12; 14]	6 [5; 7]	0.2 [0.2; 0.2]	0.1 [0.1; 0.1]	0.0 [0.0; 0.1]
Portugal	Country	10 [9; 10]	2 [2; 2]	2 [1; 2]	0.1 [0.0; 0.1]	0.0 [0.0; 0.0]	0.0 [0.0; 0.0]
Puerto Rico	Country	12 [11; 13]	4 [4; 4]	1 [1; 2]	0.1 [0.1; 0.1]	0.0 [0.0; 0.0]	0.0 [0.0; 0.0]
Qatar	Country	0.6 [0.6; 0.7]	0.4 [0.3; 0.4]	0.3 [0.2; 0.4]	0.0 [0.0; 0.0]	0.0 [0.0; 0.0]	0.0 [0.0; 0.0]
Republic of Korea	Country	9 [9; 10]	3 [2; 3]	0.6 [0.5; 0.7]	0.1 [0.0; 0.1]	0.0 [0.0; 0.0]	0.0 [0.0; 0.0]
Republic of Moldova	Country	10 [10; 11]	4 [3; 4]	2 [2; 3]	0.1 [0.0; 0.1]	0.0 [0.0; 0.0]	0.0 [0.0; 0.0]
Réunion	Country	1 [1; 1]	0.8 [0.8; 0.9]	0.5 [0.4; 0.6]	0.0 [0.0; 0.0]	0.0 [0.0; 0.0]	0.0 [0.0; 0.0]
Romania	Country	49 [46; 52]	19 [18; 21]	18 [14; 23]	0.3 [0.2; 0.3]	0.1 [0.1; 0.2]	0.1 [0.1; 0.2]
Russian Federation	Country	276 [258; 294]	81 [75; 89]	49 [41; 59]	1.5 [1.4; 1.6]	0.6 [0.5; 0.7]	0.4 [0.3; 0.5]
Rwanda	Country	22 [19; 24]	24 [21; 27]	24 [17; 37]	0.1 [0.1; 0.1]	0.2 [0.2; 0.2]	0.2 [0.1; 0.3]
Saint Lucia	Country	0.8 [0.7; 0.9]	0.3 [0.2; 0.3]	0.2 [0.1; 0.2]	0.0 [0.0; 0.0]	0.0 [0.0; 0.0]	0.0 [0.0; 0.0]

Continued on next page

Table 16 – continued from previous page

Location Name	Location Type	Age-specific births 15–19: Number (in thousands) 1990	2015	2023	Proportion (in %) 1990	2015	2023
Saint Vincent and the Grenadines	Country	0.6 [0.5; 0.7]	0.3 [0.2; 0.3]	0.2 [0.1; 0.2]	0.0 [0.0; 0.0]	0.0 [0.0; 0.0]	0.0 [0.0; 0.0]
Samoa	Country	0.5 [0.4; 0.6]	0.5 [0.4; 0.6]	0.5 [0.3; 0.7]	0.0 [0.0; 0.0]	0.0 [0.0; 0.0]	0.0 [0.0; 0.0]
Sao Tome and Principe	Country	0.8 [0.7; 1]	1 [1; 1]	1 [1; 2]	0.0 [0.0; 0.0]	0.0 [0.0; 0.0]	0.0 [0.0; 0.0]
Saudi Arabia	Country	28 [24; 33]	15 [10; 25]	13 [7; 23]	0.1 [0.1; 0.2]	0.1 [0.1; 0.2]	0.1 [0.1; 0.2]
Senegal	Country	44 [41; 48]	56 [50; 62]	59 [40; 84]	0.2 [0.2; 0.3]	0.4 [0.4; 0.5]	0.5 [0.3; 0.7]
Serbia	Country	9 [8; 10]	3 [3; 3]	2 [2; 3]	0.0 [0.0; 0.1]	0.0 [0.0; 0.0]	0.0 [0.0; 0.0]
Seychelles	Country	0.2 [0.2; 0.3]	0.2 [0.2; 0.2]	0.2 [0.1; 0.3]	0.0 [0.0; 0.0]	0.0 [0.0; 0.0]	0.0 [0.0; 0.0]
Sierra Leone	Country	33 [30; 37]	44 [40; 50]	43 [29; 62]	0.2 [0.2; 0.2]	0.3 [0.3; 0.4]	0.4 [0.2; 0.5]
Singapore	Country	0.9 [0.8; 1]	0.5 [0.4; 0.5]	0.4 [0.3; 0.5]	0.0 [0.0; 0.0]	0.0 [0.0; 0.0]	0.0 [0.0; 0.0]
Slovakia	Country	9 [9; 10]	3 [3; 4]	3 [3; 4]	0.0 [0.0; 0.1]	0.0 [0.0; 0.0]	0.0 [0.0; 0.0]
Slovenia	Country	2 [2; 2]	0.2 [0.2; 0.2]	0.2 [0.1; 0.2]	0.0 [0.0; 0.0]	0.0 [0.0; 0.0]	0.0 [0.0; 0.0]
Solomon Islands	Country	2 [2; 2]	2 [1; 3]	2 [1; 3]	0.0 [0.0; 0.0]	0.0 [0.0; 0.0]	0.0 [0.0; 0.0]
Somalia	Country	44 [37; 52]	100 [72; 136]	116 [68; 187]	0.2 [0.2; 0.3]	0.7 [0.5; 1.0]	1.0 [0.6; 1.5]
South Africa	Country	185 [166; 206]	172 [158; 186]	134 [105; 171]	1.0 [0.9; 1.1]	1.3 [1.2; 1.4]	1.1 [0.9; 1.4]
South Sudan	Country	42 [35; 50]	56 [36; 85]	68 [36; 121]	0.2 [0.2; 0.3]	0.4 [0.3; 0.6]	0.6 [0.3; 1.0]
Spain	Country	19 [18; 20]	8 [8; 9]	6 [5; 7]	0.1 [0.1; 0.1]	0.1 [0.1; 0.1]	0.0 [0.0; 0.1]
Sri Lanka	Country	25 [22; 29]	14 [13; 16]	13 [9; 22]	0.1 [0.1; 0.2]	0.1 [0.1; 0.1]	0.1 [0.1; 0.2]
State of Palestine	Country	13 [11; 15]	12 [10; 14]	10 [7; 15]	0.1 [0.1; 0.1]	0.1 [0.1; 0.1]	0.1 [0.1; 0.1]
Sudan	Country	105 [91; 122]	176 [132; 235]	180 [106; 297]	0.6 [0.5; 0.6]	1.3 [1.0; 1.7]	1.5 [0.9; 2.4]
Suriname	Country	2 [2; 2]	2 [1; 2]	1 [1; 2]	0.0 [0.0; 0.0]	0.0 [0.0; 0.0]	0.0 [0.0; 0.0]
Sweden	Country	4 [4; 4]	1 [1; 1]	0.5 [0.4; 0.6]	0.0 [0.0; 0.0]	0.0 [0.0; 0.0]	0.0 [0.0; 0.0]
Switzerland	Country	2 [1; 2]	0.6 [0.5; 0.6]	0.3 [0.3; 0.4]	0.0 [0.0; 0.0]	0.0 [0.0; 0.0]	0.0 [0.0; 0.0]
Syrian Arab Republic	Country	58 [49; 68]	43 [26; 71]	53 [26; 105]	0.3 [0.3; 0.4]	0.3 [0.2; 0.5]	0.4 [0.2; 0.9]
Tajikistan	Country	13 [12; 14]	22 [20; 24]	18 [13; 26]	0.1 [0.1; 0.1]	0.2 [0.2; 0.2]	0.2 [0.1; 0.2]
Thailand	Country	145 [132; 160]	118 [109; 128]	55 [44; 70]	0.8 [0.7; 0.8]	0.9 [0.8; 0.9]	0.5 [0.4; 0.6]
Timor-Leste	Country	2 [2; 2]	2 [2; 3]	2 [1; 3]	0.0 [0.0; 0.0]	0.0 [0.0; 0.0]	0.0 [0.0; 0.0]
Togo	Country	23 [20; 25]	32 [28; 38]	37 [24; 57]	0.1 [0.1; 0.1]	0.2 [0.2; 0.3]	0.3 [0.2; 0.5]
Tonga	Country	0.2 [0.2; 0.2]	0.1 [0.1; 0.2]	0.1 [0.1; 0.2]	0.0 [0.0; 0.0]	0.0 [0.0; 0.0]	0.0 [0.0; 0.0]
Trinidad and Tobago	Country	4 [3; 4]	2 [1; 3]	2 [1; 3]	0.0 [0.0; 0.0]	0.0 [0.0; 0.0]	0.0 [0.0; 0.0]
Tunisia	Country	8 [7; 9]	4 [3; 4]	2 [1; 2]	0.0 [0.0; 0.0]	0.0 [0.0; 0.0]	0.0 [0.0; 0.0]
Türkiye	Country	192 [172; 215]	84 [78; 90]	37 [31; 45]	1.0 [0.9; 1.1]	0.6 [0.6; 0.7]	0.3 [0.3; 0.4]
Turkmenistan	Country	4 [4; 5]	8 [7; 10]	5 [3; 8]	0.0 [0.0; 0.0]	0.1 [0.1; 0.1]	0.0 [0.0; 0.1]
Uganda	Country	178 [165; 192]	282 [254; 313]	305 [205; 442]	0.9 [0.9; 1.0]	2.1 [1.9; 2.3]	2.5 [1.7; 3.6]
Ukraine	Country	104 [97; 111]	27 [25; 29]	11 [8; 15]	0.6 [0.5; 0.6]	0.2 [0.2; 0.2]	0.1 [0.1; 0.1]
United Arab Emirates	Country	3 [2; 3]	1 [1; 1]	0.7 [0.6; 0.9]	0.0 [0.0; 0.0]	0.0 [0.0; 0.0]	0.0 [0.0; 0.0]
United Kingdom	Country	67 [63; 72]	27 [26; 29]	16 [13; 21]	0.4 [0.3; 0.4]	0.2 [0.2; 0.2]	0.1 [0.1; 0.2]
United Republic of Tanzania	Country	192 [177; 208]	346 [303; 394]	412 [267; 615]	1.0 [0.9; 1.1]	2.6 [2.2; 2.9]	3.4 [2.2; 5.0]
United States of America	Country	516 [484; 551]	225 [211; 241]	144 [119; 173]	2.7 [2.5; 2.9]	1.7 [1.6; 1.8]	1.2 [1.0; 1.4]
Uruguay	Country	8 [7; 9]	7 [6; 8]	3 [2; 4]	0.0 [0.0; 0.0]	0.1 [0.0; 0.1]	0.0 [0.0; 0.0]
Uzbekistan	Country	46 [42; 49]	34 [31; 37]	44 [37; 54]	0.2 [0.2; 0.3]	0.3 [0.2; 0.3]	0.4 [0.3; 0.4]
Vanuatu	Country	0.6 [0.5; 0.8]	0.9 [0.7; 1.3]	1 [0; 0]	0.0 [0.0; 0.0]	0.0 [0.0; 0.0]	0.0 [0.0; 0.0]
Venezuela (Bolivarian Republic of)	Country	101 [86; 118]	120 [110; 131]	97 [71; 133]	0.5 [0.5; 0.6]	0.9 [0.8; 1.0]	0.8 [0.6; 1.1]
Viet Nam	Country	157 [141; 173]	145 [119; 176]	121 [84; 174]	0.8 [0.7; 0.9]	1.1 [0.9; 1.3]	1.0 [0.7; 1.4]
Western Sahara	Country	0.6 [0.5; 0.7]	0.6 [0.3; 1.1]	0.6 [0.3; 1.3]	0.0 [0.0; 0.0]	0.0 [0.0; 0.0]	0.0 [0.0; 0.0]
Yemen	Country	110 [101; 120]	122 [92; 158]	151 [112; 200]	0.6 [0.5; 0.6]	0.9 [0.7; 1.2]	1.2 [0.9; 1.7]
Zambia	Country	68 [62; 73]	115 [99; 133]	135 [91; 197]	0.4 [0.3; 0.4]	0.9 [0.7; 1.0]	1.1 [0.7; 1.6]
Zimbabwe	Country	61 [56; 67]	80 [68; 93]	91 [62; 131]	0.3 [0.3; 0.4]	0.6 [0.5; 0.7]	0.7 [0.5; 1.1]

Table 17: **Age-specific fertility rate data sources for age group 10–14, by country.** For each country, the total number of observations and the most recent reference year are shown after the country name. For each country-specific data series, the number of observations and the most recent reference year within that series are shown before each data series name. The source type that each data series falls in is shown in parentheses after each data series name. Countries without data are shown in grey rows (if applicable). Only countries/areas with population size above 90,000 by mid-2024 are shown.

Country/Area	# obs.	Most recent obs. year	Data series name [source type]
Afghanistan	39	2021.5	
	19	2009.1	2010 DHS Special (Survey) [other DHS Direct]
	19	2014.3	2015 DHS (Survey) [DHS Direct]
	1	2021.5	2022-2023 MICS (Survey) [other DHS Direct]
Albania	23	2021.5	
	23	2021.5	Register (Register) [VR]
Algeria	78	2017.5	
	19	1991	1992 MCH (Survey) [other DHS Direct]
	19	2001.3	2002 MCH (Survey) [other DHS Direct]
	19	2011.5	2012-2013 MICS (Survey) [other DHS Direct]
	19	2017.5	2018-2019 MICS (Survey) [other DHS Direct]
	2	1979.5	Register (Register) [VR]
Angola	40	2014.5	
	2	2005.6	2006-2007 MIS (Survey) [other DHS Direct]
	19	2009.7	2011 MIS (Survey) [other DHS Direct]
	19	2014.5	2015-2016 DHS (Survey) [DHS Direct]
Antigua and Barbuda	16	2020.5	
	1	2010.9	2011 Census (Census) [Others Direct]
	15	2020.5	Register (Register) [VR]
Argentina	51	2021.5	
	51	2021.5	Register (Register) [VR]
Armenia	64	2019.5	
	13	1999.4	2000 DHS (Survey) [DHS Direct]
	13	2004.3	2005 DHS (Survey) [DHS Direct]
	18	2009.4	2010 DHS (Survey) [DHS Direct]
	15	2014.6	2015 DHS (Survey) [DHS Direct]
	5	2019.5	Register (Register) [VR]
Aruba	24	2021.5	
	24	2021.5	Register (Register) [VR]
Australia	71	2021.5	
	71	2021.5	Register (Register) [VR]
Austria	140	2021.5	
	69	2019.5	Estimates (Estimate) [VR]
	71	2021.5	Register (Register) [VR]
Azerbaijan	21	2013.5	
	15	2005.2	2006 DHS (Survey) [DHS Direct]
	6	2013.5	Register (Register) [VR]
Bahamas	4	2012.5	
	4	2012.5	Register (Register) [VR]

Continued on next page

Table 17 – continued from previous page

Country/Area	# obs.	Most recent obs. year	Data series name [source type]
Bahrain	37	2009.5	
	5	1982	1989 CHS (Survey) [other DHS Direct]
	32	2009.5	Register (Register) [VR]
Bangladesh	163	2020.8	
	2	1974.5	1975-1976 WFS (Survey) [other DHS Direct]
	12	1992.5	1993-1994 DHS (Survey) [DHS Direct]
	7	1995.5	1996-1997 DHS (Survey) [DHS Direct]
	14	1998.5	1999-2000 DHS (Survey) [DHS Direct]
	17	1999.7	2001 DHS Special (Survey) [other DHS Direct]
	16	2002.7	2004 DHS (Survey) [DHS Direct]
	18	2005.9	2007 DHS (Survey) [DHS Direct]
	19	2010.2	2011 DHS (Survey) [DHS Direct]
	19	2013.2	2014 DHS (Survey) [DHS Direct]
	19	2016.5	2017-2018 DHS (Survey) [DHS Direct]
	19	2017.7	2019 MICS (Survey) [other DHS Direct]
	1	2020.8	2022 DHS (Survey) [DHS Direct]
Barbados	42	2007.5	
	42	2007.5	Register (Register) [VR]
Belarus	71	2018.5	
	55	2018.5	Estimates (Estimate) [VR]
	16	2018.5	Register (Register) [VR]
Belgium	128	2019.5	
	59	2019.5	Estimates (Estimate) [VR]
	69	2018.5	Register (Register) [VR]
Belize	49	2022.5	
	19	2014.4	2015-2016 MICS (Survey) [other DHS Direct]
	1	2022.5	Estimates (Estimate) [VR]
	29	2021.5	Register (Register) [VR]
Benin	149	2020	
	16	1980.9	1981-1982 WFS (Survey) [other DHS Direct]
	19	1995	1996 DHS (Survey) [DHS Direct]
	19	2000.3	2001 DHS (Survey) [DHS Direct]
	19	2005.3	2006 DHS (Survey) [DHS Direct]
	19	2010.6	2011-2012 DHS (Survey) [DHS Direct]
	19	2012.8	2014 MICS (Survey) [other DHS Direct]
	19	2016.5	2017-2018 DHS (Survey) [DHS Direct]
	19	2020	2021 MICS (Survey) [other DHS Direct]
Bhutan			
Bolivia (Plurinational State of)	92	2006.8	
	19	1987.9	1989 DHS (Survey) [DHS Direct]
	19	1992.6	1994 DHS (Survey) [DHS Direct]
	18	1996	1998 DHS (Survey) [DHS Direct]
	17	2002.3	2003 DHS (Survey) [DHS Direct]

Continued on next page

Table 17 – continued from previous page

Country/Area	# obs.	Most recent obs. year	Data series name [source type]
	19	2006.8	2008 DHS (Survey) [DHS Direct]
Bosnia and Herzegovina	20	2019.5	
	20	2019.5	Register (Register) [VR]
Botswana	10	2019.5	
	1	2011.1	2011 Census (Census) [Others Direct]
	9	2019.5	Register (Register) [VR]
Brazil	55	2022.5	
	14	1985	1986 FP-MCH DHS (Survey) [DHS Direct]
	19	1994.8	1996 DHS (Survey) [DHS Direct]
	22	2022.5	Register (Register) [VR]
Brunei Darussalam	34	2021.5	
	34	2021.5	Register (Register) [VR]
Bulgaria	136	2022.5	
	71	2021.5	Estimates (Estimate) [VR]
	65	2022.5	Register (Register) [VR]
Burkina Faso	98	2020.2	
	19	1991.6	1993 DHS (Survey) [DHS Direct]
	19	1997.5	1998-1999 DHS (Survey) [DHS Direct]
	19	2002.2	2003 DHS (Survey) [DHS Direct]
	19	2009.2	2010 DHS (Survey) [DHS Direct]
	2	2016.6	2017-2018 MIS (Survey) [other DHS Direct]
	1	2019.4	2019 Census (Census) [Others Direct]
	19	2020.2	2021 DHS (Survey) [DHS Direct]
Burundi	59	2015.5	
	19	1985.9	1987 DHS (Survey) [DHS Direct]
	19	2009.3	2010 DHS (Survey) [DHS Direct]
	2	2011.5	2012 MIS (Survey) [other DHS Direct]
	19	2015.5	2016 DHS (Survey) [DHS Direct]
Cabo Verde	13	2020.5	
	13	2020.5	Register (Register) [VR]
Cambodia	94	2020.4	
	19	1998.8	2000 DHS (Survey) [DHS Direct]
	19	2004.5	2005 DHS (Survey) [DHS Direct]
	18	2009.3	2010 DHS (Survey) [DHS Direct]
	19	2013.2	2014 DHS (Survey) [DHS Direct]
	19	2020.4	2021-2022 DHS (Survey) [DHS Direct]
Cameroon	122	2017.2	
	14	1977	1978 WFS (Survey) [other DHS Direct]
	13	1990	1991 DHS (Survey) [DHS Direct]
	19	1996.8	1998 DHS (Survey) [DHS Direct]
	19	2002.9	2004 DHS (Survey) [DHS Direct]
	19	2009.8	2011 DHS (Survey) [DHS Direct]
	19	2013	2014 MICS (Survey) [other DHS Direct]
	19	2017.2	2018 DHS (Survey) [DHS Direct]
Canada	137	2021.5	

Continued on next page

Table 17 – continued from previous page

Country/Area	# obs.	Most recent obs. year	Data series name [source type]
	70	2019.5	Estimates (Estimate) [VR]
	67	2021.5	Register (Register) [VR]
Central African Republic	37	2018	
	19	1993.5	1994-1995 DHS (Survey) [DHS Direct]
	18	2018	2019 MICS (Survey) [other DHS Direct]
Chad	68	2018	
	17	1995.7	1996-1997 DHS (Survey) [DHS Direct]
	18	2003.2	2004 DHS (Survey) [DHS Direct]
	14	2013.5	2014-2015 DHS (Survey) [DHS Direct]
	19	2018	2019 MICS (Survey) [other DHS Direct]
Chile	72	2023.5	
	72	2023.5	Register (Register) [VR]
China			
China, Hong Kong SAR	53	2021.5	
	53	2021.5	Register (Register) [VR]
Macao, SAR of China	15	2009.5	
	15	2009.5	Register (Register) [VR]
China, Taiwan Province of China	44	2019.5	
	44	2019.5	Estimates (Estimate) [VR]
Colombia	90	2022.5	
	11	1985.3	1986 DHS (Survey) [DHS Direct]
	13	1993.8	1995 DHS (Survey) [DHS Direct]
	15	1998.8	2000 DHS (Survey) [DHS Direct]
	14	2003.6	2005 DHS (Survey) [DHS Direct]
	19	2008.9	2010 DHS (Survey) [DHS Direct]
	18	2022.5	Register (Register) [VR]
Comoros	38	2011.3	
	19	1994.8	1996 DHS (Survey) [DHS Direct]
	19	2011.3	2012 DHS (Survey) [DHS Direct]
Republic of the Congo	57	2013.5	
	19	2004.2	2005 DHS (Survey) [DHS Direct]
	19	2010.4	2011-2012 DHS (Survey) [DHS Direct]
	19	2013.5	2014-2015 MICS (Survey) [other DHS Direct]
Costa Rica	47	2022.5	
	19	1991.5	1993 RHS (Survey) [other DHS Direct]
	28	2022.5	Register (Register) [VR]
Côte d'Ivoire	130	2021.4	
	16	1979.5	1980-1981 WFS (Survey) [other DHS Direct]
	19	1993.2	1994 DHS (Survey) [DHS Direct]
	18	1997.5	1998-1999 DHS (Survey) [DHS Direct]
	18	2004.2	2005 EIS (Survey) [other DHS Direct]

Continued on next page

Table 17 – continued from previous page

Country/Area	# obs.	Most recent obs. year	Data series name [source type]
	19	2010.7	2011-2012 DHS (Survey) [DHS Direct]
	1	2013.7	2014 Census (Census) [Others Direct]
	19	2015.1	2016 MICS (Survey) [other DHS Direct]
	1	2021.4	2021 Census (Census) [Others Direct]
	19	2020.3	2021 DHS (Survey) [DHS Direct]
Croatia	75	2023.5	
	20	2020.5	Estimates (Estimate) [VR]
	55	2023.5	Register (Register) [VR]
Cuba	38	2021.5	
	38	2021.5	Register (Register) [VR]
Curaçao	8	2016.5	
	8	2016.5	Register (Register) [VR]
Cyprus	7	2020.5	
	7	2020.5	Register (Register) [VR]
Czechia	133	2021.5	
	72	2021.5	Estimates (Estimate) [VR]
	61	2021.5	Register (Register) [VR]
Dem. People's Republic of Korea			
Democratic Republic of the Congo	56	2016.7	
	19	2005.8	2007 DHS (Survey) [DHS Direct]
	18	2012.4	2013-2014 DHS (Survey) [DHS Direct]
	19	2016.7	2017-2018 MICS (Survey) [other DHS Direct]
Denmark	133	2021.5	
	68	2021.5	Estimates (Estimate) [VR]
	65	2021.5	Register (Register) [VR]
Djibouti	37	2011.1	
	18	2001.4	2002 FHS (Survey) [other DHS Direct]
	19	2011.1	2012 FHS (Survey) [other DHS Direct]
Dominican Republic	135	2022.5	
	15	1978	1980 WFS (Survey) [other DHS Direct]
	17	1995.3	1996 DHS (Survey) [DHS Direct]
	19	1998.3	1999 DHS (Survey) [DHS Direct]
	18	2000.3	2002 DHS (Survey) [DHS Direct]
	12	2005.9	2007 DHS (Survey) [DHS Direct]
	16	2012.2	2013 DHS (Survey) [DHS Direct]
	18	2018	2019 MICS (Survey) [other DHS Direct]
	20	2022.5	Register (Register) [VR]
Ecuador	74	2021.5	
	2	1988.1	1989 ENDEMAIN-RHS (Survey) [other DHS Direct]
	17	1993.1	1994 ENDEMAIN-RHS (Survey) [other DHS Direct]
	17	1998	1999 ENDEMAIN-RHS (Survey) [other DHS Direct]
	17	2002.8	2004 ENDEMAIN-RHS (Survey) [other DHS Direct]

Continued on next page

Table 17 – continued from previous page

Country/Area	# obs.	Most recent obs. year	Data series name [source type]
	21	2021.5	Register (Register) [VR]
Egypt	187	2012.9	
	16	1979	1980 WFS (Survey) [other DHS Direct]
	19	1987.4	1988 DHS (Survey) [DHS Direct]
	19	1989.6	1991 PAPCHILD (Survey) [other DHS Direct]
	19	1991.5	1992 DHS (Survey) [DHS Direct]
	19	1994.5	1995 DHS (Survey) [DHS Direct]
	19	1998.7	2000 DHS (Survey) [DHS Direct]
	19	2001.9	2003 DHS Interim (Survey) [other DHS Direct]
	19	2003.9	2005 DHS (Survey) [DHS Direct]
	19	2006.8	2008 DHS (Survey) [DHS Direct]
	19	2012.9	2014 DHS (Survey) [DHS Direct]
El Salvador	130	2018.5	
	2	1983.9	1985 DHS (Survey) [DHS Direct]
	2	1986.9	1988 FESAL (Survey) [other DHS Direct]
	2	1991.9	1993 FESAL (Survey) [other DHS Direct]
	19	1997.1	1998 FESAL (Survey) [other DHS Direct]
	16	2001.7	2002-2003 FESAL (Survey) [other DHS Direct]
	19	2007	2008 FESAL (Survey) [other DHS Direct]
	19	2012.9	2014 MICS (Survey) [other DHS Direct]
	51	2018.5	Register (Register) [VR]
Equatorial Guinea			
Eritrea	57	2008.8	
	19	1994.4	1995 DHS (Survey) [DHS Direct]
	19	2000.9	2002 DHS (Survey) [DHS Direct]
	19	2008.8	2010 PHS (Survey) [other DHS Direct]
Estonia	103	2021.5	
	53	2019.5	Estimates (Estimate) [VR]
	50	2021.5	Register (Register) [VR]
Eswatini	57	2013.2	
	19	2005.3	2006-2007 DHS (Survey) [DHS Direct]
	19	2009.3	2010 MICS (Survey) [other DHS Direct]
	19	2013.2	2014 MICS (Survey) [other DHS Direct]
Ethiopia	101	2017.8	
	19	1998.7	2000 DHS (Survey) [DHS Direct]
	19	2004	2005 DHS (Survey) [DHS Direct]
	19	2009.7	2011 DHS (Survey) [DHS Direct]
	19	2012.7	2014 MDHS (Survey) [Others Direct]
	19	2014.8	2016 DHS (Survey) [DHS Direct]
	6	2017.8	2019 DHS (Survey) [DHS Direct]
Fiji	60	2019.7	
	12	1973	1974 WFS (Survey) [other DHS Direct]
	17	2019.7	2021 MICS (Survey) [other DHS Direct]
	31	2017.5	Register (Register) [VR]
Finland	139	2021.5	
	70	2021.5	Estimates (Estimate) [VR]

Continued on next page

Table 17 – continued from previous page

Country/Area	# obs.	Most recent obs. year	Data series name [source type]
	69	2021.5	Register (Register) [VR]
France	142	2021.5	
	72	2021.5	Estimates (Estimate) [VR]
	70	2020.5	Register (Register) [VR]
French Guiana	37	2021.5	
	37	2021.5	Register (Register) [VR]
French Polynesia	5	2021.5	
	5	2021.5	Register (Register) [VR]
Gabon	55	2019.3	
	17	1999.3	2000 DHS (Survey) [DHS Direct]
	19	2010.7	2012 DHS (Survey) [DHS Direct]
	19	2019.3	2019-2021 DHS (Survey) [DHS Direct]
Gambia	52	2018.5	
	18	2011.7	2013 DHS (Survey) [DHS Direct]
	16	2016.7	2018 MICS (Survey) [other DHS Direct]
	18	2018.5	2019-2020 DHS (Survey) [DHS Direct]
Georgia	18	2020.5	
	18	2020.5	Register (Register) [VR]
Germany	91	2021.5	
	62	2017.5	Estimates (Estimate) [VR]
	29	2021.5	Register (Register) [VR]
Ghana	209	2021.4	
	14	1978.2	1979-1980 WFS (Survey) [other DHS Direct]
	19	1986.8	1988 DHS (Survey) [DHS Direct]
	19	1992.4	1993 DHS (Survey) [DHS Direct]
	19	1997.5	1998 DHS (Survey) [DHS Direct]
	19	2002.2	2003 DHS (Survey) [DHS Direct]
	19	2006.4	2007 DHS Special (Survey) [other DHS Direct]
	19	2007.3	2008 DHS (Survey) [DHS Direct]
	19	2010.3	2011 MICS (Survey) [other DHS Direct]
	19	2013.3	2014 DHS (Survey) [DHS Direct]
	2	2015.4	2016 MIS (Survey) [other DHS Direct]
	19	2016.4	2017-2018 MICS (Survey) [other DHS Direct]
	19	2016.1	2017 MHS (Survey) [other DHS Direct]
	2	2018.3	2019 MIS (Survey) [other DHS Direct]
	1	2021.4	2022 DHS (Survey) [DHS Direct]
Greece	95	2021.5	
	29	2009.5	Estimates (Estimate) [VR]
	66	2021.5	Register (Register) [VR]
Grenada	37	2018.5	
	37	2018.5	Register (Register) [VR]
Guadeloupe	27	2021.5	
	27	2021.5	Register (Register) [VR]
Guam	45	2018.5	
	45	2018.5	Register (Register) [VR]
Guatemala	131	2021.5	

Continued on next page

Table 17 – continued from previous page

Country/Area	# obs.	Most recent obs. year	Data series name [source type]
	16	1986.3	1987 DHS (Survey) [DHS Direct]
	19	1994.2	1995 DHS (Survey) [DHS Direct]
	19	1997.6	1998-1999 DHS Interim (Survey) [other DHS Direct]
	19	2001.1	2002 ENSMI (Survey) [other DHS Direct]
	58	2021.5	Register (Register) [VR]
Guinea	94	2020.1	
	17	1997.9	1999 DHS (Survey) [DHS Direct]
	19	2003.8	2005 DHS (Survey) [DHS Direct]
	19	2011.1	2012 DHS (Survey) [DHS Direct]
	1	2013.7	2014 Census (Census) [Others Direct]
	19	2015	2016 MICS (Survey) [other DHS Direct]
	17	2016.8	2018 DHS (Survey) [DHS Direct]
	2	2020.1	2021 MIS (Survey) [other DHS Direct]
Guinea-Bissau	38	2017.5	
	19	2012.9	2014 MICS (Survey) [other DHS Direct]
	19	2017.5	2018-2019 MICS (Survey) [other DHS Direct]
Guyana	98	2018.5	
	13	1973.9	1975 WFS (Survey) [other DHS Direct]
	19	2004.1	2005 AIS (Survey) [other DHS Direct]
	19	2007.9	2009 DHS (Survey) [DHS Direct]
	19	2013	2014 MICS (Survey) [other DHS Direct]
	19	2018.5	2019-2020 MICS (Survey) [other DHS Direct]
	9	1972.5	Register (Register) [VR]
Haiti	83	2015.6	
	11	1970	1977 WFS (Survey) [other DHS Direct]
	18	1998.8	2000 DHS (Survey) [DHS Direct]
	17	2004.6	2005-2006 DHS (Survey) [DHS Direct]
	18	2010.7	2012 DHS (Survey) [DHS Direct]
	19	2015.6	2016-2017 DHS (Survey) [DHS Direct]
Honduras	38	1999.9	
	19	1994.9	1996 ENESF (Survey) [other DHS Direct]
	19	1999.9	2001 ENESF (Survey) [other DHS Direct]
Hungary	143	2021.5	
	71	2020.5	Estimates (Estimate) [VR]
	72	2021.5	Register (Register) [VR]
Iceland	72	2018.5	
	33	2018.5	Estimates (Estimate) [VR]
	39	2018.5	Register (Register) [VR]
India	95	2018.9	
	19	1991.5	1992-1993 NFHS (Survey) [DHS Direct]
	19	1997.9	1998-1999 NFHS (Survey) [DHS Direct]
	19	2004.8	2005-2006 NFHS (Survey) [DHS Direct]
	19	2014.5	2015-2016 NFHS (Survey) [DHS Direct]
	19	2018.9	2019-2021 NFHS (Survey) [other DHS Direct]
Indonesia	152	2016.1	
	19	1986.3	1987 CPS (Survey) [DHS Direct]

Continued on next page

Table 17 – continued from previous page

Country/Area	# obs.	Most recent obs. year	Data series name [source type]
	19	1989.9	1991 DHS (Survey) [DHS Direct]
	19	1993.2	1994 DHS (Survey) [DHS Direct]
	19	1996.3	1997 DHS (Survey) [DHS Direct]
	19	2001.5	2002-2003 DHS (Survey) [DHS Direct]
	19	2006.3	2007 DHS (Survey) [DHS Direct]
	19	2011	2012 SDKI (Survey) [Others Direct]
	17	2016.1	2017 DHS (Survey) [DHS Direct]
	2	2005.1	2017 YARHS DHS Special (Survey) [Indirect]
Iran (Islamic Republic of)	13	2021.5	
	1	2016	2016 Census (Census) [Others Direct]
	12	2021.5	Register (Register) [VR]
Iraq	57	2016.8	
	19	2004.8	2006 MICS (Survey) [other DHS Direct]
	19	2009.7	2011 MICS (Survey) [other DHS Direct]
	19	2016.8	2018 MICS (Survey) [other DHS Direct]
Ireland	100	2021.5	
	33	2020.5	Estimates (Estimate) [VR]
	67	2021.5	Register (Register) [VR]
Israel	64	2021.5	
	27	2009.5	Estimates (Estimate) [VR]
	37	2021.5	Register (Register) [VR]
Italy	130	2021.5	
	67	2020.5	Estimates (Estimate) [VR]
	63	2021.5	Register (Register) [VR]
Jamaica	42	2006.5	
	10	1968.5	1975-1976 WFS (Survey) [other DHS Direct]
	32	2006.5	Register (Register) [VR]
Japan	114	2021.5	
	72	2021.5	Estimates (Estimate) [VR]
	42	2019.5	Register (Register) [VR]
Jersey	121	2021.5	
	71	2020.5	Estimates (Estimate) [VR]
	50	2021.5	Register (Register) [VR]
Jordan	138	2016.3	
	19	1989.4	1990 DHS (Survey) [DHS Direct]
	19	1996.1	1997 DHS (Survey) [DHS Direct]
	19	2001.1	2002 DHS (Survey) [DHS Direct]
	19	2006.2	2007 DHS (Survey) [DHS Direct]
	19	2008.4	2009 DHS Interim (Survey) [other DHS Direct]
	16	2011.3	2012 DHS (Survey) [DHS Direct]
	17	2016.3	2017-2018 DHS (Survey) [DHS Direct]
	10	1978.5	Register (Register) [VR]
Kazakhstan	43	2018.5	
	11	1994	1995 DHS (Survey) [DHS Direct]
	11	1998.1	1999 DHS (Survey) [DHS Direct]

Continued on next page

Table 17 – continued from previous page

Country/Area	# obs.	Most recent obs. year	Data series name [source type]
	21	2018.5	Register (Register) [VR]
Kenya	134	2022.5	
	13	1976.5	1977-1978 WFS (Survey) [other DHS Direct]
	15	1987.7	1989 DHS (Survey) [DHS Direct]
	19	1991.9	1993 DHS (Survey) [DHS Direct]
	18	1996.8	1998 DHS (Survey) [DHS Direct]
	19	2002	2003 DHS (Survey) [DHS Direct]
	19	2007.5	2008-2009 DHS (Survey) [DHS Direct]
	19	2013.1	2014 DHS (Survey) [DHS Direct]
	2	2014.1	2015 MIS (Survey) [other DHS Direct]
	1	2019.2	2019 Census (Census) [Others Direct]
	2	2019.3	2020 MIS (Survey) [other DHS Direct]
	7	2022.5	Register (Register) [VR]
Kiribati	23	2017.5	
	19	2017.5	2018-2019 MICS (Survey) [other DHS Direct]
	4	1996.5	Register (Register) [VR]
Kosovo (under UNSC res. 1244)	48	2019.5	
	48	2019.5	Register (Register) [VR]
Kuwait	3	1976.5	
	3	1976.5	Register (Register) [VR]
Kyrgyz Republic	84	2021.5	
	13	1996.2	1997 DHS (Survey) [DHS Direct]
	13	2011.3	2012 DHS (Survey) [DHS Direct]
	15	2012.9	2014 MICS (Survey) [other DHS Direct]
	17	2017.3	2018 MICS (Survey) [other DHS Direct]
	26	2021.5	Register (Register) [VR]
Lao People's Democratic Republic	38	2016.1	
	19	2010.4	2011-2012 DHS-MICS Special (Survey) [other DHS Direct]
	19	2016.1	2017 MICS (Survey) [other DHS Direct]
Latvia	64	2021.5	
	37	2017.5	Estimates (Estimate) [VR]
	27	2021.5	Register (Register) [VR]
Lebanon	38	2003.1	
	19	1994.6	1996 MCHS (Survey) [other DHS Direct]
	19	2003.1	2004 FHS (Survey) [other DHS Direct]
Lesotho	90	2017	
	14	1976.1	1977 WFS (Survey) [other DHS Direct]
	19	2003.4	2004 DHS (Survey) [DHS Direct]
	19	2008.4	2009 DHS (Survey) [DHS Direct]
	19	2013.3	2014 DHS (Survey) [DHS Direct]
	19	2017	2018 MICS (Survey) [other DHS Direct]
Liberia	98	2021.4	
	19	1984.8	1986 DHS (Survey) [DHS Direct]

Continued on next page

Table 17 – continued from previous page

Country/Area	# obs.	Most recent obs. year	Data series name [source type]
	19	2005.6	2007 DHS (Survey) [DHS Direct]
	16	2007.6	2009 MIS (Survey) [other DHS Direct]
	2	2010.3	2011 MIS (Survey) [other DHS Direct]
	19	2011.9	2013 DHS (Survey) [DHS Direct]
	2	2015.3	2016 MIS (Survey) [other DHS Direct]
	19	2018.5	2019-2020 DHS (Survey) [DHS Direct]
	2	2021.4	2022 MIS (Survey) [other DHS Direct]
Libya	19	1994	
	19	1994	1995 MCH (Survey) [other DHS Direct]
Lithuania	94	2021.5	
	62	2020.5	Estimates (Estimate) [VR]
	32	2021.5	Register (Register) [VR]
Luxembourg	77	2018.5	
	42	2014.5	Estimates (Estimate) [VR]
	35	2018.5	Register (Register) [VR]
Madagascar	126	2019.8	
	19	1991.1	1992 DHS (Survey) [DHS Direct]
	19	1996.3	1997 DHS (Survey) [DHS Direct]
	19	2002.5	2003-2004 DHS (Survey) [DHS Direct]
	19	2007.7	2008-2009 DHS (Survey) [DHS Direct]
	2	2009.8	2011 MIS (Survey) [other DHS Direct]
	2	2011.9	2013 MIS (Survey) [other DHS Direct]
	2	2015	2016 MIS (Survey) [other DHS Direct]
	19	2017.3	2018 MICS (Survey) [other DHS Direct]
	19	2019.8	2021 DHS (Survey) [DHS Direct]
	6	1972.5	Register (Register) [VR]
Malawi	159	2018.8	
	1	1977.2	1977 Census (Census) [Others Direct]
	19	1991.3	1992 DHS (Survey) [DHS Direct]
	19	1999.2	2000 DHS (Survey) [DHS Direct]
	19	2003.4	2004 DHS (Survey) [DHS Direct]
	19	2005.2	2006 MICS (Survey) [other DHS Direct]
	19	2009.2	2010 DHS (Survey) [DHS Direct]
	2	2010.8	2012 MIS (Survey) [other DHS Direct]
	19	2012.6	2013-2014 MICS (Survey) [other DHS Direct]
	2	2012.9	2014 MIS (Survey) [other DHS Direct]
	19	2014.5	2015-2016 DHS (Survey) [DHS Direct]
	2	2015.8	2017 MIS (Survey) [other DHS Direct]
	19	2018.8	2019-2020 MICS (Survey) [other DHS Direct]
Malaysia	21	2021.5	
	1	2020	2020 Census (Census) [Others Direct]
	20	2021.5	Register (Register) [VR]
Maldives	52	2019.5	
	19	2007.9	2009 DHS (Survey) [DHS Direct]
	17	2015.7	2016-2017 DHS (Survey) [DHS Direct]

Continued on next page

Table 17 – continued from previous page

Country/Area	# obs.	Most recent obs. year	Data series name [source type]
	16	2019.5	Register (Register) [VR]
Mali	129	2020.3	
	15	1985.9	1987 DHS (Survey) [DHS Direct]
	19	1994.6	1995-1996 DHS (Survey) [DHS Direct]
	19	1999.7	2001 DHS (Survey) [DHS Direct]
	19	2005.2	2006 DHS (Survey) [DHS Direct]
	17	2011.5	2012-2013 DHS (Survey) [DHS Direct]
	19	2014.2	2015 MICS (Survey) [other DHS Direct]
	2	2014.3	2015 MIS (Survey) [other DHS Direct]
	17	2017.2	2018 DHS (Survey) [DHS Direct]
	2	2020.3	2021 MIS (Survey) [other DHS Direct]
Malta	30	2020.5	
	30	2020.5	Register (Register) [VR]
Martinique	37	2020.5	
	37	2020.5	Register (Register) [VR]
Mauritania	73	2019.1	
	3	1988.5	1990-1991 MCHS (Survey) [other DHS Direct]
	8	1999.5	2000-2001 DHS (Survey) [DHS Direct]
	7	2002.4	2003-2004 DHS-NS (Survey) [other DHS Direct]
	18	2010.1	2011 MICS (Survey) [other DHS Direct]
	18	2014.2	2015 MICS (Survey) [other DHS Direct]
	19	2019.1	2019-2021 DHS (Survey) [DHS Direct]
Mauritius	31	2022.5	
	31	2022.5	Register (Register) [VR]
Mayotte			
Mexico	41	2018.5	
	8	1969.4	1976-1977 WFS (Survey) [other DHS Direct]
	18	1985.7	1987 DHS (Survey) [DHS Direct]
	15	2018.5	Register (Register) [VR]
Micronesia (Fed. States of)	8	2006.5	
	8	2006.5	Register (Register) [VR]
Mongolia	44	2022.5	
	18	2012.4	2013 MICS (Survey) [other DHS Direct]
	19	2017.4	2018 MICS (Survey) [other DHS Direct]
	1	2020	2020 Census (Census) [Others Direct]
	6	2022.5	Register (Register) [VR]
Montenegro	34	2021.5	
	34	2021.5	Register (Register) [VR]
Morocco	113	2009.5	
	16	1978.5	1980 WFS (Survey) [other DHS Direct]
	19	1985.9	1987 DHS (Survey) [DHS Direct]
	19	1990.7	1992 DHS (Survey) [DHS Direct]
	19	1993.8	1995 DHS Special (Panel) [Others Direct]
	19	1996.3	1997 ENSME (Survey) [other DHS Direct]
	19	2002.5	2003-2004 DHS (Survey) [other DHS Direct]

Continued on next page

Table 17 – continued from previous page

Country/Area	# obs.	Most recent obs. year	Data series name [source type]
	2	2009.5	2011 ENPSF (Survey) [other DHS Direct]
Mozambique	65	2021.3	
	14	1995.9	1997 DHS (Survey) [DHS Direct]
	17	2002.3	2003 DHS (Survey) [DHS Direct]
	10	2007.3	2008 MICS (Survey) [other DHS Direct]
	19	2010.2	2011 DHS (Survey) [DHS Direct]
	2	2014.1	2015 AIS (Survey) [other DHS Direct]
	2	2016.9	2018 MIS (Survey) [other DHS Direct]
	1	2021.3	2022-2023 DHS (Survey) [DHS Direct]
Myanmar	19	2014.8	
	19	2014.8	2015-2016 DHS (Survey) [DHS Direct]
Namibia	76	2012	
	19	1991.2	1992 DHS (Survey) [DHS Direct]
	19	1999.3	2000 DHS (Survey) [DHS Direct]
	19	2005.5	2006-2007 DHS (Survey) [DHS Direct]
	19	2012	2013 DHS (Survey) [DHS Direct]
Nepal	152	2020.8	
	19	1994.7	1996 DHS (Survey) [DHS Direct]
	19	1999.7	2001 DHS (Survey) [DHS Direct]
	19	2004.9	2006 DHS (Survey) [DHS Direct]
	19	2009.7	2011 DHS (Survey) [DHS Direct]
	19	2012.8	2014 MICS (Survey) [other DHS Direct]
	19	2015.2	2016 DHS (Survey) [DHS Direct]
	19	2018	2019 MICS (Survey) [other DHS Direct]
	19	2020.8	2022 DHS (Survey) [DHS Direct]
Netherlands	80	2019.5	
	70	2019.5	Estimates (Estimate) [VR]
	10	2006.5	Register (Register) [VR]
New Caledonia	25	2002.5	
	25	2002.5	Register (Register) [VR]
New Zealand	61	2022.5	
	61	2022.5	Register (Register) [VR]
Nicaragua	70	2010.5	
	15	1991.5	1992-1993 ESF-RHS (Survey) [other DHS Direct]
	17	1996.7	1998 DHS (Survey) [DHS Direct]
	17	2000.3	2001 DHS (Survey) [DHS Direct]
	17	2005.5	2006-2007 RHS-NDHS (Survey) [other DHS Direct]
	4	2010.5	Register (Register) [VR]
Niger	70	2020.2	
	10	1990.8	1992 DHS (Survey) [DHS Direct]
	19	1996.9	1998 DHS (Survey) [DHS Direct]
	19	2004.7	2006 DHS (Survey) [other DHS Direct]
	19	2010.8	2012 DHS (Survey) [DHS Direct]
	1	2020	2021 ENAFEME (Survey) [Others Direct]
	2	2020.2	2021 MIS (Survey) [other DHS Direct]
Nigeria	143	2020.4	

Continued on next page

Table 17 – continued from previous page

Country/Area	# obs.	Most recent obs. year	Data series name [source type]
	9	1980.7	1981-1982 WFS (Survey) [other DHS Direct]
	13	1989	1990 DHS (Survey) [DHS Direct]
	13	2001.9	2003 DHS (Survey) [DHS Direct]
	19	2007.1	2008 DHS (Survey) [DHS Direct]
	11	2009.4	2010 MIS (Survey) [other DHS Direct]
	19	2011.8	2013 DHS (Survey) [DHS Direct]
	2	2014.3	2015 MIS (Survey) [other DHS Direct]
	19	2015.4	2016-2017 MICS (Survey) [other DHS Direct]
	17	2017.3	2018 DHS (Survey) [DHS Direct]
	19	2020.3	2021 MICS (Survey) [other DHS Direct]
	2	2020.4	2021 MIS (Survey) [other DHS Direct]
North Macedonia	34	2022.5	
	34	2022.5	Register (Register) [VR]
Norway	105	2021.5	
	47	2014.5	Estimates (Estimate) [VR]
	58	2021.5	Register (Register) [VR]
Oman	5	2021.5	
	5	2021.5	Register (Register) [VR]
Pakistan	55	2016.4	
	19	2005.4	2006-2007 DHS (Survey) [DHS Direct]
	19	2011.5	2012-2013 DHS (Survey) [DHS Direct]
	17	2016.4	2017-2018 DHS (Survey) [DHS Direct]
Panama	48	2021.5	
	48	2021.5	Register (Register) [VR]
Papua New Guinea	17	2016.4	
	17	2016.4	2016-2018 DHS (Survey) [DHS Direct]
Paraguay	116	2015.1	
	14	1978	1979 WFS (Survey) [other DHS Direct]
	19	1989	1990 DHS (Survey) [DHS Direct]
	16	1994.5	1995-1996 ENDSR-RHS (Survey) [other DHS Direct]
	16	1997.3	1998 ENSMI-RHS (Survey) [other DHS Direct]
	16	2002.9	2004 ENDSSR-RHS (Survey) [other DHS Direct]
	16	2007.1	2008 ENDSSR-RHS (Survey) [other DHS Direct]
	19	2015.1	2016 MICS (Survey) [other DHS Direct]
Peru	140	2011.1	
	1	1981	1981 Census (Census) [Others Direct]
	19	1985.3	1986 DHS (Survey) [DHS Direct]
	18	1989.5	1991-1992 DHS (Survey) [DHS Direct]
	15	1999.2	2000 DHS (Survey) [DHS Direct]
	14	2002.8	2004 DHS (Survey) [DHS Direct]
	14	2006.4	2007-2008 DHS (Survey) [DHS Direct]
	14	2008	2009 DHS (Survey) [DHS Direct]
	15	2009.1	2010 DHS (Survey) [DHS Direct]
	16	2010.1	2011 DHS (Survey) [DHS Direct]
	14	2011.1	2012 DHS (Survey) [DHS Direct]
Philippines	198	2021.5	

Continued on next page

Table 17 – continued from previous page

Country/Area	# obs.	Most recent obs. year	Data series name [source type]
	14	1976.8	1978 WFS (Survey) [other DHS Direct]
	19	1991.8	1993 DHS (Survey) [DHS Direct]
	19	1996.7	1998 DHS (Survey) [DHS Direct]
	19	2002.1	2003 DHS (Survey) [DHS Direct]
	19	2007.2	2008 DHS (Survey) [DHS Direct]
	19	2012.2	2013 DHS (Survey) [DHS Direct]
	19	2016.2	2017 DHS (Survey) [DHS Direct]
	2	2009.9	2022 DHS (Survey) [DHS Direct]
	68	2021.5	Register (Register) [VR]
Poland	104	2021.5	
	49	2019.5	Estimates (Estimate) [VR]
	55	2021.5	Register (Register) [VR]
Portugal	157	2021.5	
	14	1978.5	1979-1980 WFS (Survey) [other DHS Direct]
	72	2021.5	Estimates (Estimate) [VR]
	71	2021.5	Register (Register) [VR]
Puerto Rico	72	2022.5	
	1	2009.7	2010 Census (Census) [Others Direct]
	71	2022.5	Register (Register) [VR]
Qatar	13	1994.5	
	3	1982	1987 CHS (Survey) [other DHS Direct]
	10	1994.5	Register (Register) [VR]
Republic of Korea	61	2021.5	
	21	2020.5	Estimates (Estimate) [VR]
	40	2021.5	Register (Register) [VR]
Republic of Moldova	39	2010.9	
	19	2004	2005 DHS (Survey) [DHS Direct]
	17	2010.9	2012 MICS (Survey) [other DHS Direct]
	3	1989.5	Register (Register) [VR]
Réunion	35	2021.5	
	35	2021.5	Register (Register) [VR]
Romania	66	2021.5	
	66	2021.5	Register (Register) [VR]
Russian Federation	65	2022.5	
	57	2018.5	Estimates (Estimate) [VR]
	8	2022.5	Register (Register) [VR]
Rwanda	152	2018.6	
	17	1982	1983 WFS (Survey) [other DHS Direct]
	19	1991.1	1992 DHS (Survey) [DHS Direct]
	19	1999	2000 DHS (Survey) [DHS Direct]
	19	2003.8	2005 DHS (Survey) [DHS Direct]
	19	2006.6	2007-2008 DHS Interim (Survey) [other DHS Direct]
	19	2009.5	2010 DHS (Survey) [DHS Direct]
	2	2011.7	2013 MIS (Survey) [other DHS Direct]
	19	2013.6	2014-2015 DHS (Survey) [DHS Direct]

Continued on next page

Table 17 – continued from previous page

Country/Area	# obs.	Most recent obs. year	Data series name [source type]
	19	2018.6	2019-2020 DHS (Survey) [DHS Direct]
Saint Lucia	35	2020.5	
	35	2020.5	Register (Register) [VR]
Saint Vincent and the Grenadines	44	2021.5	
	44	2021.5	Register (Register) [VR]
Samoa	26	2018.5	
	19	2018.5	2019-2020 MICS (Survey) [other DHS Direct]
	7	1975.5	Register (Register) [VR]
Sao Tome and Principe	64	2018	
	19	2007.4	2008-2009 DHS (Survey) [DHS Direct]
	19	2013	2014 MICS (Survey) [other DHS Direct]
	19	2018	2019 MICS (Survey) [other DHS Direct]
	7	1979.5	Register (Register) [VR]
Saudi Arabia			
Senegal	248	2019.5	
	14	1977.1	1978 WFS (Survey) [other DHS Direct]
	19	1984.9	1986 DHS (Survey) [DHS Direct]
	19	1991.7	1992-1993 DHS (Survey) [DHS Direct]
	19	1995.7	1997 DHS (Survey) [DHS Direct]
	19	2003.8	2005 DHS (Survey) [DHS Direct]
	2	2005.4	2006 MIS (Survey) [other DHS Direct]
	2	2007.5	2008-2009 MIS (Survey) [other DHS Direct]
	19	2009.5	2010-2011 DHS (Survey) [DHS Direct]
	19	2011.6	2012-2013 DHS (Survey) [DHS Direct]
	19	2012.9	2014 DHS (Survey) [DHS Direct]
	19	2013.9	2015 DHS (Survey) [DHS Direct]
	19	2014.9	2016 DHS (Survey) [DHS Direct]
	19	2016.1	2017 DHS (Survey) [DHS Direct]
	19	2017.2	2018 DHS (Survey) [DHS Direct]
	19	2018	2019 DHS (Survey) [DHS Direct]
	2	2019.5	2020-2021 MIS (Survey) [other DHS Direct]
Serbia	12	2021.5	
	12	2021.5	Register (Register) [VR]
Seychelles	49	2017.5	
	1	2010.2	2010 Census (Census) [Others Direct]
	48	2017.5	Register (Register) [VR]
Sierra Leone	69	2018	
	15	2006.9	2008 DHS (Survey) [DHS Direct]
	18	2012.1	2013 DHS (Survey) [DHS Direct]
	2	2015.1	2016 MIS (Survey) [other DHS Direct]
	15	2016	2017 MICS (Survey) [other DHS Direct]
	19	2018	2019 DHS (Survey) [DHS Direct]
Singapore	62	2022.5	

Continued on next page

Table 17 – continued from previous page

Country/Area	# obs.	Most recent obs. year	Data series name [source type]
	62	2022.5	Register (Register) [VR]
Slovakia	127	2021.5	
	65	2014.5	Estimates (Estimate) [VR]
	62	2021.5	Register (Register) [VR]
Slovenia	75	2021.5	
	36	2019.5	Estimates (Estimate) [VR]
	39	2021.5	Register (Register) [VR]
Solomon Islands			
Somalia	38	2017	
	19	2005.2	2006 MICS (Survey) [other DHS Direct]
	19	2017	2018-2019 SHDS (Survey) [Others Direct]
South Africa	49	2021.5	
	19	1996.9	1998 DHS (Survey) [DHS Direct]
	16	2015.2	2016 DHS (Survey) [DHS Direct]
	14	2021.5	Register (Register) [VR]
South Sudan	19	2008.9	
	19	2008.9	2010 MICS-HHS (Survey) [other DHS Direct]
Spain	144	2021.5	
	72	2021.5	Estimates (Estimate) [VR]
	72	2021.5	Register (Register) [VR]
Sri Lanka	87	2015.5	
	12	1974	1975 WFS (Survey) [other DHS Direct]
	18	1985.6	1987 DHS (Survey) [DHS Direct]
	57	2015.5	Register (Register) [VR]
State of Palestine	58	2018.5	
	19	2009	2010 FHS-MICS (Survey) [other DHS Direct]
	19	2012.6	2014 MICS (Survey) [other DHS Direct]
	19	2018.5	2019-2020 MICS (Survey) [other DHS Direct]
	1	2007.5	Register (Register) [VR]
Sudan	60	2013.3	
	8	1977.5	1978-1979 WFS (Survey) [other DHS Direct]
	14	1988.6	1989-1990 DHS (Survey) [DHS Direct]
	19	2009.2	2010 MICS-HHS (Survey) [other DHS Direct]
	19	2013.3	2014 MICS (Survey) [other DHS Direct]
Suriname	55	2021.5	
	19	2016.9	2018 MICS (Survey) [other DHS Direct]
	36	2021.5	Register (Register) [VR]
Sweden	143	2022.5	
	73	2022.5	Estimates (Estimate) [VR]
	70	2021.5	Register (Register) [VR]
Switzerland	138	2022.5	
	71	2022.5	Estimates (Estimate) [VR]
	67	2019.5	Register (Register) [VR]
Syrian Arab Republic	52	2000.4	
	14	1977	1978 WFS (Survey) [other DHS Direct]

Continued on next page

Table 17 – continued from previous page

Country/Area	# obs.	Most recent obs. year	Data series name [source type]
	19	1992	1993 MCHS (Survey) [other DHS Direct]
	19	2000.4	2001 FHS (Survey) [other DHS Direct]
Tajikistan	27	2016.2	
	14	2011.1	2012 DHS (Survey) [DHS Direct]
	13	2016.2	2017 DHS (Survey) [DHS Direct]
Thailand	61	2021.5	
	13	1973.8	1975 WFS (Survey) [other DHS Direct]
	19	1985.8	1987 DHS (Survey) [DHS Direct]
	29	2021.5	Register (Register) [VR]
Timor-Leste	38	2015.3	
	19	2008.4	2009-2010 DHS (Survey) [DHS Direct]
	19	2015.3	2016 DHS (Survey) [DHS Direct]
Togo	78	2016.3	
	19	1987.2	1988 DHS (Survey) [DHS Direct]
	19	1996.8	1998 DHS (Survey) [DHS Direct]
	19	2012.6	2013-2014 DHS (Survey) [DHS Direct]
	19	2016.2	2017 MICS (Survey) [other DHS Direct]
	2	2016.3	2017 MIS (Survey) [other DHS Direct]
Tonga	5	2000.5	
	5	2000.5	Register (Register) [VR]
Trinidad and Tobago	77	2009.5	
	11	1970	1977 WFS (Survey) [other DHS Direct]
	19	1986	1987 DHS (Survey) [DHS Direct]
	47	2009.5	Register (Register) [VR]
Tunisia	113	2016.8	
	14	1977	1978 WFS (Survey) [other DHS Direct]
	17	1987.1	1988 DHS (Survey) [DHS Direct]
	19	1993.5	1994-1995 MCHS (Survey) [other DHS Direct]
	18	2000	2001 FHS (Survey) [other DHS Direct]
	18	2010.6	2011-2012 MICS (Survey) [other DHS Direct]
	16	2016.8	2018 MICS (Survey) [other DHS Direct]
	11	1986.5	Register (Register) [VR]
Türkiye	126	2021.5	
	14	1977	1978 WFS (Survey) [other DHS Direct]
	19	1992.2	1993 DHS (Survey) [DHS Direct]
	19	1997.2	1998 DHS (Survey) [DHS Direct]
	19	2002.7	2003 DHS (Survey) [other DHS Direct]
	19	2007.3	2008 DHS (Survey) [other DHS Direct]
	2	2001.4	2013 NDHS (Survey) [Indirect]
	19	2017.4	2018 NDHS (Survey) [other DHS Direct]
	15	2021.5	Register (Register) [VR]
Turkmenistan	30	2018	
	17	2014.4	2015-2016 MICS (Survey) [other DHS Direct]
	13	2018	2019 MICS (Survey) [other DHS Direct]
Uganda	130	2017.5	

Continued on next page

Table 17 – continued from previous page

Country/Area	# obs.	Most recent obs. year	Data series name [source type]
	18	1987.4	1988-1989 DHS (Survey) [DHS Direct]
	18	1993.9	1995 DHS (Survey) [DHS Direct]
	19	1999.5	2000-2001 DHS (Survey) [DHS Direct]
	19	2005.1	2006 DHS (Survey) [DHS Direct]
	15	2008.5	2009 MIS (Survey) [other DHS Direct]
	18	2010.2	2011 DHS (Survey) [DHS Direct]
	2	2013.5	2014-2015 MIS (Survey) [other DHS Direct]
	19	2015.2	2016 DHS (Survey) [DHS Direct]
	2	2017.5	2018-2019 MIS (Survey) [other DHS Direct]
Ukraine	73	2018.5	
	55	2013.5	Estimates (Estimate) [VR]
	18	2018.5	Register (Register) [VR]
United Arab Emirates	1	1980.5	
	1	1980.5	Register (Register) [VR]
United Kingdom	118	2021.5	
	71	2020.5	Estimates (Estimate) [VR]
	47	2021.5	Register (Register) [VR]
United Republic of Tanzania	137	2016.2	
	19	1990.5	1991-1992 DHS (Survey) [DHS Direct]
	19	1995.2	1996 DHS (Survey) [DHS Direct]
	19	1998.3	1999 RCHS (Survey) [DHS Direct]
	19	2003.5	2004-2005 DHS (Survey) [DHS Direct]
	19	2006.5	2007-2008 AIS (Survey) [other DHS Direct]
	19	2008.7	2010 DHS (Survey) [DHS Direct]
	2	2010.7	2011-2012 AIS (Survey) [other DHS Direct]
	19	2014.4	2015-2016 DHS (Survey) [DHS Direct]
	2	2016.2	2017 MIS (Survey) [other DHS Direct]
United States of America	144	2022.5	
	72	2021.5	Estimates (Estimate) [VR]
	72	2022.5	Register (Register) [VR]
Uruguay	35	2021.5	
	35	2021.5	Register (Register) [VR]
Uzbekistan	34	2022.5	
	13	1995.1	1996 DHS (Survey) [DHS Direct]
	15	2020.5	2021-2022 MICS (Survey) [other DHS Direct]
	6	2022.5	Register (Register) [VR]
Vanuatu			
Venezuela (Bolivarian Republic of)	65	2019.5	
	14	1976	1977 WFS (Survey) [other DHS Direct]
	51	2019.5	Register (Register) [VR]
Viet Nam	70	2019.5	

Continued on next page

Table 17 – continued from previous page

Country/Area	# obs.	Most recent obs. year	Data series name [source type]
	17	1996.2	1997 DHS (Survey) [DHS Direct]
	15	2001.4	2002 DHS (Survey) [DHS Direct]
	19	2012.6	2013-2014 MICS (Survey) [other DHS Direct]
	19	2019.5	2020-2021 MICS (Survey) [other DHS Direct]
Western Sahara			
Yemen	76	2012.3	
	19	1990.5	1991-1992 DHS (Survey) [DHS Direct]
	19	2001.5	2003 FHS (Survey) [other DHS Direct]
	19	2005.2	2006 MICS (Survey) [other DHS Direct]
	19	2012.3	2013 DHS (Survey) [DHS Direct]
Zambia	114	2017.3	
	19	1990.7	1992 DHS (Survey) [DHS Direct]
	19	1995.3	1996 DHS (Survey) [DHS Direct]
	19	2000.6	2001-2002 DHS (Survey) [DHS Direct]
	19	2006	2007 DHS (Survey) [DHS Direct]
	19	2012.5	2013-2014 DHS (Survey) [DHS Direct]
	19	2017.3	2018 DHS (Survey) [DHS Direct]
Zimbabwe	170	2017.7	
	18	1987.4	1988 DHS (Survey) [DHS Direct]
	19	1993.2	1994 DHS (Survey) [DHS Direct]
	19	1998.3	1999 DHS (Survey) [DHS Direct]
	19	2004.4	2005-2006 DHS (Survey) [DHS Direct]
	19	2007.9	2009 MICS (Survey) [other DHS Direct]
	19	2009.5	2010-2011 DHS (Survey) [DHS Direct]
	19	2012.7	2014 MICS (Survey) [other DHS Direct]
	19	2014.3	2015 DHS (Survey) [DHS Direct]
	19	2017.7	2019 MICS (Survey) [other DHS Direct]

Table 18: **Age-specific fertility rate data sources for age group 15–19, by country.** For each country, the total number of observations and the most recent reference year are shown after the country name. For each country-specific data series, the number of observations and the most recent reference year within that series are shown before each data series name. The source type that each data series falls in is shown in parentheses after each data series name. Countries without data are shown in grey rows (if applicable). Only countries/areas with population size above 90,000 by mid-2024 are shown.

Country/Area	# obs.	Most recent obs. year	Data series name [source type]
Afghanistan	61	2021.5	
	2	1973	1972-1974 NDFGS (Survey) [Indirect]
	2	1979	1979 Census (Census) [Indirect]
	2	2003	2003 MICS (Survey) [Indirect]
	2	2006.3	2006 HS (Survey) [Indirect]
	17	2007.7	2007-2008 NRVA (Survey) [Indirect]
	1	2010.6	2010-2011 MICS (Survey) [Indirect]
	16	2009.1	2010 DHS Special (Survey) [other DHS Direct]
	1	2011.2	2011-2012 NRVA (Survey) [Indirect]
	16	2014.3	2015 DHS (Survey) [DHS Direct]
	1	2017	2018 AHS (Survey) [Others Direct]
	1	2021.5	2022-2023 MICS (Survey) [other DHS Direct]
Albania	119	2021.5	
	54	2008.5	Estimates (Estimate) [VR]
	65	2021.5	Register (Register) [VR]
Algeria	97	2019.5	
	1	1970	1970-1971 EF (Survey) [Others Direct]
	1	1984.5	1986-1987 ENF (Survey) [other DHS Direct]
	1	1986.7	1987 Census (Census) [Others Direct]
	16	1991	1992 MCH (Survey) [other DHS Direct]
	1	1998	1998 Census (Census) [Others Direct]
	16	2001.3	2002 MCH (Survey) [other DHS Direct]
	1	2005.8	2006 MICS (Survey) [other DHS Direct]
	1	2007.8	2008 Census (Census) [Others Direct]
	16	2011.5	2012-2013 MICS (Survey) [other DHS Direct]
	16	2017.5	2018-2019 MICS (Survey) [other DHS Direct]
	1	2015.5	Estimates (Estimate) [VR]
	26	2019.5	Register (Register) [VR]
Angola	36	2014.5	
	1	1996.3	1996 MICS (Survey) [other DHS Direct]
	1	2001	2001 MICS (Survey) [other DHS Direct]
	2	2005.6	2006-2007 MIS (Survey) [other DHS Direct]
	16	2009.7	2011 MIS (Survey) [other DHS Direct]
	16	2014.5	2015-2016 DHS (Survey) [DHS Direct]
Antigua and Barbuda	40	2020.5	
	2	2010.9	2011 Census (Census) [Others Direct]
	38	2020.5	Register (Register) [VR]
Argentina	58	2022.5	
	1	2010.9	2011-2012 MICS (Survey) [other DHS Direct]

Continued on next page

Table 18 – continued from previous page

Country/Area	# obs.	Most recent obs. year	Data series name [source type]
	57	2022.5	Register (Register) [VR]
Armenia	173	2021.5	
	16	1999.4	2000 DHS (Survey) [DHS Direct]
	16	2004.3	2005 DHS (Survey) [DHS Direct]
	16	2009.4	2010 DHS (Survey) [DHS Direct]
	16	2014.6	2015 DHS (Survey) [DHS Direct]
	47	2009.5	Estimates (Estimate) [VR]
	62	2021.5	Register (Register) [VR]
Aruba	54	2021.5	
	20	2000.5	Estimates (Estimate) [VR]
	34	2021.5	Register (Register) [VR]
Australia	131	2022.5	
	58	2007.5	Estimates (Estimate) [VR]
	73	2022.5	Register (Register) [VR]
Austria	140	2021.5	
	69	2019.5	Estimates (Estimate) [VR]
	71	2021.5	Register (Register) [VR]
Azerbaijan	120	2022.5	
	1	1999.9	2001 RHS (Survey) [other DHS Direct]
	16	2005.2	2006 DHS (Survey) [DHS Direct]
	1	2010.2	2011 DHS-NS (Survey) [other DHS Direct]
	45	2005.5	Estimates (Estimate) [VR]
	57	2022.5	Register (Register) [VR]
Bahamas	6	2012.5	
	2	2009.9	2010 Census (Census) [Others Direct]
	4	2012.5	Register (Register) [VR]
Bahrain	70	2020.5	
	8	1989	1989 CHS (Survey) [other DHS Direct]
	5	1994	1995 FHS (Survey) [other DHS Direct]
	13	2009.5	Estimates (Estimate) [VR]
	44	2020.5	Register (Register) [VR]
Bangladesh	197	2022	
	1	1961	1961-1962 DSEP (Survey) [Others Direct]
	2	1962.5	1962-1965 PGEE (Survey) [Others Direct]
	2	1974	1974 RSFM (Survey) [Others Direct]
	3	1975.5	1975-1976 WFS (Survey) [other DHS Direct]
	2	1980	1980 BLDS (Survey) [Indirect]
	1	1982.9	1983-1984 CPS (Survey) [Others Direct]
	2	1985.1	1985-1986 CPS (Survey) [Indirect]
	17	1988.6	1988-1989 FS (Survey) [Indirect]
	6	1988.8	1989 CPS (Survey) [Indirect]
	1	1991	1991 CPS (Survey) [Others Direct]
	11	1992.5	1993-1994 DHS (Survey) [DHS Direct]
	11	1995.5	1996-1997 DHS (Survey) [DHS Direct]
	13	1998.5	1999-2000 DHS (Survey) [DHS Direct]
	16	1999.7	2001 DHS Special (Survey) [other DHS Direct]

Continued on next page

Table 18 – continued from previous page

Country/Area	# obs.	Most recent obs. year	Data series name [source type]
	16	2002.7	2004 DHS (Survey) [DHS Direct]
	1	2005.6	2006 MICS (Survey) [other DHS Direct]
	16	2005.9	2007 DHS (Survey) [DHS Direct]
	1	2009	2010 DHS Special (Survey) [DHS Direct]
	16	2010.2	2011 DHS (Survey) [DHS Direct]
	1	2011.6	2012-2013 MICS (Survey) [other DHS Direct]
	16	2013.2	2014 DHS (Survey) [DHS Direct]
	16	2016.5	2017-2018 DHS (Survey) [DHS Direct]
	16	2017.7	2019 MICS (Survey) [other DHS Direct]
	1	2020.8	2022 DHS (Survey) [DHS Direct]
	9	2022	SVRS (SRS) [VR]
Barbados	49	2007.5	
	1	1980.5	1980-1981 CPS (Survey) [Others Direct]
	48	2007.5	Register (Register) [VR]
Belarus	115	2018.5	
	59	2018.5	Estimates (Estimate) [VR]
	56	2018.5	Register (Register) [VR]
Belgium	141	2021.5	
	69	2020.5	Estimates (Estimate) [VR]
	72	2021.5	Register (Register) [VR]
Belize	52	2022.5	
	1	1997.4	1999 FHS (Survey) [Others Direct]
	1	2010	2011 MICS (Survey) [other DHS Direct]
	16	2014.4	2015-2016 MICS (Survey) [other DHS Direct]
	2	2022.5	Estimates (Estimate) [VR]
	32	2021.5	Register (Register) [VR]
Benin	134	2020	
	2	1961.1	1961 ED (Survey) [Indirect]
	17	1980.9	1981-1982 WFS (Survey) [Indirect]
	1	1991.6	1992 Census (Census) [Others Direct]
	16	1995	1996 DHS (Survey) [DHS Direct]
	16	2000.3	2001 DHS (Survey) [DHS Direct]
	2	2001.6	2002 Census (Census) [Indirect]
	16	2005.3	2006 DHS (Survey) [DHS Direct]
	16	2010.6	2011-2012 DHS (Survey) [DHS Direct]
	16	2012.8	2014 MICS (Survey) [other DHS Direct]
	16	2016.5	2017-2018 DHS (Survey) [DHS Direct]
	16	2020	2021 MICS (Survey) [other DHS Direct]
Bhutan	8	2021.5	
	1	2006.8	2007 LSS (Survey) [Others Direct]
	1	2009.4	2010 MICS (Survey) [other DHS Direct]
	1	2011.8	2012 LSS (Survey) [Others Direct]
	1	2012.5	2012 NHS (Survey) [Others Direct]
	1	2016.9	2017 Census (Census) [Others Direct]
	3	2021.5	Register (Register) [VR]

Continued on next page

Table 18 – continued from previous page

Country/Area	# obs.	Most recent obs. year	Data series name [source type]
Bolivia (Plurinational State of)	85	2015	
	1	1976.2	1976 Census (Census) [Indirect]
	2	1980.3	1980 EDN (Survey) [Others Direct]
	16	1987.9	1989 DHS (Survey) [DHS Direct]
	2	1992.4	1992 Census (Census) [Indirect]
	16	1992.6	1994 DHS (Survey) [DHS Direct]
	15	1996	1998 DHS (Survey) [DHS Direct]
	1	2001.7	2001 Census (Census) [Indirect]
	14	2002.3	2003 DHS (Survey) [DHS Direct]
	16	2006.8	2008 DHS (Survey) [DHS Direct]
	1	2012.7	2012 Census (Census) [Indirect]
	1	2015	2016 DHS (Survey) [Others Direct]
Bosnia and Herzegovina	104	2019.5	
	41	1990.5	Estimates (Estimate) [VR]
	63	2019.5	Register (Register) [VR]
Botswana	33	2019.5	
	2	1971.2	1971 Census (Census) [Others Direct]
	2	1981.1	1981 Census (Census) [Others Direct]
	1	1983.8	1984 FHS (CPS) (Survey) [Others Direct]
	1	1986	1987 BDS (Survey) [Others Direct]
	5	1987.3	1988 DHS (Survey) [DHS Direct]
	2	1991.1	1991 Census (Census) [Others Direct]
	1	2001.1	2001 Census (Census) [Others Direct]
	1	2006	2006 BDS (Survey) [Others Direct]
	5	2006.4	2007 FHS-MICS (Survey) [other DHS Direct]
	2	2011.1	2011 Census (Census) [Others Direct]
	1	2017.2	2017 BDS (Survey) [Others Direct]
	10	2019.5	Register (Register) [VR]
Brazil	54	2022.5	
	1	1980.7	1980 Census (Census) [Indirect]
	13	1985	1986 FP-MCH DHS (Survey) [DHS Direct]
	1	1991.7	1991 Census (Census) [Indirect]
	16	1994.8	1996 DHS (Survey) [DHS Direct]
	1	2000.6	2000 Census (Census) [Indirect]
	22	2022.5	Register (Register) [VR]
Brunei Darussalam	35	2021.5	
	35	2021.5	Register (Register) [VR]
Bulgaria	143	2022.5	
	71	2021.5	Estimates (Estimate) [VR]
	72	2022.5	Register (Register) [VR]
Burkina Faso	101	2020.2	
	3	1976	1976 PES (PES) [Indirect]
	1	1985.5	1985 Census (Census) [Others Direct]

Continued on next page

Table 18 – continued from previous page

Country/Area	# obs.	Most recent obs. year	Data series name [source type]
	1	1989	1991 ED (Survey) [Indirect]
	16	1991.6	1993 DHS (Survey) [DHS Direct]
	1	1993.4	1996 Census (Census) [Indirect]
	16	1997.5	1998-1999 DHS (Survey) [DHS Direct]
	16	2002.2	2003 DHS (Survey) [DHS Direct]
	2	2006.5	2006 Census (Census) [Indirect]
	2	2005.3	2006 MICS (Survey) [Indirect]
	16	2009.2	2010 DHS (Survey) [DHS Direct]
	1	2013.3	2014 MIS (Survey) [other DHS Direct]
	1	2013.8	2014 PMA-R1 (Panel) [other DHS Direct]
	2	2014.2	2015 PMA-R2 (Panel) [other DHS Direct]
	2	2015.3	2016 PMA-R3 (Panel) [other DHS Direct]
	2	2016.6	2017-2018 MIS (Survey) [other DHS Direct]
	1	2017	2017-2018 PMA-R5 (Panel) [other DHS Direct]
	1	2018	2018-2019 PMA-R6 (Panel) [other DHS Direct]
	1	2019.4	2019 Census (Census) [Others Direct]
	16	2020.2	2021 DHS (Survey) [DHS Direct]
Burundi	65	2015.5	
	1	1964.7	1965 PS (Survey) [Others Direct]
	1	1970.5	1970-1971 DS (Panel) [Indirect]
	1	1980	1980-1981 PES (PES) [Others Direct]
	1	1983	1983 EDCEA (Survey) [Others Direct]
	16	1985.9	1987 DHS (Survey) [DHS Direct]
	1	1990.1	1990 Census (Census) [Others Direct]
	9	2001	2002 Survey (Survey) [Others Direct]
	1	2008.1	2008 Census (Census) [Others Direct]
	16	2009.3	2010 DHS (Survey) [DHS Direct]
	2	2011.5	2012 MIS (Survey) [other DHS Direct]
	16	2015.5	2016 DHS (Survey) [DHS Direct]
Cabo Verde	33	2020.5	
	2	1979.9	1980 Census (Census) [Indirect]
	2	1990	1990 Census (Census) [Indirect]
	7	1997.9	1998 DHS-RHS (Survey) [DHS Direct]
	2	2000	2000 Census (Census) [Indirect]
	5	2004	2005 DHS-RHS (Survey) [DHS Direct]
	2	2010	2010 Census (Census) [Indirect]
	13	2020.5	Register (Register) [VR]
Cambodia	97	2020.4	
	1	1959	1959 Survey (Survey) [Others Direct]
	2	1961.8	1962 Census (Census) [Others Direct]
	2	1993	1995 KAP (Survey) [Others Direct]
	2	1996	1996 Survey (Survey) [Indirect]
	2	1997.7	1998 Census (Census) [Indirect]
	1	1996.9	1998 DHS-NS (Survey) [other DHS Direct]
	16	1998.8	2000 DHS (Survey) [DHS Direct]
	16	2004.5	2005 DHS (Survey) [DHS Direct]

Continued on next page

Table 18 – continued from previous page

Country/Area	# obs.	Most recent obs. year	Data series name [source type]
	16	2009.3	2010 DHS (Survey) [DHS Direct]
	16	2013.2	2014 DHS (Survey) [DHS Direct]
	16	2020.4	2021-2022 DHS (Survey) [DHS Direct]
	7	1993.5	Estimates (Estimate) [VR]
Cameroon	113	2017.2	
	1	1975.8	1976 Census (Census) [Indirect]
	14	1977	1978 WFS (Survey) [other DHS Direct]
	1	1986.8	1987 Census (Census) [Others Direct]
	16	1990	1991 DHS (Survey) [DHS Direct]
	16	1996.8	1998 DHS (Survey) [DHS Direct]
	16	2002.9	2004 DHS (Survey) [DHS Direct]
	1	2005.4	2005 Census (Census) [Indirect]
	16	2009.8	2011 DHS (Survey) [DHS Direct]
	16	2013	2014 MICS (Survey) [other DHS Direct]
	16	2017.2	2018 DHS (Survey) [DHS Direct]
Canada	142	2021.5	
	70	2019.5	Estimates (Estimate) [VR]
	72	2021.5	Register (Register) [VR]
Central African Republic	36	2018	
	1	1959	1959-1960 Survey (Survey) [Others Direct]
	1	1975.5	1975 Census (Census) [Indirect]
	1	1988.4	1988 Census (Census) [Indirect]
	16	1993.5	1994-1995 DHS (Survey) [DHS Direct]
	1	2010.2	2010 MICS (Survey) [other DHS Direct]
	16	2018	2019 MICS (Survey) [other DHS Direct]
Chad	63	2018	
	1	1963.8	1963-1964 PS (Survey) [Others Direct]
	1	1992.8	1993 Census (Census) [Indirect]
	16	1995.7	1996-1997 DHS (Survey) [DHS Direct]
	14	2003.2	2004 DHS (Survey) [DHS Direct]
	1	2007.8	2010 MICS (Survey) [other DHS Direct]
	14	2013.5	2014-2015 DHS (Survey) [DHS Direct]
	16	2018	2019 MICS (Survey) [other DHS Direct]
Chile	82	2023.5	
	2	1982.3	1982 Census (Census) [Others Direct]
	2	1992.3	1992 Census (Census) [Others Direct]
	2	2002.3	2002 Census (Census) [Others Direct]
	2	2017.3	2017 Census (Census) [Others Direct]
	74	2023.5	Register (Register) [VR]
China	199	2020.8	
	1	1982	1982 Census (Census) [Others Direct]
	32	1982	1982 One-per-Thousand FS (Survey) [Others Direct]
	1	1987	1987 One-percent Survey (Survey) [Others Direct]
	8	1988	1988 Two-per-Thousand FS (Survey) [Others Direct]
	16	1990	1990 Census (Census) [Indirect]

Continued on next page

Table 18 – continued from previous page

Country/Area	# obs.	Most recent obs. year	Data series name [source type]
	3	1992.2	1992 Fertility Survey (Survey) [Others Direct]
	1	1995.2	1995 One-percent Survey (Survey) [Others Direct]
	7	1997	1997 FPRHS (Survey) [Others Direct]
	27	2000.3	2000 Census (Census) [Indirect]
	10	2001	2001 FPRHS (Survey) [Others Direct]
	1	2005.3	2005 One-percent Survey (Survey) [Others Direct]
	6	2006	2006 FPRHS (Survey) [Others Direct]
	1	2010.3	2010 Census (Census) [Others Direct]
	1	2015.5	2015 One-percent Survey (Survey) [Others Direct]
	16	2017	2017 NFS (Survey) [Indirect]
	1	2018.3	2018 NHSS (Survey) [Others Direct]
	1	2020	2020 Census (Census) [Others Direct]
	24	2020.8	Annual APC (Survey) [Others Direct]
	42	2005.5	Estimates (Estimate) [VR]
China, Hong Kong SAR	100	2022.5	
	43	2007.5	Estimates (Estimate) [VR]
	57	2022.5	Register (Register) [VR]
Macao, SAR of China	68	2022.5	
	1	1991.2	1991 Census (Census) [Others Direct]
	2	2011.1	2011 Census (Census) [Others Direct]
	1	2016.1	2016 ICS (by-census) (Survey) [Others Direct]
	64	2022.5	Register (Register) [VR]
China, Taiwan Province of China	127	2022.5	
	54	2019.5	Estimates (Estimate) [VR]
	73	2022.5	Register (Register) [VR]
Colombia	85	2022.5	
	1	1985.3	1985 Census (Census) [Indirect]
	8	1985.3	1986 DHS (Survey) [DHS Direct]
	1	1993.3	1993 Census (Census) [Indirect]
	13	1993.8	1995 DHS (Survey) [DHS Direct]
	12	1998.8	2000 DHS (Survey) [DHS Direct]
	2	2005.7	2005 Census (Census) [Others Direct]
	14	2003.6	2005 DHS (Survey) [DHS Direct]
	16	2008.9	2010 DHS (Survey) [DHS Direct]
	18	2022.5	Register (Register) [VR]
Comoros	38	2017	
	2	1966.1	1966 Census (Census) [Others Direct]
	1	1980.2	1980 Census (Census) [Others Direct]
	16	1994.8	1996 DHS (Survey) [DHS Direct]
	1	2003.1	2003 Census (Census) [Others Direct]
	16	2011.3	2012 DHS (Survey) [DHS Direct]
	2	2017	2017 Census (Census) [Others Direct]

Continued on next page

Table 18 – continued from previous page

Country/Area	# obs.	Most recent obs. year	Data series name [source type]
Republic of the Congo	51	2013.5	
	1	1960	1960-1961 DS (Survey) [Others Direct]
	1	1973.6	1974 Census (Census) [Indirect]
	1	1984	1984 Census (Census) [Indirect]
	16	2004.2	2005 DHS (Survey) [DHS Direct]
	16	2010.4	2011-2012 DHS (Survey) [DHS Direct]
	16	2013.5	2014-2015 MICS (Survey) [other DHS Direct]
Costa Rica	46	2022.5	
	16	1991.5	1993 RHS (Survey) [other DHS Direct]
	30	2022.5	Register (Register) [VR]
Côte d'Ivoire	149	2021.4	
	1	1963	1962-1964 Survey (Survey) [Others Direct]
	2	1978.5	1978-1979 EDPR (Panel) [Indirect]
	17	1979.5	1980-1981 WFS (Survey) [Indirect]
	1	1984.7	1988 Census (Census) [Indirect]
	16	1993.2	1994 DHS (Survey) [DHS Direct]
	16	1997.5	1998-1999 DHS (Survey) [DHS Direct]
	31	2005.2	2005 EIS (Survey) [Indirect]
	1	2005.7	2006 MICS (Survey) [other DHS Direct]
	16	2010.7	2011-2012 DHS (Survey) [DHS Direct]
	1	2013.7	2014 Census (Census) [Others Direct]
	16	2015.1	2016 MICS (Survey) [other DHS Direct]
	2	2016.8	2017 PMA-R1 (Panel) [other DHS Direct]
	1	2017.6	2018 PMA-R2 (Panel) [other DHS Direct]
	1	2021.4	2021 Census (Census) [Others Direct]
	16	2020.3	2021 DHS (Survey) [DHS Direct]
	11	2010.5	Estimates (Estimate) [VR]
Croatia	125	2023.5	
	70	2020.5	Estimates (Estimate) [VR]
	55	2023.5	Register (Register) [VR]
Cuba	44	2021.5	
	44	2021.5	Register (Register) [VR]
Curaçao	38	2019.5	
	1	1960	1960 Census (Census) [Others Direct]
	1	1972	1972 Census (Census) [Others Direct]
	1	1981	1981 Census (Census) [Others Direct]
	1	1992	1992 Census (Census) [Others Direct]
	1	2001	2001 Census (Census) [Others Direct]
	1	2010.7	2011 Census (Census) [Others Direct]
	18	2009.5	Estimates (Estimate) [VR]
	14	2019.5	Register (Register) [VR]
Cyprus	103	2021.5	
	31	2007.5	Estimates (Estimate) [VR]
	72	2021.5	Register (Register) [VR]
Czechia	145	2022.5	

Continued on next page

Table 18 – continued from previous page

Country/Area	# obs.	Most recent obs. year	Data series name [source type]
	72	2021.5	Estimates (Estimate) [VR]
	73	2022.5	Register (Register) [VR]
Dem. People's Republic of Korea	3	2008.5	
	1	2008.3	2008 Census (Census) [Others Direct]
	1	1950.5	Estimates (Estimate) [VR]
	1	2008.5	Register (Register) [VR]
Democratic Republic of the Congo	50	2016.7	
	1	1956.2	1955-1957 SPS (Survey) [Others Direct]
	1	1994.8	1995 MICS (Survey) [Indirect]
	1	2001	2001 MICS (Survey) [other DHS Direct]
	16	2005.8	2007 DHS (Survey) [DHS Direct]
	15	2012.4	2013-2014 DHS (Survey) [DHS Direct]
	16	2016.7	2017-2018 MICS (Survey) [other DHS Direct]
Denmark	146	2022.5	
	73	2022.5	Estimates (Estimate) [VR]
	73	2022.5	Register (Register) [VR]
Djibouti	37	2011.1	
	1	1991	1991 ICS (Survey) [Indirect]
	3	2000	2002 EDAM-IS (Survey) [Indirect]
	16	2001.4	2002 FHS (Survey) [other DHS Direct]
	1	2009.7	2012 EDAM-IS (Survey) [Others Direct]
	16	2011.1	2012 FHS (Survey) [other DHS Direct]
Dominican Republic	122	2022.5	
	15	1978	1980 WFS (Survey) [other DHS Direct]
	1	1981.9	1981 Census (Census) [Indirect]
	1	1983	1983 CPS (Survey) [Others Direct]
	14	1995.3	1996 DHS (Survey) [DHS Direct]
	16	1998.3	1999 DHS (Survey) [DHS Direct]
	15	2000.3	2002 DHS (Survey) [DHS Direct]
	1	2005	2006 ENHOGAR-MICS (Survey) [other DHS Direct]
	9	2005.9	2007 DHS (Survey) [DHS Direct]
	1	2010.9	2010 Census (Census) [Others Direct]
	14	2012.2	2013 DHS (Survey) [DHS Direct]
	15	2018	2019 MICS (Survey) [other DHS Direct]
	20	2022.5	Register (Register) [VR]
Ecuador	72	2021.5	
	2	1982.9	1982 Census (Census) [Others Direct]
	2	1988.1	1989 ENDEMAIN-RHS (Survey) [other DHS Direct]
	2	1990.9	1990 Census (Census) [Others Direct]
	14	1993.1	1994 ENDEMAIN-RHS (Survey) [other DHS Direct]
	14	1998	1999 ENDEMAIN-RHS (Survey) [other DHS Direct]
	2	2001.9	2001 Census (Census) [Others Direct]

Continued on next page

Table 18 – continued from previous page

Country/Area	# obs.	Most recent obs. year	Data series name [source type]
	14	2002.8	2004 ENDEMAIN-RHS (Survey) [other DHS Direct]
	1	2010.9	2010 Census (Census) [Others Direct]
	21	2021.5	Register (Register) [VR]
Egypt	164	2020	
	16	1979	1980 WFS (Survey) [other DHS Direct]
	16	1987.4	1988 DHS (Survey) [DHS Direct]
	16	1989.6	1991 PAPCHILD (Survey) [other DHS Direct]
	16	1991.5	1992 DHS (Survey) [DHS Direct]
	16	1994.5	1995 DHS (Survey) [DHS Direct]
	1	1996.5	1997 DHS (Survey) [DHS Direct]
	1	1997.4	1998 DHS Interim (Survey) [other DHS Direct]
	16	1998.7	2000 DHS (Survey) [DHS Direct]
	16	2001.9	2003 DHS Interim (Survey) [other DHS Direct]
	16	2003.9	2005 DHS (Survey) [DHS Direct]
	16	2006.8	2008 DHS (Survey) [DHS Direct]
	16	2012.9	2014 DHS (Survey) [DHS Direct]
	1	2020	2021 FHS (Survey) [Others Direct]
	1	2019.5	Register (Register) [VR]
El Salvador	125	2018.5	
	2	1983.9	1985 DHS (Survey) [DHS Direct]
	2	1986.9	1988 FESAL (Survey) [other DHS Direct]
	2	1992.8	1992 Census (Census) [Others Direct]
	2	1991.9	1993 FESAL (Survey) [other DHS Direct]
	16	1997.1	1998 FESAL (Survey) [other DHS Direct]
	13	2001.7	2002-2003 FESAL (Survey) [other DHS Direct]
	2	2007.4	2007 Census (Census) [Others Direct]
	16	2007	2008 FESAL (Survey) [other DHS Direct]
	16	2012.9	2014 MICS (Survey) [other DHS Direct]
	54	2018.5	Register (Register) [VR]
Equatorial Guinea	9	2010.3	
	2	1983	1983 Census (Census) [Indirect]
	1	1994	1994 Census (Census) [Others Direct]
	1	2001.6	2001 Census (Census) [Others Direct]
	5	2010.3	2011 DHS (Survey) [DHS Direct]
Eritrea	48	2008.8	
	16	1994.4	1995 DHS (Survey) [DHS Direct]
	16	2000.9	2002 DHS (Survey) [DHS Direct]
	16	2008.8	2010 PHS (Survey) [other DHS Direct]
Estonia	133	2021.5	
	61	2019.5	Estimates (Estimate) [VR]
	72	2021.5	Register (Register) [VR]
Eswatini	58	2013.2	
	2	1965.9	1966 Census (Census) [Others Direct]
	1	1976.2	1976 Census (Census) [Others Direct]
	2	1986.1	1986 Census (Census) [Indirect]
	1	1986	1988 FHS (Survey) [other DHS Direct]

Continued on next page

Table 18 – continued from previous page

Country/Area	# obs.	Most recent obs. year	Data series name [source type]
	1	1990	1991 DHS (Survey) [Indirect]
	1	1996.9	1997 Census (Census) [Others Direct]
	16	2005.3	2006-2007 DHS (Survey) [DHS Direct]
	1	2006.8	2007 Census (Census) [Others Direct]
	16	2009.3	2010 MICS (Survey) [other DHS Direct]
	1	2012	2012 IS (Survey) [Others Direct]
	16	2013.2	2014 MICS (Survey) [other DHS Direct]
Ethiopia	121	2018.8	
	1	1965.5	1964-1967 NSS Rural (Survey) [Others Direct]
	1	1969	1968-1971 NSS Rural (Survey) [Indirect]
	1	1981	1981 DS (Panel) [Others Direct]
	1	1983.9	1984 Census (Census) [Indirect]
	8	1988	1990 FFS (Survey) [Indirect]
	1	1994.3	1994 Census (Census) [Indirect]
	1	1998	1998 MGHS (Survey) [Indirect]
	1	1998	1998 NHS (Survey) [Indirect]
	16	1998.7	2000 DHS (Survey) [DHS Direct]
	16	2004	2005 DHS (Survey) [DHS Direct]
	2	2006.9	2007 Census (Census) [Others Direct]
	16	2009.7	2011 DHS (Survey) [DHS Direct]
	16	2012.7	2014 MDHS (Survey) [Others Direct]
	1	2013.1	2014 PMA-R1 (Panel) [other DHS Direct]
	1	2013.8	2014 PMA-R2 (Panel) [other DHS Direct]
	1	2014.3	2015 PMA-R3 (Panel) [other DHS Direct]
	16	2014.8	2016 DHS (Survey) [DHS Direct]
	1	2015.3	2016 PMA-R4 (Panel) [other DHS Direct]
	2	2016.3	2017 PMA-R5 (Panel) [other DHS Direct]
	1	2017.5	2018 PMA-R6 (Panel) [other DHS Direct]
	16	2017.8	2019 DHS (Survey) [DHS Direct]
	1	2018.8	2019 PMA (Panel) [other DHS Direct]
Fiji	116	2019.7	
	3	1964.2	1966 Census (Census) [Indirect]
	12	1973	1974 WFS (Survey) [other DHS Direct]
	3	1974.2	1976 Census (Census) [Indirect]
	4	1986.2	1986 Census (Census) [Others Direct]
	3	1994.2	1996 Census (Census) [Indirect]
	3	2005.2	2007 Census (Census) [Indirect]
	3	2011.4	2013-2014 HH Listing (Census) [Indirect]
	16	2019.7	2021 MICS (Survey) [other DHS Direct]
	13	2013.5	Estimates (Estimate) [VR]
	56	2017.5	Register (Register) [VR]
Finland	146	2022.5	
	73	2022.5	Estimates (Estimate) [VR]
	73	2022.5	Register (Register) [VR]
France	145	2023.5	
	72	2021.5	Estimates (Estimate) [VR]

Continued on next page

Table 18 – continued from previous page

Country/Area	# obs.	Most recent obs. year	Data series name [source type]
	73	2023.5	Register (Register) [VR]
French Guiana	49	2021.5	
	49	2021.5	Register (Register) [VR]
French Polynesia	44	2021.5	
	44	2021.5	Register (Register) [VR]
Gabon	49	2019.3	
	1	1993	1993 Census (Census) [Others Direct]
	16	1999.3	2000 DHS (Survey) [DHS Direct]
	16	2010.7	2012 DHS (Survey) [DHS Direct]
	16	2019.3	2019-2021 DHS (Survey) [DHS Direct]
Gambia	66	2018.5	
	1	1972.8	1973 Census (Census) [Indirect]
	1	1972	1973 PES (PES) [Indirect]
	4	1982.8	1983 Census (Census) [Indirect]
	3	1988	1990 GCPFDS (Survey) [Others Direct]
	1	1990.6	1993-1994 HEHS (Survey) [Indirect]
	4	1992.8	1993 Census (Census) [Indirect]
	2	1999.9	2000 MICS (Survey) [Indirect]
	3	2001.2	2001 NSMCP (Survey) [Indirect]
	1	2002.8	2003 Census (Census) [Indirect]
	2	2005.1	2005-2006 MICS (Survey) [Indirect]
	1	2009.5	2010 MICS (Survey) [other DHS Direct]
	15	2011.7	2013 DHS (Survey) [DHS Direct]
	13	2016.7	2018 MICS (Survey) [other DHS Direct]
	15	2018.5	2019-2020 DHS (Survey) [DHS Direct]
Georgia	86	2022.5	
	1	1998.5	1999 RHS (Survey) [other DHS Direct]
	1	2004	2005 RHS (Survey) [other DHS Direct]
	32	2013.5	Estimates (Estimate) [VR]
	52	2022.5	Register (Register) [VR]
Germany	132	2021.5	
	66	2017.5	Estimates (Estimate) [VR]
	66	2021.5	Register (Register) [VR]
Ghana	230	2021.4	
	3	1959.8	1960 PES (PES) [Indirect]
	4	1968.8	1968-1969 DSS (Panel) [Indirect]
	2	1971.2	1971 Survey (Survey) [Others Direct]
	14	1978.2	1979-1980 WFS (Survey) [other DHS Direct]
	16	1986.8	1988 DHS (Survey) [DHS Direct]
	16	1992.4	1993 DHS (Survey) [DHS Direct]
	16	1997.5	1998 DHS (Survey) [DHS Direct]
	16	1999.7	2000 Census (Census) [Indirect]
	16	2002.2	2003 DHS (Survey) [DHS Direct]
	2	2005.7	2006 MICS (Survey) [other DHS Direct]
	31	2007.4	2007 DHS Special (Survey) [Indirect]
	16	2007.3	2008 DHS (Survey) [DHS Direct]

Continued on next page

Table 18 – continued from previous page

Country/Area	# obs.	Most recent obs. year	Data series name [source type]
	1	2010.3	2010 Census (Census) [Others Direct]
	16	2010.3	2011 MICS (Survey) [other DHS Direct]
	2	2013.2	2013 PMA-R1 (Panel) [other DHS Direct]
	16	2013.3	2014 DHS (Survey) [DHS Direct]
	2	2013.2	2014 PMA-R2 (Panel) [other DHS Direct]
	1	2013.8	2014 PMA-R3 (Panel) [other DHS Direct]
	1	2014.4	2015 PMA-R4 (Panel) [other DHS Direct]
	2	2015.4	2016 MIS (Survey) [other DHS Direct]
	2	2015.8	2016 PMA-R5 (Panel) [other DHS Direct]
	16	2016.4	2017-2018 MICS (Survey) [other DHS Direct]
	16	2016.1	2017 MHS (Survey) [other DHS Direct]
	2	2018.3	2019 MIS (Survey) [other DHS Direct]
	1	2021.4	2022 DHS (Survey) [DHS Direct]
Greece	118	2021.5	
	50	2009.5	Estimates (Estimate) [VR]
	68	2021.5	Register (Register) [VR]
Grenada	43	2018.5	
	43	2018.5	Register (Register) [VR]
Guadeloupe	45	2021.5	
	45	2021.5	Register (Register) [VR]
Guam	62	2018.5	
	3	1977.8	1980 Census (Census) [Indirect]
	3	1987.7	1990 Census (Census) [Indirect]
	1	1994	1994 Survey (Survey) [Others Direct]
	3	1997.8	2000 Census (Census) [Indirect]
	3	2007.7	2010 Census (Census) [Indirect]
	49	2018.5	Register (Register) [VR]
Guatemala	124	2021.5	
	13	1986.3	1987 DHS (Survey) [DHS Direct]
	1	1994.3	1994 Census (Census) [Indirect]
	16	1994.2	1995 DHS (Survey) [DHS Direct]
	16	1997.6	1998-1999 DHS Interim (Survey) [other DHS Direct]
	1	2002.9	2002 Census (Census) [Indirect]
	16	2001.1	2002 ENSMI (Survey) [other DHS Direct]
	61	2021.5	Register (Register) [VR]
Guinea	85	2020.1	
	1	1954.5	1954-1955 SDS (Survey) [Others Direct]
	1	1982.6	1983 Census (Census) [Indirect]
	2	1996.4	1996 Census (Census) [Indirect]
	16	1997.9	1999 DHS (Survey) [DHS Direct]
	16	2003.8	2005 DHS (Survey) [DHS Direct]
	16	2011.1	2012 DHS (Survey) [DHS Direct]
	1	2013.7	2014 Census (Census) [Others Direct]
	16	2015	2016 MICS (Survey) [other DHS Direct]
	14	2016.8	2018 DHS (Survey) [DHS Direct]
	2	2020.1	2021 MIS (Survey) [other DHS Direct]

Continued on next page

Table 18 – continued from previous page

Country/Area	# obs.	Most recent obs. year	Data series name [source type]
Guinea-Bissau	33	2017.5	
	1	2005.4	2006 MICS (Survey) [other DHS Direct]
	16	2012.9	2014 MICS (Survey) [other DHS Direct]
	16	2017.5	2018-2019 MICS (Survey) [other DHS Direct]
Guyana	92	2018.5	
	13	1973.9	1975 WFS (Survey) [other DHS Direct]
	1	1979.9	1980 Census (Census) [Others Direct]
	16	2004.1	2005 AIS (Survey) [other DHS Direct]
	16	2007.9	2009 DHS (Survey) [DHS Direct]
	1	2012.2	2012 Census (Census) [Others Direct]
	16	2013	2014 MICS (Survey) [other DHS Direct]
	16	2018.5	2019-2020 MICS (Survey) [other DHS Direct]
	13	1980.5	Register (Register) [VR]
Haiti	76	2015.6	
	11	1970	1977 WFS (Survey) [other DHS Direct]
	15	1998.8	2000 DHS (Survey) [DHS Direct]
	2	2003	2003 Census (Census) [Others Direct]
	16	2004.6	2005-2006 DHS (Survey) [DHS Direct]
	15	2010.7	2012 DHS (Survey) [DHS Direct]
	1	2011.9	2013 DHS-NS (Survey) [other DHS Direct]
	16	2015.6	2016-2017 DHS (Survey) [DHS Direct]
Honduras	39	2013.6	
	1	1988.4	1988 Census (Census) [Indirect]
	16	1994.9	1996 ENESF (Survey) [other DHS Direct]
	1	2001.6	2001 Census (Census) [Indirect]
	16	1999.9	2001 ENESF (Survey) [other DHS Direct]
	2	2013.6	2013 Census (Census) [Others Direct]
	3	1974.5	Register (Register) [VR]
Hungary	143	2021.5	
	71	2020.5	Estimates (Estimate) [VR]
	72	2021.5	Register (Register) [VR]
Iceland	134	2021.5	
	62	2021.5	Estimates (Estimate) [VR]
	72	2021.5	Register (Register) [VR]
India	97	2020.5	
	16	1991.5	1992-1993 NFHS (Survey) [DHS Direct]
	16	1997.9	1998-1999 NFHS (Survey) [DHS Direct]
	16	2004.8	2005-2006 NFHS (Survey) [DHS Direct]
	1	2007.5	2007-2008 DLHS (Survey) [Others Direct]
	16	2014.5	2015-2016 NFHS (Survey) [DHS Direct]
	16	2018.9	2019-2021 NFHS (Survey) [other DHS Direct]
	16	2020.5	SRS (SRS) [VR]
Indonesia	149	2020.5	
	1	1969.2	1971 Census (Census) [Indirect]
	2	1973.5	1976 SUPAS (Survey) [Indirect]
	2	1973.5	1976 WFS (Survey) [other DHS Direct]

Continued on next page

Table 18 – continued from previous page

Country/Area	# obs.	Most recent obs. year	Data series name [source type]
	1	1977.8	1980 Census (Census) [Indirect]
	1	1983	1985 IPS (Survey) [Indirect]
	16	1986.3	1987 CPS (Survey) [DHS Direct]
	1	1987.8	1990 Census (Census) [Indirect]
	16	1989.9	1991 DHS (Survey) [DHS Direct]
	16	1993.2	1994 DHS (Survey) [DHS Direct]
	16	1996.3	1997 DHS (Survey) [DHS Direct]
	1	1998	2000 Census (Census) [Indirect]
	16	2001.5	2002-2003 DHS (Survey) [DHS Direct]
	16	2006.3	2007 DHS (Survey) [DHS Direct]
	1	2009.9	2010 Census (Census) [Indirect]
	5	2011	2012 DHS Special (Survey) [other DHS Direct]
	16	2011	2012 SDKI (Survey) [Others Direct]
	1	2014	2015 SUPAS (Survey) [Indirect]
	14	2016.1	2017 DHS (Survey) [DHS Direct]
	5	2016.1	2017 YARHS DHS Special (Survey) [Indirect]
	1	1998	Annual SUSENAS (Survey) [Indirect]
	1	2020.5	Estimates (Estimate) [VR]
Iran (Islamic Republic of)	97	2021.5	
	1	1966.3	1966 Census (Census) [Others Direct]
	2	1974.5	1973-1976 PGS (Panel) [Others Direct]
	1	1976.3	1976 Census (Census) [Others Direct]
	15	1986.2	1986 Census (Census) [Indirect]
	1	1988	1988 PS (Survey) [Others Direct]
	1	1991.4	1991 Census (Census) [Others Direct]
	1	1993	1993 PS (Survey) [Others Direct]
	16	1996.3	1996 Census (Census) [Indirect]
	1	1996	1996 PES (PES) [Others Direct]
	1	2001.8	2002 SCIH (Panel) [Others Direct]
	15	2006.3	2006 Census (Census) [Indirect]
	14	2011.3	2011 Census (Census) [Indirect]
	16	2016	2016 Census (Census) [Others Direct]
	12	2021.5	Register (Register) [VR]
Iraq	92	2016.8	
	1	1965.3	1965 Census (Census) [Indirect]
	1	1974	1974 FS (Survey) [Indirect]
	1	1987.3	1987 Census (Census) [Indirect]
	2	1988.8	1989 CHS (Survey) [Indirect]
	17	1997.3	1997 Census (Census) [Indirect]
	2	1997.7	1999 CMMS (Survey) [Others Direct]
	1	2000.2	2000 MICS (Survey) [other DHS Direct]
	1	2006.4	2006-2007 FHS (Survey) [Indirect]
	32	2005.8	2006 MICS (Survey) [Indirect]
	16	2009.7	2011 MICS (Survey) [other DHS Direct]
	16	2016.8	2018 MICS (Survey) [other DHS Direct]

Continued on next page

Table 18 – continued from previous page

Country/Area	# obs.	Most recent obs. year	Data series name [source type]
	2	1987.5	Estimates (Estimate) [VR]
Ireland	133	2021.5	
	66	2020.5	Estimates (Estimate) [VR]
	67	2021.5	Register (Register) [VR]
Israel	94	2021.5	
	27	2009.5	Estimates (Estimate) [VR]
	67	2021.5	Register (Register) [VR]
Italy	143	2021.5	
	71	2020.5	Estimates (Estimate) [VR]
	72	2021.5	Register (Register) [VR]
Jamaica	67	2018.5	
	1	1969.8	1970 Census (Census) [Indirect]
	10	1968.5	1975-1976 WFS (Survey) [other DHS Direct]
	1	1982.5	1983 CPS (Survey) [Others Direct]
	2	1988.5	1989 CPS (Survey) [Others Direct]
	1	1992.5	1993 CPS (Survey) [Others Direct]
	1	1996.5	1997 RHS (Survey) [other DHS Direct]
	1	2002	2002-2003 RHS (Survey) [other DHS Direct]
	1	2007.4	2008 RHS (Survey) [other DHS Direct]
	2	2011	2011 Census (Census) [Others Direct]
	1	2010.6	2011 MICS (Survey) [other DHS Direct]
	46	2018.5	Register (Register) [VR]
Japan	145	2022.5	
	72	2021.5	Estimates (Estimate) [VR]
	73	2022.5	Register (Register) [VR]
Jersey	153	2021.5	
	71	2020.5	Estimates (Estimate) [VR]
	82	2021.5	Register (Register) [VR]
Jordan	134	2021.7	
	1	1961.4	1961 Census (Census) [Others Direct]
	1	1976	1976 WFS (Survey) [other DHS Direct]
	10	1980.9	1981 DS (Survey) [Indirect]
	1	1982	1982 MS (Survey) [Others Direct]
	16	1989.4	1990 DHS (Survey) [DHS Direct]
	16	1996.1	1997 DHS (Survey) [DHS Direct]
	16	2001.1	2002 DHS (Survey) [DHS Direct]
	16	2006.2	2007 DHS (Survey) [DHS Direct]
	16	2008.4	2009 DHS Interim (Survey) [other DHS Direct]
	16	2011.3	2012 DHS (Survey) [DHS Direct]
	14	2016.3	2017-2018 DHS (Survey) [DHS Direct]
	1	2021.7	2023 DHS (Survey) [DHS Direct]
	10	1978.5	Register (Register) [VR]
Kazakhstan	81	2022.5	
	1	1988.5	1989 Census (Census) [Others Direct]
	16	1994	1995 DHS (Survey) [DHS Direct]
	16	1998.1	1999 DHS (Survey) [DHS Direct]

Continued on next page

Table 18 – continued from previous page

Country/Area	# obs.	Most recent obs. year	Data series name [source type]
	1	2015.3	2015 MICS (Survey) [other DHS Direct]
	47	2022.5	Register (Register) [VR]
Kenya	134	2022.5	
	1	1962.1	1962 Census (Census) [Others Direct]
	1	1969.1	1969 Census (Census) [Others Direct]
	14	1976.5	1977-1978 WFS (Survey) [other DHS Direct]
	1	1984	1984 CPS (Survey) [Others Direct]
	1	1989.1	1989 Census (Census) [Indirect]
	16	1987.7	1989 DHS (Survey) [DHS Direct]
	16	1991.9	1993 DHS (Survey) [DHS Direct]
	16	1996.8	1998 DHS (Survey) [DHS Direct]
	1	1999.1	1999 Census (Census) [Indirect]
	16	2002	2003 DHS (Survey) [DHS Direct]
	16	2007.5	2008-2009 DHS (Survey) [DHS Direct]
	1	2009.1	2009 Census (Census) [Others Direct]
	16	2013.1	2014 DHS (Survey) [DHS Direct]
	2	2014.1	2015 MIS (Survey) [other DHS Direct]
	2	2014.5	2015 PMA-R3 (Panel) [other DHS Direct]
	2	2015.9	2016 PMA-R5 (Panel) [other DHS Direct]
	1	2016.9	2017 PMA-R6 (Panel) [other DHS Direct]
	1	2017.9	2018 PMA-R7 (Panel) [other DHS Direct]
	1	2019.2	2019 Census (Census) [Others Direct]
	2	2019.3	2020 MIS (Survey) [other DHS Direct]
	7	2022.5	Register (Register) [VR]
Kiribati	59	2017.5	
	3	1966.4	1968 Census (Census) [Indirect]
	4	1973.4	1973 Census (Census) [Others Direct]
	5	1978.4	1978 Census (Census) [Others Direct]
	4	1984.9	1985 Census (Census) [Others Direct]
	4	1990.4	1990 Census (Census) [Others Direct]
	3	1993.4	1995 Census (Census) [Indirect]
	4	2000.4	2000 Census (Census) [Indirect]
	4	2005.3	2005 Census (Census) [Indirect]
	5	2010.3	2010 Census (Census) [Indirect]
	3	2013.4	2015 Census (Census) [Indirect]
	16	2017.5	2018-2019 MICS (Survey) [other DHS Direct]
	4	1996.5	Register (Register) [VR]
Kosovo (under UNSC res. 1244)	67	2019.5	
	1	2001.5	2003 KDHS (Survey) [Others Direct]
	1	2009.4	2009 KDHS (Survey) [Others Direct]
	65	2019.5	Register (Register) [VR]
Kuwait	59	2022.5	
	5	1995	1996 FHS (Survey) [other DHS Direct]
	54	2022.5	Register (Register) [VR]
Kyrgyz Republic	103	2022.5	

Continued on next page

Table 18 – continued from previous page

Country/Area	# obs.	Most recent obs. year	Data series name [source type]
	16	1996.2	1997 DHS (Survey) [DHS Direct]
	16	2011.3	2012 DHS (Survey) [DHS Direct]
	16	2012.9	2014 MICS (Survey) [other DHS Direct]
	16	2017.3	2018 MICS (Survey) [other DHS Direct]
	39	2022.5	Register (Register) [VR]
Lao People's Democratic Republic	87	2022	
	2	1992.9	1993 SIS (Survey) [Others Direct]
	1	1994.7	1995 Census (Census) [Indirect]
	15	1999.7	2000 MICS (Survey) [Indirect]
	1	2000	2000 RHS (Survey) [Indirect]
	15	2003	2003 WHS (Survey) [Indirect]
	2	2004.7	2005 Census (Census) [Indirect]
	1	2004.3	2005 RHS (Survey) [Indirect]
	16	2005.8	2006 MICS (Survey) [Indirect]
	16	2010.4	2011-2012 DHS-MICS Special (Survey) [other DHS Direct]
	1	2014.7	2015 Census (Census) [Others Direct]
	16	2016.1	2017 MICS (Survey) [other DHS Direct]
	1	2022	2023 MICS (Survey) [other DHS Direct]
Latvia	133	2022.5	
	59	2017.5	Estimates (Estimate) [VR]
	1	2022.5	Pop. Register (Register) [VR]
	73	2022.5	Register (Register) [VR]
Lebanon	49	2003.1	
	17	1995.7	1995-1996 PHS (Survey) [Indirect]
	16	1994.6	1996 MCHS (Survey) [other DHS Direct]
	16	2003.1	2004 FHS (Survey) [other DHS Direct]
Lesotho	97	2020.9	
	1	1971.9	1971-1973 DS (Panel) [Others Direct]
	2	1975.8	1976 Census (Census) [Others Direct]
	14	1976.1	1977 WFS (Survey) [other DHS Direct]
	1	1983.8	1986 Census (Census) [Others Direct]
	3	1995.8	1996 Census (Census) [Others Direct]
	1	2000.9	2001 DS (Survey) [Others Direct]
	4	2002	2002 RHS (Survey) [Others Direct]
	16	2003.4	2004 DHS (Survey) [DHS Direct]
	1	2005.8	2006 Census (Census) [Others Direct]
	16	2008.4	2009 DHS (Survey) [DHS Direct]
	2	2010.8	2011 DS (Survey) [Others Direct]
	16	2013.3	2014 DHS (Survey) [DHS Direct]
	2	2015.8	2016 Census (Census) [Indirect]
	16	2017	2018 MICS (Survey) [other DHS Direct]
	2	2020.9	2021 DS (Survey) [Others Direct]
Liberia	90	2021.4	
	2	1969.3	1970 PGS (Panel) [Indirect]

Continued on next page

Table 18 – continued from previous page

Country/Area	# obs.	Most recent obs. year	Data series name [source type]
	1	1970.3	1971 PGS (Panel) [Others Direct]
	2	1973.6	1974 Census (Census) [Indirect]
	1	1980.6	1984 Census (Census) [Indirect]
	16	1984.8	1986 DHS (Survey) [DHS Direct]
	16	2005.6	2007 DHS (Survey) [DHS Direct]
	1	2006.7	2008 Census (Census) [Indirect]
	13	2007.6	2009 MIS (Survey) [other DHS Direct]
	2	2010.3	2011 MIS (Survey) [other DHS Direct]
	16	2011.9	2013 DHS (Survey) [DHS Direct]
	2	2015.3	2016 MIS (Survey) [other DHS Direct]
	16	2018.5	2019-2020 DHS (Survey) [DHS Direct]
	2	2021.4	2022 MIS (Survey) [other DHS Direct]
Libya	32	2013	
	16	1994	1995 MCH (Survey) [other DHS Direct]
	5	2005	2007 FHS (Survey) [other DHS Direct]
	1	2013	2014 NFHS (Survey) [other DHS Direct]
	10	2009.5	Register (Register) [VR]
Lithuania	126	2022.5	
	62	2020.5	Estimates (Estimate) [VR]
	64	2022.5	Register (Register) [VR]
Luxembourg	127	2021.5	
	55	2014.5	Estimates (Estimate) [VR]
	72	2021.5	Register (Register) [VR]
Madagascar	115	2019.8	
	1	1966.1	1966 ED (Survey) [Others Direct]
	1	1974.9	1975 Census (Census) [Others Direct]
	1	1980	1980 Survey (Survey) [Others Direct]
	16	1991.1	1992 DHS (Survey) [DHS Direct]
	1	1993.1	1993 Census (Census) [Indirect]
	16	1996.3	1997 DHS (Survey) [DHS Direct]
	16	2002.5	2003-2004 DHS (Survey) [DHS Direct]
	16	2007.7	2008-2009 DHS (Survey) [DHS Direct]
	2	2009.8	2011 MIS (Survey) [other DHS Direct]
	2	2011.9	2013 MIS (Survey) [other DHS Direct]
	2	2015	2016 MIS (Survey) [other DHS Direct]
	1	2017.9	2018 Census (Census) [Others Direct]
	16	2017.3	2018 MICS (Survey) [other DHS Direct]
	16	2019.8	2021 DHS (Survey) [DHS Direct]
	8	1972.5	Register (Register) [VR]
Malawi	142	2018.8	
	1	1970.6	1970-1972 PCS (Panel) [Others Direct]
	1	1977.2	1977 Census (Census) [Others Direct]
	1	1982.4	1982 MDS (Survey) [Others Direct]
	1	1982.4	1984 FFS (Survey) [Others Direct]
	2	1987.2	1987 Census (Census) [Others Direct]
	16	1991.3	1992 DHS (Survey) [DHS Direct]

Continued on next page

Table 18 – continued from previous page

Country/Area	# obs.	Most recent obs. year	Data series name [source type]
	1	1998.2	1998 Census (Census) [Others Direct]
	16	1999.2	2000 DHS (Survey) [DHS Direct]
	16	2003.4	2004 DHS (Survey) [DHS Direct]
	16	2005.2	2006 MICS (Survey) [other DHS Direct]
	1	2008	2008 Census (Census) [Others Direct]
	16	2009.2	2010 DHS (Survey) [DHS Direct]
	2	2010.8	2012 MIS (Survey) [other DHS Direct]
	16	2012.6	2013-2014 MICS (Survey) [other DHS Direct]
	2	2012.9	2014 MIS (Survey) [other DHS Direct]
	16	2014.5	2015-2016 DHS (Survey) [DHS Direct]
	2	2015.8	2017 MIS (Survey) [other DHS Direct]
	16	2018.8	2019-2020 MICS (Survey) [other DHS Direct]
Malaysia	43	2022.5	
	5	1973	1974 WFS (Survey) [other DHS Direct]
	1	2020	2020 Census (Census) [Others Direct]
	37	2022.5	Register (Register) [VR]
Maldives	64	2019.5	
	1	1967	1967 Census (Census) [Indirect]
	1	1984.7	1985 Census (Census) [Indirect]
	1	1989.7	1990 Census (Census) [Others Direct]
	3	1992.7	1995 Census (Census) [Indirect]
	4	1999.8	2000 Census (Census) [Others Direct]
	2	2005.7	2006 Census (Census) [Indirect]
	16	2007.9	2009 DHS (Survey) [DHS Direct]
	1	2014.2	2014 Census (Census) [Others Direct]
	14	2015.7	2016-2017 DHS (Survey) [DHS Direct]
	21	2019.5	Register (Register) [VR]
Mali	121	2020.3	
	2	1957.2	1956-1958 ED (Survey) [Indirect]
	2	1960.4	1960-1961 ED (Survey) [Indirect]
	1	1983.8	1987 Census (Census) [Indirect]
	16	1985.9	1987 DHS (Survey) [DHS Direct]
	16	1994.6	1995-1996 DHS (Survey) [DHS Direct]
	1	1997.8	1998 Census (Census) [Others Direct]
	16	1999.7	2001 DHS (Survey) [DHS Direct]
	16	2005.2	2006 DHS (Survey) [DHS Direct]
	1	2008.8	2009 Census (Census) [Others Direct]
	16	2011.5	2012-2013 DHS (Survey) [DHS Direct]
	16	2014.2	2015 MICS (Survey) [other DHS Direct]
	2	2014.3	2015 MIS (Survey) [other DHS Direct]
	14	2017.2	2018 DHS (Survey) [DHS Direct]
	2	2020.3	2021 MIS (Survey) [other DHS Direct]
Malta	88	2021.5	
	23	2009.5	Estimates (Estimate) [VR]
	65	2021.5	Register (Register) [VR]
Martinique	56	2021.5	

Continued on next page

Table 18 – continued from previous page

Country/Area	# obs.	Most recent obs. year	Data series name [source type]
	56	2021.5	Register (Register) [VR]
Mauritania	115	2019.1	
	2	1964.9	1964-1965 PS (Survey) [Indirect]
	2	1976.6	1976-1977 Census (Census) [Indirect]
	8	1981.1	1981 WFS (Survey) [Indirect]
	3	1987.8	1988 Census (Census) [Indirect]
	4	1988.5	1990-1991 MCHS (Survey) [other DHS Direct]
	16	1999.5	2000-2001 DHS (Survey) [DHS Direct]
	1	2000.4	2000 Census (Census) [Others Direct]
	16	2002.4	2003-2004 DHS-NS (Survey) [other DHS Direct]
	15	2003	2003 WHS (Survey) [Indirect]
	16	2010.1	2011 MICS (Survey) [other DHS Direct]
	16	2014.2	2015 MICS (Survey) [other DHS Direct]
	16	2019.1	2019-2021 DHS (Survey) [DHS Direct]
Mauritius	55	2022.5	
	1	1984.6	1985 CPS (Survey) [Others Direct]
	1	1990	1990 Census (Census) [Indirect]
	1	1990.5	1991 CPS (Survey) [Others Direct]
	1	2000	2000 Census (Census) [Indirect]
	51	2022.5	Register (Register) [VR]
Mayotte	7	2017.5	
	1	1978	1978 Census (Census) [Indirect]
	6	2017.5	Register (Register) [VR]
Mexico	53	2018.5	
	8	1969.4	1976-1977 WFS (Survey) [other DHS Direct]
	1	1977.2	1978 ENPUMA (Survey) [Others Direct]
	2	1979.3	1979 ENPUMA (Survey) [Others Direct]
	16	1985.7	1987 DHS (Survey) [DHS Direct]
	3	2000.1	2000 Census (Census) [Others Direct]
	1	2008	2009 ENADID (Panel) [Others Direct]
	3	2010.4	2010 Census (Census) [Others Direct]
	1	2013.2	2014 ENADID (Survey) [Others Direct]
	1	2014.5	2015 Intercensal Survey (Survey) [Others Direct]
	2	2016.2	2018 ENADID (Survey) [Others Direct]
	15	2018.5	Register (Register) [VR]
Micronesia (Fed. States of)	41	2009.8	
	3	1965	1967 Census (Census) [Indirect]
	7	1973	1973 Census (Census) [Indirect]
	5	1980	1980 Census (Census) [Indirect]
	6	1994.2	1994 Census (Census) [Indirect]
	6	1999.7	2000 Census (Census) [Others Direct]
	4	2009.8	2010 Census (Census) [Indirect]
	10	2006.5	Register (Register) [VR]
Mongolia	89	2022.5	
	3	1992	1994 DS (Survey) [Others Direct]

Continued on next page

Table 18 – continued from previous page

Country/Area	# obs.	Most recent obs. year	Data series name [source type]
	3	1994	1996 DS (Survey) [Others Direct]
	5	1997	1998 RHS (Survey) [Others Direct]
	1	1999.5	2000 Census (Census) [Others Direct]
	1	2002	2003 RHS (Survey) [Others Direct]
	1	2007	2008 RHS (Survey) [Others Direct]
	1	2009.1	2010-2011 MICS (Survey) [other DHS Direct]
	1	2010.4	2010 Census (Census) [Others Direct]
	16	2012.4	2013 MICS (Survey) [other DHS Direct]
	16	2017.4	2018 MICS (Survey) [other DHS Direct]
	1	2020	2020 Census (Census) [Others Direct]
	1	2020.5	Estimates (Estimate) [VR]
	39	2022.5	Register (Register) [VR]
Montenegro	118	2022.5	
	54	2006.5	Estimates (Estimate) [VR]
	64	2022.5	Register (Register) [VR]
Morocco	109	2009.5	
	1	1961.9	1961-1963 MPS (Panel) [Others Direct]
	2	1972	1972-1973 VR (Panel) [Indirect]
	17	1978.5	1980 WFS (Survey) [Indirect]
	1	1982.2	1982 Census (Census) [Others Direct]
	1	1983.5	1983-1984 CPS (Survey) [Others Direct]
	1	1986	1986-1988 ENDPR (Panel) [Others Direct]
	16	1985.9	1987 DHS (Survey) [DHS Direct]
	16	1990.7	1992 DHS (Survey) [DHS Direct]
	1	1994.2	1994 Census (Census) [Others Direct]
	16	1993.8	1995 DHS Special (Panel) [Others Direct]
	1	1995	1996-1998 ENDPR (Panel) [Others Direct]
	16	1996.3	1997 ENSME (Survey) [other DHS Direct]
	1	1996	1998-1999 ENNV (Survey) [Others Direct]
	16	2002.5	2003-2004 DHS (Survey) [other DHS Direct]
	1	2004.2	2004 Census (Census) [Others Direct]
	2	2009.5	2011 ENPSF (Survey) [other DHS Direct]
Mozambique	73	2021.3	
	1	1970.5	1970 Census (Census) [Others Direct]
	1	1980.1	1980 Census (Census) [Others Direct]
	1	1997.1	1997 Census (Census) [Others Direct]
	16	1995.9	1997 DHS (Survey) [DHS Direct]
	16	2002.3	2003 DHS (Survey) [DHS Direct]
	1	2007.1	2007 Census (Census) [Others Direct]
	16	2007.3	2008 MICS (Survey) [other DHS Direct]
	16	2010.2	2011 DHS (Survey) [DHS Direct]
	2	2014.1	2015 AIS (Survey) [other DHS Direct]
	2	2016.9	2018 MIS (Survey) [other DHS Direct]
	1	2021.3	2022-2023 DHS (Survey) [DHS Direct]
Myanmar	31	2014.8	
	1	1982.7	1983 Census (Census) [Others Direct]

Continued on next page

Table 18 – continued from previous page

Country/Area	# obs.	Most recent obs. year	Data series name [source type]
	5	1989	1991 PCFS (Survey) [Others Direct]
	3	1996.8	1997 FRHS (Survey) [Others Direct]
	3	2001	2001 FRHS (Survey) [Others Direct]
	1	2006.5	2007 FRHS (Survey) [Others Direct]
	2	2013.8	2014 Census (Census) [Others Direct]
	16	2014.8	2015-2016 DHS (Survey) [DHS Direct]
Namibia	75	2016.3	
	4	1989	1989 Demographic and Health Survey (Survey) [Indirect]
	2	1991.3	1991 Census (Census) [Indirect]
	16	1991.2	1992 DHS (Survey) [DHS Direct]
	16	1999.3	2000 DHS (Survey) [DHS Direct]
	2	2001.1	2001 Census (Census) [Others Direct]
	16	2005.5	2006-2007 DHS (Survey) [DHS Direct]
	2	2011.2	2011 Census (Census) [Others Direct]
	16	2012	2013 DHS (Survey) [DHS Direct]
	1	2016.3	2016 NIDS (Survey) [Others Direct]
Nepal	199	2020.8	
	2	1961	1961 Census (Census) [Indirect]
	2	1971	1971 Census (Census) [Indirect]
	2	1976	1974-1978 DSS (Panel) [Others Direct]
	5	1975.9	1976 WFS (Survey) [Indirect]
	2	1981	1981 Census (Census) [Others Direct]
	1	1980.3	1981 CPS (Survey) [Others Direct]
	5	1984.5	1985-1986 FFPS (Survey) [Others Direct]
	3	1986	1986-1987 DSS (Survey) [Indirect]
	5	1990.8	1991-1992 FFPHS (Survey) [Indirect]
	5	1995.5	1995-1996 LSMS (Survey) [Indirect]
	16	1994.7	1996 DHS (Survey) [DHS Direct]
	1	2001	2001 Census (Census) [Others Direct]
	16	1999.7	2001 DHS (Survey) [DHS Direct]
	1	2003.5	2003-2004 LSMS (Survey) [Indirect]
	16	2004.9	2006 DHS (Survey) [DHS Direct]
	2	2011	2011 Census (Census) [Indirect]
	16	2009.7	2011 DHS (Survey) [DHS Direct]
	16	2012.8	2014 MICS (Survey) [other DHS Direct]
	16	2015.2	2016 DHS (Survey) [DHS Direct]
	16	2018	2019 MICS (Survey) [other DHS Direct]
	16	2020.8	2022 DHS (Survey) [DHS Direct]
	35	1995.5	Estimates (Estimate) [VR]
Netherlands	142	2021.5	
	70	2019.5	Estimates (Estimate) [VR]
	72	2021.5	Register (Register) [VR]
New Caledonia	54	2021.5	
	54	2021.5	Register (Register) [VR]
New Zealand	119	2022.5	
	58	2007.5	Estimates (Estimate) [VR]

Continued on next page

Table 18 – continued from previous page

Country/Area	# obs.	Most recent obs. year	Data series name [source type]
	61	2022.5	Register (Register) [VR]
Nicaragua	68	2010.5	
	2	1977.5	1978 RETRO-EDENIC (Survey) [Indirect]
	12	1991.5	1992-1993 ESF-RHS (Survey) [other DHS Direct]
	5	1997.4	1998-1999 LSMS (Survey) [Others Direct]
	14	1996.7	1998 DHS (Survey) [DHS Direct]
	14	2000.3	2001 DHS (Survey) [DHS Direct]
	2	2005.4	2005 Census (Census) [Others Direct]
	14	2005.5	2006-2007 RHS-NDHS (Survey) [other DHS Direct]
	1	2009.7	2011-2012 NDHS (Survey) [Others Direct]
	4	2010.5	Register (Register) [VR]
Niger	65	2020.2	
	1	1959.5	1959-1960 ED (Survey) [Others Direct]
	1	1987.9	1988 Census (Census) [Others Direct]
	12	1990.8	1992 DHS (Survey) [DHS Direct]
	12	1996.9	1998 DHS (Survey) [DHS Direct]
	1	1999.9	2000 MICS (Survey) [other DHS Direct]
	1	2000.9	2001 Census (Census) [Others Direct]
	14	2004.7	2006 DHS (Survey) [other DHS Direct]
	1	2012.5	2012 Census (Census) [Others Direct]
	14	2010.8	2012 DHS (Survey) [DHS Direct]
	1	2015.1	2015 ENISED (Survey) [Others Direct]
	2	2015.2	2016 PMA-R2 (Panel) [other DHS Direct]
	2	2016.5	2017 PMA-R4 (Panel) [other DHS Direct]
	1	2020	2021 ENAFEME (Survey) [Others Direct]
	2	2020.2	2021 MIS (Survey) [other DHS Direct]
Nigeria	160	2020.4	
	1	1968	1971-1975 FFFP (Survey) [Indirect]
	16	1980.7	1981-1982 WFS (Survey) [other DHS Direct]
	16	1989	1990 DHS (Survey) [DHS Direct]
	1	1990.4	1991 Census (Census) [Indirect]
	5	1997.8	1999 DHS (Survey) [DHS Direct]
	2	2000	2000 SSNPP (Survey) [Indirect]
	16	2001.9	2003 DHS (Survey) [DHS Direct]
	1	2004.7	2007 MICS (Survey) [Indirect]
	16	2007.1	2008 DHS (Survey) [DHS Direct]
	16	2009.4	2010 MIS (Survey) [other DHS Direct]
	1	2010.7	2011 MICS (Survey) [other DHS Direct]
	16	2011.8	2013 DHS (Survey) [DHS Direct]
	2	2014.3	2015 MIS (Survey) [other DHS Direct]
	16	2015.4	2016-2017 MICS (Survey) [other DHS Direct]
	2	2015.4	2016 PMA-R3 (Panel) [other DHS Direct]
	1	2016.3	2017 PMA-R4 (Panel) [other DHS Direct]
	14	2017.3	2018 DHS (Survey) [DHS Direct]
	16	2020.3	2021 MICS (Survey) [other DHS Direct]
	2	2020.4	2021 MIS (Survey) [other DHS Direct]

Continued on next page

Table 18 – continued from previous page

Country/Area	# obs.	Most recent obs. year	Data series name [source type]
North Macedonia	133	2022.5	
	60	2009.5	Estimates (Estimate) [VR]
	73	2022.5	Register (Register) [VR]
Norway	146	2022.5	
	73	2022.5	Estimates (Estimate) [VR]
	73	2022.5	Register (Register) [VR]
Oman	32	2022.5	
	1	1985.5	1984-1989 DSS (Survey) [Others Direct]
	4	1995.4	1995 FHS (Survey) [other DHS Direct]
	1	1999.5	2000 NHS (Survey) [Others Direct]
	1	2003.4	2003 Census (Census) [Others Direct]
	1	2013	2014 MICS (Survey) [Others Direct]
	1	2020	2020 Census (Census) [Others Direct]
	23	2022.5	Register (Register) [VR]
Pakistan	95	2021.1	
	1	1963.5	1962-1965 PGE (Survey) [Others Direct]
	5	1967.5	1968-1969 NIS (Survey) [Others Direct]
	2	1969.5	1968-1971 PGS (Panel) [Indirect]
	7	1975.2	1975 WFS (Survey) [Indirect]
	1	1976.5	1976-1979 PGS (Panel) [Others Direct]
	3	1977.5	1979-1980 LFMS (Survey) [Others Direct]
	2	1984	1984-1985 CPS (Survey) [Indirect]
	1	1992.9	1993 CPS (Survey) [Others Direct]
	2	1994	1994-1995 CPS (Survey) [Indirect]
	2	1994.5	1995-1996 IHS (Survey) [Indirect]
	4	1994.5	1996-1997 FFPS (Survey) [Indirect]
	1	1995.5	1996-1997 IHS (Survey) [Indirect]
	2	1997.5	1998-1999 IHS (Survey) [Indirect]
	2	1997.7	1998 Census (Census) [Indirect]
	2	1998.4	2000-2001 RHFPS (Survey) [Indirect]
	2	2000.5	2001-2002 IHS (Survey) [Indirect]
	1	2002	2003 SWRHFPS (Survey) [Others Direct]
	2	2004	2005-2006 LSMS (Survey) [Indirect]
	16	2005.4	2006-2007 DHS (Survey) [DHS Direct]
	16	2011.5	2012-2013 DHS (Survey) [DHS Direct]
	14	2016.4	2017-2018 DHS (Survey) [DHS Direct]
	1	2018	2018-2019 LSMS (Survey) [Others Direct]
	6	2021.1	Annual PDS (Survey) [Others Direct]
Panama	56	2021.5	
	2	1990.4	1990 Census (Census) [Others Direct]
	2	2000.4	2000 Census (Census) [Others Direct]
	2	2010.4	2010 Census (Census) [Others Direct]
	50	2021.5	Register (Register) [VR]
Papua New Guinea	37	2016.4	
	1	1971	1971 Census (Census) [Others Direct]
	1	1979	1979-1980 DS (Survey) [Others Direct]

Continued on next page

Table 18 – continued from previous page

Country/Area	# obs.	Most recent obs. year	Data series name [source type]
	4	1980.2	1980 Census (Census) [Others Direct]
	3	1988	1990 Census (Census) [Indirect]
	4	1994.5	1996 DHS (Survey) [Others Direct]
	3	1998	2000 Census (Census) [Indirect]
	4	2004	2006 DHS (Survey) [Others Direct]
	3	2009	2011 Census (Census) [Indirect]
	14	2016.4	2016-2018 DHS (Survey) [DHS Direct]
Paraguay	104	2015.1	
	14	1978	1979 WFS (Survey) [other DHS Direct]
	2	1982.5	1982 Census (Census) [Others Direct]
	16	1989	1990 DHS (Survey) [DHS Direct]
	2	1992.7	1992 Census (Census) [Others Direct]
	13	1994.5	1995-1996 ENDSR-RHS (Survey) [other DHS Direct]
	13	1997.3	1998 ENSMI-RHS (Survey) [other DHS Direct]
	2	2002.7	2002 Census (Census) [Others Direct]
	13	2002.9	2004 ENDSSR-RHS (Survey) [other DHS Direct]
	13	2007.1	2008 ENDSSR-RHS (Survey) [other DHS Direct]
	16	2015.1	2016 MICS (Survey) [other DHS Direct]
Peru	125	2021	
	1	1981	1981 Census (Census) [Others Direct]
	16	1985.3	1986 DHS (Survey) [DHS Direct]
	15	1989.5	1991-1992 DHS (Survey) [DHS Direct]
	12	1999.2	2000 DHS (Survey) [DHS Direct]
	3	2003.8	2004-2006 DHS (Survey) [DHS Direct]
	11	2002.8	2004 DHS (Survey) [DHS Direct]
	11	2006.4	2007-2008 DHS (Survey) [DHS Direct]
	11	2008	2009 DHS (Survey) [DHS Direct]
	12	2009.1	2010 DHS (Survey) [DHS Direct]
	13	2010.1	2011 DHS (Survey) [DHS Direct]
	11	2011.1	2012 DHS (Survey) [DHS Direct]
	1	2012	2013 DHS (Survey) [other DHS Direct]
	1	2013.1	2014 DHS (Survey) [other DHS Direct]
	1	2015.1	2016 DHS (Survey) [other DHS Direct]
	1	2017.8	2017 Census (Census) [Others Direct]
	1	2016.1	2017 DHS (Survey) [other DHS Direct]
	1	2017	2018 DHS (Survey) [other DHS Direct]
	1	2018	2019 DHS (Survey) [other DHS Direct]
	1	2020	2021 DHS (Survey) [other DHS Direct]
	1	2021	2022 DHS (Survey) [other DHS Direct]
Philippines	186	2021.5	
	14	1976.8	1978 WFS (Survey) [other DHS Direct]
	1	1981	1983 DS (Survey) [Others Direct]
	1	1985	1986 CPS (Survey) [Others Direct]
	16	1991.8	1993 DHS (Survey) [DHS Direct]
	16	1996.7	1998 DHS (Survey) [DHS Direct]
	16	2002.1	2003 DHS (Survey) [DHS Direct]

Continued on next page

Table 18 – continued from previous page

Country/Area	# obs.	Most recent obs. year	Data series name [source type]
	16	2007.2	2008 DHS (Survey) [DHS Direct]
	16	2012.2	2013 DHS (Survey) [DHS Direct]
	16	2016.2	2017 DHS (Survey) [DHS Direct]
	5	2020.9	2022 DHS (Survey) [DHS Direct]
	69	2021.5	Register (Register) [VR]
Poland	128	2022.5	
	55	2019.5	Estimates (Estimate) [VR]
	73	2022.5	Register (Register) [VR]
Portugal	160	2021.5	
	16	1978.5	1979-1980 WFS (Survey) [other DHS Direct]
	72	2021.5	Estimates (Estimate) [VR]
	72	2021.5	Register (Register) [VR]
Puerto Rico	72	2022.5	
	1	2009.7	2010 Census (Census) [Others Direct]
	71	2022.5	Register (Register) [VR]
Qatar	48	2021.5	
	5	1986	1987 CHS (Survey) [other DHS Direct]
	5	1997	1998 FHS (Survey) [other DHS Direct]
	38	2021.5	Register (Register) [VR]
Republic of Korea	106	2022.5	
	6	1966.3	1966 Census (Census) [Indirect]
	6	1970.3	1970 Census (Census) [Indirect]
	1	1971	1971 FAS (Survey) [Others Direct]
	45	2020.5	Estimates (Estimate) [VR]
	48	2022.5	Register (Register) [VR]
Republic of Moldova	108	2022.5	
	13	1996.2	1997 RHS (Survey) [other DHS Direct]
	16	2004	2005 DHS (Survey) [DHS Direct]
	16	2010.9	2012 MICS (Survey) [other DHS Direct]
	45	2022.5	Estimates (Estimate) [VR]
	18	1989.5	Register (Register) [VR]
Réunion	56	2021.5	
	56	2021.5	Register (Register) [VR]
Romania	123	2021.5	
	1	1998.2	1999 RHS (Survey) [other DHS Direct]
	55	2009.5	Estimates (Estimate) [VR]
	67	2021.5	Register (Register) [VR]
Russian Federation	111	2022.5	
	57	2018.5	Estimates (Estimate) [VR]
	54	2022.5	Register (Register) [VR]
Rwanda	140	2018.6	
	1	1970	1970 ED (Survey) [Others Direct]
	1	1978.1	1978 Census (Census) [Others Direct]
	2	1981	1981 ED (Survey) [Others Direct]
	16	1982	1983 WFS (Survey) [other DHS Direct]

Continued on next page

Table 18 – continued from previous page

Country/Area	# obs.	Most recent obs. year	Data series name [source type]
	2	1991.1	1991 Census (Census) [Others Direct]
	16	1991.1	1992 DHS (Survey) [DHS Direct]
	1	1996	1996 SDS (Survey) [Indirect]
	16	1999	2000 DHS (Survey) [DHS Direct]
	1	2002.1	2002 Census (Census) [Others Direct]
	16	2003.8	2005 DHS (Survey) [DHS Direct]
	16	2006.6	2007-2008 DHS Interim (Survey) [other DHS Direct]
	16	2009.5	2010 DHS (Survey) [DHS Direct]
	2	2011.7	2013 MIS (Survey) [other DHS Direct]
	16	2013.6	2014-2015 DHS (Survey) [DHS Direct]
	2	2016.3	2017 MIS (Survey) [other DHS Direct]
	16	2018.6	2019-2020 DHS (Survey) [DHS Direct]
Saint Lucia	43	2020.5	
	1	2000.9	2001 Census (Census) [Others Direct]
	42	2020.5	Register (Register) [VR]
Saint Vincent and the Grenadines	49	2021.5	
	49	2021.5	Register (Register) [VR]
Samoa	67	2018.5	
	3	1959.2	1961 Census (Census) [Indirect]
	5	1964.4	1966 Census (Census) [Indirect]
	5	1969.3	1971 Census (Census) [Indirect]
	1	1981.3	1981 Census (Census) [Others Direct]
	3	1984.3	1986 Census (Census) [Indirect]
	5	1991.3	1991 Census (Census) [Others Direct]
	2	2001.3	2001 Census (Census) [Others Direct]
	2	2006.3	2006 Census (Census) [Others Direct]
	3	2008.2	2009 DHS (Survey) [Indirect]
	3	2011.3	2011 Census (Census) [Others Direct]
	5	2013	2014 DHS (Survey) [Others Direct]
	2	2016.4	2016 Census (Census) [Others Direct]
	16	2018.5	2019-2020 MICS (Survey) [other DHS Direct]
	12	1975.5	Register (Register) [VR]
Sao Tome and Principe	58	2018	
	1	1991.1	1991 Census (Census) [Others Direct]
	1	2001.1	2001 Census (Census) [Others Direct]
	16	2007.4	2008-2009 DHS (Survey) [DHS Direct]
	17	2014	2014 MICS (Survey) [other DHS Direct]
	16	2018	2019 MICS (Survey) [other DHS Direct]
	7	1979.5	Register (Register) [VR]
Saudi Arabia	17	2021.9	
	1	1987	1987 CHS (Survey) [other DHS Direct]
	6	1996.4	1996-1997 FHS (Survey) [other DHS Direct]
	1	1998.6	1999 DS (Survey) [Others Direct]
	1	1999.6	2000 DS (Survey) [Others Direct]

Continued on next page

Table 18 – continued from previous page

Country/Area	# obs.	Most recent obs. year	Data series name [source type]
	1	2004.2	2004 Census (Census) [Others Direct]
	1	2006.6	2007 DS (Survey) [Others Direct]
	1	2021.9	2022 Census (Census) [Others Direct]
	4	2000.5	Estimates (Estimate) [VR]
	1	2005.5	Register (Register) [VR]
Senegal	252	2019.5	
	3	1960.5	1960-1961 ED (Survey) [Indirect]
	1	1970.6	1970-1971 ED (Panel) [Others Direct]
	15	1977.1	1978 WFS (Survey) [Indirect]
	16	1984.9	1986 DHS (Survey) [DHS Direct]
	15	1987.9	1988 Census (Census) [Indirect]
	16	1991.7	1992-1993 DHS (Survey) [DHS Direct]
	16	1995.7	1997 DHS (Survey) [DHS Direct]
	1	1999.9	2000 MICS (Survey) [other DHS Direct]
	17	2002.5	2002 Census (Census) [Indirect]
	16	2003.8	2005 DHS (Survey) [DHS Direct]
	2	2005.4	2006 MIS (Survey) [other DHS Direct]
	2	2007.5	2008-2009 MIS (Survey) [other DHS Direct]
	16	2009.5	2010-2011 DHS (Survey) [DHS Direct]
	16	2011.6	2012-2013 DHS (Survey) [DHS Direct]
	2	2012.4	2013 Census (Census) [Others Direct]
	16	2012.9	2014 DHS (Survey) [DHS Direct]
	16	2013.9	2015 DHS (Survey) [DHS Direct]
	16	2014.9	2016 DHS (Survey) [DHS Direct]
	16	2016.1	2017 DHS (Survey) [DHS Direct]
	16	2017.2	2018 DHS (Survey) [DHS Direct]
	16	2018	2019 DHS (Survey) [DHS Direct]
	2	2019.5	2020-2021 MIS (Survey) [other DHS Direct]
Serbia	71	2021.5	
	59	2009.5	Estimates (Estimate) [VR]
	12	2021.5	Register (Register) [VR]
Seychelles	53	2017.5	
	1	2010.2	2010 Census (Census) [Others Direct]
	52	2017.5	Register (Register) [VR]
Sierra Leone	121	2018	
	2	1974.4	1974 Census (Census) [Indirect]
	2	1985.5	1985 Census (Census) [Indirect]
	1	1991.9	1992 DSMS (Survey) [Others Direct]
	1	1997.8	2000 MICS (Survey) [Indirect]
	1	2000.7	2003 Pilot Census (Survey) [Indirect]
	16	2004.4	2004 Census (Census) [Indirect]
	16	2005.3	2005-2006 MICS (Survey) [Indirect]
	16	2006.9	2008 DHS (Survey) [DHS Direct]
	16	2010.4	2010 MICS (Survey) [Indirect]
	16	2012.1	2013 DHS (Survey) [DHS Direct]
	2	2015.1	2016 MIS (Survey) [other DHS Direct]

Continued on next page

Table 18 – continued from previous page

Country/Area	# obs.	Most recent obs. year	Data series name [source type]
	16	2016	2017 MICS (Survey) [other DHS Direct]
	16	2018	2019 DHS (Survey) [DHS Direct]
Singapore	82	2022.5	
	19	1975.5	Estimates (Estimate) [VR]
	63	2022.5	Register (Register) [VR]
Slovakia	137	2021.5	
	65	2014.5	Estimates (Estimate) [VR]
	72	2021.5	Register (Register) [VR]
Slovenia	137	2021.5	
	67	2019.5	Estimates (Estimate) [VR]
	70	2021.5	Register (Register) [VR]
Solomon Islands	28	2019.4	
	3	1967.6	1970 Census (Census) [Indirect]
	3	1973.6	1976 Census (Census) [Indirect]
	4	1986.4	1986 Census (Census) [Others Direct]
	4	1999.4	1999 Census (Census) [Others Direct]
	5	2005.5	2006-2007 DHS (Survey) [DHS Direct]
	5	2009.4	2009 Census (Census) [Indirect]
	1	2014	2015 DHS (Survey) [Others Direct]
	3	2019.4	2019 Census (Census) [Others Direct]
Somalia	35	2017	
	1	1974.6	1975 Census (Census) [Others Direct]
	1	1997	1999-2000 MICS (Survey) [Indirect]
	1	2000	2002-2003 Survey (Survey) [Indirect]
	16	2005.2	2006 MICS (Survey) [other DHS Direct]
	16	2017	2018-2019 SHDS (Survey) [Others Direct]
South Africa	79	2021.5	
	1	1987	1987-1989 DHS (Survey) [Others Direct]
	17	1996.3	1996 Census (Census) [Indirect]
	16	1996.9	1998 DHS (Survey) [DHS Direct]
	1	2001.3	2001 Census (Census) [Indirect]
	5	2002.7	2003 DHS (Survey) [DHS Direct]
	1	2006.6	2007 CS (Survey) [Indirect]
	1	2011.3	2011 Census (Census) [Others Direct]
	13	2015.2	2016 DHS (Survey) [DHS Direct]
	8	1985.5	Estimates (Estimate) [VR]
	16	2021.5	Register (Register) [VR]
South Sudan	21	2008.9	
	1	1992.8	1993 Census (Census) [Others Direct]
	4	2007.9	2008 Census (Census) [Indirect]
	16	2008.9	2010 MICS-HHS (Survey) [other DHS Direct]
Spain	144	2021.5	
	72	2021.5	Estimates (Estimate) [VR]
	72	2021.5	Register (Register) [VR]
Sri Lanka	102	2015.5	
	13	1974	1975 WFS (Survey) [Indirect]

Continued on next page

Table 18 – continued from previous page

Country/Area	# obs.	Most recent obs. year	Data series name [source type]
	1	1981.5	1982 CPS (Survey) [Others Direct]
	15	1985.6	1987 DHS (Survey) [DHS Direct]
	5	1992	1993 DHS (Survey) [Indirect]
	4	1998	2000 DHS (Survey) [Indirect]
	5	2005.7	2006-2007 DHS (Survey) [Indirect]
	59	2015.5	Register (Register) [VR]
State of Palestine	70	2018.5	
	1	1992	1992 LCS (Survey) [Others Direct]
	1	1991	1992 UNICEF-JFPPA Survey (Survey) [Others Direct]
	5	1995	1996 HS (Survey) [Others Direct]
	1	1997.4	1997 Census (Census) [Others Direct]
	2	2000	2000 HS (Survey) [Others Direct]
	1	2003.4	2004 DHS (Survey) [Others Direct]
	2	2006.4	2006 FHS-MICS (Survey) [Indirect]
	17	2009	2010 FHS-MICS (Survey) [Indirect]
	17	2013.6	2014 MICS (Survey) [other DHS Direct]
	16	2018.5	2019-2020 MICS (Survey) [other DHS Direct]
	7	2007.5	Register (Register) [VR]
Sudan	74	2013.3	
	2	1972.8	1973 Census (Census) [Others Direct]
	14	1977.5	1978-1979 WFS (Survey) [other DHS Direct]
	16	1988.6	1989-1990 DHS (Survey) [DHS Direct]
	2	1991.5	1992-1993 MCH (Survey) [other DHS Direct]
	3	1992.8	1993 Census (Census) [Indirect]
	3	1996.9	1999 SMS (Survey) [Others Direct]
	2	2006.8	2008 Census (Census) [Indirect]
	16	2009.2	2010 MICS-HHS (Survey) [other DHS Direct]
	16	2013.3	2014 MICS (Survey) [other DHS Direct]
Suriname	55	2021.5	
	1	2004.1	2004 Census (Census) [Others Direct]
	1	2012.1	2012 Census (Census) [Others Direct]
	16	2016.9	2018 MICS (Survey) [other DHS Direct]
	37	2021.5	Register (Register) [VR]
Sweden	146	2022.5	
	73	2022.5	Estimates (Estimate) [VR]
	73	2022.5	Register (Register) [VR]
Switzerland	146	2022.5	
	73	2022.5	Estimates (Estimate) [VR]
	73	2022.5	Register (Register) [VR]
Syrian Arab Republic	69	2007.5	
	1	1970.2	1970 Census (Census) [Others Direct]
	3	1977.5	1976-1979 PFS (Panel) [Others Direct]
	14	1977	1978 WFS (Survey) [other DHS Direct]
	1	1981.2	1981 Census (Census) [Others Direct]
	16	1992	1993 MCHS (Survey) [other DHS Direct]

Continued on next page

Table 18 – continued from previous page

Country/Area	# obs.	Most recent obs. year	Data series name [source type]
	1	1999.5	2000 MICS (Survey) [other DHS Direct]
	16	2000.4	2001 FHS (Survey) [other DHS Direct]
	1	2004.1	2004 Census (Census) [Others Direct]
	15	2005.9	2006 MICS (Survey) [Indirect]
	1	2007.5	2009 FHS (Survey) [other DHS Direct]
Tajikistan	49	2019.5	
	1	2002	2003 LSMS (Survey) [Others Direct]
	16	2011.1	2012 DHS (Survey) [DHS Direct]
	13	2016.2	2017 DHS (Survey) [DHS Direct]
	19	2019.5	Register (Register) [VR]
Thailand	100	2022	
	2	1959.8	1960 Census (Census) [Indirect]
	5	1969.7	1970 Census (Census) [Indirect]
	1	1972.5	1974-1975 SPC (Survey) [Indirect]
	1	1973.4	1975-1976 SPC (Survey) [Others Direct]
	14	1973.8	1975 WFS (Survey) [Indirect]
	1	1977.9	1978 CPS (Survey) [Others Direct]
	1	1980.8	1981 CPS (Survey) [Others Direct]
	1	1984	1984 CPS (Survey) [Others Direct]
	1	1985.6	1985-1986 SPC (Survey) [Others Direct]
	16	1985.8	1987 DHS (Survey) [DHS Direct]
	1	1989	1989 SPC (Survey) [Others Direct]
	1	1990.5	1991 SPC (Survey) [Others Direct]
	1	1995.5	1995-1996 SPC (Survey) [Others Direct]
	1	1996.5	1996-1997 CPS (Survey) [Others Direct]
	1	2005	2005-2006 MICS (Survey) [other DHS Direct]
	1	2005.5	2006 RHS (Survey) [other DHS Direct]
	1	2012.3	2012-2013 MICS (Survey) [other DHS Direct]
	1	2015.5	2015-2016 MICS (Survey) [other DHS Direct]
	1	2019.1	2019 MICS (Survey) [other DHS Direct]
	1	2022	2022 MICS (Survey) [other DHS Direct]
	47	2021.5	Register (Register) [VR]
Timor-Leste	42	2021.5	
	5	2002	2003 DHS (Survey) [Others Direct]
	2	2004.5	2004 Census (Census) [Indirect]
	16	2008.4	2009-2010 DHS (Survey) [DHS Direct]
	2	2010	2010 Census (Census) [Indirect]
	16	2015.3	2016 DHS (Survey) [DHS Direct]
	1	2021.5	2022 Census (Census) [Others Direct]
Togo	73	2016.3	
	2	1961.5	1961 ED (Survey) [Others Direct]
	2	1969.7	1970 Census (Census) [Indirect]
	16	1987.2	1988 DHS (Survey) [DHS Direct]
	16	1996.8	1998 DHS (Survey) [DHS Direct]
	2	2005.9	2006 MICS (Survey) [Indirect]
	1	2010.3	2010 MICS (Survey) [other DHS Direct]

Continued on next page

Table 18 – continued from previous page

Country/Area	# obs.	Most recent obs. year	Data series name [source type]
	16	2012.6	2013-2014 DHS (Survey) [DHS Direct]
	16	2016.2	2017 MICS (Survey) [other DHS Direct]
	2	2016.3	2017 MIS (Survey) [other DHS Direct]
Tonga	41	2017	
	5	1964.4	1966 Census (Census) [Indirect]
	5	1974.4	1976 Census (Census) [Indirect]
	4	1986.4	1986 Census (Census) [Others Direct]
	4	1996.4	1996 Census (Census) [Others Direct]
	5	2006.4	2006 Census (Census) [Others Direct]
	2	2011.4	2011 Census (Census) [Others Direct]
	4	2010	2012 DHS-NS (Survey) [other DHS Direct]
	1	2017	2019 MICS (Survey) [other DHS Direct]
	11	2003.5	Register (Register) [VR]
Trinidad and Tobago	79	2009.5	
	11	1970	1977 WFS (Survey) [other DHS Direct]
	16	1986	1987 DHS (Survey) [DHS Direct]
	52	2009.5	Register (Register) [VR]
Tunisia	135	2021.5	
	14	1977	1978 WFS (Survey) [other DHS Direct]
	16	1987.1	1988 DHS (Survey) [DHS Direct]
	16	1993.5	1994-1995 MCHS (Survey) [other DHS Direct]
	16	2000	2001 FHS (Survey) [other DHS Direct]
	16	2010.6	2011-2012 MICS (Survey) [other DHS Direct]
	16	2016.8	2018 MICS (Survey) [other DHS Direct]
	41	2021.5	Register (Register) [VR]
Türkiye	156	2022.5	
	14	1977	1978 WFS (Survey) [other DHS Direct]
	5	1981	1983 PHS (Survey) [Others Direct]
	16	1992.2	1993 DHS (Survey) [DHS Direct]
	16	1997.2	1998 DHS (Survey) [DHS Direct]
	16	2002.7	2003 DHS (Survey) [other DHS Direct]
	16	2007.3	2008 DHS (Survey) [other DHS Direct]
	8	2012.4	2013 NDHS (Survey) [other DHS Direct]
	16	2017.4	2018 NDHS (Survey) [other DHS Direct]
	20	2020.5	Estimates (Estimate) [VR]
	29	2022.5	Register (Register) [VR]
Turkmenistan	69	2018	
	5	1999.1	2000 DHS (Survey) [DHS Direct]
	16	2014.4	2015-2016 MICS (Survey) [other DHS Direct]
	16	2018	2019 MICS (Survey) [other DHS Direct]
	32	2006.5	Register (Register) [VR]
Uganda	127	2018.5	
	1	1969.1	1969 Census (Census) [Others Direct]
	16	1987.4	1988-1989 DHS (Survey) [DHS Direct]
	1	1990.5	1991 Census (Census) [Others Direct]

Continued on next page

Table 18 – continued from previous page

Country/Area	# obs.	Most recent obs. year	Data series name [source type]
	16	1993.9	1995 DHS (Survey) [DHS Direct]
	16	1999.5	2000-2001 DHS (Survey) [DHS Direct]
	1	2002.2	2002 Census (Census) [Others Direct]
	16	2005.1	2006 DHS (Survey) [DHS Direct]
	16	2008.5	2009 MIS (Survey) [other DHS Direct]
	16	2010.2	2011 DHS (Survey) [DHS Direct]
	2	2013.5	2014-2015 MIS (Survey) [other DHS Direct]
	1	2013.5	2014 PMA-R1 (Panel) [other DHS Direct]
	2	2014.7	2015 PMA-R3 (Panel) [other DHS Direct]
	16	2015.2	2016 DHS (Survey) [DHS Direct]
	1	2015.3	2016 PMA-R4 (Panel) [other DHS Direct]
	2	2016.3	2017 PMA-R5 (Panel) [other DHS Direct]
	2	2017.5	2018-2019 MIS (Survey) [other DHS Direct]
	1	2017.3	2018 PMA-R6 (Panel) [other DHS Direct]
	1	2018.5	2019 PMA-R6-2 (Panel) [other DHS Direct]
Ukraine	133	2020.5	
	71	2020.5	Estimates (Estimate) [VR]
	62	2018.5	Register (Register) [VR]
United Arab Emirates	34	2022.5	
	1	1975.5	1975 Census (Census) [Others Direct]
	17	2022.5	Estimates (Estimate) [VR]
	16	2004.5	Register (Register) [VR]
United Kingdom	120	2021.5	
	71	2020.5	Estimates (Estimate) [VR]
	49	2021.5	Register (Register) [VR]
United Republic of Tanzania	141	2016.2	
	1	1967.2	1967 Census (Census) [Others Direct]
	1	1973	1973 NDS (Survey) [Others Direct]
	1	1978.2	1978 Census (Census) [Others Direct]
	1	1988.2	1988 Census (Census) [Others Direct]
	16	1990.5	1991-1992 DHS (Survey) [DHS Direct]
	1	1993.1	1994 KAP Survey (Survey) [other DHS Direct]
	1	1994.1	1995 SUMVE In Depth Study (Survey) [other DHS Direct]
	16	1995.2	1996 DHS (Survey) [DHS Direct]
	16	1998.3	1999 RCHS (Survey) [DHS Direct]
	1	2002.1	2002 Census (Census) [Indirect]
	16	2003.5	2004-2005 DHS (Survey) [DHS Direct]
	16	2006.5	2007-2008 AIS (Survey) [other DHS Direct]
	16	2008.7	2010 DHS (Survey) [DHS Direct]
	2	2010.7	2011-2012 AIS (Survey) [other DHS Direct]
	4	2012.1	2012 Census (Census) [Indirect]
	16	2014.4	2015-2016 DHS (Survey) [DHS Direct]
	2	2016.2	2017 MIS (Survey) [other DHS Direct]
	14	2014.5	Estimates (Estimate) [VR]

Continued on next page

Table 18 – continued from previous page

Country/Area	# obs.	Most recent obs. year	Data series name [source type]
United States of America	145	2022.5	
	72	2021.5	Estimates (Estimate) [VR]
	73	2022.5	Register (Register) [VR]
Uruguay	37	2021.5	
	1	1996.4	1996 Census (Census) [Indirect]
	1	2011.8	2011 Census (Census) [Indirect]
	35	2021.5	Register (Register) [VR]
Uzbekistan	94	2022.5	
	16	1995.1	1996 DHS (Survey) [DHS Direct]
	16	2001.3	2002 DHS Special (Survey) [other DHS Direct]
	16	2020.5	2021-2022 MICS (Survey) [other DHS Direct]
	46	2022.5	Register (Register) [VR]
Vanuatu	20	2012	
	1	1966.9	1967 Census (Census) [Others Direct]
	1	1978.5	1979 Census (Census) [Others Direct]
	4	1988.9	1989 Census (Census) [Others Direct]
	4	1999.4	1999 Census (Census) [Indirect]
	5	2009.4	2009 Census (Census) [Indirect]
	5	2012	2013 DHS-NS (Survey) [other DHS Direct]
Venezuela (Bolivarian Republic of)	79	2019.5	
	13	1976	1977 WFS (Survey) [other DHS Direct]
	3	1996	1998 ENPOFAM (Survey) [Others Direct]
	1	2001.8	2001 Census (Census) [Indirect]
	1	2008	2010 ENDEVE (Survey) [Others Direct]
	1	2011.8	2011 Census (Census) [Indirect]
	60	2019.5	Register (Register) [VR]
Viet Nam	195	2020.5	
	6	1986.9	1988 DHS (Survey) [Others Direct]
	16	1988.7	1989 Census (Census) [Indirect]
	2	1993.9	1994 IDS (Survey) [Others Direct]
	16	1996.2	1997 DHS (Survey) [DHS Direct]
	16	1998.7	1999 Census (Census) [Indirect]
	16	2001.4	2002 DHS (Survey) [DHS Direct]
	15	2003	2003 WHS (Survey) [Indirect]
	16	2006.2	2006 MICS (Survey) [Indirect]
	16	2008.8	2009 Census (Census) [Indirect]
	16	2010.5	2010-2011 MICS (Survey) [other DHS Direct]
	16	2012.6	2013-2014 MICS (Survey) [other DHS Direct]
	1	2014	2014 IPHS (Survey) [Others Direct]
	2	2019	2019 Census (Census) [Others Direct]
	16	2019.5	2020-2021 MICS (Survey) [other DHS Direct]
	25	2020.5	Annual APC (Survey) [Others Direct]
Western Sahara	67	2004.2	

Continued on next page

Table 18 – continued from previous page

Country/Area	# obs.	Most recent obs. year	Data series name [source type]
	16	1982.2	1982 Census (Census) [Indirect]
	16	1994.2	1994 Census (Census) [Indirect]
	16	2004.2	2004 Census (Census) [Indirect]
	19	2001.5	Estimates (Estimate) [VR]
Yemen	77	2023.5	
	6	1979	1979 WFS (Survey) [other DHS Direct]
	1	1987.7	1988 Census (Census) [Others Direct]
	16	1990.5	1991-1992 DHS (Survey) [DHS Direct]
	5	1996.4	1997 DHS (Survey) [DHS Direct]
	16	2001.5	2003 FHS (Survey) [other DHS Direct]
	16	2005.2	2006 MICS (Survey) [other DHS Direct]
	16	2012.3	2013 DHS (Survey) [DHS Direct]
	1	2023.5	2022-2023 MICS (Survey) [other DHS Direct]
Zambia	106	2017.3	
	1	1969.2	1969 Census (Census) [Indirect]
	1	1974.2	1974 Sample Census (Survey) [Others Direct]
	2	1980.1	1980 Census (Census) [Indirect]
	2	1990.1	1990 Census (Census) [Indirect]
	16	1990.7	1992 DHS (Survey) [DHS Direct]
	16	1995.3	1996 DHS (Survey) [DHS Direct]
	2	2000.3	2000 Census (Census) [Indirect]
	16	2000.6	2001-2002 DHS (Survey) [DHS Direct]
	16	2006	2007 DHS (Survey) [DHS Direct]
	2	2010.3	2010 Census (Census) [Others Direct]
	16	2012.5	2013-2014 DHS (Survey) [DHS Direct]
	16	2017.3	2018 DHS (Survey) [DHS Direct]
Zimbabwe	155	2021.8	
	2	1968.8	1969 Census (Census) [Others Direct]
	2	1982.1	1982 Census (Census) [Others Direct]
	2	1984.2	1984 RHS (Survey) [Others Direct]
	16	1987.4	1988 DHS (Survey) [DHS Direct]
	1	1992.1	1992 Census (Census) [Others Direct]
	16	1993.2	1994 DHS (Survey) [DHS Direct]
	16	1998.3	1999 DHS (Survey) [DHS Direct]
	1	2002.1	2002 Census (Census) [Others Direct]
	16	2004.4	2005-2006 DHS (Survey) [DHS Direct]
	16	2007.9	2009 MICS (Survey) [other DHS Direct]
	16	2009.5	2010-2011 DHS (Survey) [DHS Direct]
	1	2012.1	2012 Census (Census) [Others Direct]
	16	2012.7	2014 MICS (Survey) [other DHS Direct]
	16	2014.3	2015 DHS (Survey) [DHS Direct]
	1	2017.1	2017 ICDS (Survey) [Others Direct]
	16	2017.7	2019 MICS (Survey) [other DHS Direct]
	1	2021.8	2022 Census (Census) [Others Direct]

12 Supplementary figures

- Figure 15: ASFR 10–14 estimates and uncertainty intervals during 1950–2023, by country (page 111–313).
- Figure 16: ASFR 15–19 estimates and uncertainty intervals during 1950–2023, by country (page 313–515).

Figure 15: **ASFR 10–14 estimates and uncertainty intervals during 1950–2023, by country.** Red curves and shades: ASFR 10–14 median estimates and 95% uncertainty intervals. Black curves: GBD 2021 estimates. Dots with connection lines: input data series differentiated by colors. Shades around the data series: sampling errors (for non-VR data) or stochastic errors (for VR data). Bayesian model elements results are shown at the bottom of each page.

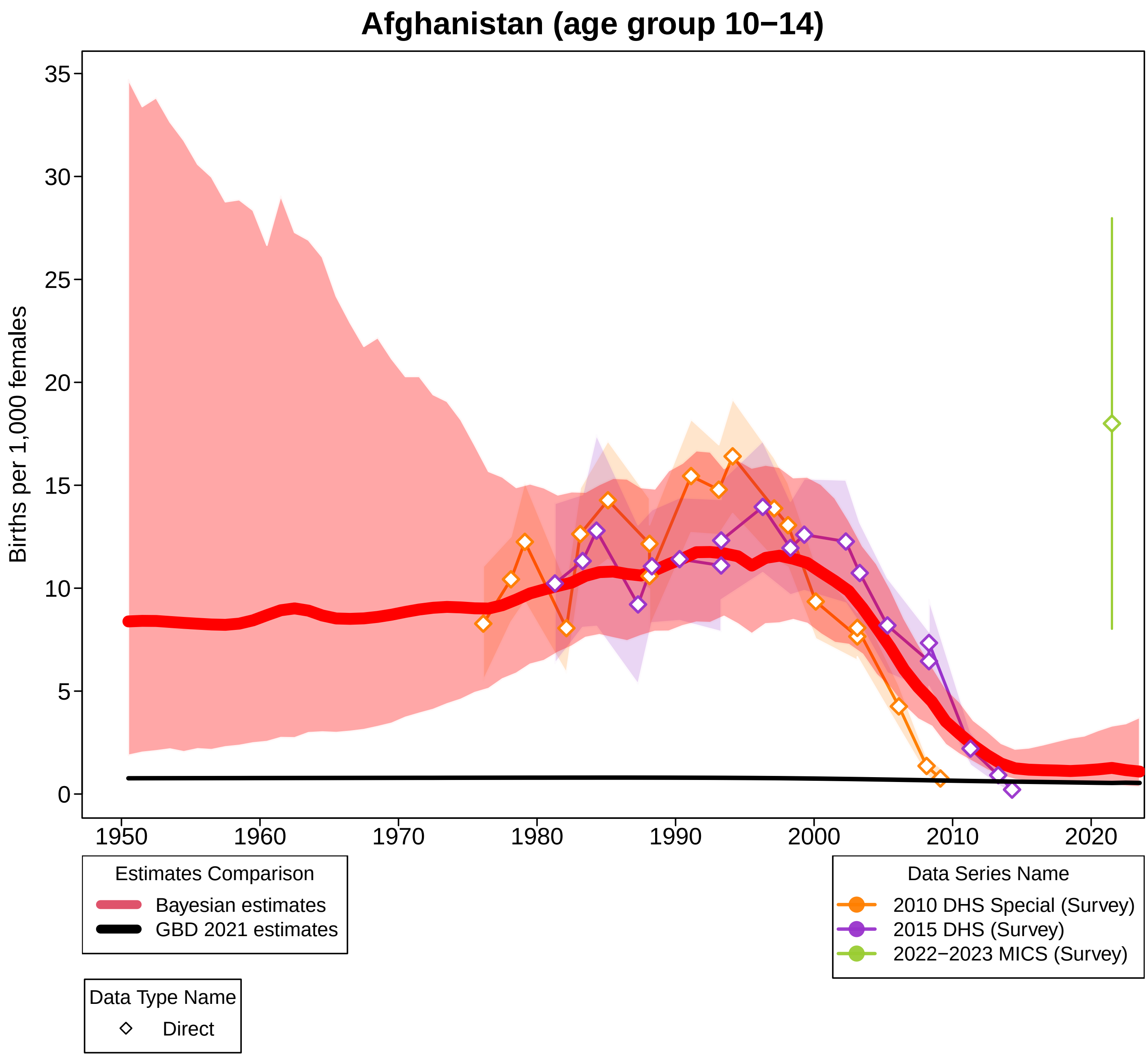

Afghanistan (age group 10−14)
Births per 1,000 females
35
30
25
20
15
10
5
0
1950
1960
1970
1980
1990
2000
2010
2020
Estimates Comparison
Bayesian estimates
GBD 2021 estimates
Data Series Name
2010 DHS Special (Survey)
2015 DHS (Survey)
2022−2023 MICS (Survey)
Data Type Name
Direct

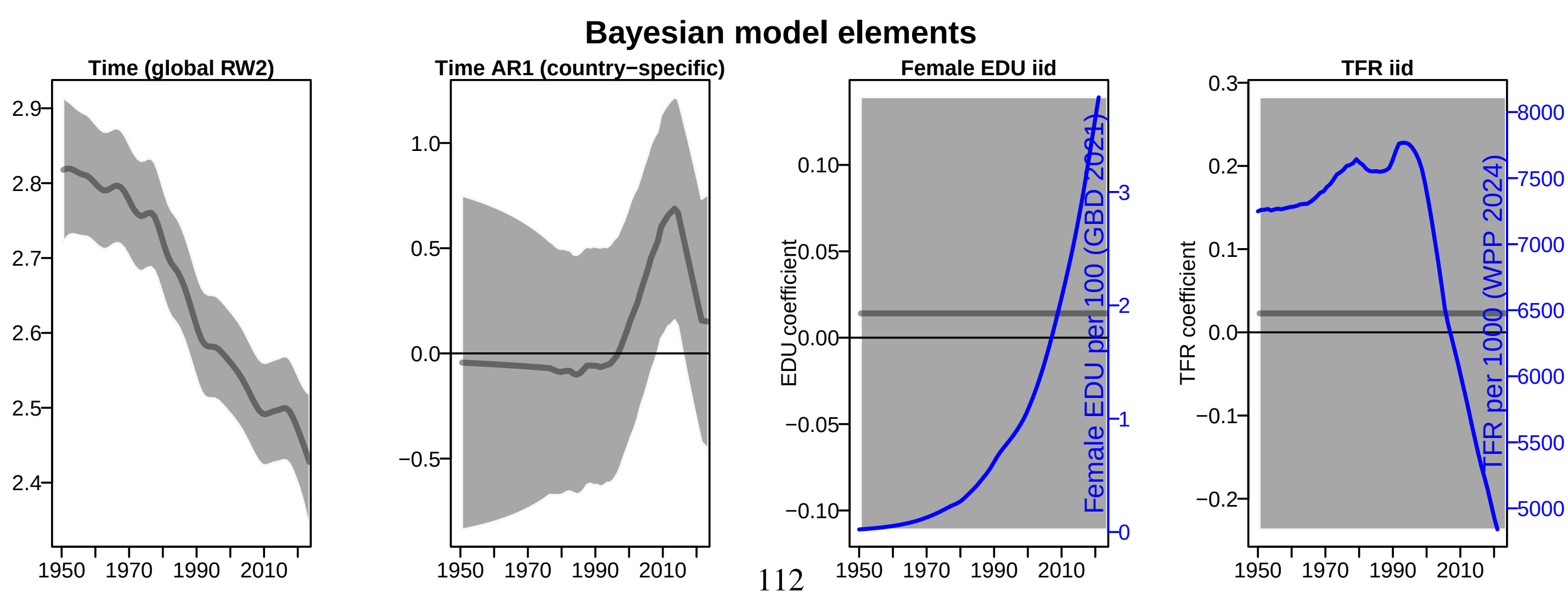

Bayesian model elements
Time (global RW2)
Time AR1 (country−specific)
Female EDU iid
TFR iid
EDU coefficient
Female EDU per 100 (GBD 2021)
TFR coefficient
TFR per 1000 (WPP 2024)
1950
1970
1990
2010

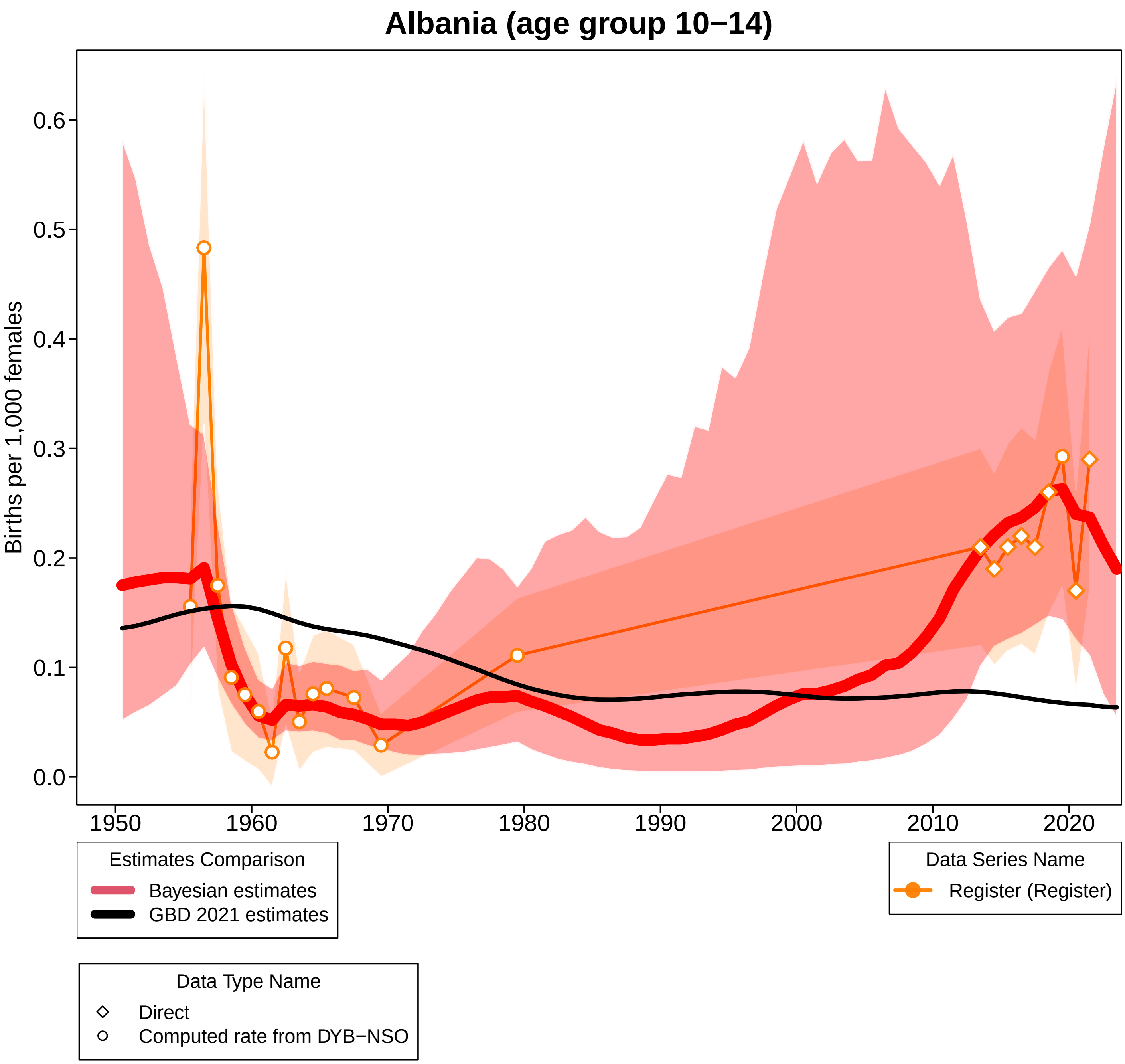
Albania (age group 10−14)
Births per 1,000 females
0.0
0.1
0.2
0.3
0.4
0.5
0.6
1950
1960
1970
1980
1990
2000
2010
2020
Estimates Comparison
Bayesian estimates
GBD 2021 estimates
Data Series Name
Register (Register)
Data Type Name
Direct
Computed rate from DYB−NSO

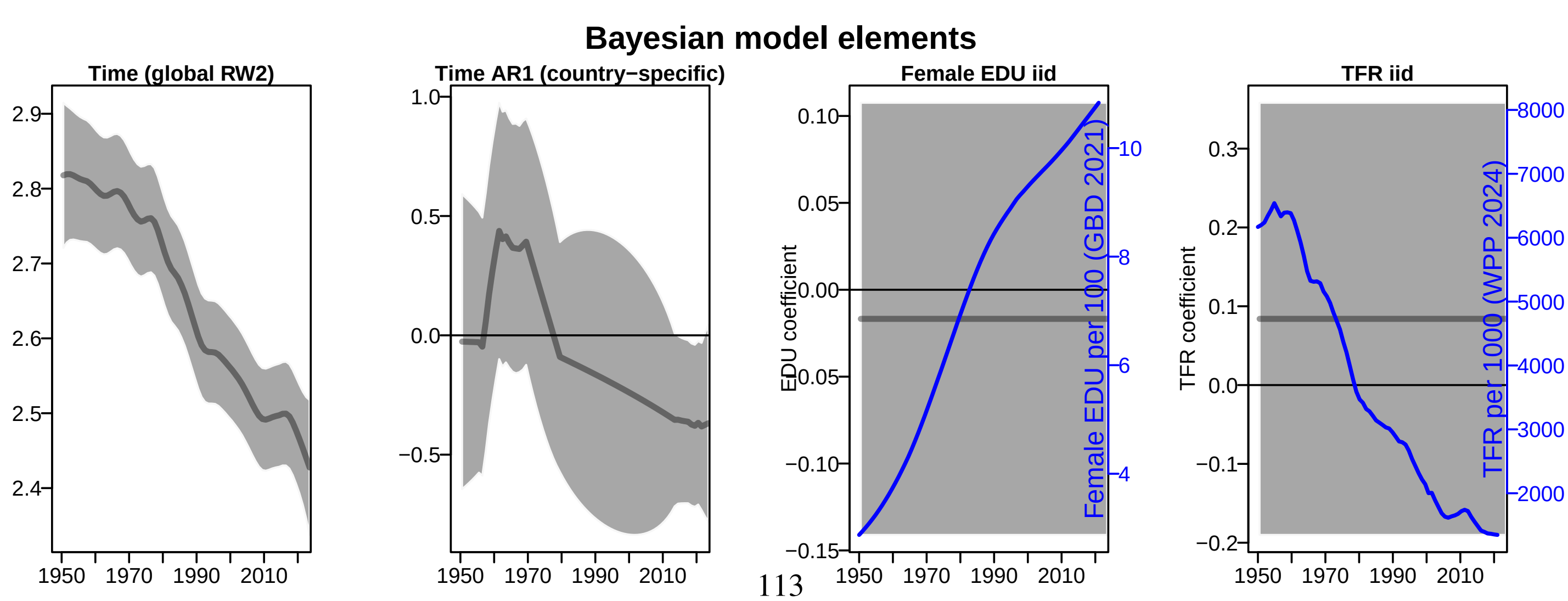
Bayesian model elements
Time (global RW2)
Time AR1 (country−specific)
Female EDU iid
TFR iid
EDU coefficient
Female EDU per 100 (GBD 2021)
TFR coefficient
TFR per 1000 (WPP 2024)

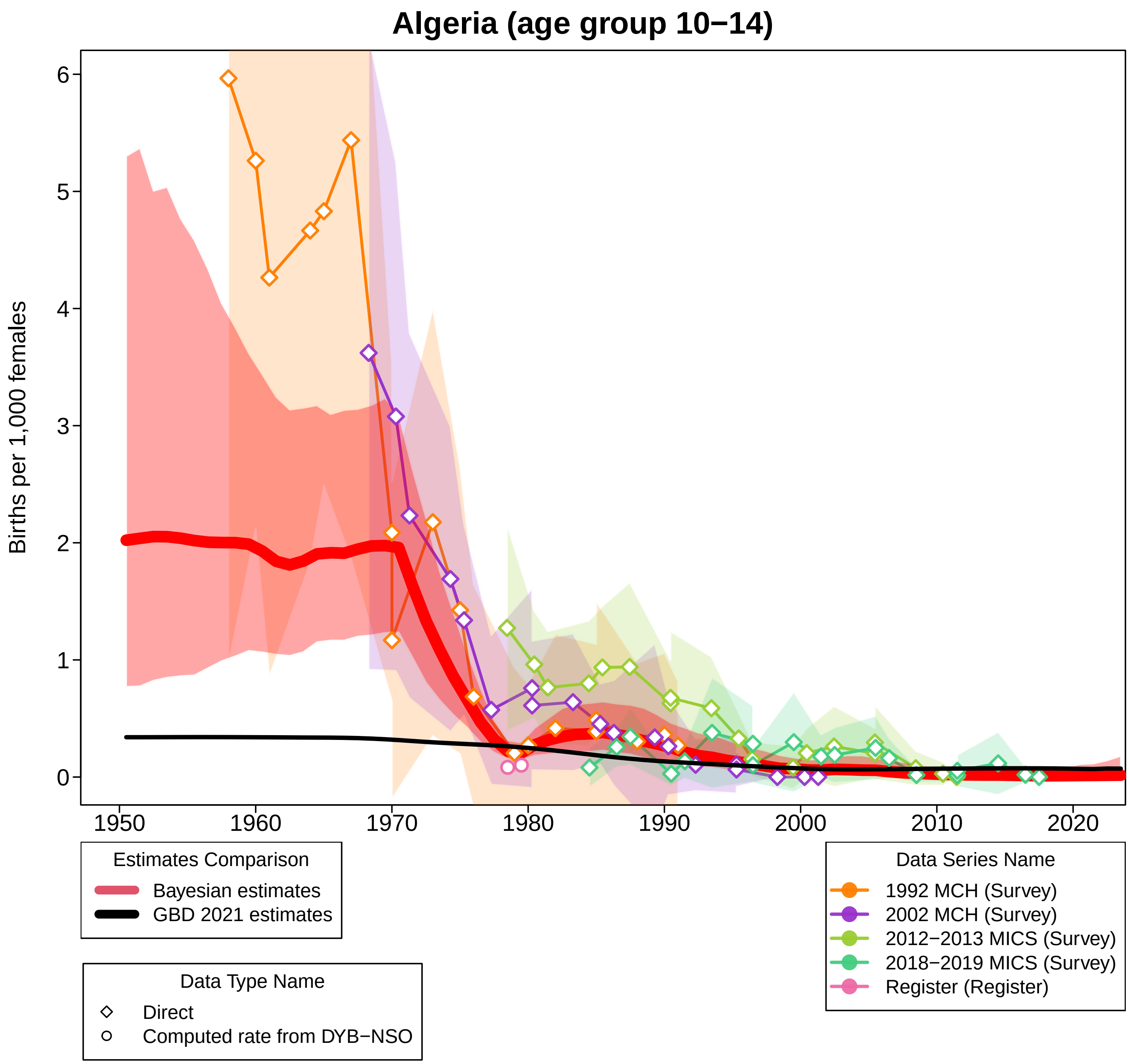
Algeria (age group 10−14)
Births per 1,000 females
0
1
2
3
4
5
6
1950
1960
1970
1980
1990
2000
2010
2020
Estimates Comparison
Bayesian estimates
GBD 2021 estimates
Data Series Name
1992 MCH (Survey)
2002 MCH (Survey)
2012−2013 MICS (Survey)
2018−2019 MICS (Survey)
Register (Register)
Data Type Name
Direct
Computed rate from DYB−NSO

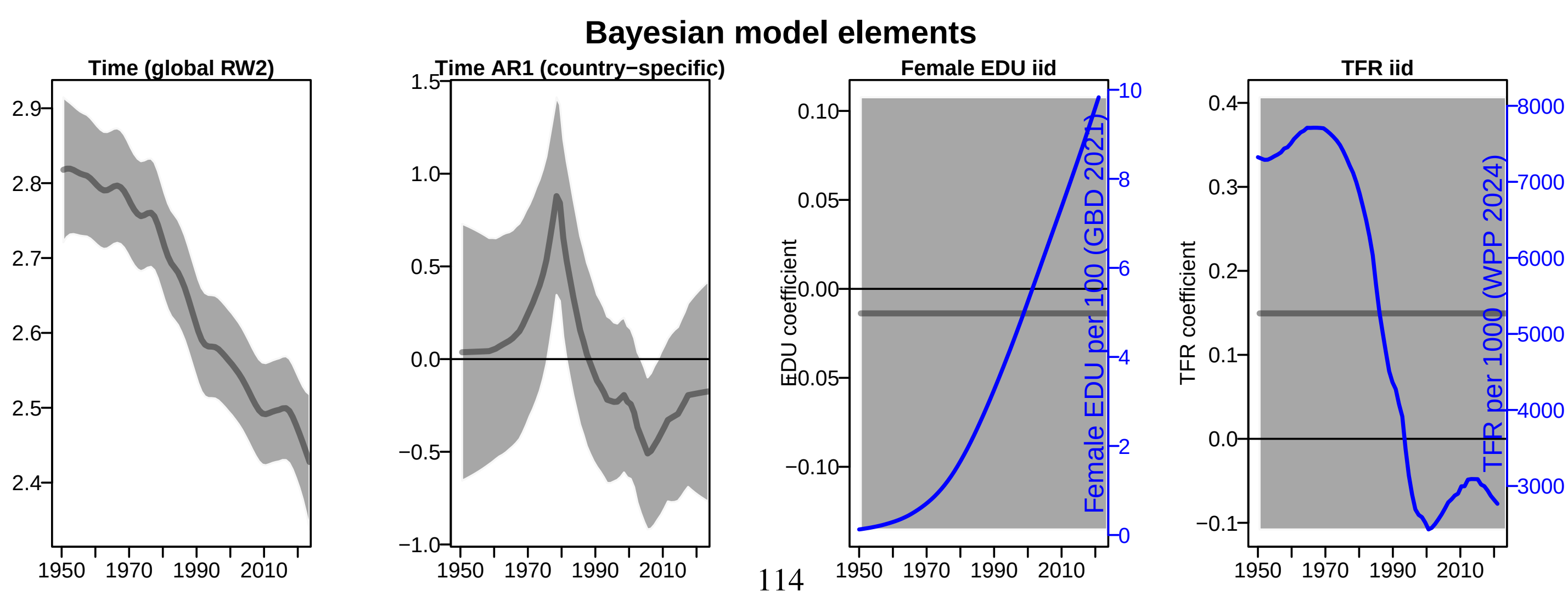
Bayesian model elements
Time (global RW2)
Time AR1 (country−specific)
Female EDU iid
TFR iid
EDU coefficient
Female EDU per 100 (GBD 2021)
TFR coefficient
TFR per 1000 (WPP 2024)
1950
1970
1990
2010

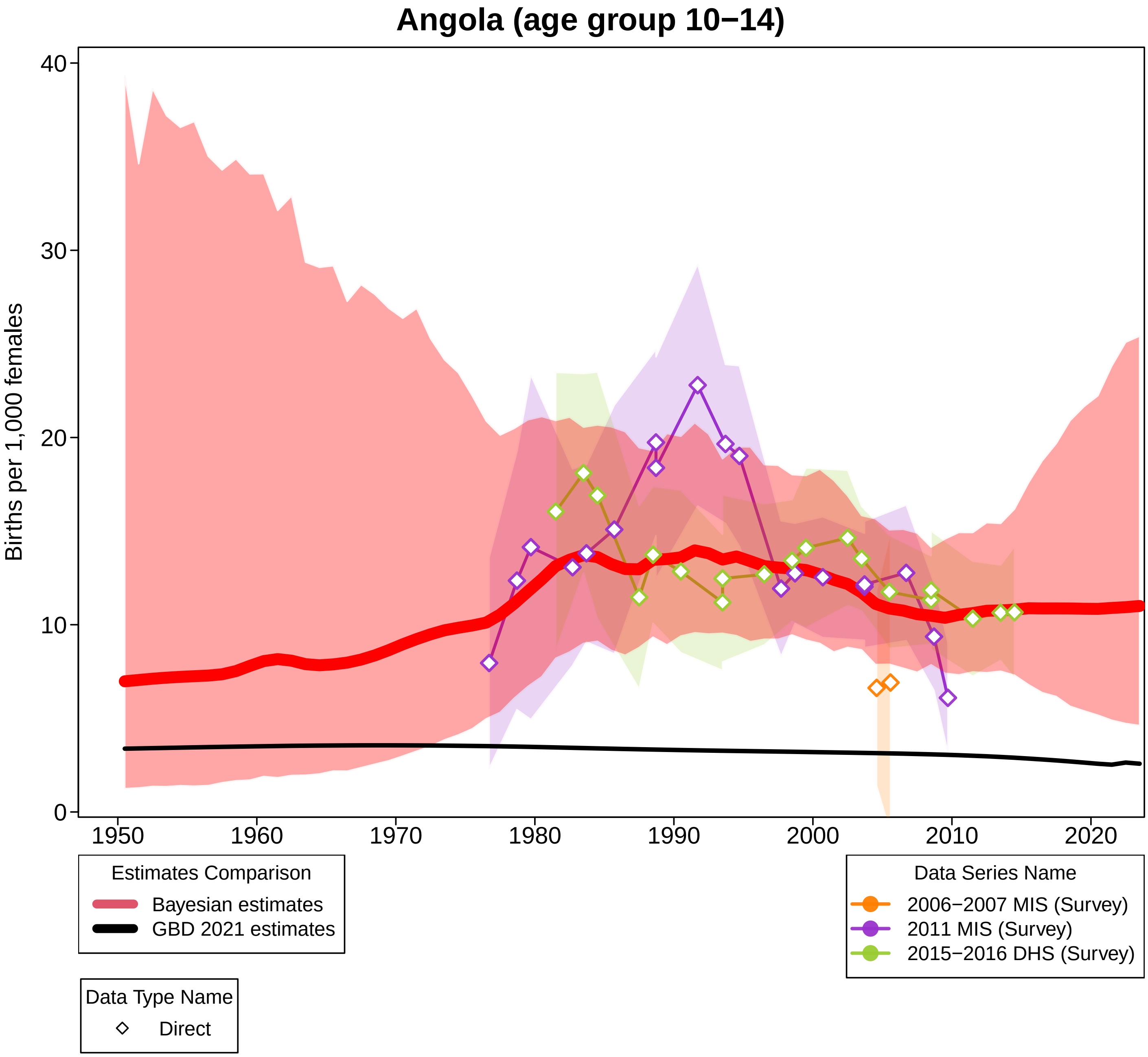
Angola (age group 10−14)
Births per 1,000 females
40
30
20
10
0
1950
1960
1970
1980
1990
2000
2010
2020
Estimates Comparison
Bayesian estimates
GBD 2021 estimates
Data Series Name
2006−2007 MIS (Survey)
2011 MIS (Survey)
2015−2016 DHS (Survey)
Data Type Name
Direct

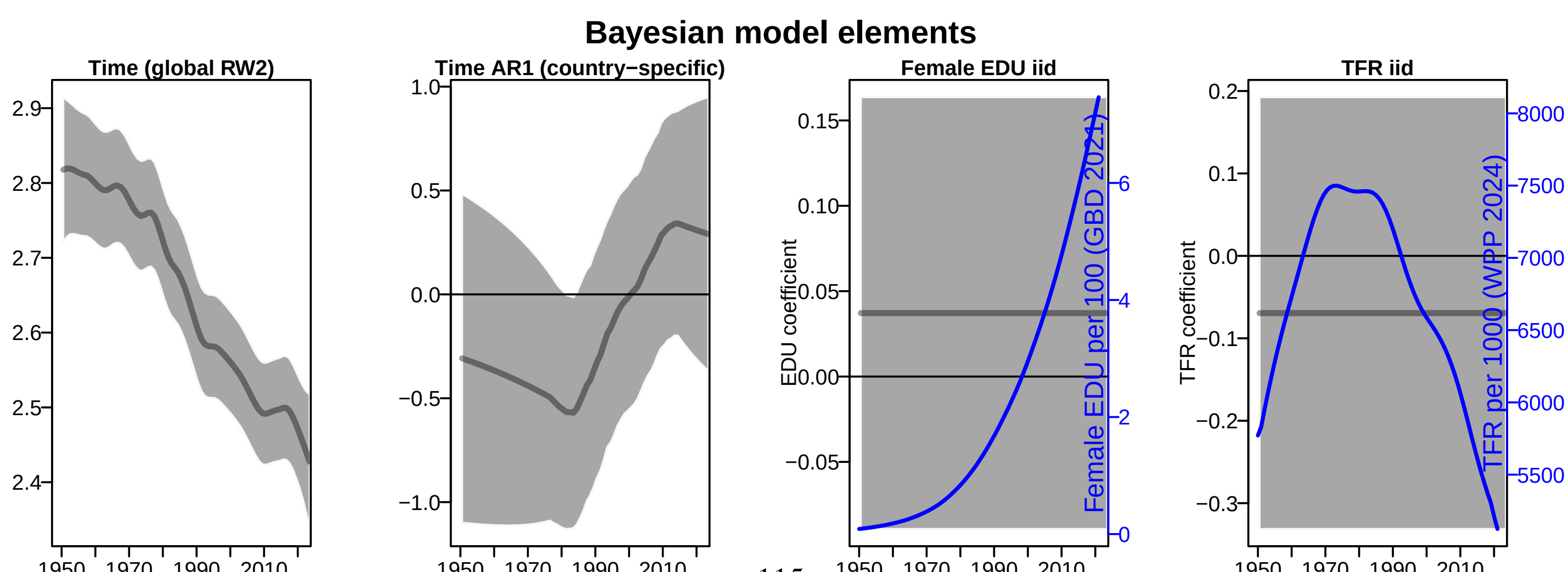
Bayesian model elements
Time (global RW2)
Time AR1 (country−specific)
Female EDU iid
TFR iid
EDU coefficient
Female EDU per 100 (GBD 2021)
TFR coefficient
TFR per 1000 (WPP 2024)
1950
1970
1990
2010

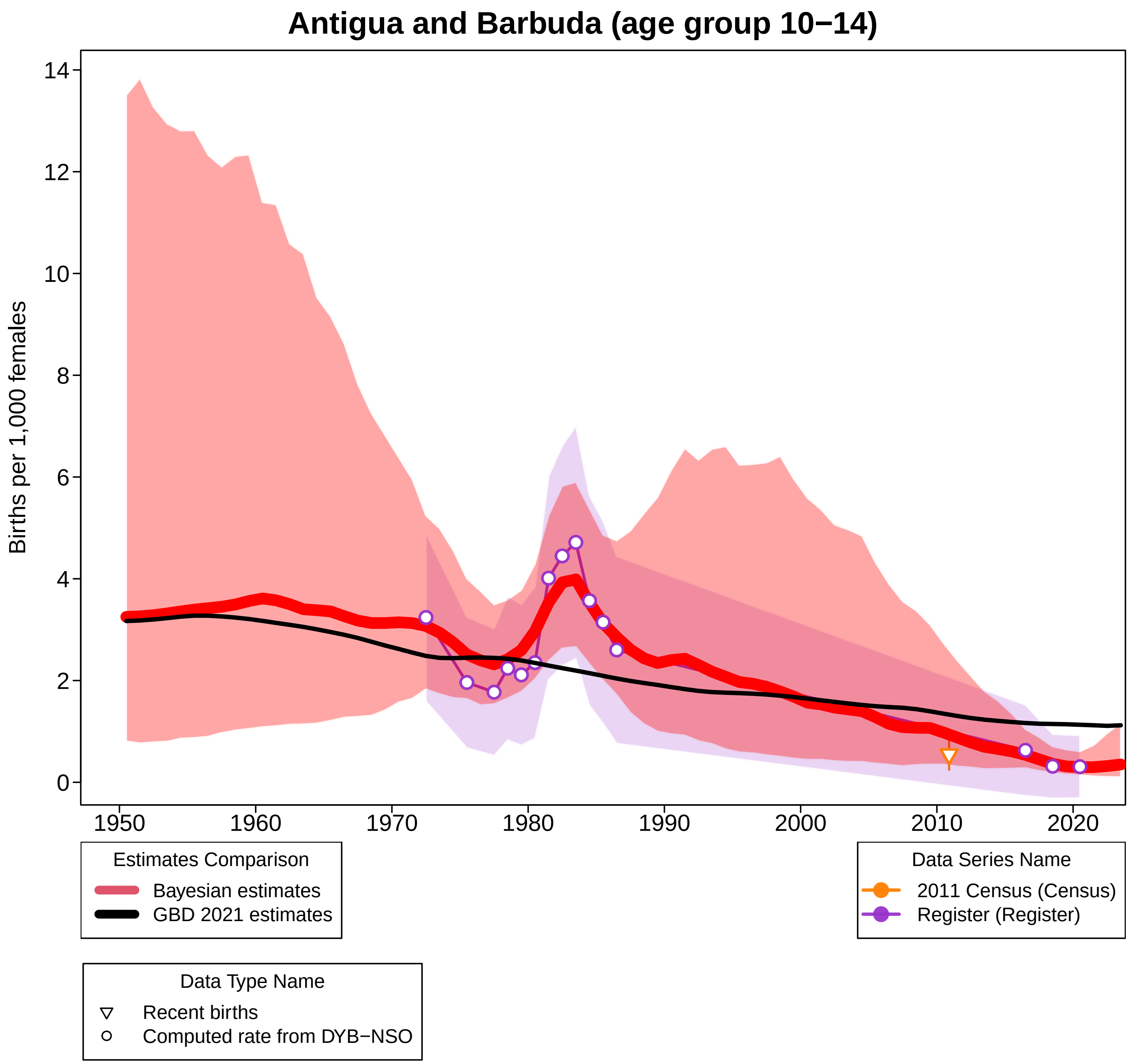

Antigua and Barbuda (age group 10−14)
Births per 1,000 females
0
2
4
6
8
10
12
14
1950
1960
1970
1980
1990
2000
2010
2020
Estimates Comparison
Bayesian estimates
GBD 2021 estimates
Data Series Name
2011 Census (Census)
Register (Register)
Data Type Name
Recent births
Computed rate from DYB−NSO

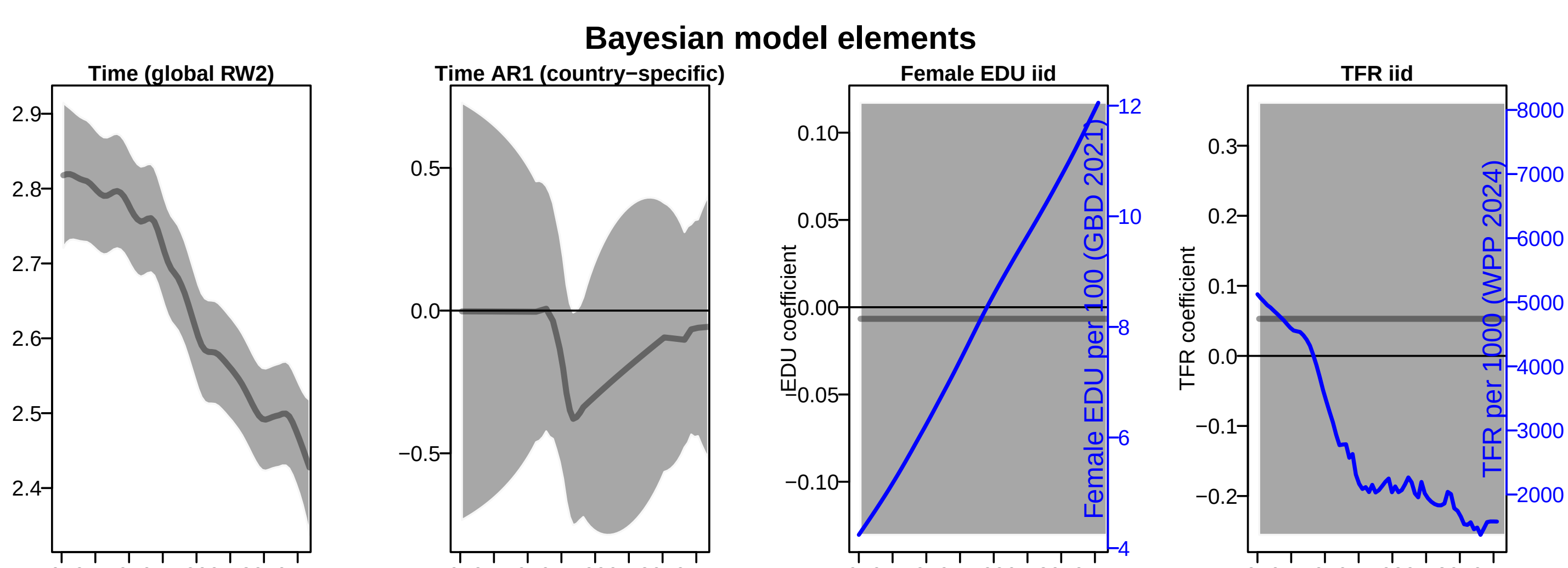

Bayesian model elements
Time (global RW2)
Time AR1 (country−specific)
Female EDU iid
TFR iid
EDU coefficient
Female EDU per 100 (GBD 2021)
TFR coefficient
TFR per 1000 (WPP 2024)
1950
1970
1990
2010

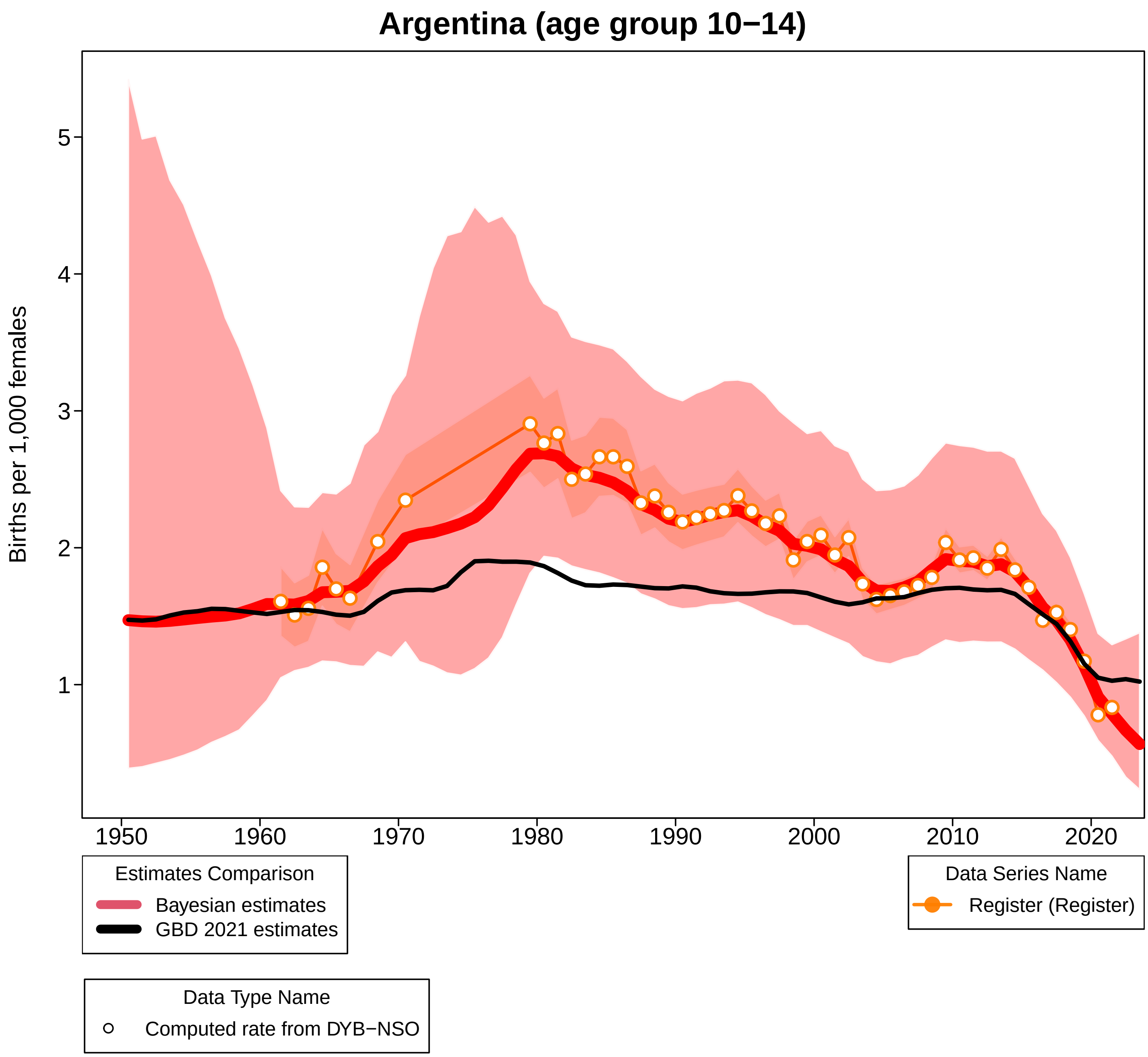
Argentina (age group 10−14)
Births per 1,000 females
5
4
3
2
1
1950
1960
1970
1980
1990
2000
2010
2020
Estimates Comparison
Bayesian estimates
GBD 2021 estimates
Data Series Name
Register (Register)
Data Type Name
Computed rate from DYB−NSO

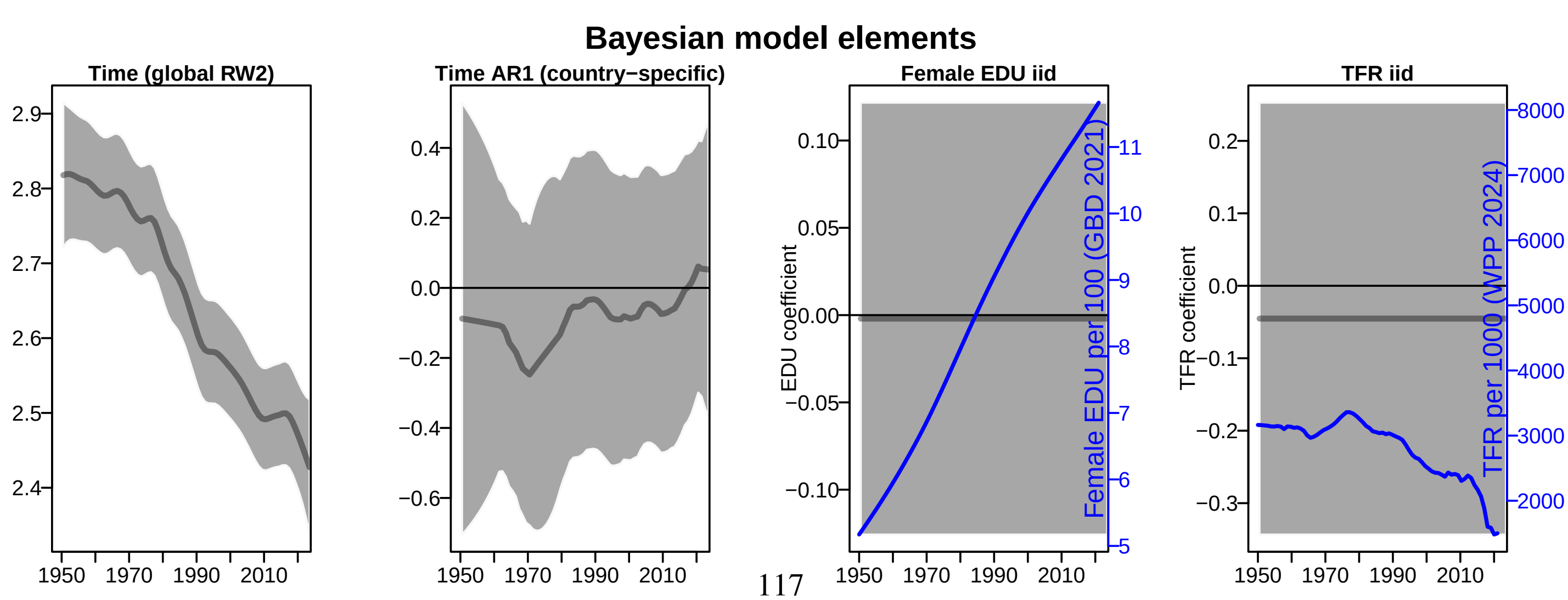
Bayesian model elements
Time (global RW2)
Time AR1 (country−specific)
Female EDU iid
TFR iid
EDU coefficient
Female EDU per 100 (GBD 2021)
TFR coefficient
TFR per 1000 (WPP 2024)
1950
1970
1990
2010

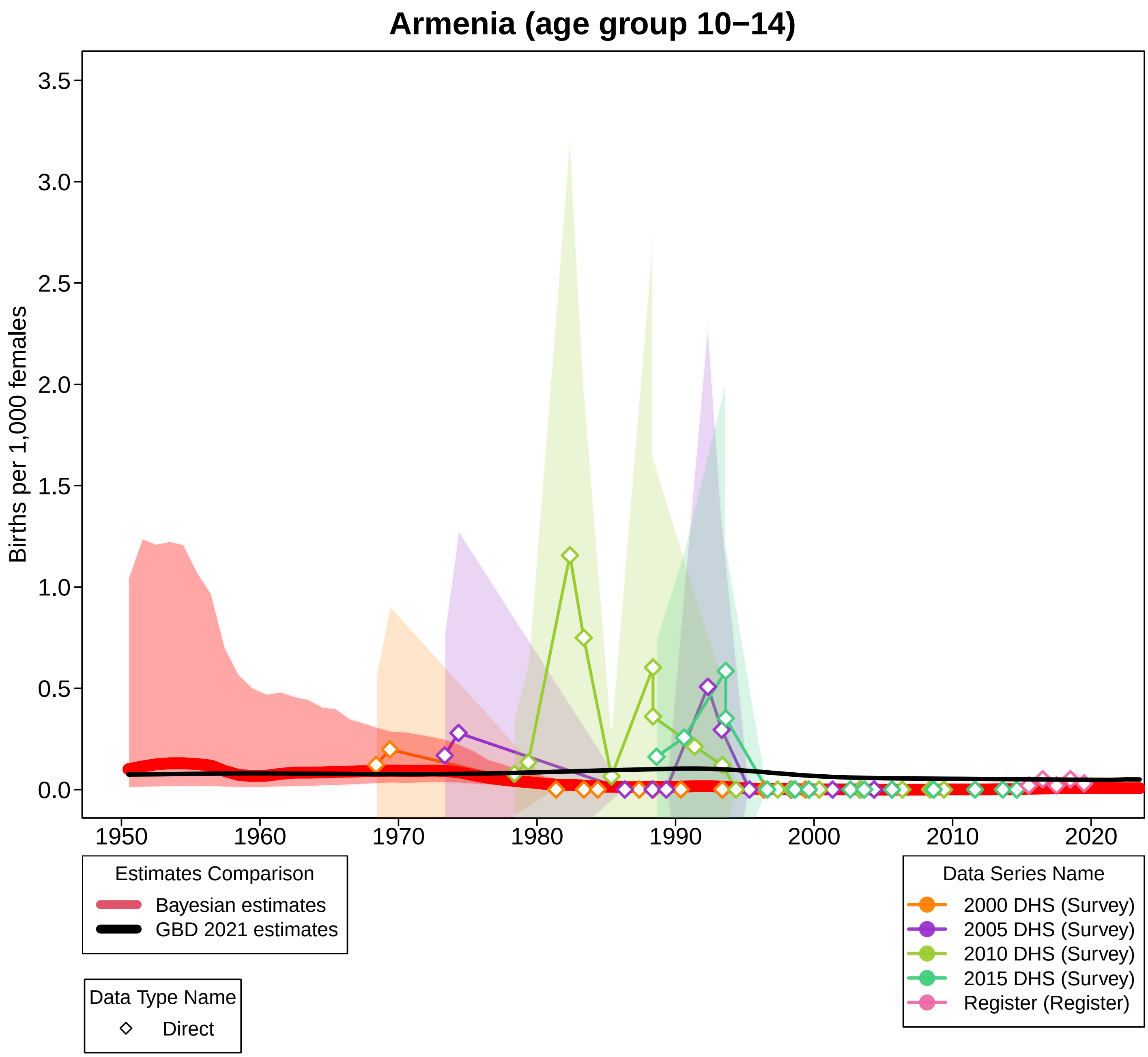
Armenia (age group 10−14)
Births per 1,000 females
3.5
3.0
2.5
2.0
1.5
1.0
0.5
0.0
1950
1960
1970
1980
1990
2000
2010
2020
Estimates Comparison
Bayesian estimates
GBD 2021 estimates
Data Type Name
Direct
Data Series Name
2000 DHS (Survey)
2005 DHS (Survey)
2010 DHS (Survey)
2015 DHS (Survey)
Register (Register)

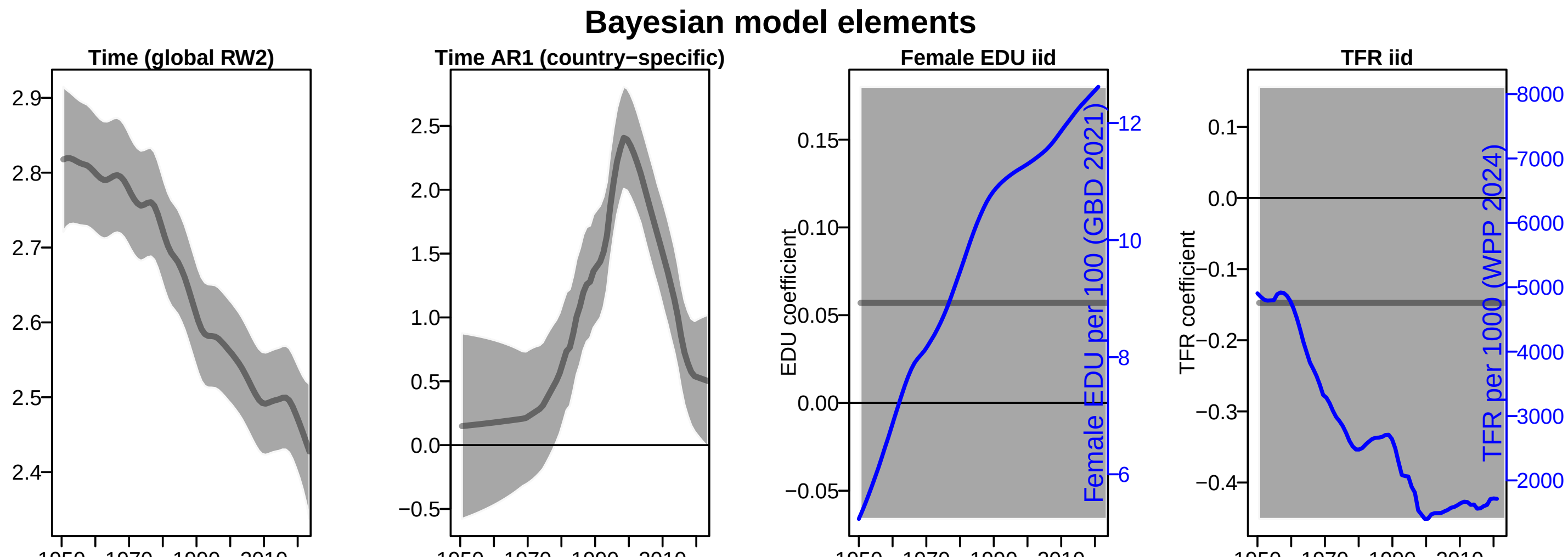
Bayesian model elements
Time (global RW2)
Time AR1 (country−specific)
Female EDU iid
TFR iid
EDU coefficient
Female EDU per 100 (GBD 2021)
TFR coefficient
TFR per 1000 (WPP 2024)
1950
1970
1990
2010

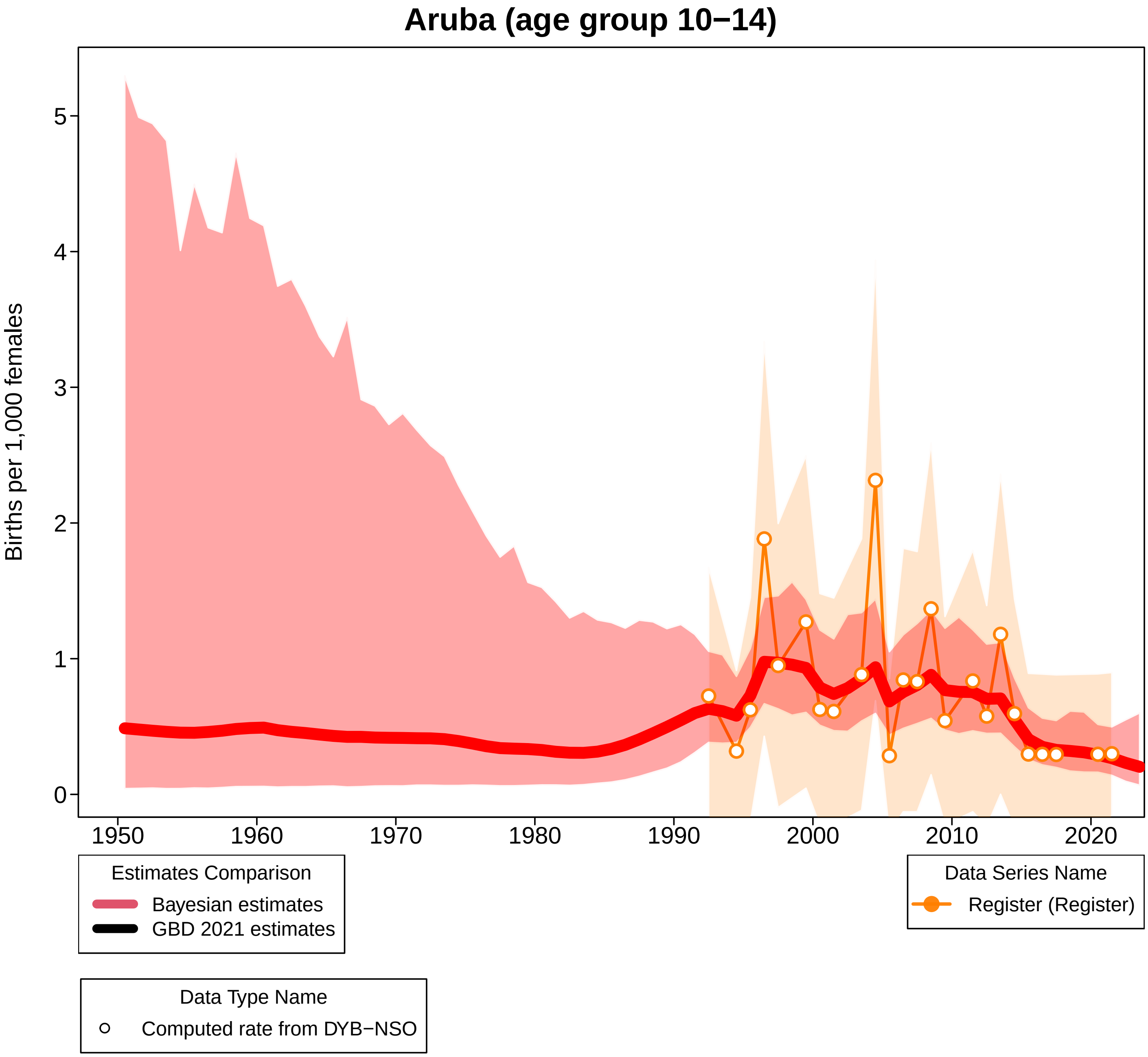
Aruba (age group 10−14)
Births per 1,000 females
0
1
2
3
4
5
1950
1960
1970
1980
1990
2000
2010
2020
Estimates Comparison
Bayesian estimates
GBD 2021 estimates
Data Series Name
Register (Register)
Data Type Name
Computed rate from DYB−NSO

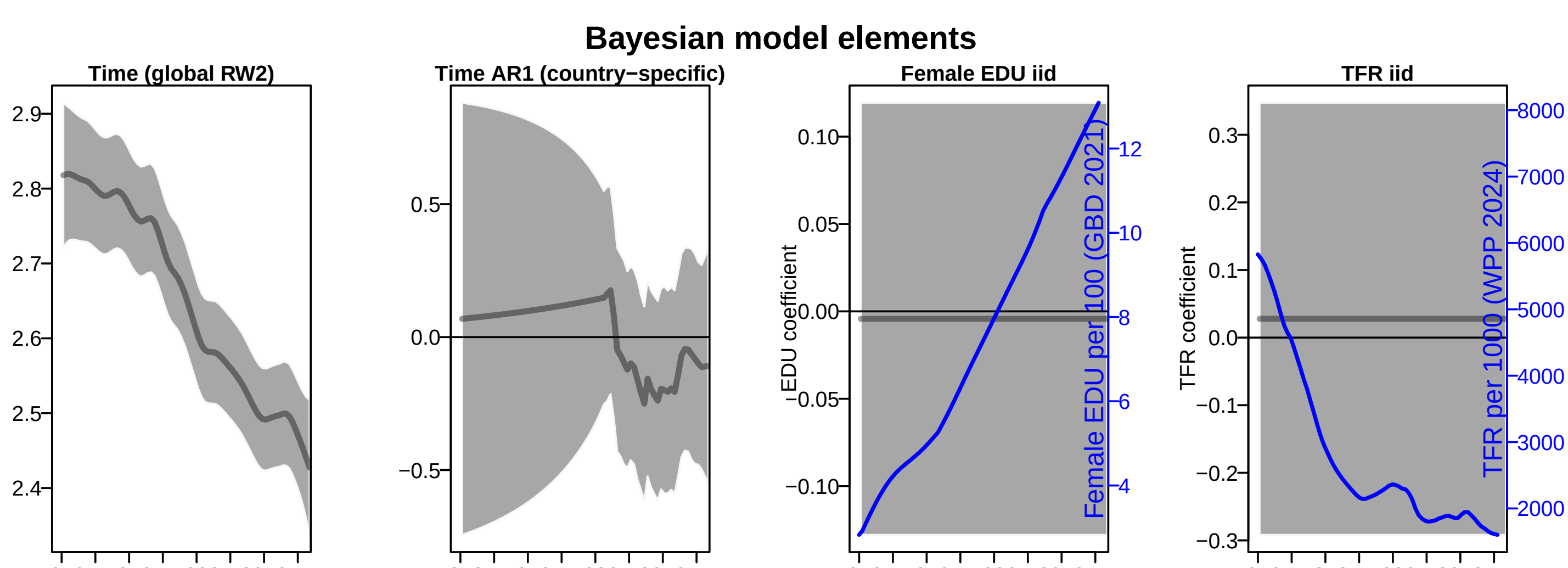
Bayesian model elements
Time (global RW2)
Time AR1 (country−specific)
Female EDU iid
TFR iid
EDU coefficient
Female EDU per 100 (GBD 2021)
TFR coefficient
TFR per 1000 (WPP 2024)
1950
1970
1990
2010

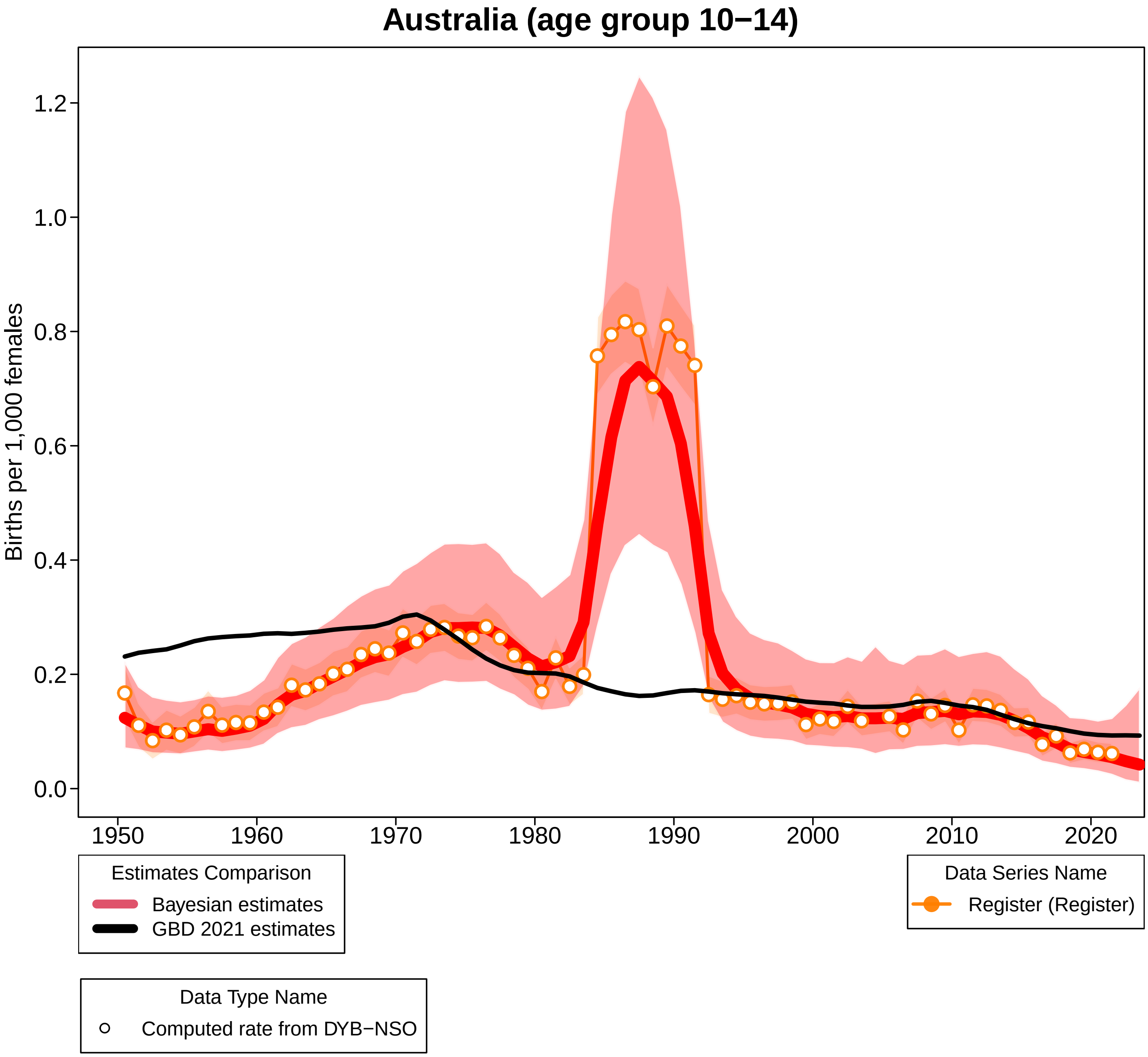
Australia (age group 10−14)
Births per 1,000 females
1.2
1.0
0.8
0.6
0.4
0.2
0.0
1950
1960
1970
1980
1990
2000
2010
2020
Estimates Comparison
Bayesian estimates
GBD 2021 estimates
Data Series Name
Register (Register)
Data Type Name
Computed rate from DYB−NSO

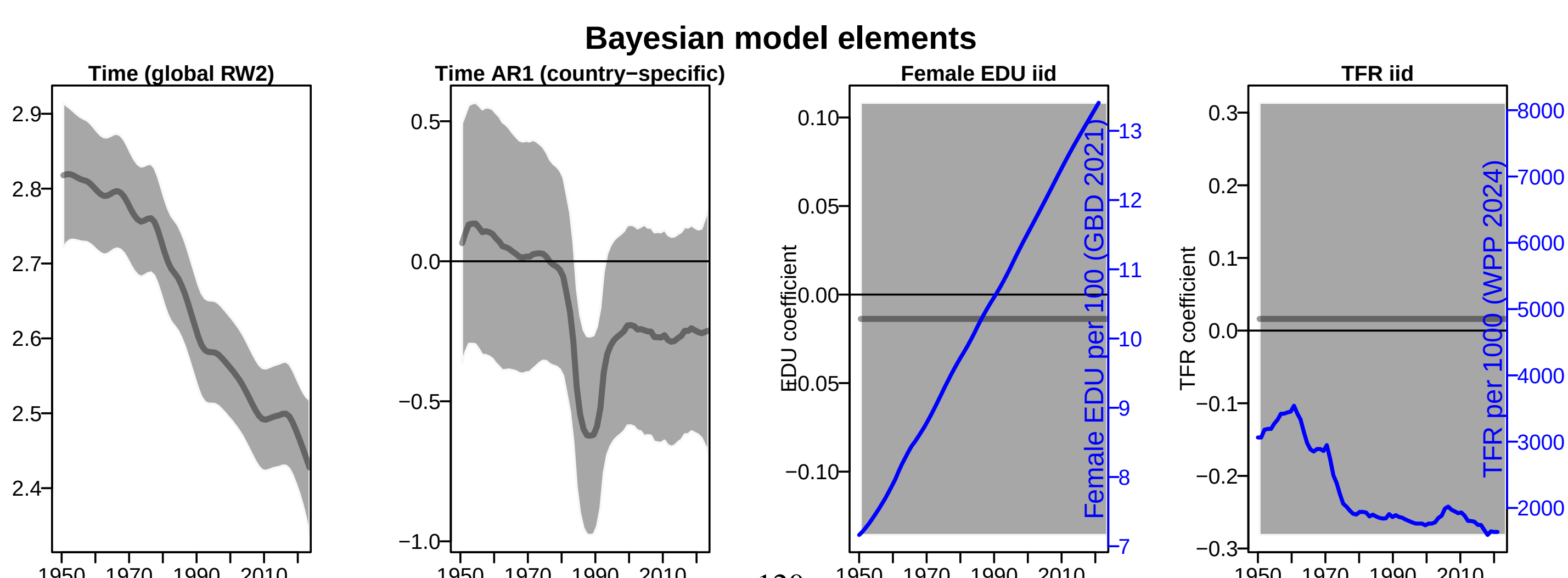
Bayesian model elements
Time (global RW2)
Time AR1 (country−specific)
Female EDU iid
TFR iid
EDU coefficient
Female EDU per 100 (GBD 2021)
TFR coefficient
TFR per 1000 (WPP 2024)
1950
1970
1990
2010

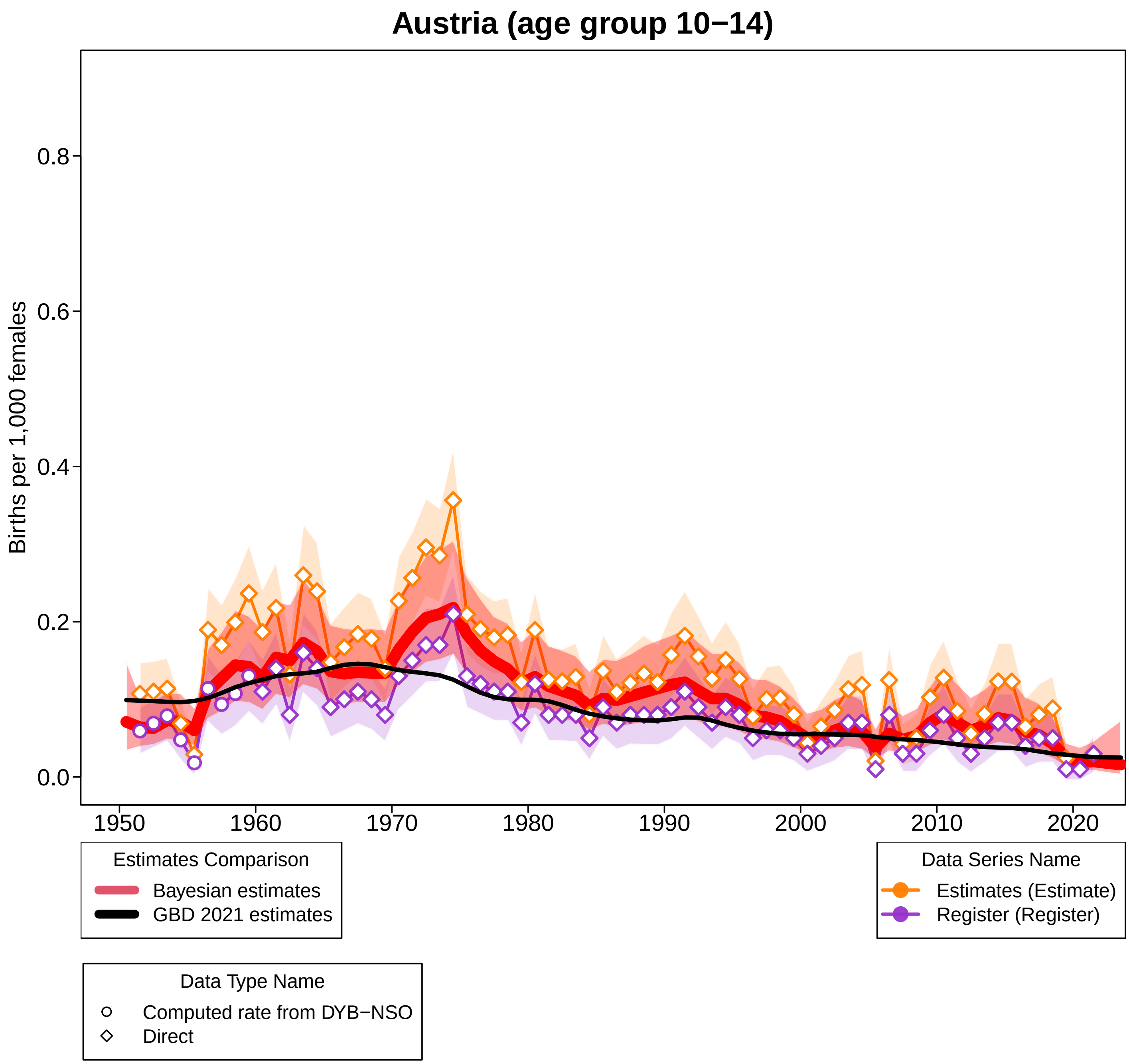
Austria (age group 10−14)
Births per 1,000 females
0.0
0.2
0.4
0.6
0.8
1950
1960
1970
1980
1990
2000
2010
2020
Estimates Comparison
Bayesian estimates
GBD 2021 estimates
Data Series Name
Estimates (Estimate)
Register (Register)
Data Type Name
Computed rate from DYB−NSO
Direct

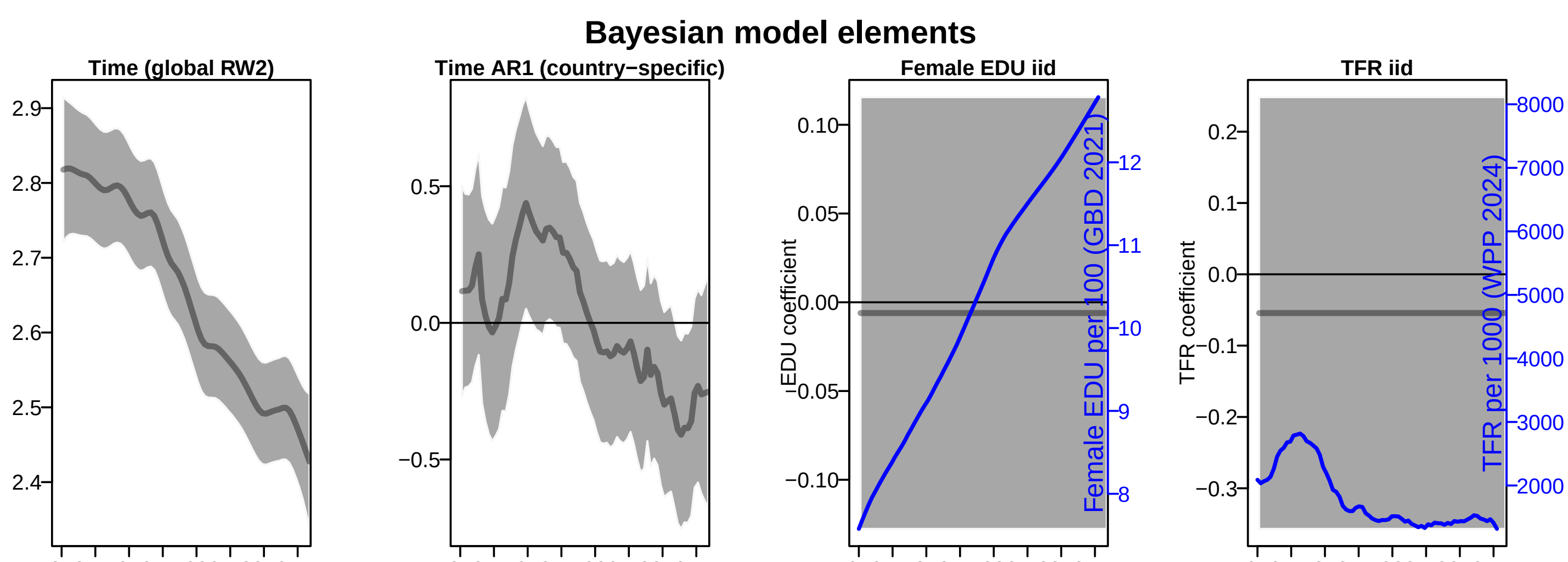
Bayesian model elements
Time (global RW2)
Time AR1 (country−specific)
Female EDU iid
TFR iid
EDU coefficient
Female EDU per 100 (GBD 2021)
TFR coefficient
TFR per 1000 (WPP 2024)
1950
1970
1990
2010

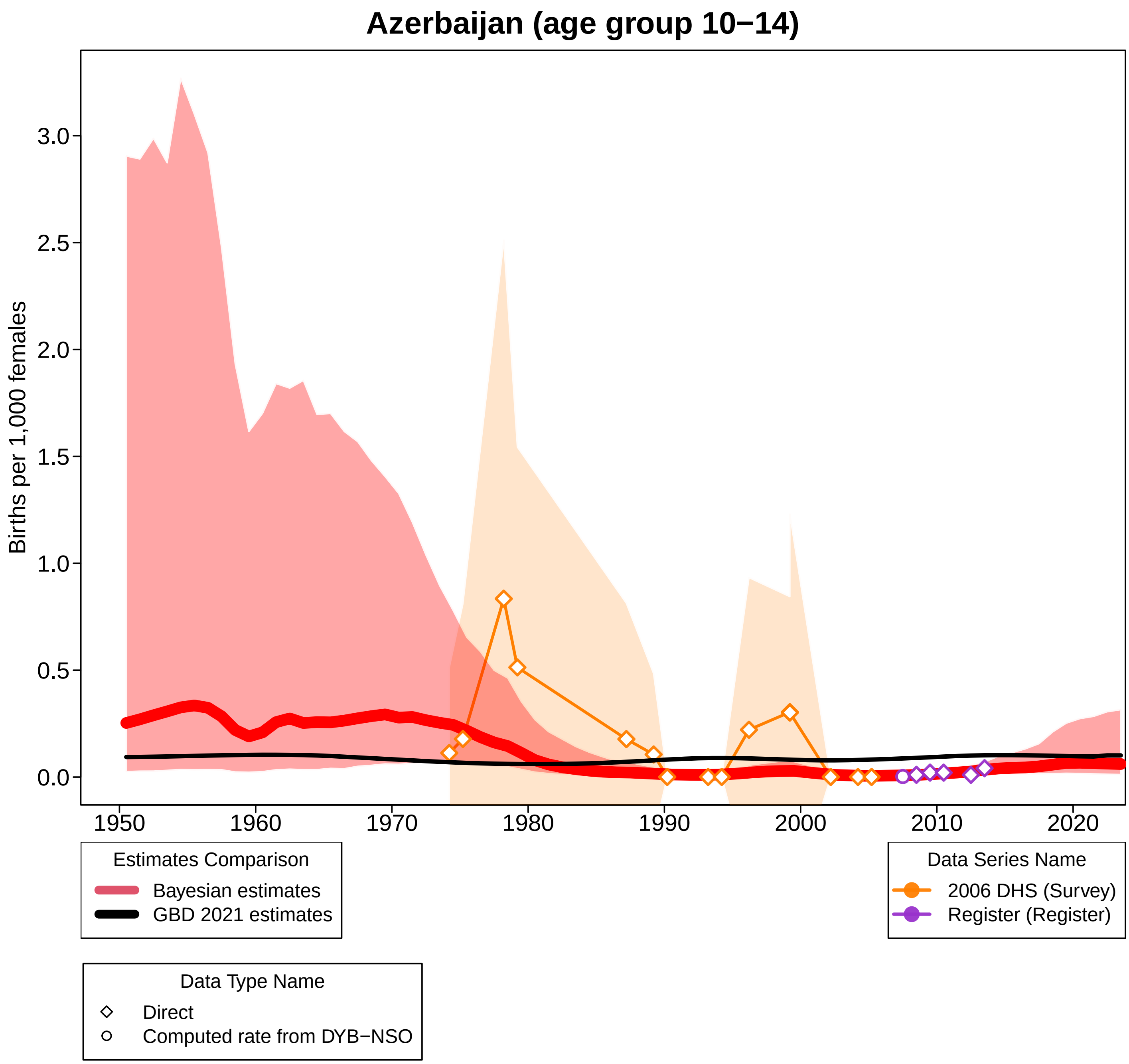

Azerbaijan (age group 10−14)
Births per 1,000 females
3.0
2.5
2.0
1.5
1.0
0.5
0.0
1950
1960
1970
1980
1990
2000
2010
2020
Estimates Comparison
Bayesian estimates
GBD 2021 estimates
Data Series Name
2006 DHS (Survey)
Register (Register)
Data Type Name
Direct
Computed rate from DYB−NSO

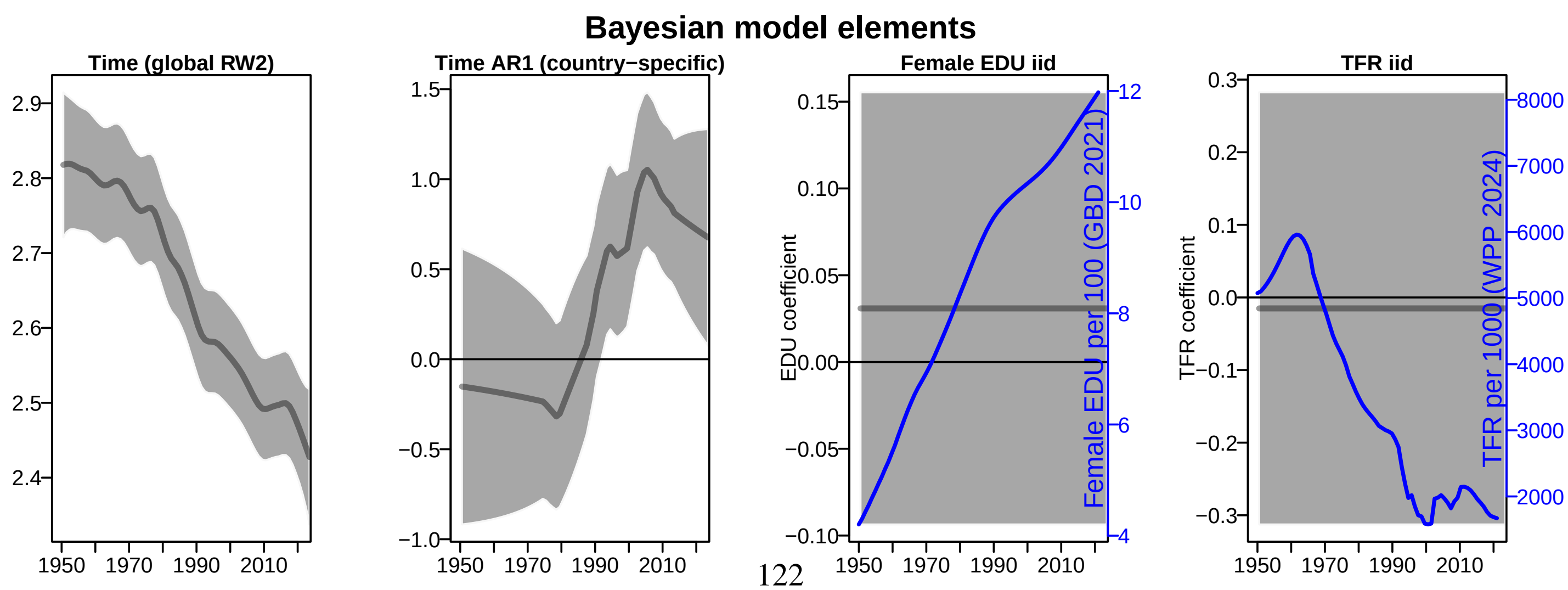

Bayesian model elements
Time (global RW2)
Time AR1 (country−specific)
Female EDU iid
TFR iid
EDU coefficient
Female EDU per 100 (GBD 2021)
TFR coefficient
TFR per 1000 (WPP 2024)
1950
1970
1990
2010

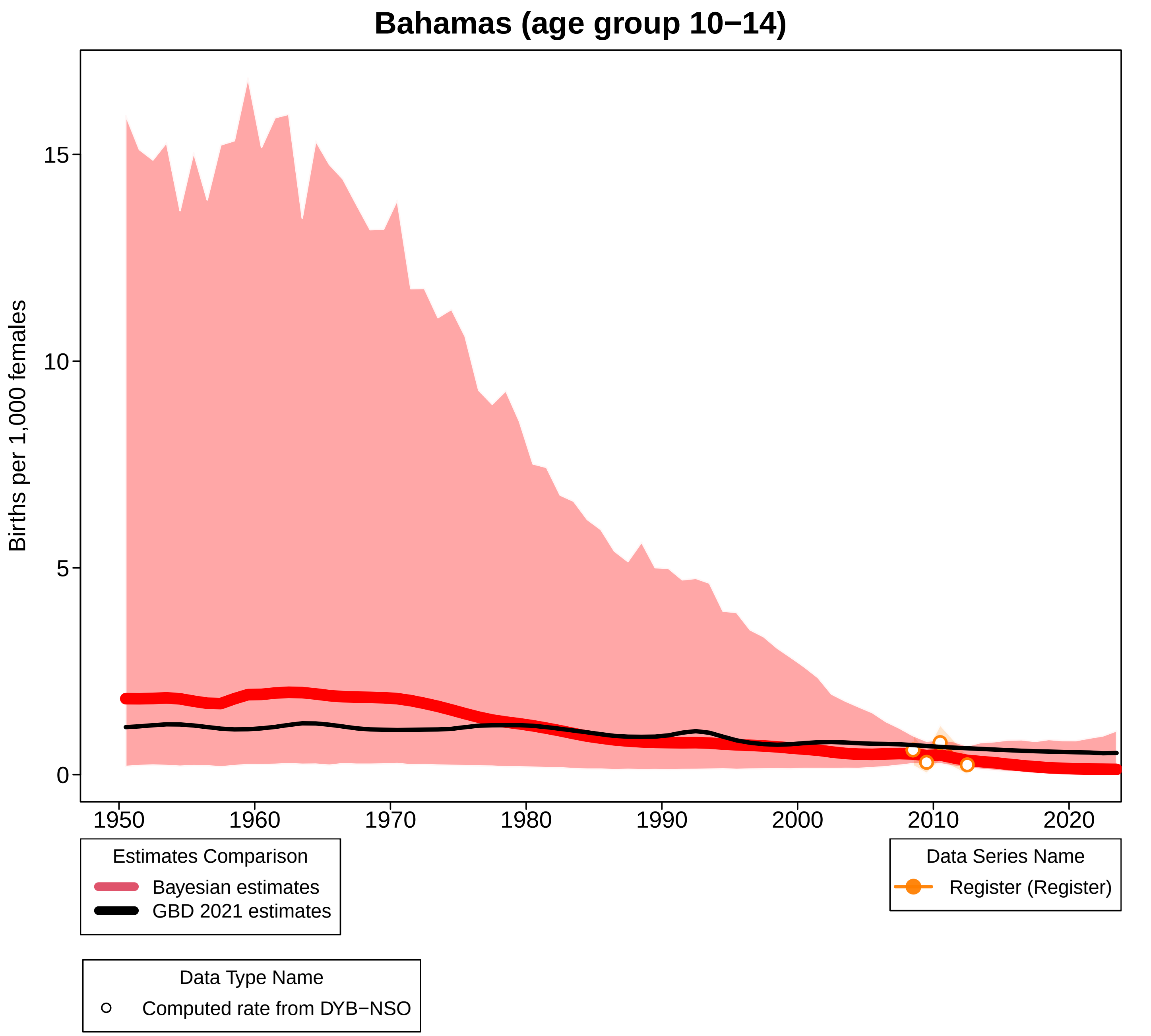
Bahamas (age group 10−14)
Births per 1,000 females
15
10
5
0
1950
1960
1970
1980
1990
2000
2010
2020
Estimates Comparison
Bayesian estimates
GBD 2021 estimates
Data Series Name
Register (Register)
Data Type Name
Computed rate from DYB−NSO

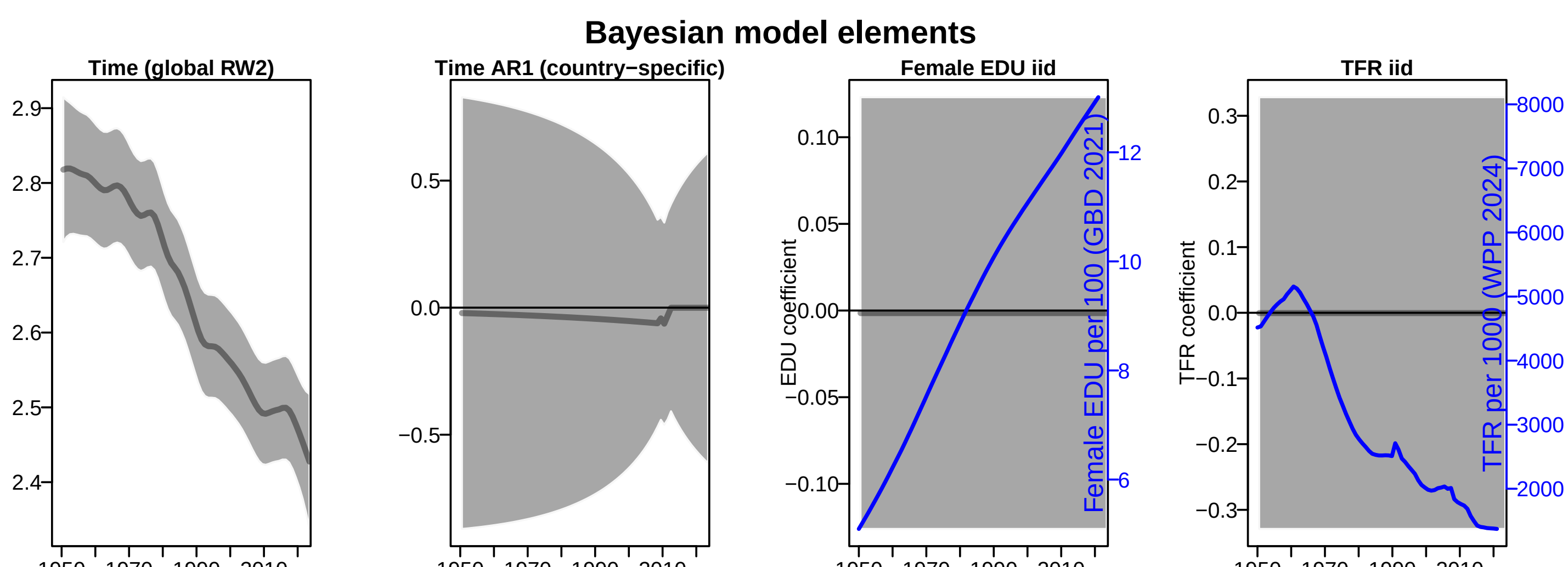
Bayesian model elements
Time (global RW2)
Time AR1 (country−specific)
Female EDU iid
TFR iid
EDU coefficient
Female EDU per 100 (GBD 2021)
TFR coefficient
TFR per 1000 (WPP 2024)
1950
1970
1990
2010

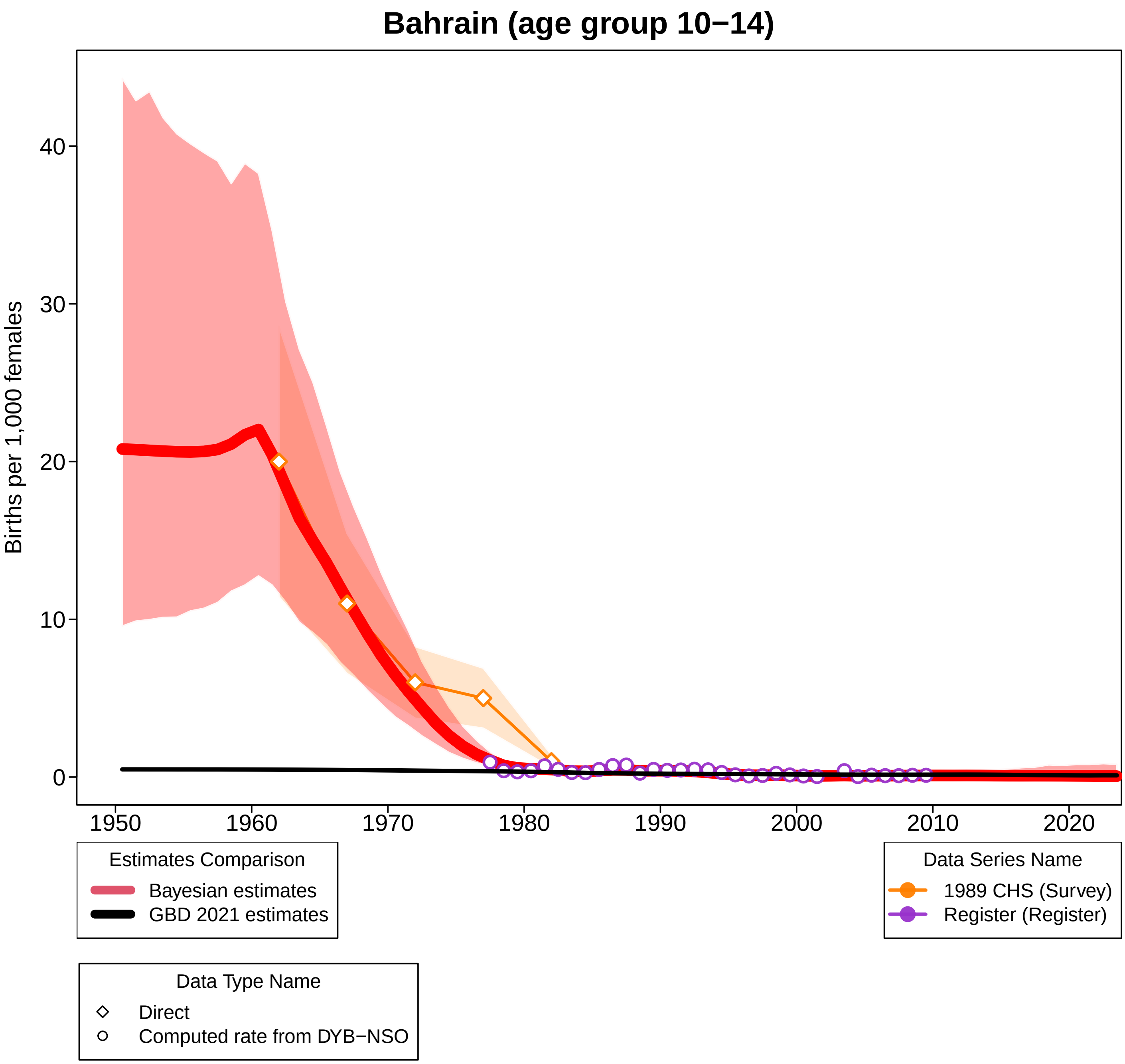
Bahrain (age group 10−14)
Births per 1,000 females
0
10
20
30
40
1950
1960
1970
1980
1990
2000
2010
2020
Estimates Comparison
Bayesian estimates
GBD 2021 estimates
Data Series Name
1989 CHS (Survey)
Register (Register)
Data Type Name
Direct
Computed rate from DYB−NSO

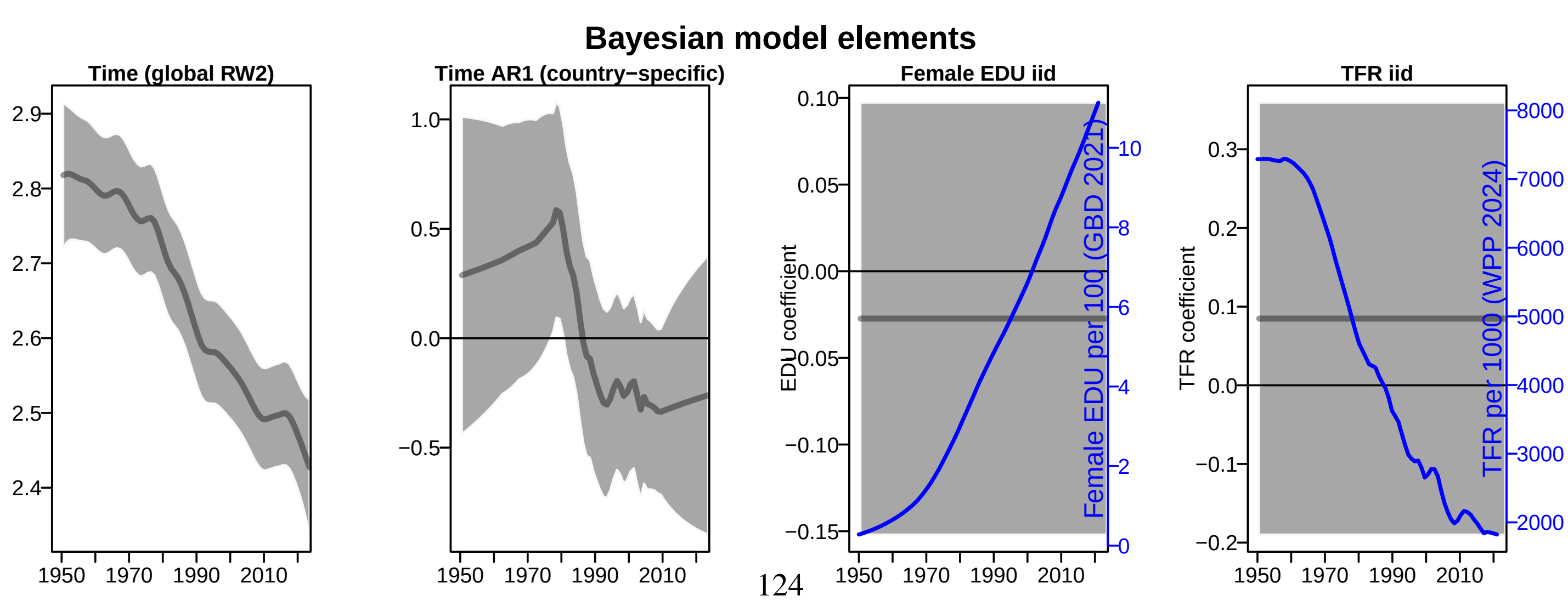
Bayesian model elements
Time (global RW2)
Time AR1 (country−specific)
Female EDU iid
TFR iid
EDU coefficient
Female EDU per 100 (GBD 2021)
TFR coefficient
TFR per 1000 (WPP 2024)
1950
1970
1990
2010

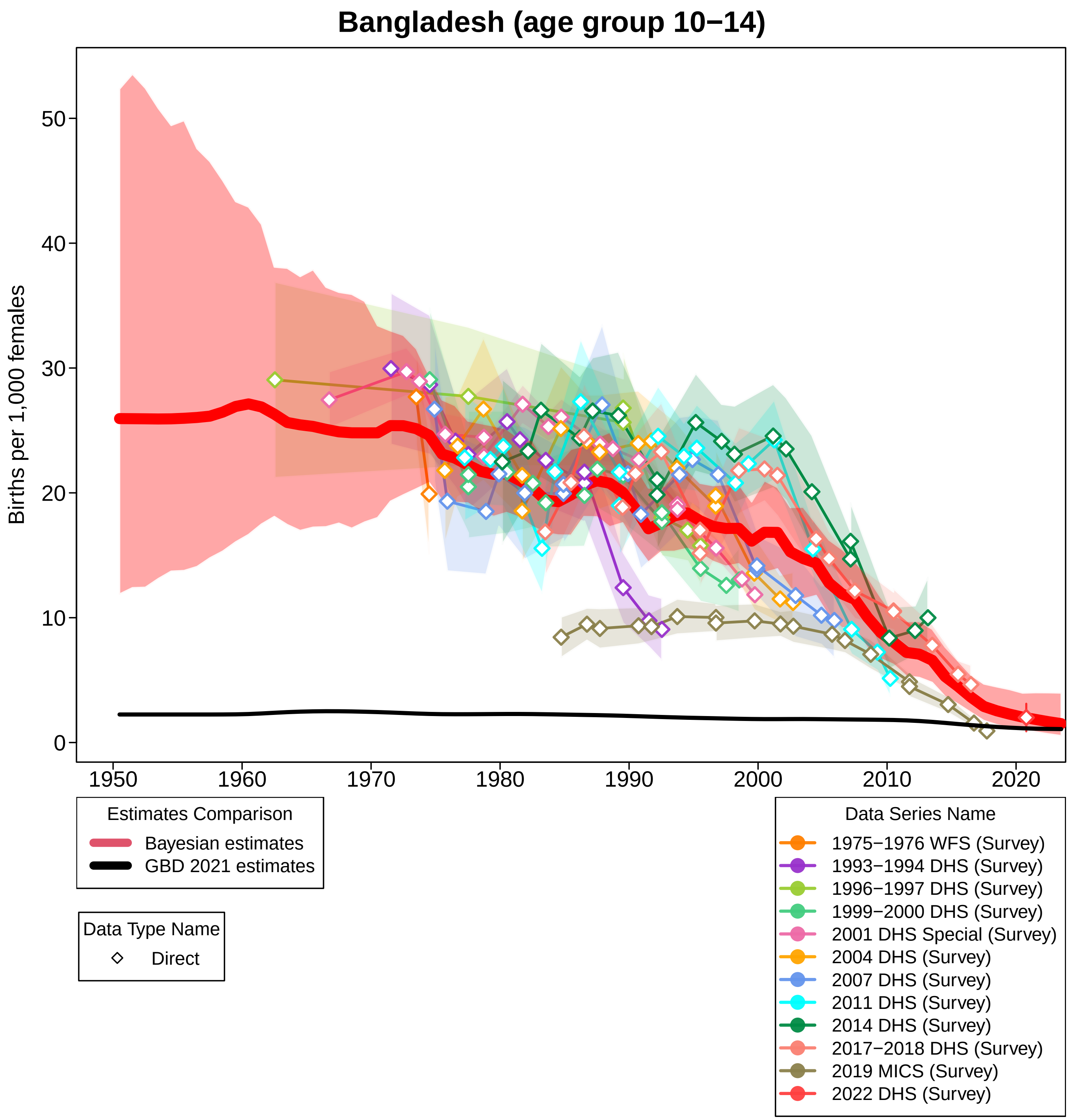
Bangladesh (age group 10−14)
Births per 1,000 females
0
10
20
30
40
50
1950
1960
1970
1980
1990
2000
2010
2020
Estimates Comparison
Bayesian estimates
GBD 2021 estimates
Data Type Name
Direct
Data Series Name
1975−1976 WFS (Survey)
1993−1994 DHS (Survey)
1996−1997 DHS (Survey)
1999−2000 DHS (Survey)
2001 DHS Special (Survey)
2004 DHS (Survey)
2007 DHS (Survey)
2011 DHS (Survey)
2014 DHS (Survey)
2017−2018 DHS (Survey)
2019 MICS (Survey)
2022 DHS (Survey)

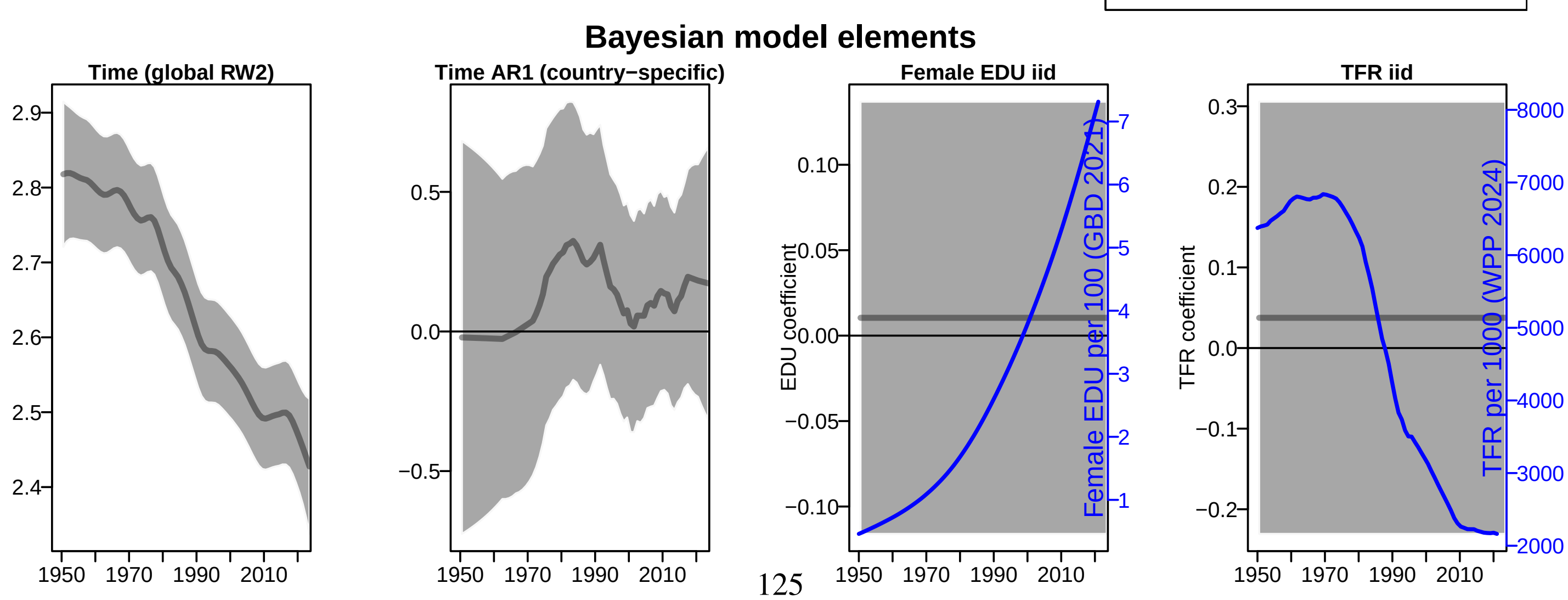
Bayesian model elements
Time (global RW2)
Time AR1 (country−specific)
Female EDU iid
TFR iid
EDU coefficient
Female EDU per 100 (GBD 2021)
TFR coefficient
TFR per 1000 (WPP 2024)
1950
1970
1990
2010

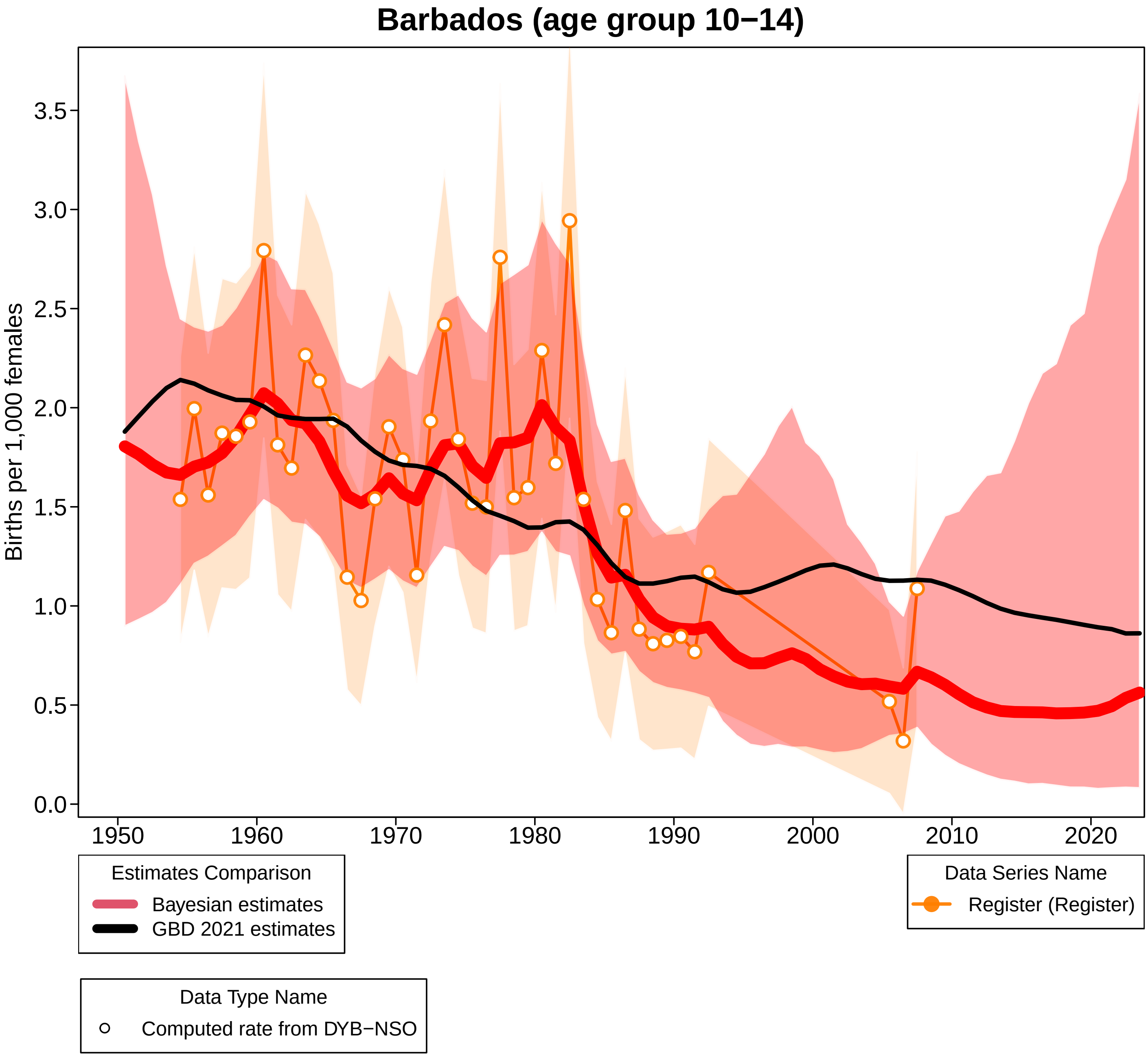
Barbados (age group 10−14)
Births per 1,000 females
3.5
3.0
2.5
2.0
1.5
1.0
0.5
0.0
1950
1960
1970
1980
1990
2000
2010
2020
Estimates Comparison
Bayesian estimates
GBD 2021 estimates
Data Series Name
Register (Register)
Data Type Name
Computed rate from DYB−NSO

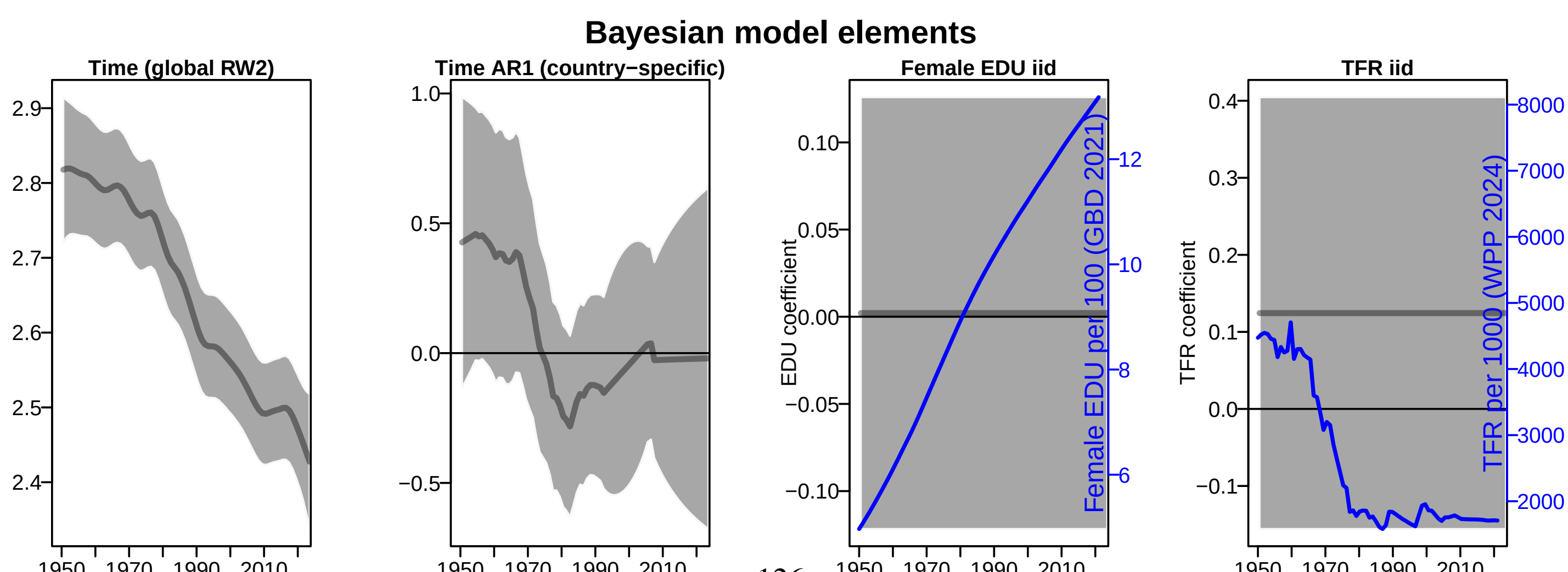
Bayesian model elements
Time (global RW2)
Time AR1 (country−specific)
Female EDU iid
TFR iid
EDU coefficient
Female EDU per 100 (GBD 2021)
TFR coefficient
TFR per 1000 (WPP 2024)
1950
1970
1990
2010

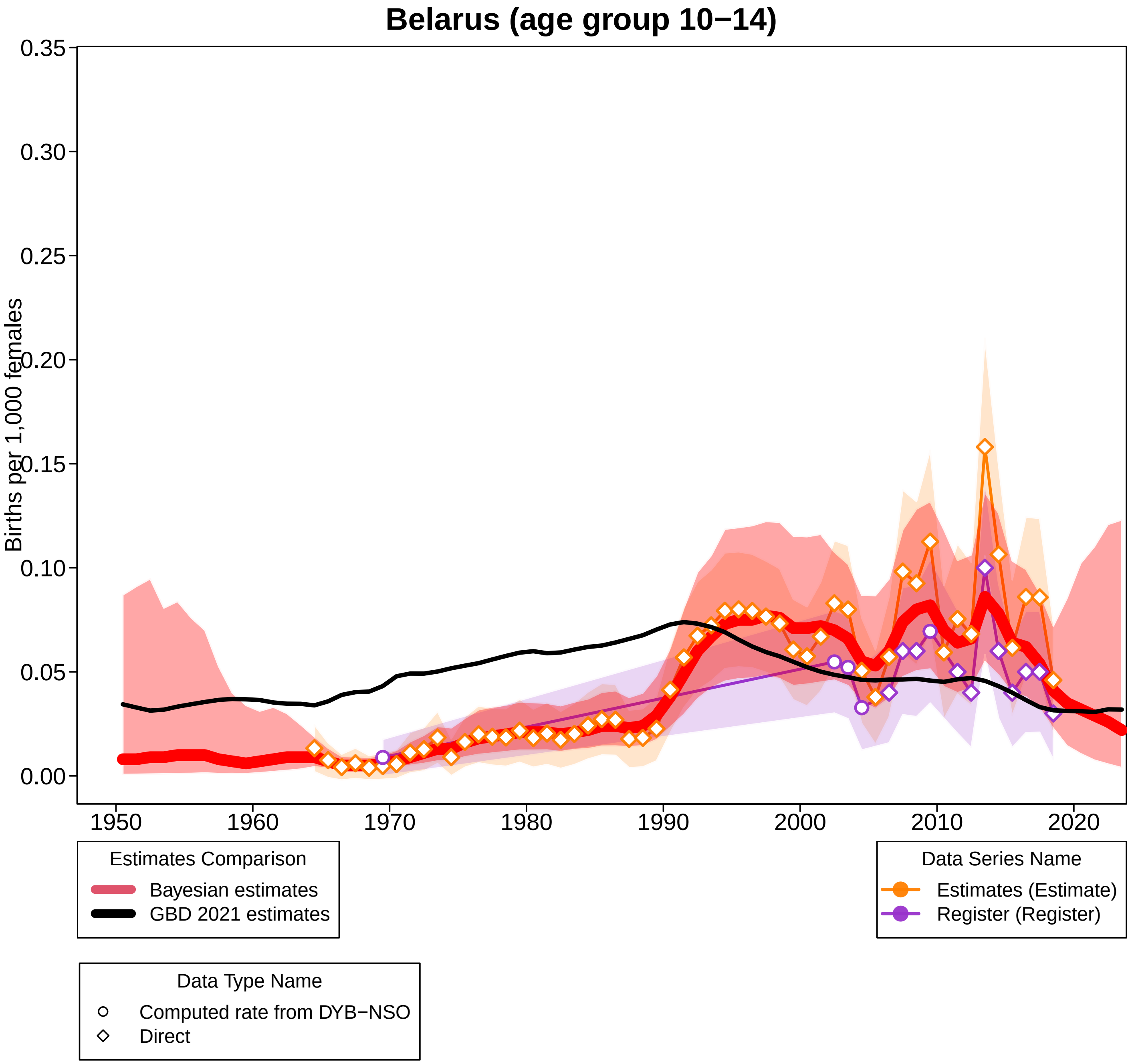

Belarus (age group 10−14)
Births per 1,000 females
0.35
0.30
0.25
0.20
0.15
0.10
0.05
0.00
1950
1960
1970
1980
1990
2000
2010
2020
Estimates Comparison
Bayesian estimates
GBD 2021 estimates
Data Series Name
Estimates (Estimate)
Register (Register)
Data Type Name
Computed rate from DYB−NSO
Direct

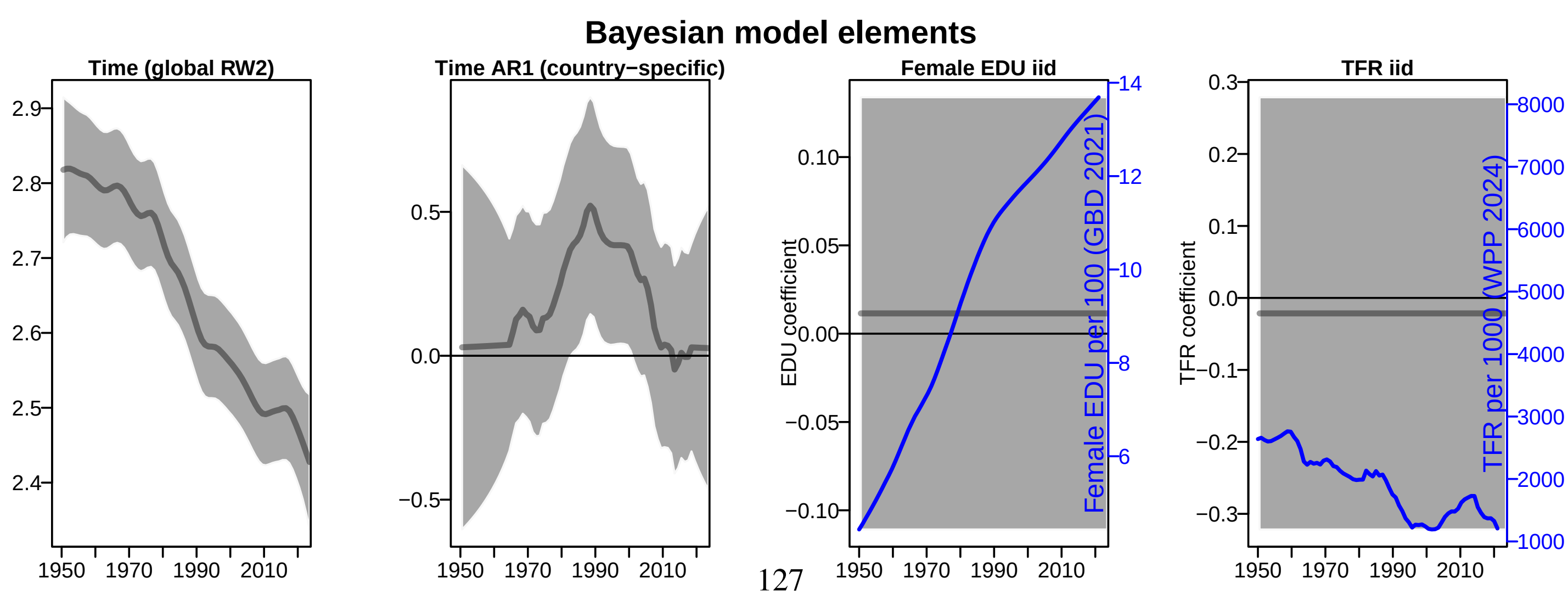

Bayesian model elements
Time (global RW2)
Time AR1 (country−specific)
Female EDU iid
TFR iid
EDU coefficient
Female EDU per 100 (GBD 2021)
TFR coefficient
TFR per 1000 (WPP 2024)
1950
1970
1990
2010

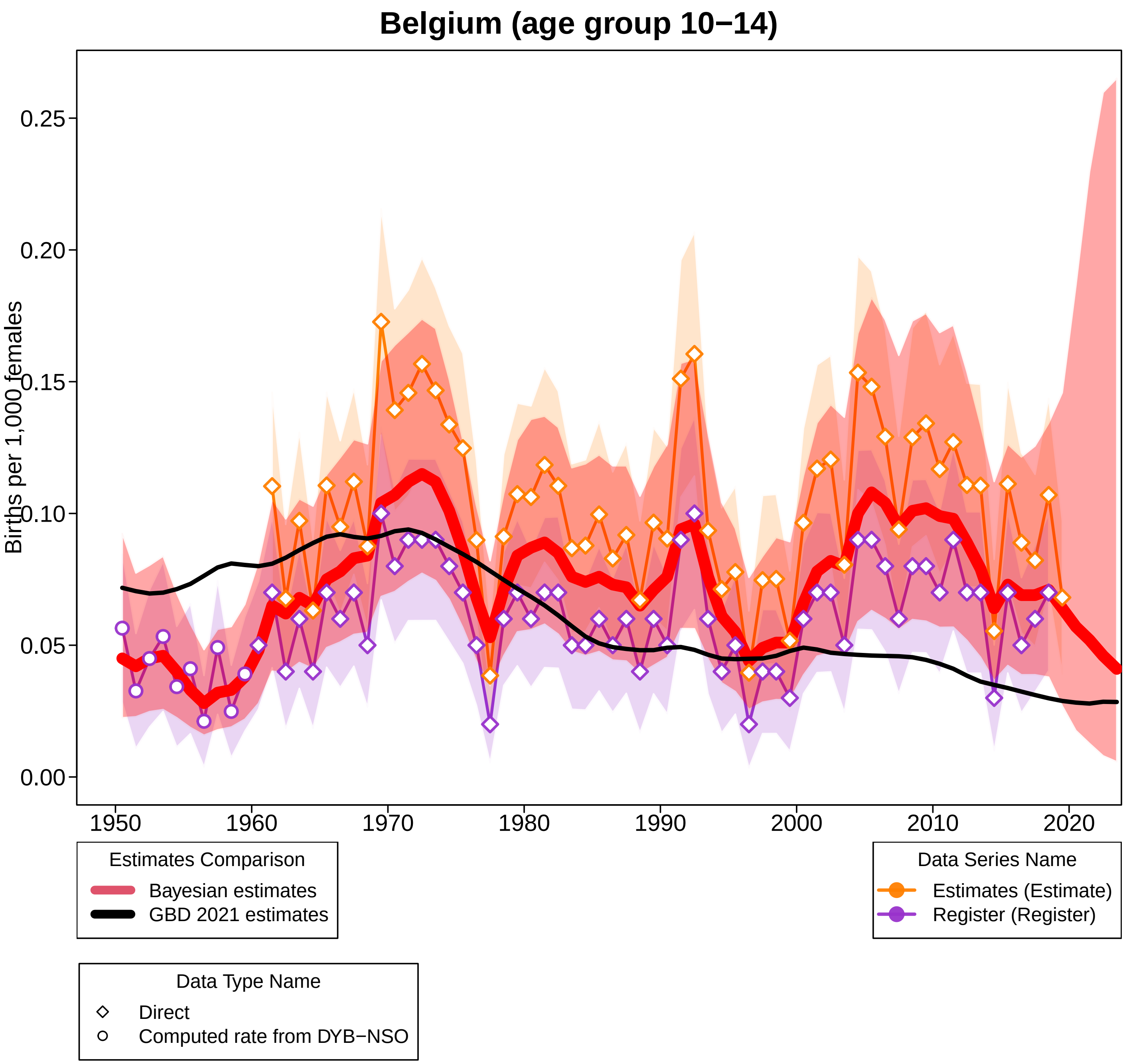
Belgium (age group 10−14)
Births per 1,000 females
0.25
0.20
0.15
0.10
0.05
0.00
1950
1960
1970
1980
1990
2000
2010
2020
Estimates Comparison
Bayesian estimates
GBD 2021 estimates
Data Series Name
Estimates (Estimate)
Register (Register)
Data Type Name
Direct
Computed rate from DYB−NSO

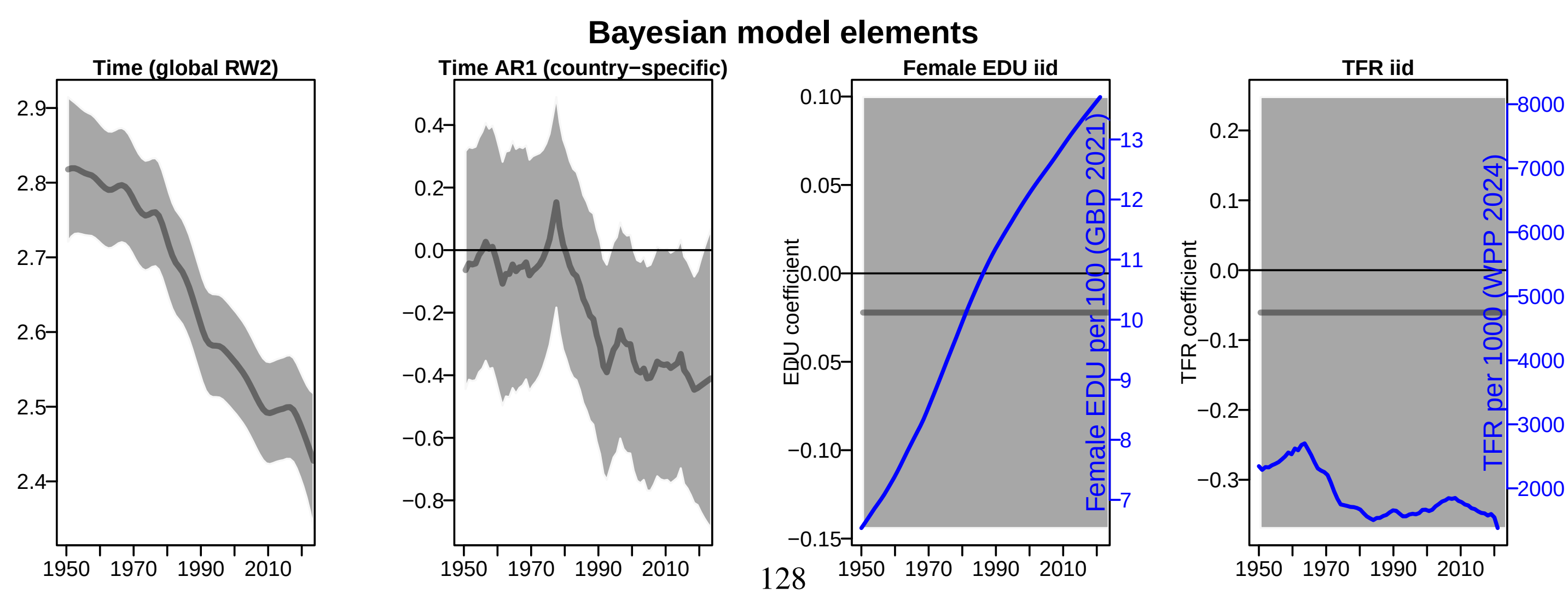
Bayesian model elements
Time (global RW2)
Time AR1 (country−specific)
Female EDU iid
TFR iid
EDU coefficient
Female EDU per 100 (GBD 2021)
TFR coefficient
TFR per 1000 (WPP 2024)

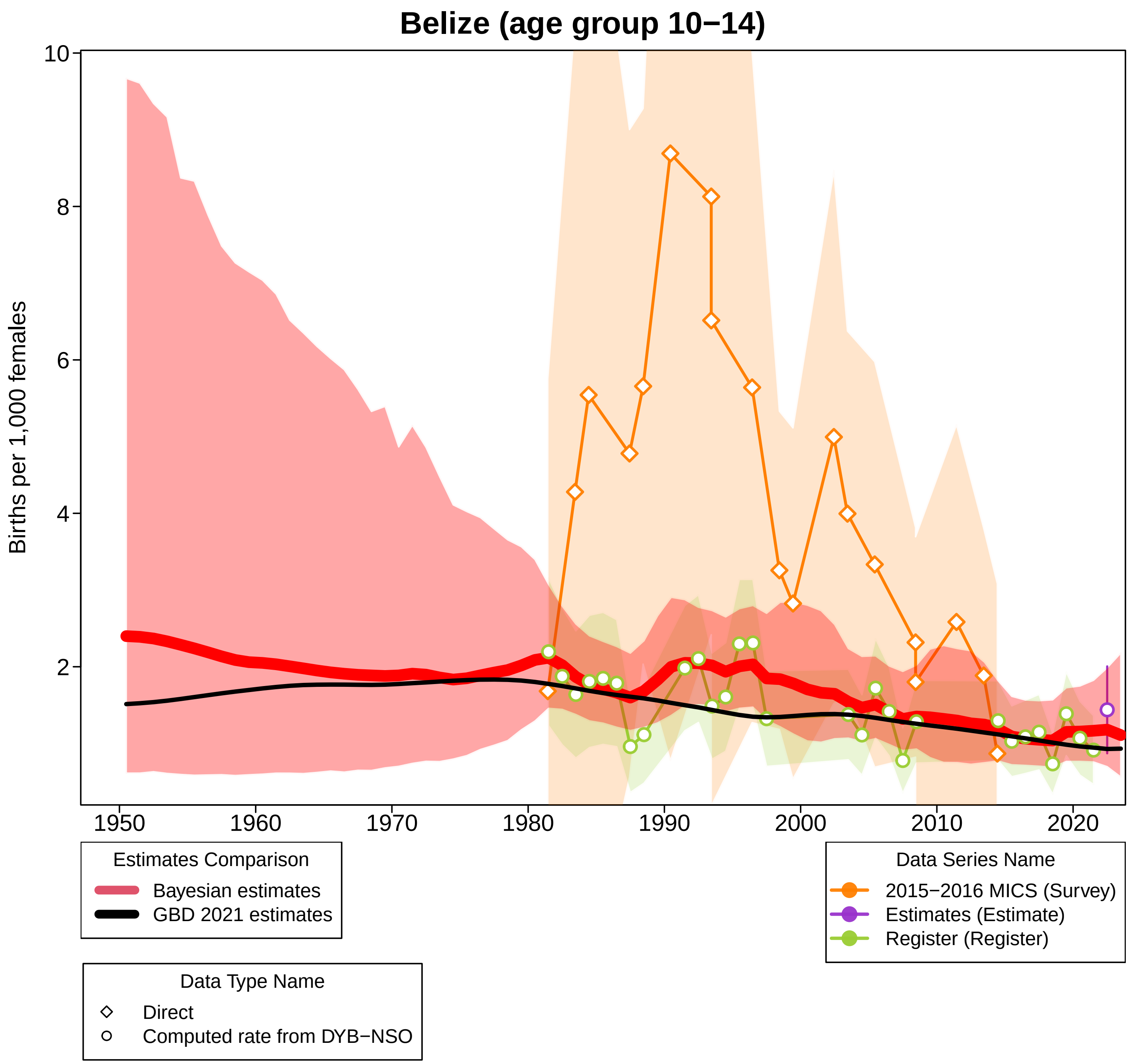
Belize (age group 10−14)
Births per 1,000 females
10
8
6
4
2
1950
1960
1970
1980
1990
2000
2010
2020
Estimates Comparison
Bayesian estimates
GBD 2021 estimates
Data Series Name
2015−2016 MICS (Survey)
Estimates (Estimate)
Register (Register)
Data Type Name
Direct
Computed rate from DYB−NSO

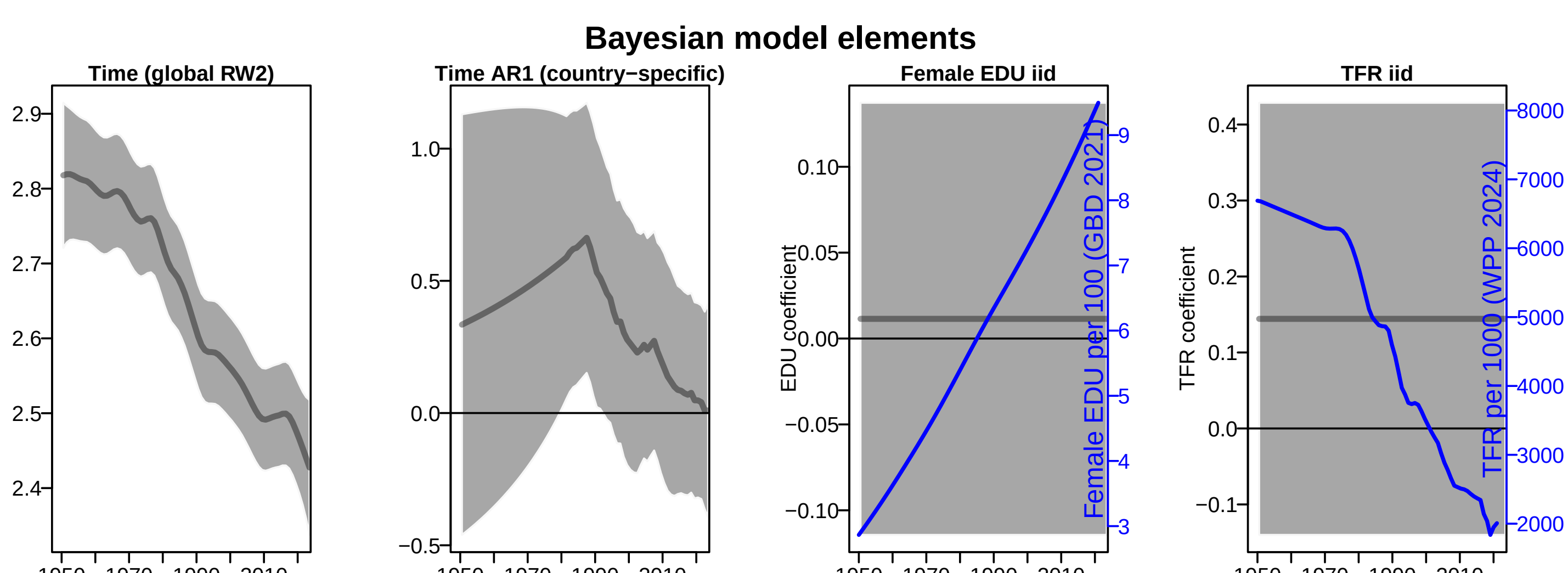
Bayesian model elements
Time (global RW2)
Time AR1 (country−specific)
Female EDU iid
TFR iid
EDU coefficient
Female EDU per 100 (GBD 2021)
TFR coefficient
TFR per 1000 (WPP 2024)
1950
1970
1990
2010

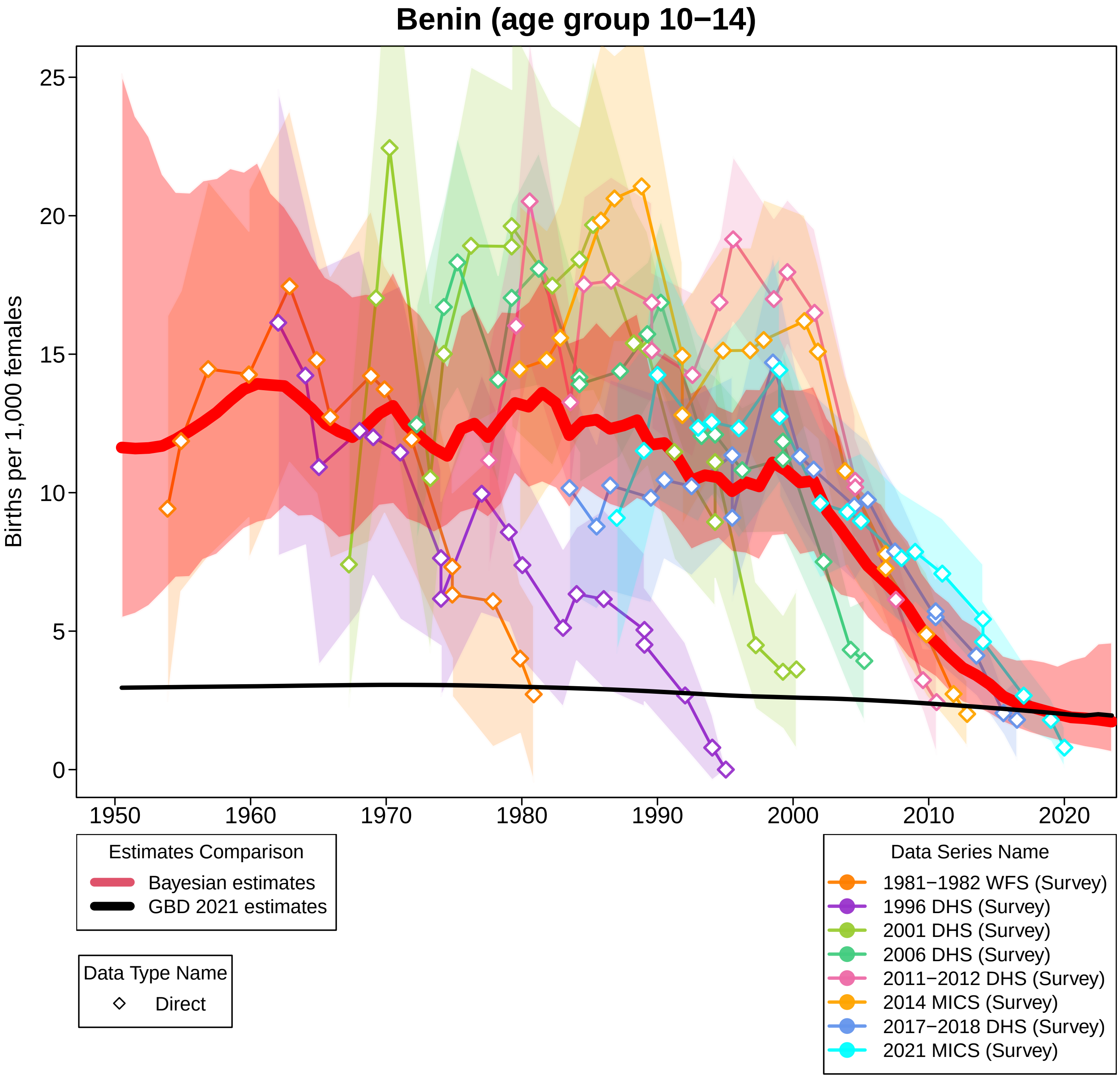

Benin (age group 10−14)
Births per 1,000 females
0
5
10
15
20
25
1950
1960
1970
1980
1990
2000
2010
2020
Estimates Comparison
Bayesian estimates
GBD 2021 estimates
Data Type Name
Direct
Data Series Name
1981−1982 WFS (Survey)
1996 DHS (Survey)
2001 DHS (Survey)
2006 DHS (Survey)
2011−2012 DHS (Survey)
2014 MICS (Survey)
2017−2018 DHS (Survey)
2021 MICS (Survey)

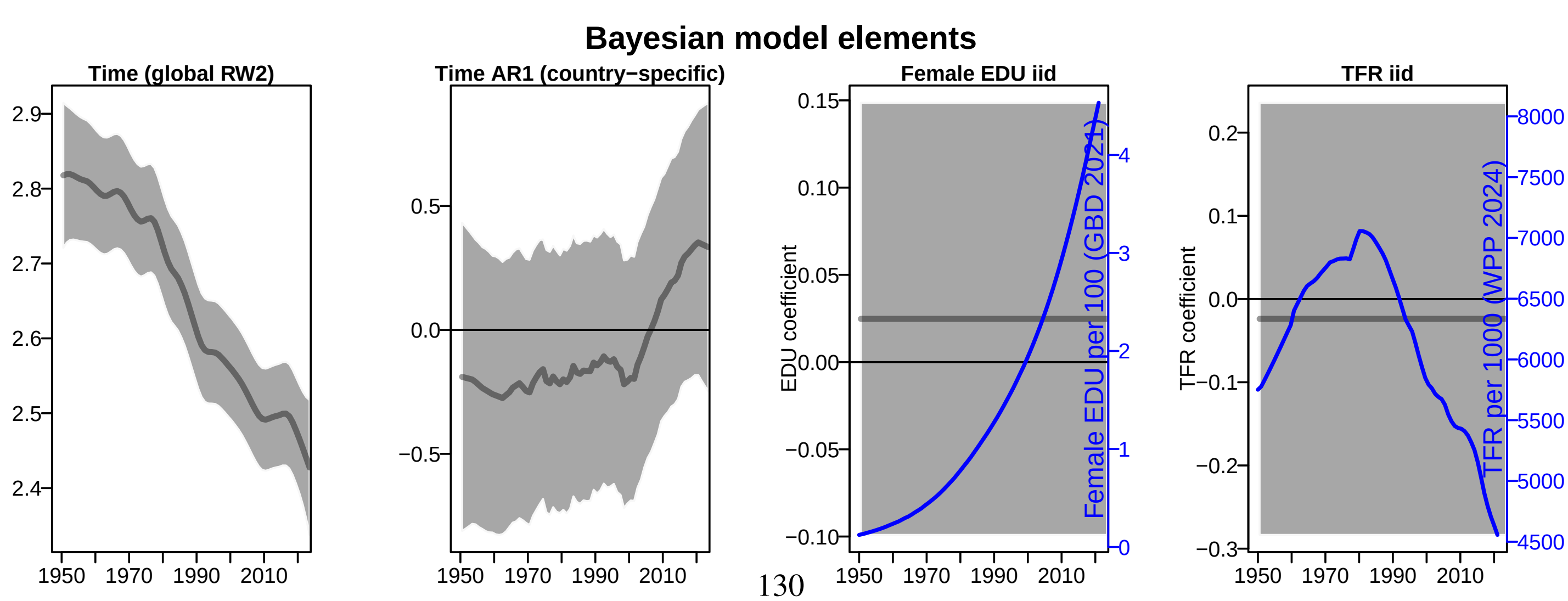

Bayesian model elements
Time (global RW2)
Time AR1 (country−specific)
Female EDU iid
TFR iid
EDU coefficient
Female EDU per 100 (GBD 2021)
TFR coefficient
TFR per 1000 (WPP 2024)
1950
1970
1990
2010

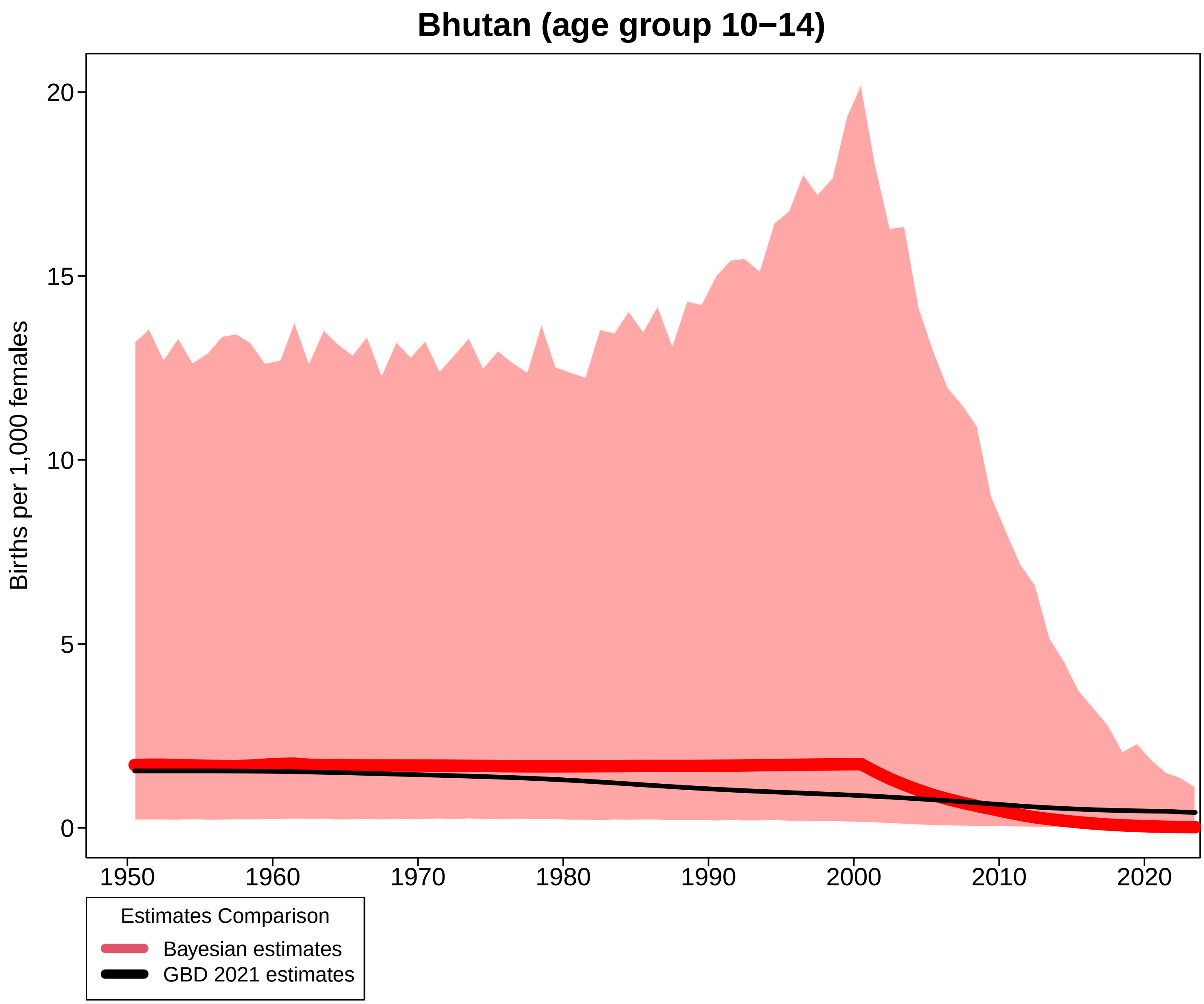

Bhutan (age group 10−14)
Births per 1,000 females
20
15
10
5
0
1950
1960
1970
1980
1990
2000
2010
2020
Estimates Comparison
Bayesian estimates
GBD 2021 estimates

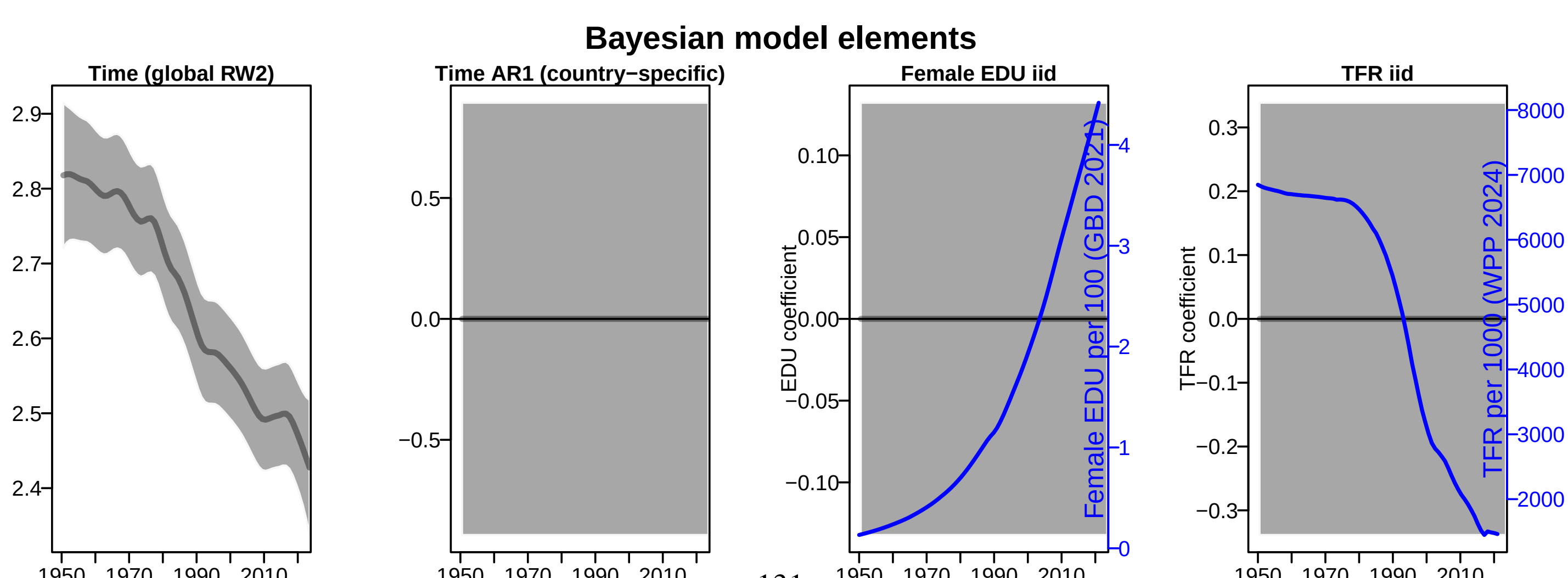

Bayesian model elements
Time (global RW2)
Time AR1 (country−specific)
Female EDU iid
TFR iid
EDU coefficient
TFR coefficient
Female EDU per 100 (GBD 2021)
TFR per 1000 (WPP 2024)
1950
1970
1990
2010

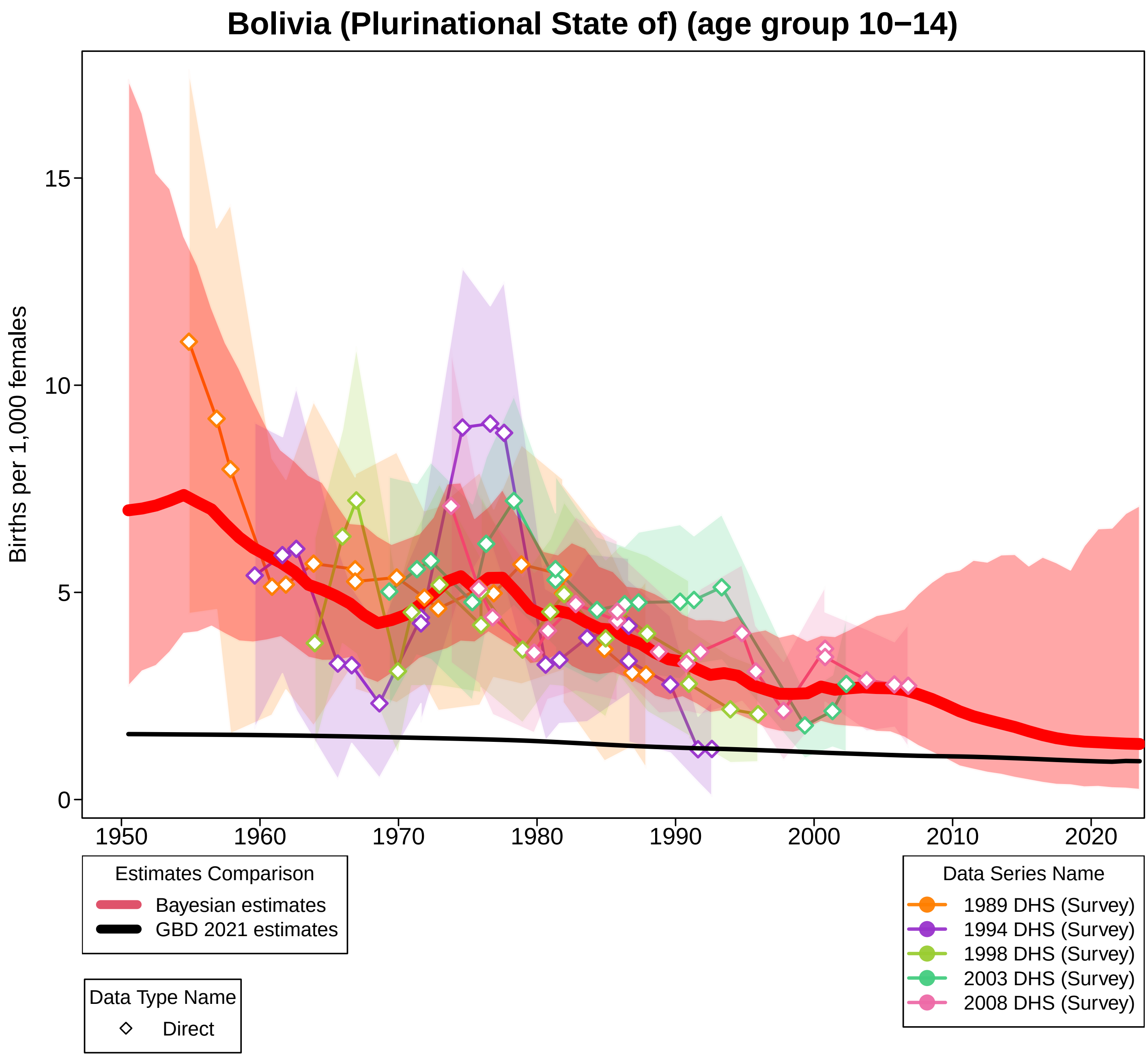
Bolivia (Plurinational State of) (age group 10−14)
Births per 1,000 females
0
5
10
15
1950
1960
1970
1980
1990
2000
2010
2020
Estimates Comparison
Bayesian estimates
GBD 2021 estimates
Data Series Name
1989 DHS (Survey)
1994 DHS (Survey)
1998 DHS (Survey)
2003 DHS (Survey)
2008 DHS (Survey)
Data Type Name
Direct

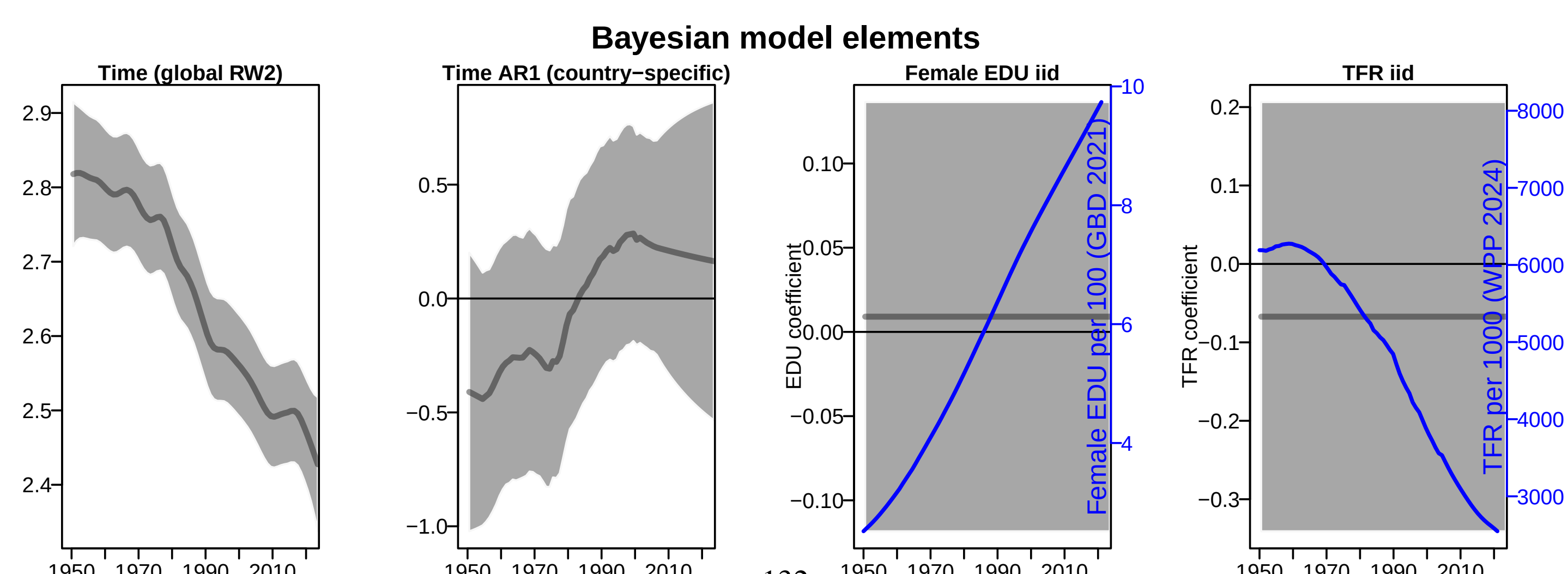
Bayesian model elements
Time (global RW2)
Time AR1 (country−specific)
Female EDU iid
TFR iid
EDU coefficient
Female EDU per 100 (GBD 2021)
TFR coefficient
TFR per 1000 (WPP 2024)
1950
1970
1990
2010

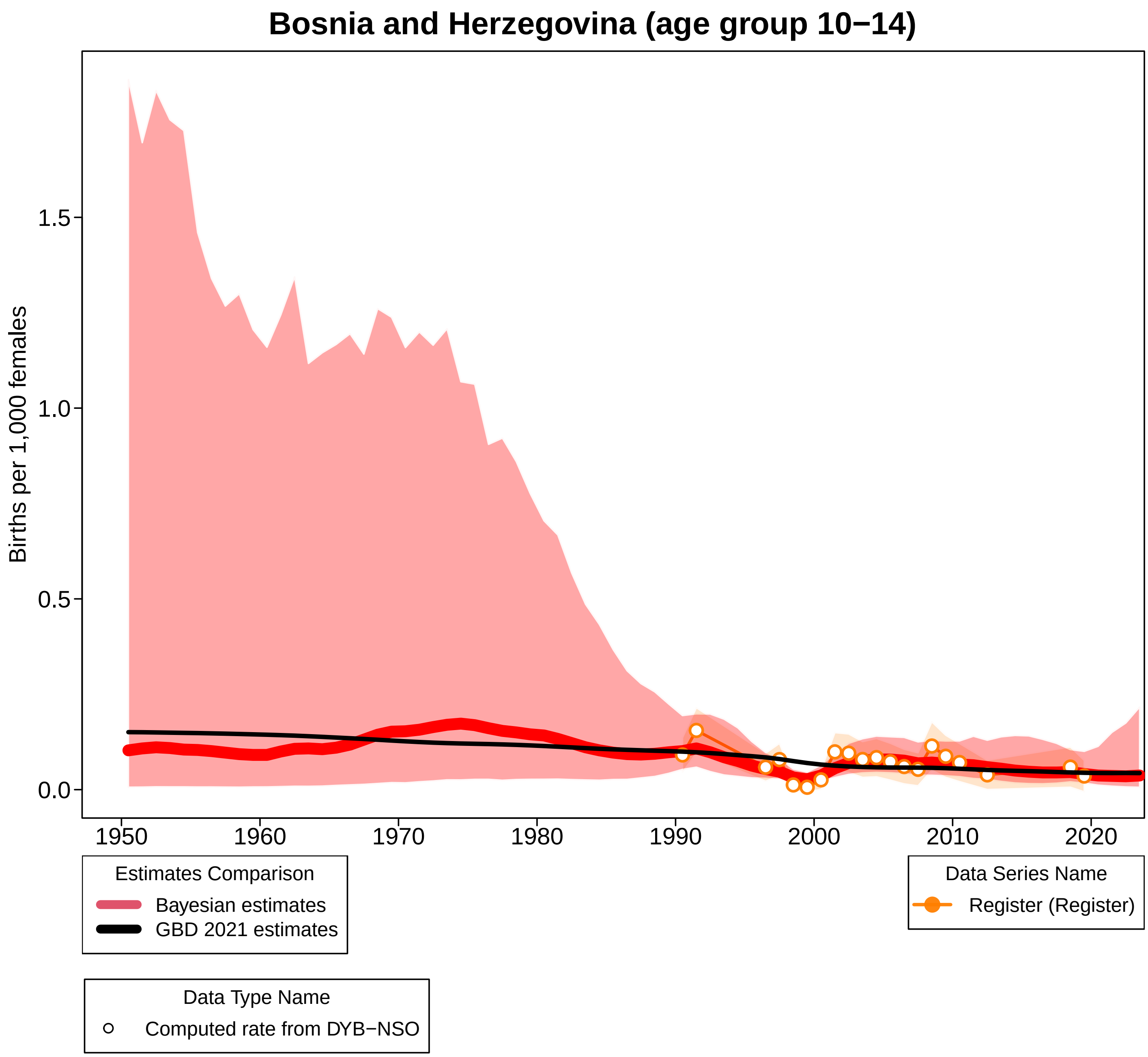
Bosnia and Herzegovina (age group 10−14)
Births per 1,000 females
0.0
0.5
1.0
1.5
1950
1960
1970
1980
1990
2000
2010
2020
Estimates Comparison
Bayesian estimates
GBD 2021 estimates
Data Series Name
Register (Register)
Data Type Name
Computed rate from DYB−NSO

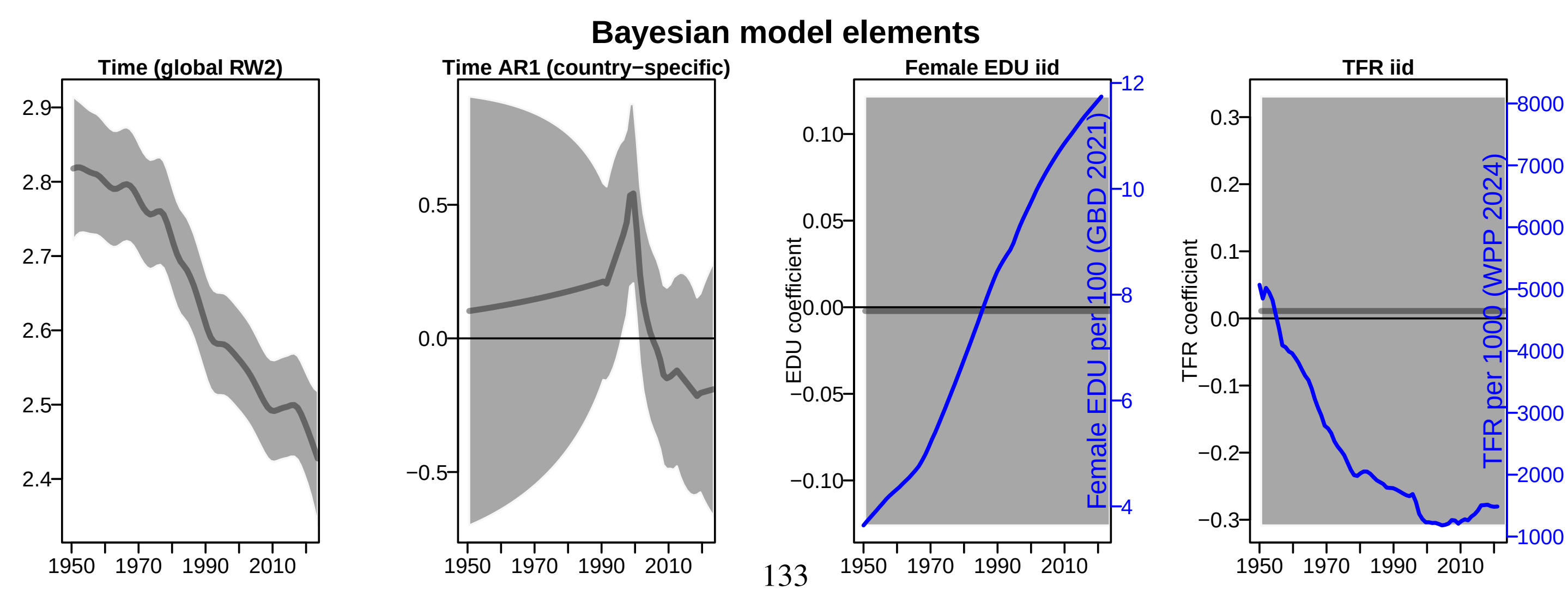
Bayesian model elements
Time (global RW2)
Time AR1 (country−specific)
Female EDU iid
TFR iid
EDU coefficient
Female EDU per 100 (GBD 2021)
TFR coefficient
TFR per 1000 (WPP 2024)
1950
1970
1990
2010

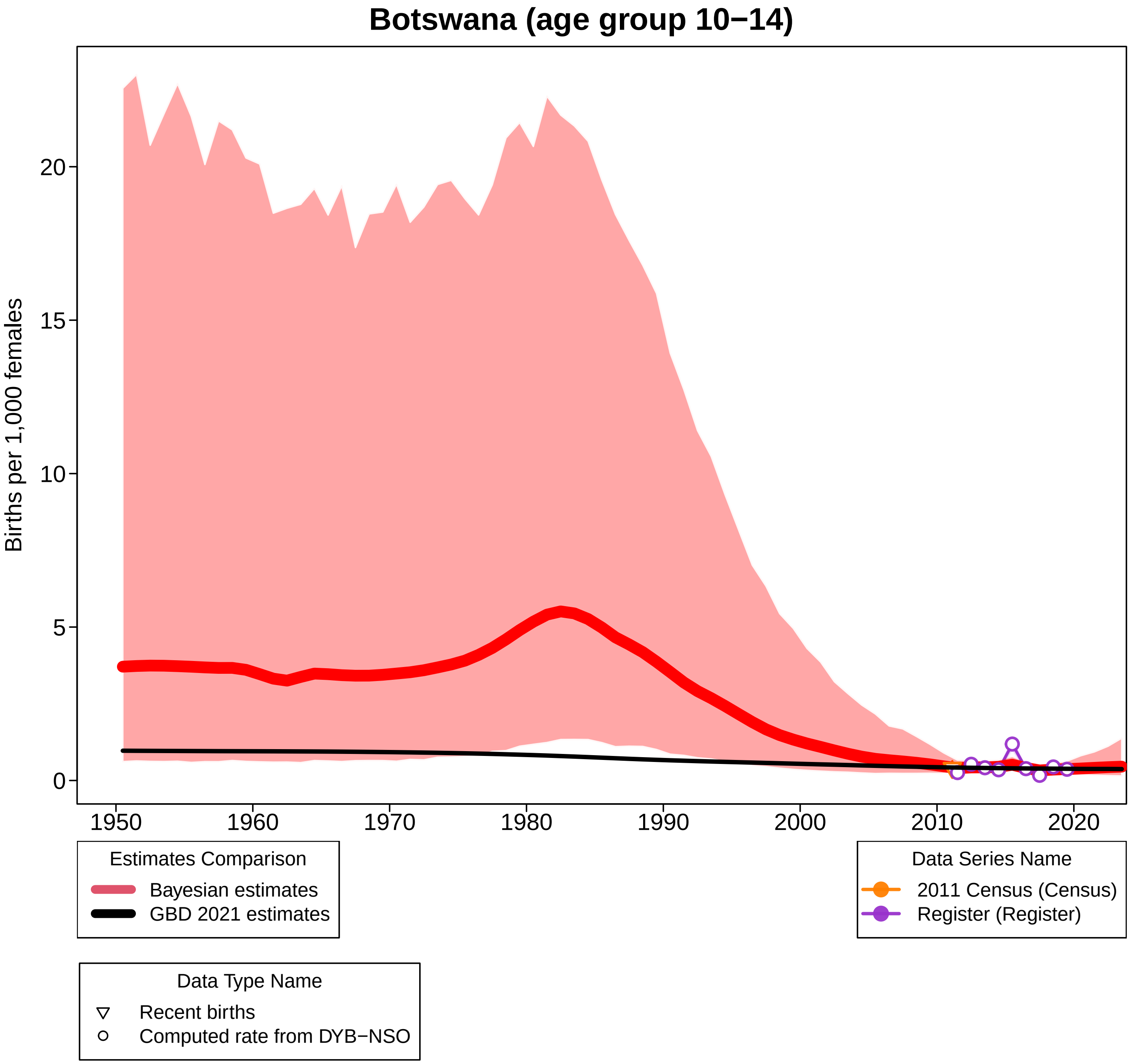
Botswana (age group 10−14)
Births per 1,000 females
0
5
10
15
20
1950
1960
1970
1980
1990
2000
2010
2020
Estimates Comparison
Bayesian estimates
GBD 2021 estimates
Data Series Name
2011 Census (Census)
Register (Register)
Data Type Name
Recent births
Computed rate from DYB−NSO

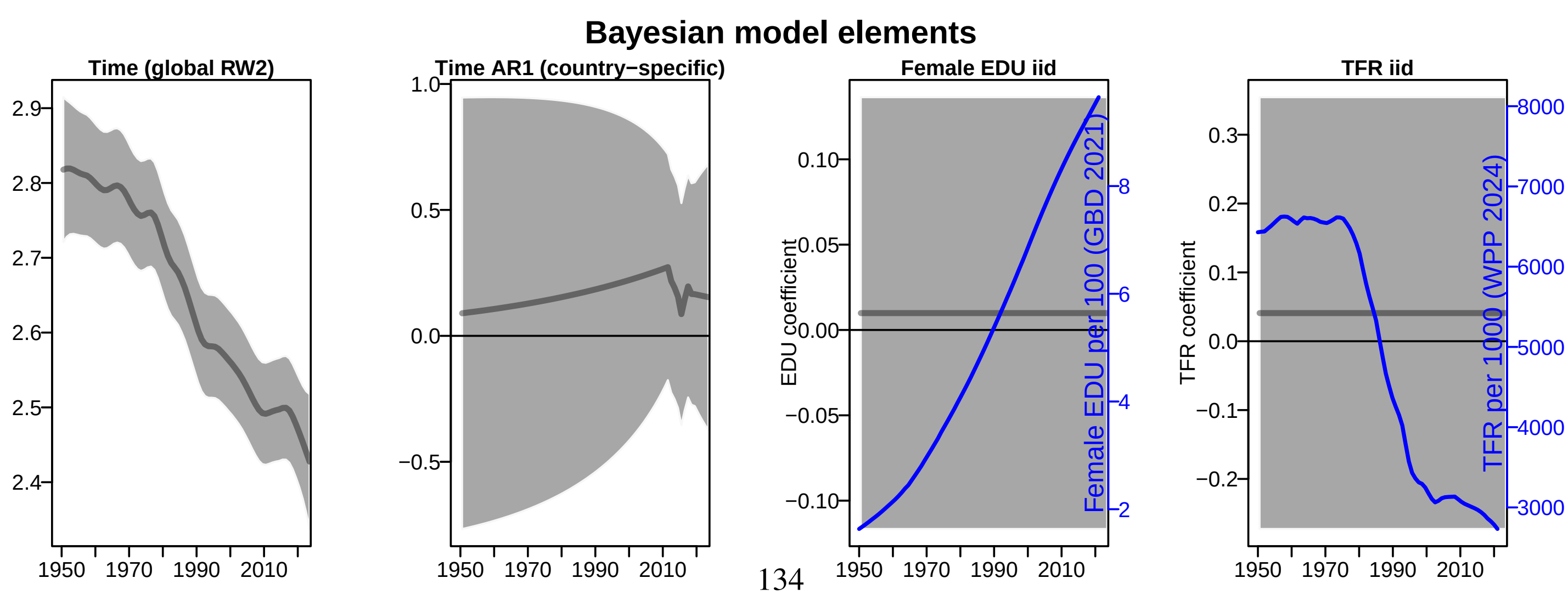
Bayesian model elements
Time (global RW2)
Time AR1 (country−specific)
Female EDU iid
TFR iid
EDU coefficient
Female EDU per 100 (GBD 2021)
TFR coefficient
TFR per 1000 (WPP 2024)
1950
1970
1990
2010

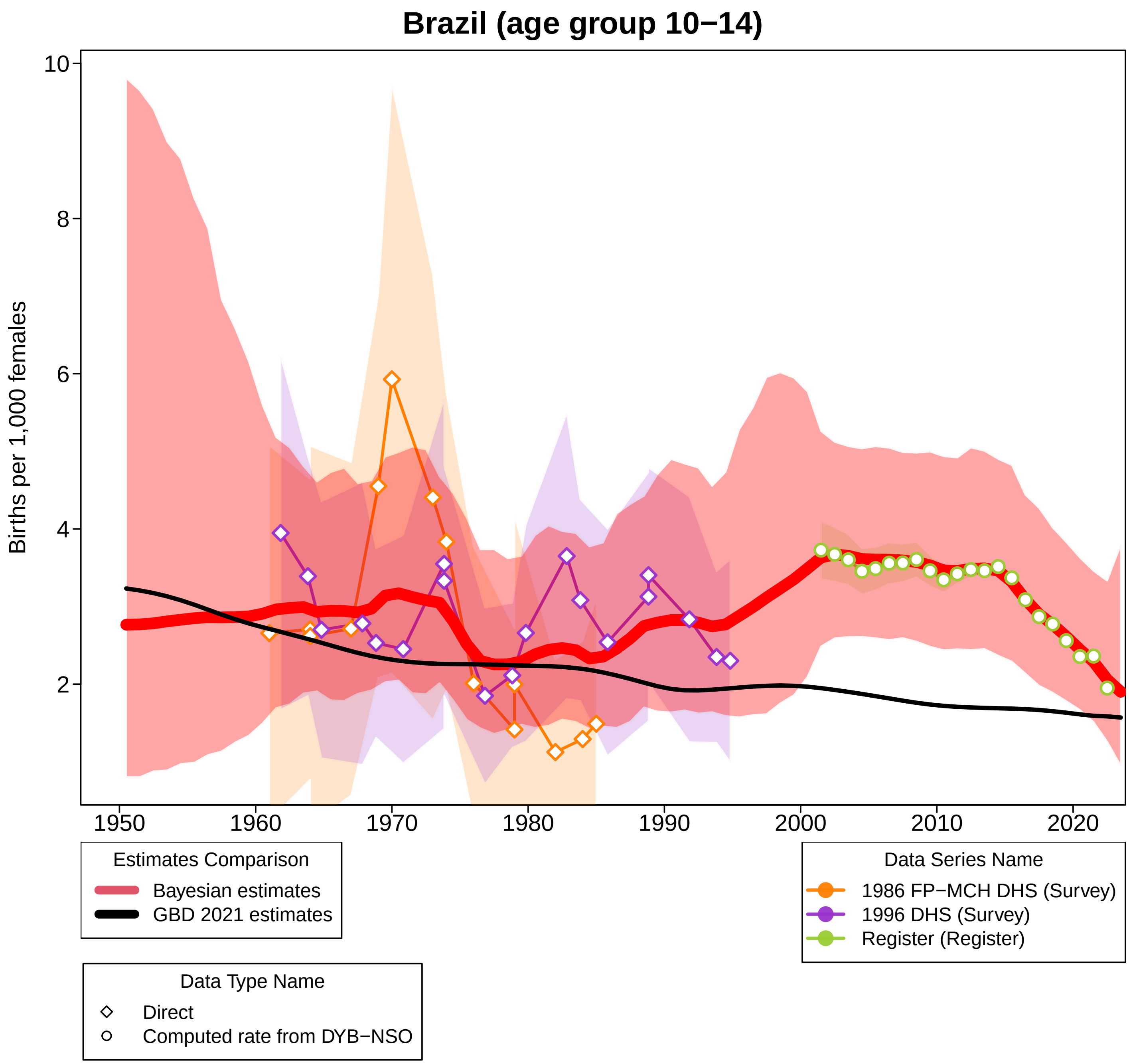
Brazil (age group 10−14)
Births per 1,000 females
10
8
6
4
2
1950
1960
1970
1980
1990
2000
2010
2020
Estimates Comparison
Bayesian estimates
GBD 2021 estimates
Data Series Name
1986 FP−MCH DHS (Survey)
1996 DHS (Survey)
Register (Register)
Data Type Name
Direct
Computed rate from DYB−NSO

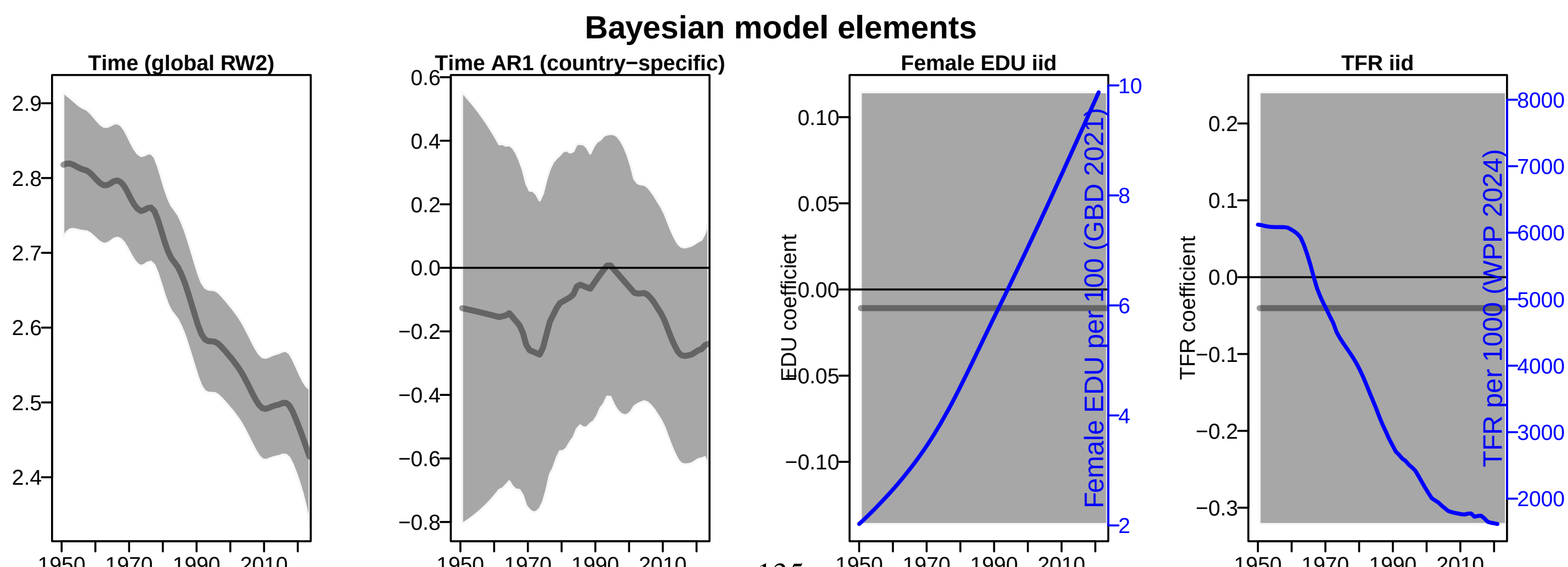
Bayesian model elements
Time (global RW2)
Time AR1 (country−specific)
Female EDU iid
TFR iid
EDU coefficient
Female EDU per 100 (GBD 2021)
TFR coefficient
TFR per 1000 (WPP 2024)
1950
1970
1990
2010

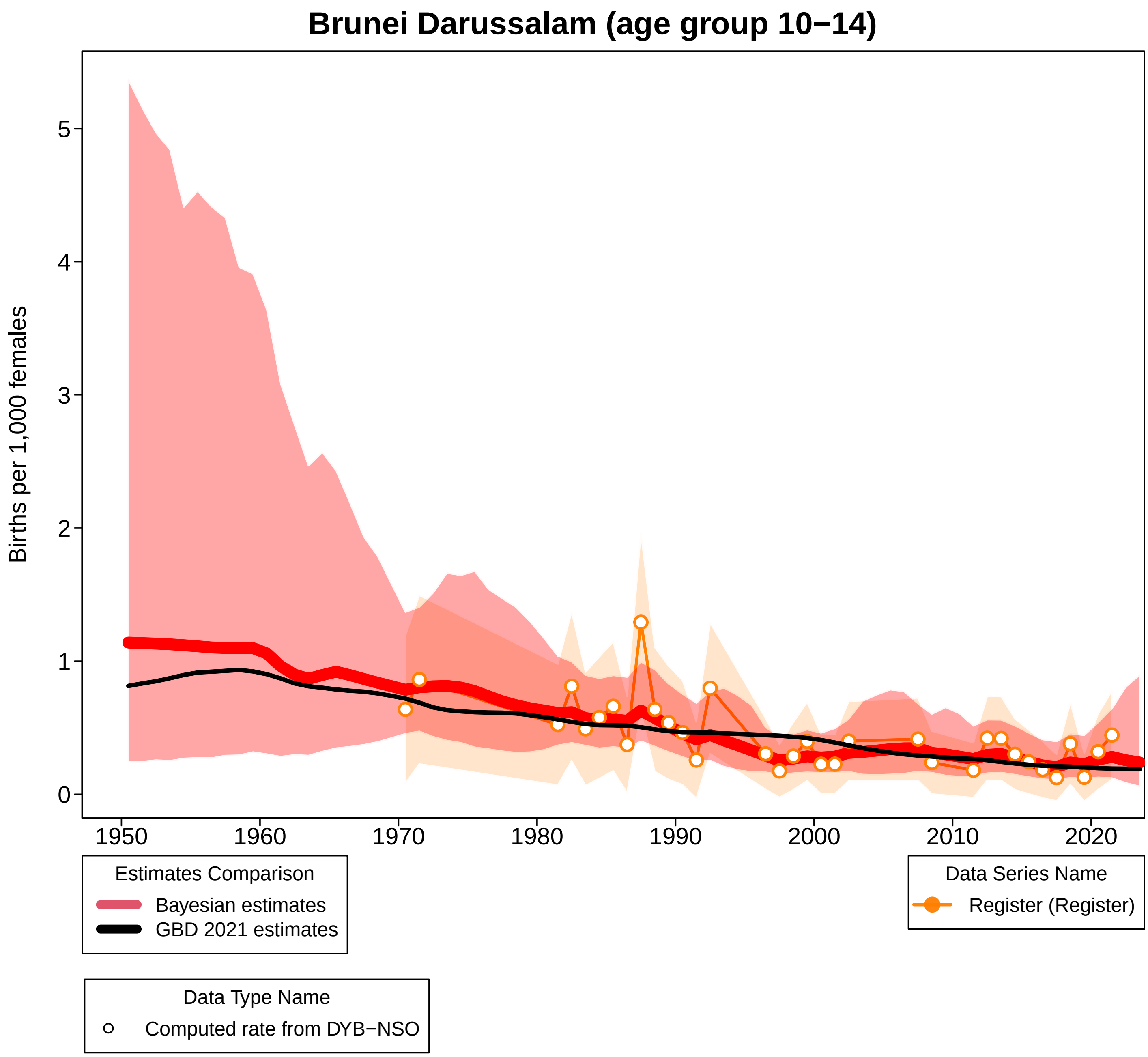

Brunei Darussalam (age group 10−14)
Births per 1,000 females
0
1
2
3
4
5
1950
1960
1970
1980
1990
2000
2010
2020
Estimates Comparison
Bayesian estimates
GBD 2021 estimates
Data Series Name
Register (Register)
Data Type Name
Computed rate from DYB−NSO

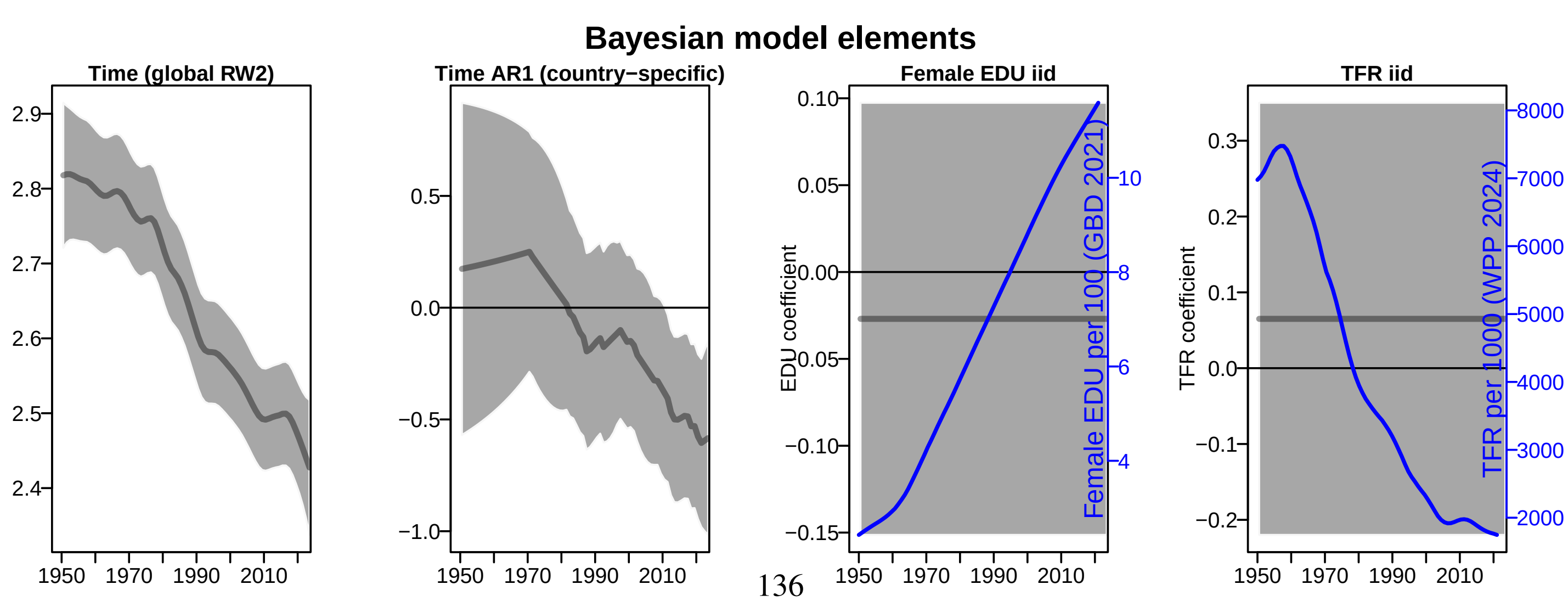

Bayesian model elements
Time (global RW2)
Time AR1 (country−specific)
Female EDU iid
TFR iid
EDU coefficient
Female EDU per 100 (GBD 2021)
TFR coefficient
TFR per 1000 (WPP 2024)
1950
1970
1990
2010

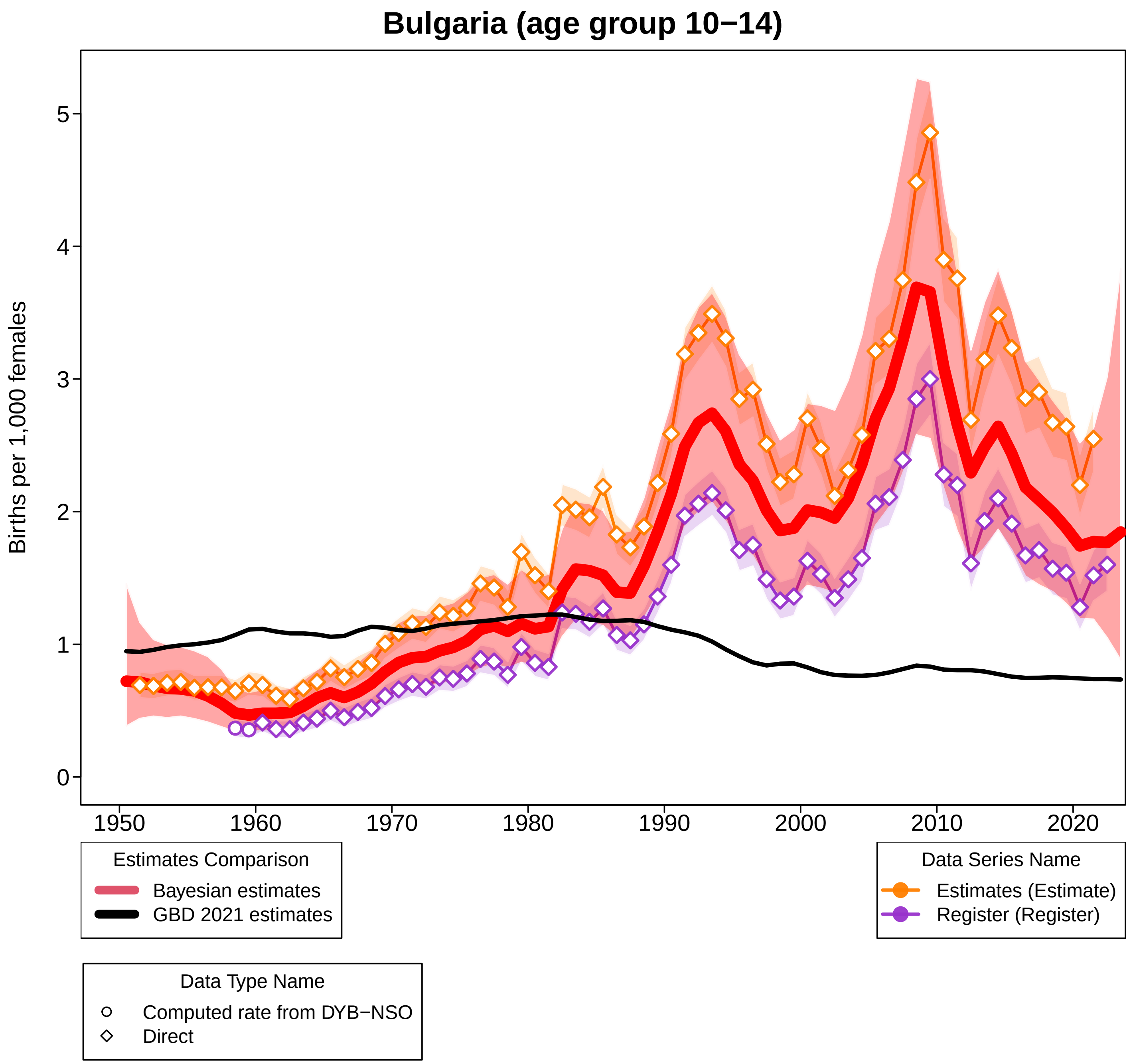
Bulgaria (age group 10−14)
Births per 1,000 females
Estimates Comparison
Bayesian estimates
GBD 2021 estimates
Data Series Name
Estimates (Estimate)
Register (Register)
Data Type Name
Computed rate from DYB−NSO
Direct

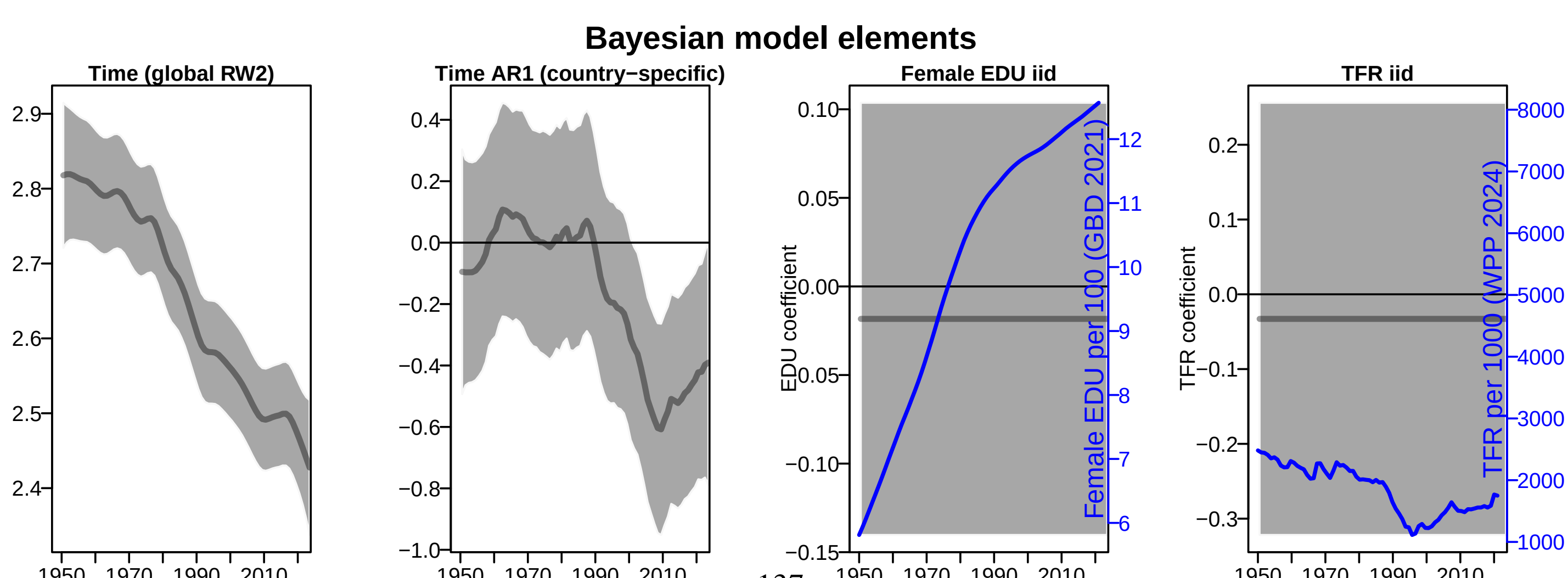
Bayesian model elements
Time (global RW2)
Time AR1 (country−specific)
Female EDU iid
TFR iid
EDU coefficient
Female EDU per 100 (GBD 2021)
TFR coefficient
TFR per 1000 (WPP 2024)

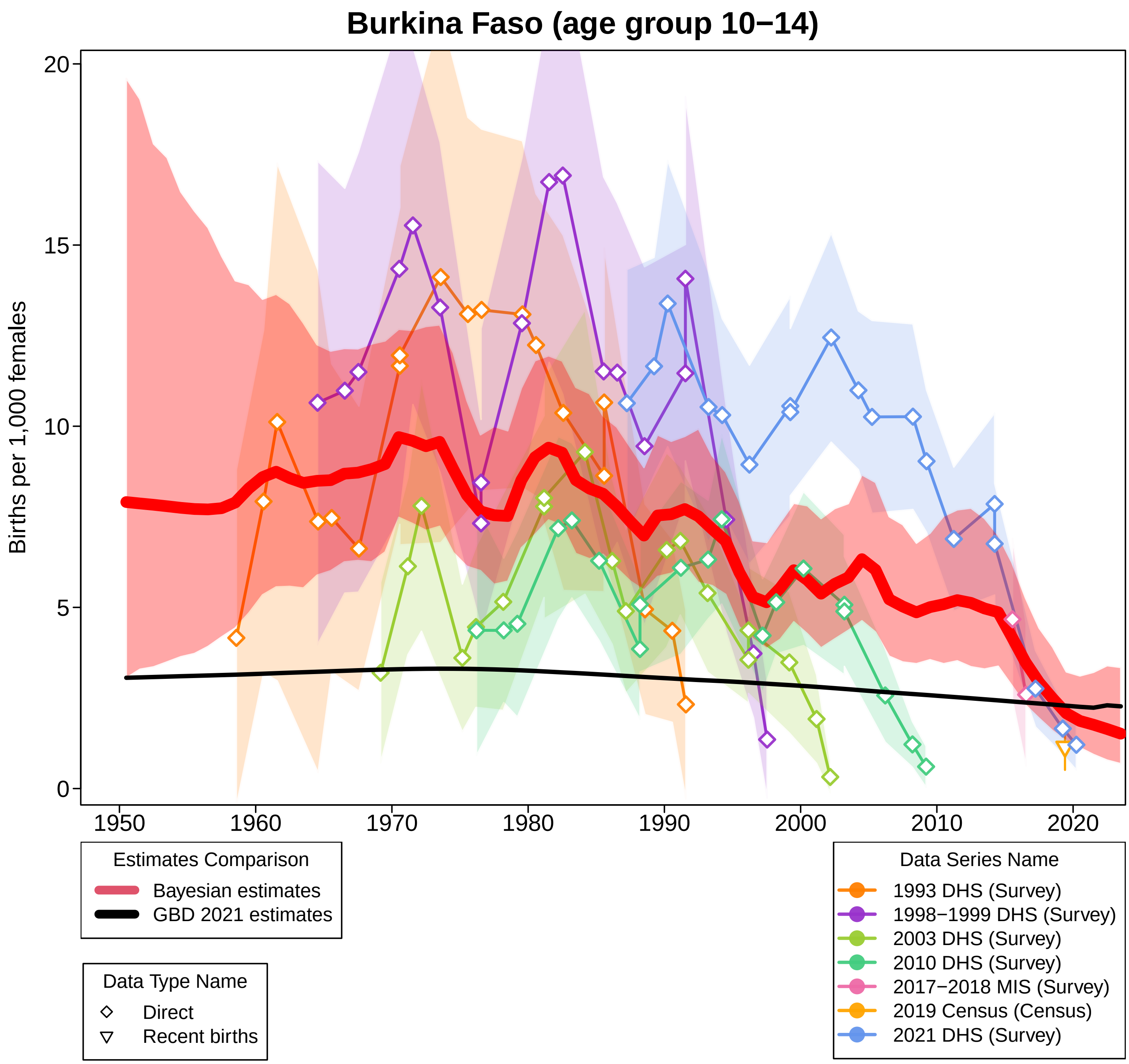
Burkina Faso (age group 10−14)
Births per 1,000 females
0
5
10
15
20
1950
1960
1970
1980
1990
2000
2010
2020
Estimates Comparison
Bayesian estimates
GBD 2021 estimates
Data Type Name
Direct
Recent births
Data Series Name
1993 DHS (Survey)
1998−1999 DHS (Survey)
2003 DHS (Survey)
2010 DHS (Survey)
2017−2018 MIS (Survey)
2019 Census (Census)
2021 DHS (Survey)

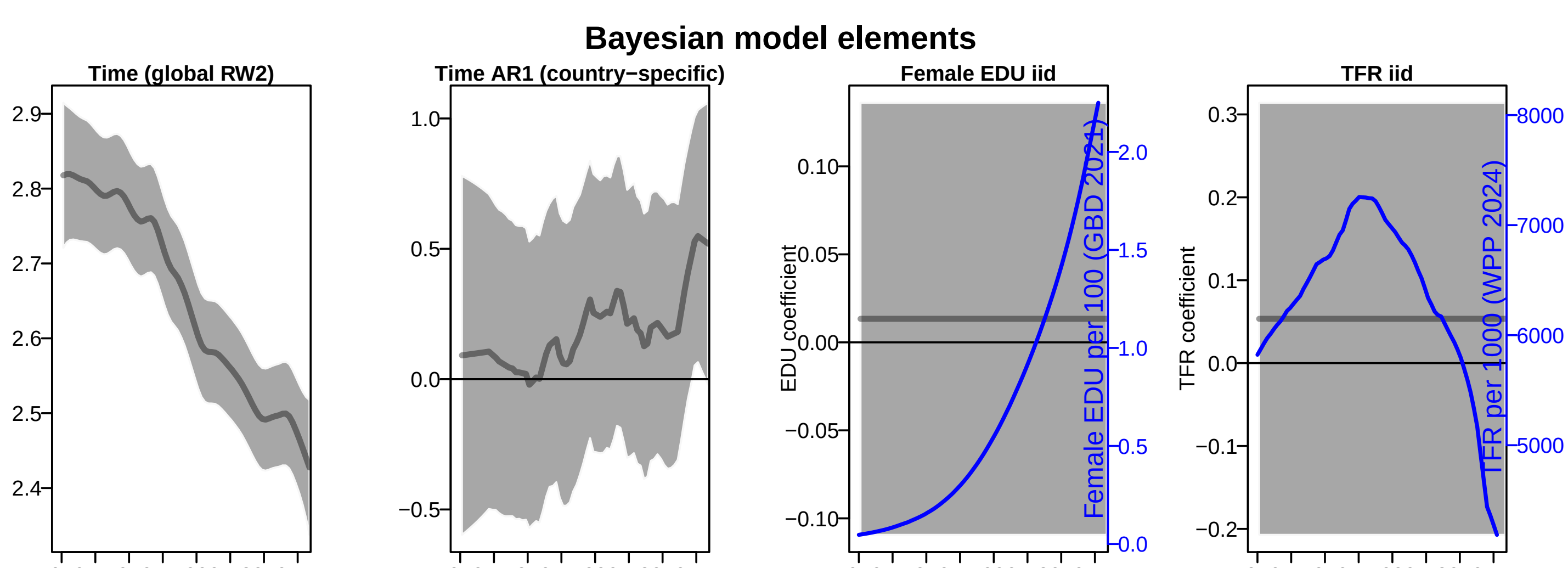
Bayesian model elements
Time (global RW2)
Time AR1 (country−specific)
Female EDU iid
TFR iid
EDU coefficient
Female EDU per 100 (GBD 2021)
TFR coefficient
TFR per 1000 (WPP 2024)
1950
1970
1990
2010

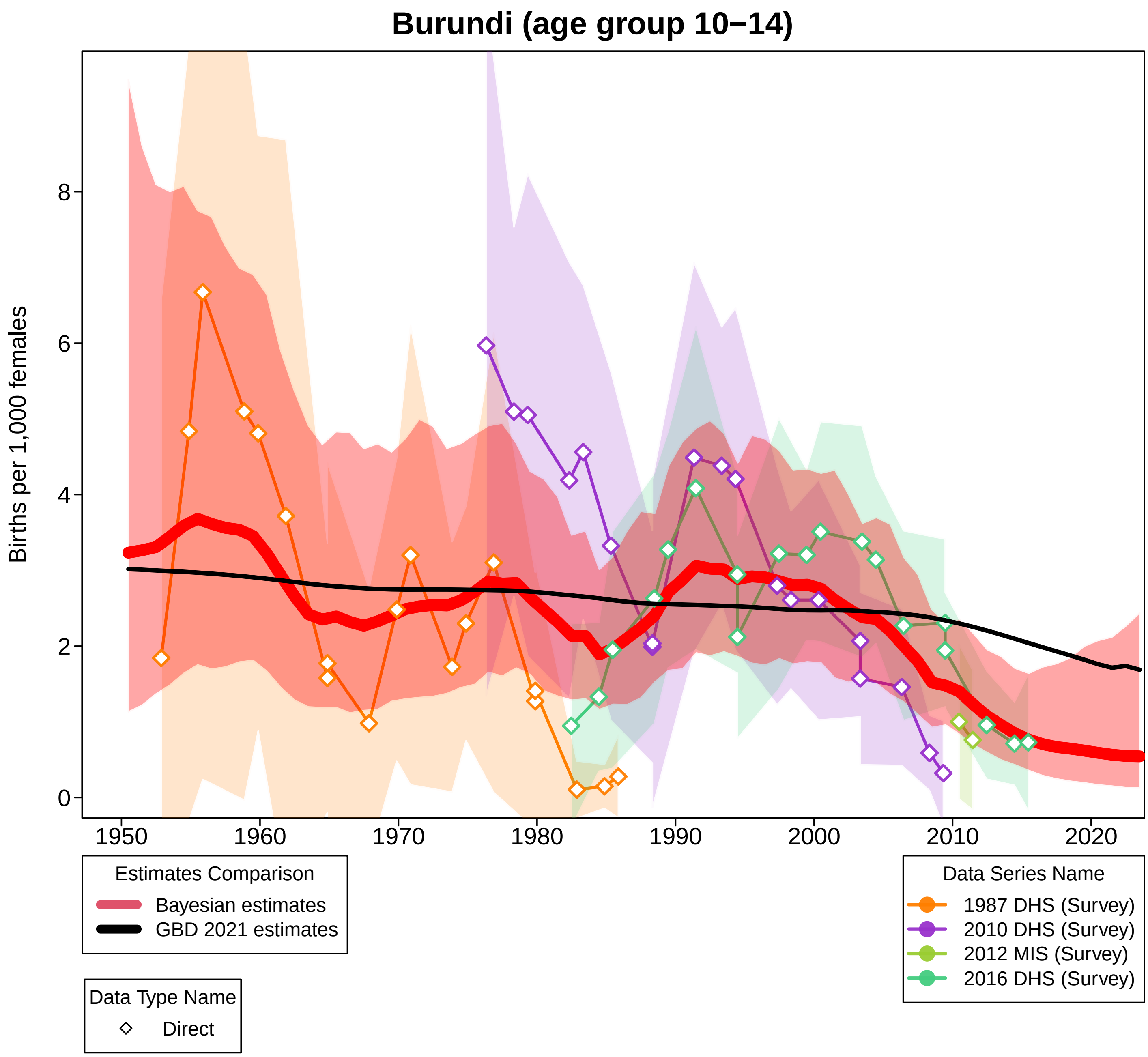
Burundi (age group 10−14)
Births per 1,000 females
0
2
4
6
8
1950
1960
1970
1980
1990
2000
2010
2020
Estimates Comparison
Bayesian estimates
GBD 2021 estimates
Data Type Name
Direct
Data Series Name
1987 DHS (Survey)
2010 DHS (Survey)
2012 MIS (Survey)
2016 DHS (Survey)

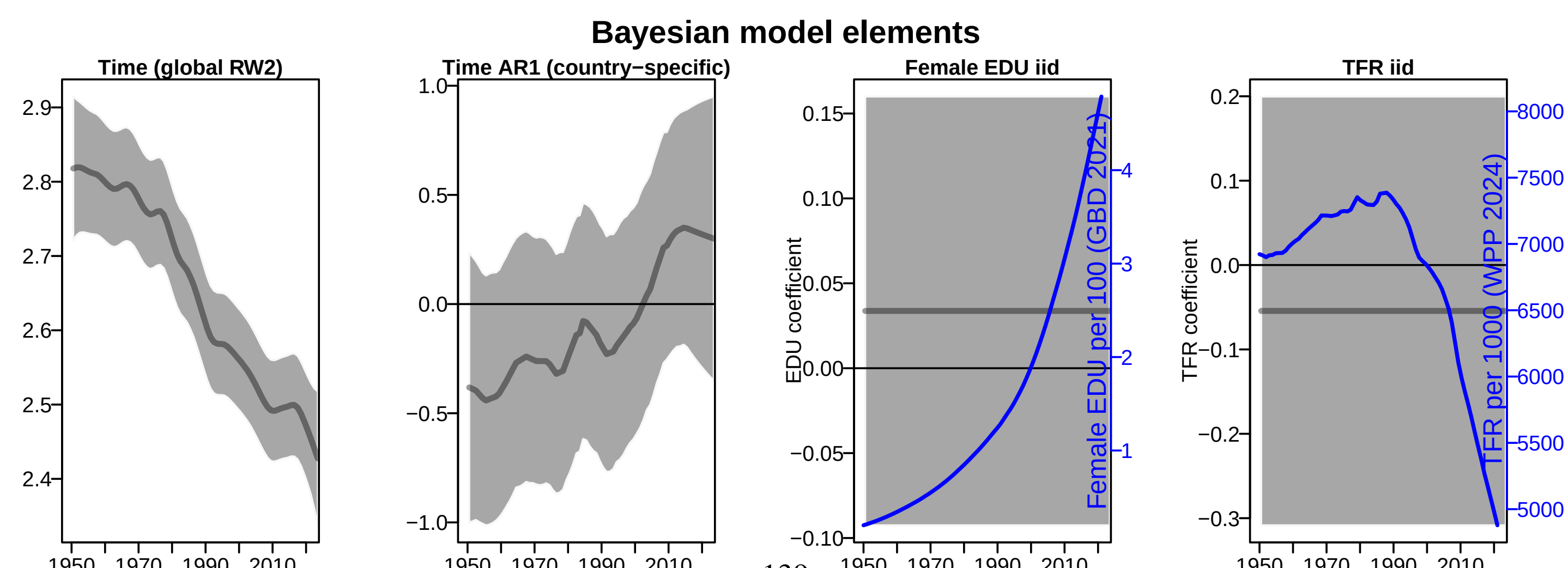
Bayesian model elements
Time (global RW2)
Time AR1 (country−specific)
Female EDU iid
TFR iid
EDU coefficient
Female EDU per 100 (GBD 2021)
TFR coefficient
TFR per 1000 (WPP 2024)
1950
1970
1990
2010

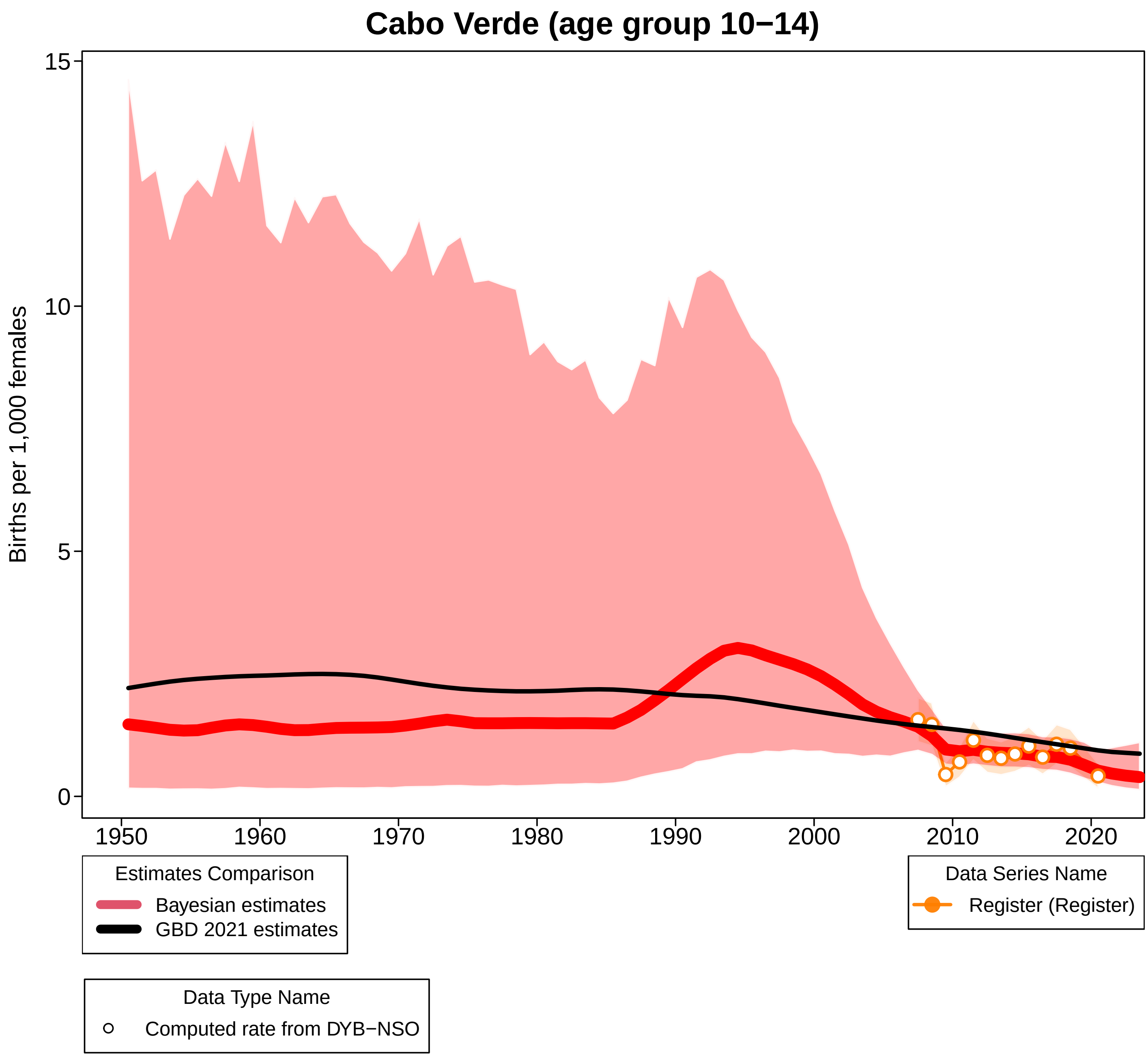
Cabo Verde (age group 10−14)
Births per 1,000 females
15
10
5
0
1950
1960
1970
1980
1990
2000
2010
2020
Estimates Comparison
Bayesian estimates
GBD 2021 estimates
Data Series Name
Register (Register)
Data Type Name
Computed rate from DYB−NSO

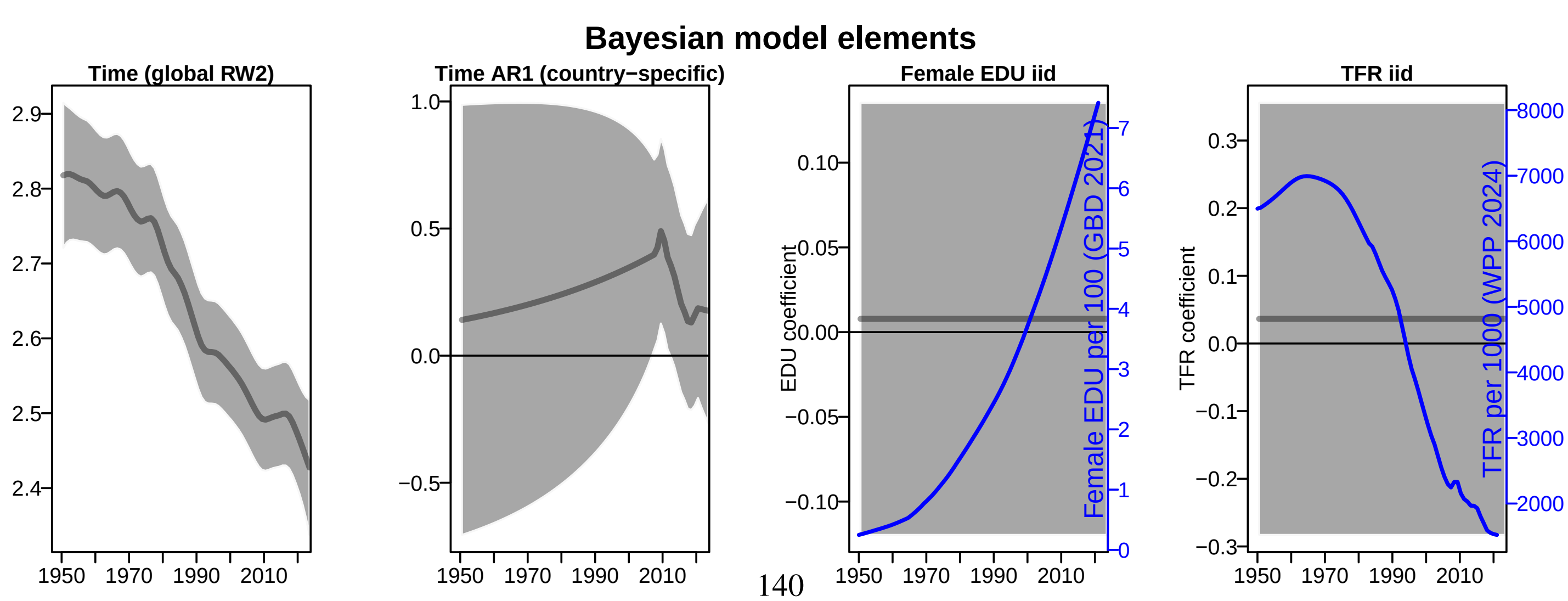
Bayesian model elements
Time (global RW2)
Time AR1 (country−specific)
Female EDU iid
TFR iid
EDU coefficient
Female EDU per 100 (GBD 2021)
TFR coefficient
TFR per 1000 (WPP 2024)
1950
1970
1990
2010

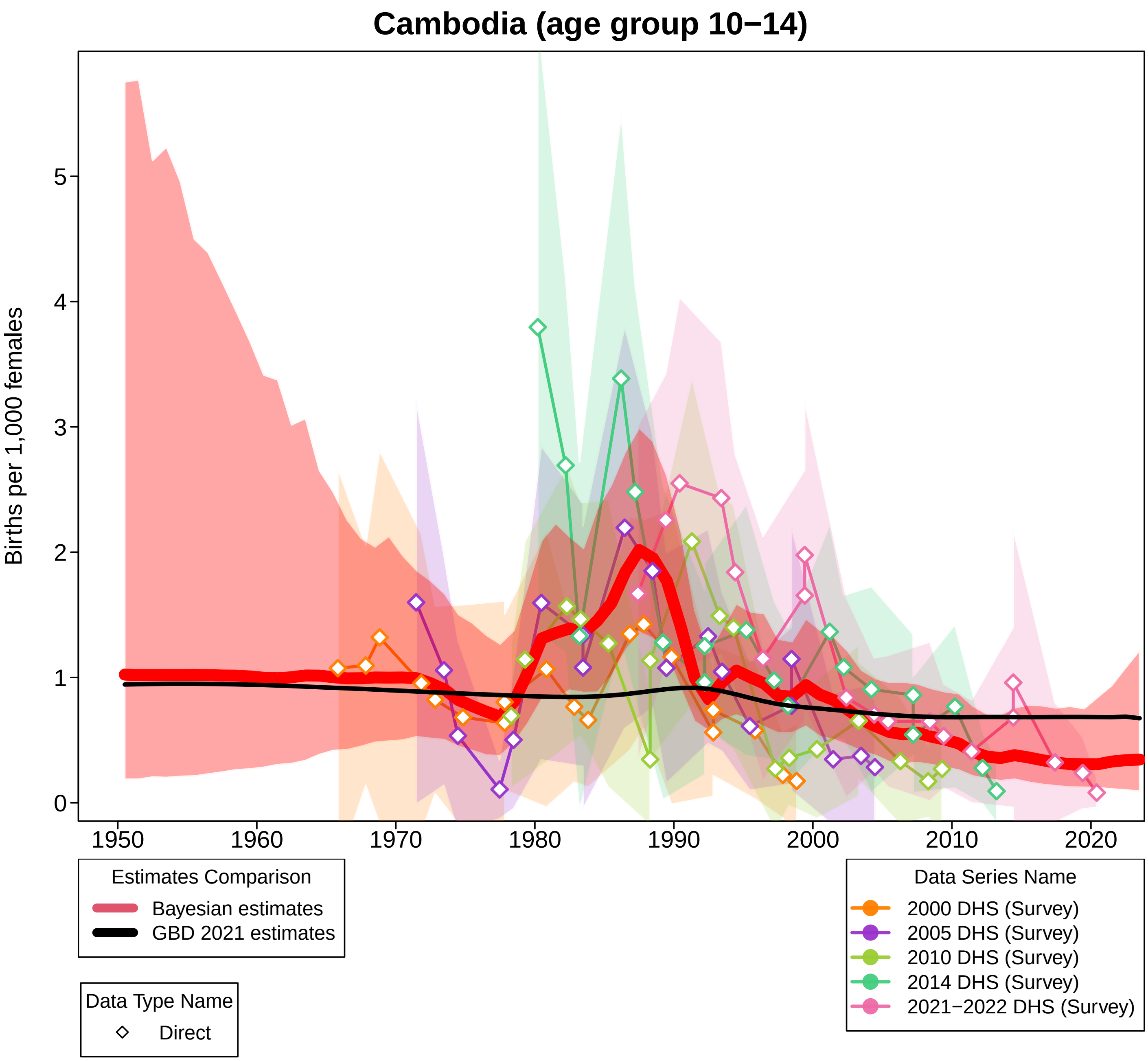
Cambodia (age group 10−14)
Births per 1,000 females
0
1
2
3
4
5
1950
1960
1970
1980
1990
2000
2010
2020
Estimates Comparison
Bayesian estimates
GBD 2021 estimates
Data Type Name
Direct
Data Series Name
2000 DHS (Survey)
2005 DHS (Survey)
2010 DHS (Survey)
2014 DHS (Survey)
2021−2022 DHS (Survey)

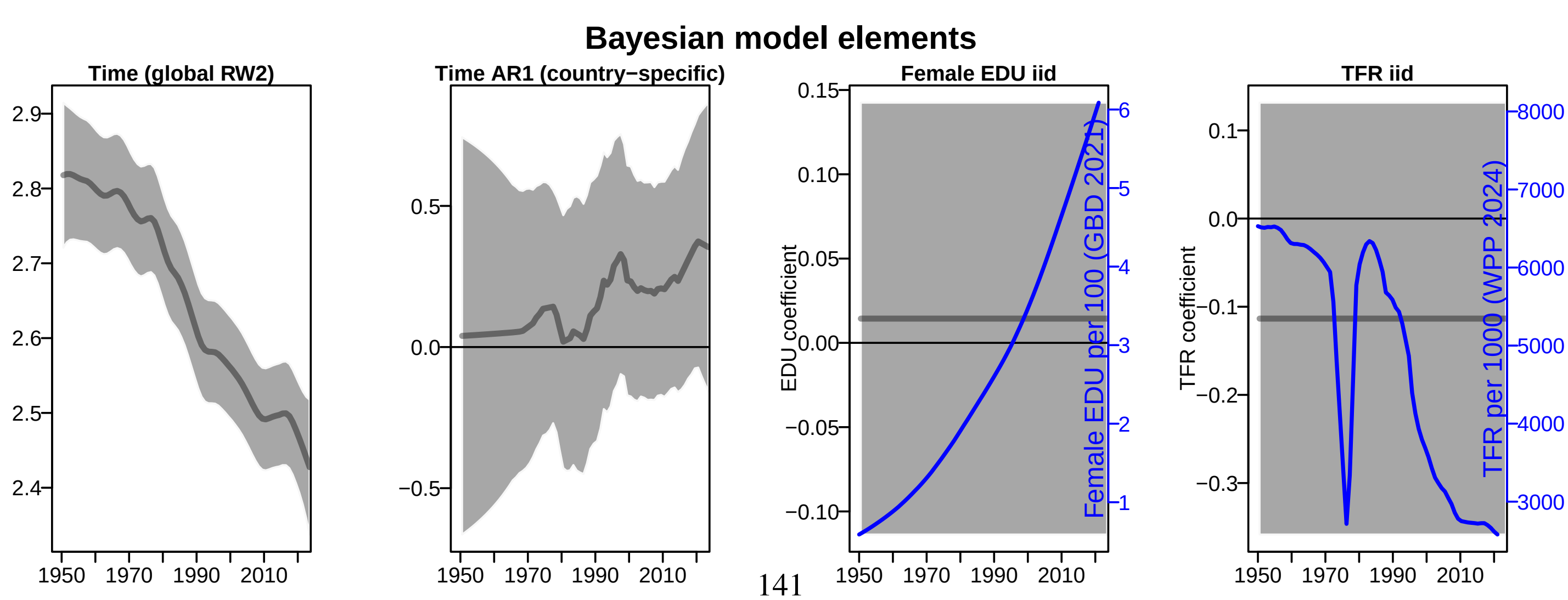
Bayesian model elements
Time (global RW2)
Time AR1 (country−specific)
Female EDU iid
TFR iid
EDU coefficient
Female EDU per 100 (GBD 2021)
TFR coefficient
TFR per 1000 (WPP 2024)
1950
1970
1990
2010

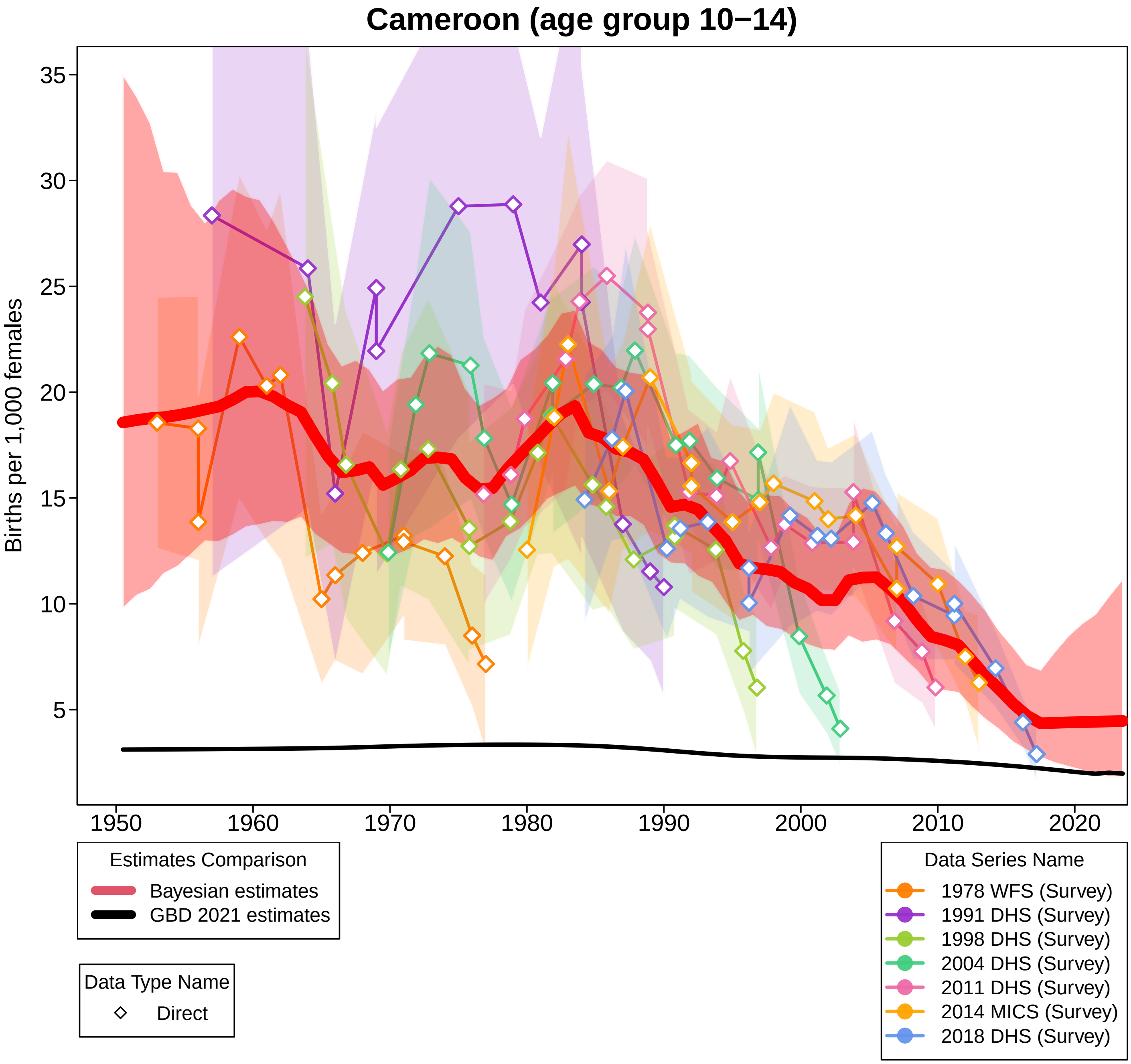
Cameroon (age group 10−14)
Births per 1,000 females
35
30
25
20
15
10
5
1950
1960
1970
1980
1990
2000
2010
2020
Estimates Comparison
Bayesian estimates
GBD 2021 estimates
Data Type Name
Direct
Data Series Name
1978 WFS (Survey)
1991 DHS (Survey)
1998 DHS (Survey)
2004 DHS (Survey)
2011 DHS (Survey)
2014 MICS (Survey)
2018 DHS (Survey)

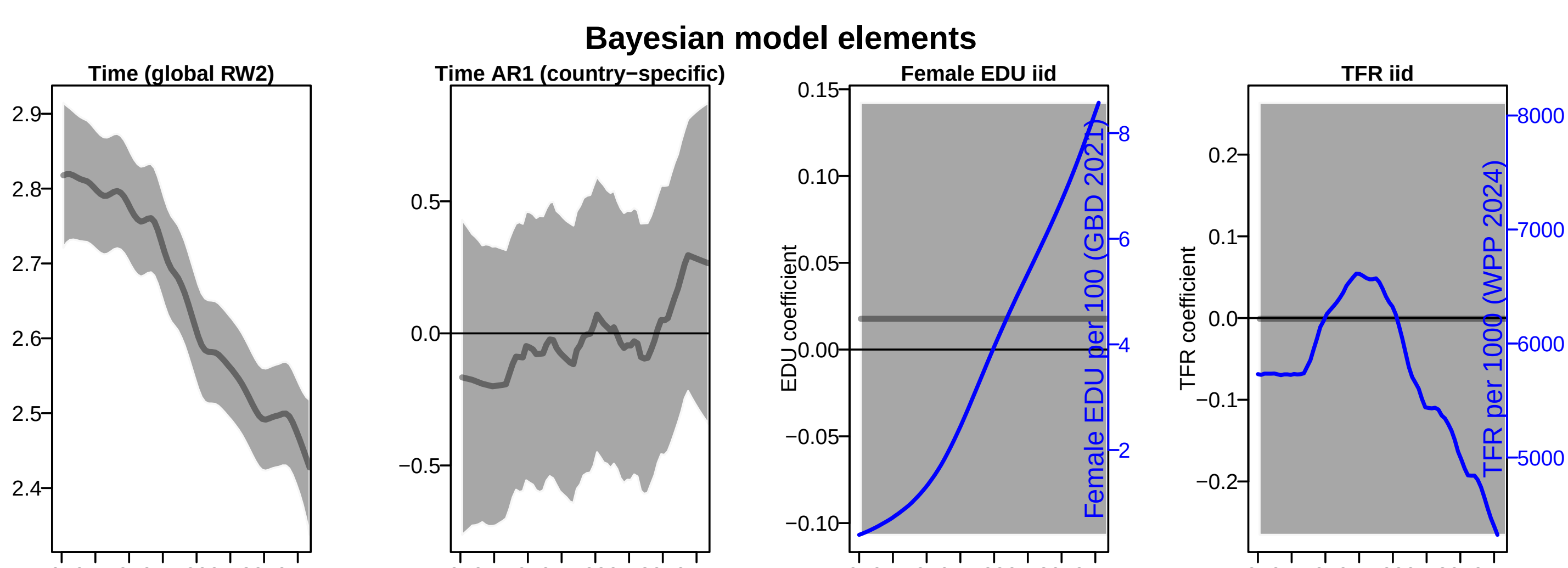
Bayesian model elements
Time (global RW2)
Time AR1 (country−specific)
Female EDU iid
TFR iid
EDU coefficient
Female EDU per 100 (GBD 2021)
TFR coefficient
TFR per 1000 (WPP 2024)
2.9
2.8
2.7
2.6
2.5
2.4
0.5
0.0
−0.5
0.15
0.10
0.05
0.00
−0.05
−0.10
8
6
4
2
0.2
0.1
0.0
−0.1
−0.2
8000
7000
6000
5000
1950
1970
1990
2010

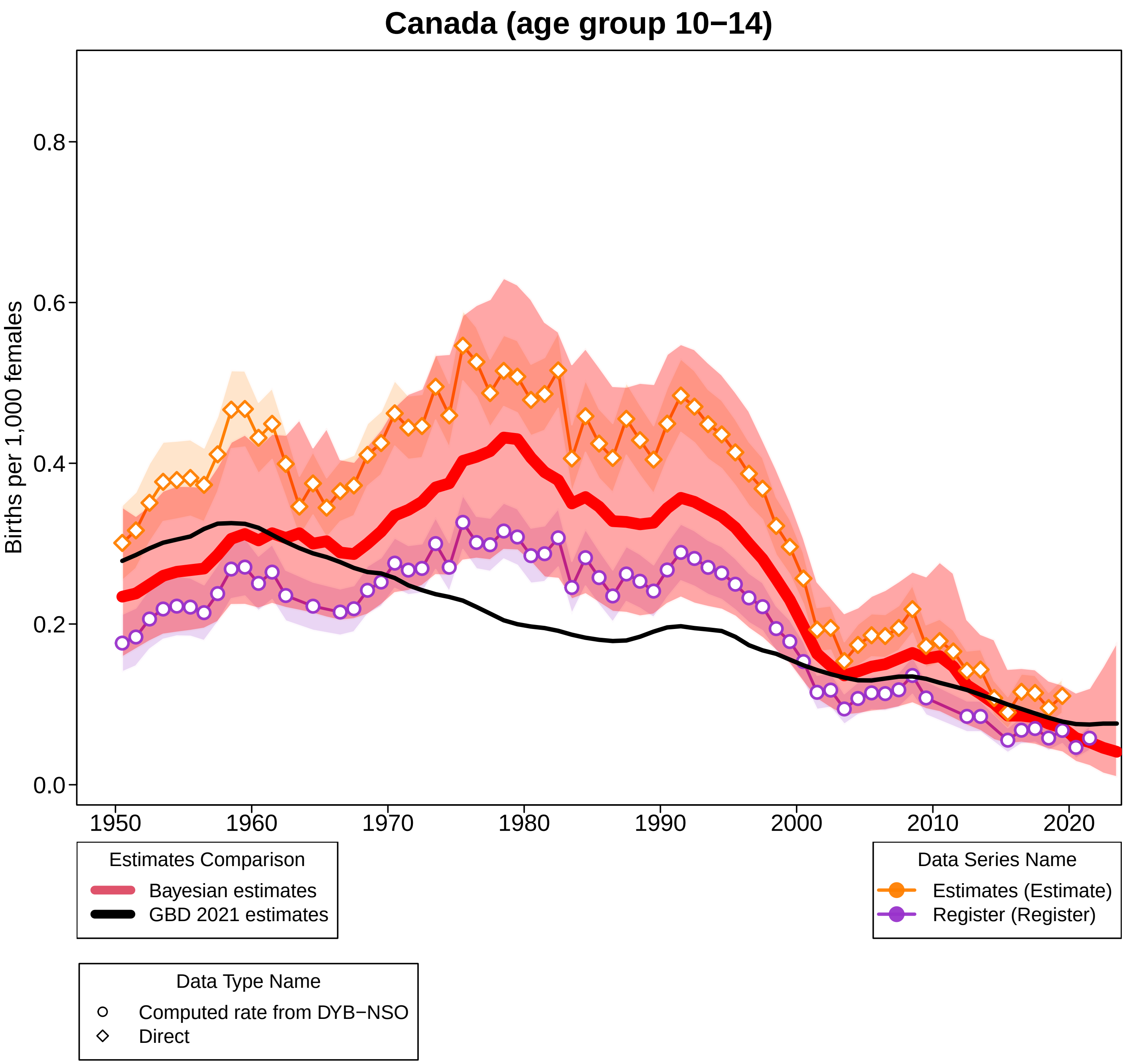
Canada (age group 10−14)
Births per 1,000 females
0.0
0.2
0.4
0.6
0.8
1950
1960
1970
1980
1990
2000
2010
2020
Estimates Comparison
Bayesian estimates
GBD 2021 estimates
Data Series Name
Estimates (Estimate)
Register (Register)
Data Type Name
Computed rate from DYB−NSO
Direct

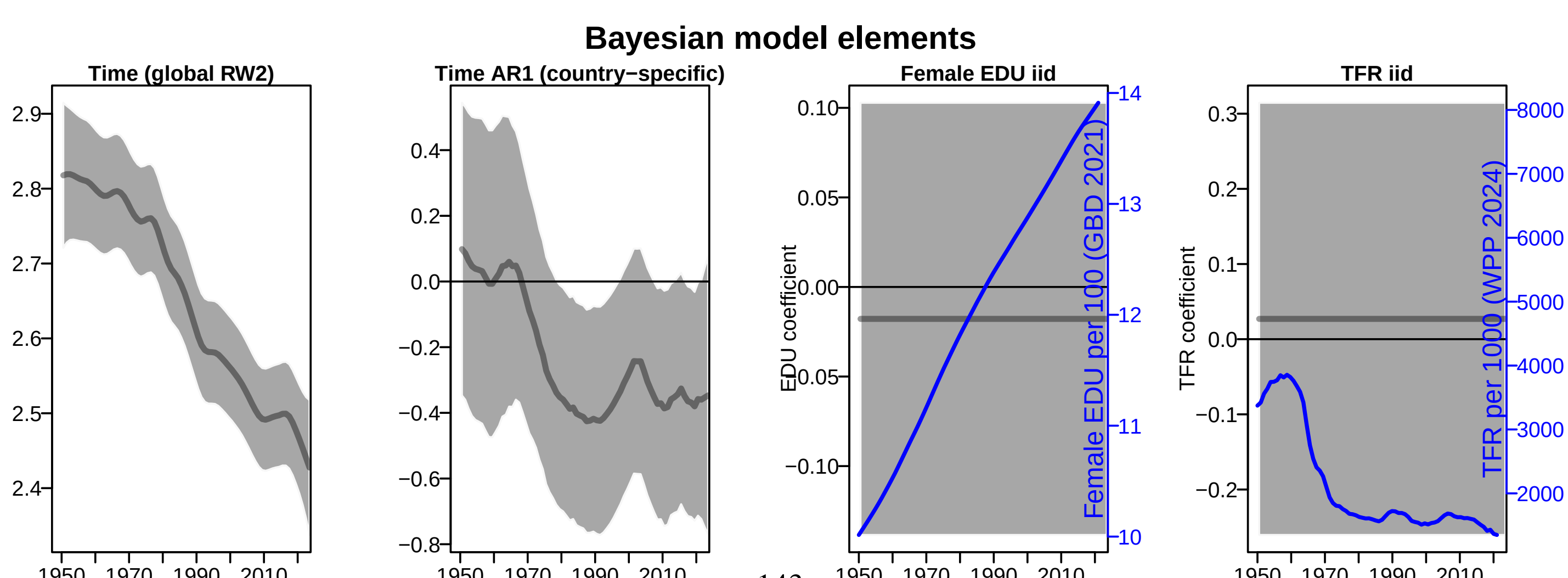
Bayesian model elements
Time (global RW2)
Time AR1 (country−specific)
Female EDU iid
TFR iid
EDU coefficient
Female EDU per 100 (GBD 2021)
TFR coefficient
TFR per 1000 (WPP 2024)
1950
1970
1990
2010

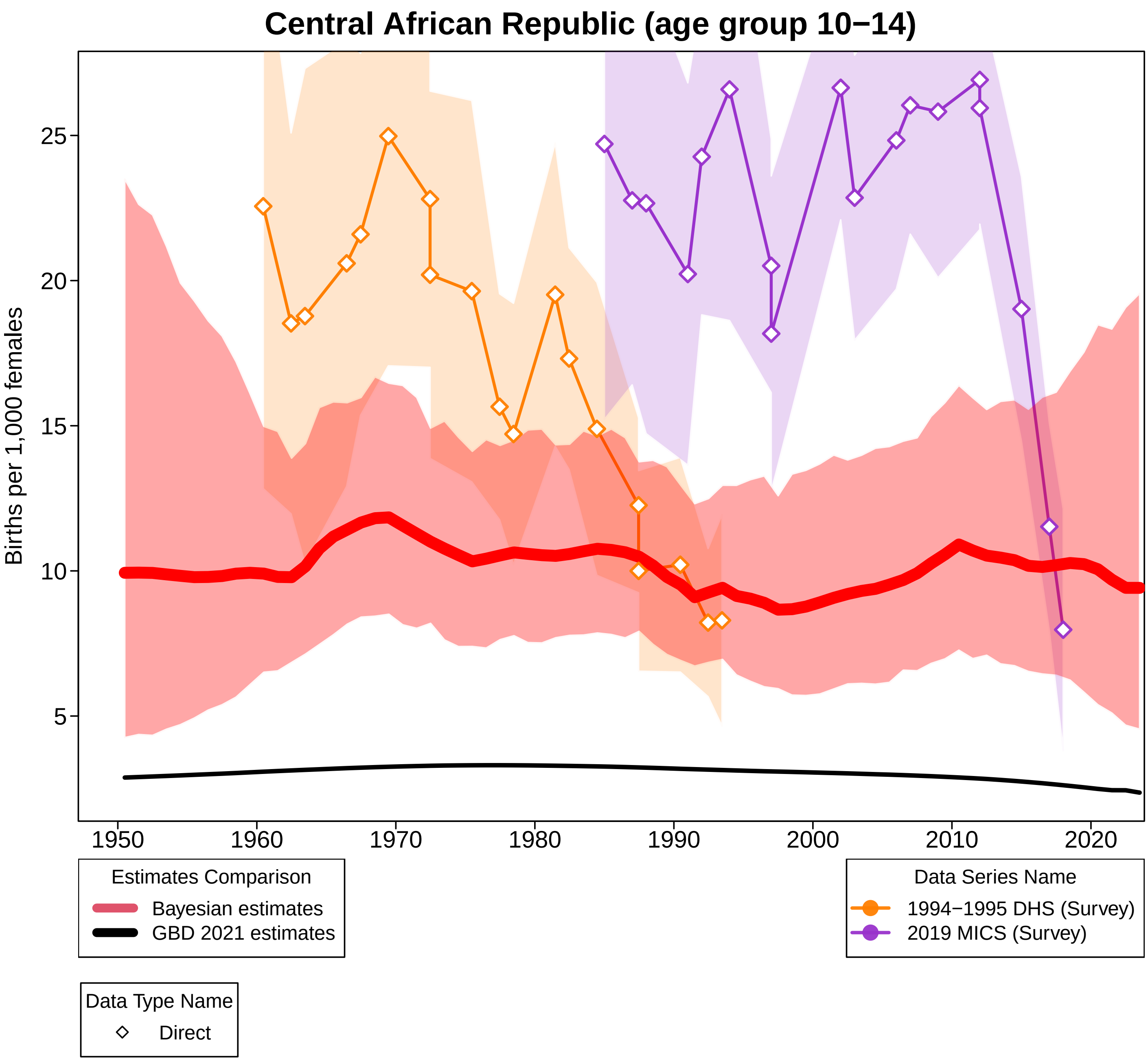

Central African Republic (age group 10−14)
Births per 1,000 females
Estimates Comparison
Bayesian estimates
GBD 2021 estimates
Data Series Name
1994−1995 DHS (Survey)
2019 MICS (Survey)
Data Type Name
Direct

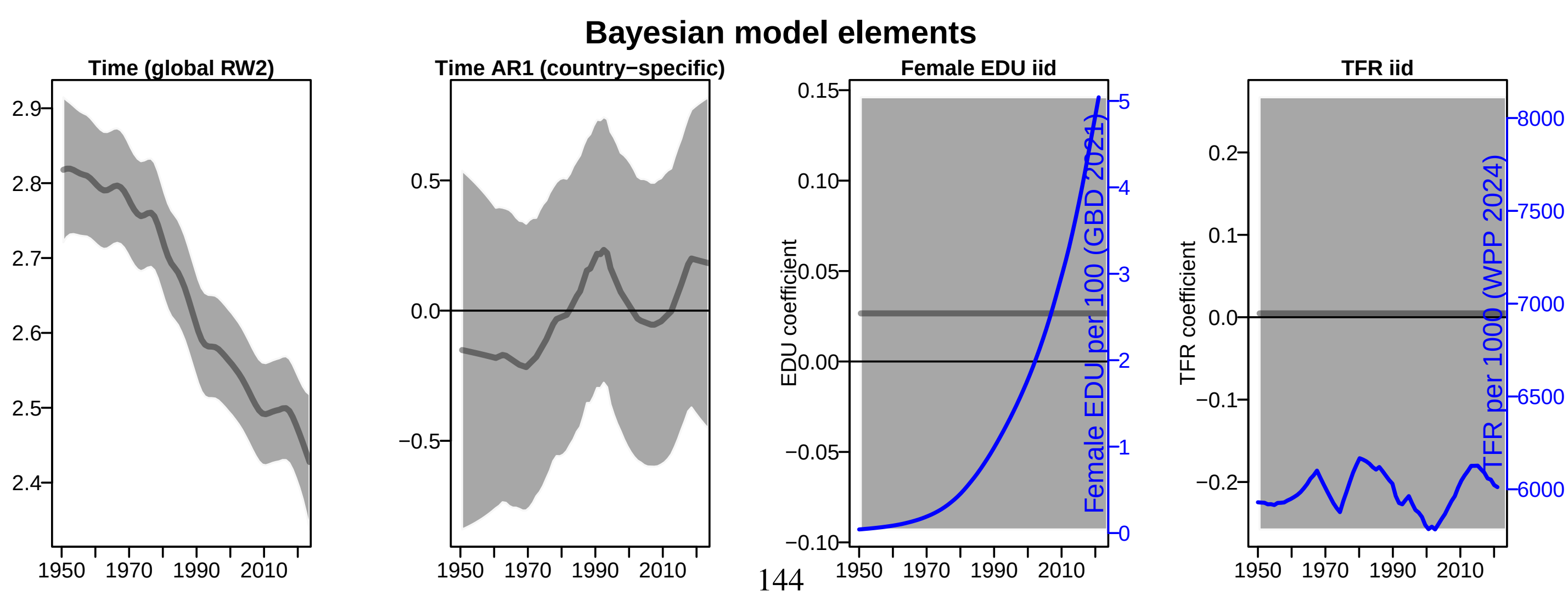

Bayesian model elements
Time (global RW2)
Time AR1 (country−specific)
Female EDU iid
EDU coefficient
Female EDU per 100 (GBD 2021)
TFR iid
TFR coefficient
TFR per 1000 (WPP 2024)

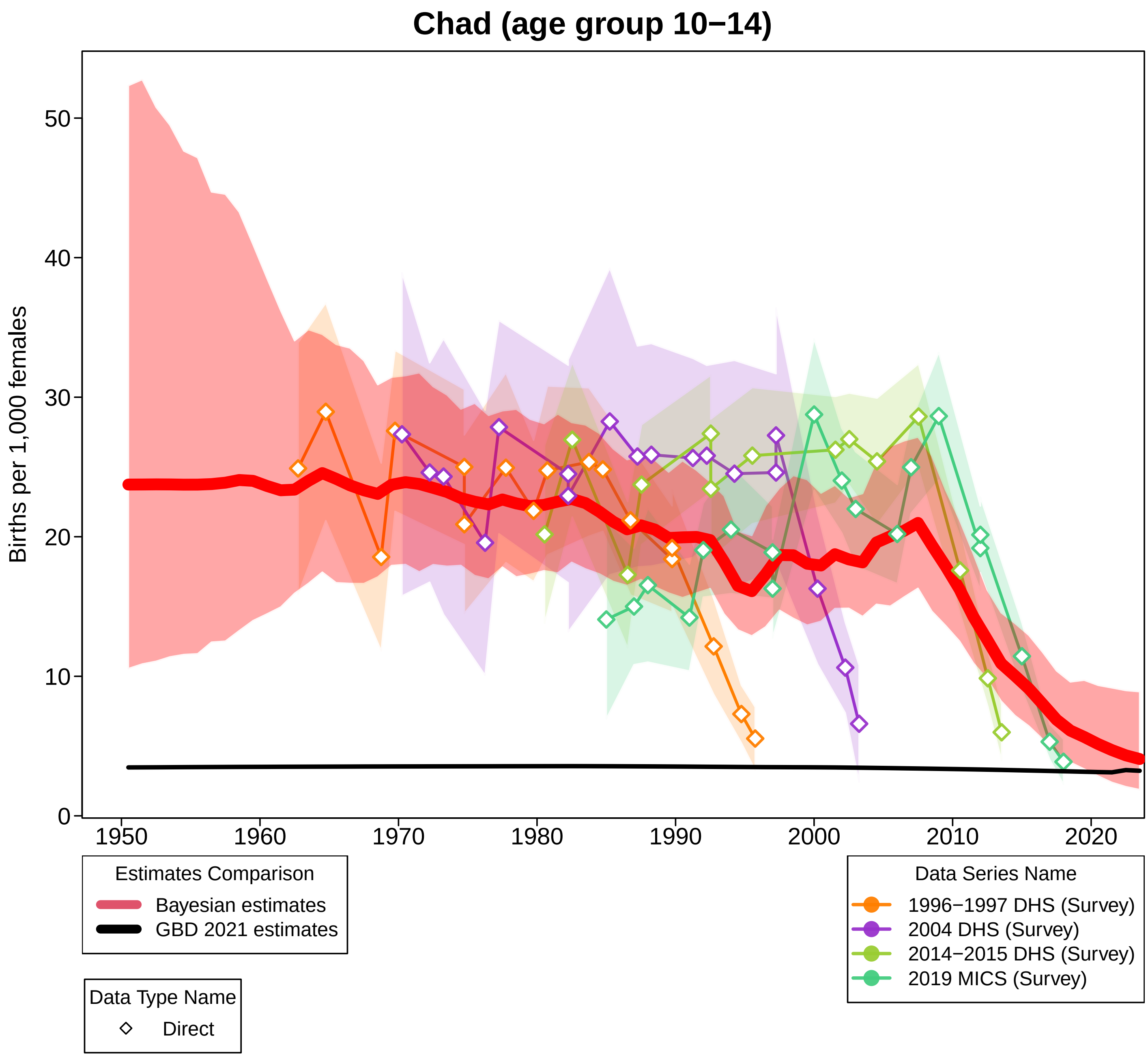

Chad (age group 10−14)
Births per 1,000 females
0
10
20
30
40
50
1950
1960
1970
1980
1990
2000
2010
2020
Estimates Comparison
Bayesian estimates
GBD 2021 estimates
Data Series Name
1996−1997 DHS (Survey)
2004 DHS (Survey)
2014−2015 DHS (Survey)
2019 MICS (Survey)
Data Type Name
Direct

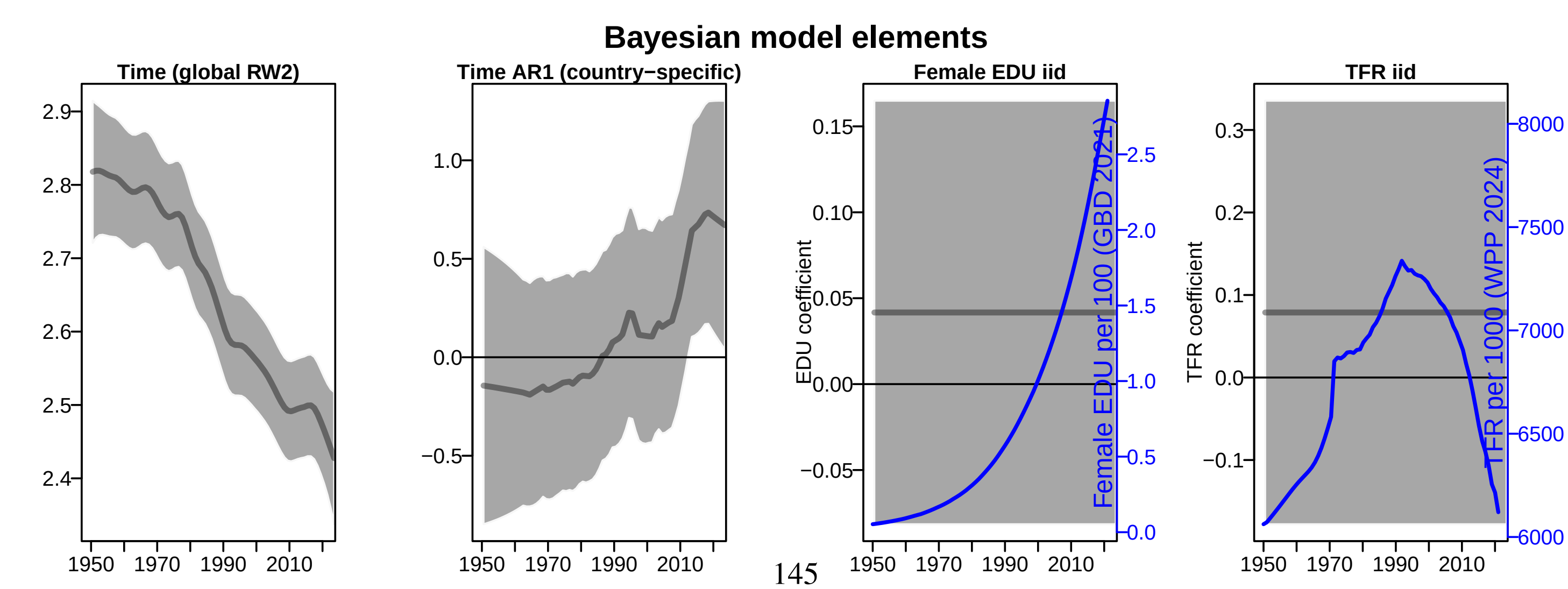

Bayesian model elements
Time (global RW2)
Time AR1 (country−specific)
Female EDU iid
TFR iid
EDU coefficient
Female EDU per 100 (GBD 2021)
TFR coefficient
TFR per 1000 (WPP 2024)
1950
1970
1990
2010

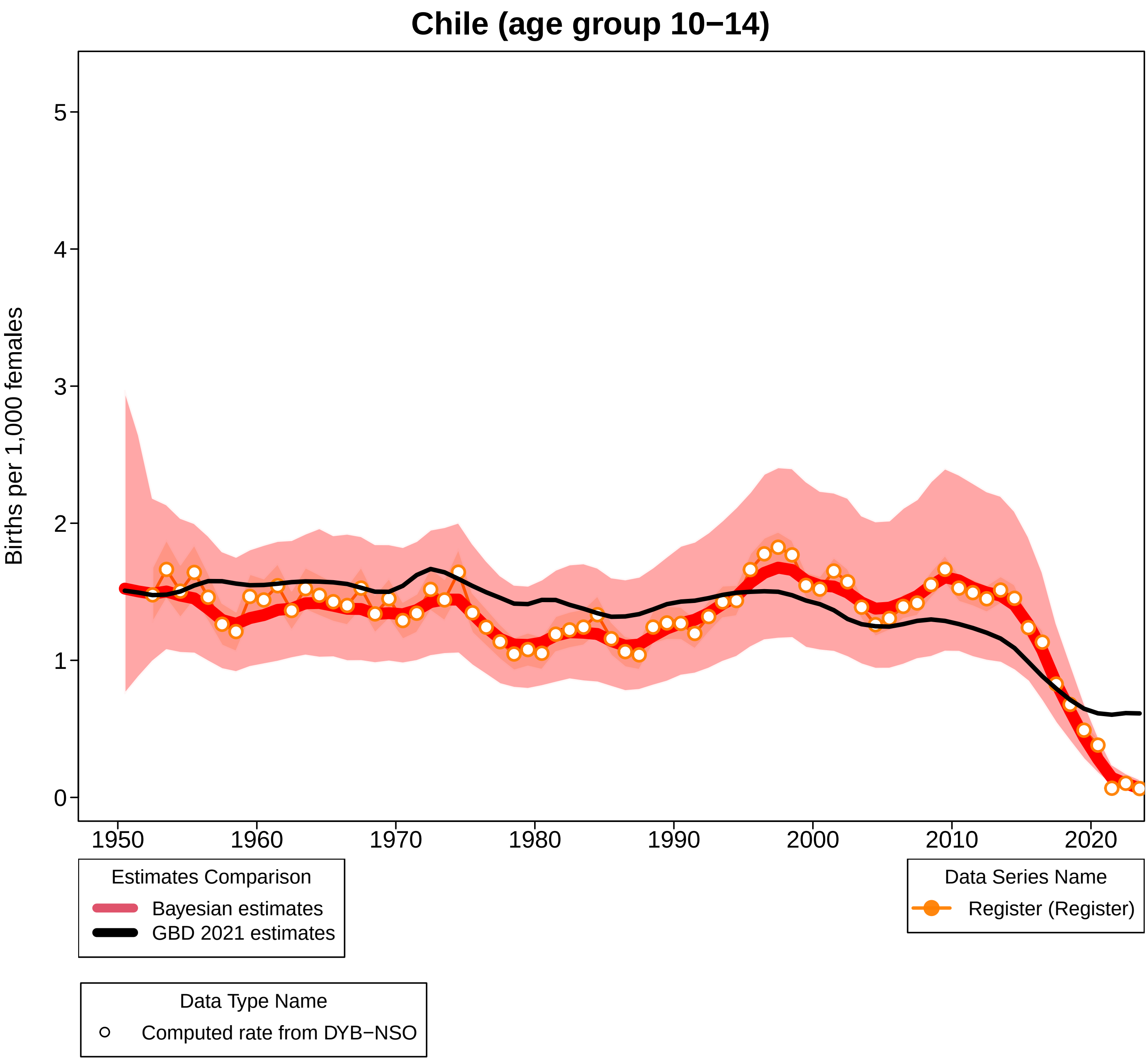
Chile (age group 10−14)
Births per 1,000 females
0
1
2
3
4
5
1950
1960
1970
1980
1990
2000
2010
2020
Estimates Comparison
Bayesian estimates
GBD 2021 estimates
Data Series Name
Register (Register)
Data Type Name
Computed rate from DYB−NSO

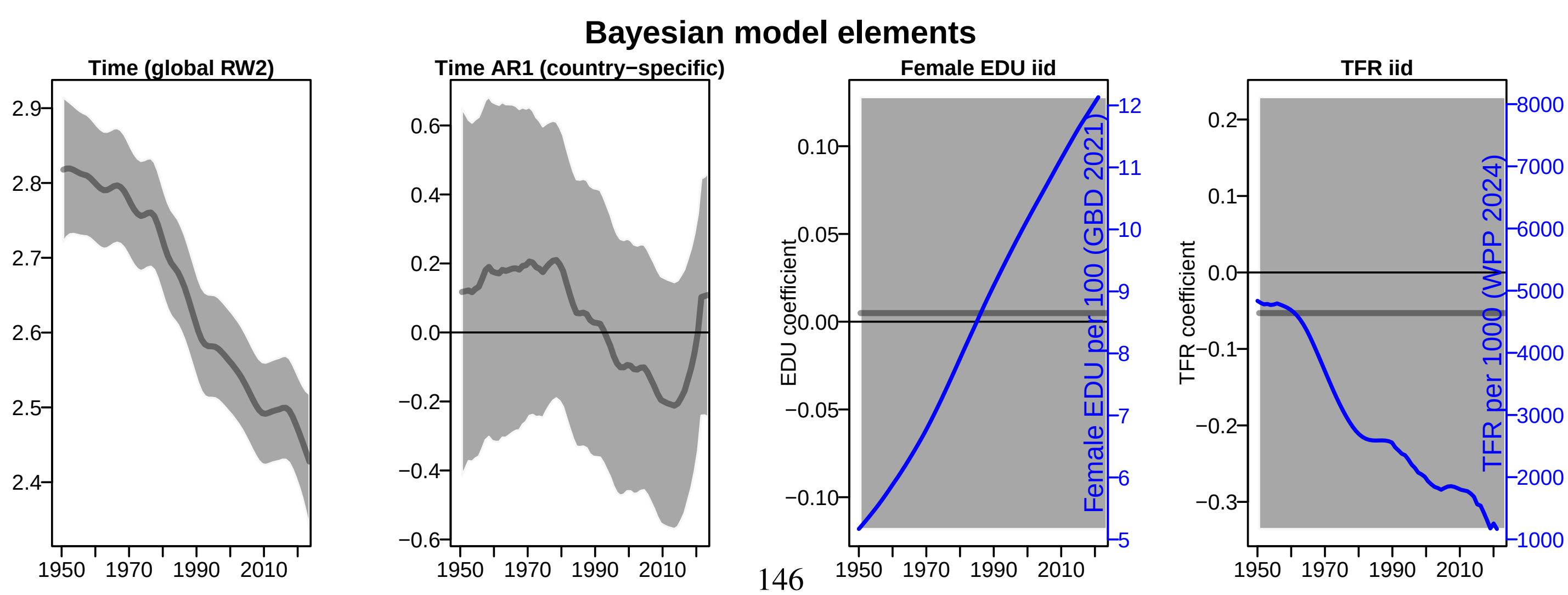
Bayesian model elements
Time (global RW2)
Time AR1 (country−specific)
Female EDU iid
TFR iid
EDU coefficient
Female EDU per 100 (GBD 2021)
TFR coefficient
TFR per 1000 (WPP 2024)
1950
1970
1990
2010

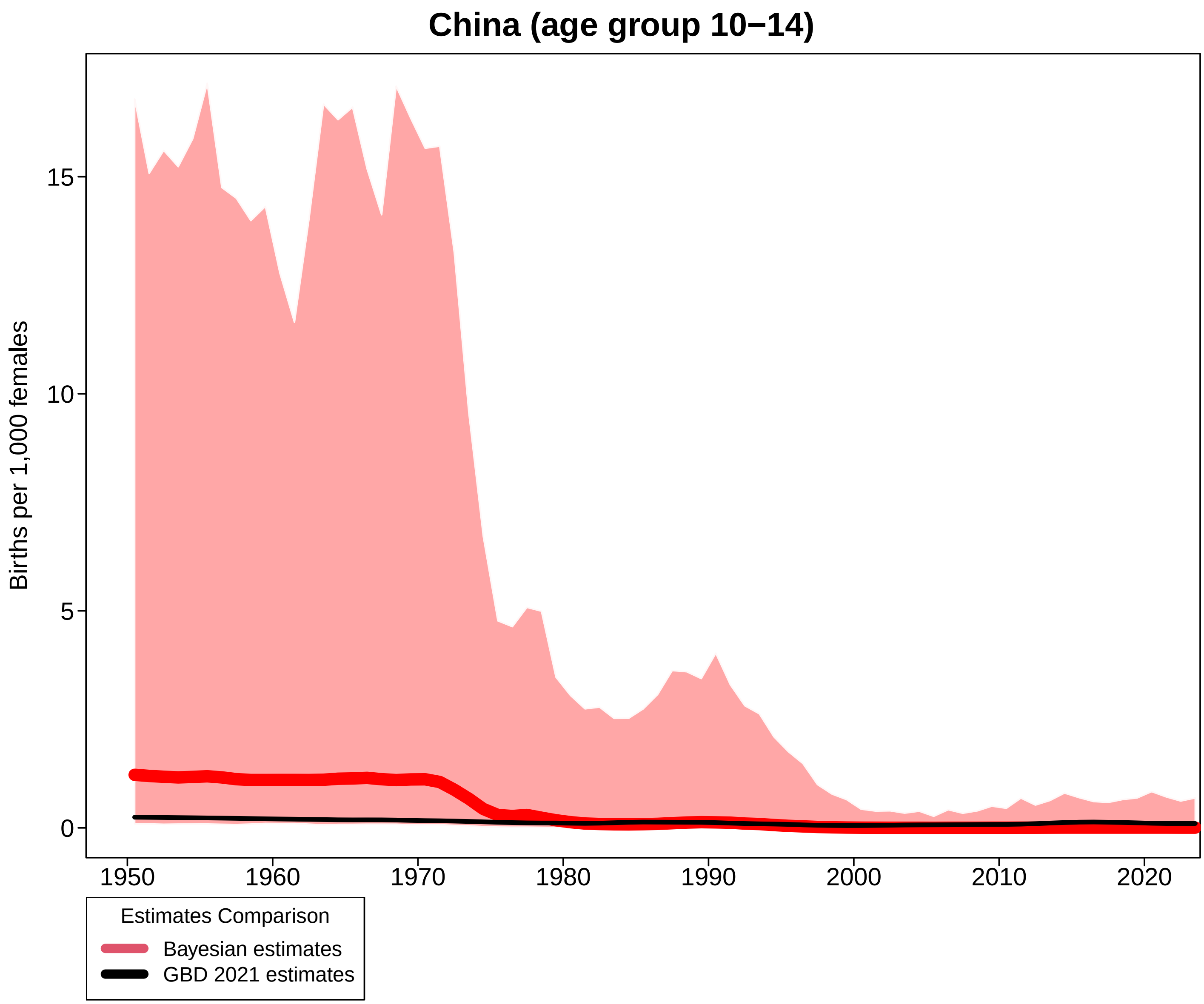
China (age group 10−14)
Births per 1,000 females
15
10
5
0
1950
1960
1970
1980
1990
2000
2010
2020
Estimates Comparison
Bayesian estimates
GBD 2021 estimates

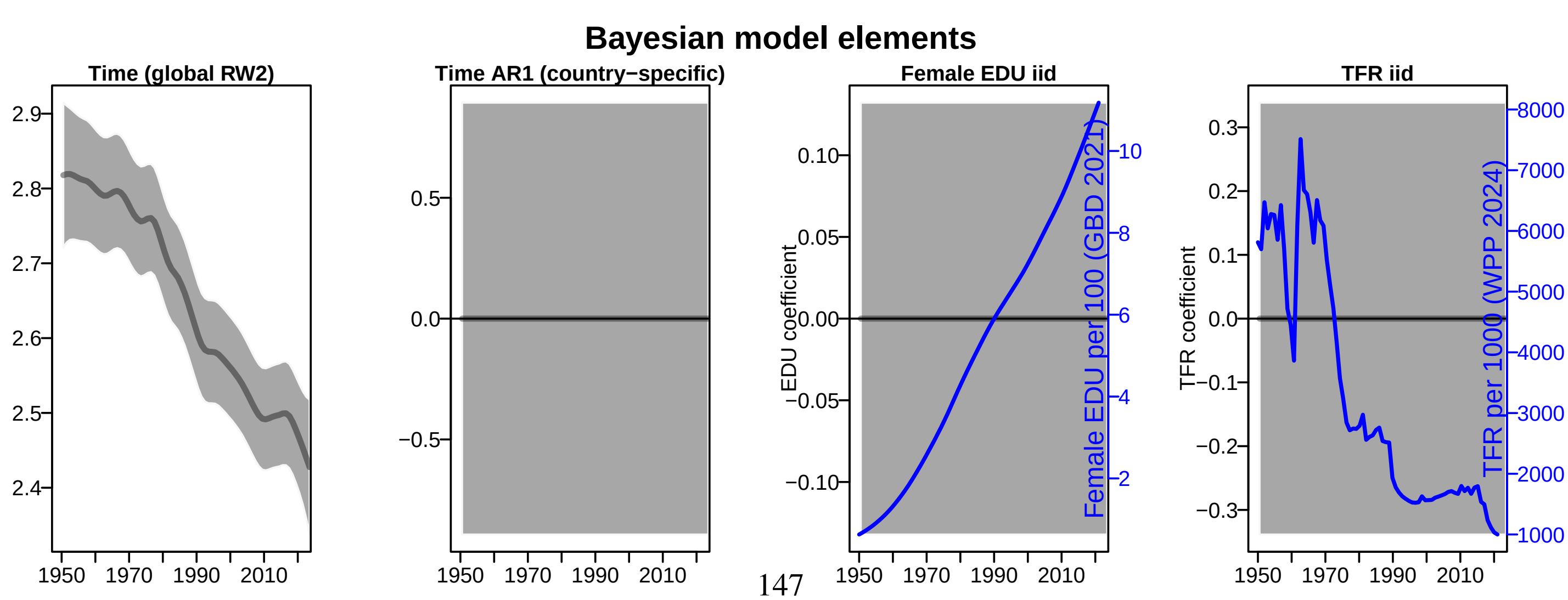
Bayesian model elements
Time (global RW2)
Time AR1 (country−specific)
Female EDU iid
TFR iid
EDU coefficient
Female EDU per 100 (GBD 2021)
TFR coefficient
TFR per 1000 (WPP 2024)
1950
1970
1990
2010

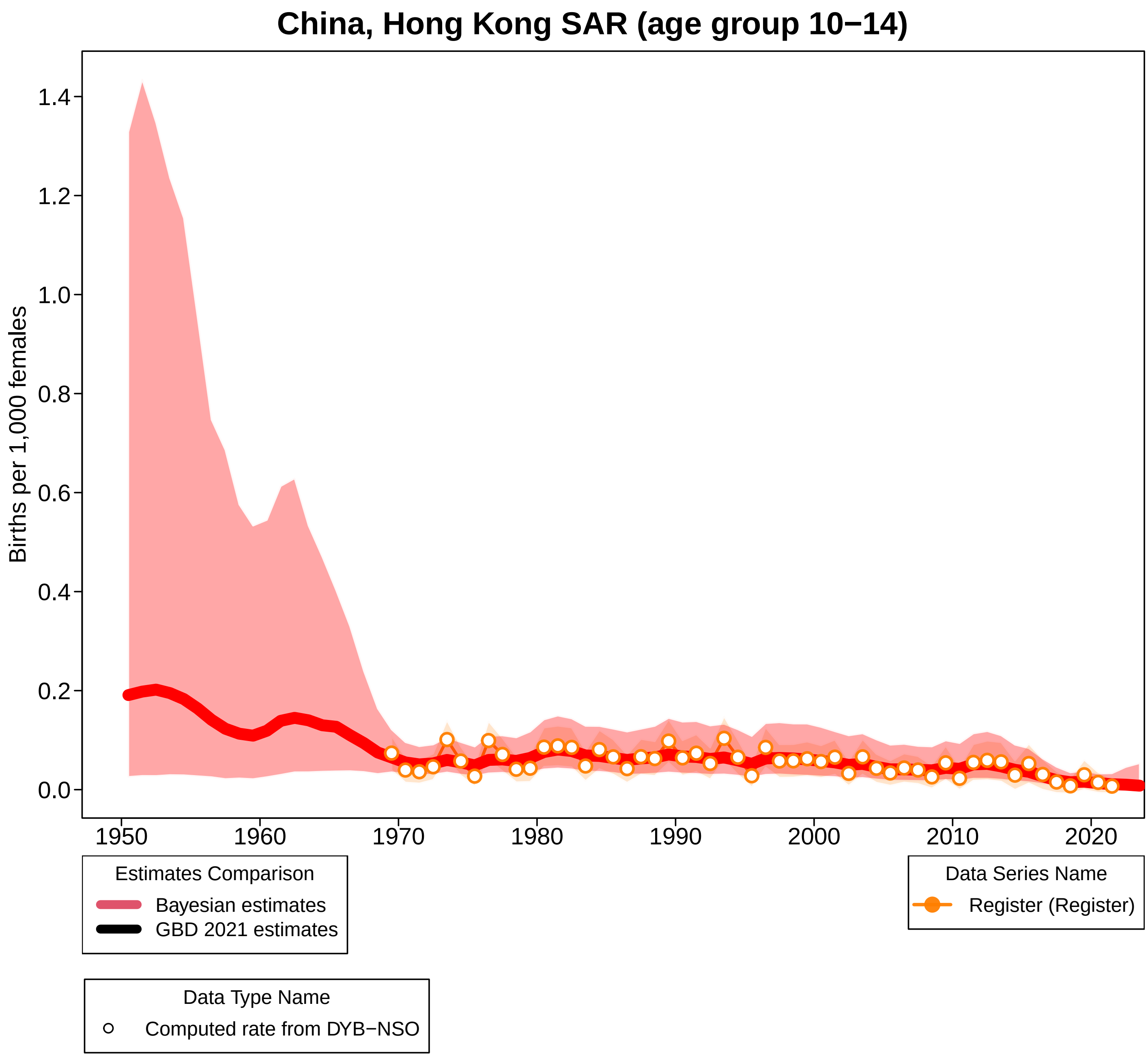
China, Hong Kong SAR (age group 10−14)
Births per 1,000 females
0.0
0.2
0.4
0.6
0.8
1.0
1.2
1.4
1950
1960
1970
1980
1990
2000
2010
2020
Estimates Comparison
Bayesian estimates
GBD 2021 estimates
Data Series Name
Register (Register)
Data Type Name
Computed rate from DYB−NSO

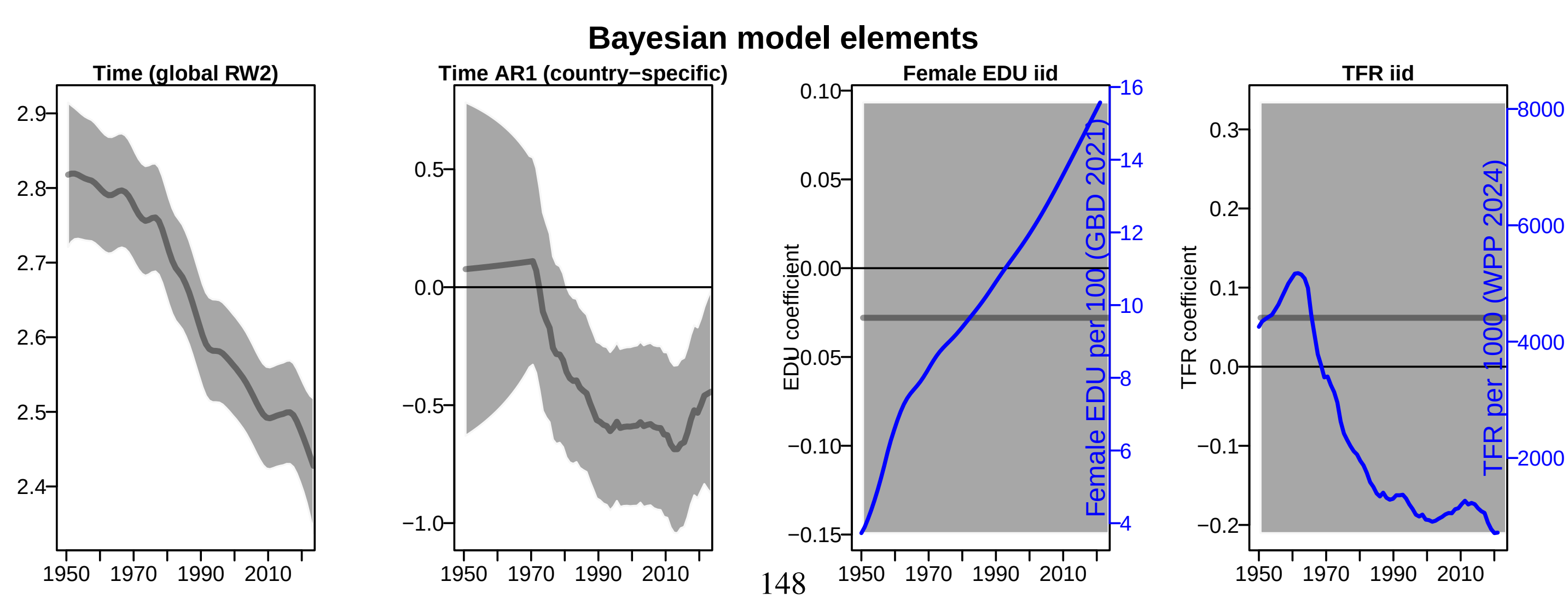
Bayesian model elements
Time (global RW2)
Time AR1 (country−specific)
Female EDU iid
TFR iid
EDU coefficient
Female EDU per 100 (GBD 2021)
TFR coefficient
TFR per 1000 (WPP 2024)
1950
1970
1990
2010

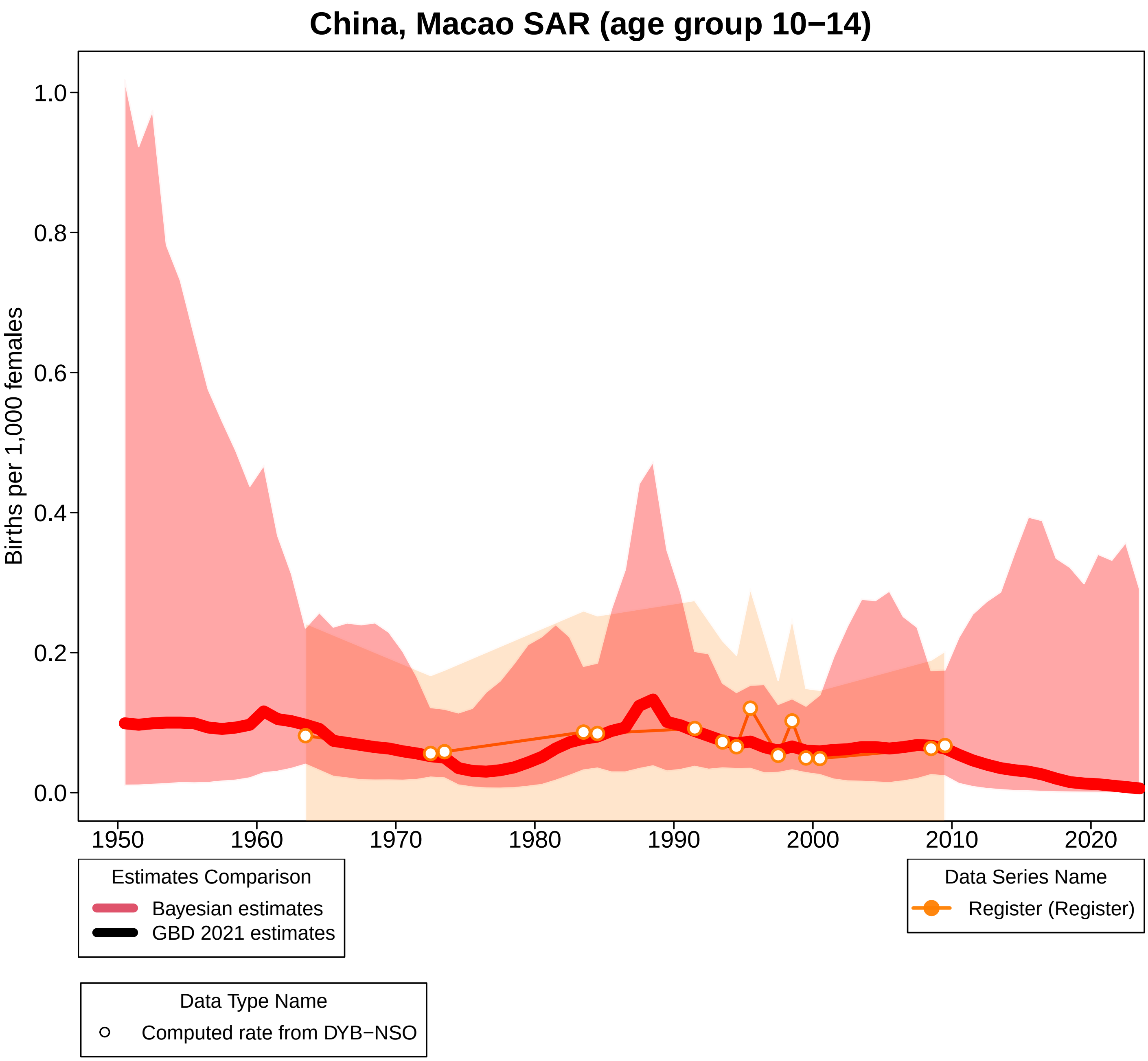
China, Macao SAR (age group 10−14)
Births per 1,000 females
1.0
0.8
0.6
0.4
0.2
0.0
1950
1960
1970
1980
1990
2000
2010
2020
Estimates Comparison
Bayesian estimates
GBD 2021 estimates
Data Series Name
Register (Register)
Data Type Name
Computed rate from DYB−NSO

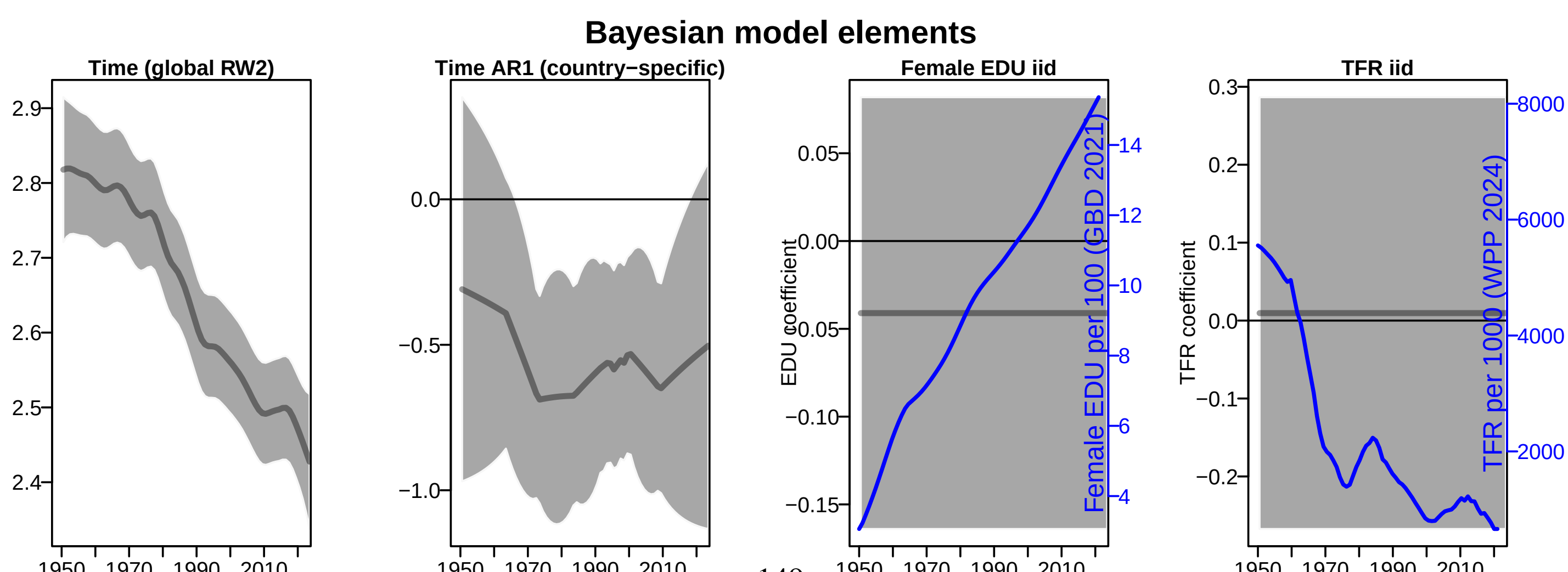
Bayesian model elements
Time (global RW2)
Time AR1 (country−specific)
Female EDU iid
TFR iid
EDU coefficient
Female EDU per 100 (GBD 2021)
TFR coefficient
TFR per 1000 (WPP 2024)

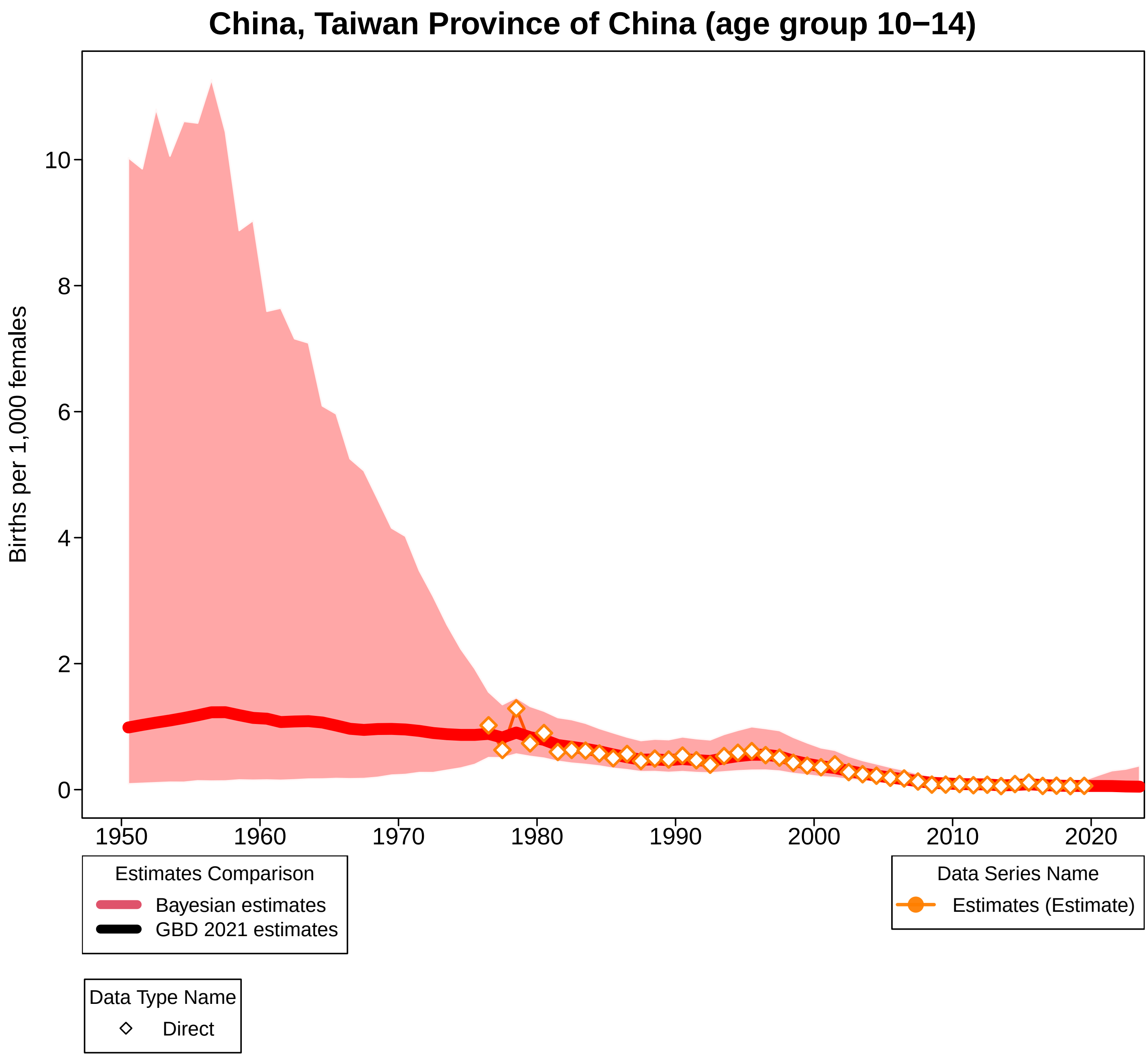
China, Taiwan Province of China (age group 10−14)
Births per 1,000 females
0
2
4
6
8
10
1950
1960
1970
1980
1990
2000
2010
2020
Estimates Comparison
Bayesian estimates
GBD 2021 estimates
Data Series Name
Estimates (Estimate)
Data Type Name
Direct

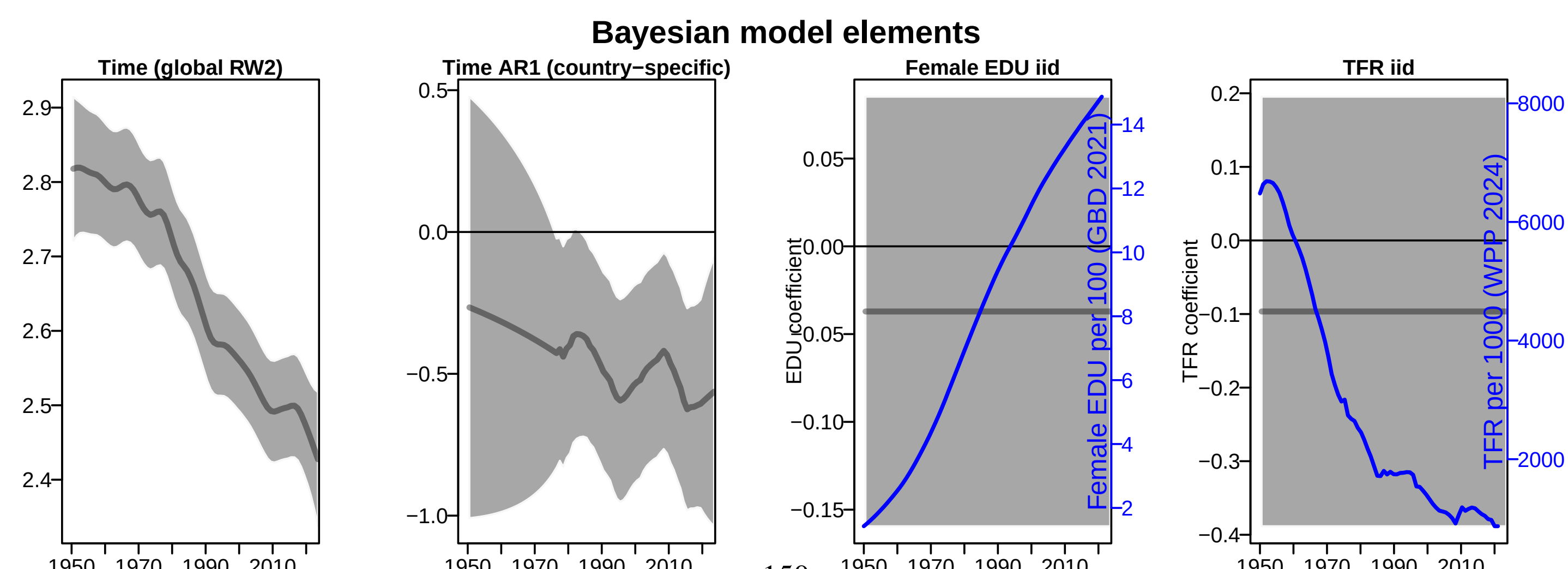
Bayesian model elements
Time (global RW2)
Time AR1 (country−specific)
Female EDU iid
TFR iid
EDU coefficient
Female EDU per 100 (GBD 2021)
TFR coefficient
TFR per 1000 (WPP 2024)
1950
1970
1990
2010

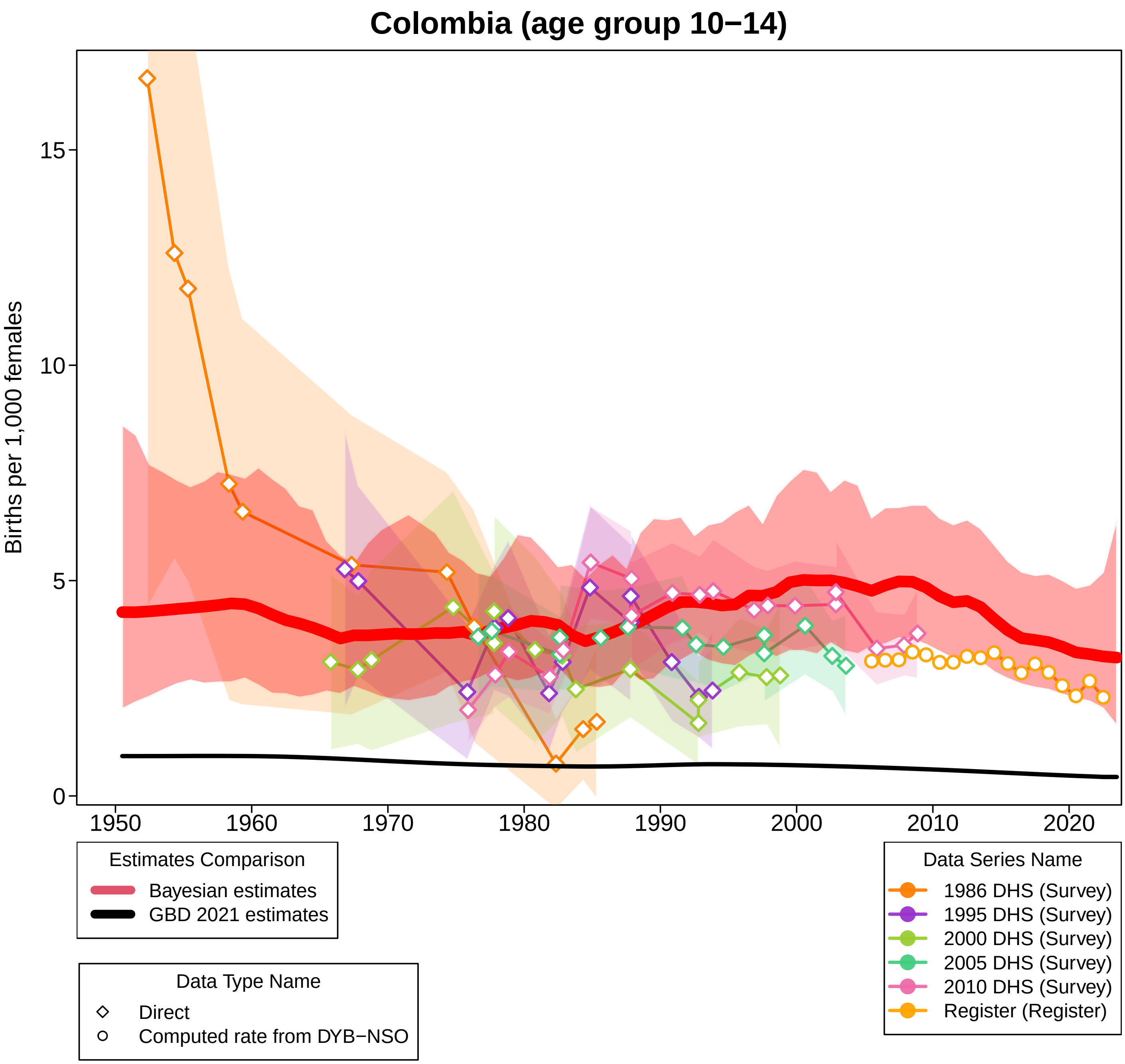

Colombia (age group 10−14)
Births per 1,000 females
0
5
10
15
1950
1960
1970
1980
1990
2000
2010
2020
Estimates Comparison
Bayesian estimates
GBD 2021 estimates
Data Type Name
Direct
Computed rate from DYB−NSO
Data Series Name
1986 DHS (Survey)
1995 DHS (Survey)
2000 DHS (Survey)
2005 DHS (Survey)
2010 DHS (Survey)
Register (Register)

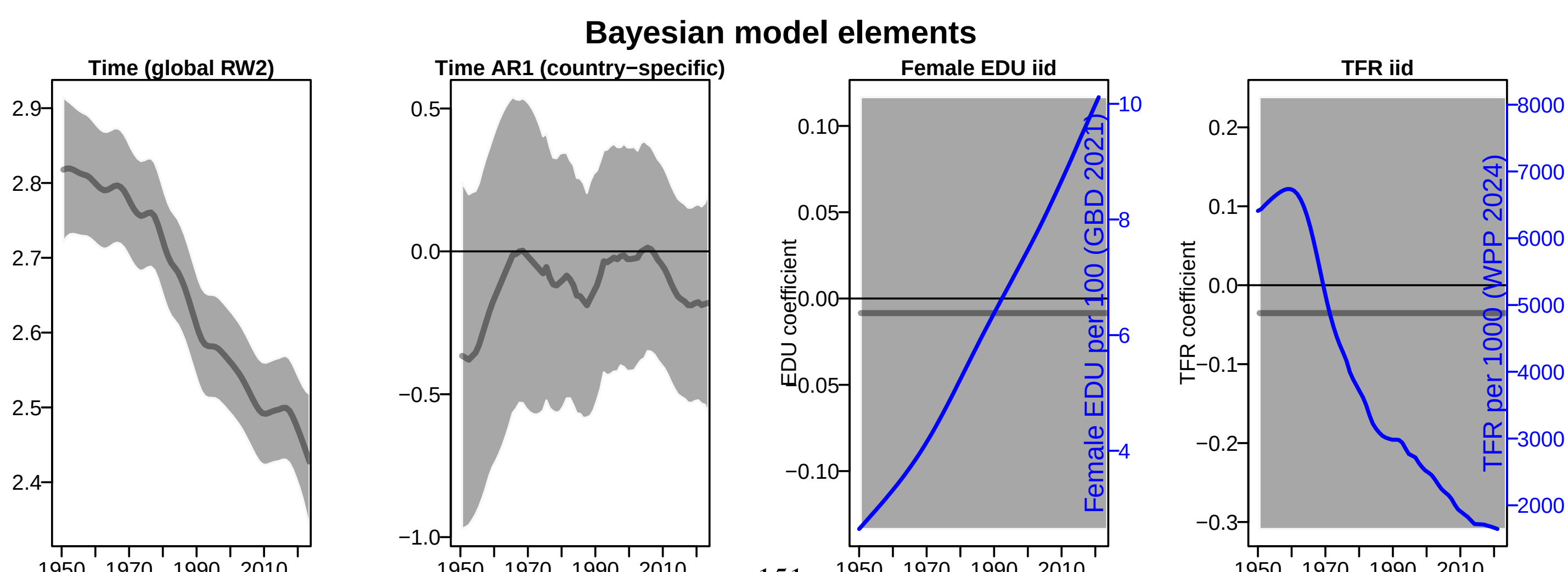

Bayesian model elements
Time (global RW2)
Time AR1 (country−specific)
Female EDU iid
TFR iid
EDU coefficient
Female EDU per 100 (GBD 2021)
TFR coefficient
TFR per 1000 (WPP 2024)

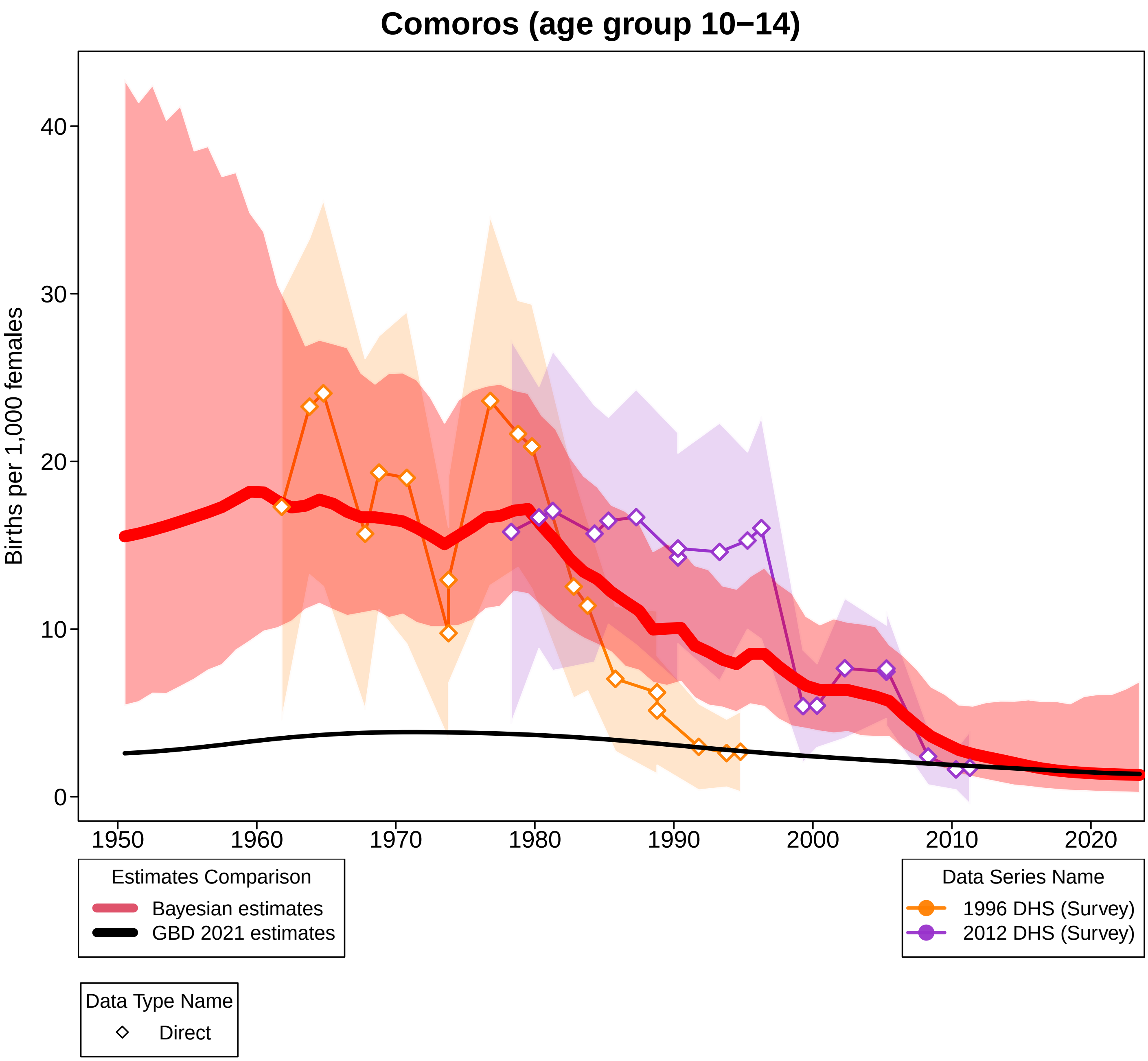

Comoros (age group 10−14)
Births per 1,000 females
0
10
20
30
40
1950
1960
1970
1980
1990
2000
2010
2020
Estimates Comparison
Bayesian estimates
GBD 2021 estimates
Data Series Name
1996 DHS (Survey)
2012 DHS (Survey)
Data Type Name
Direct

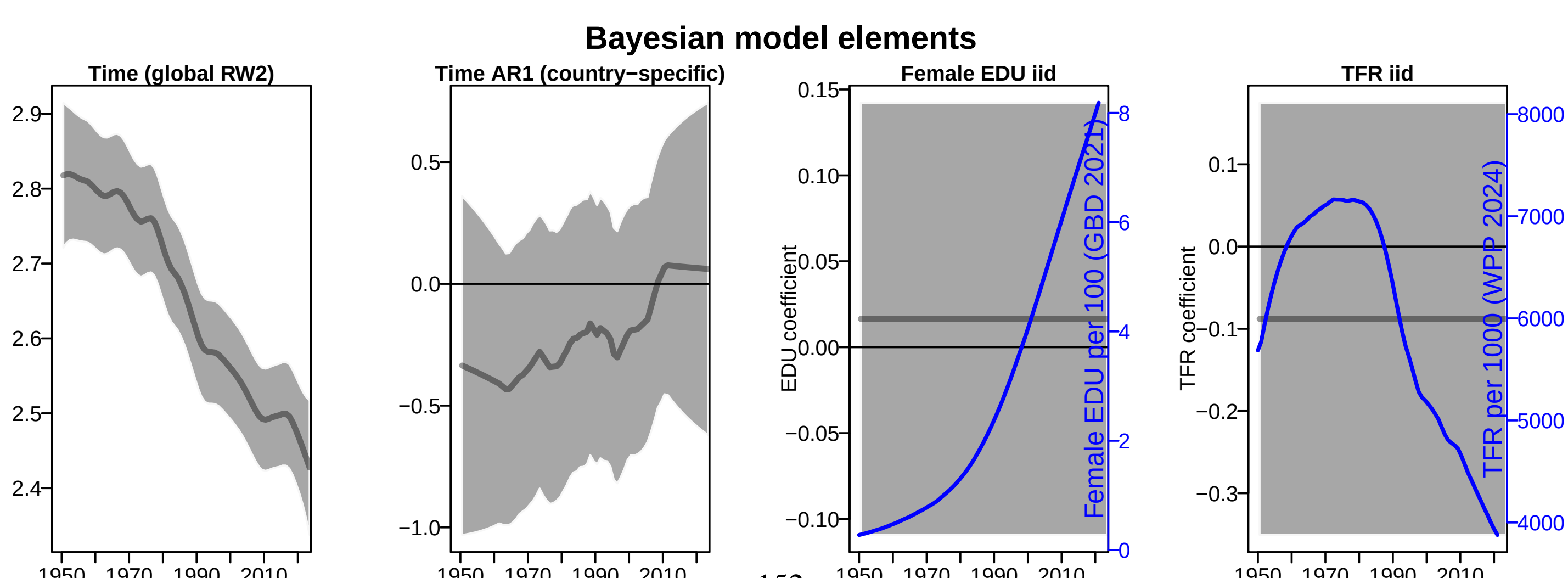

Bayesian model elements
Time (global RW2)
Time AR1 (country−specific)
Female EDU iid
TFR iid
EDU coefficient
Female EDU per 100 (GBD 2021)
TFR coefficient
TFR per 1000 (WPP 2024)
1950
1970
1990
2010

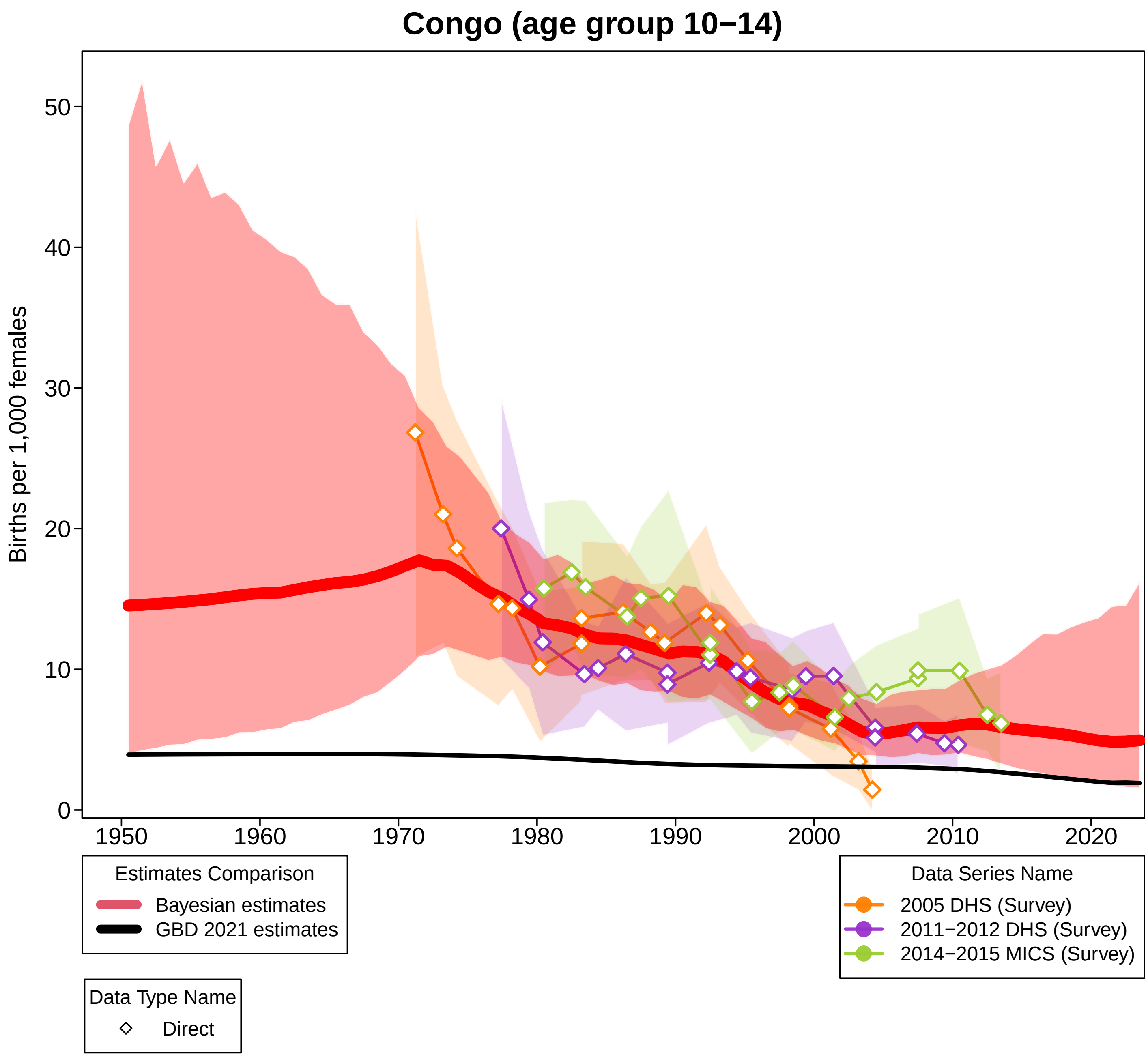
Congo (age group 10−14)
Births per 1,000 females
0
10
20
30
40
50
1950
1960
1970
1980
1990
2000
2010
2020
Estimates Comparison
Bayesian estimates
GBD 2021 estimates
Data Series Name
2005 DHS (Survey)
2011−2012 DHS (Survey)
2014−2015 MICS (Survey)
Data Type Name
Direct

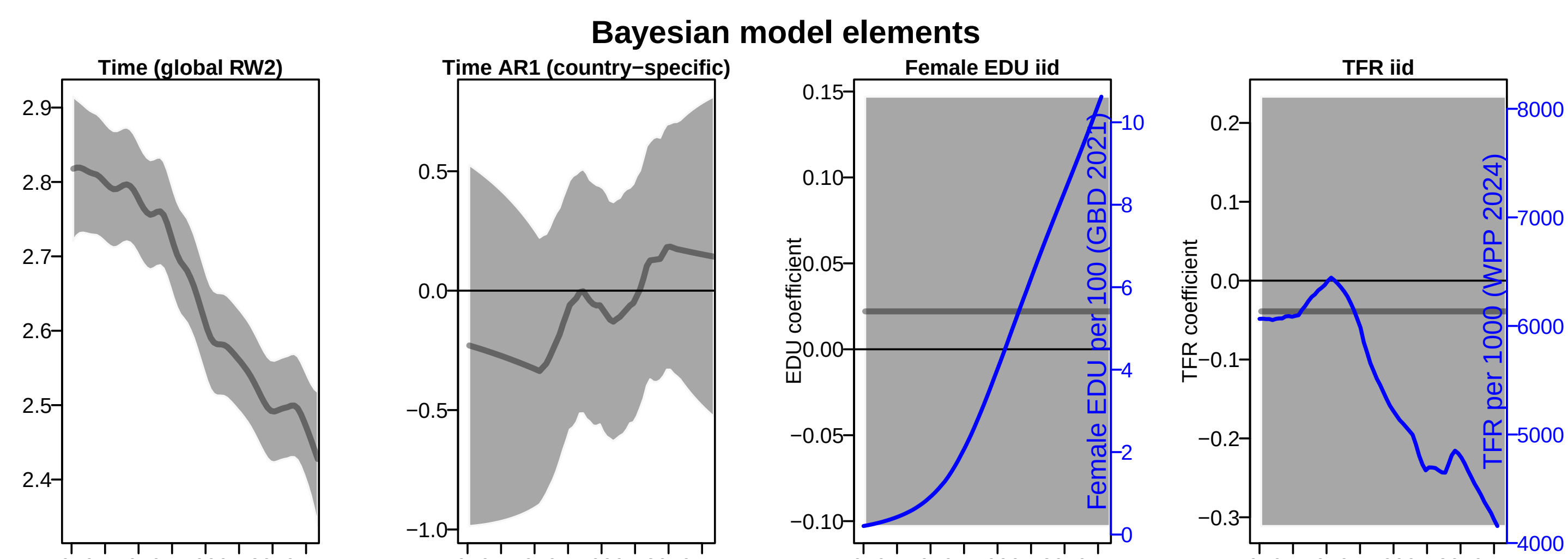
Bayesian model elements
Time (global RW2)
Time AR1 (country−specific)
Female EDU iid
TFR iid
EDU coefficient
Female EDU per 100 (GBD 2021)
TFR coefficient
TFR per 1000 (WPP 2024)
1950
1970
1990
2010

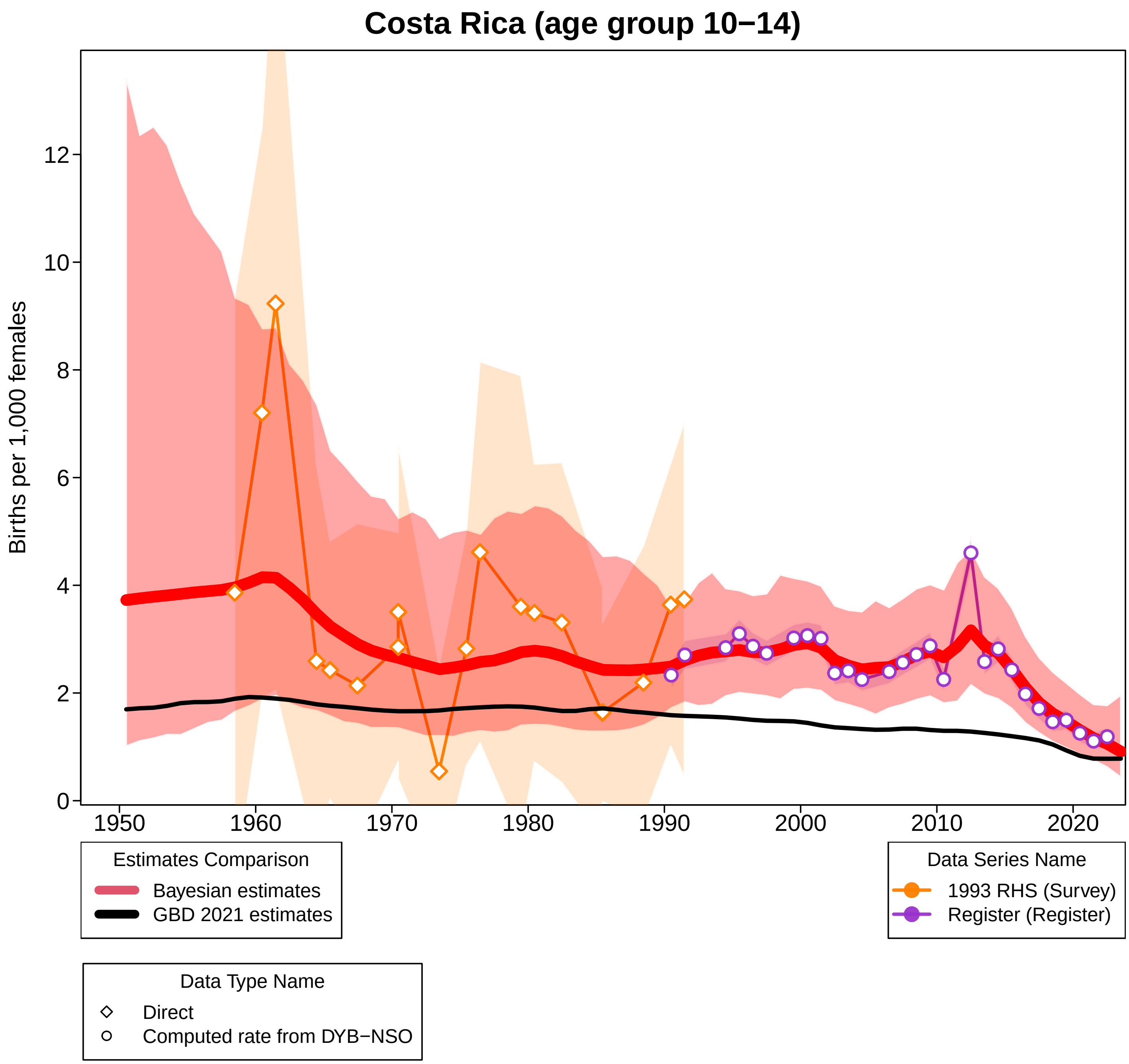
Costa Rica (age group 10−14)
Births per 1,000 females
0
2
4
6
8
10
12
1950
1960
1970
1980
1990
2000
2010
2020
Estimates Comparison
Bayesian estimates
GBD 2021 estimates
Data Series Name
1993 RHS (Survey)
Register (Register)
Data Type Name
Direct
Computed rate from DYB−NSO

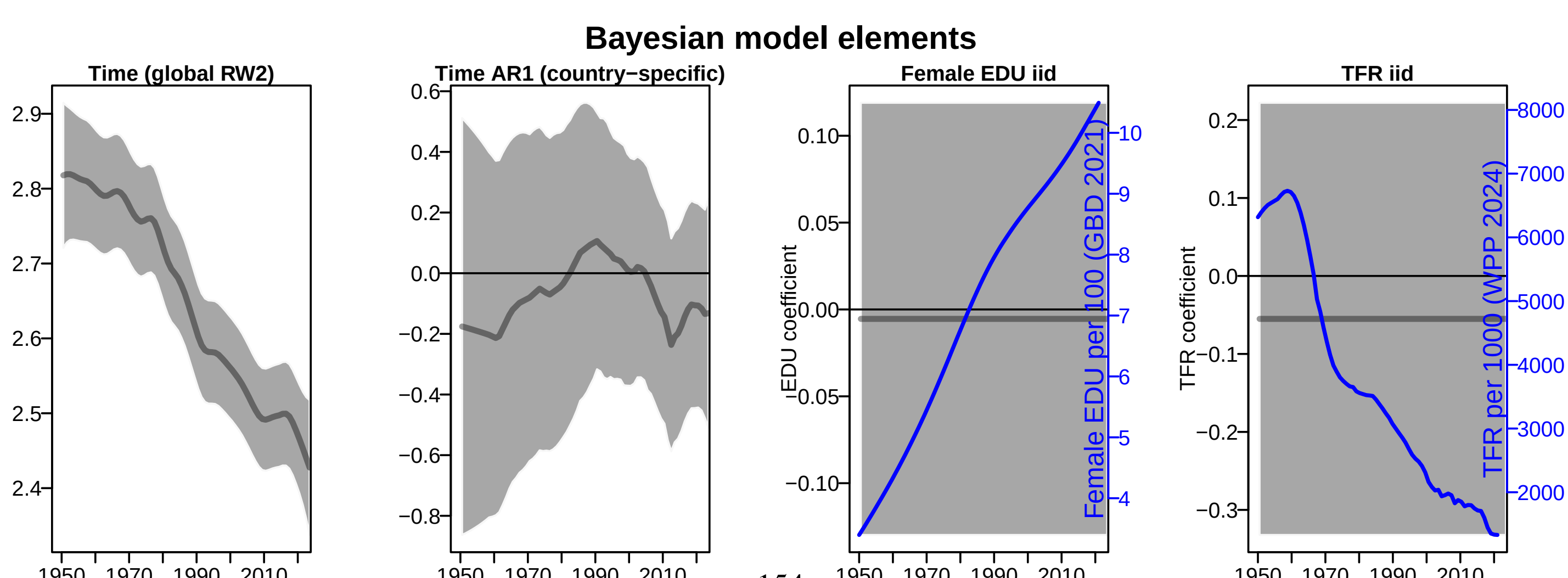
Bayesian model elements
Time (global RW2)
Time AR1 (country−specific)
Female EDU iid
TFR iid
EDU coefficient
Female EDU per 100 (GBD 2021)
TFR coefficient
TFR per 1000 (WPP 2024)
1950
1970
1990
2010

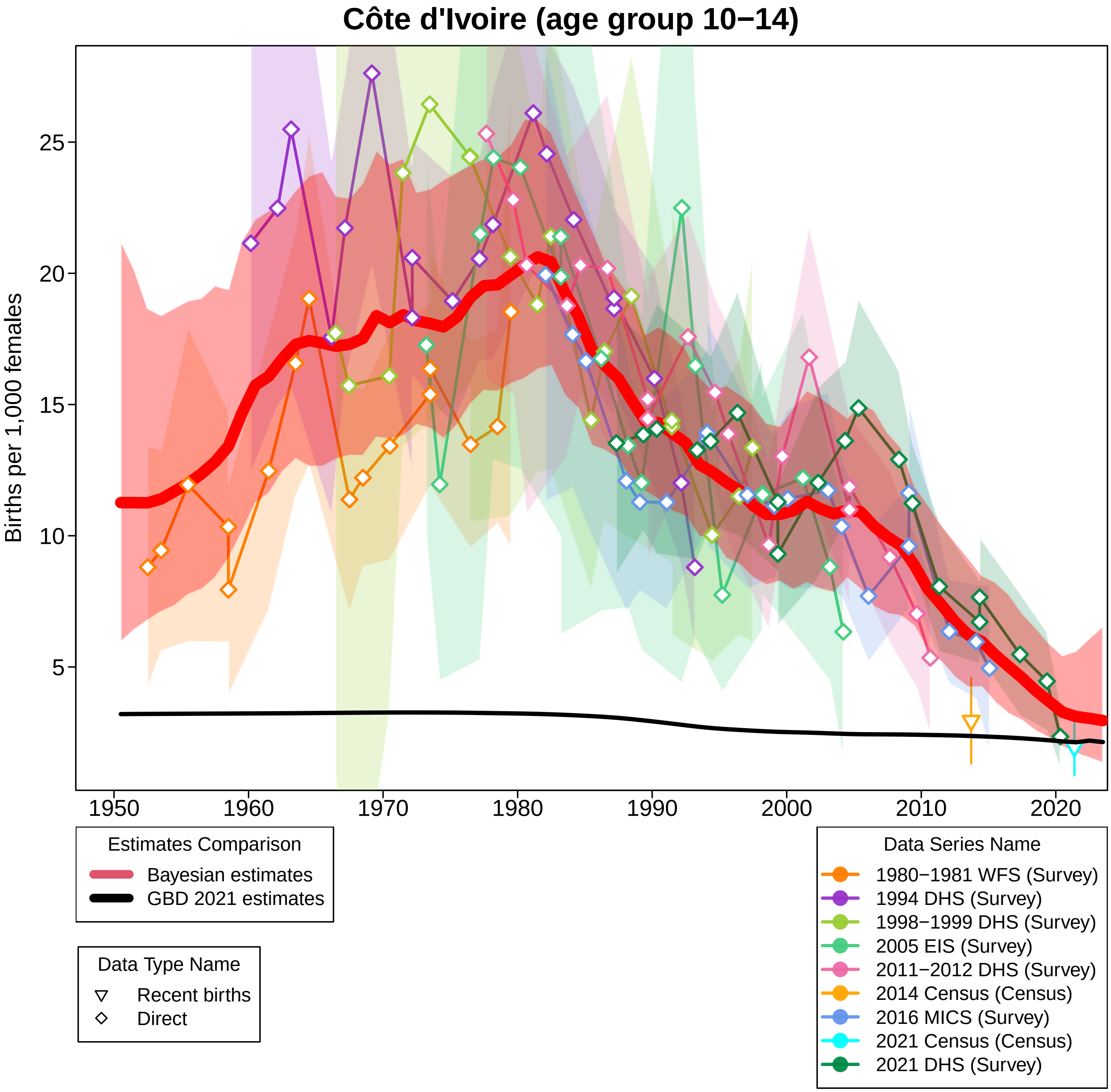
Côte d'Ivoire (age group 10−14)
Births per 1,000 females
25
20
15
10
5
1950
1960
1970
1980
1990
2000
2010
2020
Estimates Comparison
Bayesian estimates
GBD 2021 estimates
Data Type Name
Recent births
Direct
Data Series Name
1980−1981 WFS (Survey)
1994 DHS (Survey)
1998−1999 DHS (Survey)
2005 EIS (Survey)
2011−2012 DHS (Survey)
2014 Census (Census)
2016 MICS (Survey)
2021 Census (Census)
2021 DHS (Survey)

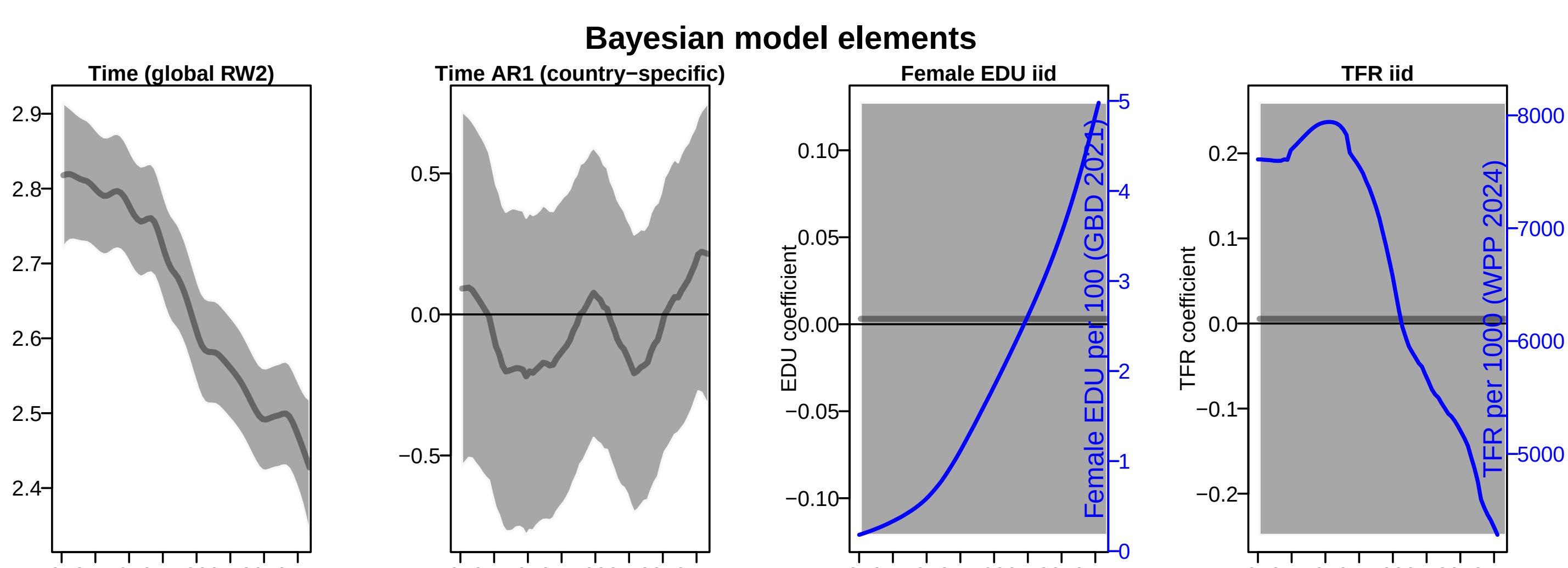
Bayesian model elements
Time (global RW2)
Time AR1 (country−specific)
Female EDU iid
TFR iid
EDU coefficient
Female EDU per 100 (GBD 2021)
TFR coefficient
TFR per 1000 (WPP 2024)
1950
1970
1990
2010

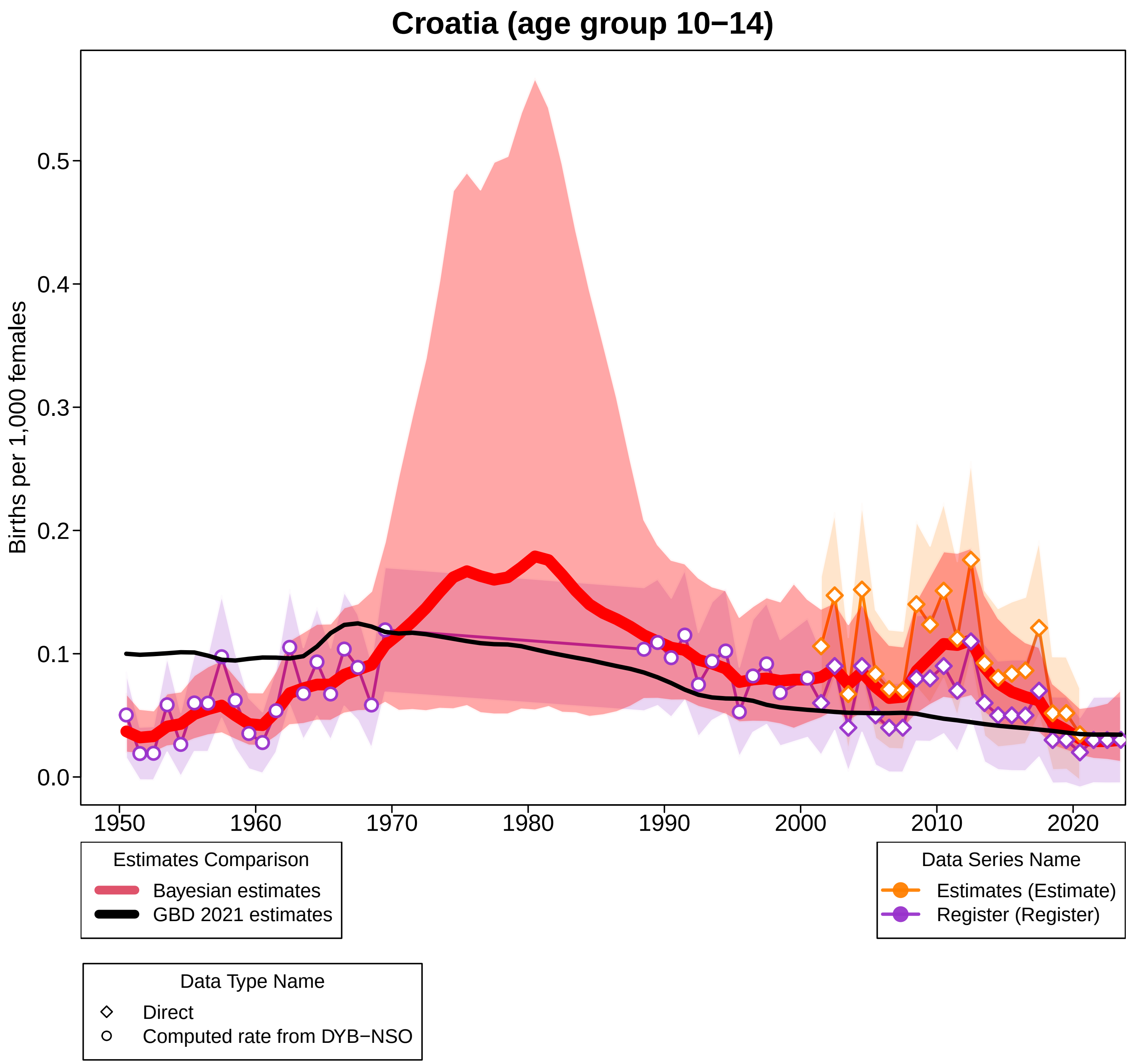
Croatia (age group 10−14)
Births per 1,000 females
0.0
0.1
0.2
0.3
0.4
0.5
1950
1960
1970
1980
1990
2000
2010
2020
Estimates Comparison
Bayesian estimates
GBD 2021 estimates
Data Series Name
Estimates (Estimate)
Register (Register)
Data Type Name
Direct
Computed rate from DYB−NSO

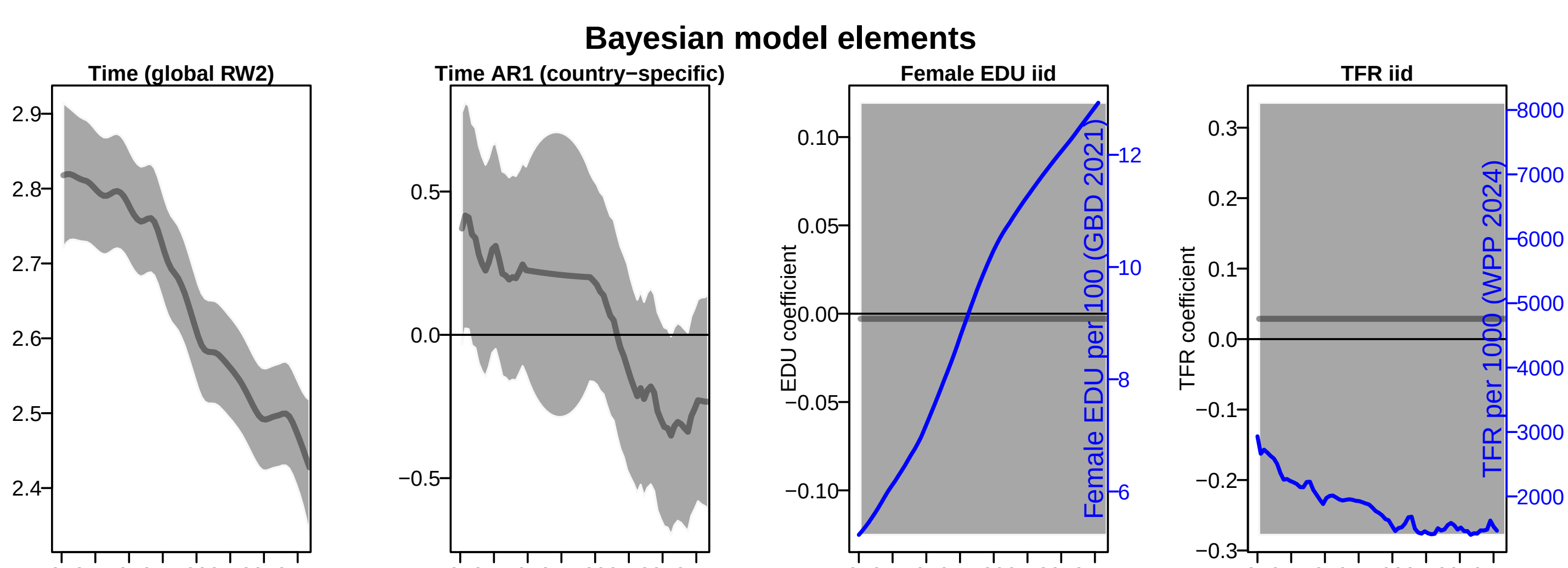
Bayesian model elements
Time (global RW2)
Time AR1 (country−specific)
Female EDU iid
TFR iid
EDU coefficient
Female EDU per 100 (GBD 2021)
TFR coefficient
TFR per 1000 (WPP 2024)
1950
1970
1990
2010

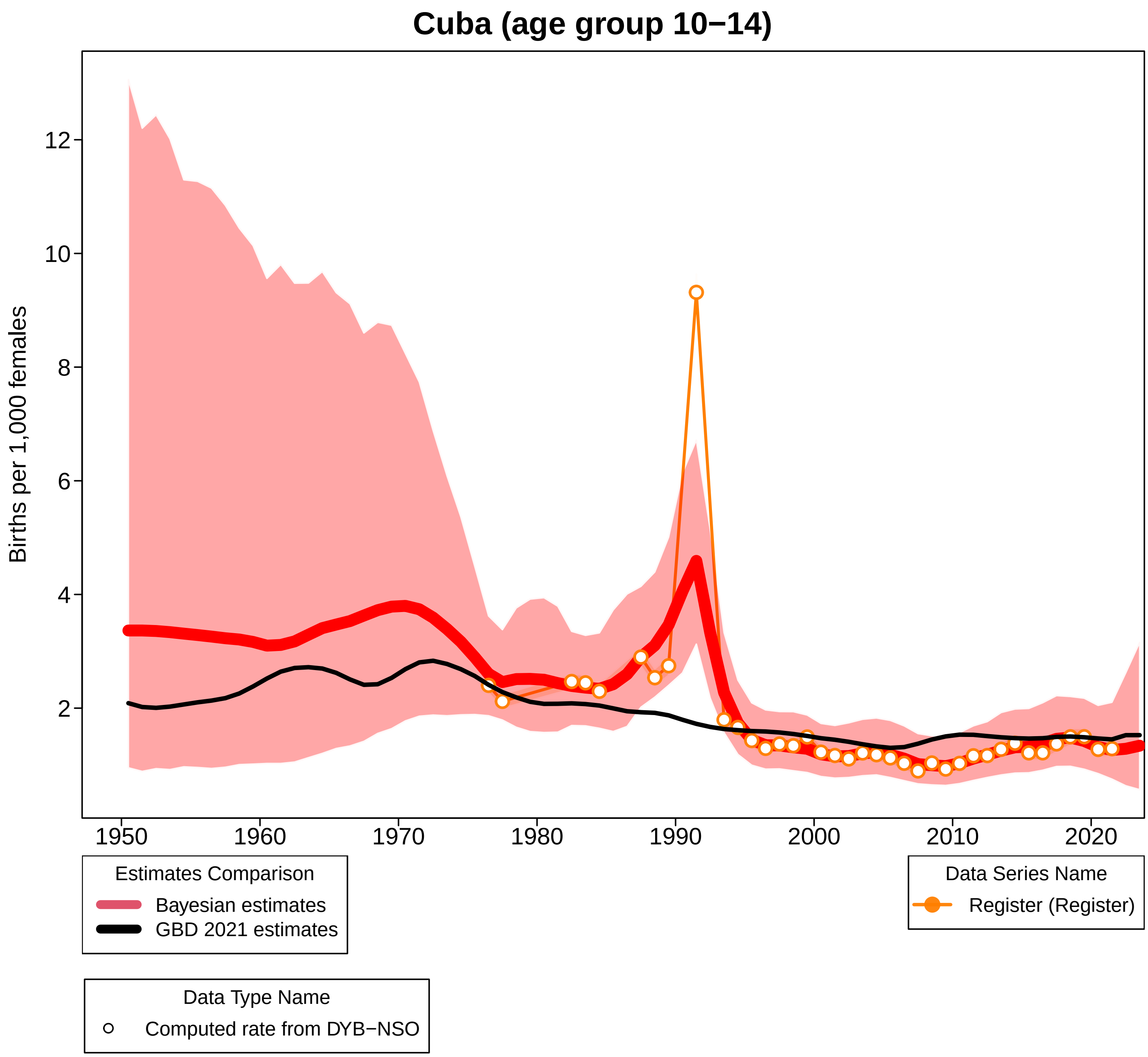
Cuba (age group 10−14)
Births per 1,000 females
Estimates Comparison
Bayesian estimates
GBD 2021 estimates
Data Series Name
Register (Register)
Data Type Name
Computed rate from DYB−NSO

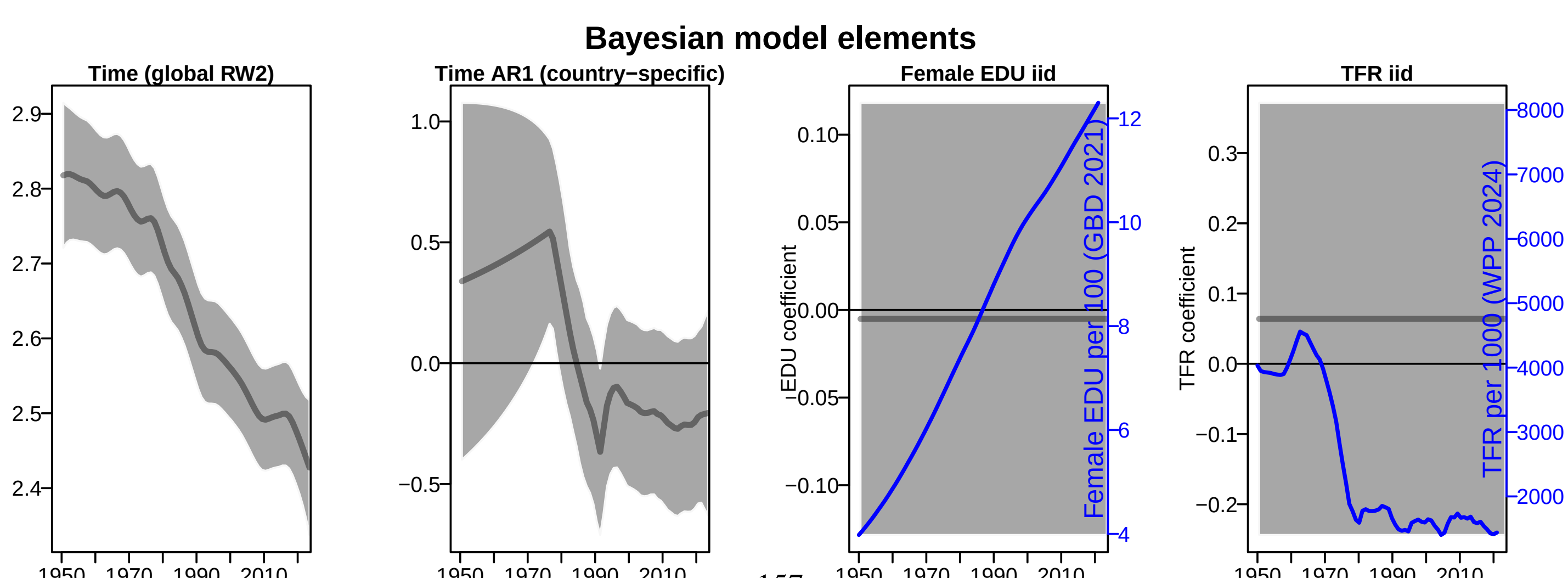
Bayesian model elements
Time (global RW2)
Time AR1 (country−specific)
Female EDU iid
EDU coefficient
Female EDU per 100 (GBD 2021)
TFR iid
TFR coefficient
TFR per 1000 (WPP 2024)

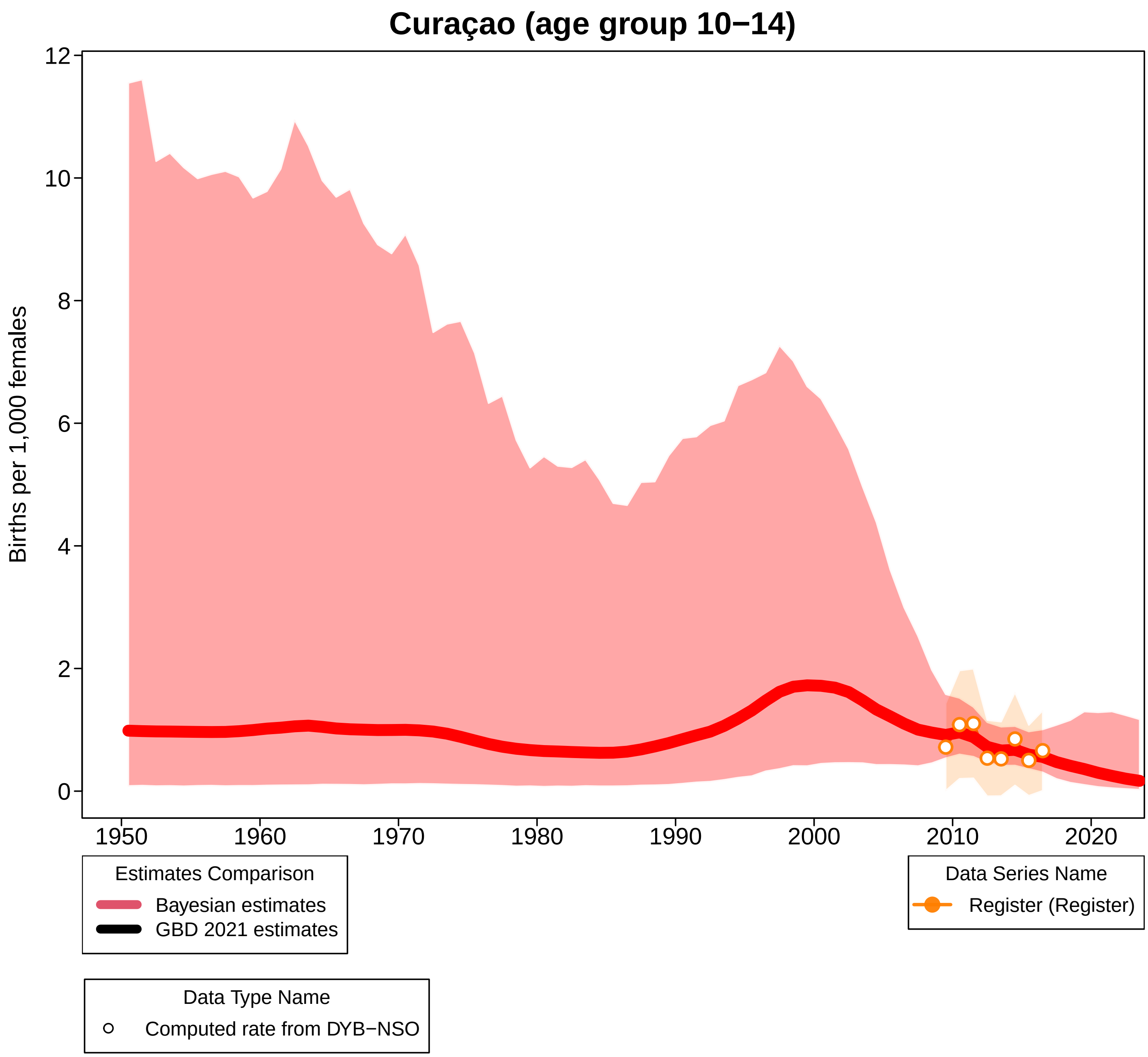

Curaçao (age group 10−14)
Births per 1,000 females
12
10
8
6
4
2
0
1950
1960
1970
1980
1990
2000
2010
2020
Estimates Comparison
Bayesian estimates
GBD 2021 estimates
Data Series Name
Register (Register)
Data Type Name
Computed rate from DYB−NSO

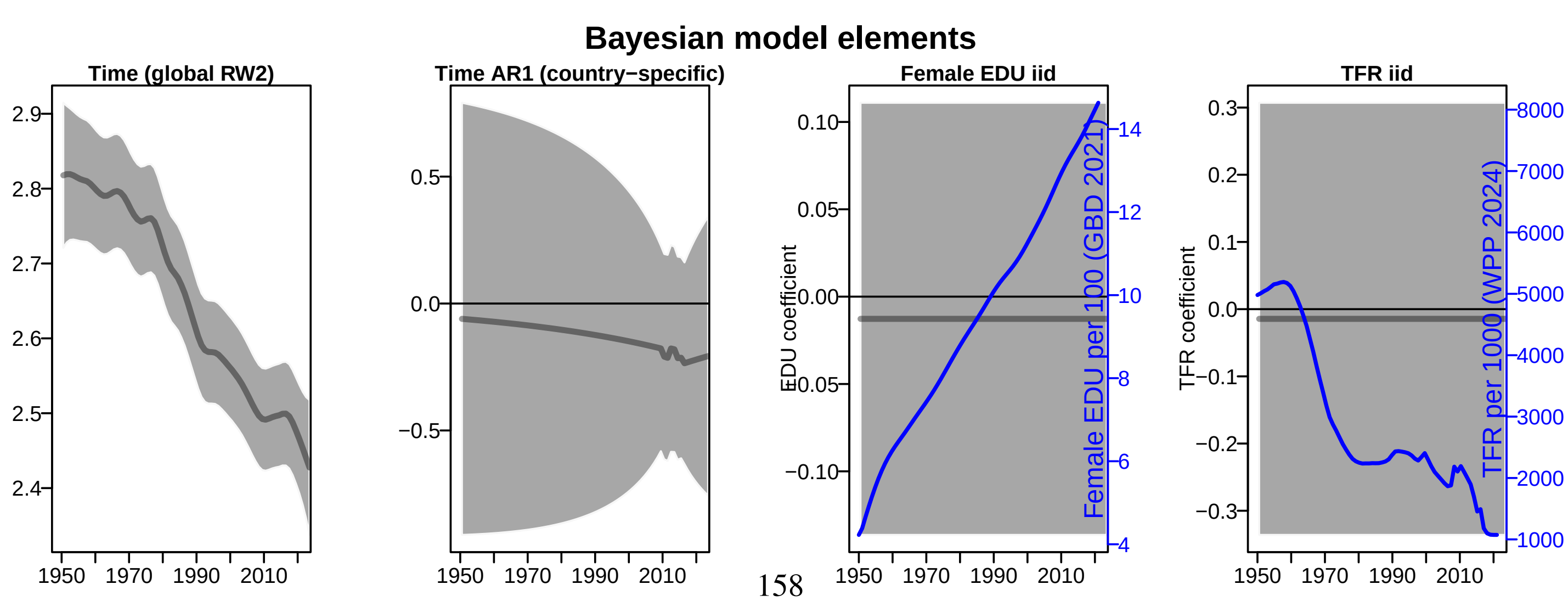

Bayesian model elements
Time (global RW2)
Time AR1 (country−specific)
Female EDU iid
TFR iid
EDU coefficient
Female EDU per 100 (GBD 2021)
TFR coefficient
TFR per 1000 (WPP 2024)
1950
1970
1990
2010

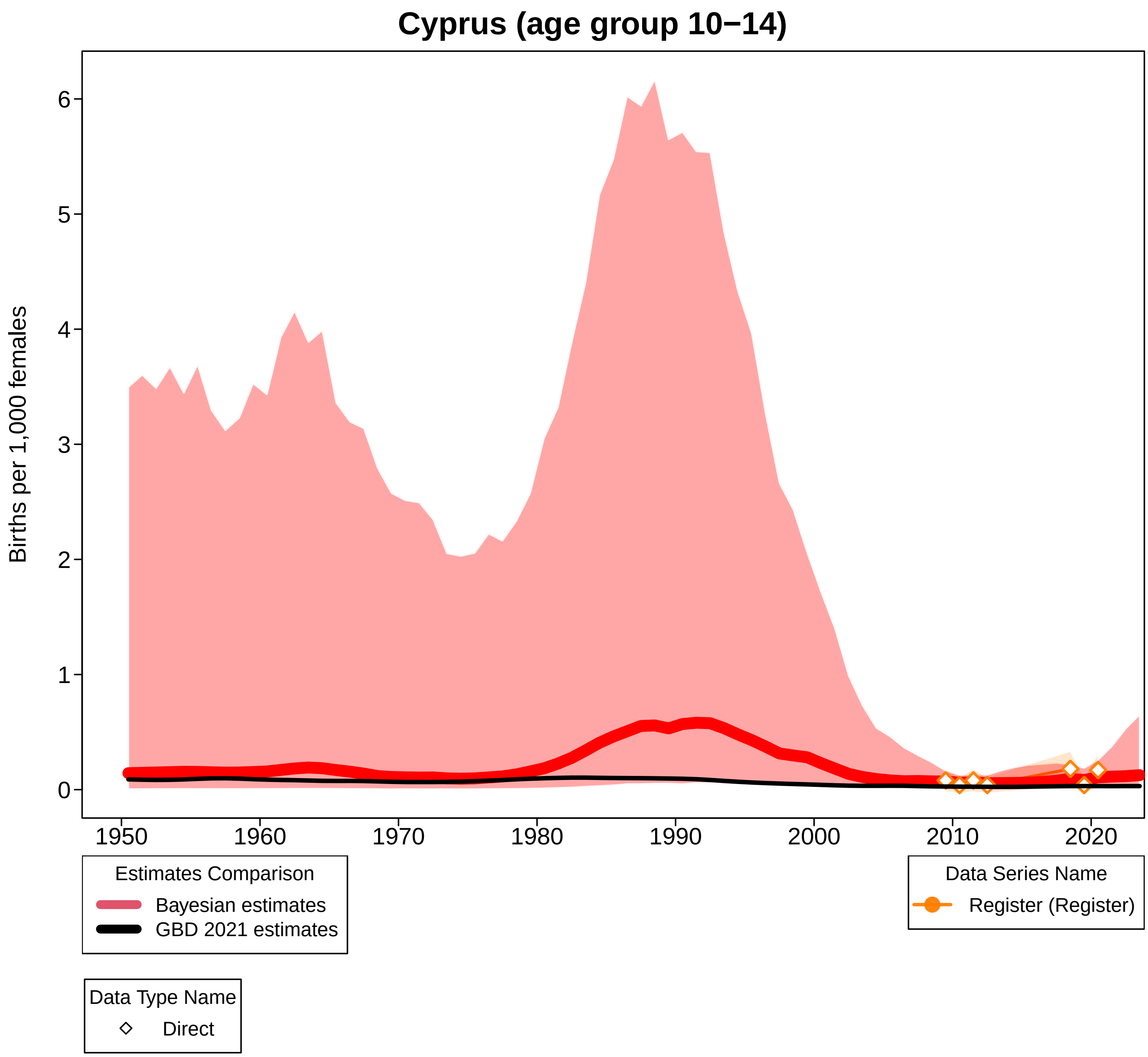

Cyprus (age group 10−14)
Births per 1,000 females
0
1
2
3
4
5
6
1950
1960
1970
1980
1990
2000
2010
2020
Estimates Comparison
Bayesian estimates
GBD 2021 estimates
Data Series Name
Register (Register)
Data Type Name
Direct

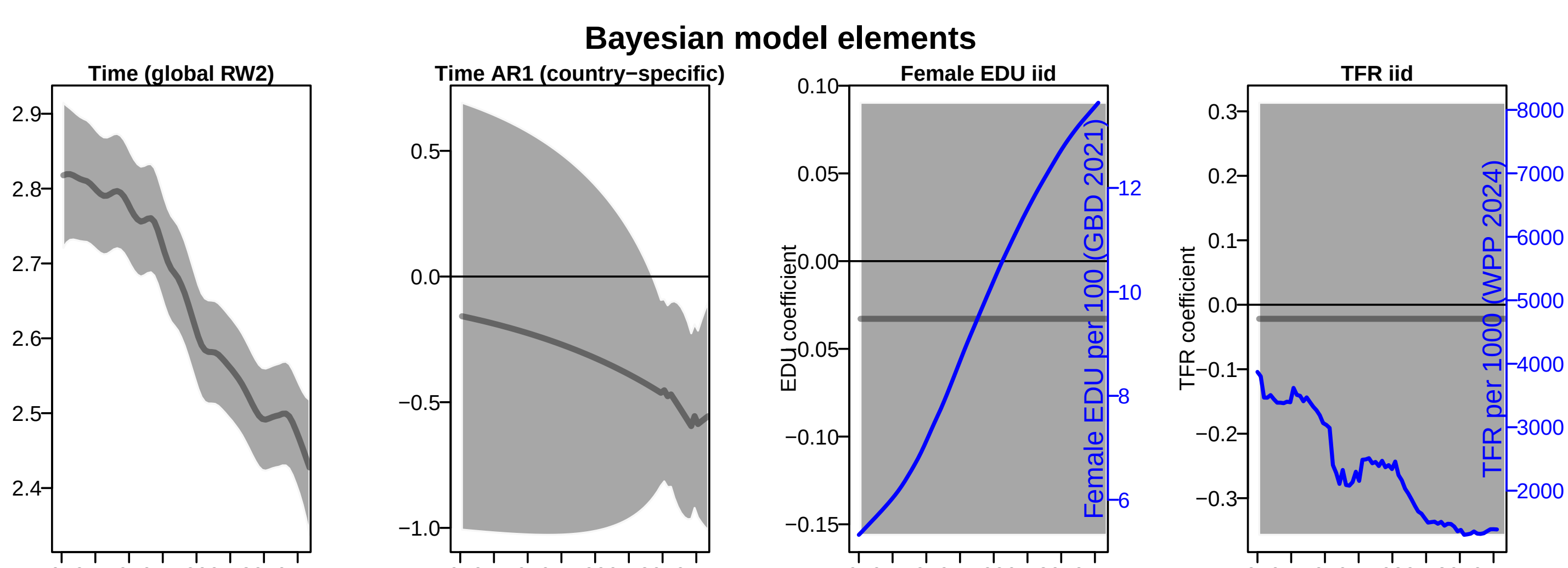

Bayesian model elements
Time (global RW2)
Time AR1 (country−specific)
Female EDU iid
TFR iid
EDU coefficient
Female EDU per 100 (GBD 2021)
TFR coefficient
TFR per 1000 (WPP 2024)
1950
1970
1990
2010

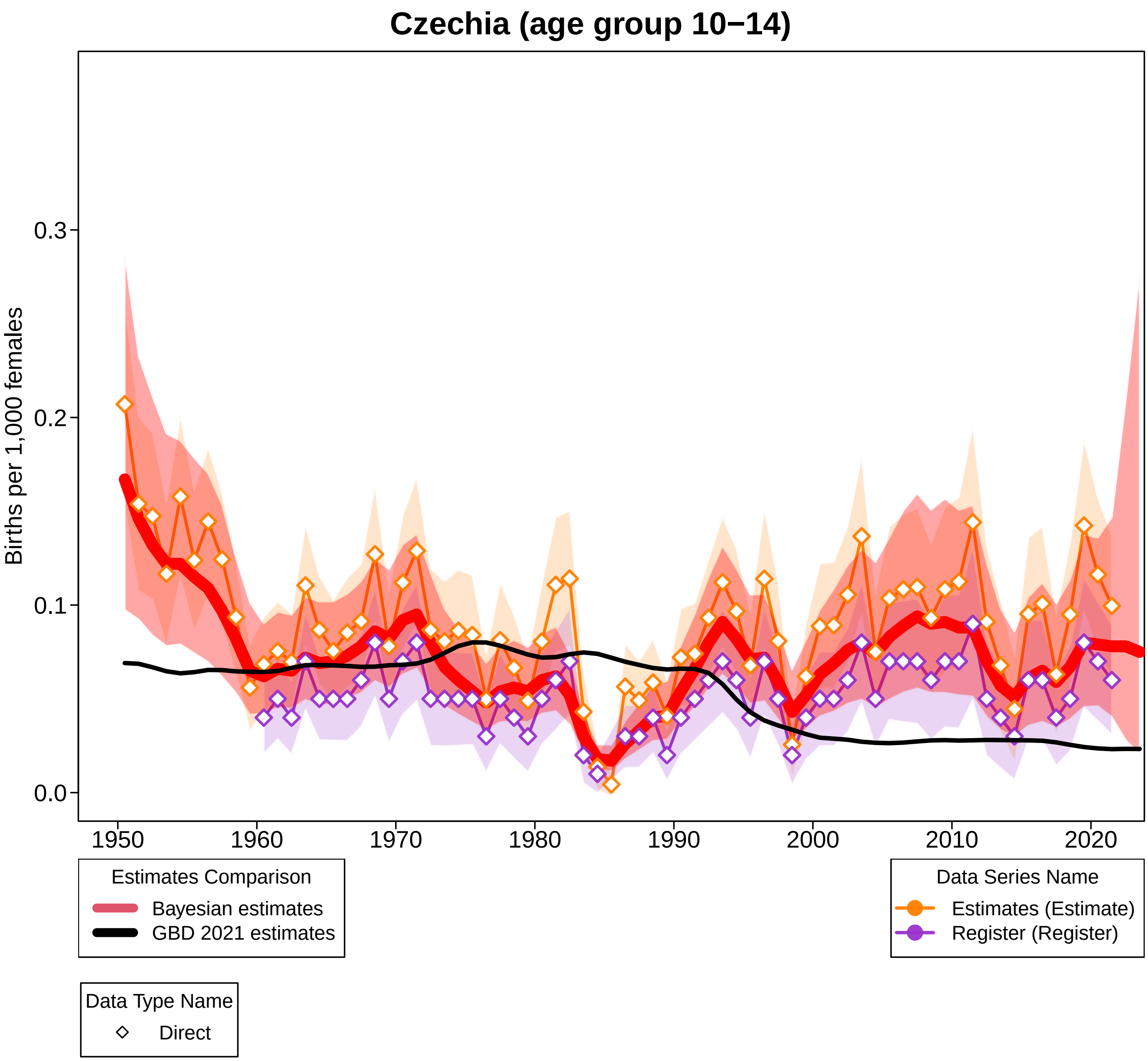

Czechia (age group 10−14)
Births per 1,000 females
0.0
0.1
0.2
0.3
1950
1960
1970
1980
1990
2000
2010
2020
Estimates Comparison
Bayesian estimates
GBD 2021 estimates
Data Series Name
Estimates (Estimate)
Register (Register)
Data Type Name
Direct

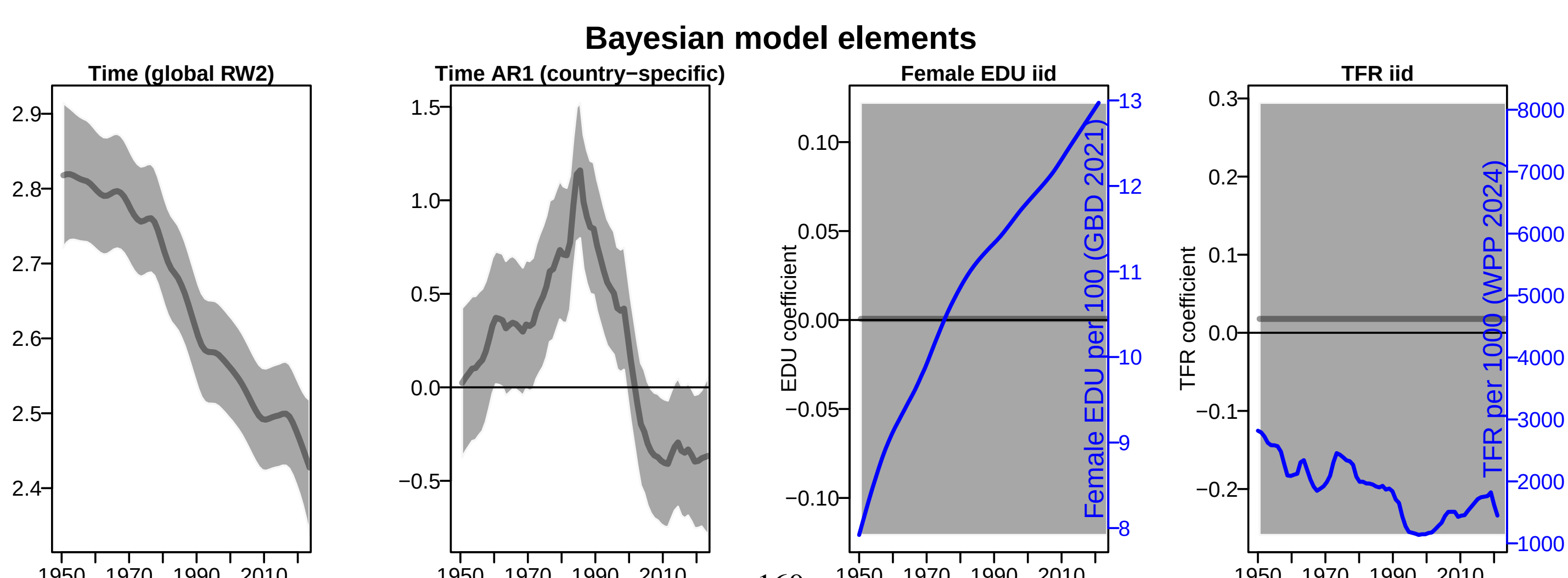

Bayesian model elements
Time (global RW2)
Time AR1 (country−specific)
Female EDU iid
TFR iid
EDU coefficient
Female EDU per 100 (GBD 2021)
TFR coefficient
TFR per 1000 (WPP 2024)
1950
1970
1990
2010

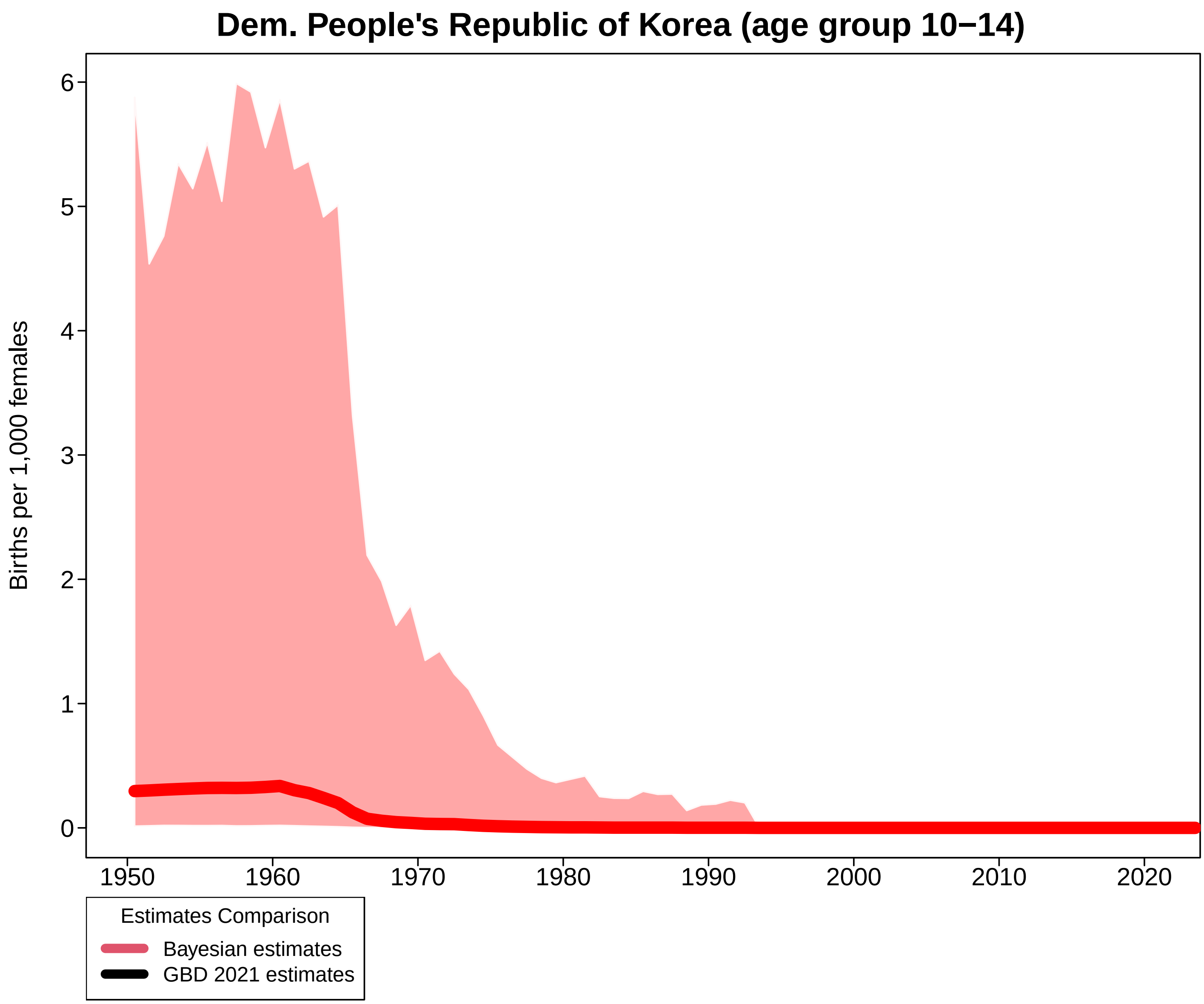
Dem. People's Republic of Korea (age group 10−14)
Births per 1,000 females
Estimates Comparison
Bayesian estimates
GBD 2021 estimates

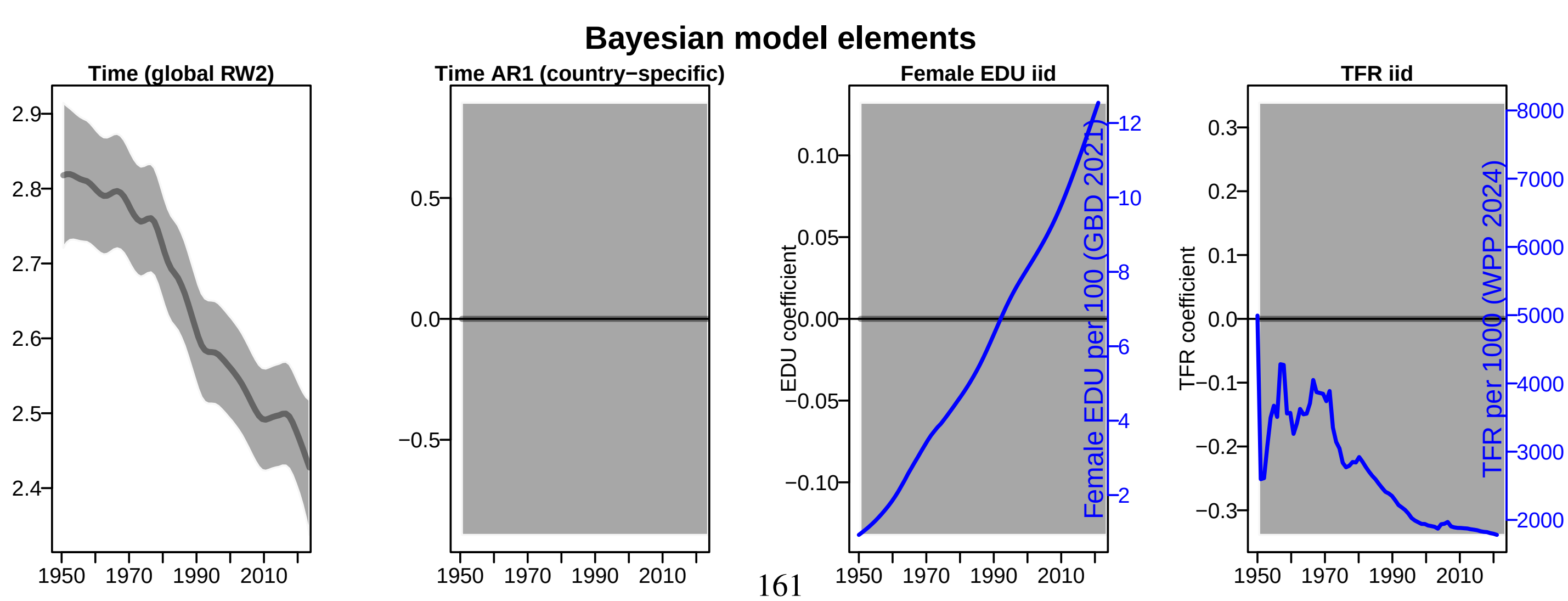
Bayesian model elements
Time (global RW2)
Time AR1 (country−specific)
Female EDU iid
TFR iid
EDU coefficient
Female EDU per 100 (GBD 2021)
TFR coefficient
TFR per 1000 (WPP 2024)

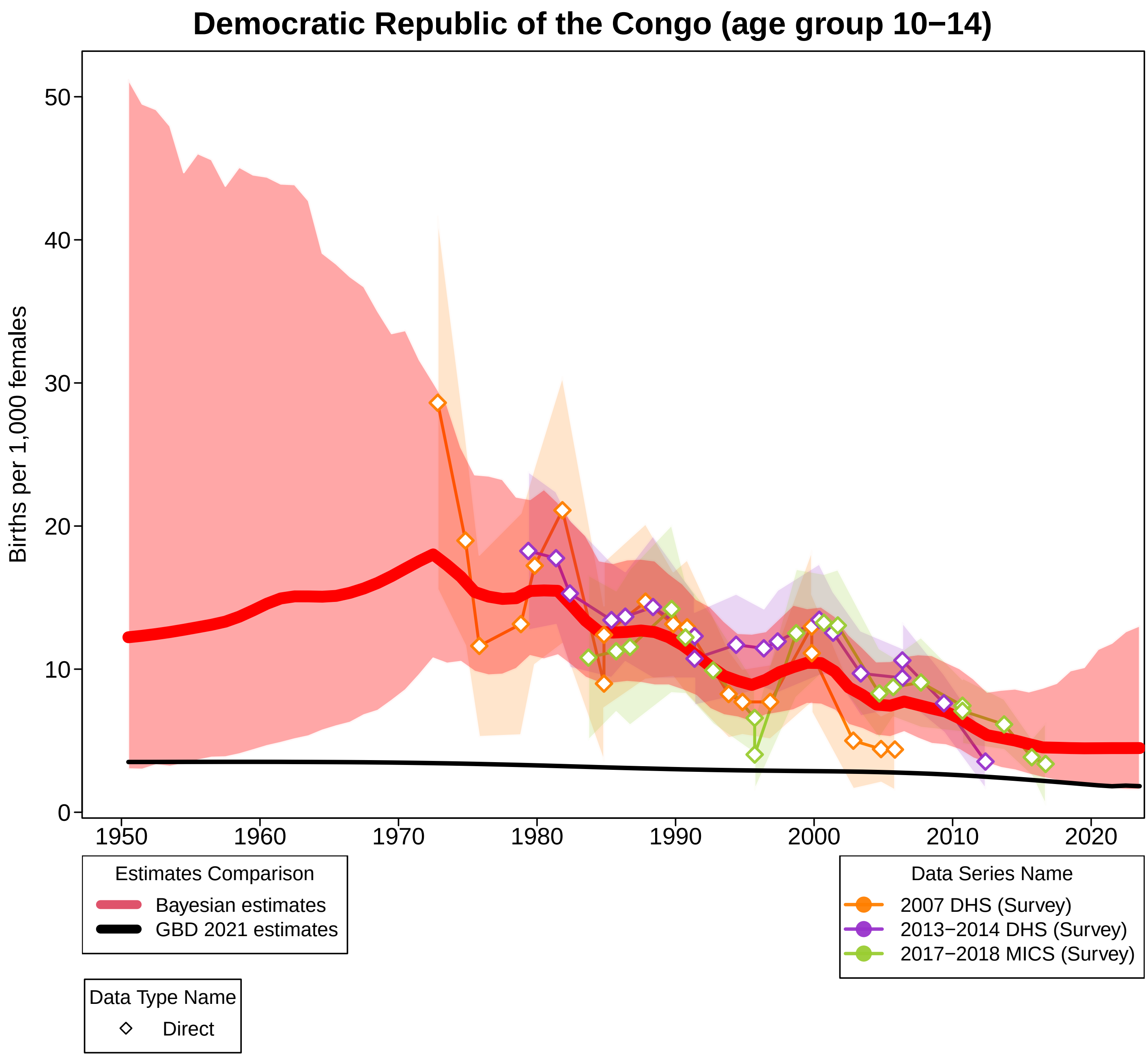
Democratic Republic of the Congo (age group 10−14)
Births per 1,000 females
0
10
20
30
40
50
1950
1960
1970
1980
1990
2000
2010
2020
Estimates Comparison
Bayesian estimates
GBD 2021 estimates
Data Series Name
2007 DHS (Survey)
2013−2014 DHS (Survey)
2017−2018 MICS (Survey)
Data Type Name
Direct

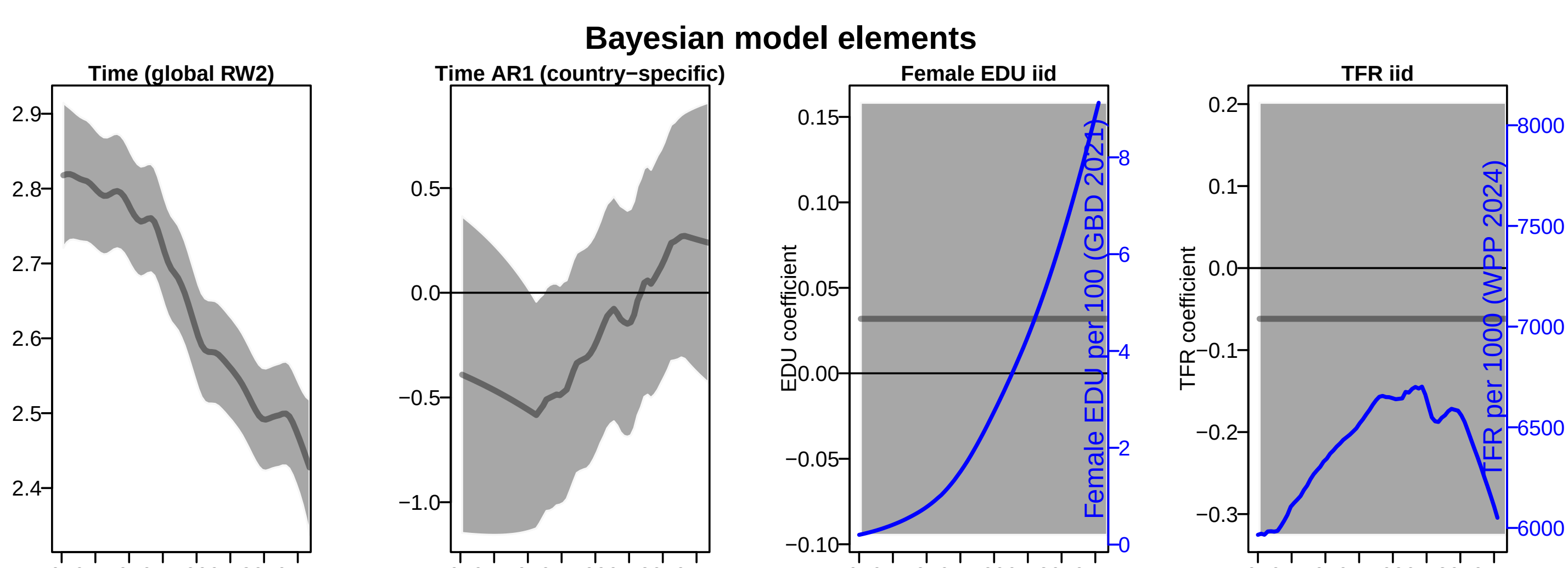
Bayesian model elements
Time (global RW2)
Time AR1 (country−specific)
Female EDU iid
TFR iid
EDU coefficient
Female EDU per 100 (GBD 2021)
TFR coefficient
TFR per 1000 (WPP 2024)
1950
1970
1990
2010

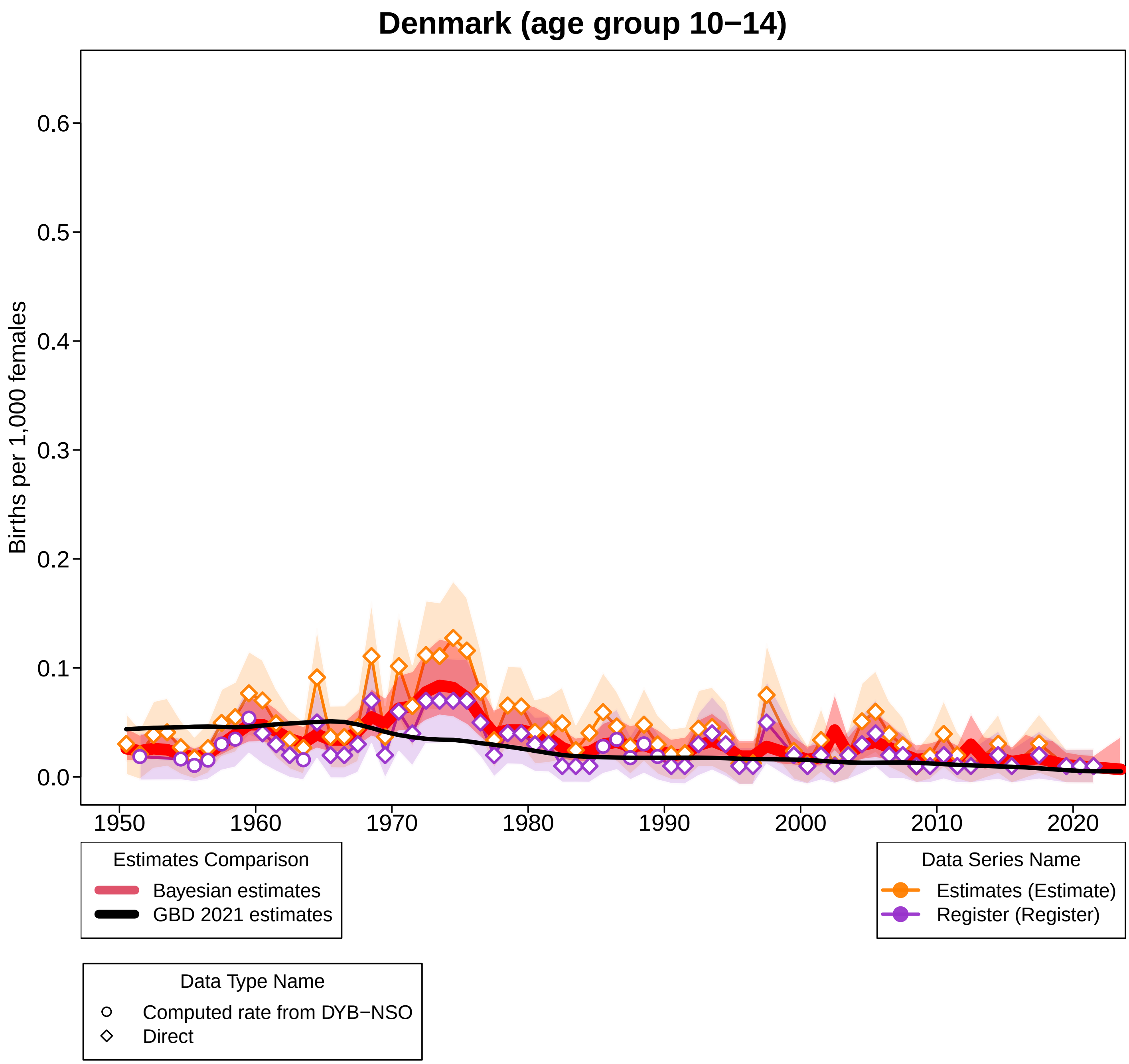

Denmark (age group 10−14)
Births per 1,000 females
0.0
0.1
0.2
0.3
0.4
0.5
0.6
1950
1960
1970
1980
1990
2000
2010
2020
Estimates Comparison
Bayesian estimates
GBD 2021 estimates
Data Series Name
Estimates (Estimate)
Register (Register)
Data Type Name
Computed rate from DYB−NSO
Direct

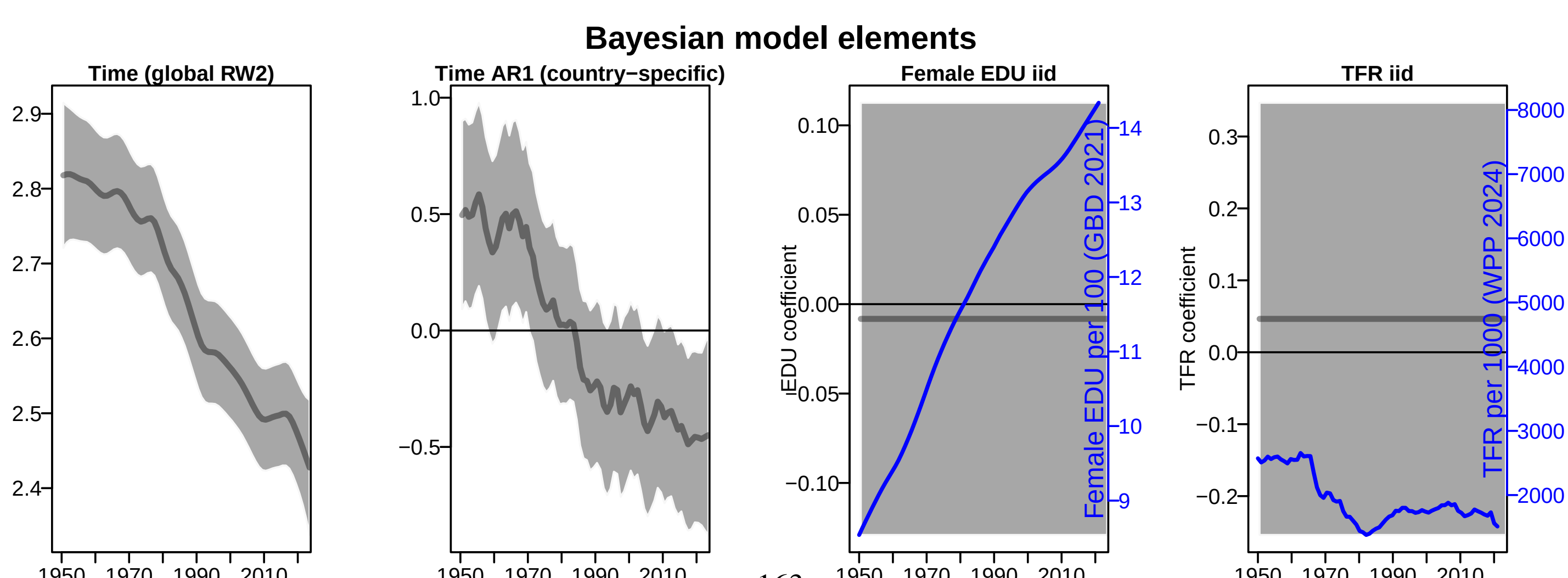

Bayesian model elements
Time (global RW2)
Time AR1 (country−specific)
Female EDU iid
TFR iid
EDU coefficient
Female EDU per 100 (GBD 2021)
TFR coefficient
TFR per 1000 (WPP 2024)
1950
1970
1990
2010

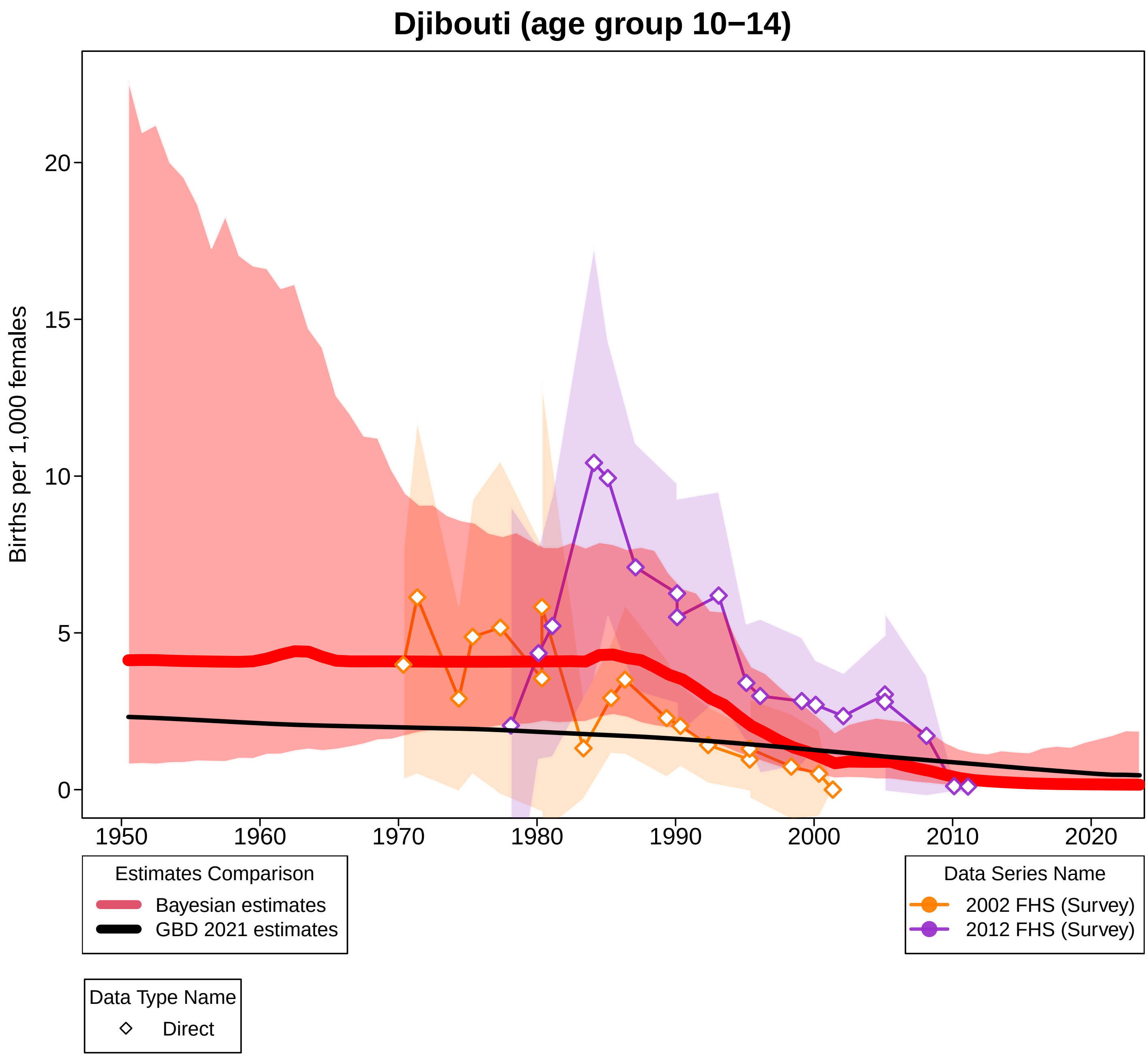

Djibouti (age group 10−14)
Births per 1,000 females
0
5
10
15
20
1950
1960
1970
1980
1990
2000
2010
2020
Estimates Comparison
Bayesian estimates
GBD 2021 estimates
Data Series Name
2002 FHS (Survey)
2012 FHS (Survey)
Data Type Name
Direct

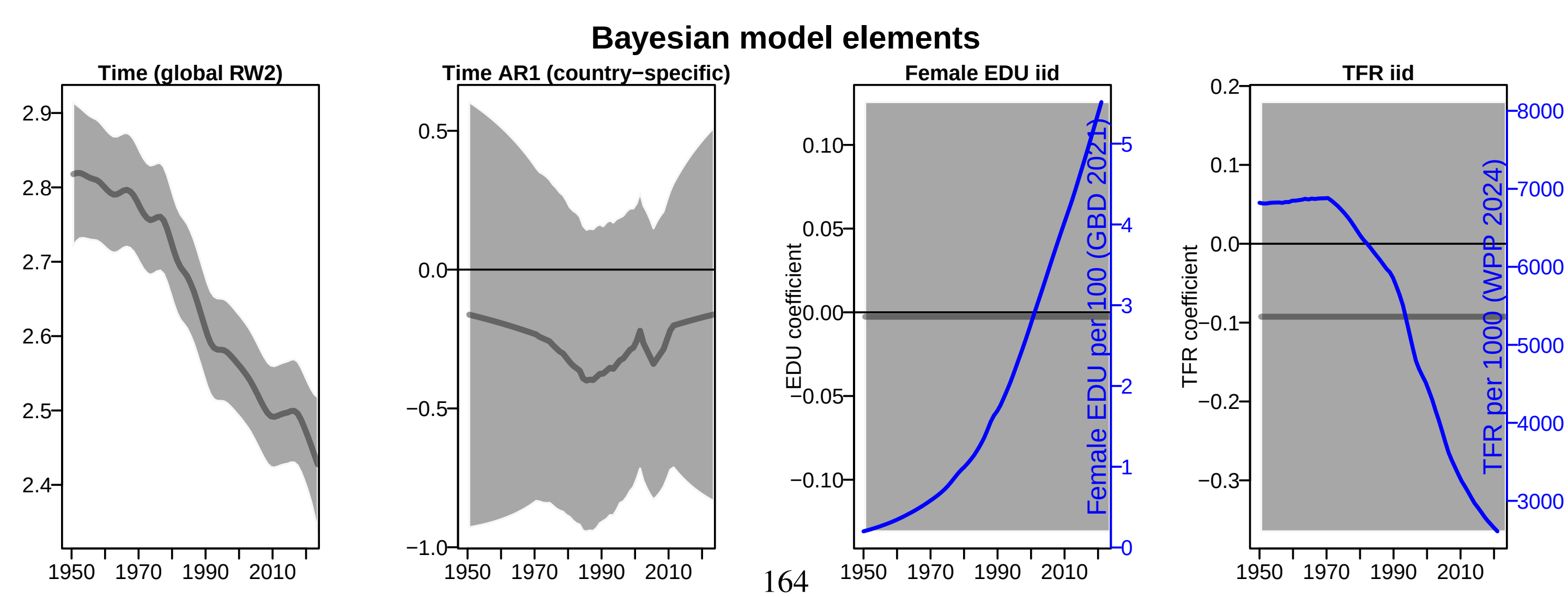

Bayesian model elements
Time (global RW2)
Time AR1 (country−specific)
Female EDU iid
TFR iid
EDU coefficient
Female EDU per 100 (GBD 2021)
TFR coefficient
TFR per 1000 (WPP 2024)
1950
1970
1990
2010

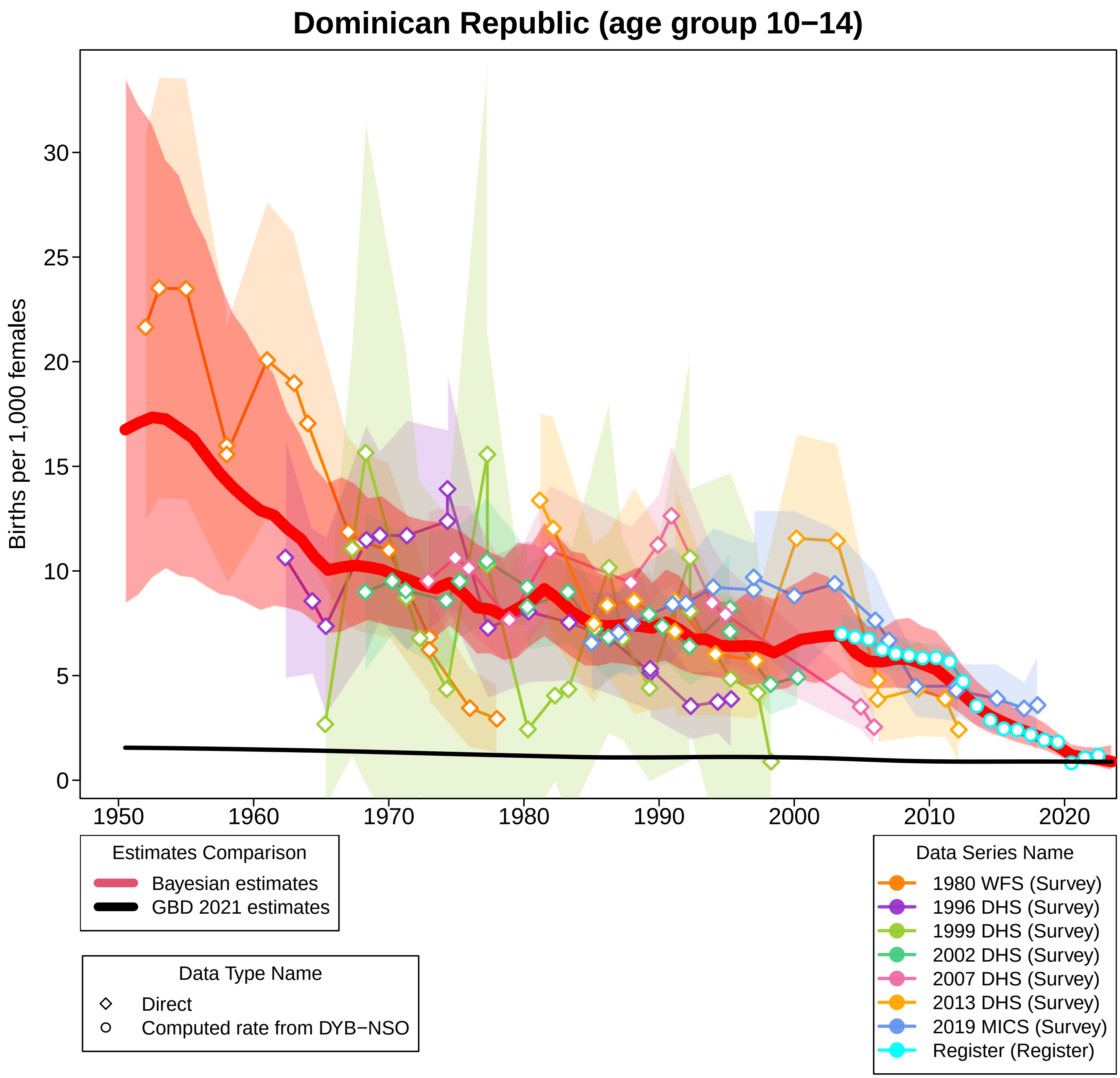
Dominican Republic (age group 10−14)
Births per 1,000 females
0
5
10
15
20
25
30
1950
1960
1970
1980
1990
2000
2010
2020
Estimates Comparison
Bayesian estimates
GBD 2021 estimates
Data Type Name
Direct
Computed rate from DYB−NSO
Data Series Name
1980 WFS (Survey)
1996 DHS (Survey)
1999 DHS (Survey)
2002 DHS (Survey)
2007 DHS (Survey)
2013 DHS (Survey)
2019 MICS (Survey)
Register (Register)

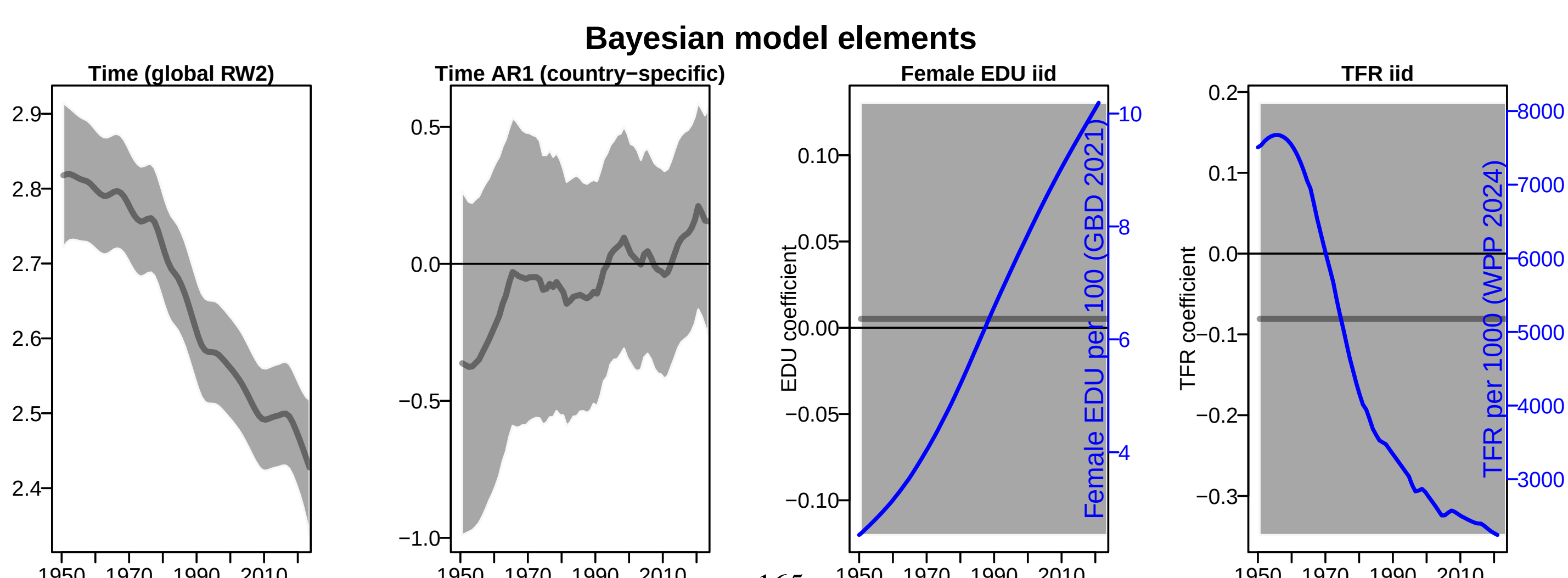
Bayesian model elements
Time (global RW2)
Time AR1 (country−specific)
Female EDU iid
TFR iid
EDU coefficient
Female EDU per 100 (GBD 2021)
TFR coefficient
TFR per 1000 (WPP 2024)
1950
1970
1990
2010

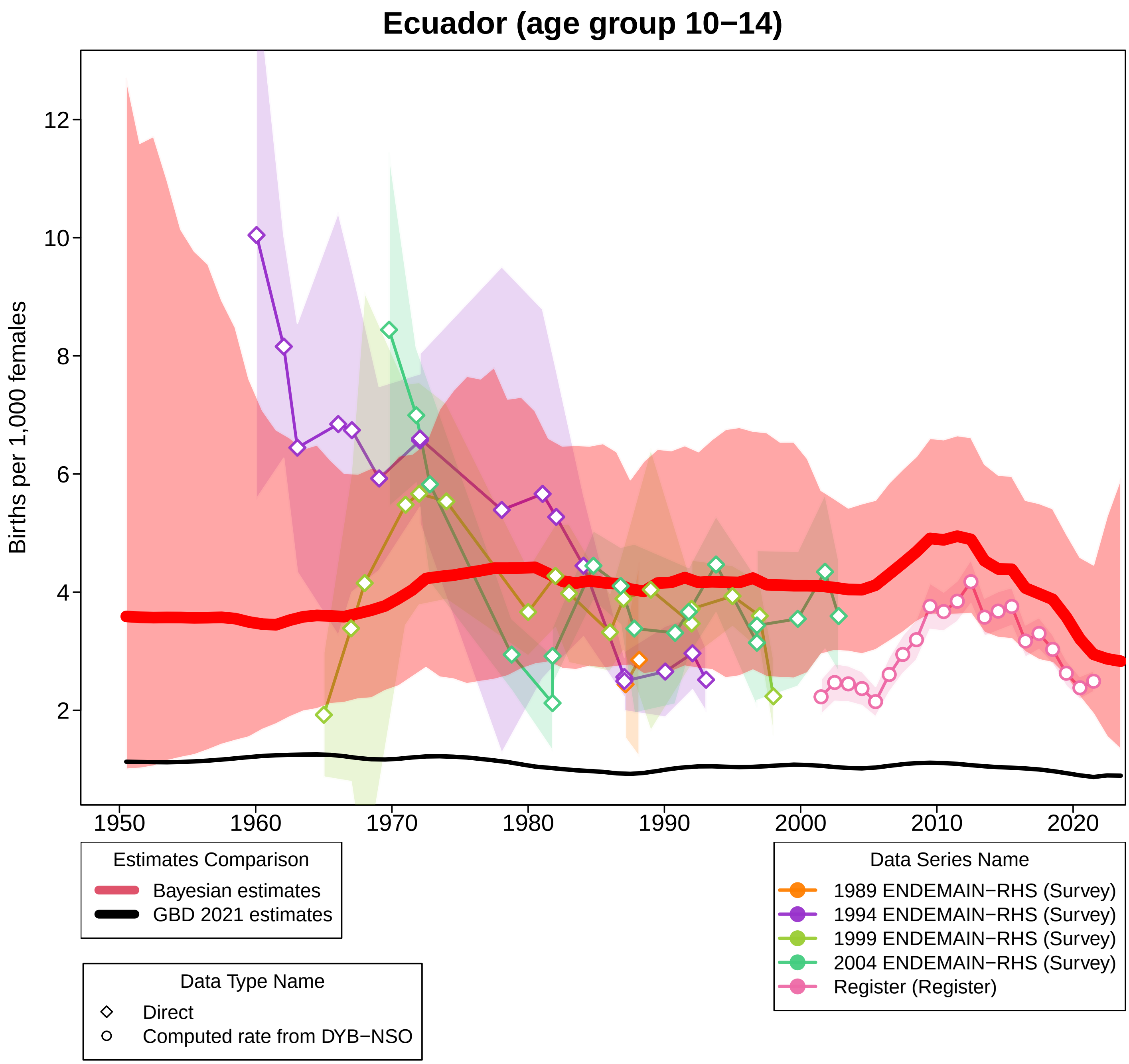
Ecuador (age group 10−14)
Births per 1,000 females
2
4
6
8
10
12
1950
1960
1970
1980
1990
2000
2010
2020
Estimates Comparison
Bayesian estimates
GBD 2021 estimates
Data Series Name
1989 ENDEMAIN−RHS (Survey)
1994 ENDEMAIN−RHS (Survey)
1999 ENDEMAIN−RHS (Survey)
2004 ENDEMAIN−RHS (Survey)
Register (Register)
Data Type Name
Direct
Computed rate from DYB−NSO

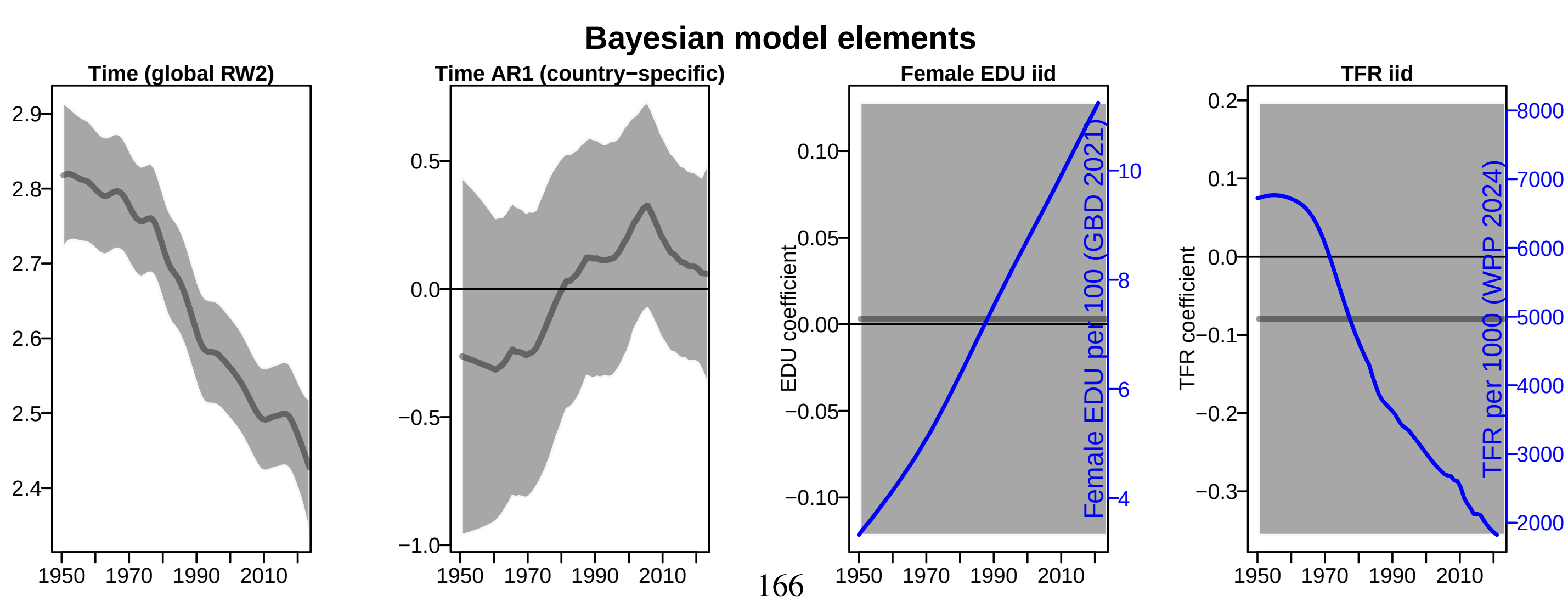
Bayesian model elements
Time (global RW2)
Time AR1 (country−specific)
Female EDU iid
TFR iid
EDU coefficient
Female EDU per 100 (GBD 2021)
TFR coefficient
TFR per 1000 (WPP 2024)
1950
1970
1990
2010

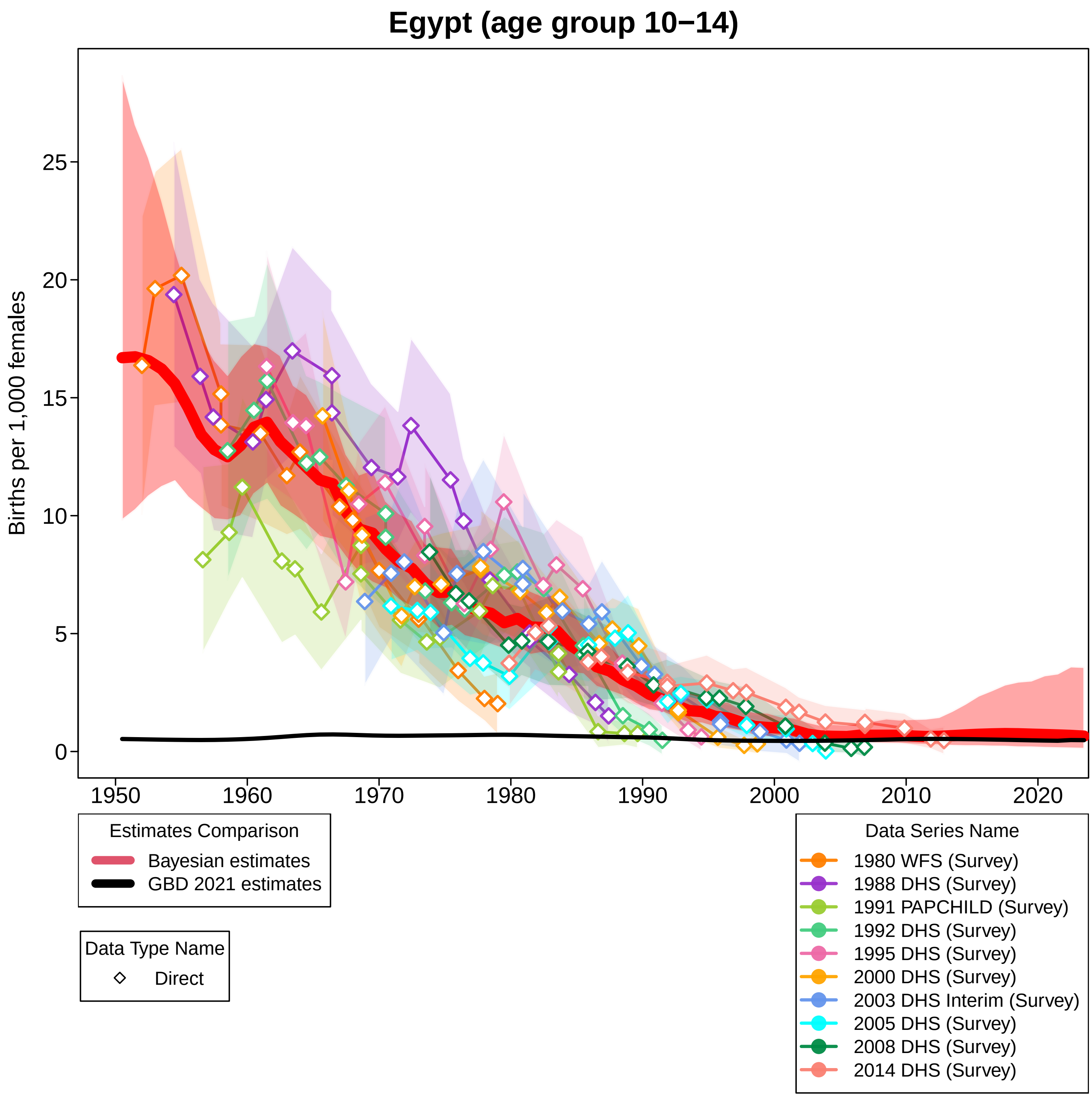
Egypt (age group 10−14)
Births per 1,000 females
0
5
10
15
20
25
1950
1960
1970
1980
1990
2000
2010
2020
Estimates Comparison
Bayesian estimates
GBD 2021 estimates
Data Type Name
Direct
Data Series Name
1980 WFS (Survey)
1988 DHS (Survey)
1991 PAPCHILD (Survey)
1992 DHS (Survey)
1995 DHS (Survey)
2000 DHS (Survey)
2003 DHS Interim (Survey)
2005 DHS (Survey)
2008 DHS (Survey)
2014 DHS (Survey)

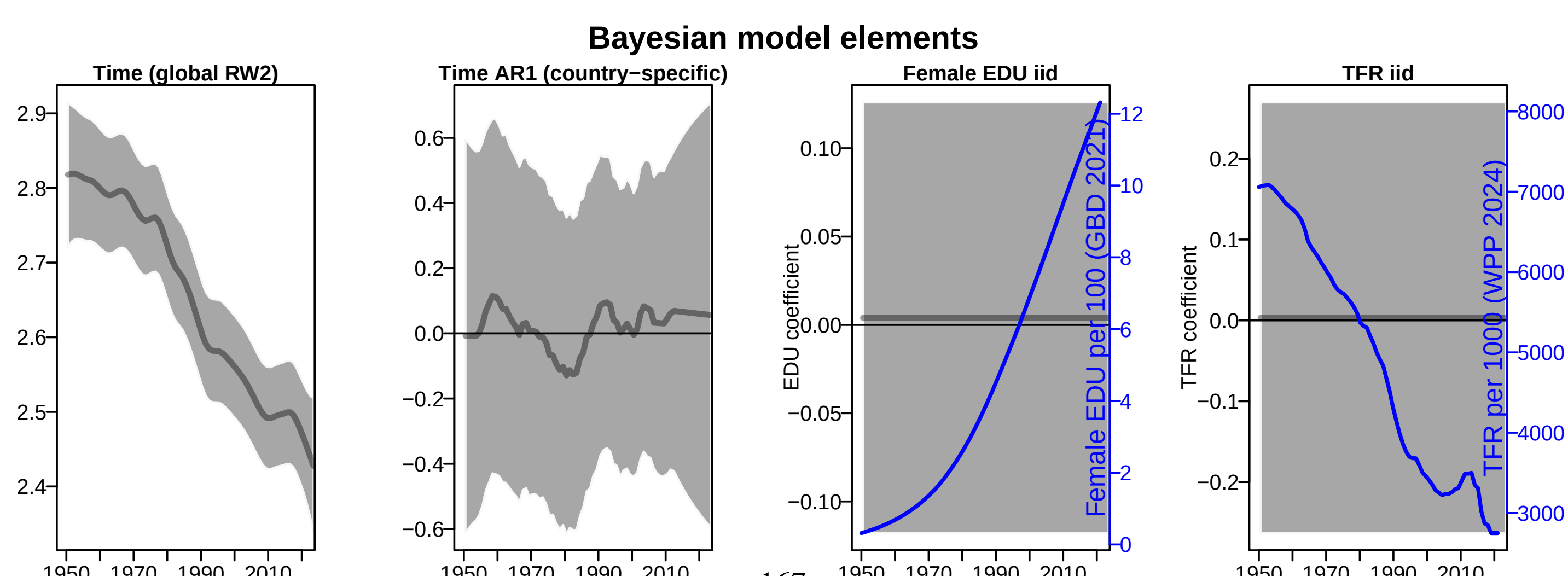
Bayesian model elements
Time (global RW2)
Time AR1 (country−specific)
Female EDU iid
TFR iid
EDU coefficient
Female EDU per 100 (GBD 2021)
TFR coefficient
TFR per 1000 (WPP 2024)
1950
1970
1990
2010

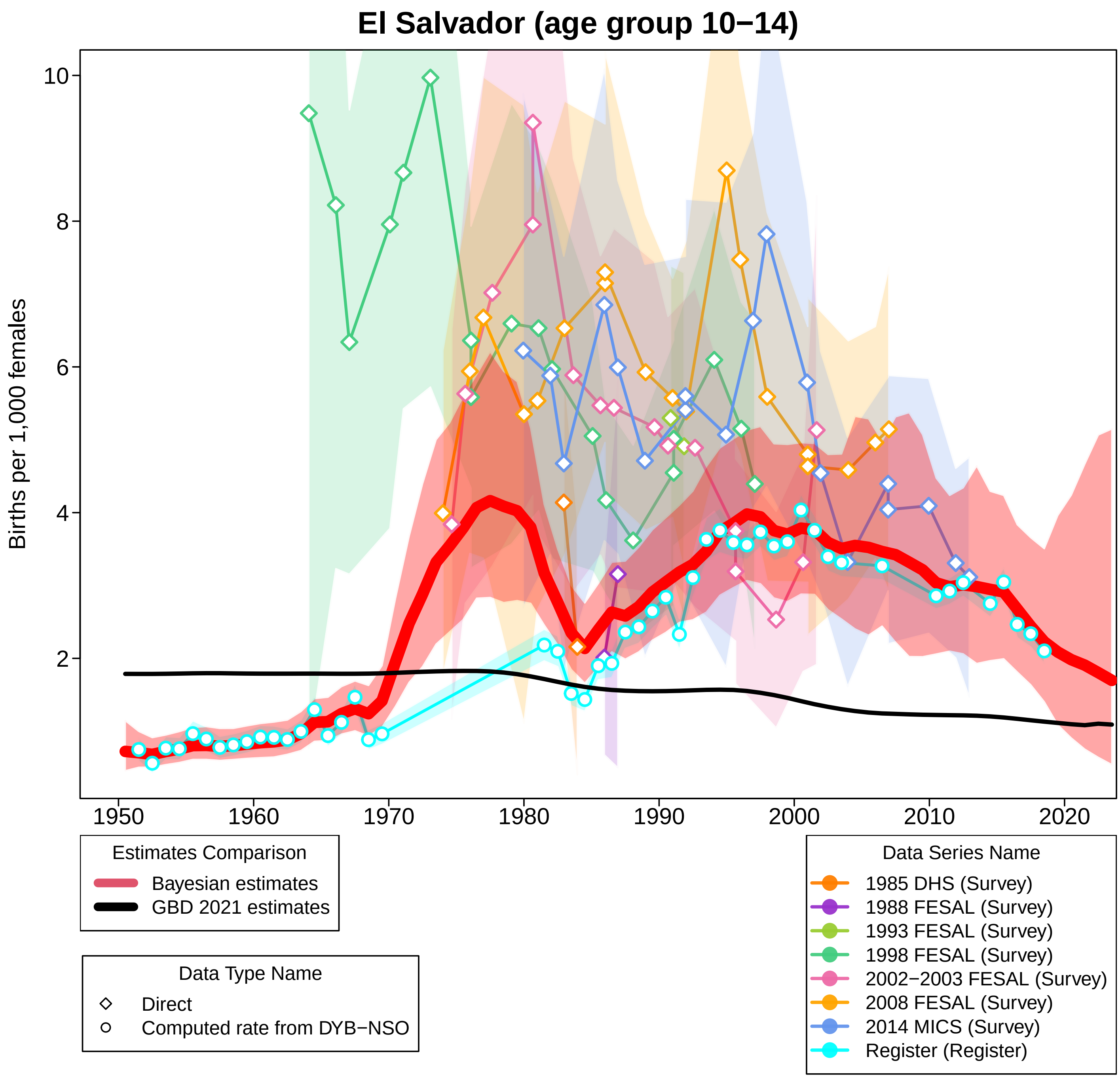
El Salvador (age group 10−14)
Births per 1,000 females
10
8
6
4
2
1950
1960
1970
1980
1990
2000
2010
2020
Estimates Comparison
Bayesian estimates
GBD 2021 estimates
Data Type Name
Direct
Computed rate from DYB−NSO
Data Series Name
1985 DHS (Survey)
1988 FESAL (Survey)
1993 FESAL (Survey)
1998 FESAL (Survey)
2002−2003 FESAL (Survey)
2008 FESAL (Survey)
2014 MICS (Survey)
Register (Register)

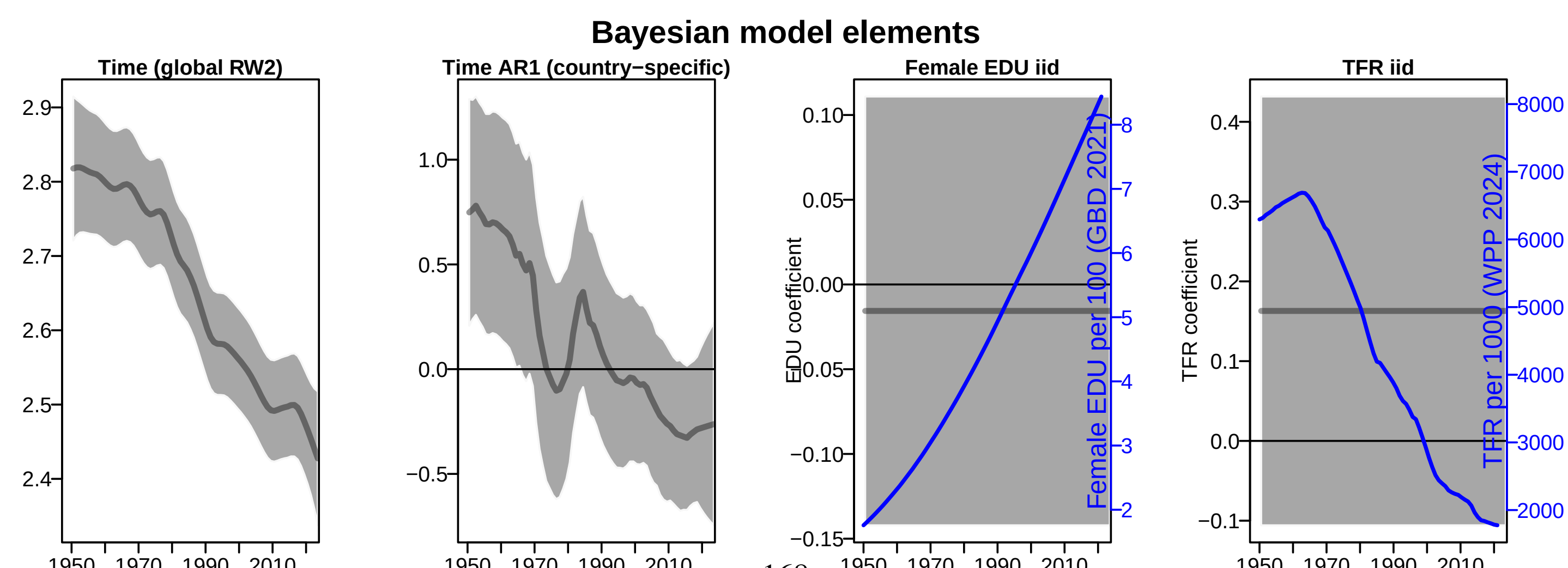
Bayesian model elements
Time (global RW2)
Time AR1 (country−specific)
Female EDU iid
TFR iid
EDU coefficient
Female EDU per 100 (GBD 2021)
TFR coefficient
TFR per 1000 (WPP 2024)
1950
1970
1990
2010

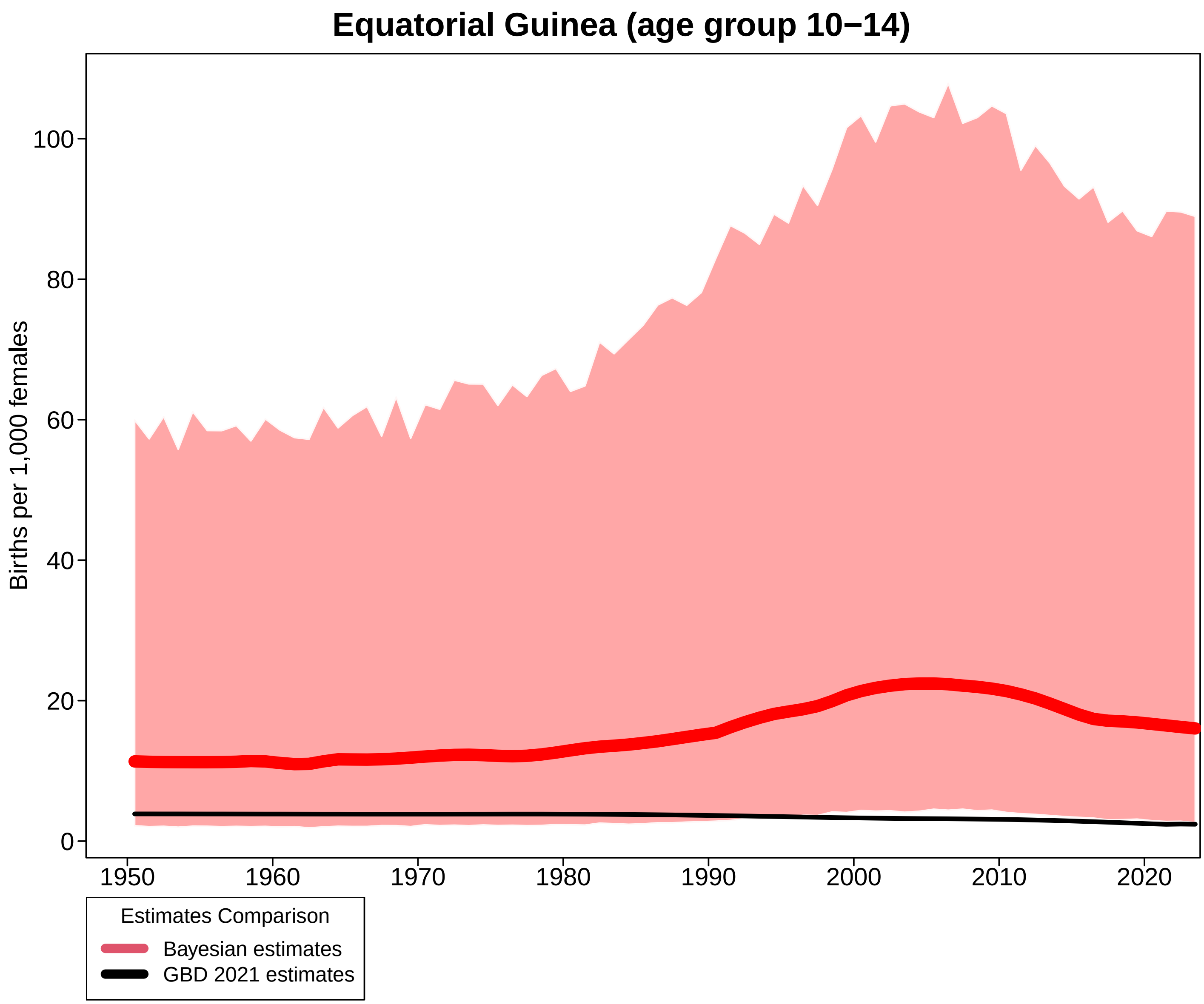

Equatorial Guinea (age group 10−14)
Births per 1,000 females
0
20
40
60
80
100
1950
1960
1970
1980
1990
2000
2010
2020
Estimates Comparison
Bayesian estimates
GBD 2021 estimates

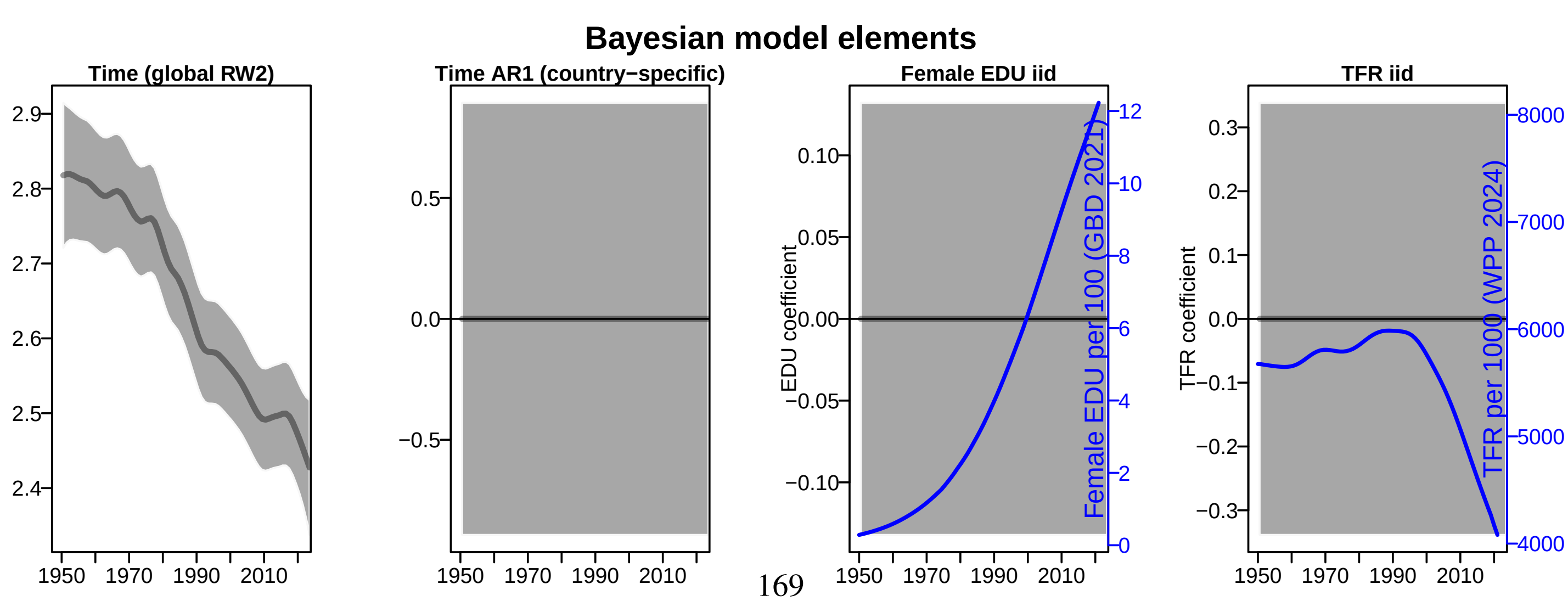

Bayesian model elements
Time (global RW2)
Time AR1 (country−specific)
Female EDU iid
TFR iid
EDU coefficient
Female EDU per 100 (GBD 2021)
TFR coefficient
TFR per 1000 (WPP 2024)
1950
1970
1990
2010

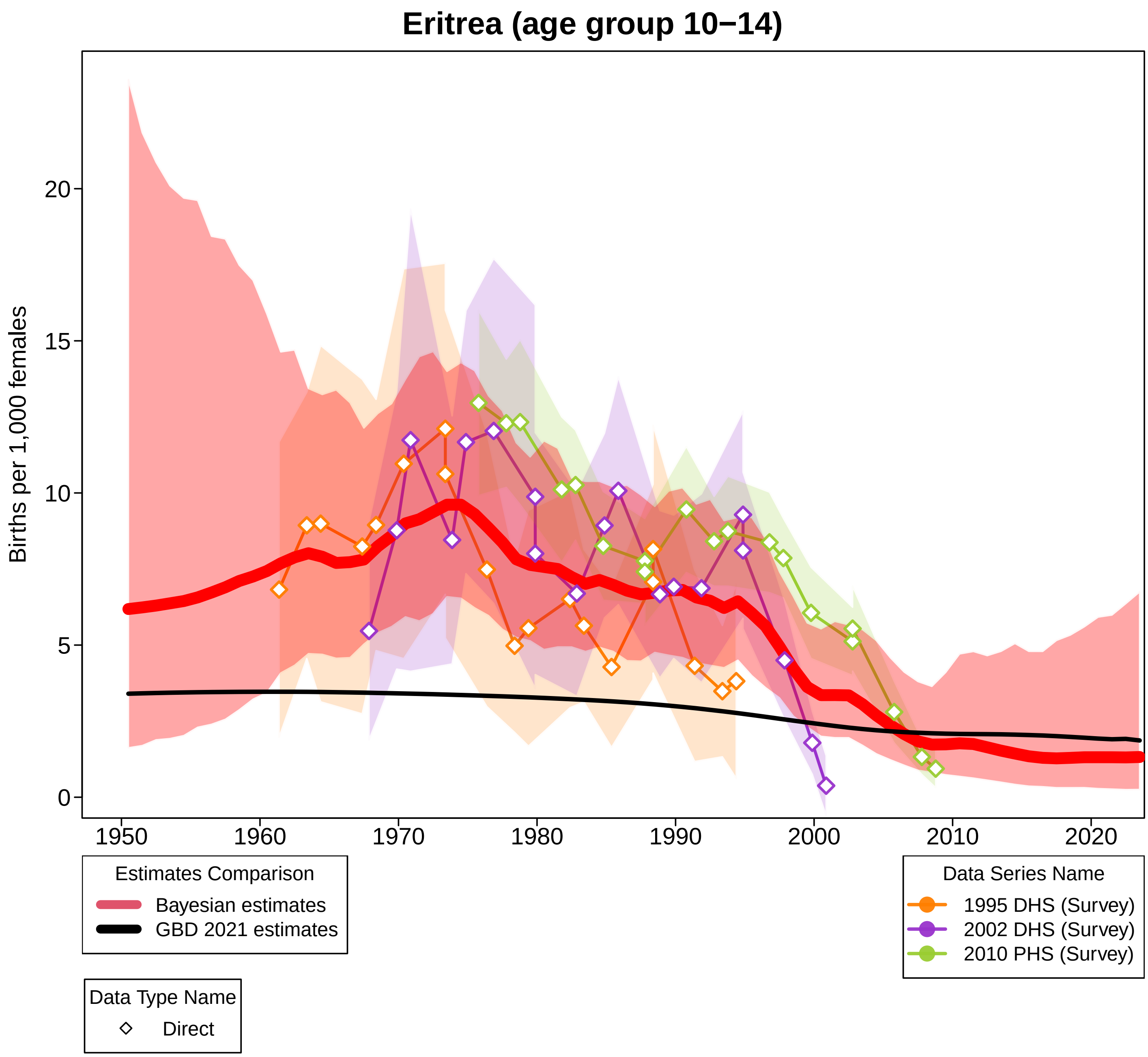
Eritrea (age group 10−14)
Births per 1,000 females
0
5
10
15
20
1950
1960
1970
1980
1990
2000
2010
2020
Estimates Comparison
Bayesian estimates
GBD 2021 estimates
Data Series Name
1995 DHS (Survey)
2002 DHS (Survey)
2010 PHS (Survey)
Data Type Name
Direct

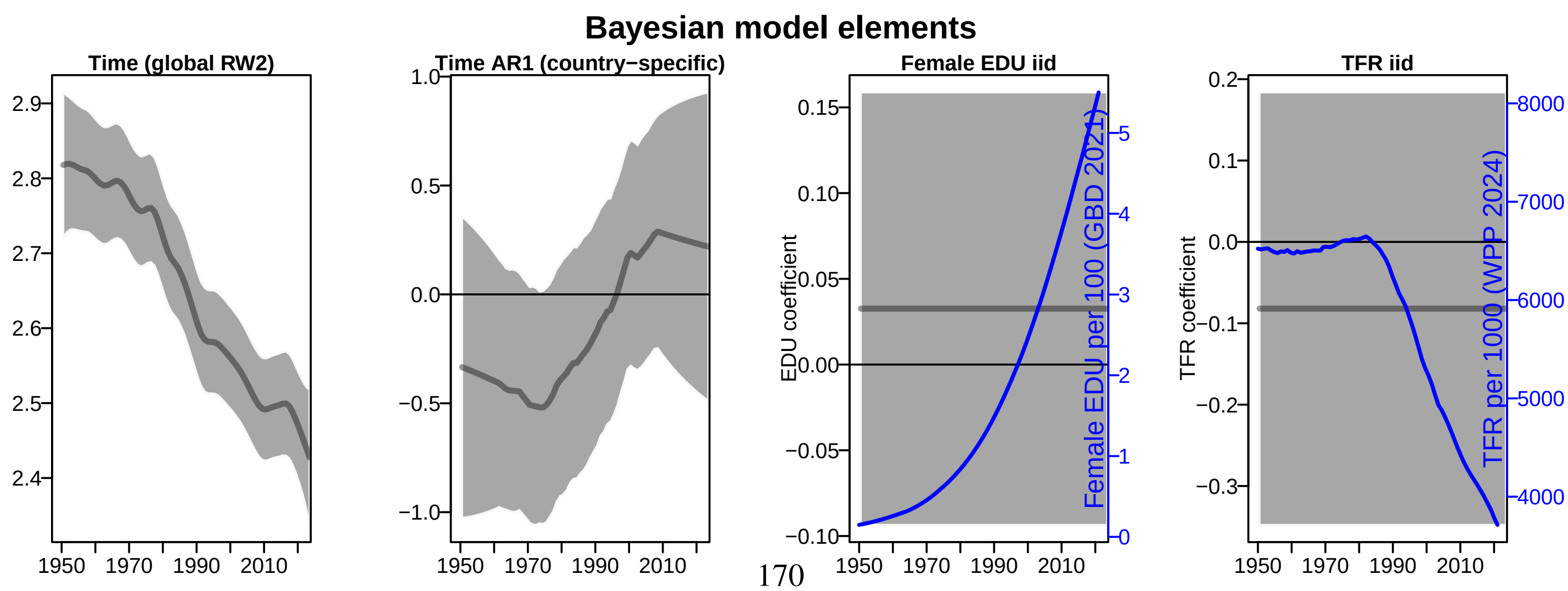
Bayesian model elements
Time (global RW2)
Time AR1 (country−specific)
Female EDU iid
TFR iid
EDU coefficient
Female EDU per 100 (GBD 2021)
TFR coefficient
TFR per 1000 (WPP 2024)
1950
1970
1990
2010

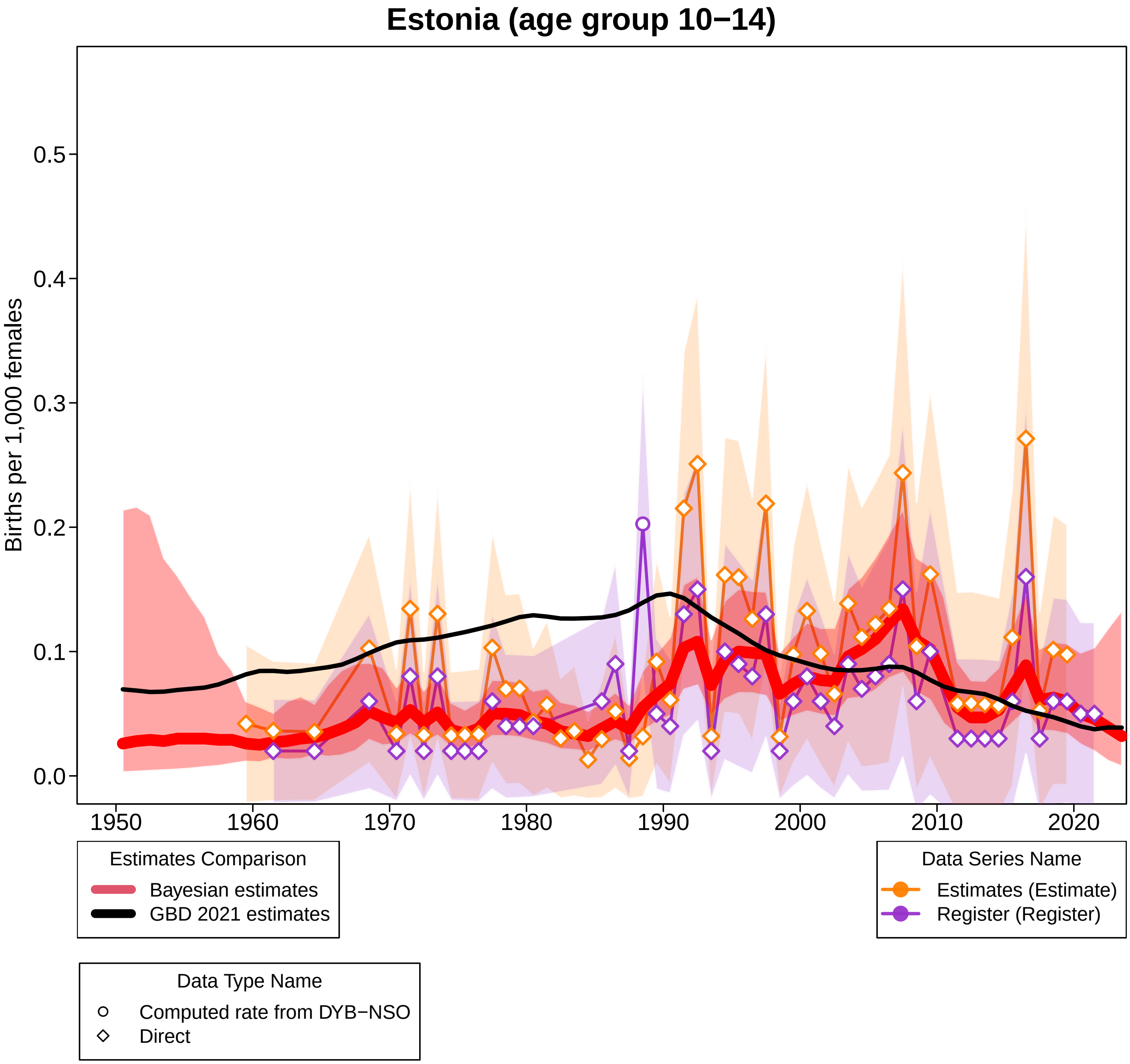

Estonia (age group 10−14)
Births per 1,000 females
0.0
0.1
0.2
0.3
0.4
0.5
1950
1960
1970
1980
1990
2000
2010
2020
Estimates Comparison
Bayesian estimates
GBD 2021 estimates
Data Series Name
Estimates (Estimate)
Register (Register)
Data Type Name
Computed rate from DYB−NSO
Direct

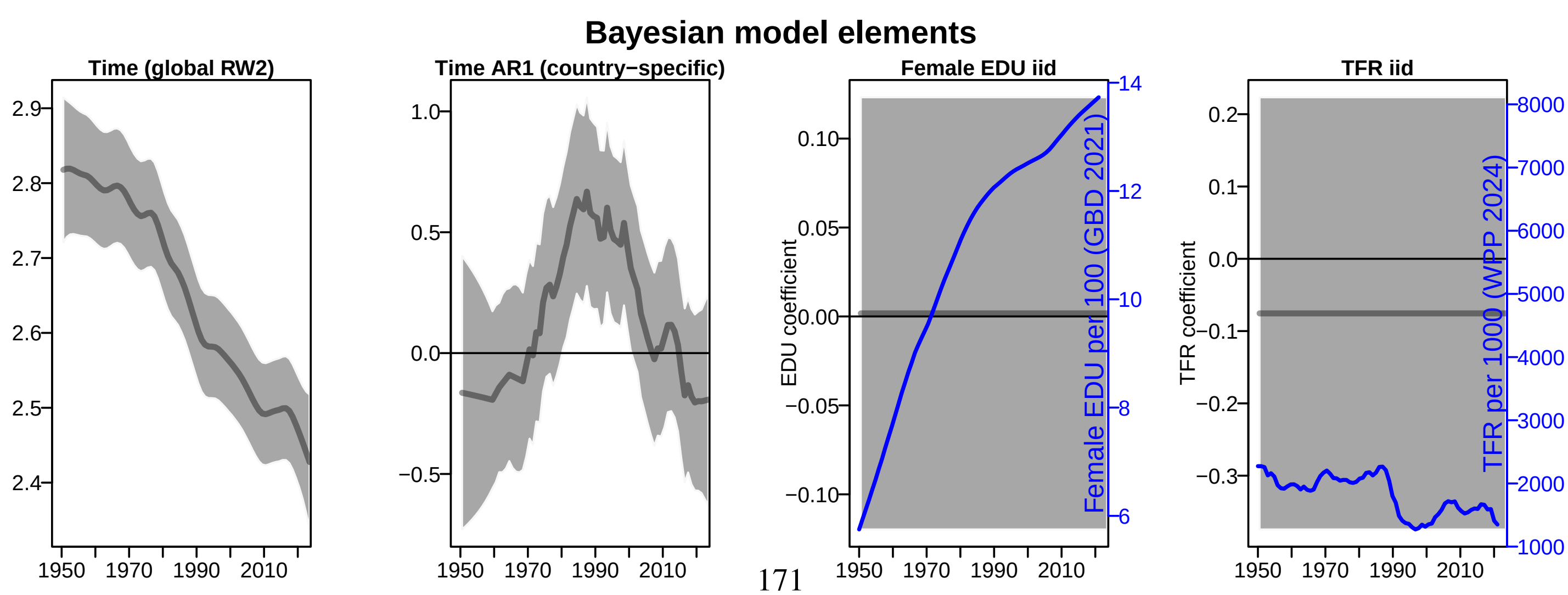

Bayesian model elements
Time (global RW2)
Time AR1 (country−specific)
Female EDU iid
TFR iid
EDU coefficient
Female EDU per 100 (GBD 2021)
TFR coefficient
TFR per 1000 (WPP 2024)
1950
1970
1990
2010

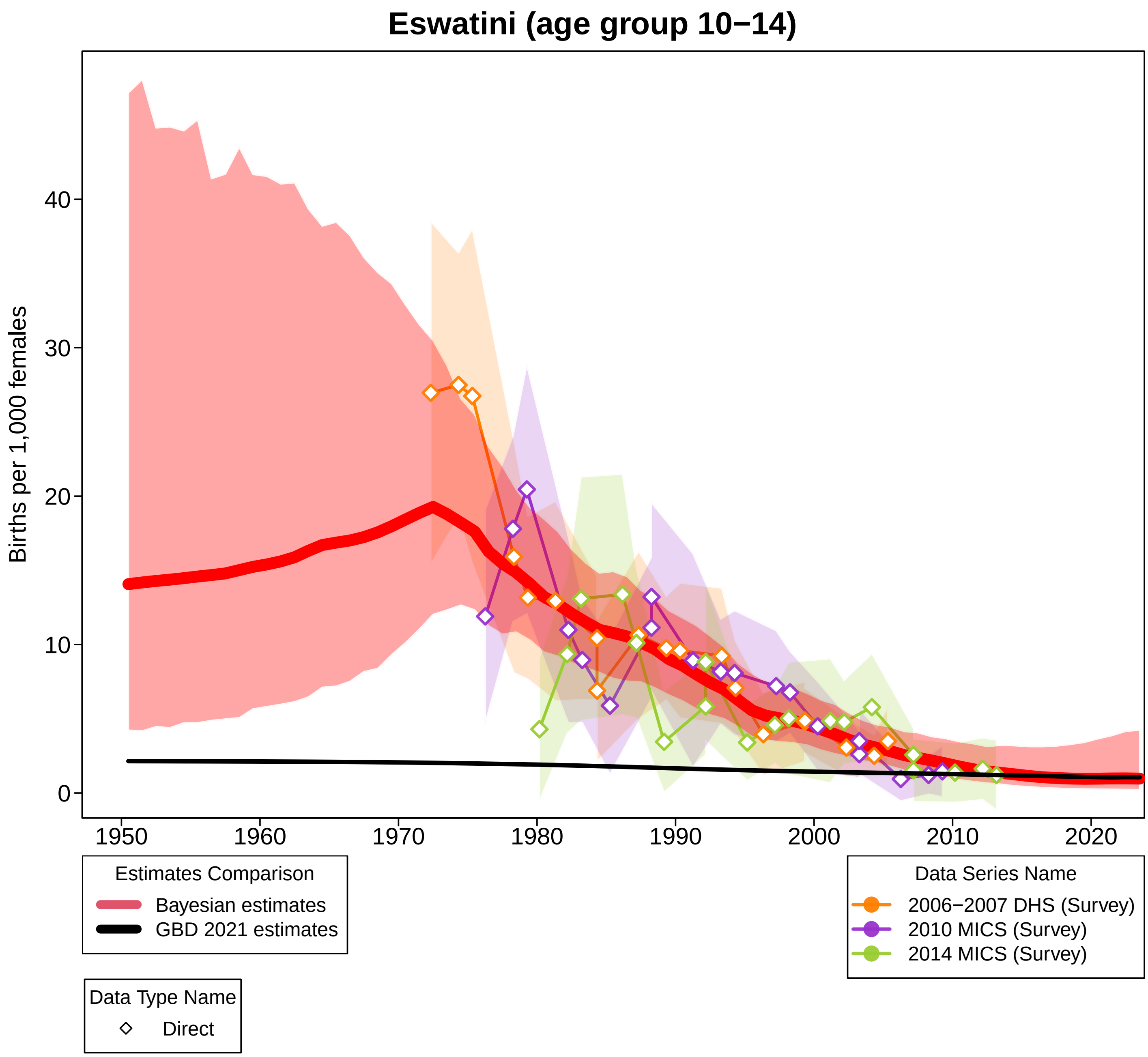
Eswatini (age group 10−14)
Births per 1,000 females
0
10
20
30
40
1950
1960
1970
1980
1990
2000
2010
2020
Estimates Comparison
Bayesian estimates
GBD 2021 estimates
Data Series Name
2006−2007 DHS (Survey)
2010 MICS (Survey)
2014 MICS (Survey)
Data Type Name
Direct

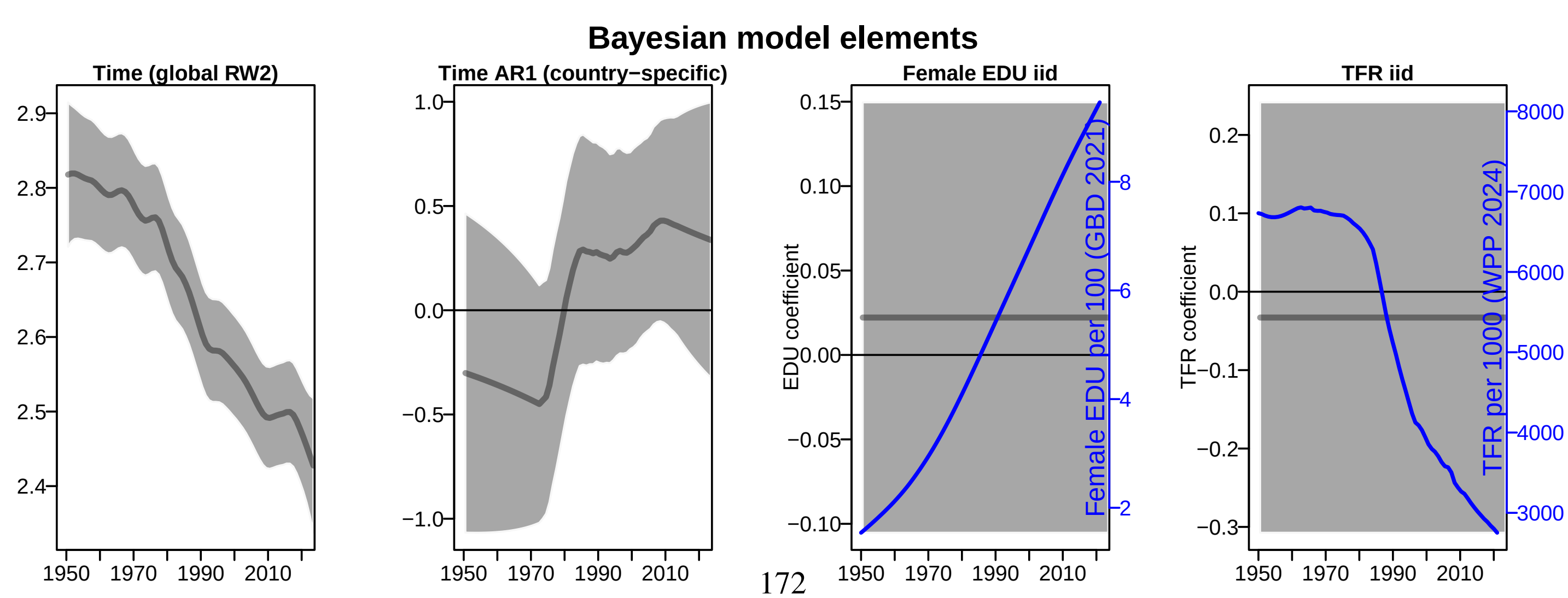
Bayesian model elements
Time (global RW2)
Time AR1 (country−specific)
Female EDU iid
TFR iid
EDU coefficient
Female EDU per 100 (GBD 2021)
TFR coefficient
TFR per 1000 (WPP 2024)
1950
1970
1990
2010

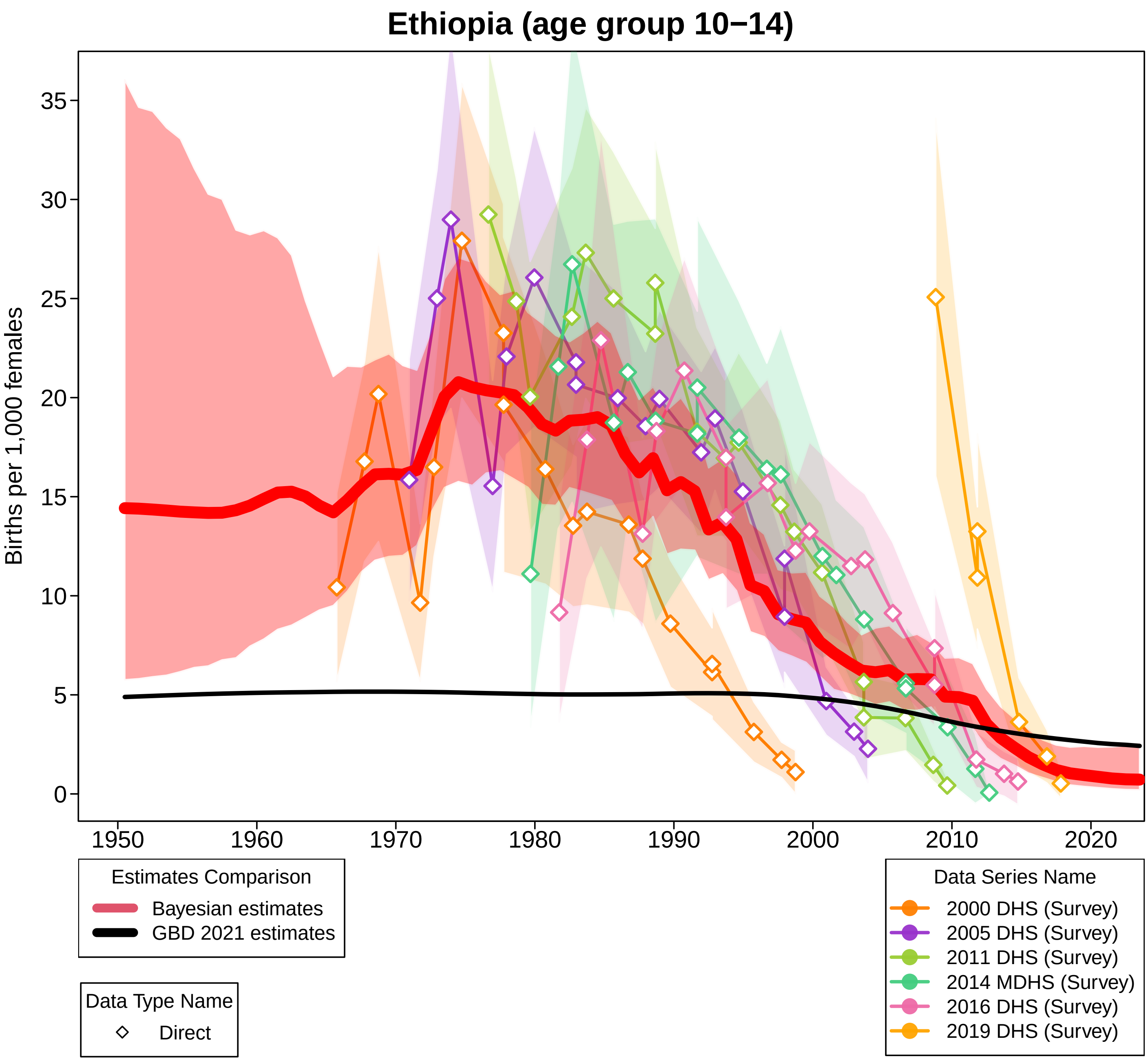
Ethiopia (age group 10−14)
Births per 1,000 females
0
5
10
15
20
25
30
35
1950
1960
1970
1980
1990
2000
2010
2020
Estimates Comparison
Bayesian estimates
GBD 2021 estimates
Data Type Name
Direct
Data Series Name
2000 DHS (Survey)
2005 DHS (Survey)
2011 DHS (Survey)
2014 MDHS (Survey)
2016 DHS (Survey)
2019 DHS (Survey)

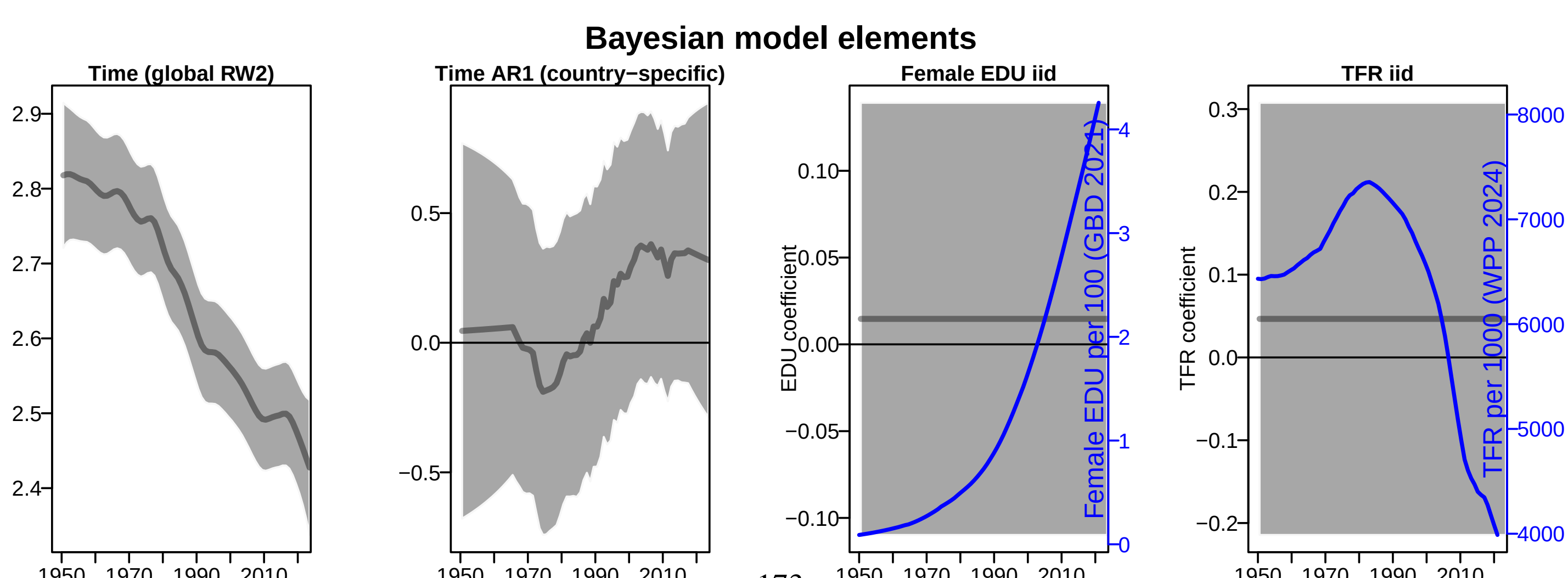
Bayesian model elements
Time (global RW2)
Time AR1 (country−specific)
Female EDU iid
TFR iid
EDU coefficient
Female EDU per 100 (GBD 2021)
TFR coefficient
TFR per 1000 (WPP 2024)
1950
1970
1990
2010

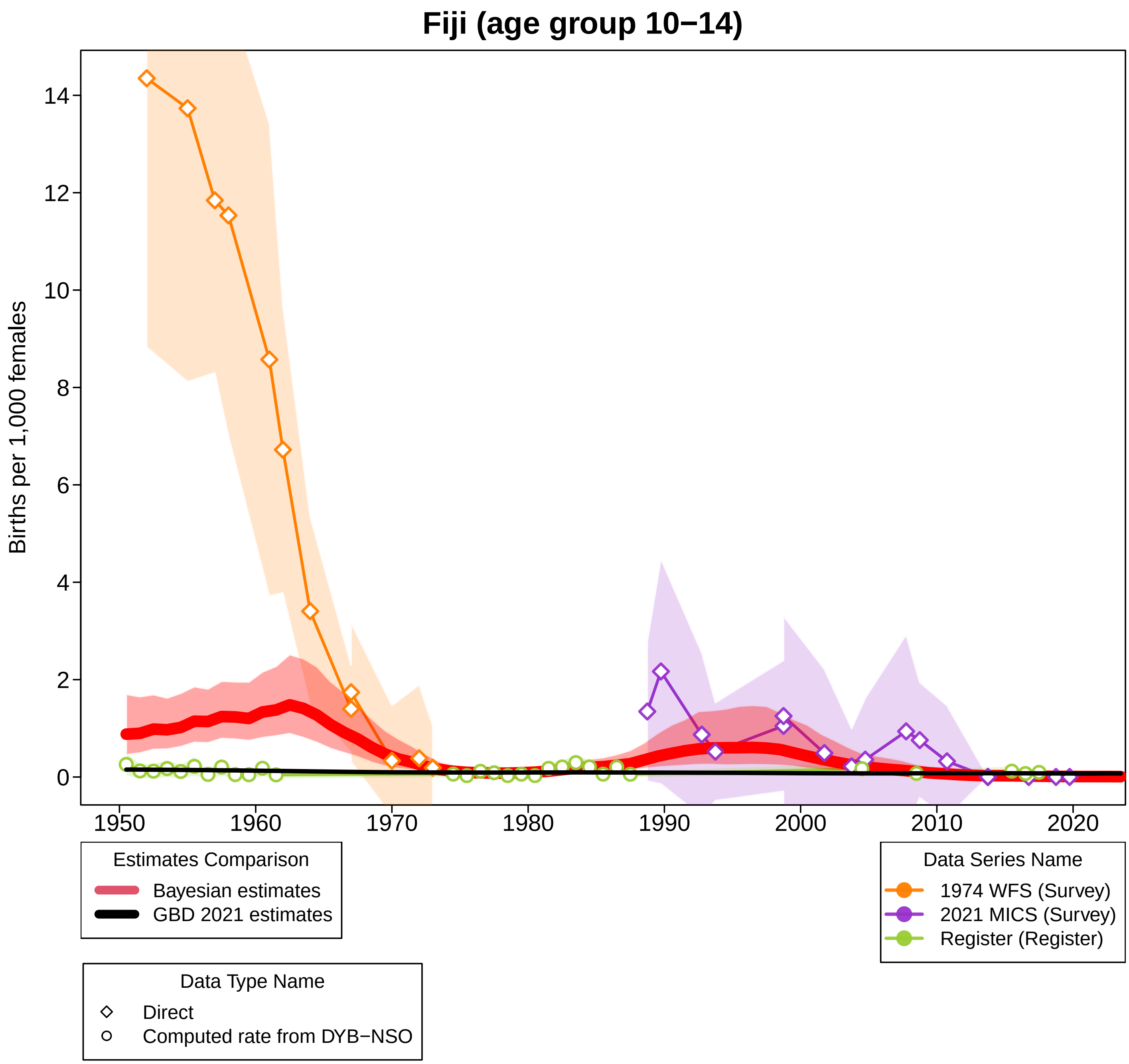
Fiji (age group 10−14)
Births per 1,000 females
0
2
4
6
8
10
12
14
1950
1960
1970
1980
1990
2000
2010
2020
Estimates Comparison
Bayesian estimates
GBD 2021 estimates
Data Series Name
1974 WFS (Survey)
2021 MICS (Survey)
Register (Register)
Data Type Name
Direct
Computed rate from DYB−NSO

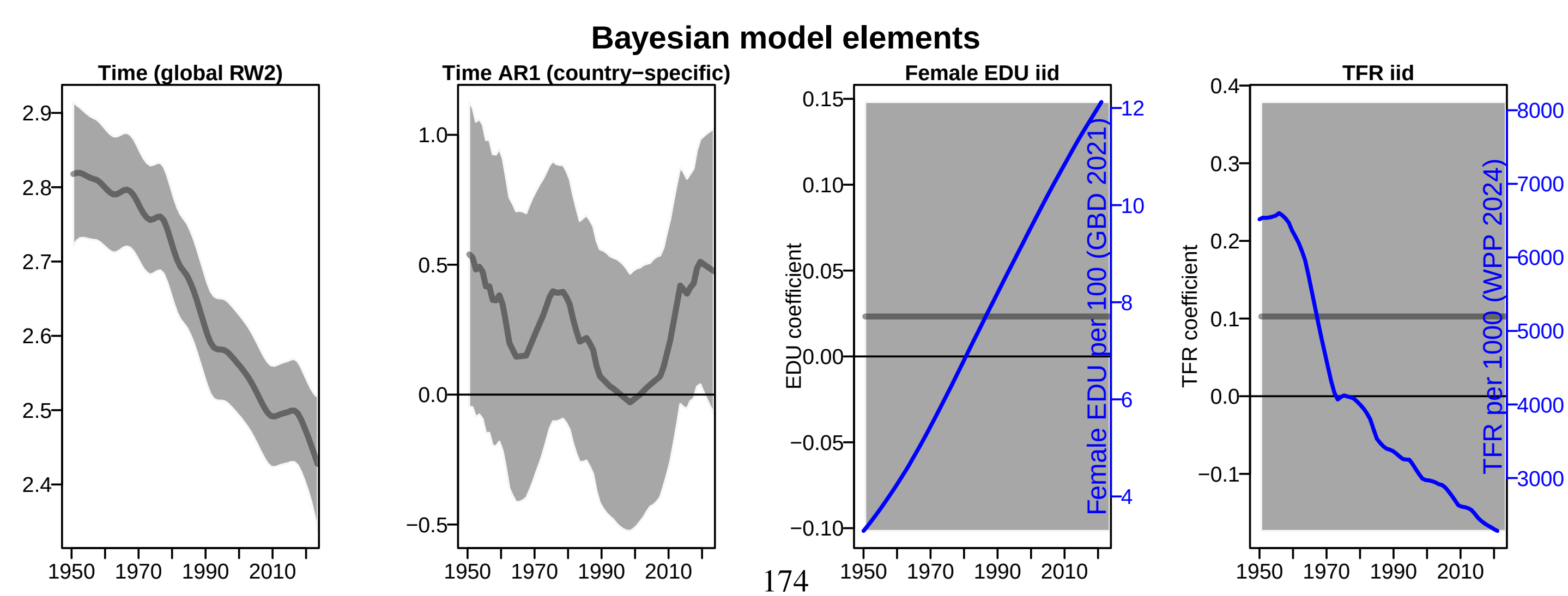
Bayesian model elements
Time (global RW2)
Time AR1 (country−specific)
Female EDU iid
TFR iid
EDU coefficient
Female EDU per 100 (GBD 2021)
TFR coefficient
TFR per 1000 (WPP 2024)
1950
1970
1990
2010

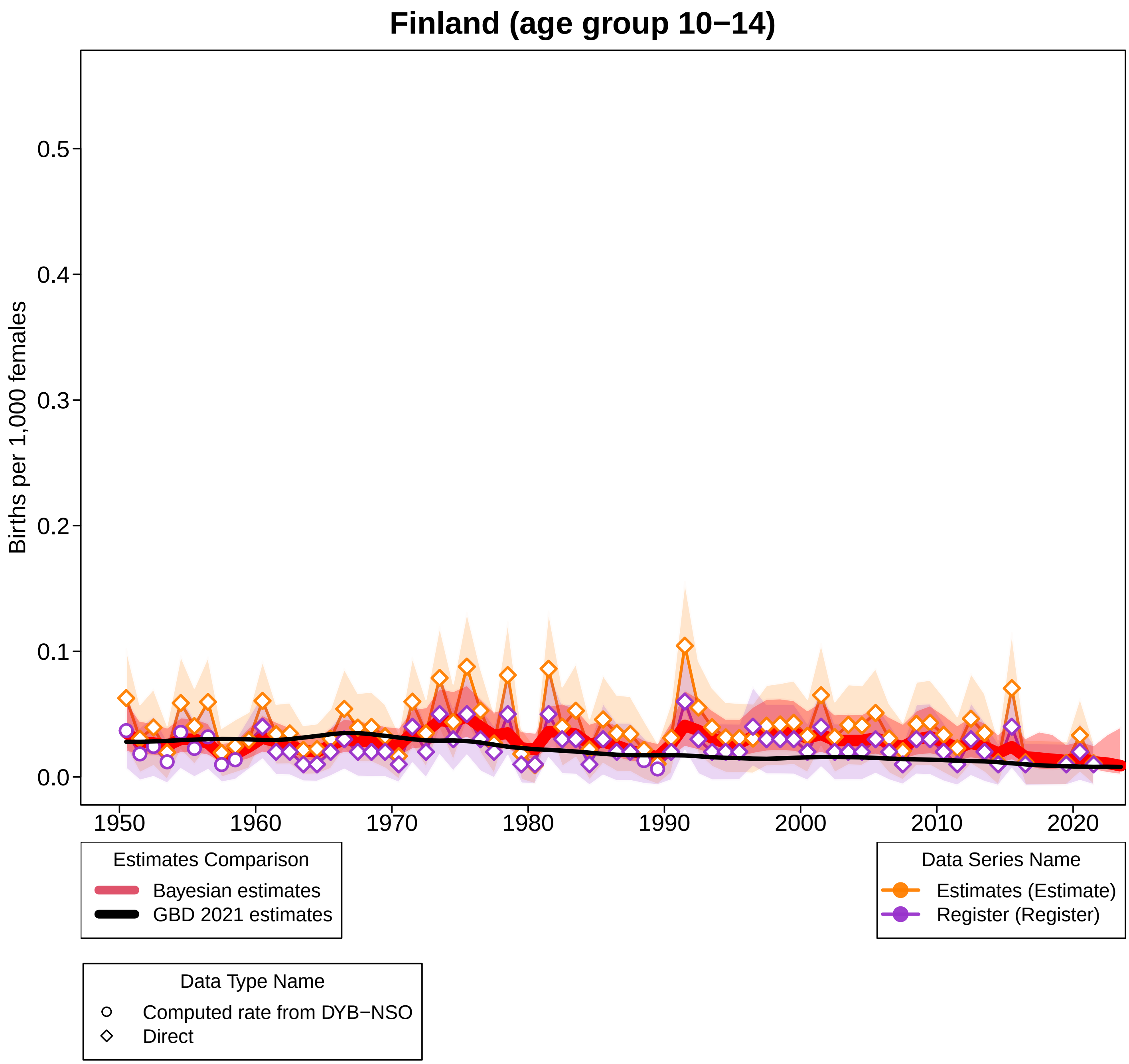
Finland (age group 10−14)
Births per 1,000 females
0.0
0.1
0.2
0.3
0.4
0.5
1950
1960
1970
1980
1990
2000
2010
2020
Estimates Comparison
Bayesian estimates
GBD 2021 estimates
Data Series Name
Estimates (Estimate)
Register (Register)
Data Type Name
Computed rate from DYB−NSO
Direct

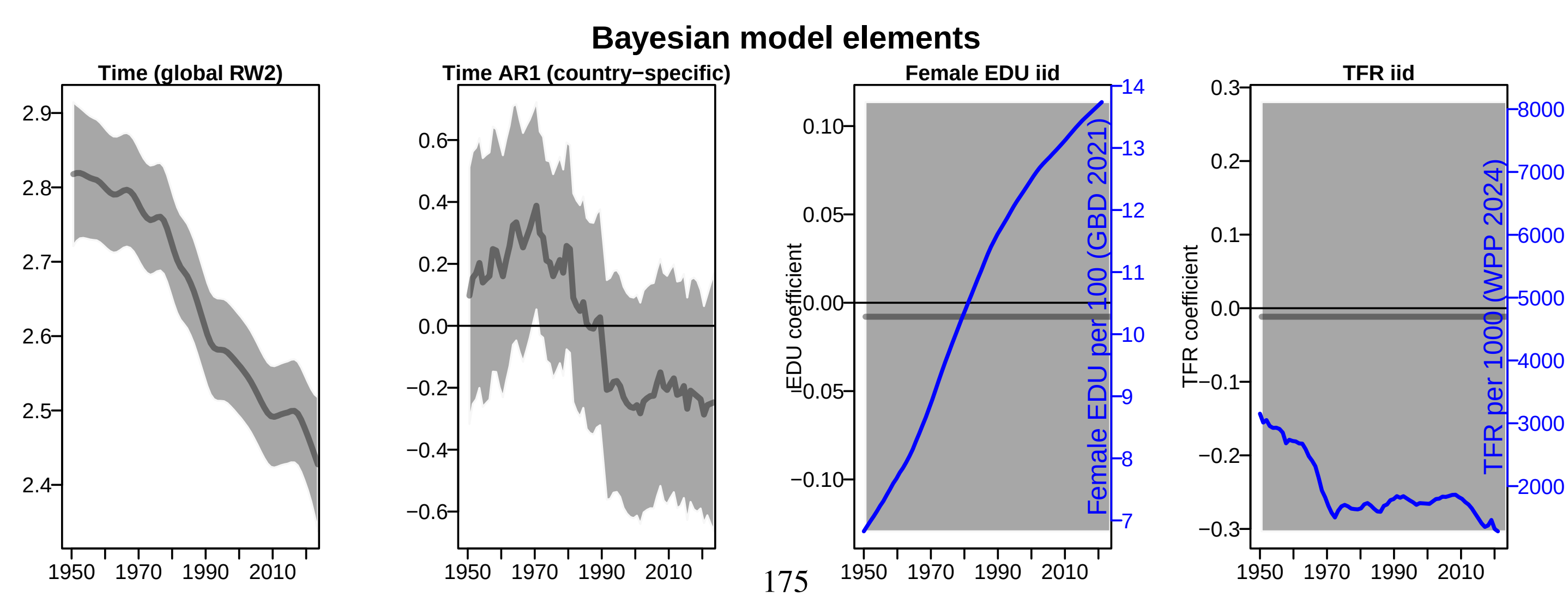
Bayesian model elements
Time (global RW2)
Time AR1 (country−specific)
Female EDU iid
TFR iid
EDU coefficient
TFR coefficient
Female EDU per 100 (GBD 2021)
TFR per 1000 (WPP 2024)
1950
1970
1990
2010

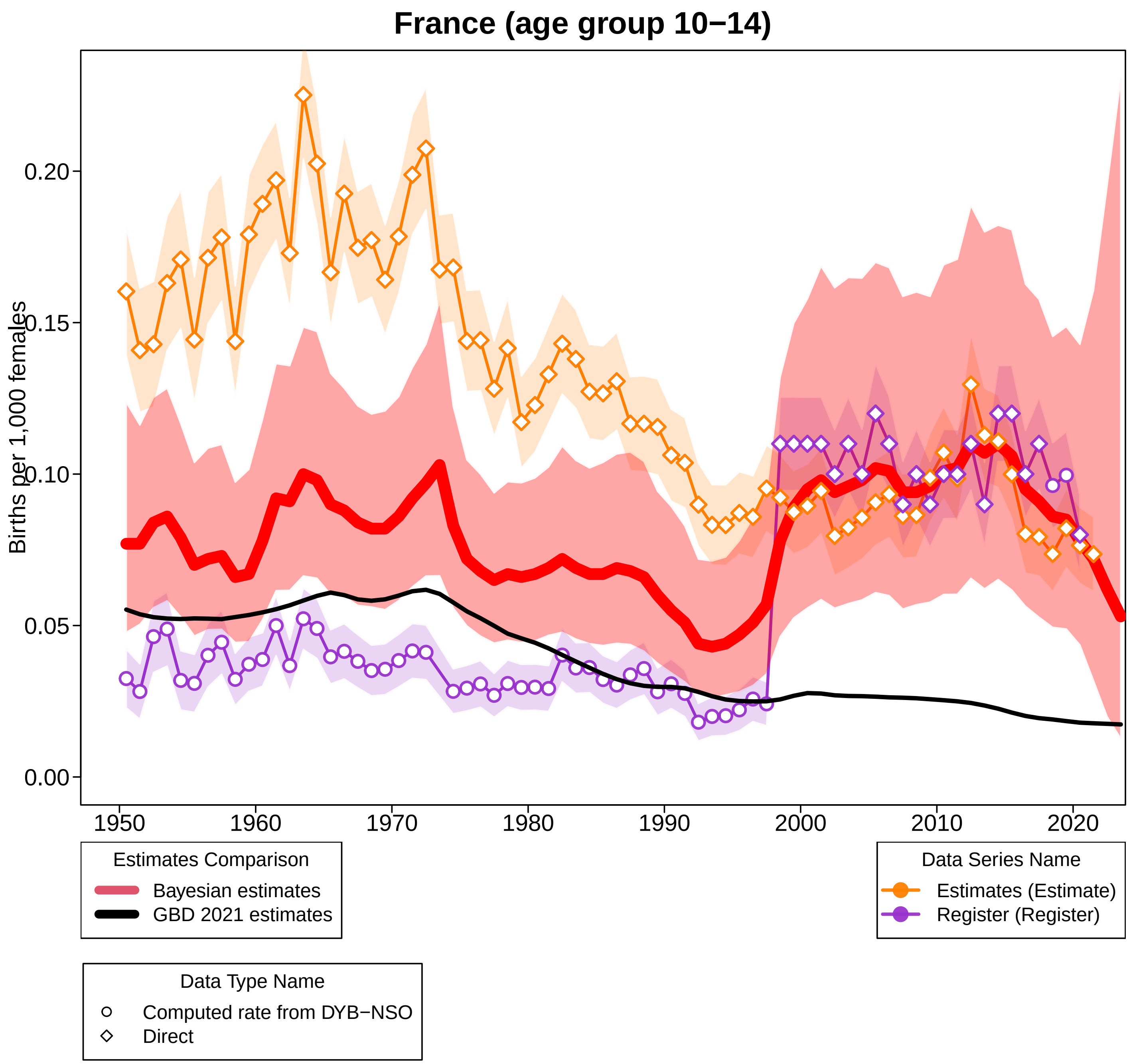
France (age group 10−14)
Births per 1,000 females
0.20
0.15
0.10
0.05
0.00
1950
1960
1970
1980
1990
2000
2010
2020
Estimates Comparison
Bayesian estimates
GBD 2021 estimates
Data Series Name
Estimates (Estimate)
Register (Register)
Data Type Name
Computed rate from DYB−NSO
Direct

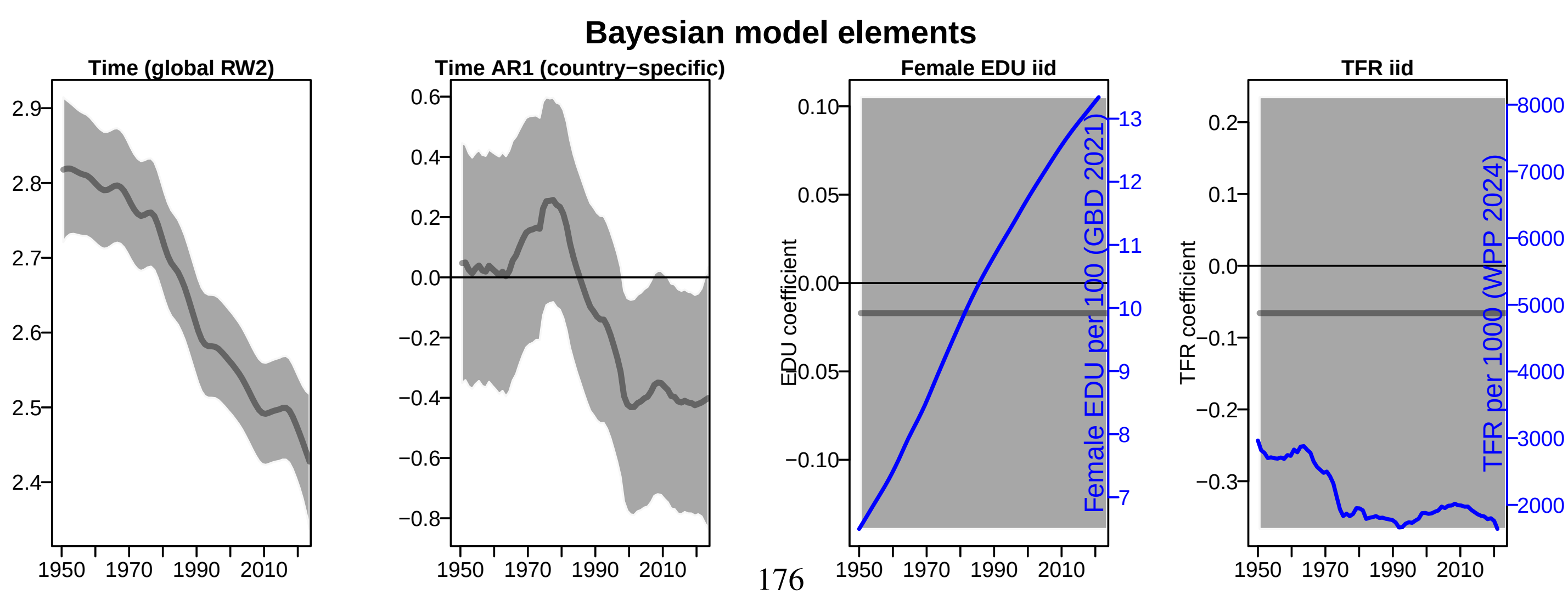
Bayesian model elements
Time (global RW2)
Time AR1 (country−specific)
Female EDU iid
TFR iid
EDU coefficient
Female EDU per 100 (GBD 2021)
TFR coefficient
TFR per 1000 (WPP 2024)
1950
1970
1990
2010

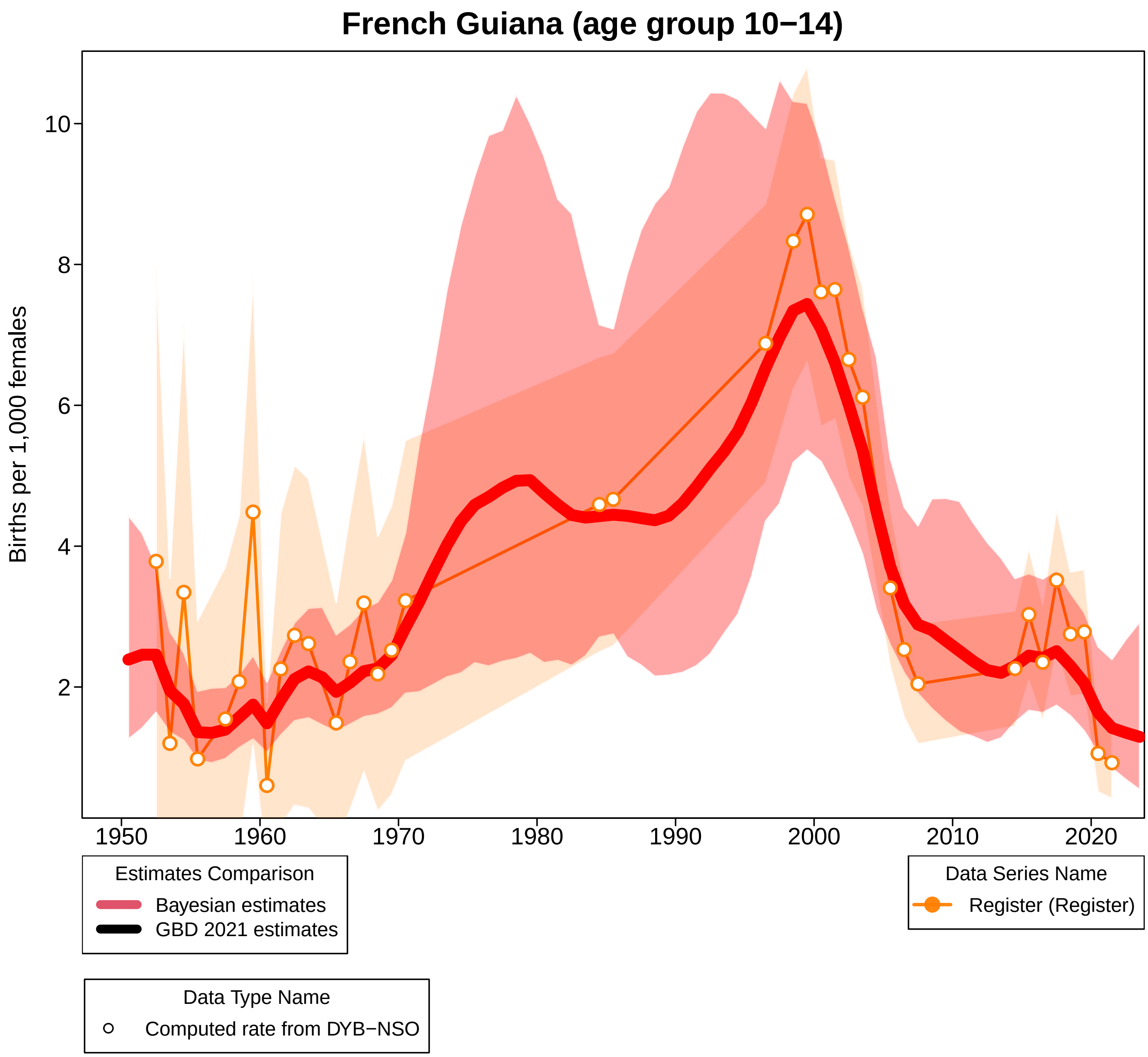
French Guiana (age group 10−14)
Births per 1,000 females
10
8
6
4
2
1950
1960
1970
1980
1990
2000
2010
2020
Estimates Comparison
Bayesian estimates
GBD 2021 estimates
Data Series Name
Register (Register)
Data Type Name
Computed rate from DYB−NSO

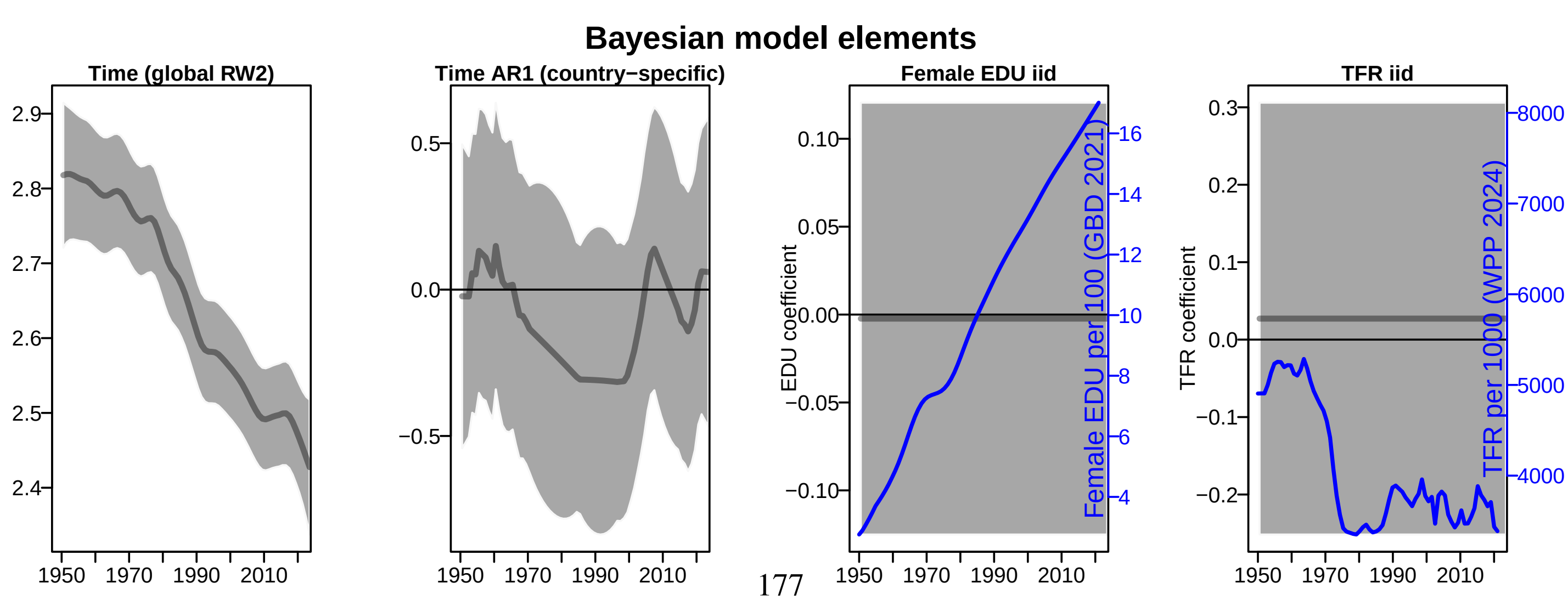
Bayesian model elements
Time (global RW2)
Time AR1 (country−specific)
Female EDU iid
TFR iid
EDU coefficient
Female EDU per 100 (GBD 2021)
TFR coefficient
TFR per 1000 (WPP 2024)
1950
1970
1990
2010

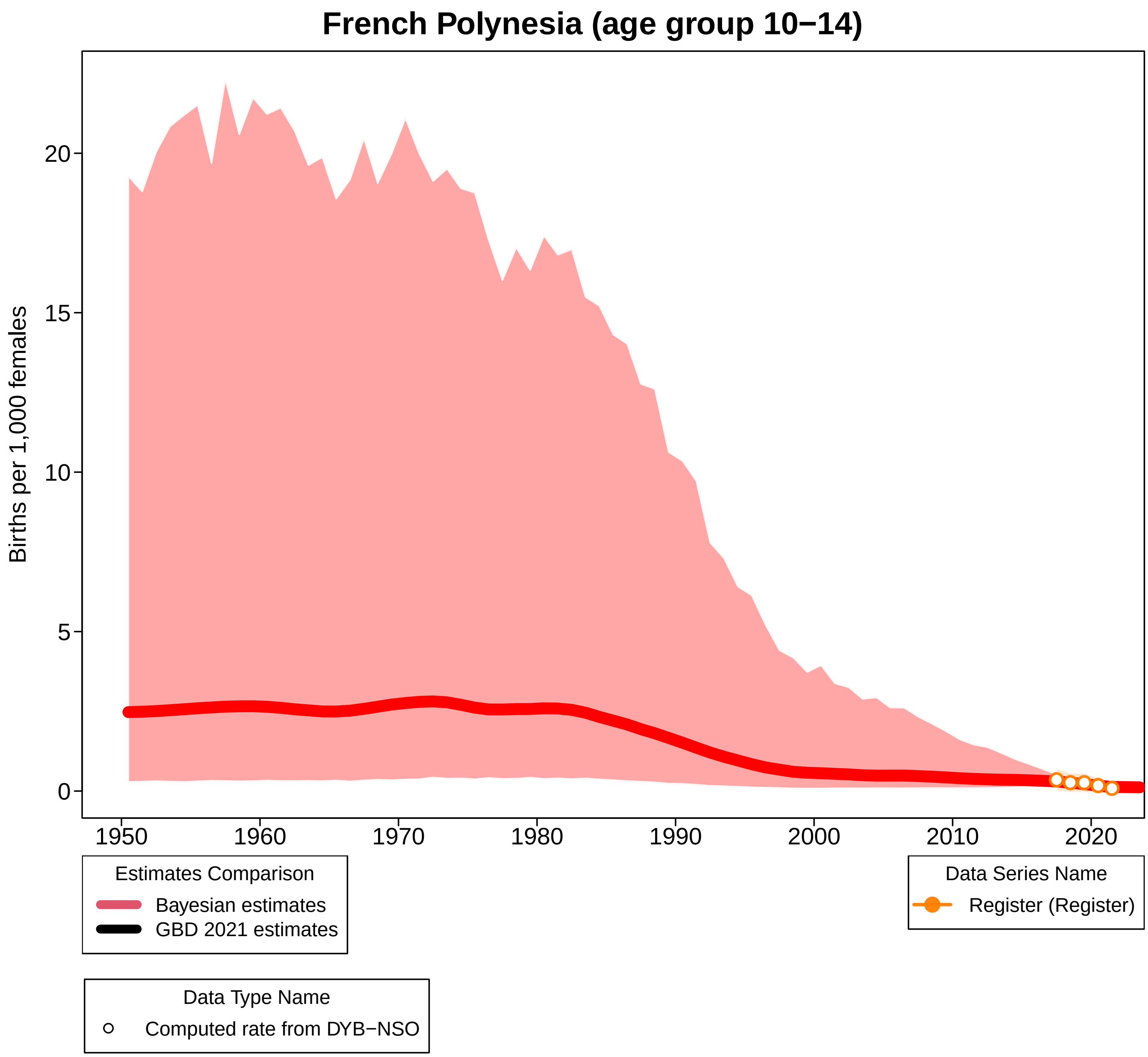
French Polynesia (age group 10−14)
Births per 1,000 females
0
5
10
15
20
1950
1960
1970
1980
1990
2000
2010
2020
Estimates Comparison
Bayesian estimates
GBD 2021 estimates
Data Series Name
Register (Register)
Data Type Name
Computed rate from DYB−NSO

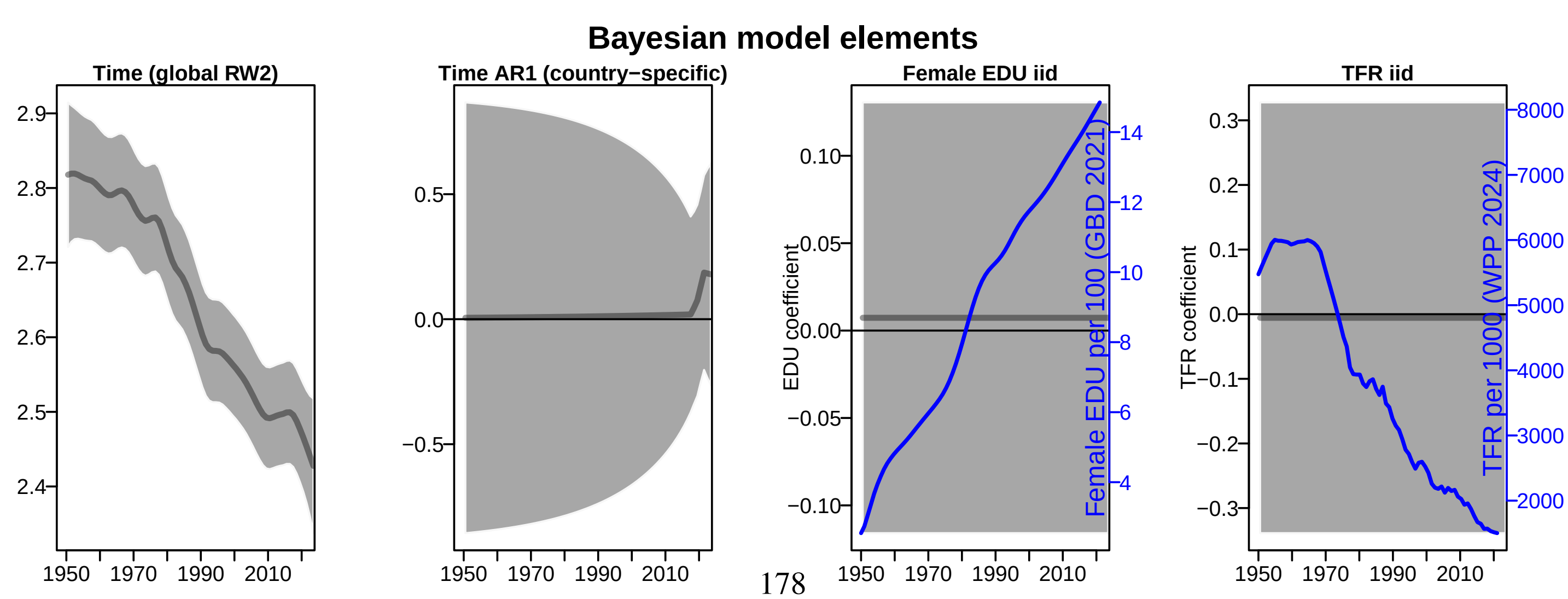
Bayesian model elements
Time (global RW2)
Time AR1 (country−specific)
Female EDU iid
TFR iid
EDU coefficient
Female EDU per 100 (GBD 2021)
TFR coefficient
TFR per 1000 (WPP 2024)
1950
1970
1990
2010

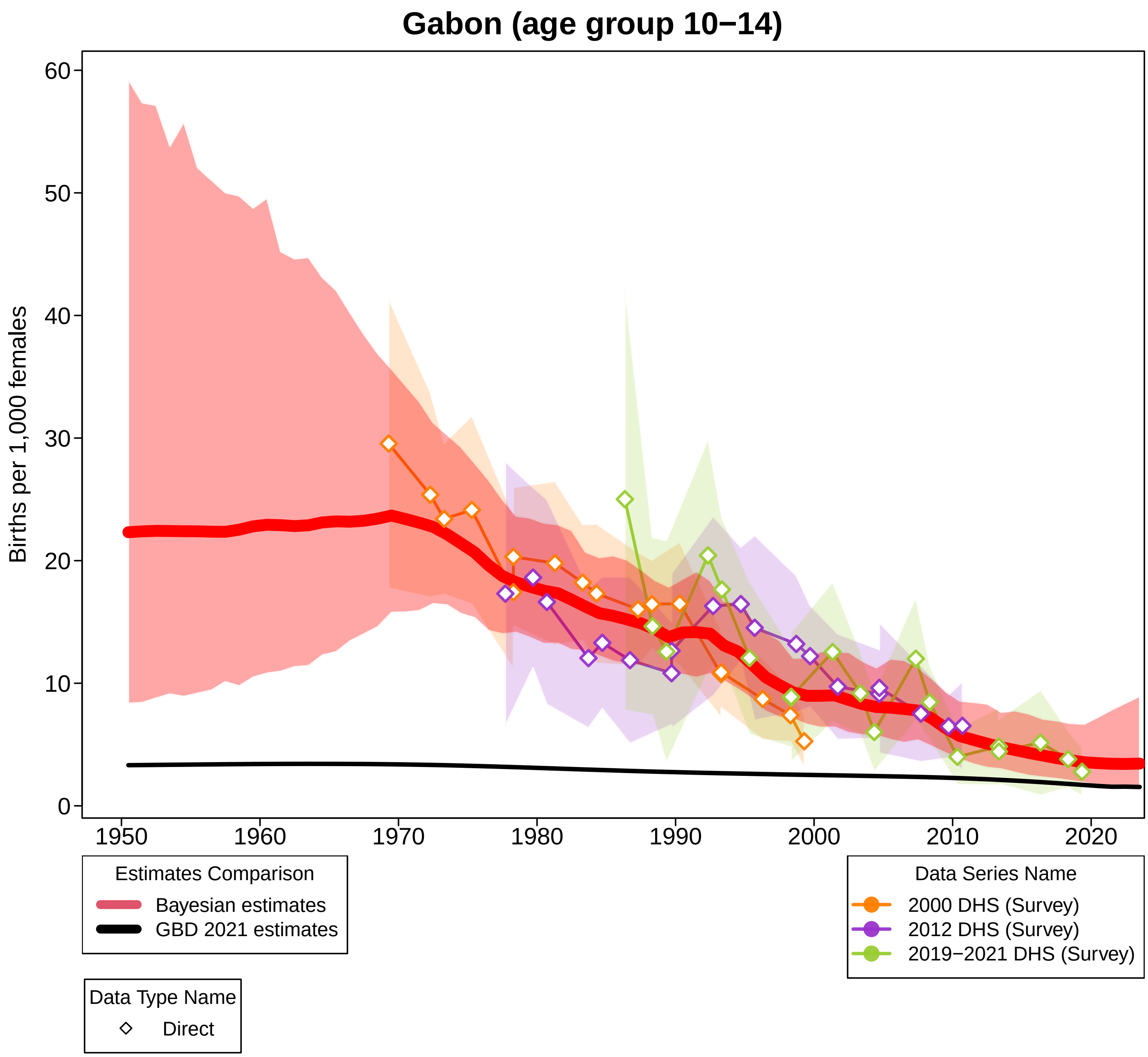

Gabon (age group 10−14)
Births per 1,000 females
0
10
20
30
40
50
60
1950
1960
1970
1980
1990
2000
2010
2020
Estimates Comparison
Bayesian estimates
GBD 2021 estimates
Data Series Name
2000 DHS (Survey)
2012 DHS (Survey)
2019−2021 DHS (Survey)
Data Type Name
Direct

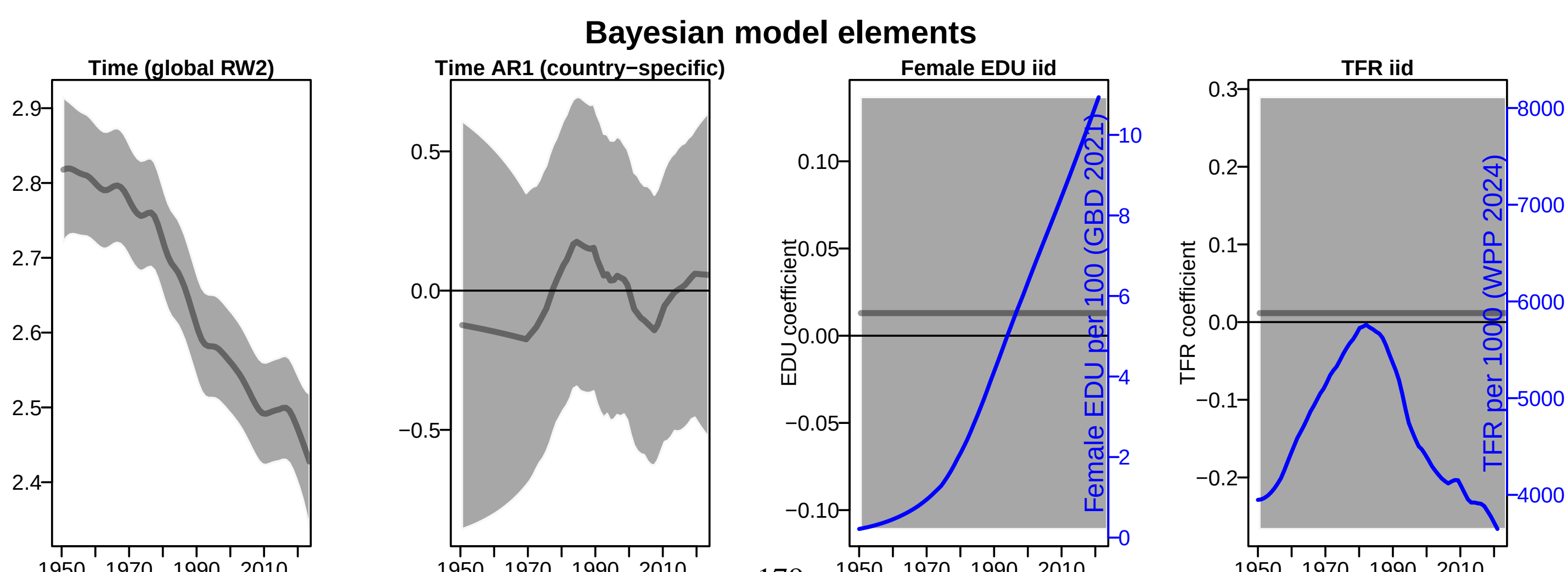

Bayesian model elements
Time (global RW2)
Time AR1 (country−specific)
Female EDU iid
TFR iid
EDU coefficient
Female EDU per 100 (GBD 2021)
TFR coefficient
TFR per 1000 (WPP 2024)
1950
1970
1990
2010

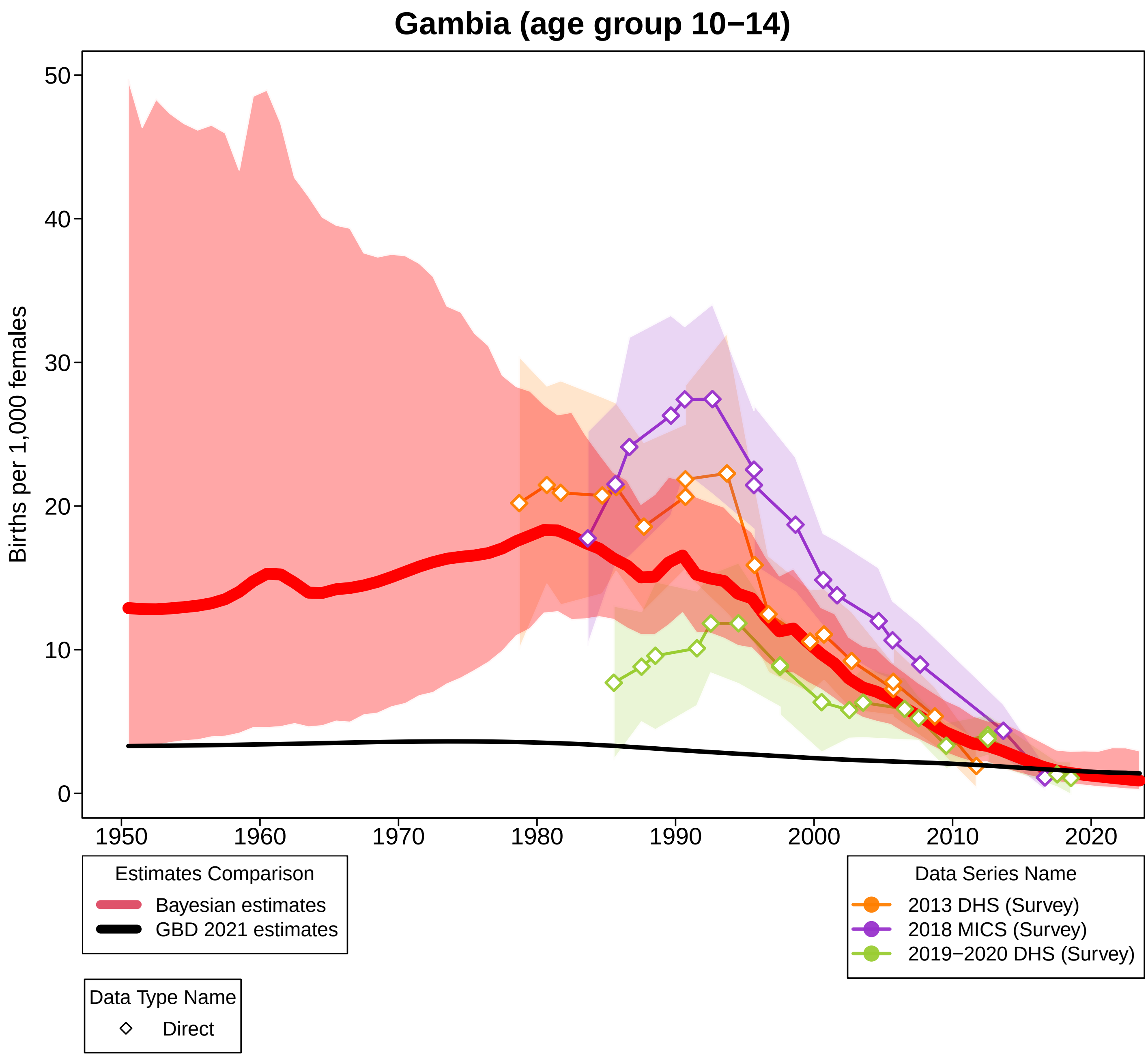
Gambia (age group 10−14)
Births per 1,000 females
0
10
20
30
40
50
1950
1960
1970
1980
1990
2000
2010
2020
Estimates Comparison
Bayesian estimates
GBD 2021 estimates
Data Series Name
2013 DHS (Survey)
2018 MICS (Survey)
2019−2020 DHS (Survey)
Data Type Name
Direct

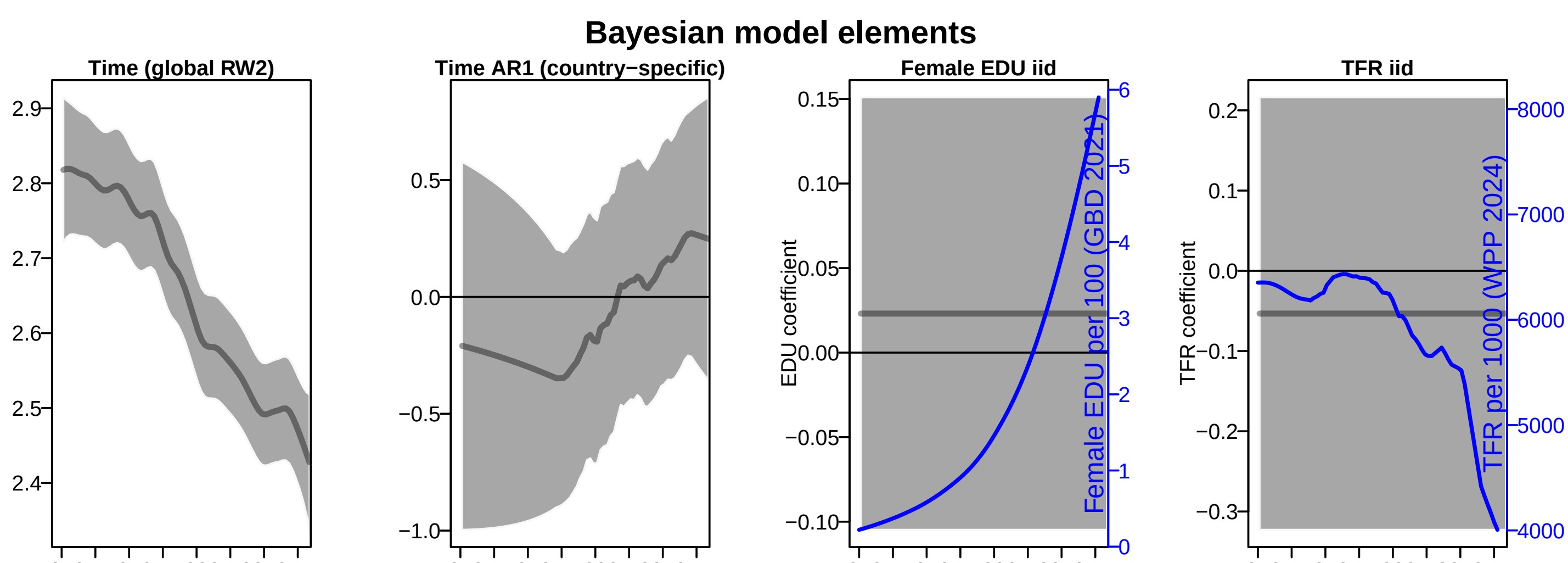
Bayesian model elements
Time (global RW2)
Time AR1 (country−specific)
Female EDU iid
TFR iid
EDU coefficient
Female EDU per 100 (GBD 2021)
TFR coefficient
TFR per 1000 (WPP 2024)
1950
1970
1990
2010

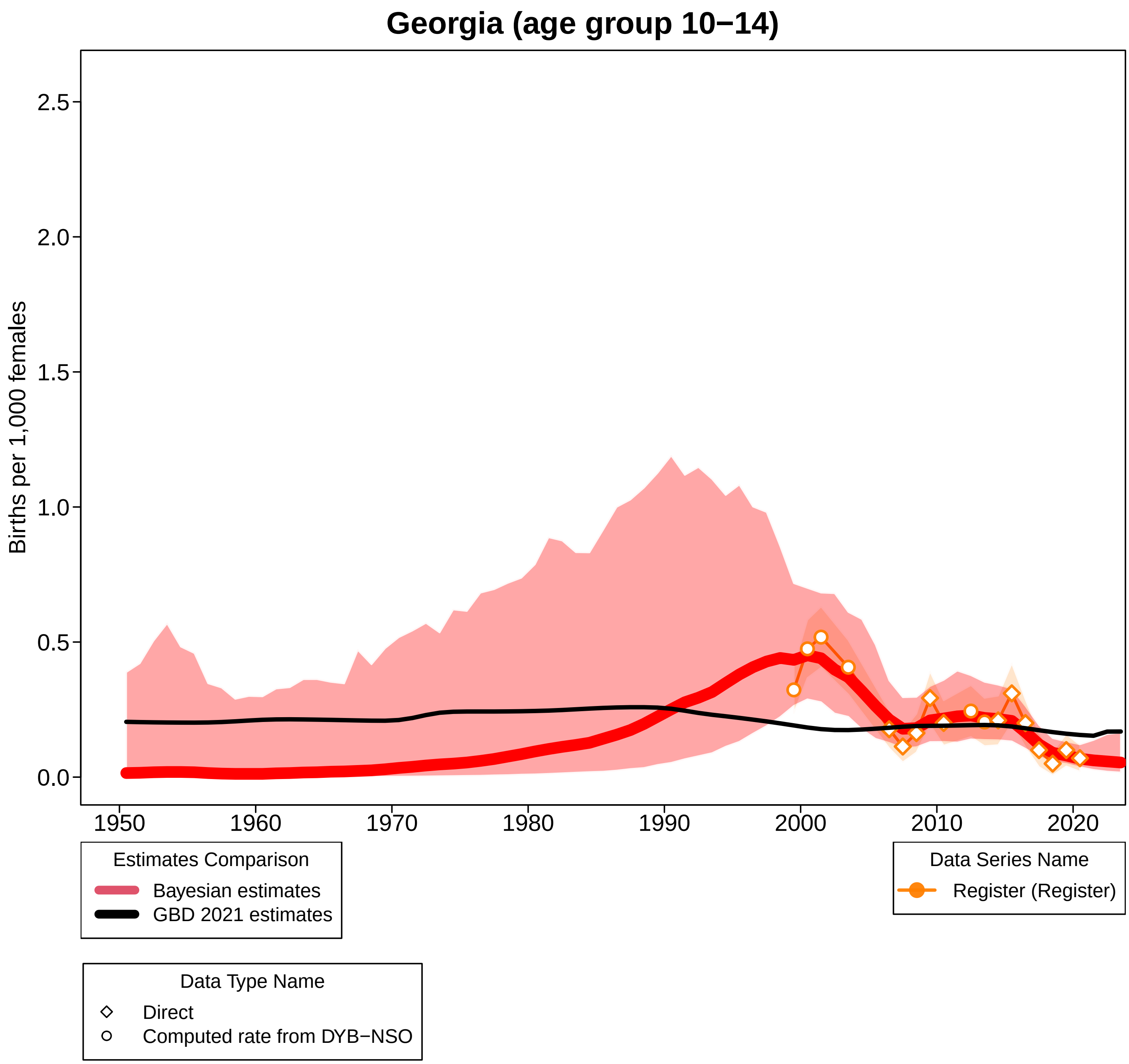

Georgia (age group 10−14)
Births per 1,000 females
2.5
2.0
1.5
1.0
0.5
0.0
1950
1960
1970
1980
1990
2000
2010
2020
Estimates Comparison
Bayesian estimates
GBD 2021 estimates
Data Series Name
Register (Register)
Data Type Name
Direct
Computed rate from DYB−NSO

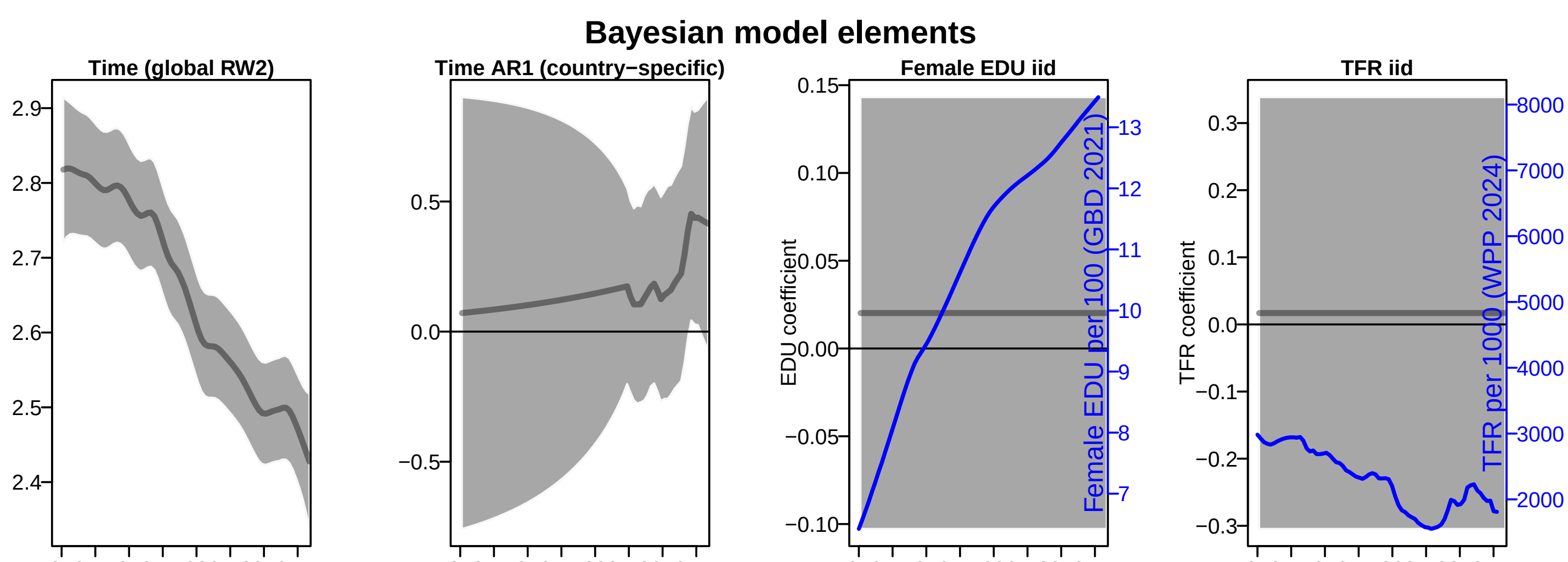

Bayesian model elements
Time (global RW2)
Time AR1 (country−specific)
Female EDU iid
TFR iid
EDU coefficient
Female EDU per 100 (GBD 2021)
TFR coefficient
TFR per 1000 (WPP 2024)
1950
1970
1990
2010

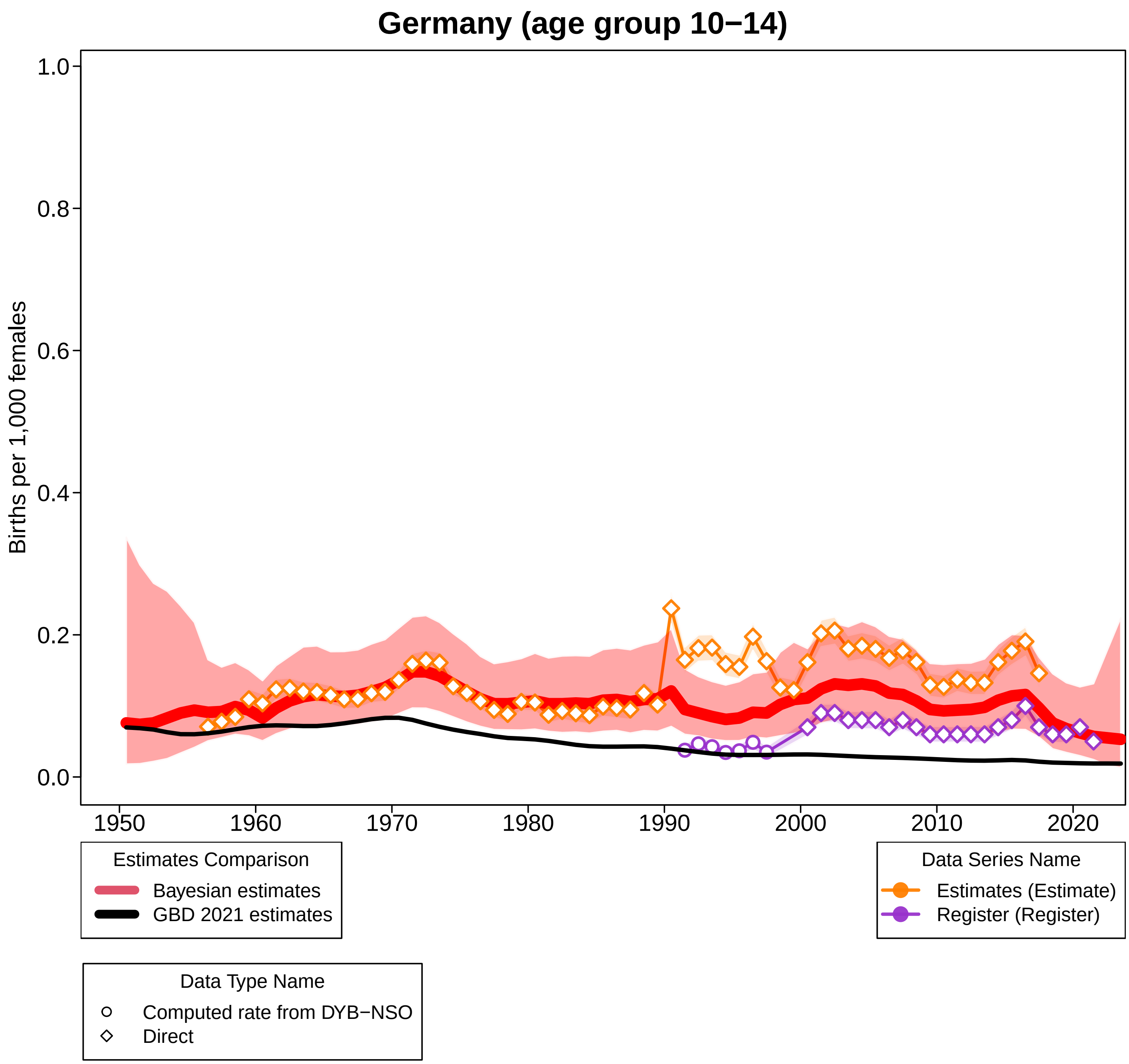
Germany (age group 10−14)
Births per 1,000 females
1.0
0.8
0.6
0.4
0.2
0.0
1950
1960
1970
1980
1990
2000
2010
2020
Estimates Comparison
Bayesian estimates
GBD 2021 estimates
Data Series Name
Estimates (Estimate)
Register (Register)
Data Type Name
Computed rate from DYB−NSO
Direct

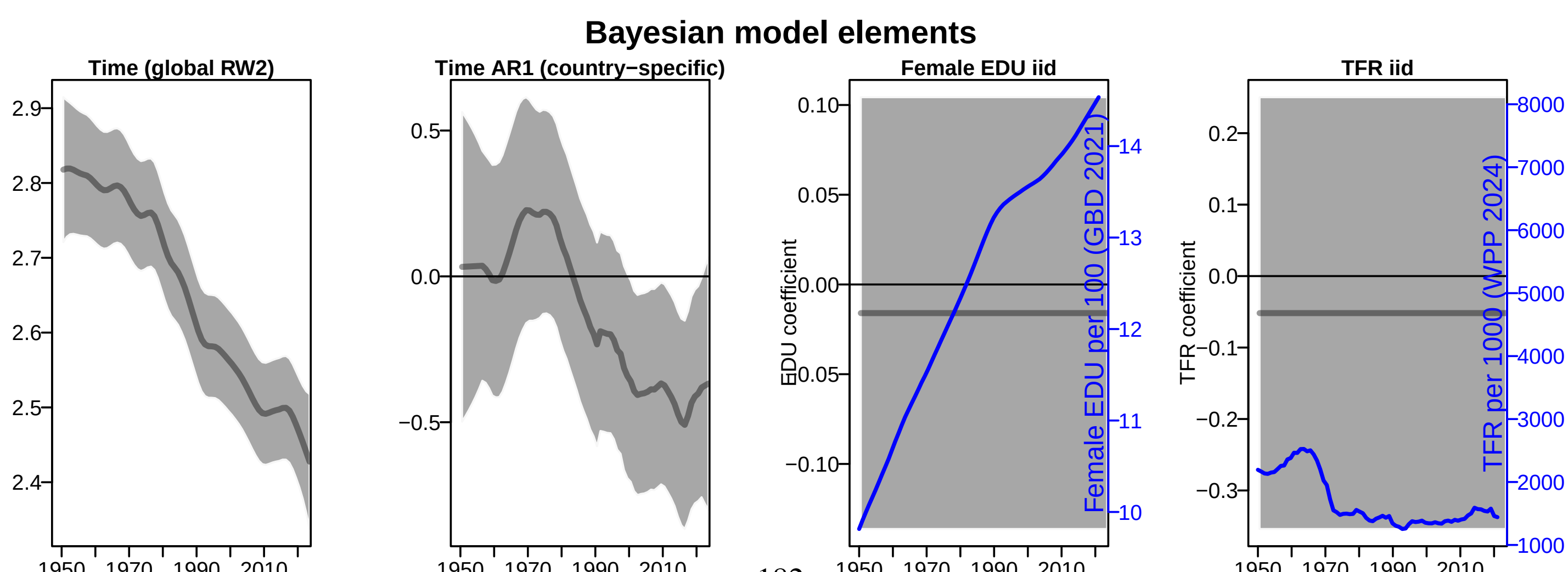
Bayesian model elements
Time (global RW2)
Time AR1 (country−specific)
Female EDU iid
TFR iid
EDU coefficient
Female EDU per 100 (GBD 2021)
TFR coefficient
TFR per 1000 (WPP 2024)

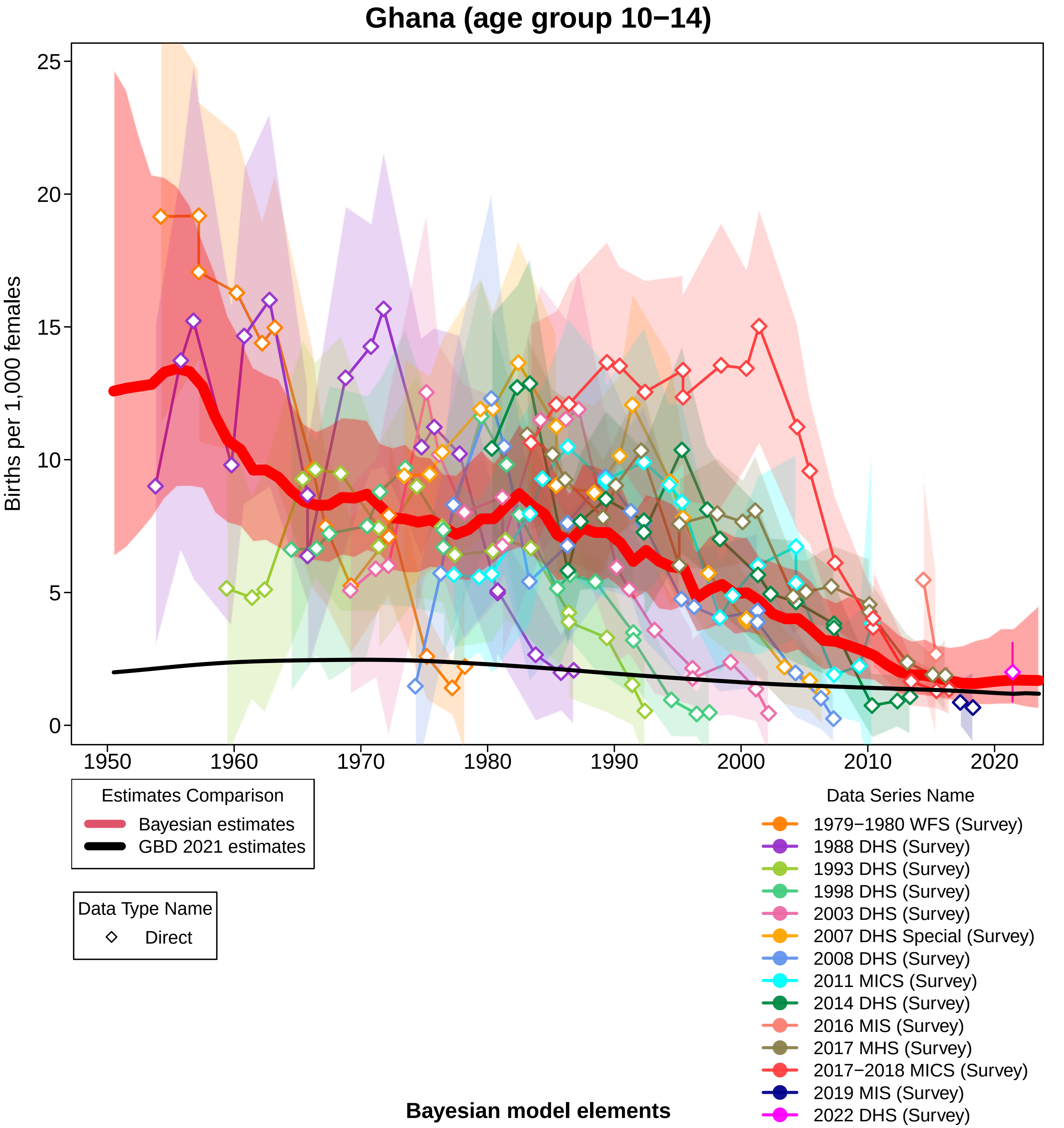
Ghana (age group 10−14)
Births per 1,000 females
0
5
10
15
20
25
1950
1960
1970
1980
1990
2000
2010
2020
Estimates Comparison
Bayesian estimates
GBD 2021 estimates
Data Type Name
Direct
Data Series Name
1979−1980 WFS (Survey)
1988 DHS (Survey)
1993 DHS (Survey)
1998 DHS (Survey)
2003 DHS (Survey)
2007 DHS Special (Survey)
2008 DHS (Survey)
2011 MICS (Survey)
2014 DHS (Survey)
2016 MIS (Survey)
2017 MHS (Survey)
2017−2018 MICS (Survey)
2019 MIS (Survey)
2022 DHS (Survey)

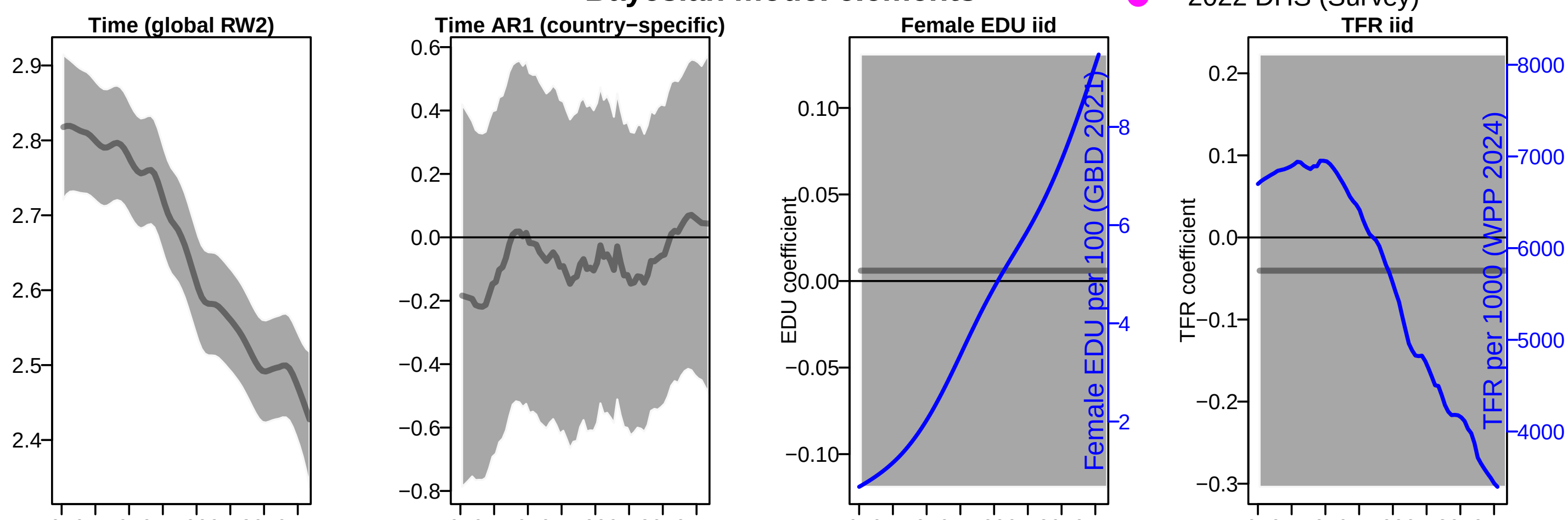
Bayesian model elements
Time (global RW2)
Time AR1 (country−specific)
Female EDU iid
TFR iid
EDU coefficient
Female EDU per 100 (GBD 2021)
TFR coefficient
TFR per 1000 (WPP 2024)
1950
1970
1990
2010

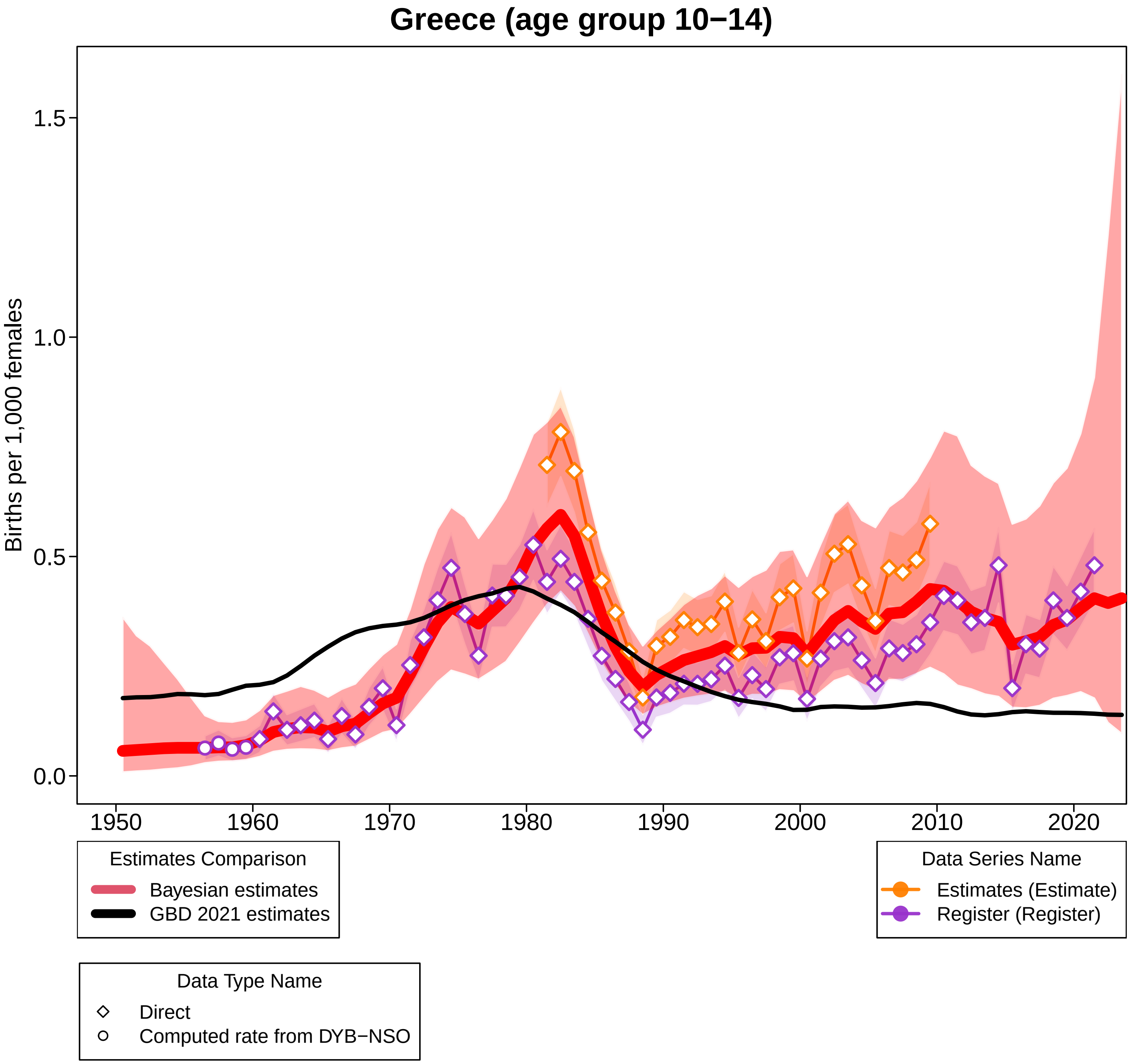
Greece (age group 10−14)
Births per 1,000 females
0.0
0.5
1.0
1.5
1950
1960
1970
1980
1990
2000
2010
2020
Estimates Comparison
Bayesian estimates
GBD 2021 estimates
Data Series Name
Estimates (Estimate)
Register (Register)
Data Type Name
Direct
Computed rate from DYB−NSO

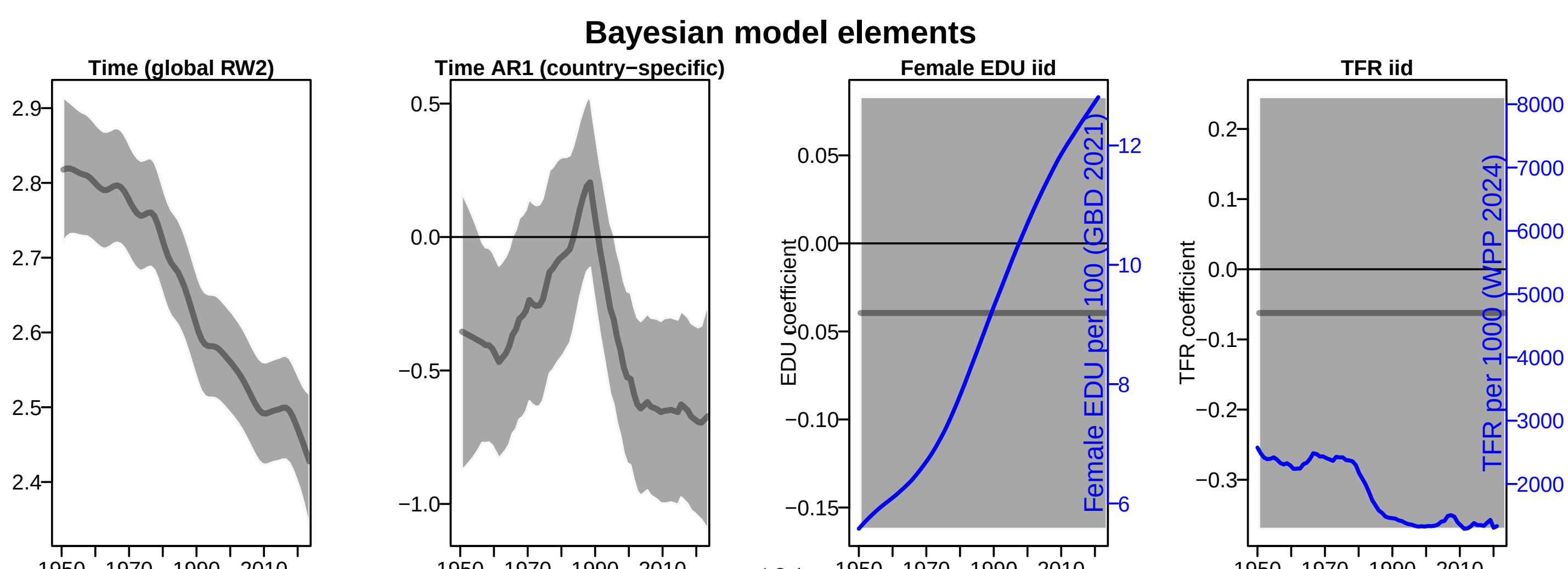
Bayesian model elements
Time (global RW2)
Time AR1 (country−specific)
Female EDU iid
TFR iid
EDU coefficient
Female EDU per 100 (GBD 2021)
TFR coefficient
TFR per 1000 (WPP 2024)
1950
1970
1990
2010

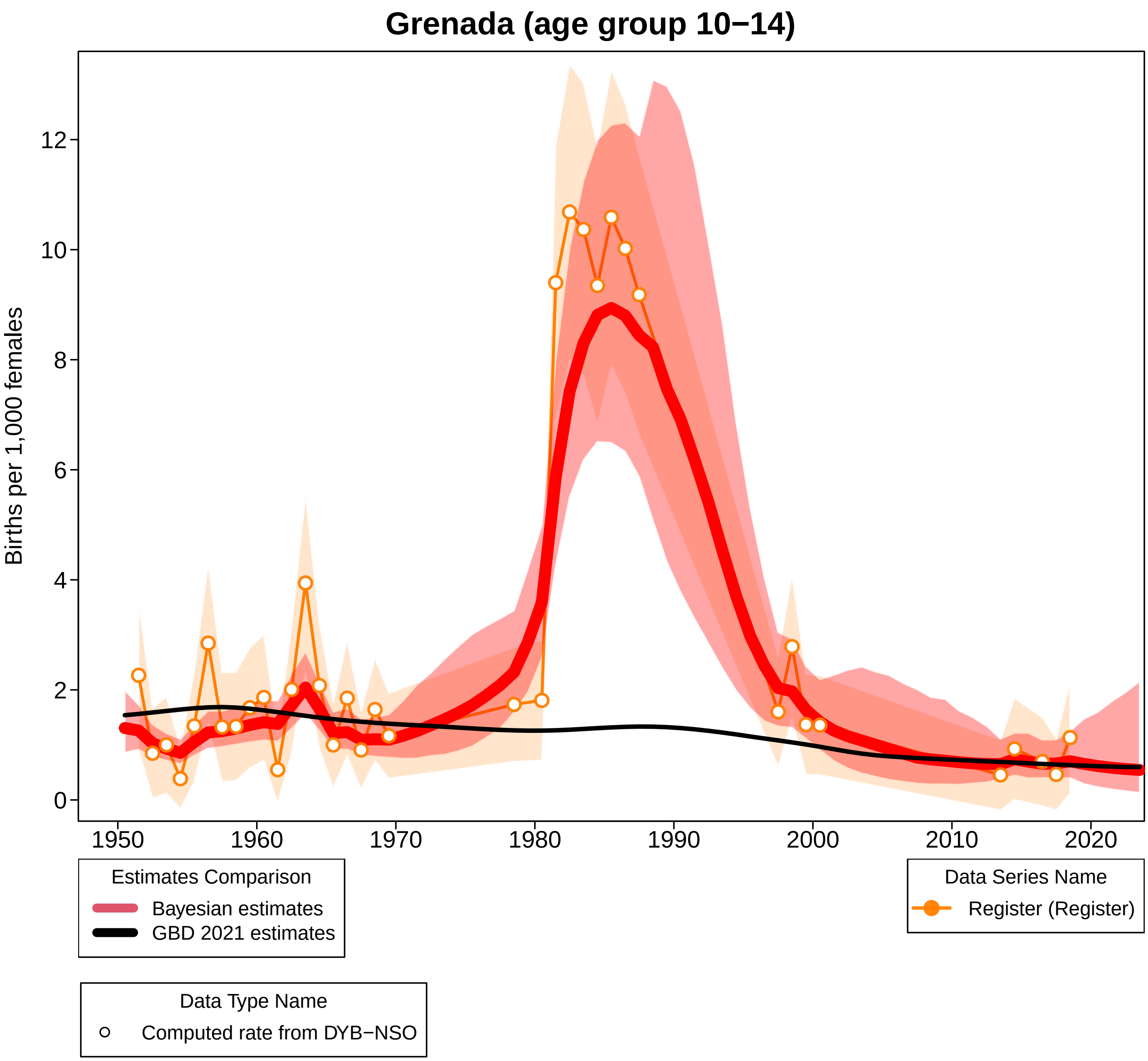

Grenada (age group 10−14)
Births per 1,000 females
0
2
4
6
8
10
12
1950
1960
1970
1980
1990
2000
2010
2020
Estimates Comparison
Bayesian estimates
GBD 2021 estimates
Data Series Name
Register (Register)
Data Type Name
Computed rate from DYB−NSO

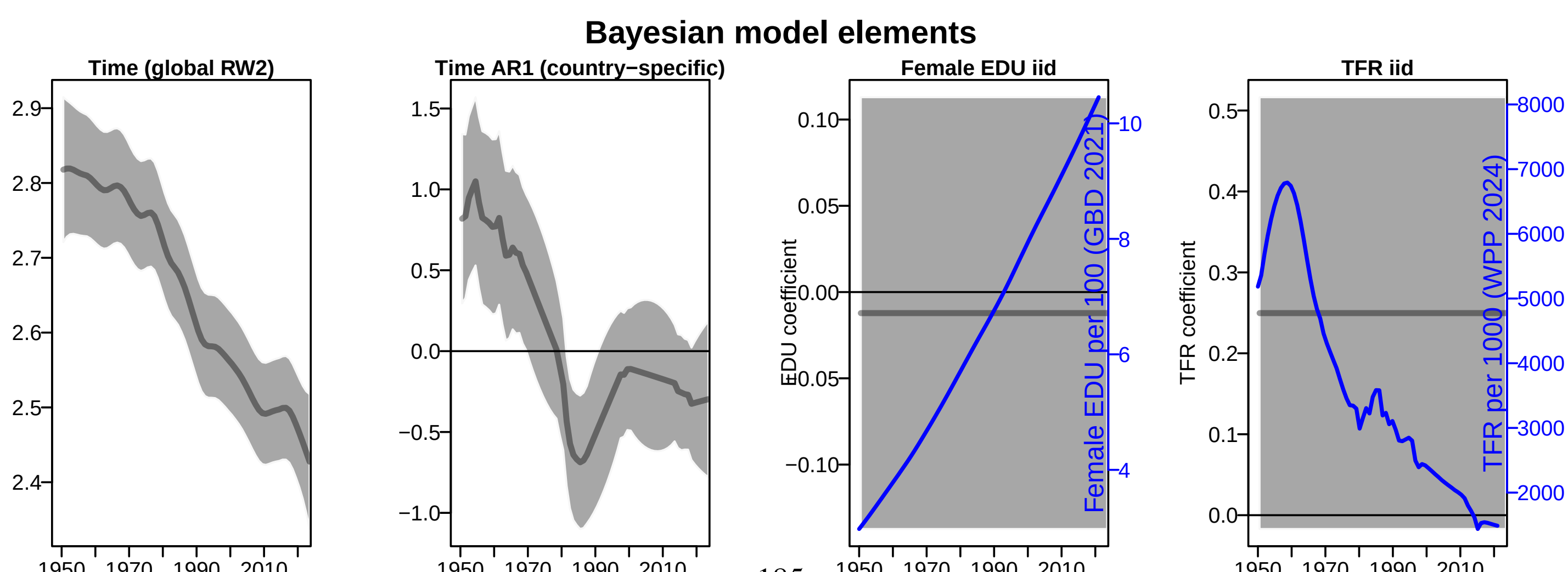

Bayesian model elements
Time (global RW2)
Time AR1 (country−specific)
Female EDU iid
TFR iid
EDU coefficient
Female EDU per 100 (GBD 2021)
TFR coefficient
TFR per 1000 (WPP 2024)
1950
1970
1990
2010

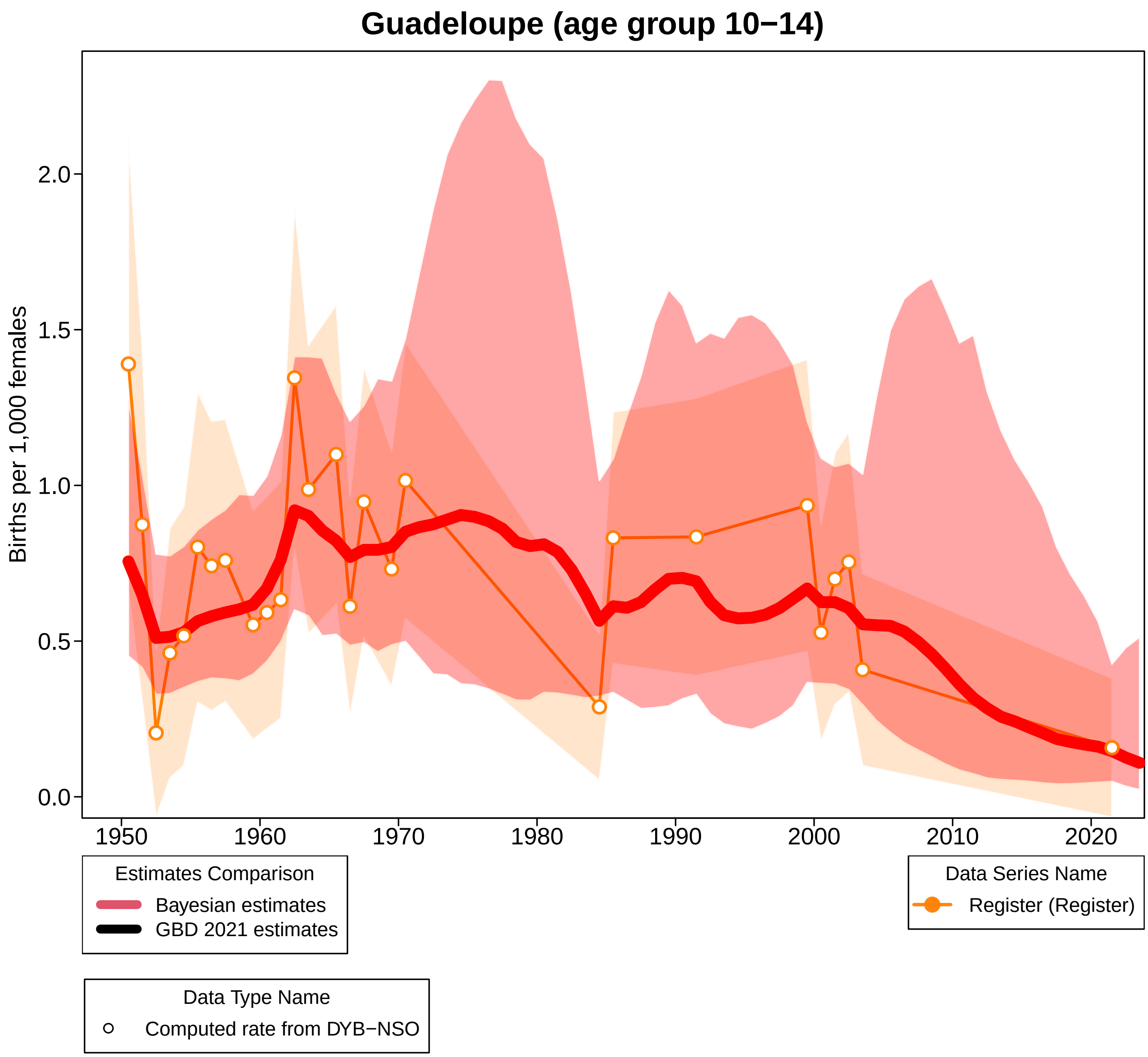
Guadeloupe (age group 10−14)
Births per 1,000 females
0.0
0.5
1.0
1.5
2.0
1950
1960
1970
1980
1990
2000
2010
2020
Estimates Comparison
Bayesian estimates
GBD 2021 estimates
Data Series Name
Register (Register)
Data Type Name
Computed rate from DYB−NSO

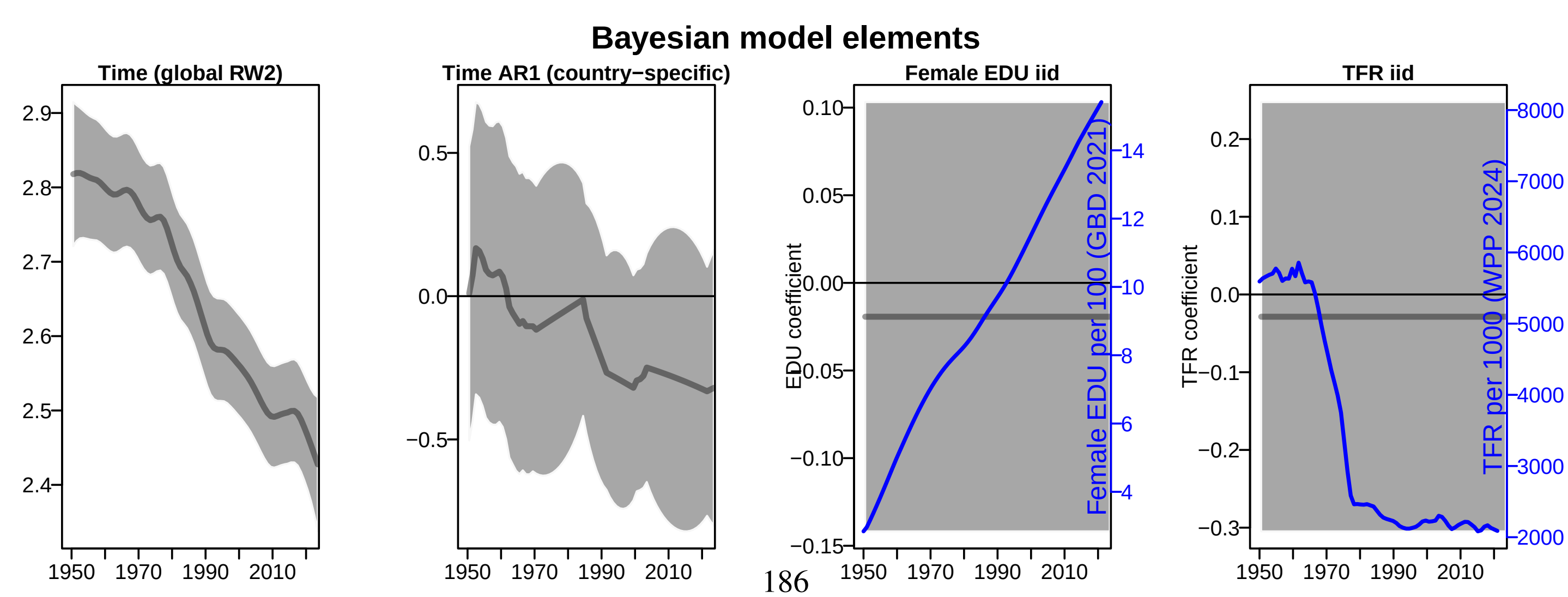
Bayesian model elements
Time (global RW2)
Time AR1 (country−specific)
Female EDU iid
TFR iid
EDU coefficient
Female EDU per 100 (GBD 2021)
TFR coefficient
TFR per 1000 (WPP 2024)
1950
1970
1990
2010

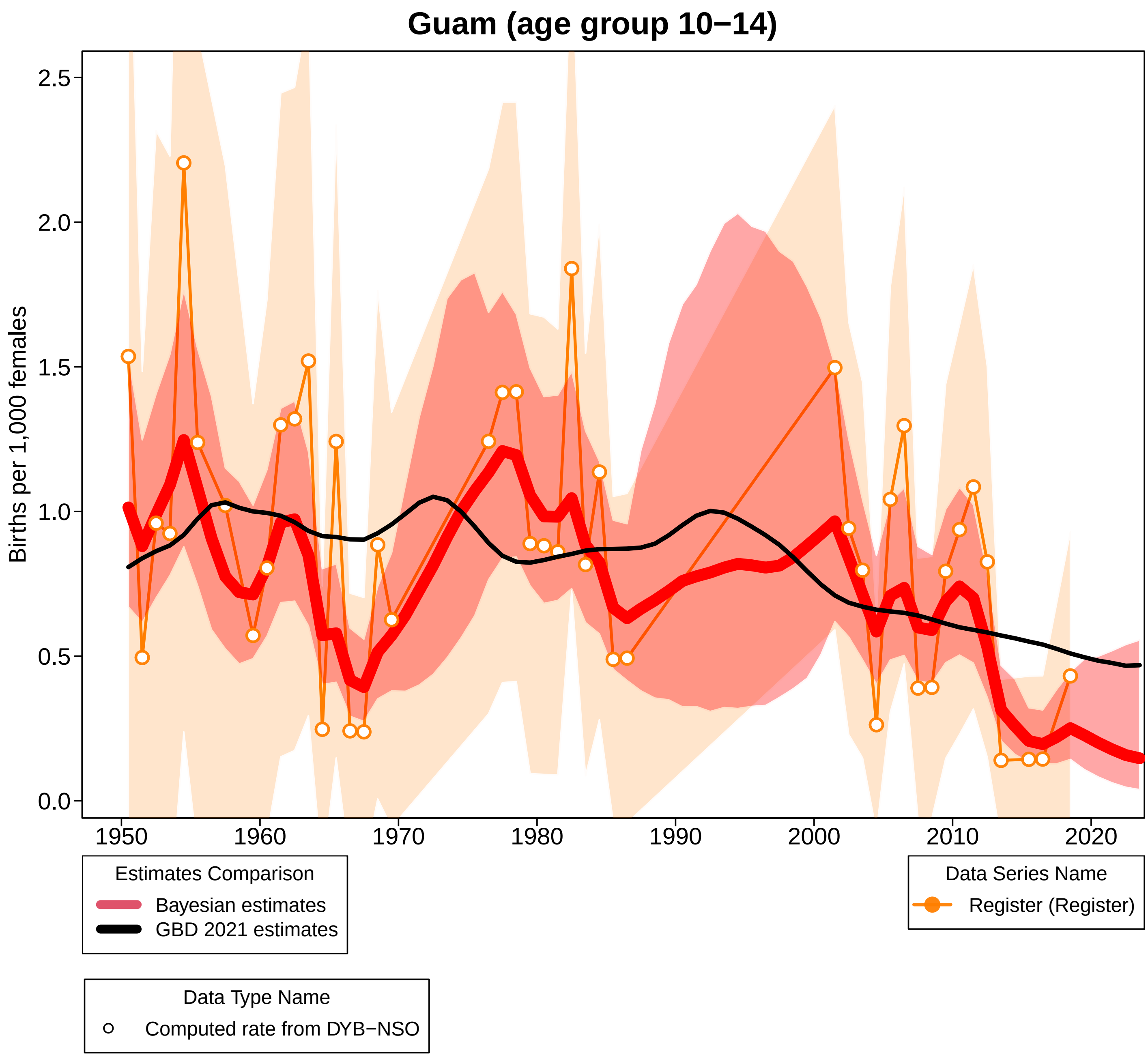
Guam (age group 10−14)
Births per 1,000 females
Estimates Comparison
Bayesian estimates
GBD 2021 estimates
Data Series Name
Register (Register)
Data Type Name
Computed rate from DYB−NSO

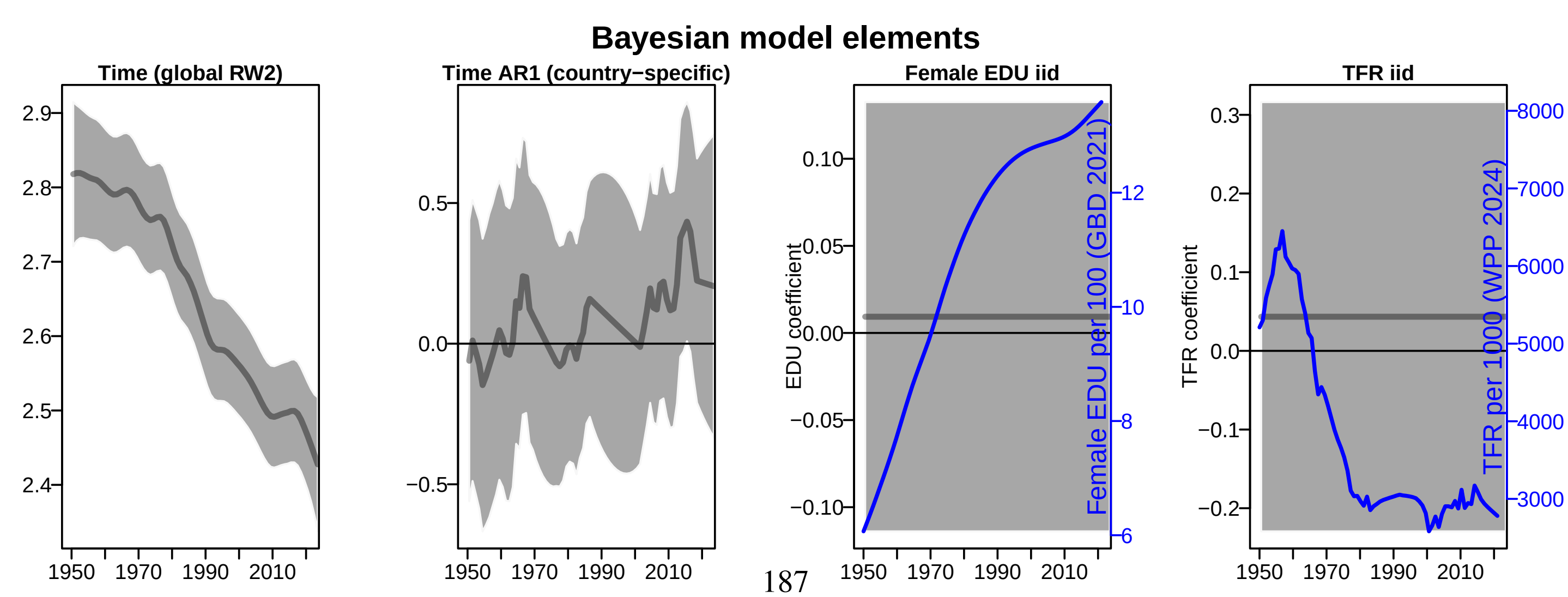
Bayesian model elements
Time (global RW2)
Time AR1 (country−specific)
Female EDU iid
EDU coefficient
Female EDU per 100 (GBD 2021)
TFR iid
TFR coefficient
TFR per 1000 (WPP 2024)

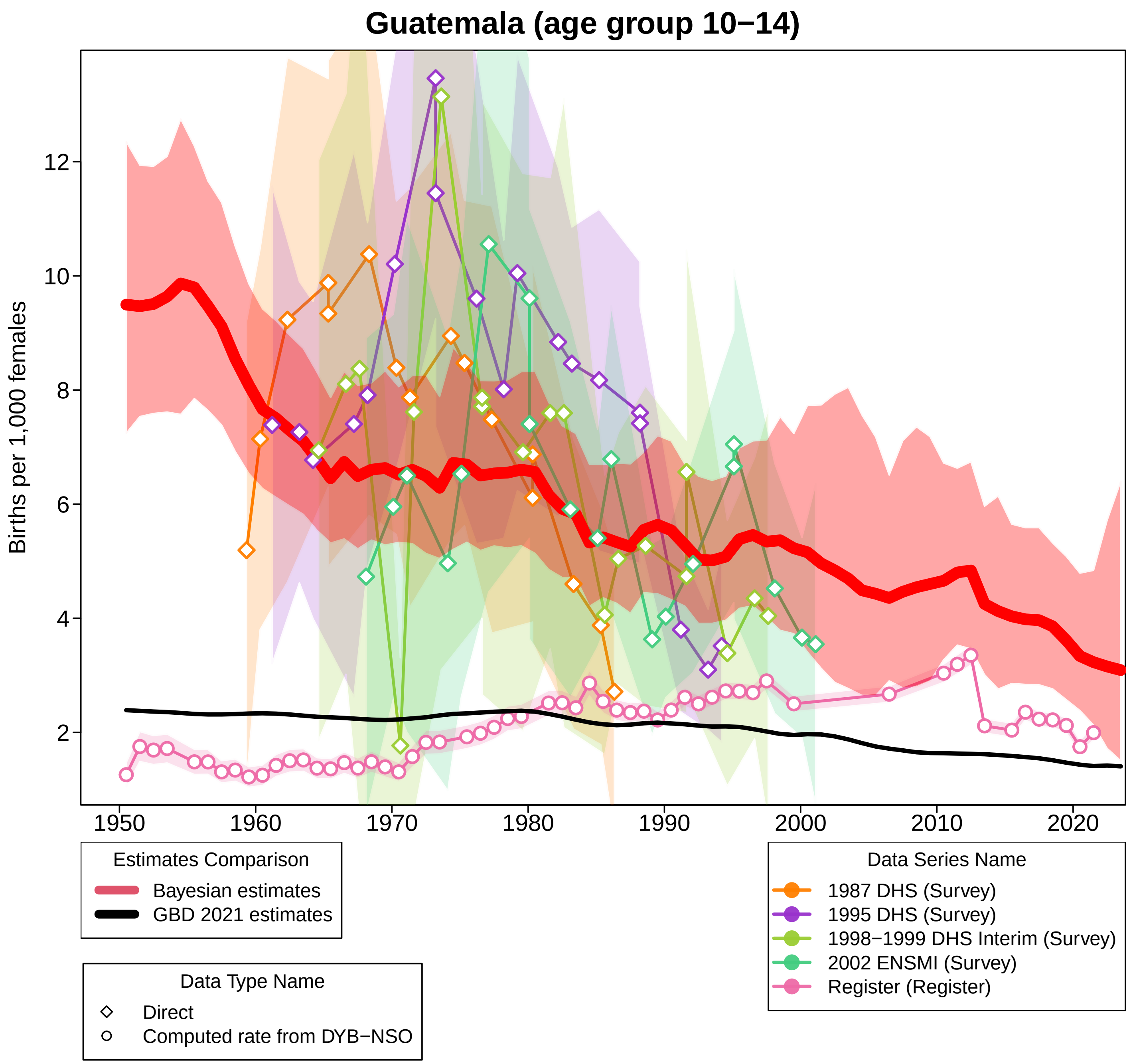

Guatemala (age group 10−14)
Births per 1,000 females
12
10
8
6
4
2
1950
1960
1970
1980
1990
2000
2010
2020
Estimates Comparison
Bayesian estimates
GBD 2021 estimates
Data Series Name
1987 DHS (Survey)
1995 DHS (Survey)
1998−1999 DHS Interim (Survey)
2002 ENSMI (Survey)
Register (Register)
Data Type Name
Direct
Computed rate from DYB−NSO

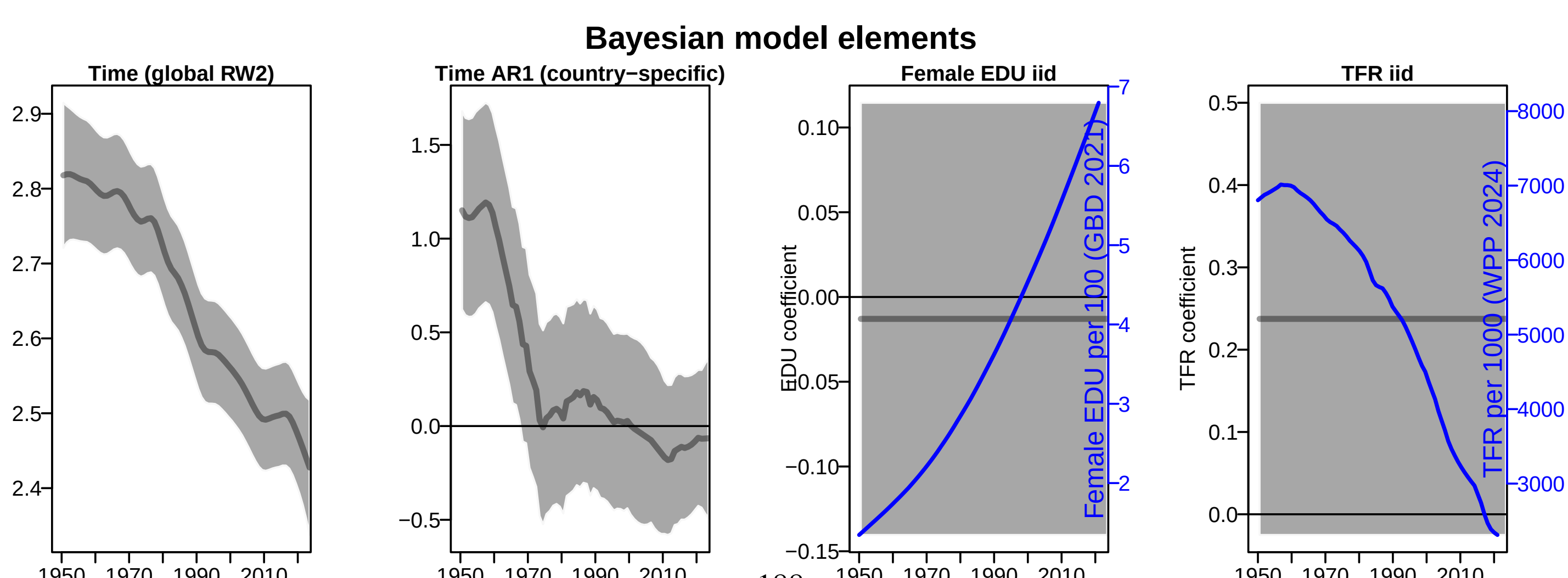

Bayesian model elements
Time (global RW2)
Time AR1 (country−specific)
Female EDU iid
TFR iid
EDU coefficient
Female EDU per 100 (GBD 2021)
TFR coefficient
TFR per 1000 (WPP 2024)
1950
1970
1990
2010

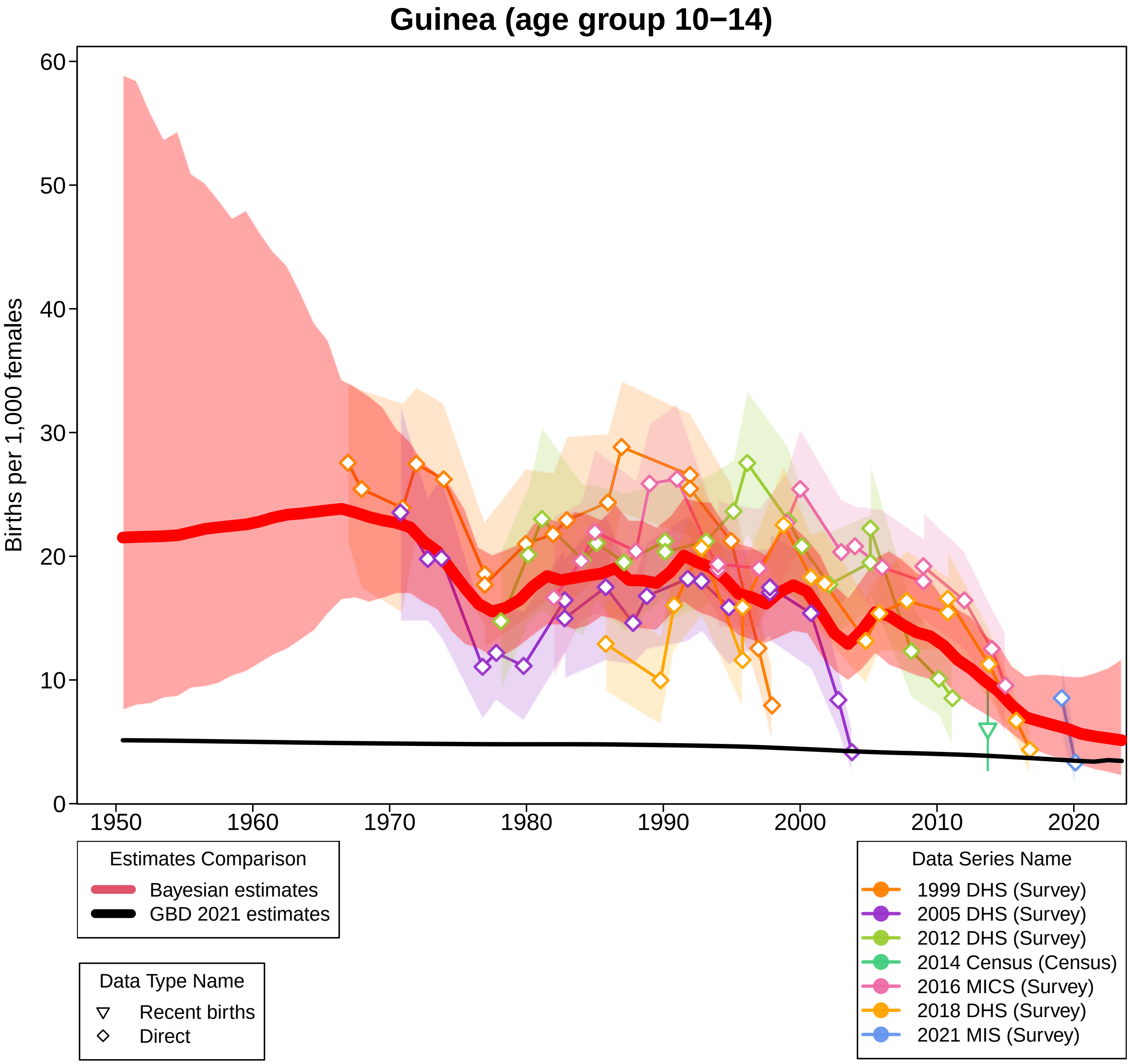
Guinea (age group 10−14)
Births per 1,000 females
0
10
20
30
40
50
60
1950
1960
1970
1980
1990
2000
2010
2020
Estimates Comparison
Bayesian estimates
GBD 2021 estimates
Data Type Name
Recent births
Direct
Data Series Name
1999 DHS (Survey)
2005 DHS (Survey)
2012 DHS (Survey)
2014 Census (Census)
2016 MICS (Survey)
2018 DHS (Survey)
2021 MIS (Survey)

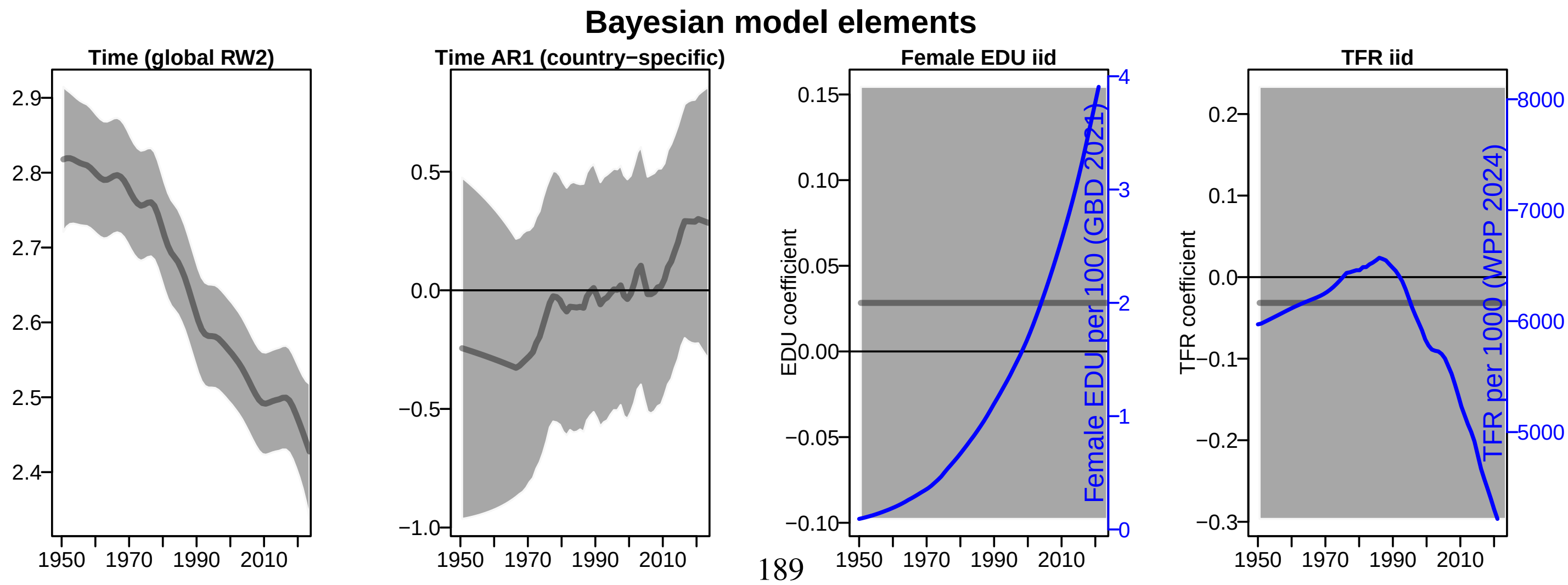
Bayesian model elements
Time (global RW2)
Time AR1 (country−specific)
Female EDU iid
TFR iid
EDU coefficient
Female EDU per 100 (GBD 2021)
TFR coefficient
TFR per 1000 (WPP 2024)
1950
1970
1990
2010

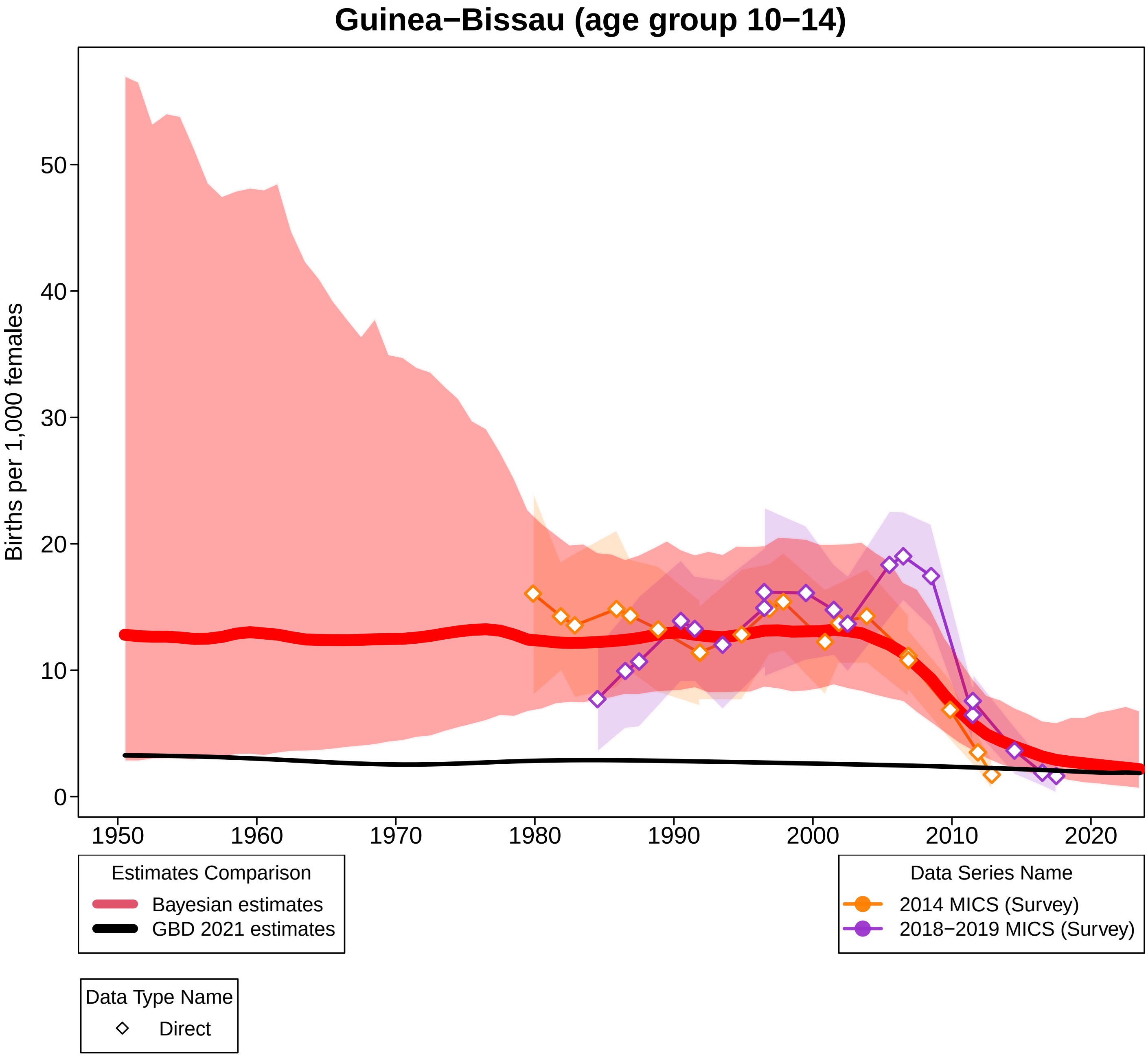

Guinea−Bissau (age group 10−14)
Births per 1,000 females
0
10
20
30
40
50
1950
1960
1970
1980
1990
2000
2010
2020
Estimates Comparison
Bayesian estimates
GBD 2021 estimates
Data Series Name
2014 MICS (Survey)
2018−2019 MICS (Survey)
Data Type Name
Direct

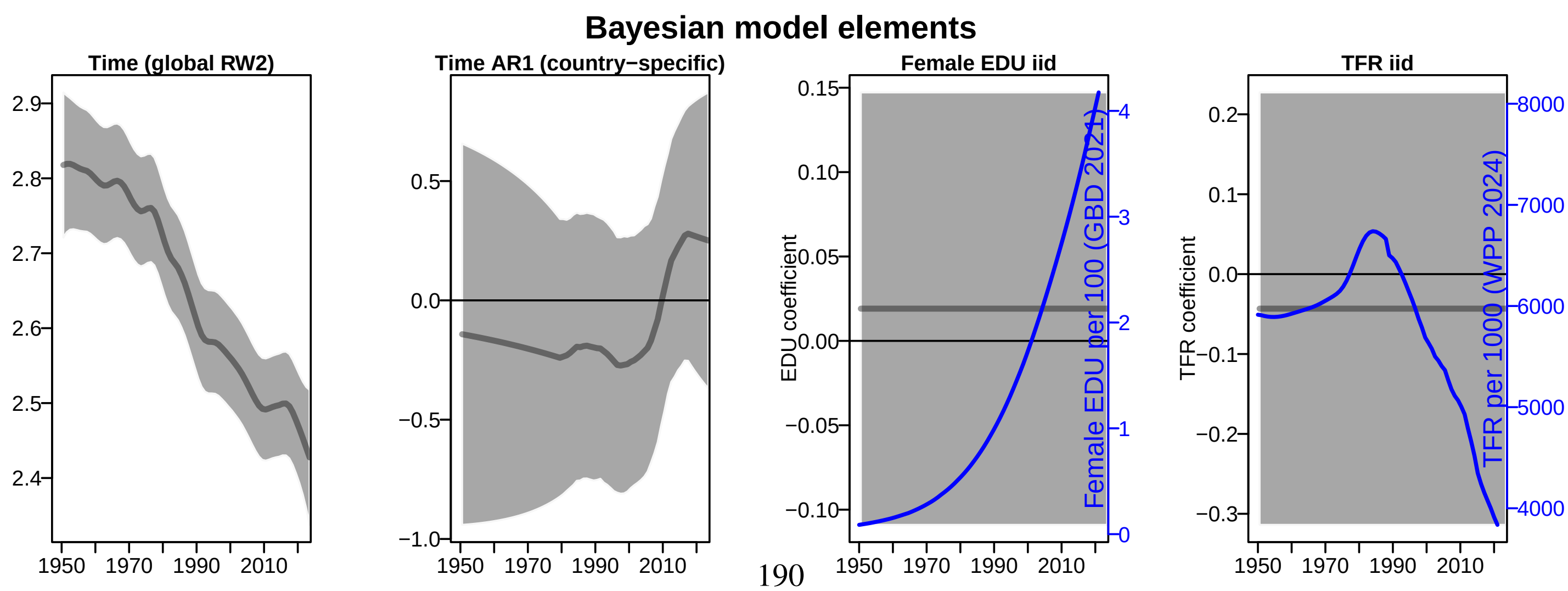

Bayesian model elements
Time (global RW2)
Time AR1 (country−specific)
Female EDU iid
TFR iid
EDU coefficient
Female EDU per 100 (GBD 2021)
TFR coefficient
TFR per 1000 (WPP 2024)
1950
1970
1990
2010

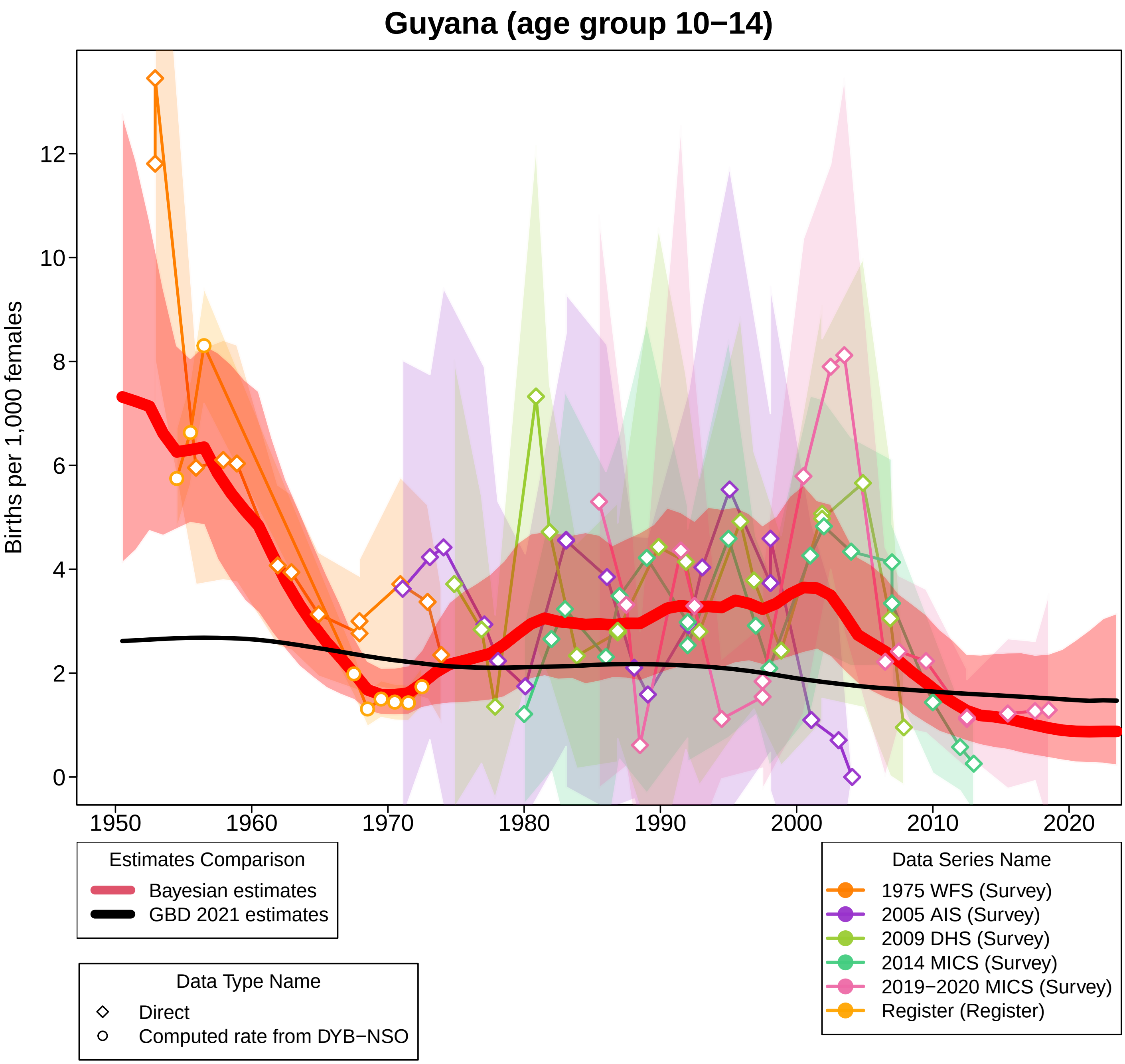
Guyana (age group 10−14)
Births per 1,000 females
0
2
4
6
8
10
12
1950
1960
1970
1980
1990
2000
2010
2020
Estimates Comparison
Bayesian estimates
GBD 2021 estimates
Data Type Name
Direct
Computed rate from DYB−NSO
Data Series Name
1975 WFS (Survey)
2005 AIS (Survey)
2009 DHS (Survey)
2014 MICS (Survey)
2019−2020 MICS (Survey)
Register (Register)

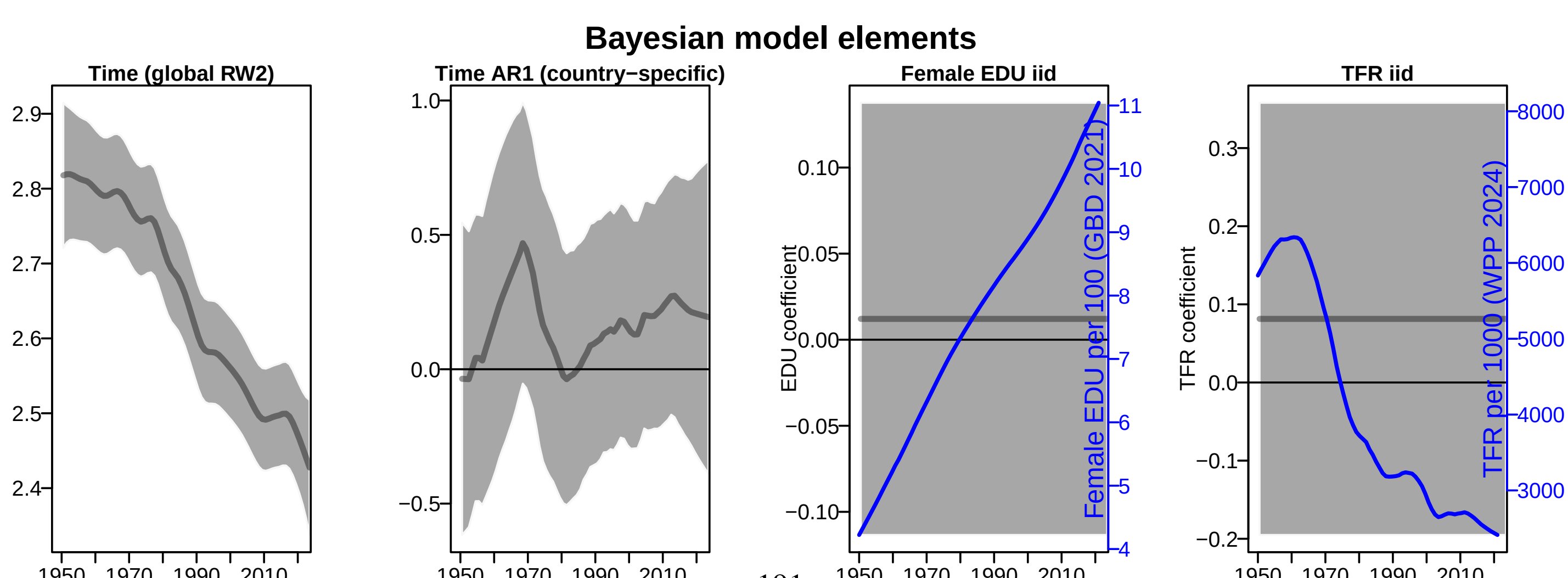
Bayesian model elements
Time (global RW2)
Time AR1 (country−specific)
Female EDU iid
TFR iid
EDU coefficient
Female EDU per 100 (GBD 2021)
TFR coefficient
TFR per 1000 (WPP 2024)
1950
1970
1990
2010

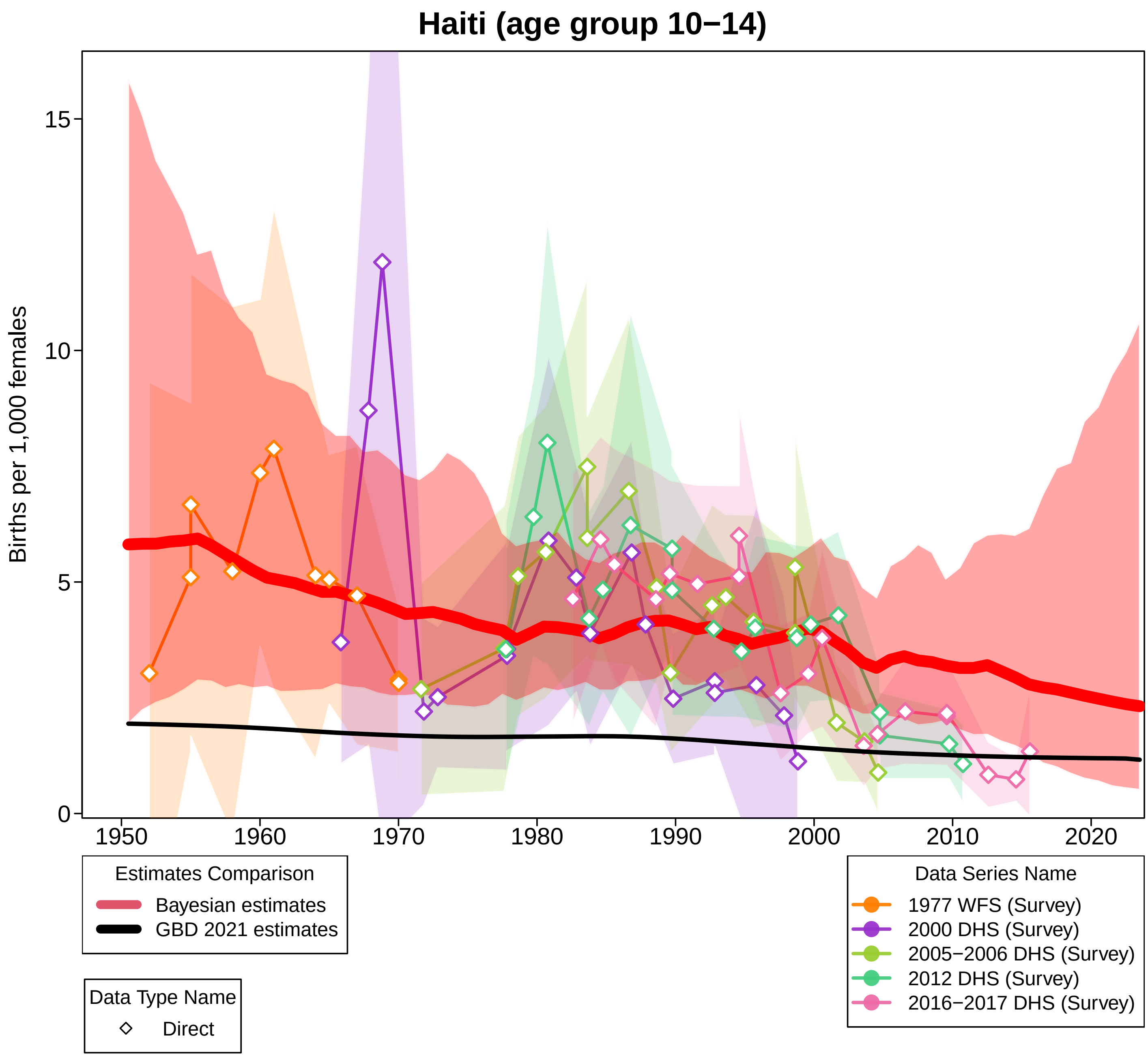
Haiti (age group 10−14)
Births per 1,000 females
0
5
10
15
1950
1960
1970
1980
1990
2000
2010
2020
Estimates Comparison
Bayesian estimates
GBD 2021 estimates
Data Type Name
Direct
Data Series Name
1977 WFS (Survey)
2000 DHS (Survey)
2005−2006 DHS (Survey)
2012 DHS (Survey)
2016−2017 DHS (Survey)

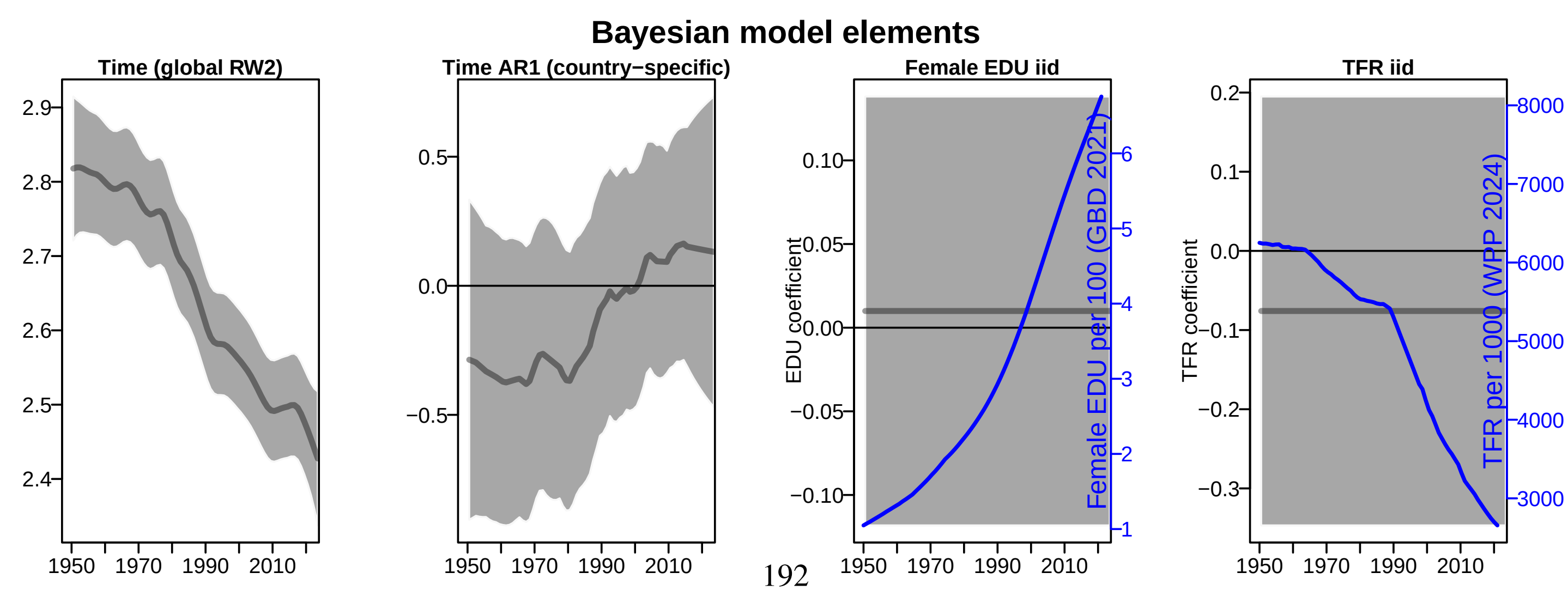
Bayesian model elements
Time (global RW2)
Time AR1 (country−specific)
Female EDU iid
TFR iid
EDU coefficient
Female EDU per 100 (GBD 2021)
TFR coefficient
TFR per 1000 (WPP 2024)
1950
1970
1990
2010

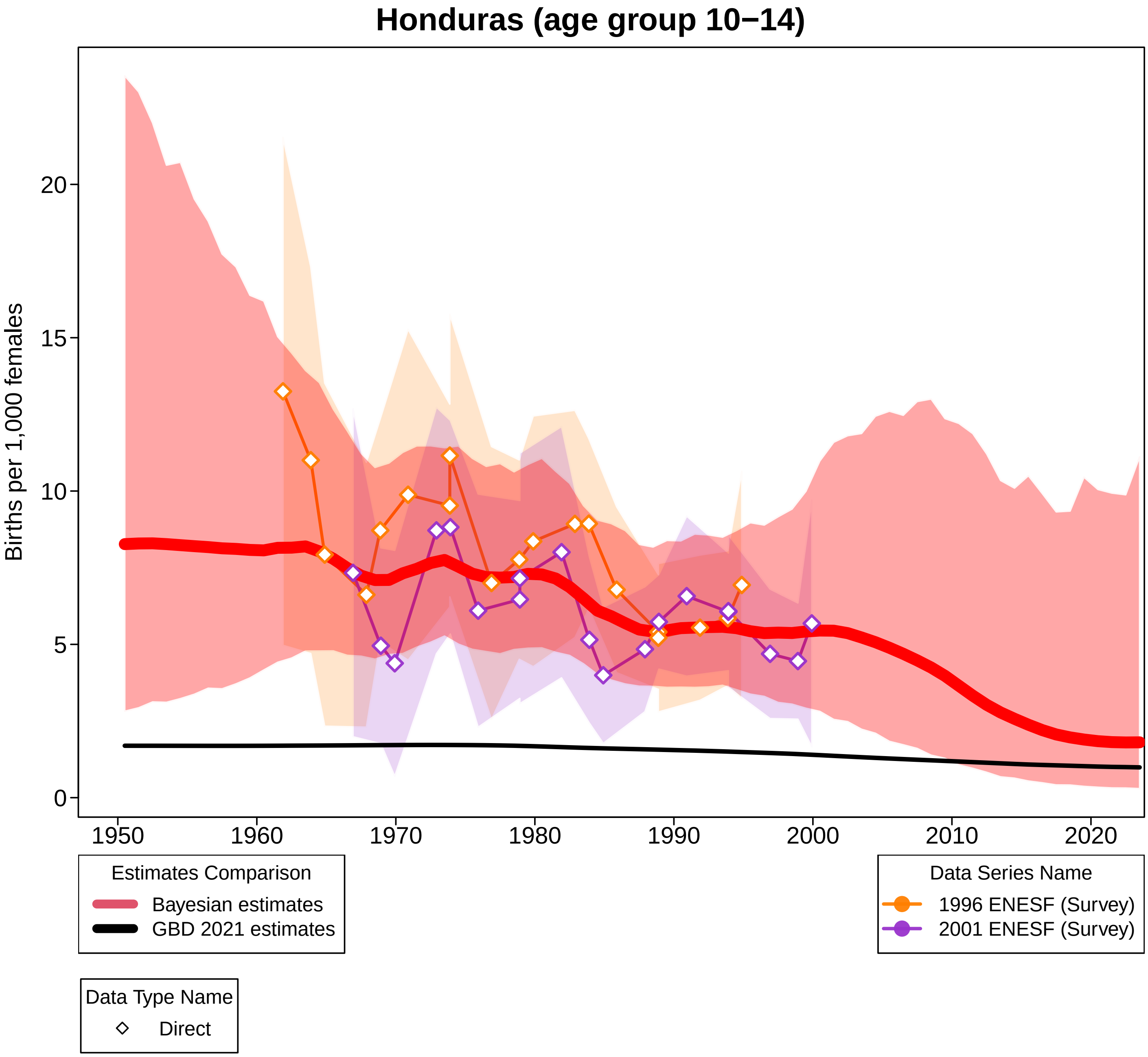
Honduras (age group 10−14)
Births per 1,000 females
Estimates Comparison
Bayesian estimates
GBD 2021 estimates
Data Series Name
1996 ENESF (Survey)
2001 ENESF (Survey)
Data Type Name
Direct

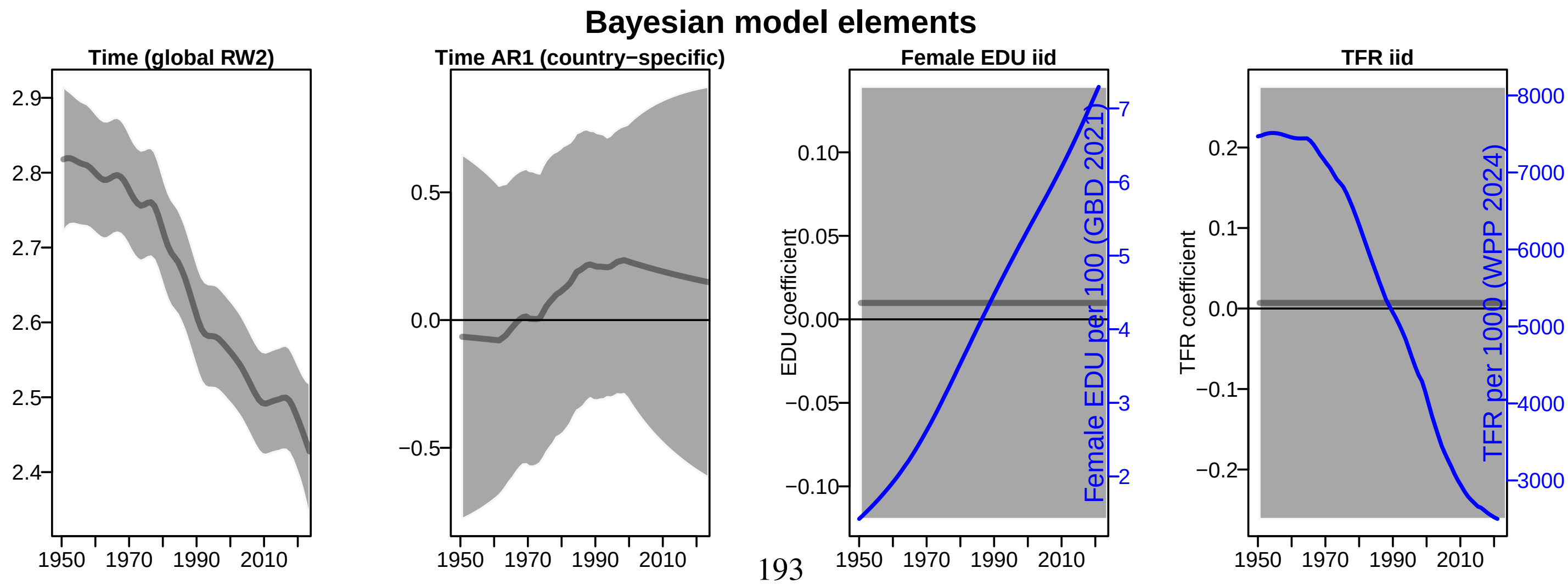
Bayesian model elements
Time (global RW2)
Time AR1 (country−specific)
Female EDU iid
TFR iid
EDU coefficient
Female EDU per 100 (GBD 2021)
TFR coefficient
TFR per 1000 (WPP 2024)

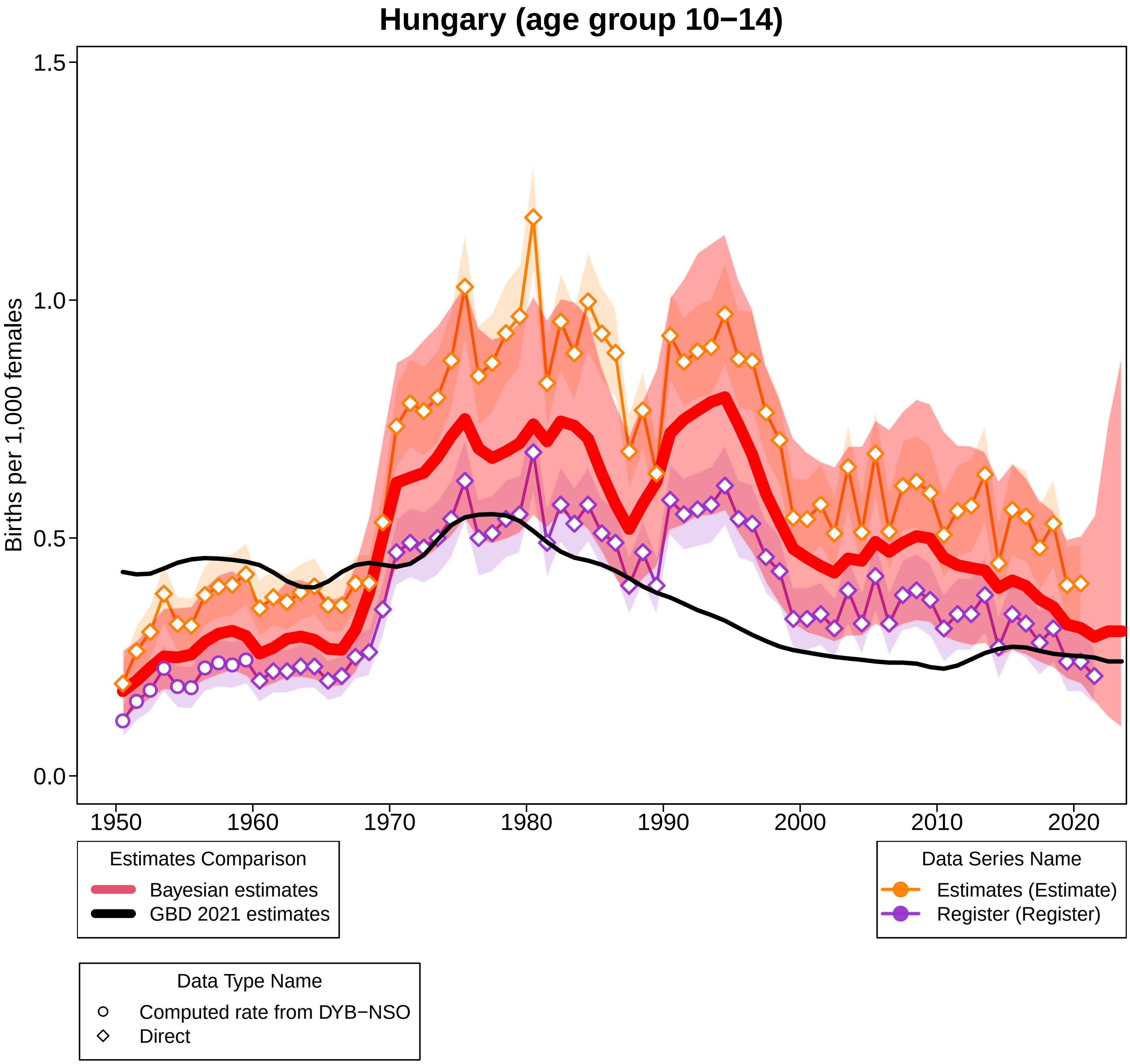

Hungary (age group 10−14)
Births per 1,000 females
1.5
1.0
0.5
0.0
1950
1960
1970
1980
1990
2000
2010
2020
Estimates Comparison
Bayesian estimates
GBD 2021 estimates
Data Series Name
Estimates (Estimate)
Register (Register)
Data Type Name
Computed rate from DYB−NSO
Direct

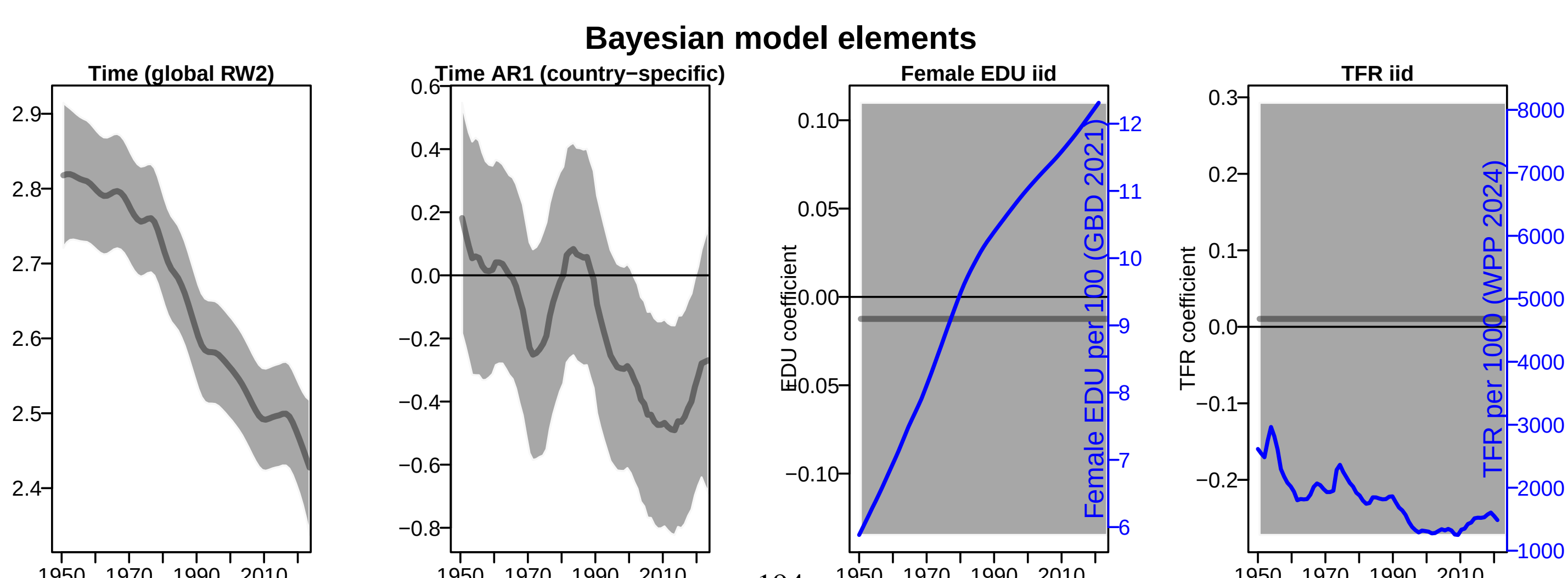

Bayesian model elements
Time (global RW2)
Time AR1 (country−specific)
Female EDU iid
TFR iid
EDU coefficient
Female EDU per 100 (GBD 2021)
TFR coefficient
TFR per 1000 (WPP 2024)
1950
1970
1990
2010

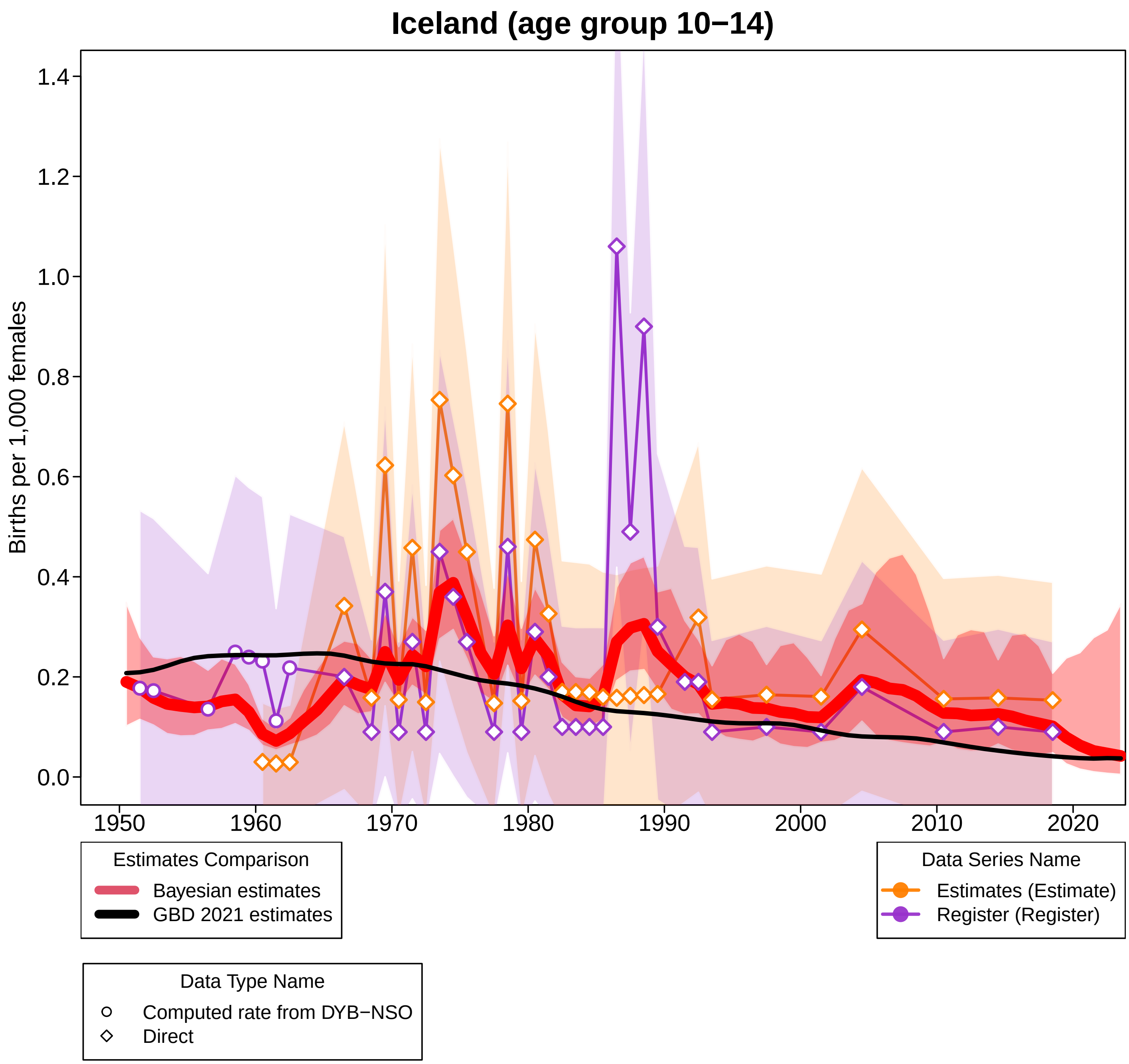
Iceland (age group 10−14)
Births per 1,000 females
1.4
1.2
1.0
0.8
0.6
0.4
0.2
0.0
1950
1960
1970
1980
1990
2000
2010
2020
Estimates Comparison
Bayesian estimates
GBD 2021 estimates
Data Series Name
Estimates (Estimate)
Register (Register)
Data Type Name
Computed rate from DYB−NSO
Direct

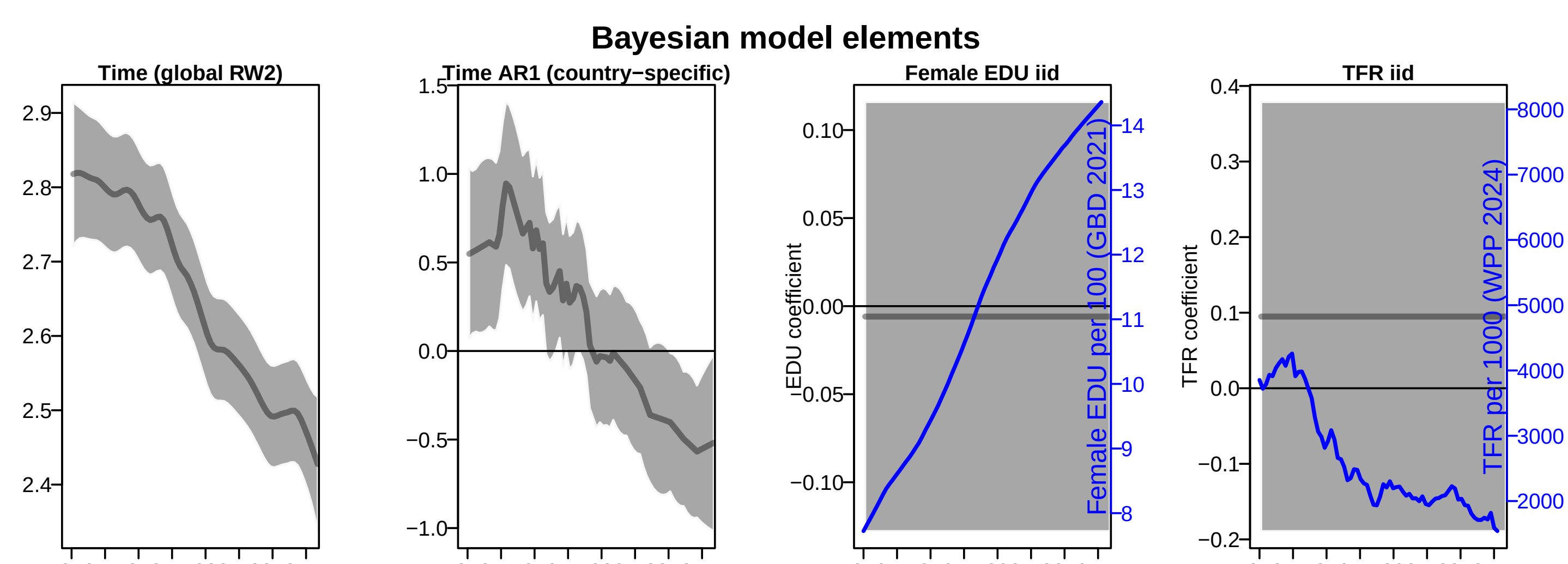
Bayesian model elements
Time (global RW2)
Time AR1 (country−specific)
Female EDU iid
TFR iid
EDU coefficient
Female EDU per 100 (GBD 2021)
TFR coefficient
TFR per 1000 (WPP 2024)
1950
1970
1990
2010

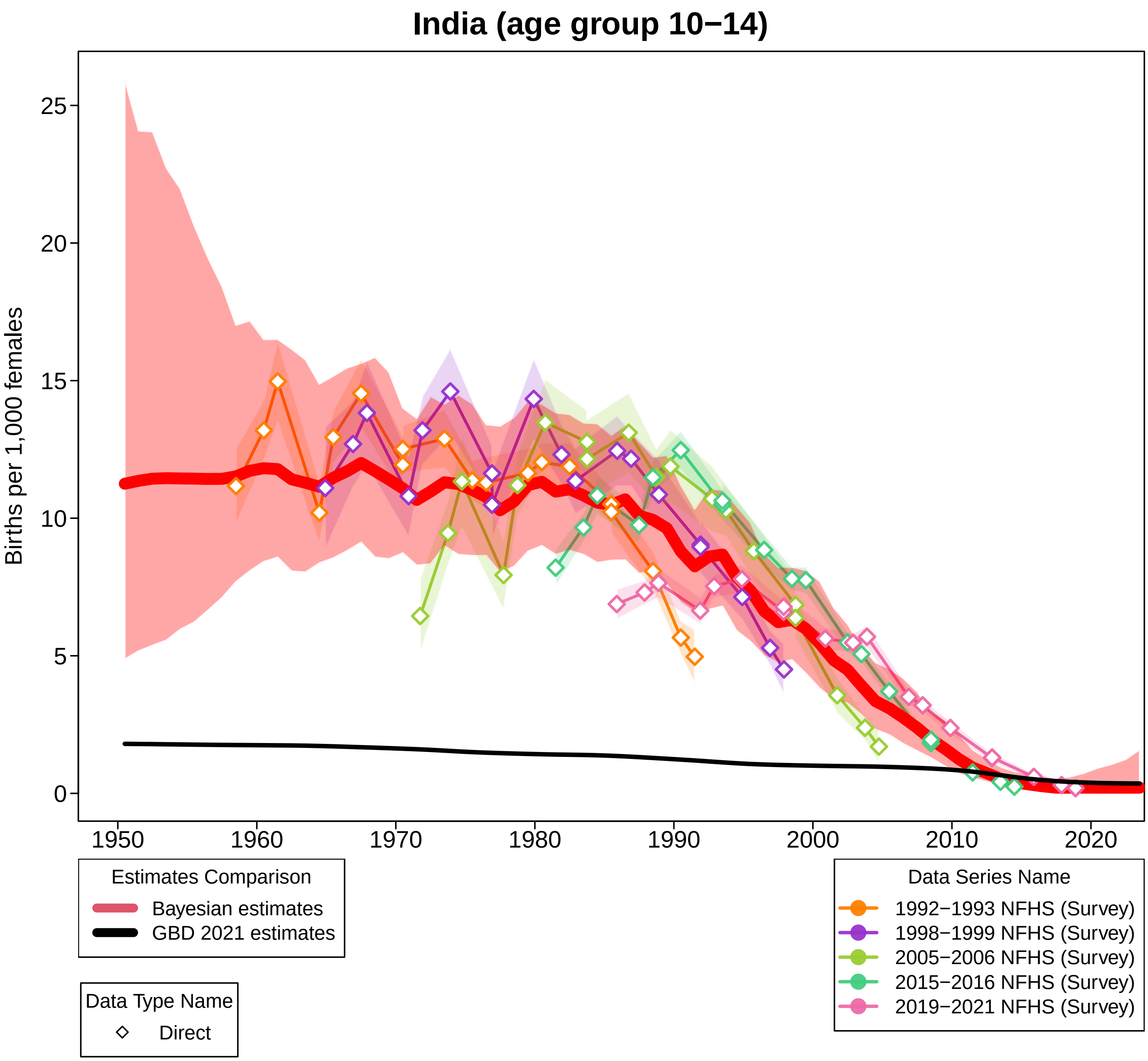
India (age group 10−14)
Births per 1,000 females
25
20
15
10
5
0
1950
1960
1970
1980
1990
2000
2010
2020
Estimates Comparison
Bayesian estimates
GBD 2021 estimates
Data Type Name
Direct
Data Series Name
1992−1993 NFHS (Survey)
1998−1999 NFHS (Survey)
2005−2006 NFHS (Survey)
2015−2016 NFHS (Survey)
2019−2021 NFHS (Survey)

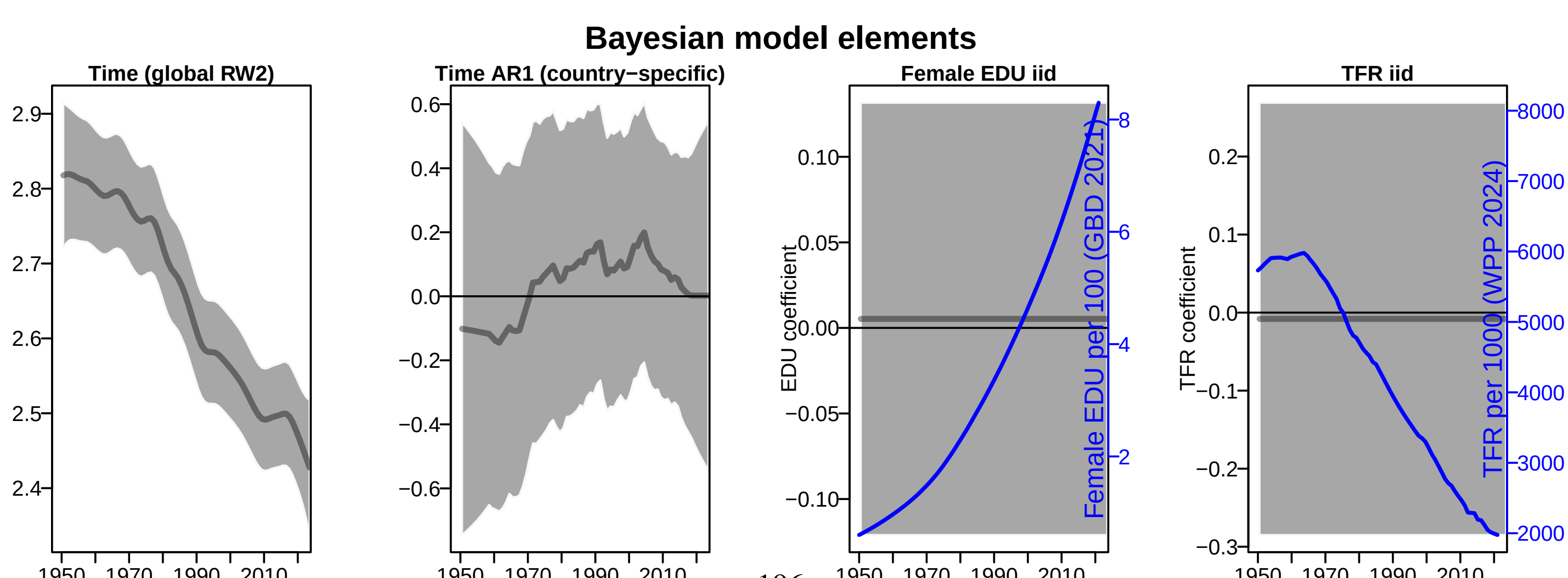
Bayesian model elements
Time (global RW2)
Time AR1 (country−specific)
Female EDU iid
TFR iid
EDU coefficient
Female EDU per 100 (GBD 2021)
TFR coefficient
TFR per 1000 (WPP 2024)
1950
1970
1990
2010

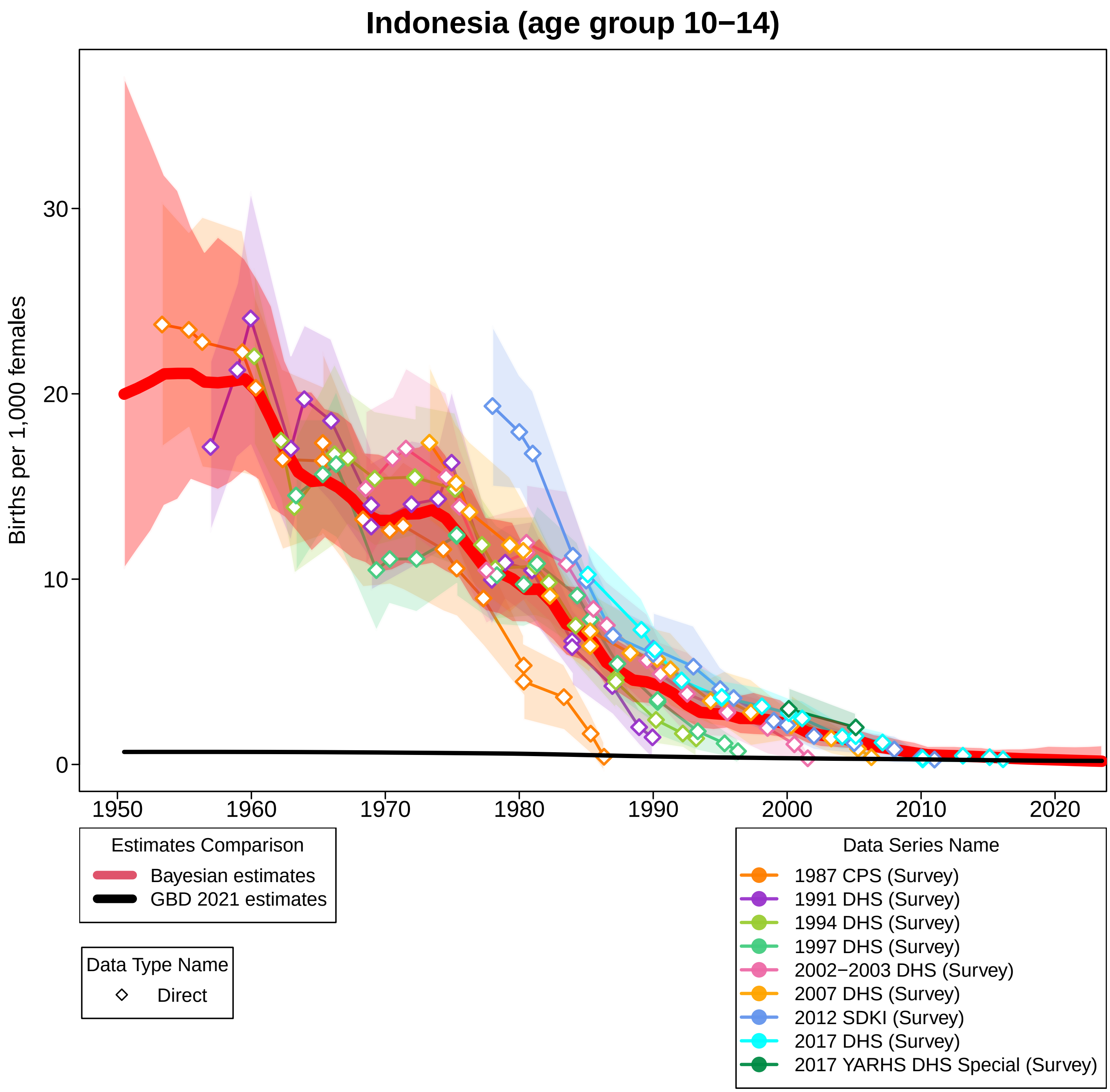
Indonesia (age group 10−14)
Births per 1,000 females
0
10
20
30
1950
1960
1970
1980
1990
2000
2010
2020
Estimates Comparison
Bayesian estimates
GBD 2021 estimates
Data Type Name
Direct
Data Series Name
1987 CPS (Survey)
1991 DHS (Survey)
1994 DHS (Survey)
1997 DHS (Survey)
2002−2003 DHS (Survey)
2007 DHS (Survey)
2012 SDKI (Survey)
2017 DHS (Survey)
2017 YARHS DHS Special (Survey)

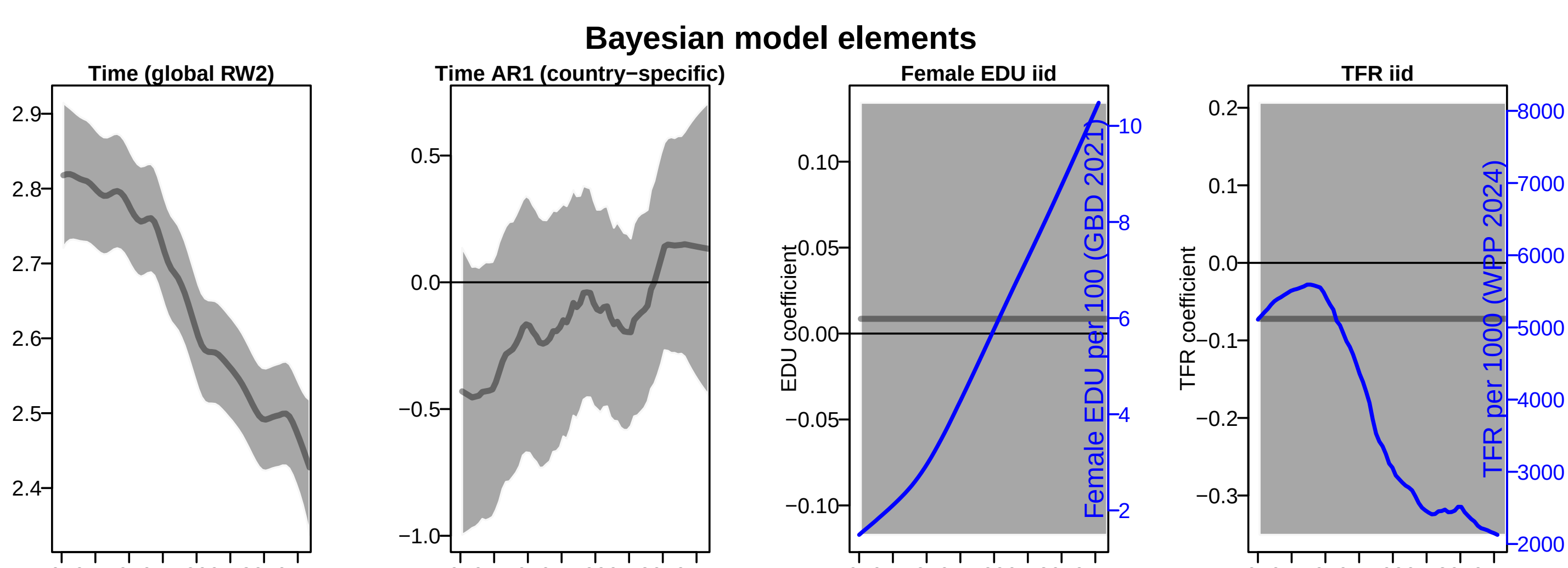
Bayesian model elements
Time (global RW2)
Time AR1 (country−specific)
Female EDU iid
TFR iid
EDU coefficient
Female EDU per 100 (GBD 2021)
TFR coefficient
TFR per 1000 (WPP 2024)
1950
1970
1990
2010

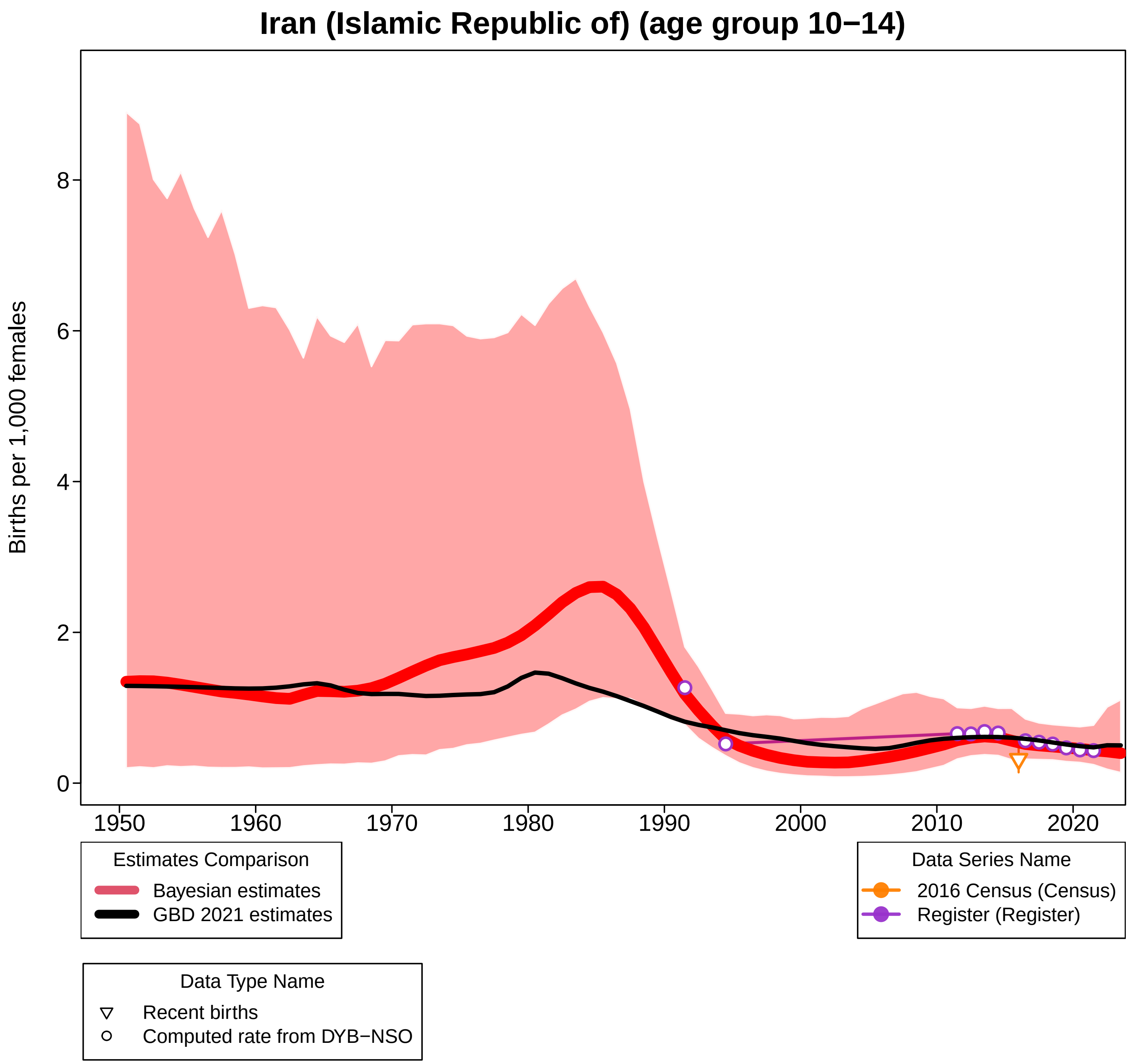
Iran (Islamic Republic of) (age group 10−14)
Births per 1,000 females
0
2
4
6
8
1950
1960
1970
1980
1990
2000
2010
2020
Estimates Comparison
Bayesian estimates
GBD 2021 estimates
Data Series Name
2016 Census (Census)
Register (Register)
Data Type Name
Recent births
Computed rate from DYB−NSO

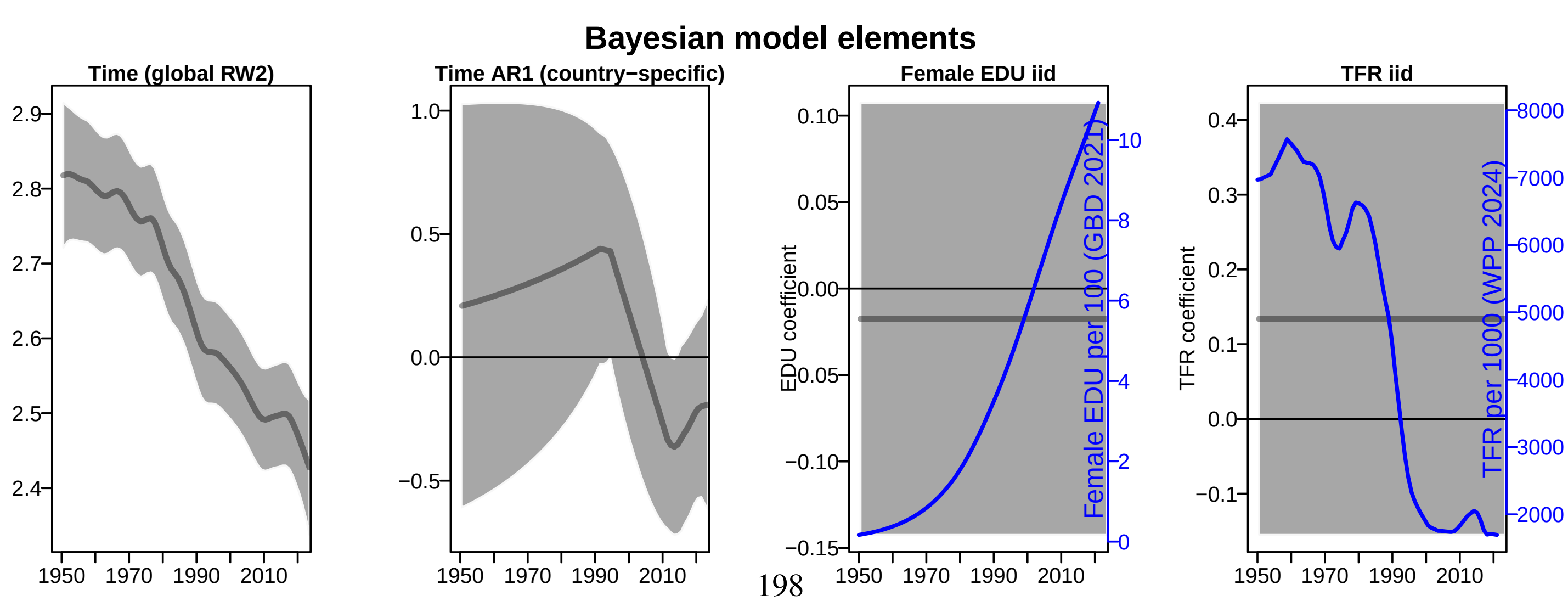
Bayesian model elements
Time (global RW2)
Time AR1 (country−specific)
Female EDU iid
TFR iid
EDU coefficient
Female EDU per 100 (GBD 2021)
TFR coefficient
TFR per 1000 (WPP 2024)
1950
1970
1990
2010

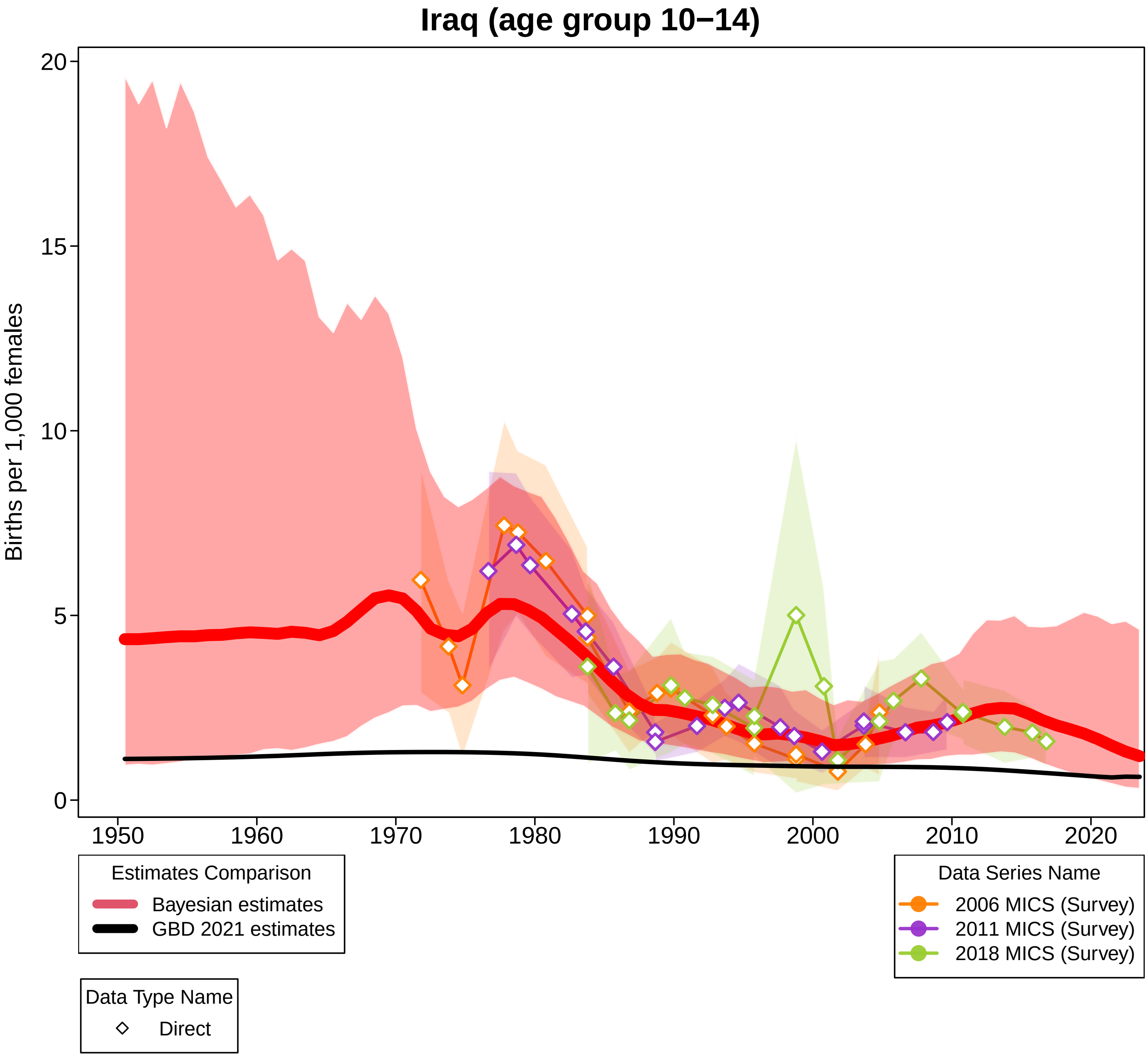

Iraq (age group 10−14)
Births per 1,000 females
0
5
10
15
20
1950
1960
1970
1980
1990
2000
2010
2020
Estimates Comparison
Bayesian estimates
GBD 2021 estimates
Data Series Name
2006 MICS (Survey)
2011 MICS (Survey)
2018 MICS (Survey)
Data Type Name
Direct

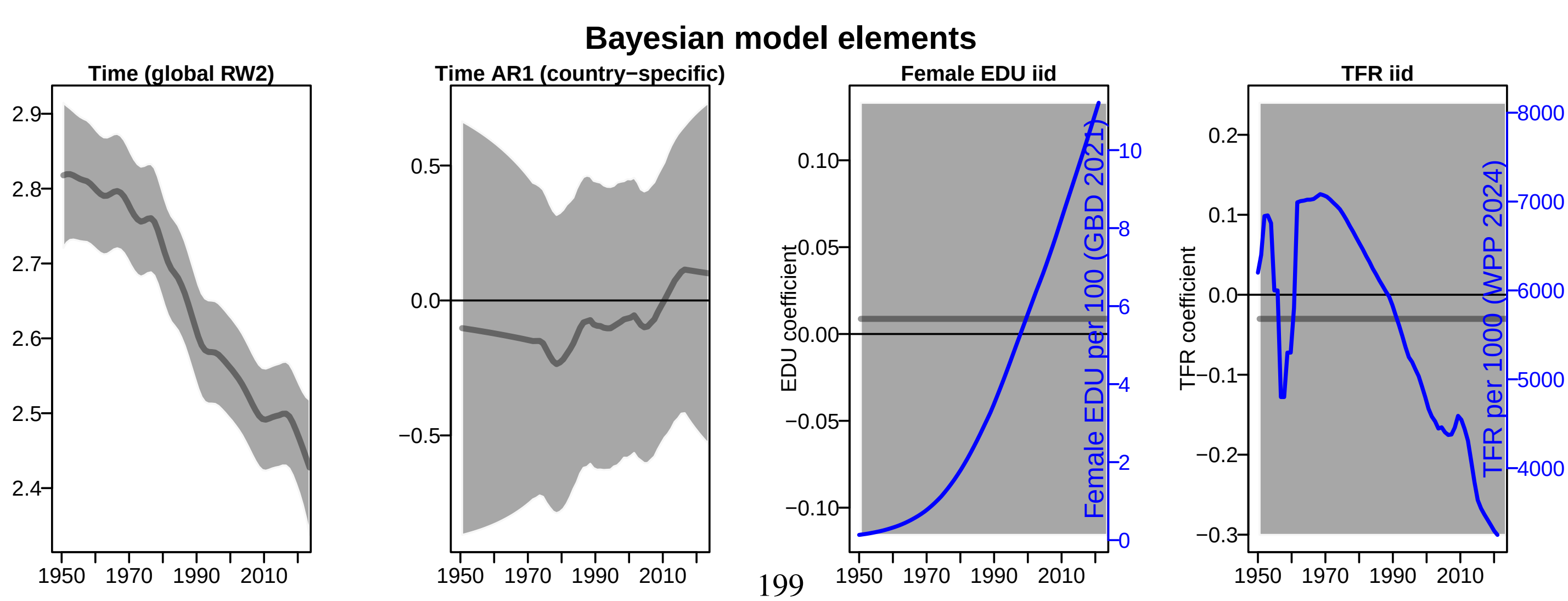

Bayesian model elements
Time (global RW2)
Time AR1 (country−specific)
Female EDU iid
TFR iid
EDU coefficient
Female EDU per 100 (GBD 2021)
TFR coefficient
TFR per 1000 (WPP 2024)
1950
1970
1990
2010

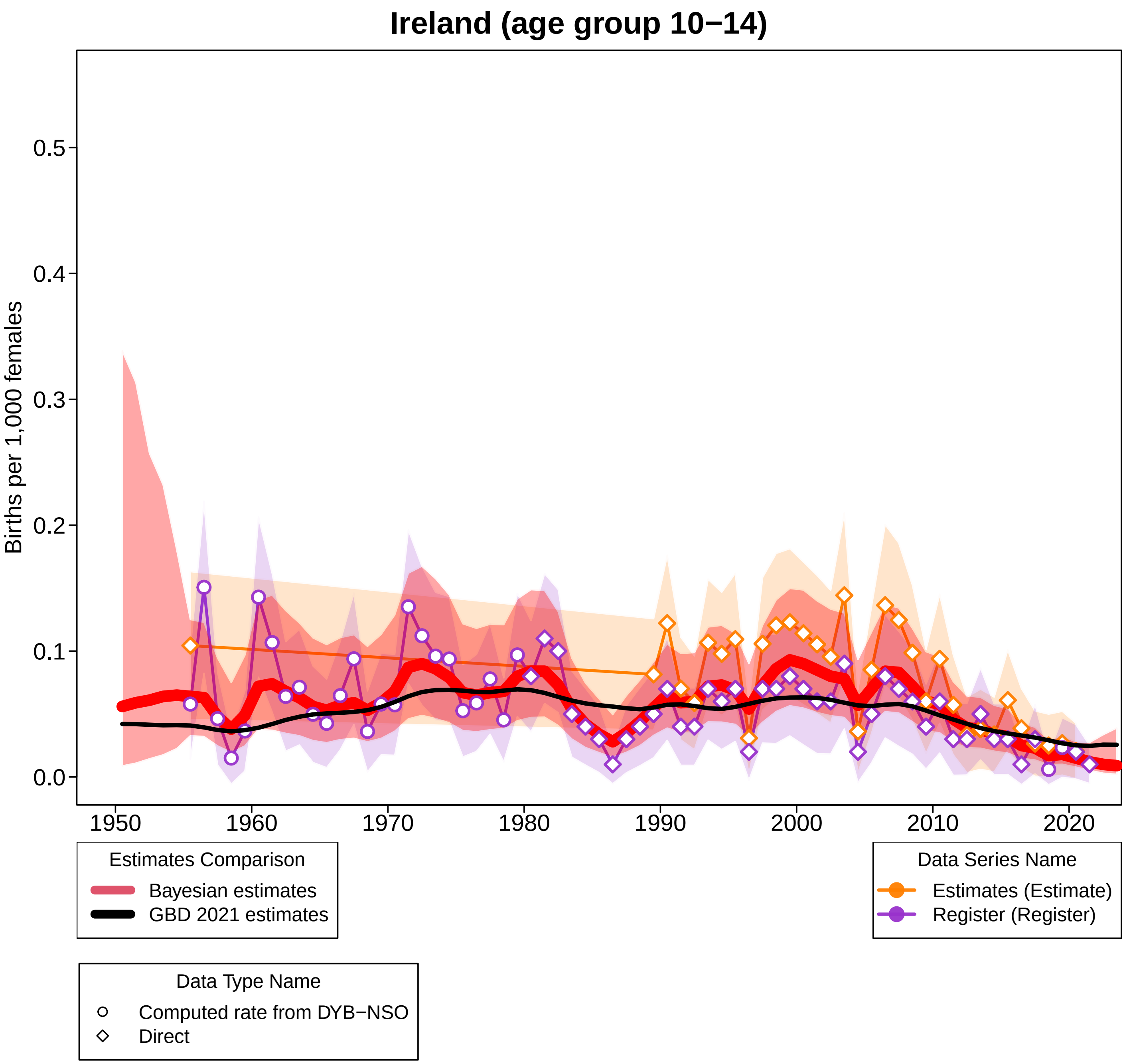
Ireland (age group 10−14)
Births per 1,000 females
0.0
0.1
0.2
0.3
0.4
0.5
1950
1960
1970
1980
1990
2000
2010
2020
Estimates Comparison
Bayesian estimates
GBD 2021 estimates
Data Series Name
Estimates (Estimate)
Register (Register)
Data Type Name
Computed rate from DYB−NSO
Direct

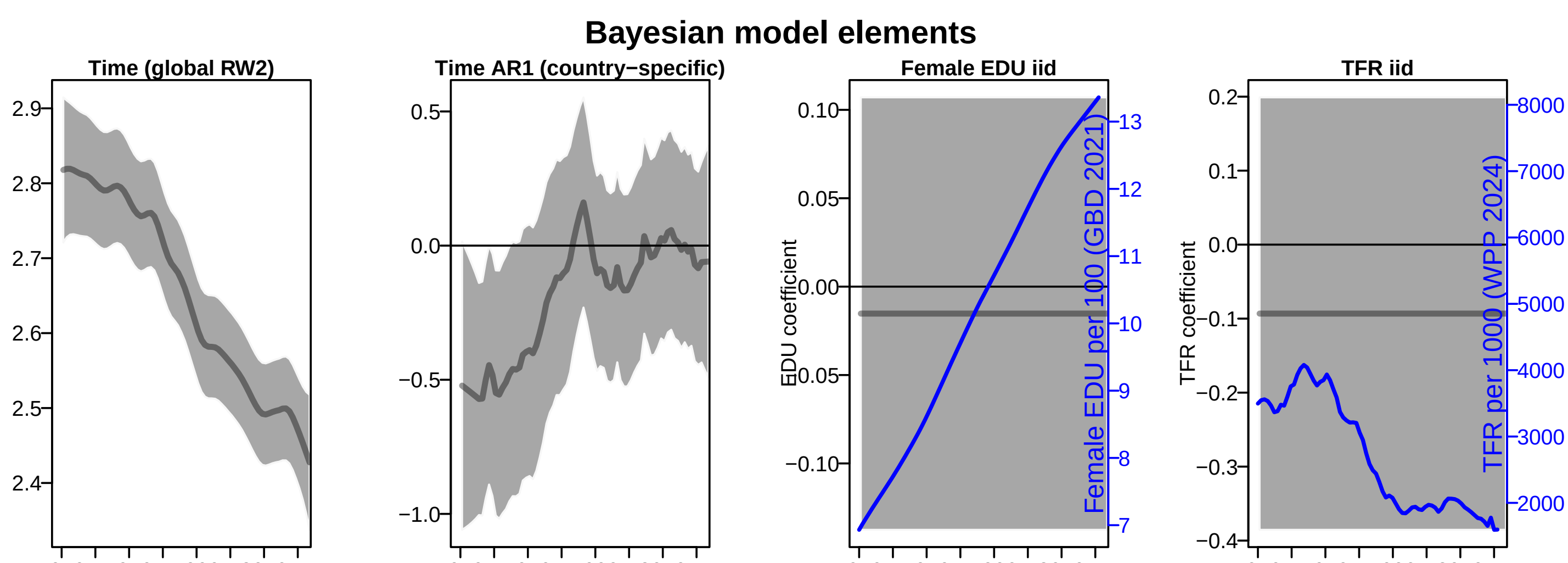
Bayesian model elements
Time (global RW2)
Time AR1 (country−specific)
Female EDU iid
TFR iid
EDU coefficient
Female EDU per 100 (GBD 2021)
TFR coefficient
TFR per 1000 (WPP 2024)
1950
1970
1990
2010

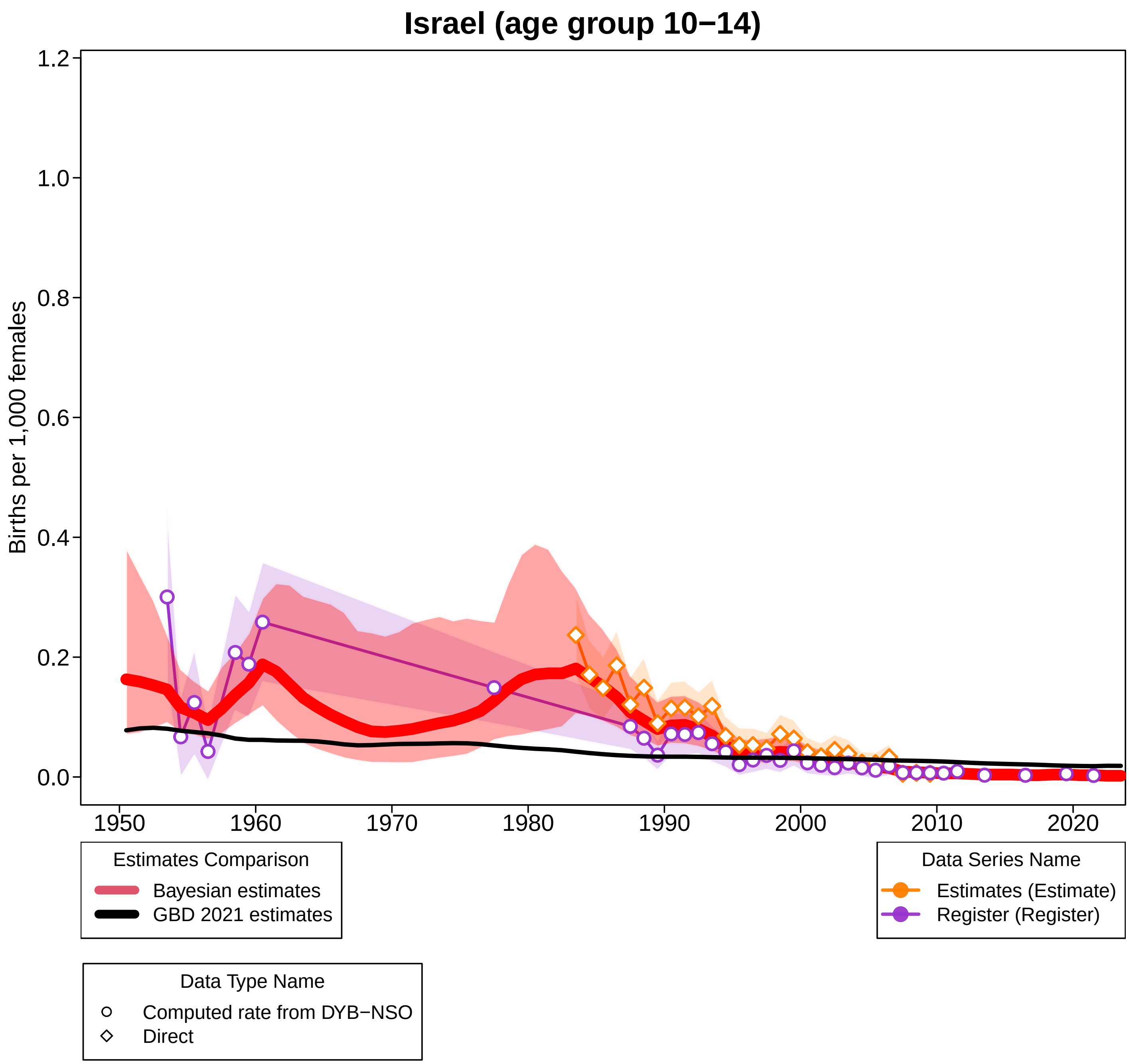
Israel (age group 10−14)
Births per 1,000 females
1.2
1.0
0.8
0.6
0.4
0.2
0.0
1950
1960
1970
1980
1990
2000
2010
2020
Estimates Comparison
Bayesian estimates
GBD 2021 estimates
Data Series Name
Estimates (Estimate)
Register (Register)
Data Type Name
Computed rate from DYB−NSO
Direct

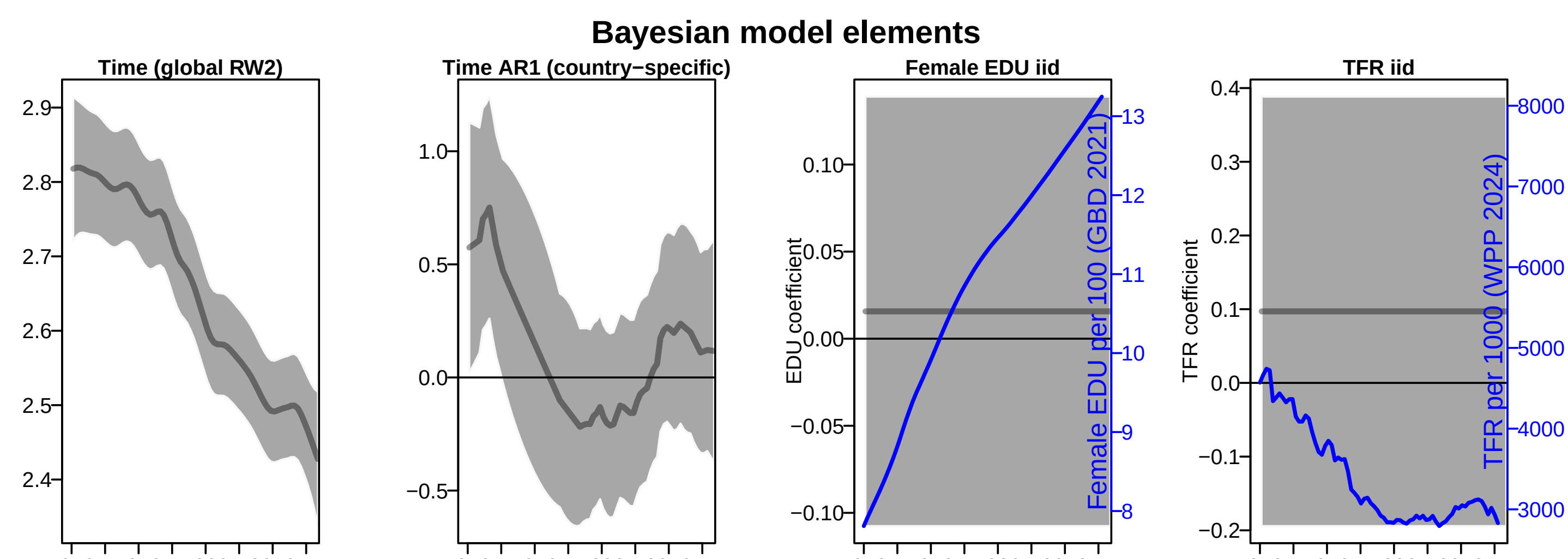
Bayesian model elements
Time (global RW2)
Time AR1 (country−specific)
Female EDU iid
TFR iid
EDU coefficient
Female EDU per 100 (GBD 2021)
TFR coefficient
TFR per 1000 (WPP 2024)
1950
1970
1990
2010

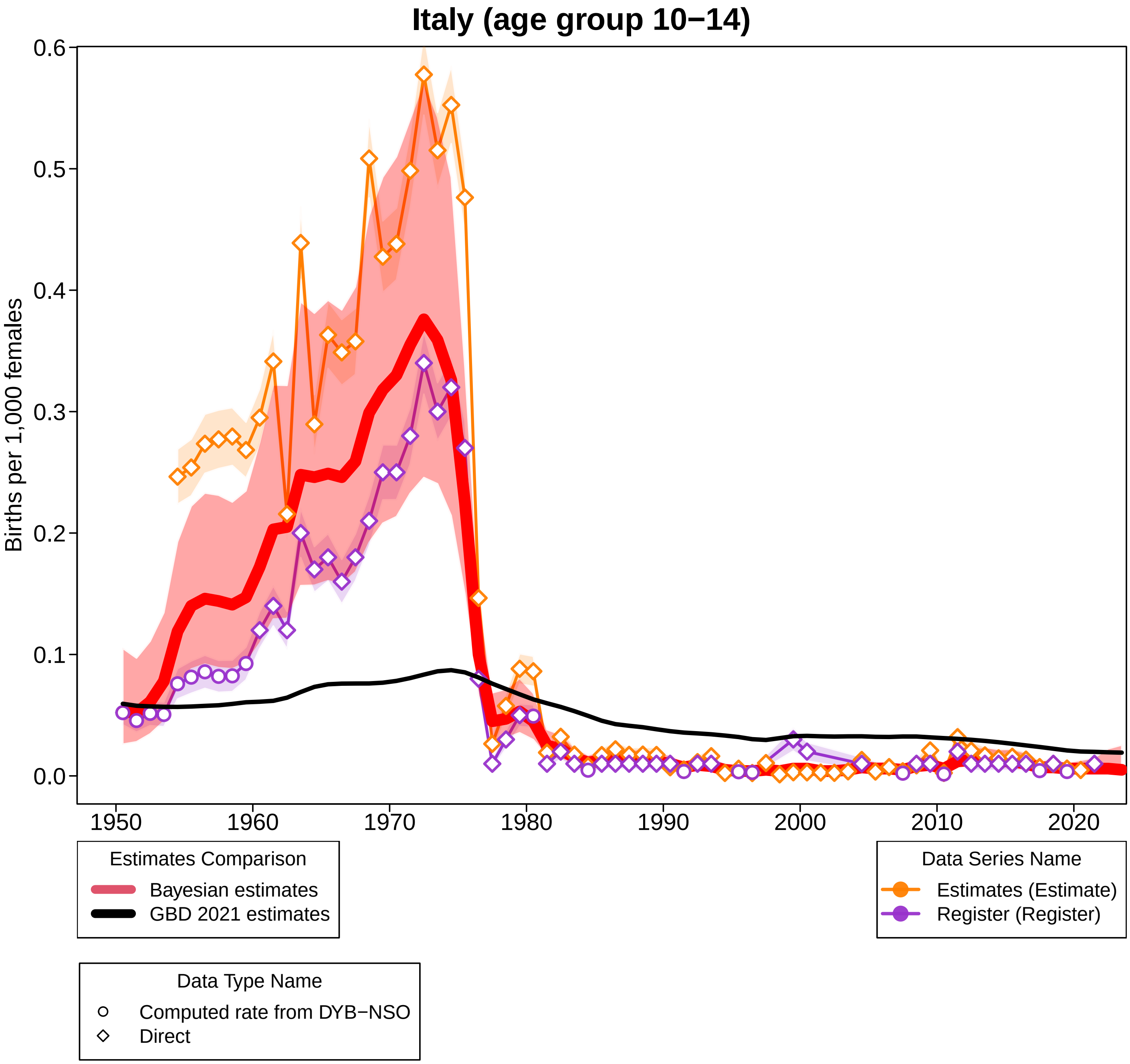
Italy (age group 10−14)
Births per 1,000 females
0.0
0.1
0.2
0.3
0.4
0.5
0.6
1950
1960
1970
1980
1990
2000
2010
2020
Estimates Comparison
Bayesian estimates
GBD 2021 estimates
Data Series Name
Estimates (Estimate)
Register (Register)
Data Type Name
Computed rate from DYB−NSO
Direct

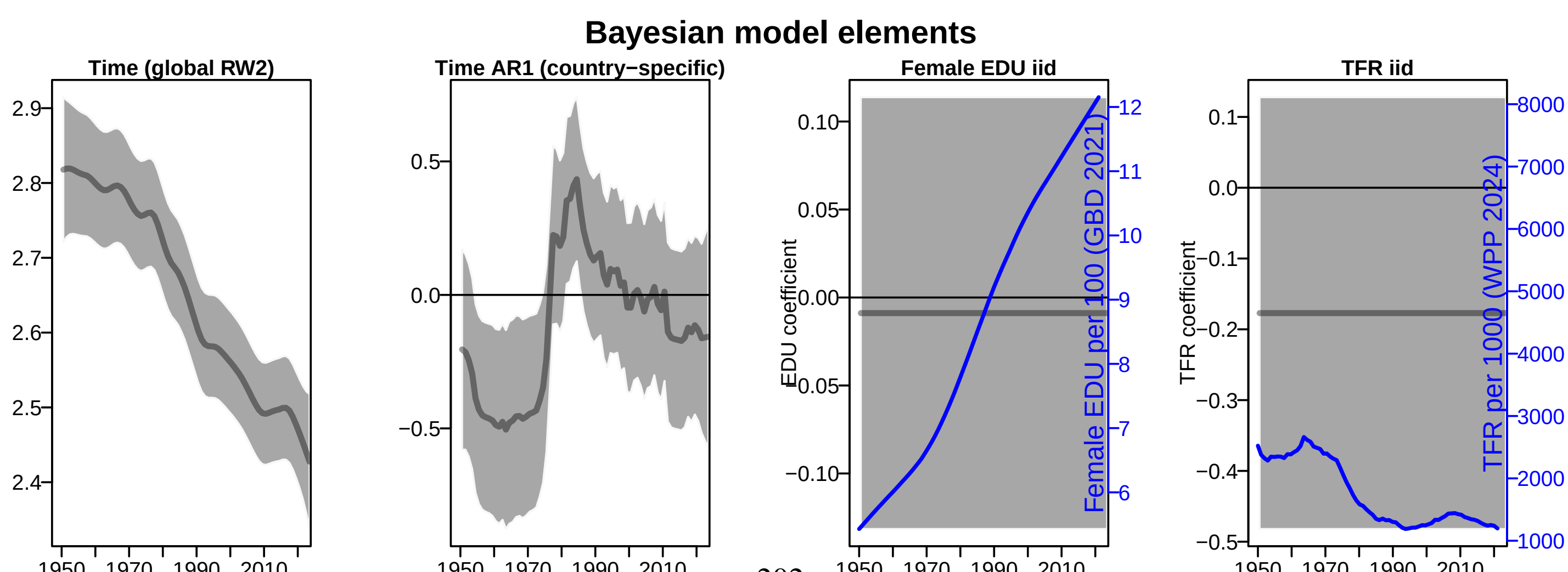
Bayesian model elements
Time (global RW2)
Time AR1 (country−specific)
Female EDU iid
TFR iid
EDU coefficient
Female EDU per 100 (GBD 2021)
TFR coefficient
TFR per 1000 (WPP 2024)

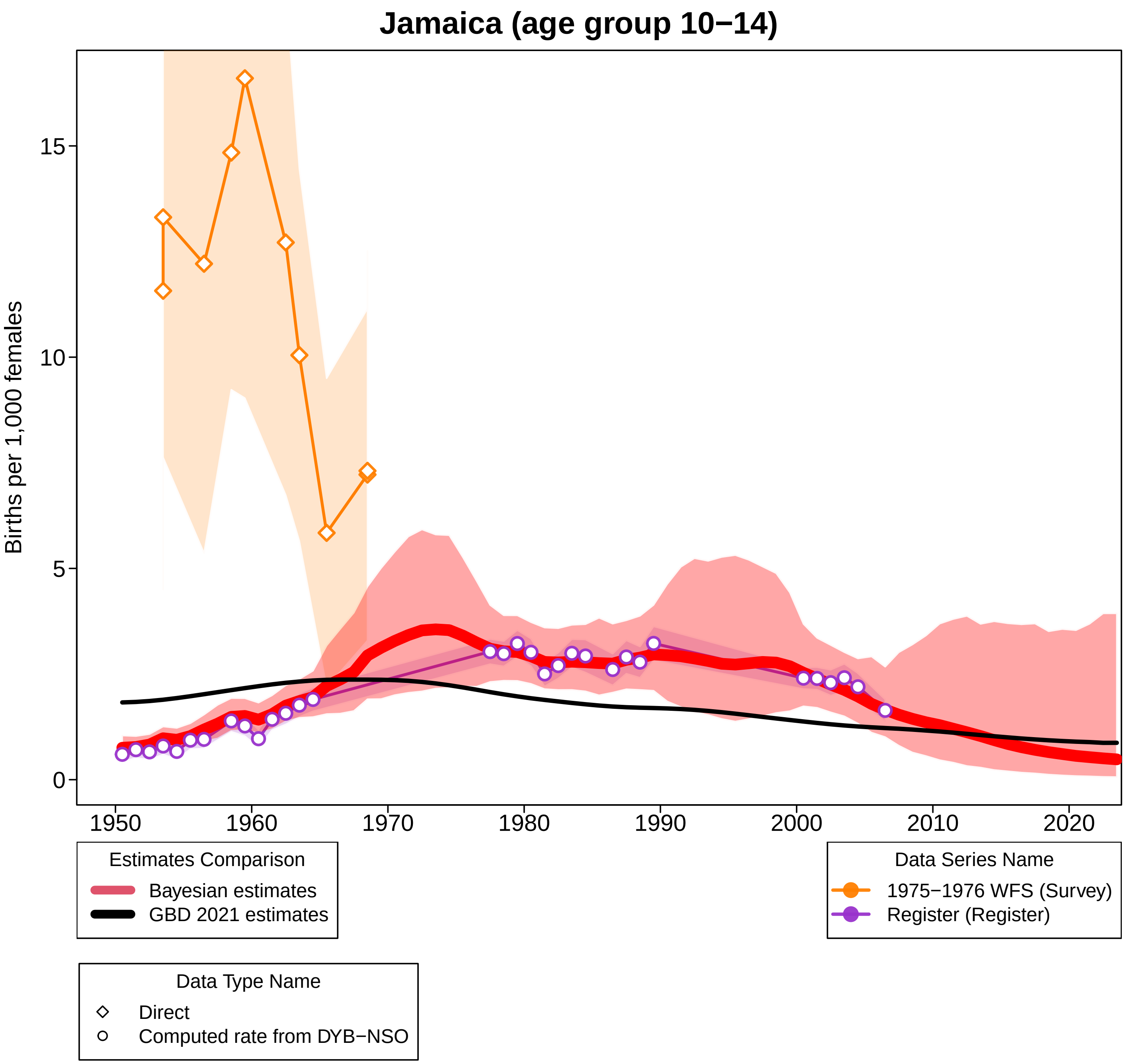

Jamaica (age group 10−14)
Births per 1,000 females
0
5
10
15
1950
1960
1970
1980
1990
2000
2010
2020
Estimates Comparison
Bayesian estimates
GBD 2021 estimates
Data Series Name
1975−1976 WFS (Survey)
Register (Register)
Data Type Name
Direct
Computed rate from DYB−NSO

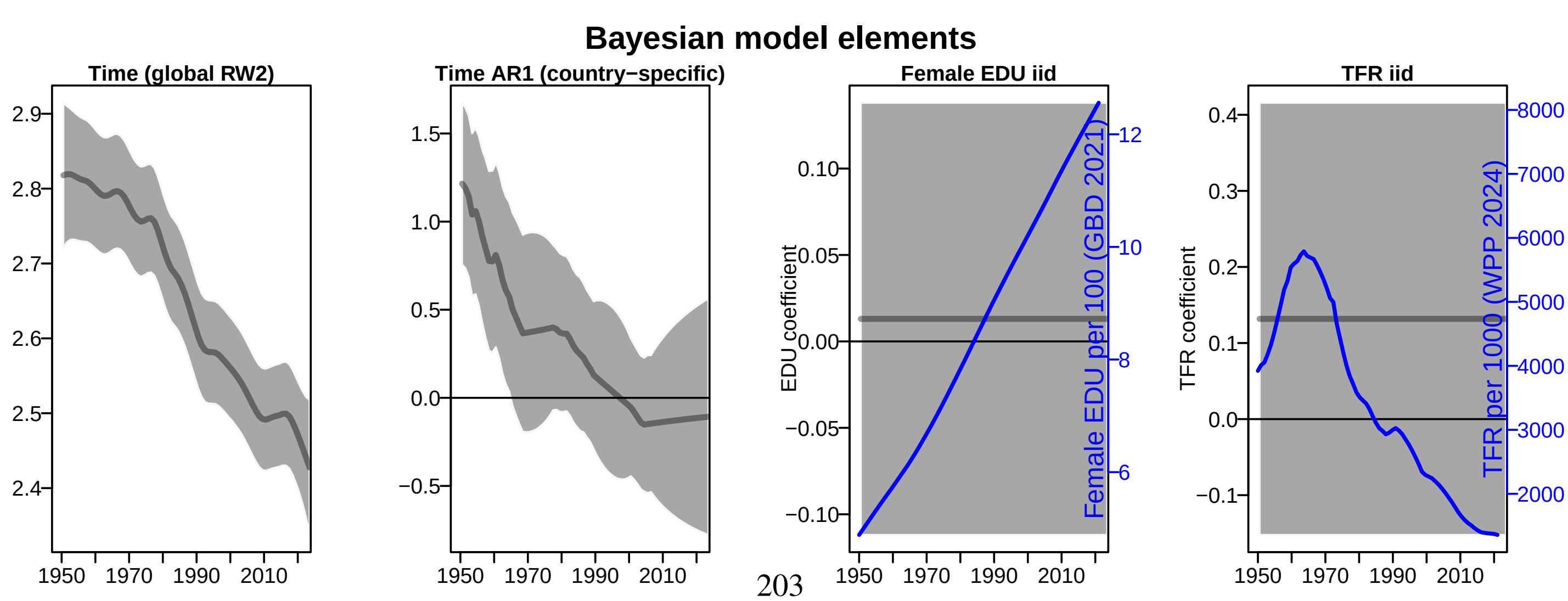

Bayesian model elements
Time (global RW2)
Time AR1 (country−specific)
Female EDU iid
TFR iid
EDU coefficient
Female EDU per 100 (GBD 2021)
TFR coefficient
TFR per 1000 (WPP 2024)
1950
1970
1990
2010

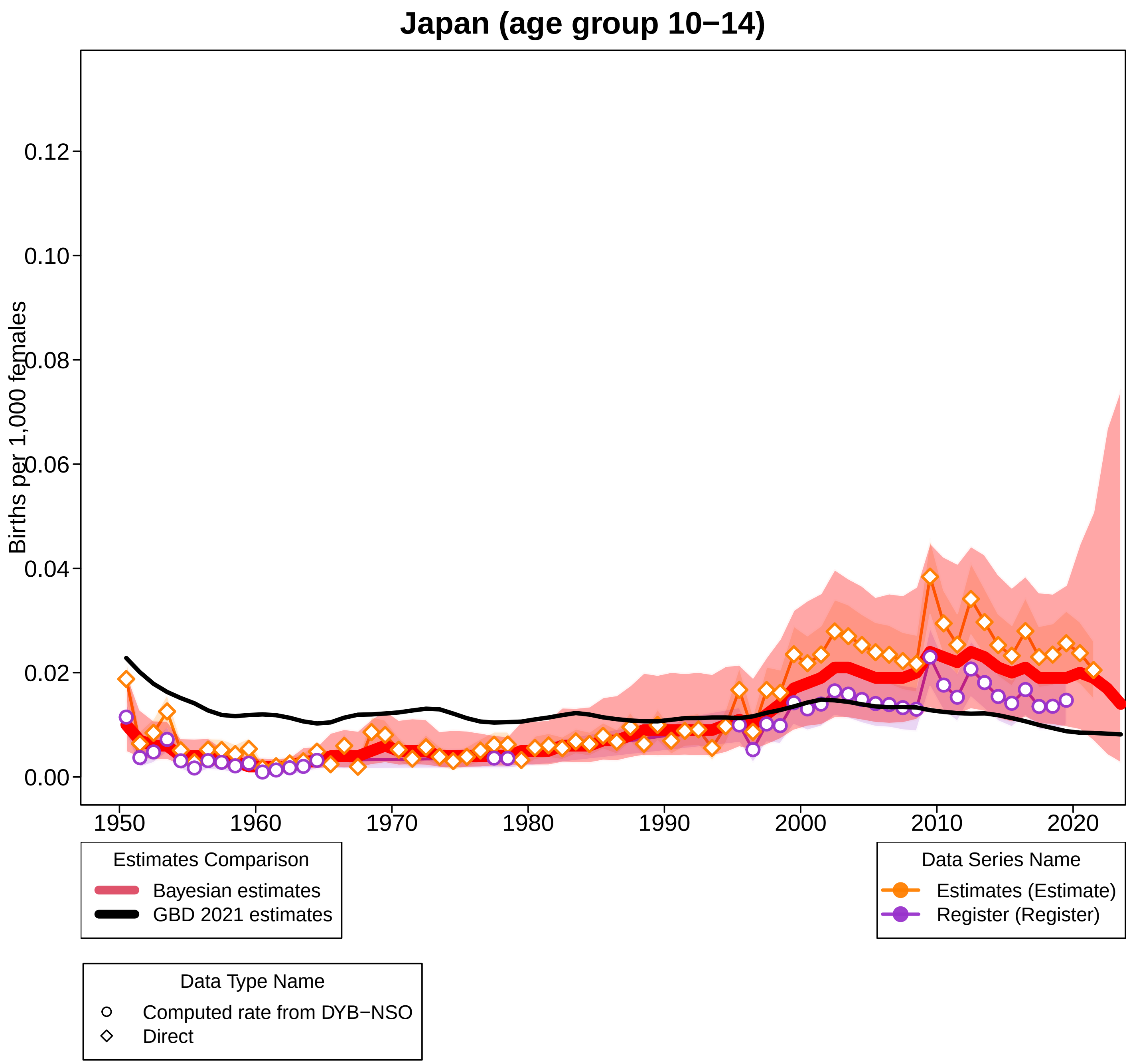

Japan (age group 10−14)
Births per 1,000 females
0.12
0.10
0.08
0.06
0.04
0.02
0.00
1950
1960
1970
1980
1990
2000
2010
2020
Estimates Comparison
Bayesian estimates
GBD 2021 estimates
Data Series Name
Estimates (Estimate)
Register (Register)
Data Type Name
Computed rate from DYB−NSO
Direct

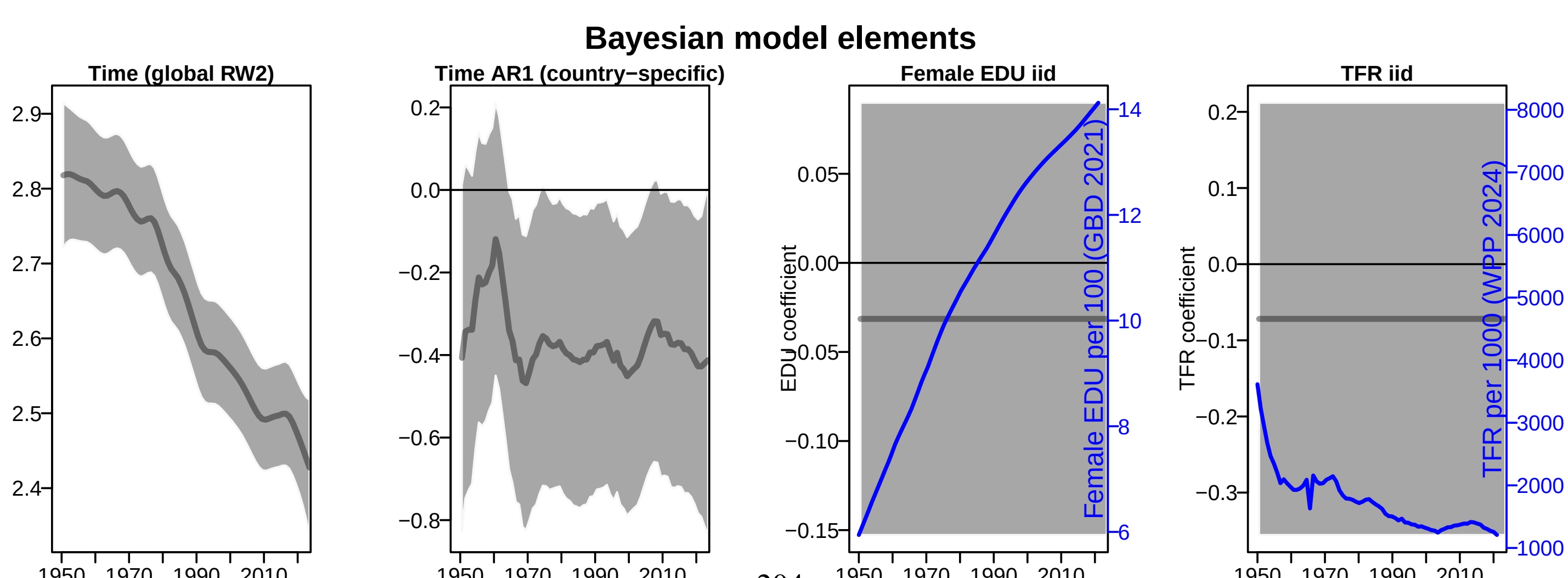

Bayesian model elements
Time (global RW2)
Time AR1 (country−specific)
Female EDU iid
TFR iid
EDU coefficient
Female EDU per 100 (GBD 2021)
TFR coefficient
TFR per 1000 (WPP 2024)
1950
1970
1990
2010

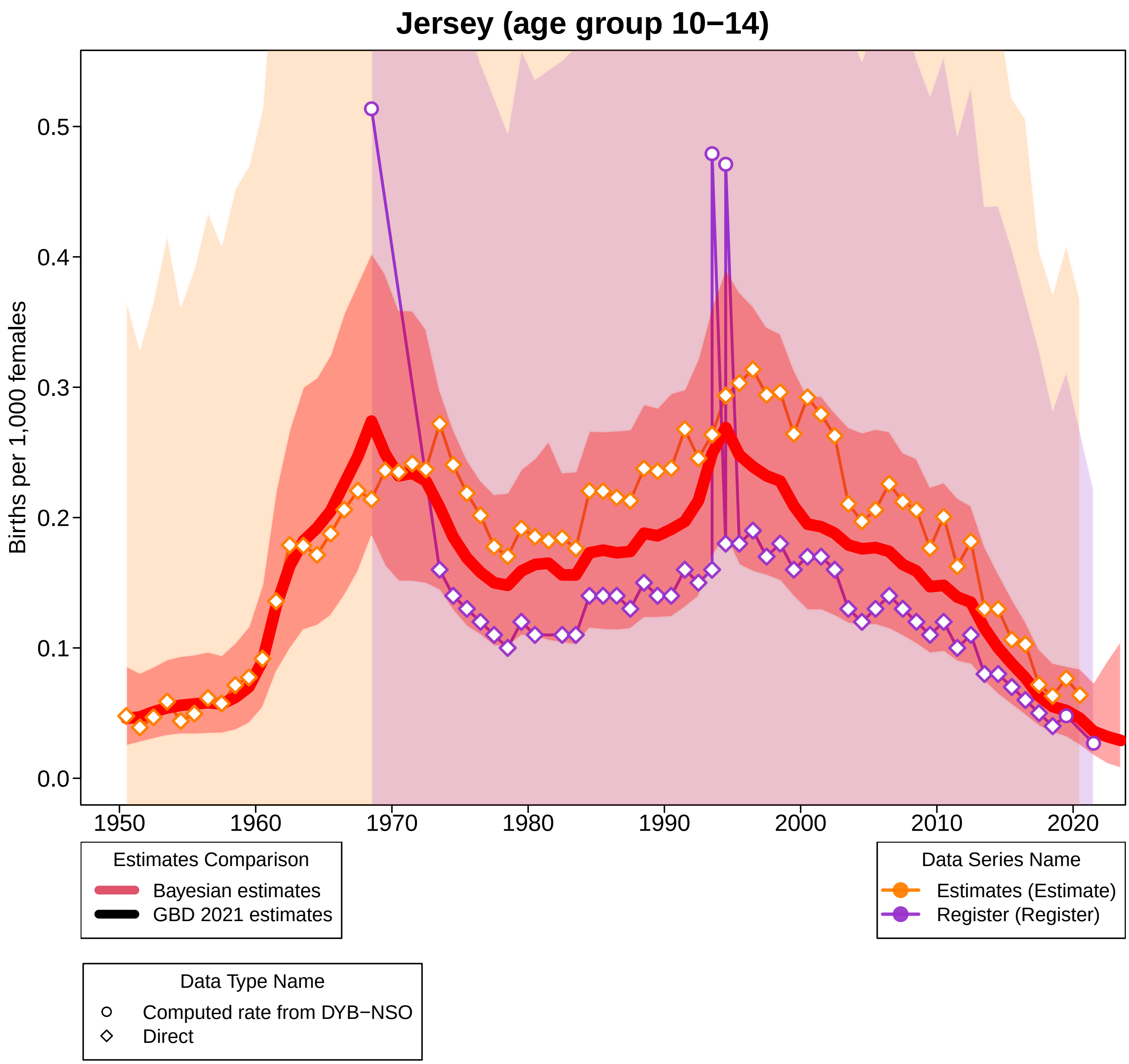

Jersey (age group 10−14)
Births per 1,000 females
0.0
0.1
0.2
0.3
0.4
0.5
1950
1960
1970
1980
1990
2000
2010
2020
Estimates Comparison
Bayesian estimates
GBD 2021 estimates
Data Series Name
Estimates (Estimate)
Register (Register)
Data Type Name
Computed rate from DYB−NSO
Direct

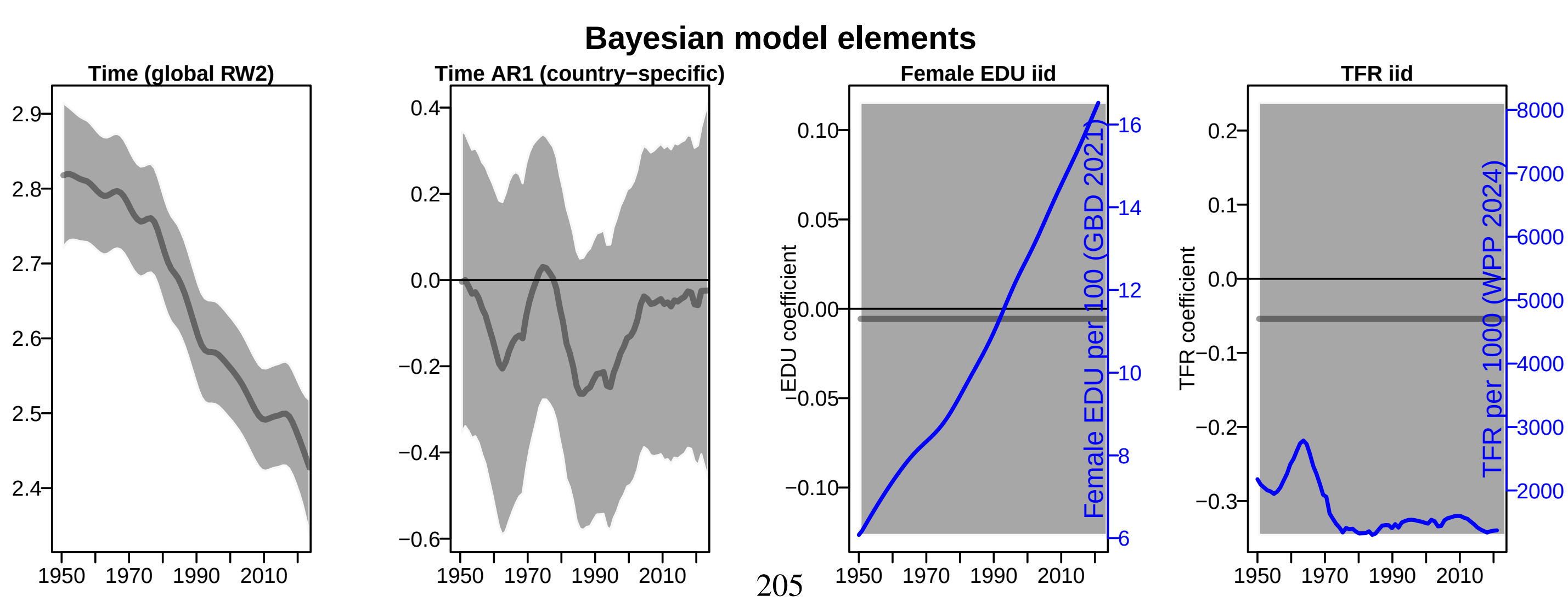

Bayesian model elements
Time (global RW2)
Time AR1 (country−specific)
Female EDU iid
TFR iid
EDU coefficient
Female EDU per 100 (GBD 2021)
TFR coefficient
TFR per 1000 (WPP 2024)
1950
1970
1990
2010

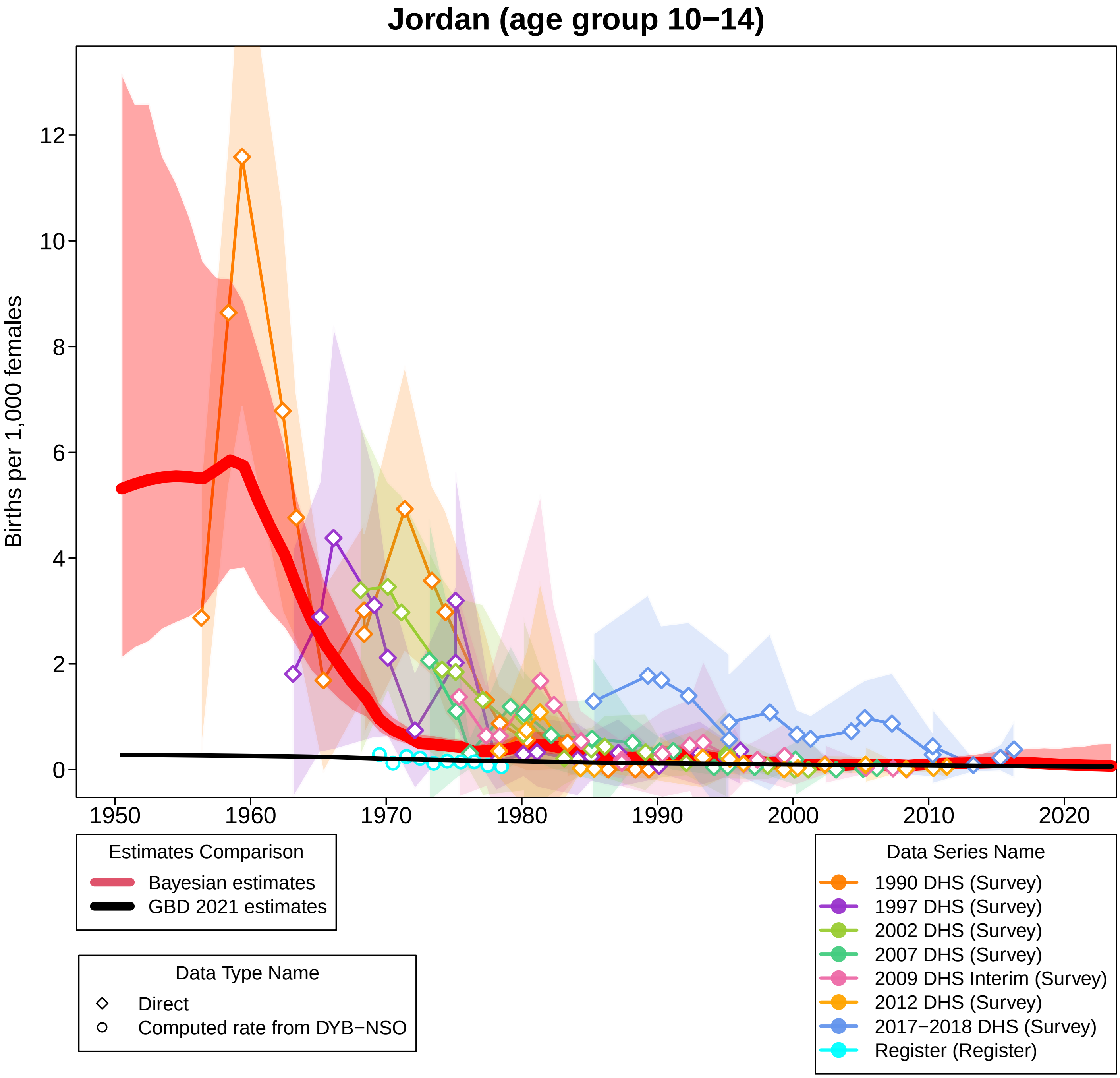
Jordan (age group 10−14)
Births per 1,000 females
Estimates Comparison
Bayesian estimates
GBD 2021 estimates
Data Type Name
Direct
Computed rate from DYB−NSO
Data Series Name
1990 DHS (Survey)
1997 DHS (Survey)
2002 DHS (Survey)
2007 DHS (Survey)
2009 DHS Interim (Survey)
2012 DHS (Survey)
2017−2018 DHS (Survey)
Register (Register)

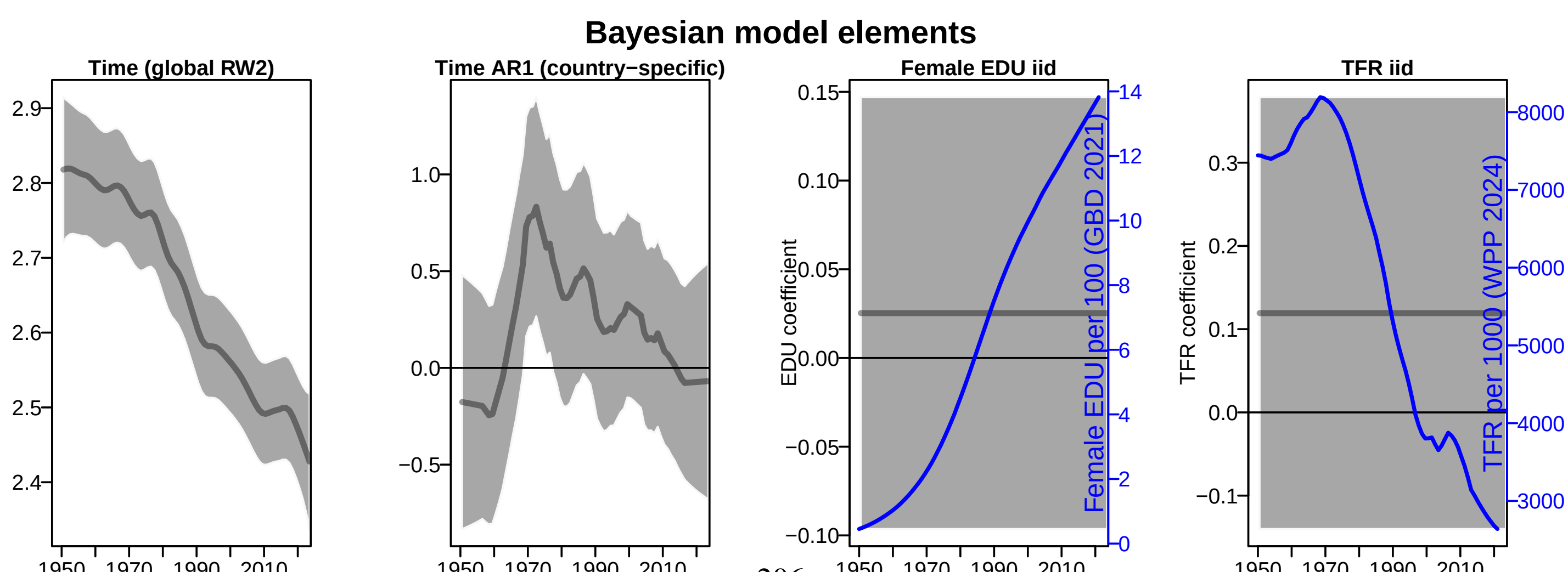
Bayesian model elements
Time (global RW2)
Time AR1 (country−specific)
Female EDU iid
EDU coefficient
Female EDU per 100 (GBD 2021)
TFR iid
TFR coefficient
TFR per 1000 (WPP 2024)

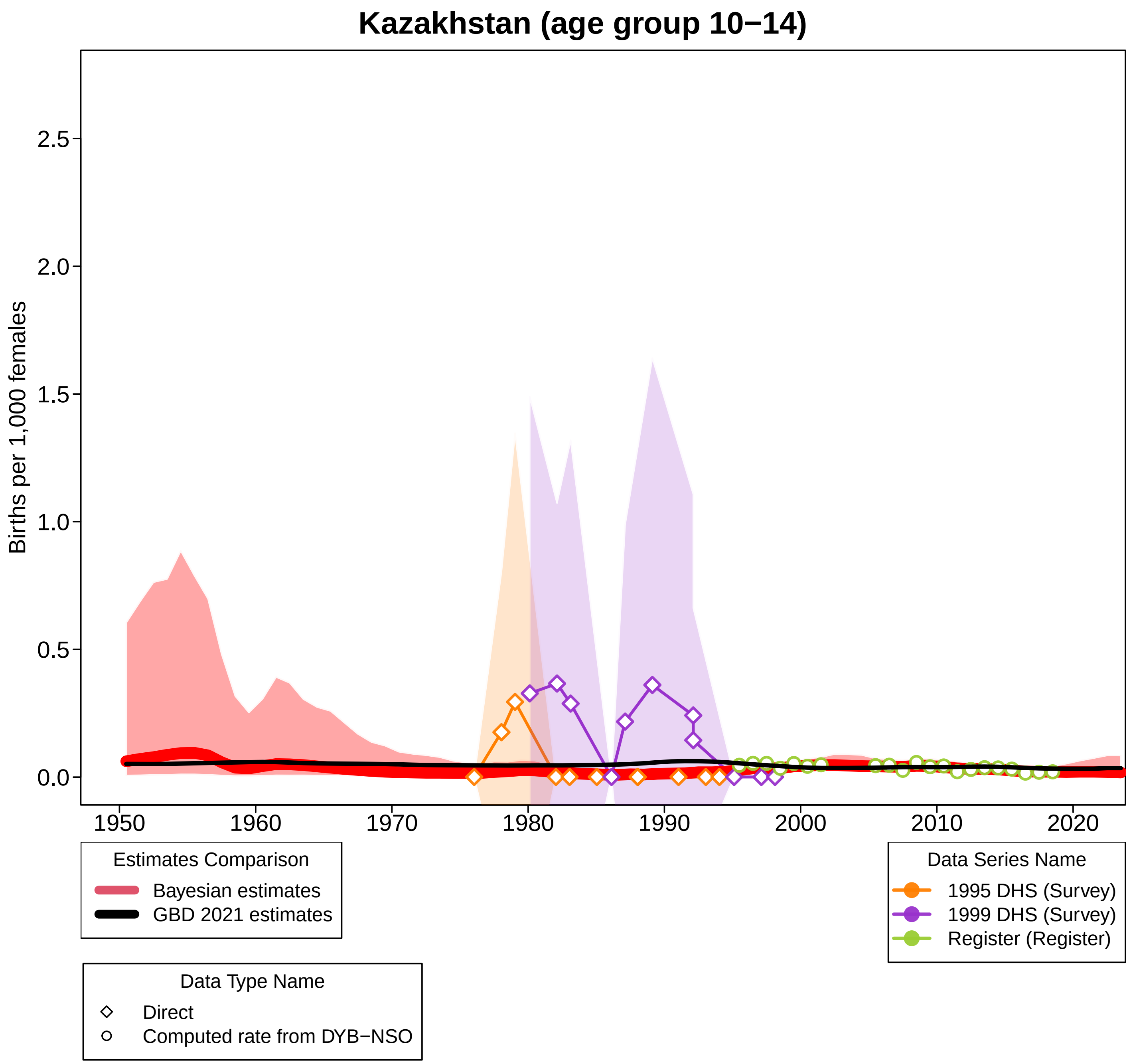
Kazakhstan (age group 10−14)
Births per 1,000 females
2.5
2.0
1.5
1.0
0.5
0.0
1950
1960
1970
1980
1990
2000
2010
2020
Estimates Comparison
Bayesian estimates
GBD 2021 estimates
Data Series Name
1995 DHS (Survey)
1999 DHS (Survey)
Register (Register)
Data Type Name
Direct
Computed rate from DYB−NSO

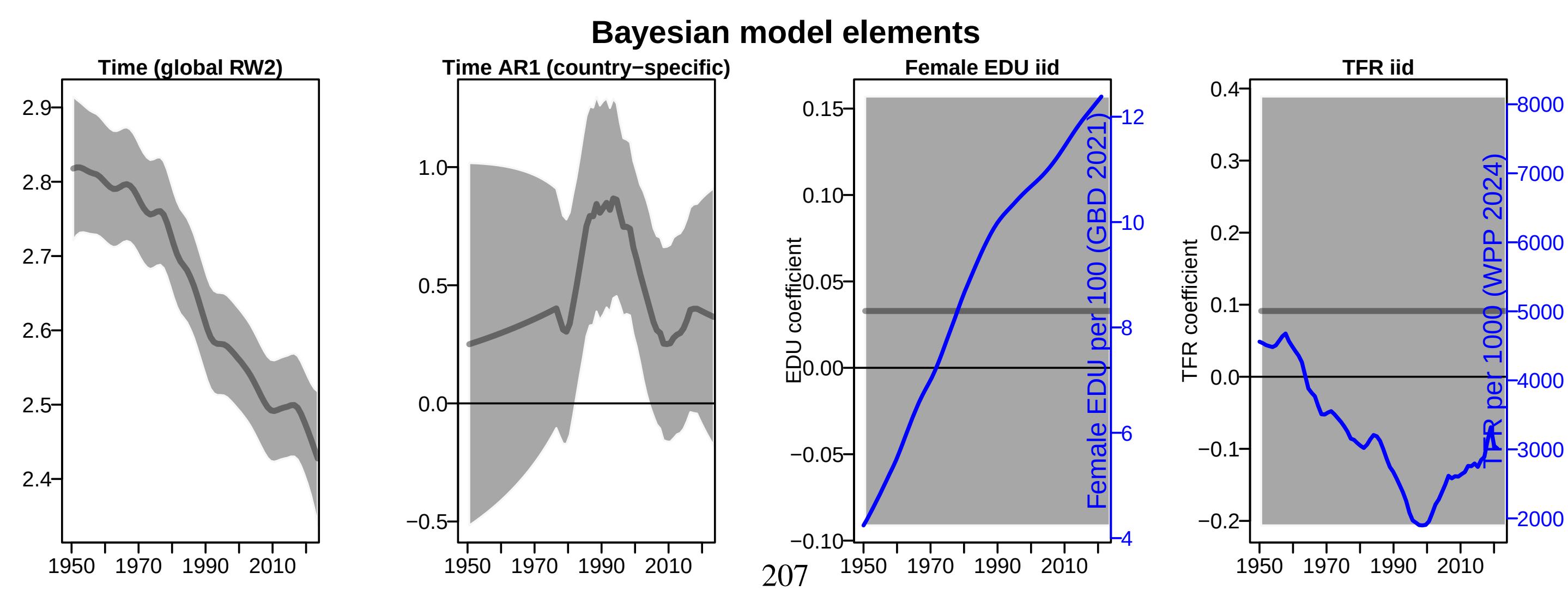
Bayesian model elements
Time (global RW2)
Time AR1 (country−specific)
Female EDU iid
TFR iid
EDU coefficient
Female EDU per 100 (GBD 2021)
TFR coefficient
TFR per 1000 (WPP 2024)

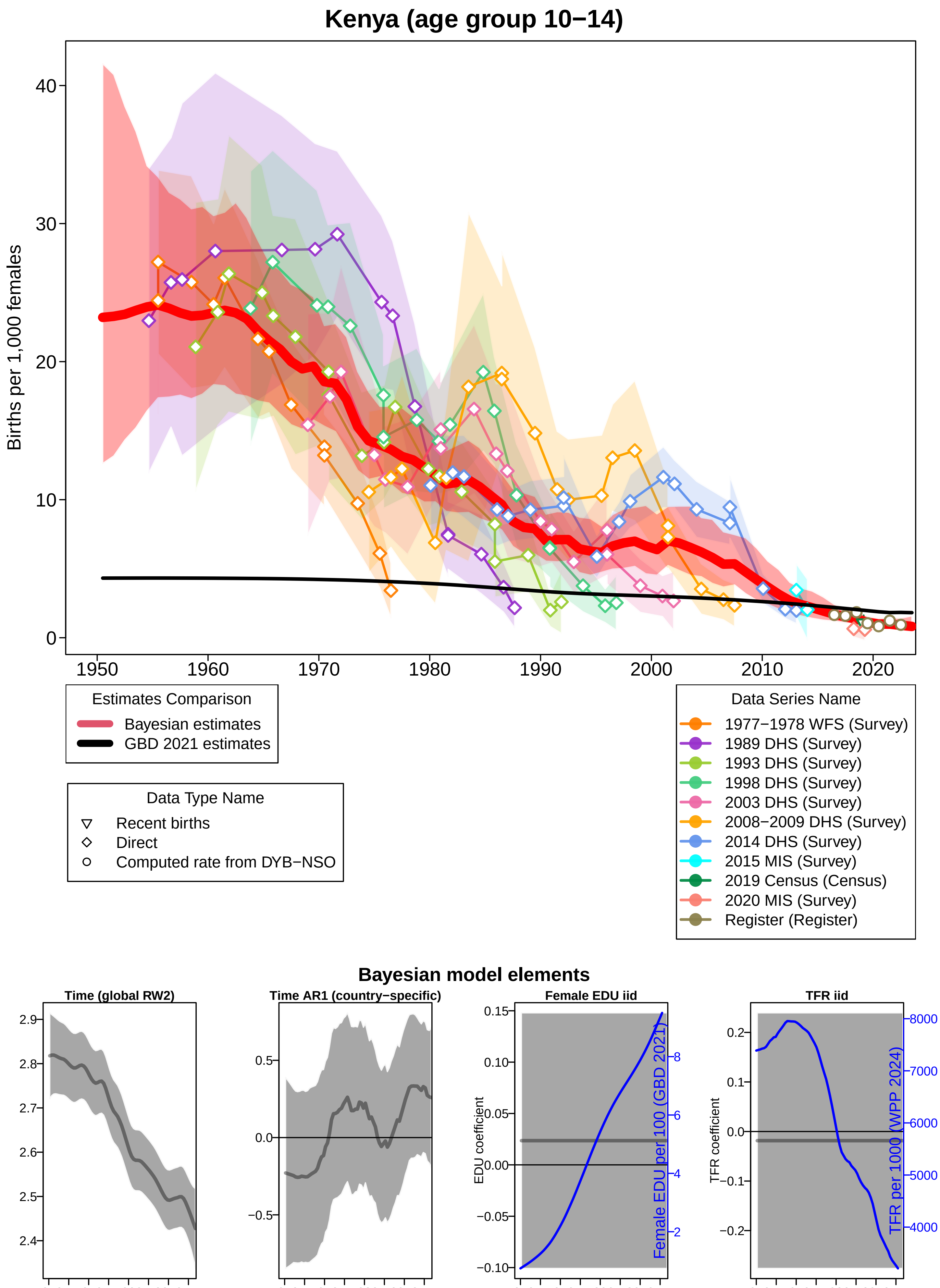
Kenya (age group 10−14)
Births per 1,000 females
0
10
20
30
40
1950
1960
1970
1980
1990
2000
2010
2020
Estimates Comparison
Bayesian estimates
GBD 2021 estimates
Data Type Name
Recent births
Direct
Computed rate from DYB−NSO
Data Series Name
1977−1978 WFS (Survey)
1989 DHS (Survey)
1993 DHS (Survey)
1998 DHS (Survey)
2003 DHS (Survey)
2008−2009 DHS (Survey)
2014 DHS (Survey)
2015 MIS (Survey)
2019 Census (Census)
2020 MIS (Survey)
Register (Register)
Bayesian model elements
Time (global RW2)
Time AR1 (country−specific)
Female EDU iid
TFR iid
EDU coefficient
Female EDU per 100 (GBD 2021)
TFR coefficient
TFR per 1000 (WPP 2024)

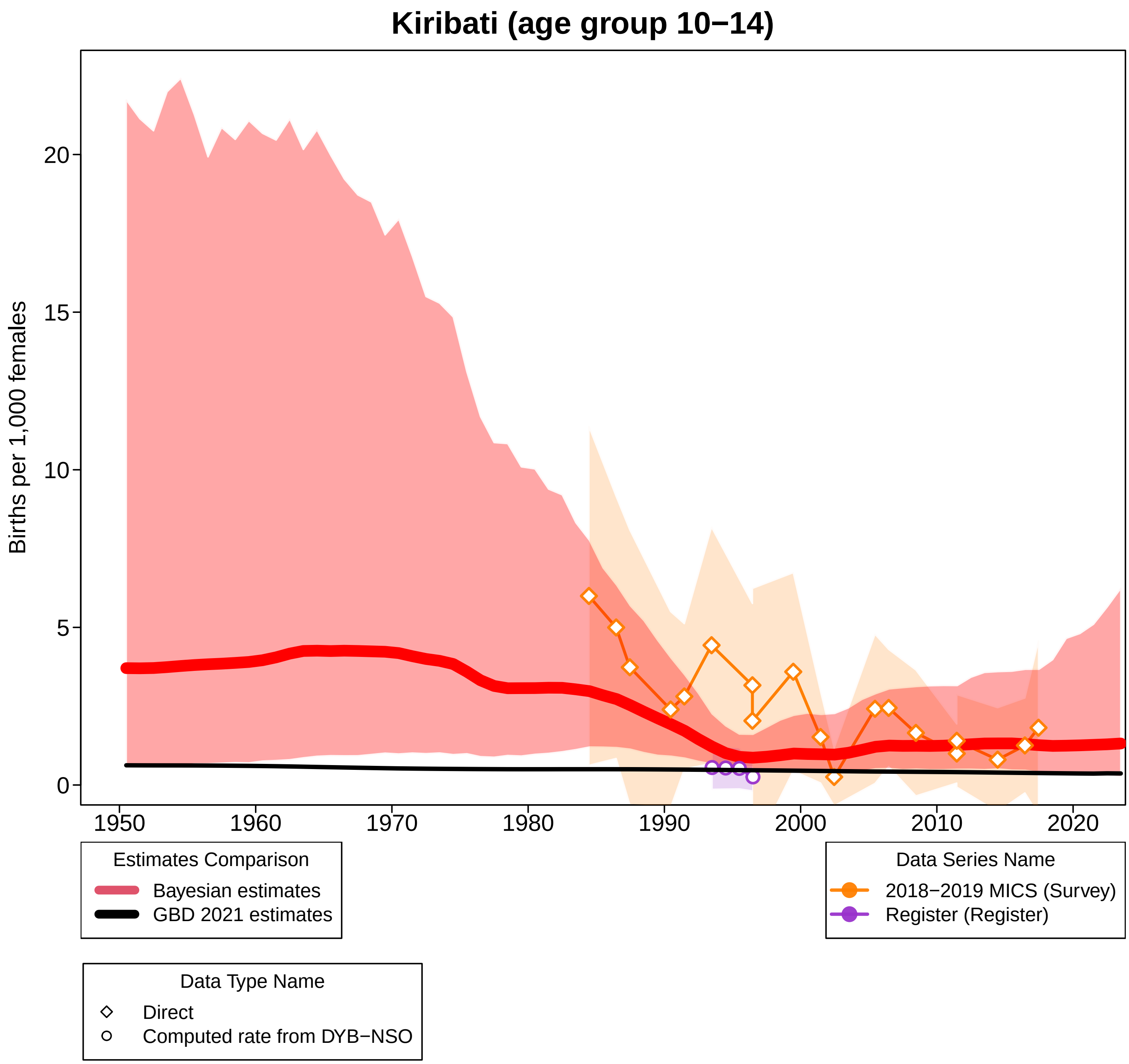

Kiribati (age group 10−14)
Births per 1,000 females
0
5
10
15
20
1950
1960
1970
1980
1990
2000
2010
2020
Estimates Comparison
Bayesian estimates
GBD 2021 estimates
Data Series Name
2018−2019 MICS (Survey)
Register (Register)
Data Type Name
Direct
Computed rate from DYB−NSO

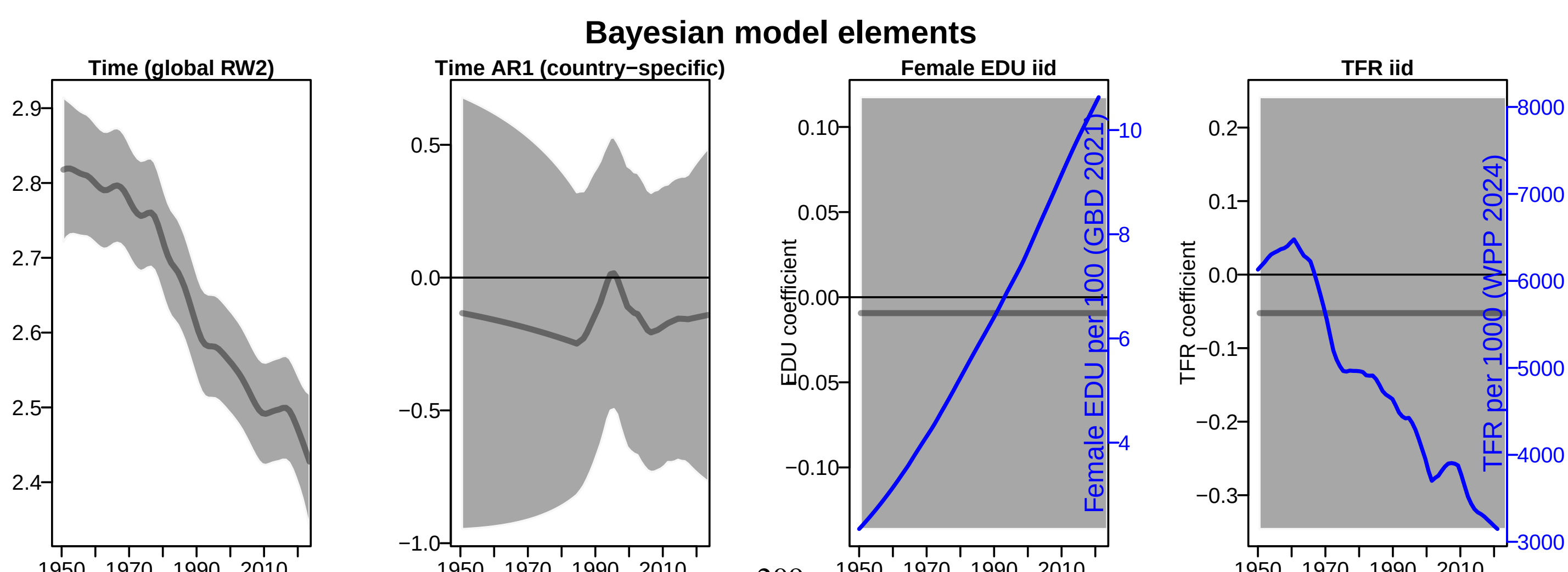

Bayesian model elements
Time (global RW2)
Time AR1 (country−specific)
Female EDU iid
TFR iid
EDU coefficient
Female EDU per 100 (GBD 2021)
TFR coefficient
TFR per 1000 (WPP 2024)
1950
1970
1990
2010

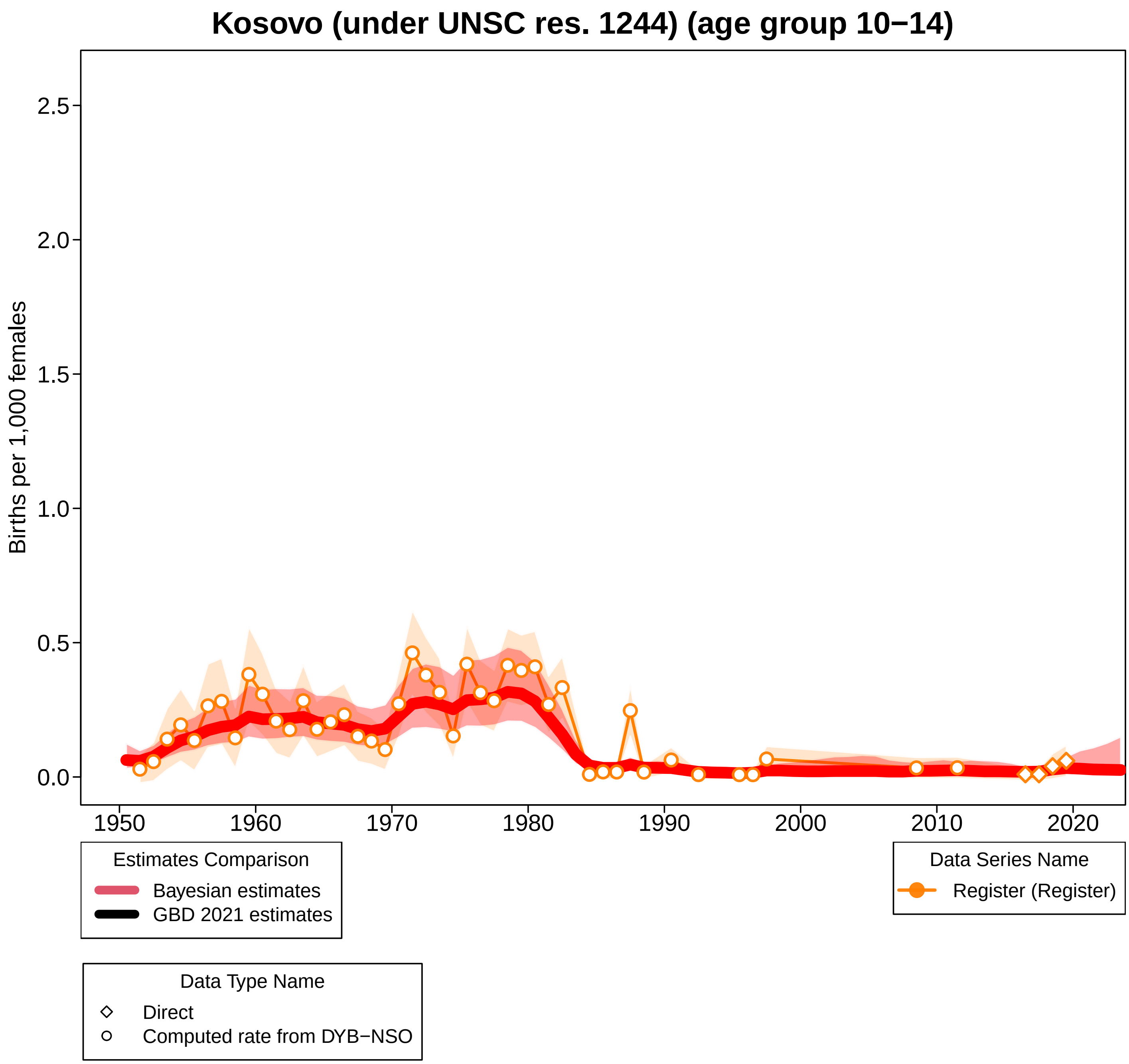
Kosovo (under UNSC res. 1244) (age group 10−14)
Births per 1,000 females
2.5
2.0
1.5
1.0
0.5
0.0
1950
1960
1970
1980
1990
2000
2010
2020
Estimates Comparison
Bayesian estimates
GBD 2021 estimates
Data Series Name
Register (Register)
Data Type Name
Direct
Computed rate from DYB−NSO

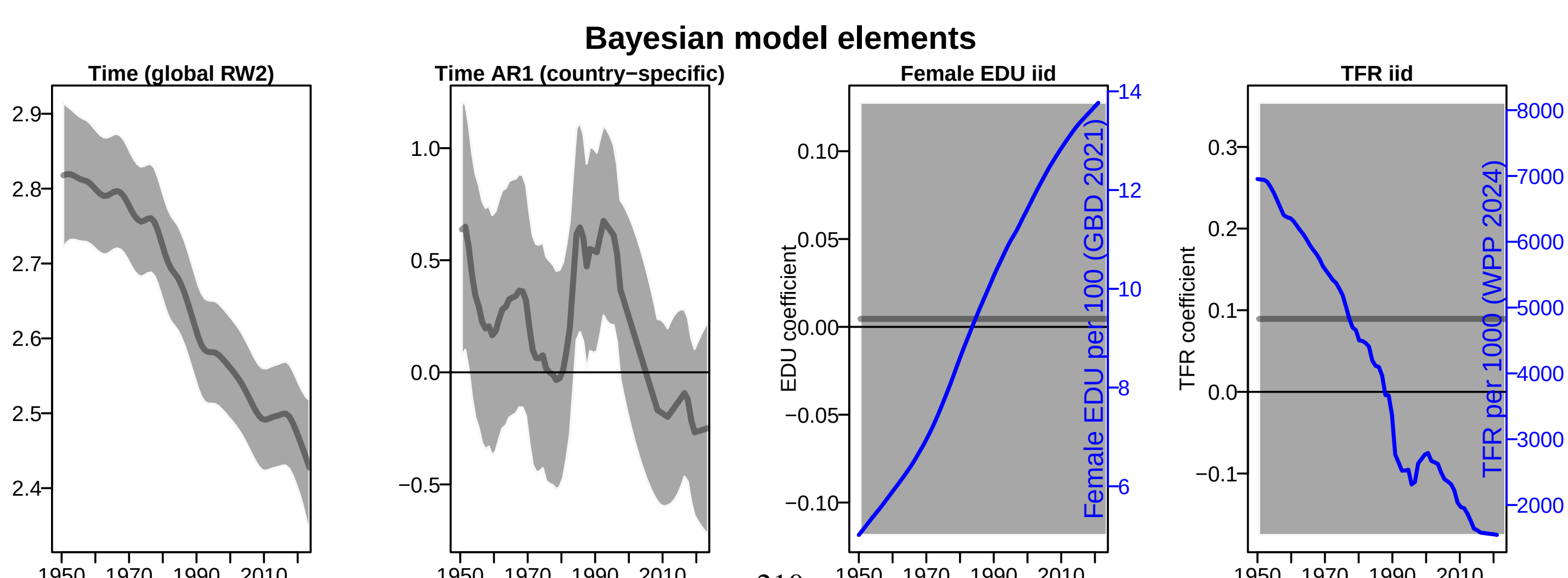
Bayesian model elements
Time (global RW2)
Time AR1 (country−specific)
Female EDU iid
TFR iid
EDU coefficient
Female EDU per 100 (GBD 2021)
TFR coefficient
TFR per 1000 (WPP 2024)
1950
1970
1990
2010

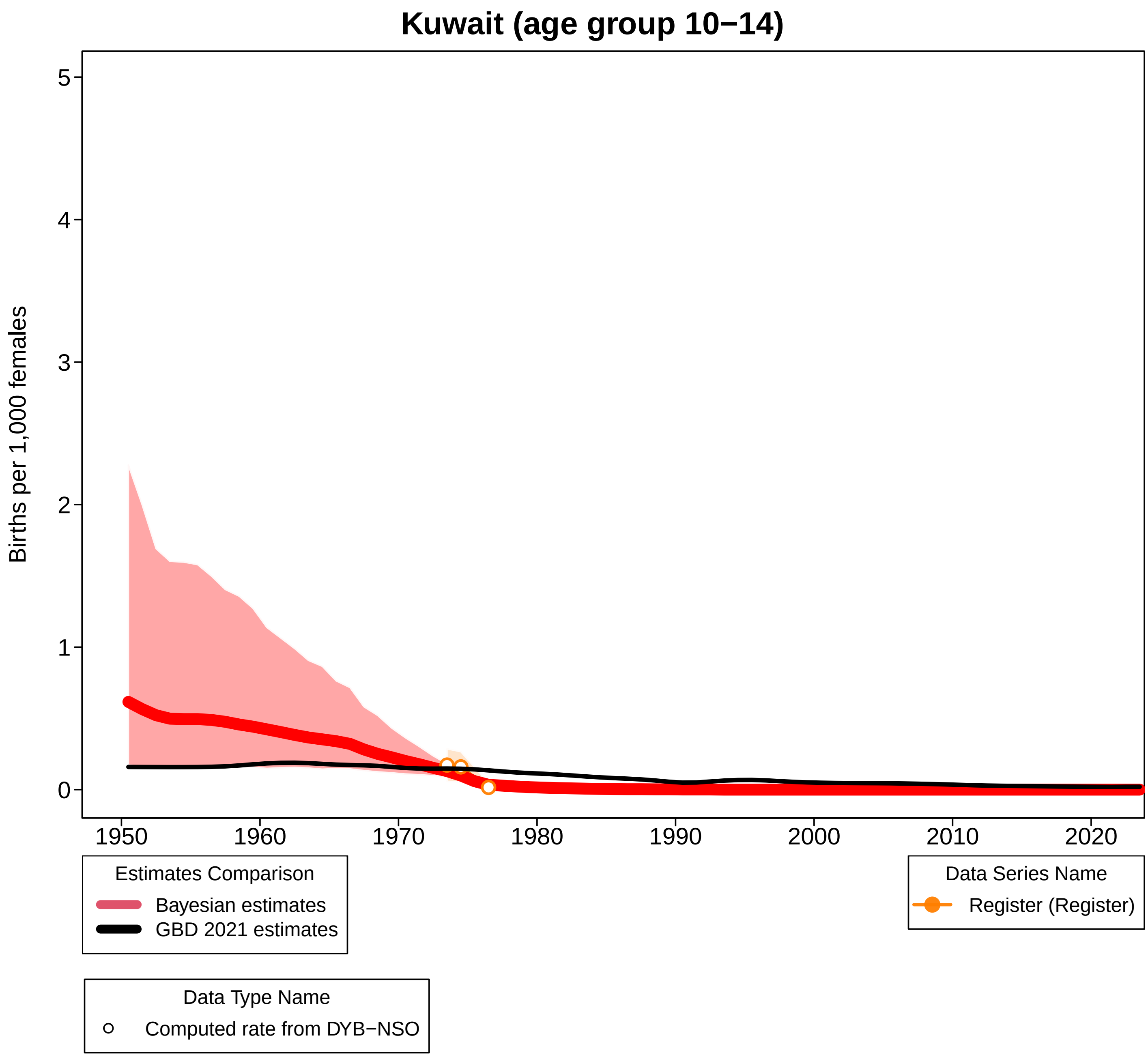
Kuwait (age group 10−14)
Births per 1,000 females
0
1
2
3
4
5
1950
1960
1970
1980
1990
2000
2010
2020
Estimates Comparison
Bayesian estimates
GBD 2021 estimates
Data Series Name
Register (Register)
Data Type Name
Computed rate from DYB−NSO

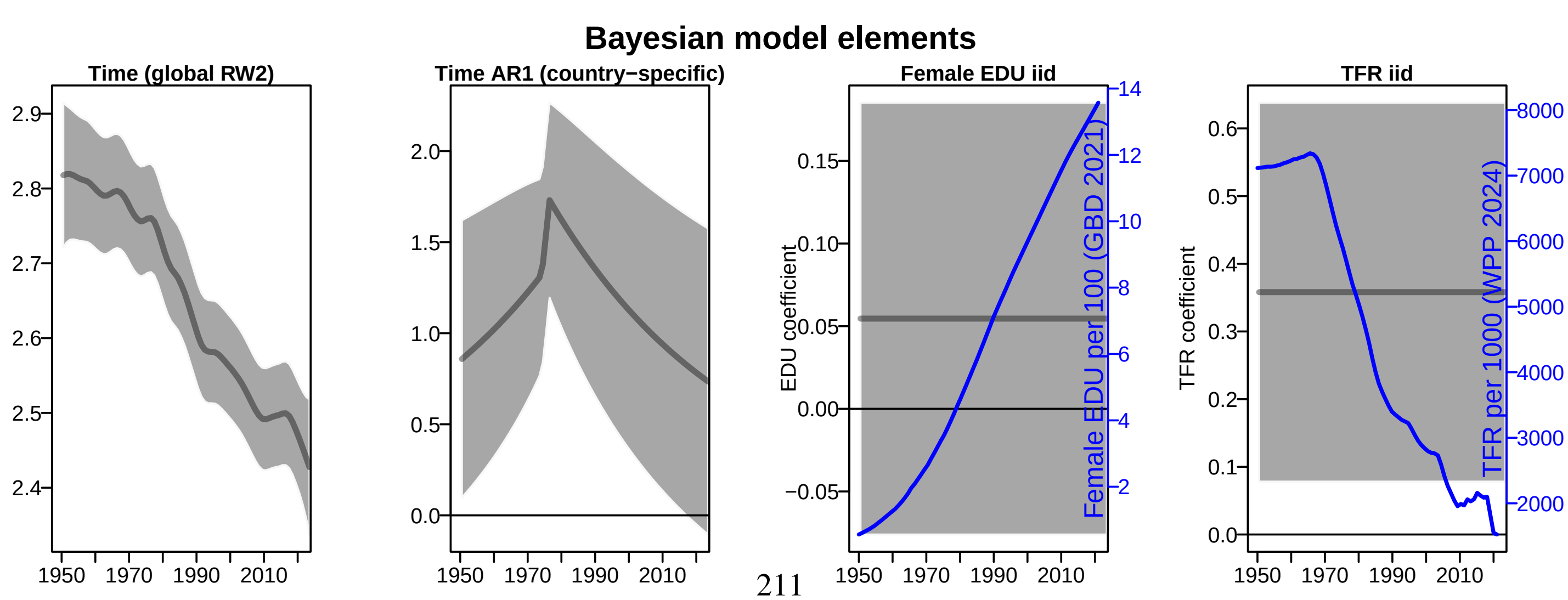
Bayesian model elements
Time (global RW2)
Time AR1 (country−specific)
Female EDU iid
TFR iid
EDU coefficient
Female EDU per 100 (GBD 2021)
TFR coefficient
TFR per 1000 (WPP 2024)
1950
1970
1990
2010

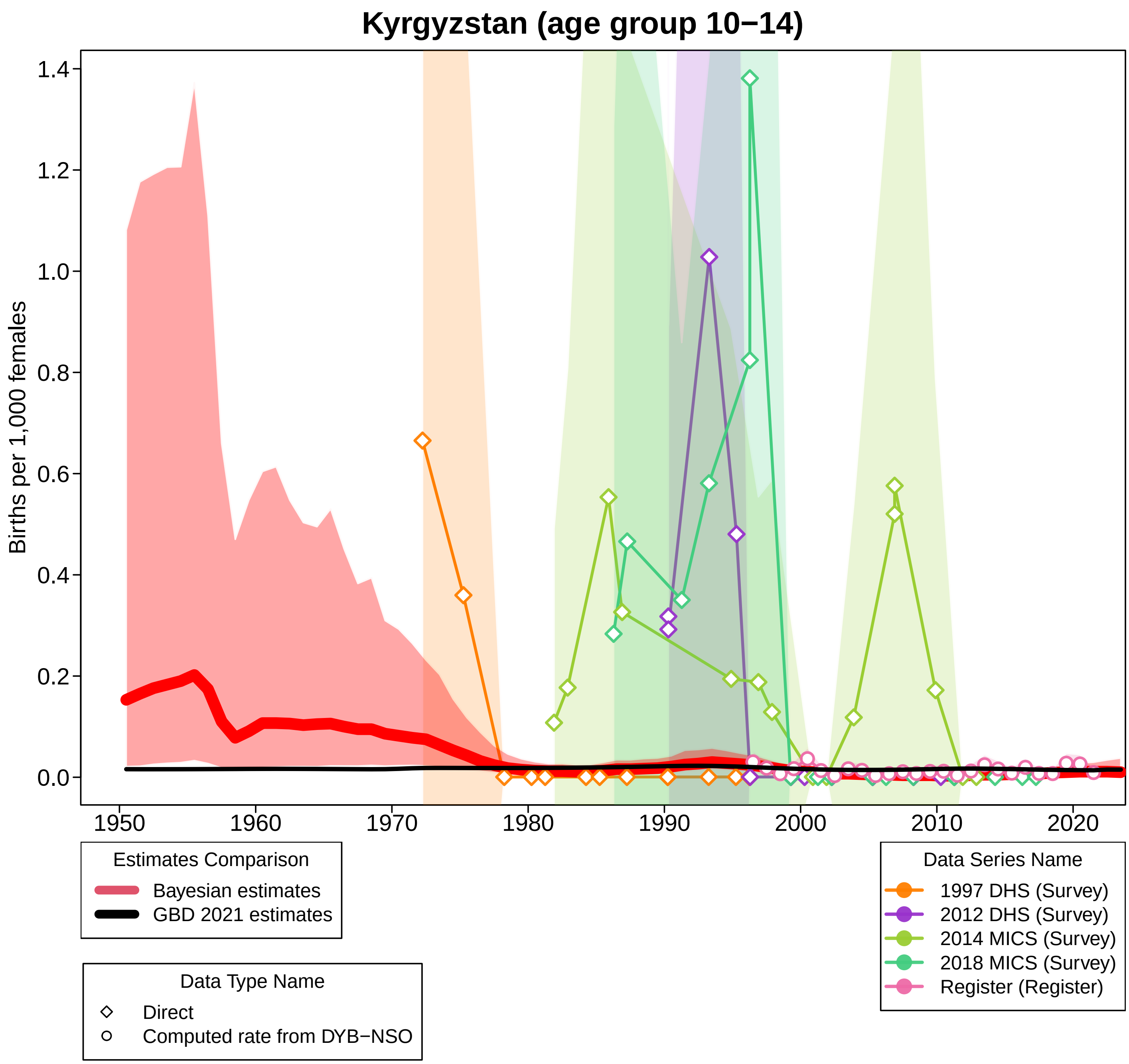

Kyrgyzstan (age group 10−14)
Births per 1,000 females
1.4
1.2
1.0
0.8
0.6
0.4
0.2
0.0
1950
1960
1970
1980
1990
2000
2010
2020
Estimates Comparison
Bayesian estimates
GBD 2021 estimates
Data Series Name
1997 DHS (Survey)
2012 DHS (Survey)
2014 MICS (Survey)
2018 MICS (Survey)
Register (Register)
Data Type Name
Direct
Computed rate from DYB−NSO

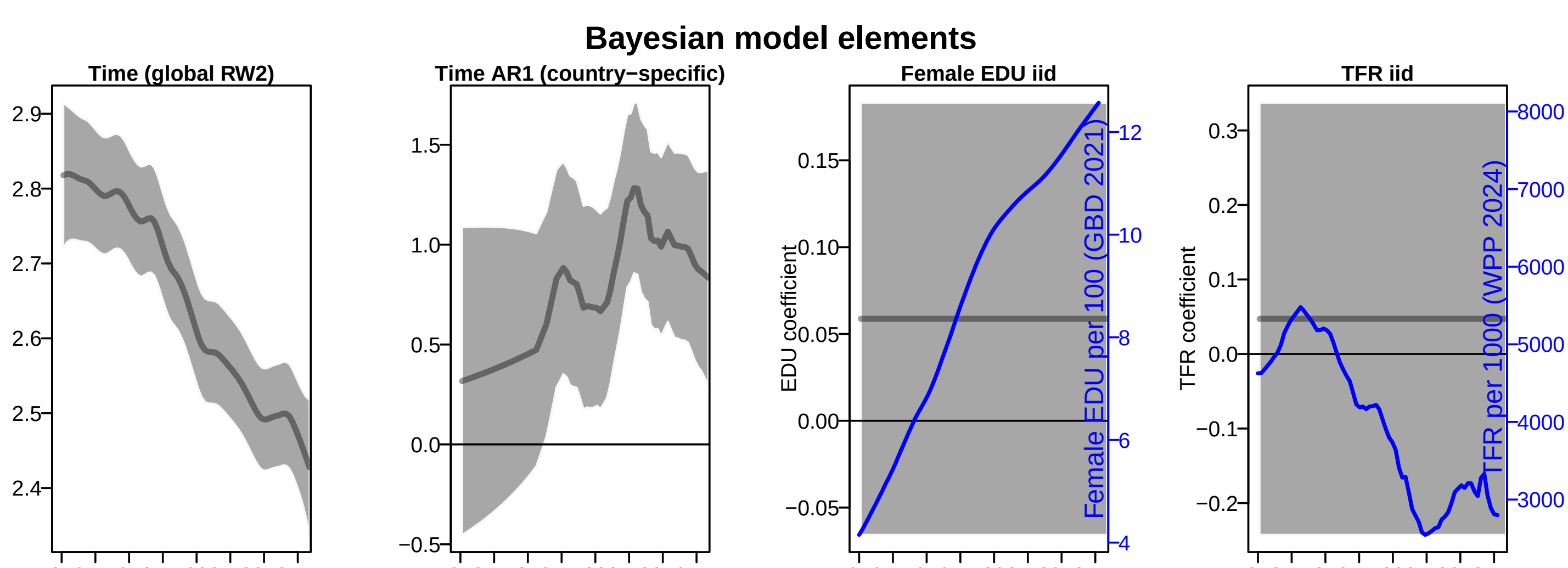

Bayesian model elements
Time (global RW2)
Time AR1 (country−specific)
Female EDU iid
TFR iid
EDU coefficient
Female EDU per 100 (GBD 2021)
TFR coefficient
TFR per 1000 (WPP 2024)
1950
1970
1990
2010

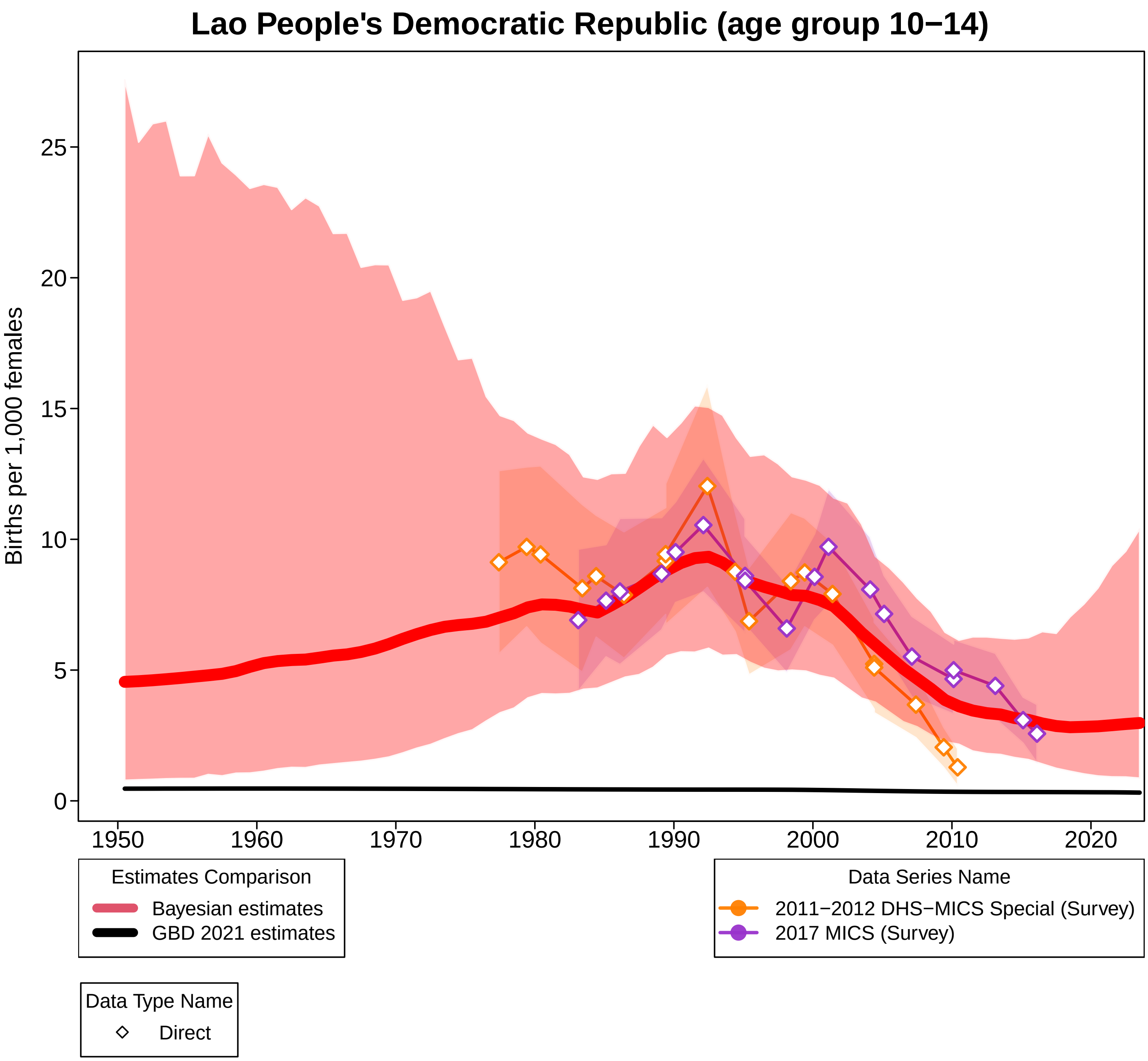
Lao People's Democratic Republic (age group 10−14)
Births per 1,000 females
0
5
10
15
20
25
1950
1960
1970
1980
1990
2000
2010
2020
Estimates Comparison
Bayesian estimates
GBD 2021 estimates
Data Series Name
2011−2012 DHS−MICS Special (Survey)
2017 MICS (Survey)
Data Type Name
Direct

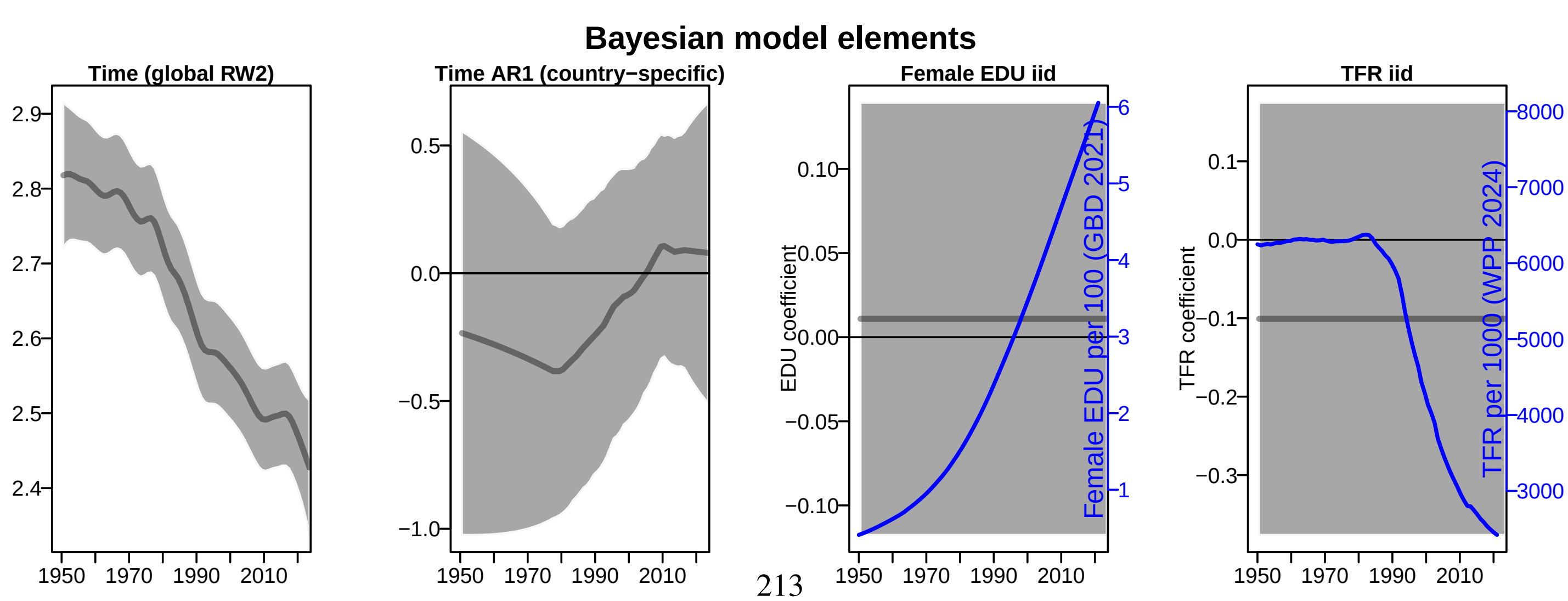
Bayesian model elements
Time (global RW2)
Time AR1 (country−specific)
Female EDU iid
TFR iid
EDU coefficient
Female EDU per 100 (GBD 2021)
TFR coefficient
TFR per 1000 (WPP 2024)
1950
1970
1990
2010

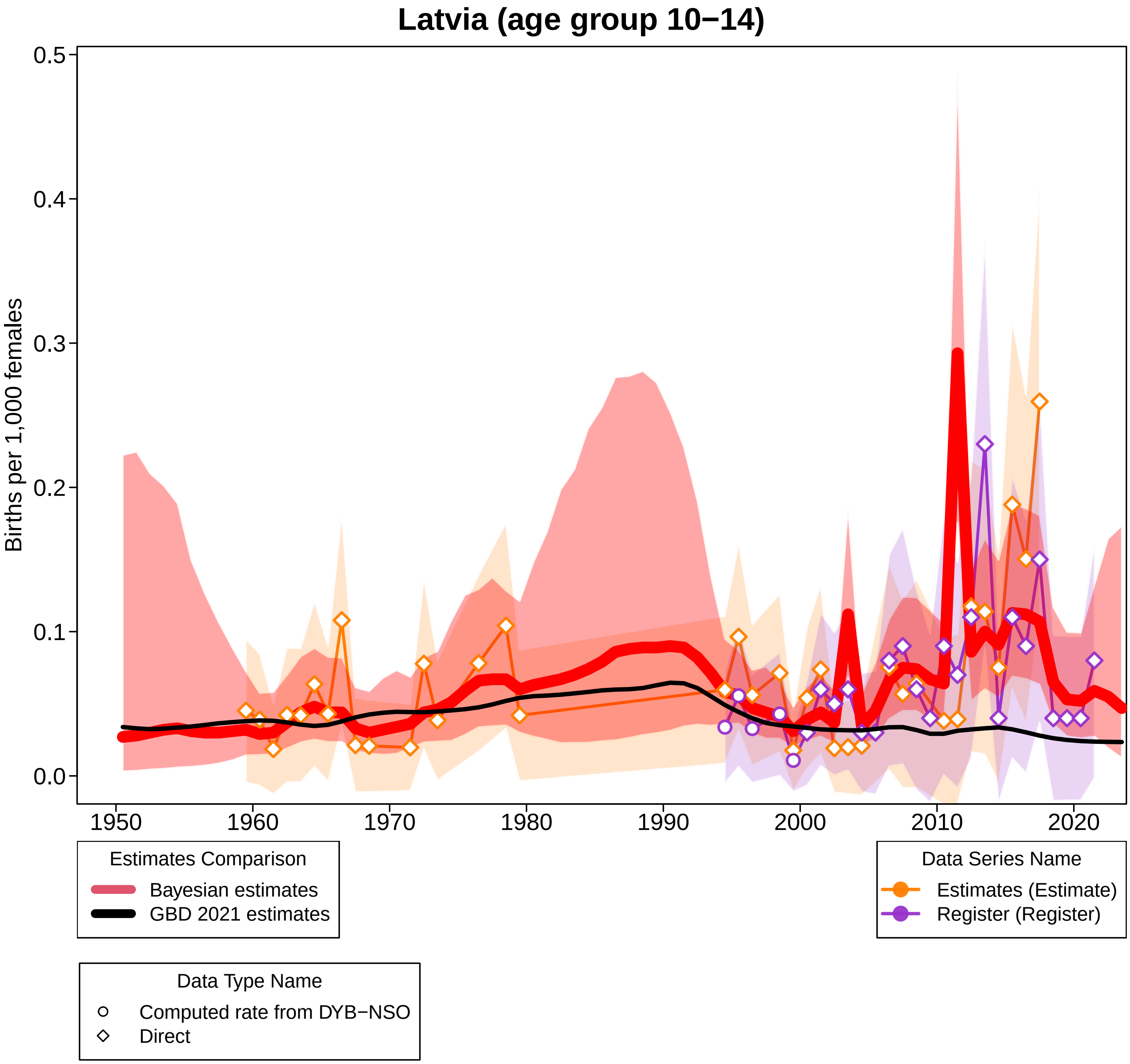

Latvia (age group 10−14)
Births per 1,000 females
0.0
0.1
0.2
0.3
0.4
0.5
1950
1960
1970
1980
1990
2000
2010
2020
Estimates Comparison
Bayesian estimates
GBD 2021 estimates
Data Series Name
Estimates (Estimate)
Register (Register)
Data Type Name
Computed rate from DYB−NSO
Direct

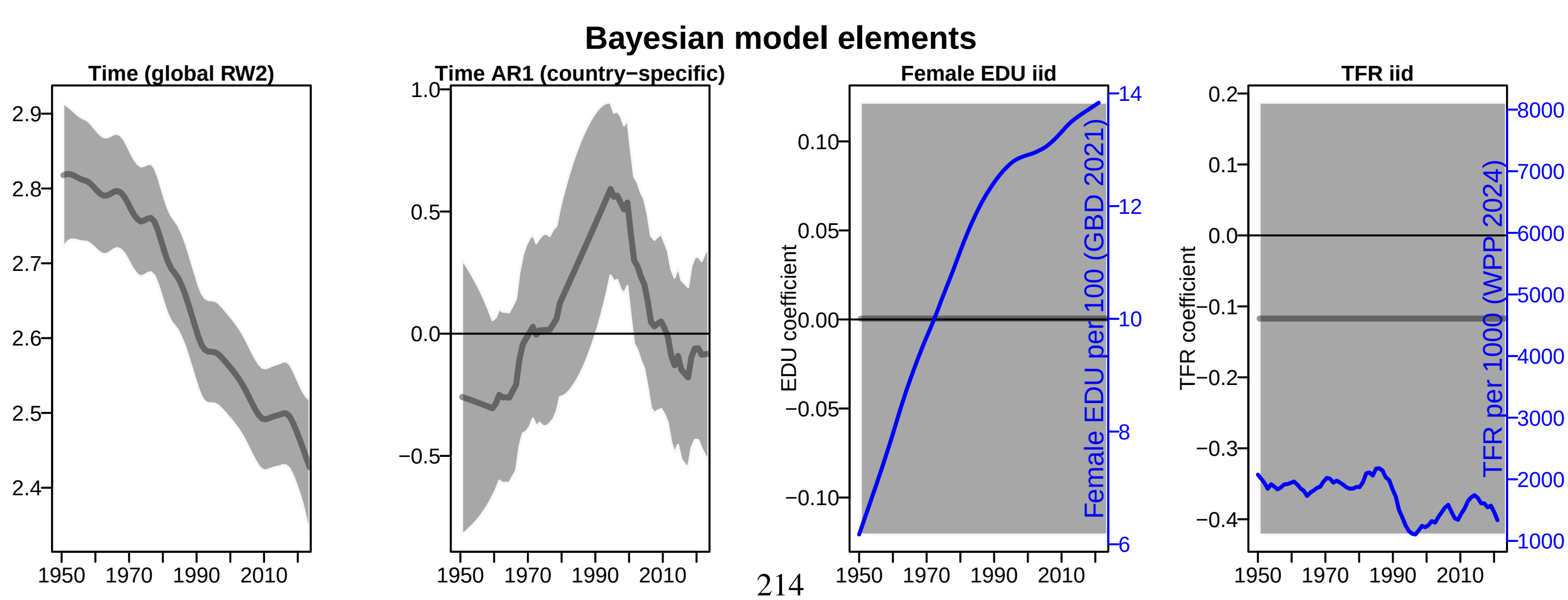

Bayesian model elements
Time (global RW2)
Time AR1 (country−specific)
Female EDU iid
TFR iid
EDU coefficient
Female EDU per 100 (GBD 2021)
TFR coefficient
TFR per 1000 (WPP 2024)
1950
1970
1990
2010

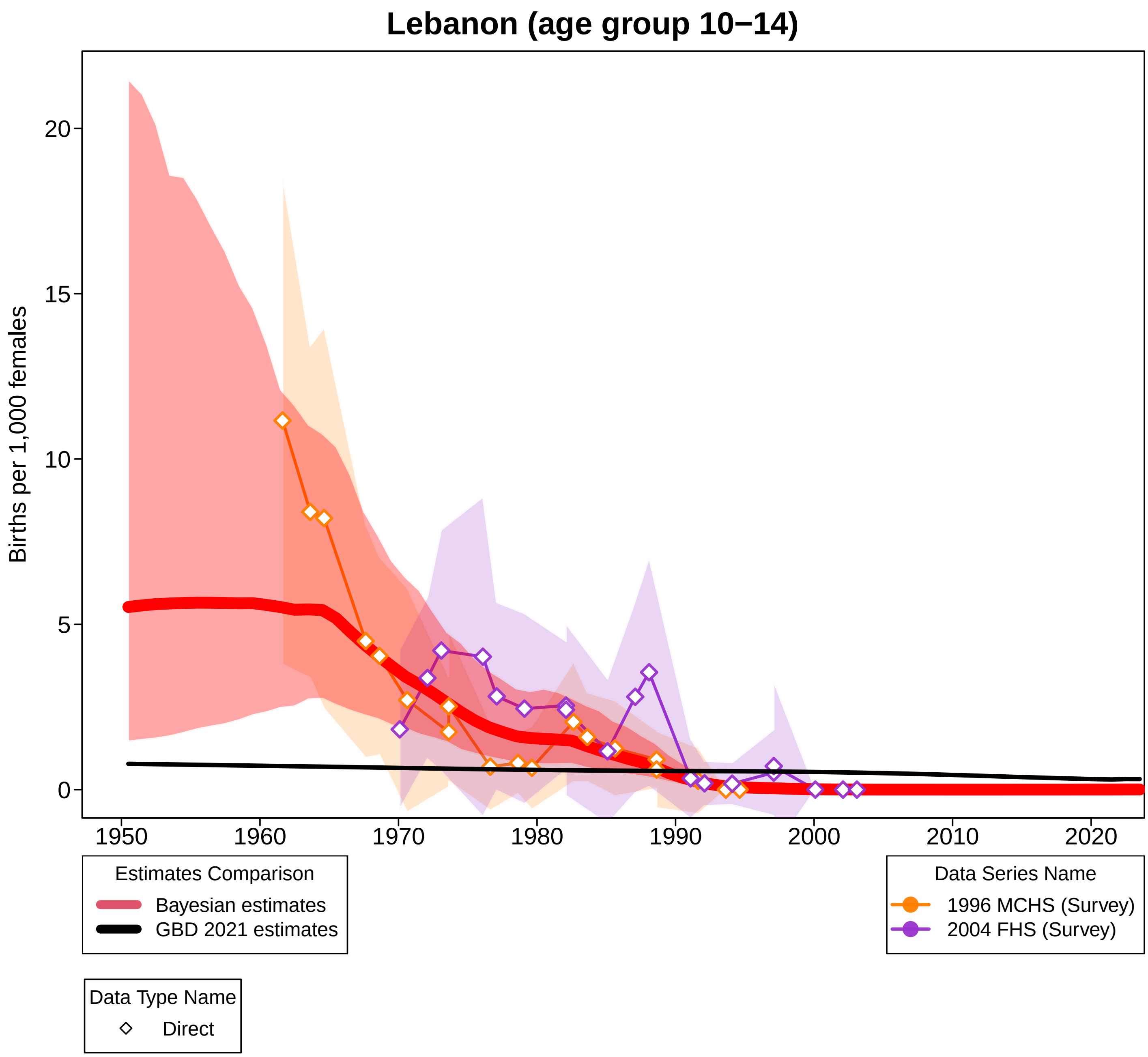
Lebanon (age group 10−14)
Births per 1,000 females
20
15
10
5
0
1950
1960
1970
1980
1990
2000
2010
2020
Estimates Comparison
Bayesian estimates
GBD 2021 estimates
Data Series Name
1996 MCHS (Survey)
2004 FHS (Survey)
Data Type Name
Direct

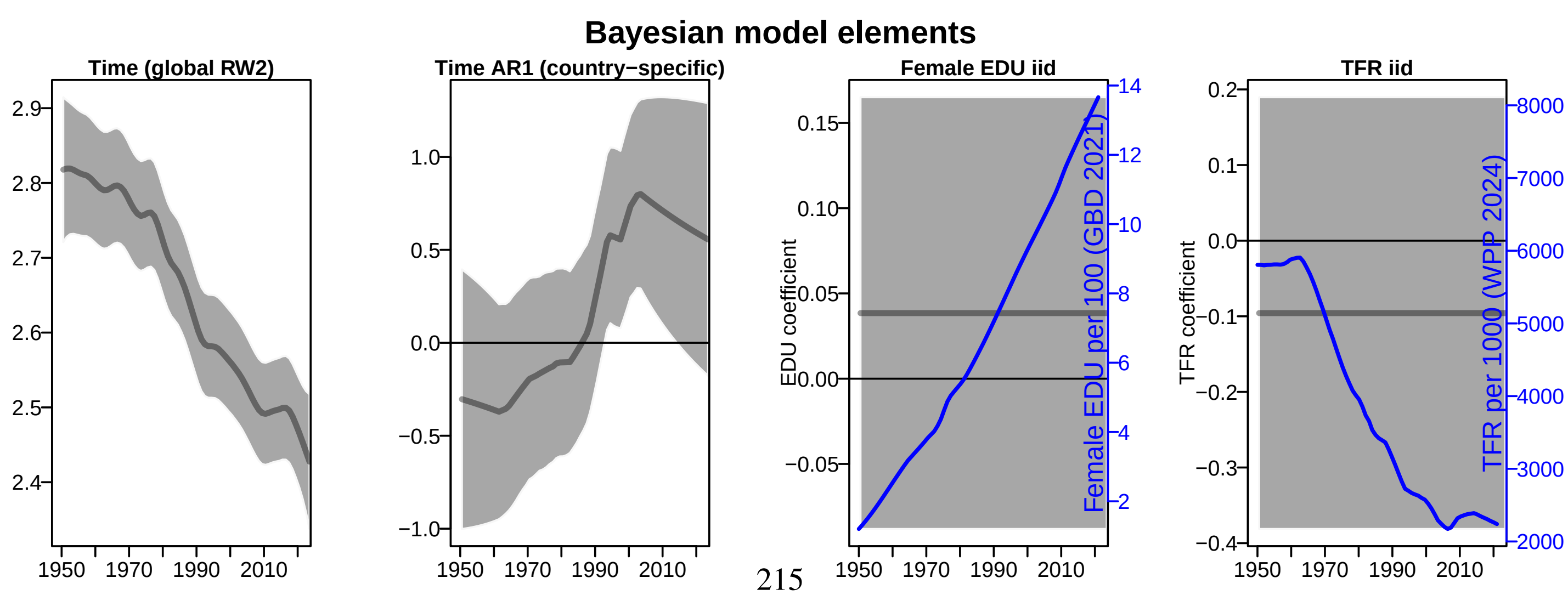
Bayesian model elements
Time (global RW2)
Time AR1 (country−specific)
Female EDU iid
TFR iid
EDU coefficient
Female EDU per 100 (GBD 2021)
TFR coefficient
TFR per 1000 (WPP 2024)
1950
1970
1990
2010

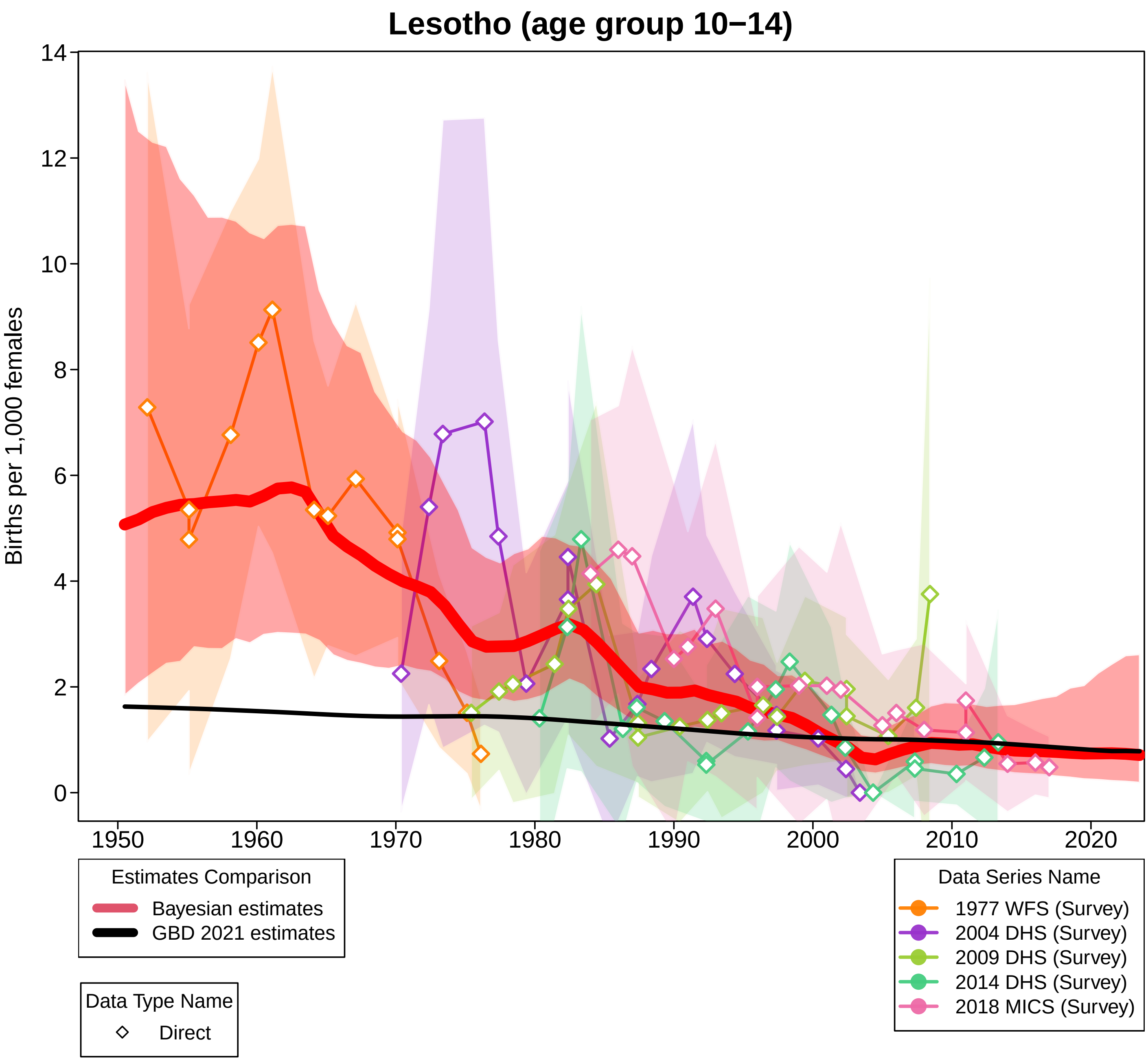

Lesotho (age group 10−14)
Births per 1,000 females
14
12
10
8
6
4
2
0
1950
1960
1970
1980
1990
2000
2010
2020
Estimates Comparison
Bayesian estimates
GBD 2021 estimates
Data Type Name
Direct
Data Series Name
1977 WFS (Survey)
2004 DHS (Survey)
2009 DHS (Survey)
2014 DHS (Survey)
2018 MICS (Survey)

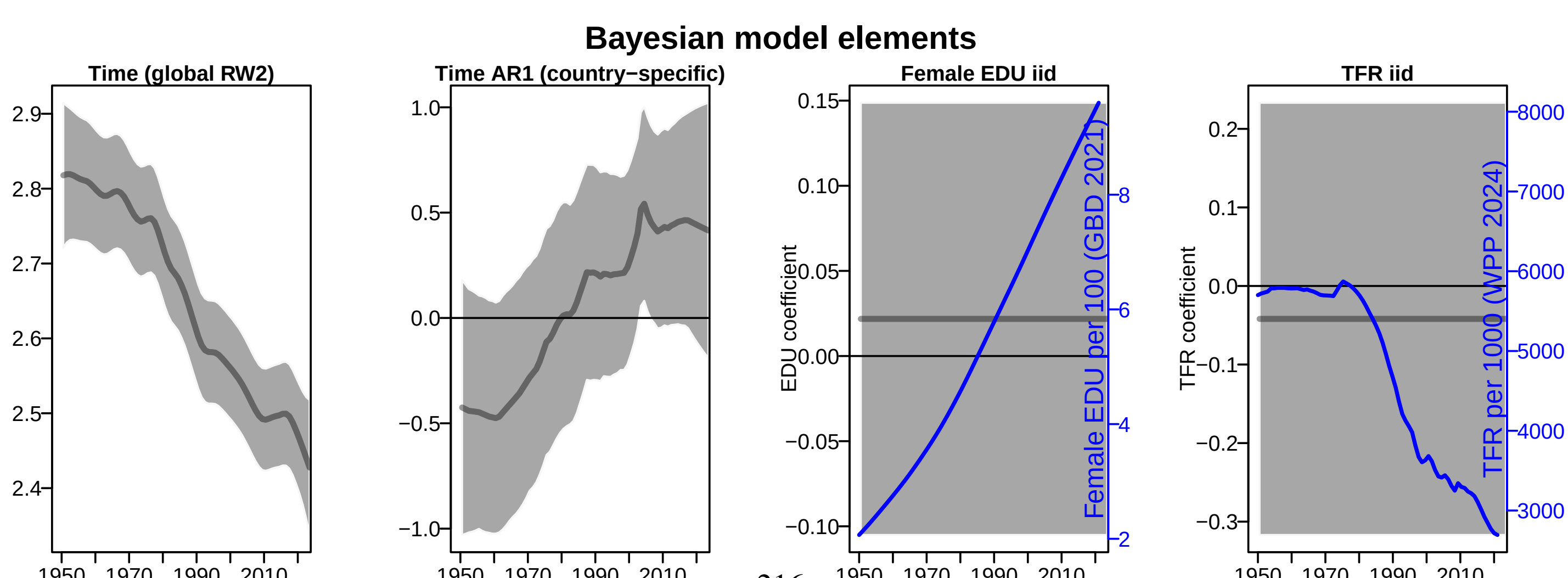

Bayesian model elements
Time (global RW2)
Time AR1 (country−specific)
Female EDU iid
TFR iid
EDU coefficient
Female EDU per 100 (GBD 2021)
TFR coefficient
TFR per 1000 (WPP 2024)

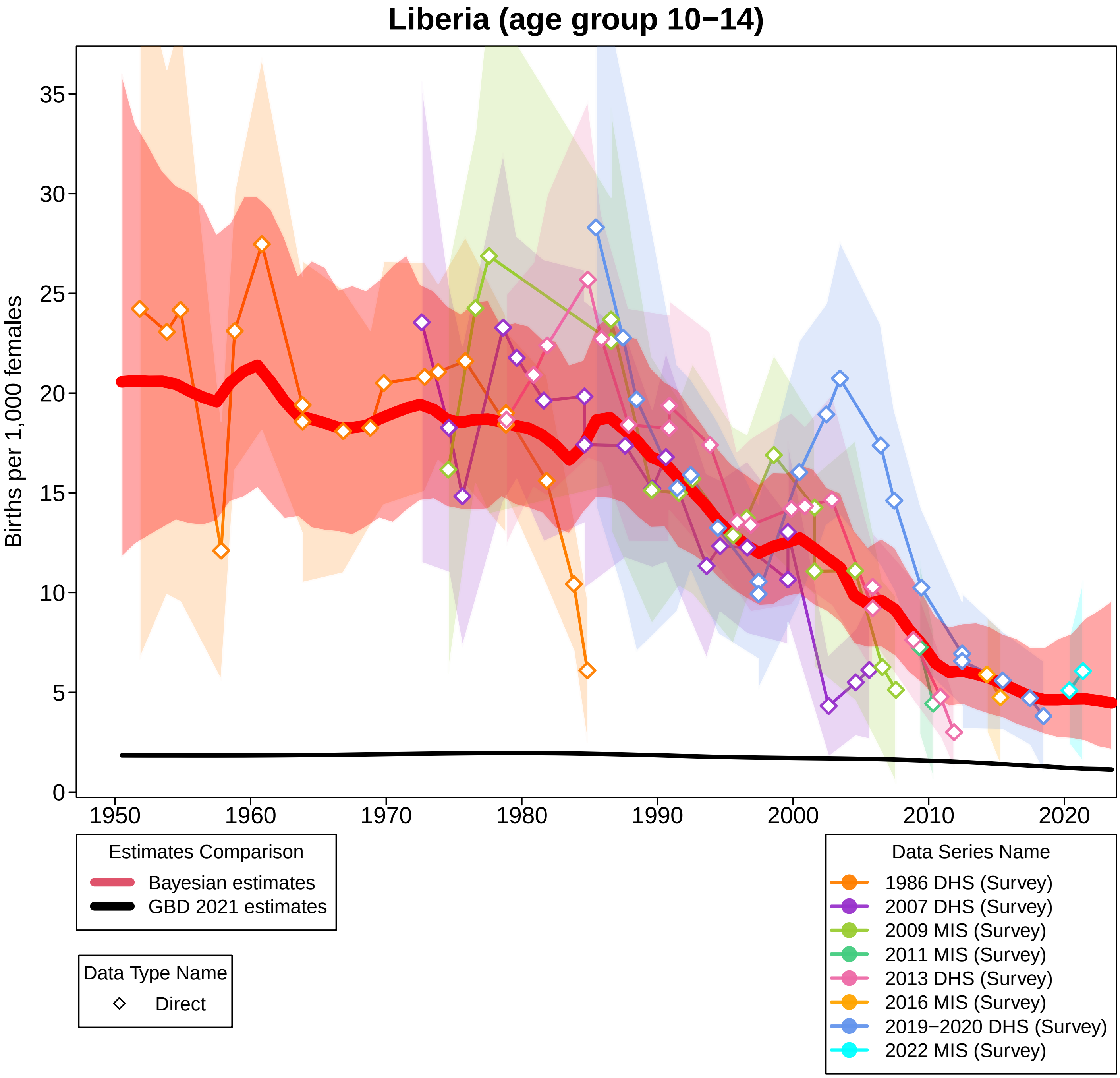

Liberia (age group 10−14)
Births per 1,000 females
35
30
25
20
15
10
5
0
1950
1960
1970
1980
1990
2000
2010
2020
Estimates Comparison
Bayesian estimates
GBD 2021 estimates
Data Type Name
Direct
Data Series Name
1986 DHS (Survey)
2007 DHS (Survey)
2009 MIS (Survey)
2011 MIS (Survey)
2013 DHS (Survey)
2016 MIS (Survey)
2019−2020 DHS (Survey)
2022 MIS (Survey)

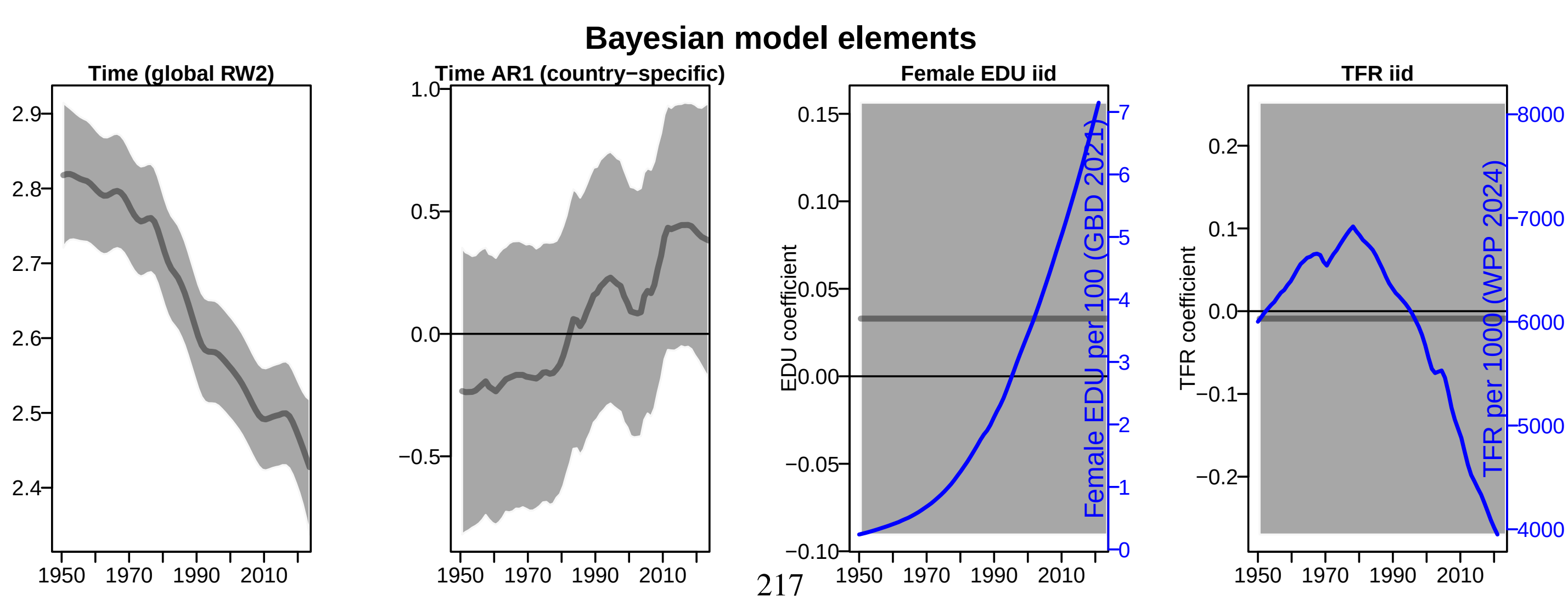

Bayesian model elements
Time (global RW2)
Time AR1 (country−specific)
Female EDU iid
TFR iid
EDU coefficient
Female EDU per 100 (GBD 2021)
TFR coefficient
TFR per 1000 (WPP 2024)
1950
1970
1990
2010

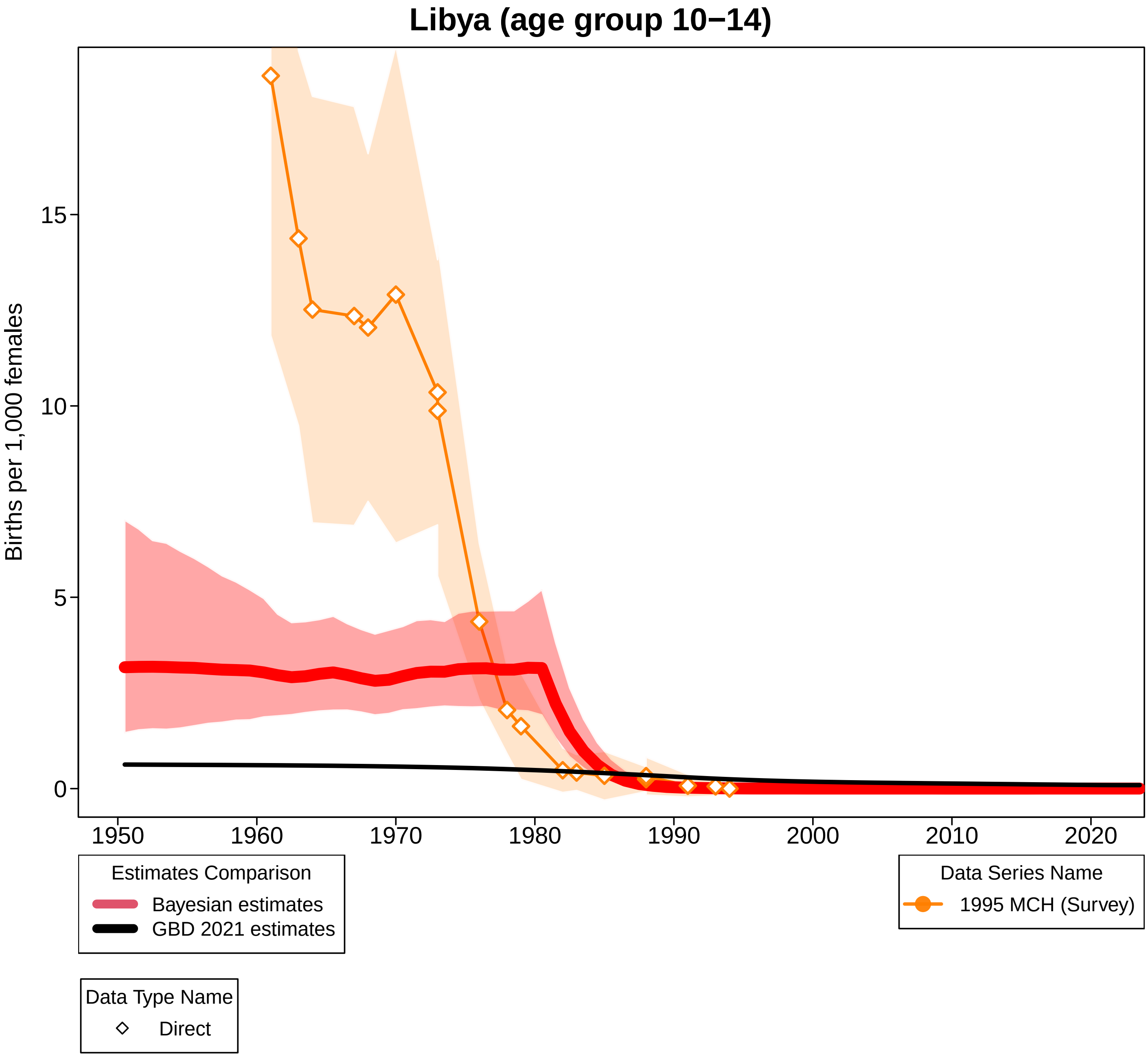

Libya (age group 10−14)
Births per 1,000 females
0
5
10
15
1950
1960
1970
1980
1990
2000
2010
2020
Estimates Comparison
Bayesian estimates
GBD 2021 estimates
Data Series Name
1995 MCH (Survey)
Data Type Name
Direct

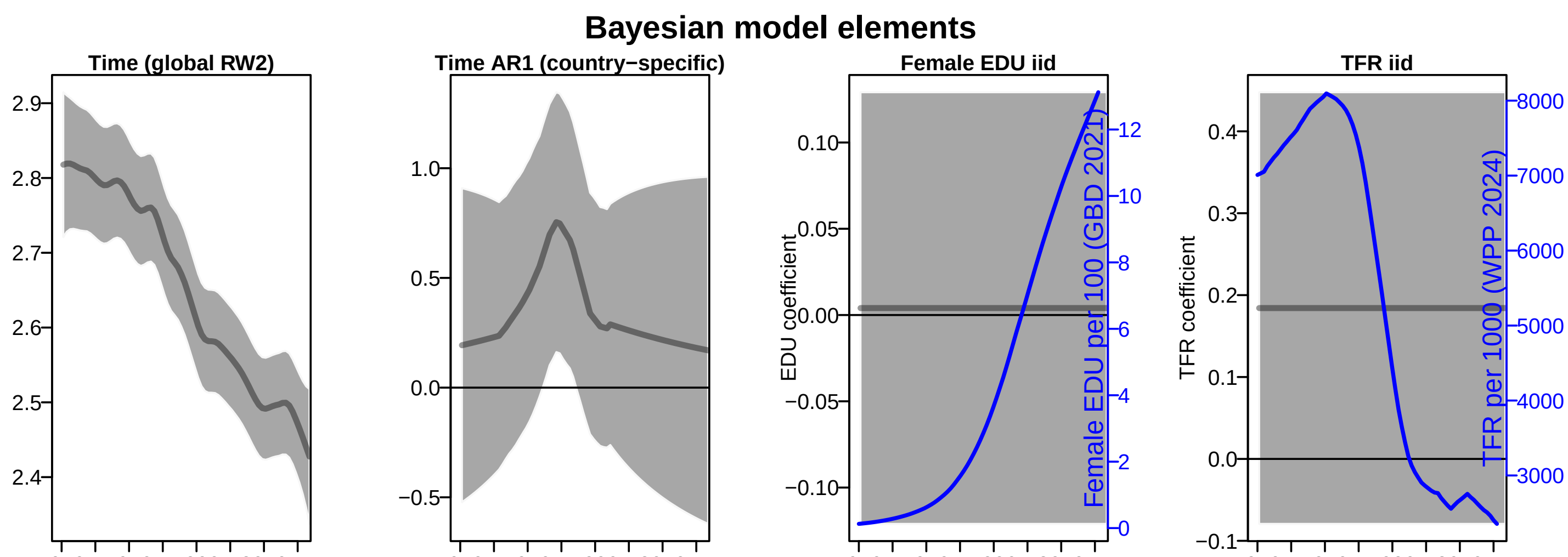

Bayesian model elements
Time (global RW2)
Time AR1 (country−specific)
Female EDU iid
TFR iid
EDU coefficient
Female EDU per 100 (GBD 2021)
TFR coefficient
TFR per 1000 (WPP 2024)
1950
1970
1990
2010

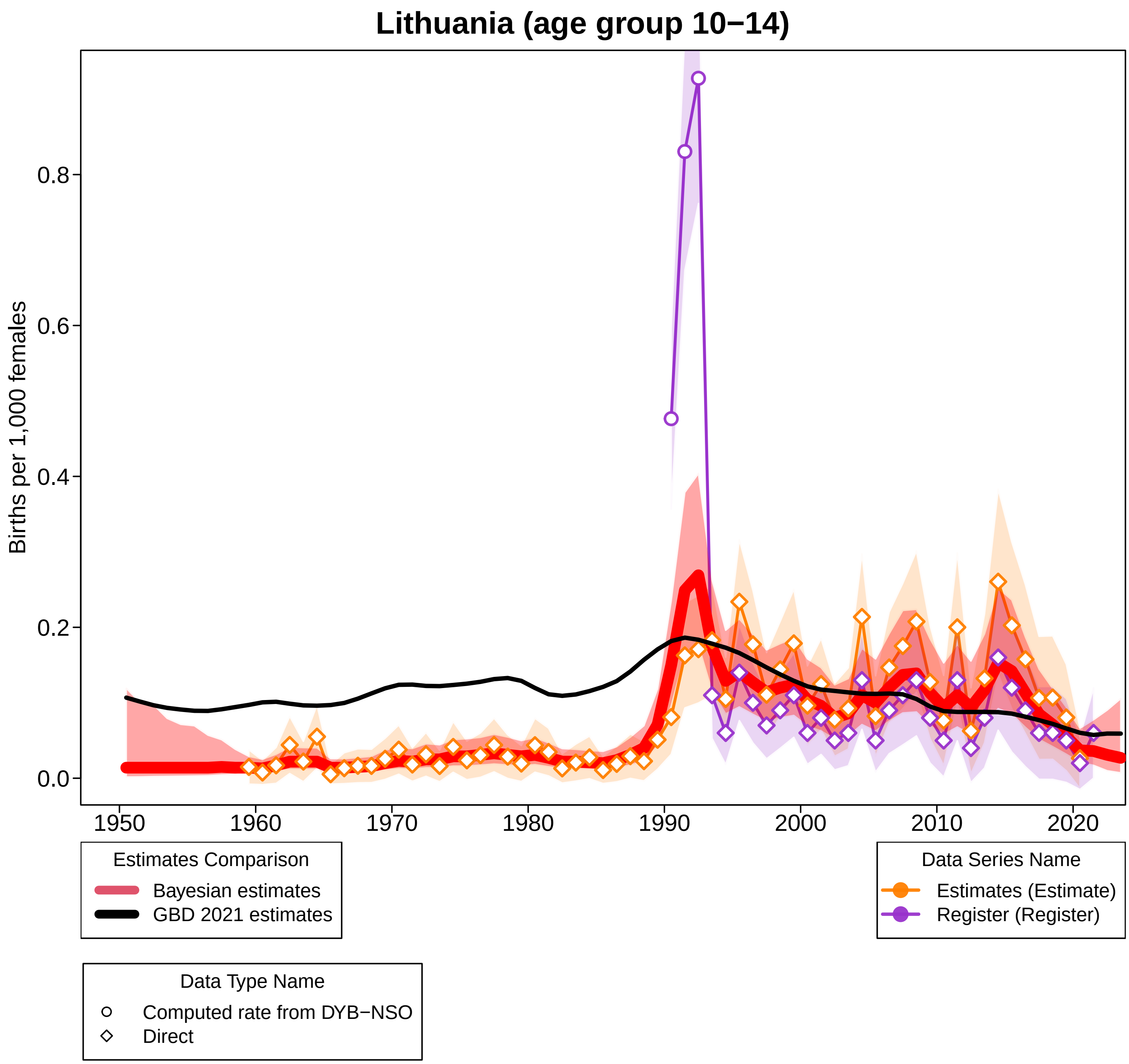
Lithuania (age group 10−14)
Births per 1,000 females
0.0
0.2
0.4
0.6
0.8
1950
1960
1970
1980
1990
2000
2010
2020
Estimates Comparison
Bayesian estimates
GBD 2021 estimates
Data Series Name
Estimates (Estimate)
Register (Register)
Data Type Name
Computed rate from DYB−NSO
Direct

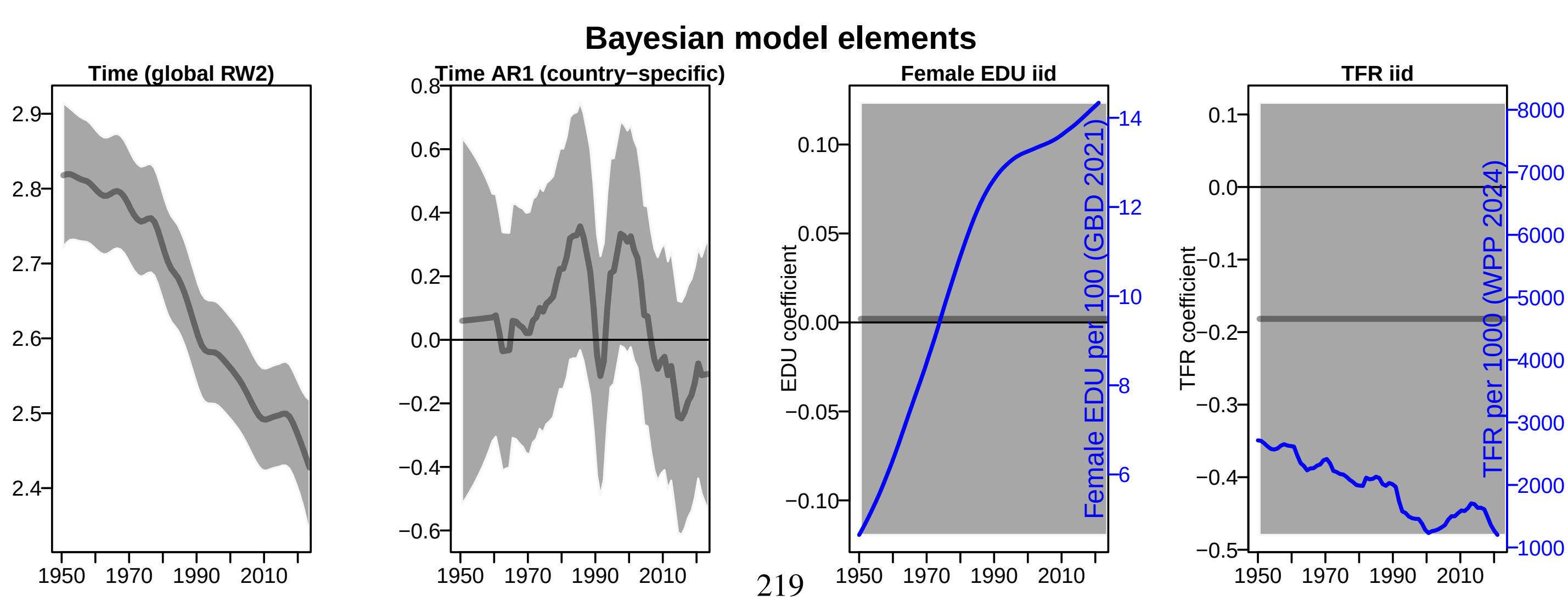
Bayesian model elements
Time (global RW2)
Time AR1 (country−specific)
Female EDU iid
TFR iid
EDU coefficient
Female EDU per 100 (GBD 2021)
TFR coefficient
TFR per 1000 (WPP 2024)

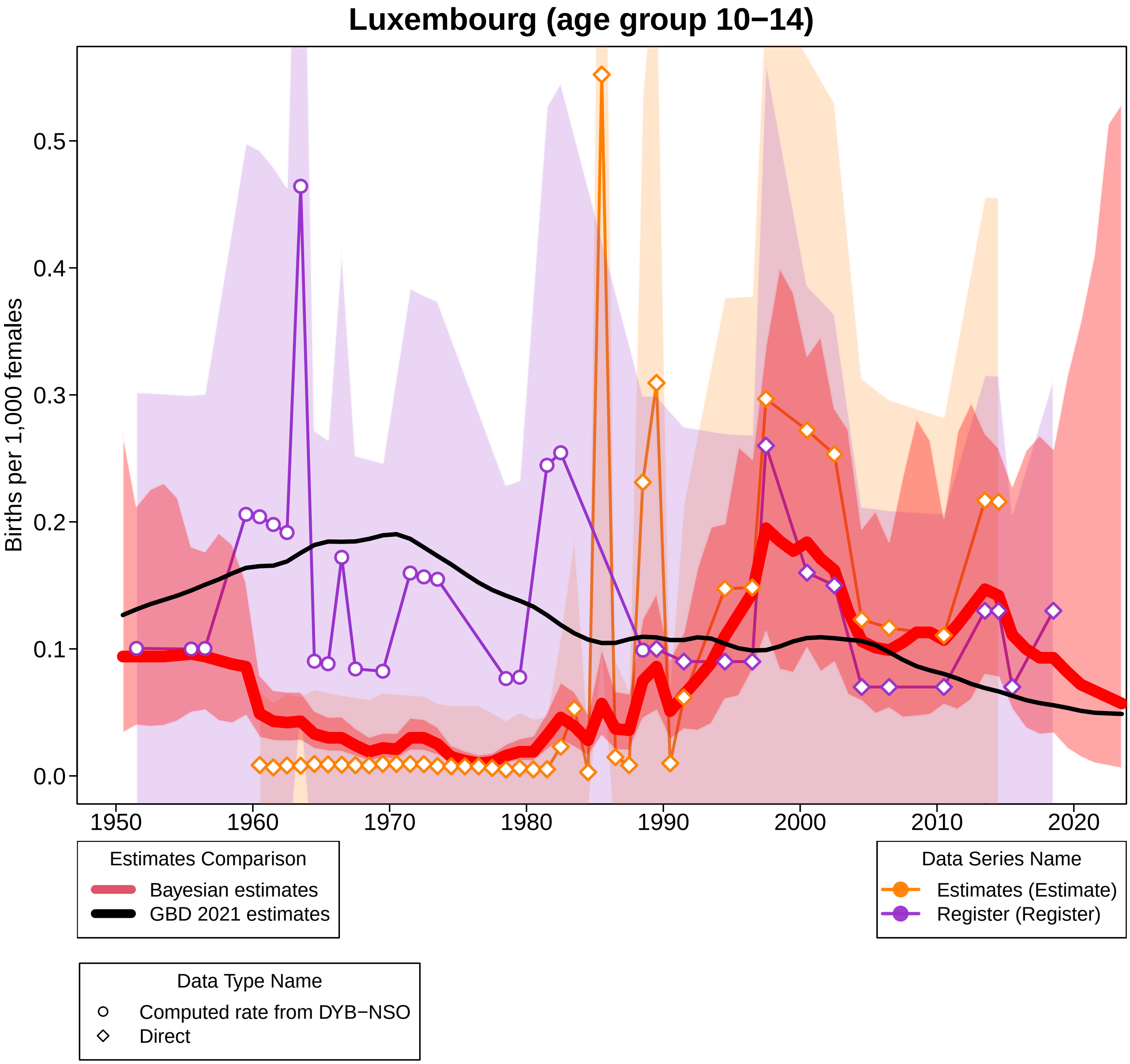
Luxembourg (age group 10−14)
Births per 1,000 females
0.0
0.1
0.2
0.3
0.4
0.5
1950
1960
1970
1980
1990
2000
2010
2020
Estimates Comparison
Bayesian estimates
GBD 2021 estimates
Data Series Name
Estimates (Estimate)
Register (Register)
Data Type Name
Computed rate from DYB−NSO
Direct

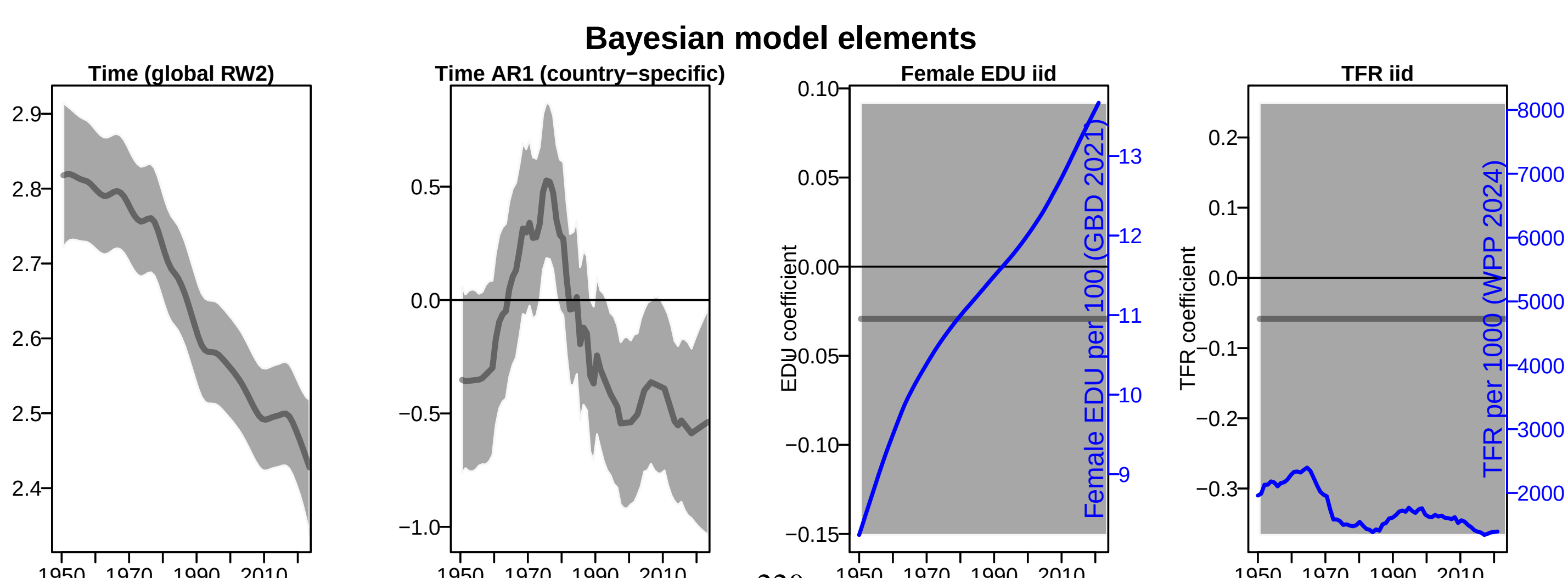
Bayesian model elements
Time (global RW2)
Time AR1 (country−specific)
Female EDU iid
TFR iid
EDU coefficient
Female EDU per 100 (GBD 2021)
TFR coefficient
TFR per 1000 (WPP 2024)
1950
1970
1990
2010

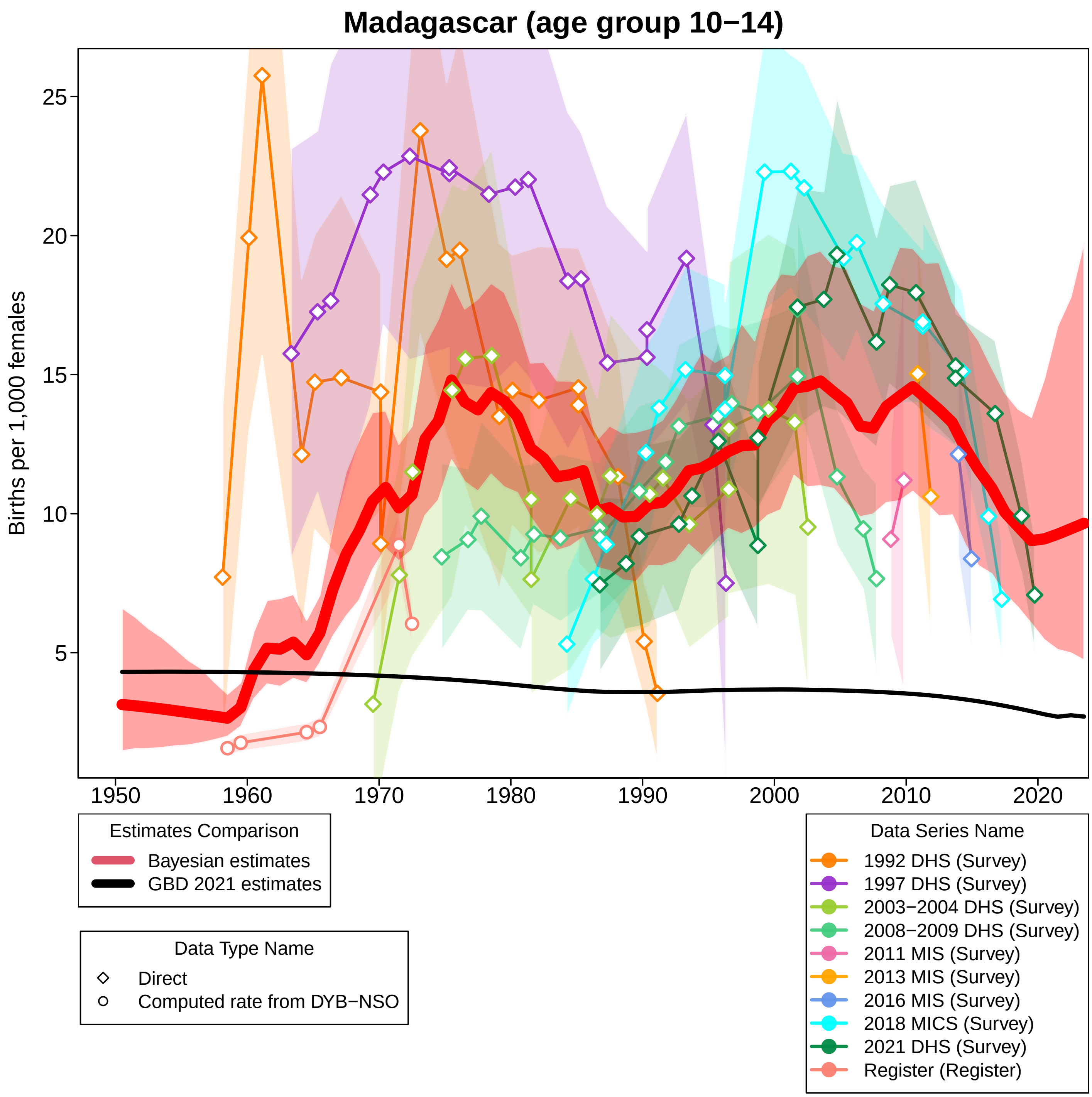
Madagascar (age group 10−14)
Births per 1,000 females
25
20
15
10
5
1950
1960
1970
1980
1990
2000
2010
2020
Estimates Comparison
Bayesian estimates
GBD 2021 estimates
Data Type Name
Direct
Computed rate from DYB−NSO
Data Series Name
1992 DHS (Survey)
1997 DHS (Survey)
2003−2004 DHS (Survey)
2008−2009 DHS (Survey)
2011 MIS (Survey)
2013 MIS (Survey)
2016 MIS (Survey)
2018 MICS (Survey)
2021 DHS (Survey)
Register (Register)

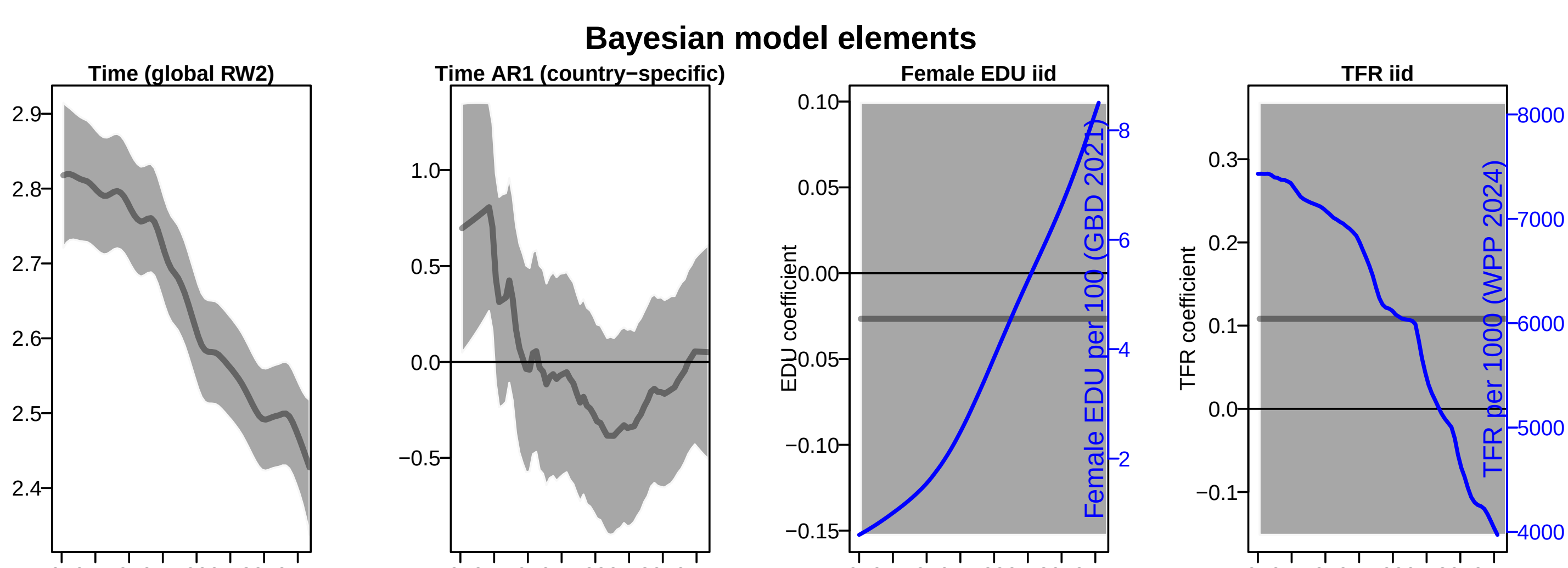
Bayesian model elements
Time (global RW2)
Time AR1 (country−specific)
Female EDU iid
TFR iid
EDU coefficient
Female EDU per 100 (GBD 2021)
TFR coefficient
TFR per 1000 (WPP 2024)
1950
1970
1990
2010

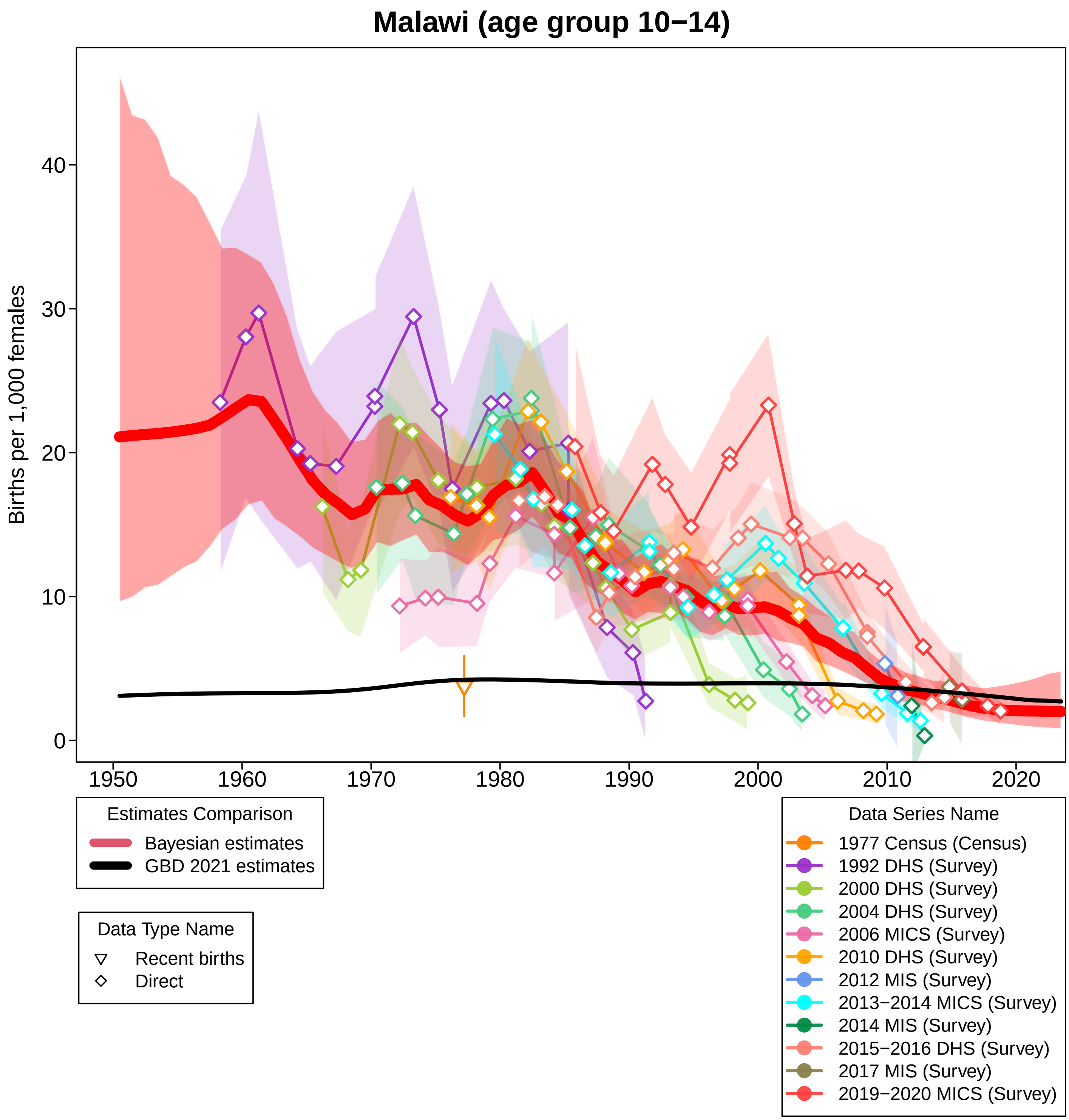
Malawi (age group 10−14)
Births per 1,000 females
0
10
20
30
40
1950
1960
1970
1980
1990
2000
2010
2020
Estimates Comparison
Bayesian estimates
GBD 2021 estimates
Data Type Name
Recent births
Direct
Data Series Name
1977 Census (Census)
1992 DHS (Survey)
2000 DHS (Survey)
2004 DHS (Survey)
2006 MICS (Survey)
2010 DHS (Survey)
2012 MIS (Survey)
2013−2014 MICS (Survey)
2014 MIS (Survey)
2015−2016 DHS (Survey)
2017 MIS (Survey)
2019−2020 MICS (Survey)

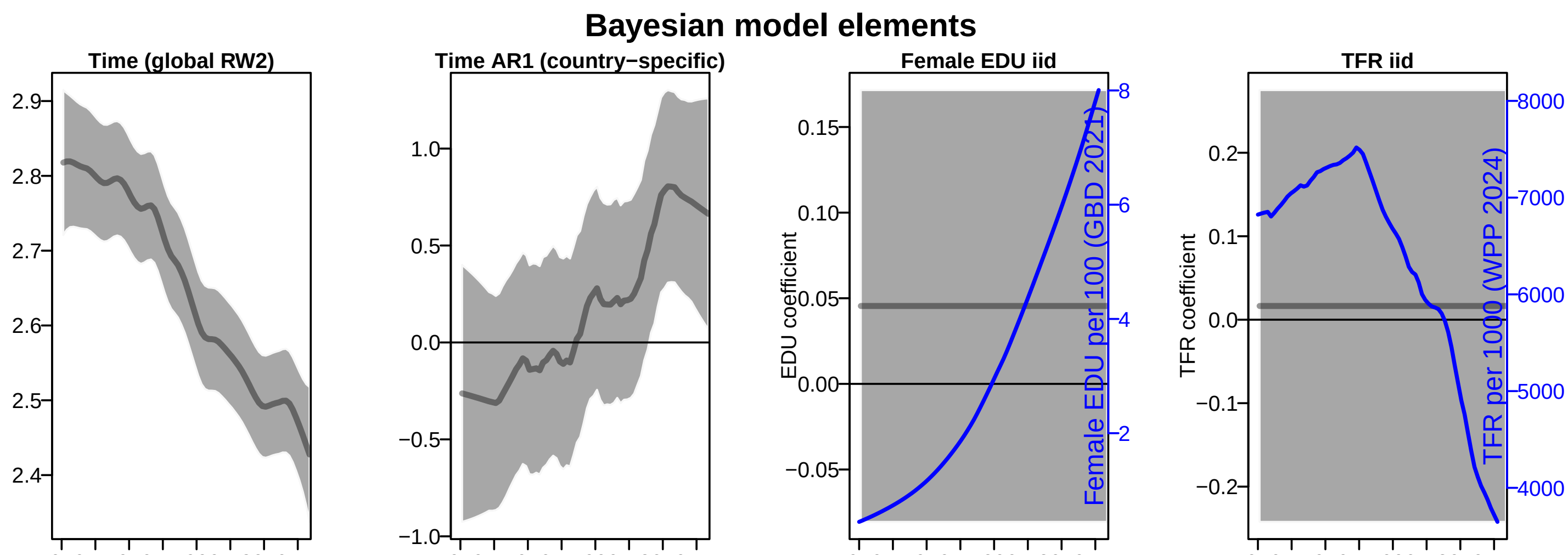
Bayesian model elements
Time (global RW2)
Time AR1 (country−specific)
Female EDU iid
TFR iid
EDU coefficient
Female EDU per 100 (GBD 2021)
TFR coefficient
TFR per 1000 (WPP 2024)
1950
1970
1990
2010

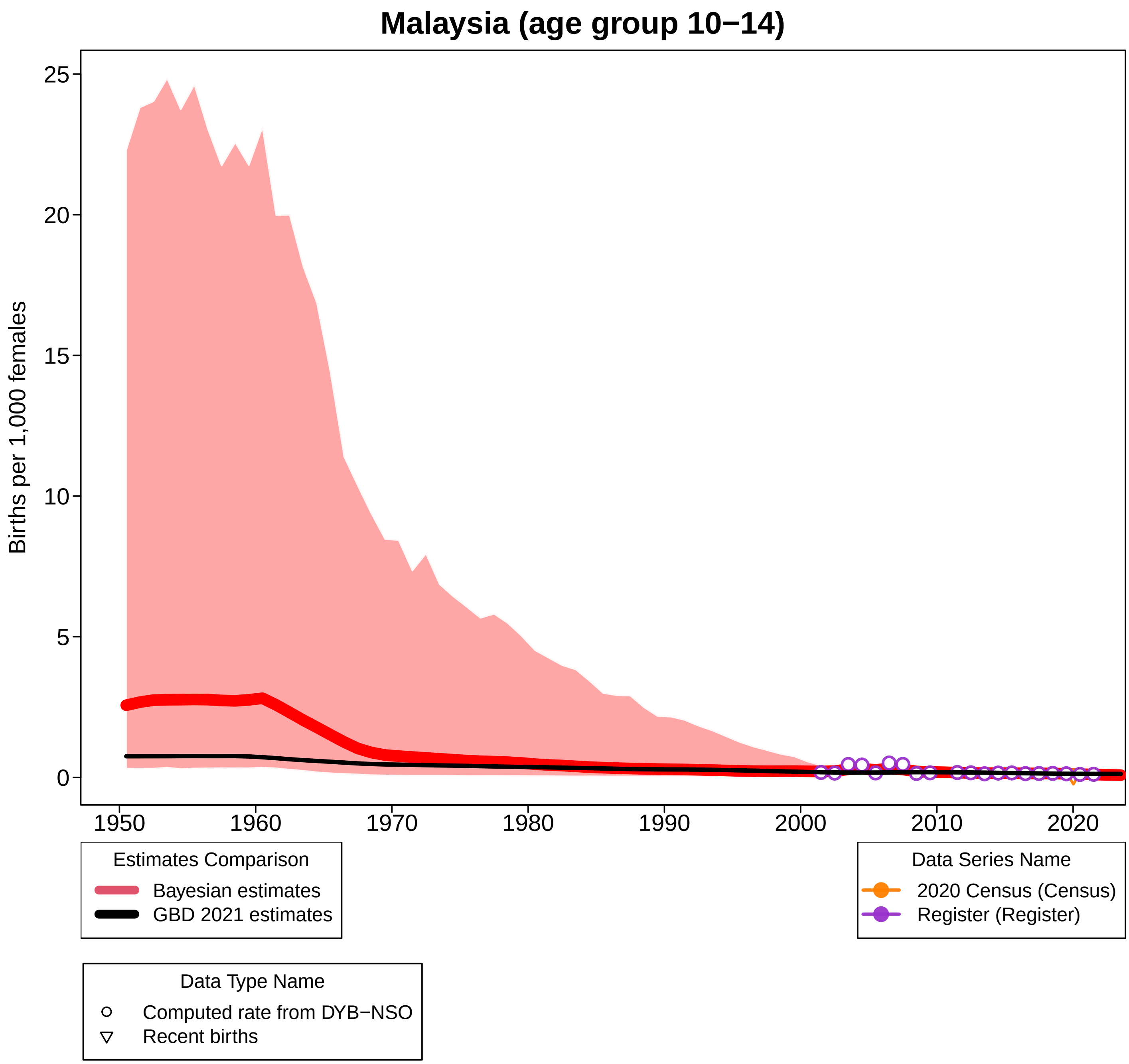

Malaysia (age group 10−14)
Births per 1,000 females
0
5
10
15
20
25
1950
1960
1970
1980
1990
2000
2010
2020
Estimates Comparison
Bayesian estimates
GBD 2021 estimates
Data Series Name
2020 Census (Census)
Register (Register)
Data Type Name
Computed rate from DYB−NSO
Recent births

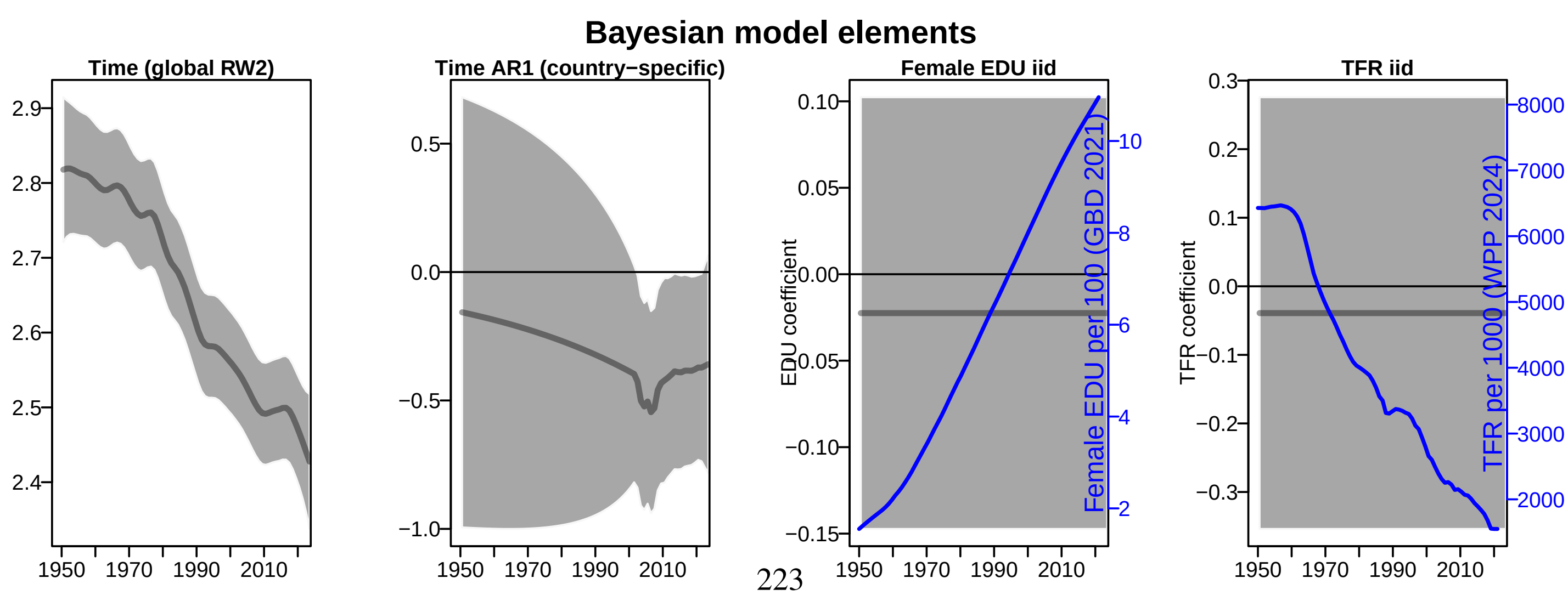

Bayesian model elements
Time (global RW2)
Time AR1 (country−specific)
Female EDU iid
TFR iid
EDU coefficient
Female EDU per 100 (GBD 2021)
TFR coefficient
TFR per 1000 (WPP 2024)
1950
1970
1990
2010

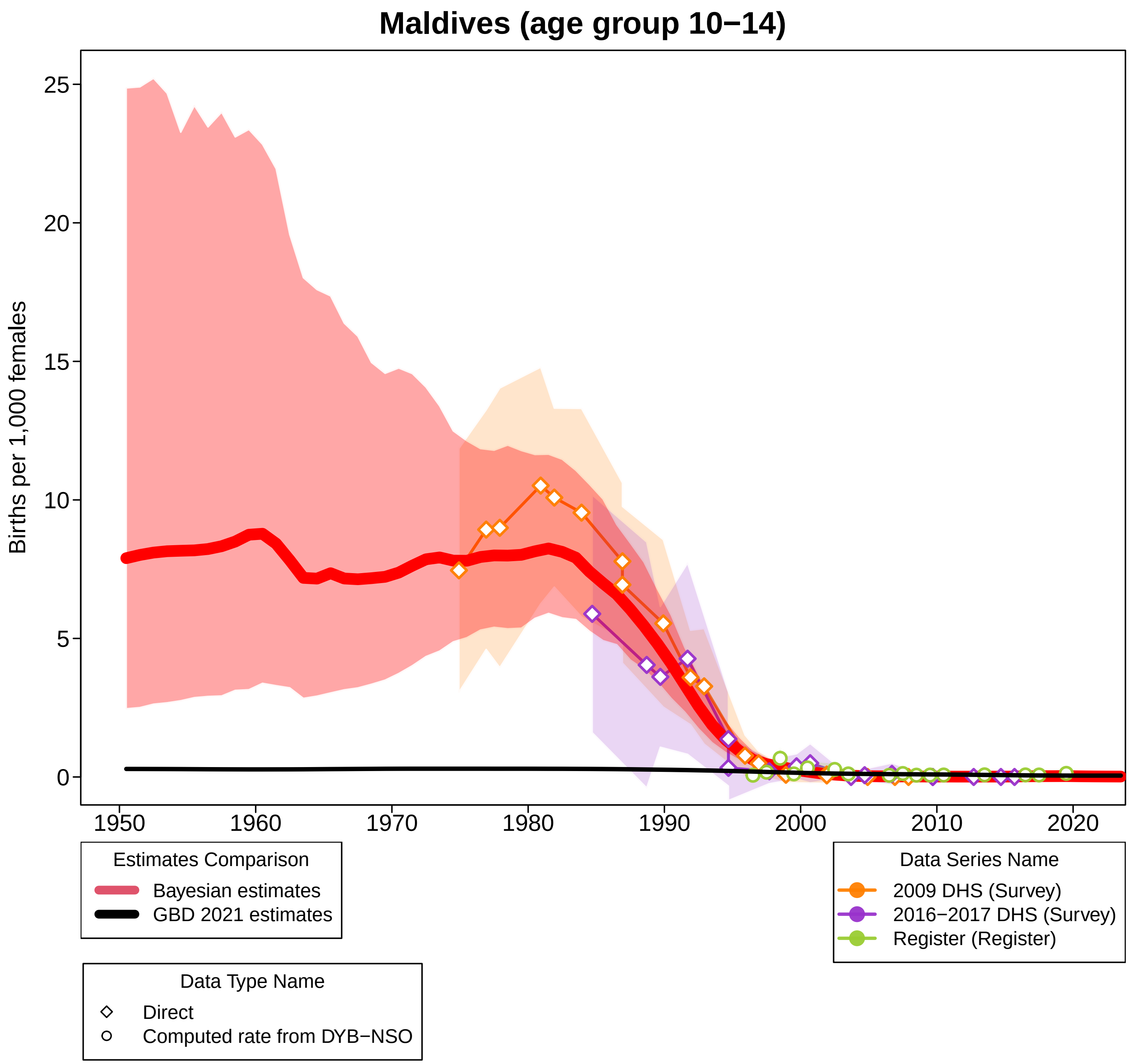
Maldives (age group 10−14)
Births per 1,000 females
25
20
15
10
5
0
1950
1960
1970
1980
1990
2000
2010
2020
Estimates Comparison
Bayesian estimates
GBD 2021 estimates
Data Series Name
2009 DHS (Survey)
2016−2017 DHS (Survey)
Register (Register)
Data Type Name
Direct
Computed rate from DYB−NSO

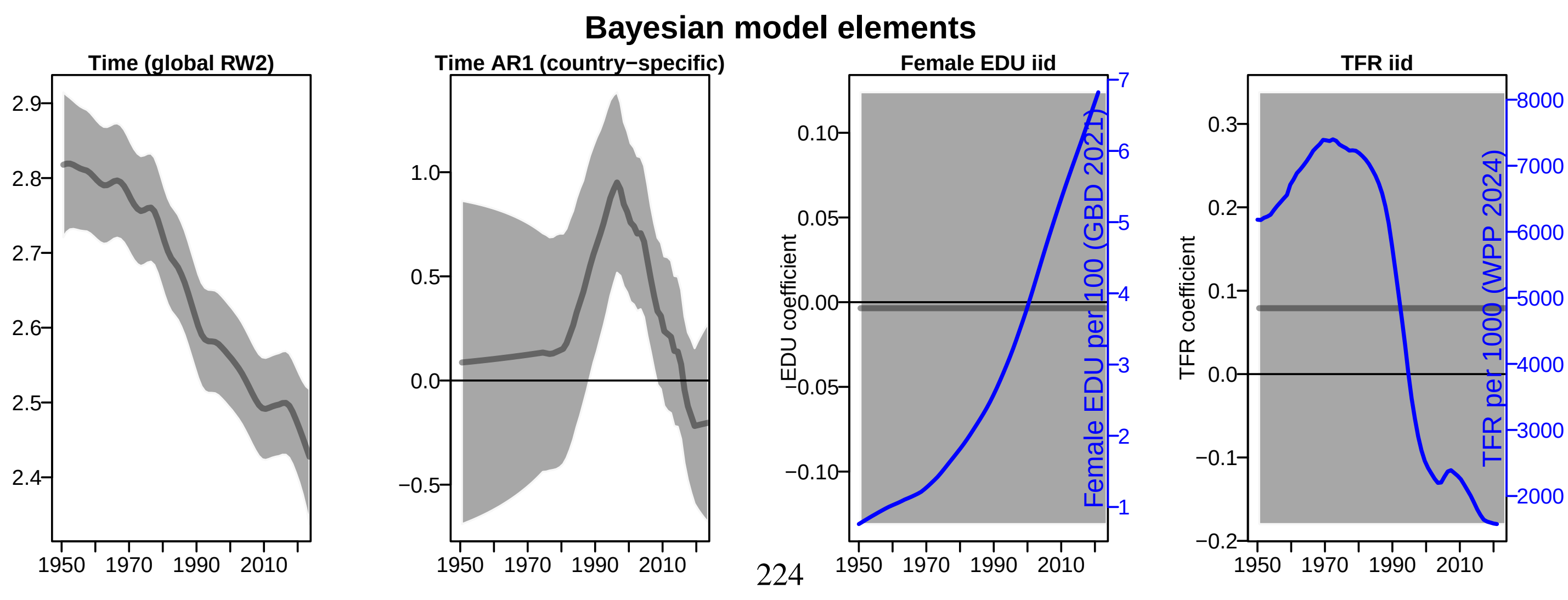
Bayesian model elements
Time (global RW2)
Time AR1 (country−specific)
Female EDU iid
TFR iid
EDU coefficient
Female EDU per 100 (GBD 2021)
TFR coefficient
TFR per 1000 (WPP 2024)
1950
1970
1990
2010

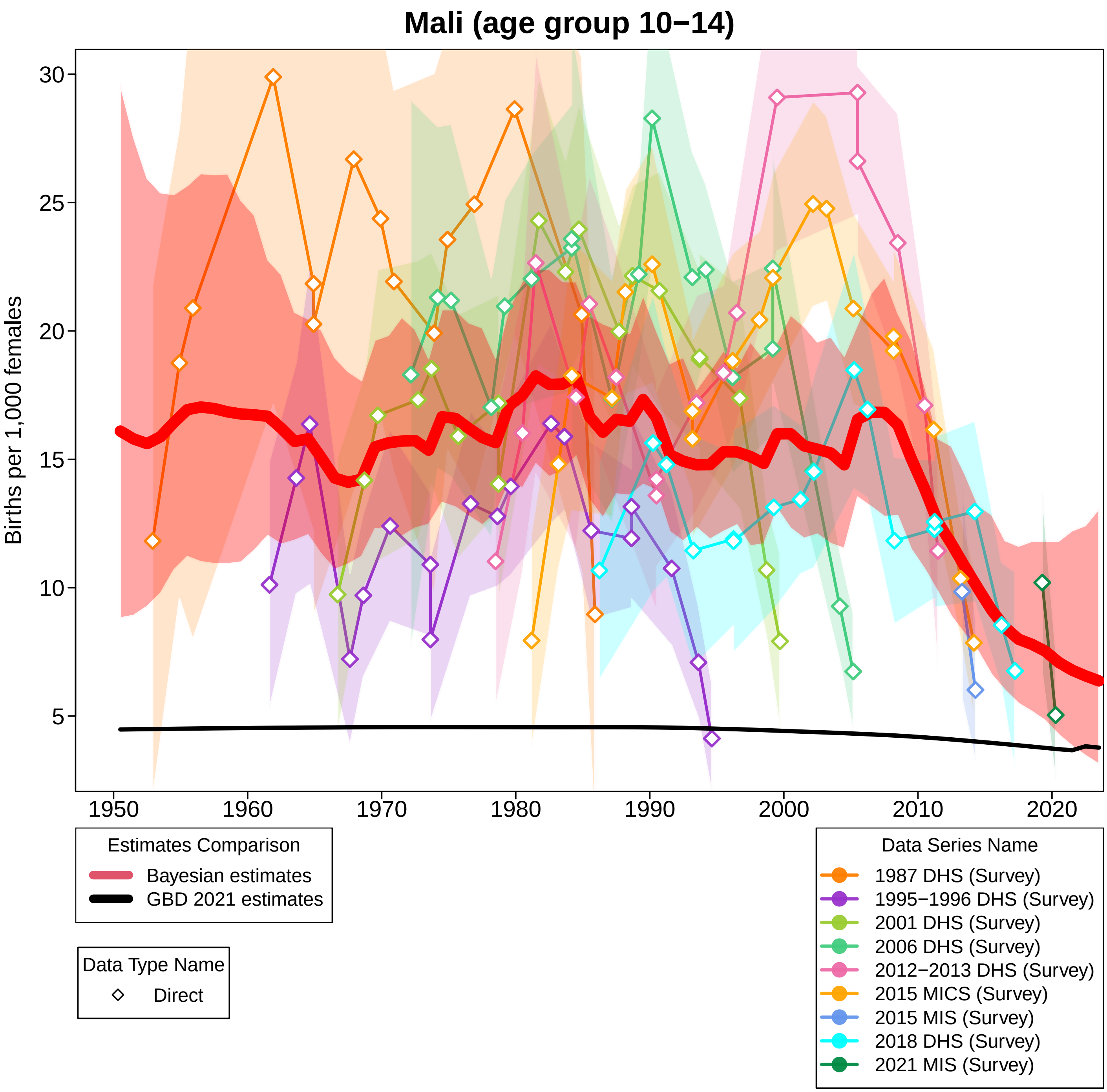
Mali (age group 10−14)
Births per 1,000 females
30
25
20
15
10
5
1950
1960
1970
1980
1990
2000
2010
2020
Estimates Comparison
Bayesian estimates
GBD 2021 estimates
Data Type Name
Direct
Data Series Name
1987 DHS (Survey)
1995−1996 DHS (Survey)
2001 DHS (Survey)
2006 DHS (Survey)
2012−2013 DHS (Survey)
2015 MICS (Survey)
2015 MIS (Survey)
2018 DHS (Survey)
2021 MIS (Survey)

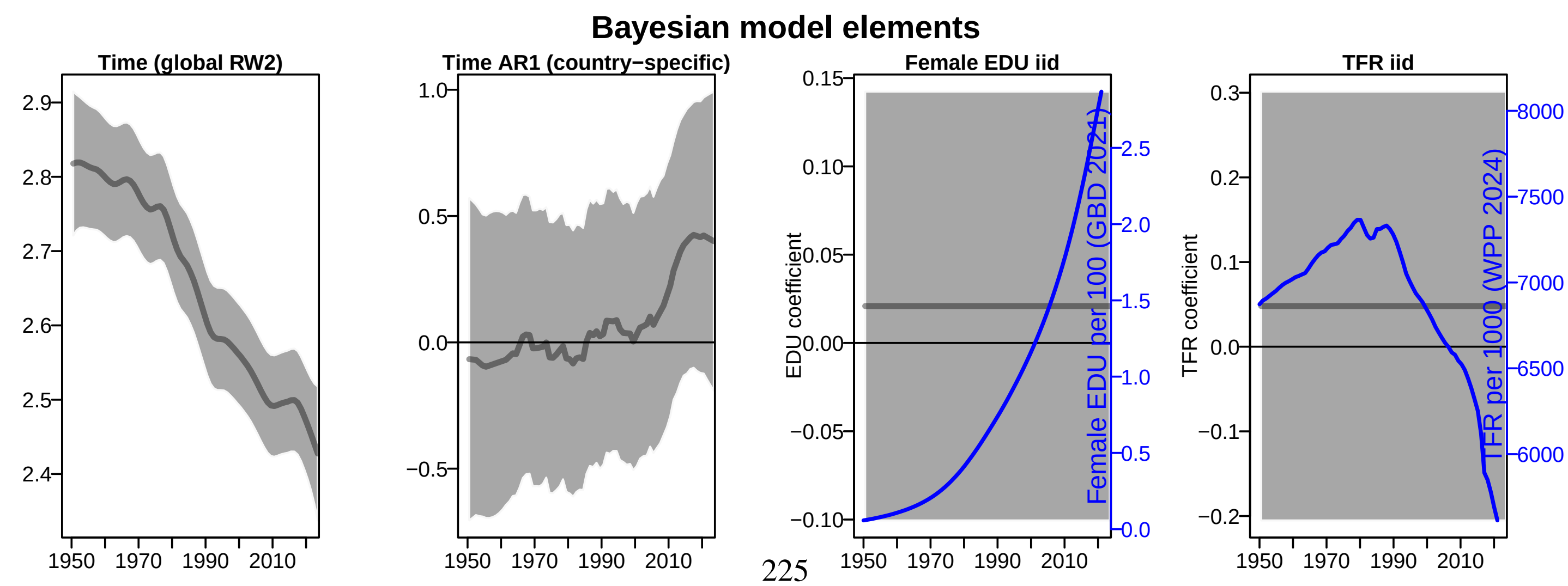
Bayesian model elements
Time (global RW2)
Time AR1 (country−specific)
Female EDU iid
TFR iid
EDU coefficient
Female EDU per 100 (GBD 2021)
TFR coefficient
TFR per 1000 (WPP 2024)
1950
1970
1990
2010

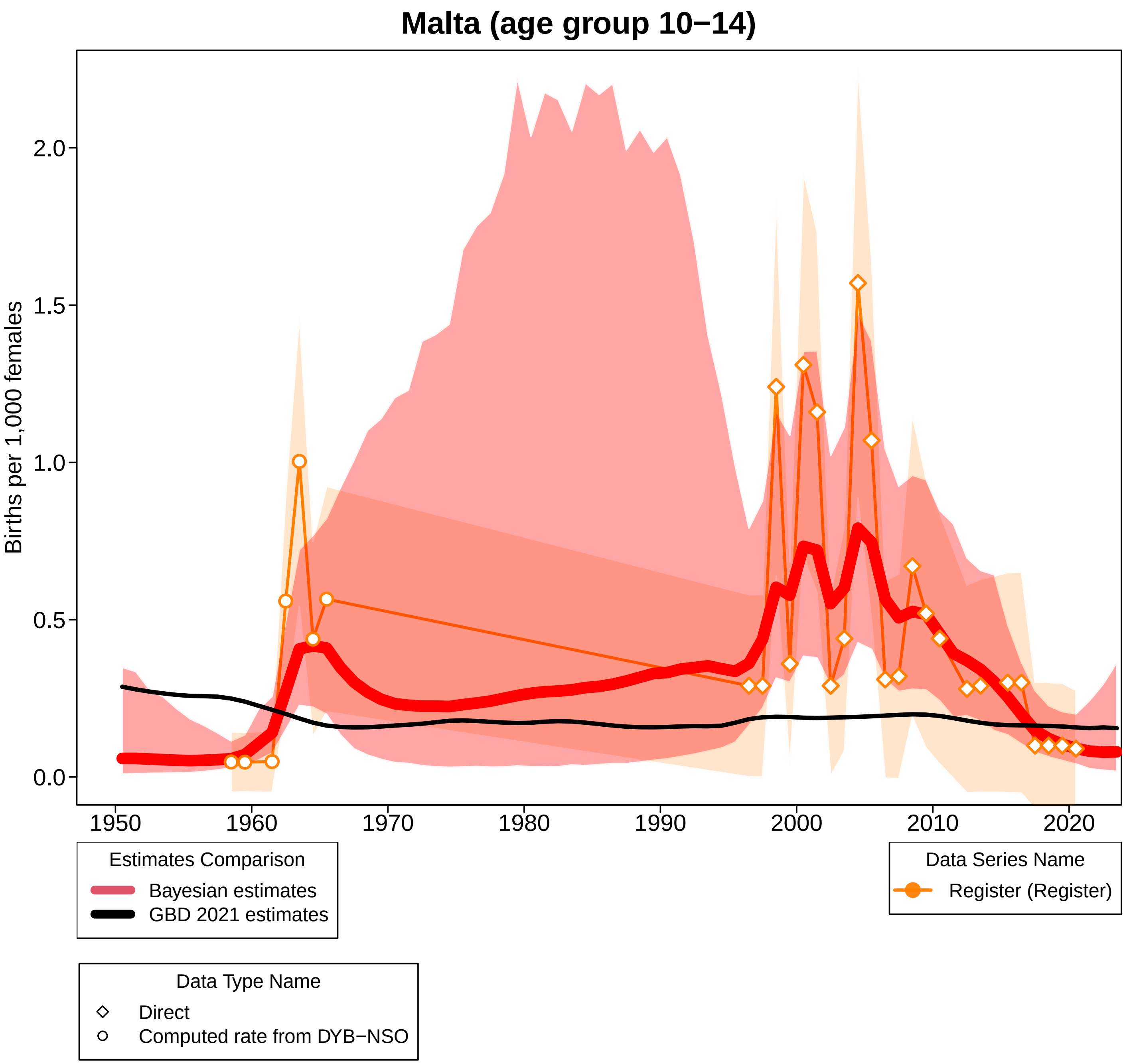
Malta (age group 10−14)
Births per 1,000 females
2.0
1.5
1.0
0.5
0.0
1950
1960
1970
1980
1990
2000
2010
2020
Estimates Comparison
Bayesian estimates
GBD 2021 estimates
Data Series Name
Register (Register)
Data Type Name
Direct
Computed rate from DYB−NSO

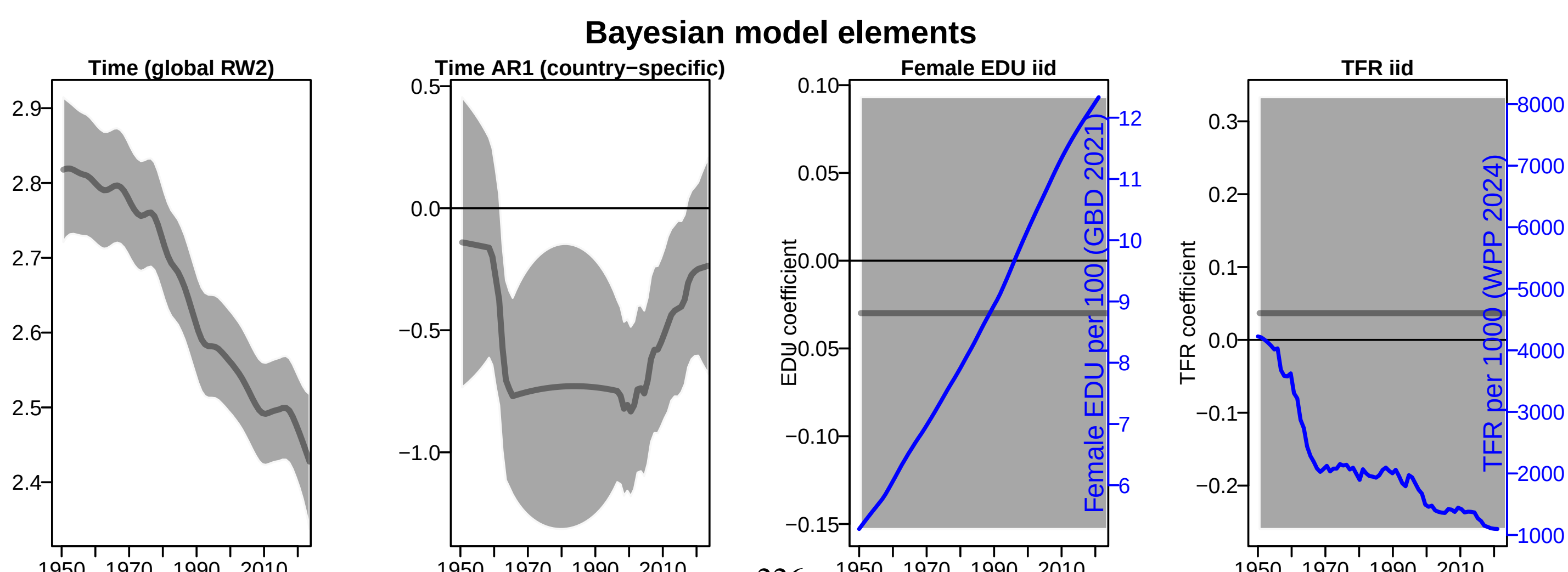
Bayesian model elements
Time (global RW2)
Time AR1 (country−specific)
Female EDU iid
TFR iid
EDU coefficient
Female EDU per 100 (GBD 2021)
TFR coefficient
TFR per 1000 (WPP 2024)
1950
1970
1990
2010

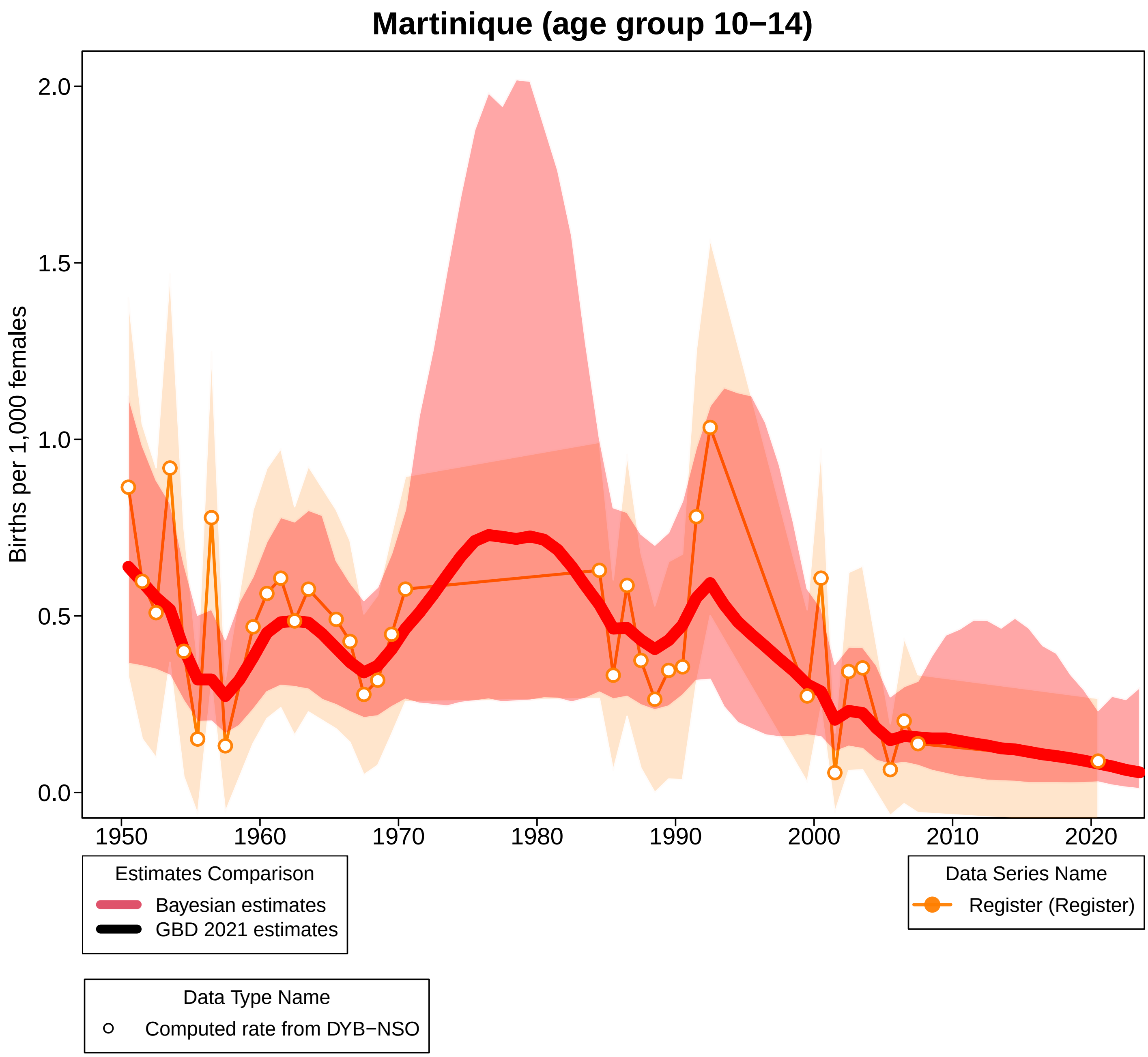
Martinique (age group 10−14)
Births per 1,000 females
2.0
1.5
1.0
0.5
0.0
1950
1960
1970
1980
1990
2000
2010
2020
Estimates Comparison
Bayesian estimates
GBD 2021 estimates
Data Series Name
Register (Register)
Data Type Name
Computed rate from DYB−NSO

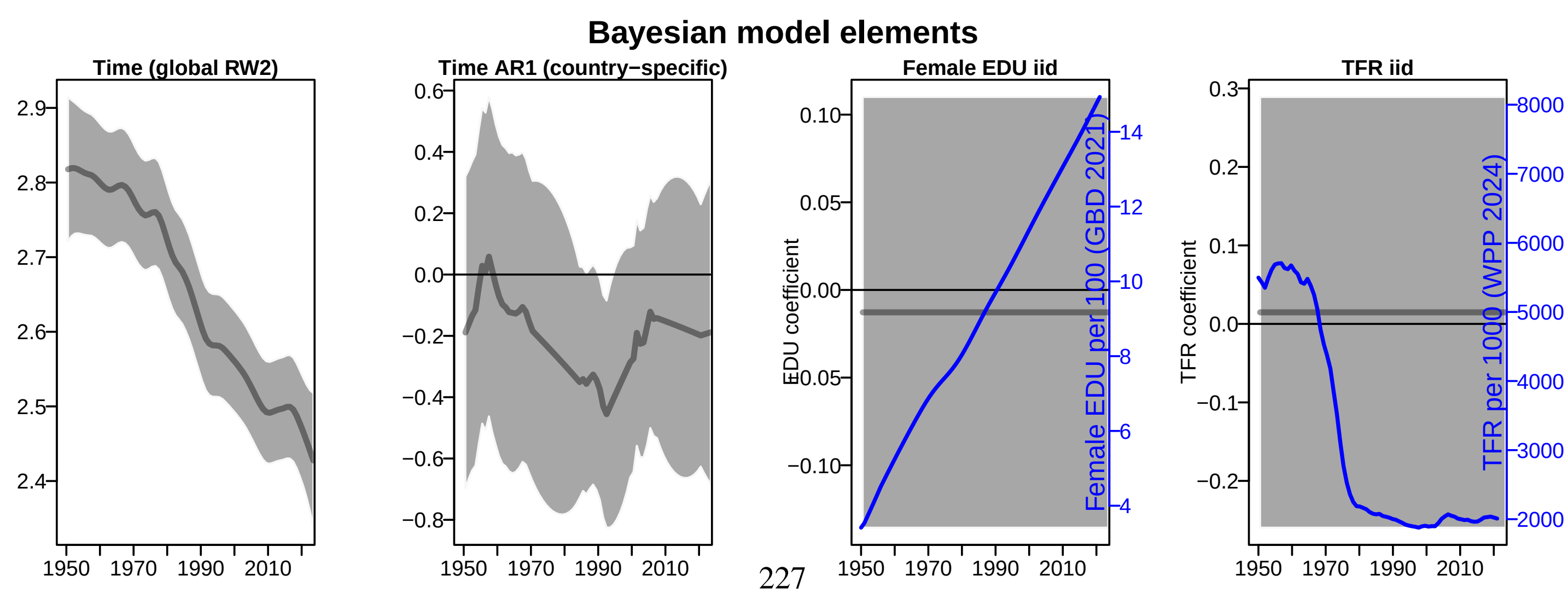
Bayesian model elements
Time (global RW2)
Time AR1 (country−specific)
Female EDU iid
TFR iid
EDU coefficient
Female EDU per 100 (GBD 2021)
TFR coefficient
TFR per 1000 (WPP 2024)
1950
1970
1990
2010

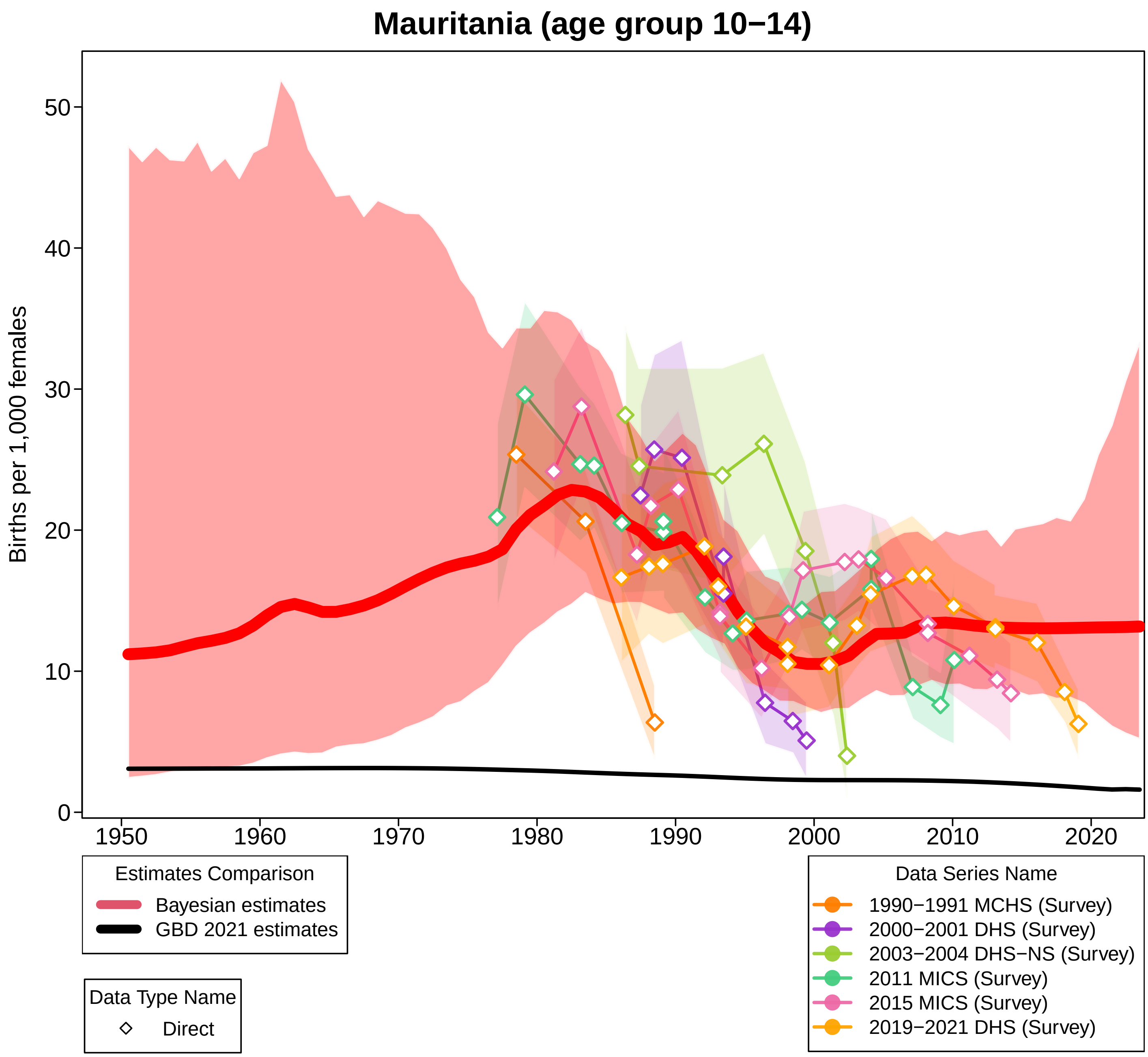

Mauritania (age group 10−14)
Births per 1,000 females
0
10
20
30
40
50
1950
1960
1970
1980
1990
2000
2010
2020
Estimates Comparison
Bayesian estimates
GBD 2021 estimates
Data Type Name
Direct
Data Series Name
1990−1991 MCHS (Survey)
2000−2001 DHS (Survey)
2003−2004 DHS−NS (Survey)
2011 MICS (Survey)
2015 MICS (Survey)
2019−2021 DHS (Survey)

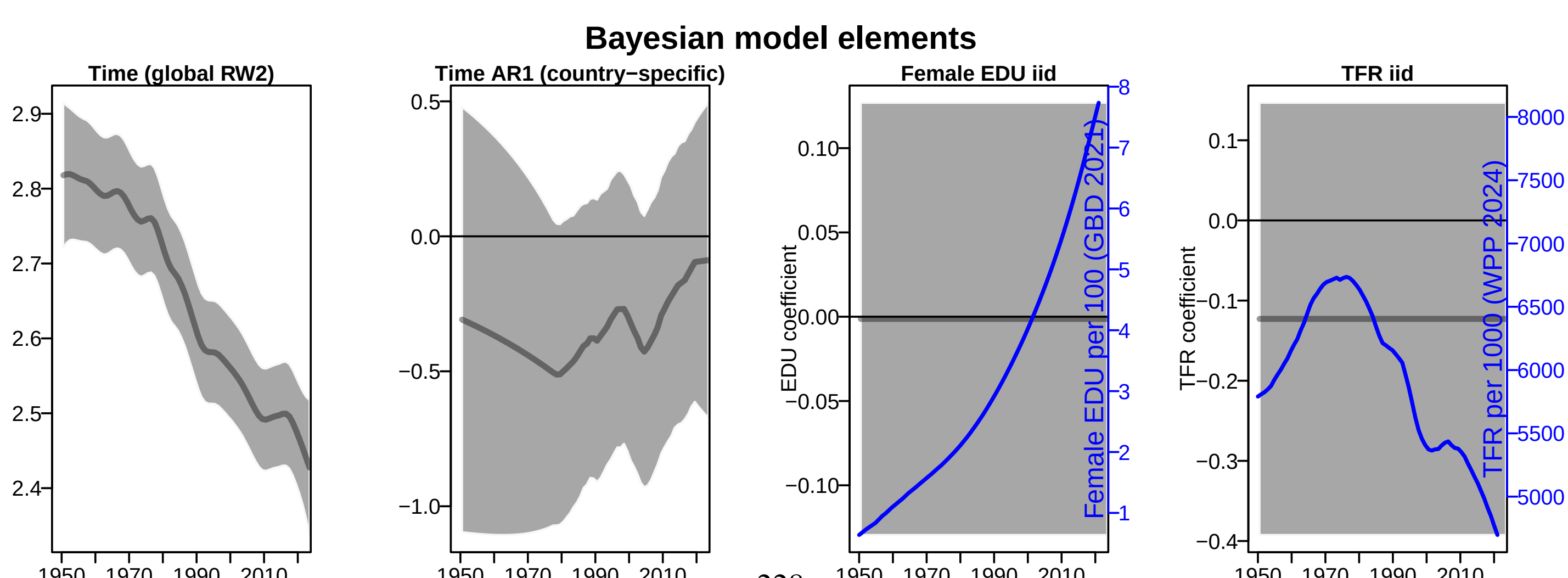

Bayesian model elements
Time (global RW2)
Time AR1 (country−specific)
Female EDU iid
TFR iid
EDU coefficient
Female EDU per 100 (GBD 2021)
TFR coefficient
TFR per 1000 (WPP 2024)
1950
1970
1990
2010

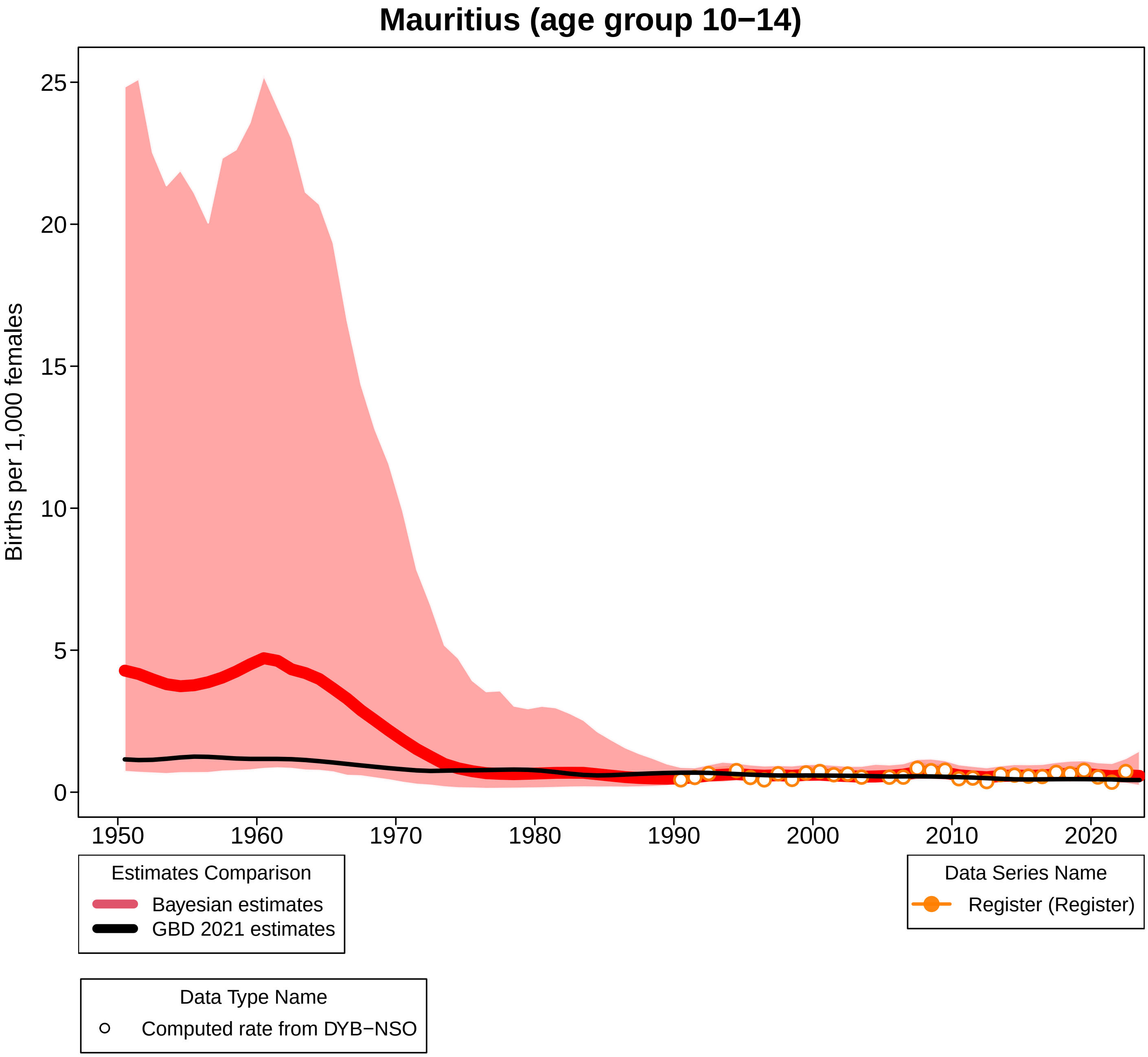
Mauritius (age group 10−14)
Births per 1,000 females
0
5
10
15
20
25
1950
1960
1970
1980
1990
2000
2010
2020
Estimates Comparison
Bayesian estimates
GBD 2021 estimates
Data Series Name
Register (Register)
Data Type Name
Computed rate from DYB−NSO

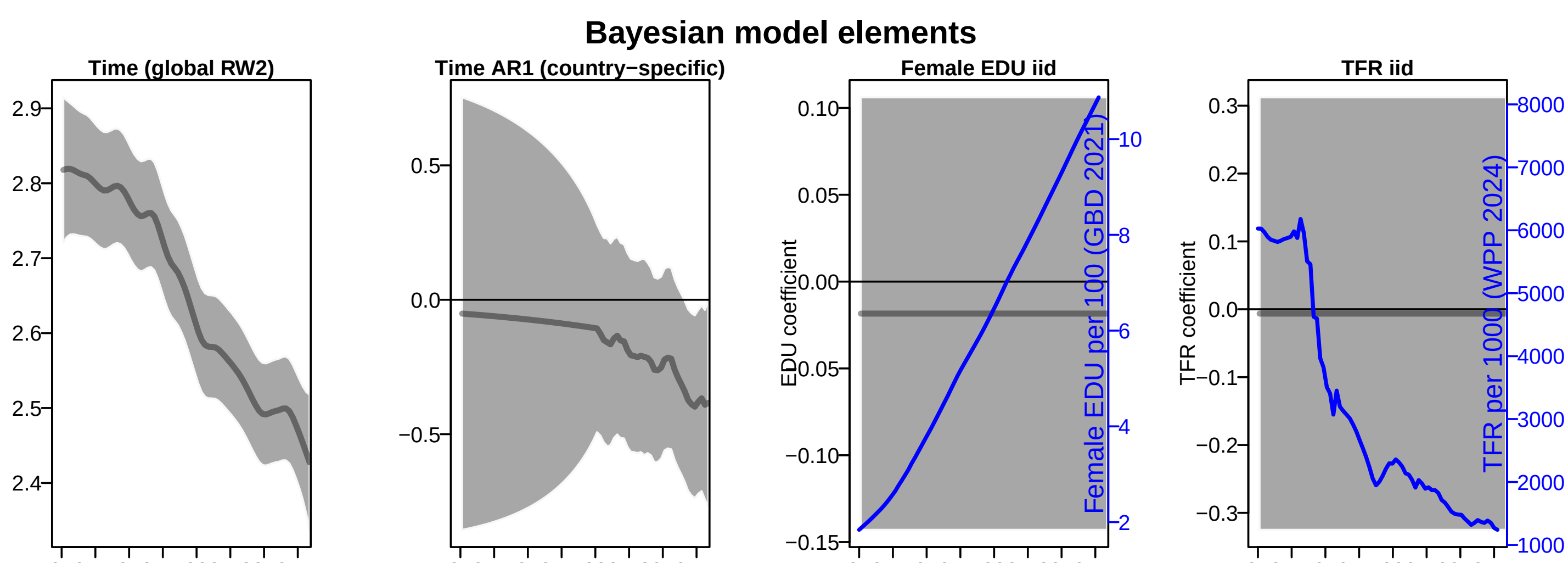
Bayesian model elements
Time (global RW2)
Time AR1 (country−specific)
Female EDU iid
TFR iid
EDU coefficient
Female EDU per 100 (GBD 2021)
TFR coefficient
TFR per 1000 (WPP 2024)
1950
1970
1990
2010

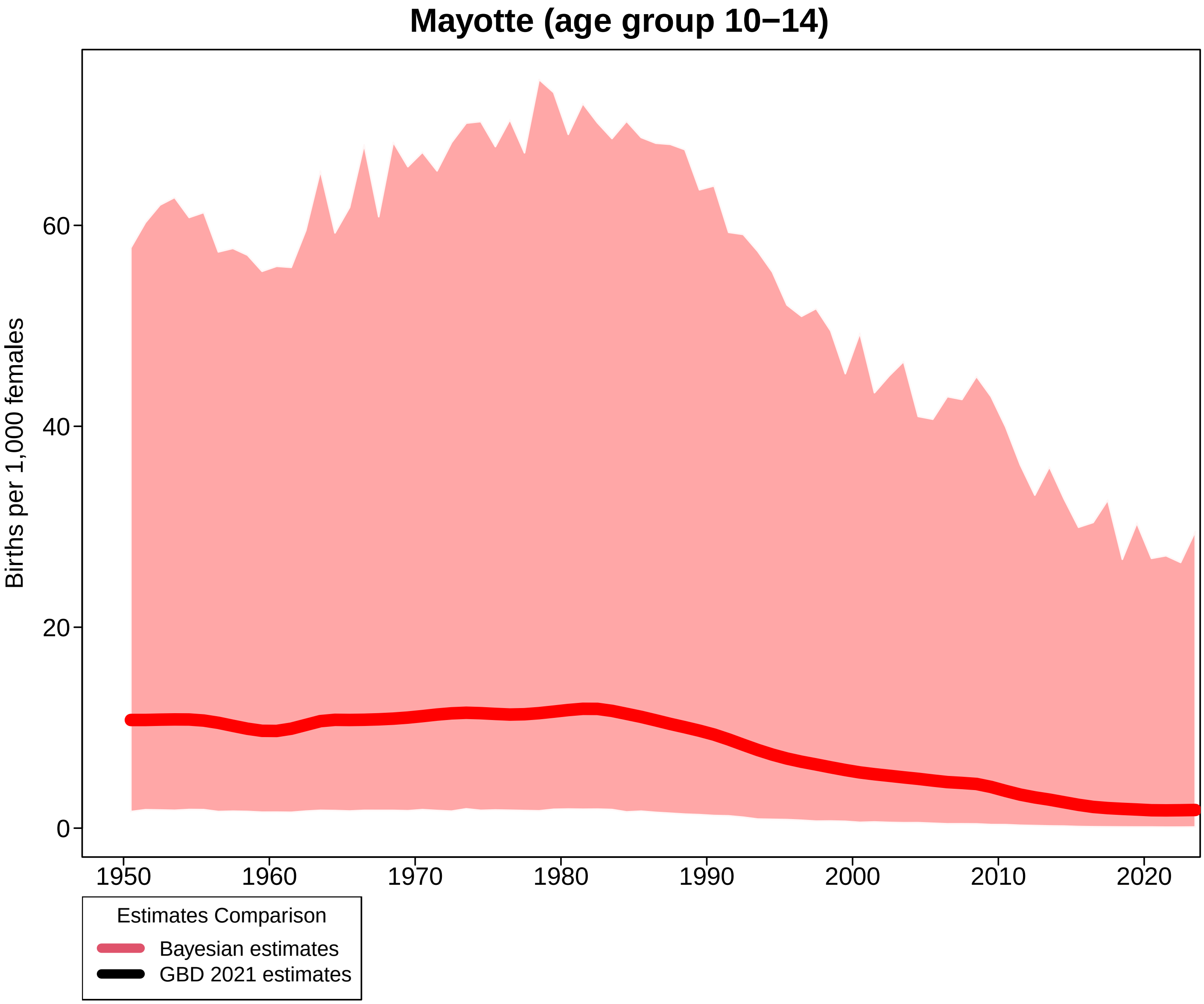

Mayotte (age group 10−14)
Births per 1,000 females
0
20
40
60
1950
1960
1970
1980
1990
2000
2010
2020
Estimates Comparison
Bayesian estimates
GBD 2021 estimates

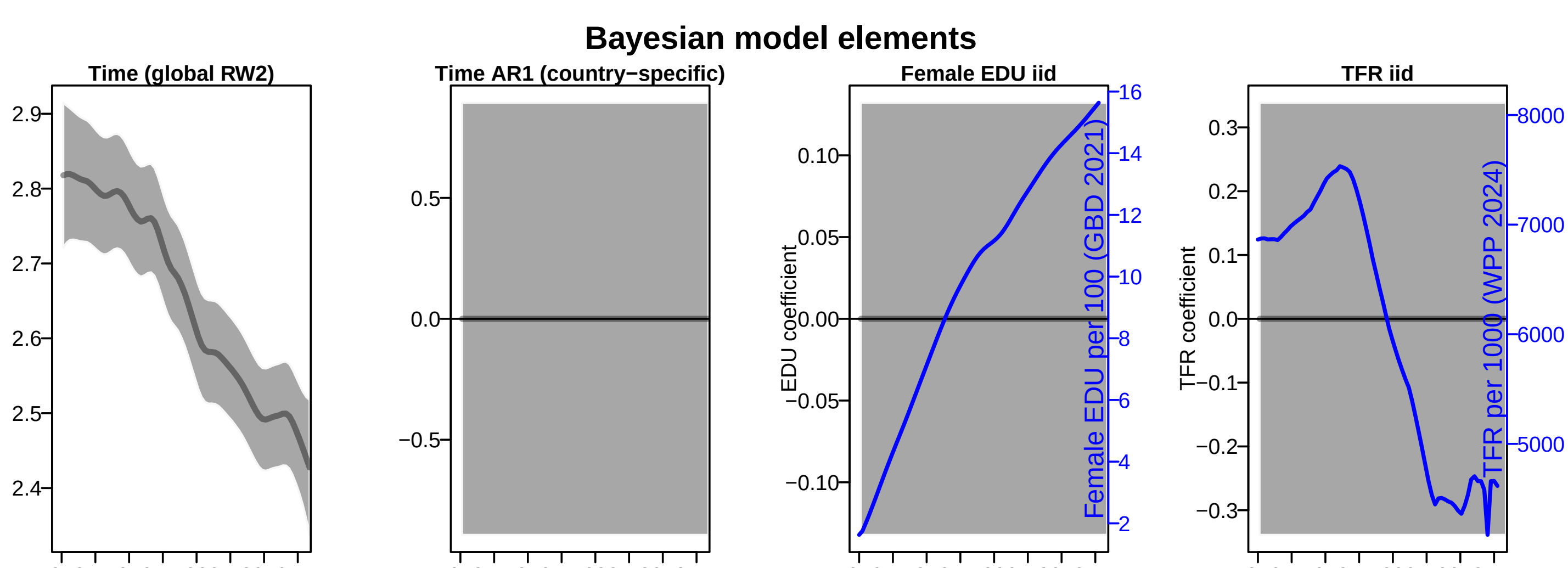

Bayesian model elements
Time (global RW2)
Time AR1 (country−specific)
Female EDU iid
TFR iid
EDU coefficient
Female EDU per 100 (GBD 2021)
TFR coefficient
TFR per 1000 (WPP 2024)
1950
1970
1990
2010

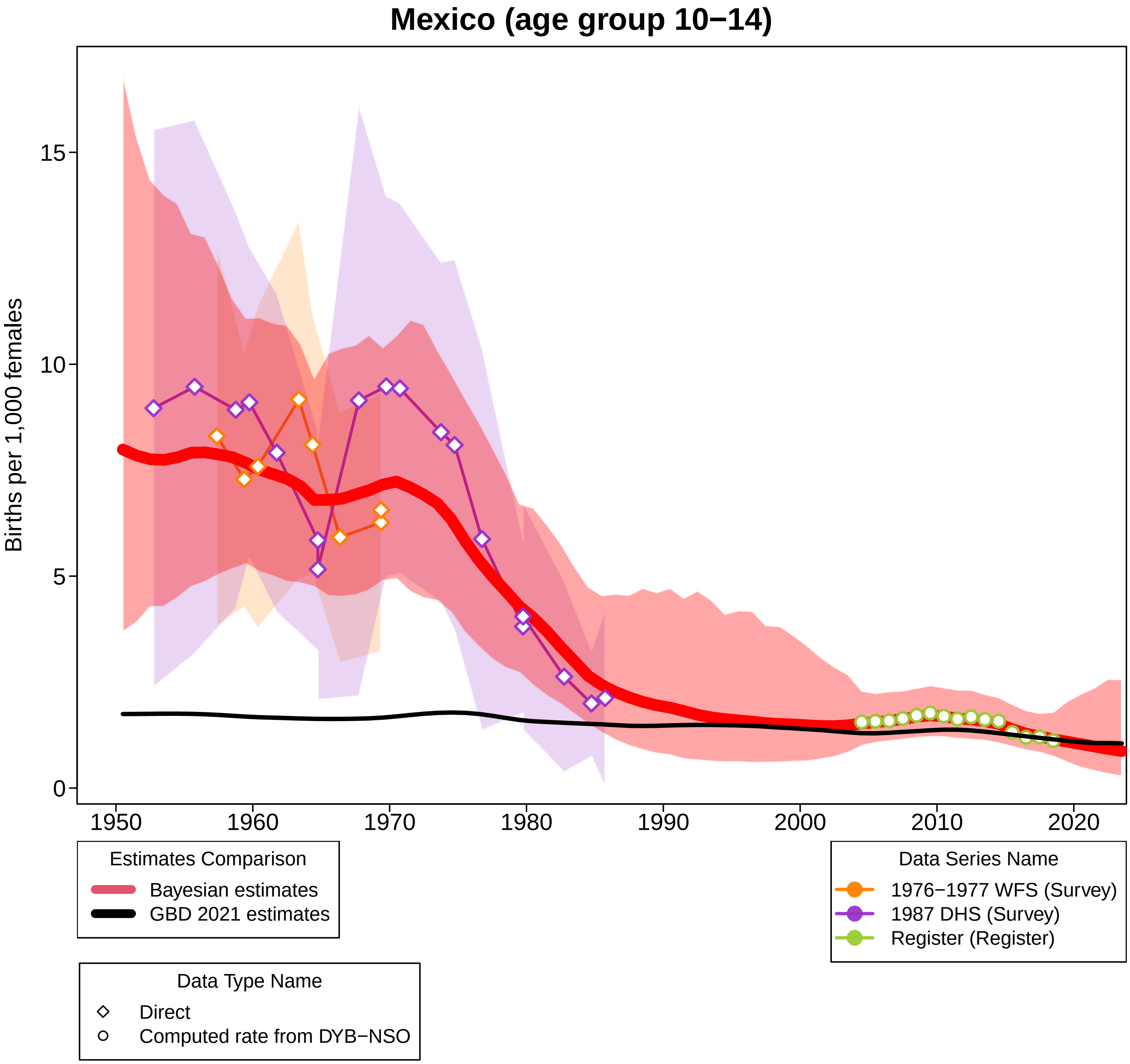
Mexico (age group 10−14)
Births per 1,000 females
15
10
5
0
1950
1960
1970
1980
1990
2000
2010
2020
Estimates Comparison
Bayesian estimates
GBD 2021 estimates
Data Series Name
1976−1977 WFS (Survey)
1987 DHS (Survey)
Register (Register)
Data Type Name
Direct
Computed rate from DYB−NSO

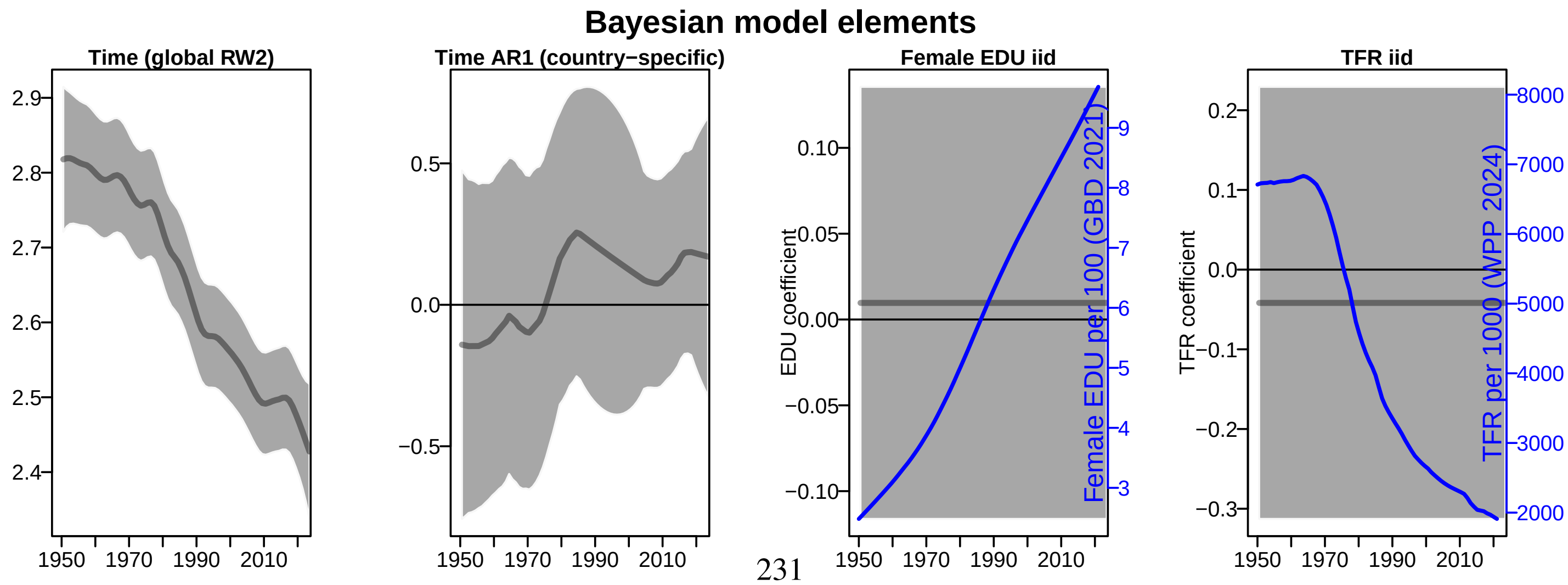
Bayesian model elements
Time (global RW2)
Time AR1 (country−specific)
Female EDU iid
TFR iid
EDU coefficient
Female EDU per 100 (GBD 2021)
TFR coefficient
TFR per 1000 (WPP 2024)
1950
1970
1990
2010

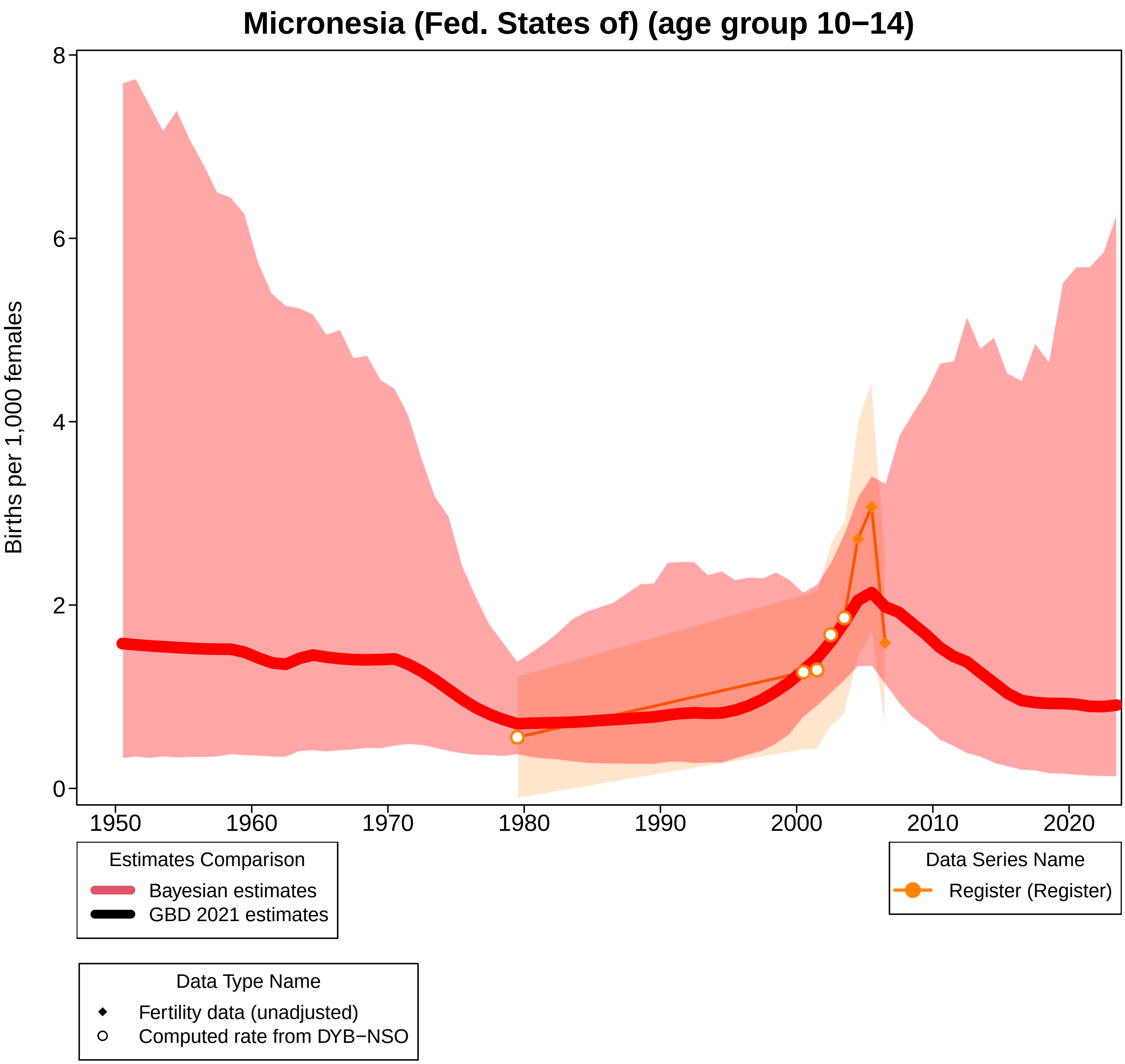

Micronesia (Fed. States of) (age group 10−14)
Births per 1,000 females
0
2
4
6
8
1950
1960
1970
1980
1990
2000
2010
2020
Estimates Comparison
Bayesian estimates
GBD 2021 estimates
Data Series Name
Register (Register)
Data Type Name
Fertility data (unadjusted)
Computed rate from DYB−NSO

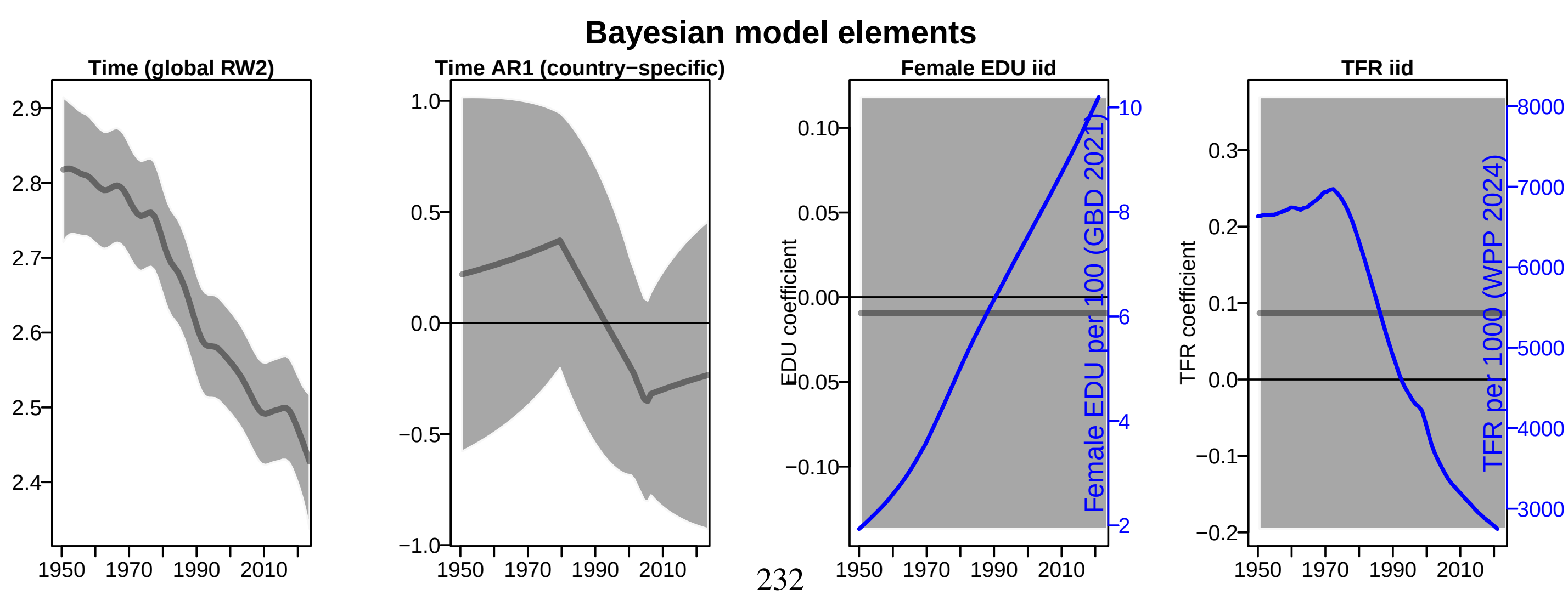

Bayesian model elements
Time (global RW2)
Time AR1 (country−specific)
Female EDU iid
TFR iid
EDU coefficient
Female EDU per 100 (GBD 2021)
TFR coefficient
TFR per 1000 (WPP 2024)
1950
1970
1990
2010

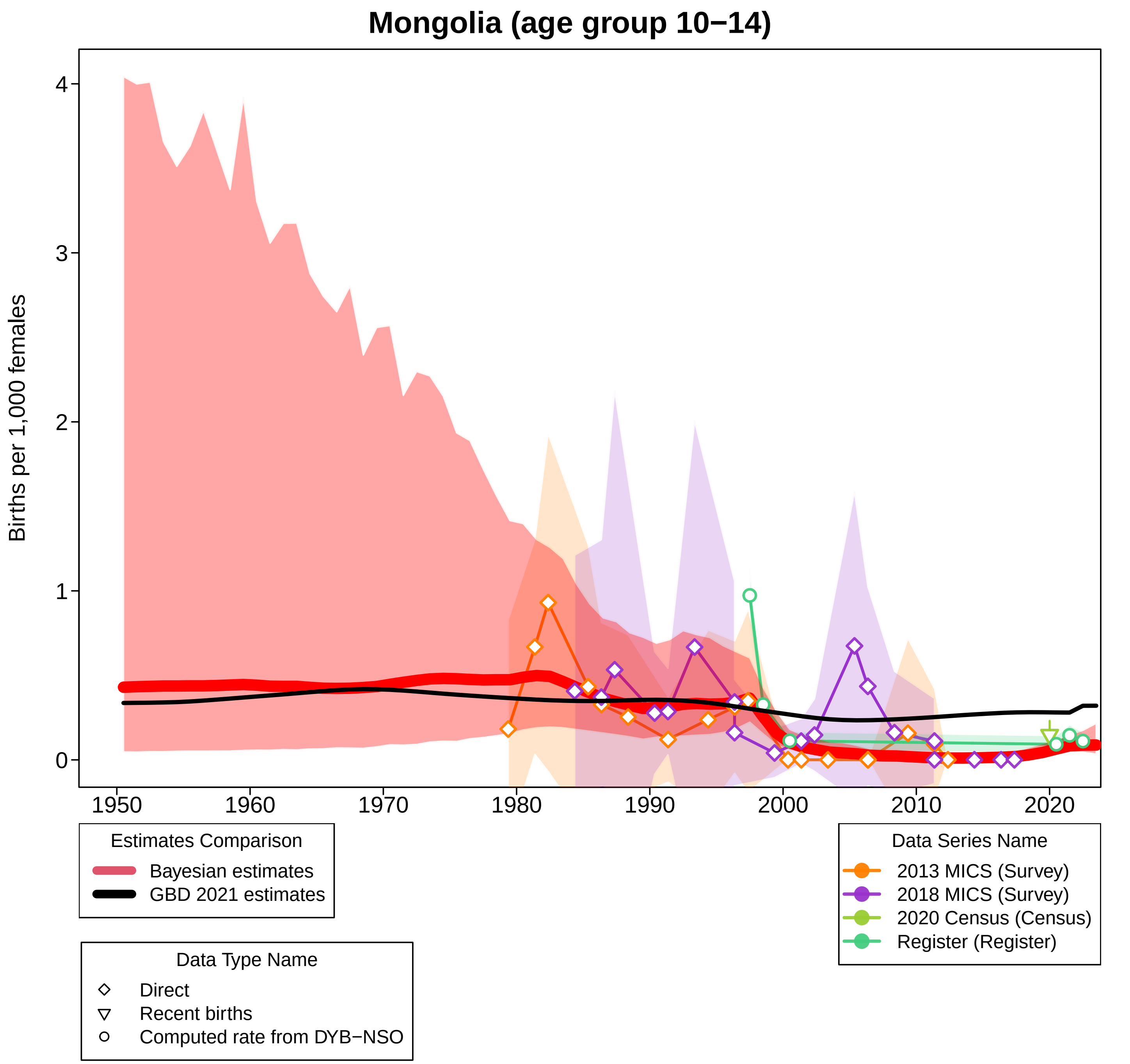
Mongolia (age group 10−14)
Births per 1,000 females
0
1
2
3
4
1950
1960
1970
1980
1990
2000
2010
2020
Estimates Comparison
Bayesian estimates
GBD 2021 estimates
Data Series Name
2013 MICS (Survey)
2018 MICS (Survey)
2020 Census (Census)
Register (Register)
Data Type Name
Direct
Recent births
Computed rate from DYB−NSO

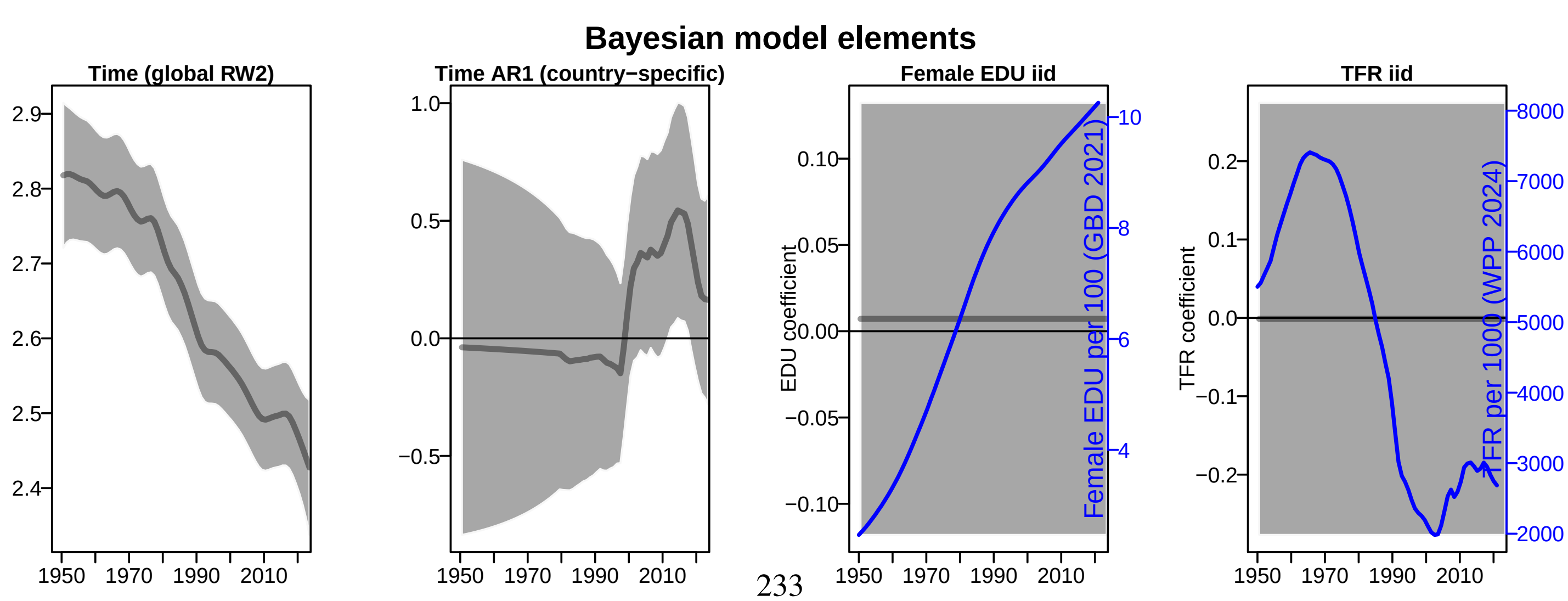
Bayesian model elements
Time (global RW2)
Time AR1 (country−specific)
Female EDU iid
TFR iid
EDU coefficient
Female EDU per 100 (GBD 2021)
TFR coefficient
TFR per 1000 (WPP 2024)
1950
1970
1990
2010

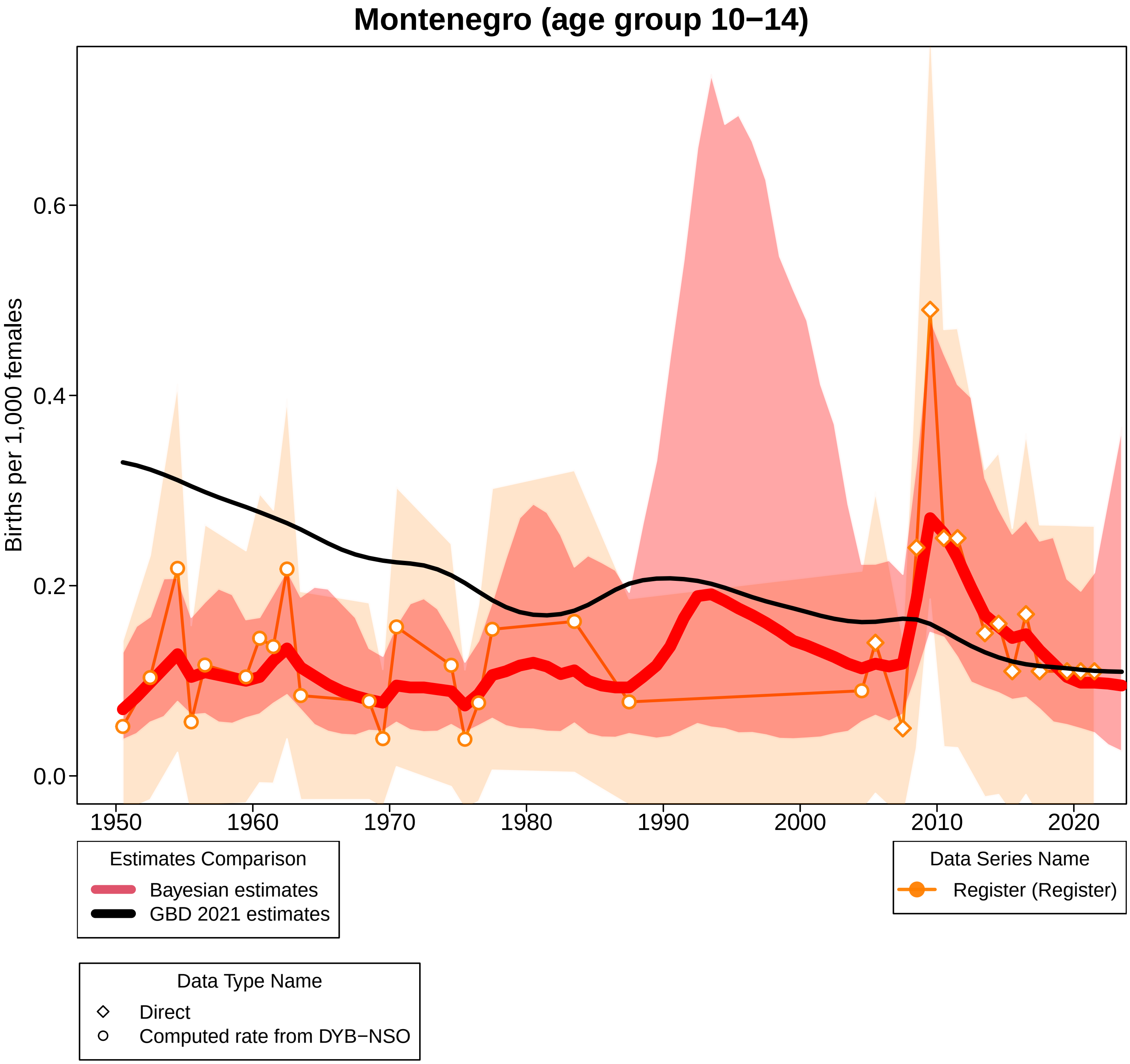
Montenegro (age group 10−14)
Births per 1,000 females
0.0
0.2
0.4
0.6
1950
1960
1970
1980
1990
2000
2010
2020
Estimates Comparison
Bayesian estimates
GBD 2021 estimates
Data Series Name
Register (Register)
Data Type Name
Direct
Computed rate from DYB−NSO

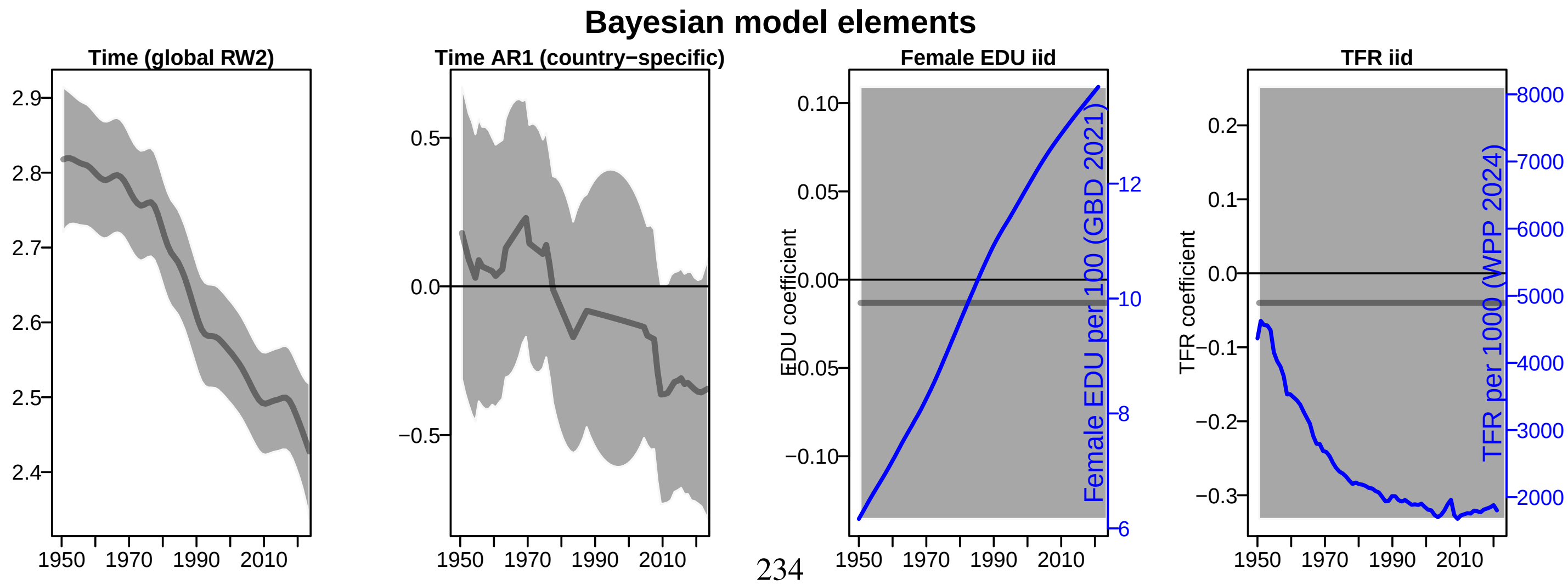
Bayesian model elements
Time (global RW2)
Time AR1 (country−specific)
Female EDU iid
TFR iid
EDU coefficient
Female EDU per 100 (GBD 2021)
TFR coefficient
TFR per 1000 (WPP 2024)
1950
1970
1990
2010

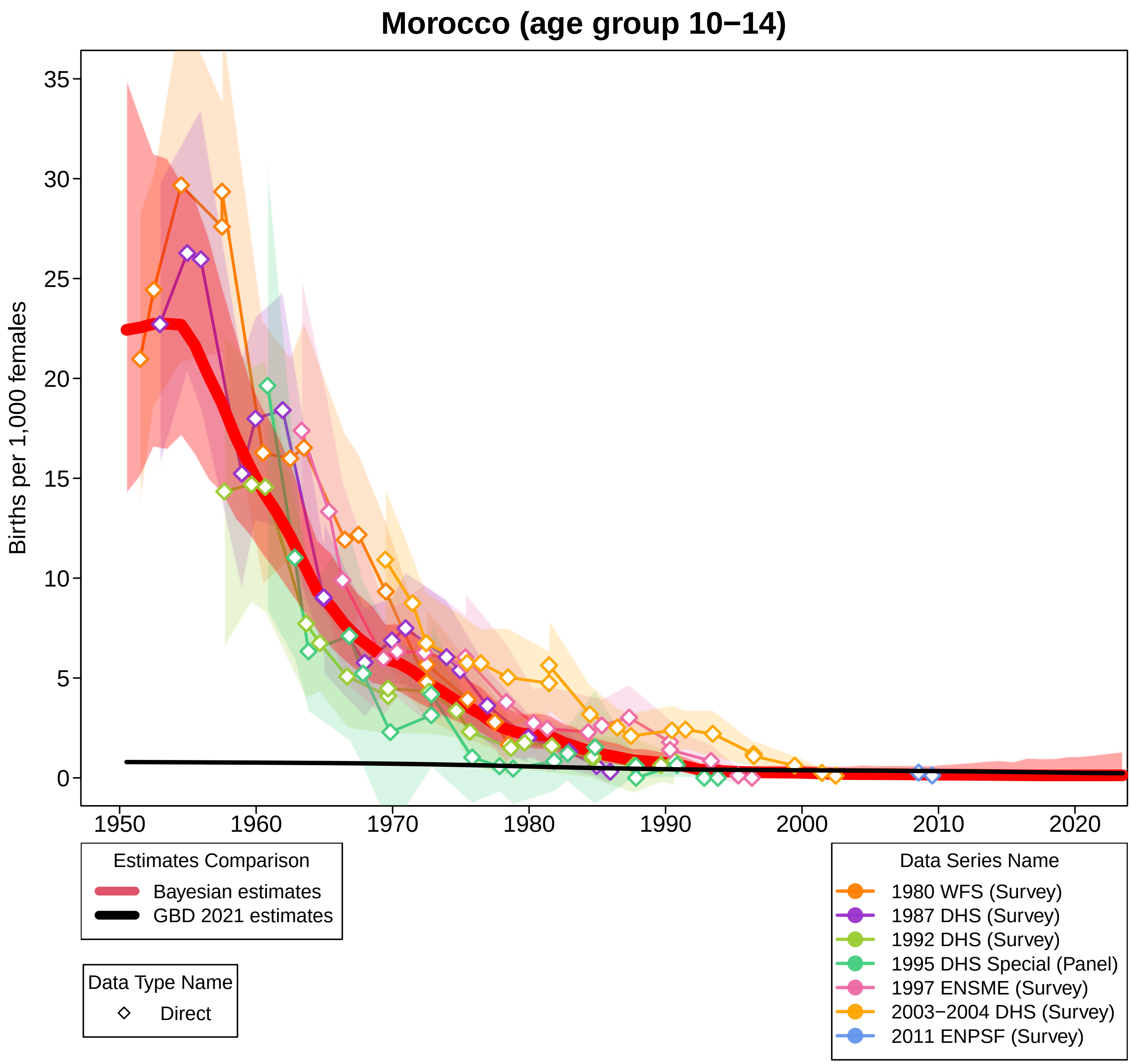
Morocco (age group 10−14)
Births per 1,000 females
Estimates Comparison
Bayesian estimates
GBD 2021 estimates
Data Type Name
Direct
Data Series Name
1980 WFS (Survey)
1987 DHS (Survey)
1992 DHS (Survey)
1995 DHS Special (Panel)
1997 ENSME (Survey)
2003−2004 DHS (Survey)
2011 ENPSF (Survey)

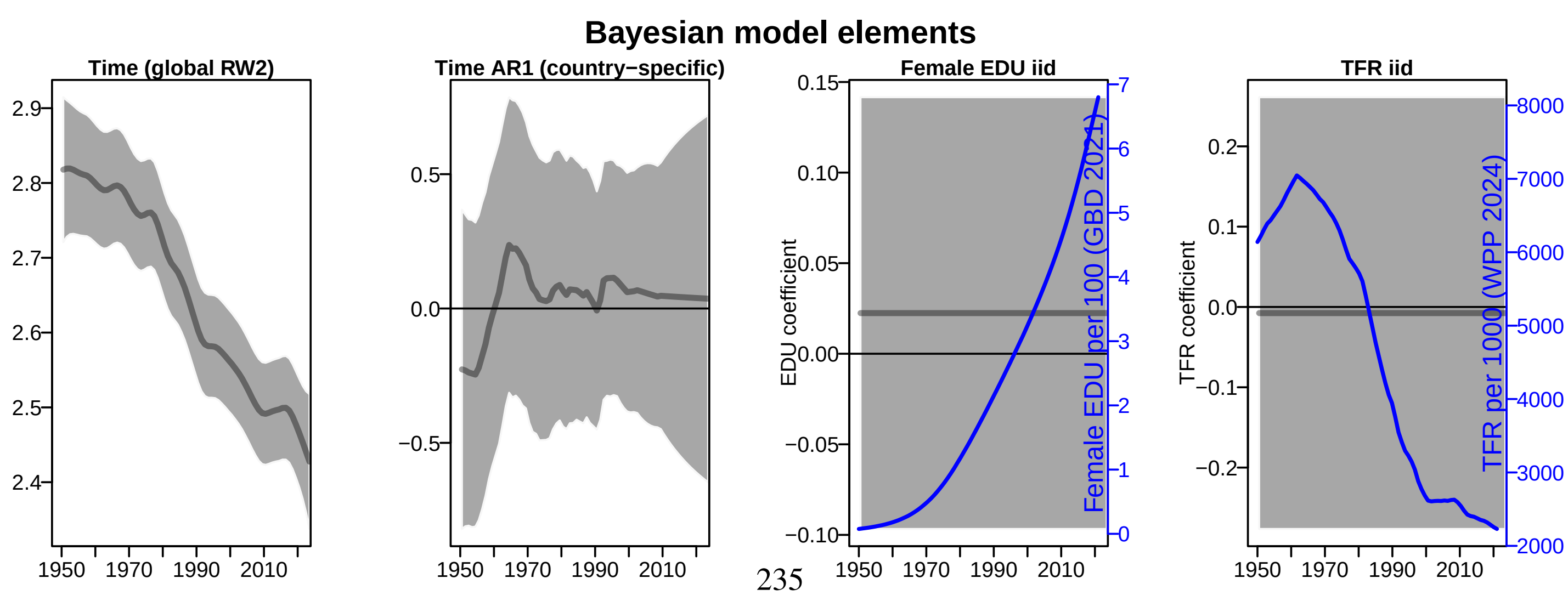
Bayesian model elements
Time (global RW2)
Time AR1 (country−specific)
Female EDU iid
TFR iid
EDU coefficient
Female EDU per 100 (GBD 2021)
TFR coefficient
TFR per 1000 (WPP 2024)

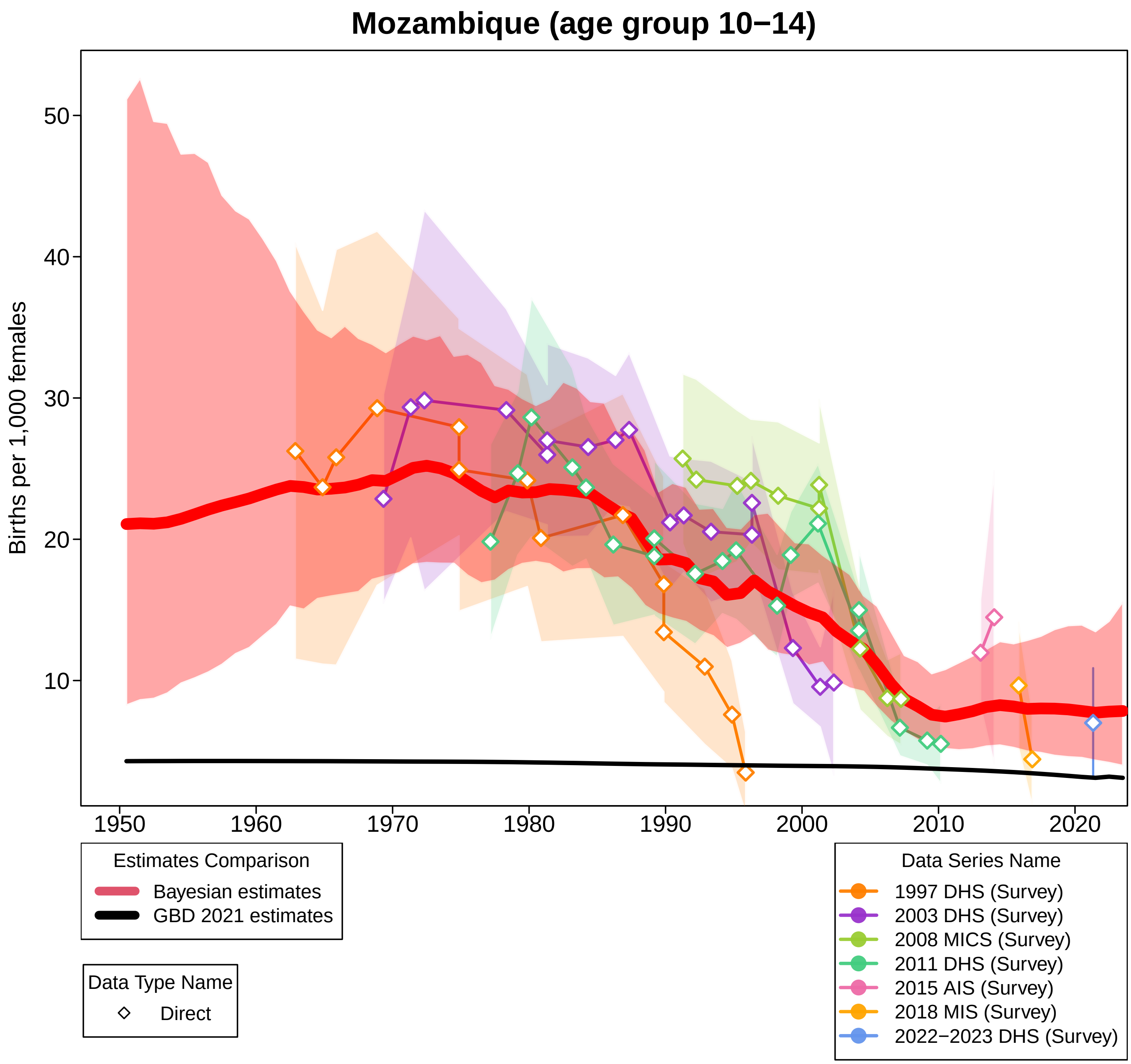
Mozambique (age group 10−14)
Births per 1,000 females
50
40
30
20
10
1950
1960
1970
1980
1990
2000
2010
2020
Estimates Comparison
Bayesian estimates
GBD 2021 estimates
Data Type Name
Direct
Data Series Name
1997 DHS (Survey)
2003 DHS (Survey)
2008 MICS (Survey)
2011 DHS (Survey)
2015 AIS (Survey)
2018 MIS (Survey)
2022−2023 DHS (Survey)

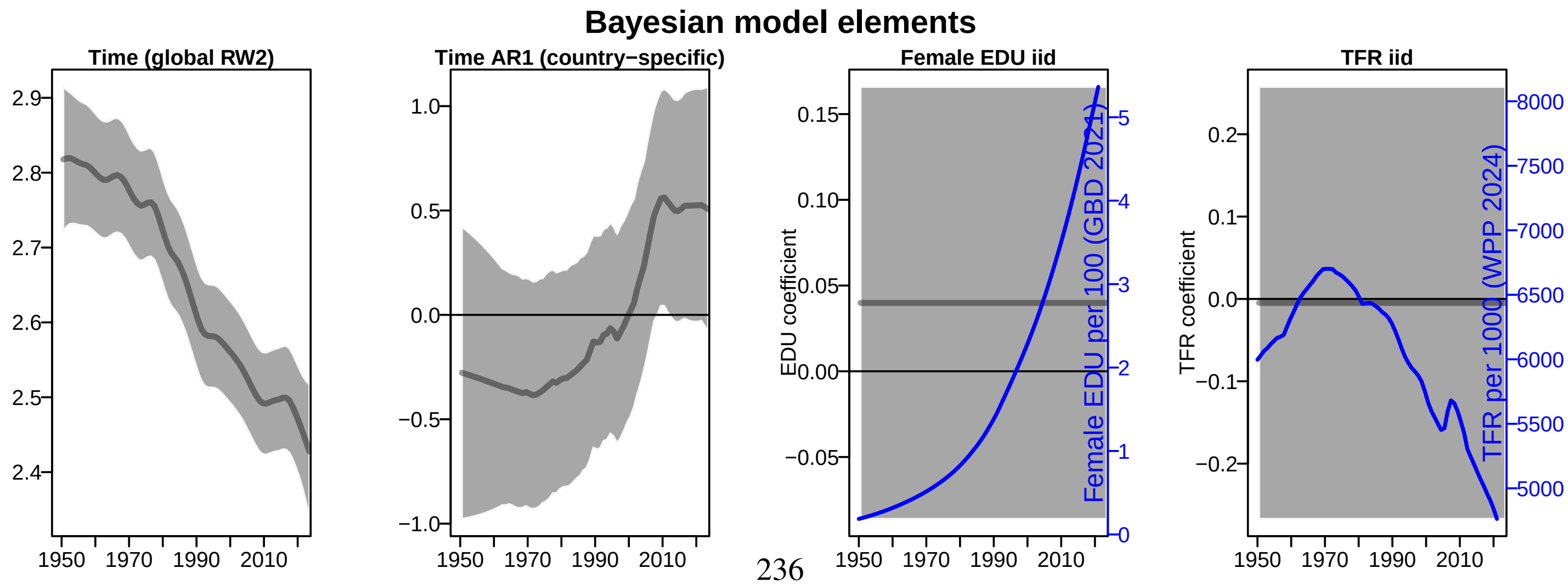
Bayesian model elements
Time (global RW2)
Time AR1 (country−specific)
Female EDU iid
TFR iid
EDU coefficient
Female EDU per 100 (GBD 2021)
TFR coefficient
TFR per 1000 (WPP 2024)

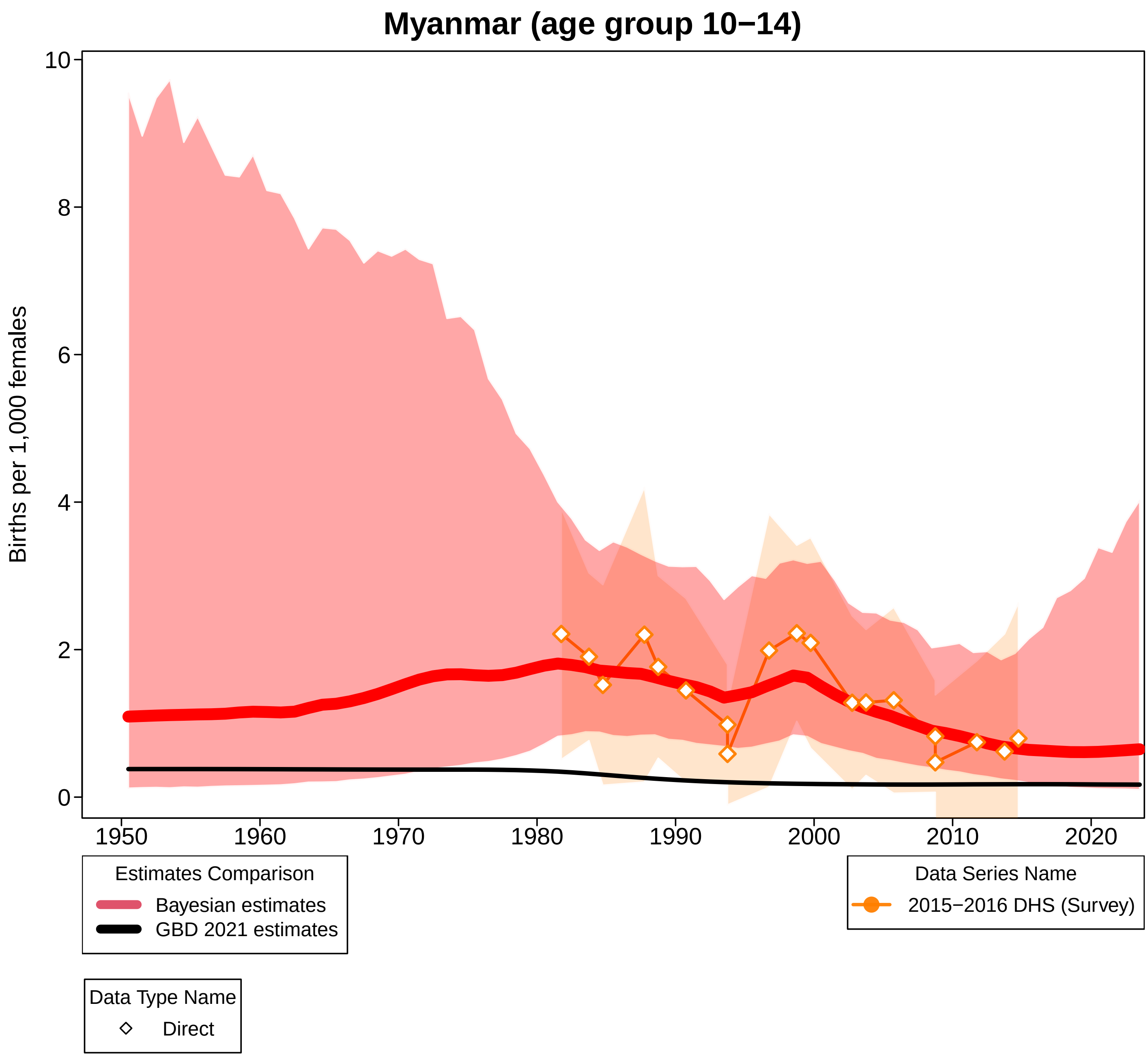

Myanmar (age group 10−14)
Births per 1,000 females
0
2
4
6
8
10
1950
1960
1970
1980
1990
2000
2010
2020
Estimates Comparison
Bayesian estimates
GBD 2021 estimates
Data Series Name
2015−2016 DHS (Survey)
Data Type Name
Direct

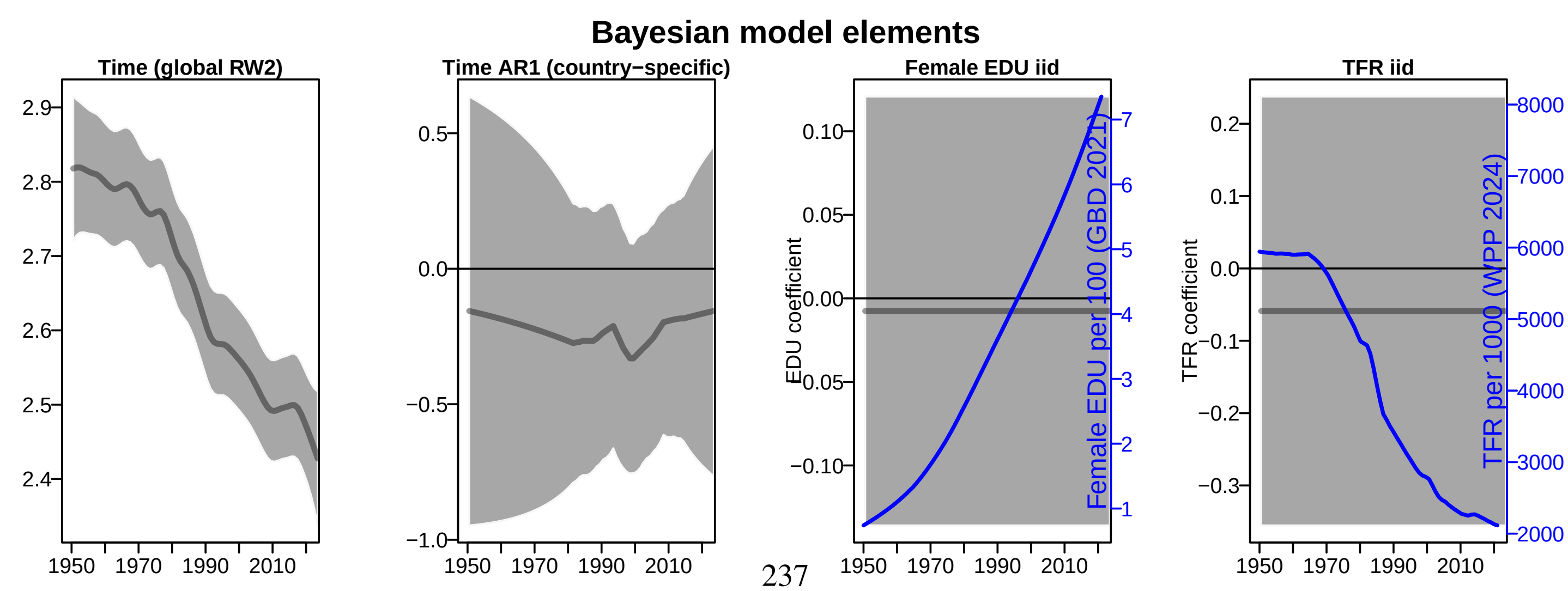

Bayesian model elements
Time (global RW2)
Time AR1 (country−specific)
Female EDU iid
TFR iid
EDU coefficient
Female EDU per 100 (GBD 2021)
TFR coefficient
TFR per 1000 (WPP 2024)
1950
1970
1990
2010

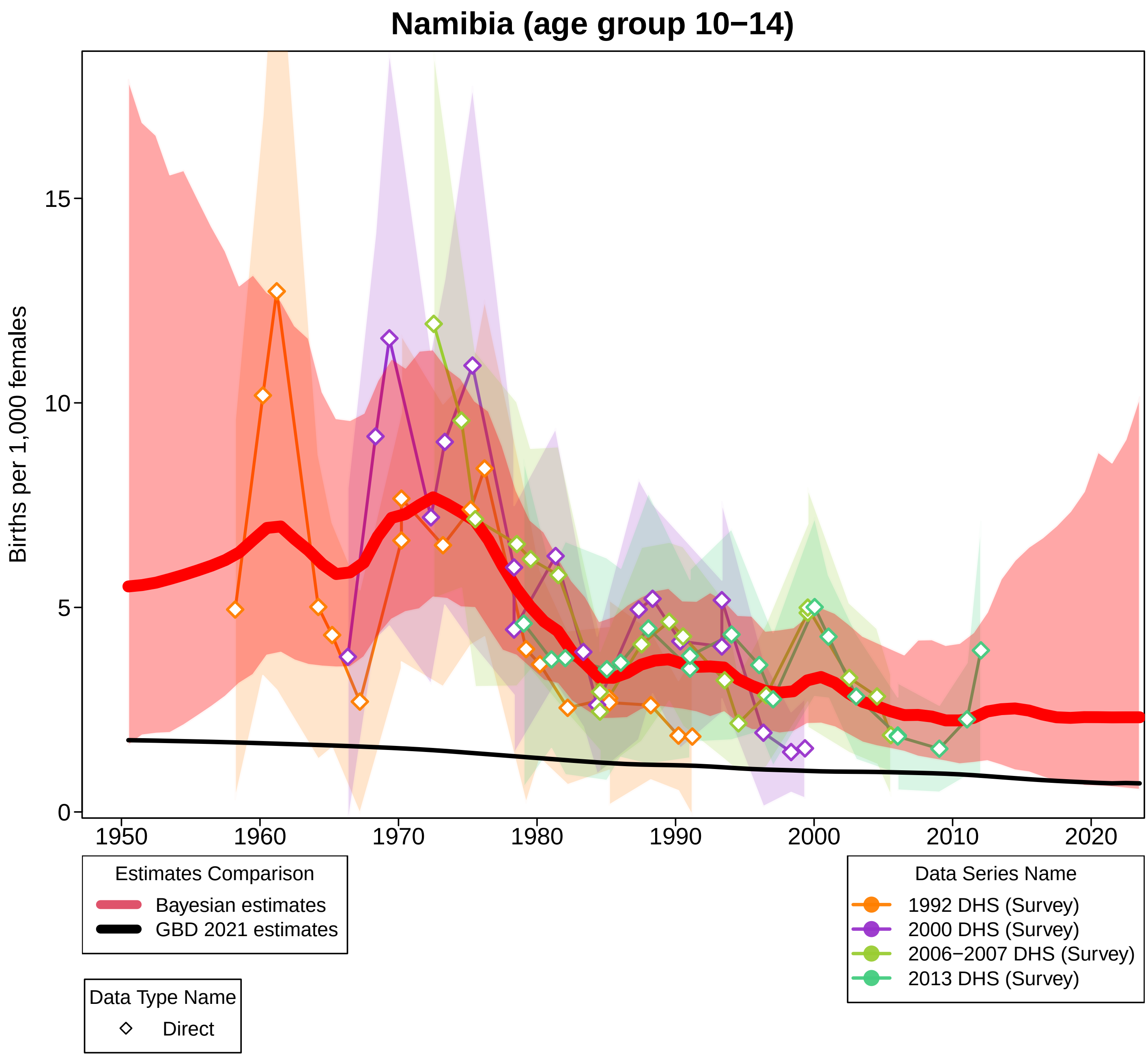
Namibia (age group 10−14)
Births per 1,000 females
0
5
10
15
1950
1960
1970
1980
1990
2000
2010
2020
Estimates Comparison
Bayesian estimates
GBD 2021 estimates
Data Type Name
Direct
Data Series Name
1992 DHS (Survey)
2000 DHS (Survey)
2006−2007 DHS (Survey)
2013 DHS (Survey)

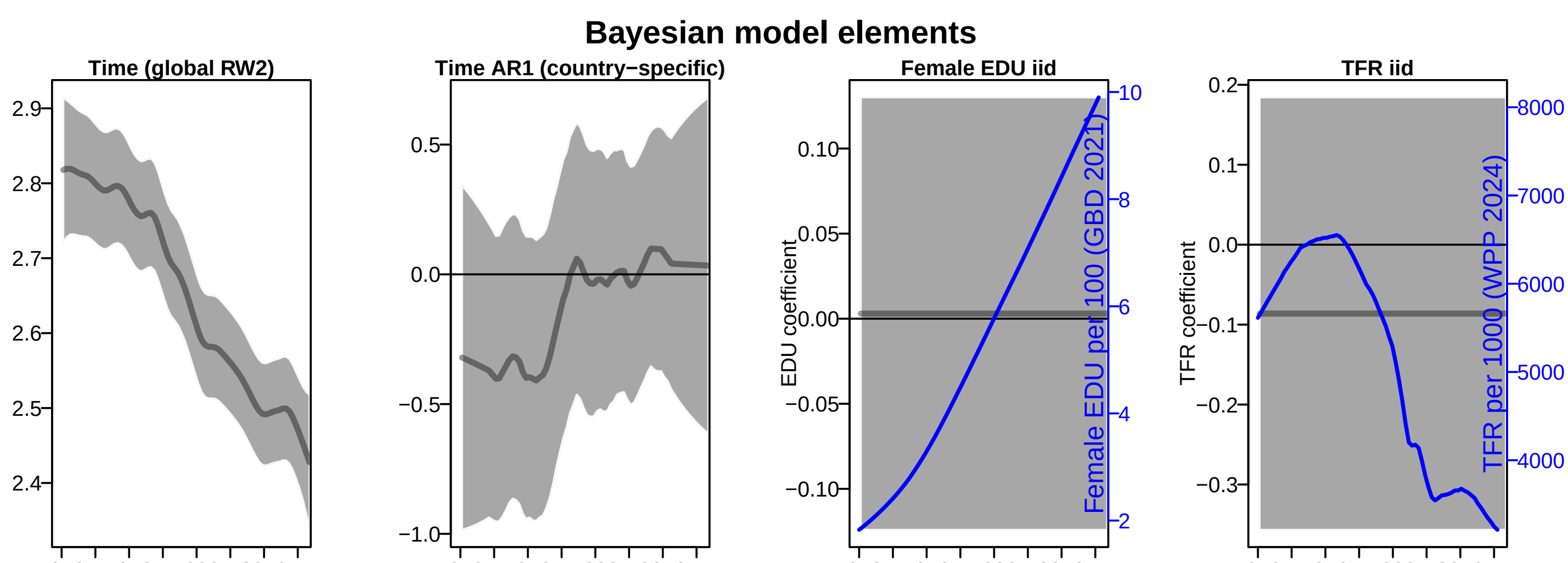
Bayesian model elements
Time (global RW2)
Time AR1 (country−specific)
Female EDU iid
TFR iid
EDU coefficient
Female EDU per 100 (GBD 2021)
TFR coefficient
TFR per 1000 (WPP 2024)
1950
1970
1990
2010

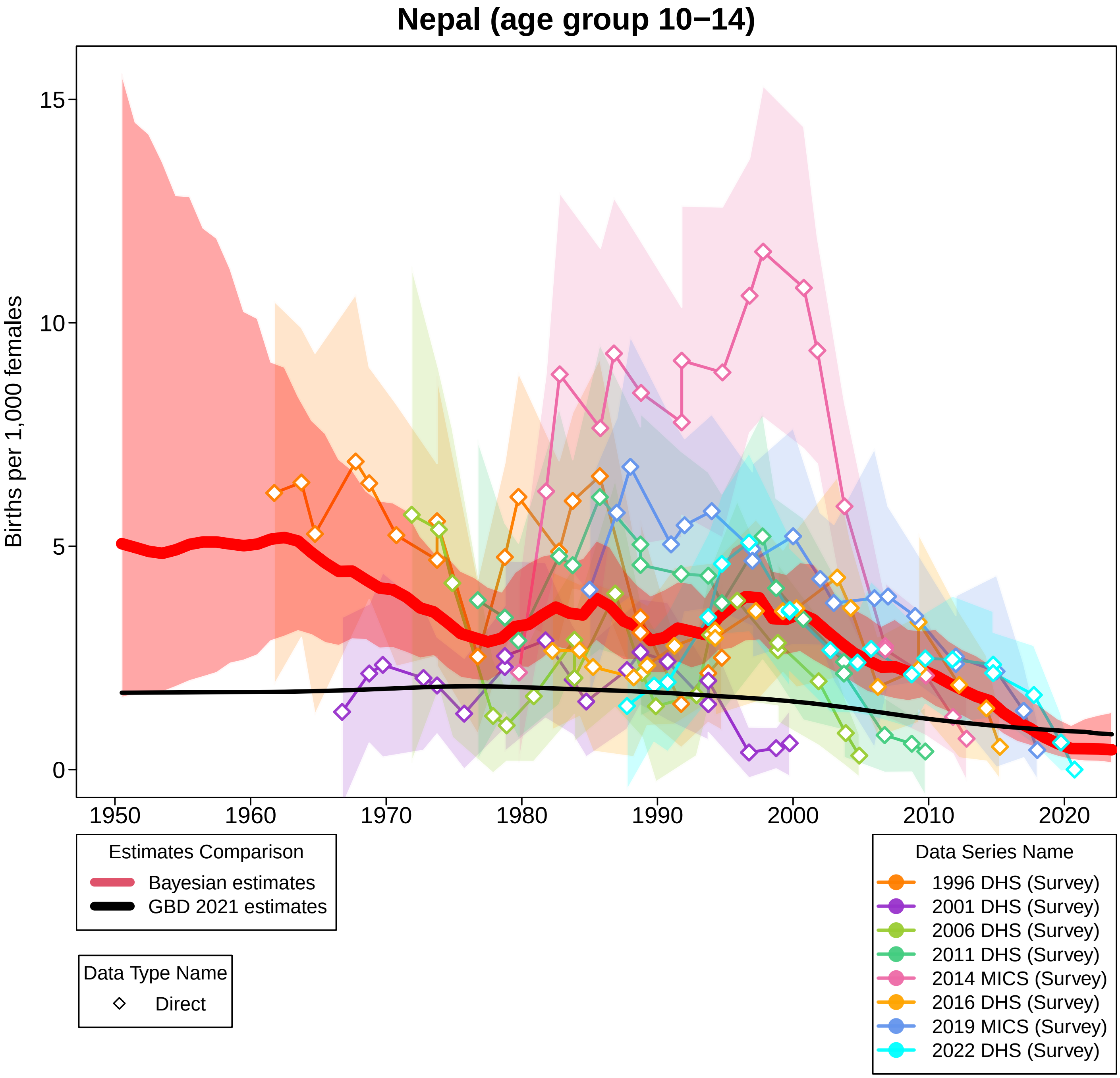

Nepal (age group 10−14)
Births per 1,000 females
0
5
10
15
1950
1960
1970
1980
1990
2000
2010
2020
Estimates Comparison
Bayesian estimates
GBD 2021 estimates
Data Type Name
Direct
Data Series Name
1996 DHS (Survey)
2001 DHS (Survey)
2006 DHS (Survey)
2011 DHS (Survey)
2014 MICS (Survey)
2016 DHS (Survey)
2019 MICS (Survey)
2022 DHS (Survey)

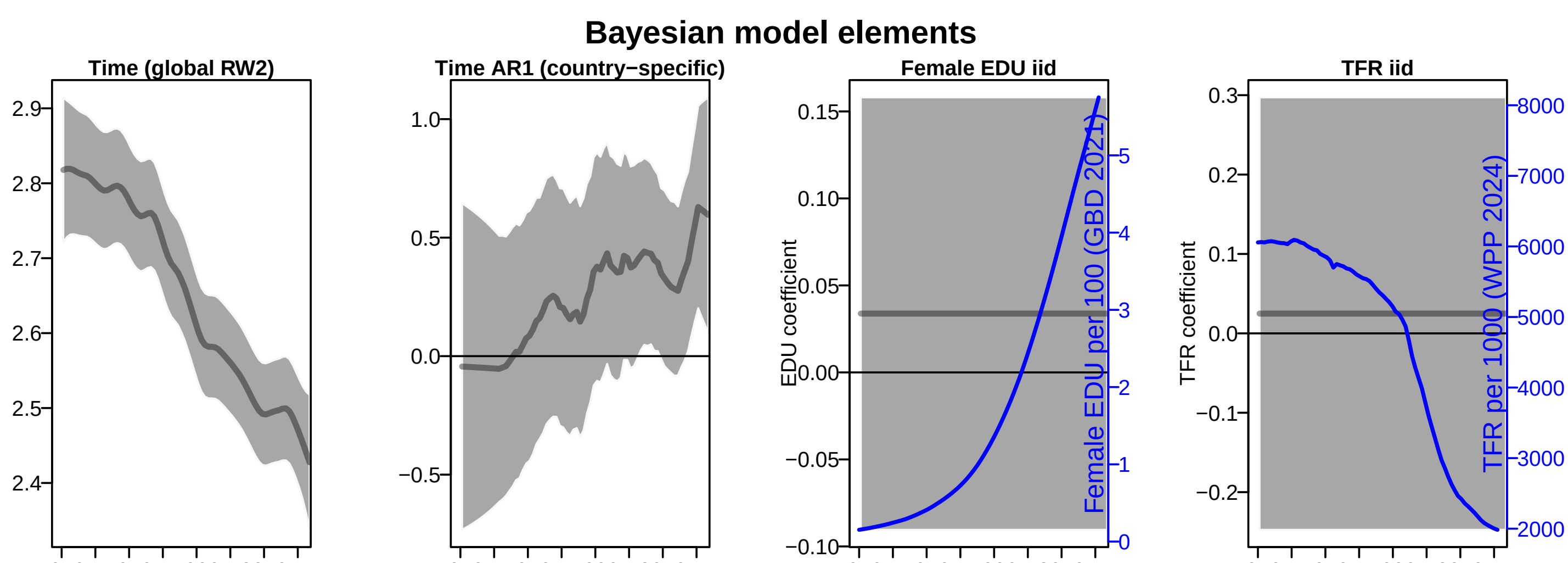

Bayesian model elements
Time (global RW2)
Time AR1 (country−specific)
Female EDU iid
TFR iid
EDU coefficient
Female EDU per 100 (GBD 2021)
TFR coefficient
TFR per 1000 (WPP 2024)
1950
1970
1990
2010

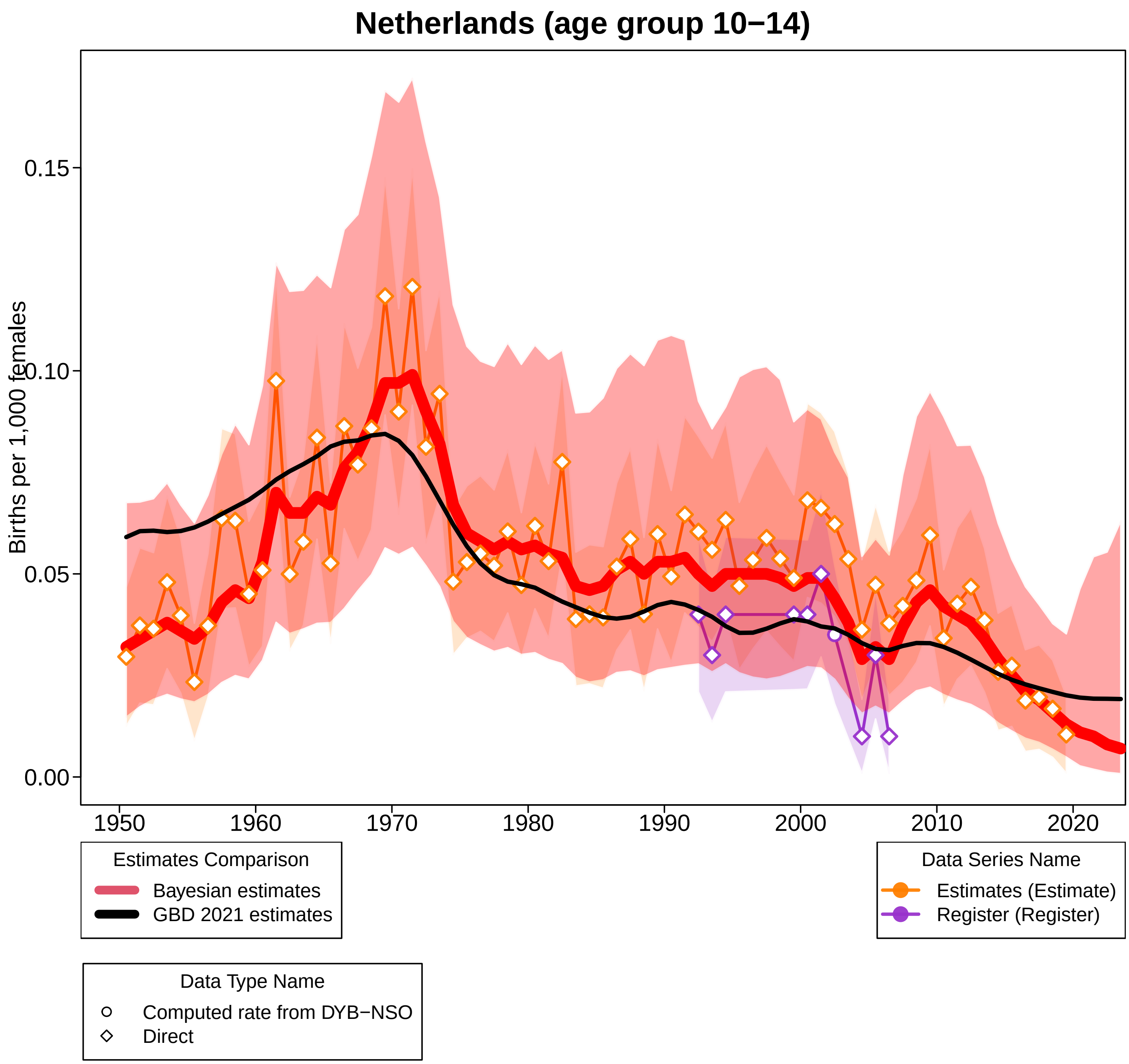

Netherlands (age group 10−14)
Births per 1,000 females
0.15
0.10
0.05
0.00
1950
1960
1970
1980
1990
2000
2010
2020
Estimates Comparison
Bayesian estimates
GBD 2021 estimates
Data Series Name
Estimates (Estimate)
Register (Register)
Data Type Name
Computed rate from DYB−NSO
Direct

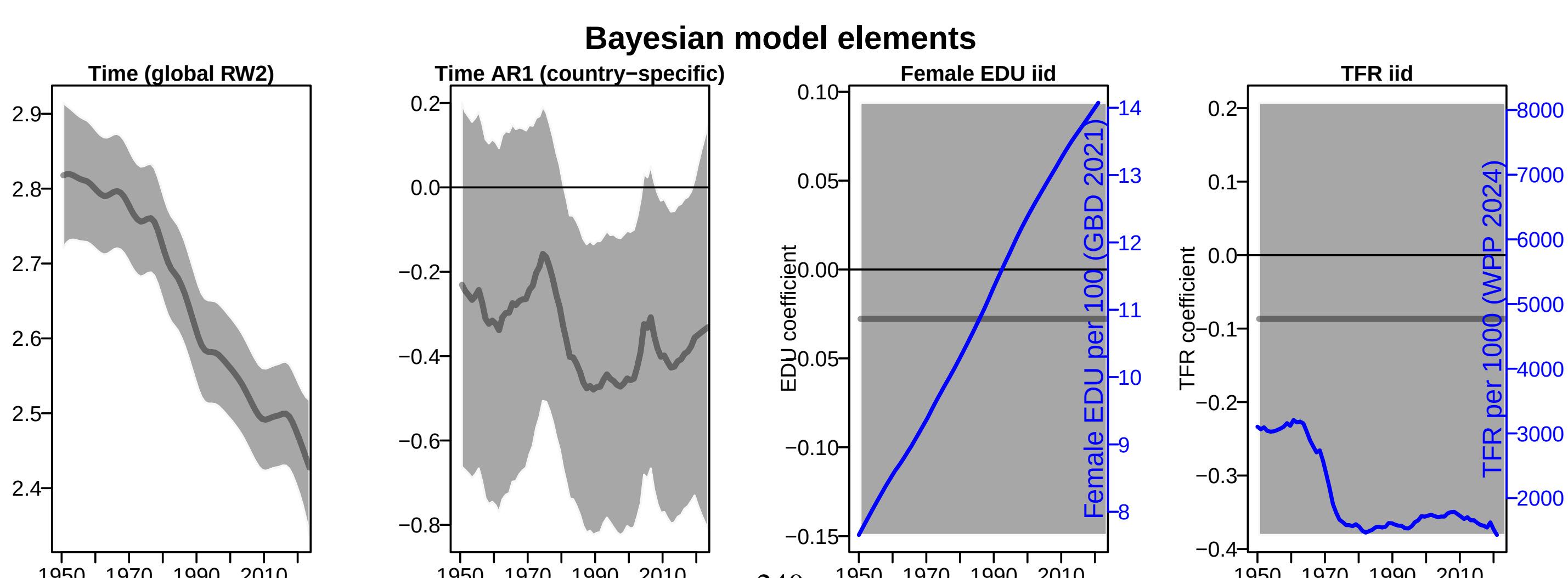

Bayesian model elements
Time (global RW2)
Time AR1 (country−specific)
Female EDU iid
TFR iid
EDU coefficient
Female EDU per 100 (GBD 2021)
TFR coefficient
TFR per 1000 (WPP 2024)

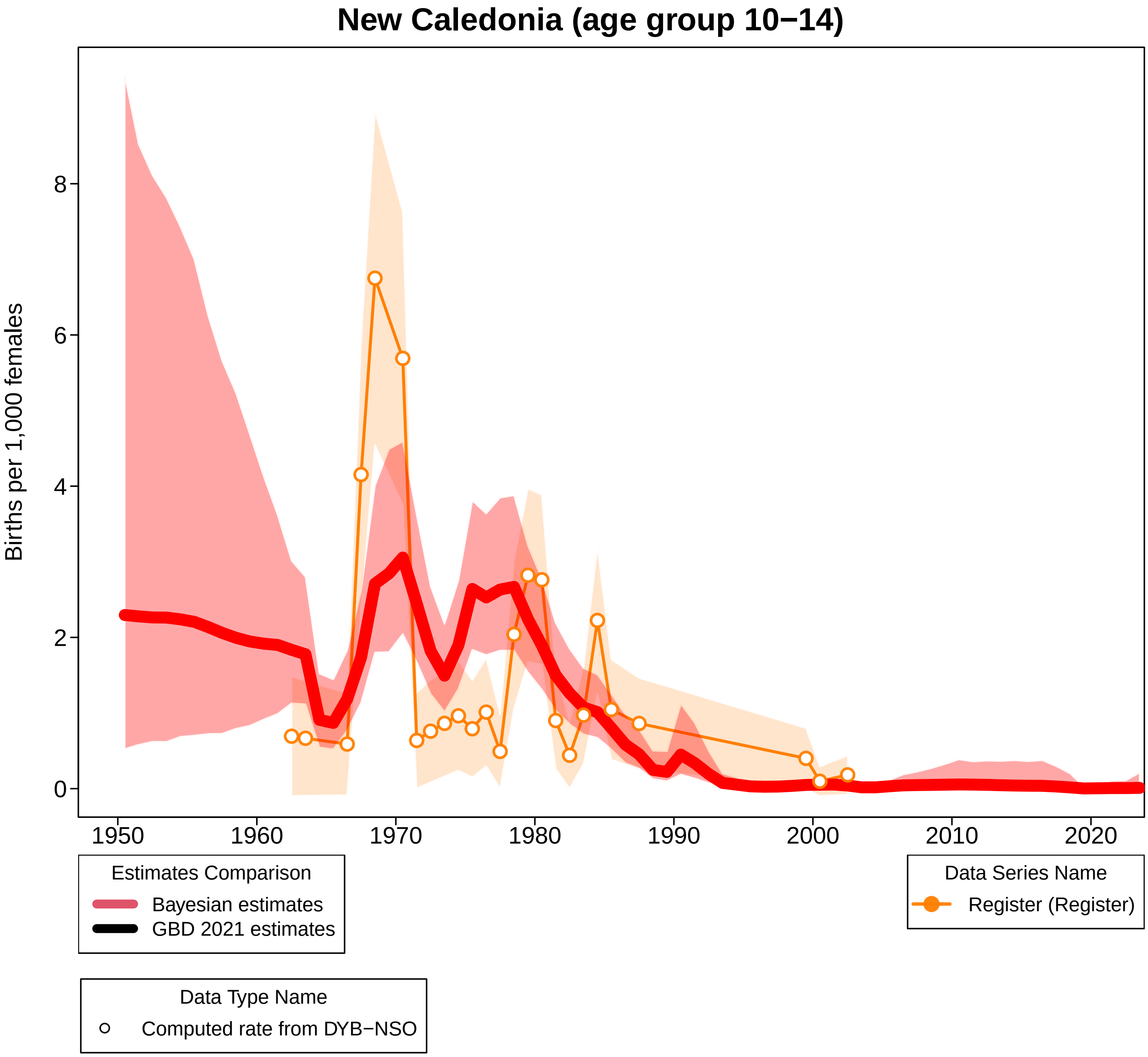
New Caledonia (age group 10−14)
Births per 1,000 females
0
2
4
6
8
1950
1960
1970
1980
1990
2000
2010
2020
Estimates Comparison
Bayesian estimates
GBD 2021 estimates
Data Series Name
Register (Register)
Data Type Name
Computed rate from DYB−NSO

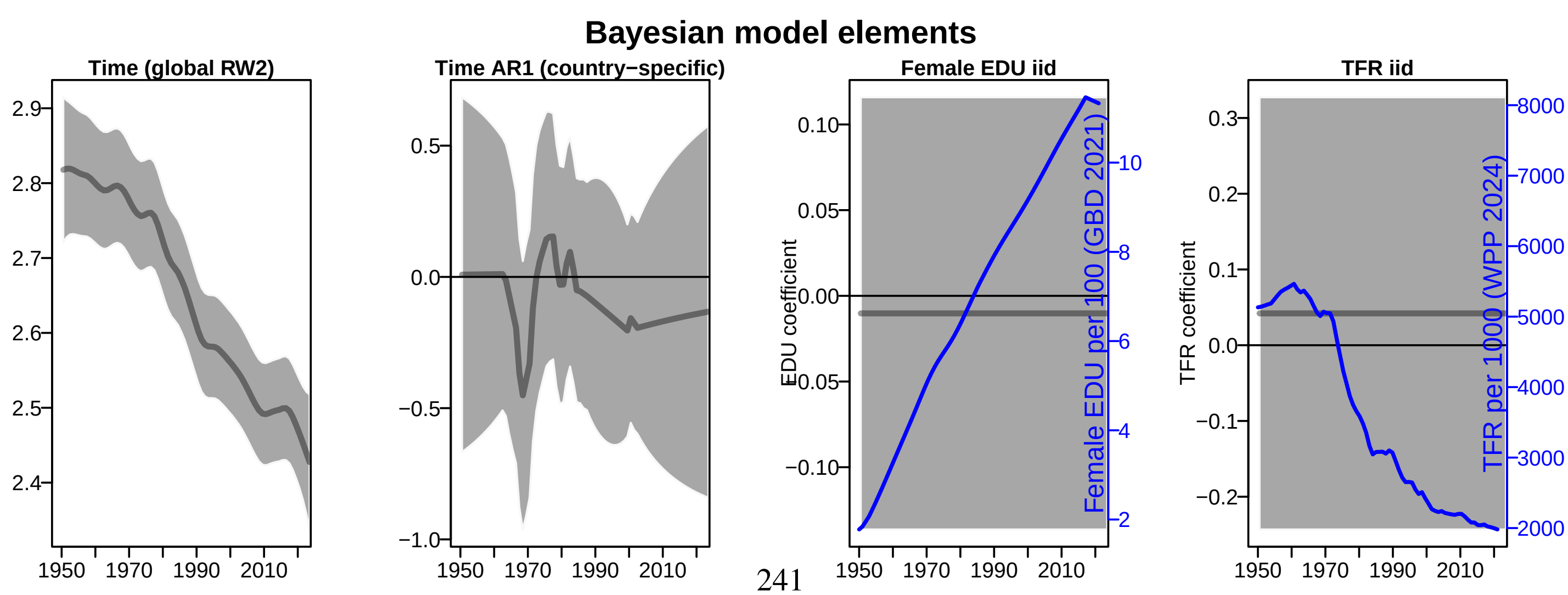
Bayesian model elements
Time (global RW2)
Time AR1 (country−specific)
Female EDU iid
TFR iid
EDU coefficient
Female EDU per 100 (GBD 2021)
TFR coefficient
TFR per 1000 (WPP 2024)
1950
1970
1990
2010

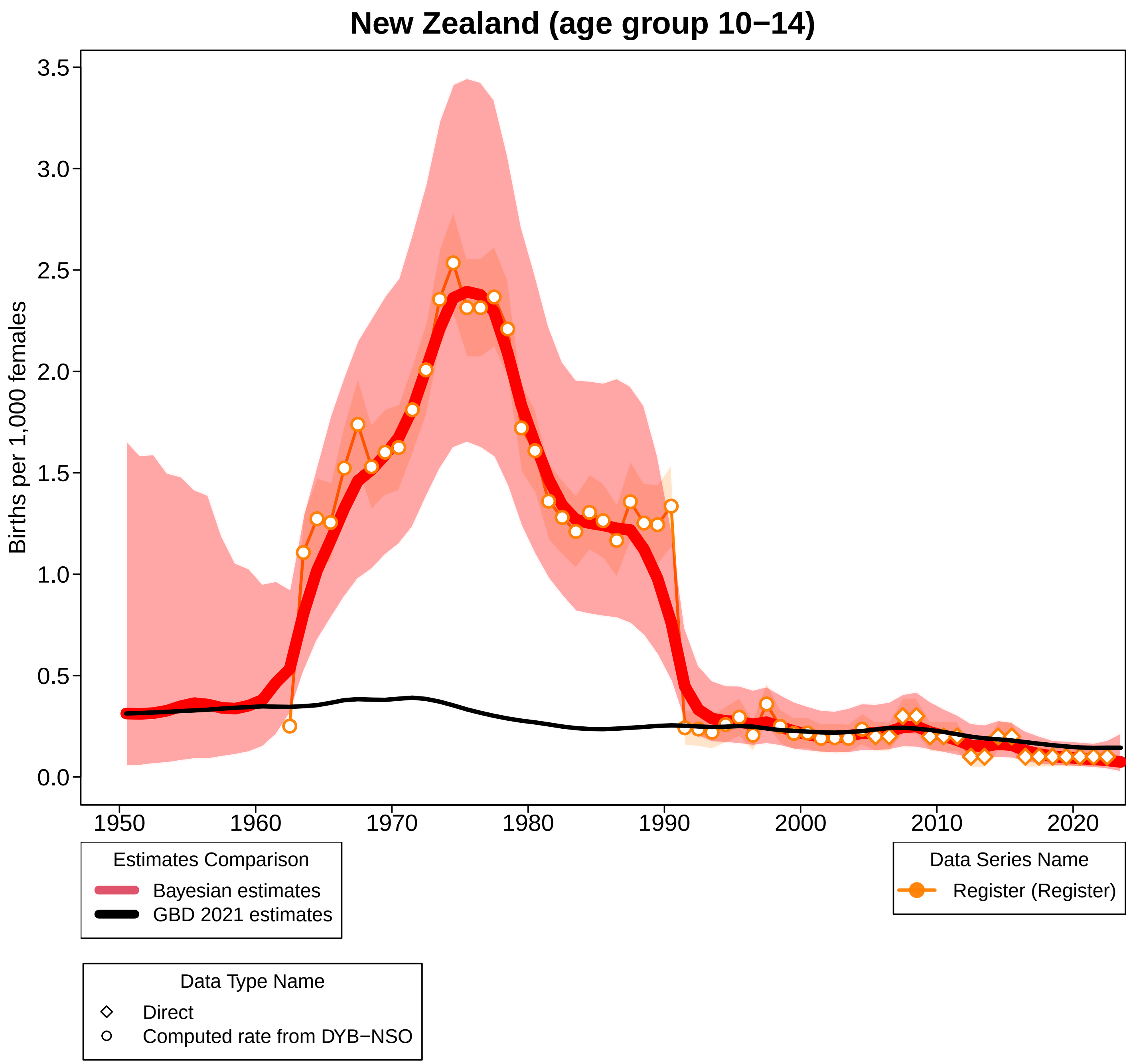
New Zealand (age group 10−14)
Births per 1,000 females
3.5
3.0
2.5
2.0
1.5
1.0
0.5
0.0
1950
1960
1970
1980
1990
2000
2010
2020
Estimates Comparison
Bayesian estimates
GBD 2021 estimates
Data Series Name
Register (Register)
Data Type Name
Direct
Computed rate from DYB−NSO

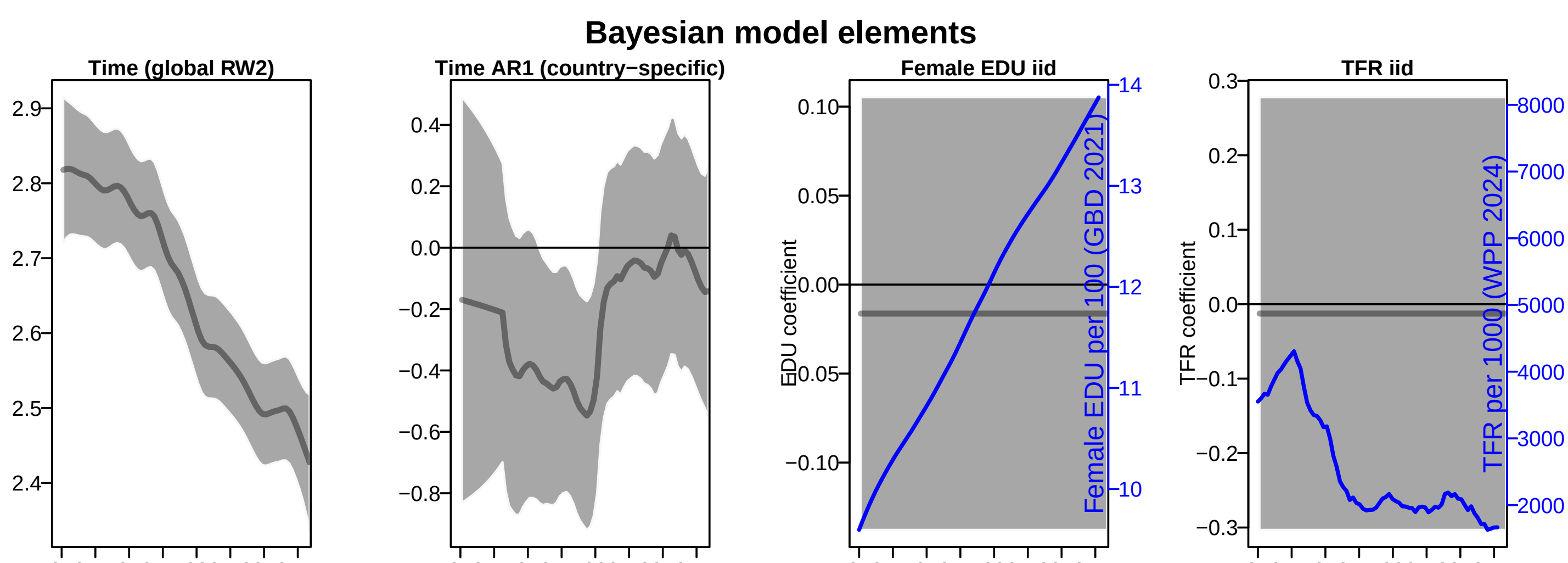
Bayesian model elements
Time (global RW2)
Time AR1 (country−specific)
Female EDU iid
TFR iid
EDU coefficient
Female EDU per 100 (GBD 2021)
TFR coefficient
TFR per 1000 (WPP 2024)
1950
1970
1990
2010

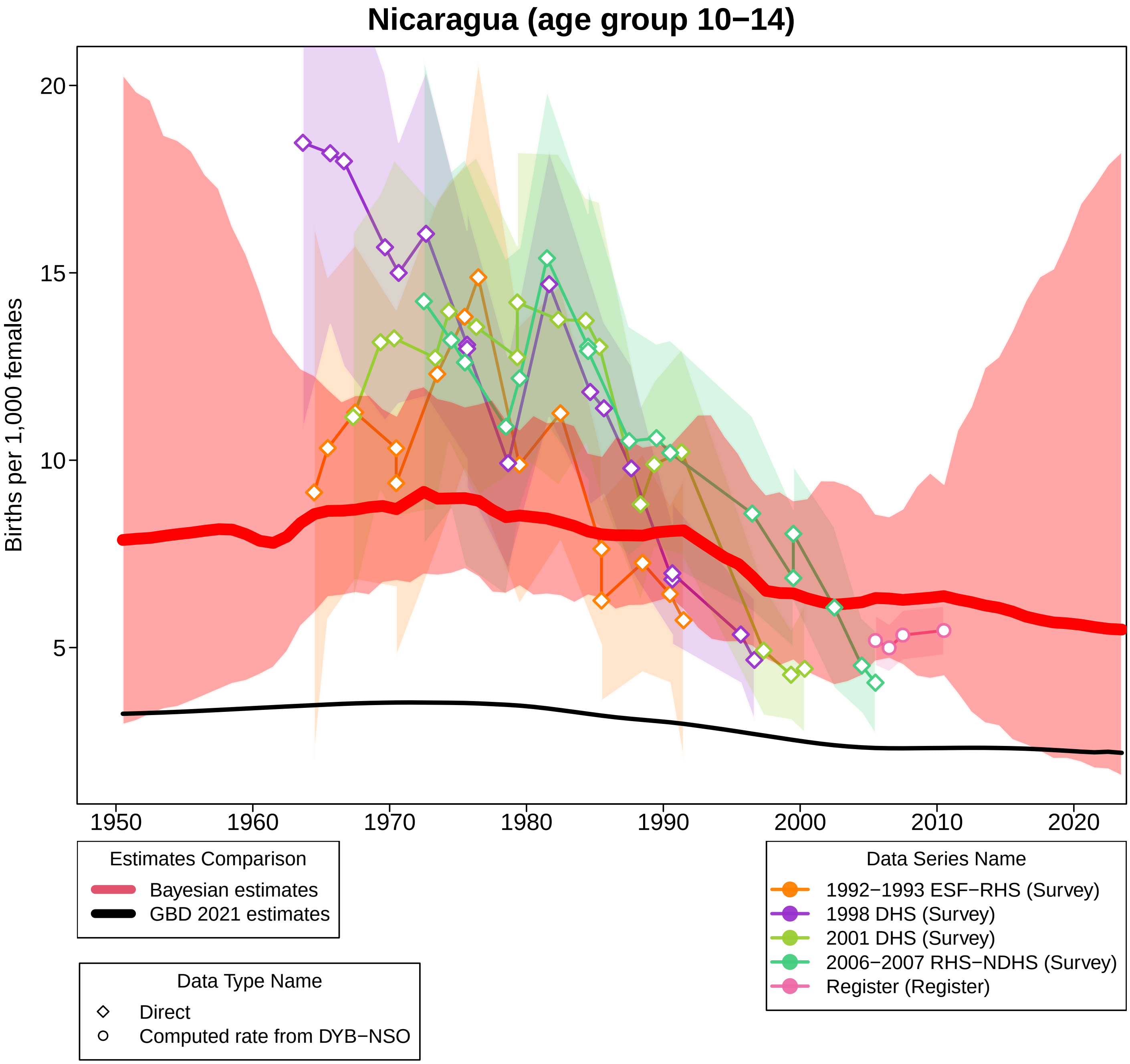

Nicaragua (age group 10−14)
Births per 1,000 females
20
15
10
5
1950
1960
1970
1980
1990
2000
2010
2020
Estimates Comparison
Bayesian estimates
GBD 2021 estimates
Data Type Name
Direct
Computed rate from DYB−NSO
Data Series Name
1992−1993 ESF−RHS (Survey)
1998 DHS (Survey)
2001 DHS (Survey)
2006−2007 RHS−NDHS (Survey)
Register (Register)

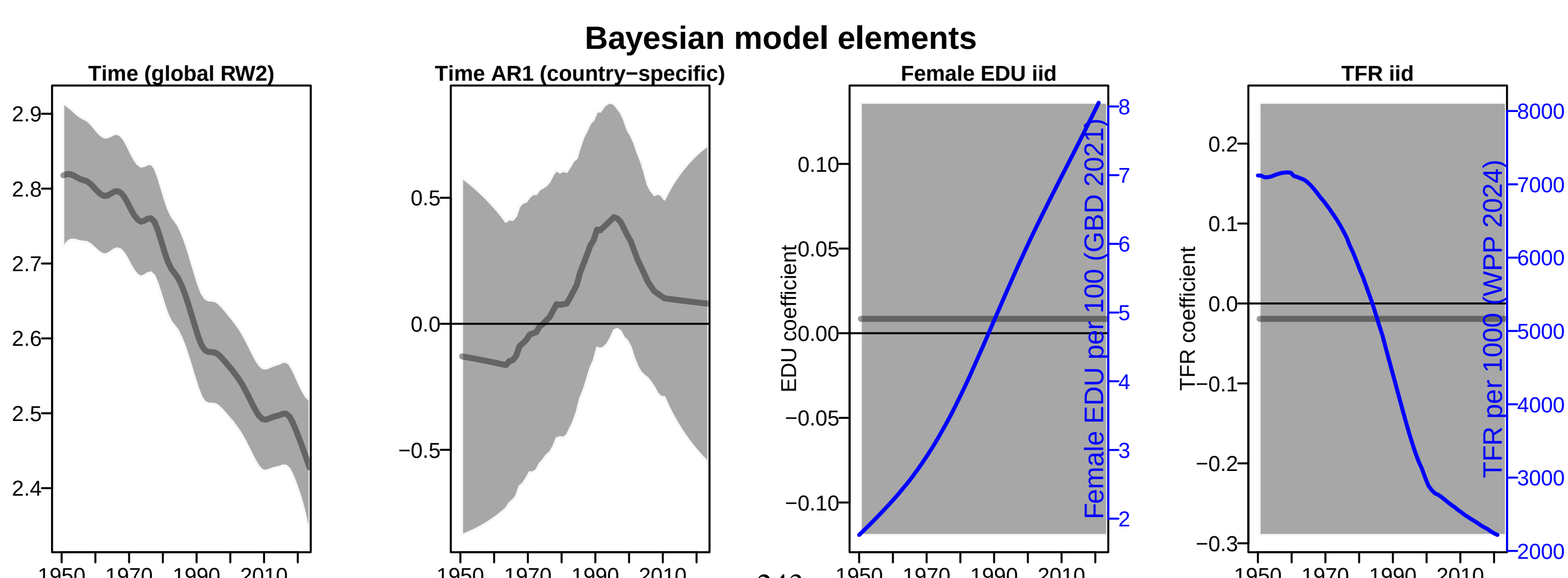

Bayesian model elements
Time (global RW2)
Time AR1 (country−specific)
Female EDU iid
TFR iid
EDU coefficient
Female EDU per 100 (GBD 2021)
TFR coefficient
TFR per 1000 (WPP 2024)
1950
1970
1990
2010

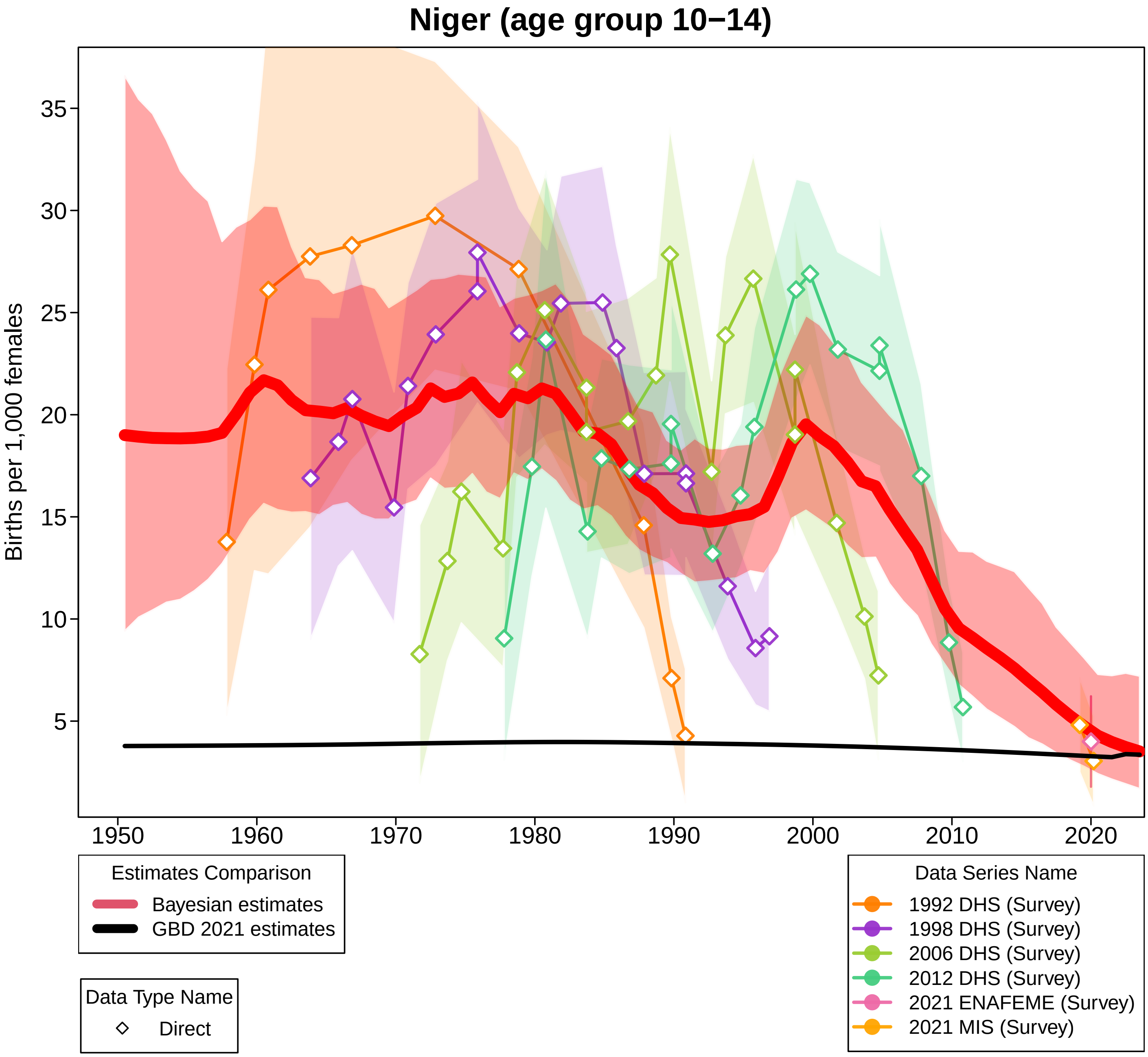
Niger (age group 10−14)
Births per 1,000 females
35
30
25
20
15
10
5
1950
1960
1970
1980
1990
2000
2010
2020
Estimates Comparison
Bayesian estimates
GBD 2021 estimates
Data Type Name
Direct
Data Series Name
1992 DHS (Survey)
1998 DHS (Survey)
2006 DHS (Survey)
2012 DHS (Survey)
2021 ENAFEME (Survey)
2021 MIS (Survey)

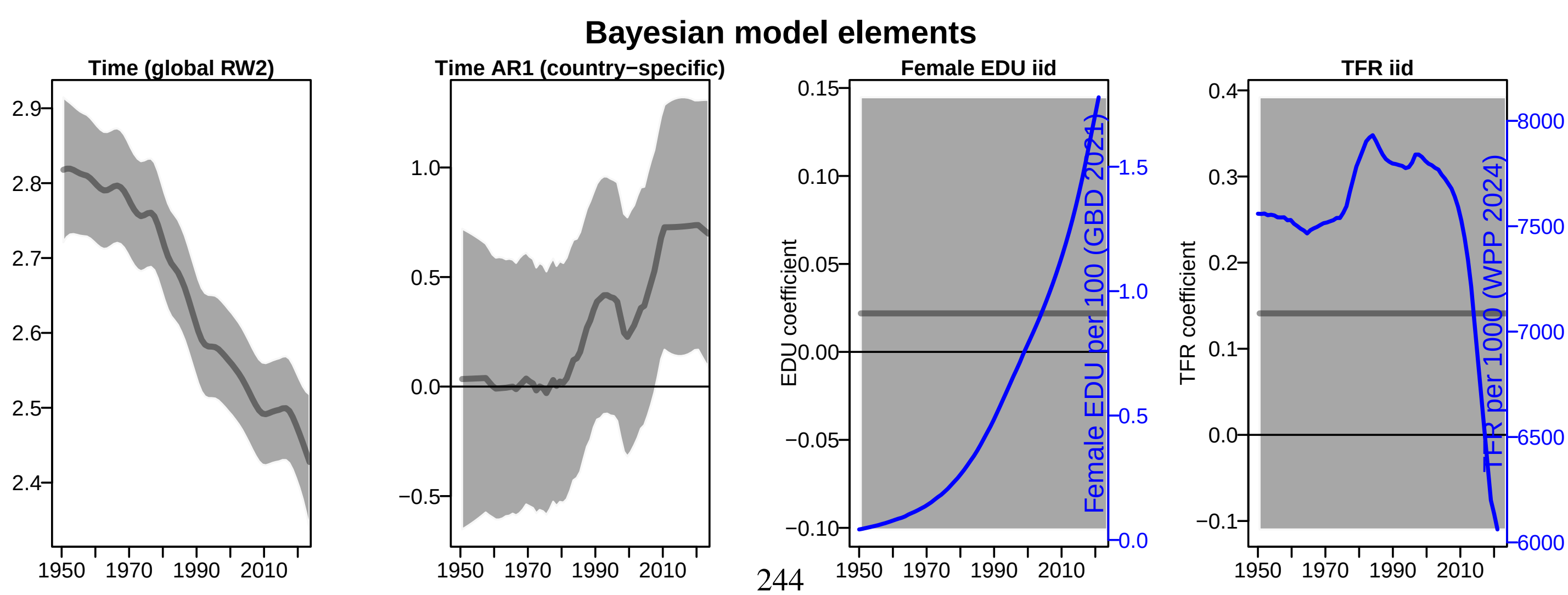
Bayesian model elements
Time (global RW2)
Time AR1 (country−specific)
Female EDU iid
TFR iid
EDU coefficient
Female EDU per 100 (GBD 2021)
TFR coefficient
TFR per 1000 (WPP 2024)

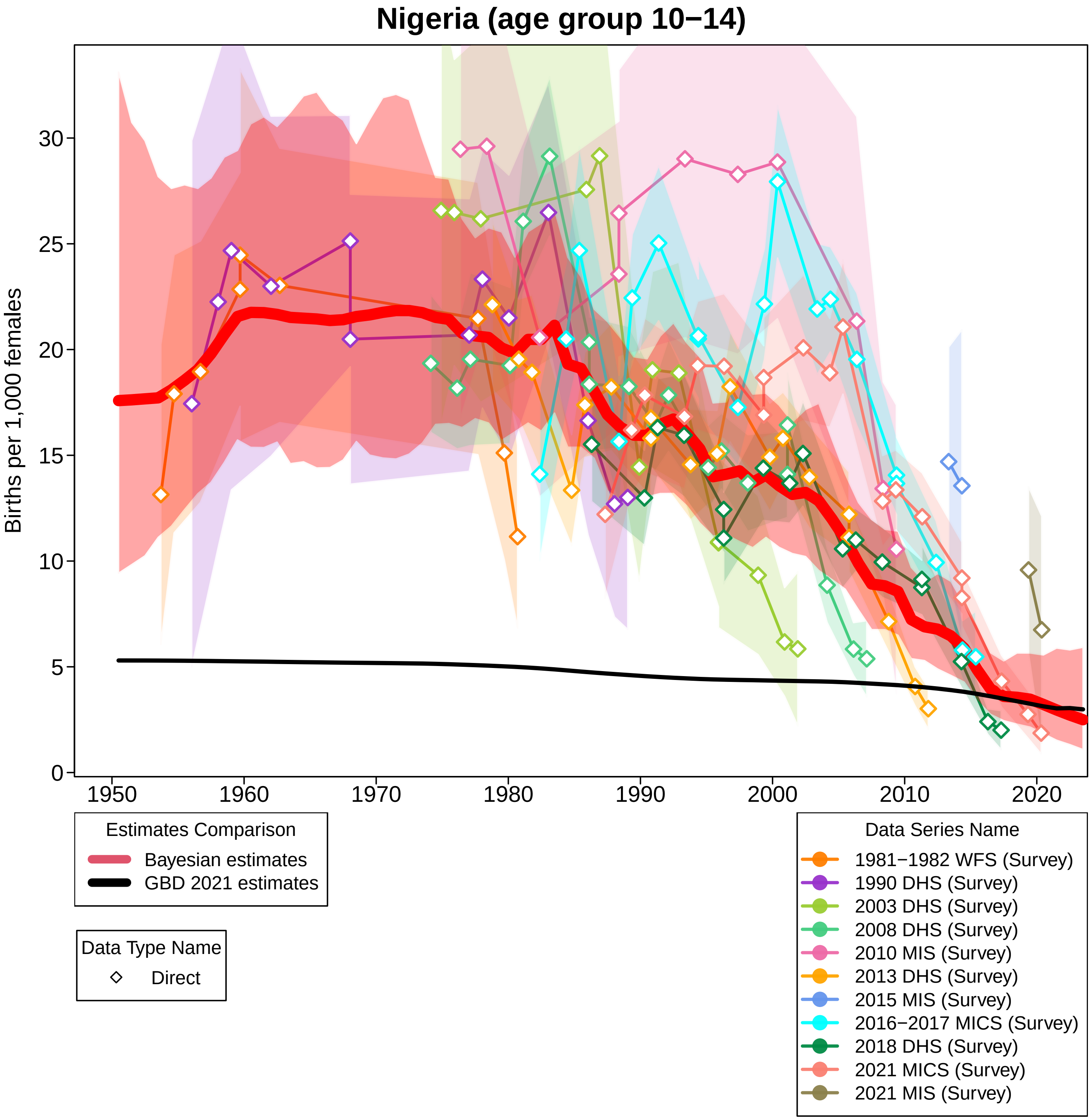

Nigeria (age group 10−14)
Births per 1,000 females
0
5
10
15
20
25
30
1950
1960
1970
1980
1990
2000
2010
2020
Estimates Comparison
Bayesian estimates
GBD 2021 estimates
Data Type Name
Direct
Data Series Name
1981−1982 WFS (Survey)
1990 DHS (Survey)
2003 DHS (Survey)
2008 DHS (Survey)
2010 MIS (Survey)
2013 DHS (Survey)
2015 MIS (Survey)
2016−2017 MICS (Survey)
2018 DHS (Survey)
2021 MICS (Survey)
2021 MIS (Survey)

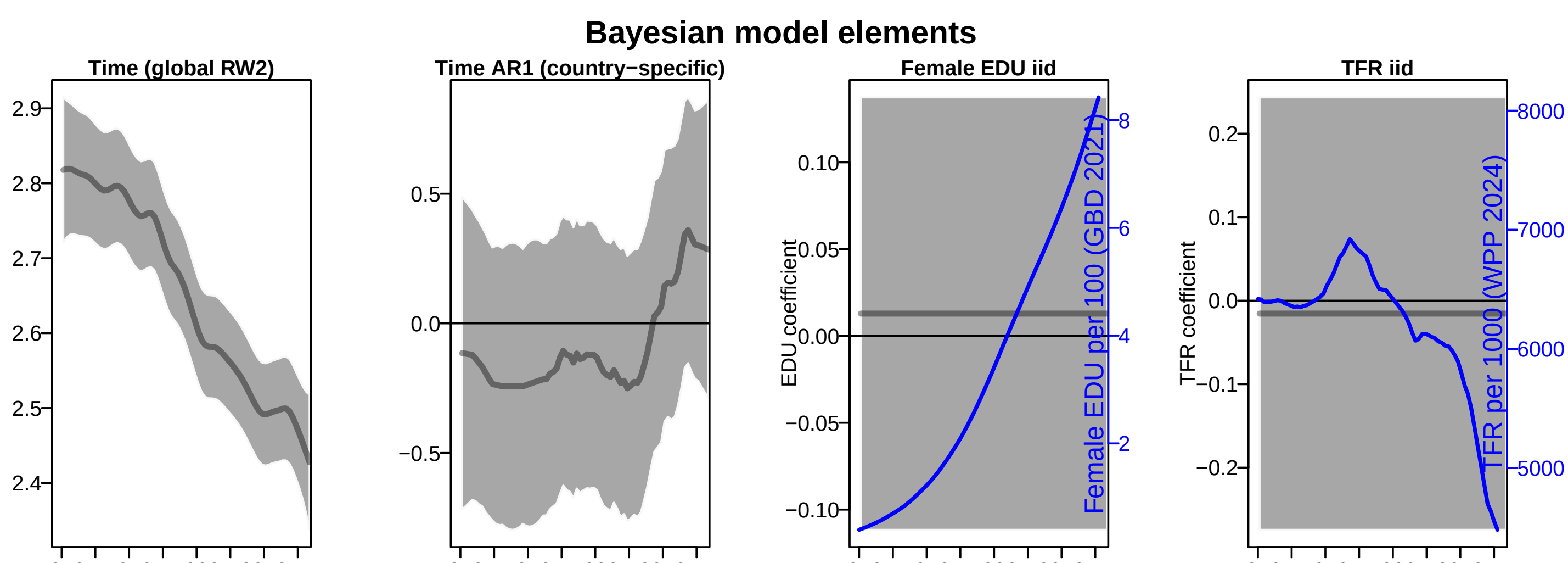

Bayesian model elements
Time (global RW2)
Time AR1 (country−specific)
Female EDU iid
TFR iid
EDU coefficient
Female EDU per 100 (GBD 2021)
TFR coefficient
TFR per 1000 (WPP 2024)
1950
1970
1990
2010

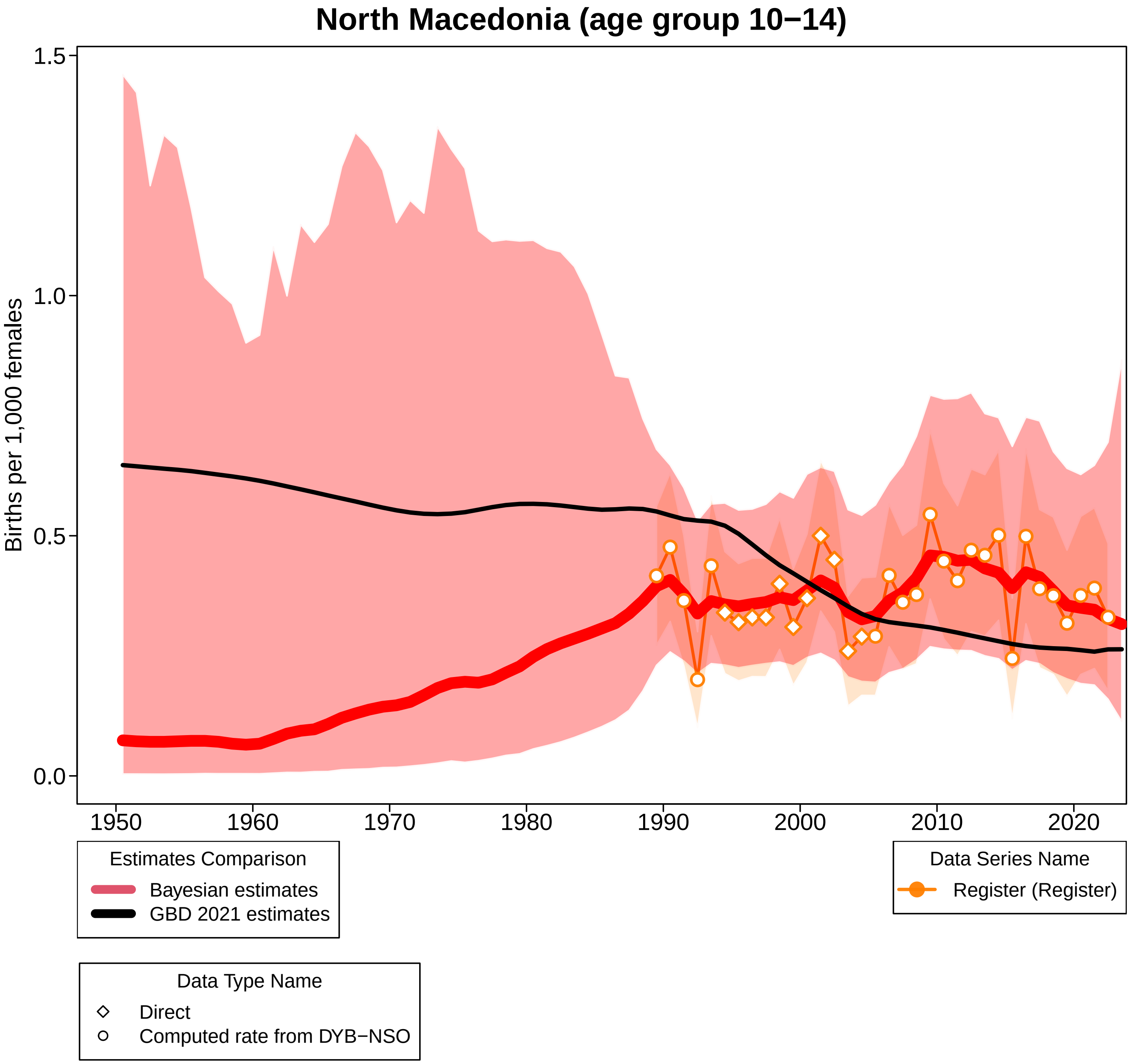

North Macedonia (age group 10−14)
Births per 1,000 females
1.5
1.0
0.5
0.0
1950
1960
1970
1980
1990
2000
2010
2020
Estimates Comparison
Bayesian estimates
GBD 2021 estimates
Data Series Name
Register (Register)
Data Type Name
Direct
Computed rate from DYB−NSO

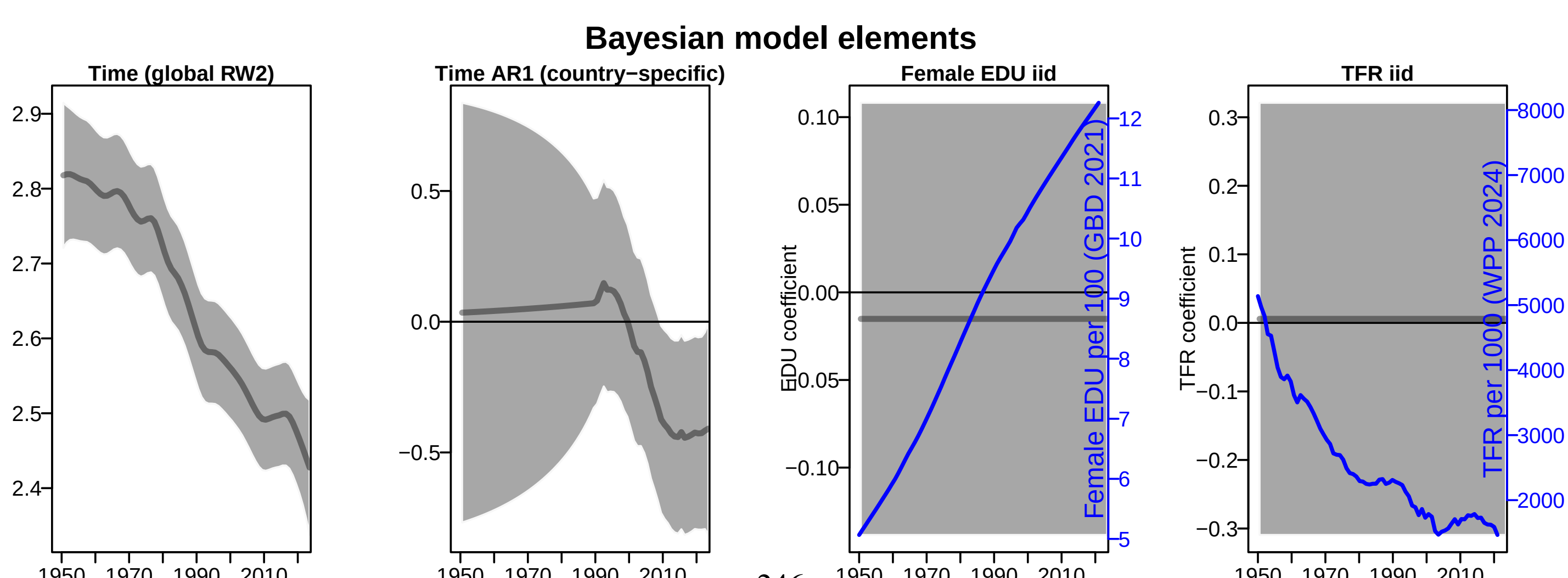

Bayesian model elements
Time (global RW2)
Time AR1 (country−specific)
Female EDU iid
TFR iid
EDU coefficient
Female EDU per 100 (GBD 2021)
TFR coefficient
TFR per 1000 (WPP 2024)
1950
1970
1990
2010

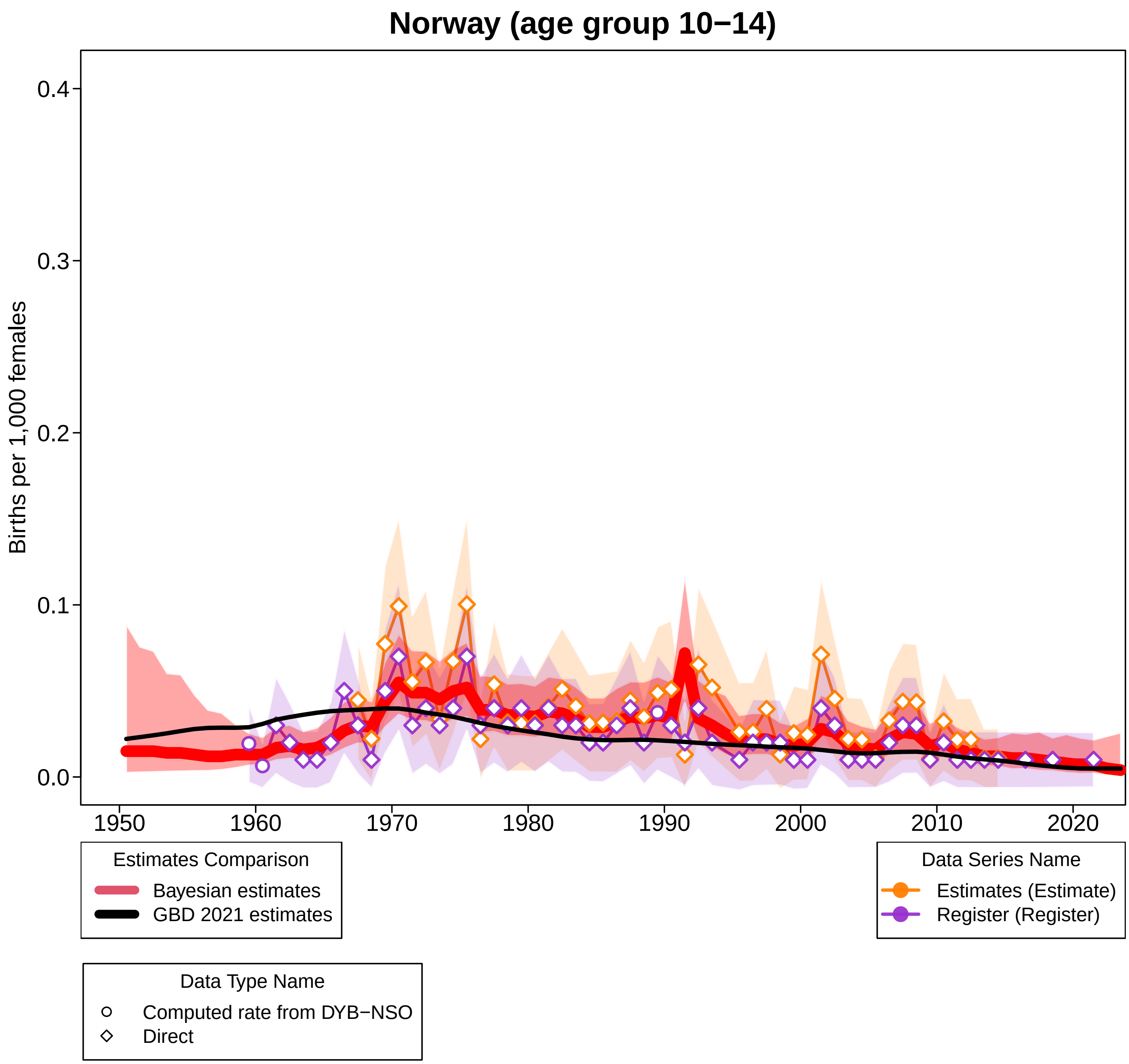
Norway (age group 10−14)
Births per 1,000 females
0.4
0.3
0.2
0.1
0.0
1950
1960
1970
1980
1990
2000
2010
2020
Estimates Comparison
Bayesian estimates
GBD 2021 estimates
Data Series Name
Estimates (Estimate)
Register (Register)
Data Type Name
Computed rate from DYB−NSO
Direct

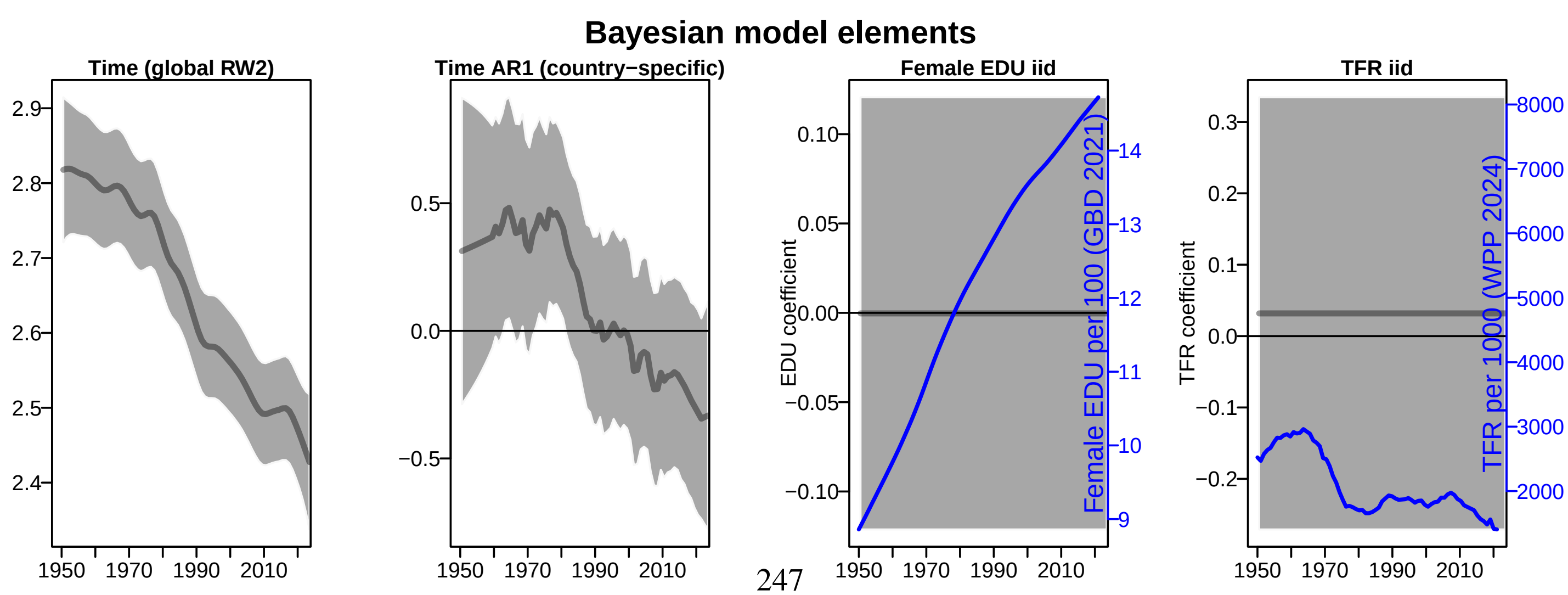
Bayesian model elements
Time (global RW2)
Time AR1 (country−specific)
Female EDU iid
TFR iid
EDU coefficient
Female EDU per 100 (GBD 2021)
TFR coefficient
TFR per 1000 (WPP 2024)

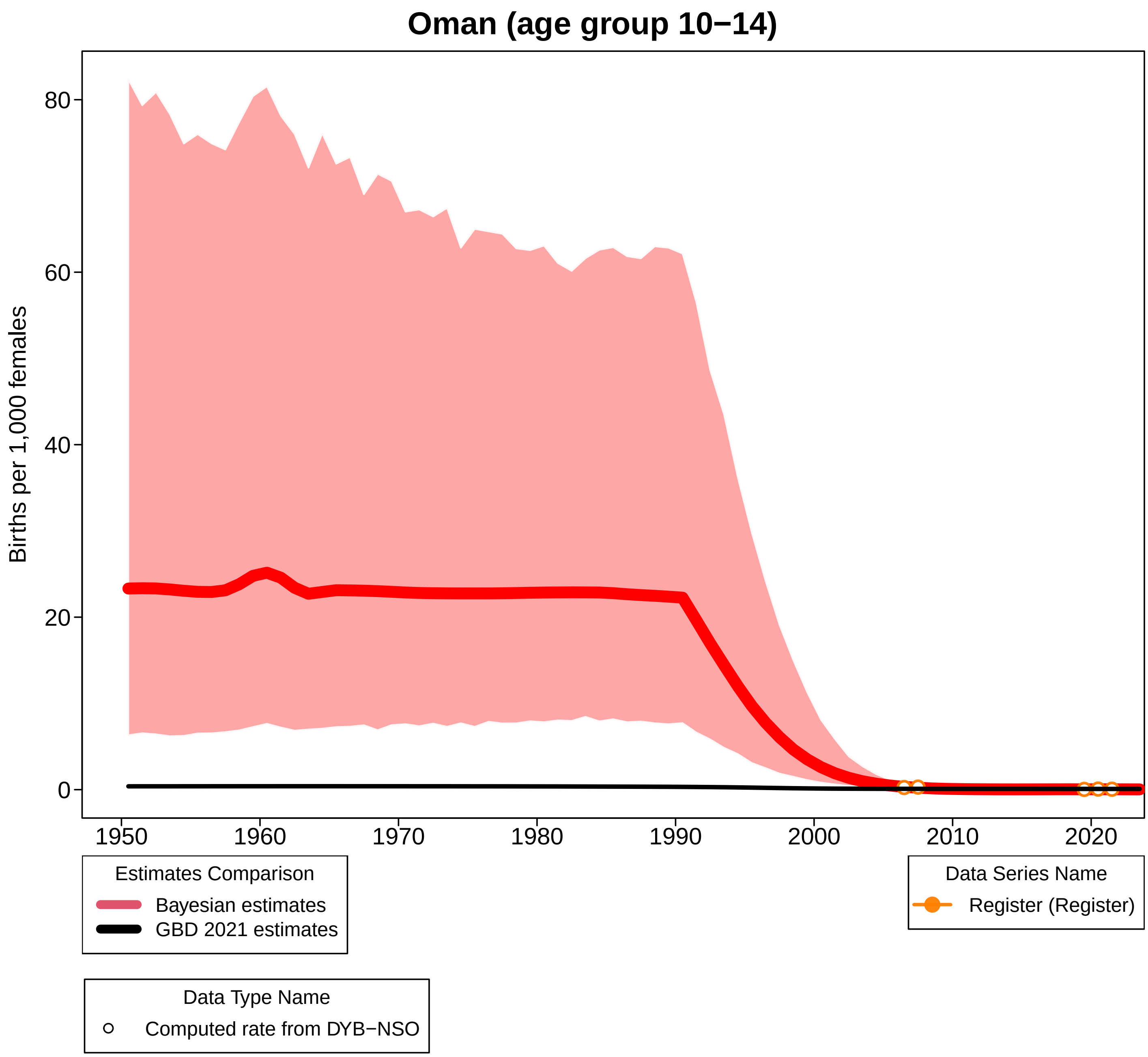
Oman (age group 10−14)
Births per 1,000 females
80
60
40
20
0
1950
1960
1970
1980
1990
2000
2010
2020
Estimates Comparison
Bayesian estimates
GBD 2021 estimates
Data Series Name
Register (Register)
Data Type Name
Computed rate from DYB−NSO

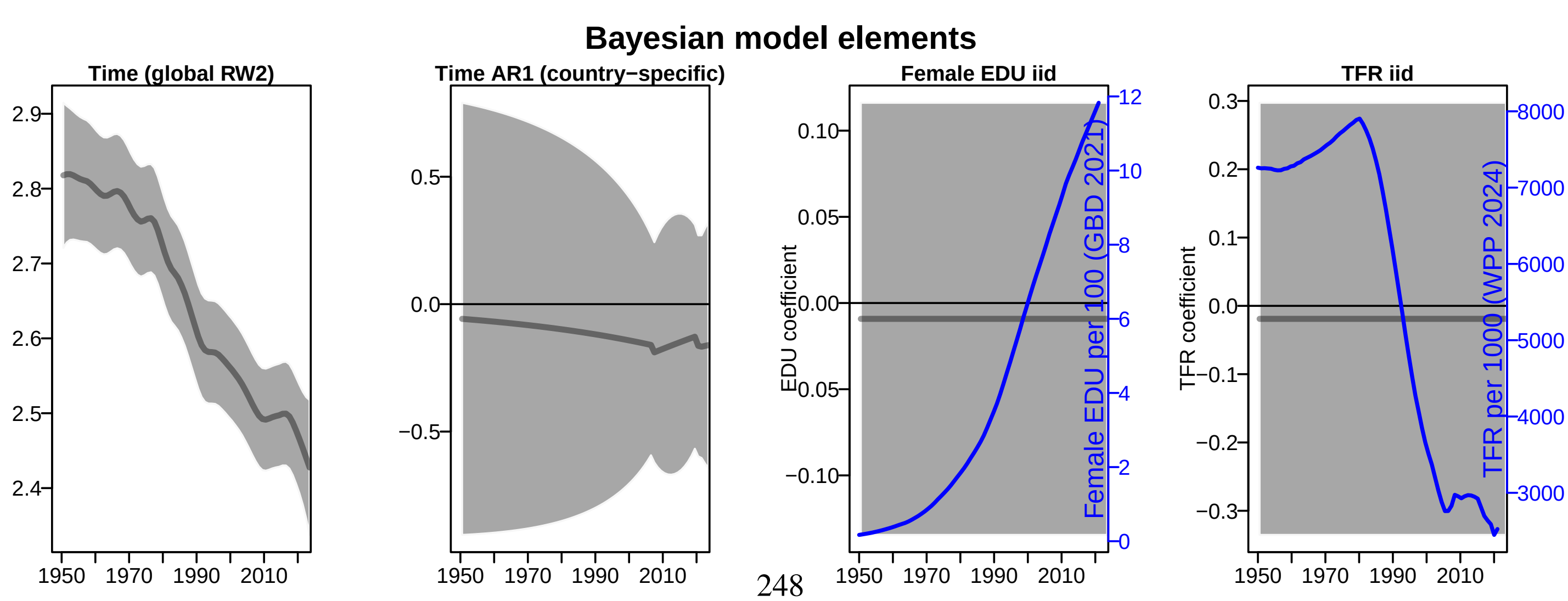
Bayesian model elements
Time (global RW2)
Time AR1 (country−specific)
Female EDU iid
TFR iid
EDU coefficient
Female EDU per 100 (GBD 2021)
TFR coefficient
TFR per 1000 (WPP 2024)
1950
1970
1990
2010

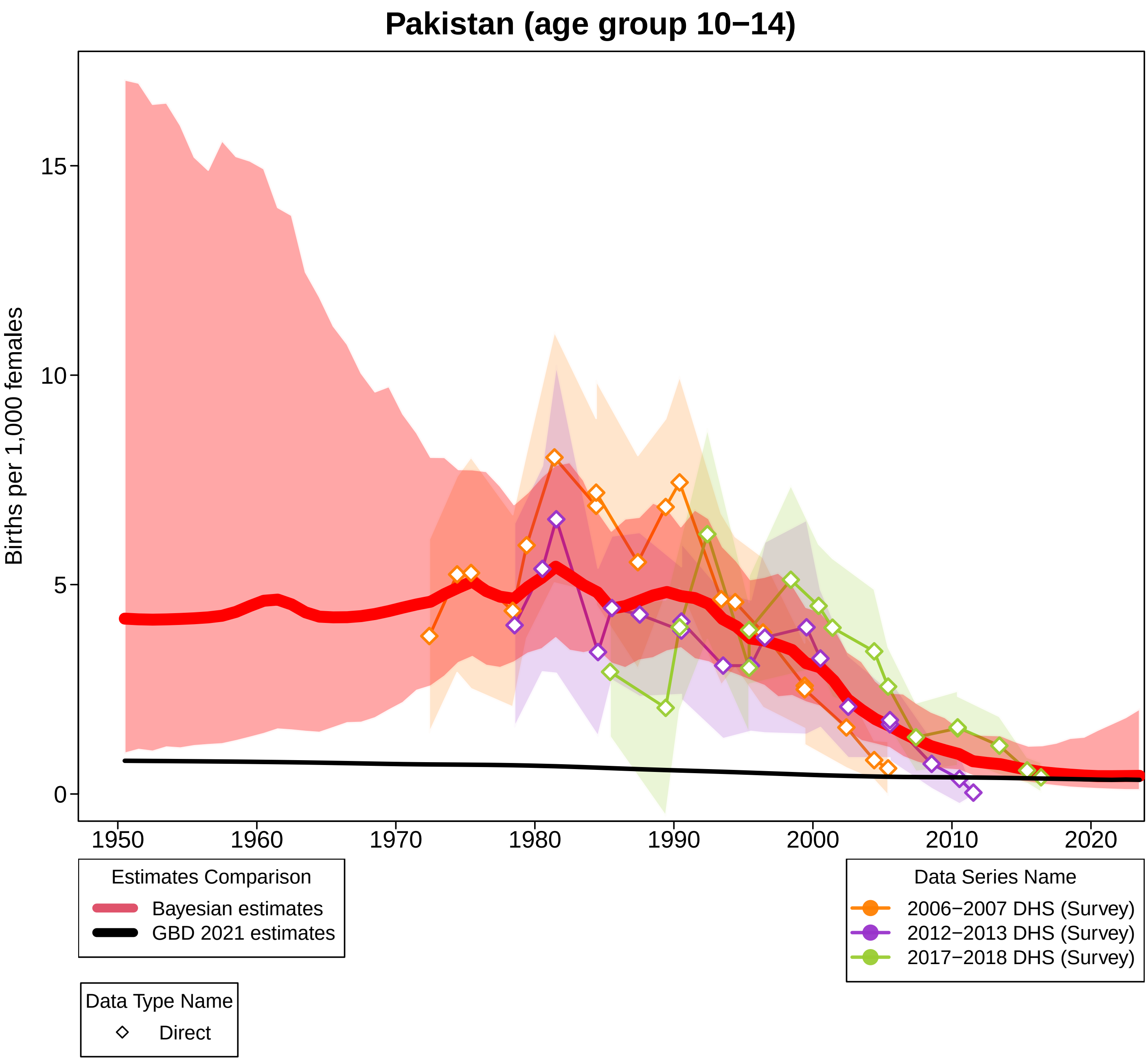

Pakistan (age group 10−14)
Births per 1,000 females
0
5
10
15
1950
1960
1970
1980
1990
2000
2010
2020
Estimates Comparison
Bayesian estimates
GBD 2021 estimates
Data Series Name
2006−2007 DHS (Survey)
2012−2013 DHS (Survey)
2017−2018 DHS (Survey)
Data Type Name
Direct

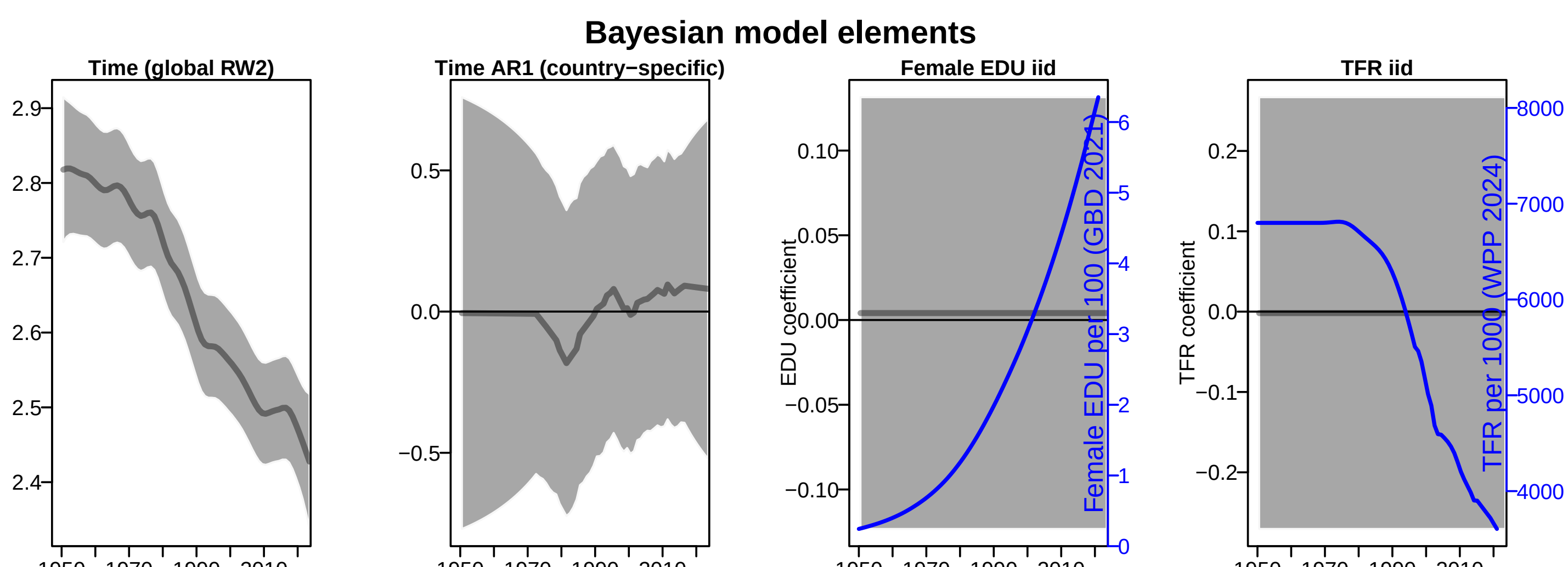

Bayesian model elements
Time (global RW2)
Time AR1 (country−specific)
Female EDU iid
TFR iid
EDU coefficient
Female EDU per 100 (GBD 2021)
TFR coefficient
TFR per 1000 (WPP 2024)
1950
1970
1990
2010

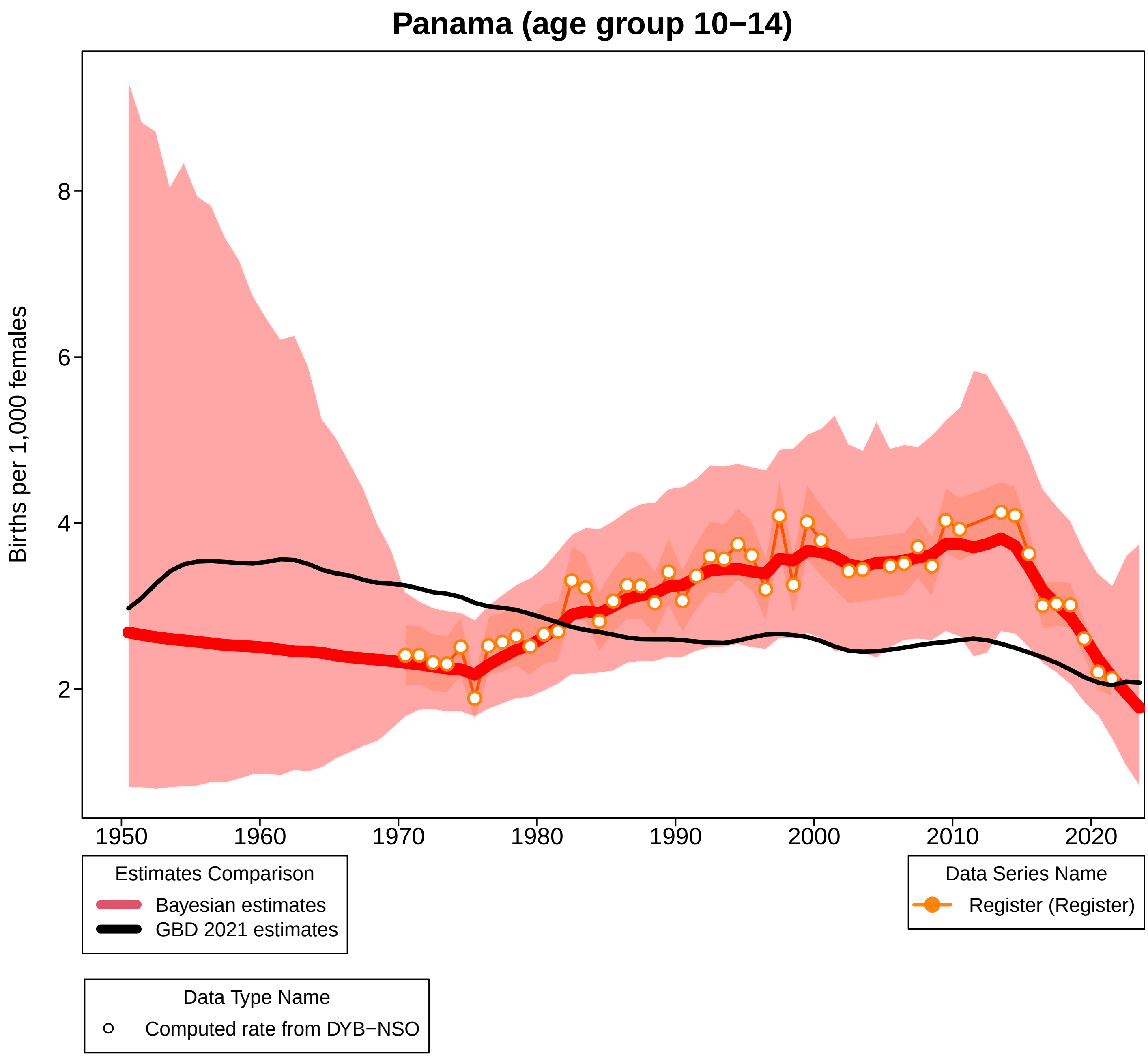
Panama (age group 10−14)
Births per 1,000 females
8
6
4
2
1950
1960
1970
1980
1990
2000
2010
2020
Estimates Comparison
Bayesian estimates
GBD 2021 estimates
Data Series Name
Register (Register)
Data Type Name
Computed rate from DYB−NSO

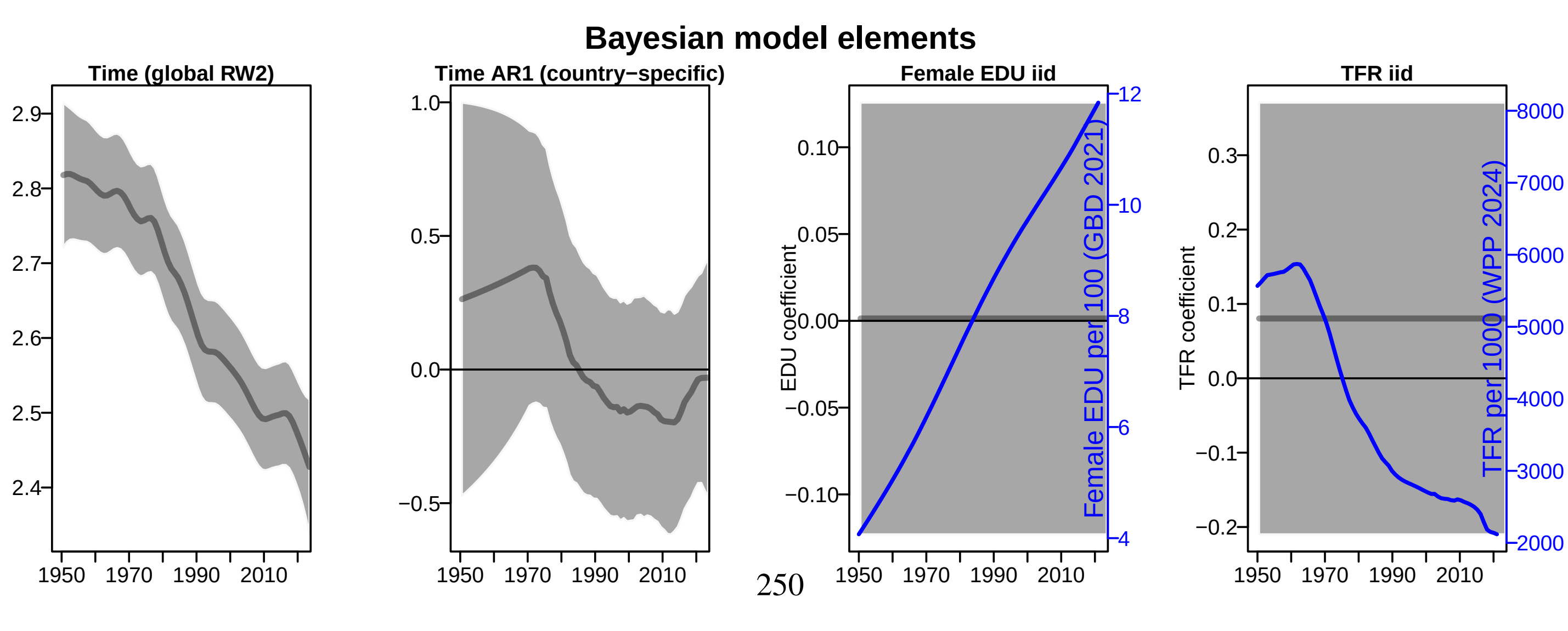
Bayesian model elements
Time (global RW2)
Time AR1 (country−specific)
Female EDU iid
TFR iid
EDU coefficient
Female EDU per 100 (GBD 2021)
TFR coefficient
TFR per 1000 (WPP 2024)
1950
1970
1990
2010

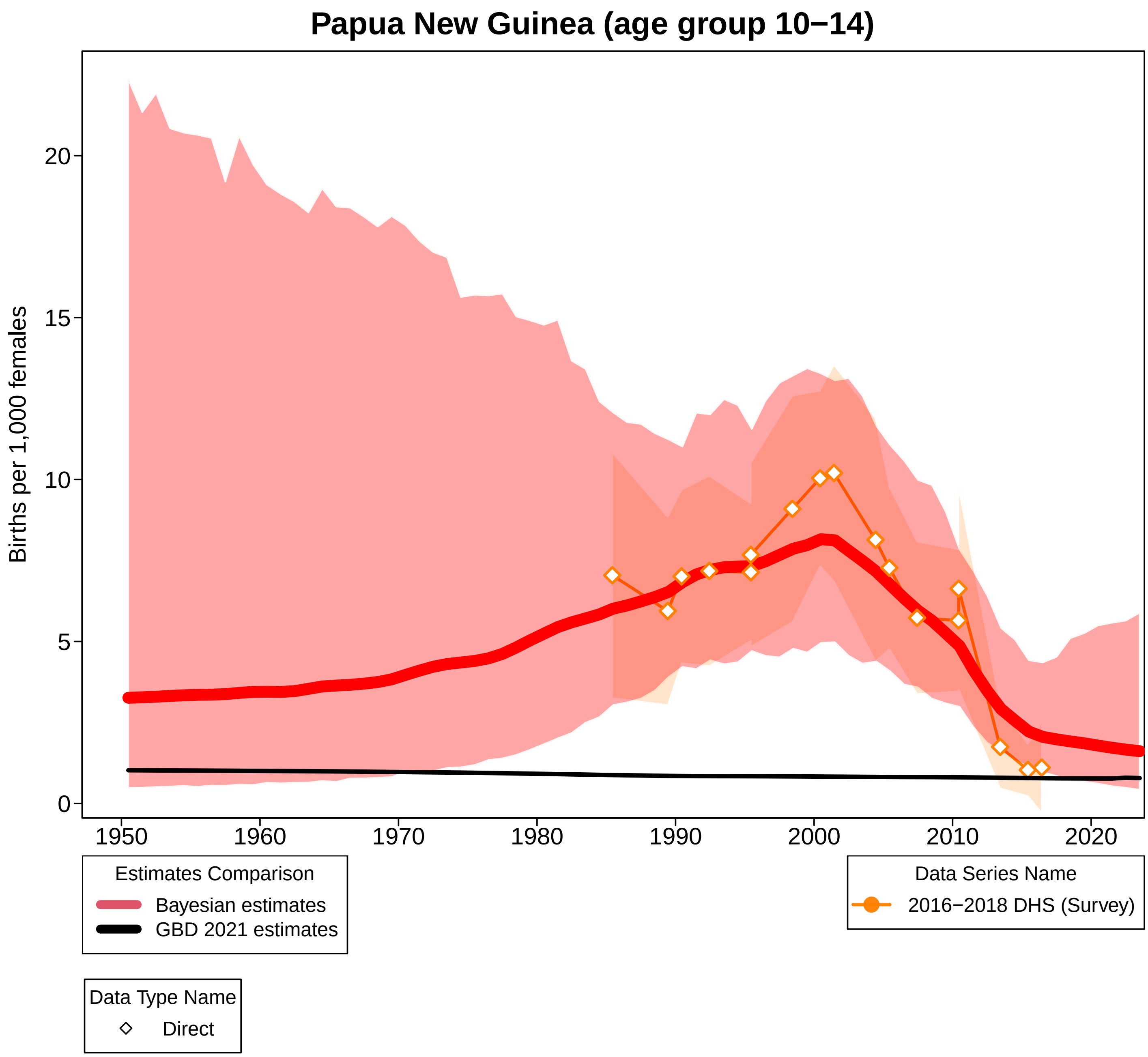
Papua New Guinea (age group 10−14)
Births per 1,000 females
20
15
10
5
0
1950
1960
1970
1980
1990
2000
2010
2020
Estimates Comparison
Bayesian estimates
GBD 2021 estimates
Data Series Name
2016−2018 DHS (Survey)
Data Type Name
Direct

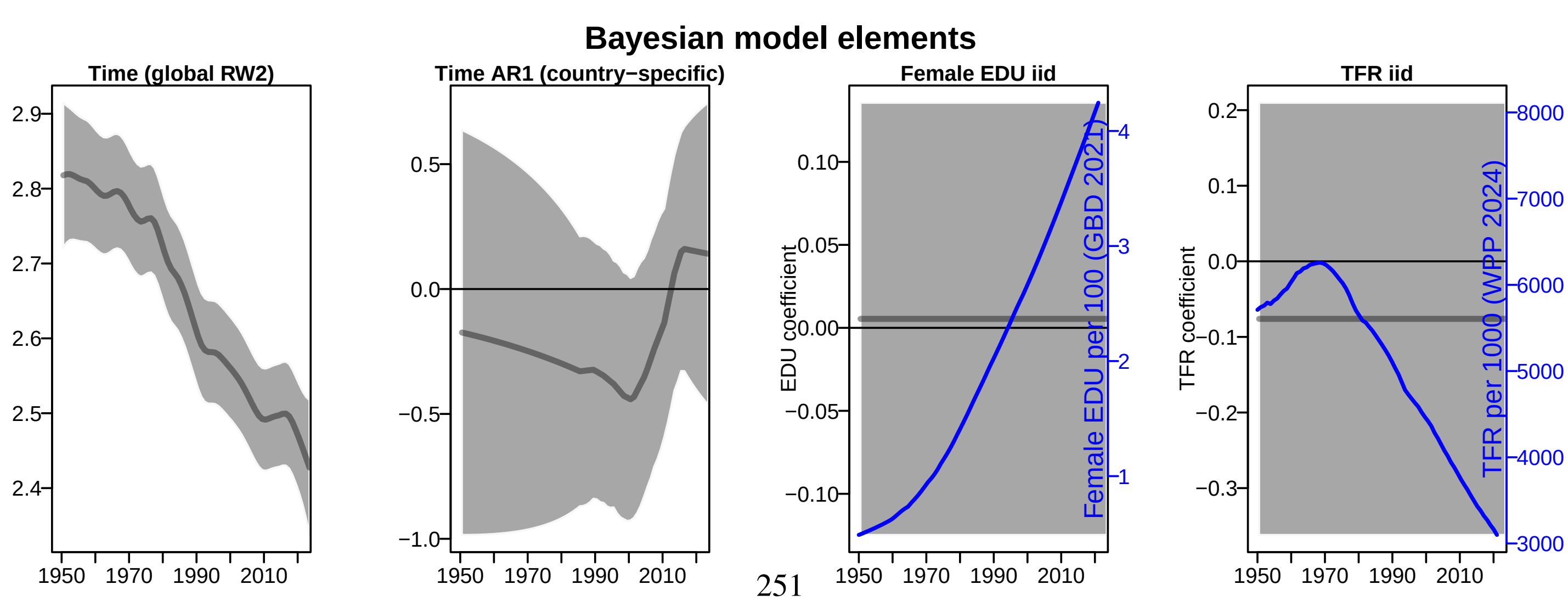
Bayesian model elements
Time (global RW2)
Time AR1 (country−specific)
Female EDU iid
TFR iid
EDU coefficient
Female EDU per 100 (GBD 2021)
TFR coefficient
TFR per 1000 (WPP 2024)
1950
1970
1990
2010

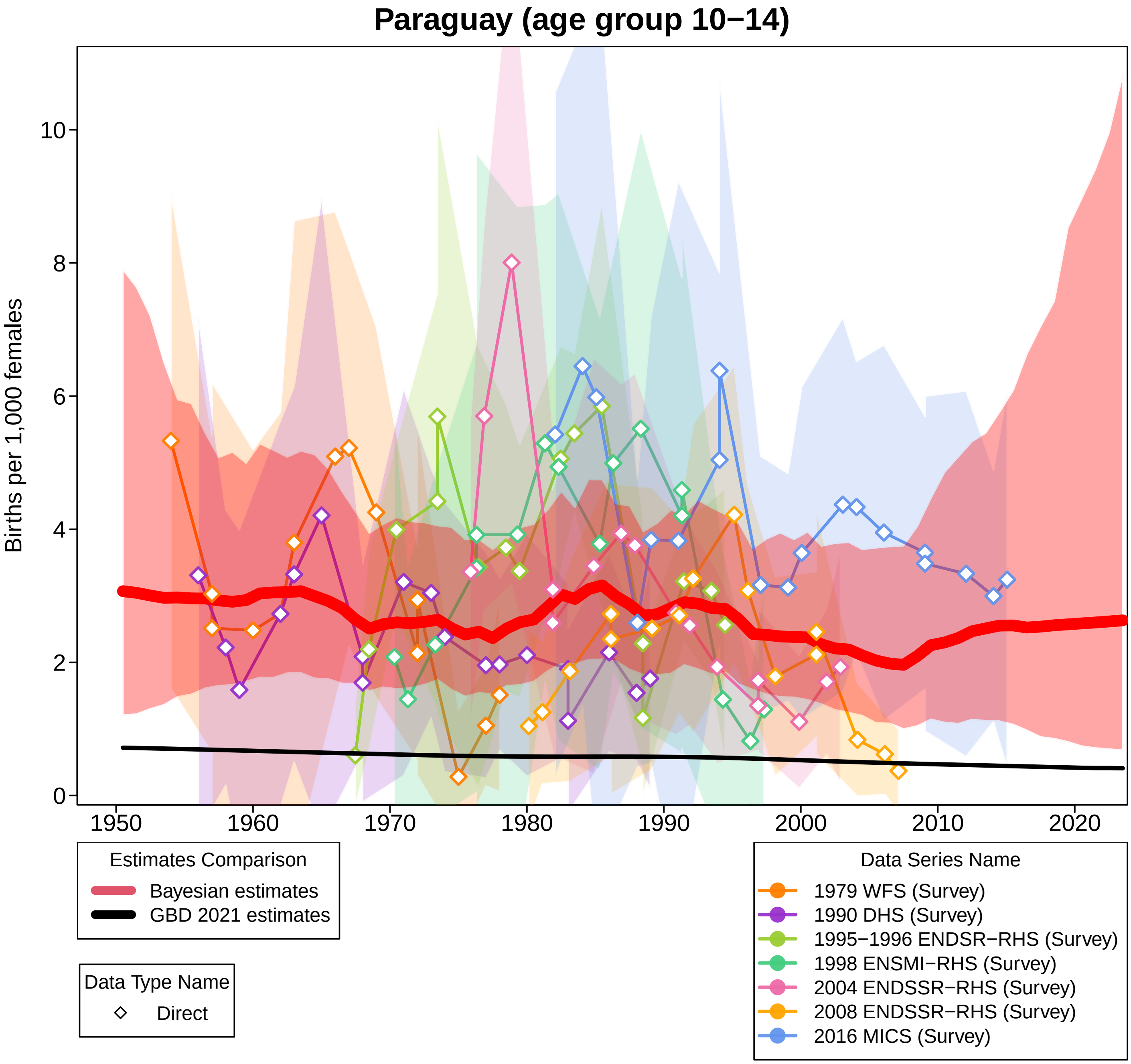

Paraguay (age group 10−14)
Births per 1,000 females
0
2
4
6
8
10
1950
1960
1970
1980
1990
2000
2010
2020
Estimates Comparison
Bayesian estimates
GBD 2021 estimates
Data Type Name
Direct
Data Series Name
1979 WFS (Survey)
1990 DHS (Survey)
1995−1996 ENDSR−RHS (Survey)
1998 ENSMI−RHS (Survey)
2004 ENDSSR−RHS (Survey)
2008 ENDSSR−RHS (Survey)
2016 MICS (Survey)

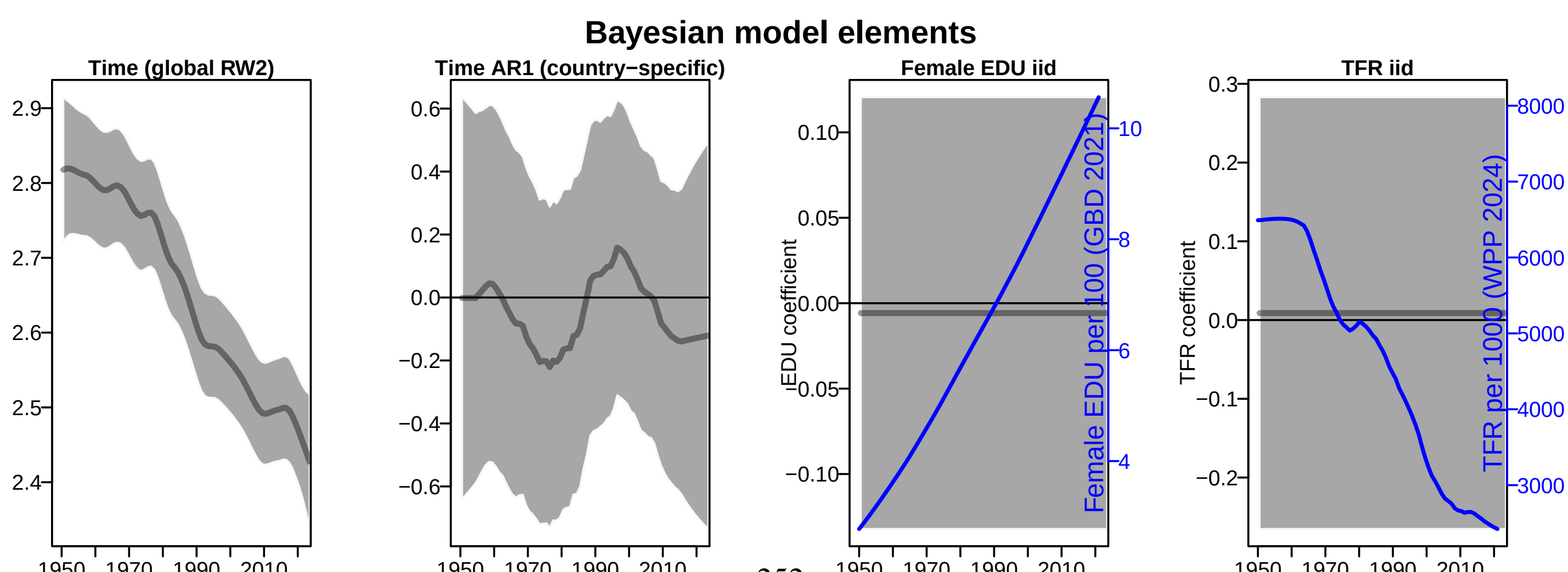

Bayesian model elements
Time (global RW2)
Time AR1 (country−specific)
Female EDU iid
TFR iid
EDU coefficient
Female EDU per 100 (GBD 2021)
TFR coefficient
TFR per 1000 (WPP 2024)

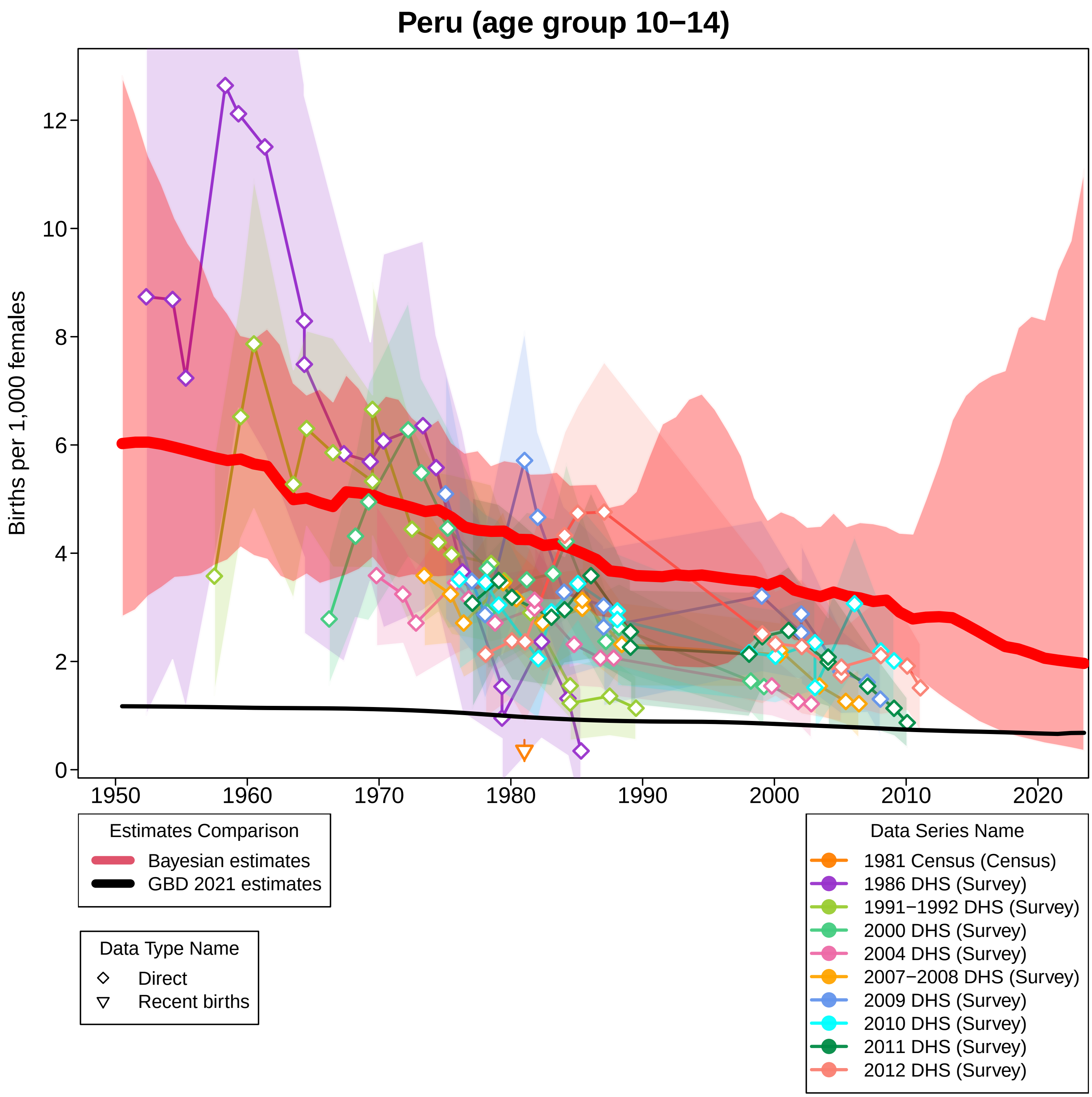

Peru (age group 10−14)
Births per 1,000 females
0
2
4
6
8
10
12
1950
1960
1970
1980
1990
2000
2010
2020
Estimates Comparison
Bayesian estimates
GBD 2021 estimates
Data Type Name
Direct
Recent births
Data Series Name
1981 Census (Census)
1986 DHS (Survey)
1991−1992 DHS (Survey)
2000 DHS (Survey)
2004 DHS (Survey)
2007−2008 DHS (Survey)
2009 DHS (Survey)
2010 DHS (Survey)
2011 DHS (Survey)
2012 DHS (Survey)

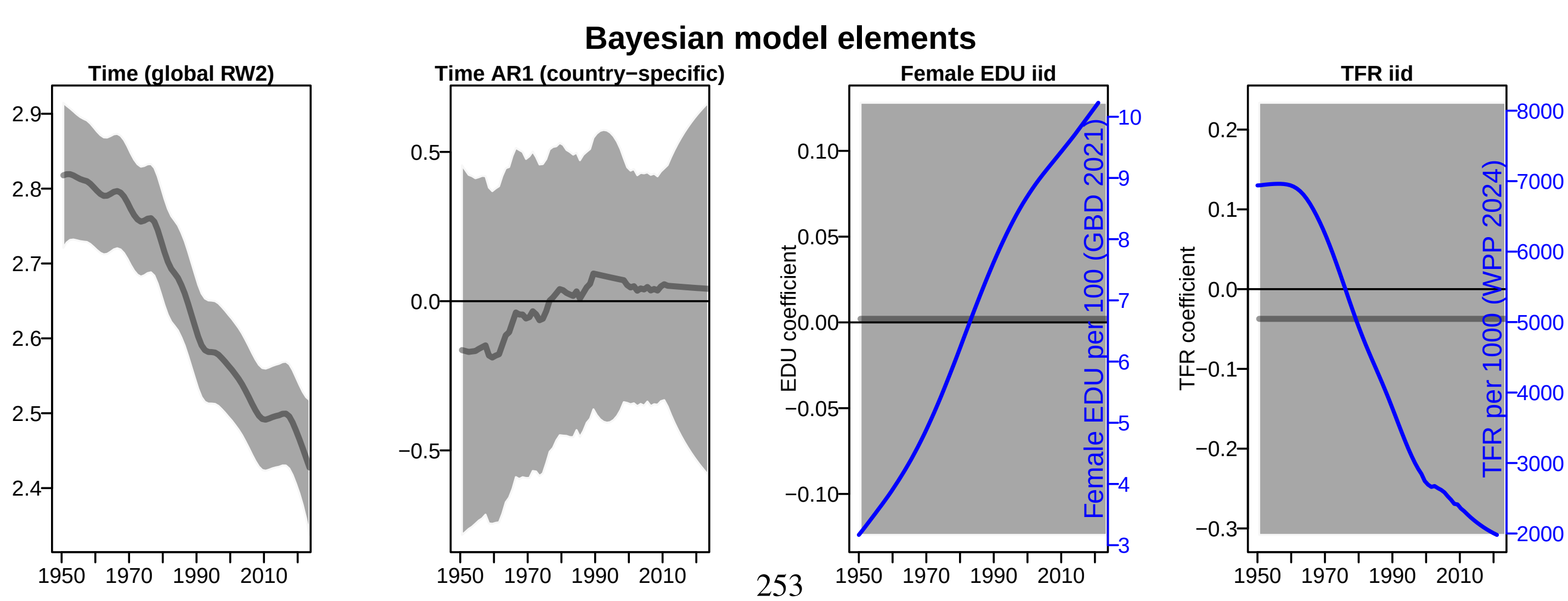

Bayesian model elements
Time (global RW2)
Time AR1 (country−specific)
Female EDU iid
TFR iid
EDU coefficient
Female EDU per 100 (GBD 2021)
TFR coefficient
TFR per 1000 (WPP 2024)
1950
1970
1990
2010

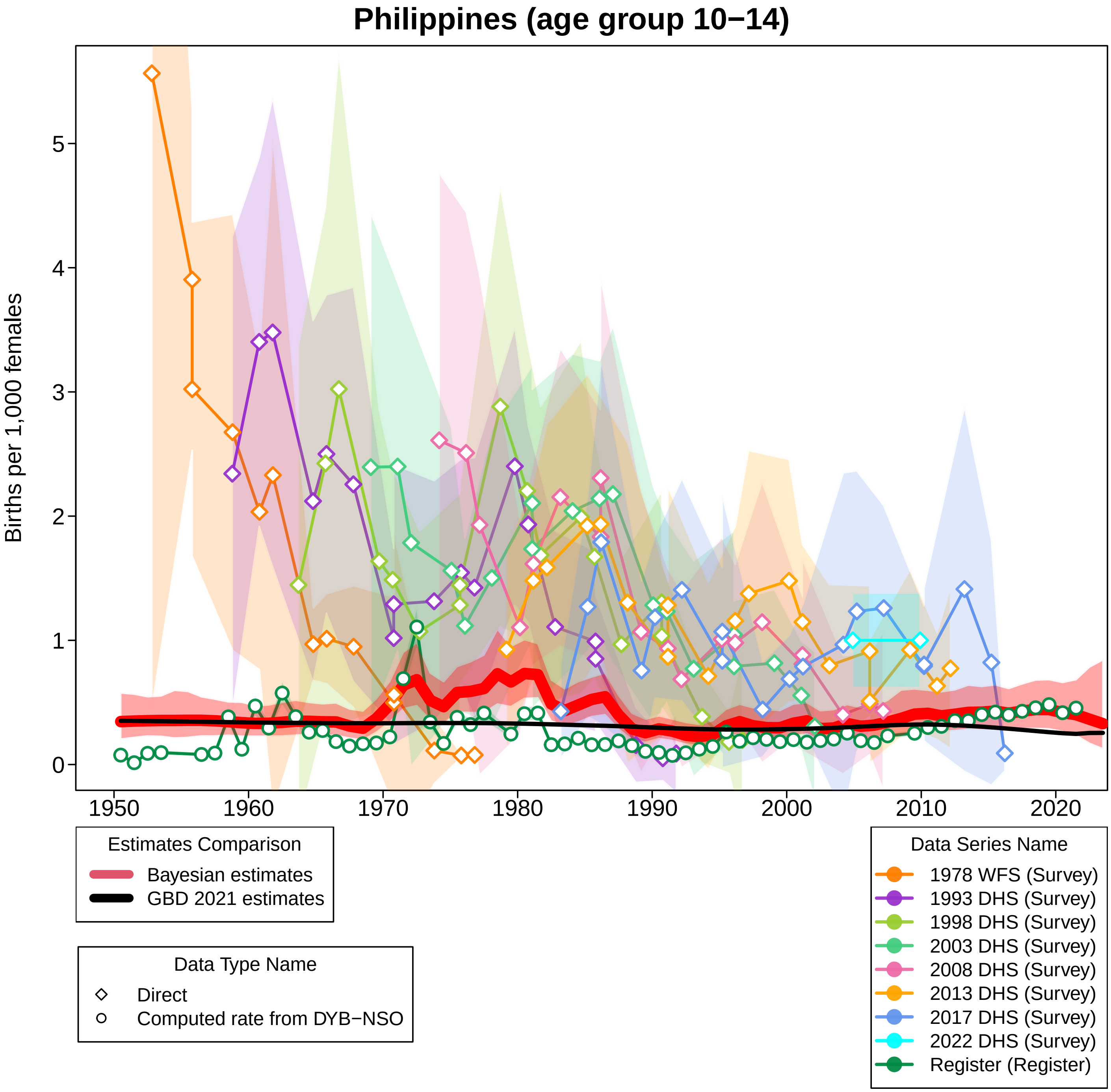
Philippines (age group 10−14)
Births per 1,000 females
0
1
2
3
4
5
1950
1960
1970
1980
1990
2000
2010
2020
Estimates Comparison
Bayesian estimates
GBD 2021 estimates
Data Type Name
Direct
Computed rate from DYB−NSO
Data Series Name
1978 WFS (Survey)
1993 DHS (Survey)
1998 DHS (Survey)
2003 DHS (Survey)
2008 DHS (Survey)
2013 DHS (Survey)
2017 DHS (Survey)
2022 DHS (Survey)
Register (Register)

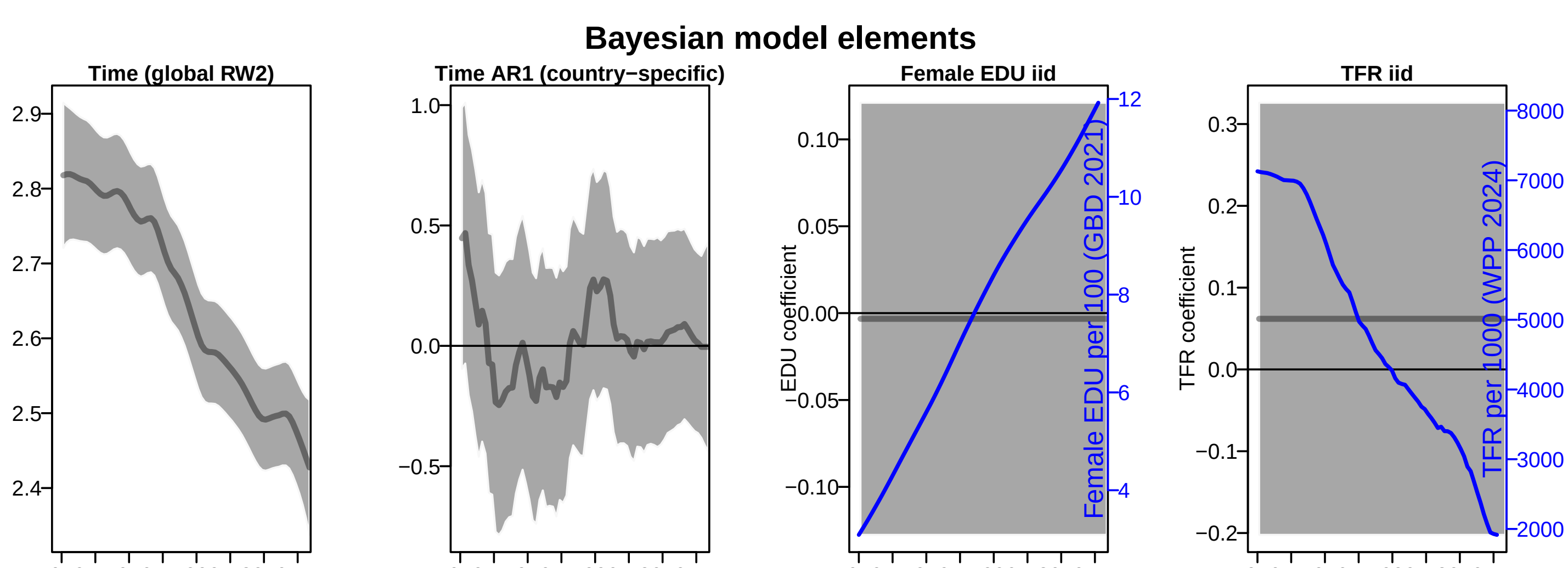
Bayesian model elements
Time (global RW2)
Time AR1 (country−specific)
Female EDU iid
TFR iid
EDU coefficient
Female EDU per 100 (GBD 2021)
TFR coefficient
TFR per 1000 (WPP 2024)

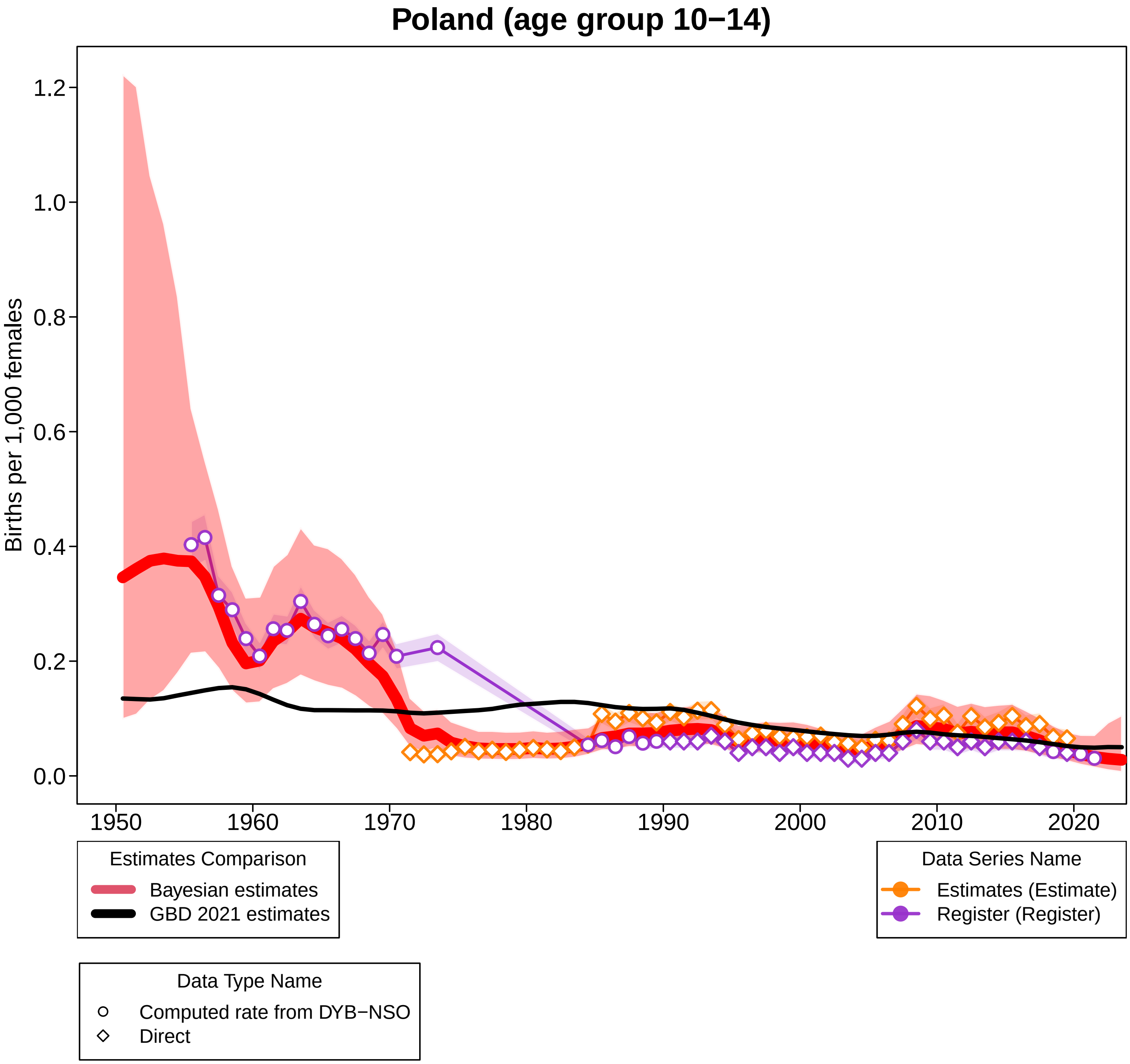

Poland (age group 10−14)
Births per 1,000 females
1.2
1.0
0.8
0.6
0.4
0.2
0.0
1950
1960
1970
1980
1990
2000
2010
2020
Estimates Comparison
Bayesian estimates
GBD 2021 estimates
Data Series Name
Estimates (Estimate)
Register (Register)
Data Type Name
Computed rate from DYB−NSO
Direct

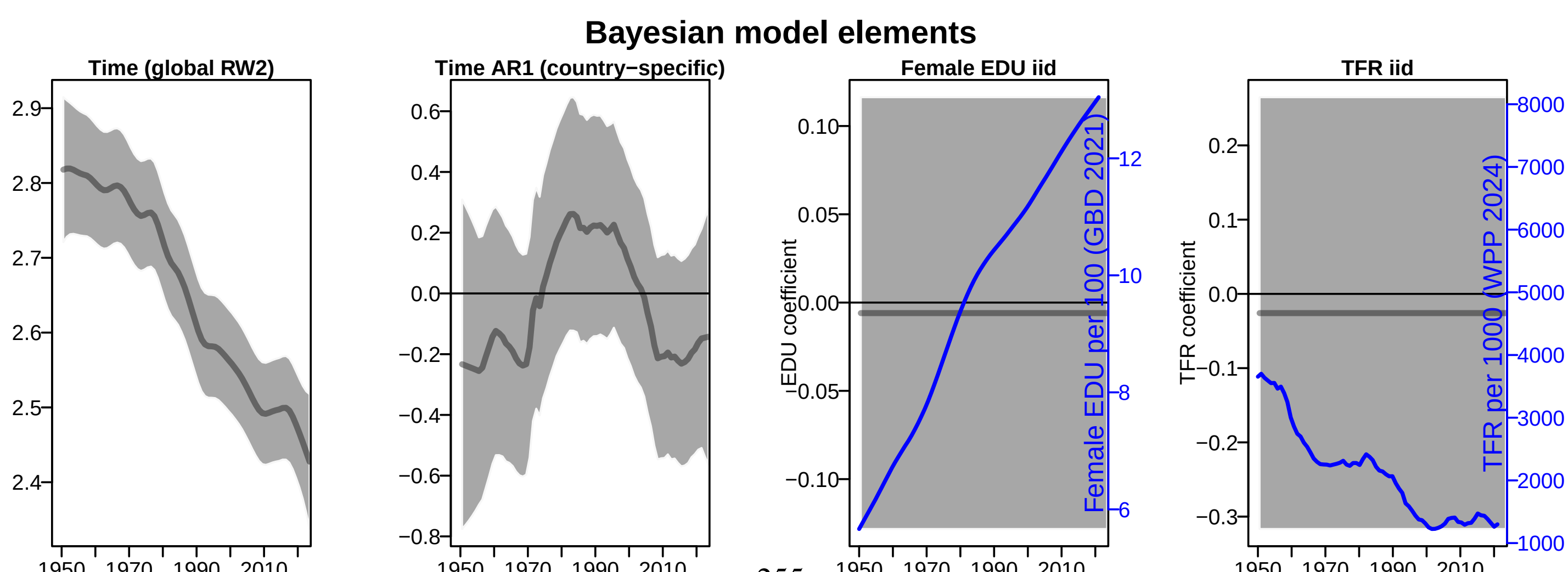

Bayesian model elements
Time (global RW2)
Time AR1 (country−specific)
Female EDU iid
TFR iid
EDU coefficient
Female EDU per 100 (GBD 2021)
TFR coefficient
TFR per 1000 (WPP 2024)

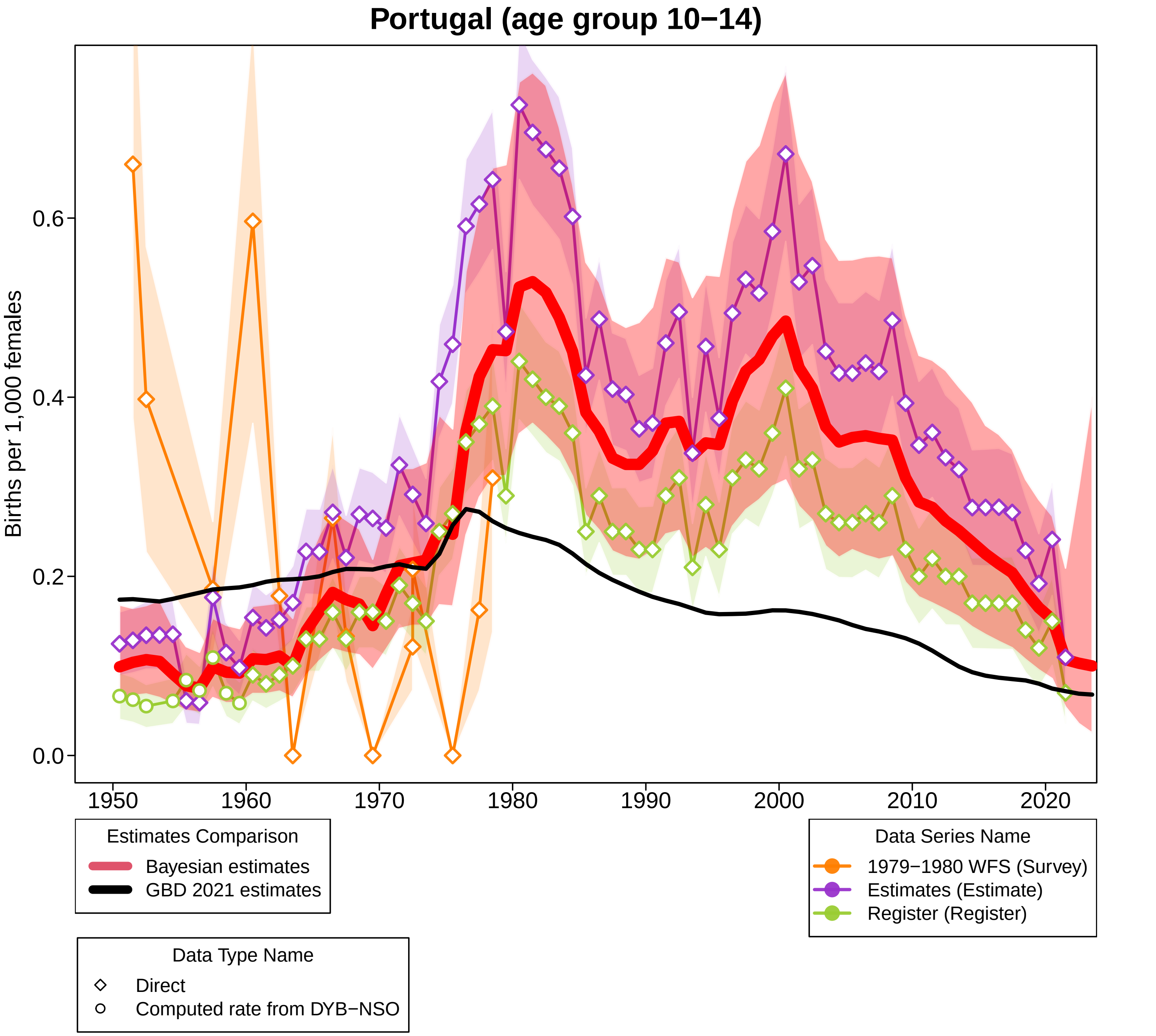

Portugal (age group 10−14)
Births per 1,000 females
0.0
0.2
0.4
0.6
1950
1960
1970
1980
1990
2000
2010
2020
Estimates Comparison
Bayesian estimates
GBD 2021 estimates
Data Series Name
1979−1980 WFS (Survey)
Estimates (Estimate)
Register (Register)
Data Type Name
Direct
Computed rate from DYB−NSO

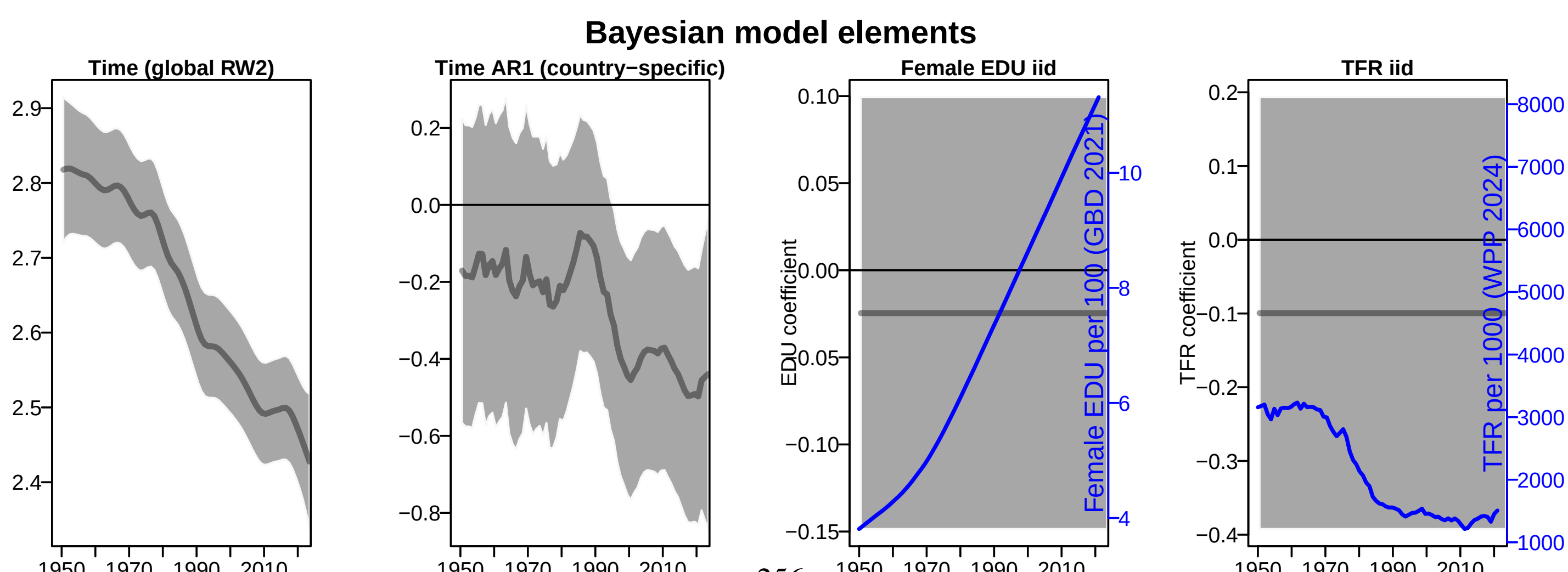

Bayesian model elements
Time (global RW2)
Time AR1 (country−specific)
Female EDU iid
TFR iid
EDU coefficient
Female EDU per 100 (GBD 2021)
TFR coefficient
TFR per 1000 (WPP 2024)

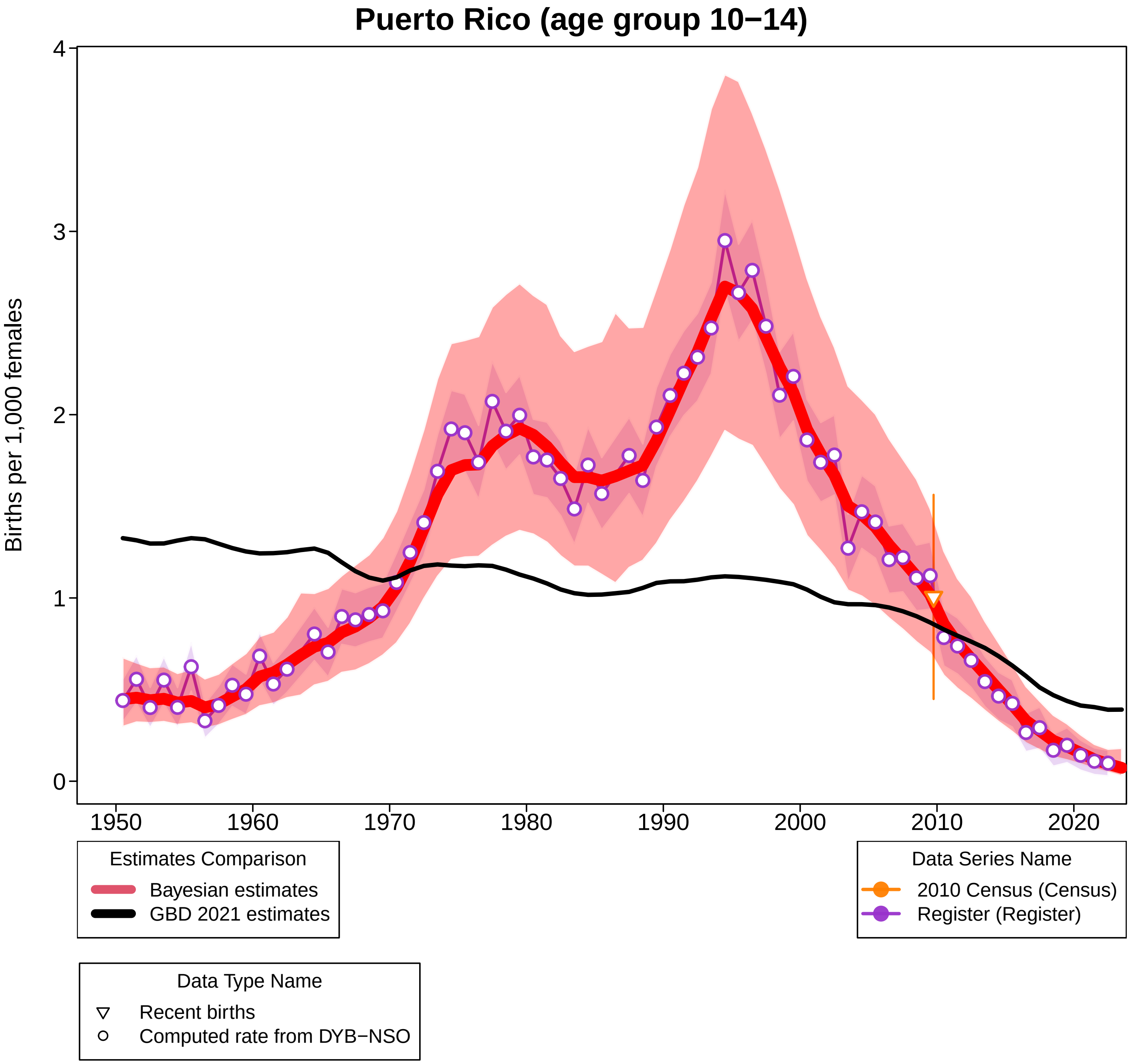
Puerto Rico (age group 10−14)
Births per 1,000 females
0
1
2
3
4
1950
1960
1970
1980
1990
2000
2010
2020
Estimates Comparison
Bayesian estimates
GBD 2021 estimates
Data Series Name
2010 Census (Census)
Register (Register)
Data Type Name
Recent births
Computed rate from DYB−NSO

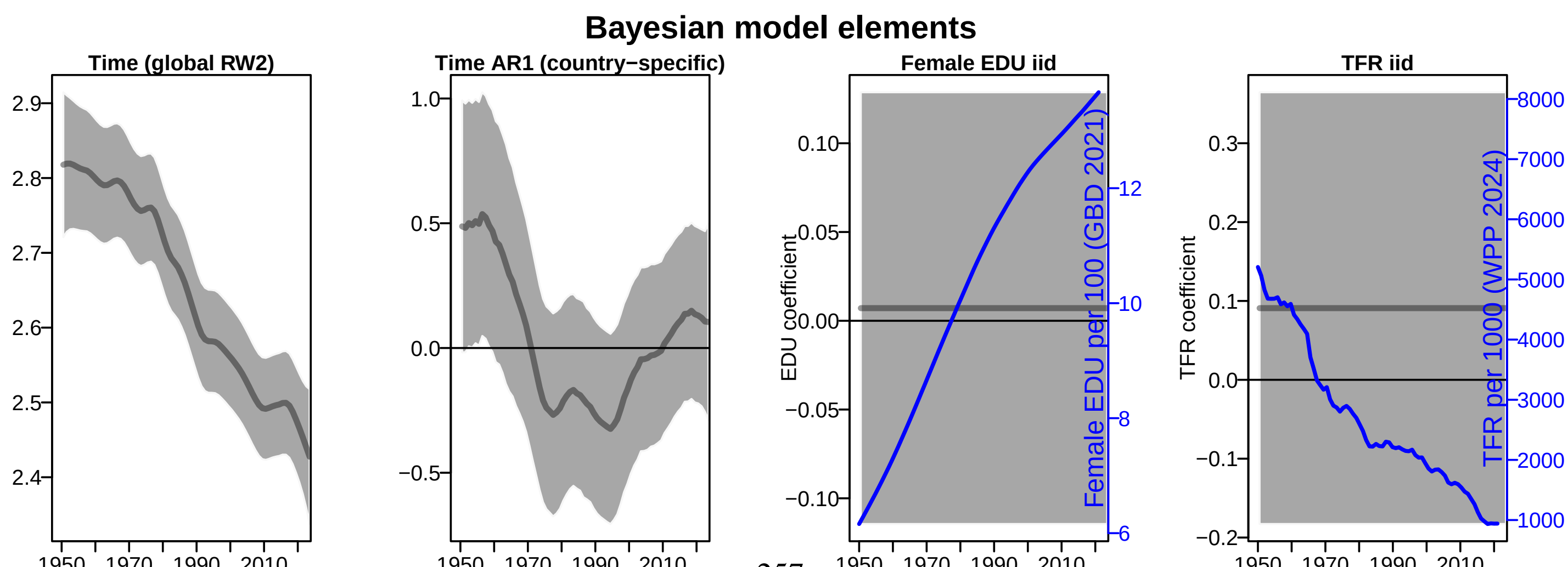
Bayesian model elements
Time (global RW2)
Time AR1 (country−specific)
Female EDU iid
TFR iid
EDU coefficient
Female EDU per 100 (GBD 2021)
TFR coefficient
TFR per 1000 (WPP 2024)
1950
1970
1990
2010

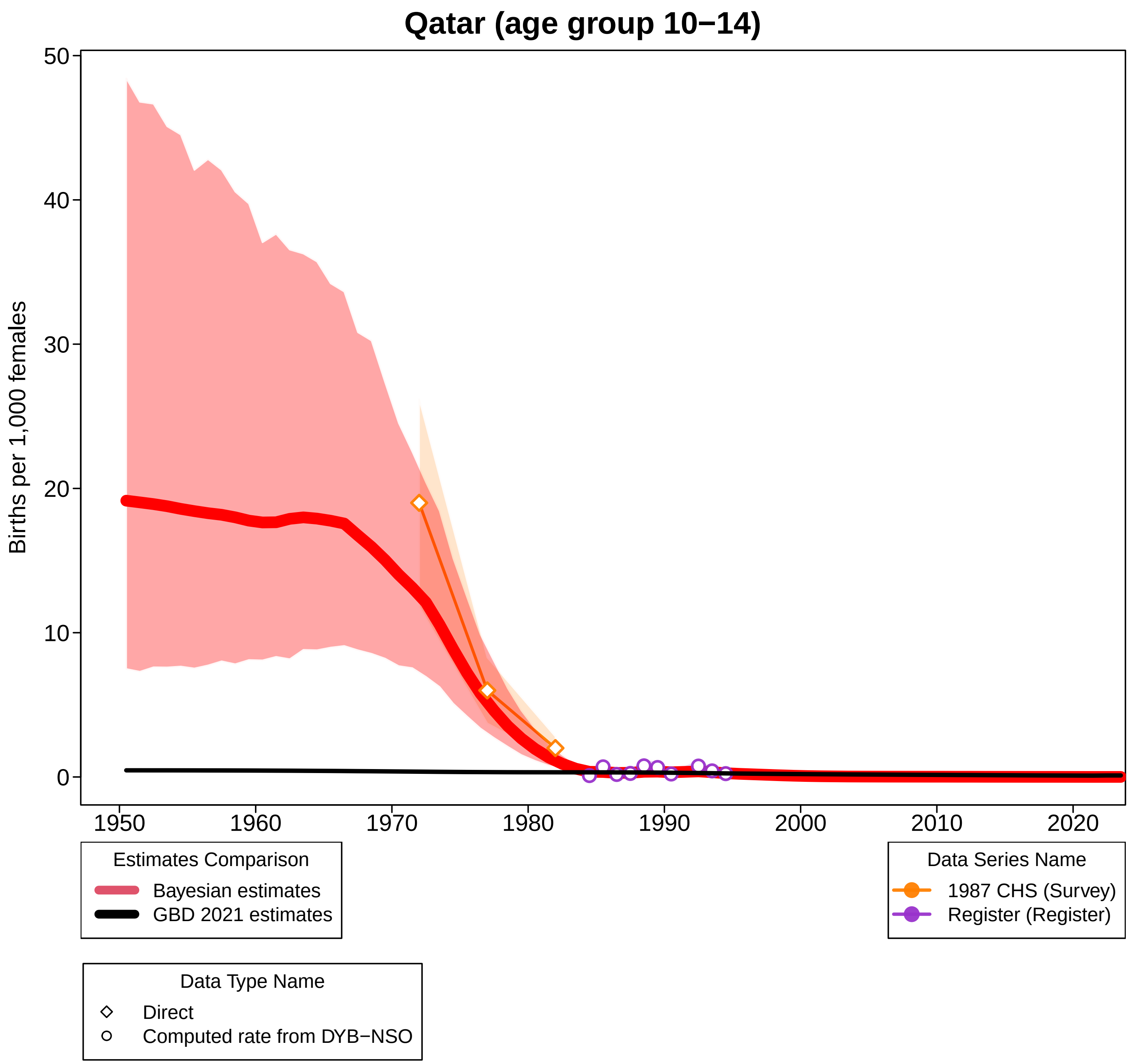
Qatar (age group 10−14)
Births per 1,000 females
0
10
20
30
40
50
1950
1960
1970
1980
1990
2000
2010
2020
Estimates Comparison
Bayesian estimates
GBD 2021 estimates
Data Series Name
1987 CHS (Survey)
Register (Register)
Data Type Name
Direct
Computed rate from DYB−NSO

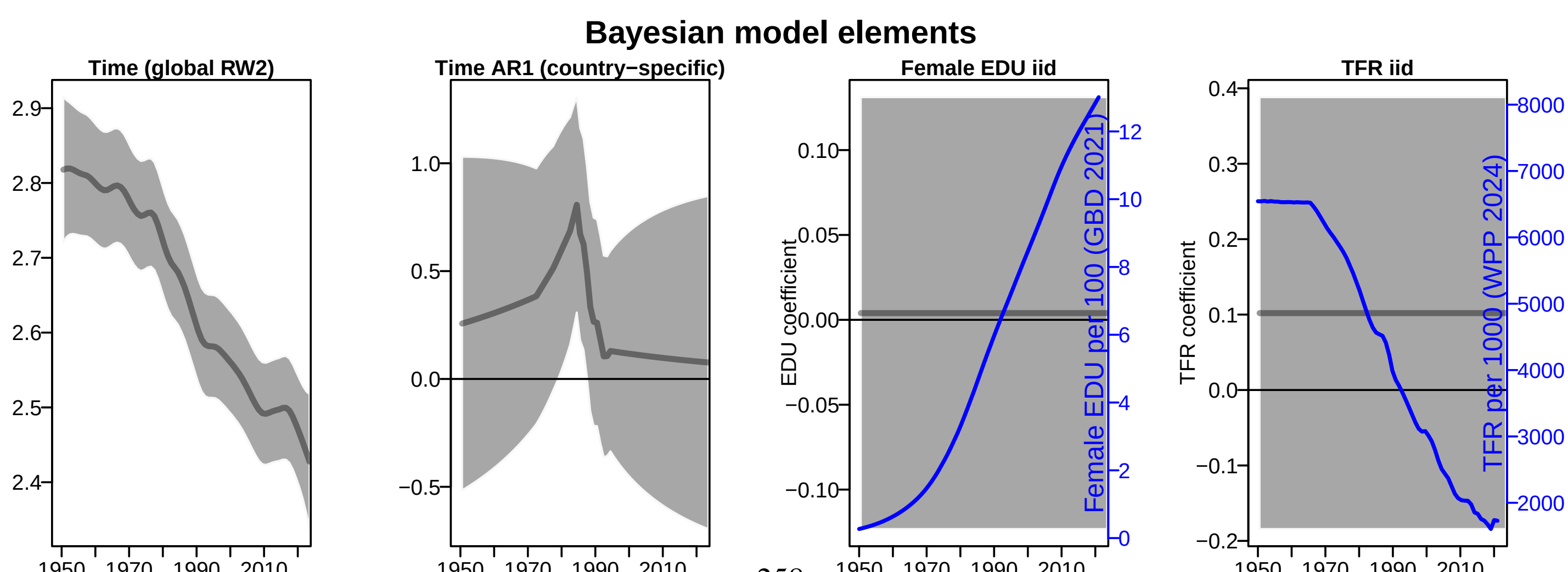
Bayesian model elements
Time (global RW2)
Time AR1 (country−specific)
Female EDU iid
TFR iid
EDU coefficient
Female EDU per 100 (GBD 2021)
TFR coefficient
TFR per 1000 (WPP 2024)
1950
1970
1990
2010

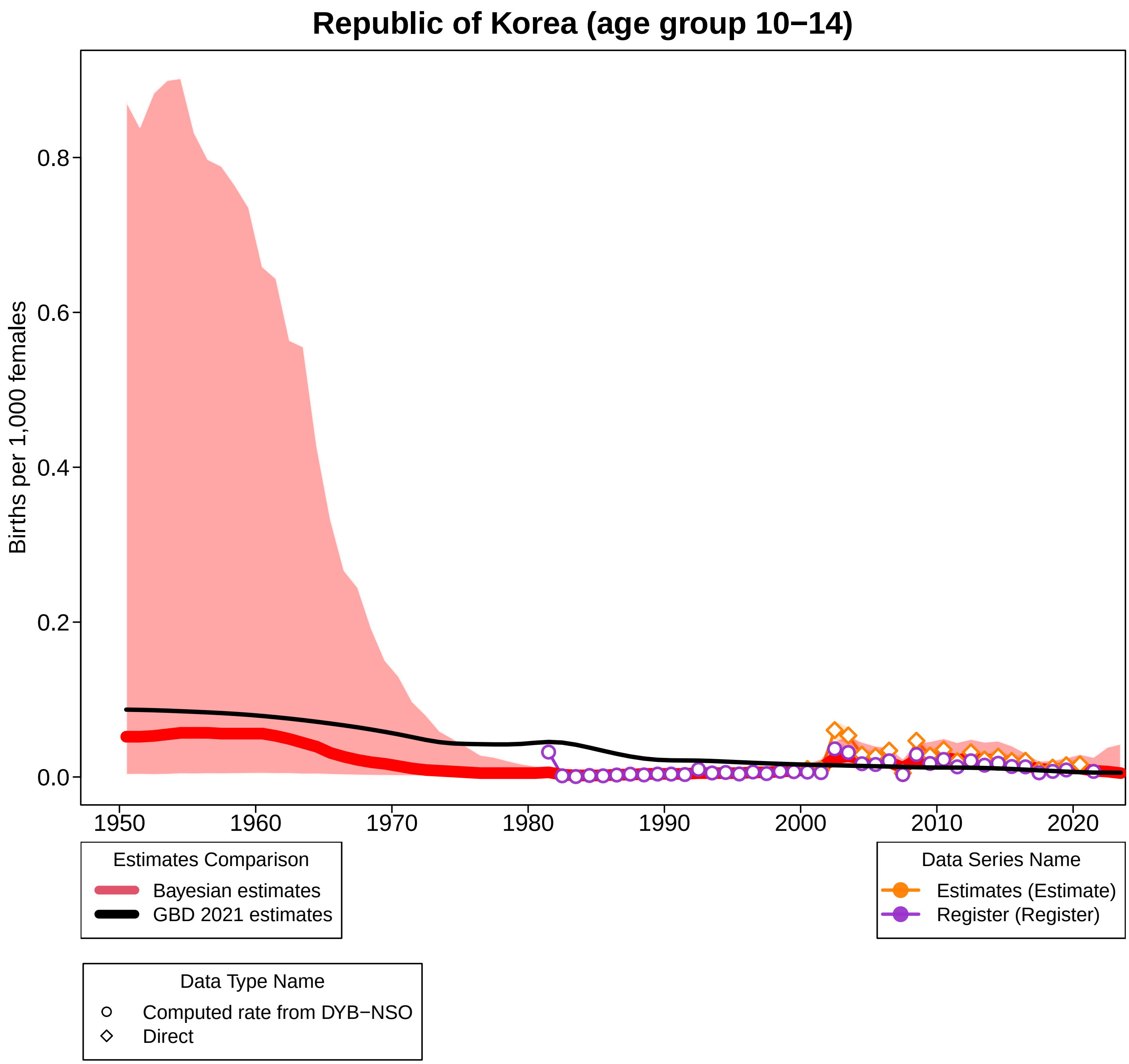
Republic of Korea (age group 10−14)
Births per 1,000 females
0.0
0.2
0.4
0.6
0.8
1950
1960
1970
1980
1990
2000
2010
2020
Estimates Comparison
Bayesian estimates
GBD 2021 estimates
Data Series Name
Estimates (Estimate)
Register (Register)
Data Type Name
Computed rate from DYB−NSO
Direct

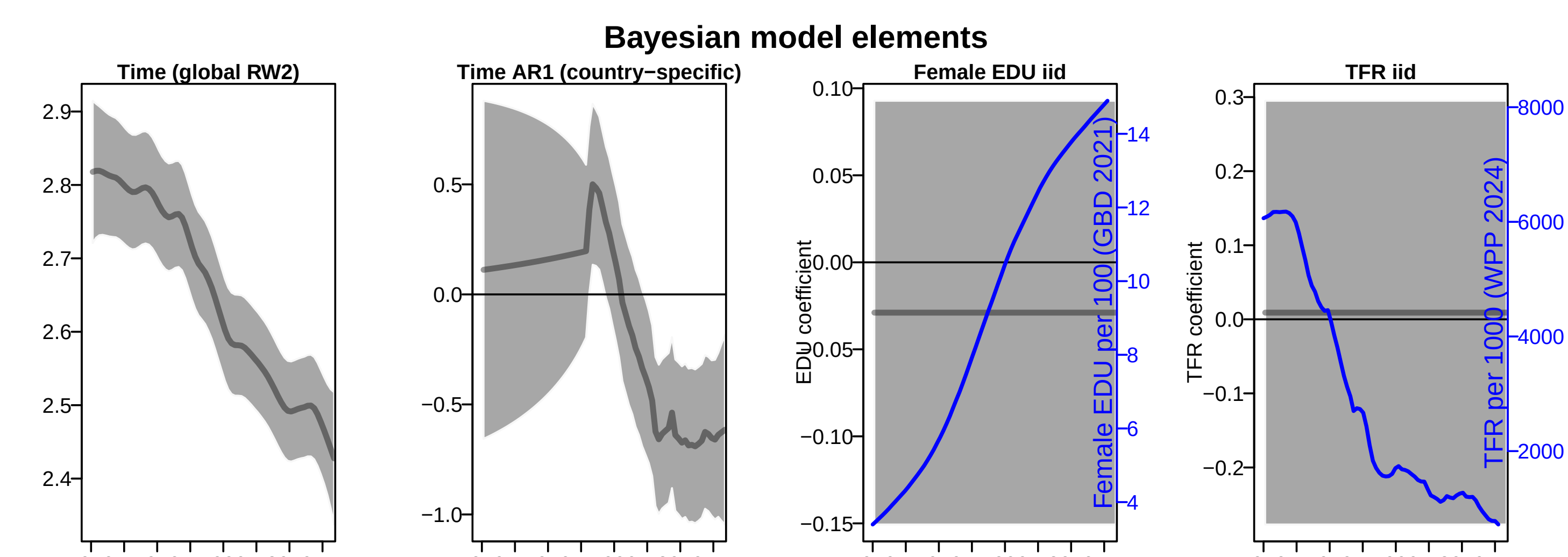
Bayesian model elements
Time (global RW2)
Time AR1 (country−specific)
Female EDU iid
TFR iid
EDU coefficient
Female EDU per 100 (GBD 2021)
TFR coefficient
TFR per 1000 (WPP 2024)
1950
1970
1990
2010

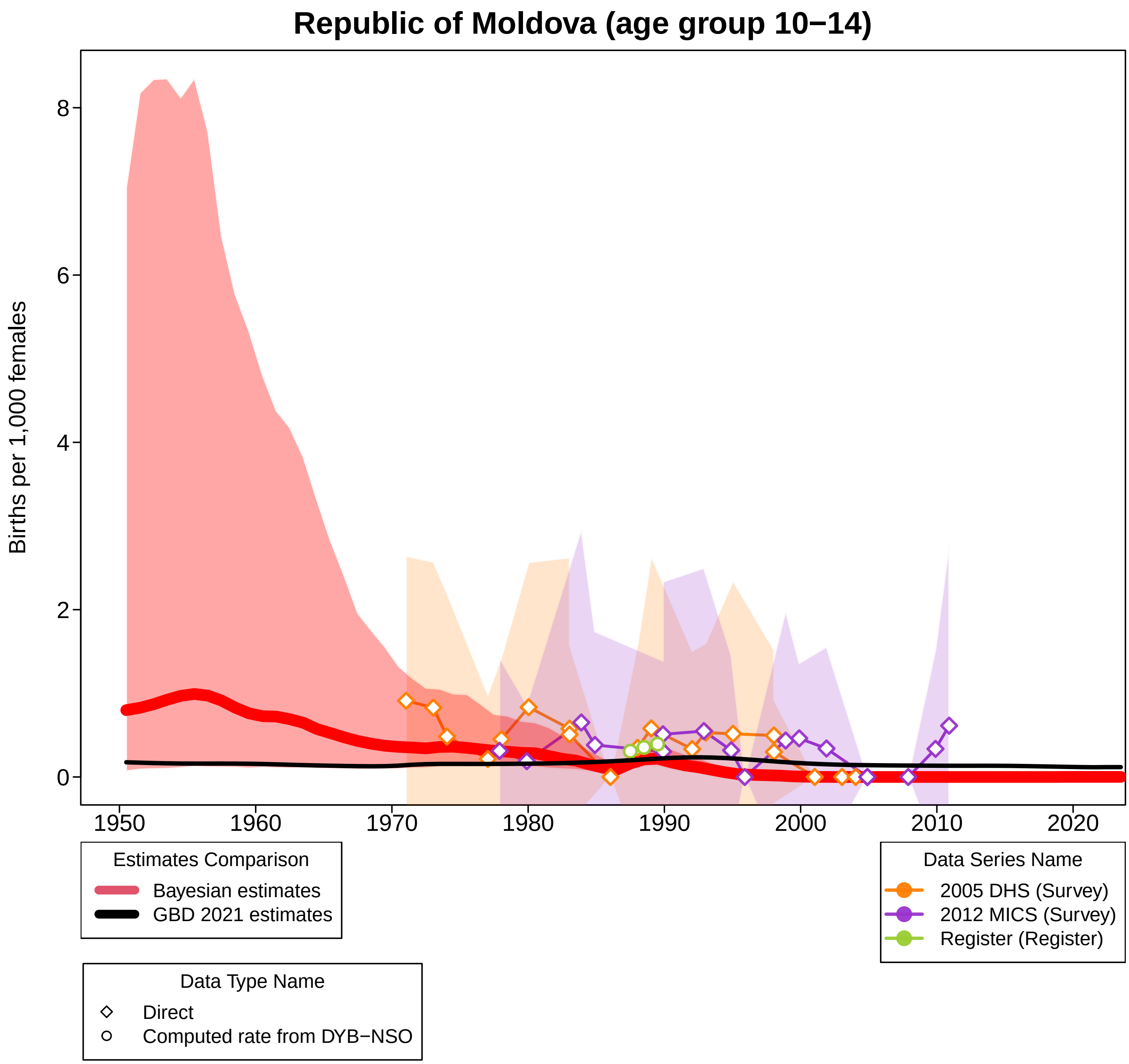
Republic of Moldova (age group 10−14)
Births per 1,000 females
0
2
4
6
8
1950
1960
1970
1980
1990
2000
2010
2020
Estimates Comparison
Bayesian estimates
GBD 2021 estimates
Data Series Name
2005 DHS (Survey)
2012 MICS (Survey)
Register (Register)
Data Type Name
Direct
Computed rate from DYB−NSO

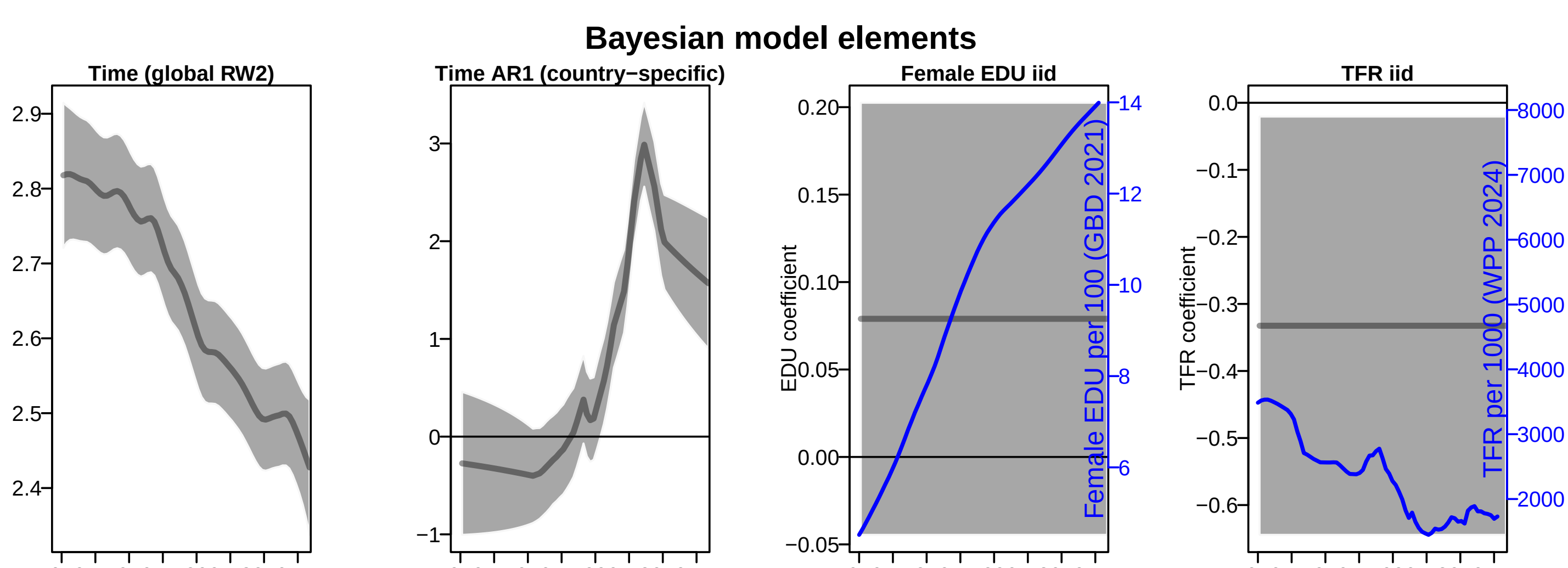
Bayesian model elements
Time (global RW2)
Time AR1 (country−specific)
Female EDU iid
TFR iid
EDU coefficient
Female EDU per 100 (GBD 2021)
TFR coefficient
TFR per 1000 (WPP 2024)
1950
1970
1990
2010

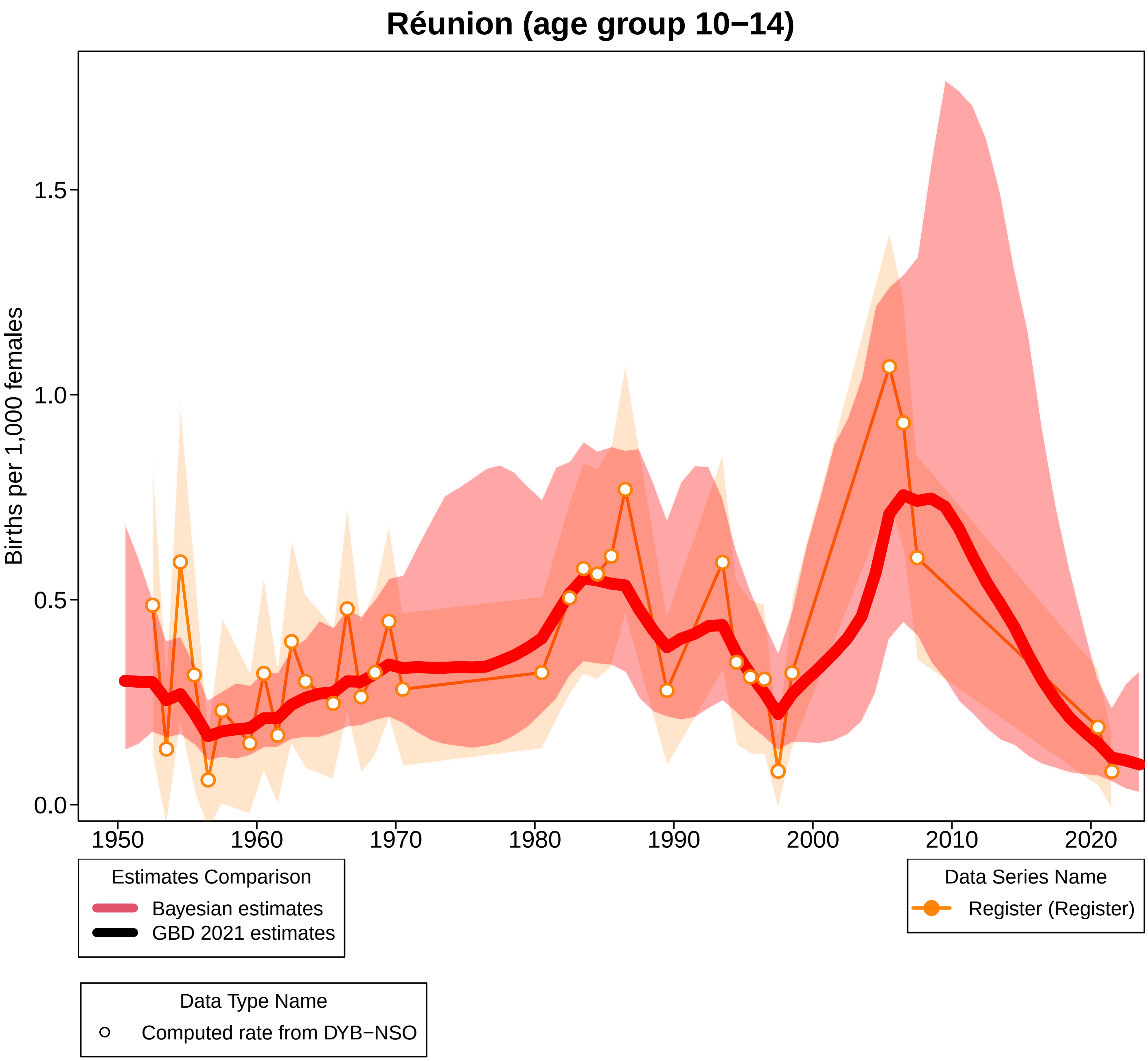

Réunion (age group 10−14)
Births per 1,000 females
1.5
1.0
0.5
0.0
1950
1960
1970
1980
1990
2000
2010
2020
Estimates Comparison
Bayesian estimates
GBD 2021 estimates
Data Series Name
Register (Register)
Data Type Name
Computed rate from DYB−NSO

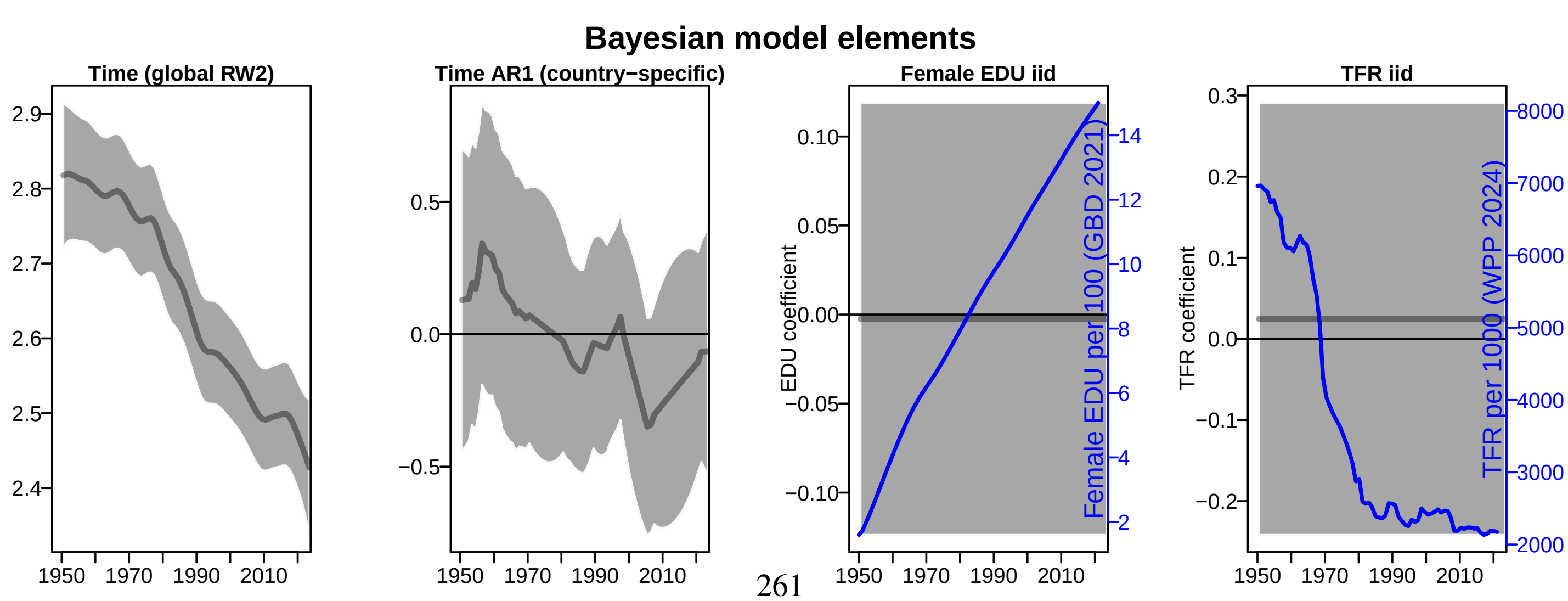

Bayesian model elements
Time (global RW2)
Time AR1 (country−specific)
Female EDU iid
TFR iid
EDU coefficient
Female EDU per 100 (GBD 2021)
TFR coefficient
TFR per 1000 (WPP 2024)
1950
1970
1990
2010

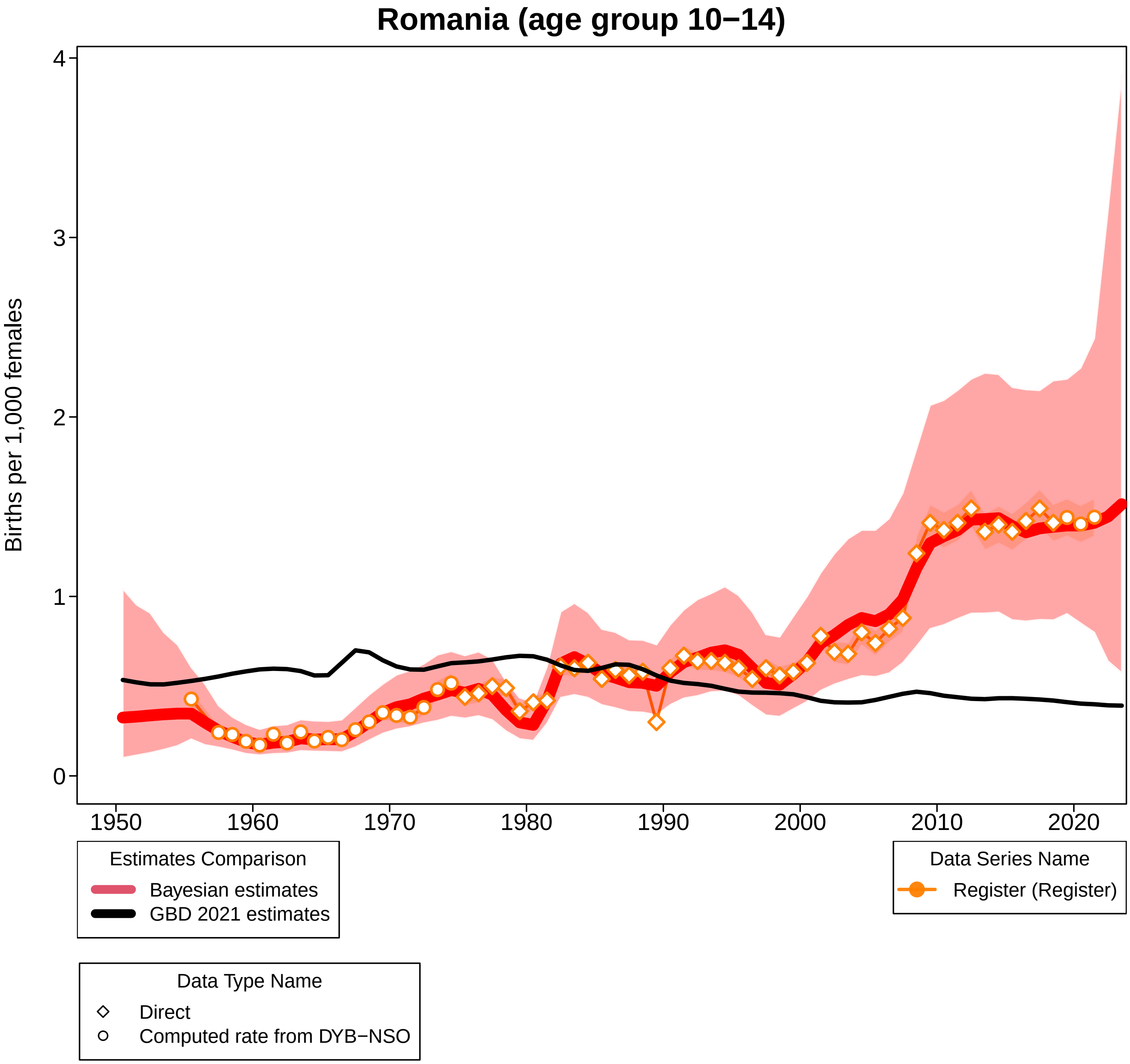
Romania (age group 10−14)
Births per 1,000 females
0
1
2
3
4
1950
1960
1970
1980
1990
2000
2010
2020
Estimates Comparison
Bayesian estimates
GBD 2021 estimates
Data Series Name
Register (Register)
Data Type Name
Direct
Computed rate from DYB−NSO

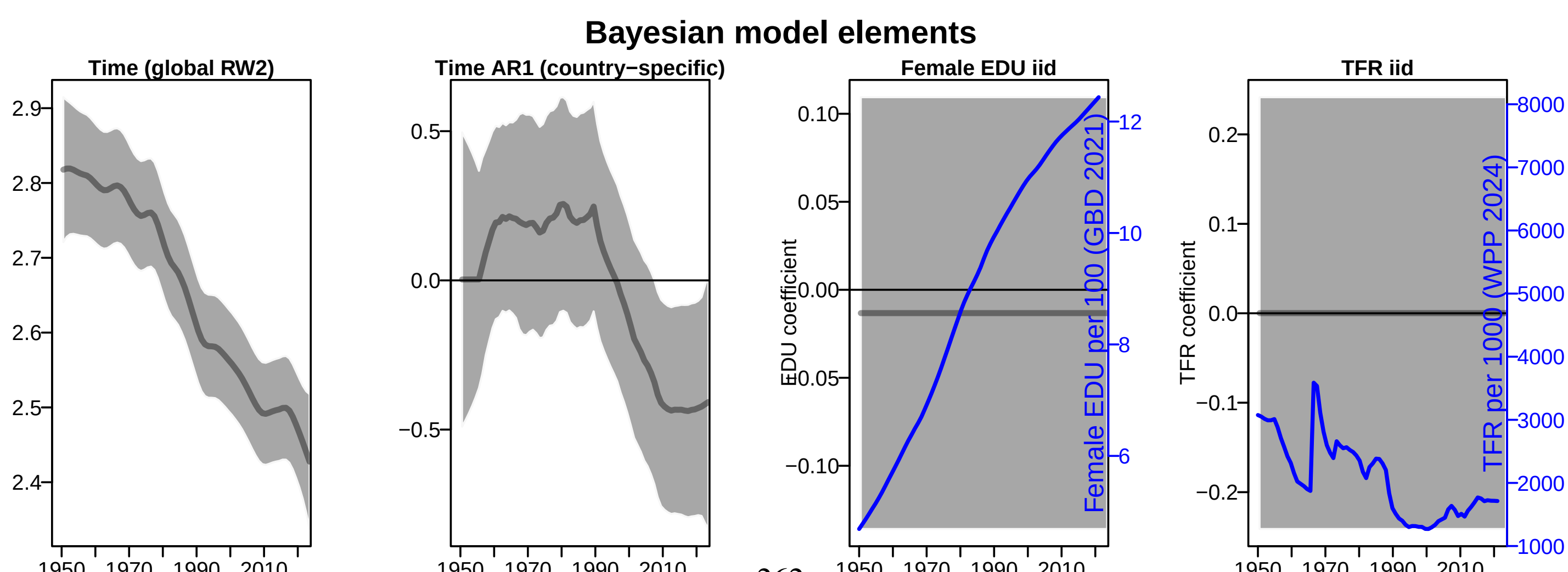
Bayesian model elements
Time (global RW2)
Time AR1 (country−specific)
Female EDU iid
TFR iid
EDU coefficient
Female EDU per 100 (GBD 2021)
TFR coefficient
TFR per 1000 (WPP 2024)
1950
1970
1990
2010

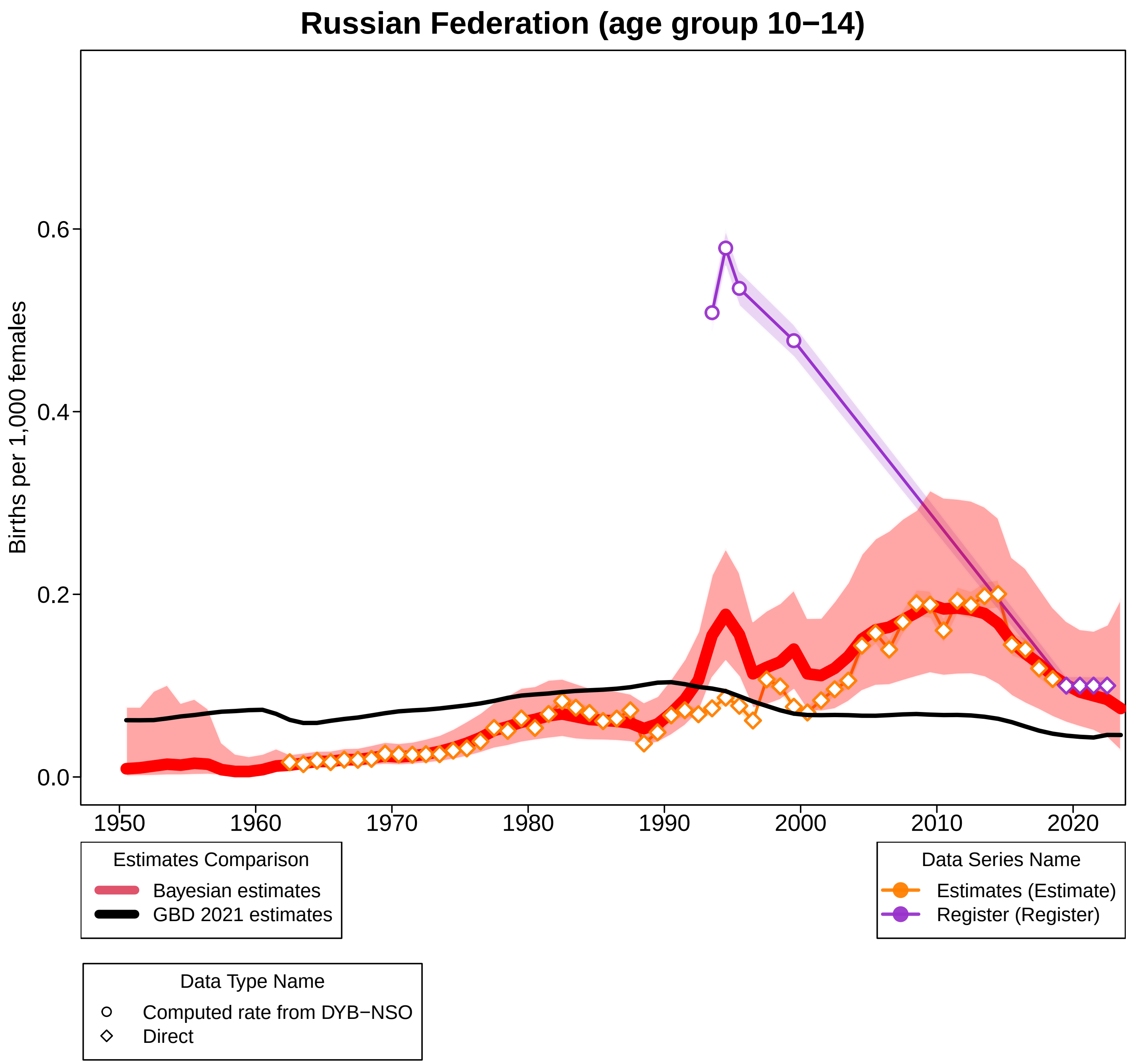

Russian Federation (age group 10−14)
Births per 1,000 females
0.0
0.2
0.4
0.6
1950
1960
1970
1980
1990
2000
2010
2020
Estimates Comparison
Bayesian estimates
GBD 2021 estimates
Data Series Name
Estimates (Estimate)
Register (Register)
Data Type Name
Computed rate from DYB−NSO
Direct

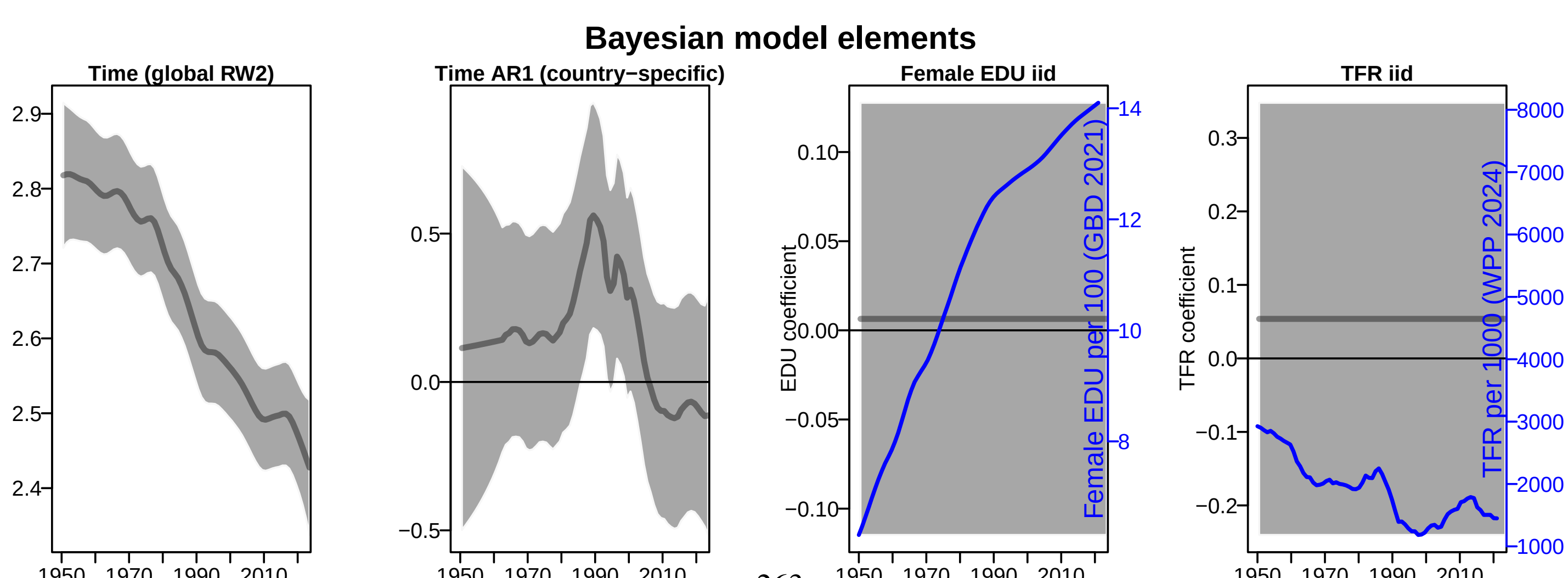

Bayesian model elements
Time (global RW2)
Time AR1 (country−specific)
Female EDU iid
TFR iid
EDU coefficient
Female EDU per 100 (GBD 2021)
TFR coefficient
TFR per 1000 (WPP 2024)
1950
1970
1990
2010

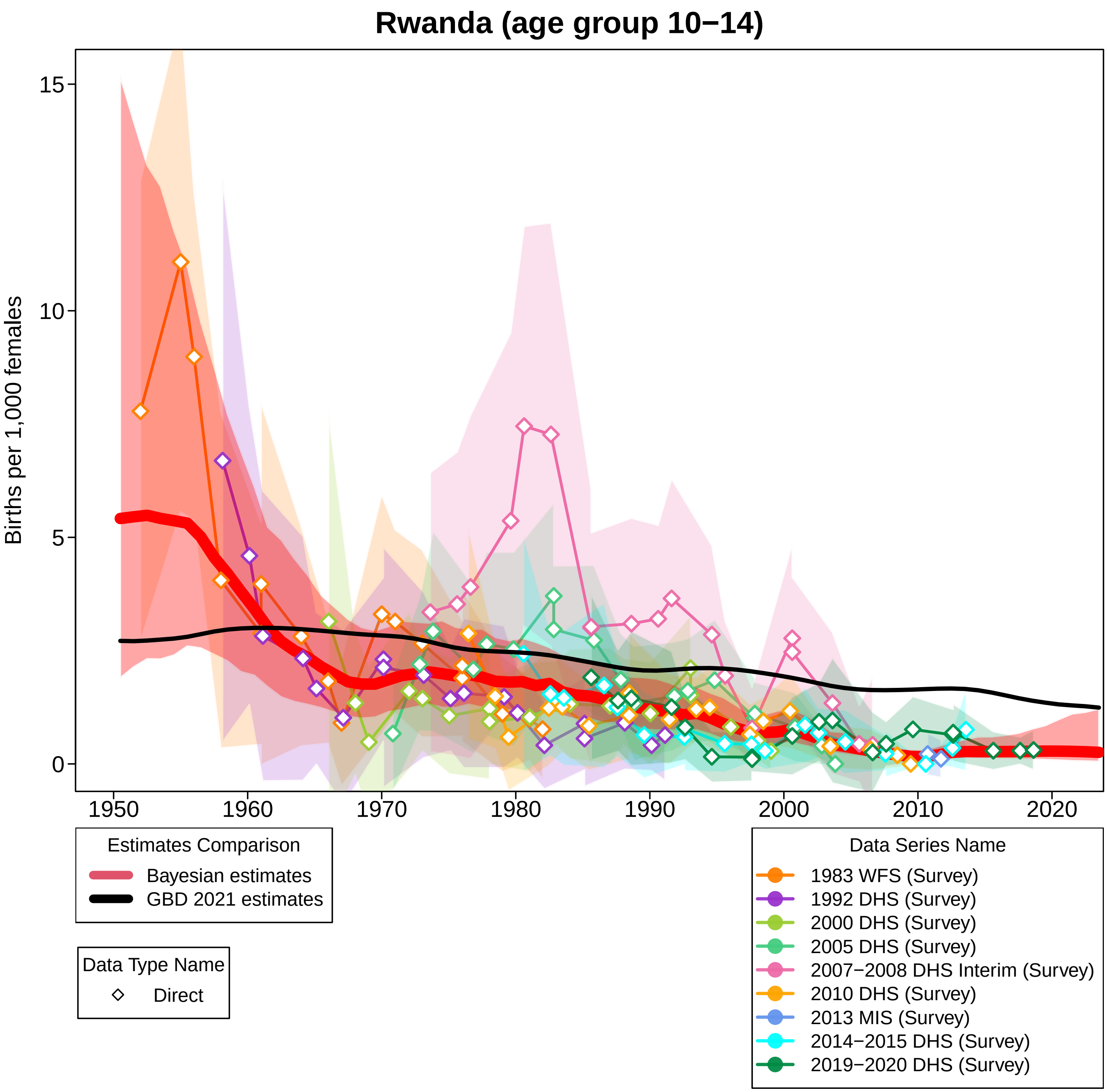
Rwanda (age group 10−14)
Births per 1,000 females
15
10
5
0
1950
1960
1970
1980
1990
2000
2010
2020
Estimates Comparison
Bayesian estimates
GBD 2021 estimates
Data Type Name
Direct
Data Series Name
1983 WFS (Survey)
1992 DHS (Survey)
2000 DHS (Survey)
2005 DHS (Survey)
2007−2008 DHS Interim (Survey)
2010 DHS (Survey)
2013 MIS (Survey)
2014−2015 DHS (Survey)
2019−2020 DHS (Survey)

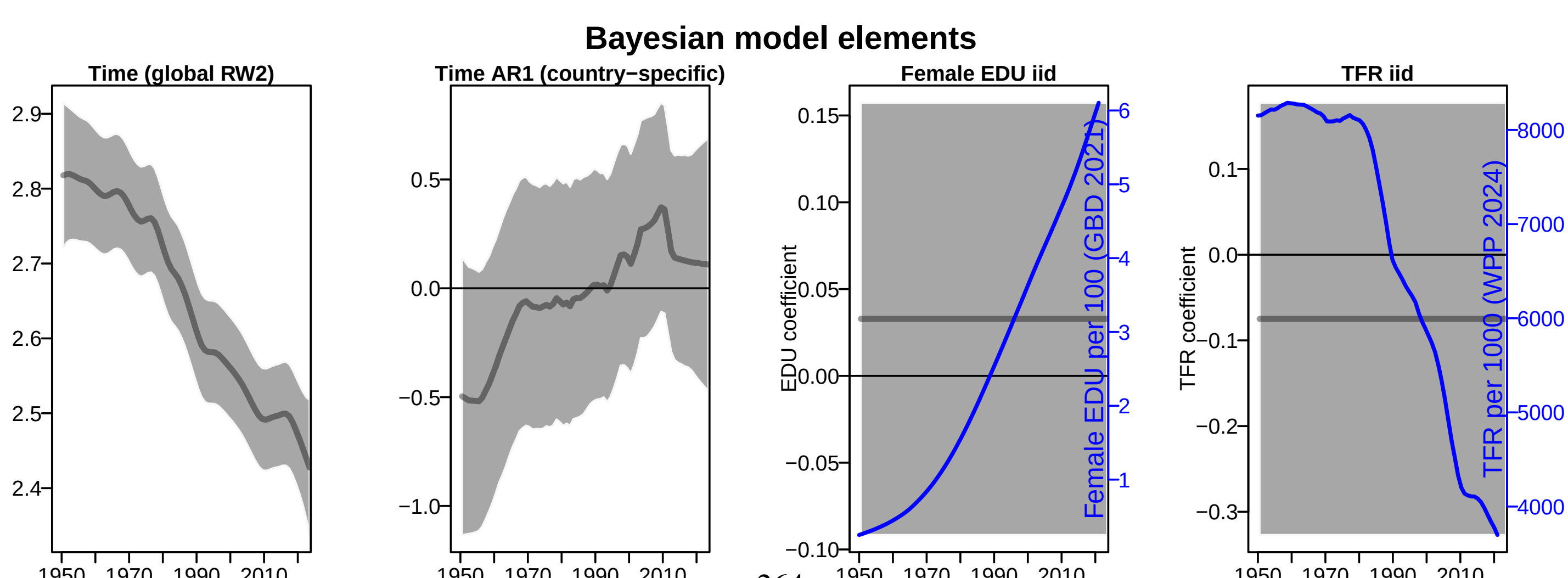
Bayesian model elements
Time (global RW2)
Time AR1 (country−specific)
Female EDU iid
TFR iid
EDU coefficient
Female EDU per 100 (GBD 2021)
TFR coefficient
TFR per 1000 (WPP 2024)
1950
1970
1990
2010

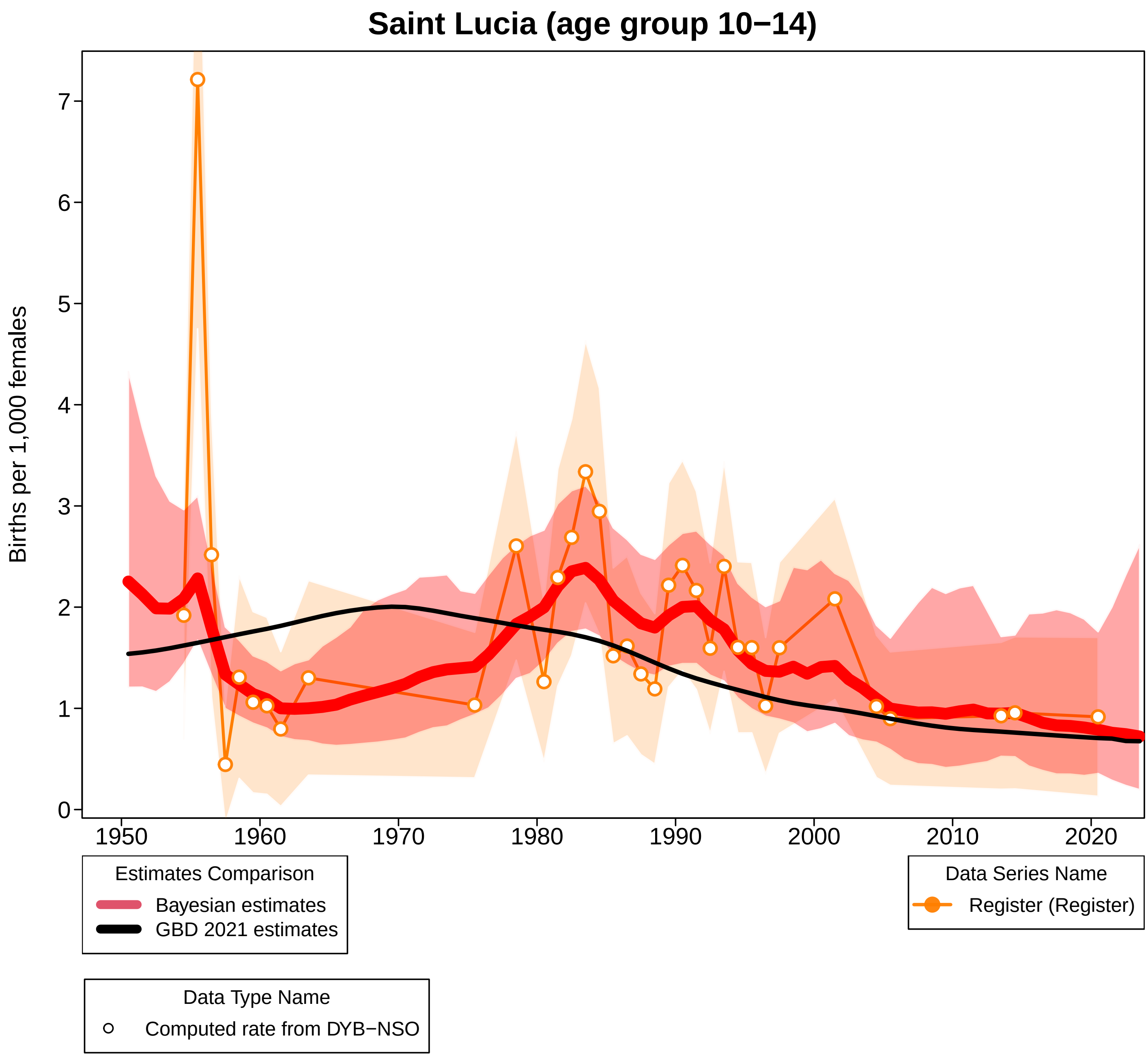
Saint Lucia (age group 10−14)
Births per 1,000 females
0
1
2
3
4
5
6
7
1950
1960
1970
1980
1990
2000
2010
2020
Estimates Comparison
Bayesian estimates
GBD 2021 estimates
Data Series Name
Register (Register)
Data Type Name
Computed rate from DYB−NSO

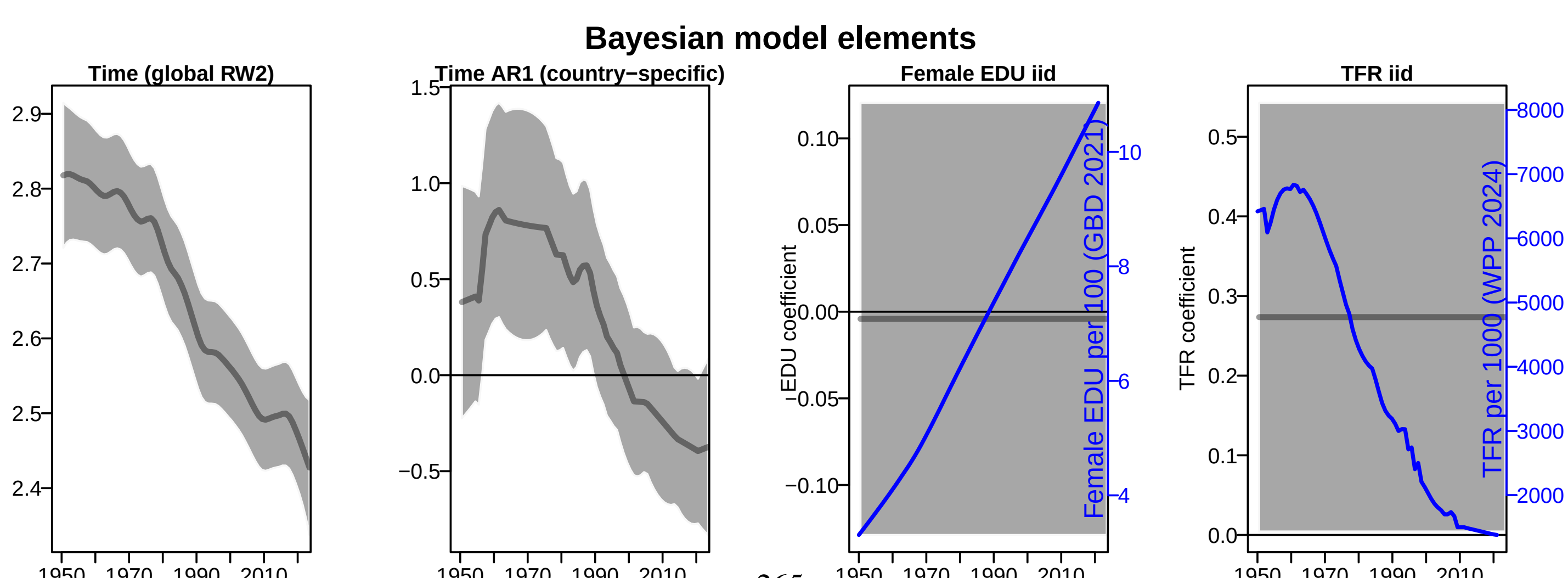
Bayesian model elements
Time (global RW2)
Time AR1 (country−specific)
Female EDU iid
TFR iid
EDU coefficient
Female EDU per 100 (GBD 2021)
TFR coefficient
TFR per 1000 (WPP 2024)
1950
1970
1990
2010

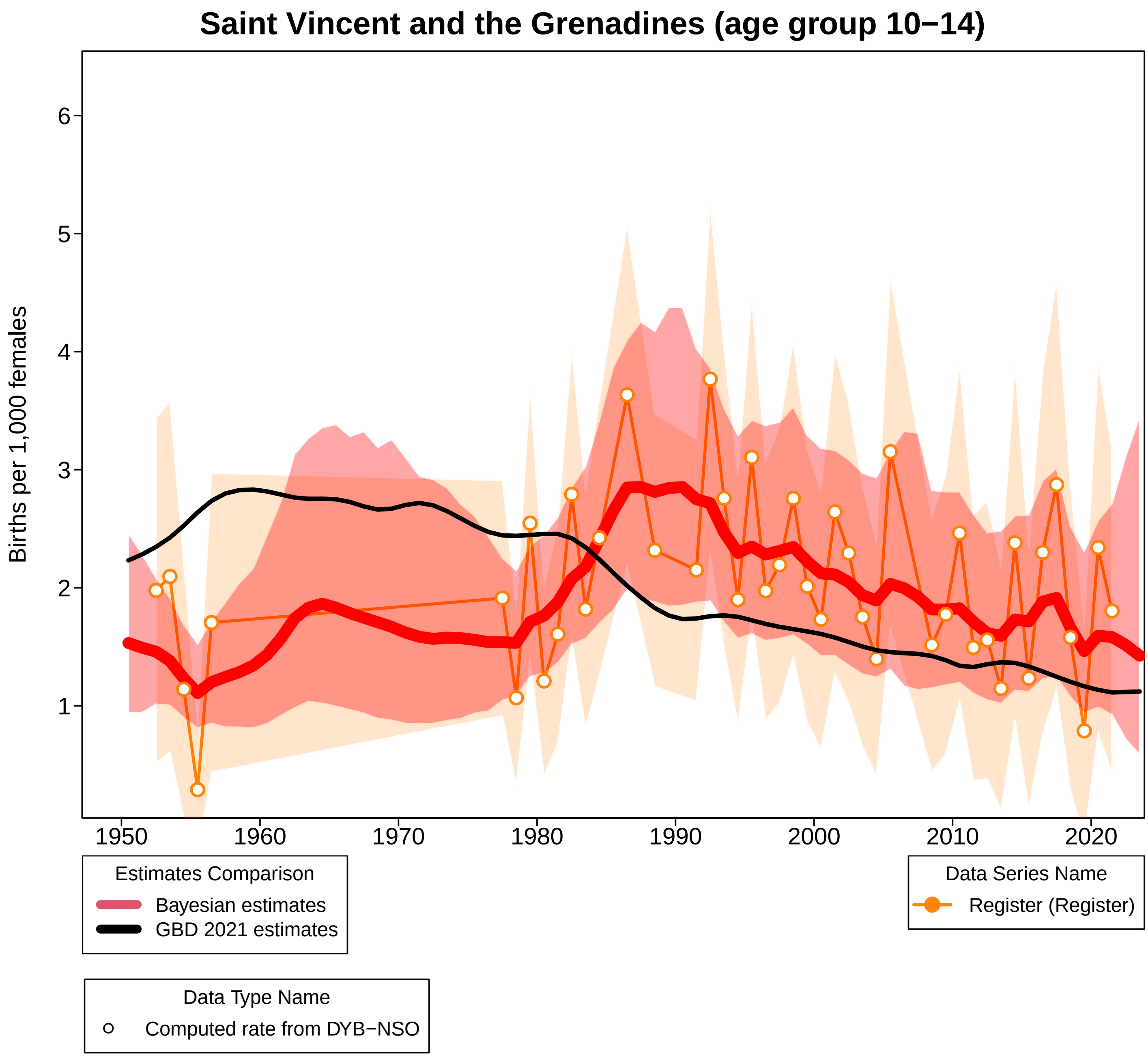

Saint Vincent and the Grenadines (age group 10−14)
Births per 1,000 females
6
5
4
3
2
1
1950
1960
1970
1980
1990
2000
2010
2020
Estimates Comparison
Bayesian estimates
GBD 2021 estimates
Data Series Name
Register (Register)
Data Type Name
Computed rate from DYB−NSO

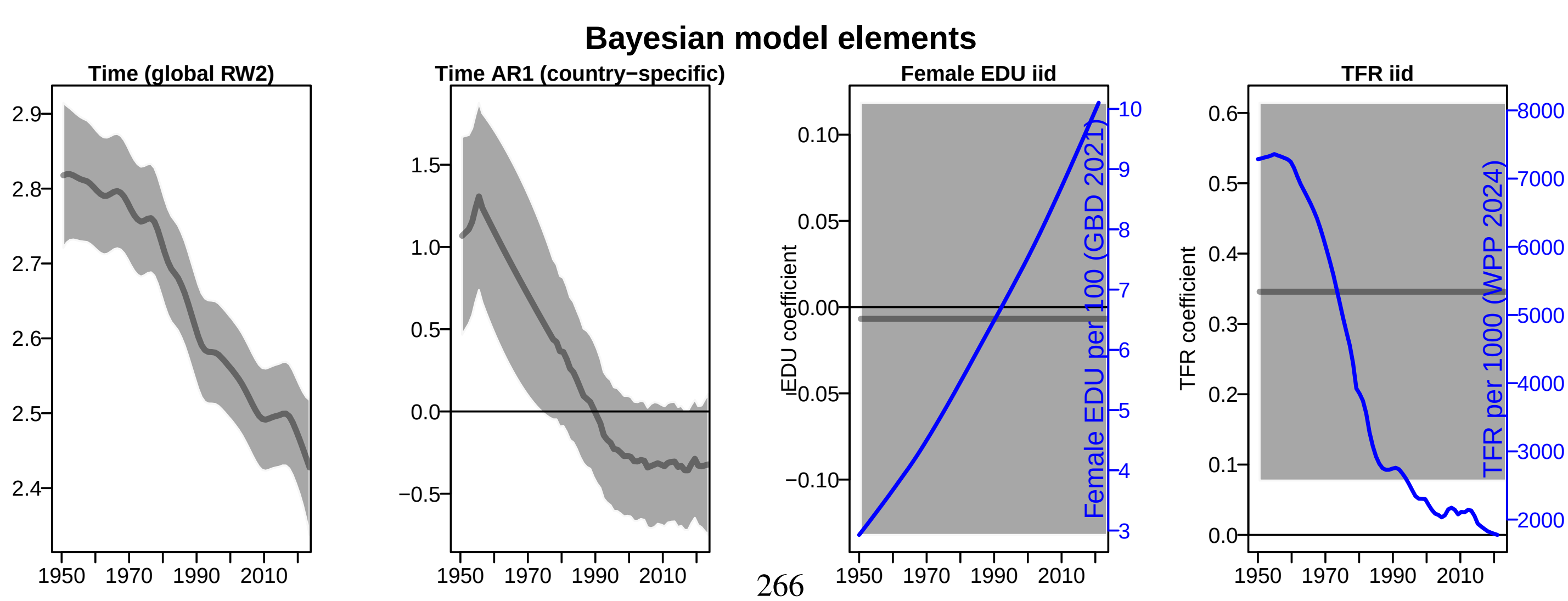

Bayesian model elements
Time (global RW2)
Time AR1 (country−specific)
Female EDU iid
TFR iid
EDU coefficient
Female EDU per 100 (GBD 2021)
TFR coefficient
TFR per 1000 (WPP 2024)
1950
1970
1990
2010

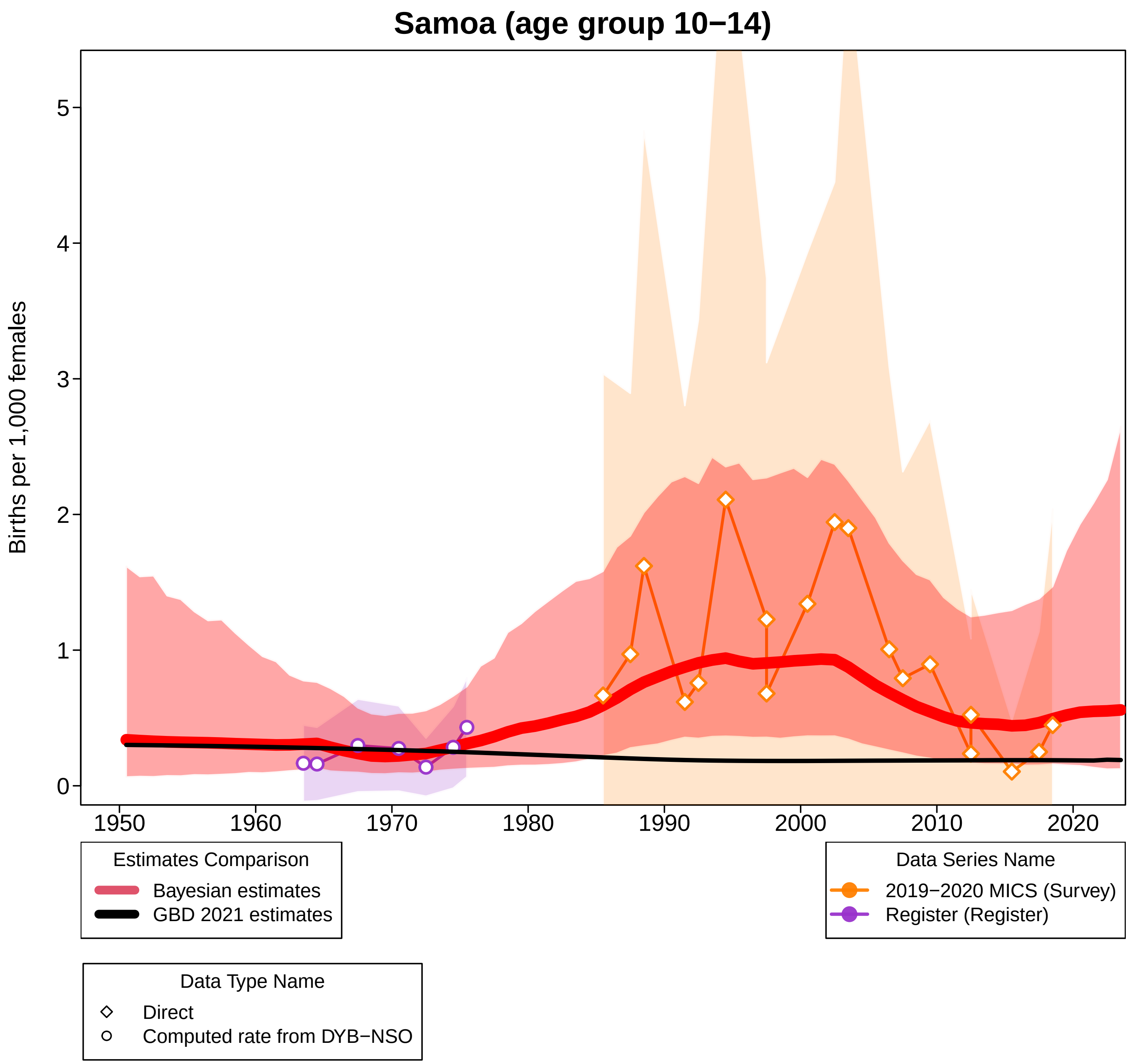
Samoa (age group 10−14)
Births per 1,000 females
0
1
2
3
4
5
1950
1960
1970
1980
1990
2000
2010
2020
Estimates Comparison
Bayesian estimates
GBD 2021 estimates
Data Series Name
2019−2020 MICS (Survey)
Register (Register)
Data Type Name
Direct
Computed rate from DYB−NSO

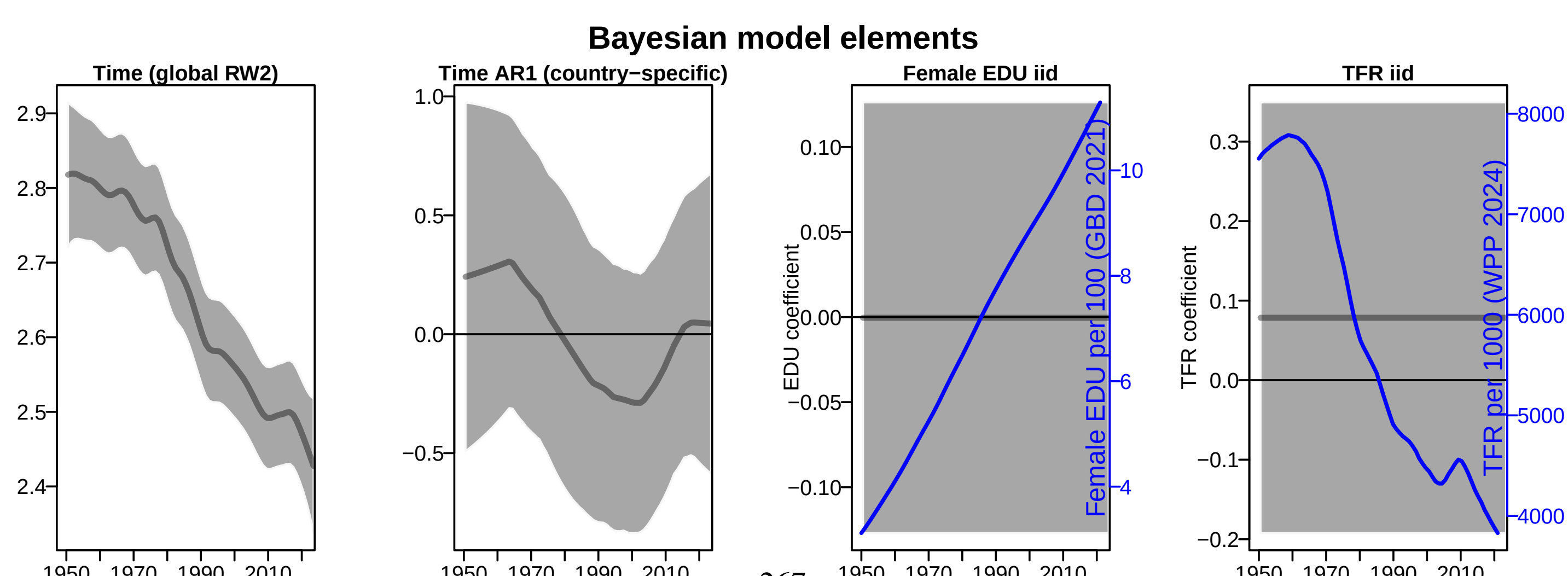
Bayesian model elements
Time (global RW2)
Time AR1 (country−specific)
Female EDU iid
TFR iid
EDU coefficient
Female EDU per 100 (GBD 2021)
TFR coefficient
TFR per 1000 (WPP 2024)
1950
1970
1990
2010

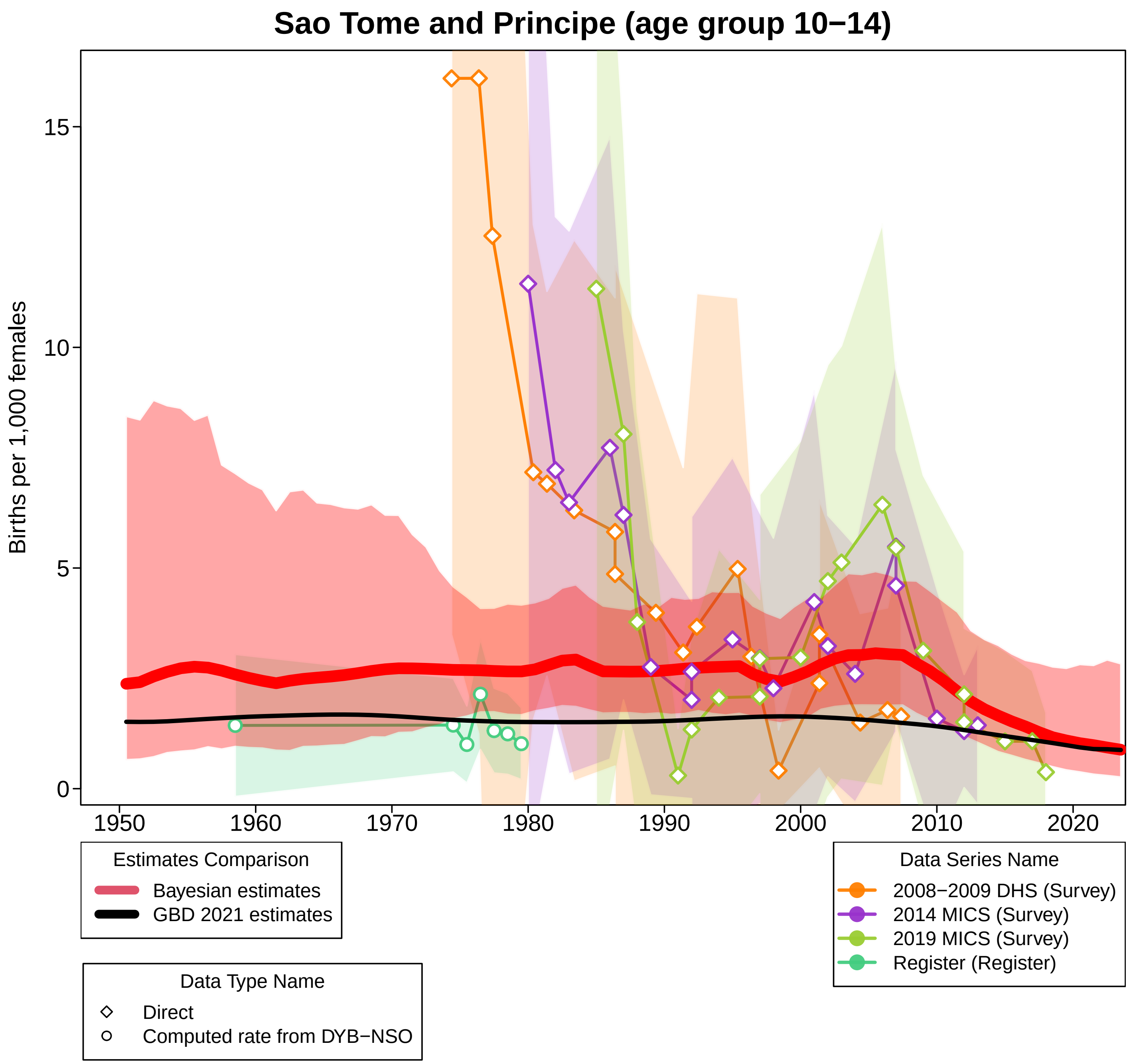
Sao Tome and Principe (age group 10−14)
Births per 1,000 females
0
5
10
15
1950
1960
1970
1980
1990
2000
2010
2020
Estimates Comparison
Bayesian estimates
GBD 2021 estimates
Data Series Name
2008−2009 DHS (Survey)
2014 MICS (Survey)
2019 MICS (Survey)
Register (Register)
Data Type Name
Direct
Computed rate from DYB−NSO

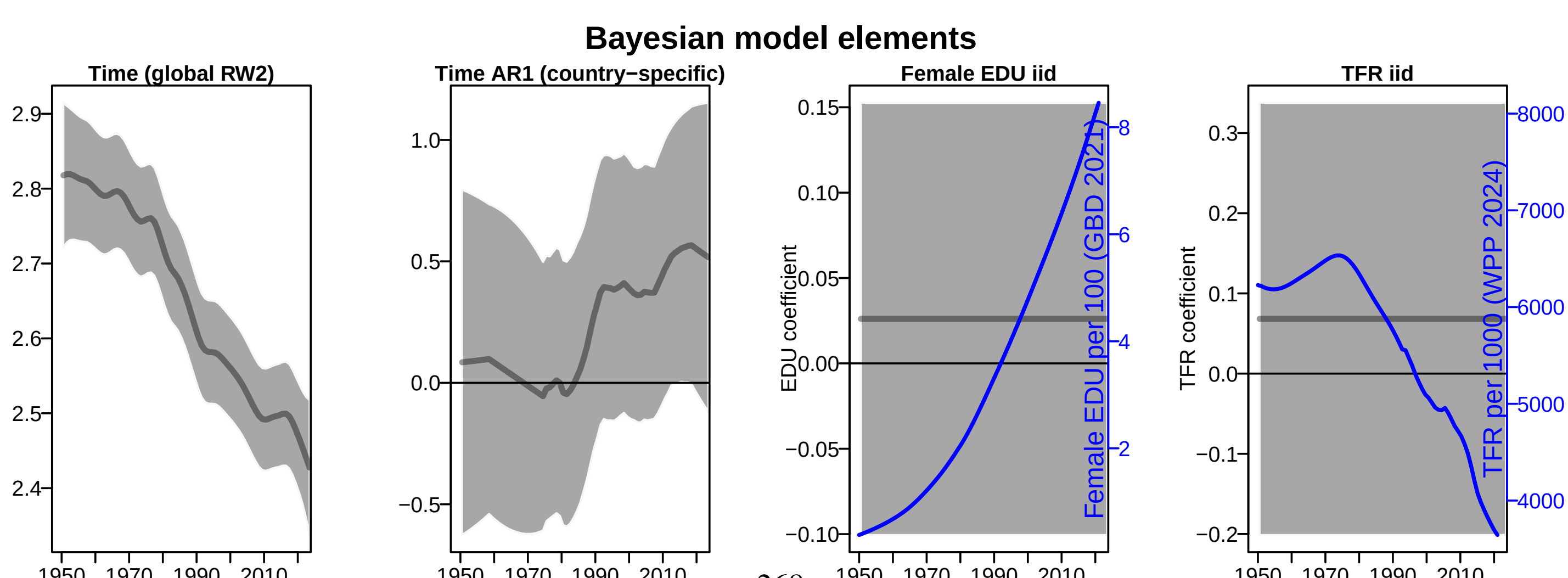
Bayesian model elements
Time (global RW2)
Time AR1 (country−specific)
Female EDU iid
TFR iid
EDU coefficient
Female EDU per 100 (GBD 2021)
TFR coefficient
TFR per 1000 (WPP 2024)
1950
1970
1990
2010

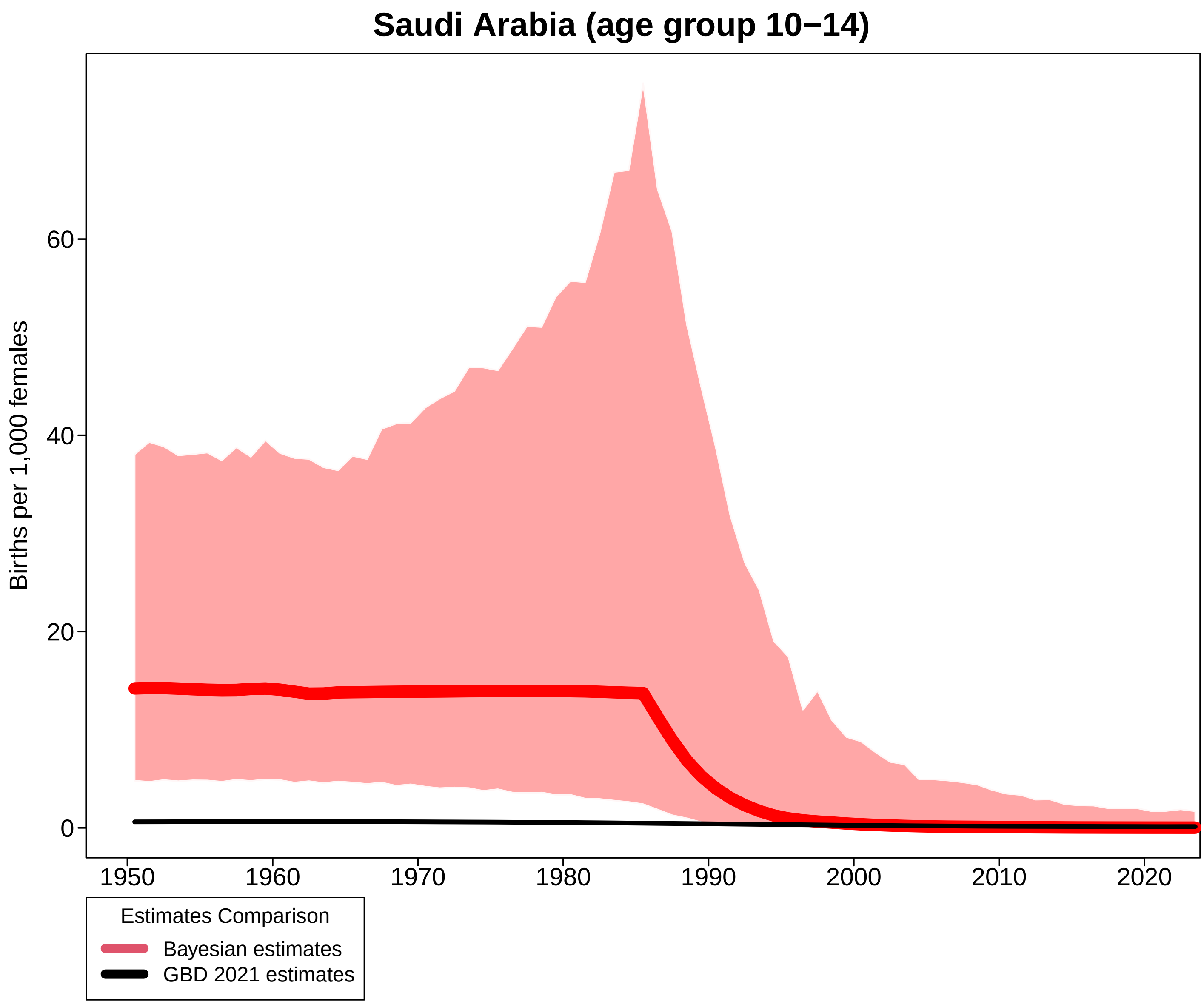
Saudi Arabia (age group 10−14)
Births per 1,000 females
0
20
40
60
1950
1960
1970
1980
1990
2000
2010
2020
Estimates Comparison
Bayesian estimates
GBD 2021 estimates

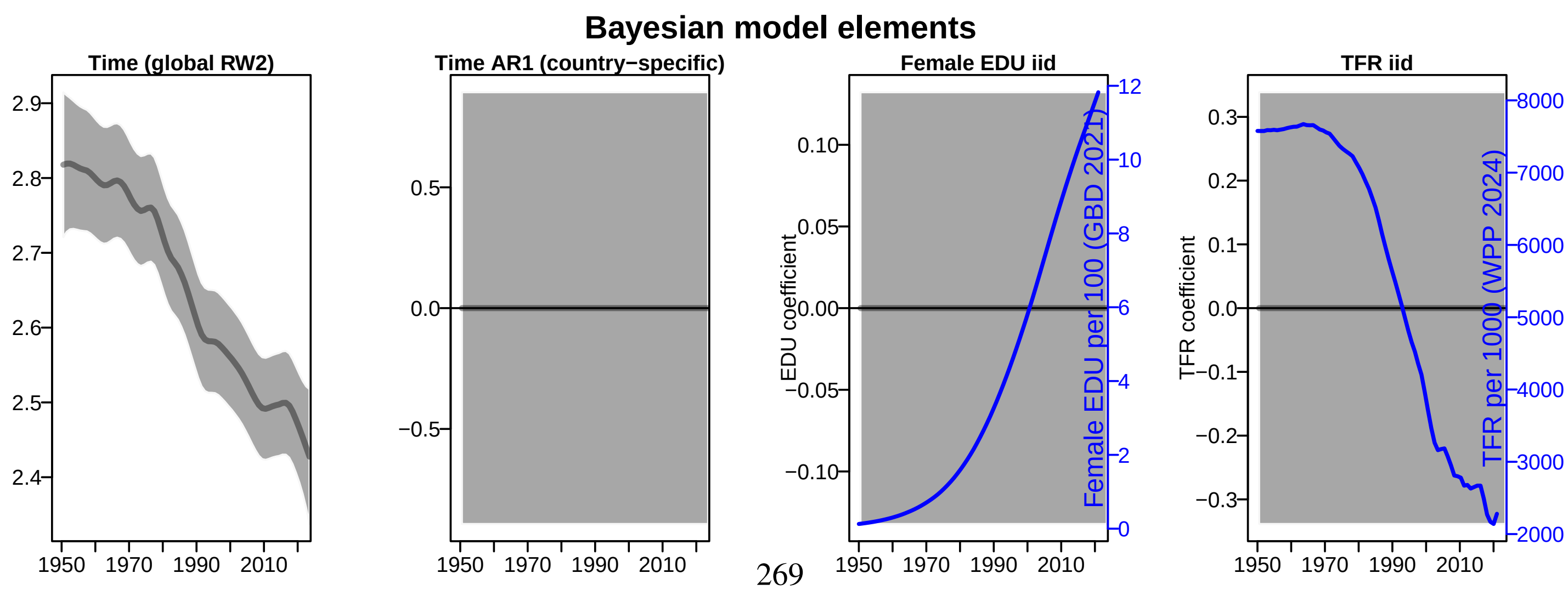
Bayesian model elements
Time (global RW2)
Time AR1 (country−specific)
Female EDU iid
TFR iid
EDU coefficient
Female EDU per 100 (GBD 2021)
TFR coefficient
TFR per 1000 (WPP 2024)
1950
1970
1990
2010

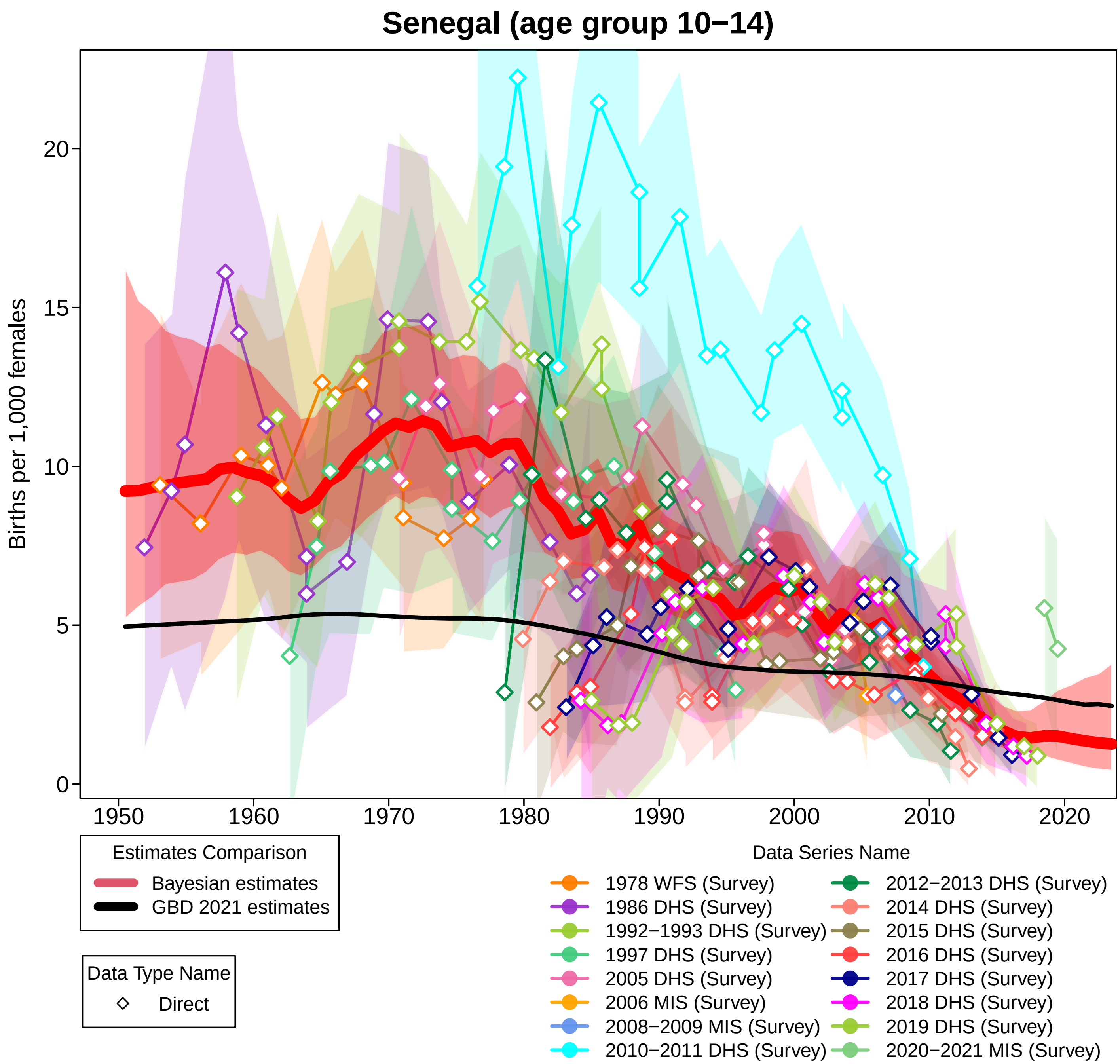

Senegal (age group 10−14)
Births per 1,000 females
0
5
10
15
20
1950
1960
1970
1980
1990
2000
2010
2020
Estimates Comparison
Bayesian estimates
GBD 2021 estimates
Data Type Name
Direct
Data Series Name
1978 WFS (Survey)
1986 DHS (Survey)
1992−1993 DHS (Survey)
1997 DHS (Survey)
2005 DHS (Survey)
2006 MIS (Survey)
2008−2009 MIS (Survey)
2010−2011 DHS (Survey)
2012−2013 DHS (Survey)
2014 DHS (Survey)
2015 DHS (Survey)
2016 DHS (Survey)
2017 DHS (Survey)
2018 DHS (Survey)
2019 DHS (Survey)
2020−2021 MIS (Survey)

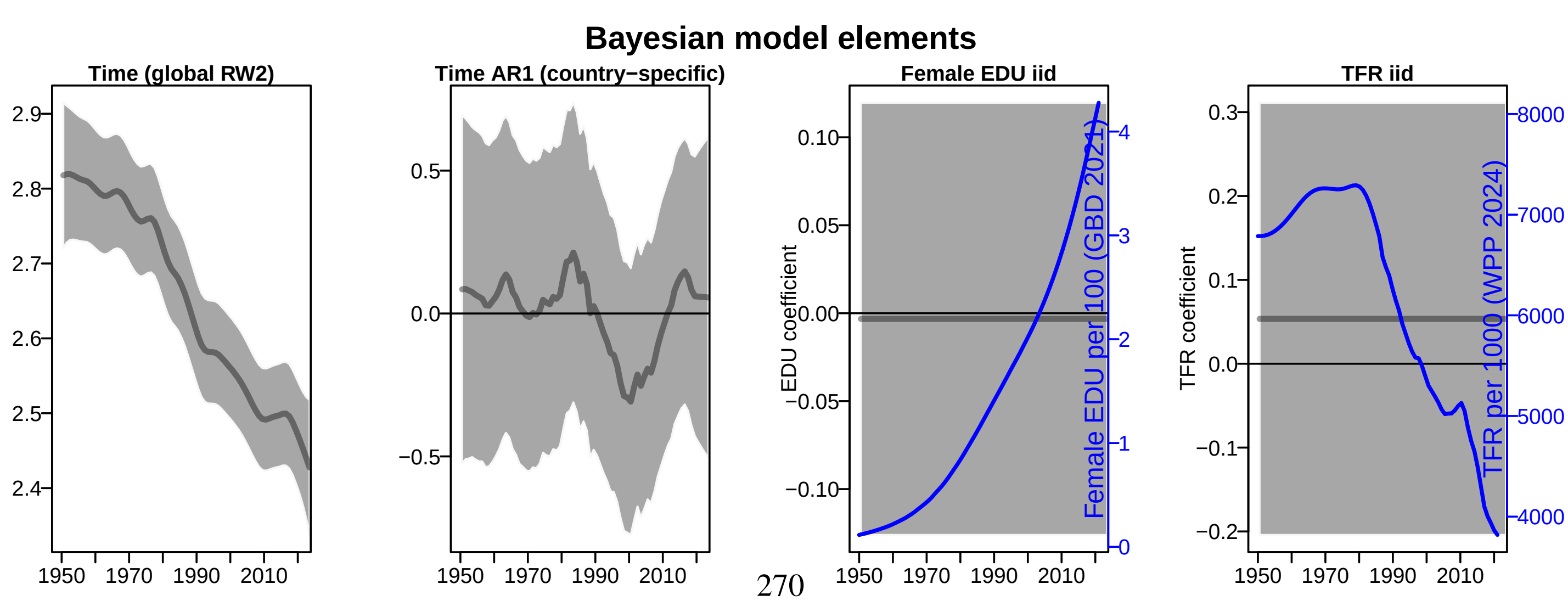

Bayesian model elements
Time (global RW2)
Time AR1 (country−specific)
Female EDU iid
TFR iid
EDU coefficient
Female EDU per 100 (GBD 2021)
TFR coefficient
TFR per 1000 (WPP 2024)
1950
1970
1990
2010

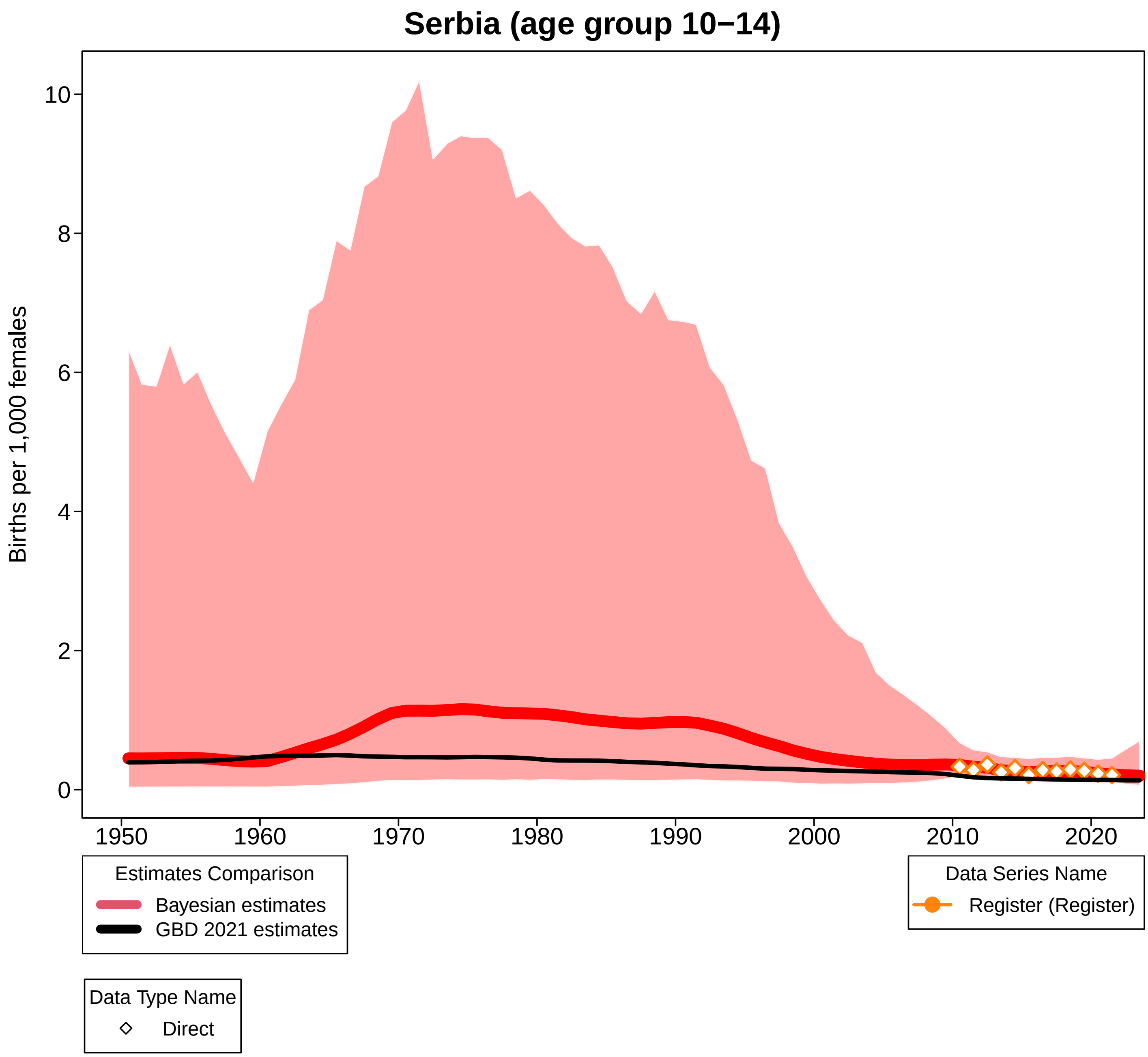

Serbia (age group 10−14)
Births per 1,000 females
0
2
4
6
8
10
1950
1960
1970
1980
1990
2000
2010
2020
Estimates Comparison
Bayesian estimates
GBD 2021 estimates
Data Series Name
Register (Register)
Data Type Name
Direct

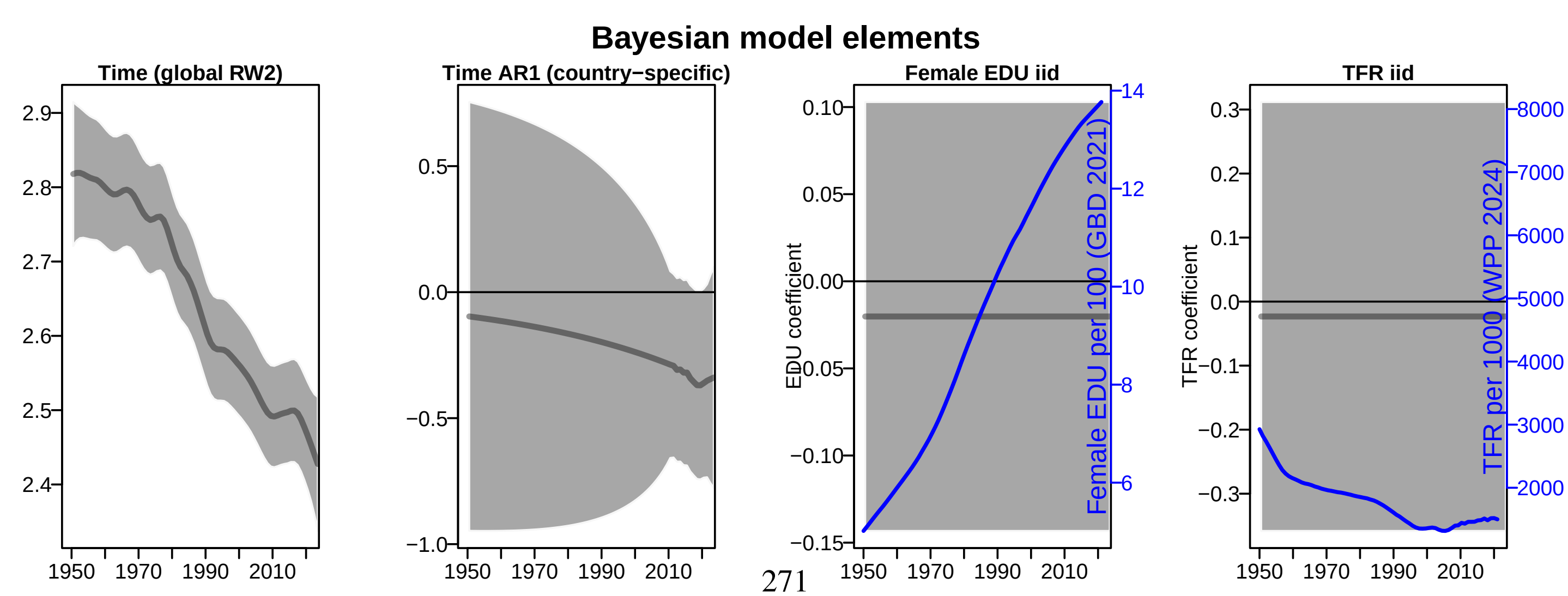

Bayesian model elements
Time (global RW2)
Time AR1 (country−specific)
Female EDU iid
TFR iid
EDU coefficient
Female EDU per 100 (GBD 2021)
TFR coefficient
TFR per 1000 (WPP 2024)
1950
1970
1990
2010

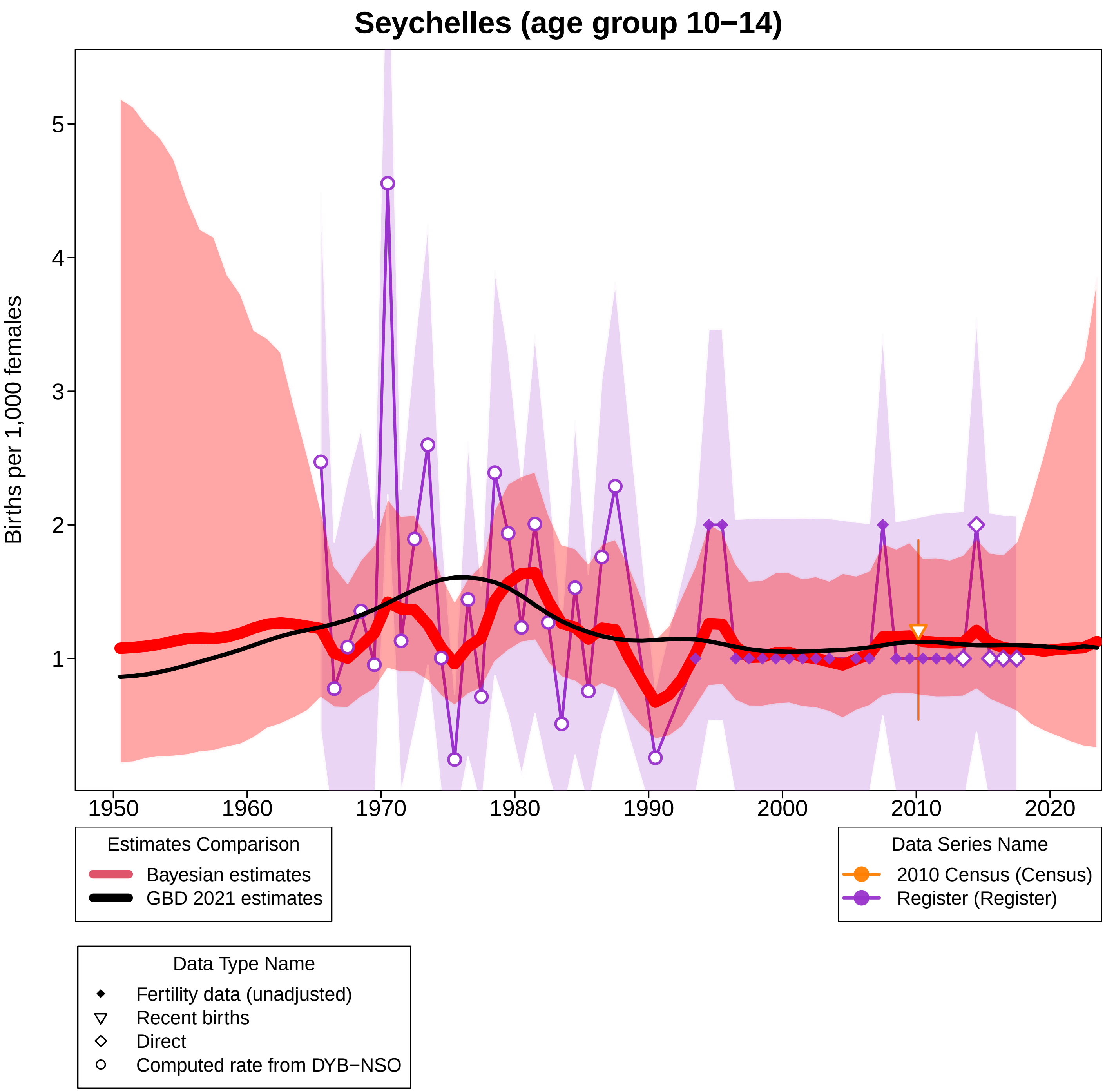

Seychelles (age group 10−14)
Births per 1,000 females
1
2
3
4
5
1950
1960
1970
1980
1990
2000
2010
2020
Estimates Comparison
Bayesian estimates
GBD 2021 estimates
Data Series Name
2010 Census (Census)
Register (Register)
Data Type Name
Fertility data (unadjusted)
Recent births
Direct
Computed rate from DYB−NSO

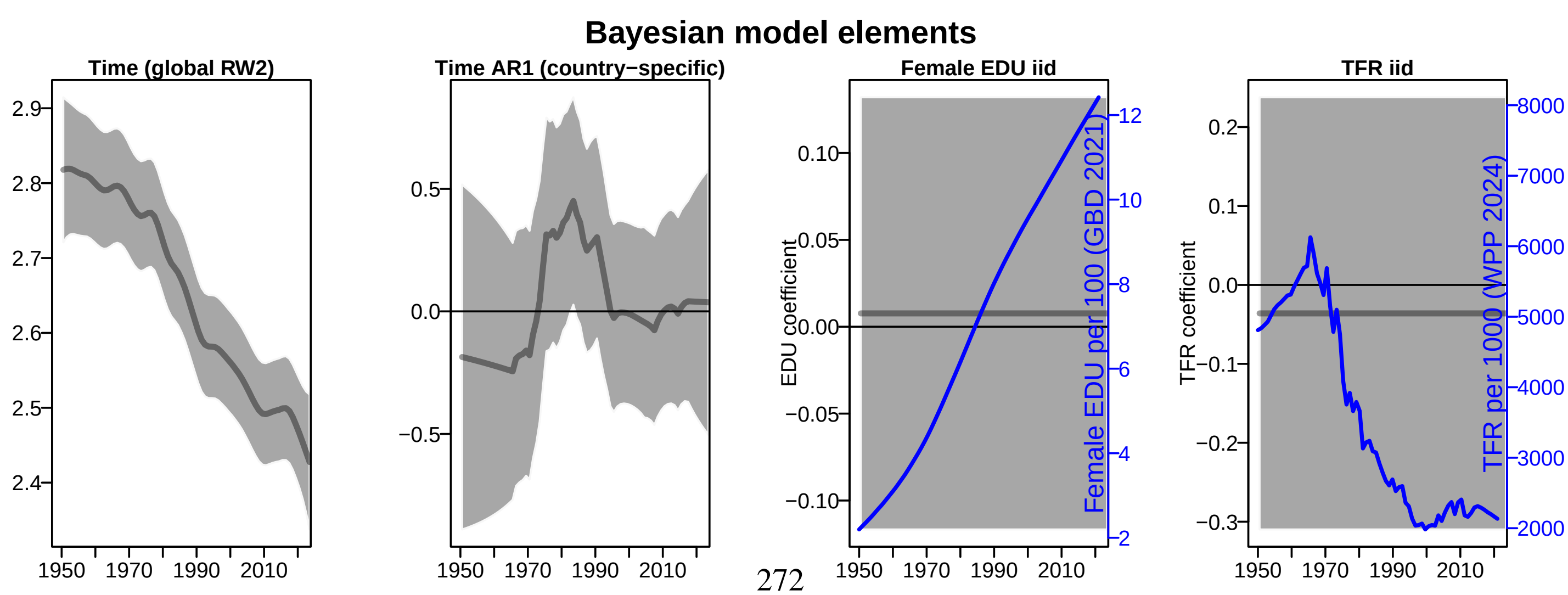

Bayesian model elements
Time (global RW2)
Time AR1 (country−specific)
Female EDU iid
TFR iid
EDU coefficient
Female EDU per 100 (GBD 2021)
TFR coefficient
TFR per 1000 (WPP 2024)

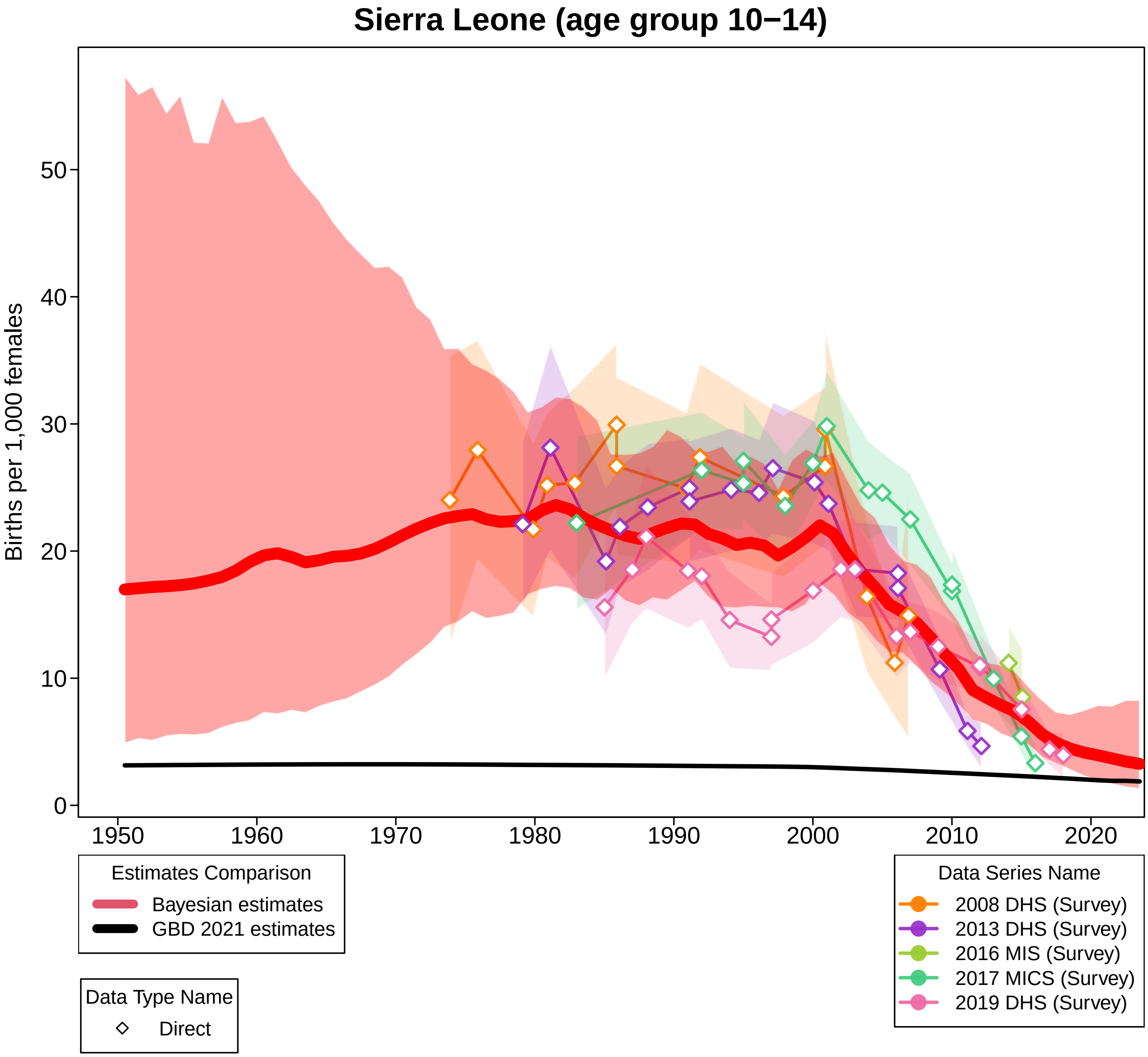

Sierra Leone (age group 10−14)
Births per 1,000 females
0
10
20
30
40
50
1950
1960
1970
1980
1990
2000
2010
2020
Estimates Comparison
Bayesian estimates
GBD 2021 estimates
Data Type Name
Direct
Data Series Name
2008 DHS (Survey)
2013 DHS (Survey)
2016 MIS (Survey)
2017 MICS (Survey)
2019 DHS (Survey)

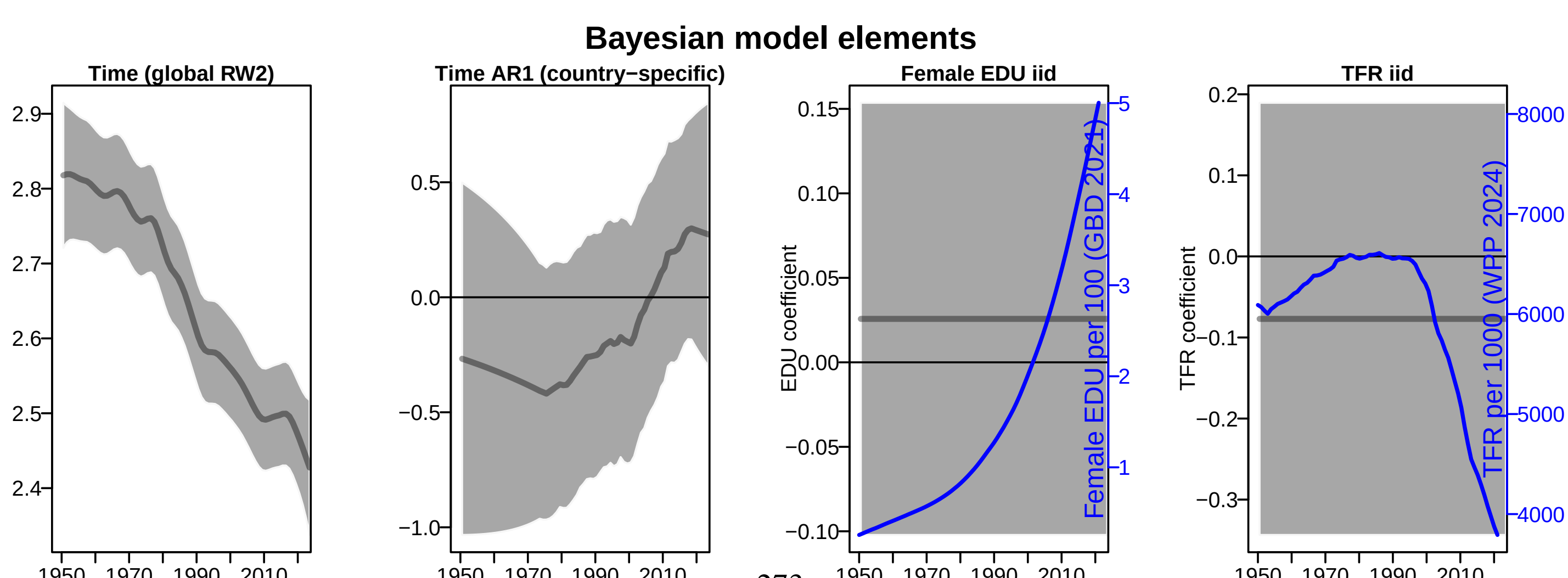

Bayesian model elements
Time (global RW2)
Time AR1 (country−specific)
Female EDU iid
TFR iid
EDU coefficient
Female EDU per 100 (GBD 2021)
TFR coefficient
TFR per 1000 (WPP 2024)
1950
1970
1990
2010

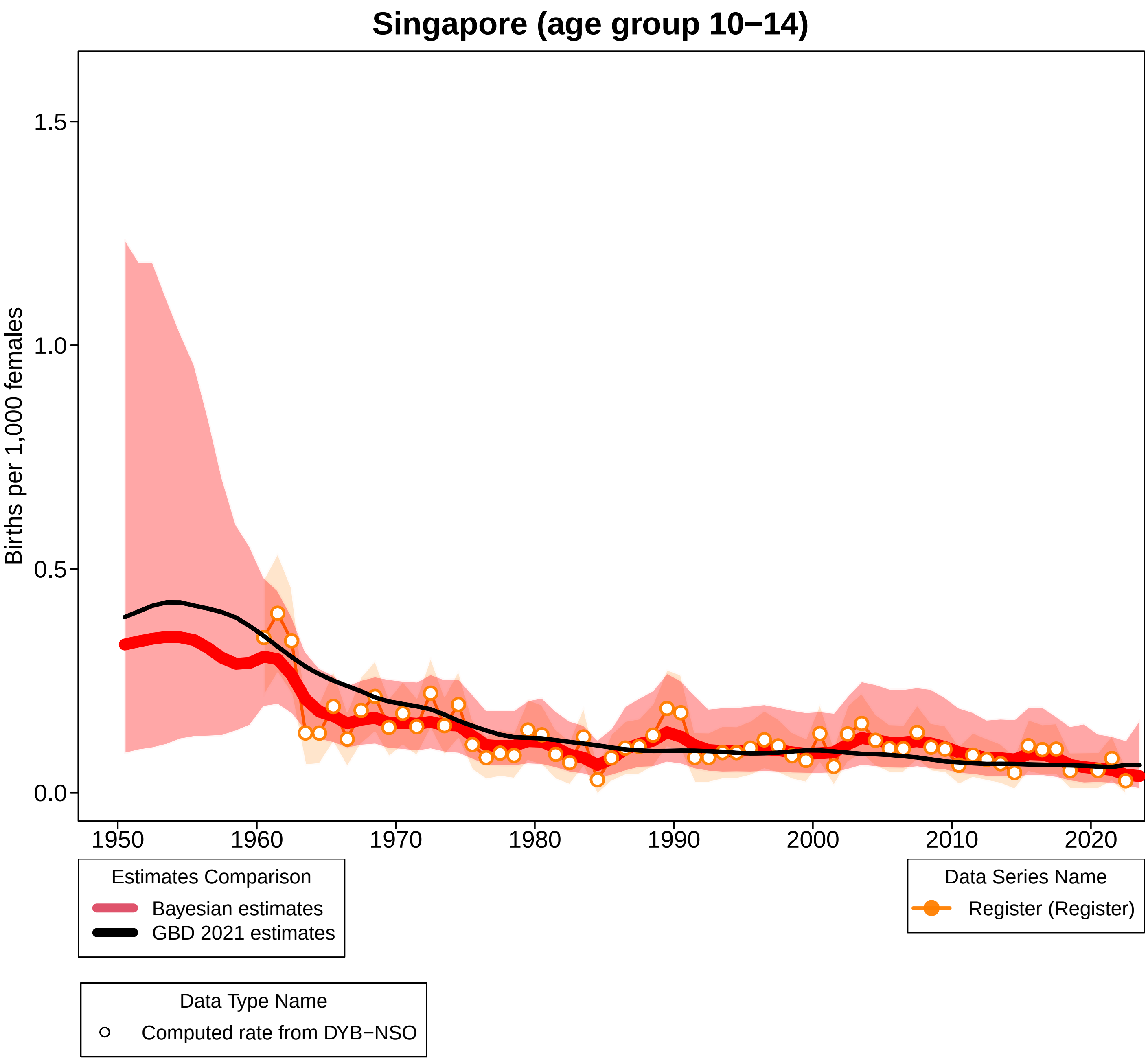
Singapore (age group 10−14)
Births per 1,000 females
1.5
1.0
0.5
0.0
1950
1960
1970
1980
1990
2000
2010
2020
Estimates Comparison
Bayesian estimates
GBD 2021 estimates
Data Series Name
Register (Register)
Data Type Name
Computed rate from DYB−NSO

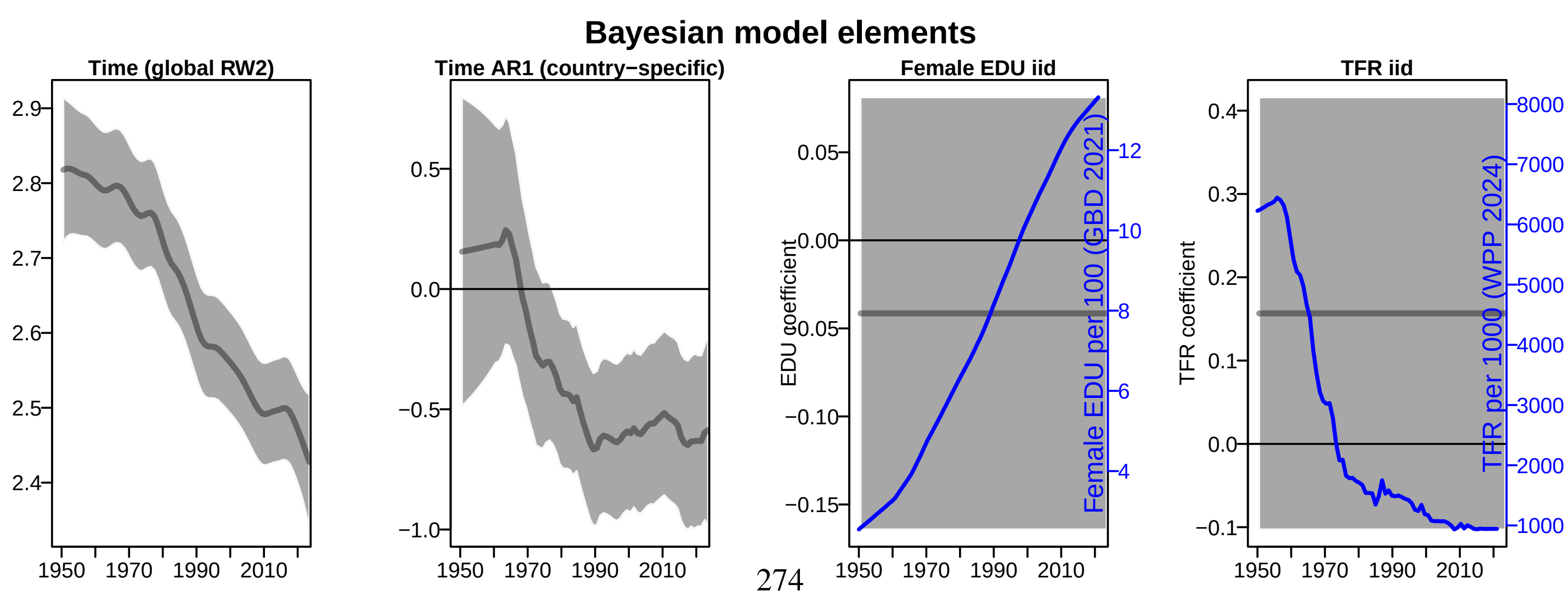
Bayesian model elements
Time (global RW2)
Time AR1 (country−specific)
Female EDU iid
TFR iid
EDU coefficient
Female EDU per 100 (GBD 2021)
TFR coefficient
TFR per 1000 (WPP 2024)

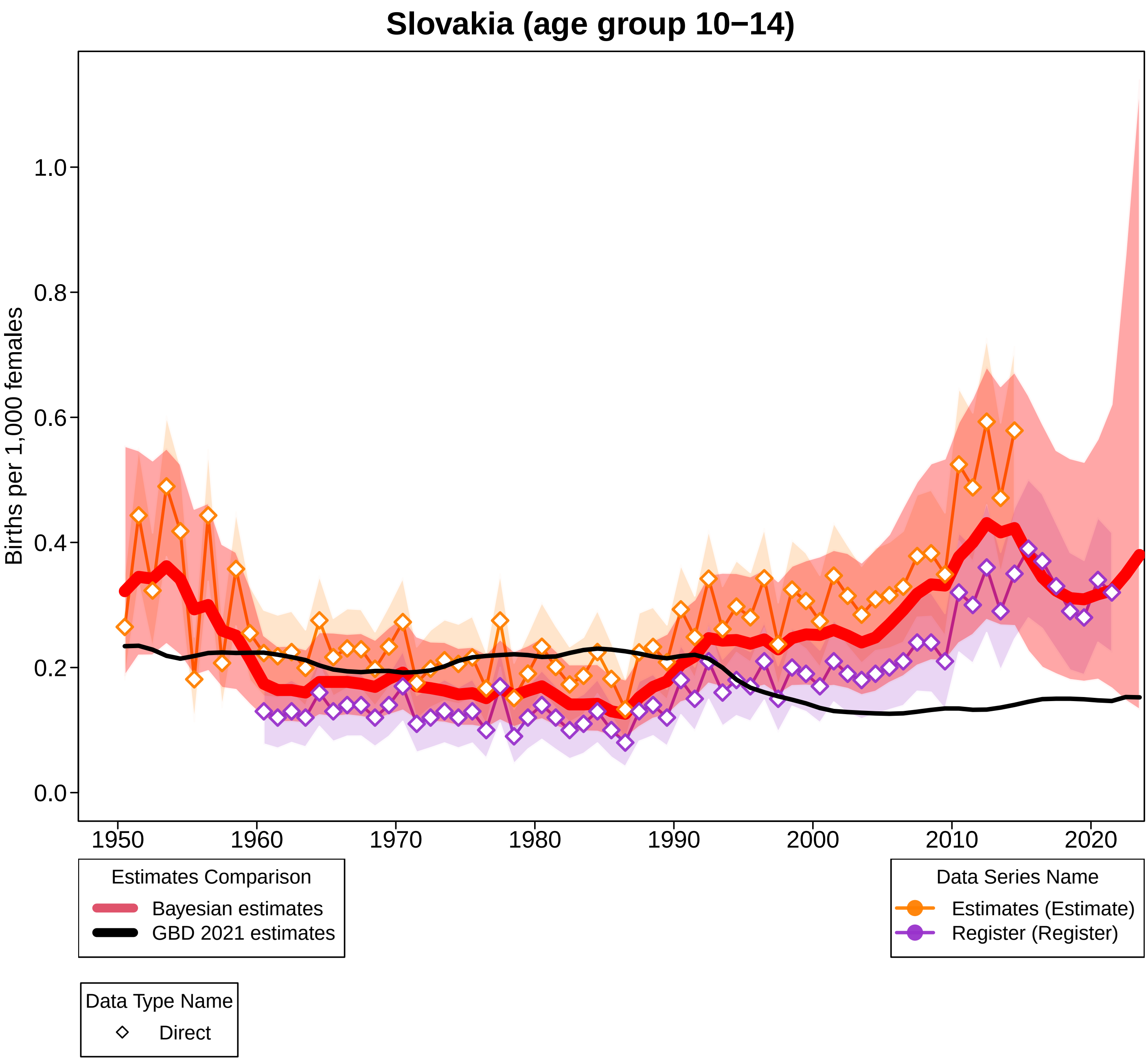
Slovakia (age group 10−14)
Births per 1,000 females
0.0
0.2
0.4
0.6
0.8
1.0
1950
1960
1970
1980
1990
2000
2010
2020
Estimates Comparison
Bayesian estimates
GBD 2021 estimates
Data Series Name
Estimates (Estimate)
Register (Register)
Data Type Name
Direct

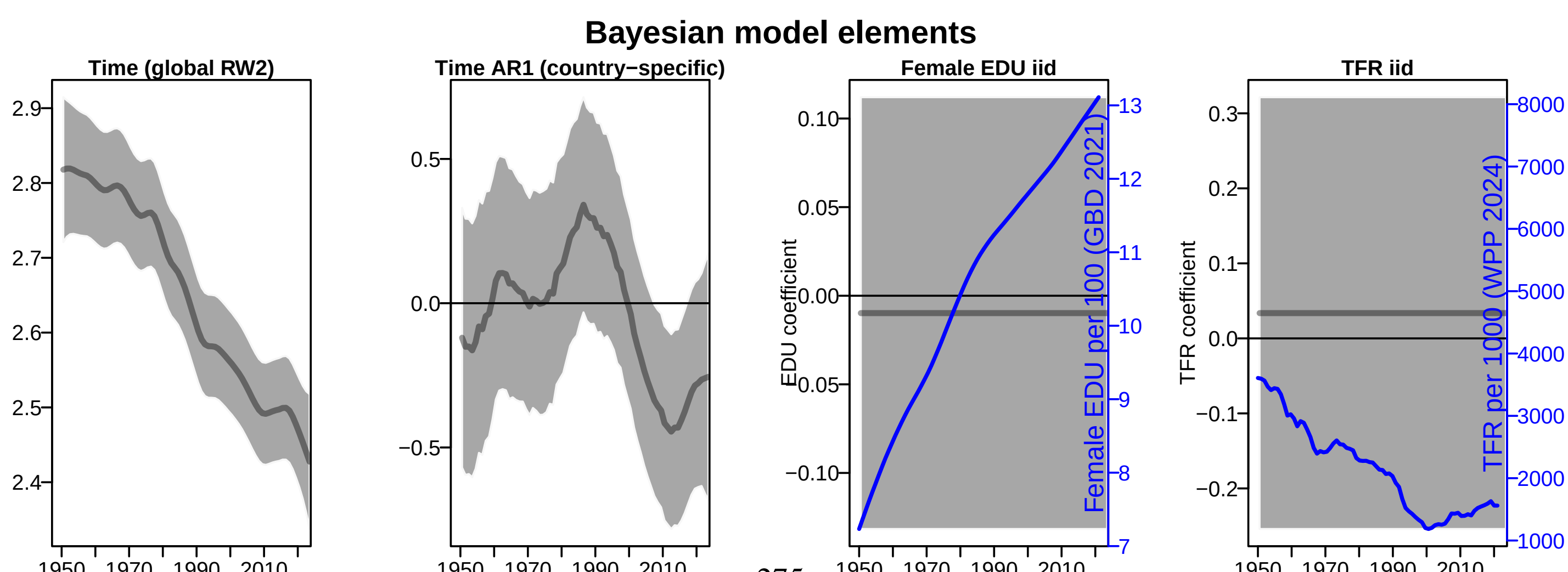
Bayesian model elements
Time (global RW2)
Time AR1 (country−specific)
Female EDU iid
TFR iid
EDU coefficient
Female EDU per 100 (GBD 2021)
TFR coefficient
TFR per 1000 (WPP 2024)
1950
1970
1990
2010

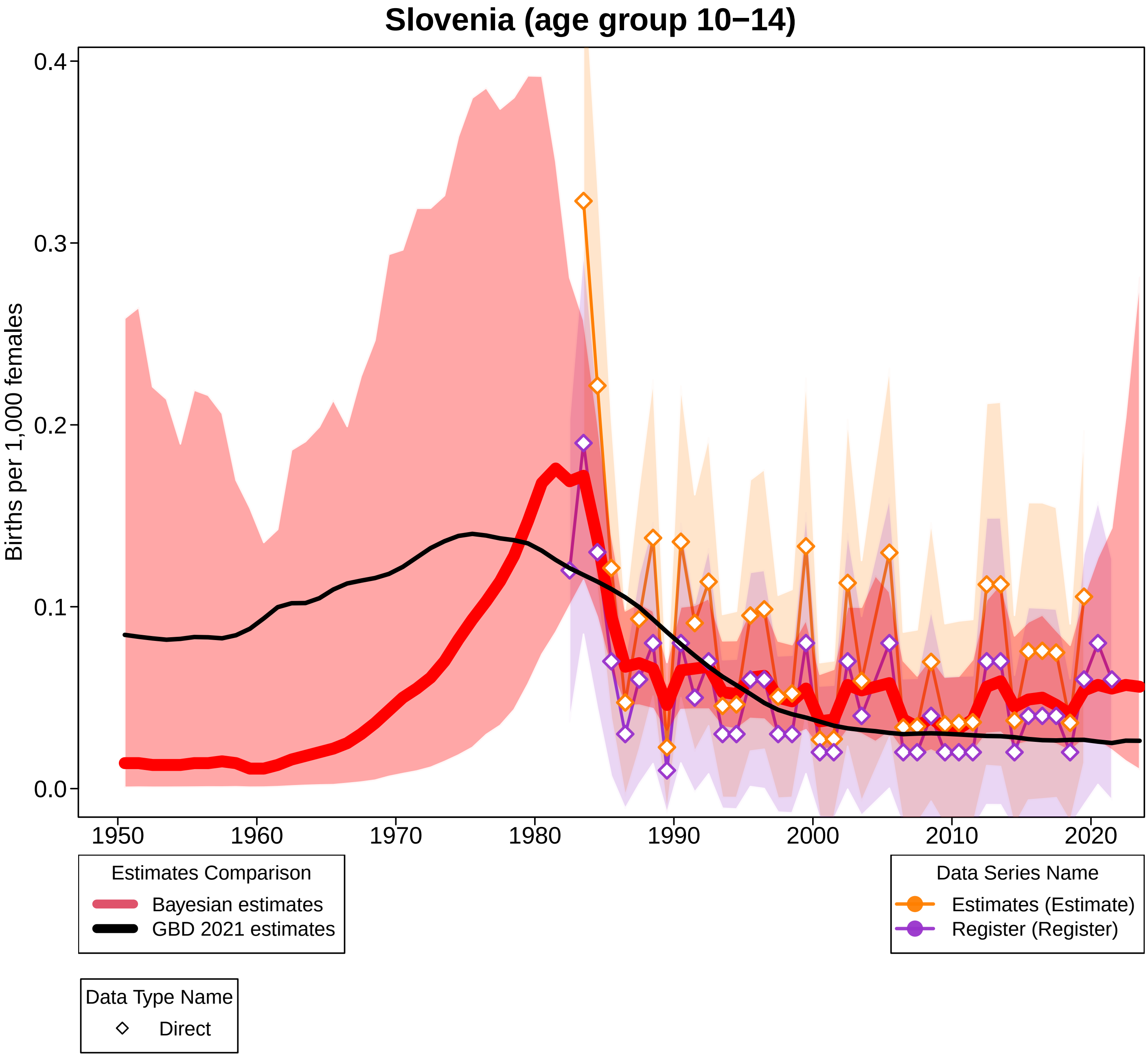
Slovenia (age group 10−14)
Births per 1,000 females
0.0
0.1
0.2
0.3
0.4
1950
1960
1970
1980
1990
2000
2010
2020
Estimates Comparison
Bayesian estimates
GBD 2021 estimates
Data Series Name
Estimates (Estimate)
Register (Register)
Data Type Name
Direct

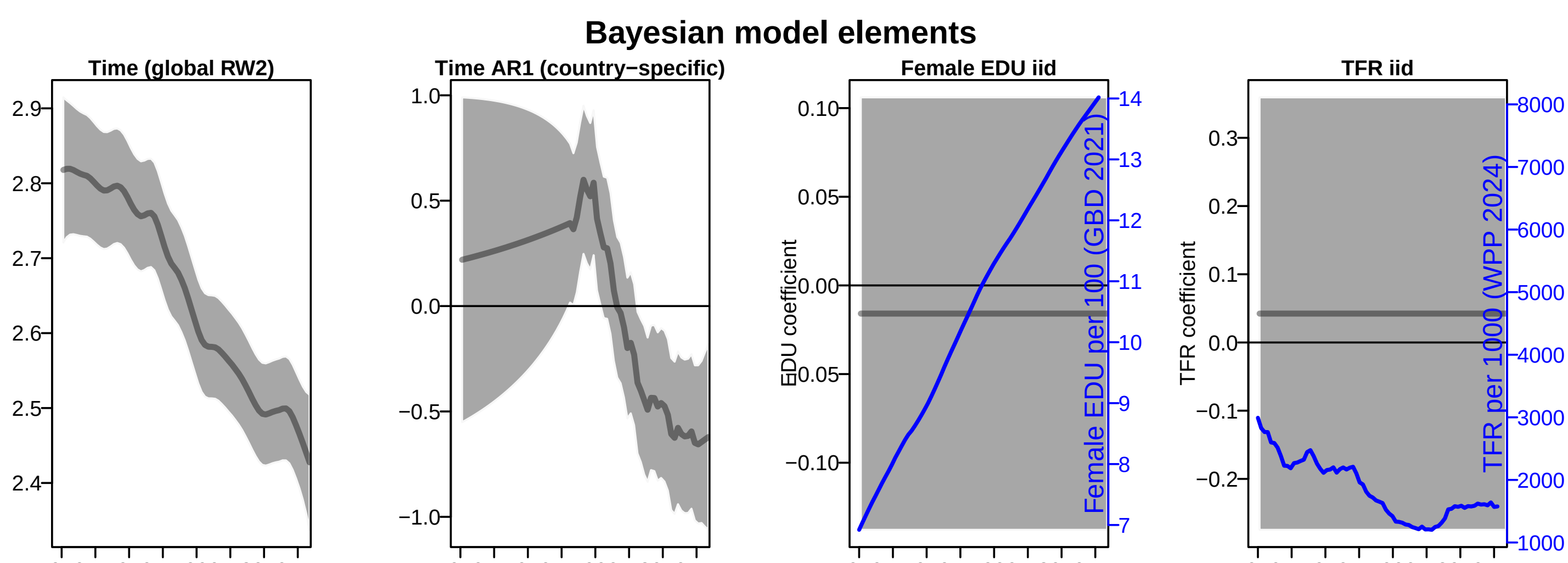
Bayesian model elements
Time (global RW2)
Time AR1 (country−specific)
Female EDU iid
TFR iid
EDU coefficient
Female EDU per 100 (GBD 2021)
TFR coefficient
TFR per 1000 (WPP 2024)
1950
1970
1990
2010

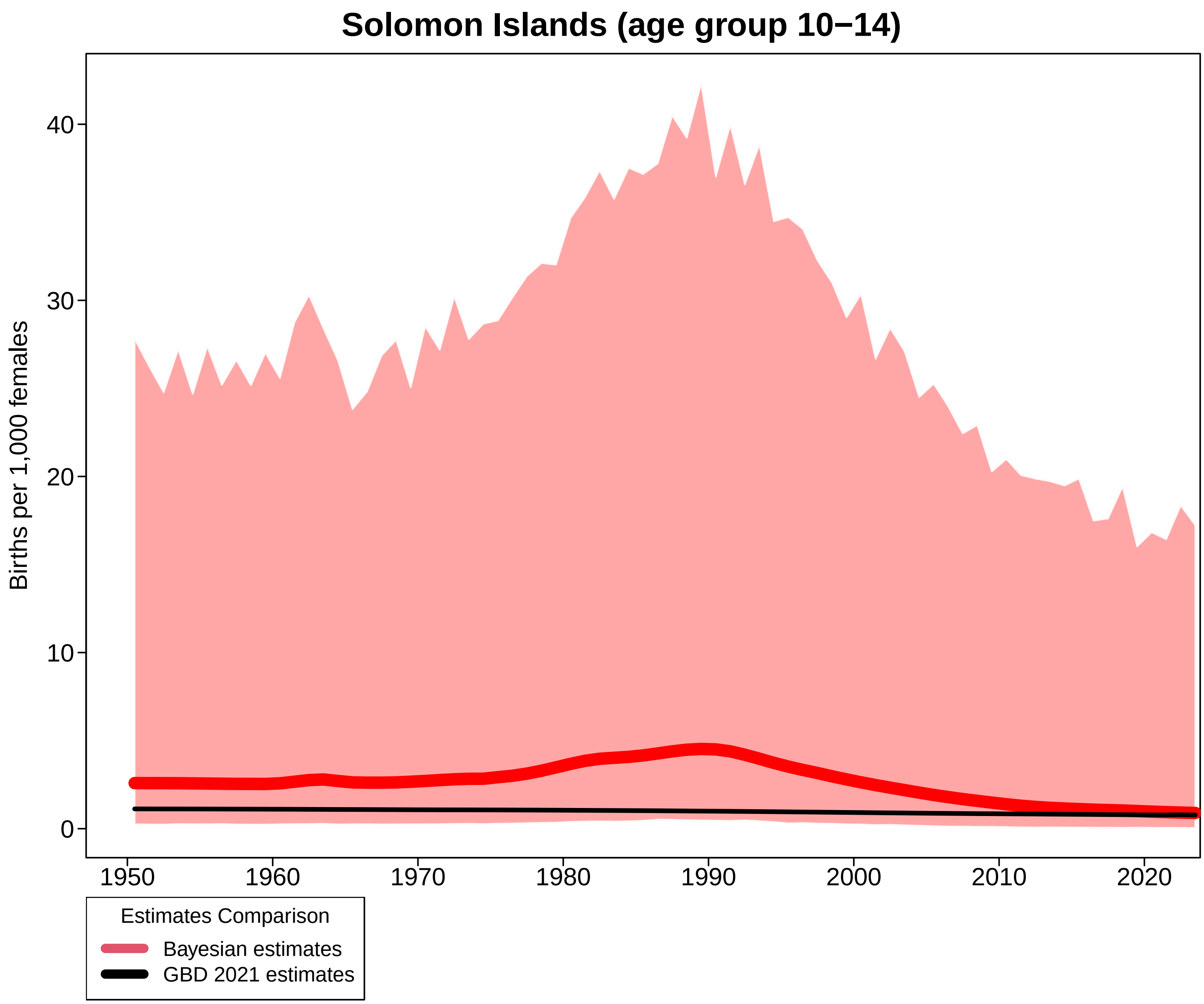

Solomon Islands (age group 10−14)
Births per 1,000 females
0
10
20
30
40
1950
1960
1970
1980
1990
2000
2010
2020
Estimates Comparison
Bayesian estimates
GBD 2021 estimates

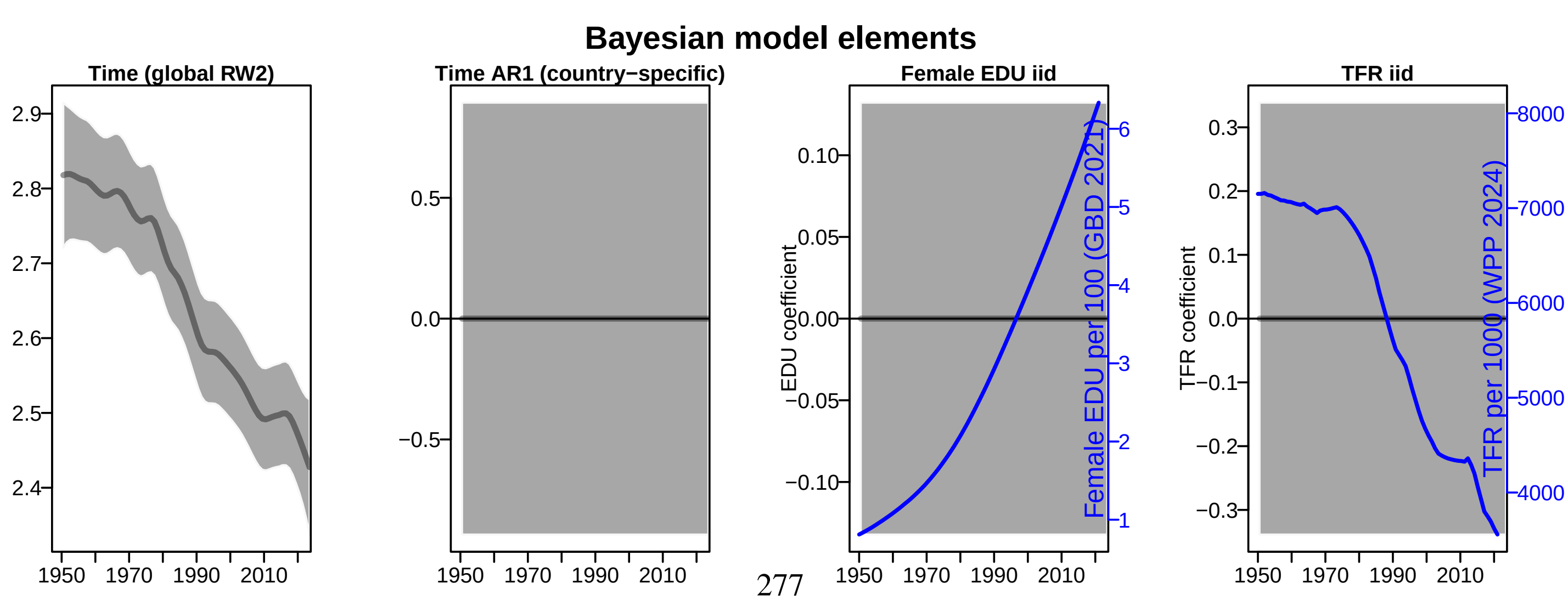

Bayesian model elements
Time (global RW2)
Time AR1 (country−specific)
Female EDU iid
TFR iid
EDU coefficient
Female EDU per 100 (GBD 2021)
TFR coefficient
TFR per 1000 (WPP 2024)
1950
1970
1990
2010

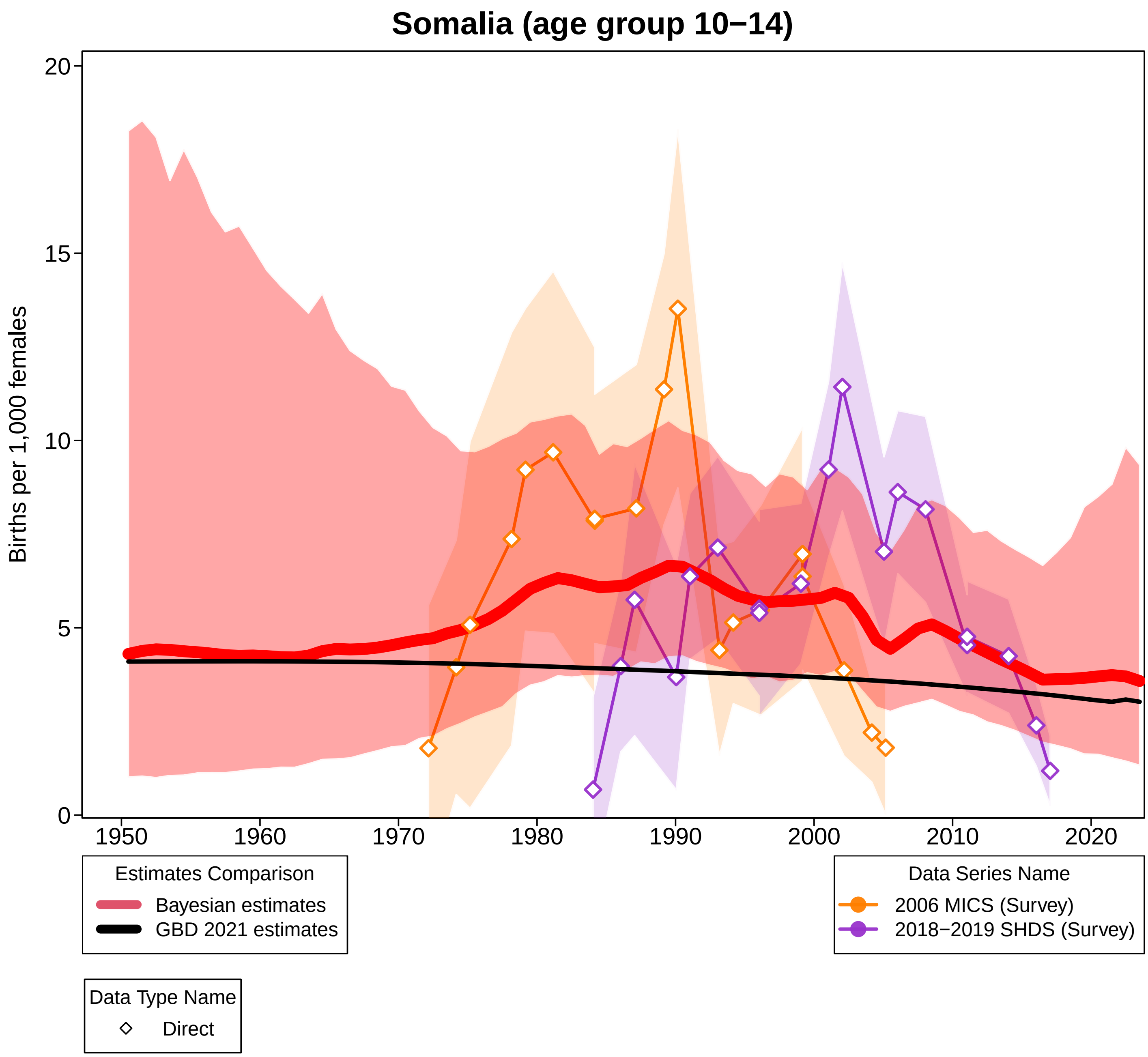
Somalia (age group 10−14)
Births per 1,000 females
0
5
10
15
20
1950
1960
1970
1980
1990
2000
2010
2020
Estimates Comparison
Bayesian estimates
GBD 2021 estimates
Data Series Name
2006 MICS (Survey)
2018−2019 SHDS (Survey)
Data Type Name
Direct

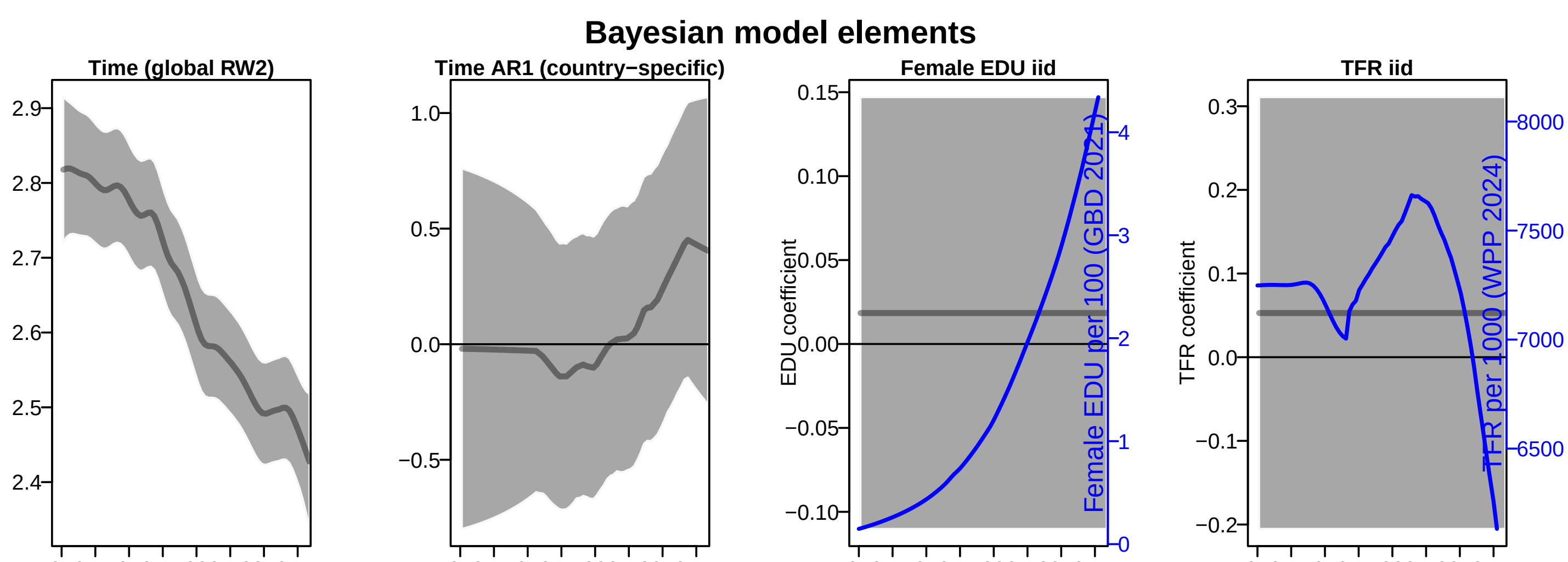
Bayesian model elements
Time (global RW2)
Time AR1 (country−specific)
Female EDU iid
TFR iid
EDU coefficient
Female EDU per 100 (GBD 2021)
TFR coefficient
TFR per 1000 (WPP 2024)
1950
1970
1990
2010

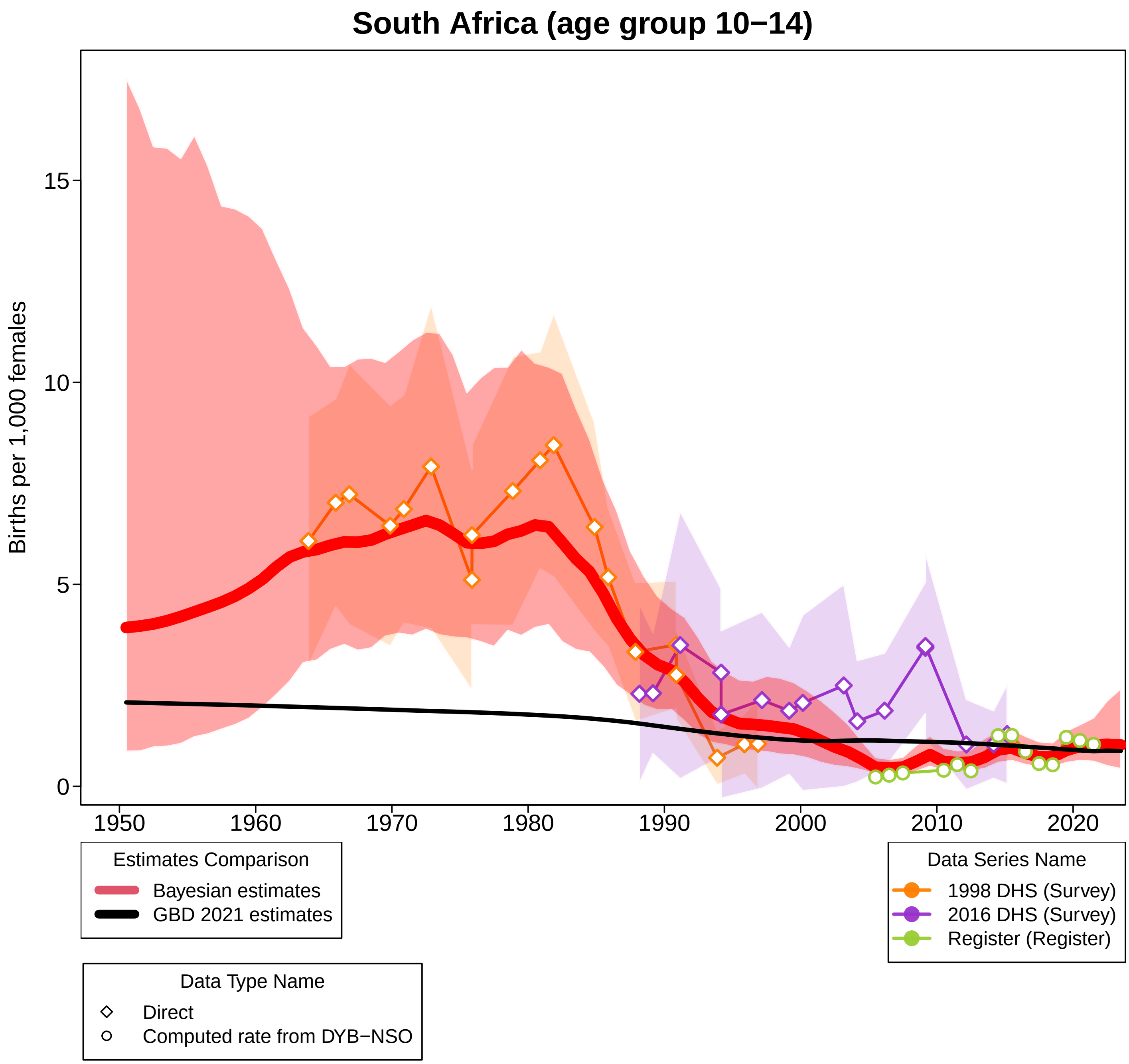
South Africa (age group 10−14)
Births per 1,000 females
15
10
5
0
1950
1960
1970
1980
1990
2000
2010
2020
Estimates Comparison
Bayesian estimates
GBD 2021 estimates
Data Series Name
1998 DHS (Survey)
2016 DHS (Survey)
Register (Register)
Data Type Name
Direct
Computed rate from DYB−NSO

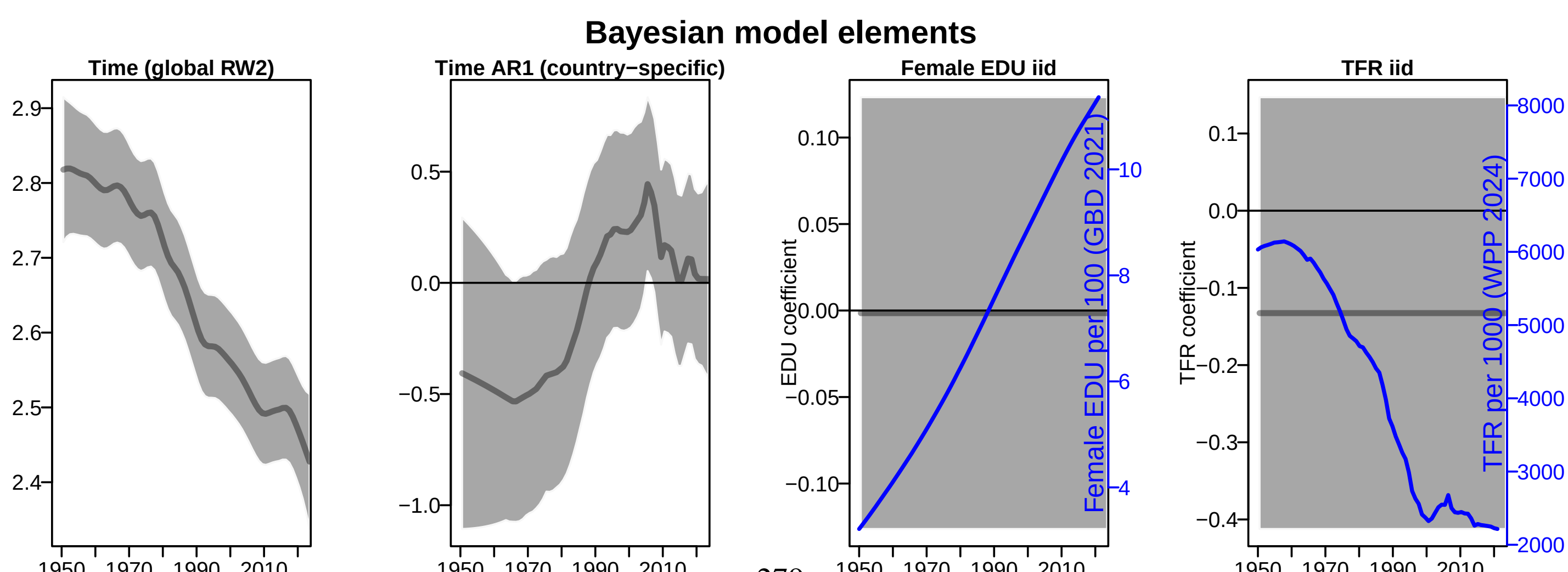
Bayesian model elements
Time (global RW2)
Time AR1 (country−specific)
Female EDU iid
TFR iid
EDU coefficient
Female EDU per 100 (GBD 2021)
TFR coefficient
TFR per 1000 (WPP 2024)
1950
1970
1990
2010

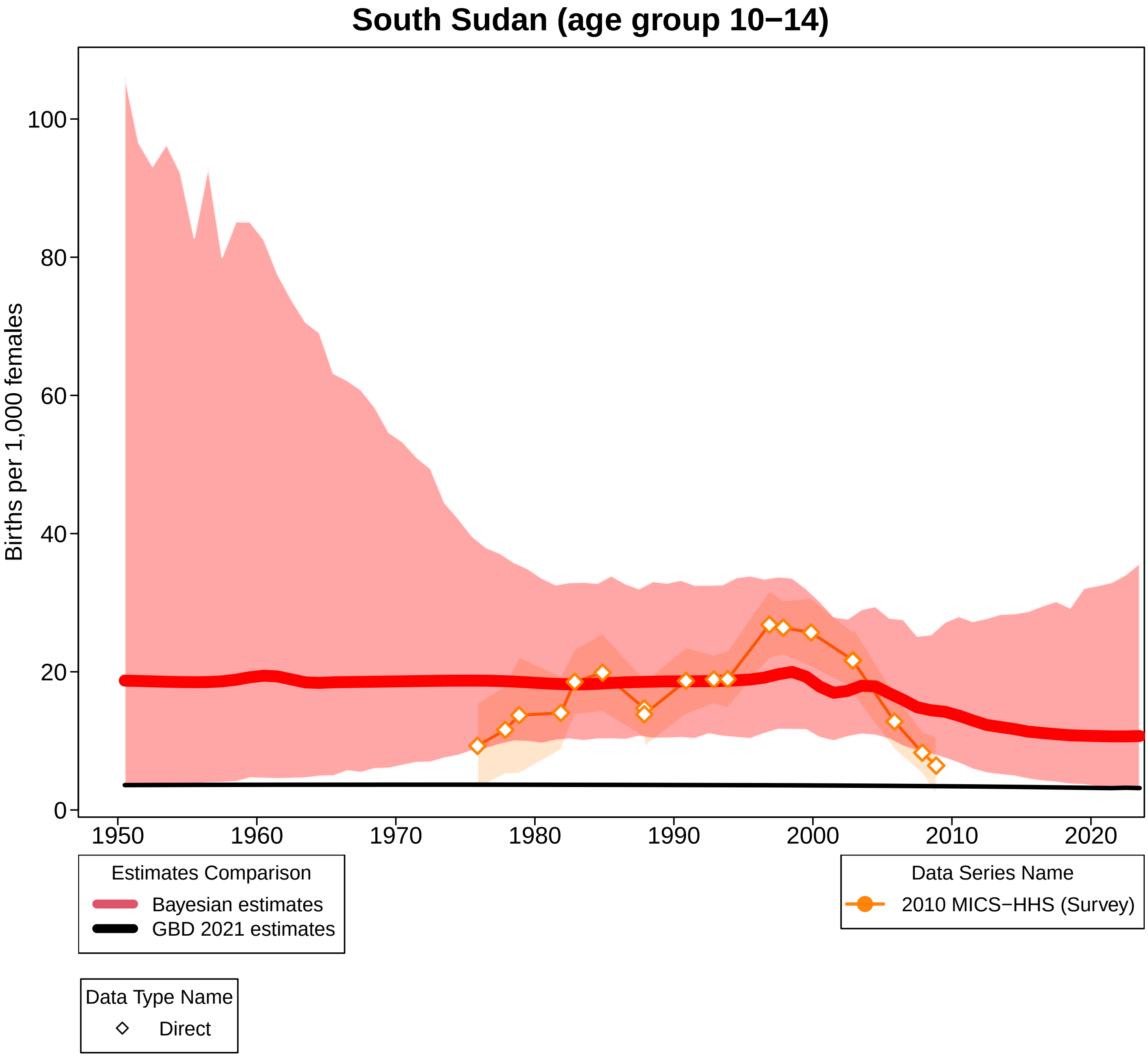

South Sudan (age group 10−14)
Births per 1,000 females
0
20
40
60
80
100
1950
1960
1970
1980
1990
2000
2010
2020
Estimates Comparison
Bayesian estimates
GBD 2021 estimates
Data Series Name
2010 MICS−HHS (Survey)
Data Type Name
Direct

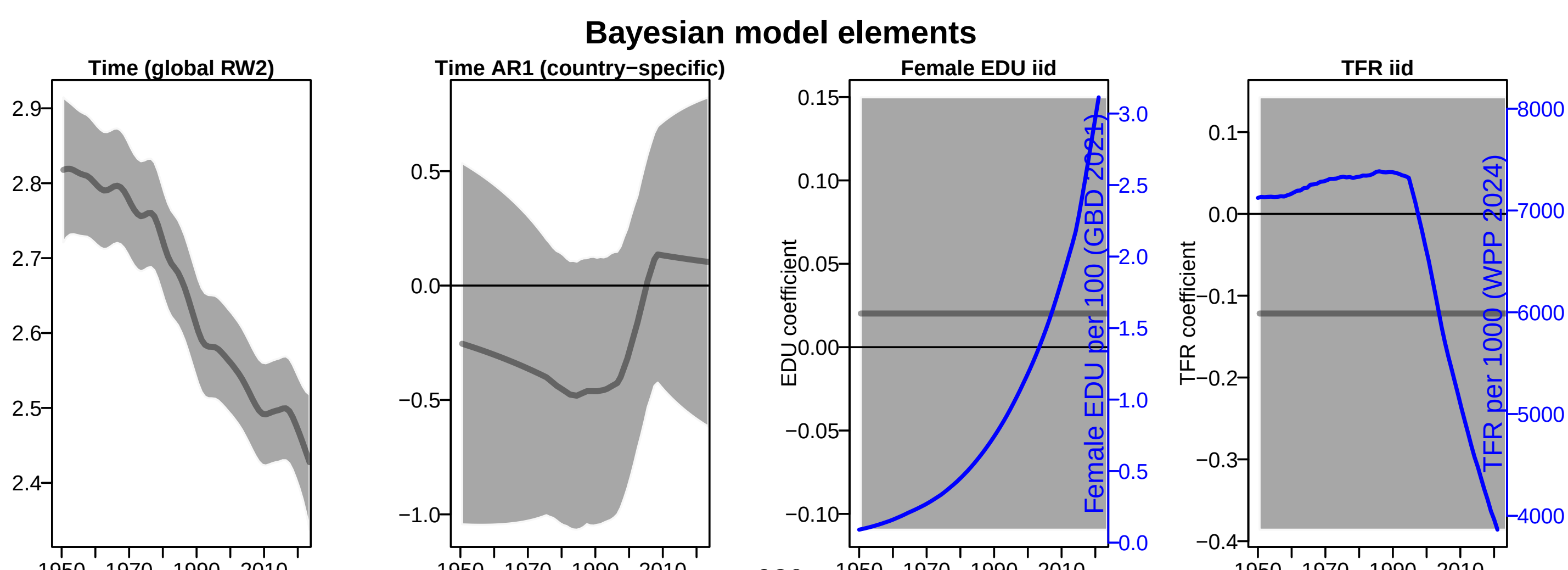

Bayesian model elements
Time (global RW2)
Time AR1 (country−specific)
Female EDU iid
TFR iid
EDU coefficient
Female EDU per 100 (GBD 2021)
TFR coefficient
TFR per 1000 (WPP 2024)
1950
1970
1990
2010

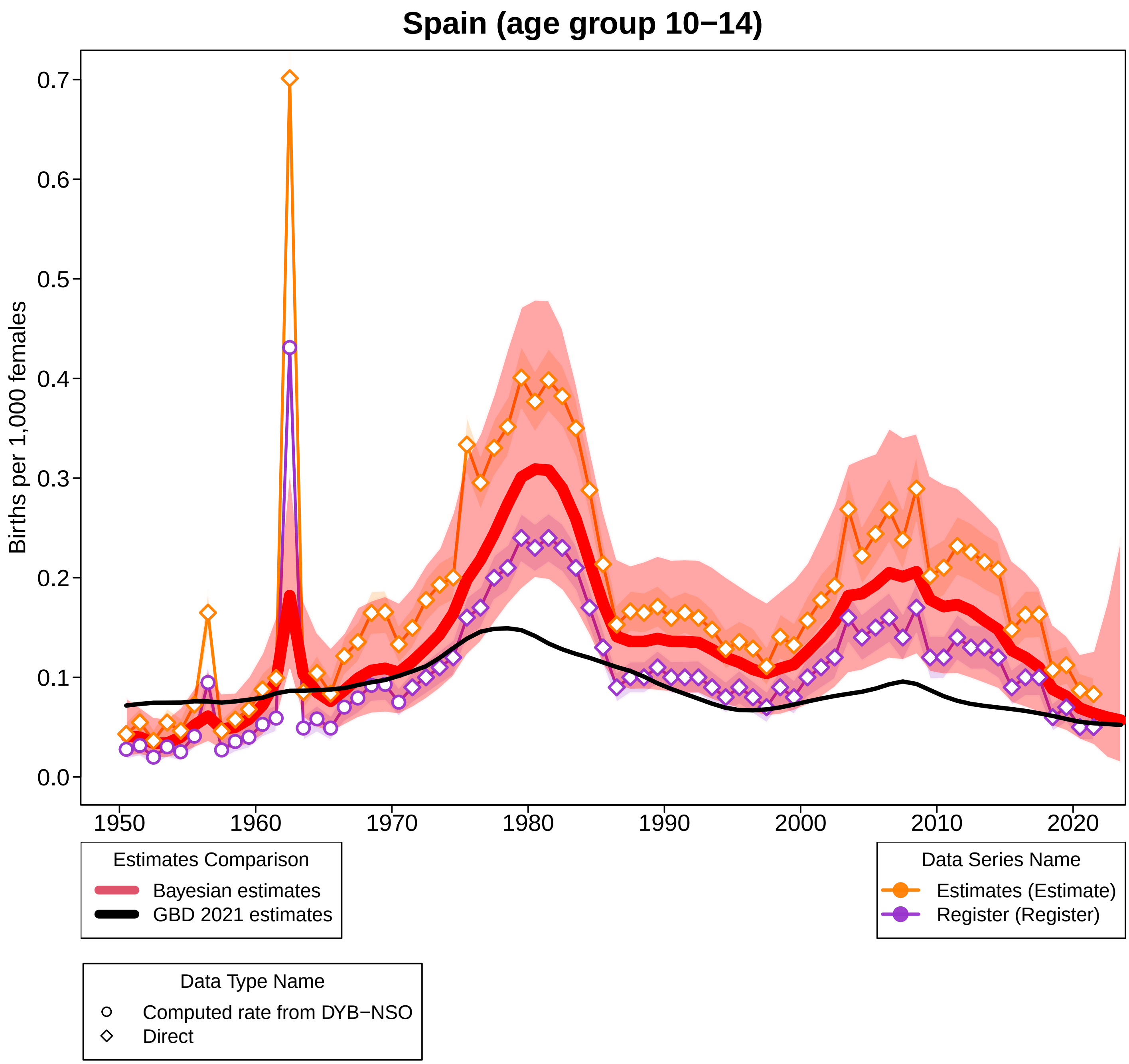
Spain (age group 10−14)
Births per 1,000 females
0.0
0.1
0.2
0.3
0.4
0.5
0.6
0.7
1950
1960
1970
1980
1990
2000
2010
2020
Estimates Comparison
Bayesian estimates
GBD 2021 estimates
Data Series Name
Estimates (Estimate)
Register (Register)
Data Type Name
Computed rate from DYB−NSO
Direct

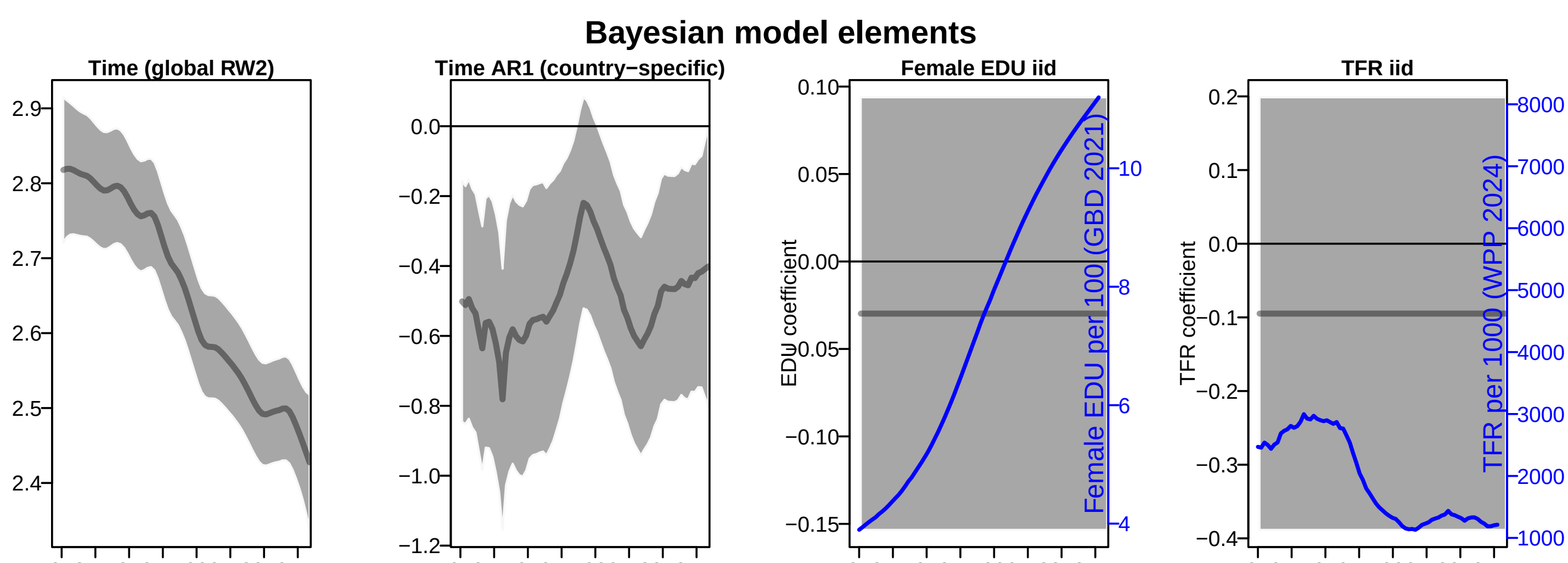
Bayesian model elements
Time (global RW2)
Time AR1 (country−specific)
Female EDU iid
TFR iid
EDU coefficient
Female EDU per 100 (GBD 2021)
TFR coefficient
TFR per 1000 (WPP 2024)
1950
1970
1990
2010

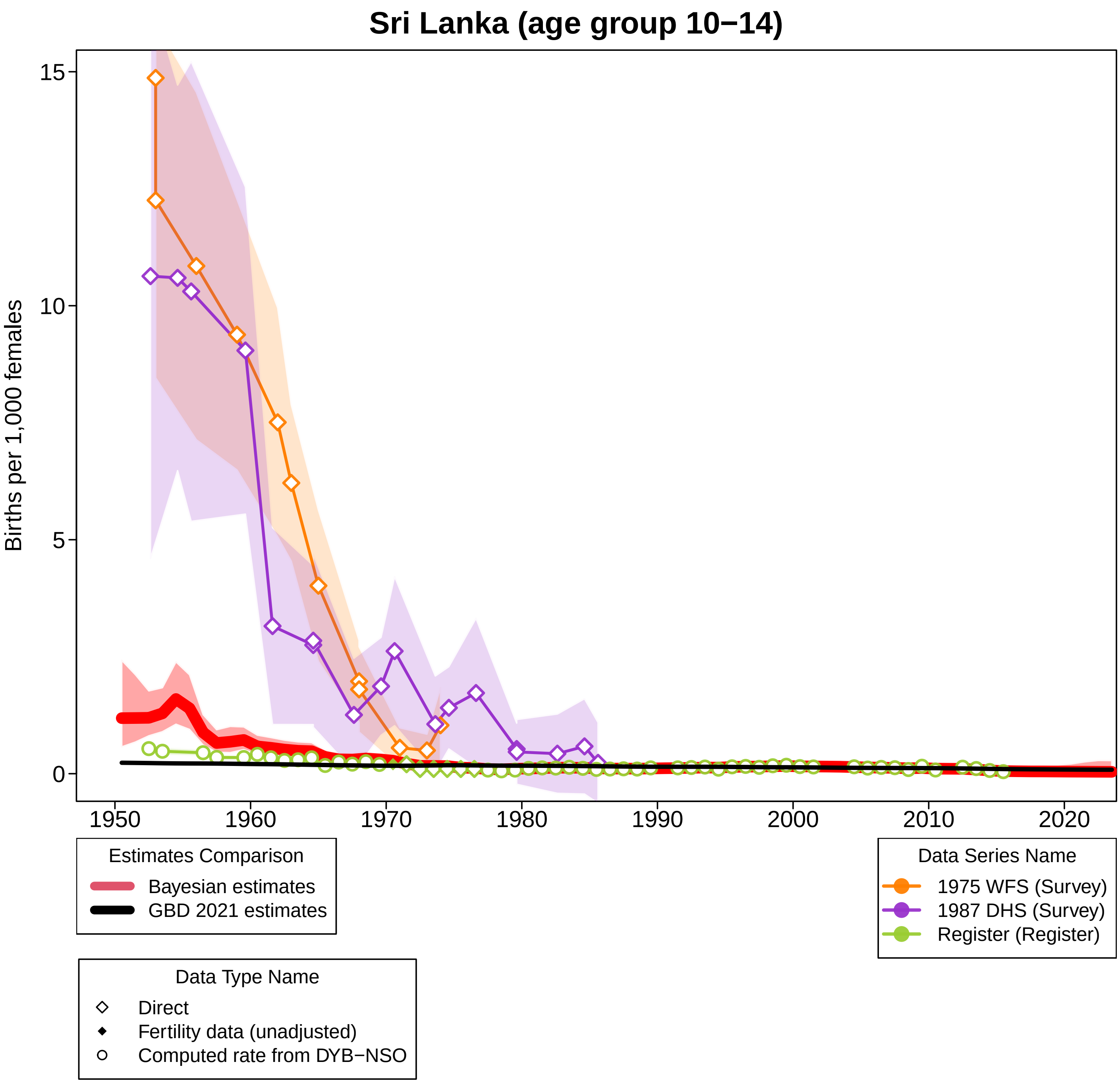

Sri Lanka (age group 10−14)
Births per 1,000 females
15
10
5
0
1950
1960
1970
1980
1990
2000
2010
2020
Estimates Comparison
Bayesian estimates
GBD 2021 estimates
Data Series Name
1975 WFS (Survey)
1987 DHS (Survey)
Register (Register)
Data Type Name
Direct
Fertility data (unadjusted)
Computed rate from DYB−NSO

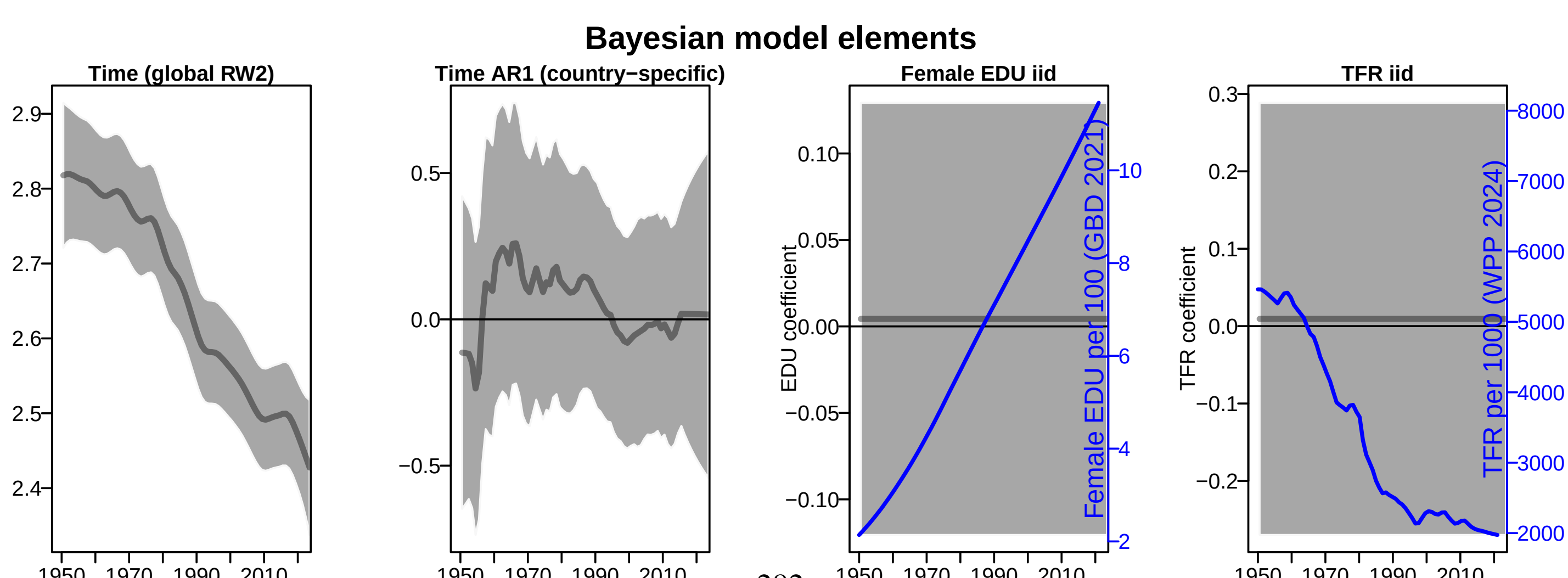

Bayesian model elements
Time (global RW2)
Time AR1 (country−specific)
Female EDU iid
TFR iid
EDU coefficient
Female EDU per 100 (GBD 2021)
TFR coefficient
TFR per 1000 (WPP 2024)

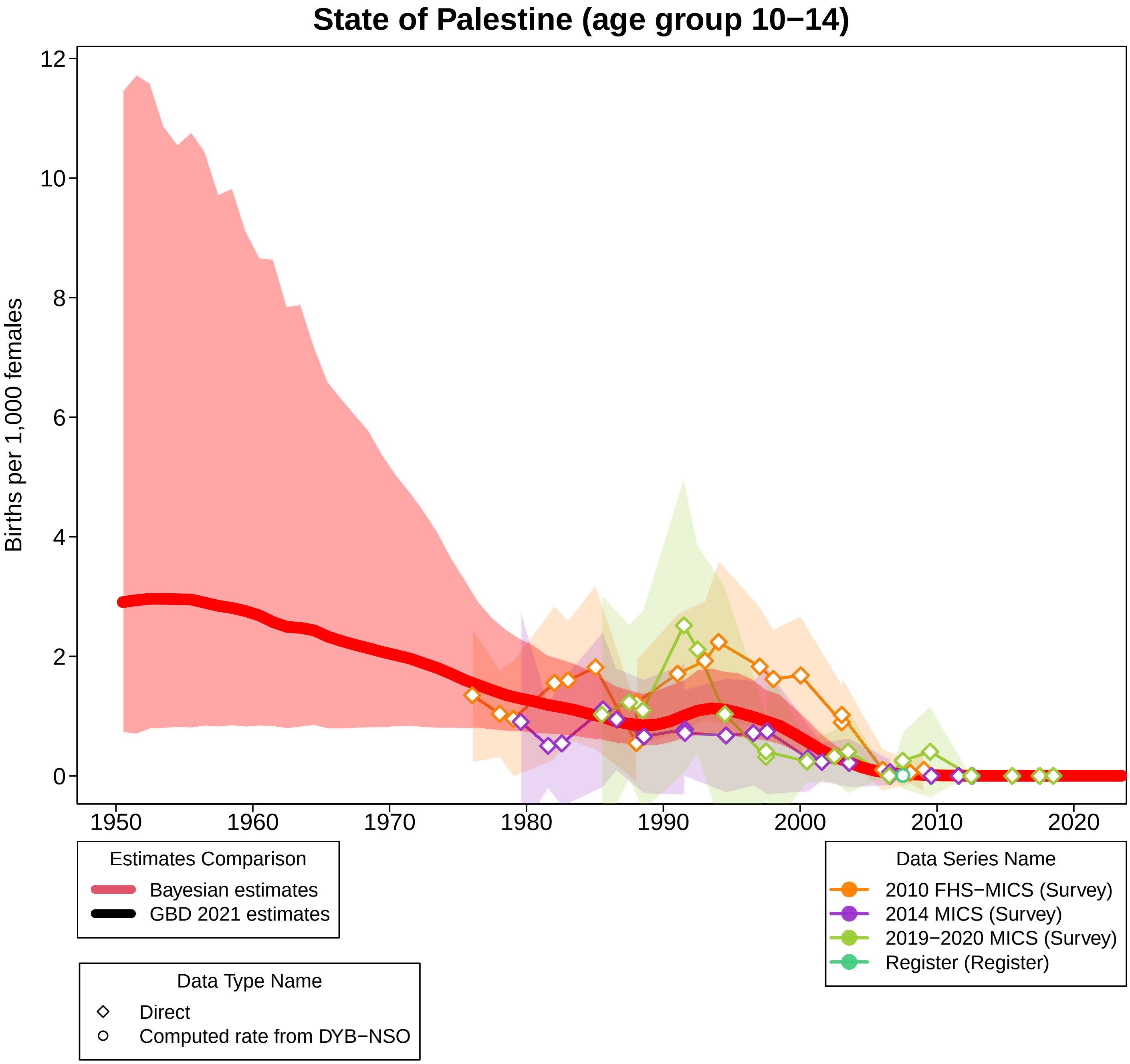
State of Palestine (age group 10−14)
Births per 1,000 females
0
2
4
6
8
10
12
1950
1960
1970
1980
1990
2000
2010
2020
Estimates Comparison
Bayesian estimates
GBD 2021 estimates
Data Type Name
Direct
Computed rate from DYB−NSO
Data Series Name
2010 FHS−MICS (Survey)
2014 MICS (Survey)
2019−2020 MICS (Survey)
Register (Register)

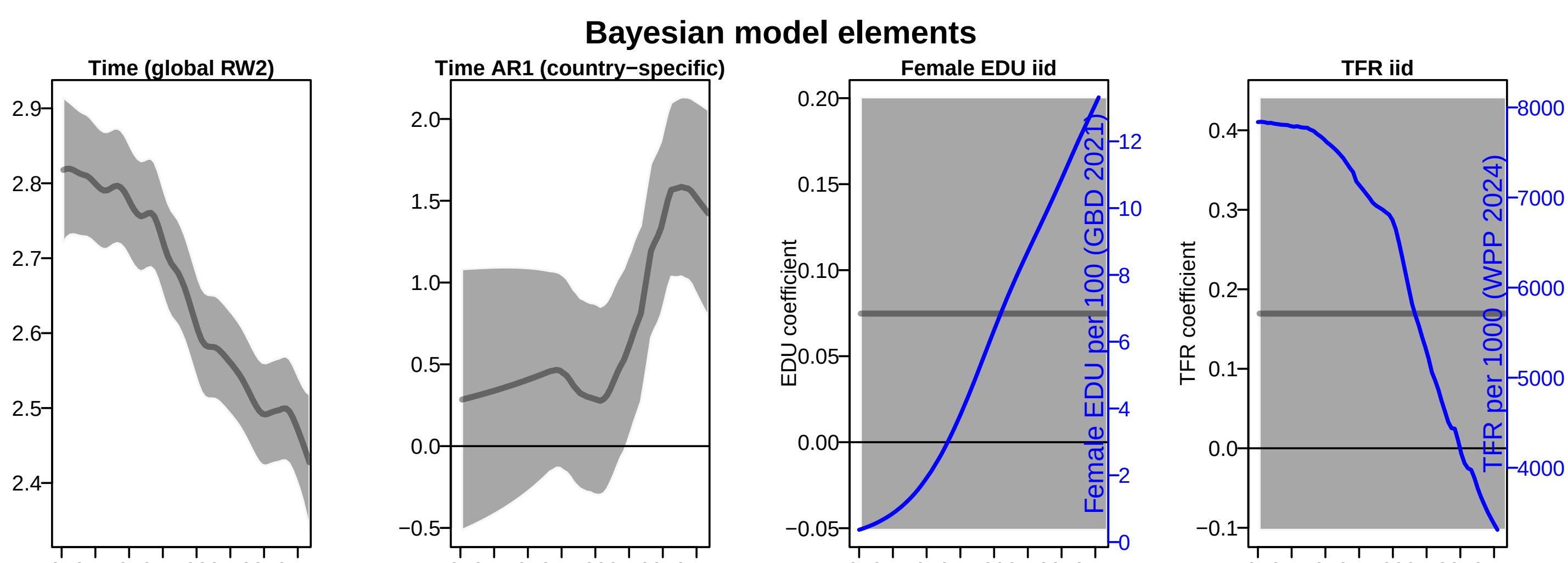
Bayesian model elements
Time (global RW2)
Time AR1 (country−specific)
Female EDU iid
TFR iid
EDU coefficient
Female EDU per 100 (GBD 2021)
TFR coefficient
TFR per 1000 (WPP 2024)
1950
1970
1990
2010

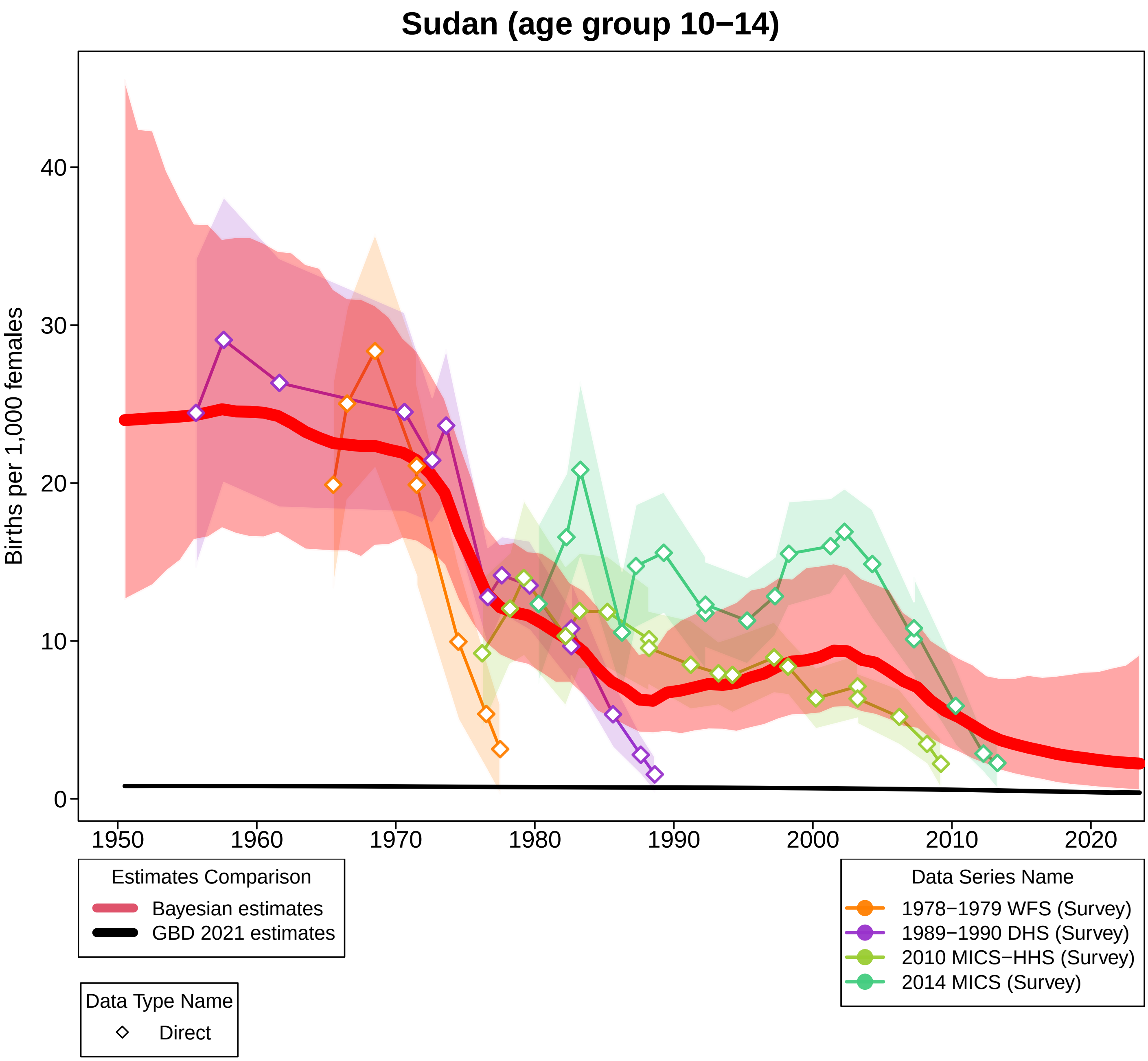
Sudan (age group 10−14)
Births per 1,000 females
0
10
20
30
40
1950
1960
1970
1980
1990
2000
2010
2020
Estimates Comparison
Bayesian estimates
GBD 2021 estimates
Data Series Name
1978−1979 WFS (Survey)
1989−1990 DHS (Survey)
2010 MICS−HHS (Survey)
2014 MICS (Survey)
Data Type Name
Direct

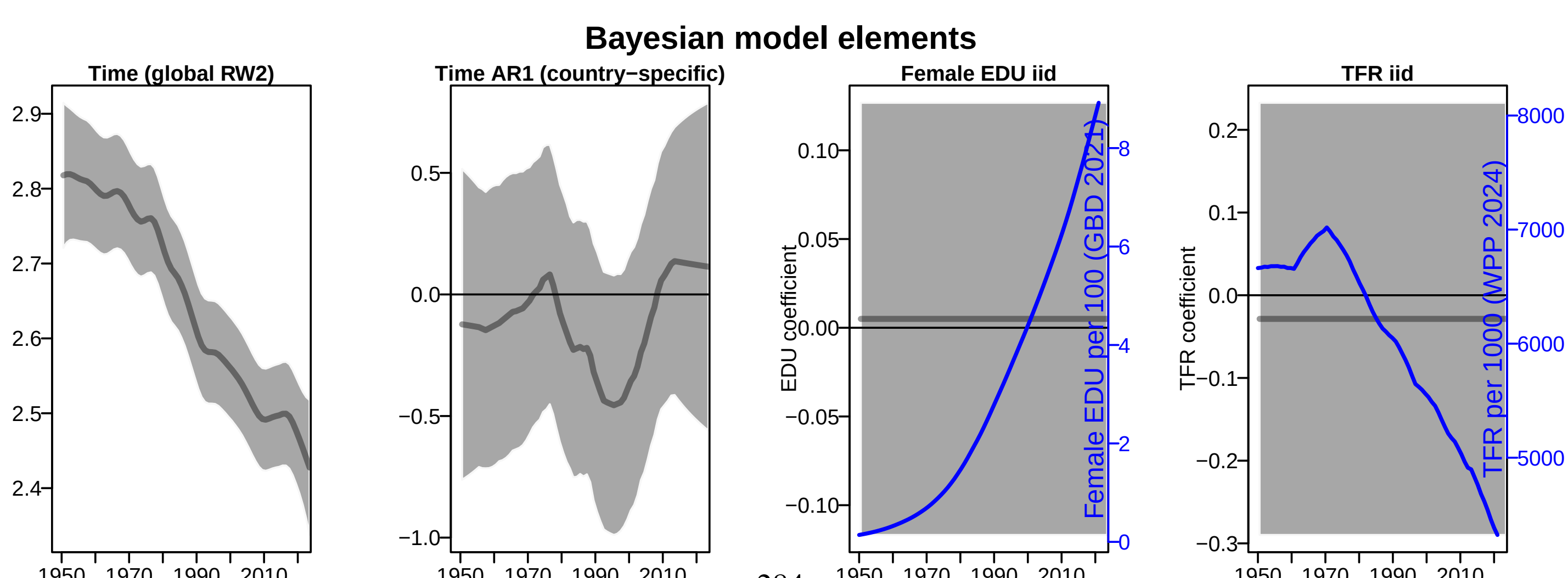
Bayesian model elements
Time (global RW2)
Time AR1 (country−specific)
Female EDU iid
TFR iid
EDU coefficient
Female EDU per 100 (GBD 2021)
TFR coefficient
TFR per 1000 (WPP 2024)
1950
1970
1990
2010

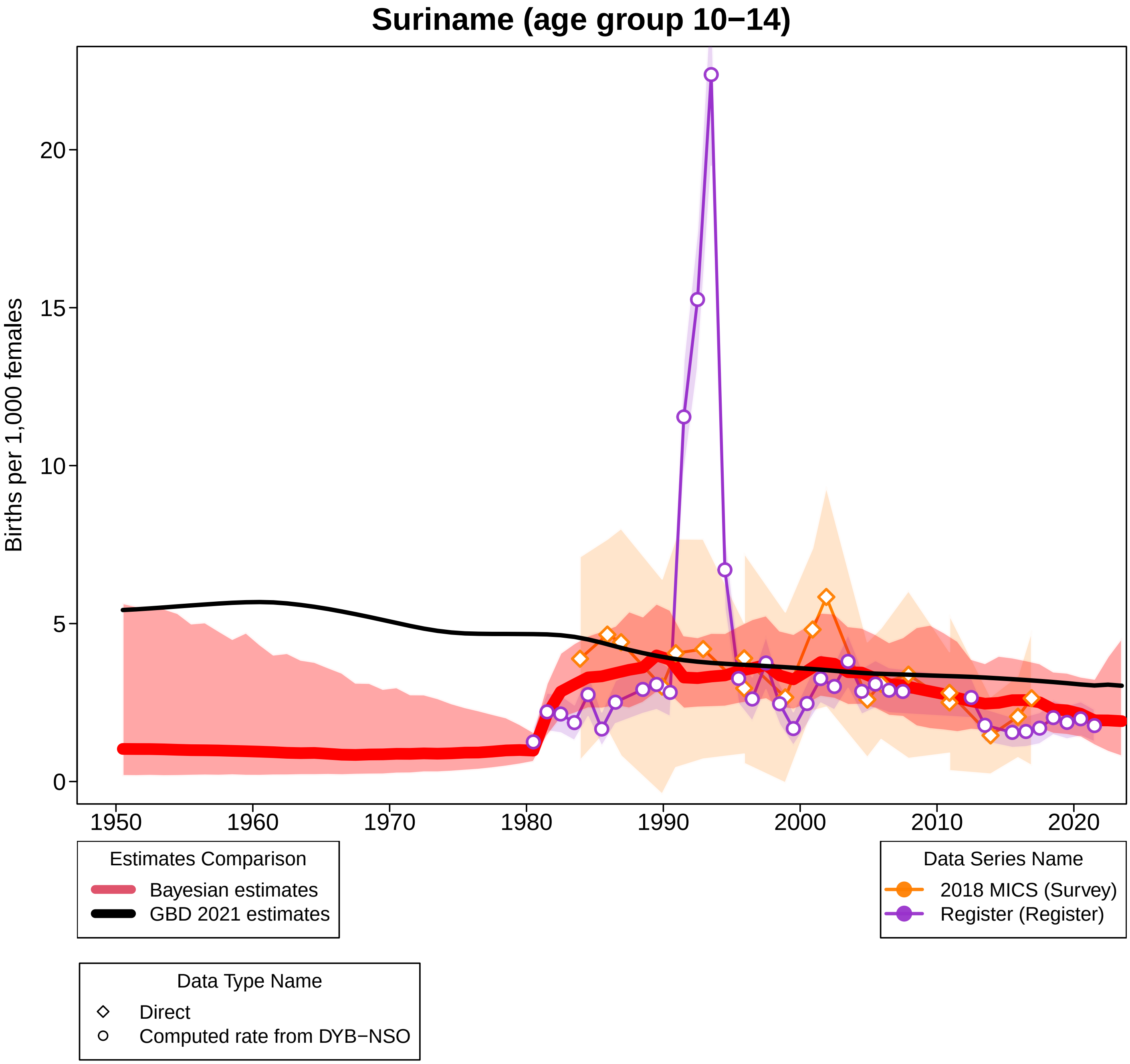
Suriname (age group 10−14)
Births per 1,000 females
0
5
10
15
20
1950
1960
1970
1980
1990
2000
2010
2020
Estimates Comparison
Bayesian estimates
GBD 2021 estimates
Data Series Name
2018 MICS (Survey)
Register (Register)
Data Type Name
Direct
Computed rate from DYB−NSO

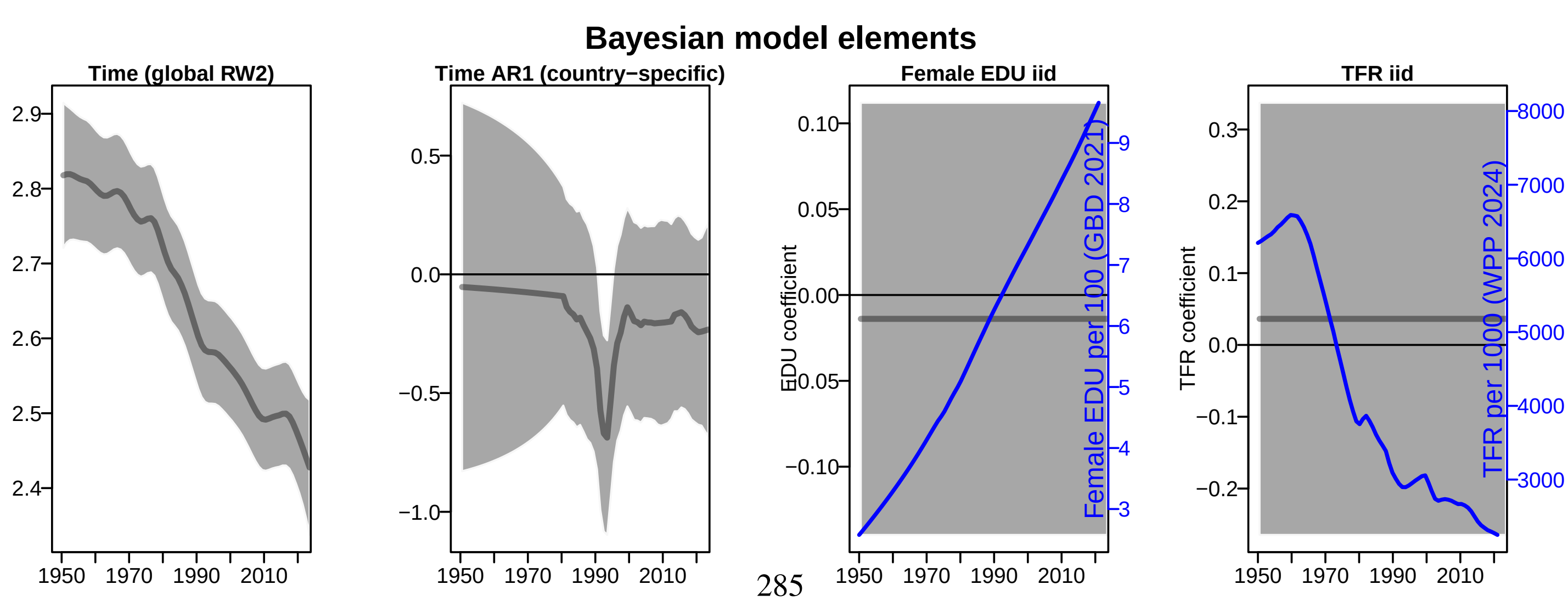
Bayesian model elements
Time (global RW2)
Time AR1 (country−specific)
Female EDU iid
TFR iid
EDU coefficient
Female EDU per 100 (GBD 2021)
TFR coefficient
TFR per 1000 (WPP 2024)
1950
1970
1990
2010

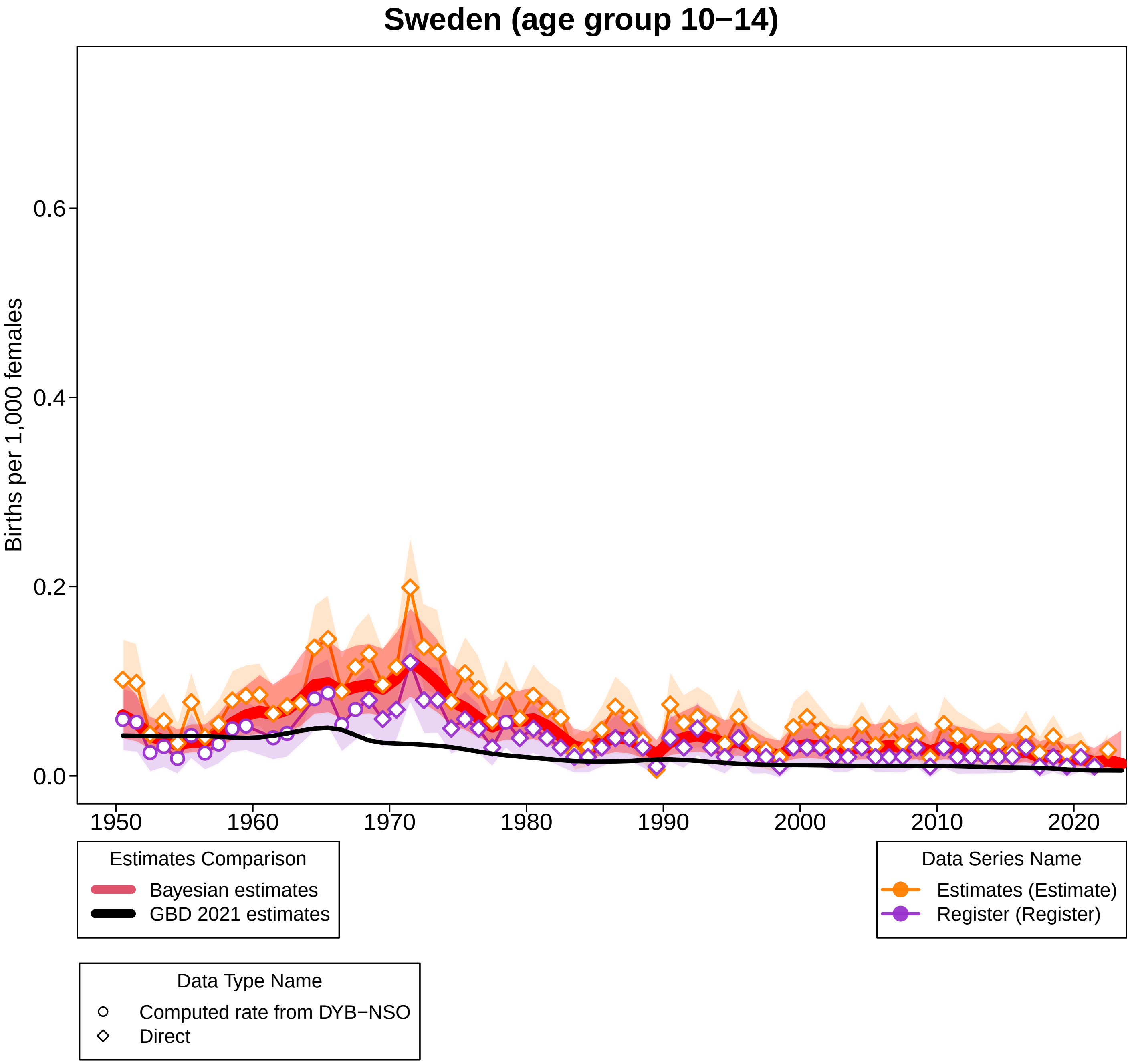
Sweden (age group 10−14)
Births per 1,000 females
0.0
0.2
0.4
0.6
1950
1960
1970
1980
1990
2000
2010
2020
Estimates Comparison
Bayesian estimates
GBD 2021 estimates
Data Series Name
Estimates (Estimate)
Register (Register)
Data Type Name
Computed rate from DYB−NSO
Direct

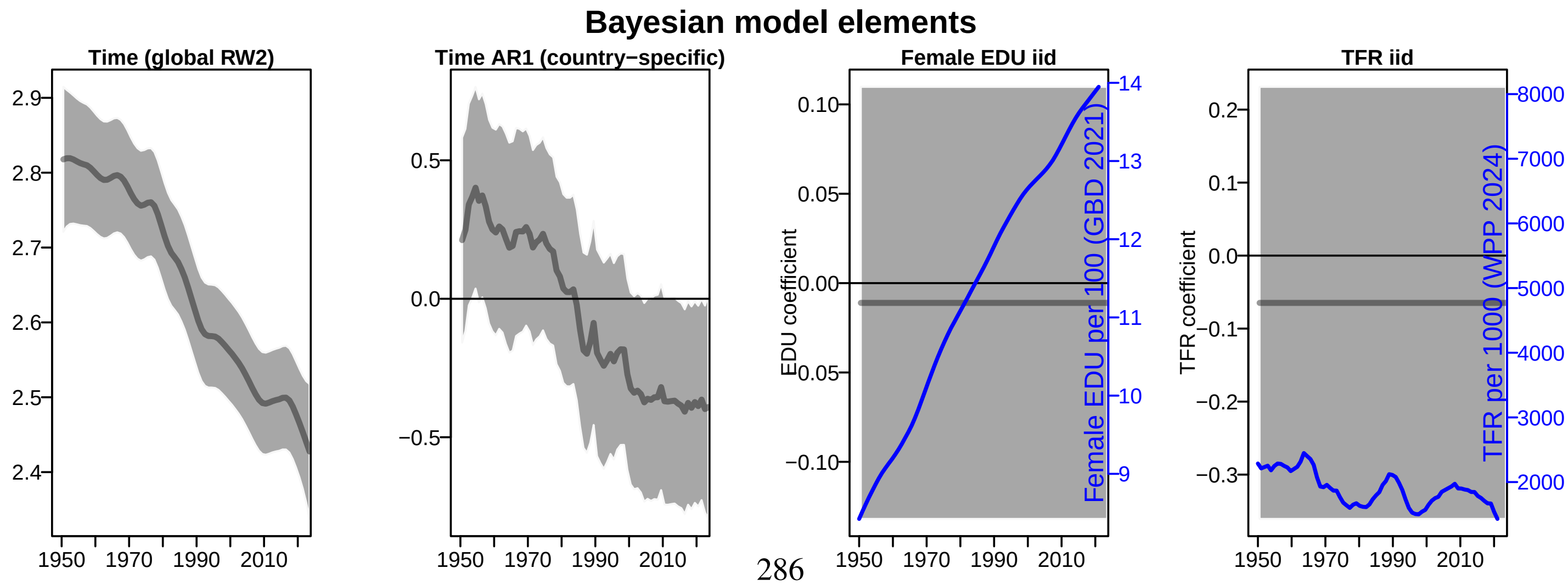
Bayesian model elements
Time (global RW2)
Time AR1 (country−specific)
Female EDU iid
TFR iid
EDU coefficient
Female EDU per 100 (GBD 2021)
TFR coefficient
TFR per 1000 (WPP 2024)
1950
1970
1990
2010

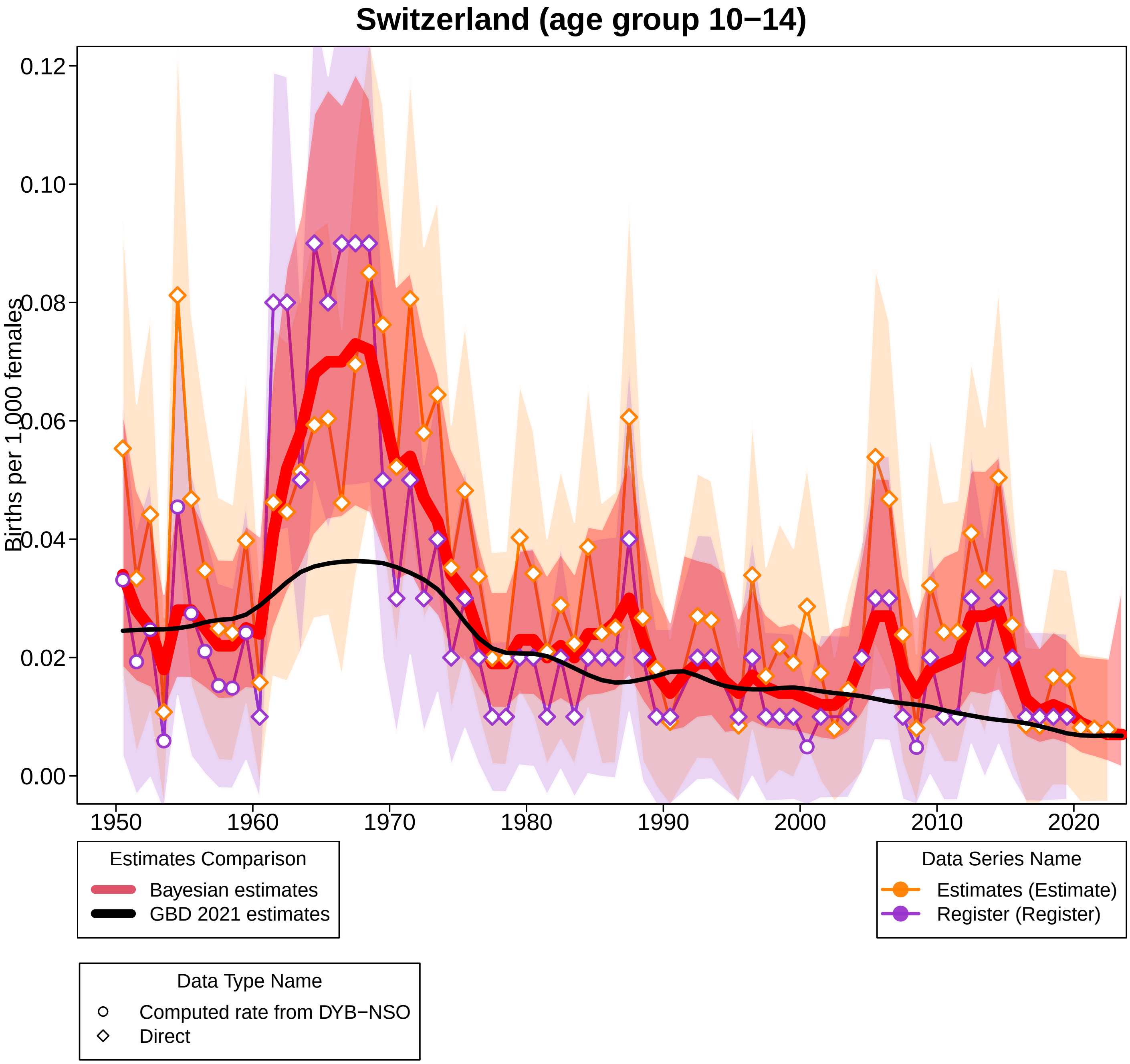

Switzerland (age group 10−14)
Births per 1,000 females
0.12
0.10
0.08
0.06
0.04
0.02
0.00
1950
1960
1970
1980
1990
2000
2010
2020
Estimates Comparison
Bayesian estimates
GBD 2021 estimates
Data Series Name
Estimates (Estimate)
Register (Register)
Data Type Name
Computed rate from DYB−NSO
Direct

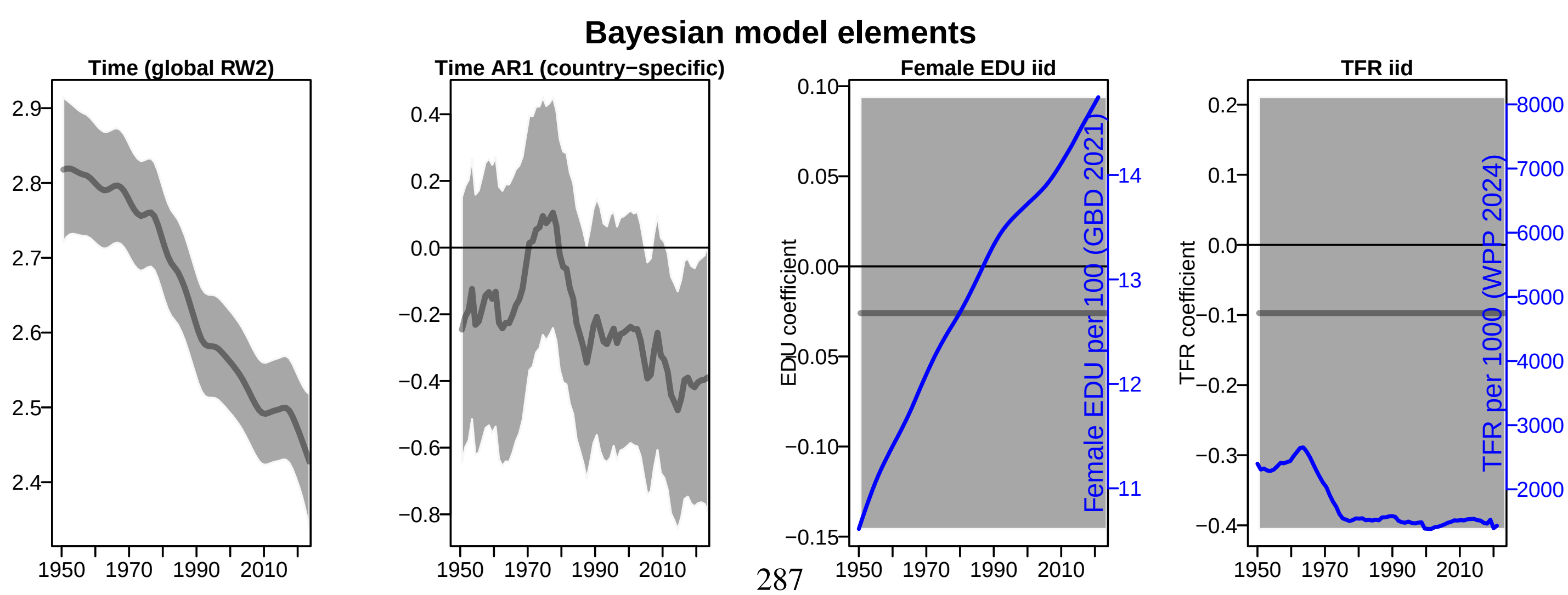

Bayesian model elements
Time (global RW2)
Time AR1 (country−specific)
Female EDU iid
TFR iid
EDU coefficient
Female EDU per 100 (GBD 2021)
TFR coefficient
TFR per 1000 (WPP 2024)

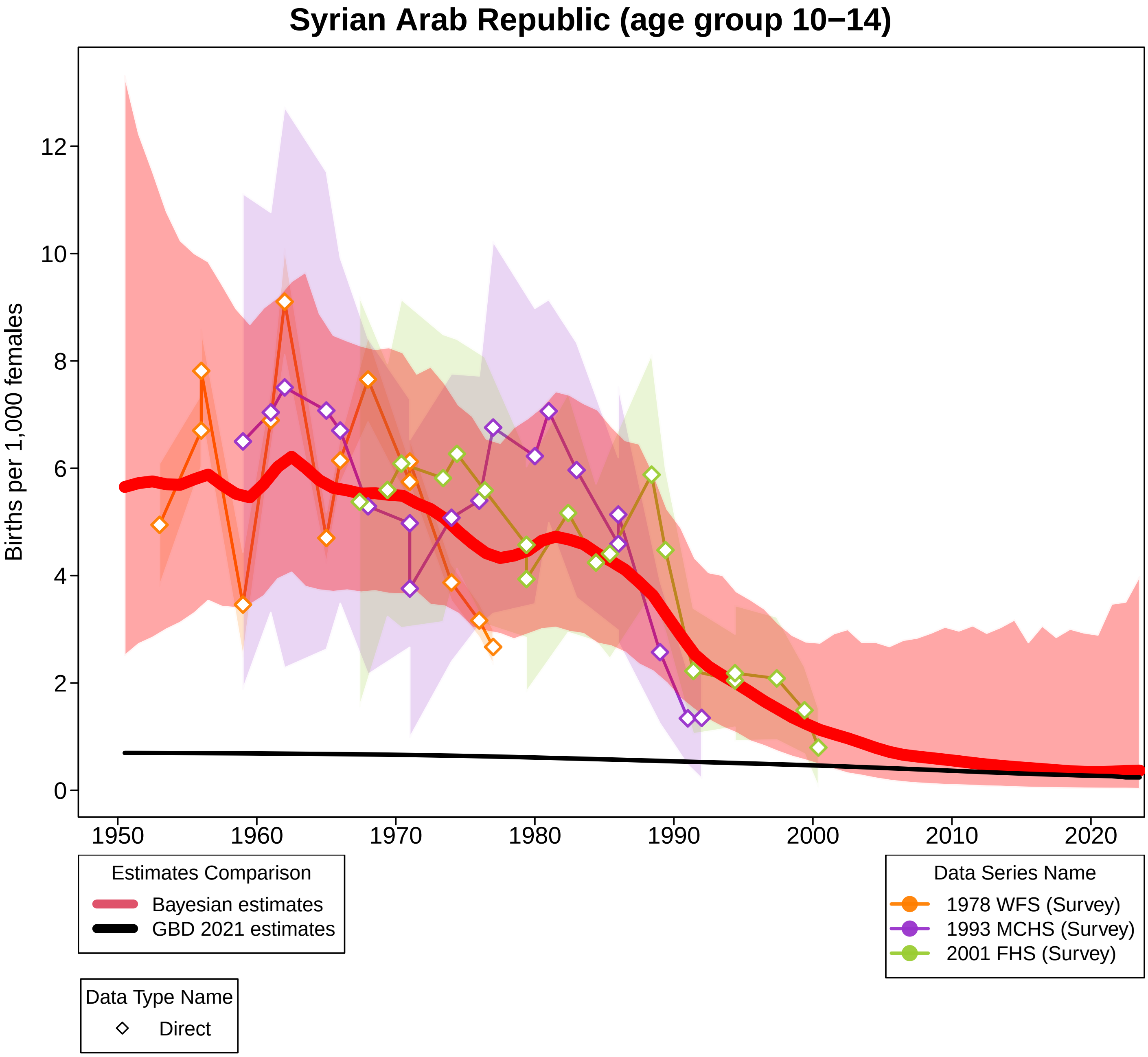

Syrian Arab Republic (age group 10−14)
Births per 1,000 females
0
2
4
6
8
10
12
1950
1960
1970
1980
1990
2000
2010
2020
Estimates Comparison
Bayesian estimates
GBD 2021 estimates
Data Series Name
1978 WFS (Survey)
1993 MCHS (Survey)
2001 FHS (Survey)
Data Type Name
Direct

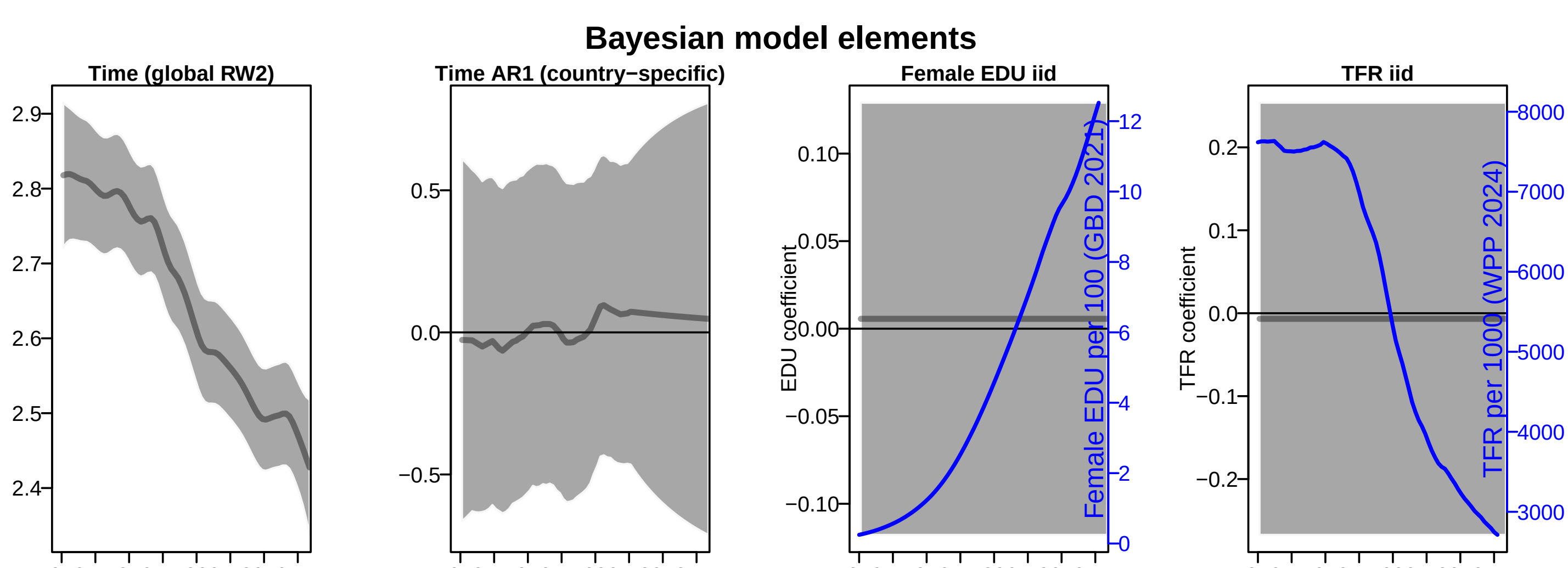

Bayesian model elements
Time (global RW2)
Time AR1 (country−specific)
Female EDU iid
TFR iid
EDU coefficient
Female EDU per 100 (GBD 2021)
TFR coefficient
TFR per 1000 (WPP 2024)
1950
1970
1990
2010

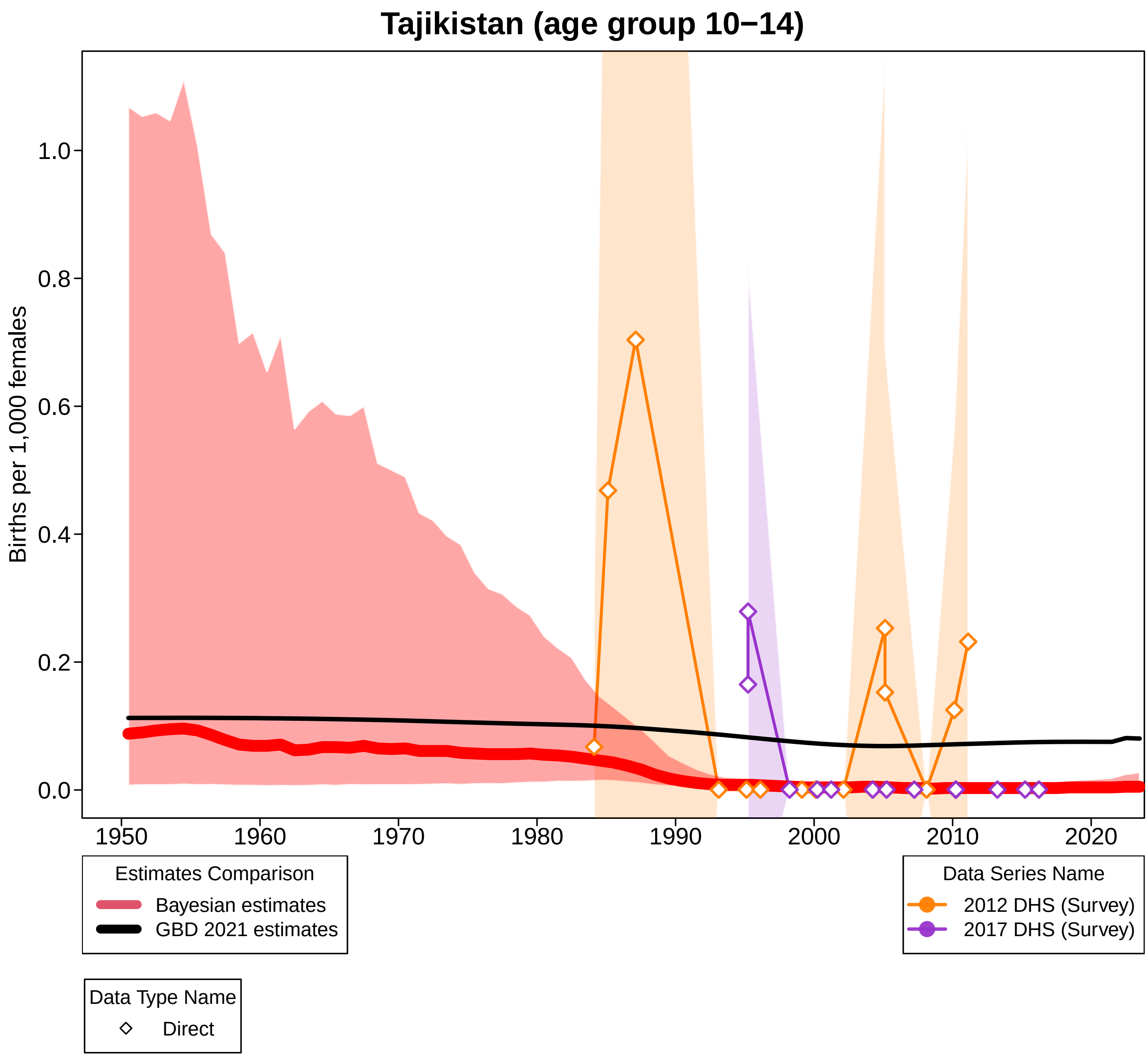
Tajikistan (age group 10−14)
Births per 1,000 females
1.0
0.8
0.6
0.4
0.2
0.0
1950
1960
1970
1980
1990
2000
2010
2020
Estimates Comparison
Bayesian estimates
GBD 2021 estimates
Data Series Name
2012 DHS (Survey)
2017 DHS (Survey)
Data Type Name
Direct

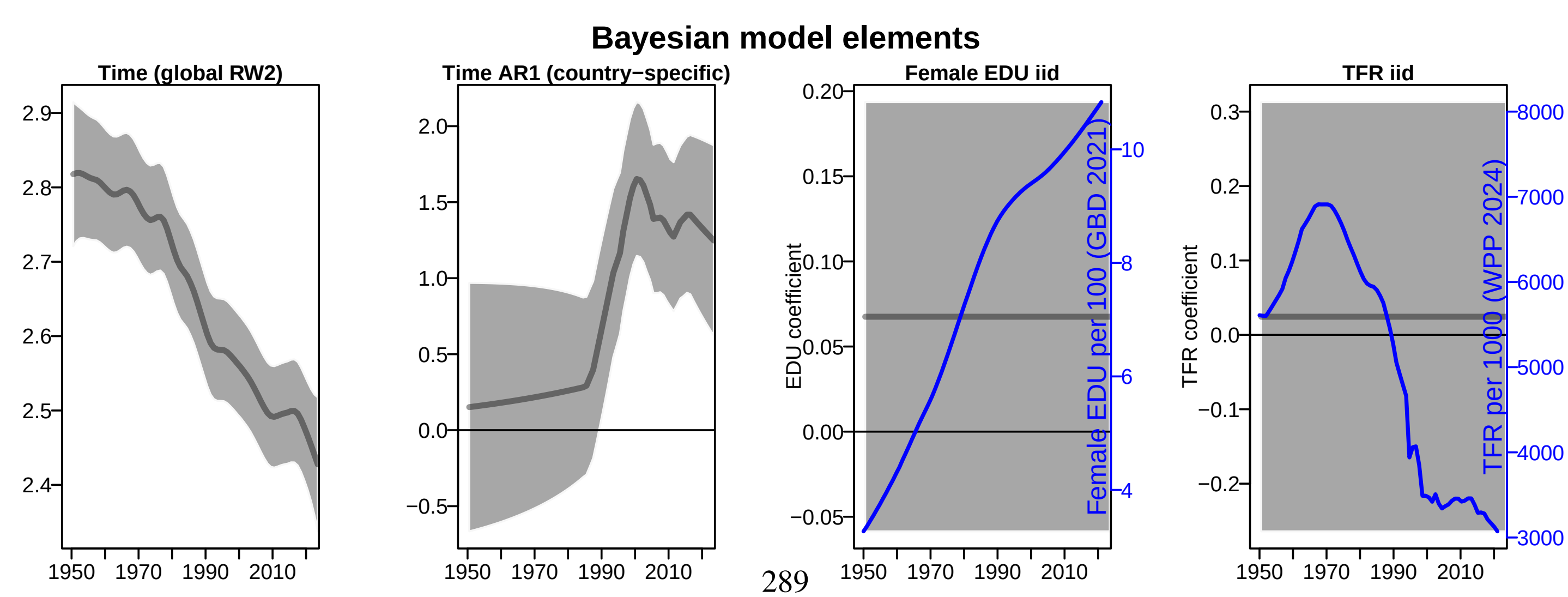
Bayesian model elements
Time (global RW2)
Time AR1 (country−specific)
Female EDU iid
TFR iid
EDU coefficient
Female EDU per 100 (GBD 2021)
TFR coefficient
TFR per 1000 (WPP 2024)
1950
1970
1990
2010

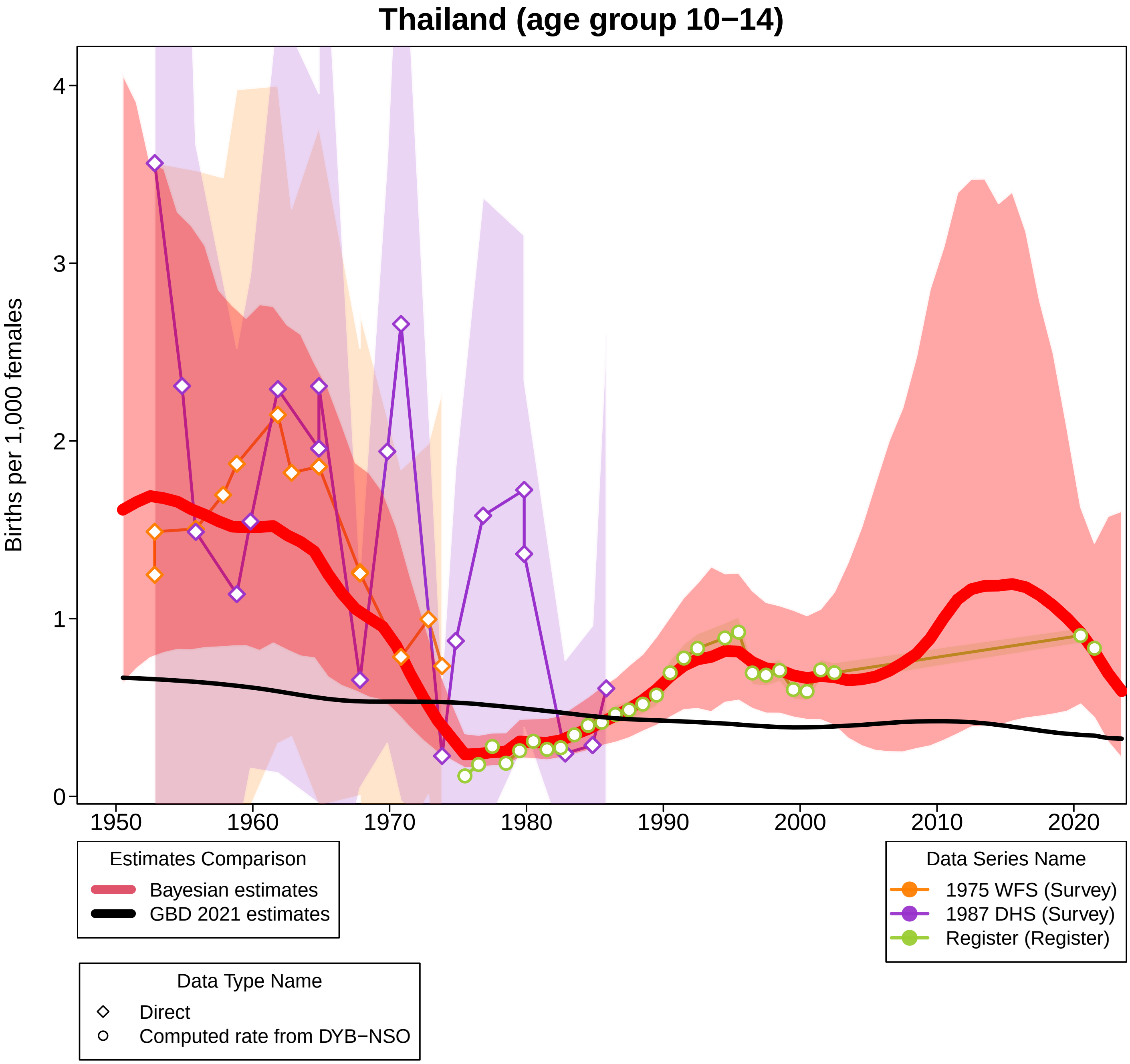
Thailand (age group 10−14)
Births per 1,000 females
0
1
2
3
4
1950
1960
1970
1980
1990
2000
2010
2020
Estimates Comparison
Bayesian estimates
GBD 2021 estimates
Data Series Name
1975 WFS (Survey)
1987 DHS (Survey)
Register (Register)
Data Type Name
Direct
Computed rate from DYB−NSO

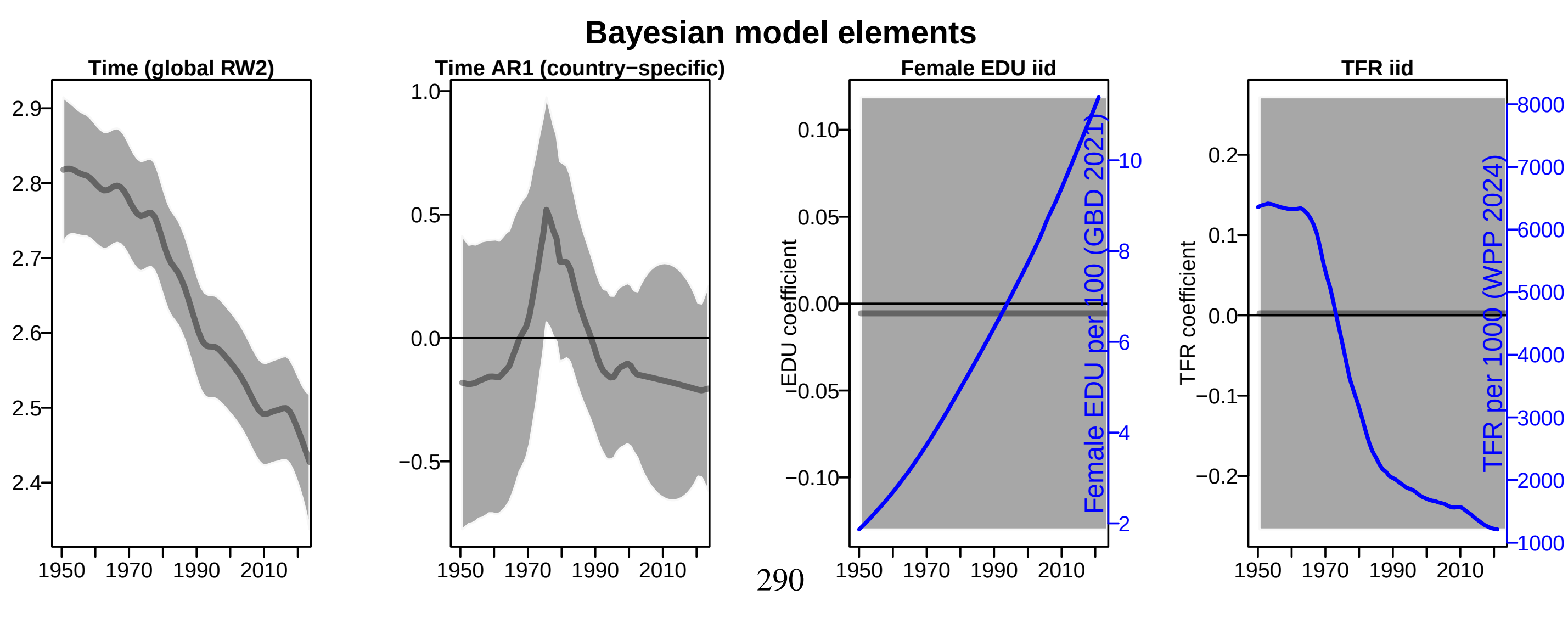
Bayesian model elements
Time (global RW2)
Time AR1 (country−specific)
Female EDU iid
TFR iid
EDU coefficient
TFR coefficient
Female EDU per 100 (GBD 2021)
TFR per 1000 (WPP 2024)
1950
1970
1990
2010

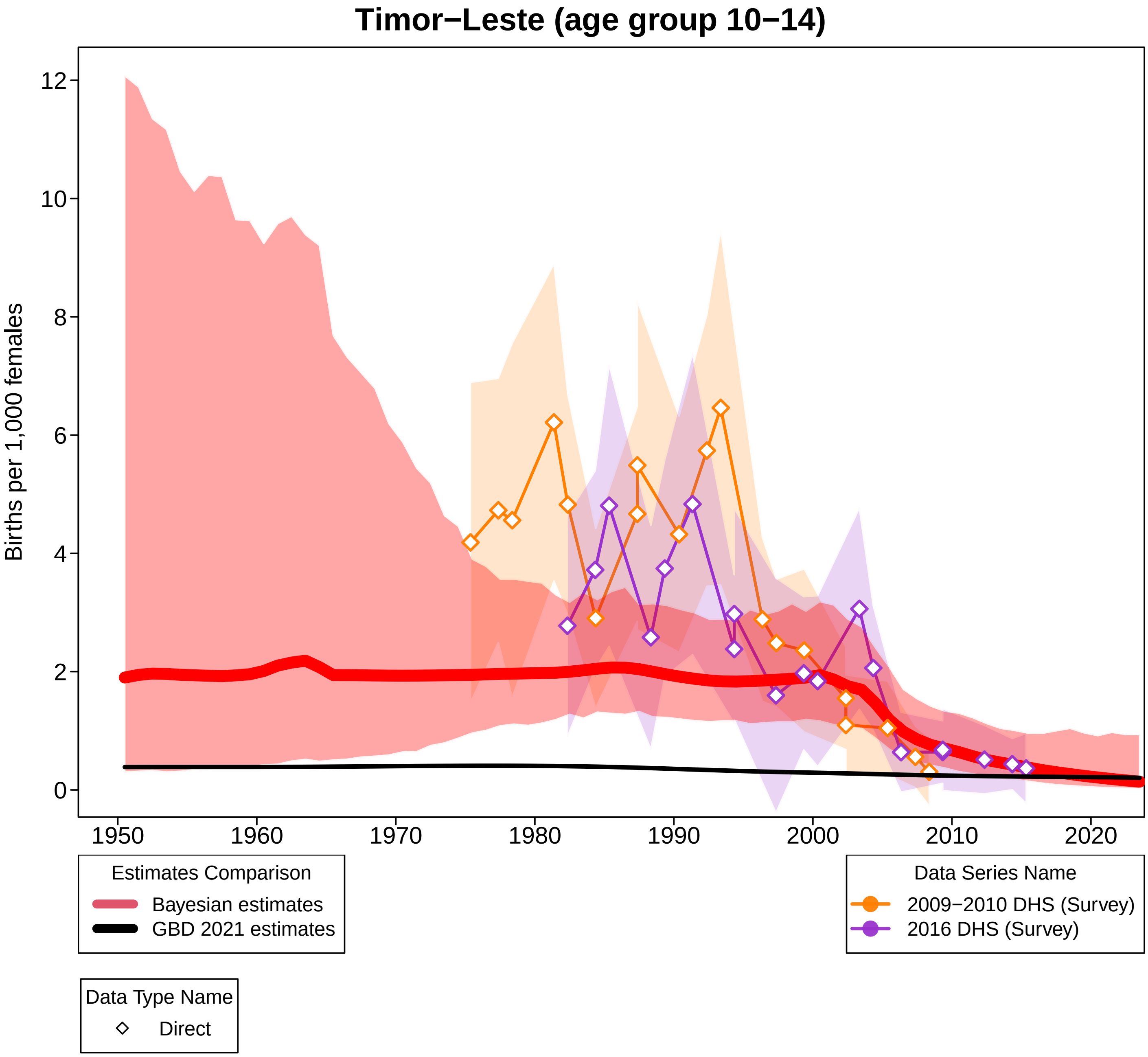
Timor−Leste (age group 10−14)
Births per 1,000 females
0
2
4
6
8
10
12
1950
1960
1970
1980
1990
2000
2010
2020
Estimates Comparison
Bayesian estimates
GBD 2021 estimates
Data Series Name
2009−2010 DHS (Survey)
2016 DHS (Survey)
Data Type Name
Direct

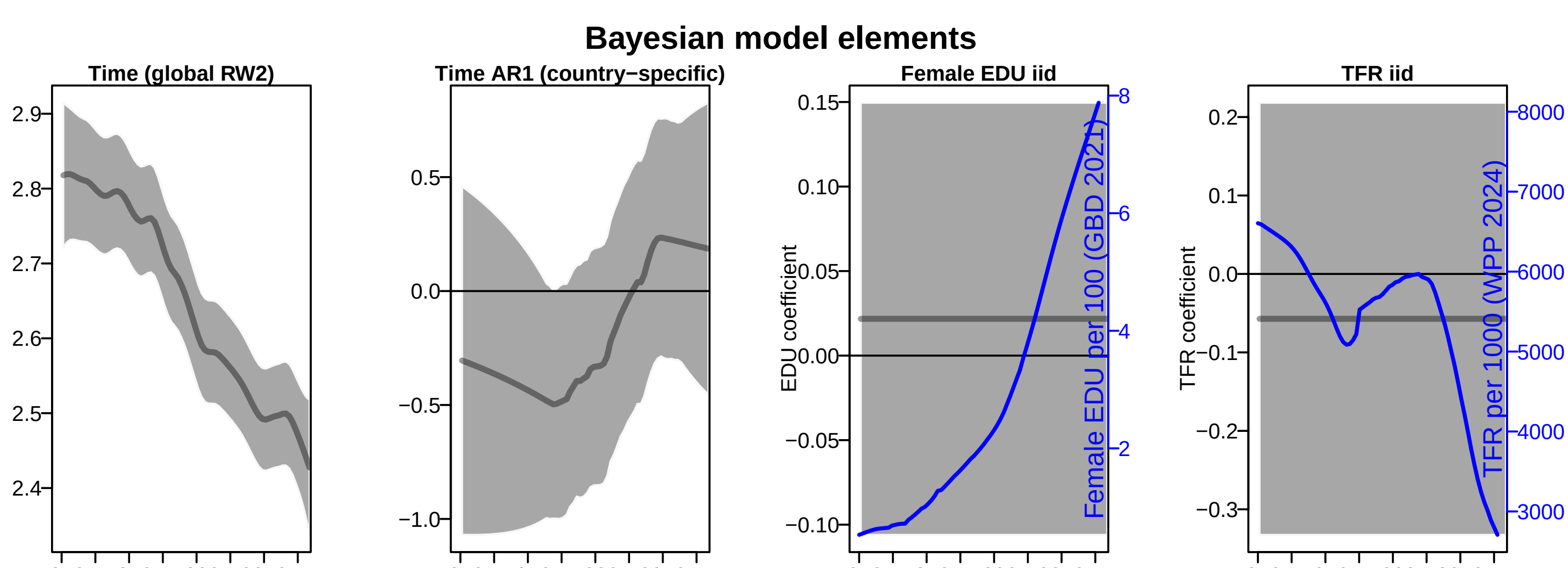
Bayesian model elements
Time (global RW2)
Time AR1 (country−specific)
Female EDU iid
TFR iid
EDU coefficient
Female EDU per 100 (GBD 2021)
TFR coefficient
TFR per 1000 (WPP 2024)
1950
1970
1990
2010

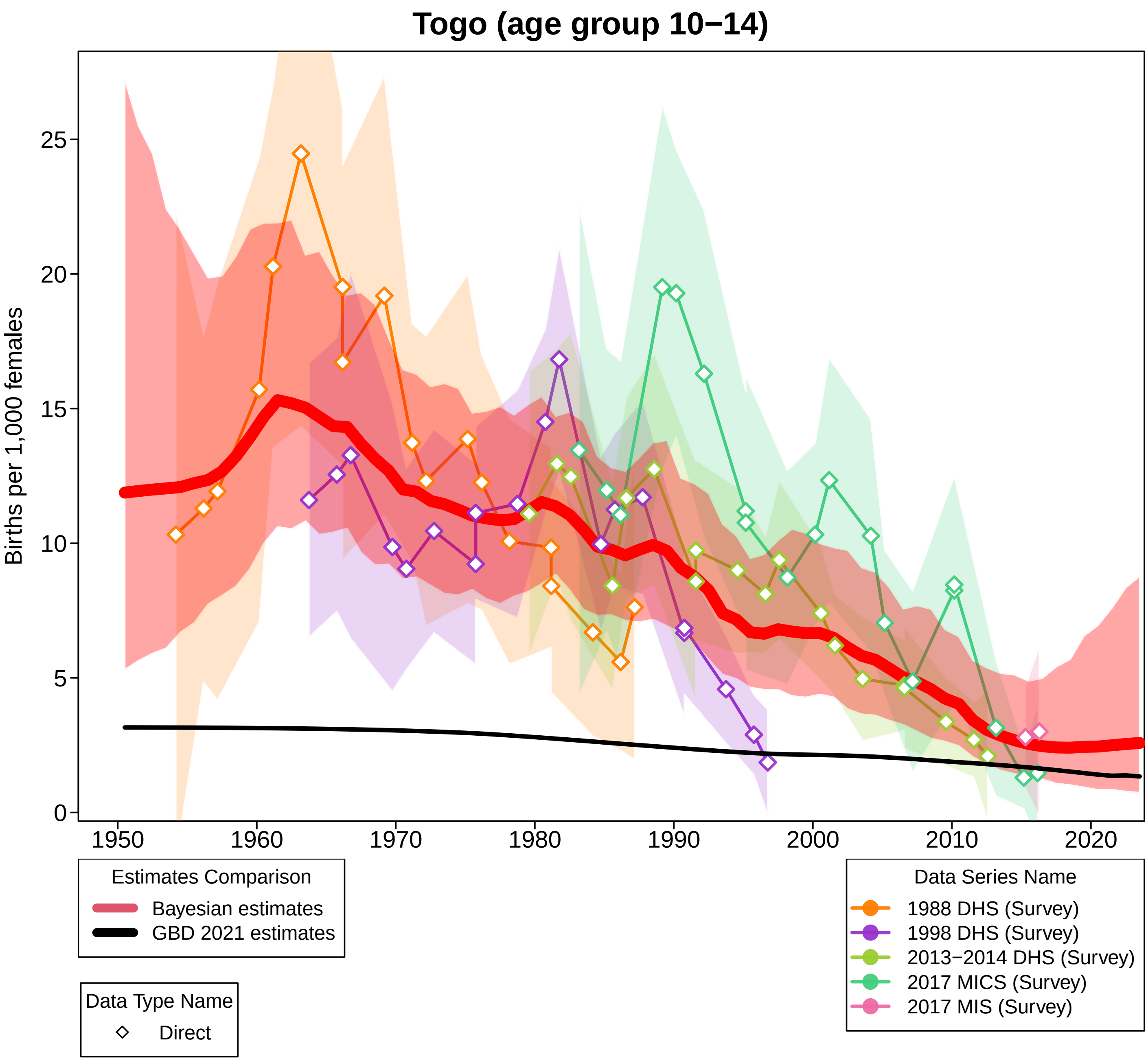
Togo (age group 10−14)
Births per 1,000 females
0
5
10
15
20
25
1950
1960
1970
1980
1990
2000
2010
2020
Estimates Comparison
Bayesian estimates
GBD 2021 estimates
Data Type Name
Direct
Data Series Name
1988 DHS (Survey)
1998 DHS (Survey)
2013−2014 DHS (Survey)
2017 MICS (Survey)
2017 MIS (Survey)

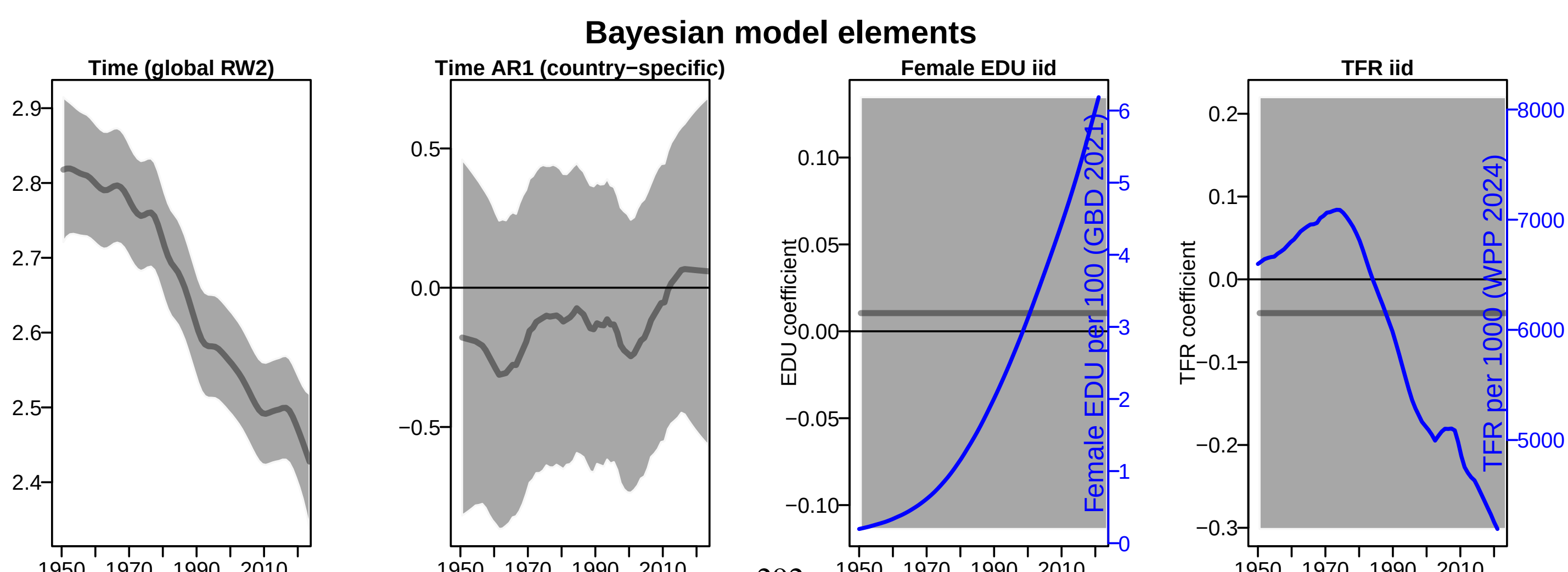
Bayesian model elements
Time (global RW2)
Time AR1 (country−specific)
Female EDU iid
TFR iid
EDU coefficient
Female EDU per 100 (GBD 2021)
TFR coefficient
TFR per 1000 (WPP 2024)
1950
1970
1990
2010

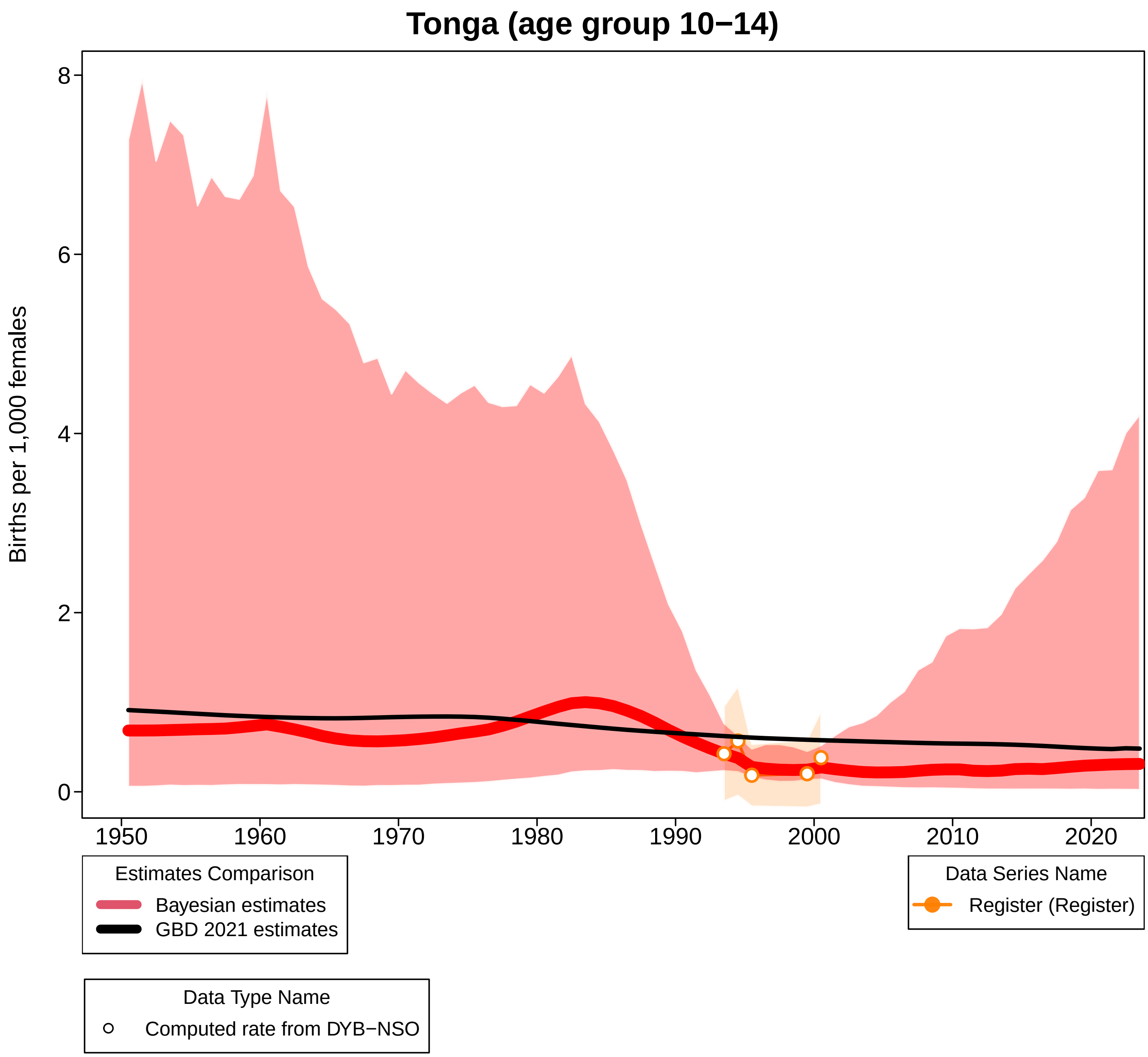

Tonga (age group 10−14)
Births per 1,000 females
0
2
4
6
8
1950
1960
1970
1980
1990
2000
2010
2020
Estimates Comparison
Bayesian estimates
GBD 2021 estimates
Data Series Name
Register (Register)
Data Type Name
Computed rate from DYB−NSO

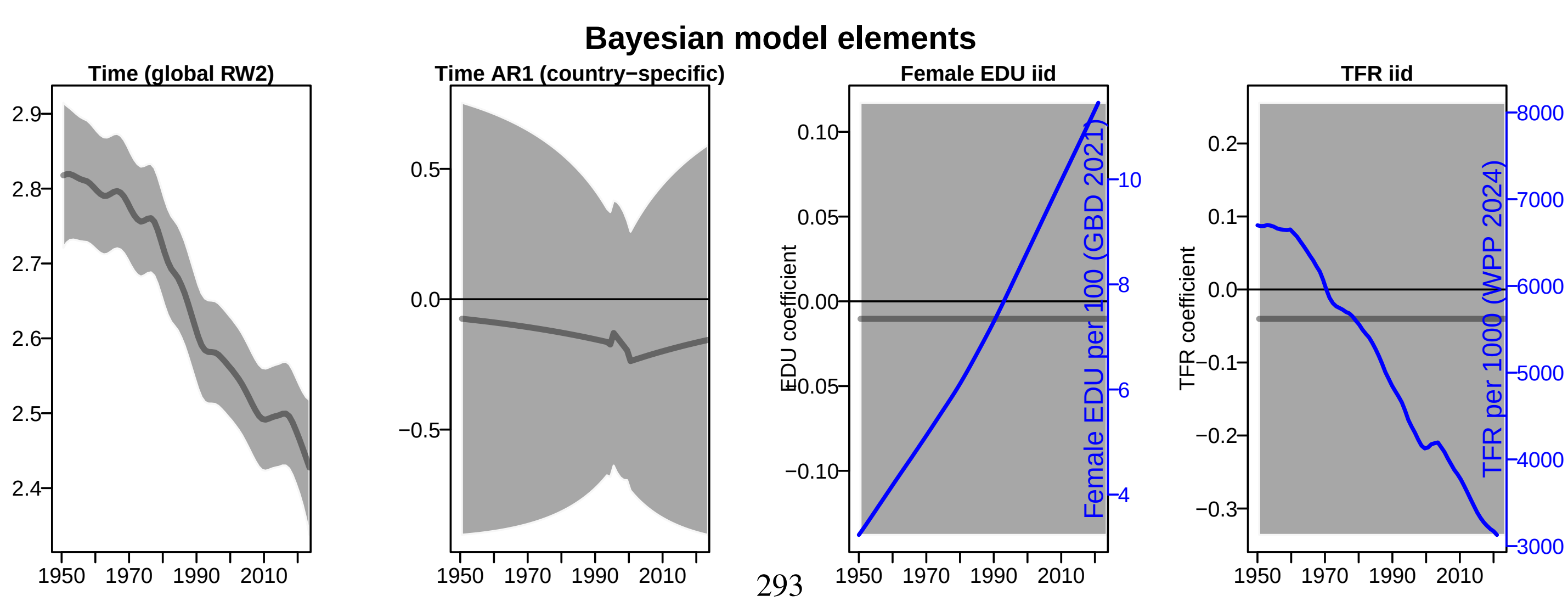

Bayesian model elements
Time (global RW2)
Time AR1 (country−specific)
Female EDU iid
TFR iid
EDU coefficient
Female EDU per 100 (GBD 2021)
TFR coefficient
TFR per 1000 (WPP 2024)
1950
1970
1990
2010

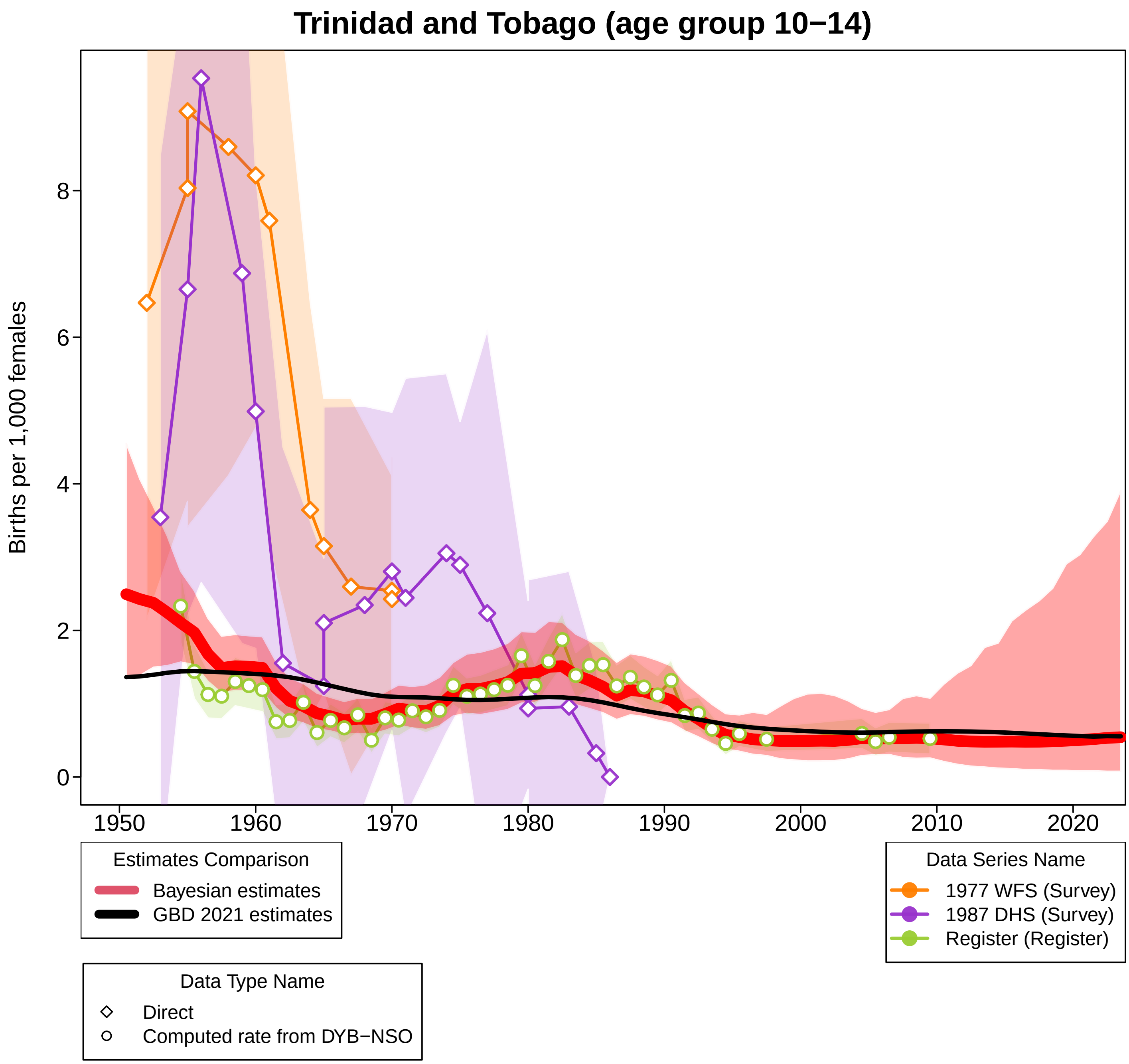
Trinidad and Tobago (age group 10−14)
Births per 1,000 females
0
2
4
6
8
1950
1960
1970
1980
1990
2000
2010
2020
Estimates Comparison
Bayesian estimates
GBD 2021 estimates
Data Series Name
1977 WFS (Survey)
1987 DHS (Survey)
Register (Register)
Data Type Name
Direct
Computed rate from DYB−NSO

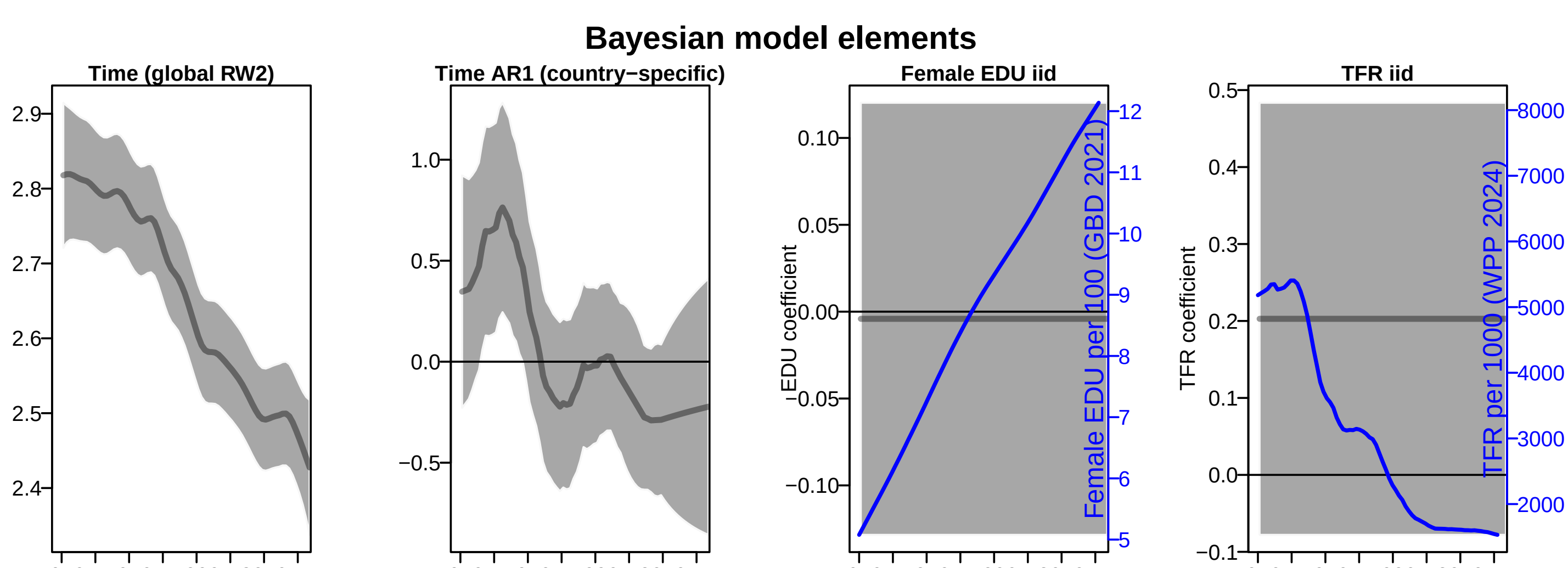
Bayesian model elements
Time (global RW2)
Time AR1 (country−specific)
Female EDU iid
TFR iid
EDU coefficient
Female EDU per 100 (GBD 2021)
TFR coefficient
TFR per 1000 (WPP 2024)
1950
1970
1990
2010

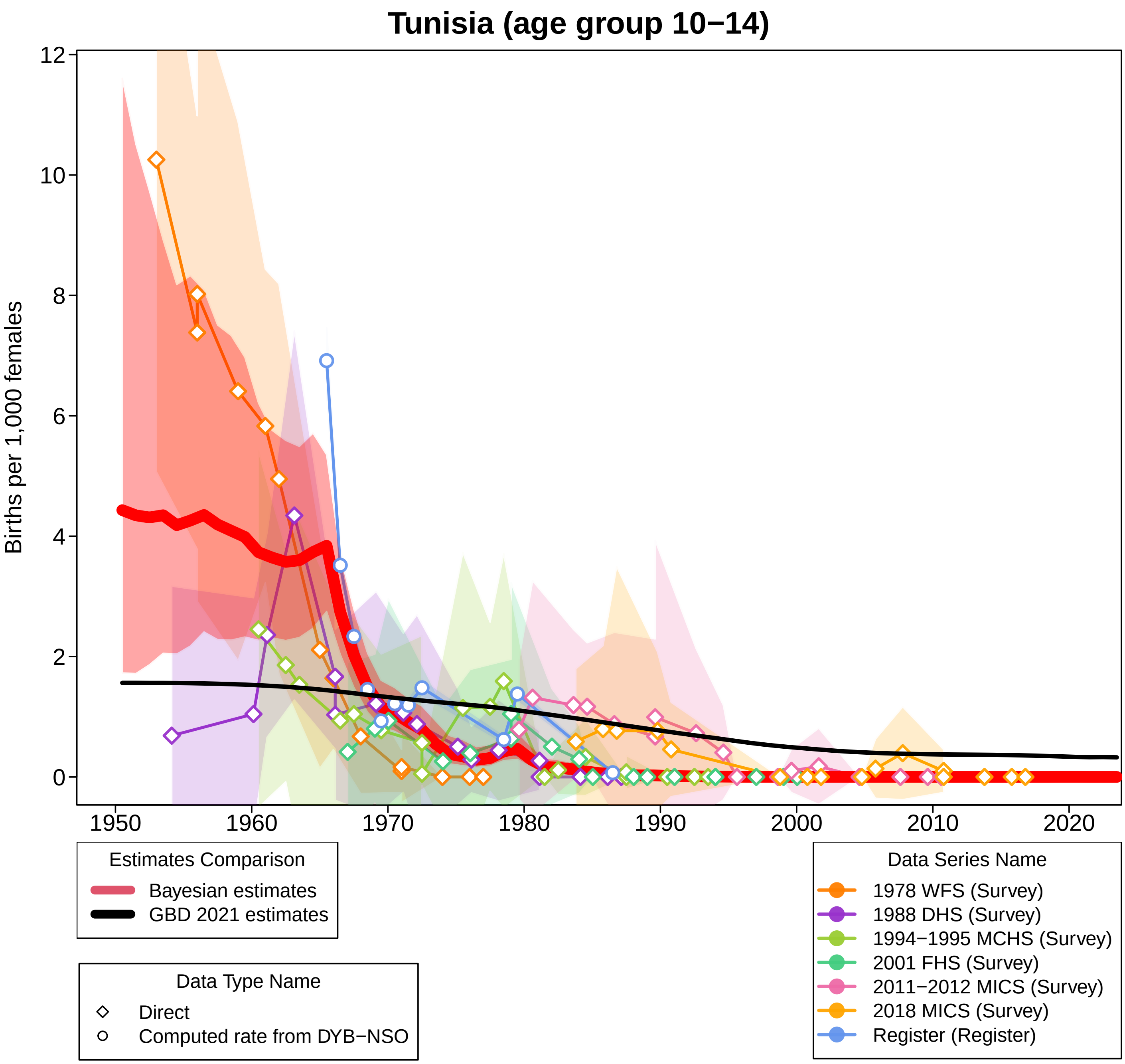
Tunisia (age group 10−14)
Births per 1,000 females
0
2
4
6
8
10
12
1950
1960
1970
1980
1990
2000
2010
2020
Estimates Comparison
Bayesian estimates
GBD 2021 estimates
Data Series Name
1978 WFS (Survey)
1988 DHS (Survey)
1994−1995 MCHS (Survey)
2001 FHS (Survey)
2011−2012 MICS (Survey)
2018 MICS (Survey)
Register (Register)
Data Type Name
Direct
Computed rate from DYB−NSO

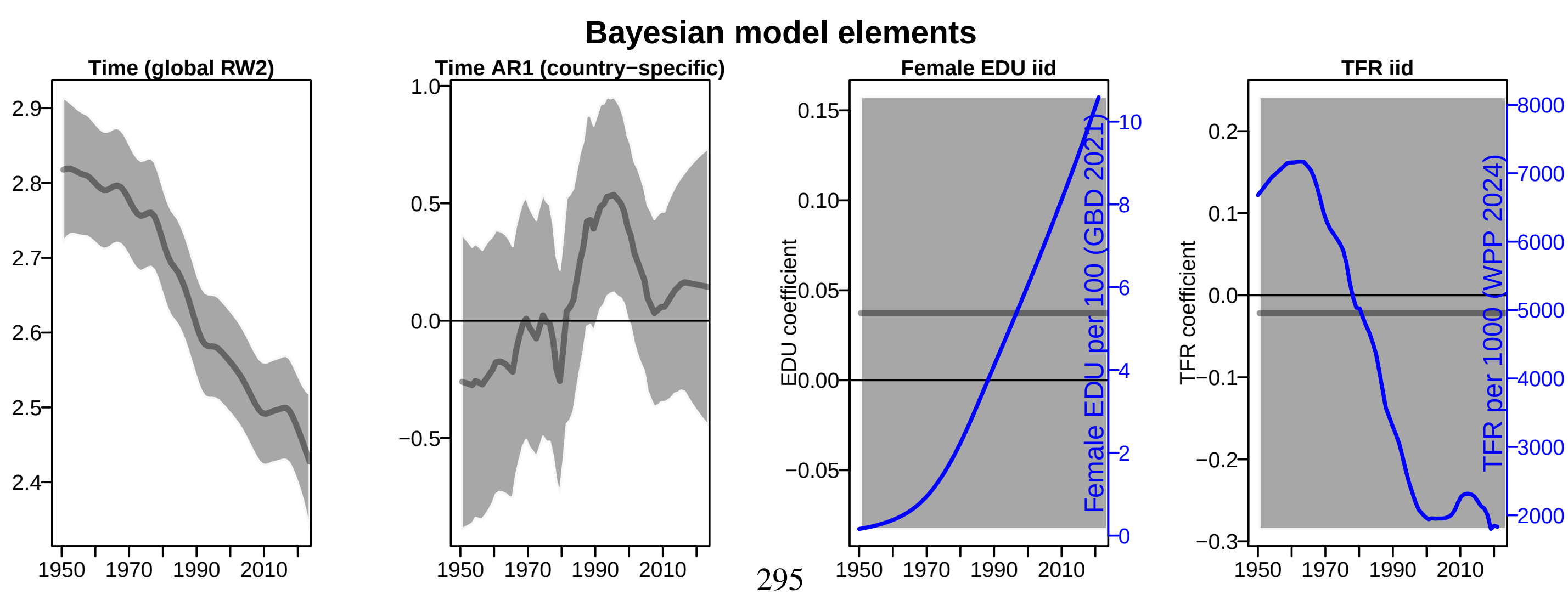
Bayesian model elements
Time (global RW2)
Time AR1 (country−specific)
Female EDU iid
TFR iid
EDU coefficient
Female EDU per 100 (GBD 2021)
TFR coefficient
TFR per 1000 (WPP 2024)
1950
1970
1990
2010

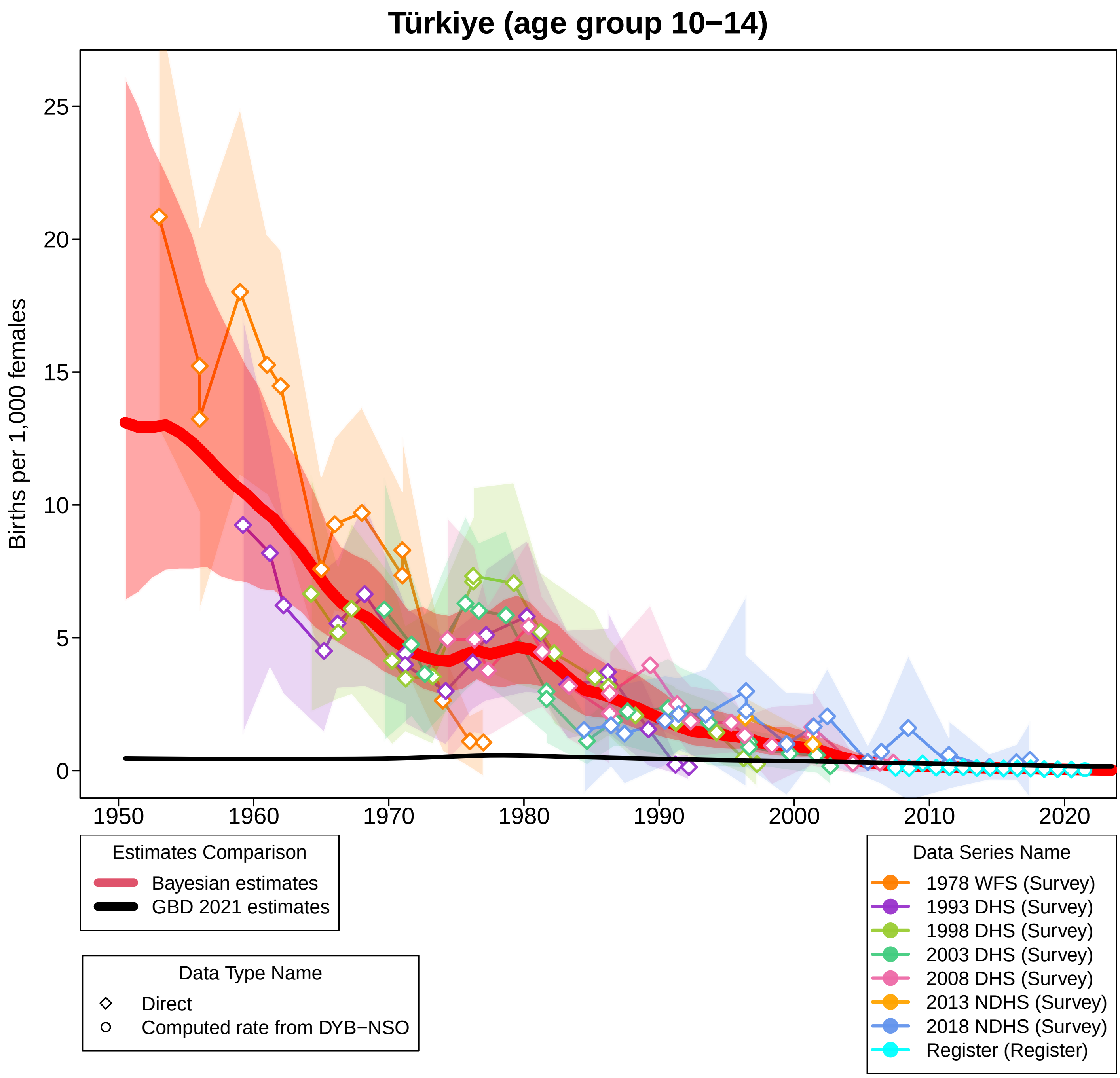

Türkiye (age group 10−14)
Births per 1,000 females
0
5
10
15
20
25
1950
1960
1970
1980
1990
2000
2010
2020
Estimates Comparison
Bayesian estimates
GBD 2021 estimates
Data Type Name
Direct
Computed rate from DYB−NSO
Data Series Name
1978 WFS (Survey)
1993 DHS (Survey)
1998 DHS (Survey)
2003 DHS (Survey)
2008 DHS (Survey)
2013 NDHS (Survey)
2018 NDHS (Survey)
Register (Register)

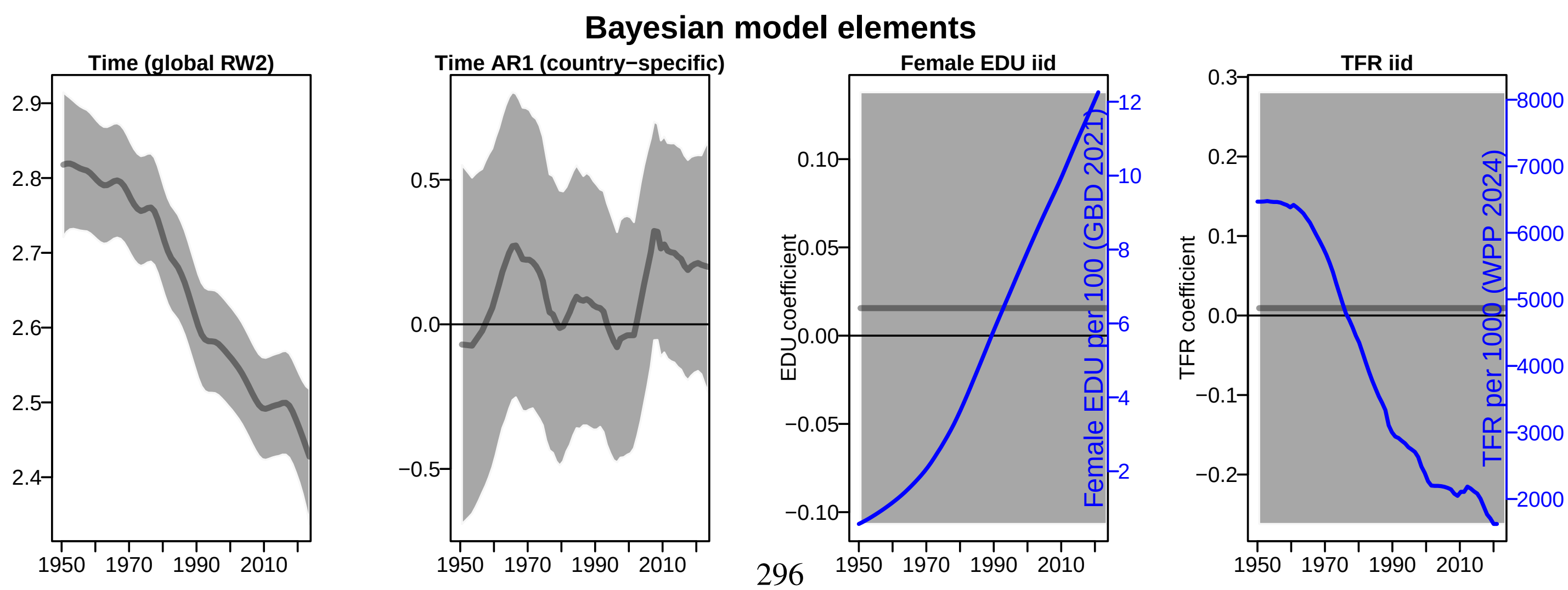

Bayesian model elements
Time (global RW2)
Time AR1 (country−specific)
Female EDU iid
TFR iid
EDU coefficient
Female EDU per 100 (GBD 2021)
TFR coefficient
TFR per 1000 (WPP 2024)
1950
1970
1990
2010

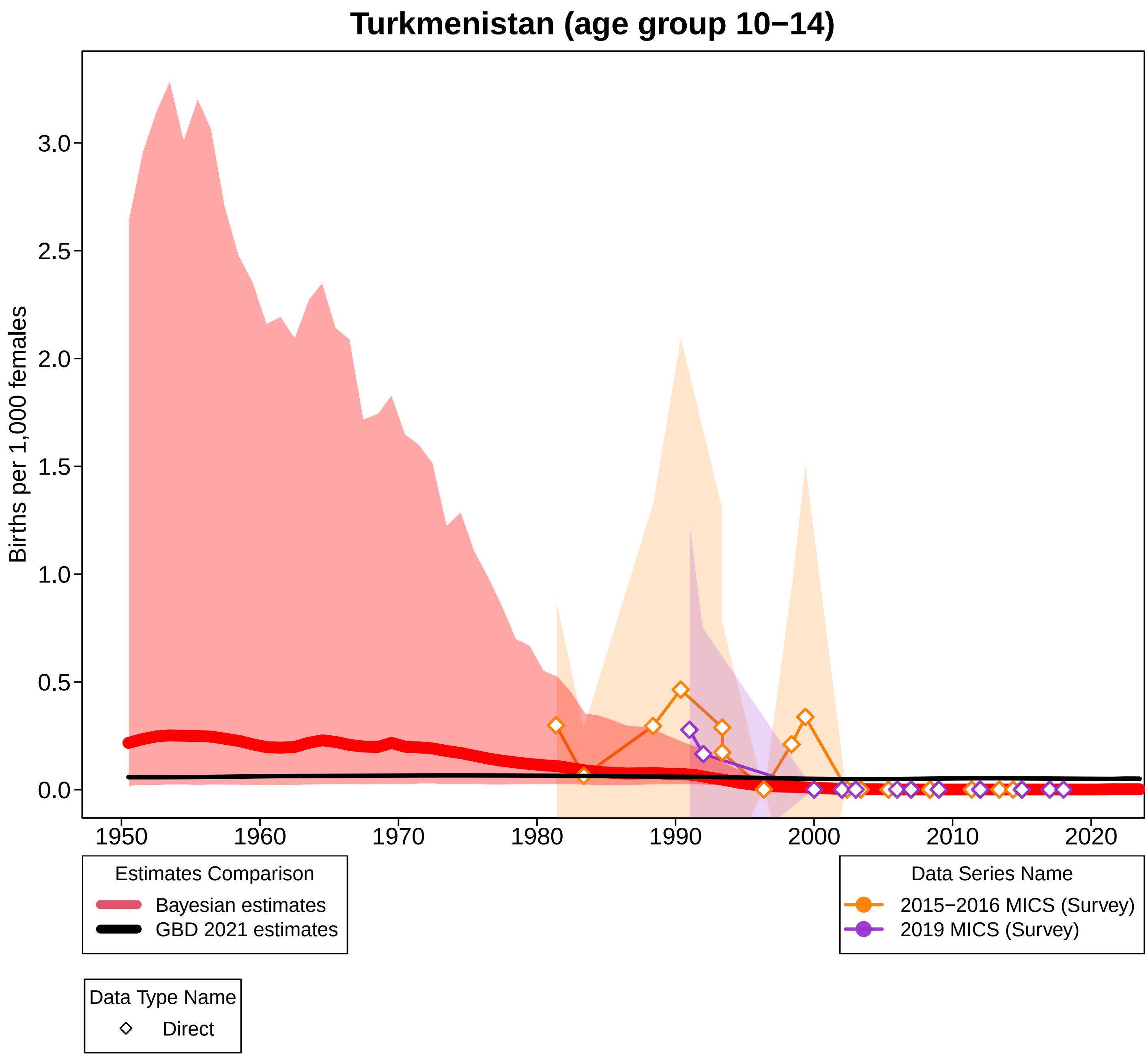
Turkmenistan (age group 10−14)
Births per 1,000 females
3.0
2.5
2.0
1.5
1.0
0.5
0.0
1950
1960
1970
1980
1990
2000
2010
2020
Estimates Comparison
Bayesian estimates
GBD 2021 estimates
Data Series Name
2015−2016 MICS (Survey)
2019 MICS (Survey)
Data Type Name
Direct

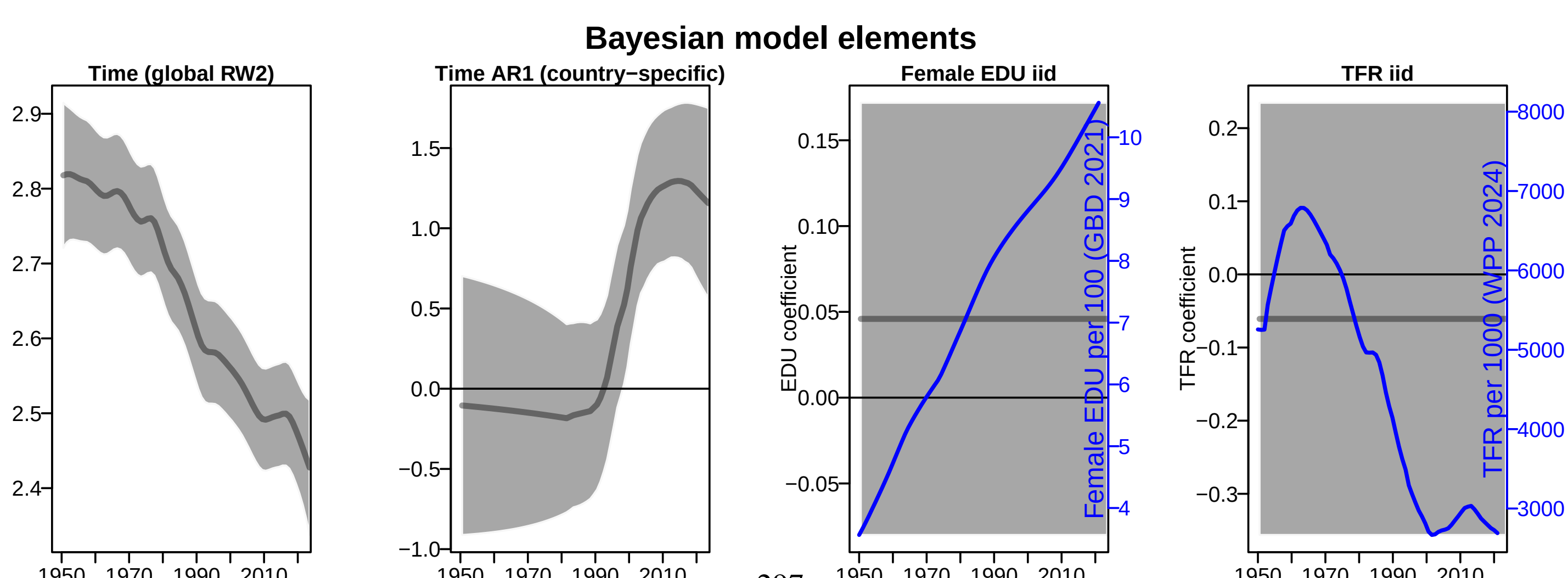
Bayesian model elements
Time (global RW2)
Time AR1 (country−specific)
Female EDU iid
TFR iid
EDU coefficient
Female EDU per 100 (GBD 2021)
TFR coefficient
TFR per 1000 (WPP 2024)
1950
1970
1990
2010

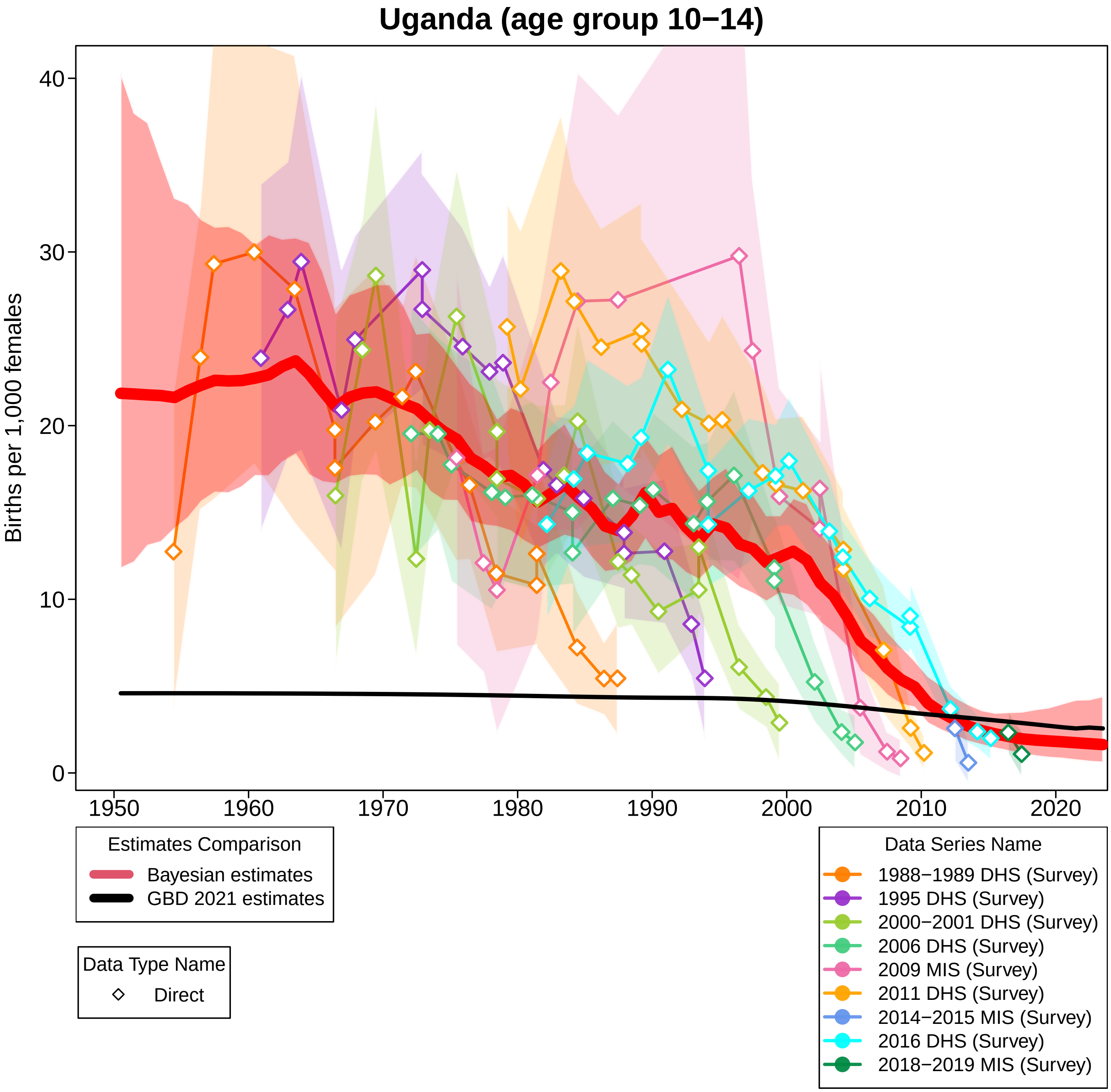

Uganda (age group 10−14)
Births per 1,000 females
0
10
20
30
40
1950
1960
1970
1980
1990
2000
2010
2020
Estimates Comparison
Bayesian estimates
GBD 2021 estimates
Data Type Name
Direct
Data Series Name
1988−1989 DHS (Survey)
1995 DHS (Survey)
2000−2001 DHS (Survey)
2006 DHS (Survey)
2009 MIS (Survey)
2011 DHS (Survey)
2014−2015 MIS (Survey)
2016 DHS (Survey)
2018−2019 MIS (Survey)

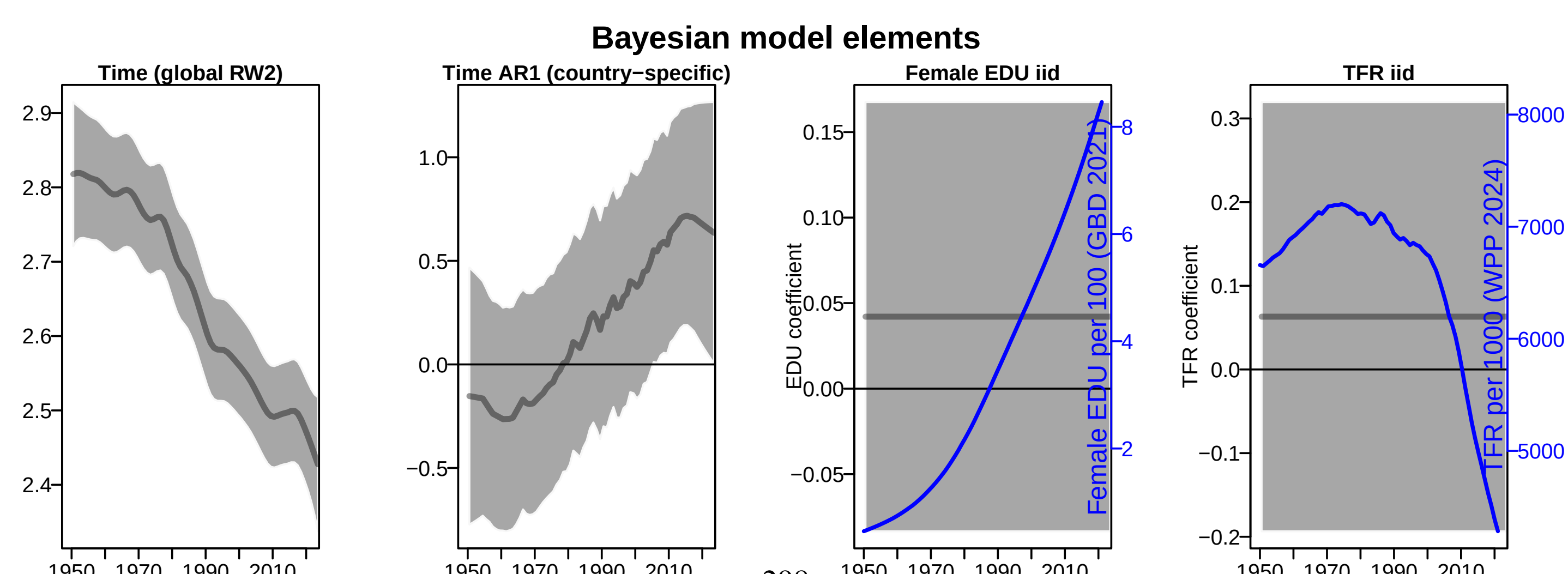

Bayesian model elements
Time (global RW2)
Time AR1 (country−specific)
Female EDU iid
TFR iid
EDU coefficient
Female EDU per 100 (GBD 2021)
TFR coefficient
TFR per 1000 (WPP 2024)
1950
1970
1990
2010

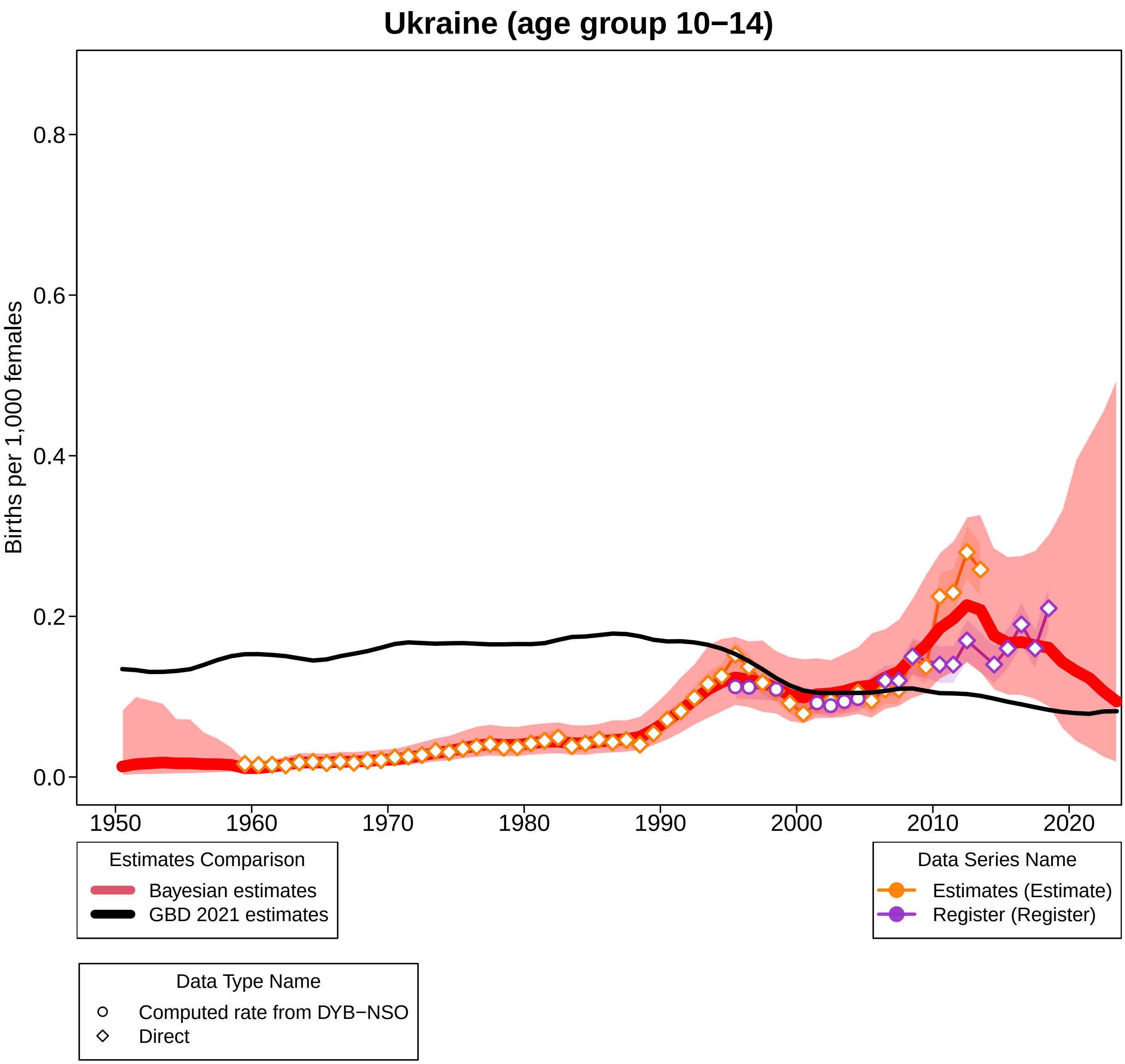

Ukraine (age group 10−14)
Births per 1,000 females
0.0
0.2
0.4
0.6
0.8
1950
1960
1970
1980
1990
2000
2010
2020
Estimates Comparison
Bayesian estimates
GBD 2021 estimates
Data Series Name
Estimates (Estimate)
Register (Register)
Data Type Name
Computed rate from DYB−NSO
Direct

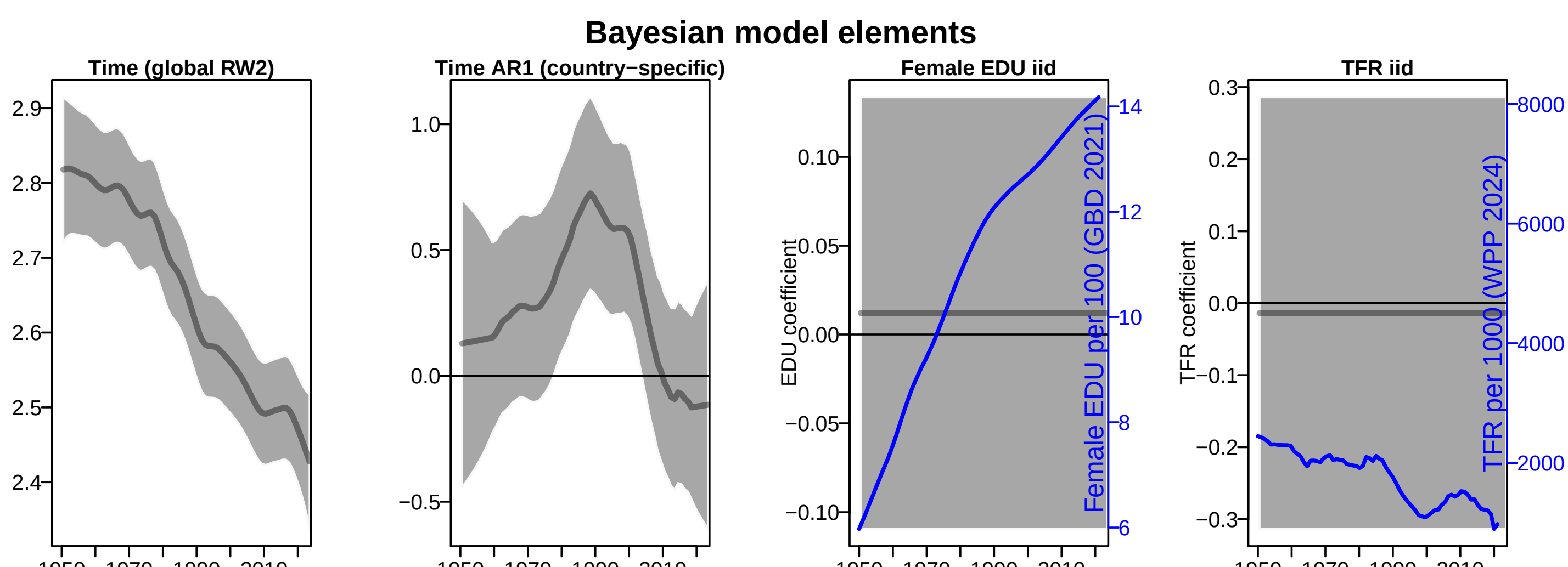

Bayesian model elements
Time (global RW2)
Time AR1 (country−specific)
Female EDU iid
TFR iid
EDU coefficient
Female EDU per 100 (GBD 2021)
TFR coefficient
TFR per 1000 (WPP 2024)

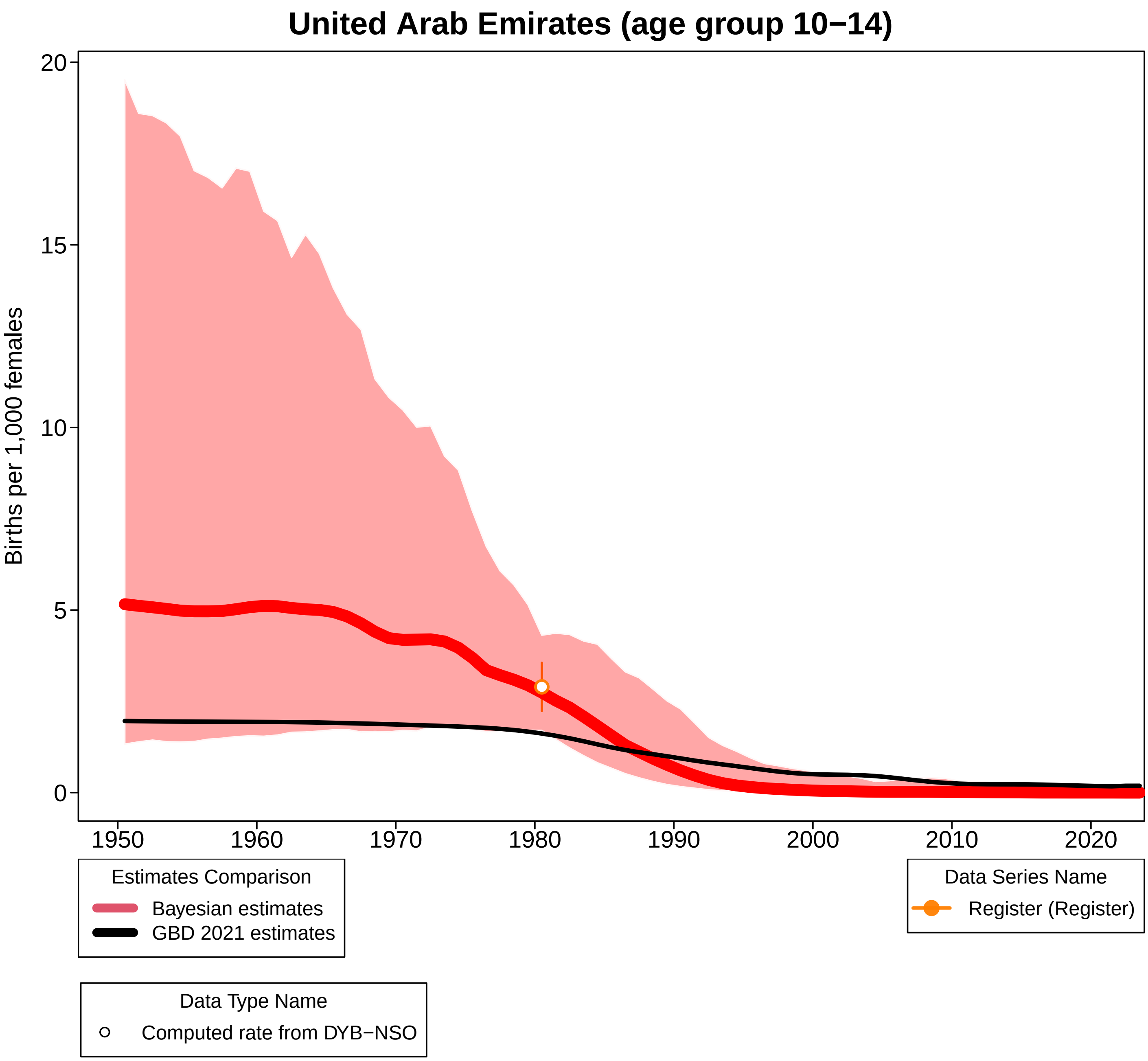
United Arab Emirates (age group 10−14)
Births per 1,000 females
20
15
10
5
0
1950
1960
1970
1980
1990
2000
2010
2020
Estimates Comparison
Bayesian estimates
GBD 2021 estimates
Data Series Name
Register (Register)
Data Type Name
Computed rate from DYB−NSO

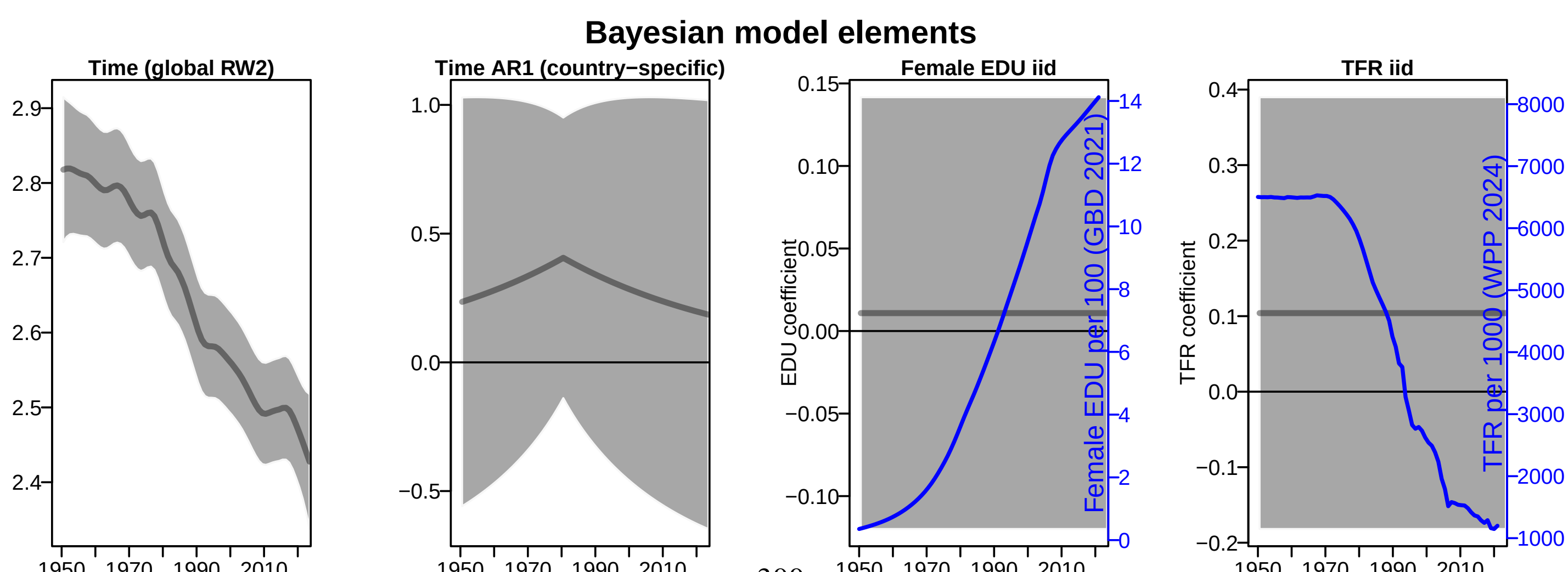
Bayesian model elements
Time (global RW2)
Time AR1 (country−specific)
Female EDU iid
TFR iid
EDU coefficient
Female EDU per 100 (GBD 2021)
TFR coefficient
TFR per 1000 (WPP 2024)
1950
1970
1990
2010

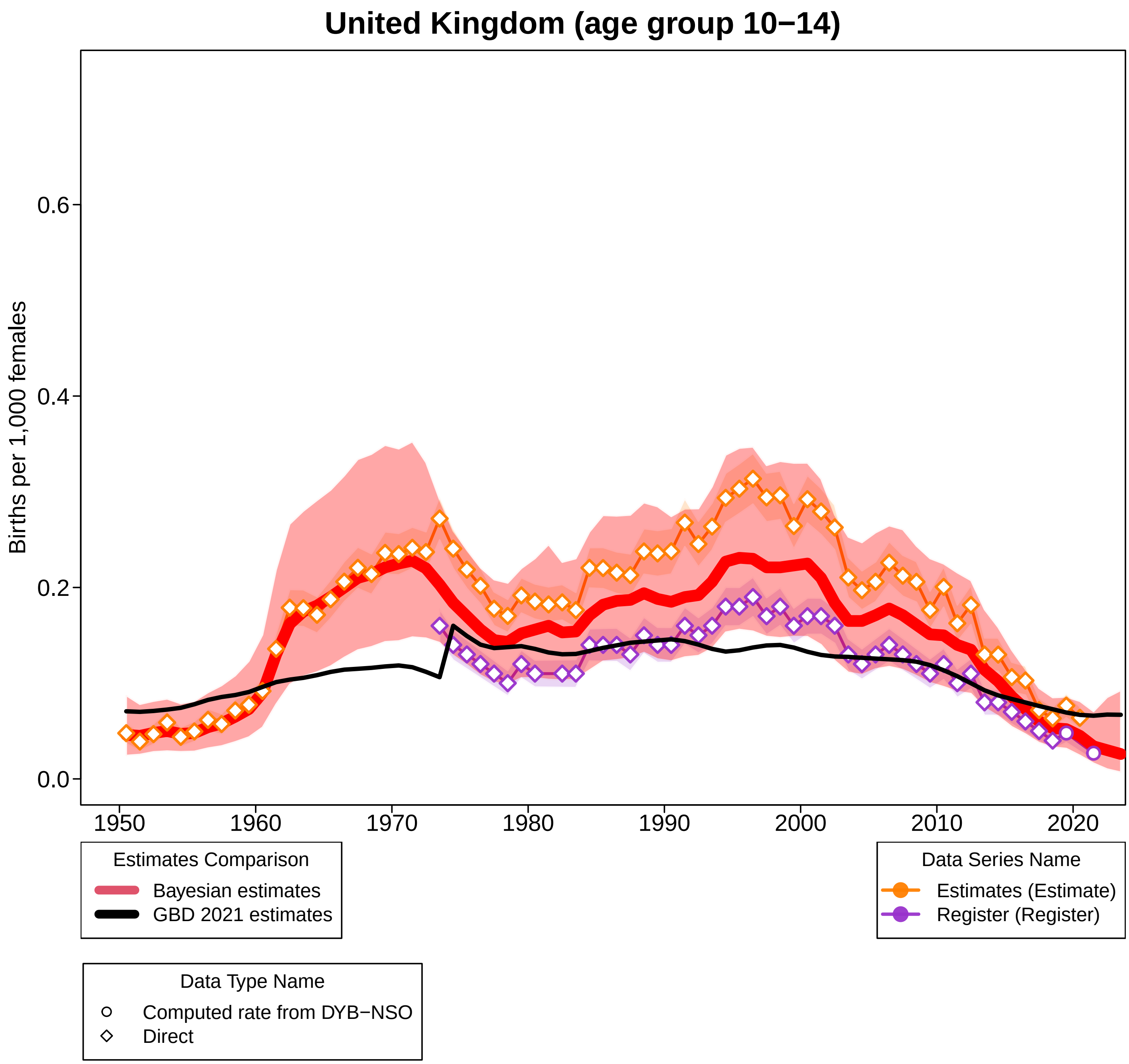
United Kingdom (age group 10−14)
Births per 1,000 females
0.0
0.2
0.4
0.6
1950
1960
1970
1980
1990
2000
2010
2020
Estimates Comparison
Bayesian estimates
GBD 2021 estimates
Data Series Name
Estimates (Estimate)
Register (Register)
Data Type Name
Computed rate from DYB−NSO
Direct

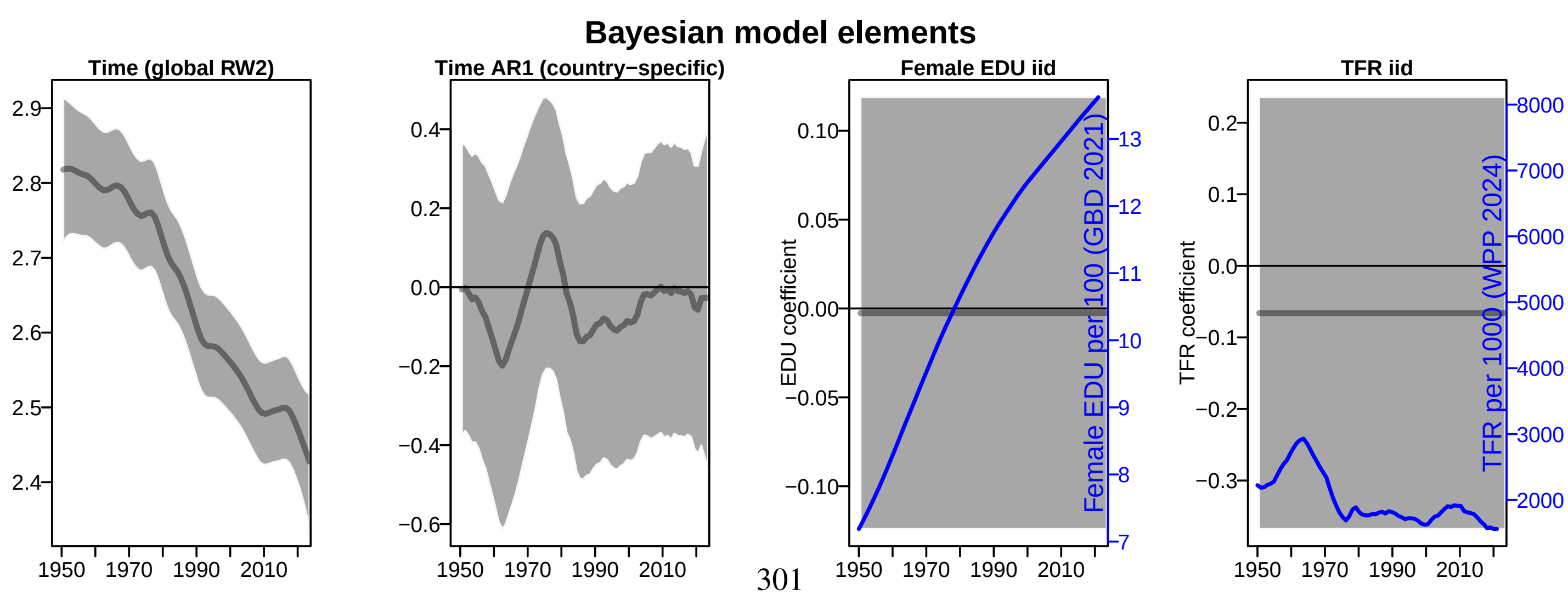
Bayesian model elements
Time (global RW2)
Time AR1 (country−specific)
Female EDU iid
TFR iid
EDU coefficient
Female EDU per 100 (GBD 2021)
TFR coefficient
TFR per 1000 (WPP 2024)

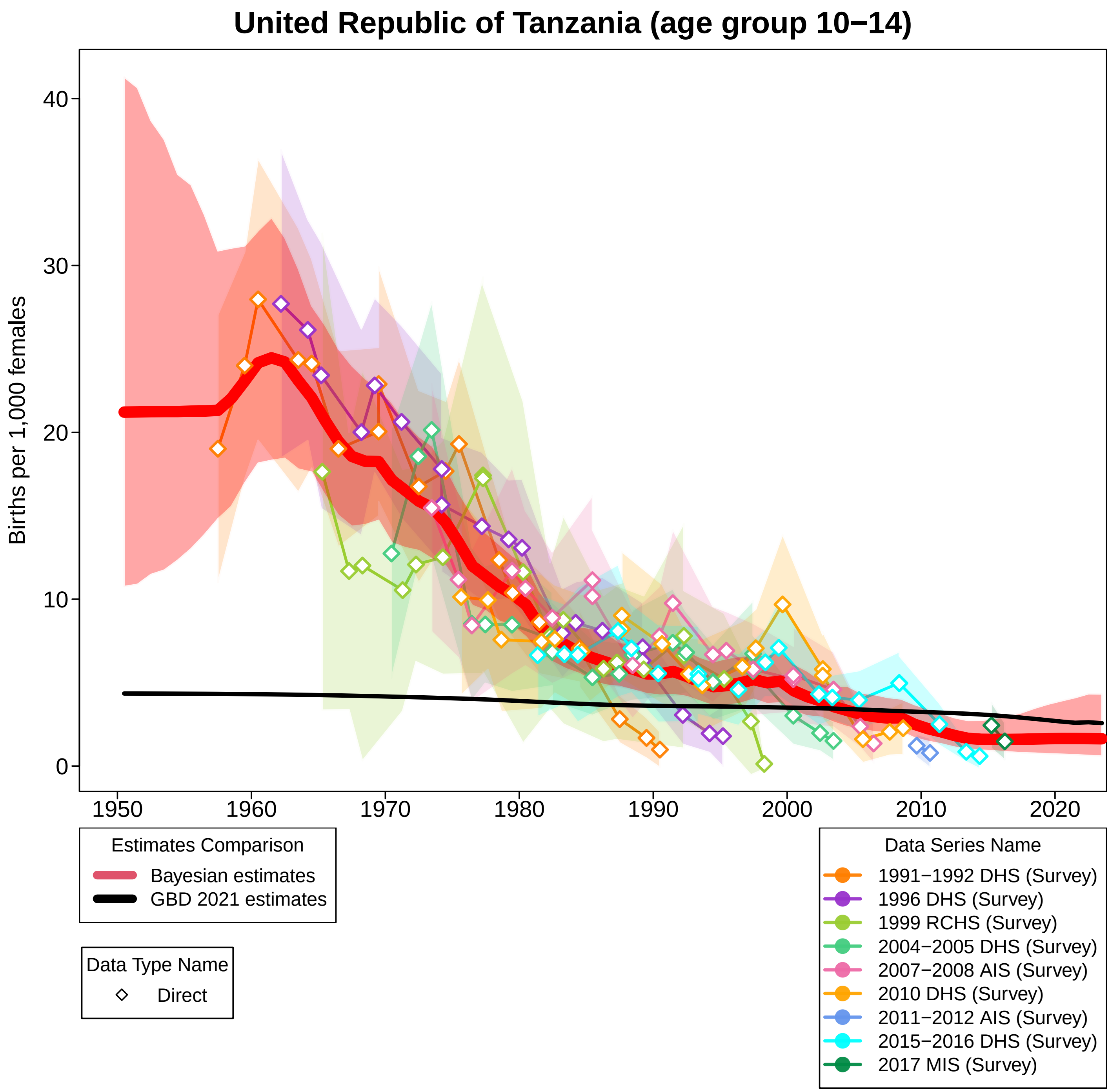
United Republic of Tanzania (age group 10−14)
Births per 1,000 females
0
10
20
30
40
1950
1960
1970
1980
1990
2000
2010
2020
Estimates Comparison
Bayesian estimates
GBD 2021 estimates
Data Type Name
Direct
Data Series Name
1991−1992 DHS (Survey)
1996 DHS (Survey)
1999 RCHS (Survey)
2004−2005 DHS (Survey)
2007−2008 AIS (Survey)
2010 DHS (Survey)
2011−2012 AIS (Survey)
2015−2016 DHS (Survey)
2017 MIS (Survey)

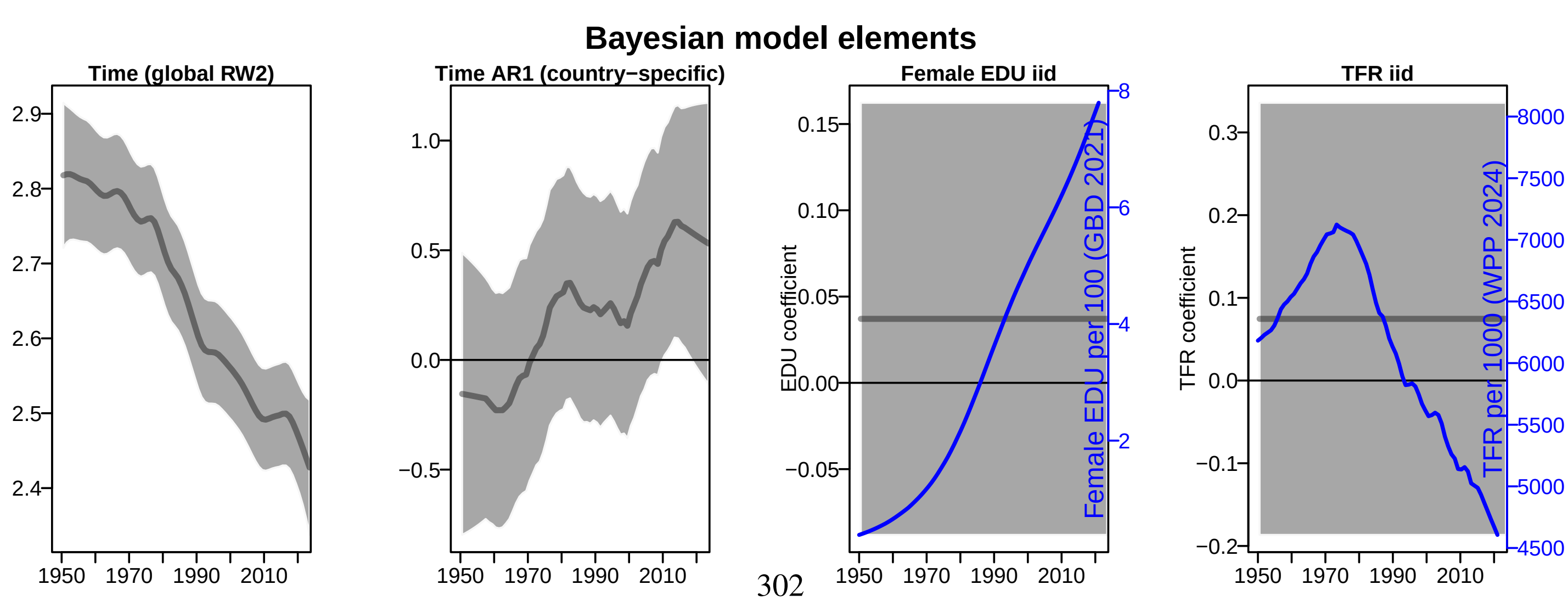
Bayesian model elements
Time (global RW2)
Time AR1 (country−specific)
Female EDU iid
TFR iid
EDU coefficient
Female EDU per 100 (GBD 2021)
TFR coefficient
TFR per 1000 (WPP 2024)

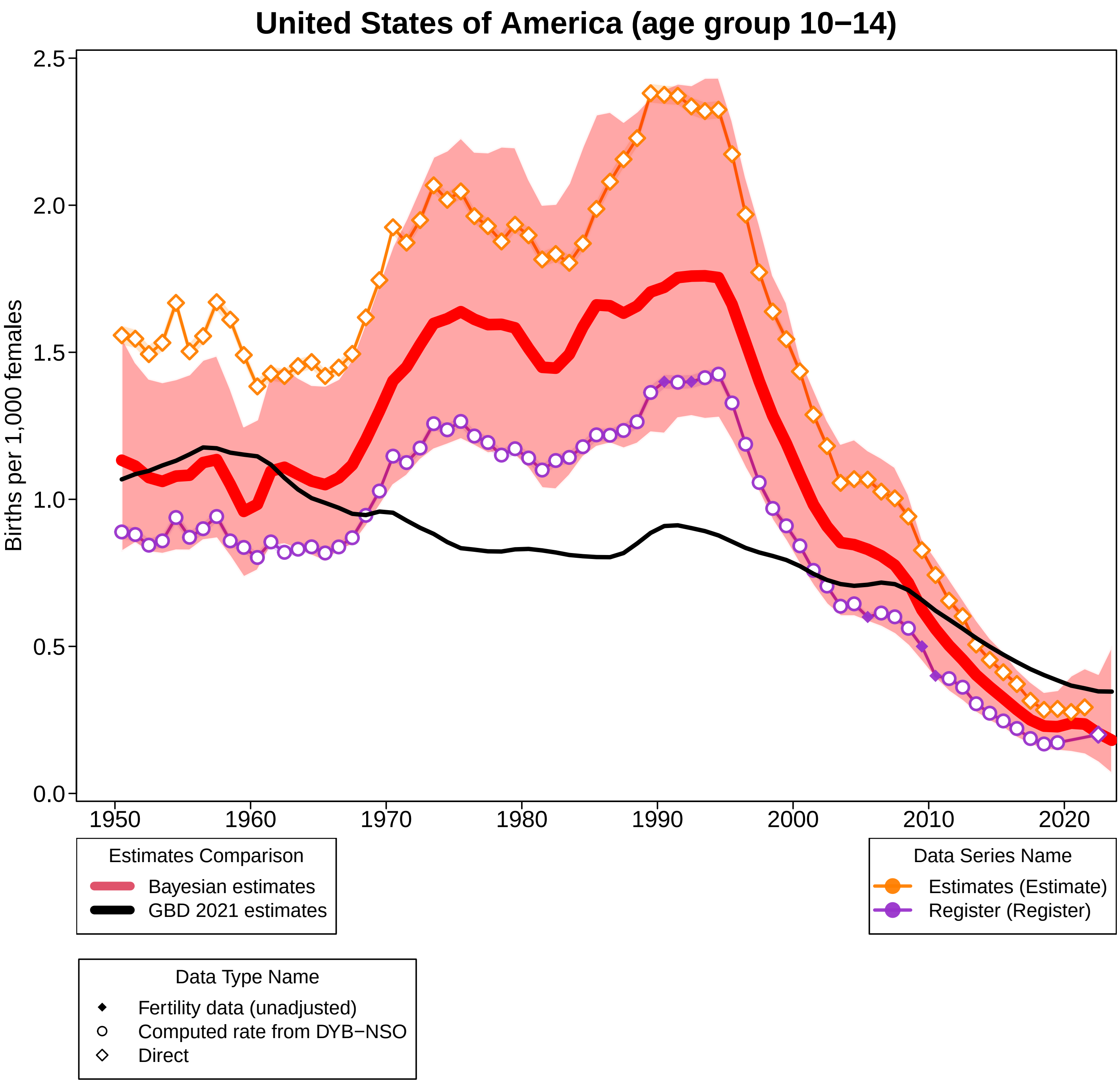
United States of America (age group 10−14)
Births per 1,000 females
2.5
2.0
1.5
1.0
0.5
0.0
1950
1960
1970
1980
1990
2000
2010
2020
Estimates Comparison
Bayesian estimates
GBD 2021 estimates
Data Series Name
Estimates (Estimate)
Register (Register)
Data Type Name
Fertility data (unadjusted)
Computed rate from DYB−NSO
Direct

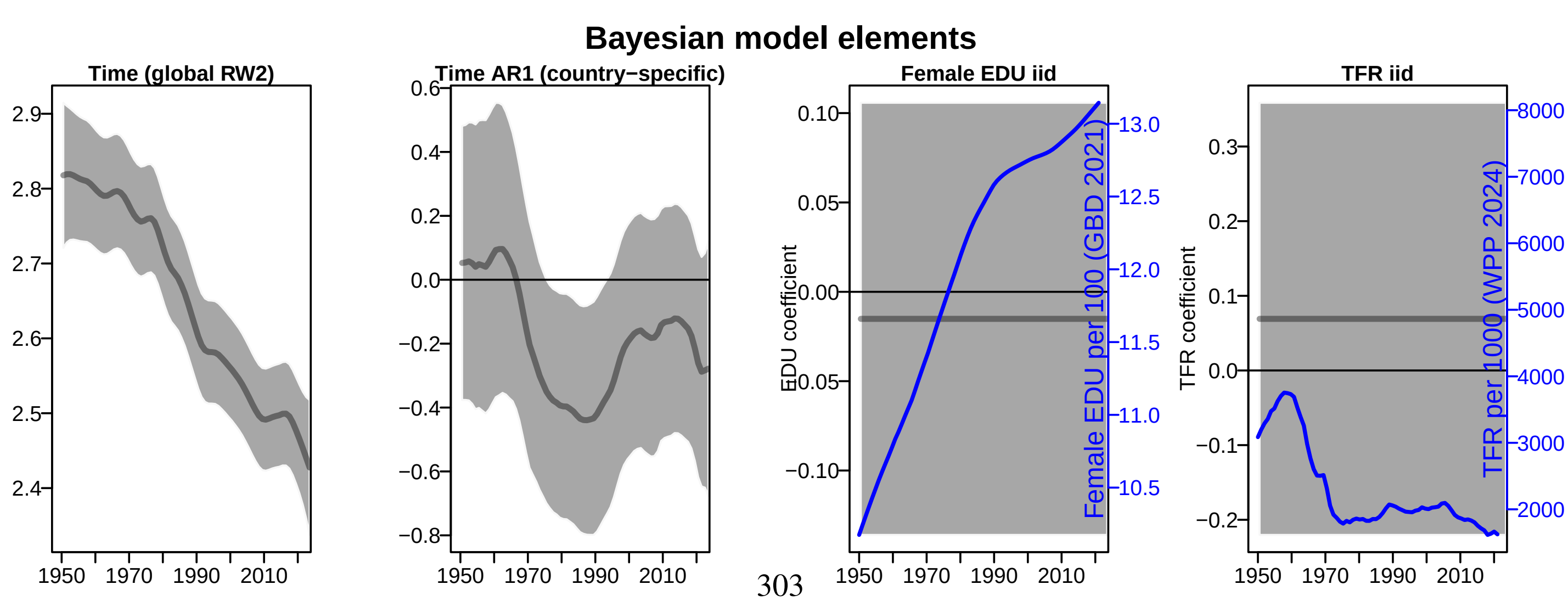
Bayesian model elements
Time (global RW2)
Time AR1 (country−specific)
Female EDU iid
TFR iid
EDU coefficient
Female EDU per 100 (GBD 2021)
TFR coefficient
TFR per 1000 (WPP 2024)

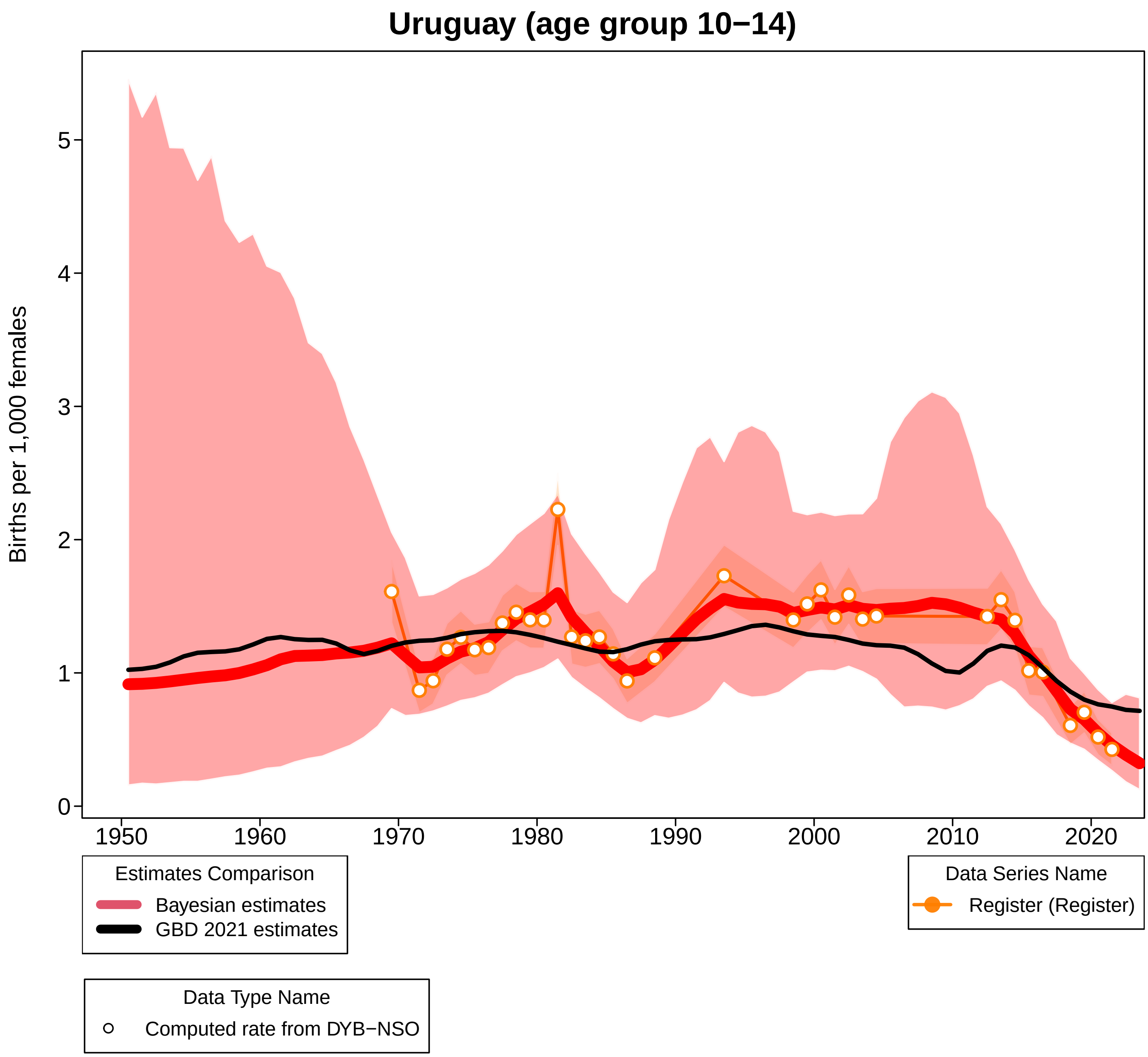

Uruguay (age group 10−14)
Births per 1,000 females
0
1
2
3
4
5
1950
1960
1970
1980
1990
2000
2010
2020
Estimates Comparison
Bayesian estimates
GBD 2021 estimates
Data Series Name
Register (Register)
Data Type Name
Computed rate from DYB−NSO

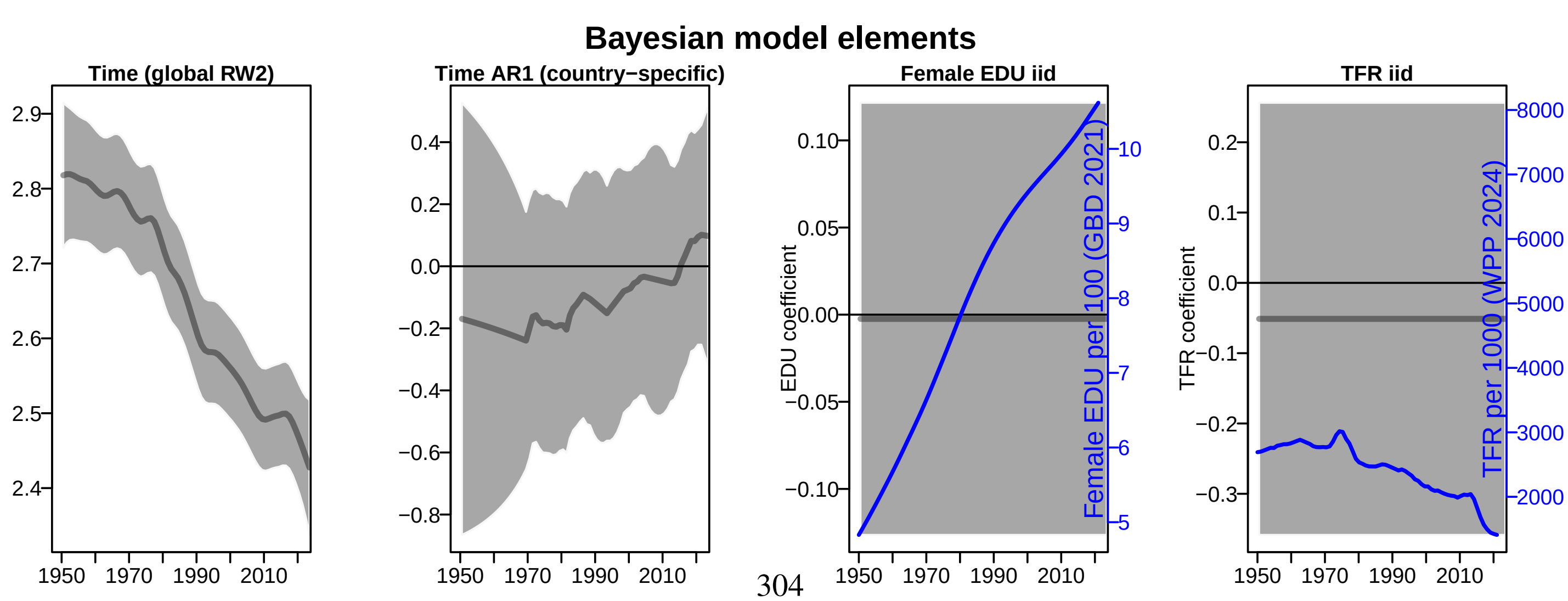

Bayesian model elements
Time (global RW2)
Time AR1 (country−specific)
Female EDU iid
TFR iid
EDU coefficient
Female EDU per 100 (GBD 2021)
TFR coefficient
TFR per 1000 (WPP 2024)
1950
1970
1990
2010

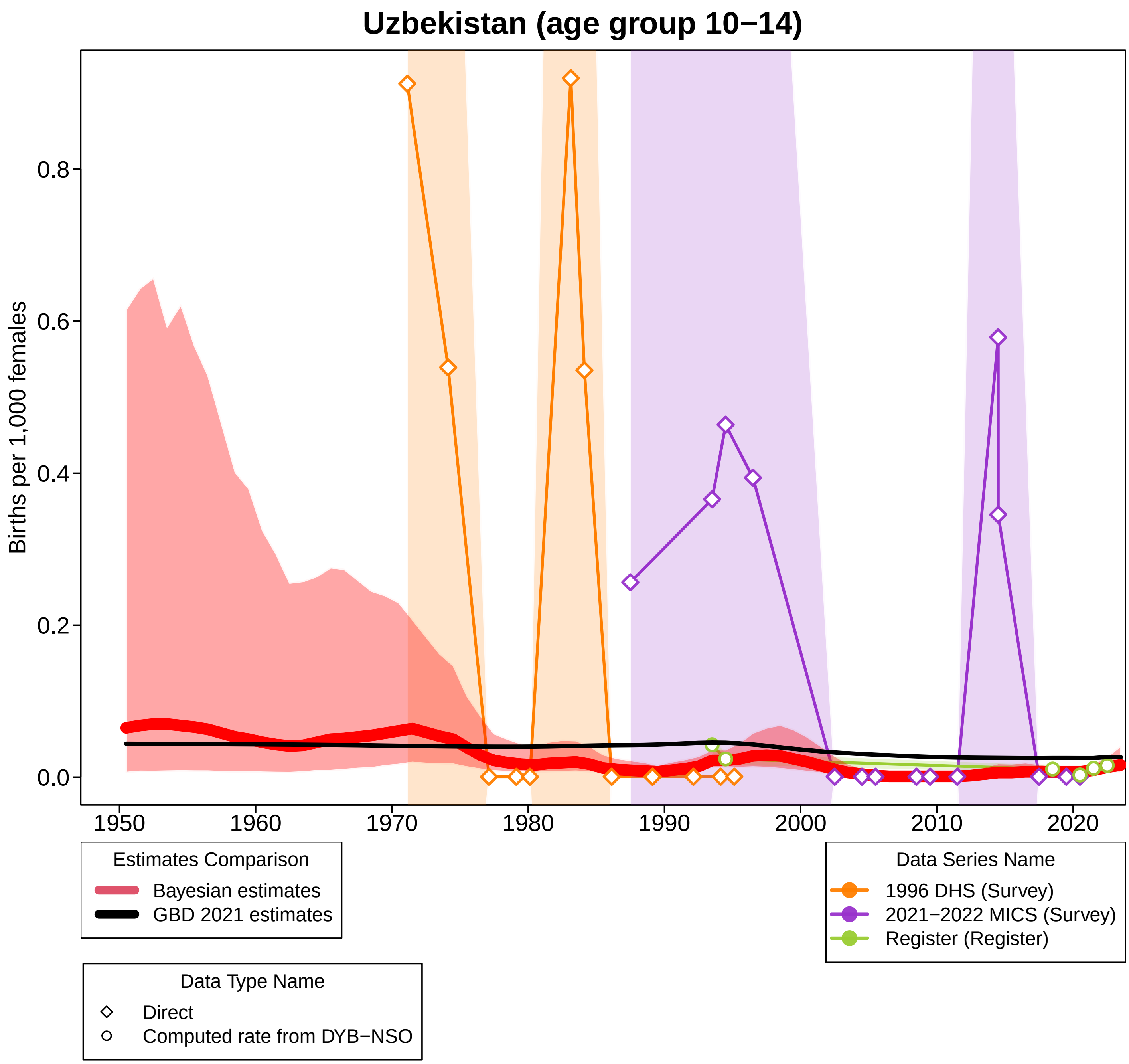
Uzbekistan (age group 10−14)
Births per 1,000 females
0.0
0.2
0.4
0.6
0.8
1950
1960
1970
1980
1990
2000
2010
2020
Estimates Comparison
Bayesian estimates
GBD 2021 estimates
Data Series Name
1996 DHS (Survey)
2021−2022 MICS (Survey)
Register (Register)
Data Type Name
Direct
Computed rate from DYB−NSO

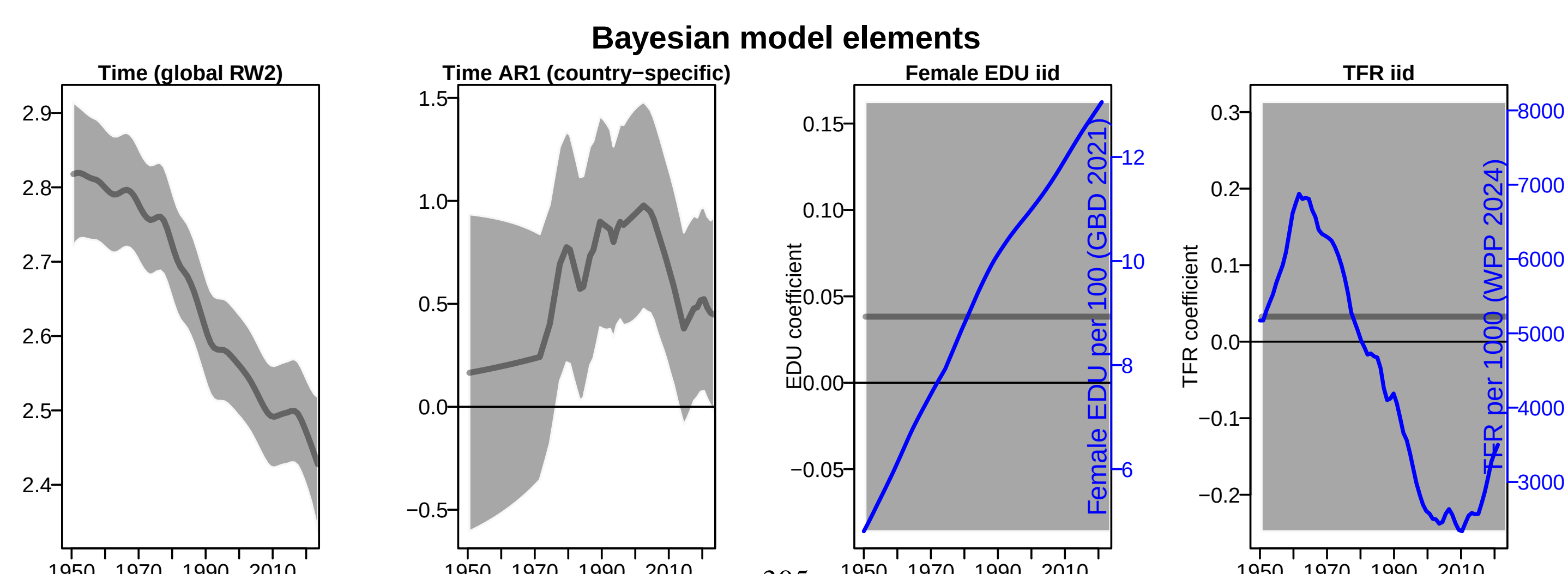
Bayesian model elements
Time (global RW2)
Time AR1 (country−specific)
Female EDU iid
TFR iid
EDU coefficient
Female EDU per 100 (GBD 2021)
TFR coefficient
TFR per 1000 (WPP 2024)
1950
1970
1990
2010

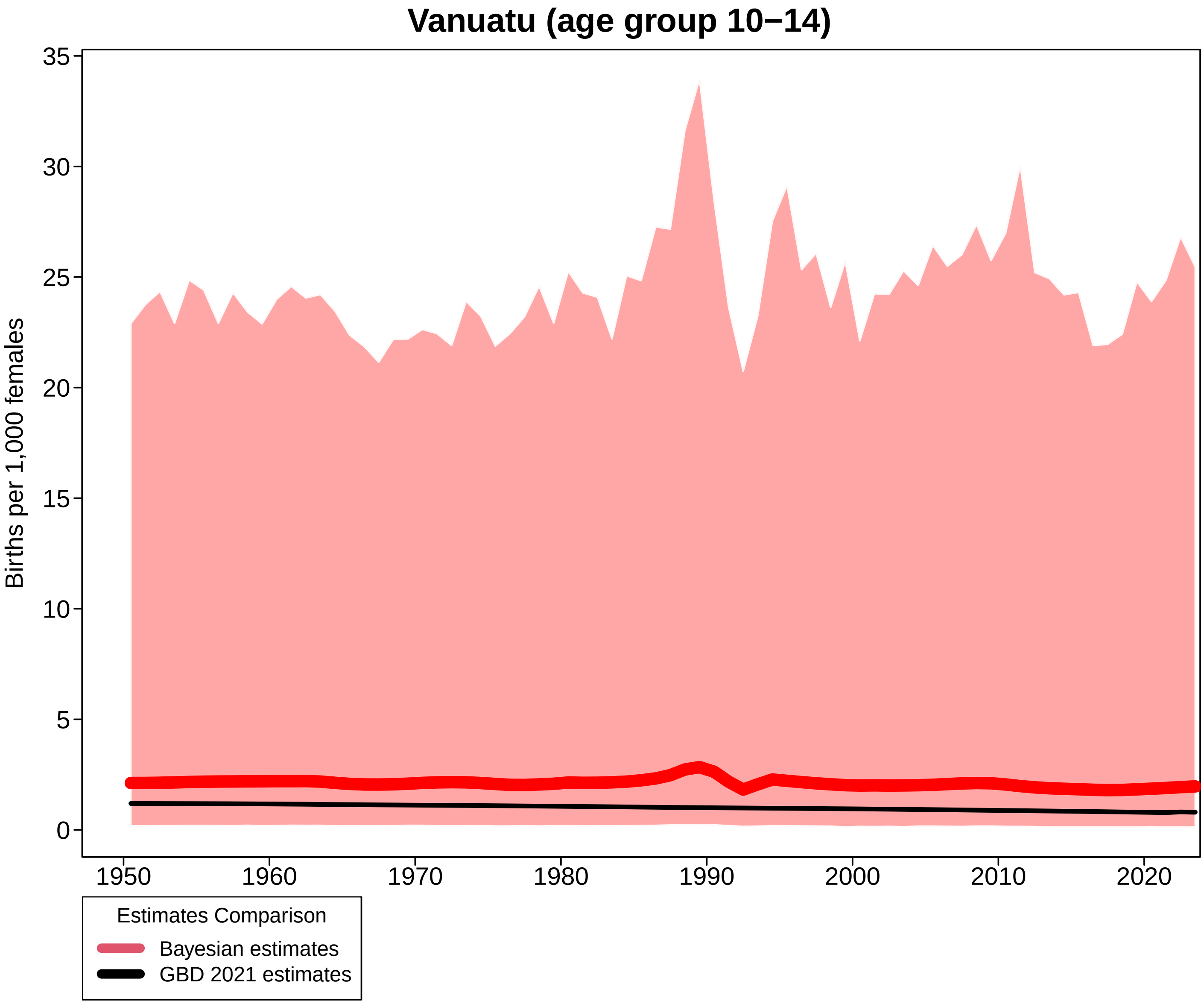
Vanuatu (age group 10−14)
Births per 1,000 females
0
5
10
15
20
25
30
35
1950
1960
1970
1980
1990
2000
2010
2020
Estimates Comparison
Bayesian estimates
GBD 2021 estimates

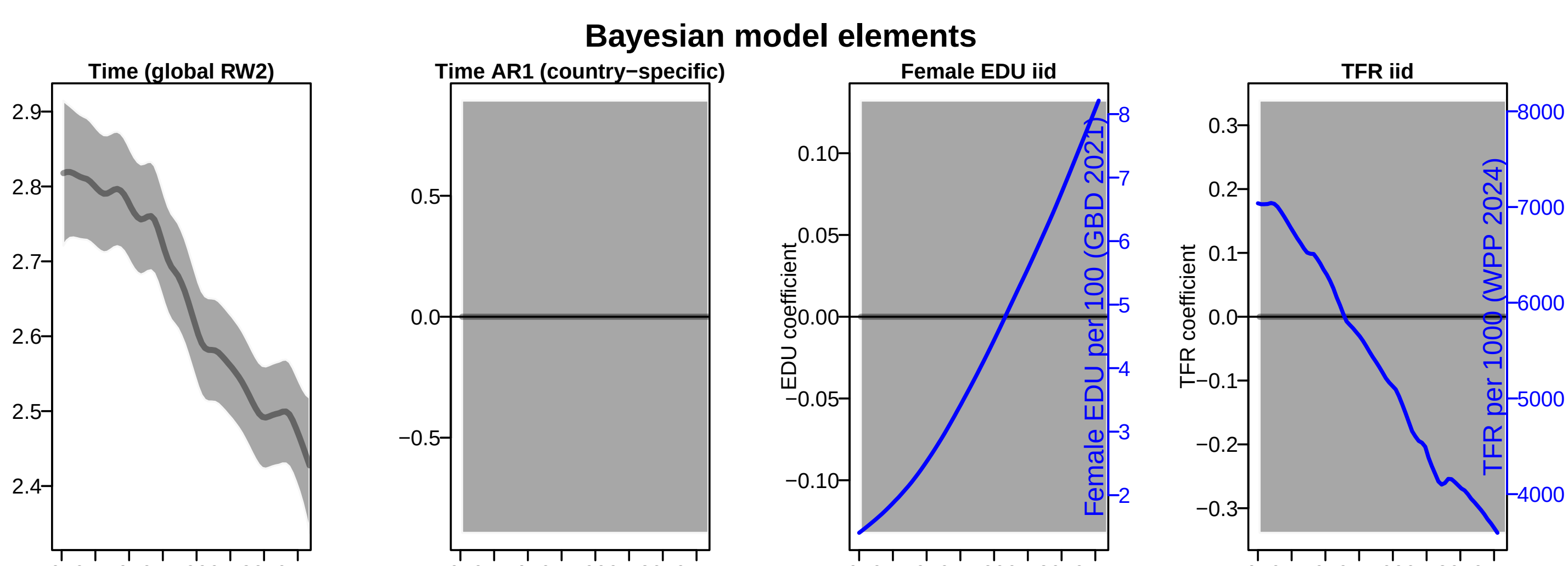
Bayesian model elements
Time (global RW2)
Time AR1 (country−specific)
Female EDU iid
TFR iid
EDU coefficient
Female EDU per 100 (GBD 2021)
TFR coefficient
TFR per 1000 (WPP 2024)
1950
1970
1990
2010

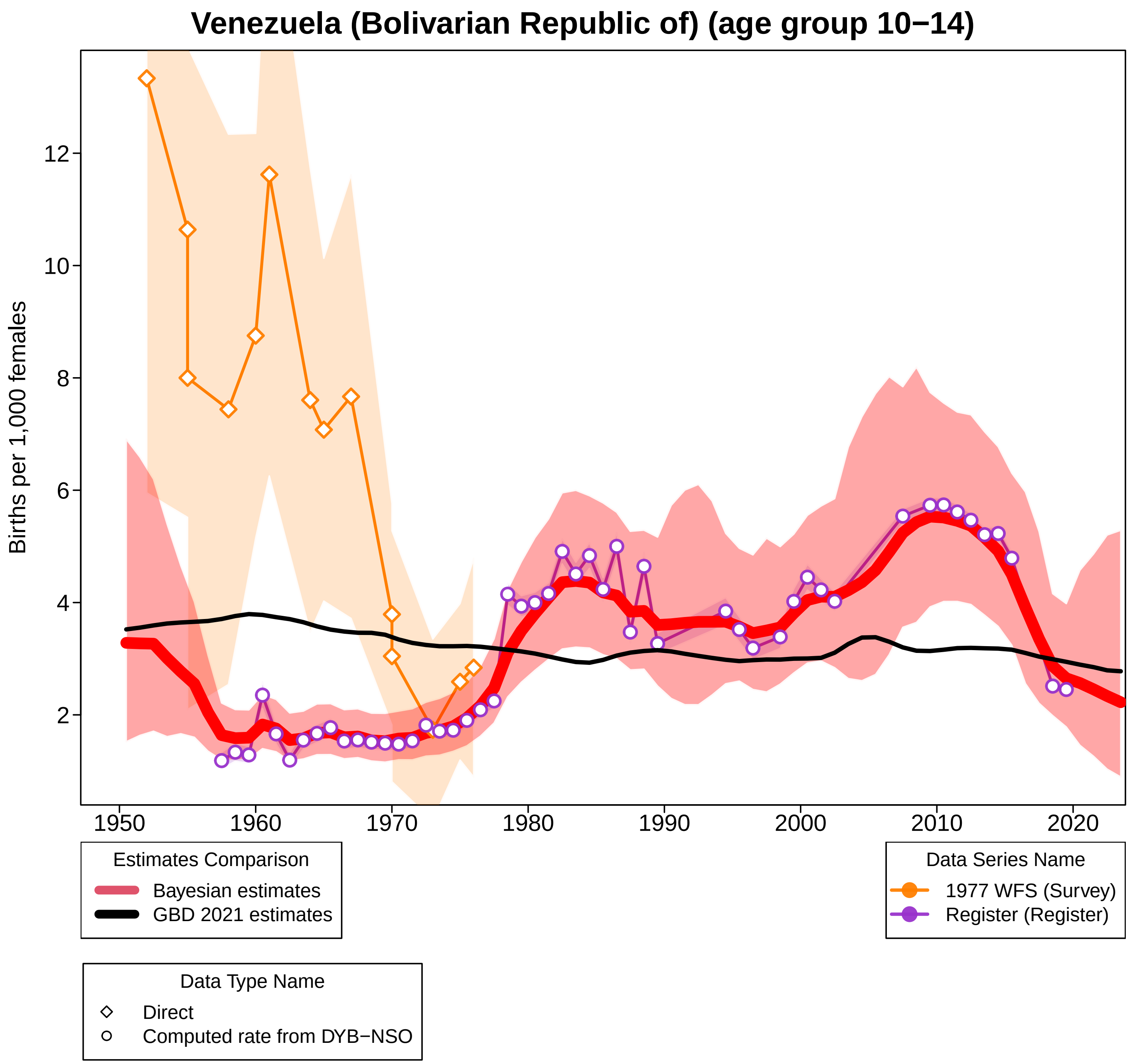

Venezuela (Bolivarian Republic of) (age group 10−14)
Births per 1,000 females
12
10
8
6
4
2
1950
1960
1970
1980
1990
2000
2010
2020
Estimates Comparison
Bayesian estimates
GBD 2021 estimates
Data Series Name
1977 WFS (Survey)
Register (Register)
Data Type Name
Direct
Computed rate from DYB−NSO

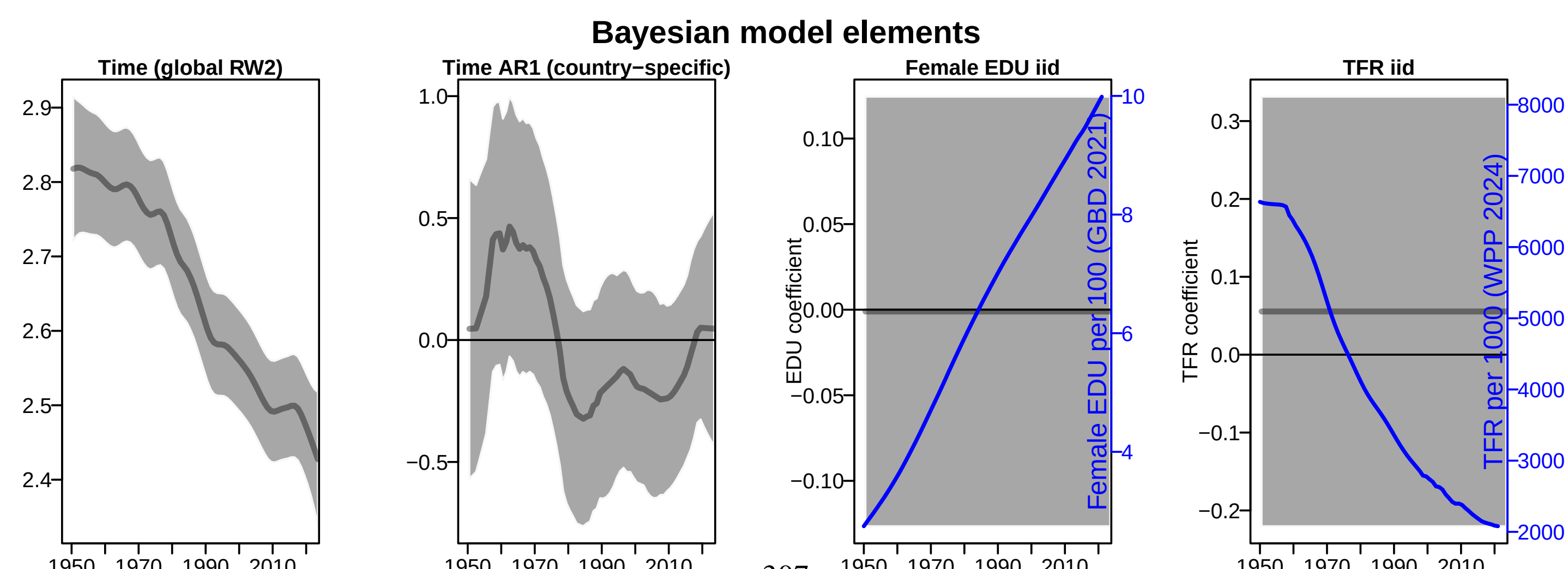

Bayesian model elements
Time (global RW2)
Time AR1 (country−specific)
Female EDU iid
TFR iid
EDU coefficient
Female EDU per 100 (GBD 2021)
TFR coefficient
TFR per 1000 (WPP 2024)

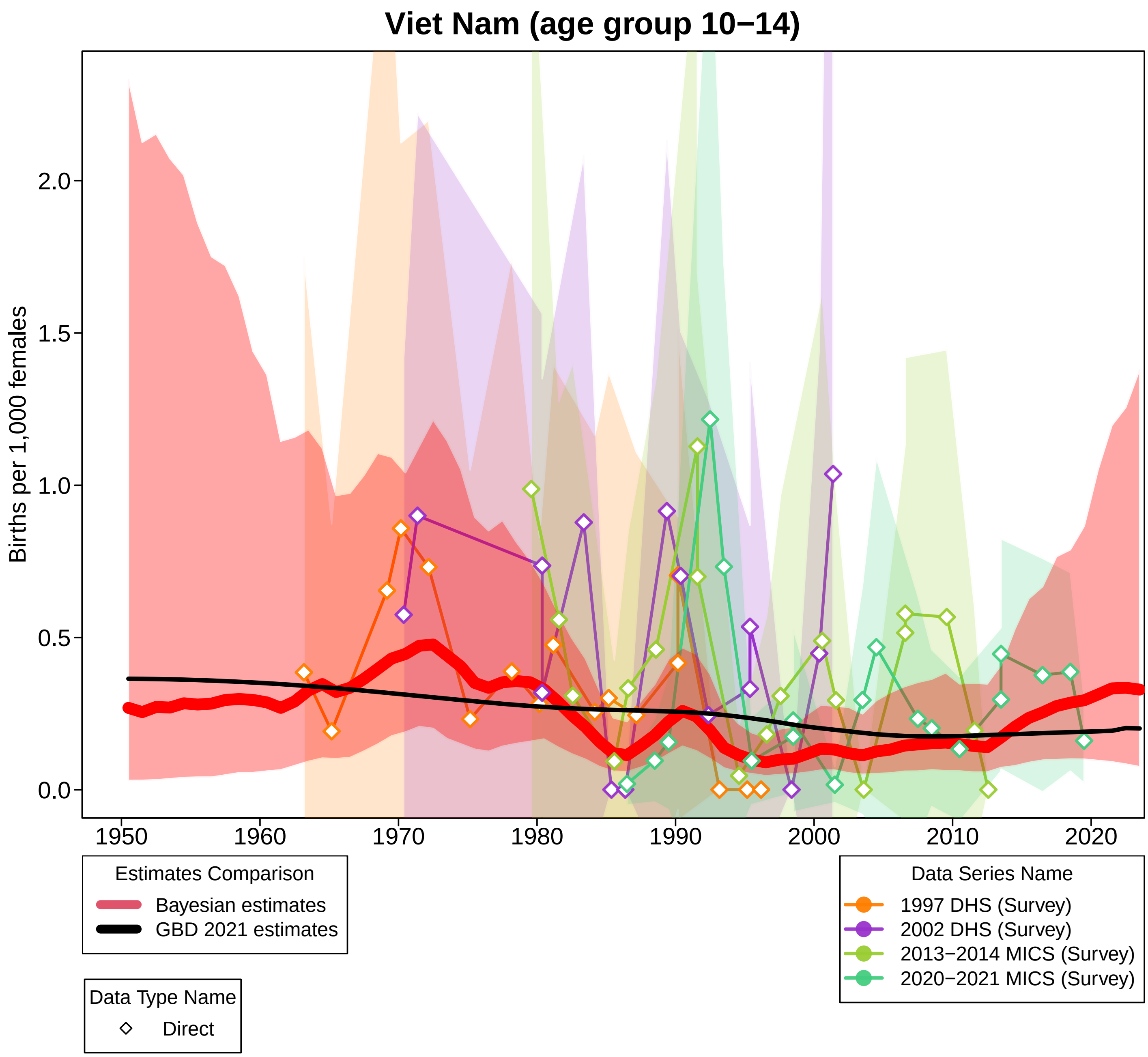
Viet Nam (age group 10−14)
Births per 1,000 females
2.0
1.5
1.0
0.5
0.0
1950
1960
1970
1980
1990
2000
2010
2020
Estimates Comparison
Bayesian estimates
GBD 2021 estimates
Data Type Name
Direct
Data Series Name
1997 DHS (Survey)
2002 DHS (Survey)
2013−2014 MICS (Survey)
2020−2021 MICS (Survey)

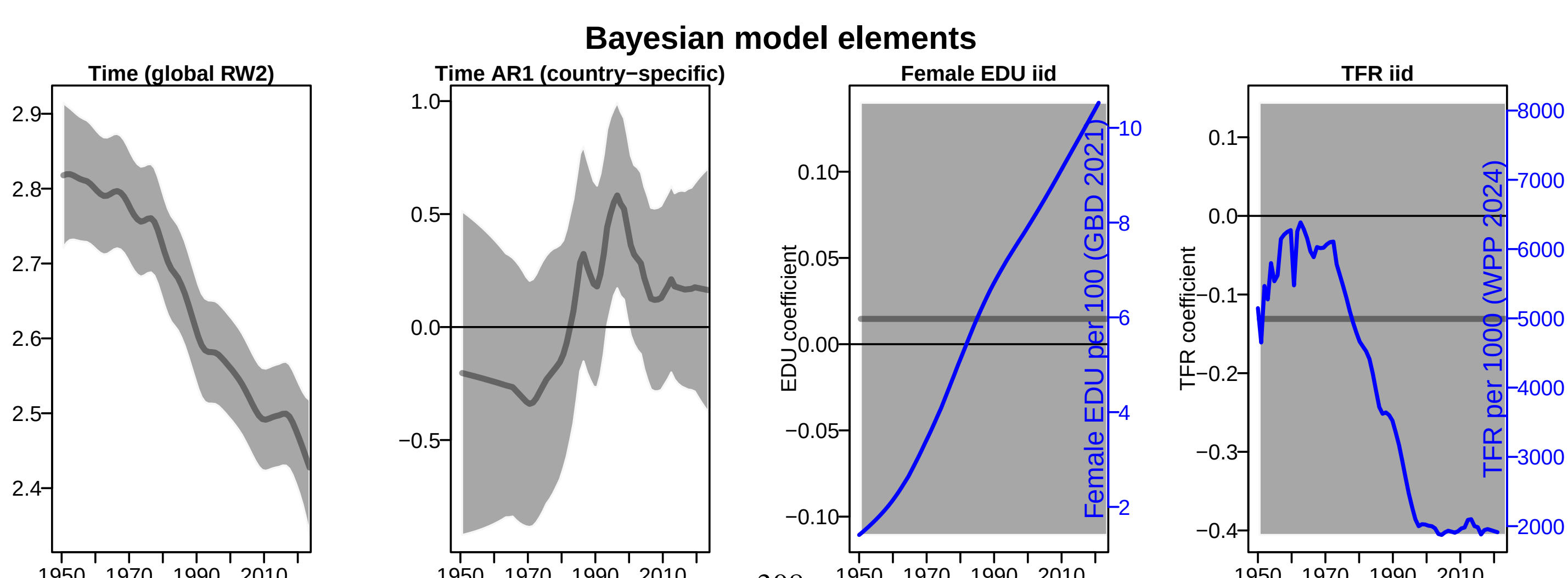
Bayesian model elements
Time (global RW2)
Time AR1 (country−specific)
Female EDU iid
TFR iid
EDU coefficient
Female EDU per 100 (GBD 2021)
TFR coefficient
TFR per 1000 (WPP 2024)
1950
1970
1990
2010

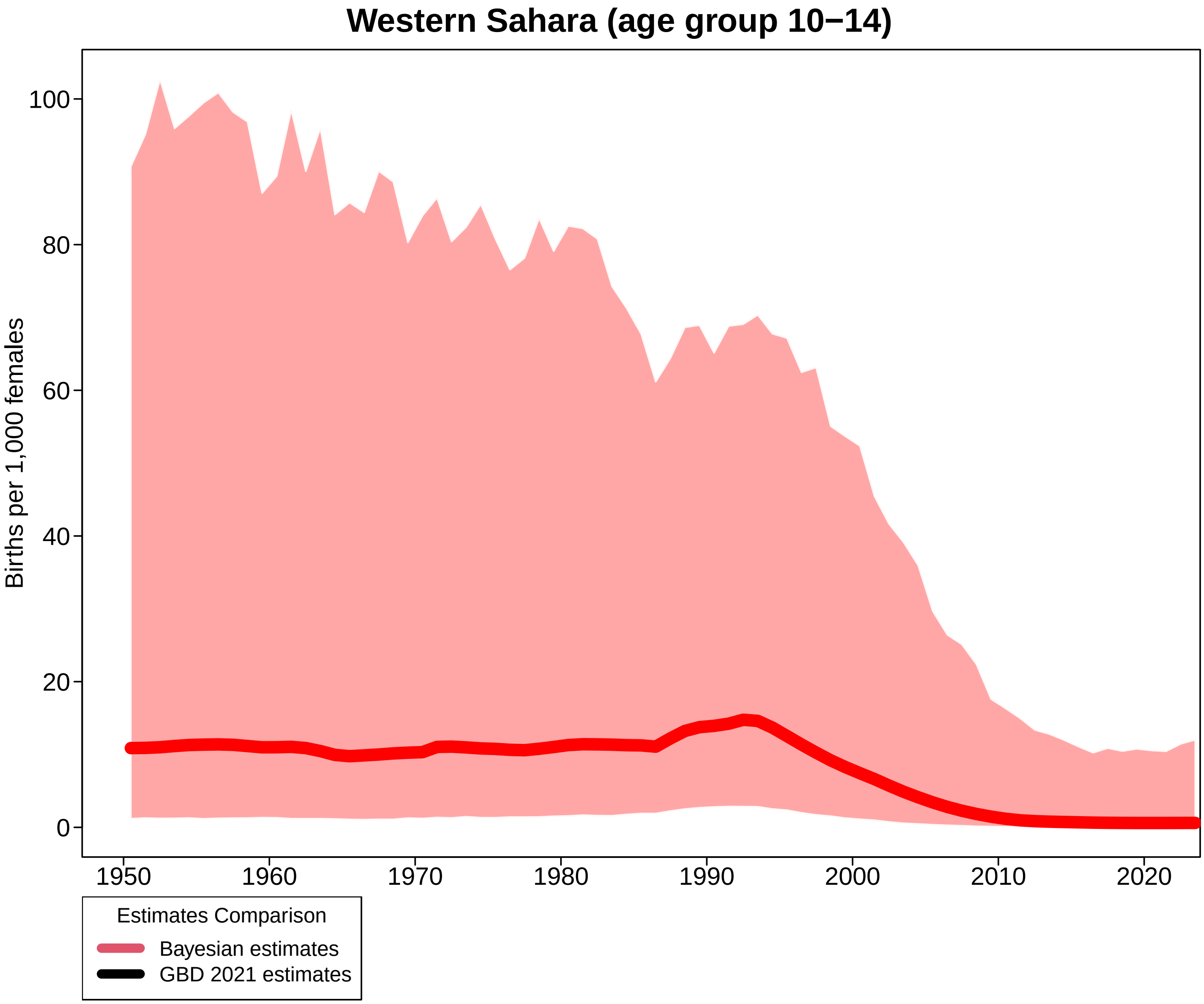
Western Sahara (age group 10−14)
Births per 1,000 females
0
20
40
60
80
100
1950
1960
1970
1980
1990
2000
2010
2020
Estimates Comparison
Bayesian estimates
GBD 2021 estimates

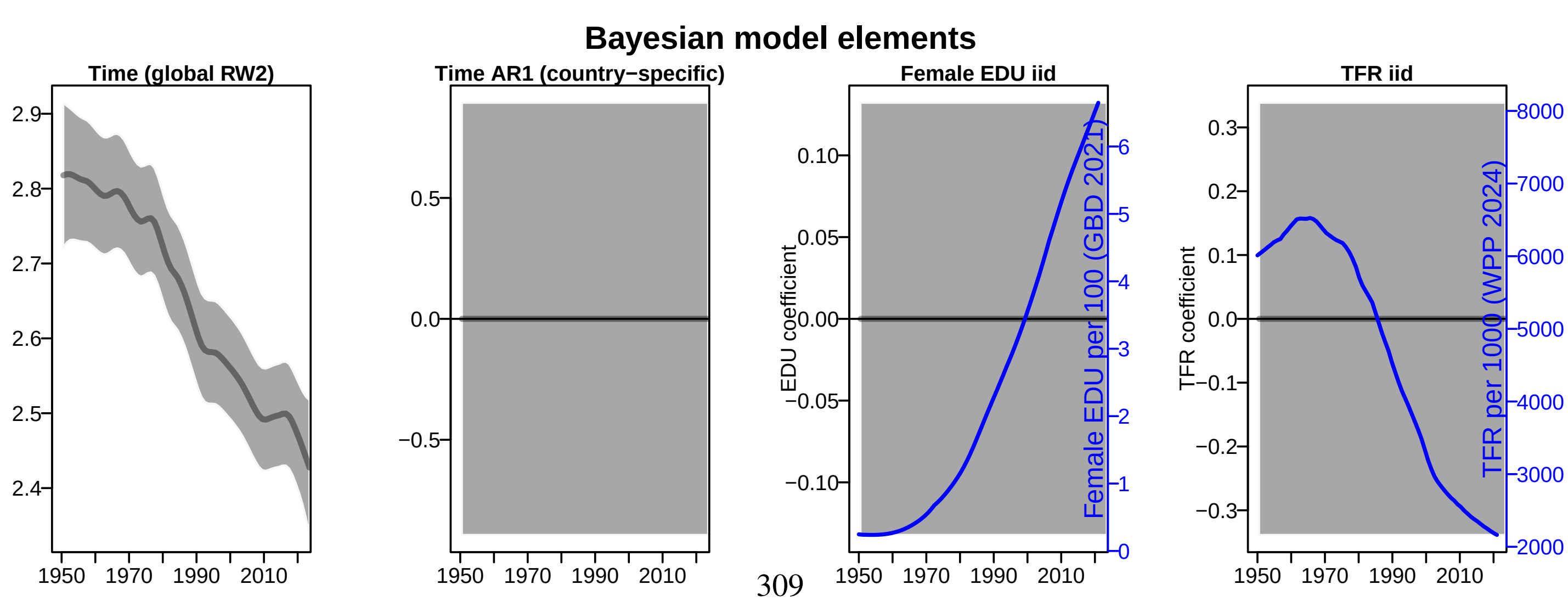
Bayesian model elements
Time (global RW2)
Time AR1 (country−specific)
Female EDU iid
TFR iid
EDU coefficient
Female EDU per 100 (GBD 2021)
TFR coefficient
TFR per 1000 (WPP 2024)
1950
1970
1990
2010

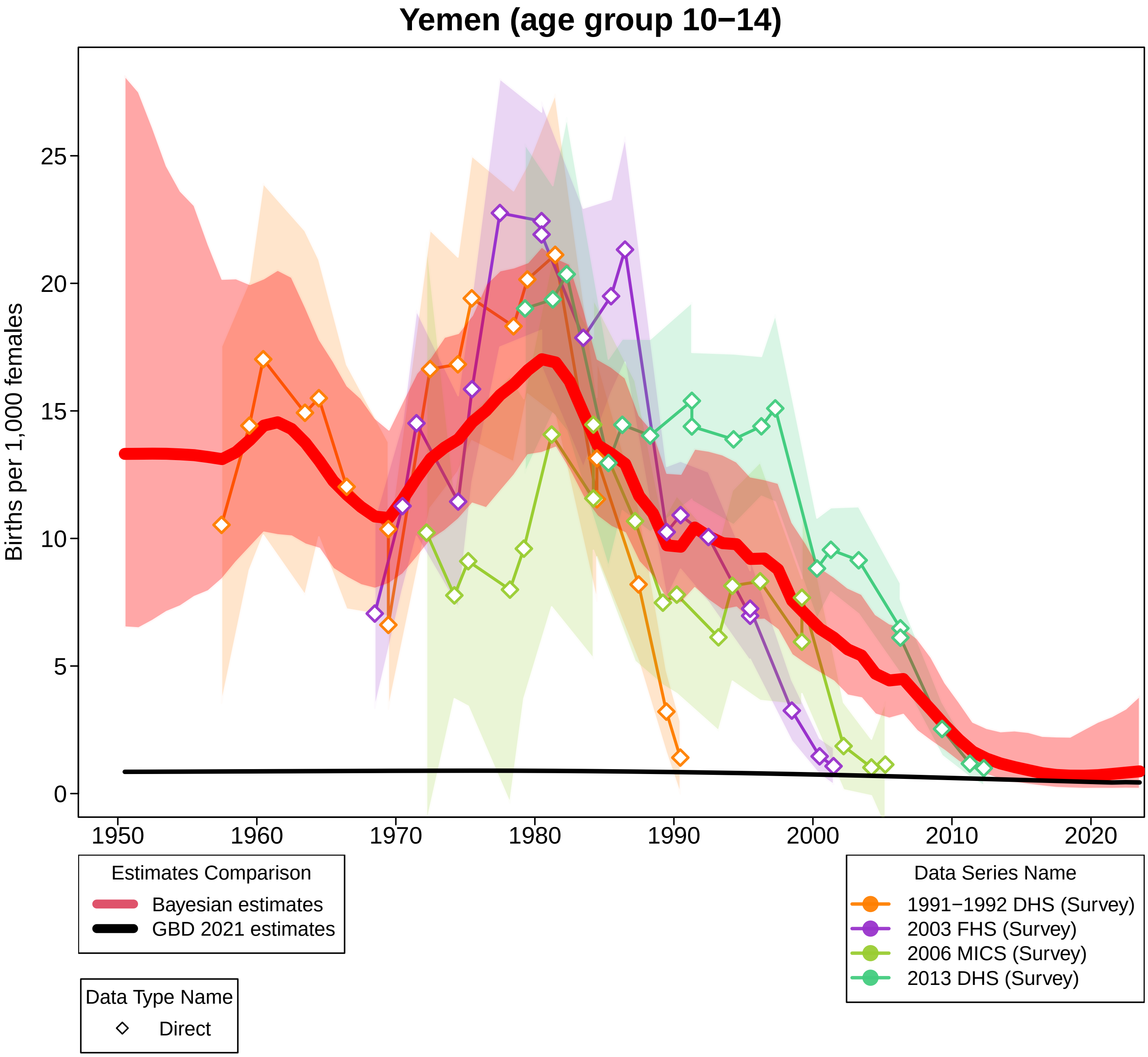

Yemen (age group 10−14)
Births per 1,000 females
0
5
10
15
20
25
1950
1960
1970
1980
1990
2000
2010
2020
Estimates Comparison
Bayesian estimates
GBD 2021 estimates
Data Series Name
1991−1992 DHS (Survey)
2003 FHS (Survey)
2006 MICS (Survey)
2013 DHS (Survey)
Data Type Name
Direct

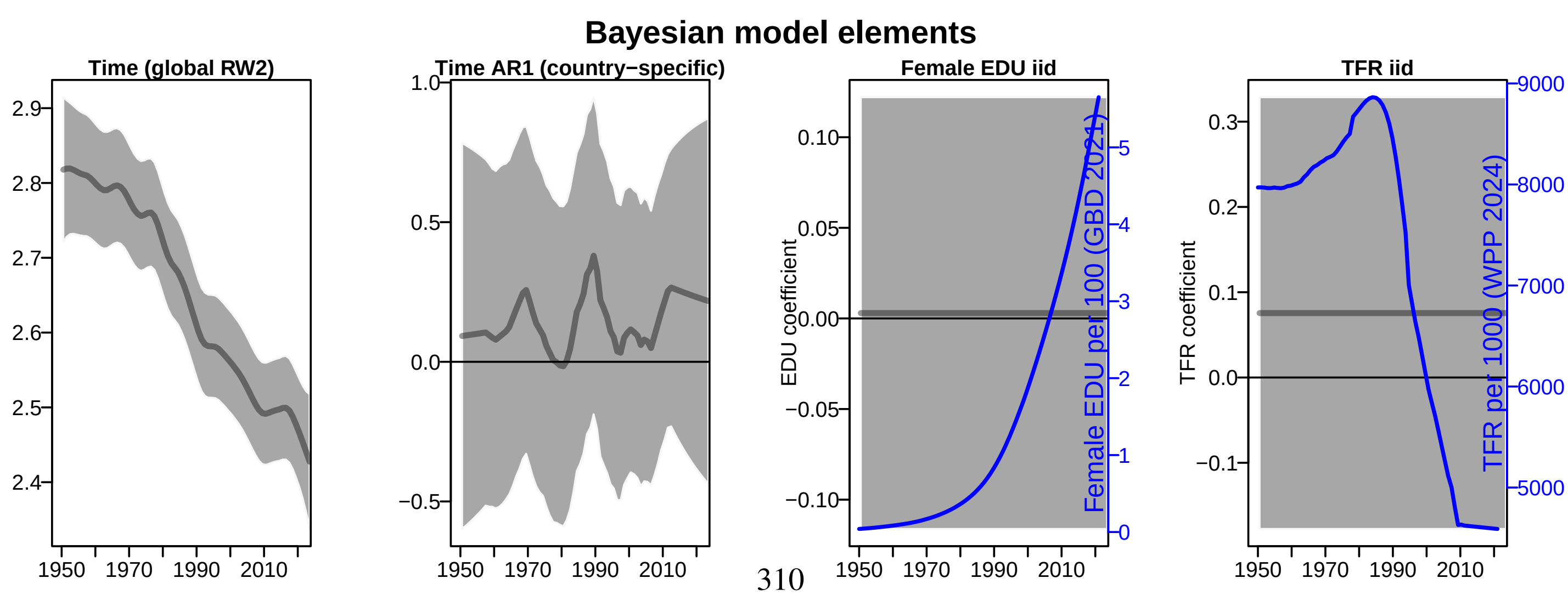

Bayesian model elements
Time (global RW2)
Time AR1 (country−specific)
Female EDU iid
TFR iid
EDU coefficient
Female EDU per 100 (GBD 2021)
TFR coefficient
TFR per 1000 (WPP 2024)
1950
1970
1990
2010

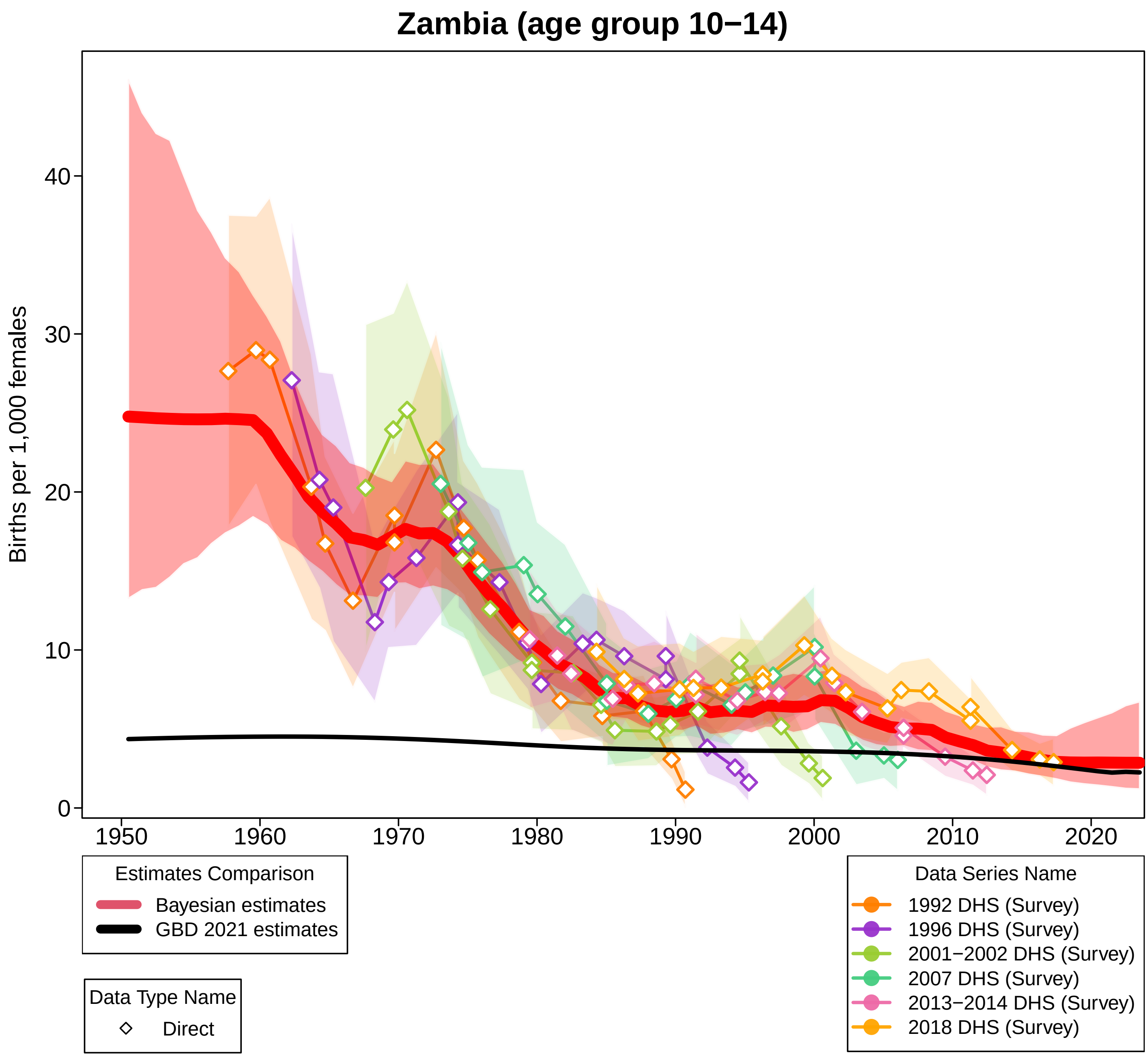
Zambia (age group 10−14)
Births per 1,000 females
0
10
20
30
40
1950
1960
1970
1980
1990
2000
2010
2020
Estimates Comparison
Bayesian estimates
GBD 2021 estimates
Data Type Name
Direct
Data Series Name
1992 DHS (Survey)
1996 DHS (Survey)
2001−2002 DHS (Survey)
2007 DHS (Survey)
2013−2014 DHS (Survey)
2018 DHS (Survey)

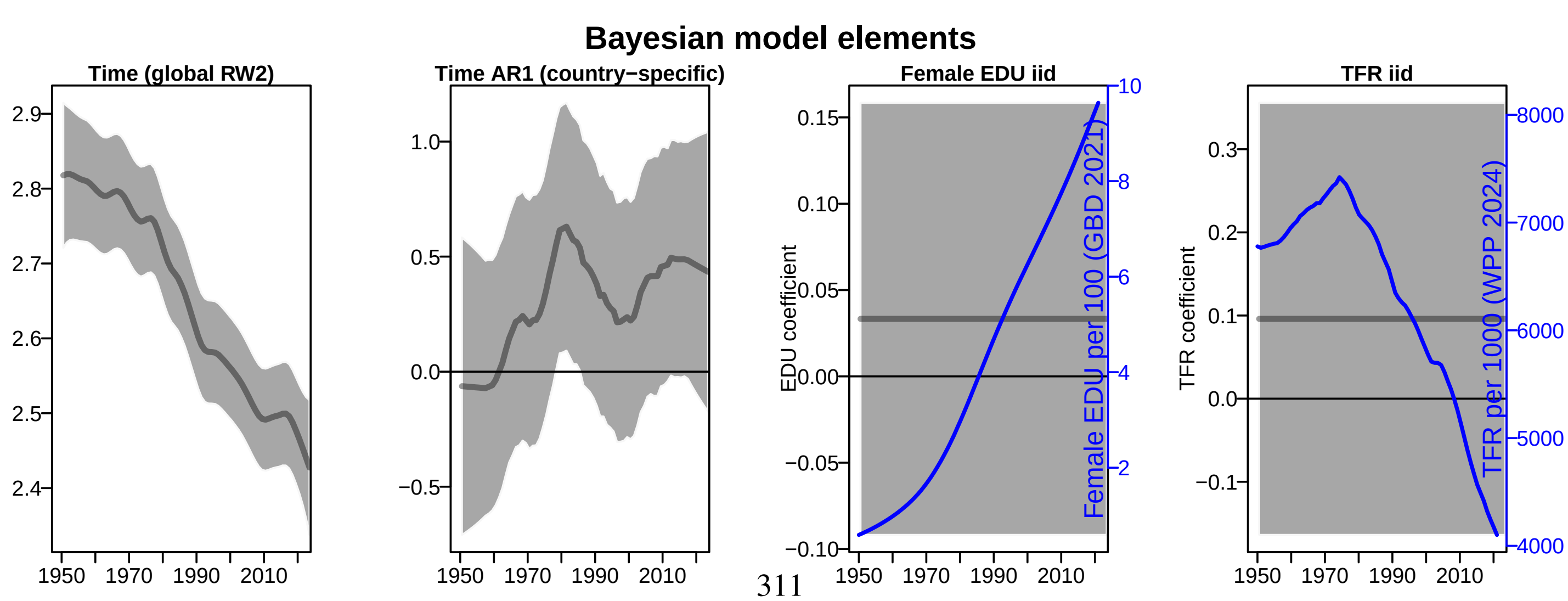
Bayesian model elements
Time (global RW2)
Time AR1 (country−specific)
Female EDU iid
TFR iid
EDU coefficient
Female EDU per 100 (GBD 2021)
TFR coefficient
TFR per 1000 (WPP 2024)
1950
1970
1990
2010

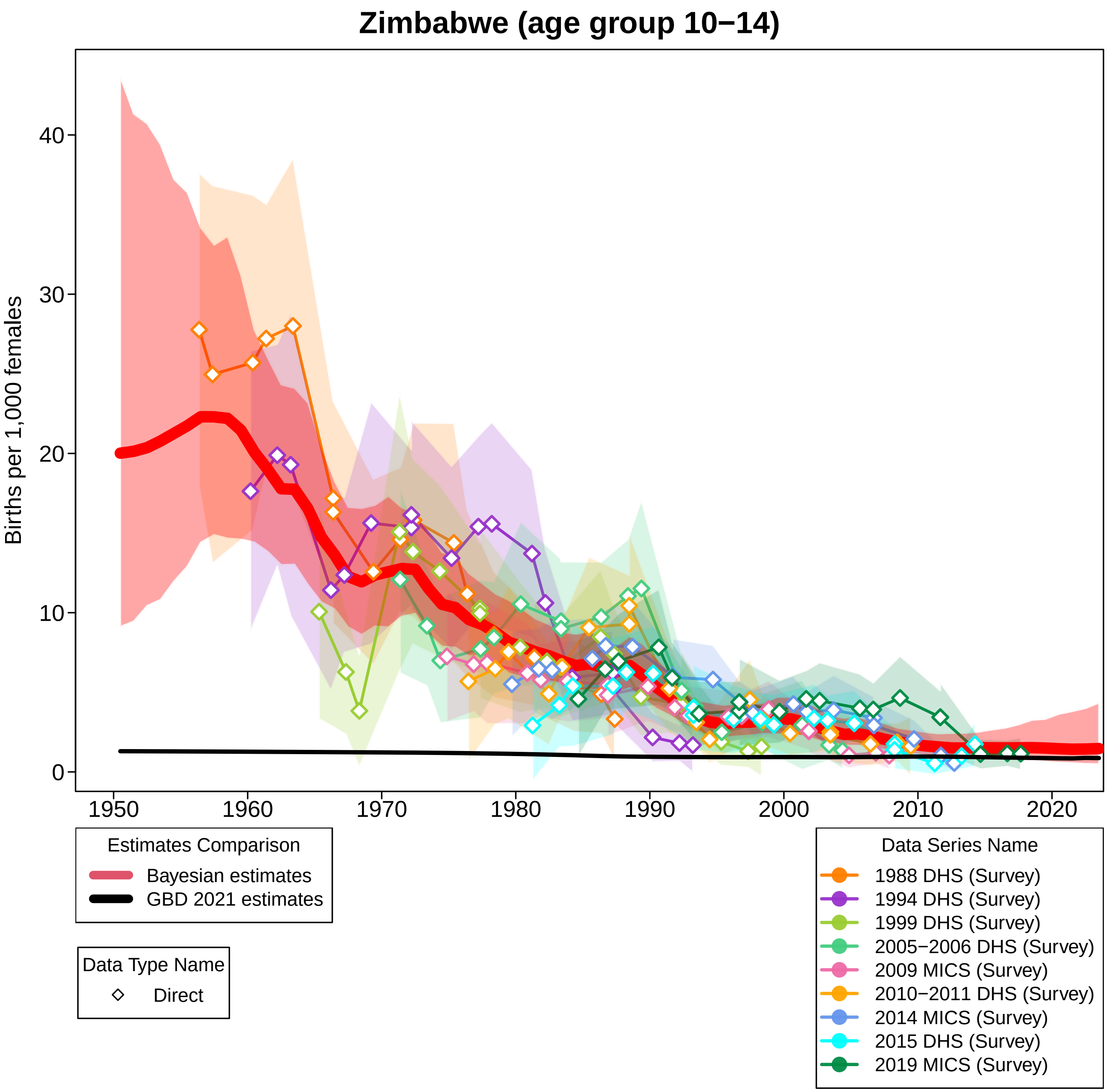
Zimbabwe (age group 10−14)
Births per 1,000 females
0
10
20
30
40
1950
1960
1970
1980
1990
2000
2010
2020
Estimates Comparison
Bayesian estimates
GBD 2021 estimates
Data Type Name
Direct
Data Series Name
1988 DHS (Survey)
1994 DHS (Survey)
1999 DHS (Survey)
2005−2006 DHS (Survey)
2009 MICS (Survey)
2010−2011 DHS (Survey)
2014 MICS (Survey)
2015 DHS (Survey)
2019 MICS (Survey)

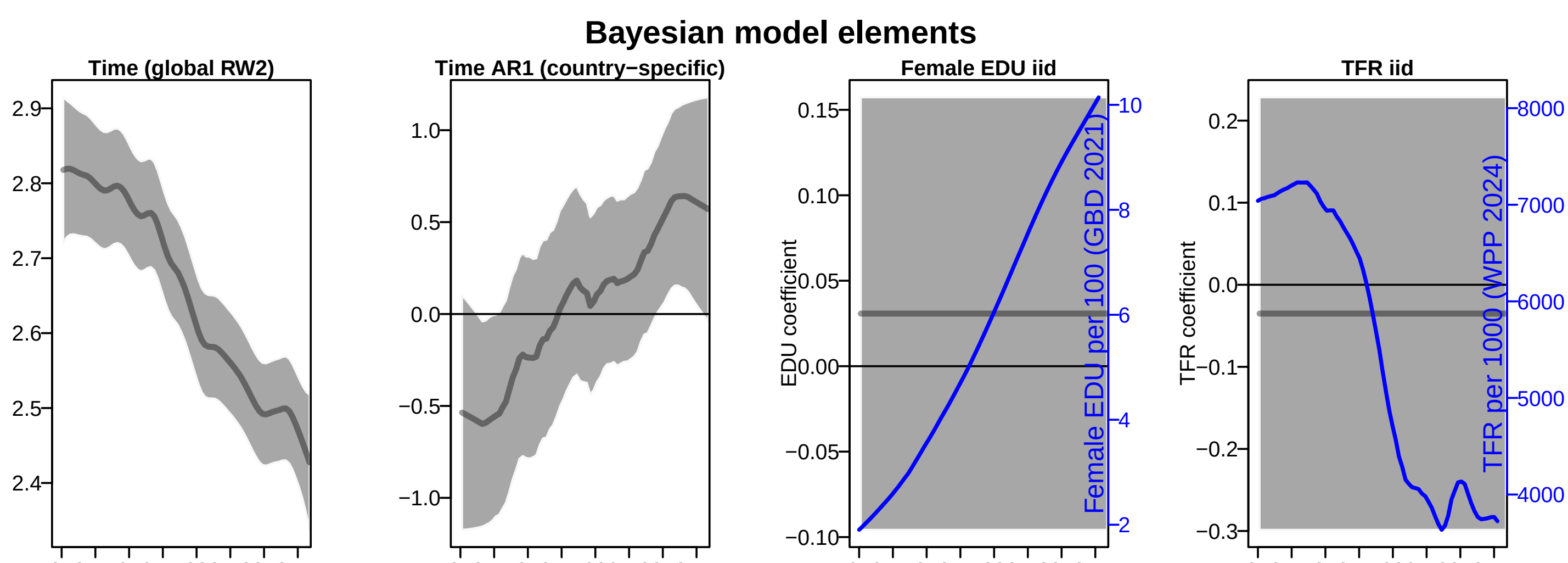
Bayesian model elements
Time (global RW2)
Time AR1 (country−specific)
Female EDU iid
TFR iid
EDU coefficient
Female EDU per 100 (GBD 2021)
TFR coefficient
TFR per 1000 (WPP 2024)
1950
1970
1990
2010

Figure 16: **ASFR 15–19 estimates and uncertainty intervals during 1950–2023, by country.** Red curves and shades: ASFR 15–19 median estimates and 95% uncertainty intervals. Black curves: GBD 2021 estimates. Dots with connection lines: input data series differentiated by colors. Shades around the data series: sampling errors (for non-VR data) or stochastic errors (for VR data). Bayesian model elements results are shown at the bottom of each page.

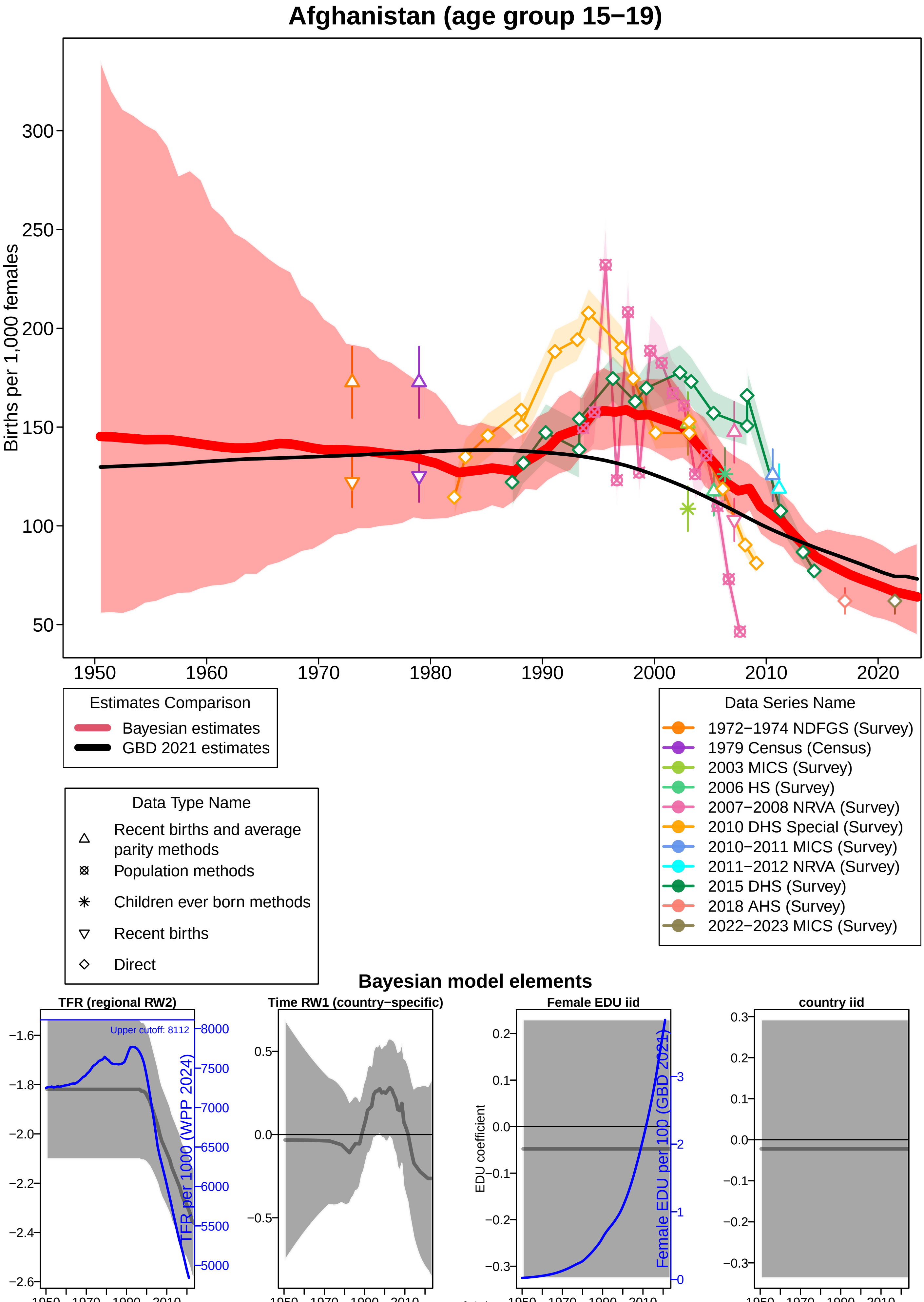

Afghanistan (age group 15−19)
Births per 1,000 females
300
250
200
150
100
50
1950
1960
1970
1980
1990
2000
2010
2020
Estimates Comparison
Bayesian estimates
GBD 2021 estimates
Data Series Name
1972−1974 NDFGS (Survey)
1979 Census (Census)
2003 MICS (Survey)
2006 HS (Survey)
2007−2008 NRVA (Survey)
2010 DHS Special (Survey)
2010−2011 MICS (Survey)
2011−2012 NRVA (Survey)
2015 DHS (Survey)
2018 AHS (Survey)
2022−2023 MICS (Survey)
Data Type Name
Recent births and average parity methods
Population methods
Children ever born methods
Recent births
Direct
Bayesian model elements
TFR (regional RW2)
Upper cutoff: 8112
TFR per 1000 (WPP 2024)
Time RW1 (country−specific)
Female EDU iid
EDU coefficient
Female EDU per 100 (GBD 2021)
country iid

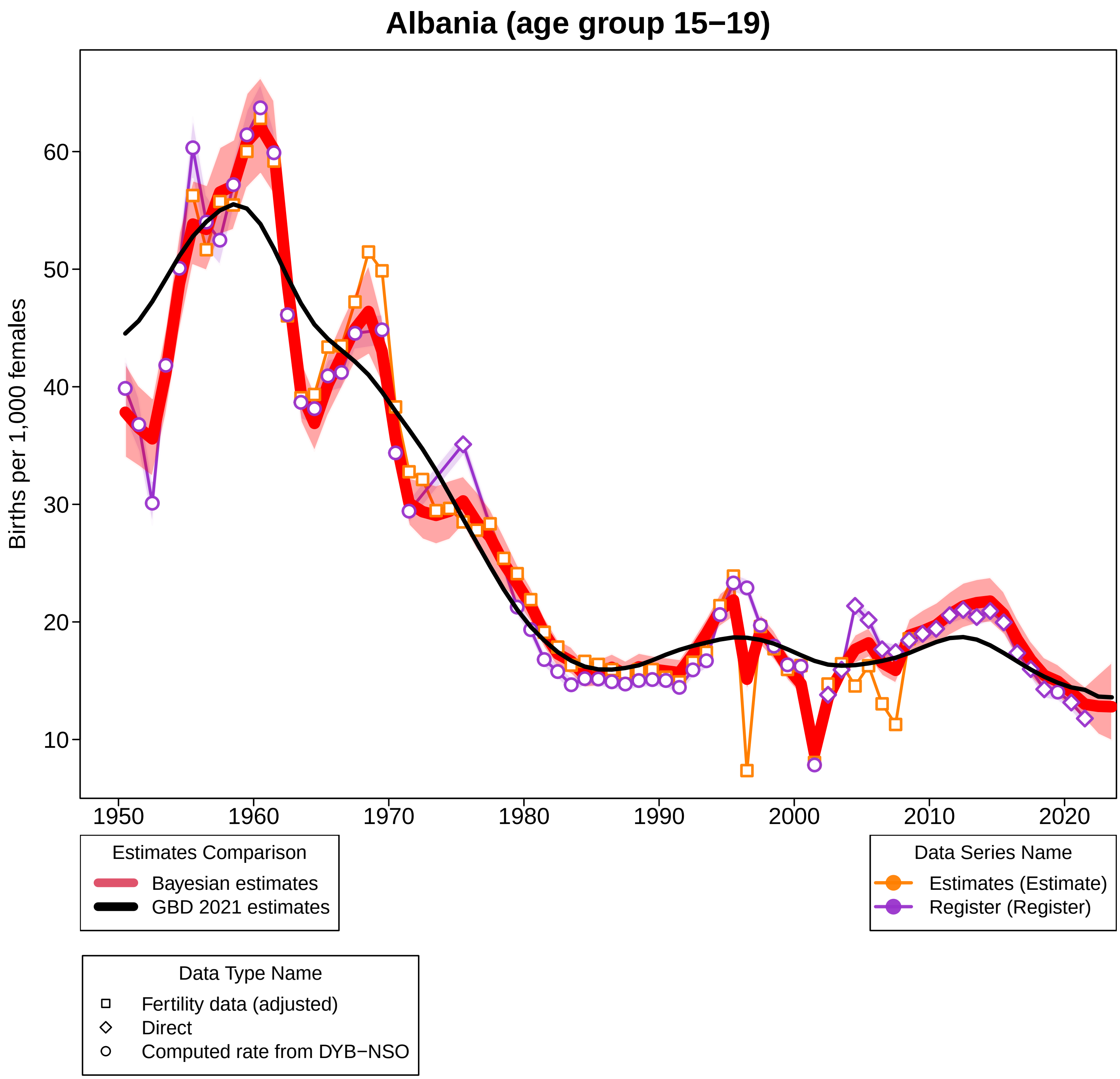
Albania (age group 15−19)
Births per 1,000 females
60
50
40
30
20
10
1950
1960
1970
1980
1990
2000
2010
2020
Estimates Comparison
Bayesian estimates
GBD 2021 estimates
Data Series Name
Estimates (Estimate)
Register (Register)
Data Type Name
Fertility data (adjusted)
Direct
Computed rate from DYB−NSO

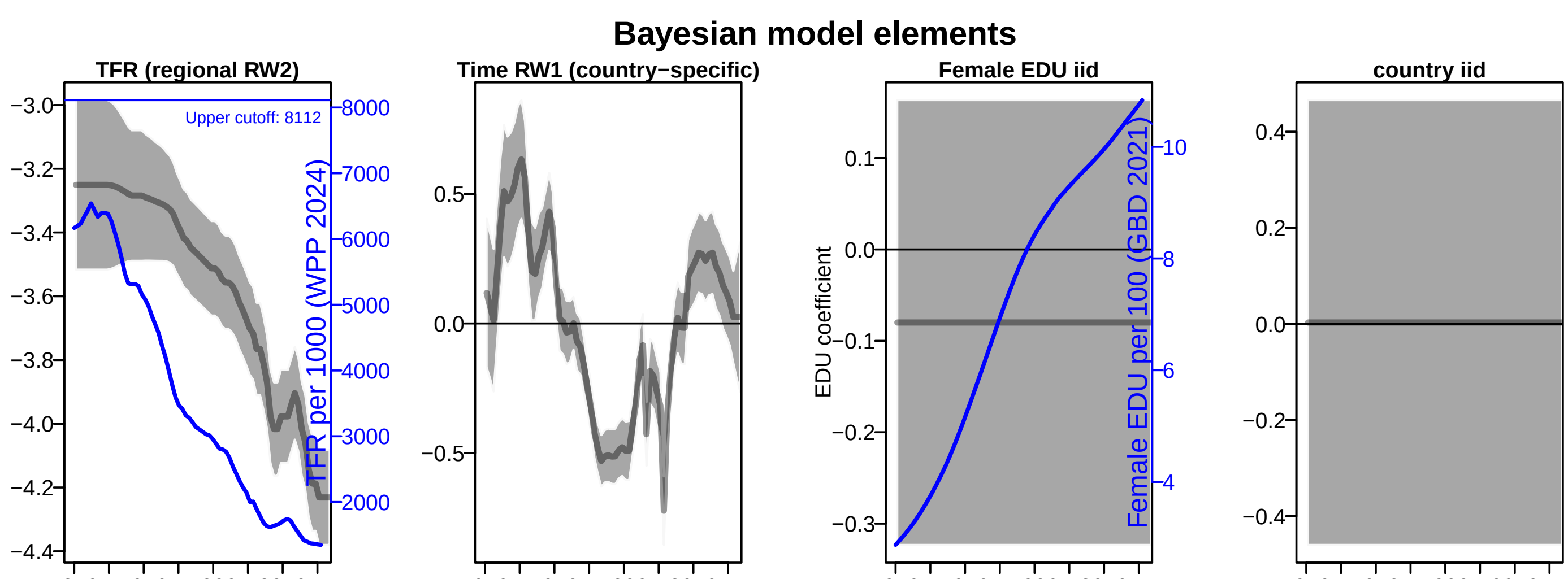
Bayesian model elements
TFR (regional RW2)
Upper cutoff: 8112
TFR per 1000 (WPP 2024)
Time RW1 (country−specific)
Female EDU iid
EDU coefficient
Female EDU per 100 (GBD 2021)
country iid

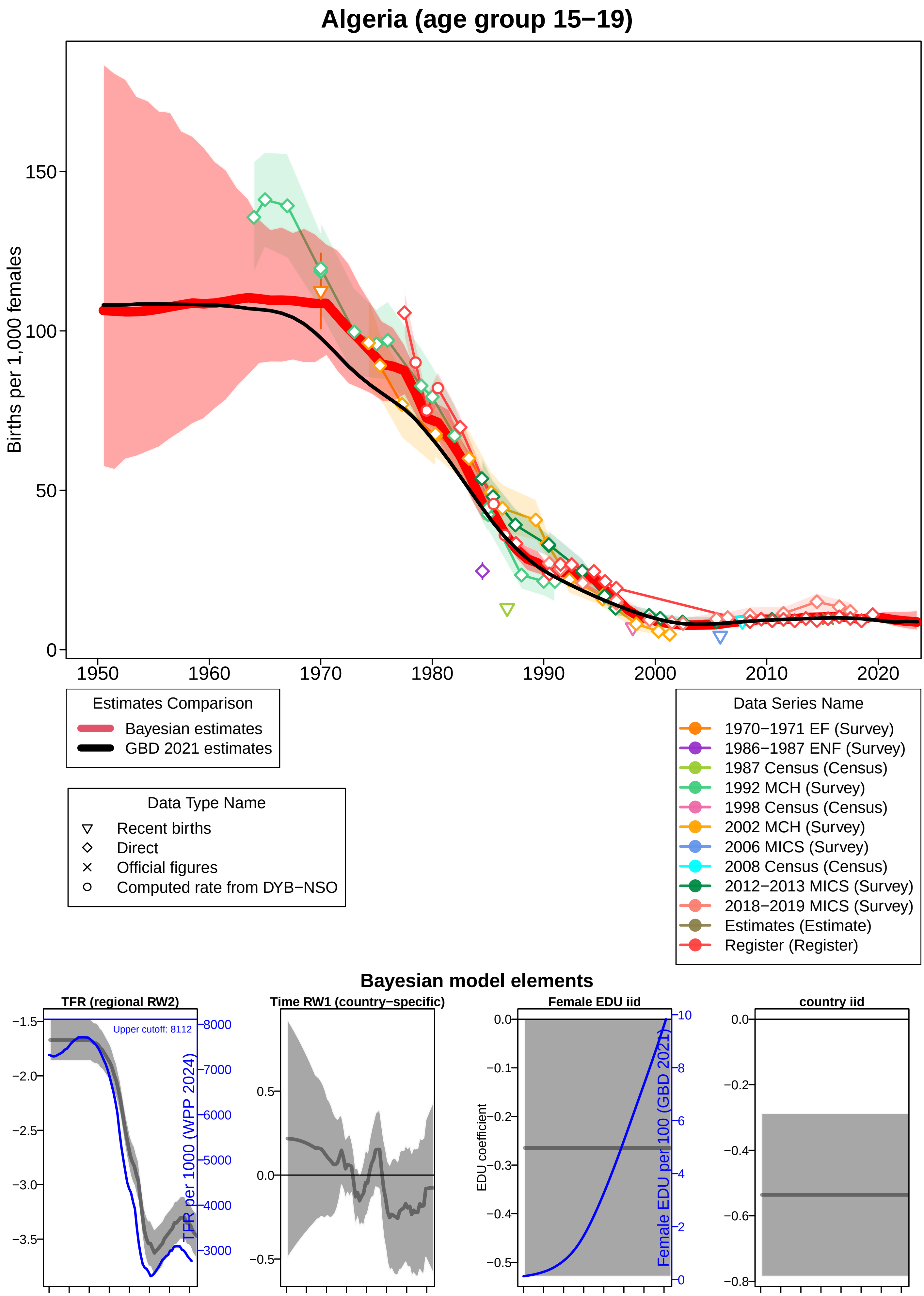
Algeria (age group 15−19)
Births per 1,000 females
Estimates Comparison
Bayesian estimates
GBD 2021 estimates
Data Type Name
Recent births
Direct
Official figures
Computed rate from DYB−NSO
Data Series Name
1970−1971 EF (Survey)
1986−1987 ENF (Survey)
1987 Census (Census)
1992 MCH (Survey)
1998 Census (Census)
2002 MCH (Survey)
2006 MICS (Survey)
2008 Census (Census)
2012−2013 MICS (Survey)
2018−2019 MICS (Survey)
Estimates (Estimate)
Register (Register)
Bayesian model elements
TFR (regional RW2)
Upper cutoff: 8112
TFR per 1000 (WPP 2024)
Time RW1 (country−specific)
Female EDU iid
EDU coefficient
Female EDU per 100 (GBD 2021)
country iid

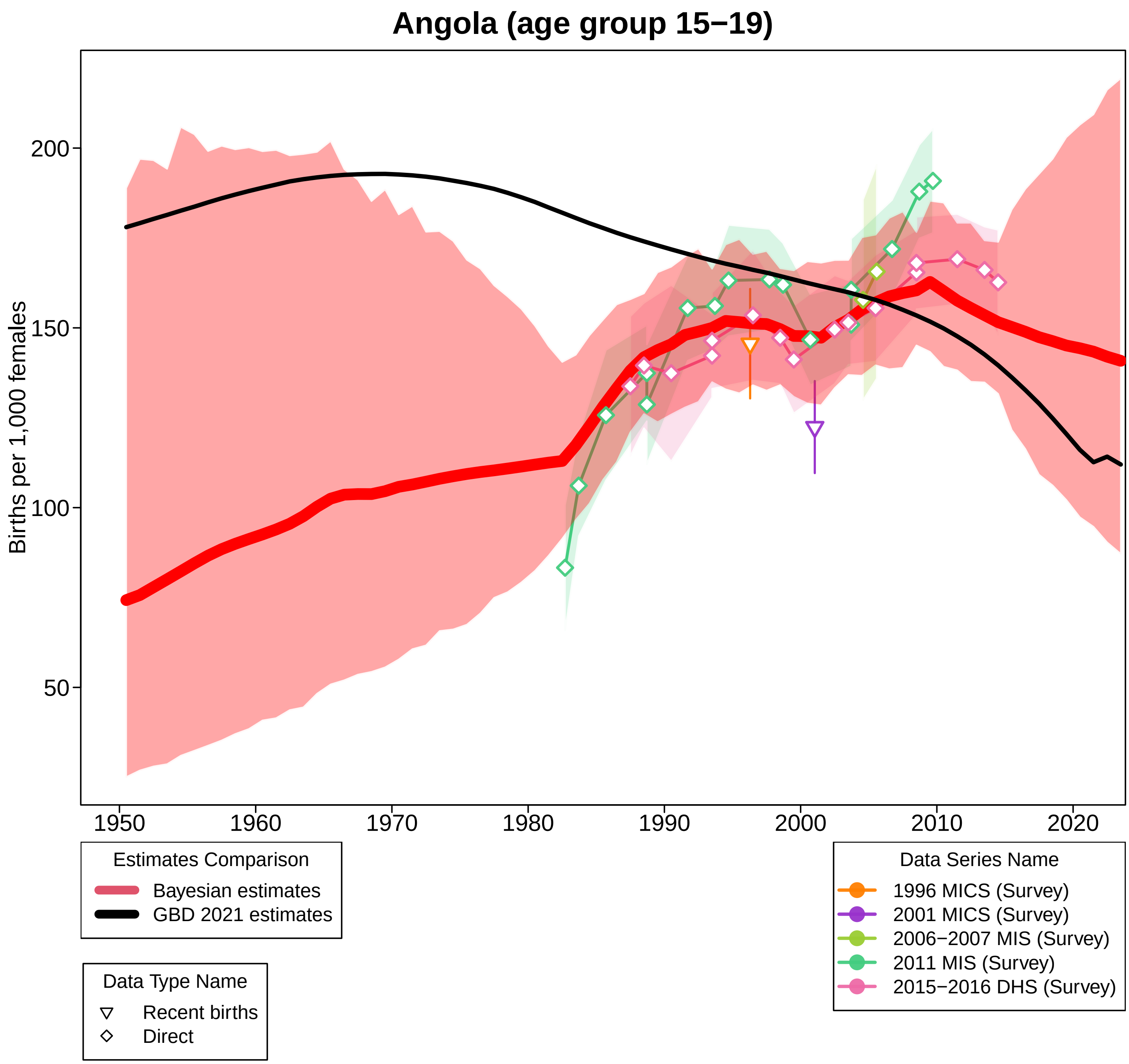

Angola (age group 15−19)
Births per 1,000 females
200
150
100
50
1950
1960
1970
1980
1990
2000
2010
2020
Estimates Comparison
Bayesian estimates
GBD 2021 estimates
Data Type Name
Recent births
Direct
Data Series Name
1996 MICS (Survey)
2001 MICS (Survey)
2006−2007 MIS (Survey)
2011 MIS (Survey)
2015−2016 DHS (Survey)

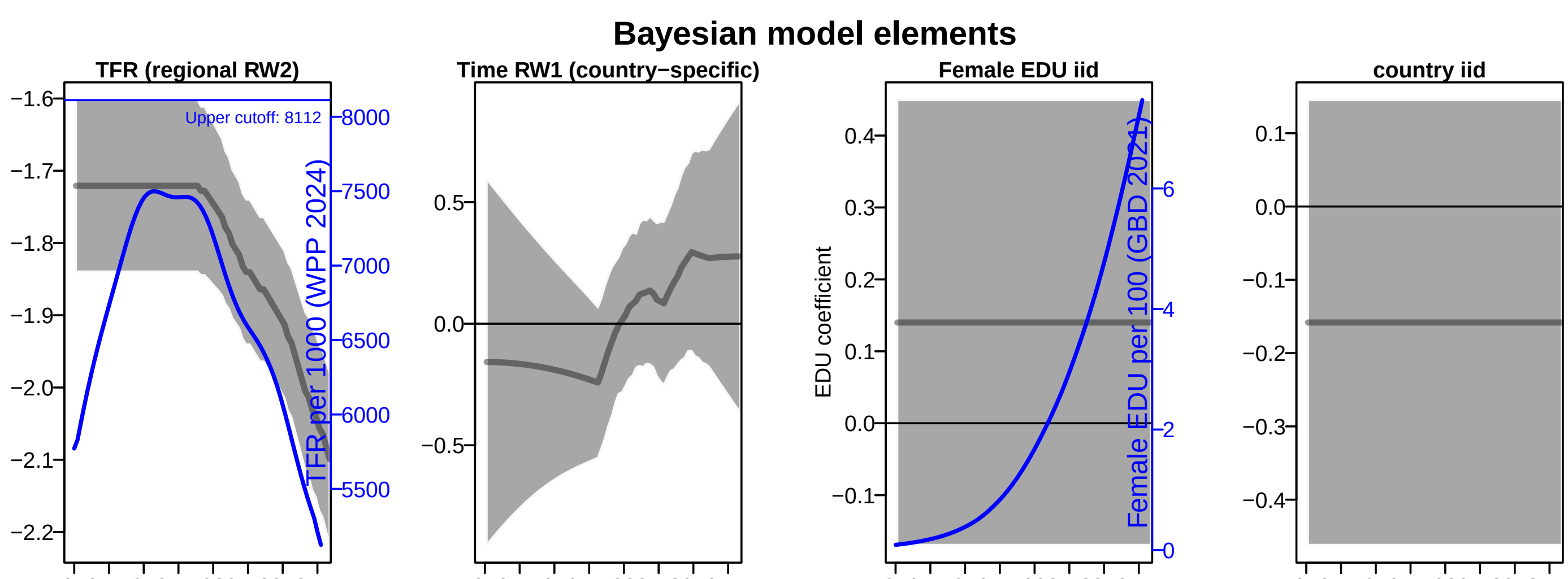

Bayesian model elements
TFR (regional RW2)
Upper cutoff: 8112
TFR per 1000 (WPP 2024)
Time RW1 (country−specific)
Female EDU iid
EDU coefficient
Female EDU per 100 (GBD 2021)
country iid
1950
1970
1990
2010

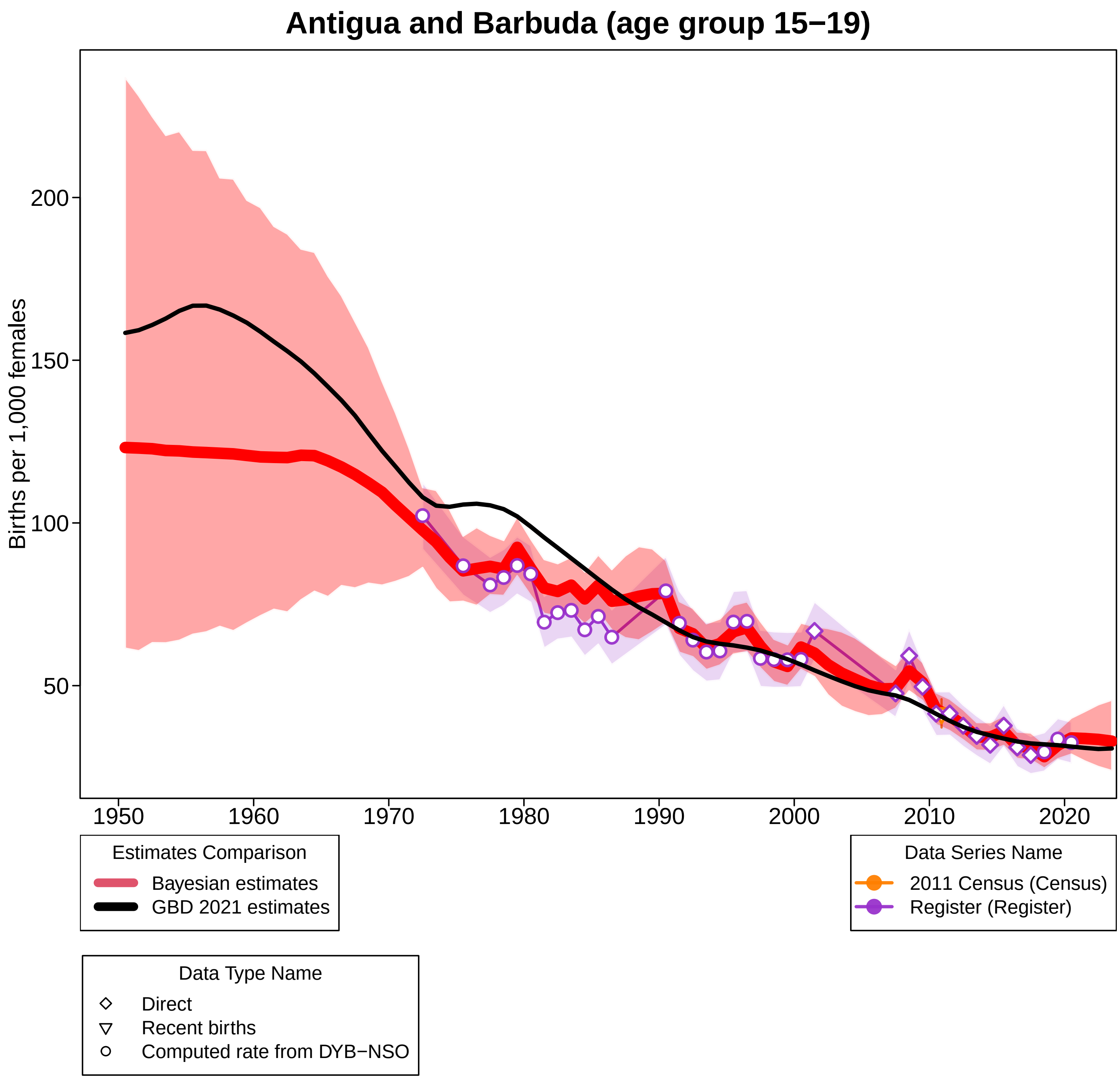

Antigua and Barbuda (age group 15−19)
Births per 1,000 females
200
150
100
50
1950
1960
1970
1980
1990
2000
2010
2020
Estimates Comparison
Bayesian estimates
GBD 2021 estimates
Data Series Name
2011 Census (Census)
Register (Register)
Data Type Name
Direct
Recent births
Computed rate from DYB−NSO

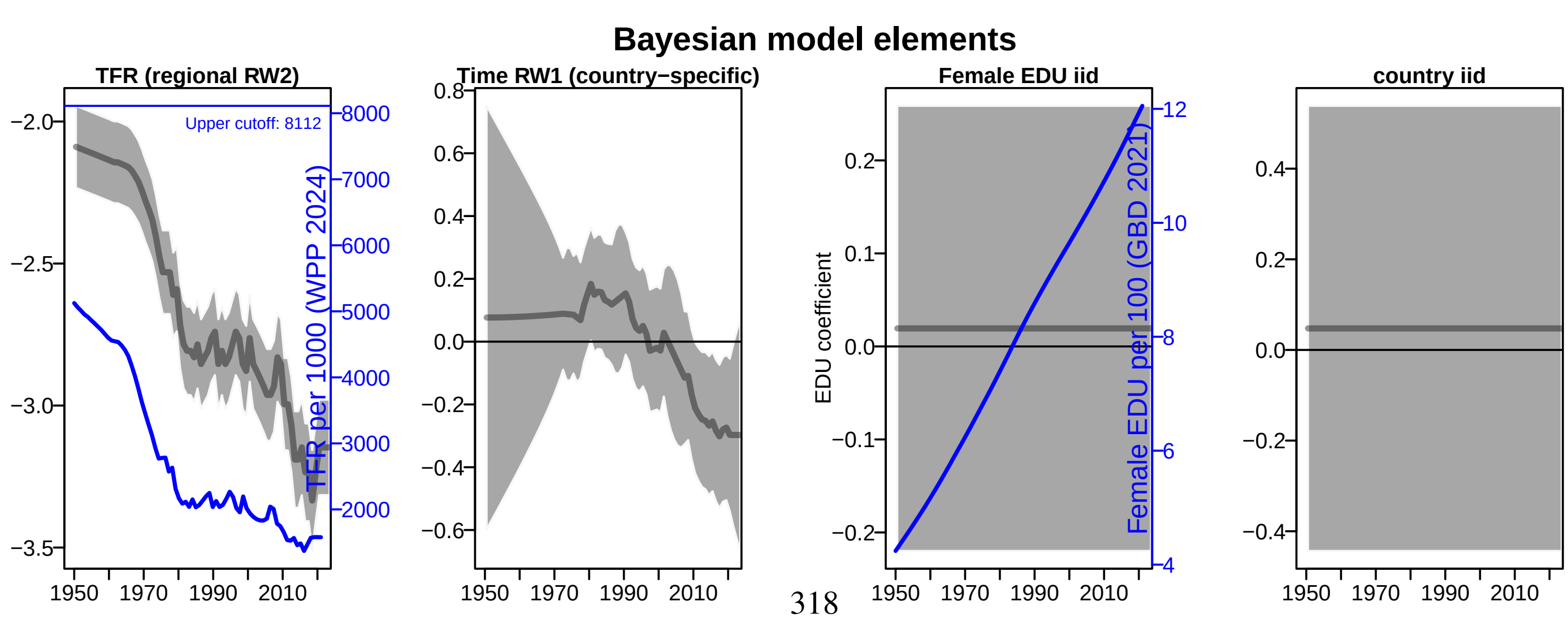

Bayesian model elements
TFR (regional RW2)
Upper cutoff: 8112
TFR per 1000 (WPP 2024)
Time RW1 (country−specific)
Female EDU iid
EDU coefficient
Female EDU per 100 (GBD 2021)
country iid

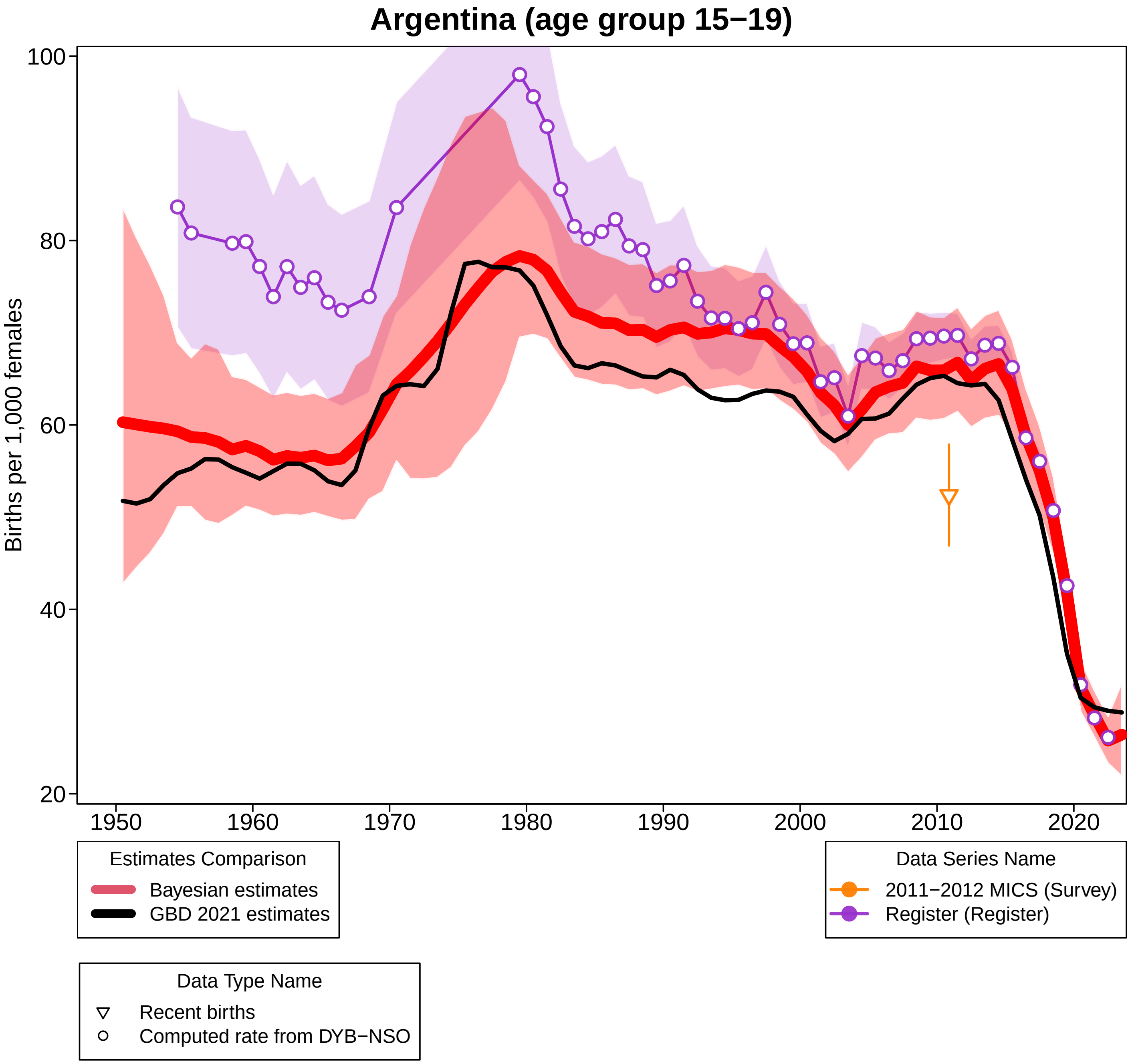
Argentina (age group 15−19)
Births per 1,000 females
100
80
60
40
20
1950
1960
1970
1980
1990
2000
2010
2020
Estimates Comparison
Bayesian estimates
GBD 2021 estimates
Data Series Name
2011−2012 MICS (Survey)
Register (Register)
Data Type Name
Recent births
Computed rate from DYB−NSO

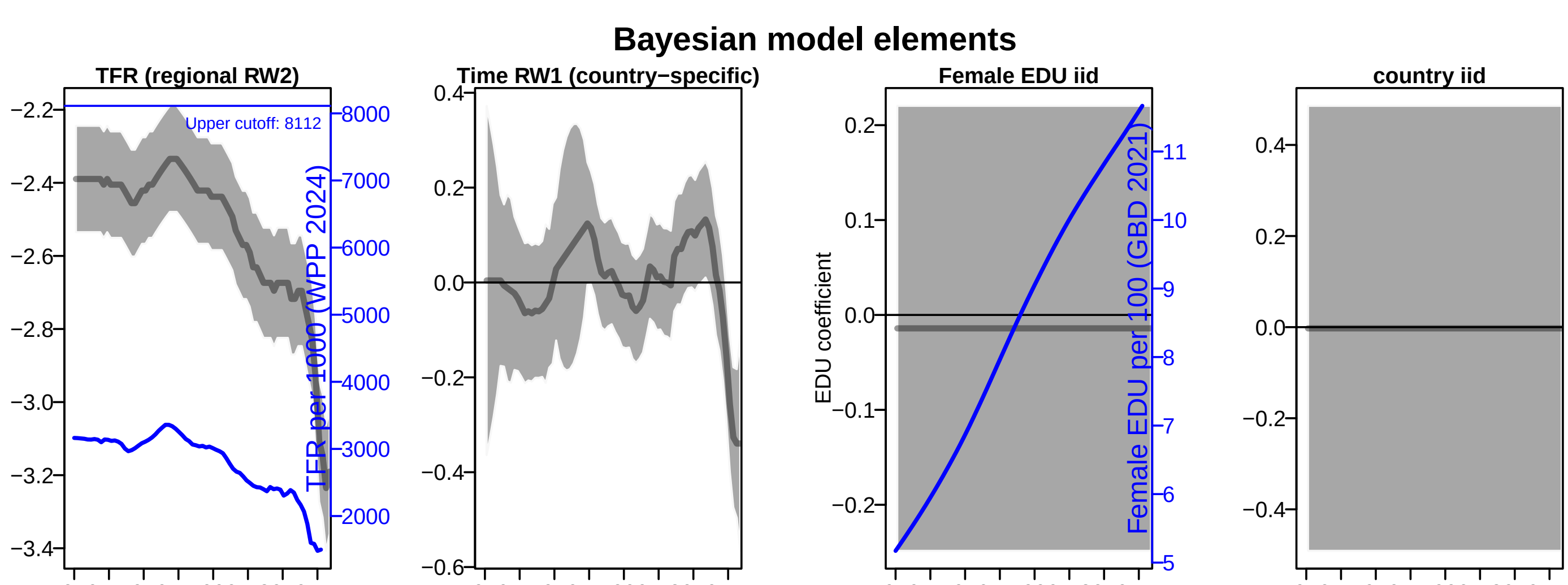
Bayesian model elements
TFR (regional RW2)
Upper cutoff: 8112
TFR per 1000 (WPP 2024)
Time RW1 (country−specific)
Female EDU iid
EDU coefficient
Female EDU per 100 (GBD 2021)
country iid
1950
1970
1990
2010

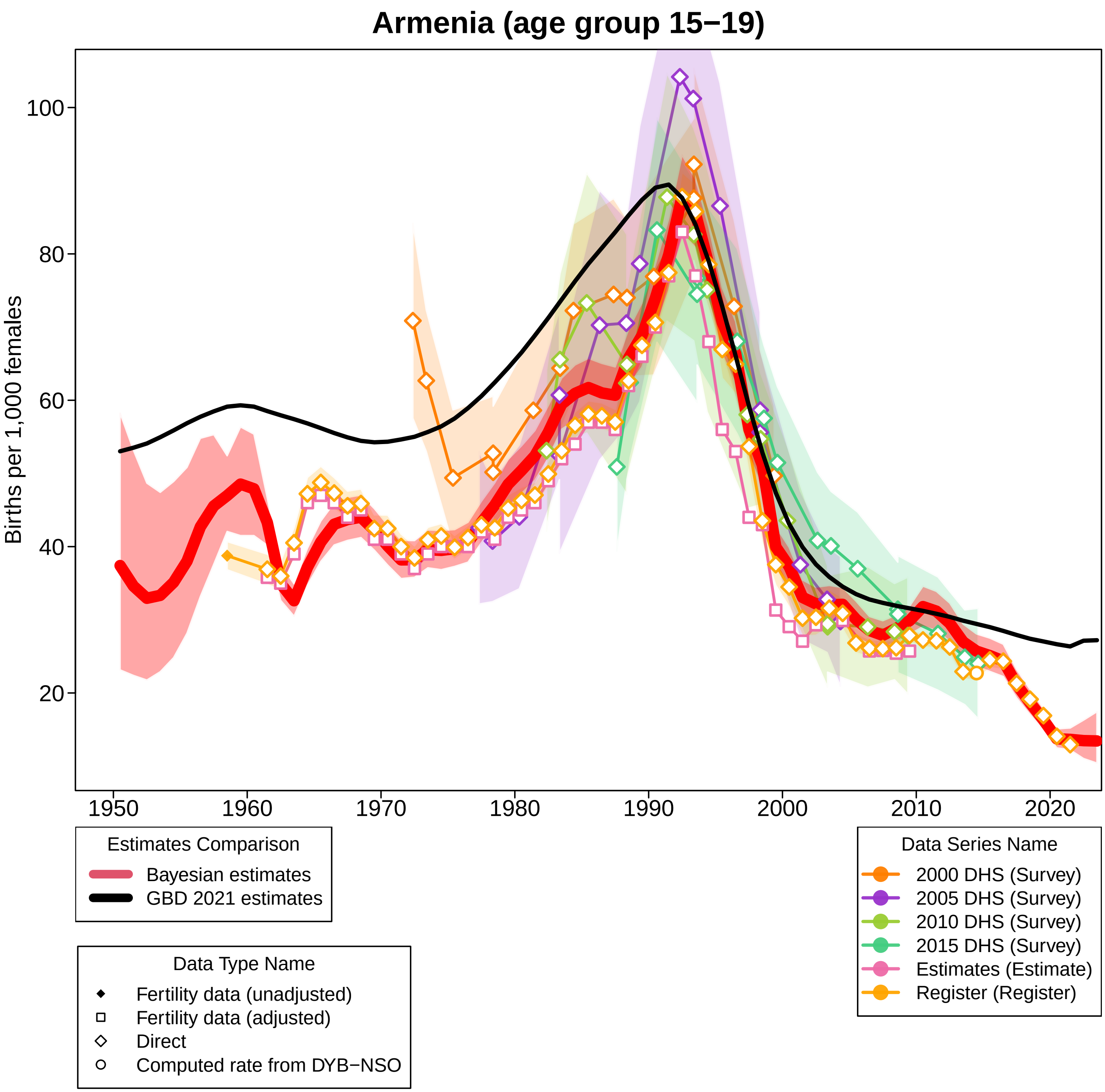
Armenia (age group 15−19)
Births per 1,000 females
100
80
60
40
20
1950
1960
1970
1980
1990
2000
2010
2020
Estimates Comparison
Bayesian estimates
GBD 2021 estimates
Data Series Name
2000 DHS (Survey)
2005 DHS (Survey)
2010 DHS (Survey)
2015 DHS (Survey)
Estimates (Estimate)
Register (Register)
Data Type Name
Fertility data (unadjusted)
Fertility data (adjusted)
Direct
Computed rate from DYB−NSO

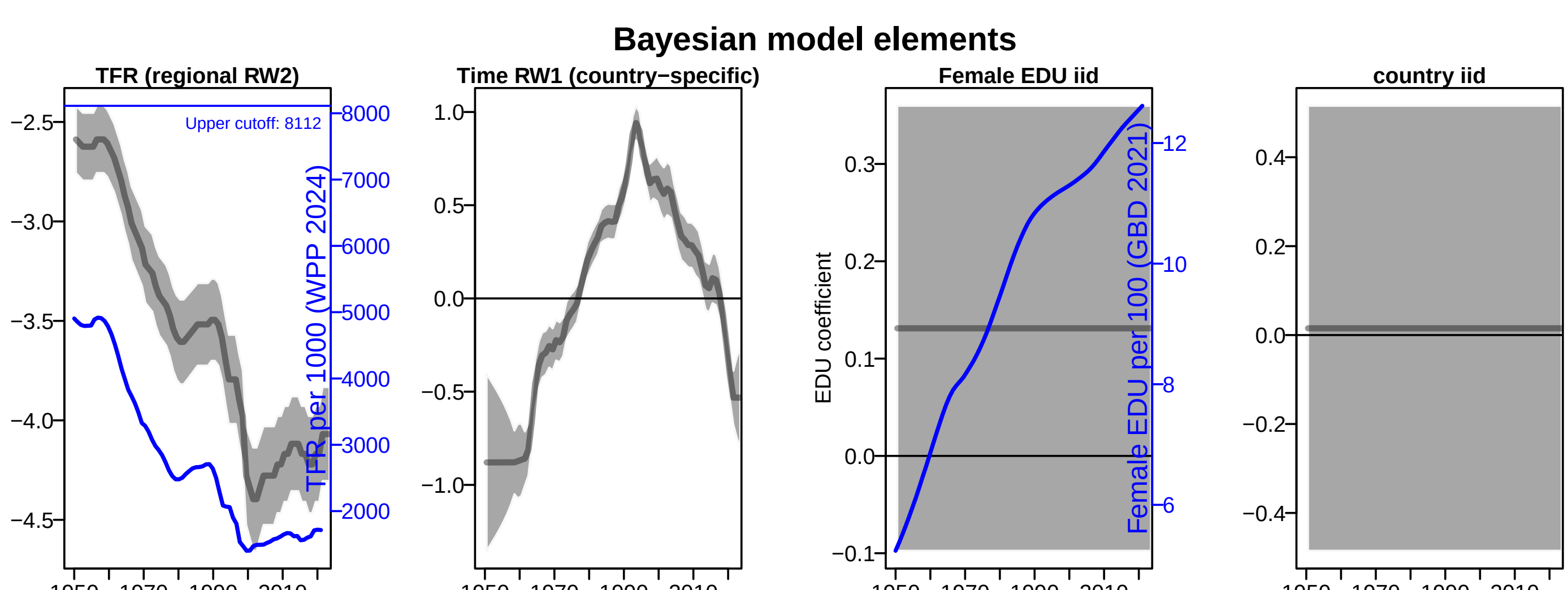
Bayesian model elements
TFR (regional RW2)
Upper cutoff: 8112
TFR per 1000 (WPP 2024)
Time RW1 (country−specific)
Female EDU iid
EDU coefficient
Female EDU per 100 (GBD 2021)
country iid
1950
1970
1990
2010

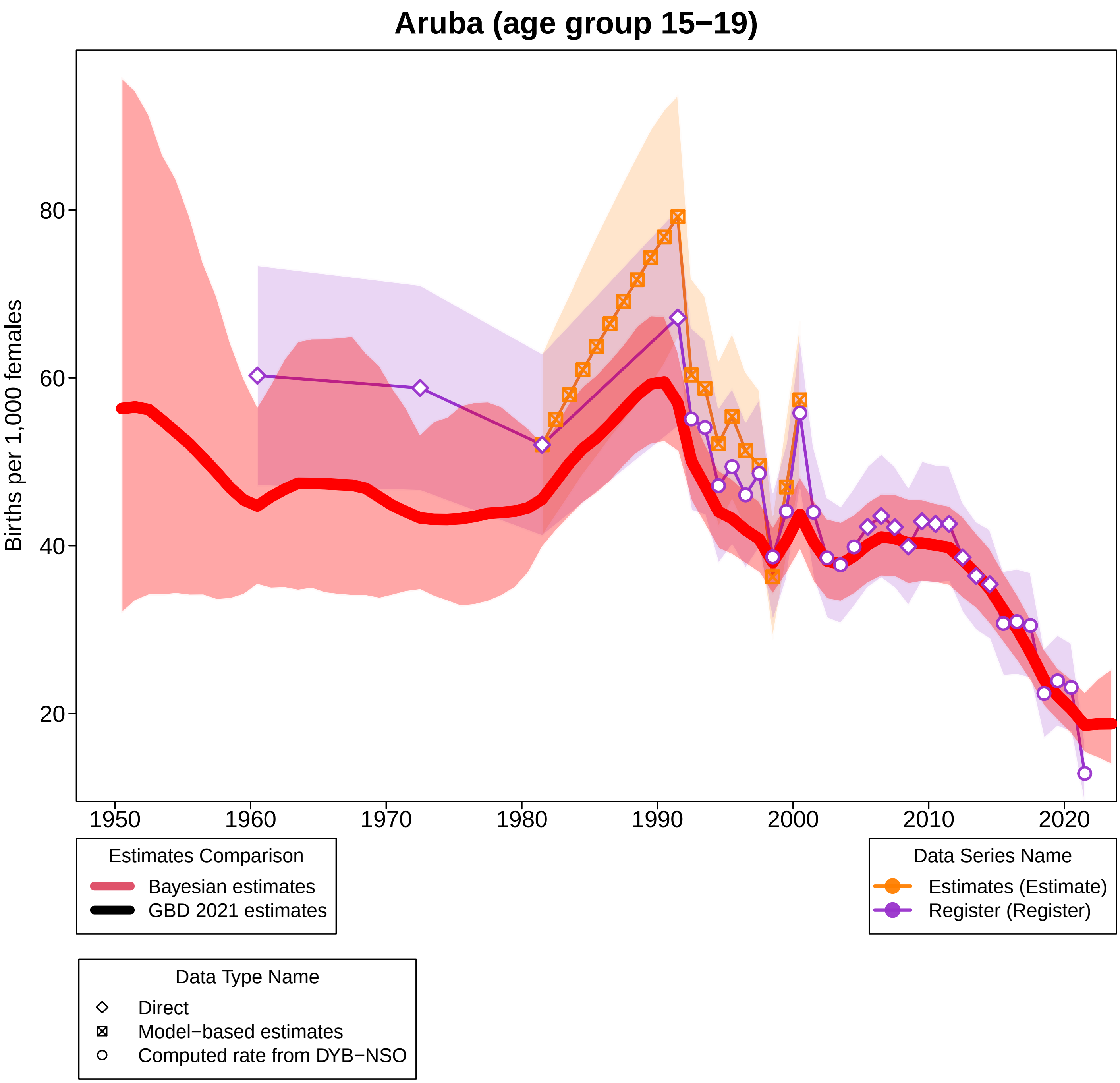
Aruba (age group 15−19)
Births per 1,000 females
80
60
40
20
1950
1960
1970
1980
1990
2000
2010
2020
Estimates Comparison
Bayesian estimates
GBD 2021 estimates
Data Series Name
Estimates (Estimate)
Register (Register)
Data Type Name
Direct
Model−based estimates
Computed rate from DYB−NSO

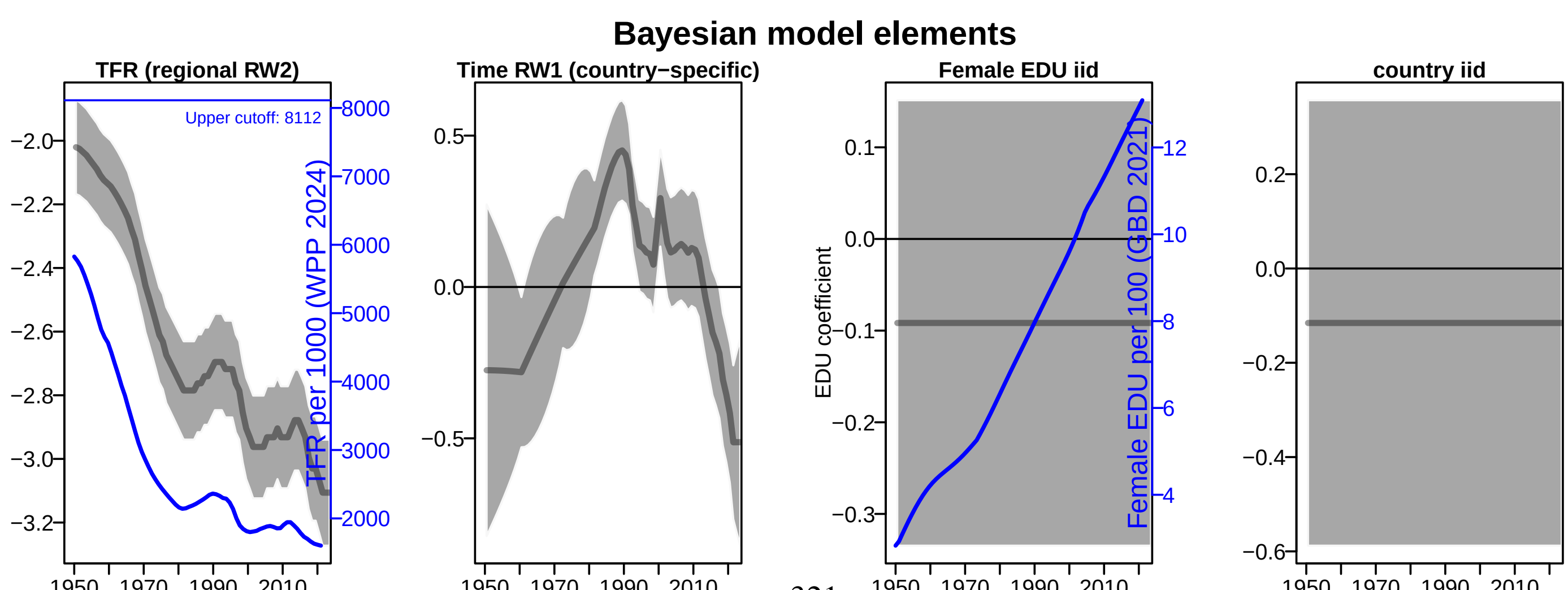
Bayesian model elements
TFR (regional RW2)
Upper cutoff: 8112
TFR per 1000 (WPP 2024)
Time RW1 (country−specific)
Female EDU iid
EDU coefficient
Female EDU per 100 (GBD 2021)
country iid

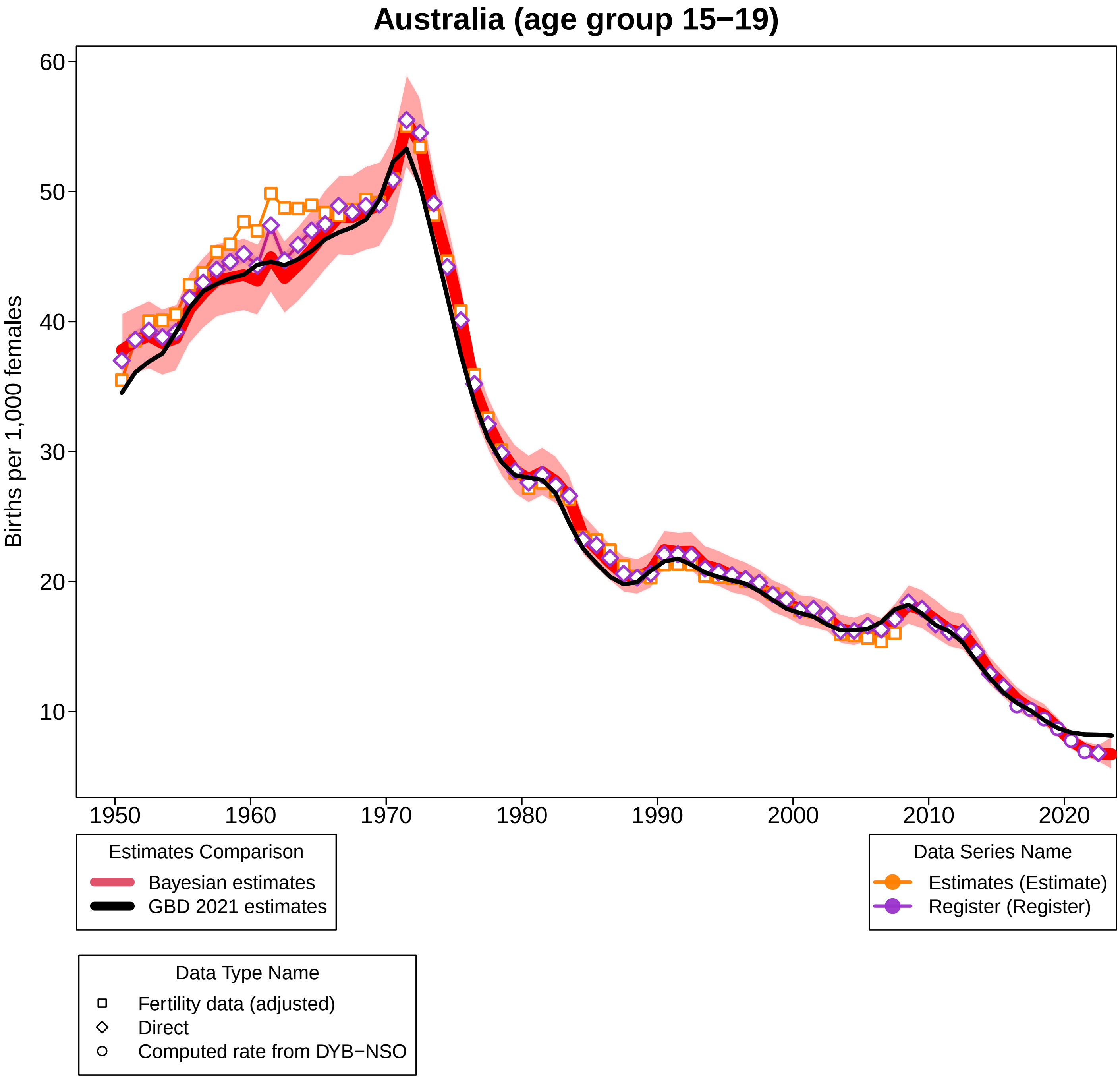
Australia (age group 15−19)
Births per 1,000 females
60
50
40
30
20
10
1950
1960
1970
1980
1990
2000
2010
2020
Estimates Comparison
Bayesian estimates
GBD 2021 estimates
Data Series Name
Estimates (Estimate)
Register (Register)
Data Type Name
Fertility data (adjusted)
Direct
Computed rate from DYB−NSO

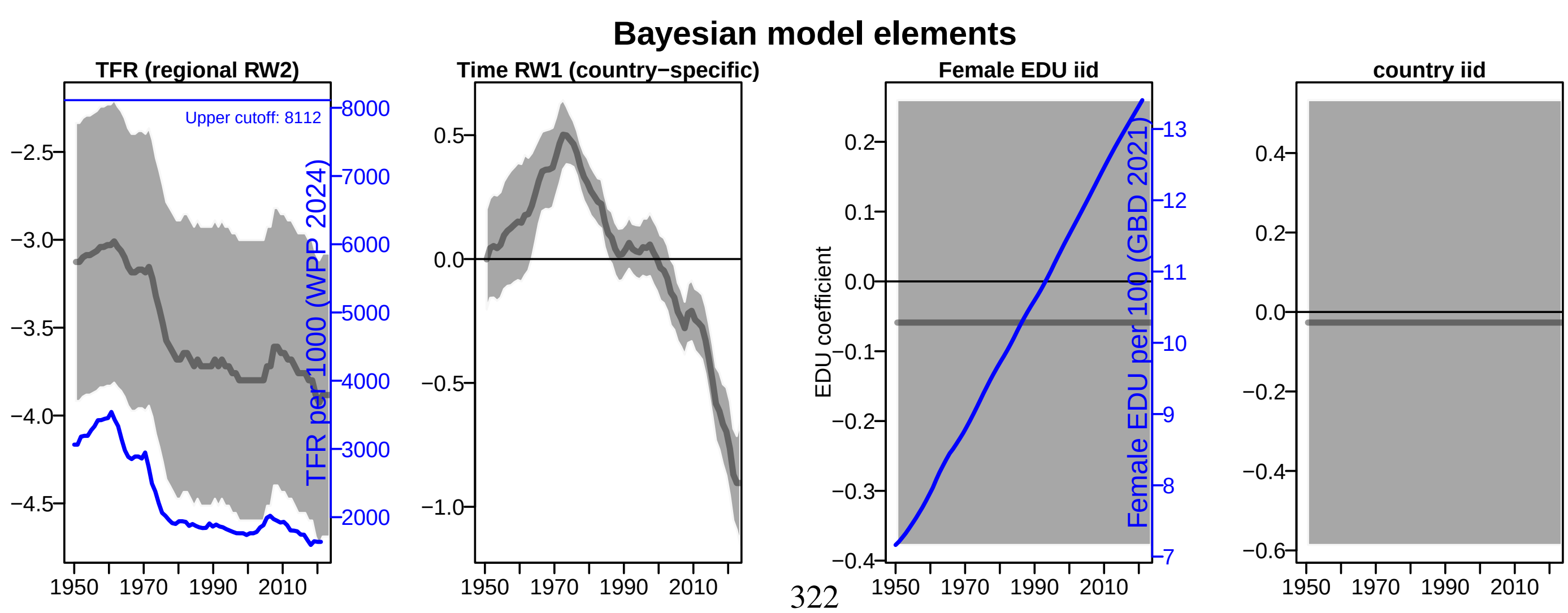
Bayesian model elements
TFR (regional RW2)
Upper cutoff: 8112
TFR per 1000 (WPP 2024)
Time RW1 (country−specific)
Female EDU iid
EDU coefficient
Female EDU per 100 (GBD 2021)
country iid
1950
1970
1990
2010

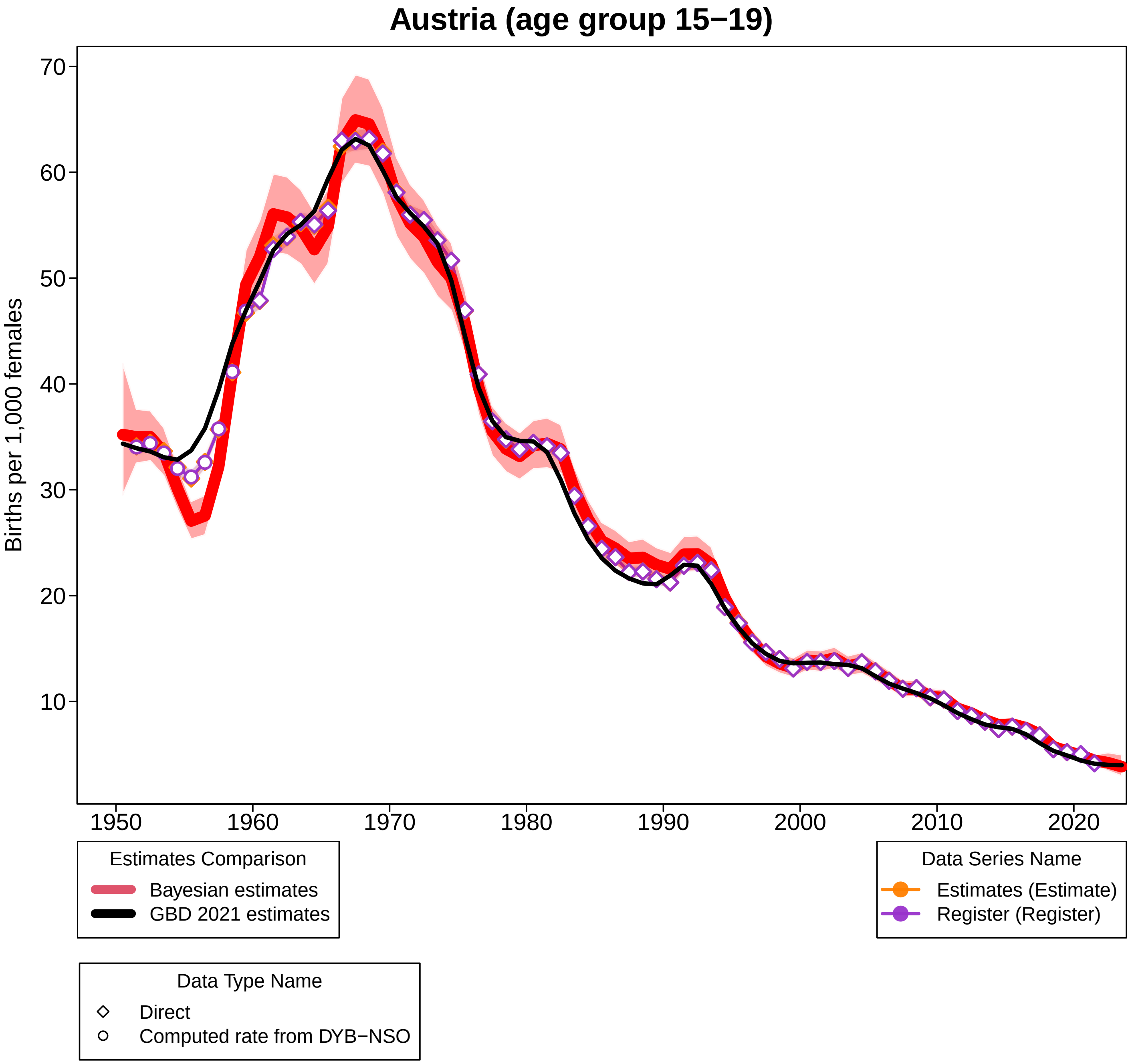
Austria (age group 15−19)
Births per 1,000 females
70
60
50
40
30
20
10
1950
1960
1970
1980
1990
2000
2010
2020
Estimates Comparison
Bayesian estimates
GBD 2021 estimates
Data Series Name
Estimates (Estimate)
Register (Register)
Data Type Name
Direct
Computed rate from DYB−NSO

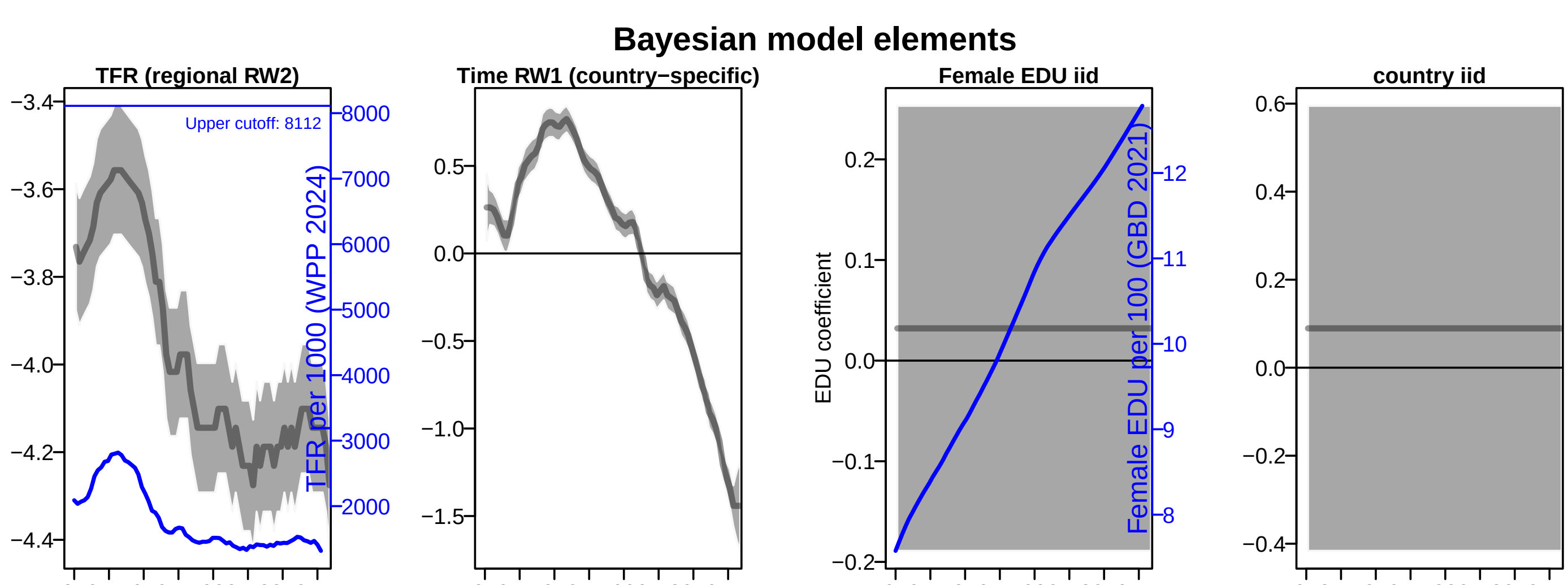
Bayesian model elements
TFR (regional RW2)
Upper cutoff: 8112
TFR per 1000 (WPP 2024)
Time RW1 (country−specific)
Female EDU iid
EDU coefficient
Female EDU per 100 (GBD 2021)
country iid

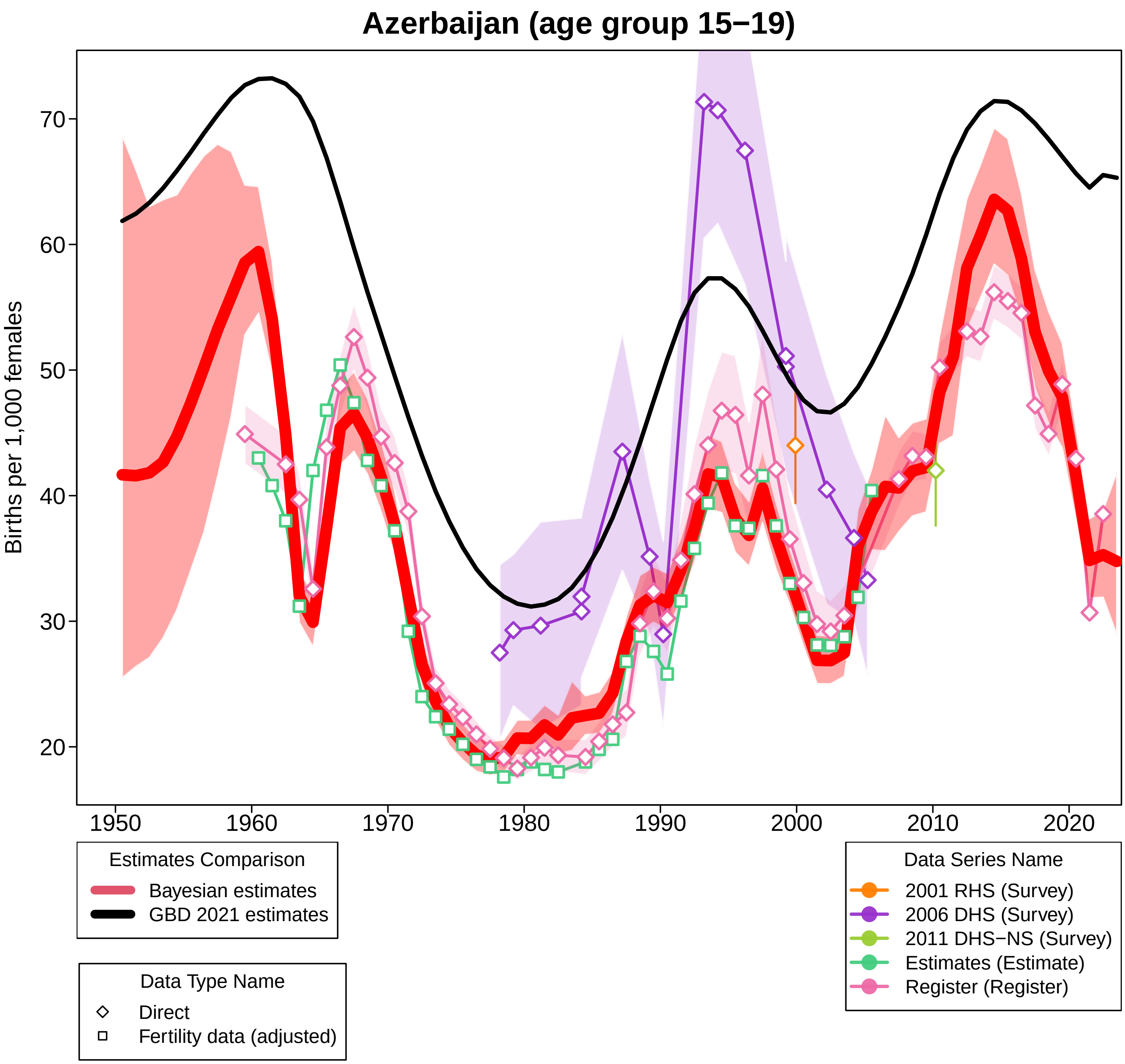
Azerbaijan (age group 15−19)
Births per 1,000 females
Estimates Comparison
Bayesian estimates
GBD 2021 estimates
Data Type Name
Direct
Fertility data (adjusted)
Data Series Name
2001 RHS (Survey)
2006 DHS (Survey)
2011 DHS−NS (Survey)
Estimates (Estimate)
Register (Register)

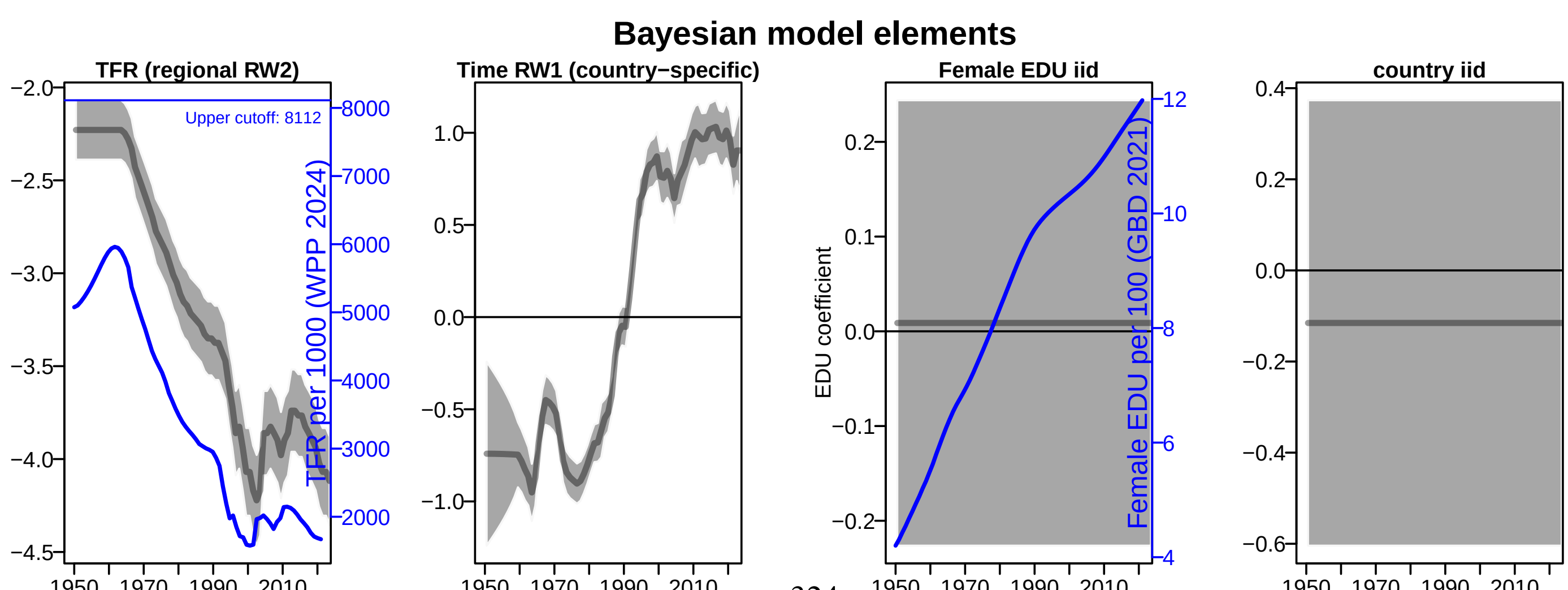
Bayesian model elements
TFR (regional RW2)
Upper cutoff: 8112
TFR per 1000 (WPP 2024)
Time RW1 (country−specific)
Female EDU iid
EDU coefficient
Female EDU per 100 (GBD 2021)
country iid

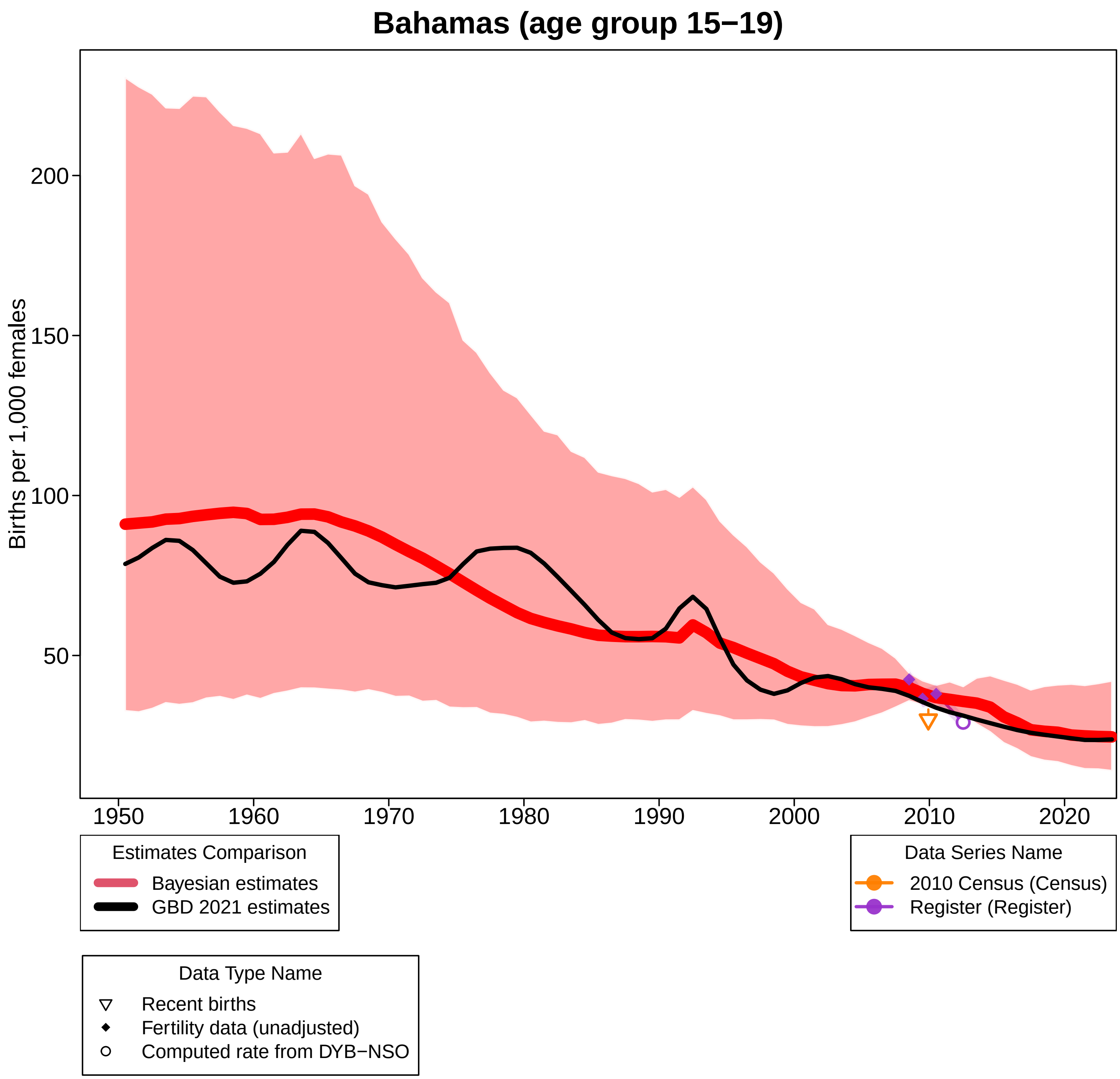
Bahamas (age group 15−19)
Births per 1,000 females
200
150
100
50
1950
1960
1970
1980
1990
2000
2010
2020
Estimates Comparison
Bayesian estimates
GBD 2021 estimates
Data Series Name
2010 Census (Census)
Register (Register)
Data Type Name
Recent births
Fertility data (unadjusted)
Computed rate from DYB−NSO

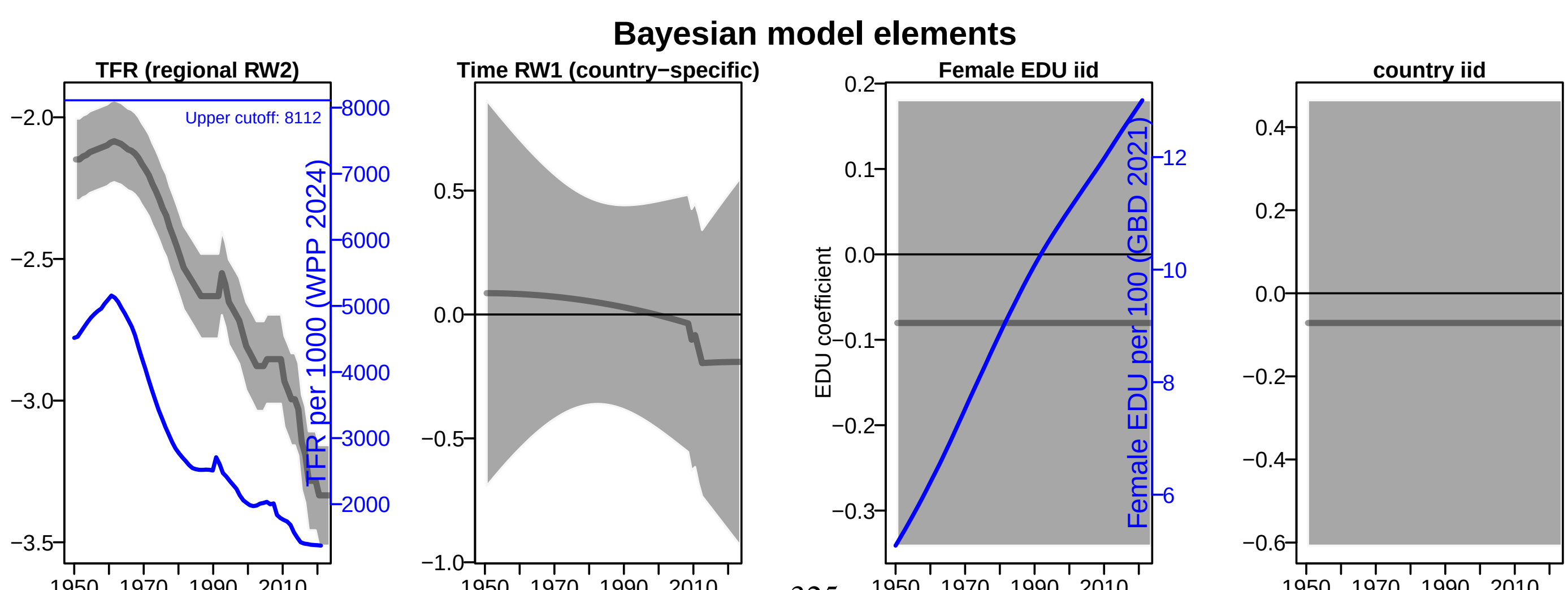
Bayesian model elements
TFR (regional RW2)
Upper cutoff: 8112
TFR per 1000 (WPP 2024)
Time RW1 (country−specific)
Female EDU iid
EDU coefficient
Female EDU per 100 (GBD 2021)
country iid

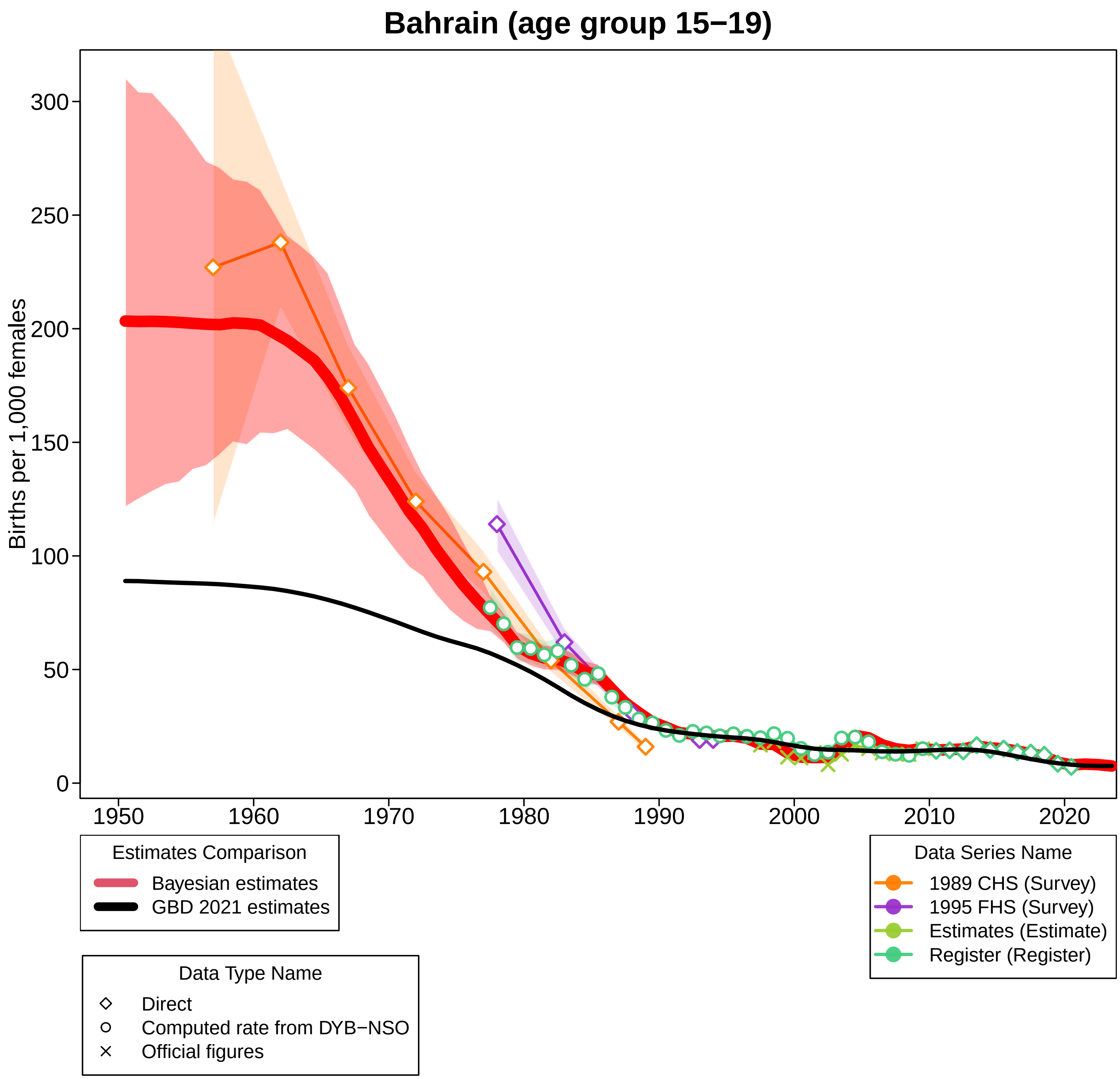
Bahrain (age group 15−19)
Births per 1,000 females
300
250
200
150
100
50
0
1950
1960
1970
1980
1990
2000
2010
2020
Estimates Comparison
Bayesian estimates
GBD 2021 estimates
Data Series Name
1989 CHS (Survey)
1995 FHS (Survey)
Estimates (Estimate)
Register (Register)
Data Type Name
Direct
Computed rate from DYB−NSO
Official figures

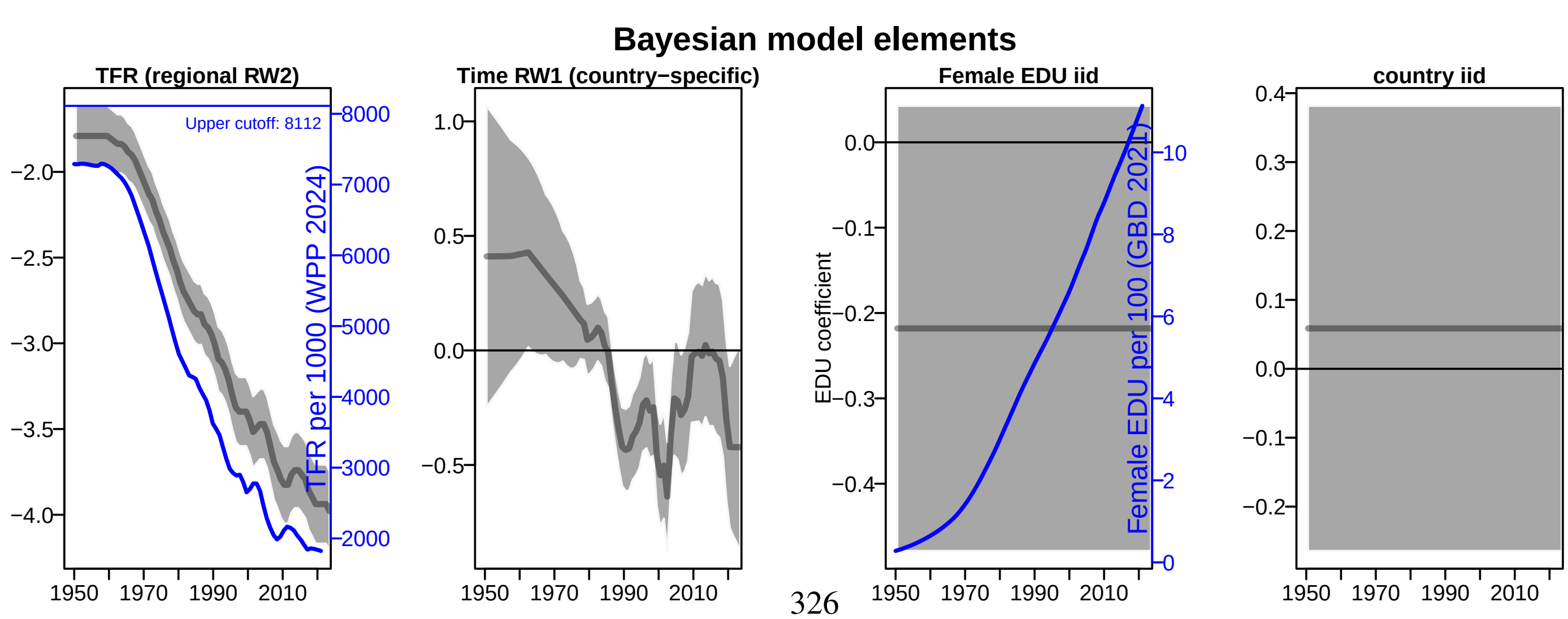
Bayesian model elements
TFR (regional RW2)
Upper cutoff: 8112
TFR per 1000 (WPP 2024)
Time RW1 (country−specific)
Female EDU iid
EDU coefficient
Female EDU per 100 (GBD 2021)
country iid

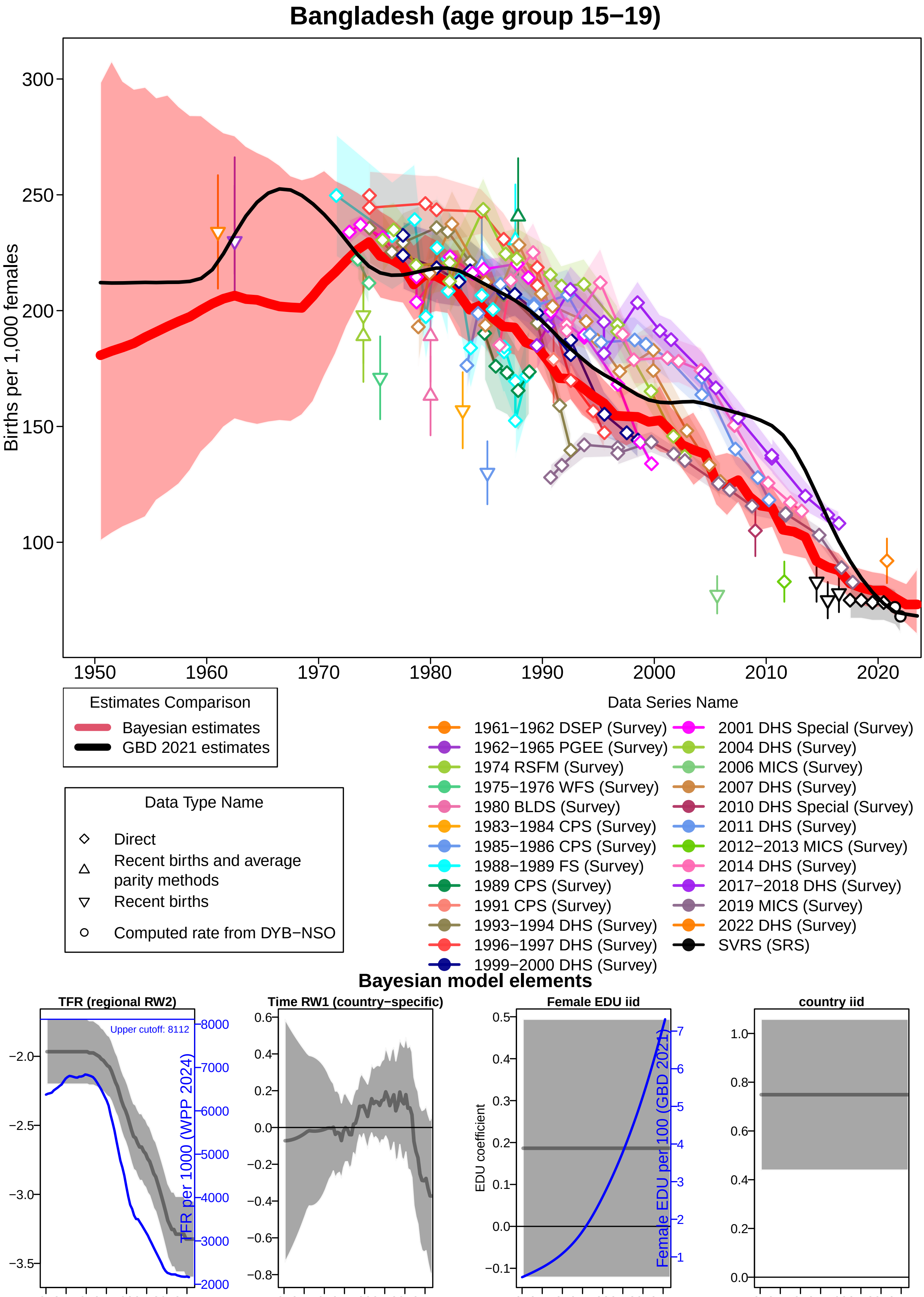
Bangladesh (age group 15−19)
Births per 1,000 females
Estimates Comparison
Bayesian estimates
GBD 2021 estimates
Data Type Name
Direct
Recent births and average parity methods
Recent births
Computed rate from DYB−NSO
Data Series Name
1961−1962 DSEP (Survey)
1962−1965 PGEE (Survey)
1974 RSFM (Survey)
1975−1976 WFS (Survey)
1980 BLDS (Survey)
1983−1984 CPS (Survey)
1985−1986 CPS (Survey)
1988−1989 FS (Survey)
1989 CPS (Survey)
1991 CPS (Survey)
1993−1994 DHS (Survey)
1996−1997 DHS (Survey)
1999−2000 DHS (Survey)
2001 DHS Special (Survey)
2004 DHS (Survey)
2006 MICS (Survey)
2007 DHS (Survey)
2010 DHS Special (Survey)
2011 DHS (Survey)
2012−2013 MICS (Survey)
2014 DHS (Survey)
2017−2018 DHS (Survey)
2019 MICS (Survey)
2022 DHS (Survey)
SVRS (SRS)
Bayesian model elements
TFR (regional RW2)
Upper cutoff: 8112
TFR per 1000 (WPP 2024)
Time RW1 (country−specific)
Female EDU iid
EDU coefficient
Female EDU per 100 (GBD 2021)
country iid

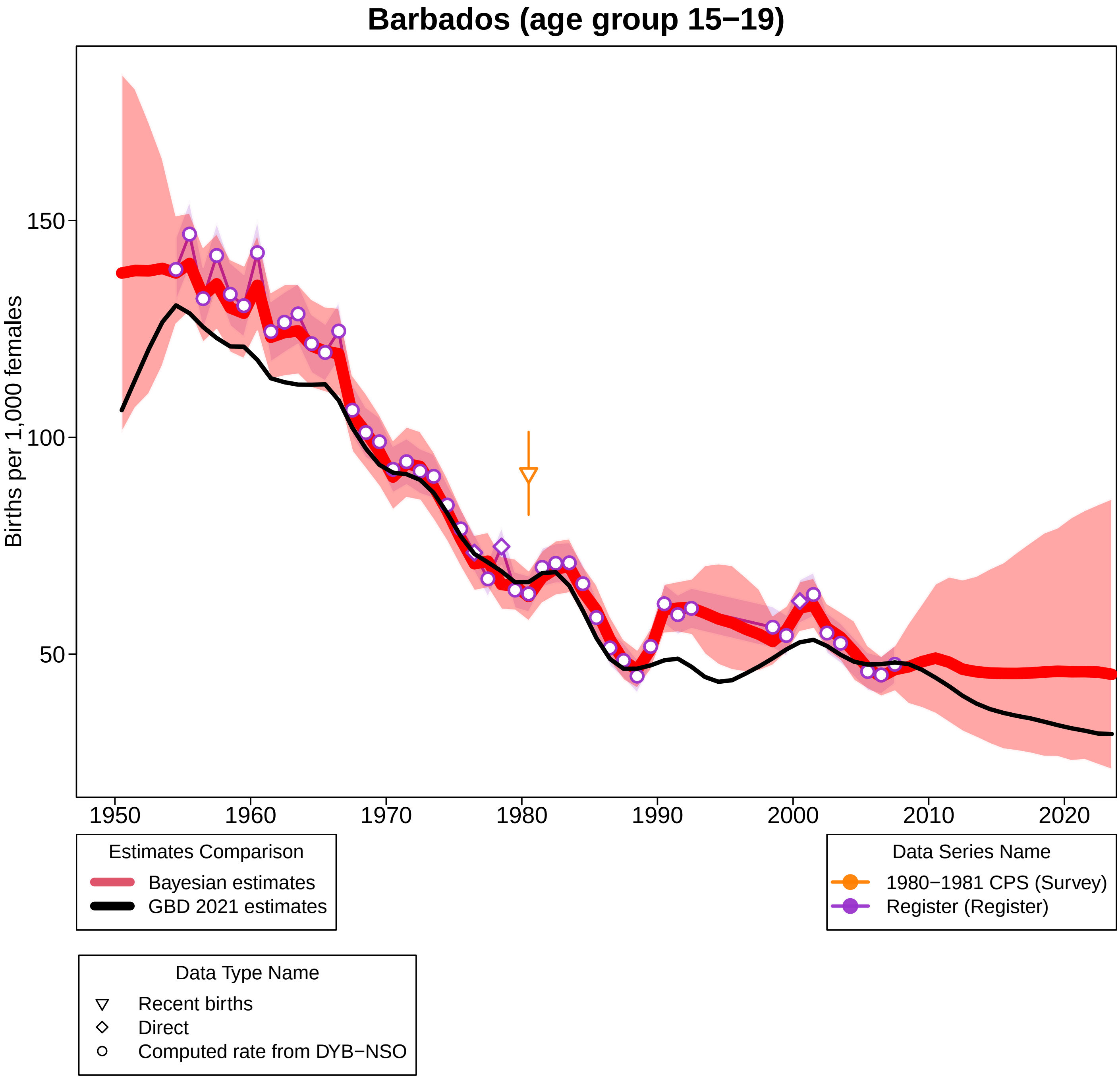

Barbados (age group 15−19)
Births per 1,000 females
150
100
50
1950
1960
1970
1980
1990
2000
2010
2020
Estimates Comparison
Bayesian estimates
GBD 2021 estimates
Data Series Name
1980−1981 CPS (Survey)
Register (Register)
Data Type Name
Recent births
Direct
Computed rate from DYB−NSO

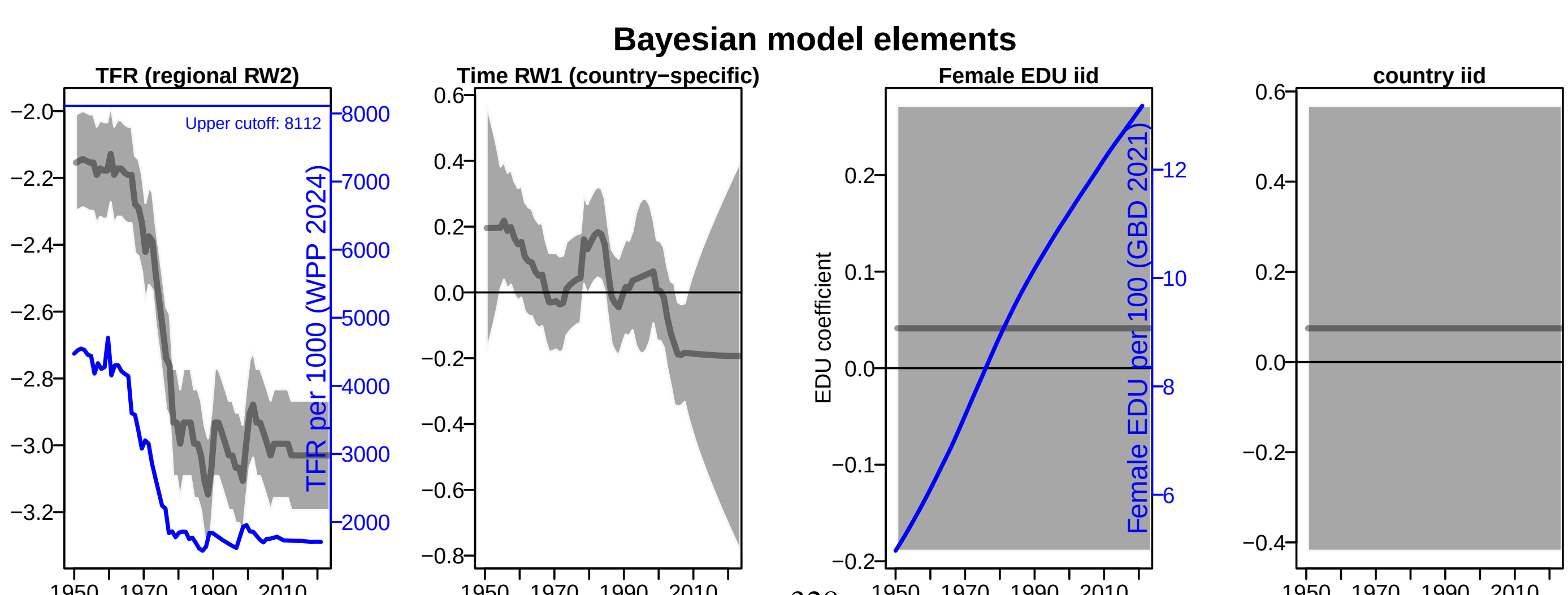

Bayesian model elements
TFR (regional RW2)
Upper cutoff: 8112
TFR per 1000 (WPP 2024)
Time RW1 (country−specific)
Female EDU iid
EDU coefficient
Female EDU per 100 (GBD 2021)
country iid

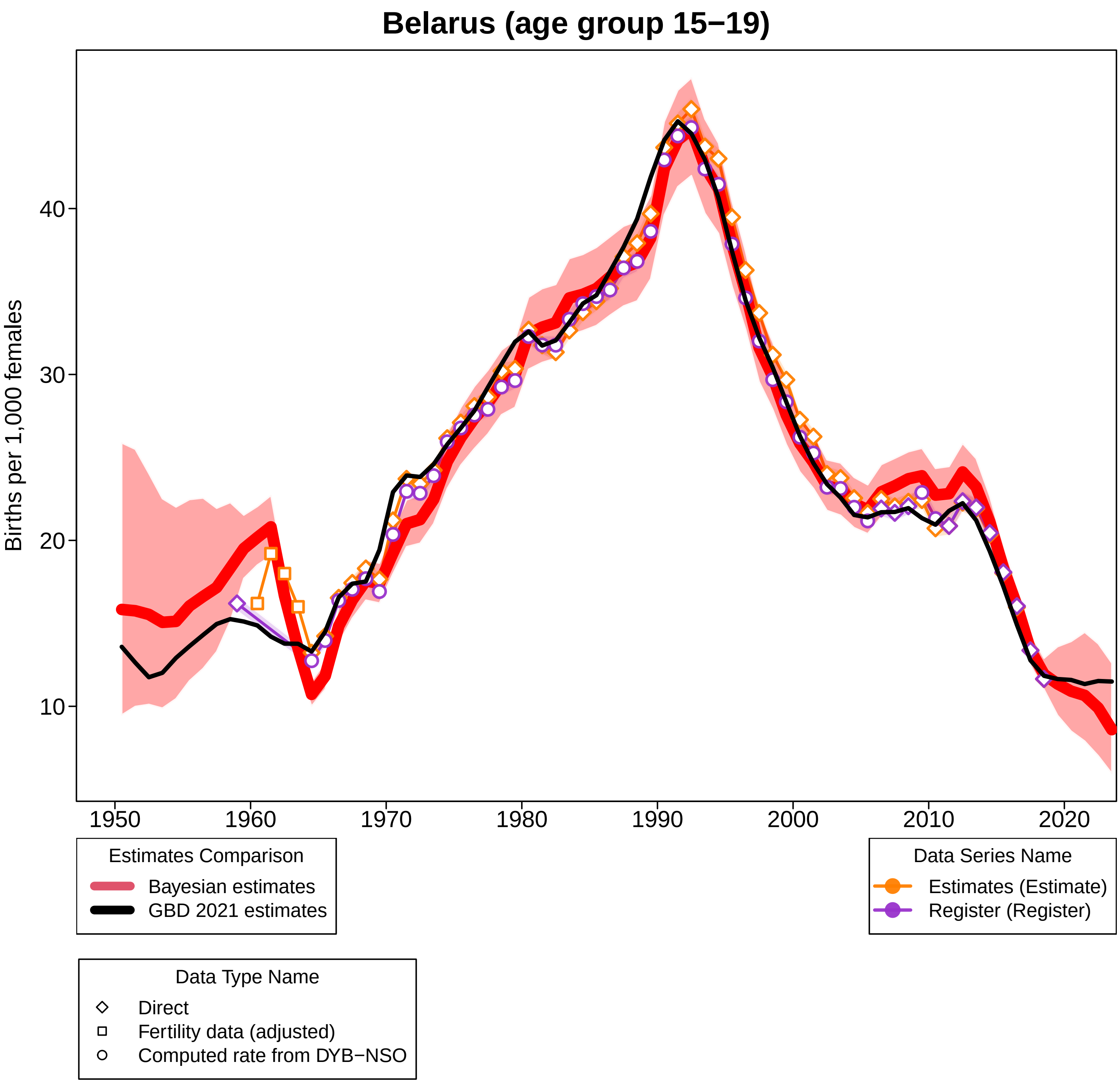
Belarus (age group 15−19)
Births per 1,000 females
40
30
20
10
1950
1960
1970
1980
1990
2000
2010
2020
Estimates Comparison
Bayesian estimates
GBD 2021 estimates
Data Series Name
Estimates (Estimate)
Register (Register)
Data Type Name
Direct
Fertility data (adjusted)
Computed rate from DYB−NSO

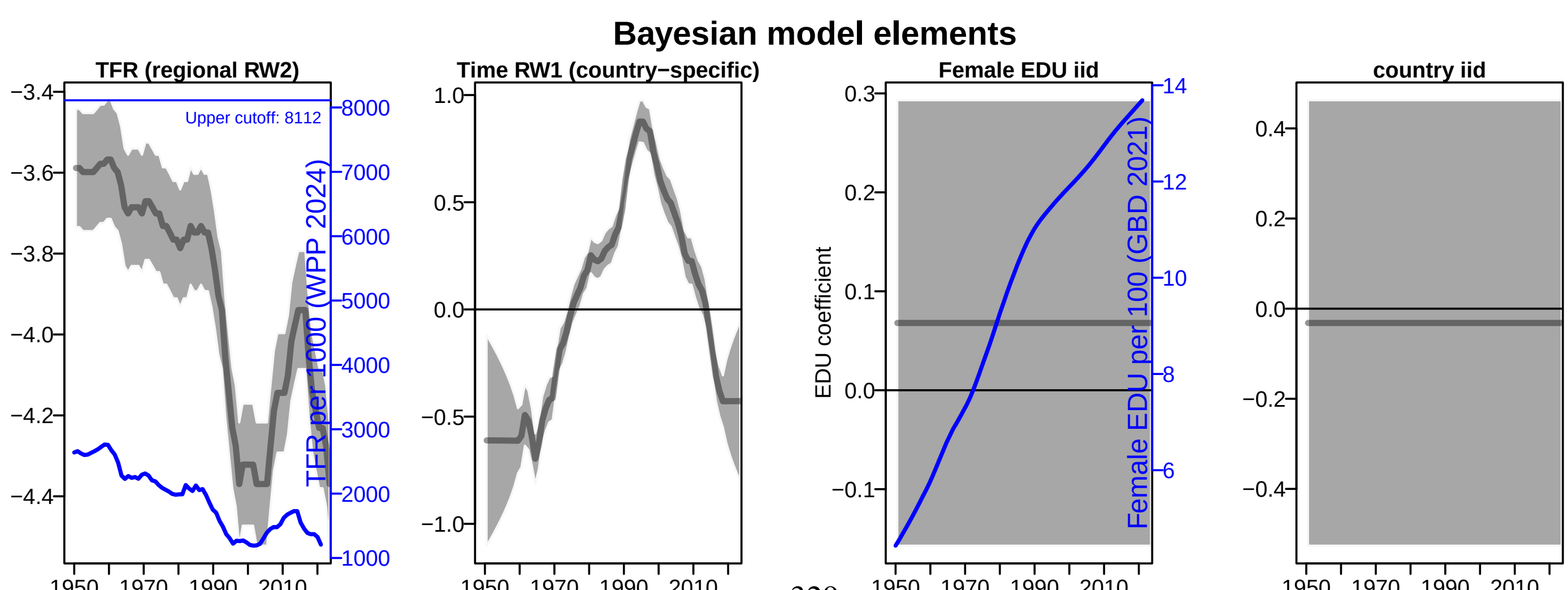
Bayesian model elements
TFR (regional RW2)
Upper cutoff: 8112
TFR per 1000 (WPP 2024)
Time RW1 (country−specific)
Female EDU iid
EDU coefficient
Female EDU per 100 (GBD 2021)
country iid
1950
1970
1990
2010

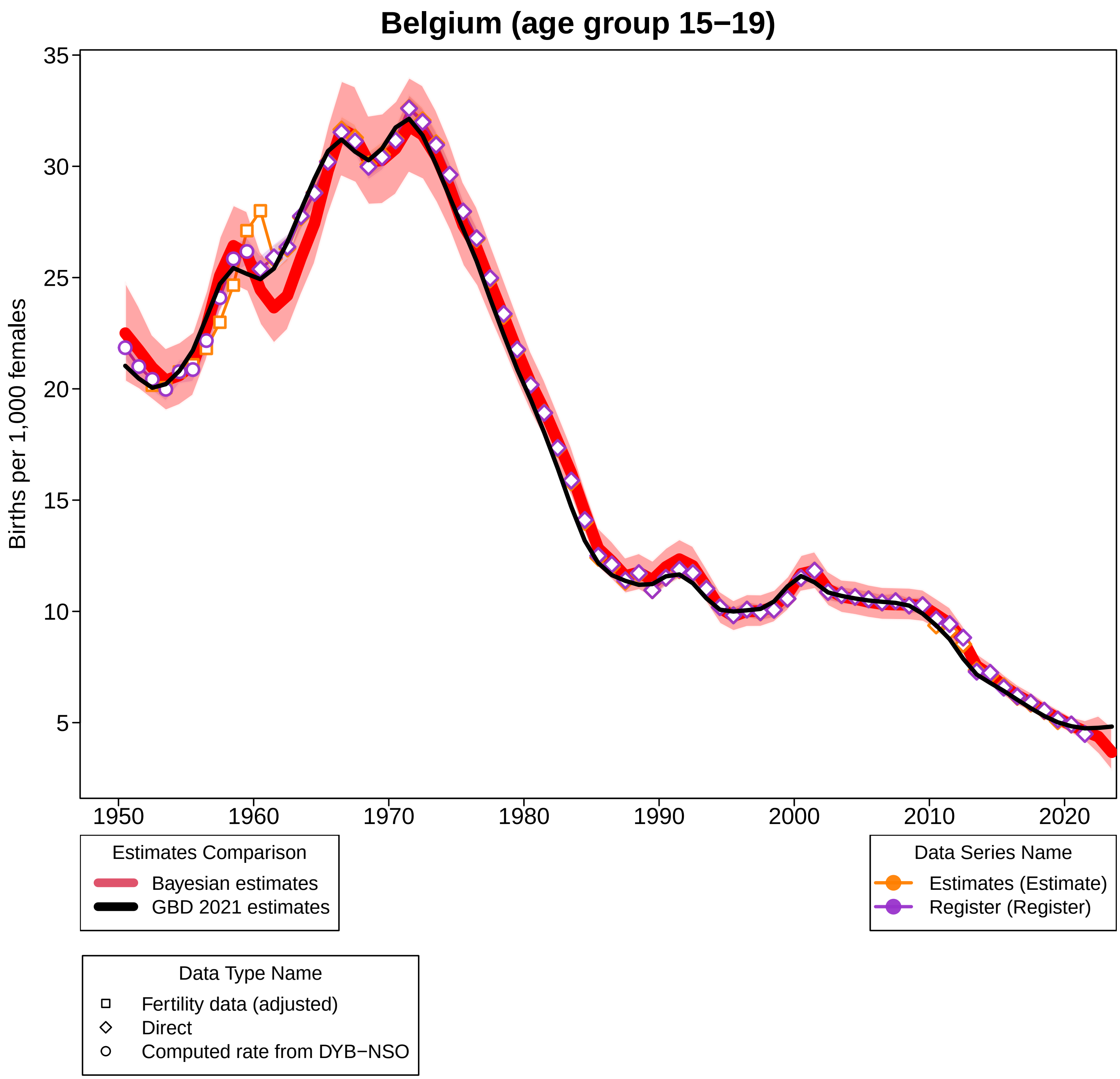
Belgium (age group 15−19)
Births per 1,000 females
35
30
25
20
15
10
5
1950
1960
1970
1980
1990
2000
2010
2020
Estimates Comparison
Bayesian estimates
GBD 2021 estimates
Data Series Name
Estimates (Estimate)
Register (Register)
Data Type Name
Fertility data (adjusted)
Direct
Computed rate from DYB−NSO

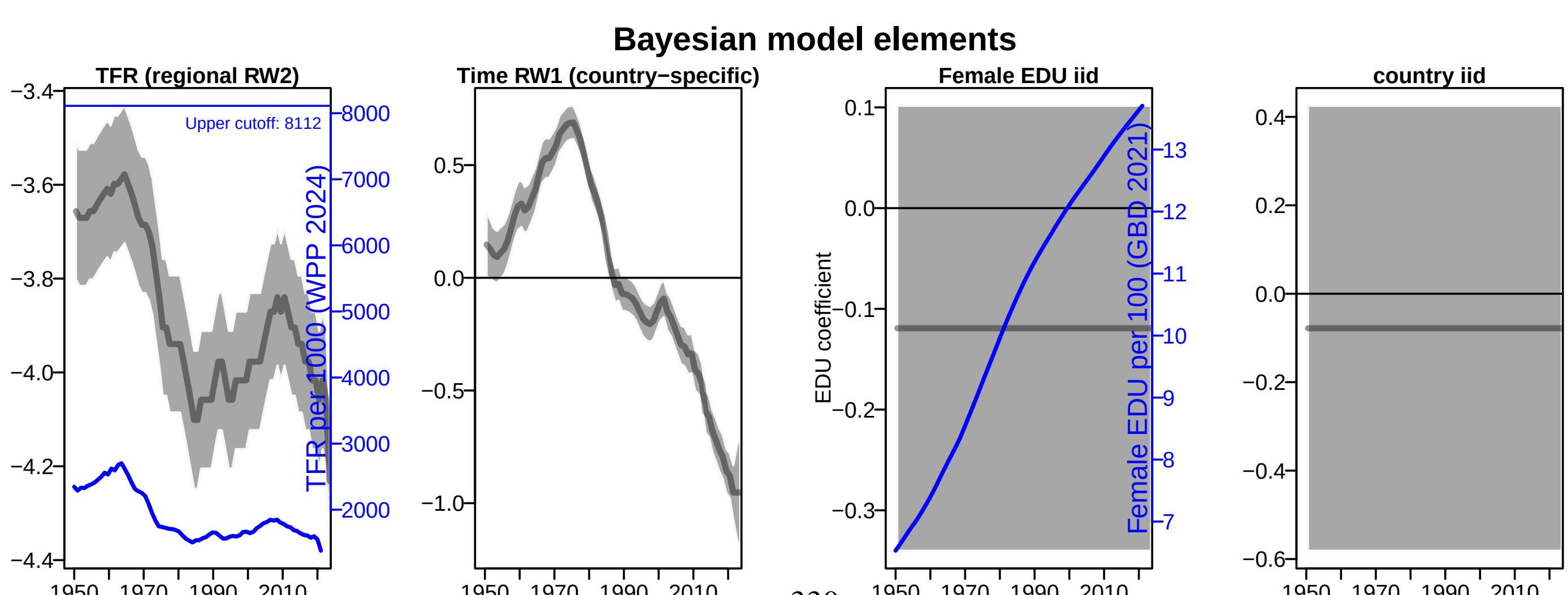
Bayesian model elements
TFR (regional RW2)
Upper cutoff: 8112
TFR per 1000 (WPP 2024)
Time RW1 (country−specific)
Female EDU iid
EDU coefficient
Female EDU per 100 (GBD 2021)
country iid

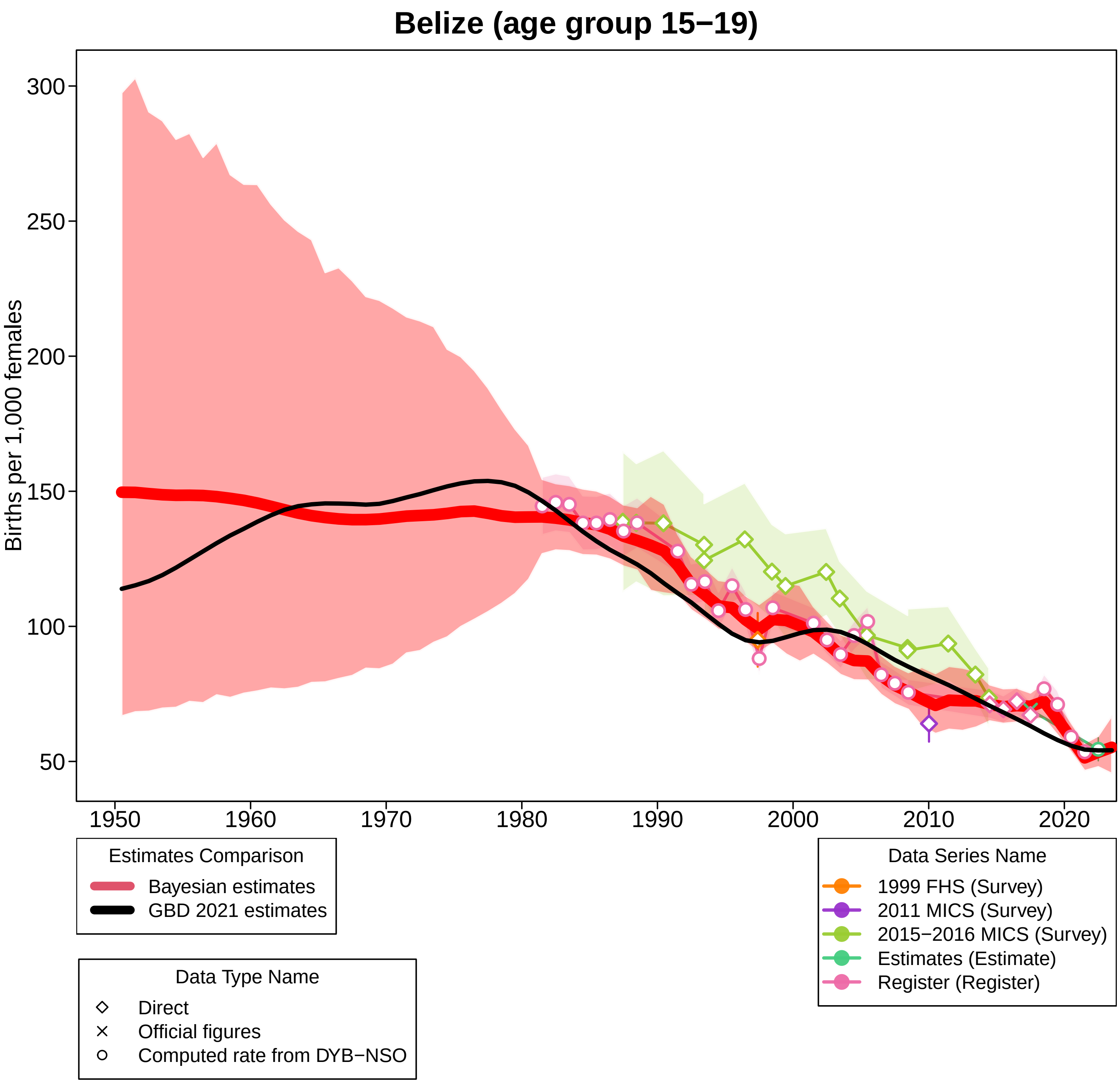
Belize (age group 15−19)
Births per 1,000 females
300
250
200
150
100
50
1950
1960
1970
1980
1990
2000
2010
2020
Estimates Comparison
Bayesian estimates
GBD 2021 estimates
Data Type Name
Direct
Official figures
Computed rate from DYB−NSO
Data Series Name
1999 FHS (Survey)
2011 MICS (Survey)
2015−2016 MICS (Survey)
Estimates (Estimate)
Register (Register)

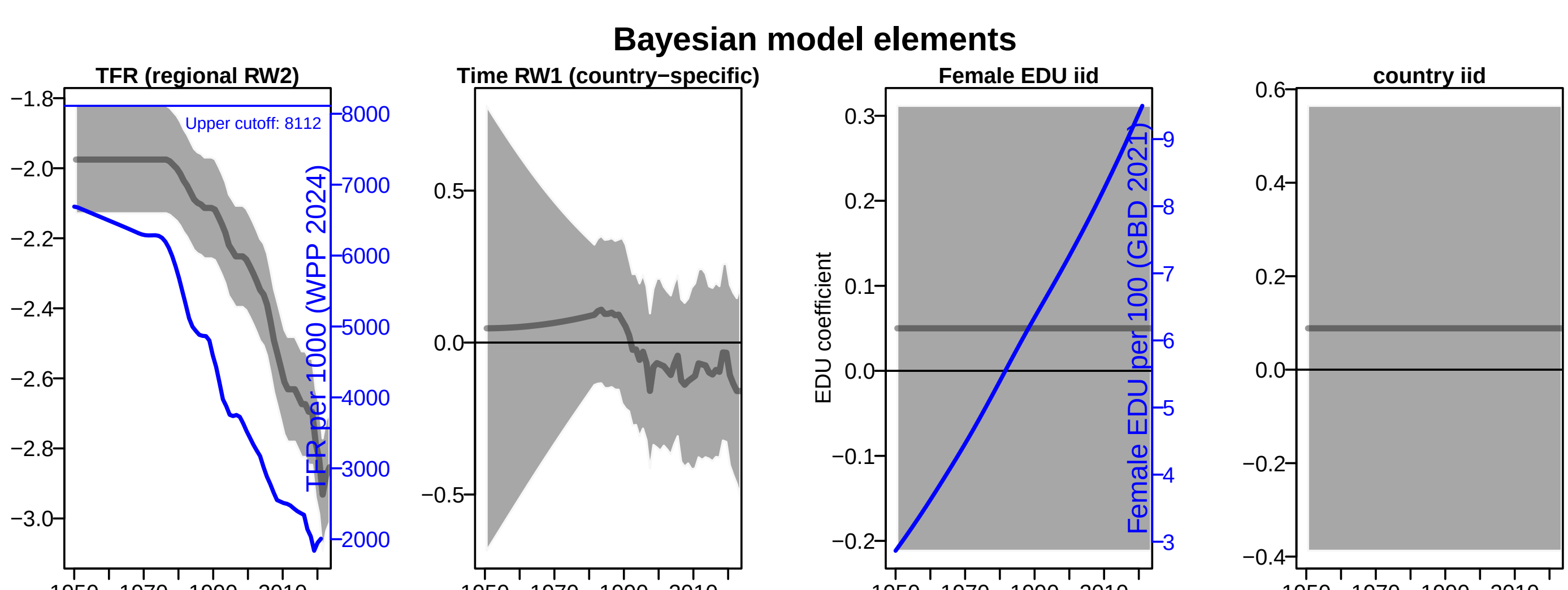
Bayesian model elements
TFR (regional RW2)
Upper cutoff: 8112
TFR per 1000 (WPP 2024)
Time RW1 (country−specific)
Female EDU iid
EDU coefficient
Female EDU per 100 (GBD 2021)
country iid
1950
1970
1990
2010

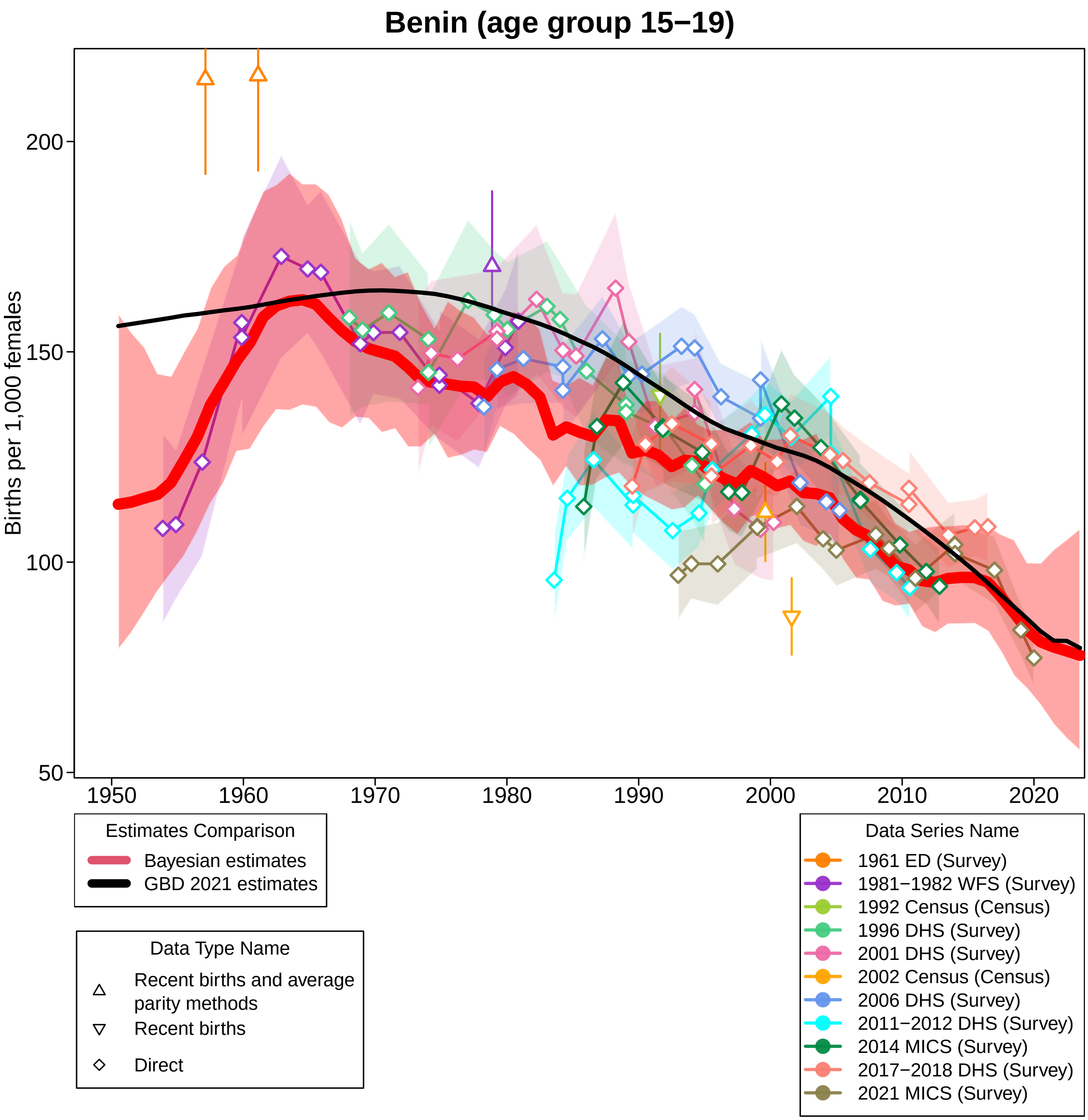
Benin (age group 15−19)
Births per 1,000 females
200
150
100
50
1950
1960
1970
1980
1990
2000
2010
2020
Estimates Comparison
Bayesian estimates
GBD 2021 estimates
Data Type Name
Recent births and average parity methods
Recent births
Direct
Data Series Name
1961 ED (Survey)
1981−1982 WFS (Survey)
1992 Census (Census)
1996 DHS (Survey)
2001 DHS (Survey)
2002 Census (Census)
2006 DHS (Survey)
2011−2012 DHS (Survey)
2014 MICS (Survey)
2017−2018 DHS (Survey)
2021 MICS (Survey)

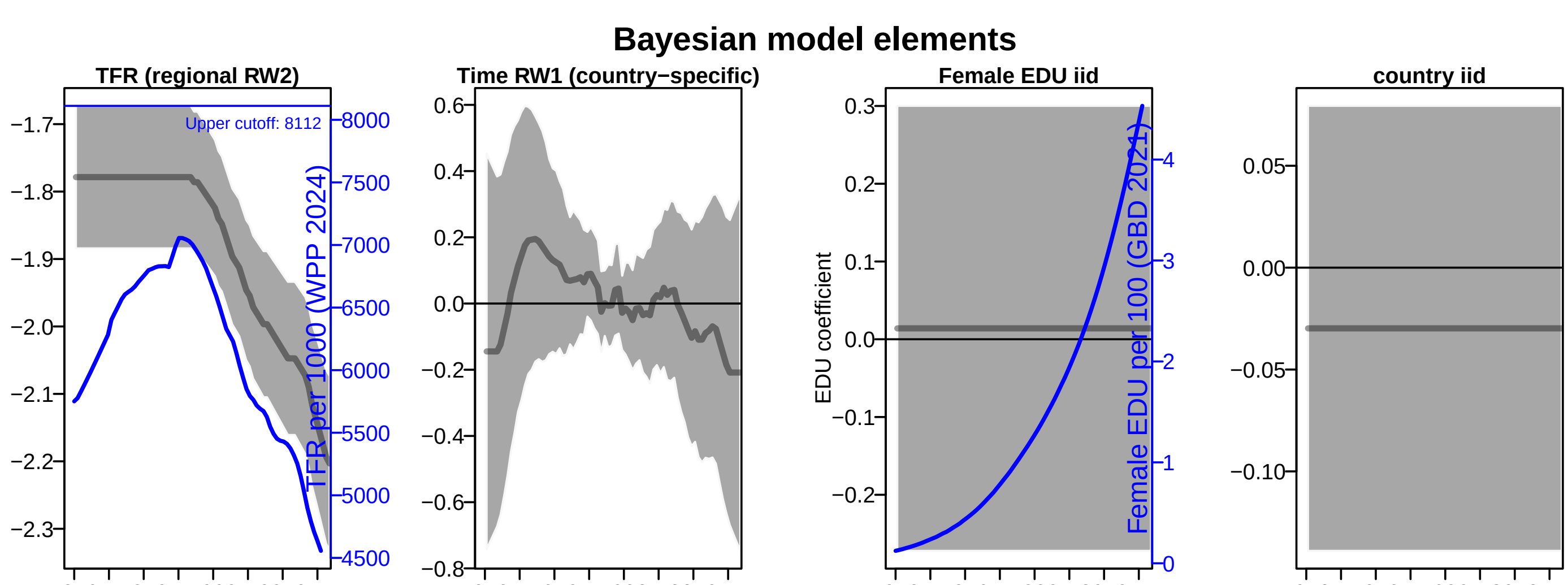
Bayesian model elements
TFR (regional RW2)
Upper cutoff: 8112
TFR per 1000 (WPP 2024)
Time RW1 (country−specific)
Female EDU iid
EDU coefficient
Female EDU per 100 (GBD 2021)
country iid

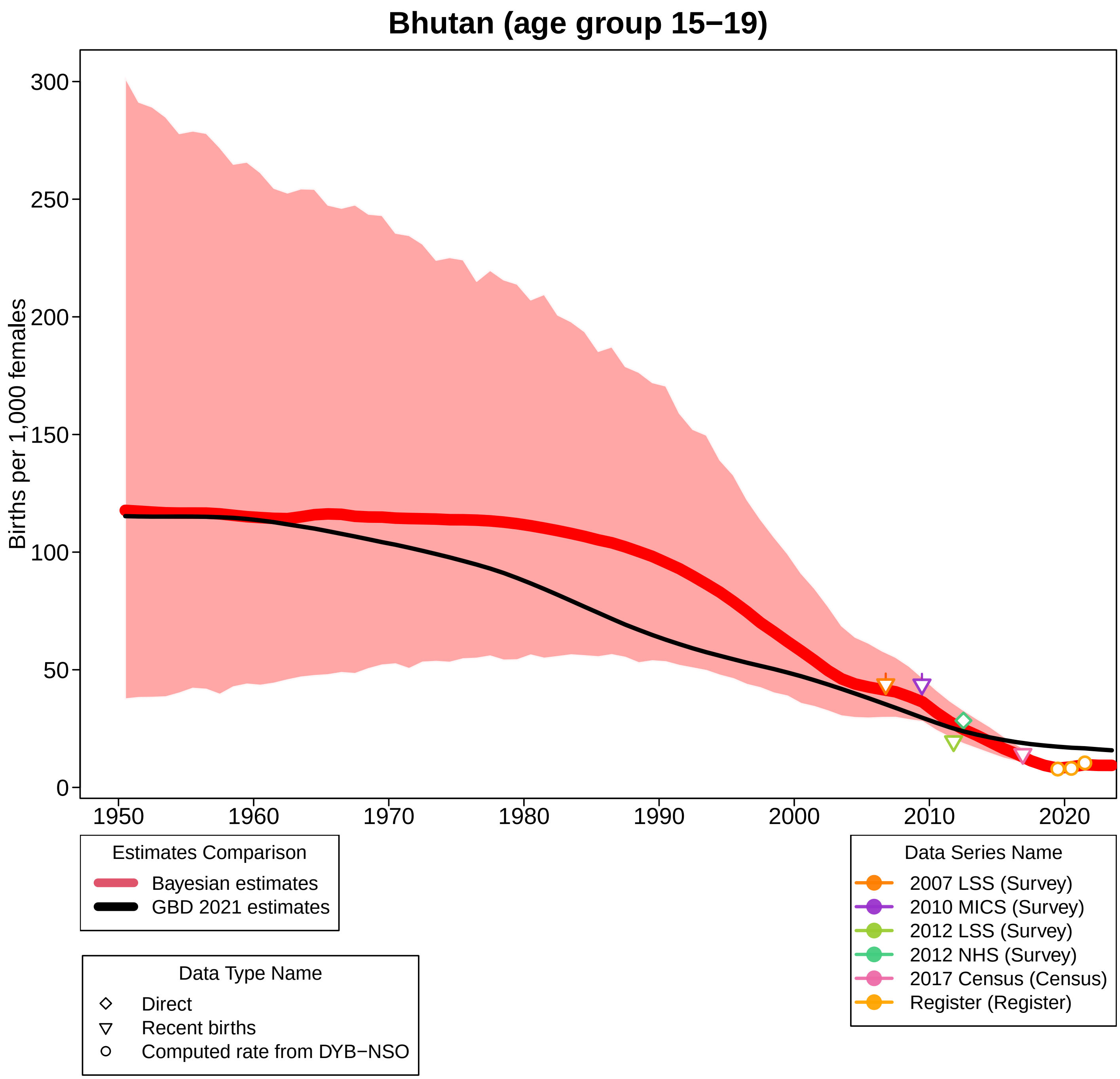

Bhutan (age group 15−19)
Births per 1,000 females
0
50
100
150
200
250
300
1950
1960
1970
1980
1990
2000
2010
2020
Estimates Comparison
Bayesian estimates
GBD 2021 estimates
Data Type Name
Direct
Recent births
Computed rate from DYB−NSO
Data Series Name
2007 LSS (Survey)
2010 MICS (Survey)
2012 LSS (Survey)
2012 NHS (Survey)
2017 Census (Census)
Register (Register)

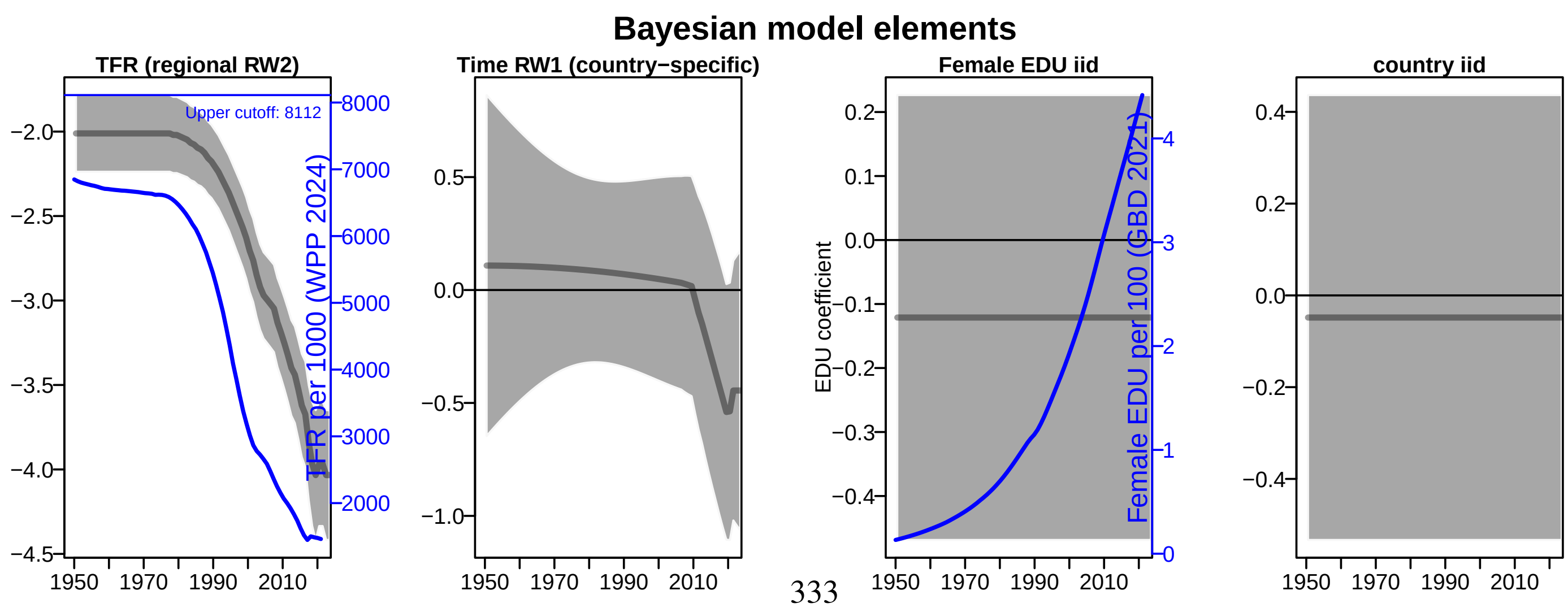

Bayesian model elements
TFR (regional RW2)
Upper cutoff: 8112
TFR per 1000 (WPP 2024)
Time RW1 (country−specific)
Female EDU iid
EDU coefficient
Female EDU per 100 (GBD 2021)
country iid

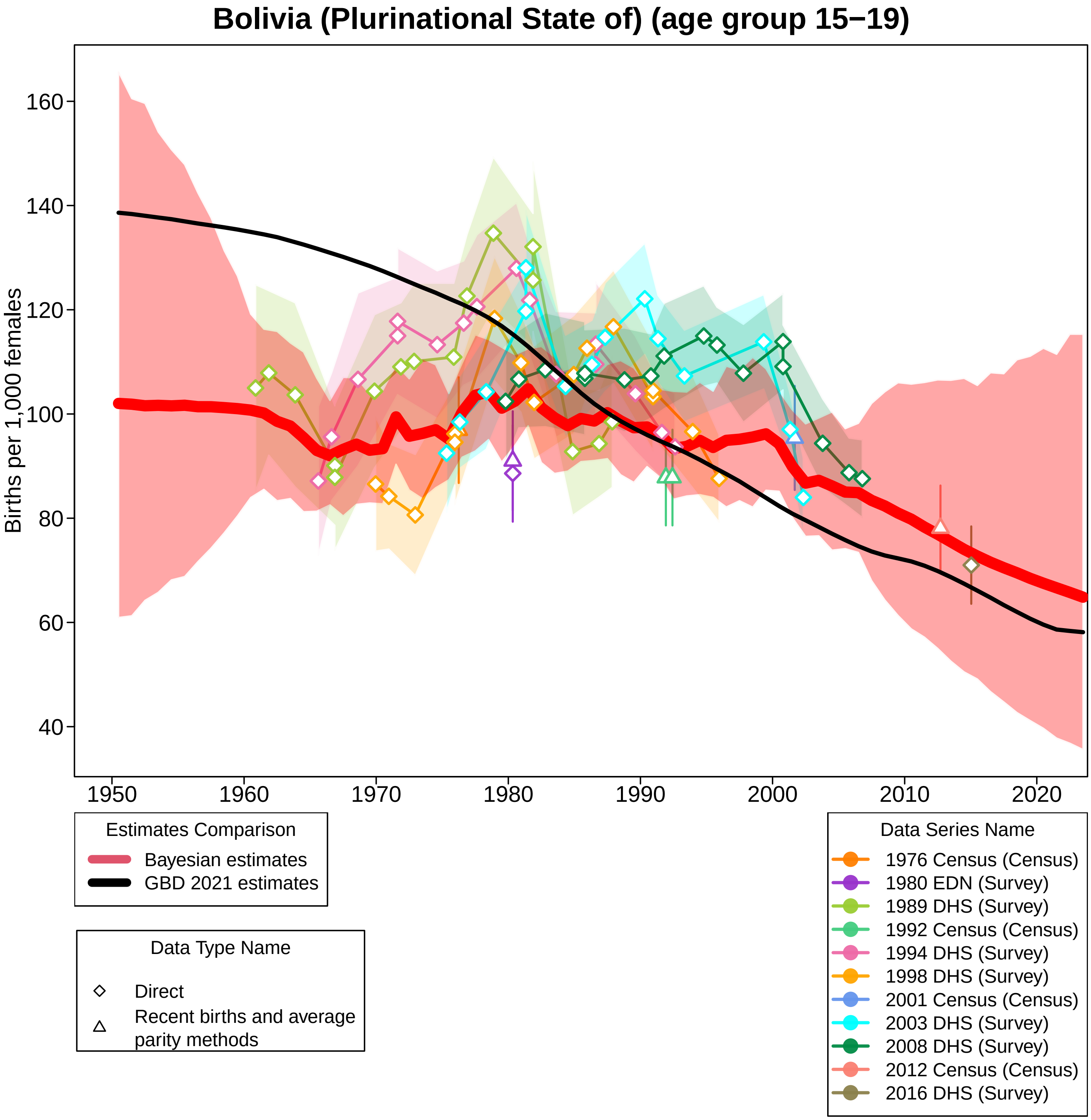
Bolivia (Plurinational State of) (age group 15−19)
Births per 1,000 females
160
140
120
100
80
60
40
1950
1960
1970
1980
1990
2000
2010
2020
Estimates Comparison
Bayesian estimates
GBD 2021 estimates
Data Type Name
Direct
Recent births and average parity methods
Data Series Name
1976 Census (Census)
1980 EDN (Survey)
1989 DHS (Survey)
1992 Census (Census)
1994 DHS (Survey)
1998 DHS (Survey)
2001 Census (Census)
2003 DHS (Survey)
2008 DHS (Survey)
2012 Census (Census)
2016 DHS (Survey)

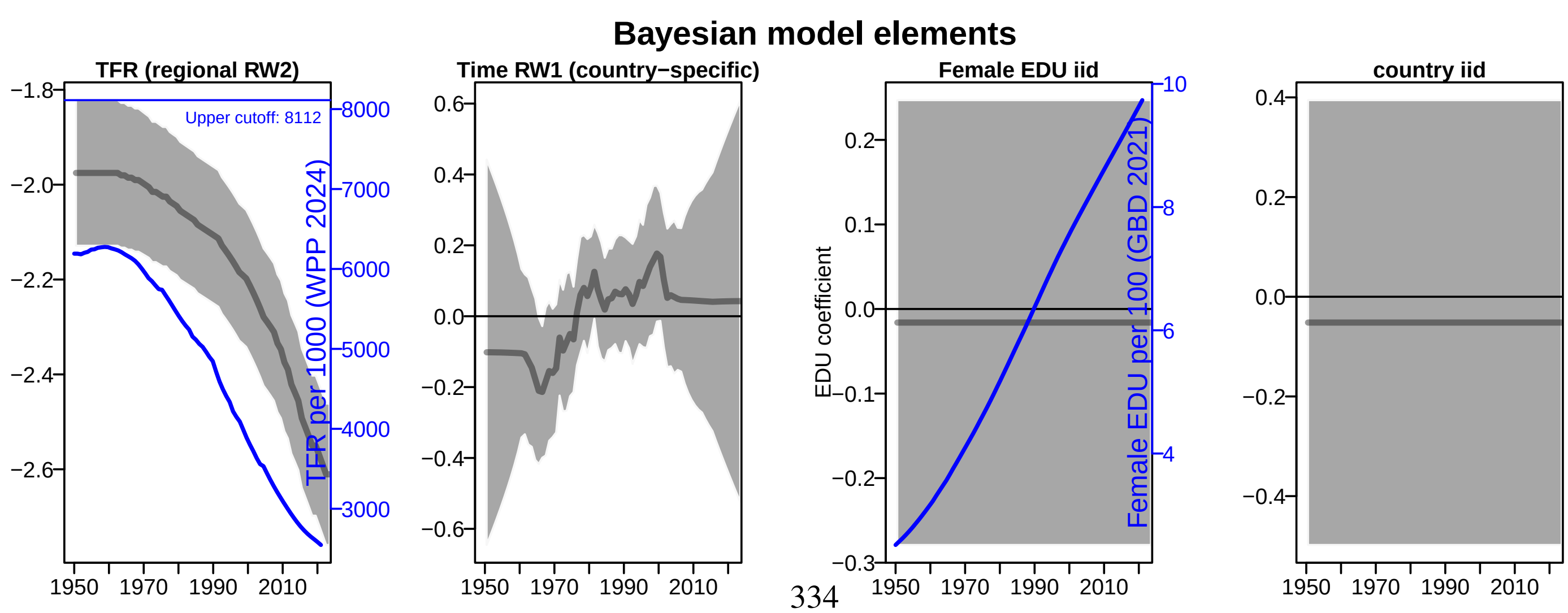
Bayesian model elements
TFR (regional RW2)
Upper cutoff: 8112
TFR per 1000 (WPP 2024)
Time RW1 (country−specific)
Female EDU iid
EDU coefficient
Female EDU per 100 (GBD 2021)
country iid

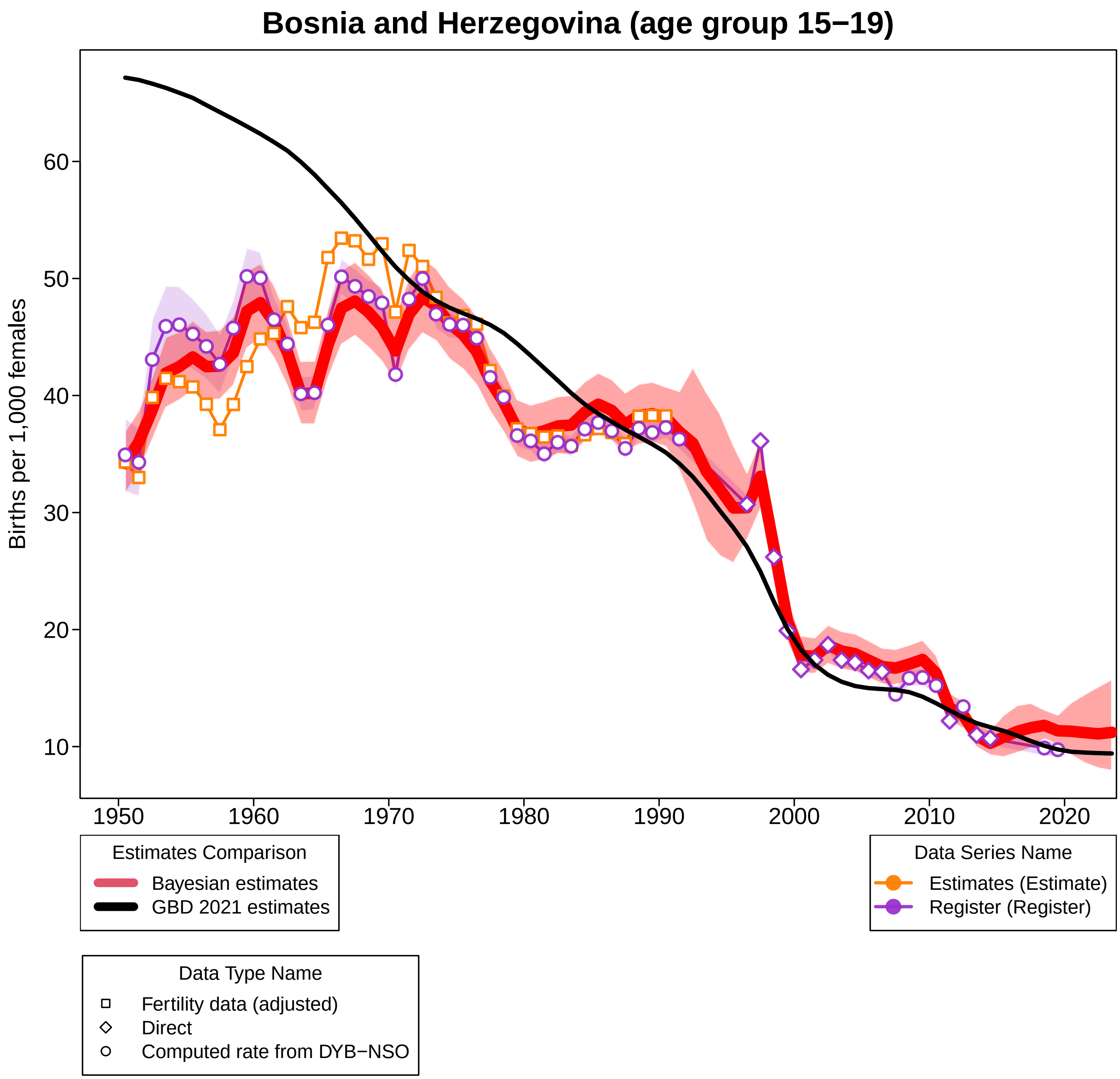
Bosnia and Herzegovina (age group 15−19)
Births per 1,000 females
60
50
40
30
20
10
1950
1960
1970
1980
1990
2000
2010
2020
Estimates Comparison
Bayesian estimates
GBD 2021 estimates
Data Series Name
Estimates (Estimate)
Register (Register)
Data Type Name
Fertility data (adjusted)
Direct
Computed rate from DYB−NSO

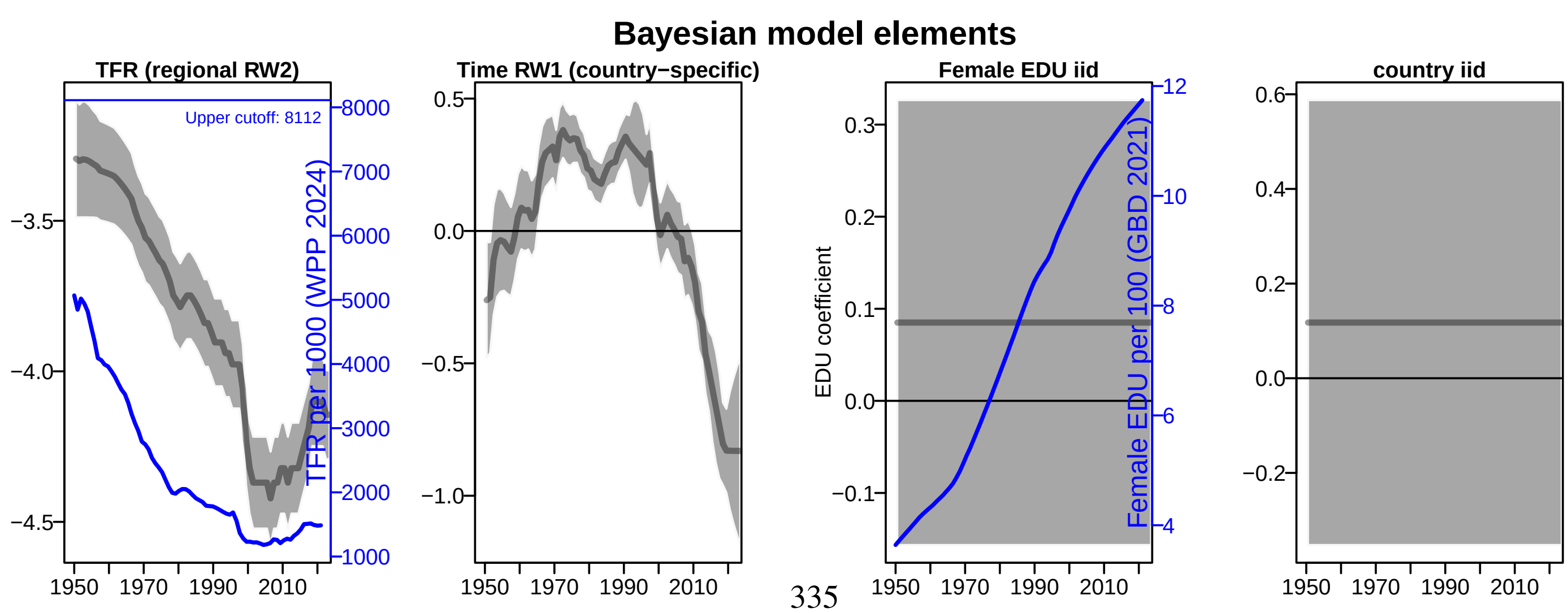
Bayesian model elements
TFR (regional RW2)
Upper cutoff: 8112
TFR per 1000 (WPP 2024)
−3.5
−4.0
−4.5
8000
7000
6000
5000
4000
3000
2000
1000
Time RW1 (country−specific)
0.5
0.0
−0.5
−1.0
Female EDU iid
EDU coefficient
Female EDU per 100 (GBD 2021)
0.3
0.2
0.1
0.0
−0.1
12
10
8
6
4
country iid
0.6
0.4
0.2
0.0
−0.2
1950
1970
1990
2010

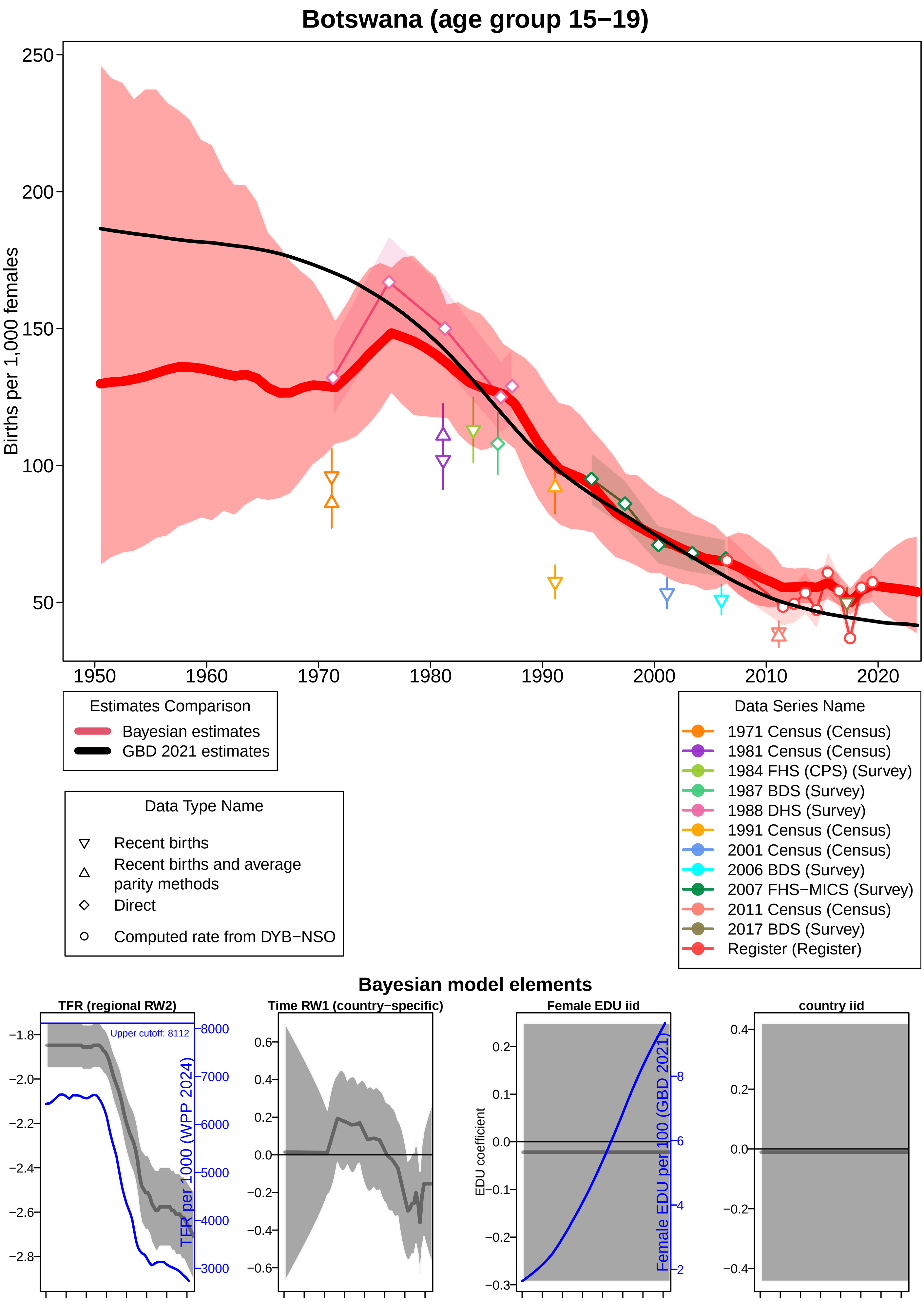

Botswana (age group 15−19)
Births per 1,000 females
250
200
150
100
50
1950
1960
1970
1980
1990
2000
2010
2020
Estimates Comparison
Bayesian estimates
GBD 2021 estimates
Data Type Name
Recent births
Recent births and average parity methods
Direct
Computed rate from DYB−NSO
Data Series Name
1971 Census (Census)
1981 Census (Census)
1984 FHS (CPS) (Survey)
1987 BDS (Survey)
1988 DHS (Survey)
1991 Census (Census)
2001 Census (Census)
2006 BDS (Survey)
2007 FHS−MICS (Survey)
2011 Census (Census)
2017 BDS (Survey)
Register (Register)
Bayesian model elements
TFR (regional RW2)
Upper cutoff: 8112
TFR per 1000 (WPP 2024)
Time RW1 (country−specific)
Female EDU iid
EDU coefficient
Female EDU per 100 (GBD 2021)
country iid

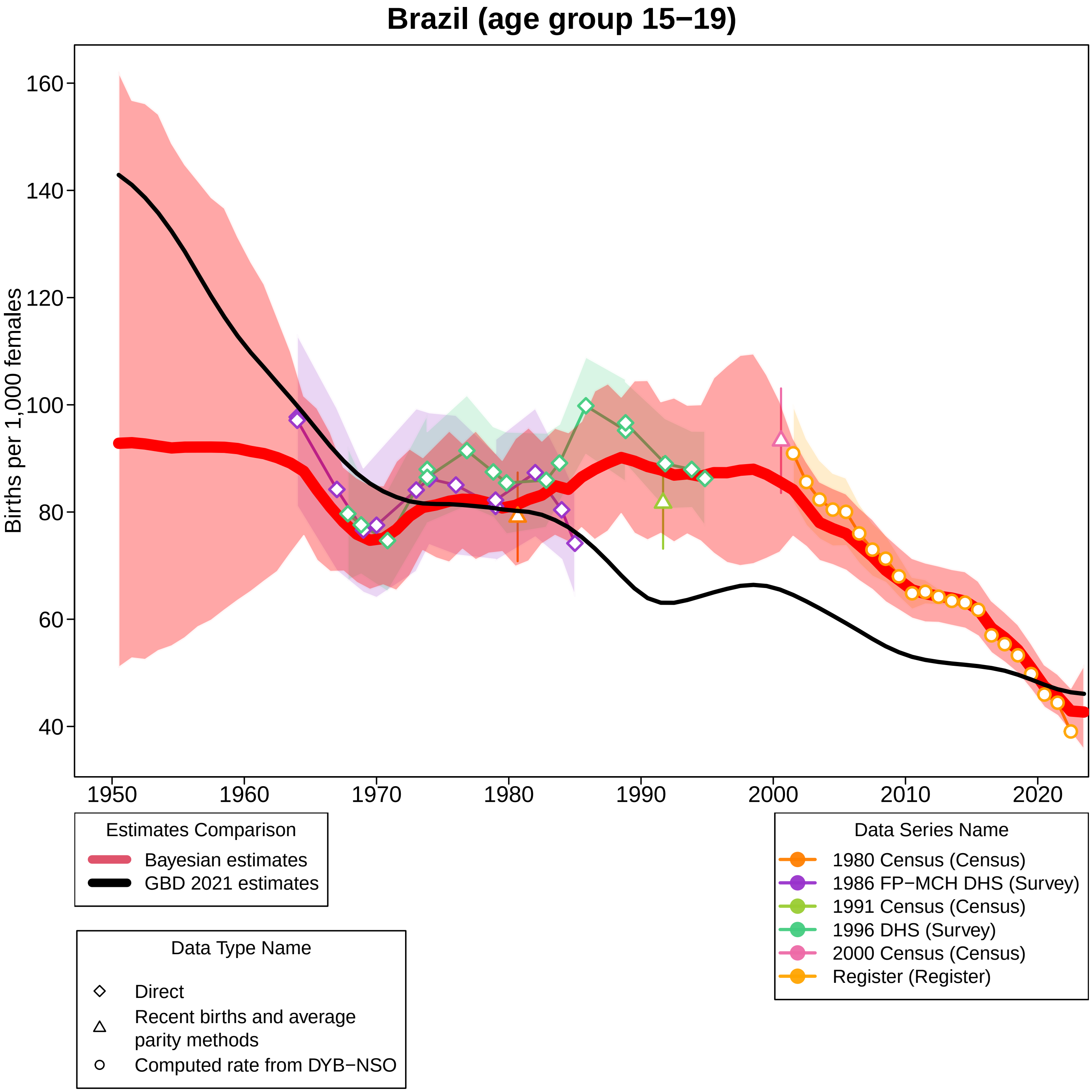
Brazil (age group 15−19)
Births per 1,000 females
160
140
120
100
80
60
40
1950
1960
1970
1980
1990
2000
2010
2020
Estimates Comparison
Bayesian estimates
GBD 2021 estimates
Data Series Name
1980 Census (Census)
1986 FP−MCH DHS (Survey)
1991 Census (Census)
1996 DHS (Survey)
2000 Census (Census)
Register (Register)
Data Type Name
Direct
Recent births and average parity methods
Computed rate from DYB−NSO

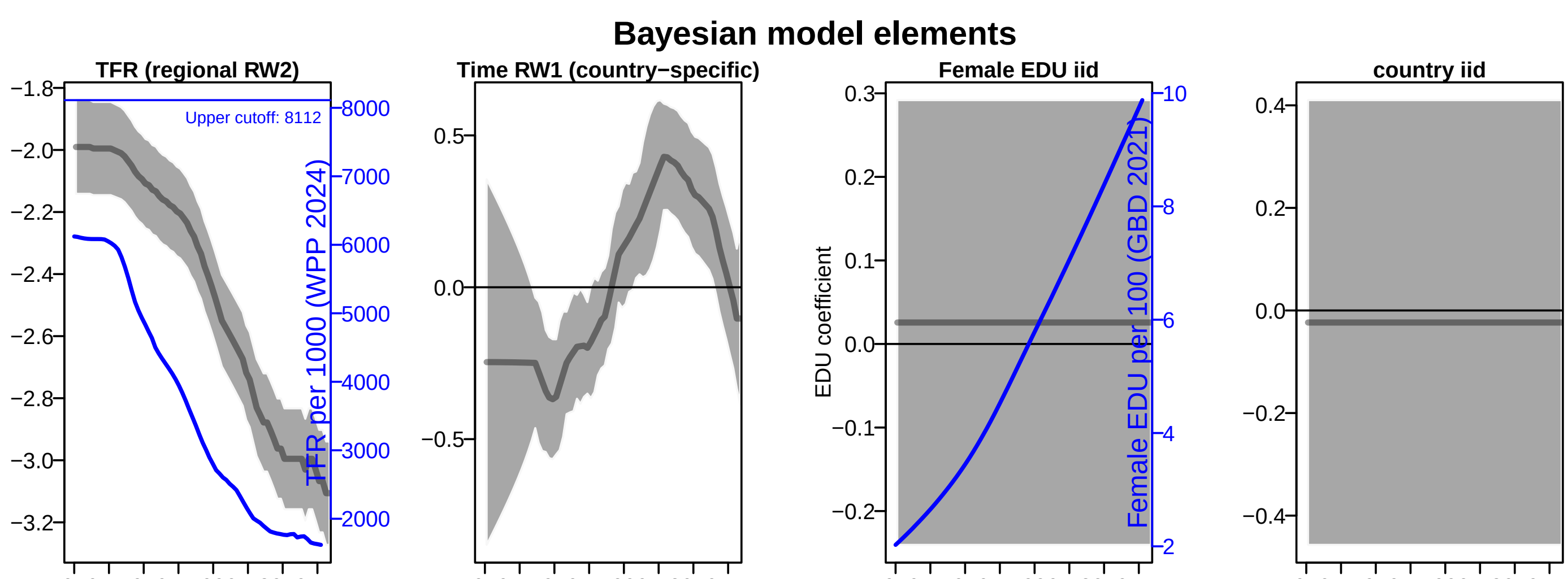
Bayesian model elements
TFR (regional RW2)
Upper cutoff: 8112
TFR per 1000 (WPP 2024)
−1.8
−2.0
−2.2
−2.4
−2.6
−2.8
−3.0
−3.2
8000
7000
6000
5000
4000
3000
2000
Time RW1 (country−specific)
0.5
0.0
−0.5
Female EDU iid
EDU coefficient
Female EDU per 100 (GBD 2021)
0.3
0.2
0.1
0.0
−0.1
−0.2
10
8
6
4
2
country iid
0.4
0.2
0.0
−0.2
−0.4
1950
1970
1990
2010

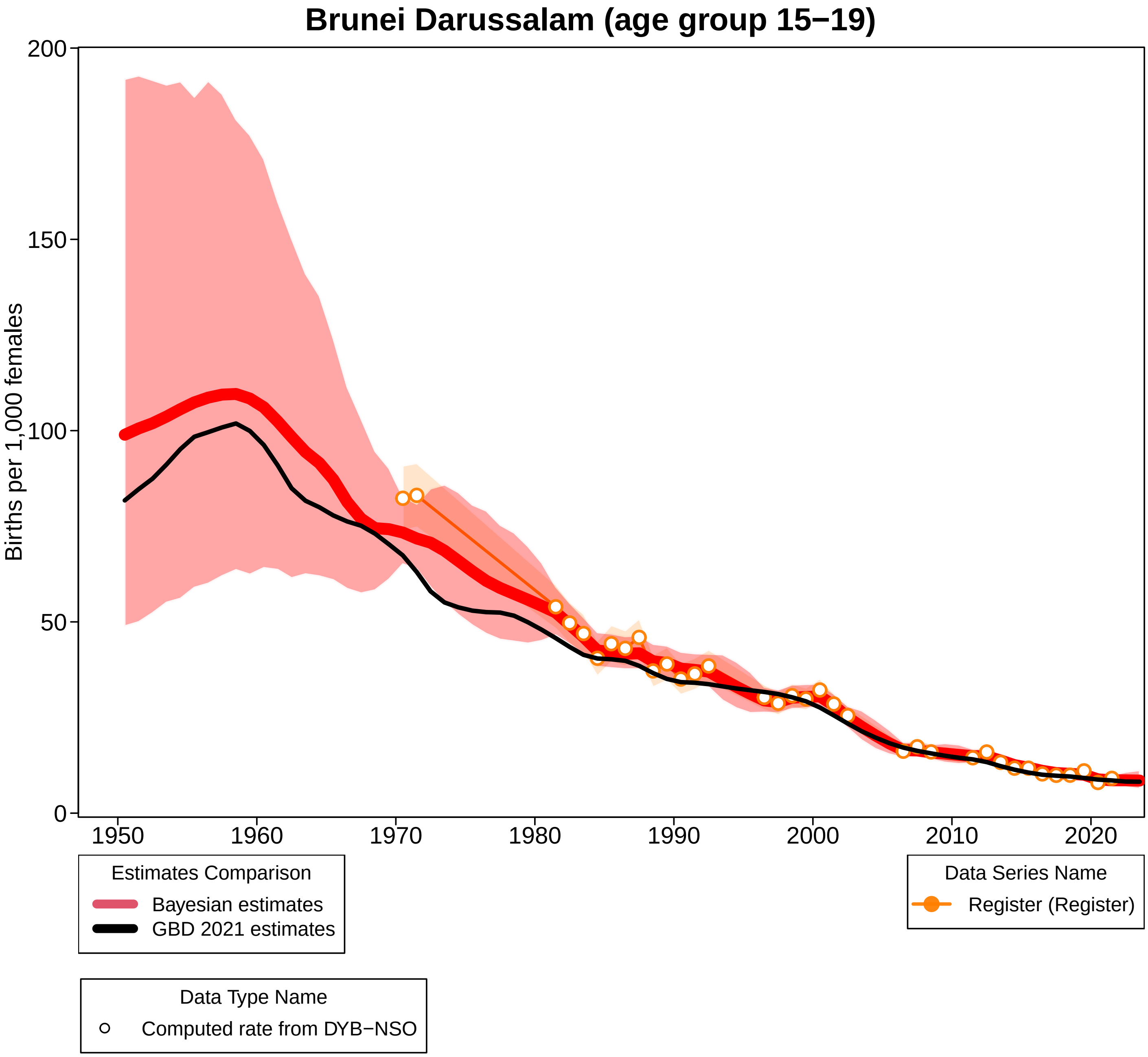
Brunei Darussalam (age group 15−19)
Births per 1,000 females
200
150
100
50
0
1950
1960
1970
1980
1990
2000
2010
2020
Estimates Comparison
Bayesian estimates
GBD 2021 estimates
Data Series Name
Register (Register)
Data Type Name
Computed rate from DYB−NSO

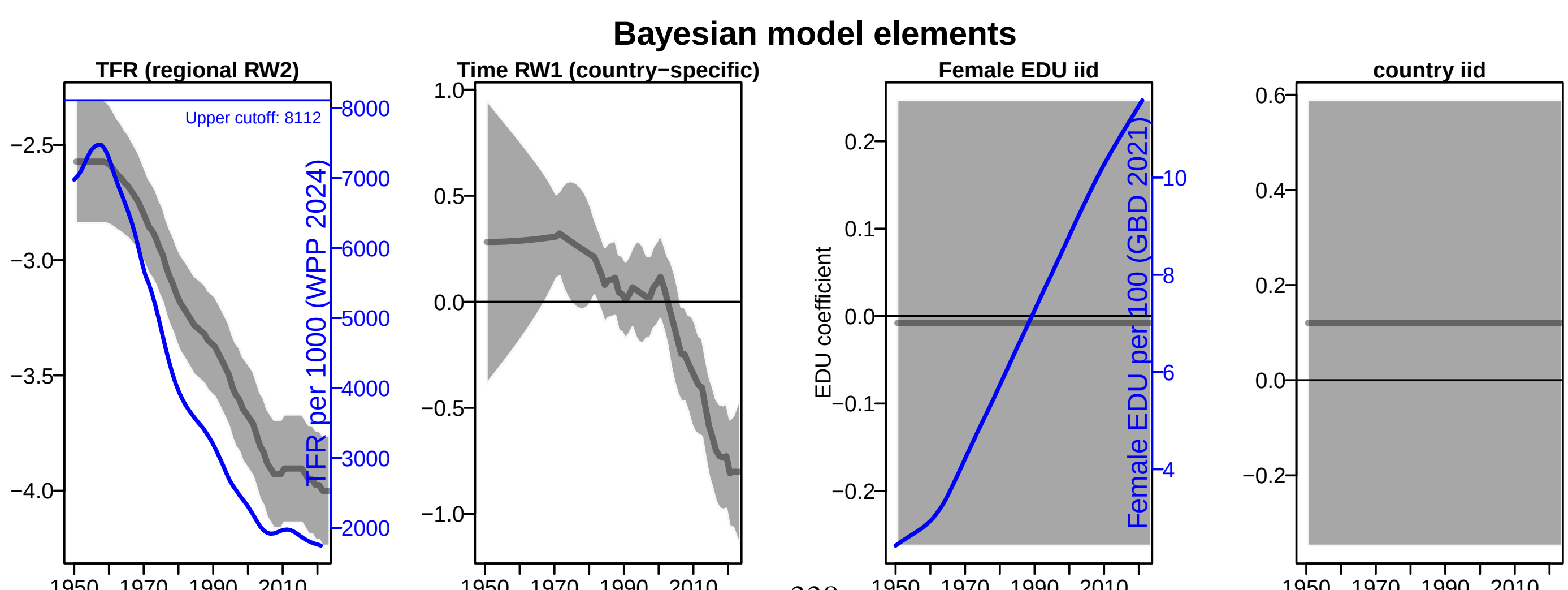
Bayesian model elements
TFR (regional RW2)
Upper cutoff: 8112
TFR per 1000 (WPP 2024)
Time RW1 (country−specific)
Female EDU iid
EDU coefficient
Female EDU per 100 (GBD 2021)
country iid

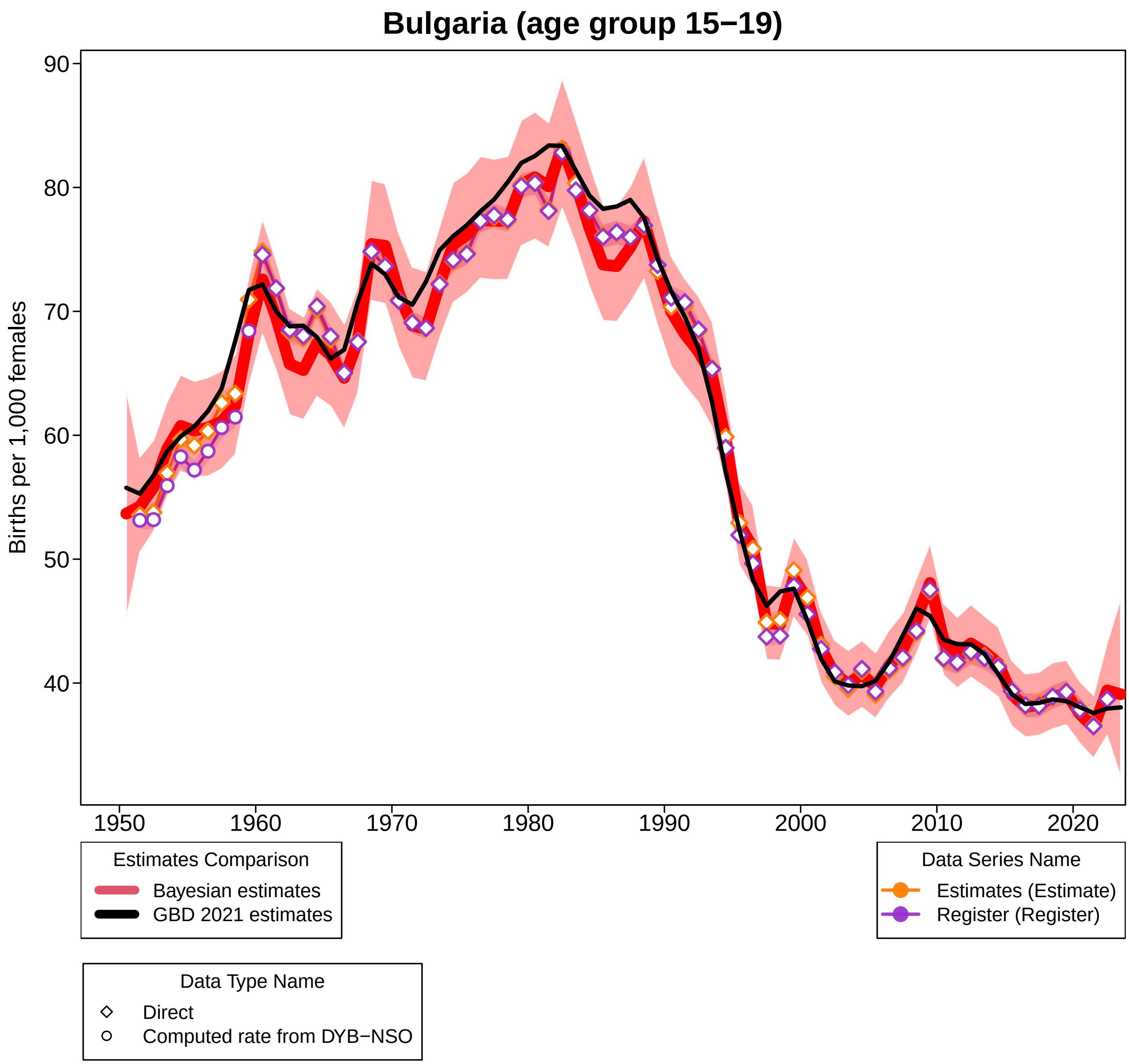

Bulgaria (age group 15−19)
Births per 1,000 females
90
80
70
60
50
40
1950
1960
1970
1980
1990
2000
2010
2020
Estimates Comparison
Bayesian estimates
GBD 2021 estimates
Data Series Name
Estimates (Estimate)
Register (Register)
Data Type Name
Direct
Computed rate from DYB−NSO

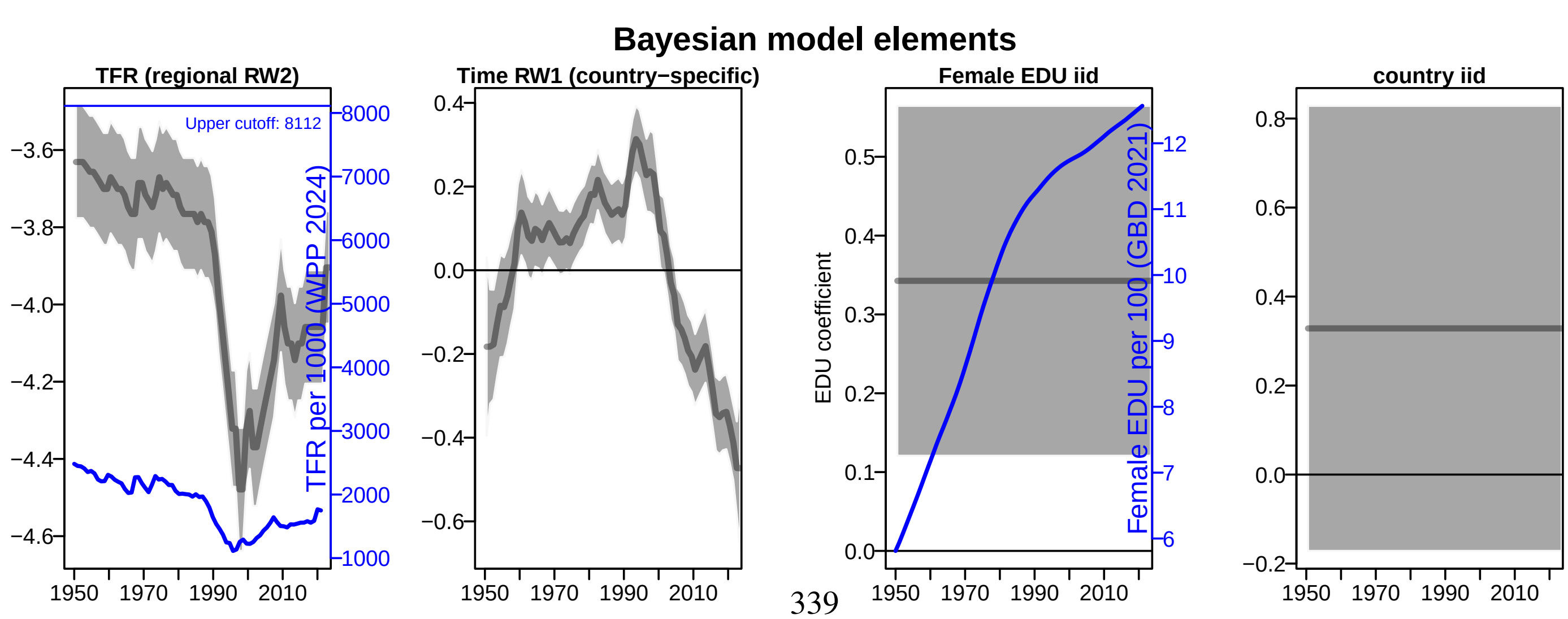

Bayesian model elements
TFR (regional RW2)
Upper cutoff: 8112
TFR per 1000 (WPP 2024)
Time RW1 (country−specific)
Female EDU iid
EDU coefficient
Female EDU per 100 (GBD 2021)
country iid

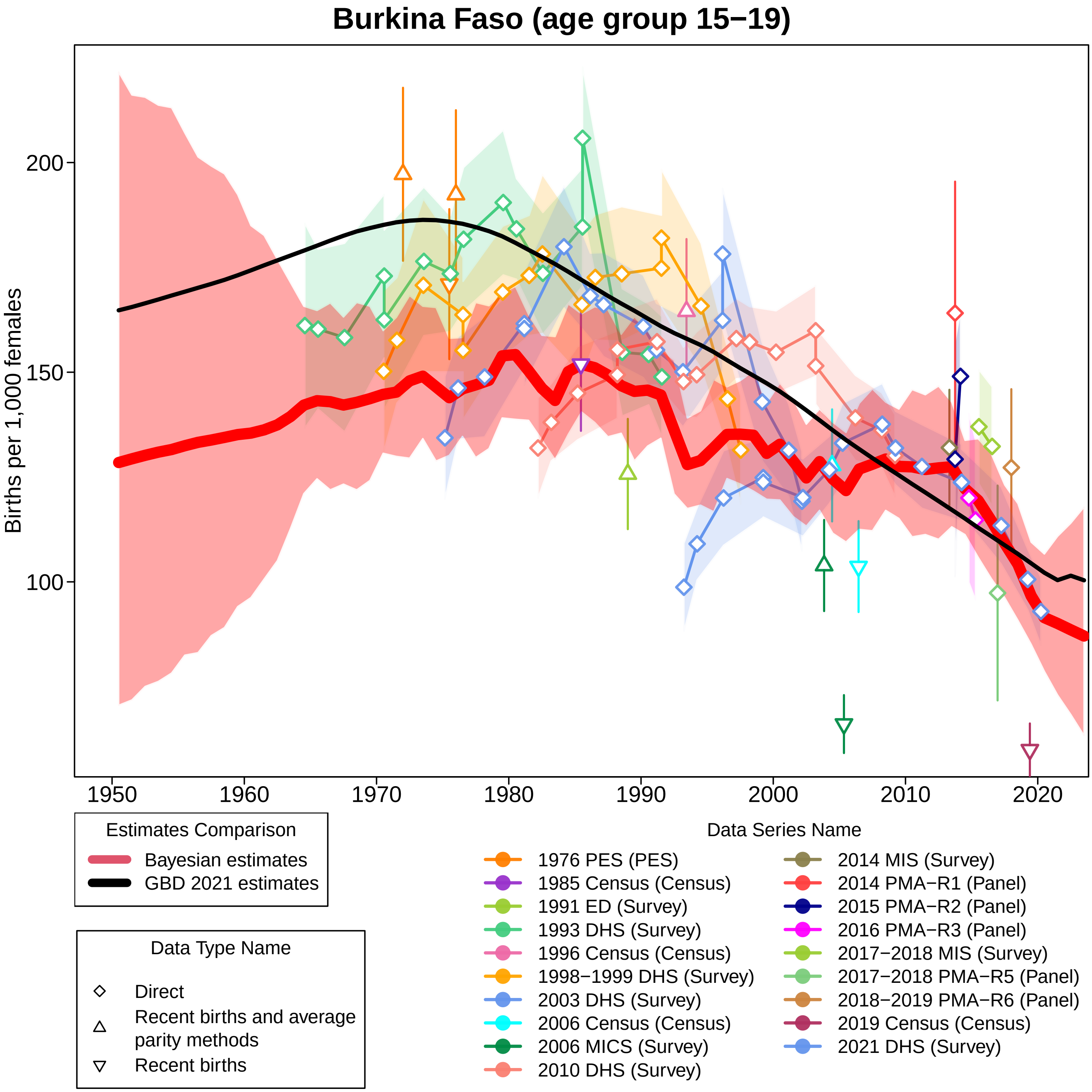
Burkina Faso (age group 15−19)
Births per 1,000 females
200
150
100
1950
1960
1970
1980
1990
2000
2010
2020
Estimates Comparison
Bayesian estimates
GBD 2021 estimates
Data Type Name
Direct
Recent births and average parity methods
Recent births
Data Series Name
1976 PES (PES)
1985 Census (Census)
1991 ED (Survey)
1993 DHS (Survey)
1996 Census (Census)
1998−1999 DHS (Survey)
2003 DHS (Survey)
2006 Census (Census)
2006 MICS (Survey)
2010 DHS (Survey)
2014 MIS (Survey)
2014 PMA−R1 (Panel)
2015 PMA−R2 (Panel)
2016 PMA−R3 (Panel)
2017−2018 MIS (Survey)
2017−2018 PMA−R5 (Panel)
2018−2019 PMA−R6 (Panel)
2019 Census (Census)
2021 DHS (Survey)

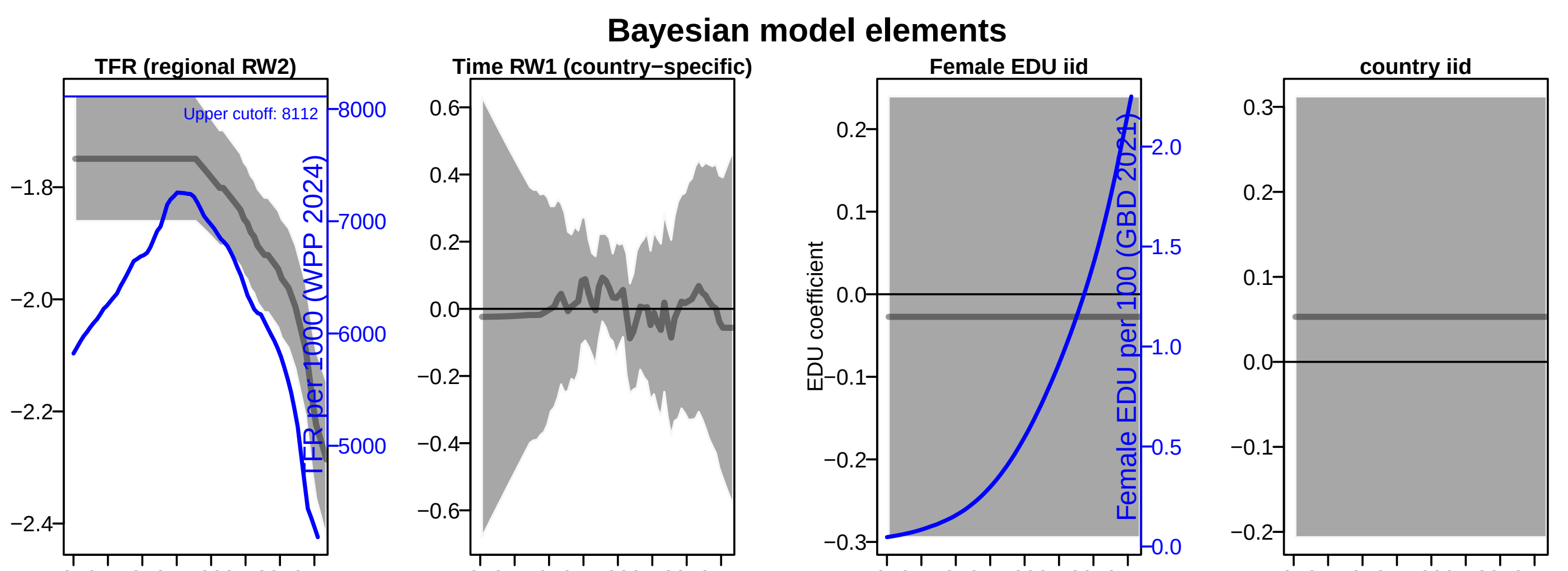
Bayesian model elements
TFR (regional RW2)
Upper cutoff: 8112
TFR per 1000 (WPP 2024)
−1.8
−2.0
−2.2
−2.4
8000
7000
6000
5000
Time RW1 (country−specific)
0.6
0.4
0.2
0.0
−0.2
−0.4
−0.6
Female EDU iid
EDU coefficient
Female EDU per 100 (GBD 2021)
0.2
0.1
0.0
−0.1
−0.2
−0.3
2.0
1.5
1.0
0.5
0.0
country iid
0.3
0.2
0.1
0.0
−0.1
−0.2
1950
1970
1990
2010

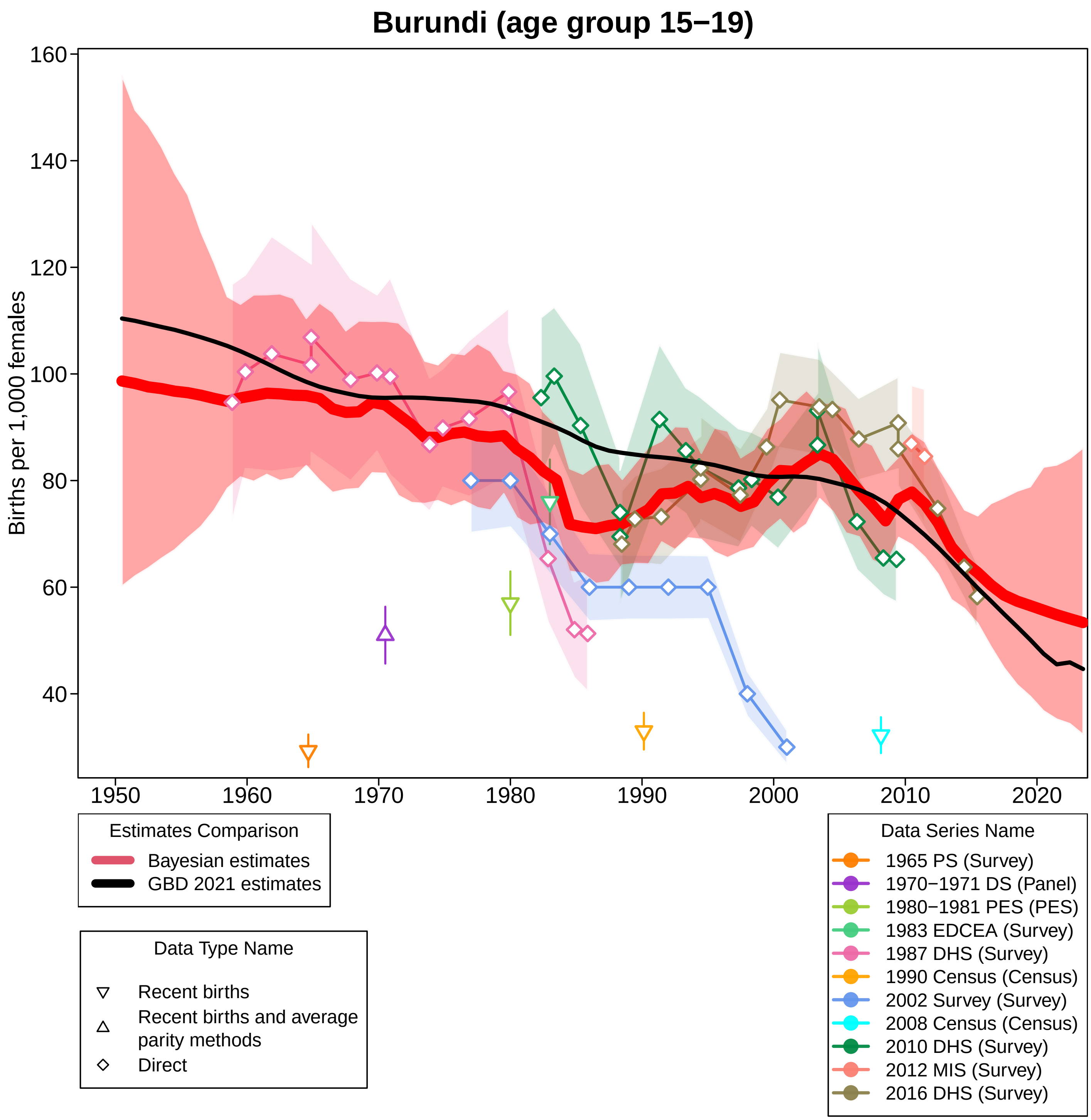
Burundi (age group 15−19)
Births per 1,000 females
160
140
120
100
80
60
40
1950
1960
1970
1980
1990
2000
2010
2020
Estimates Comparison
Bayesian estimates
GBD 2021 estimates
Data Type Name
Recent births
Recent births and average parity methods
Direct
Data Series Name
1965 PS (Survey)
1970−1971 DS (Panel)
1980−1981 PES (PES)
1983 EDCEA (Survey)
1987 DHS (Survey)
1990 Census (Census)
2002 Survey (Survey)
2008 Census (Census)
2010 DHS (Survey)
2012 MIS (Survey)
2016 DHS (Survey)

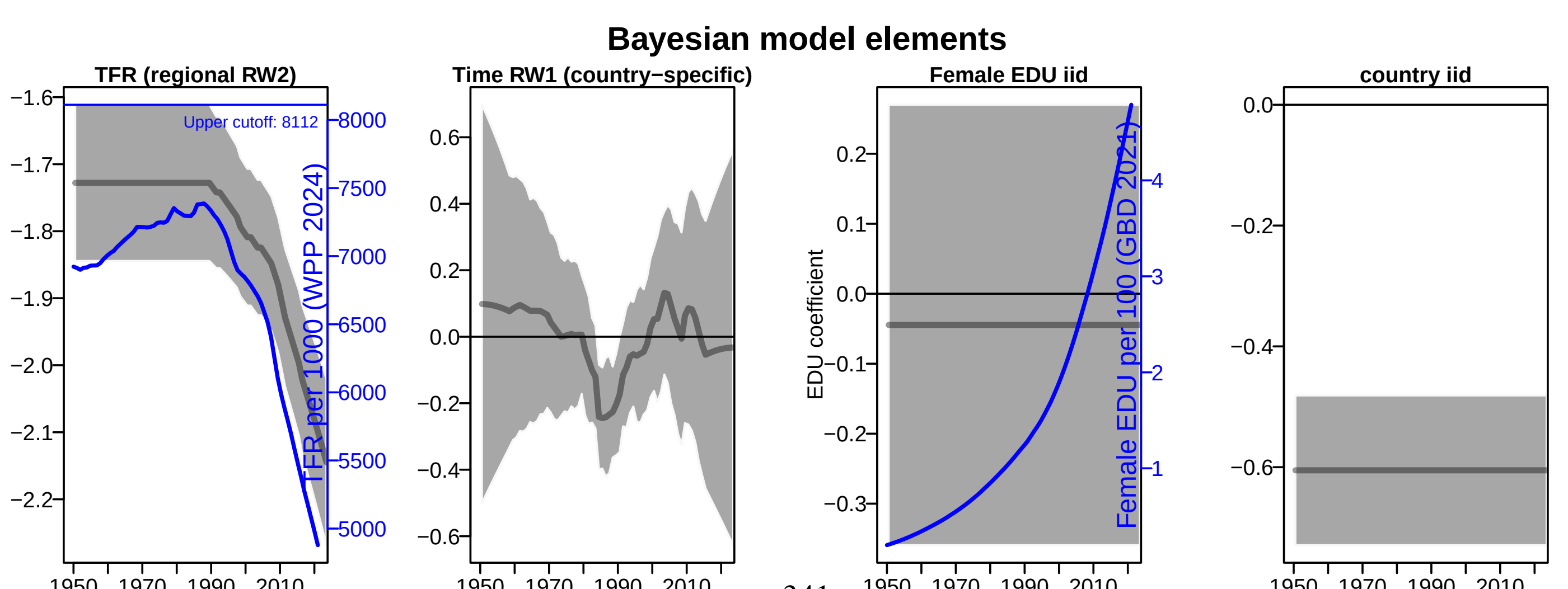
Bayesian model elements
TFR (regional RW2)
Upper cutoff: 8112
TFR per 1000 (WPP 2024)
Time RW1 (country−specific)
Female EDU iid
EDU coefficient
Female EDU per 100 (GBD 2021)
country iid

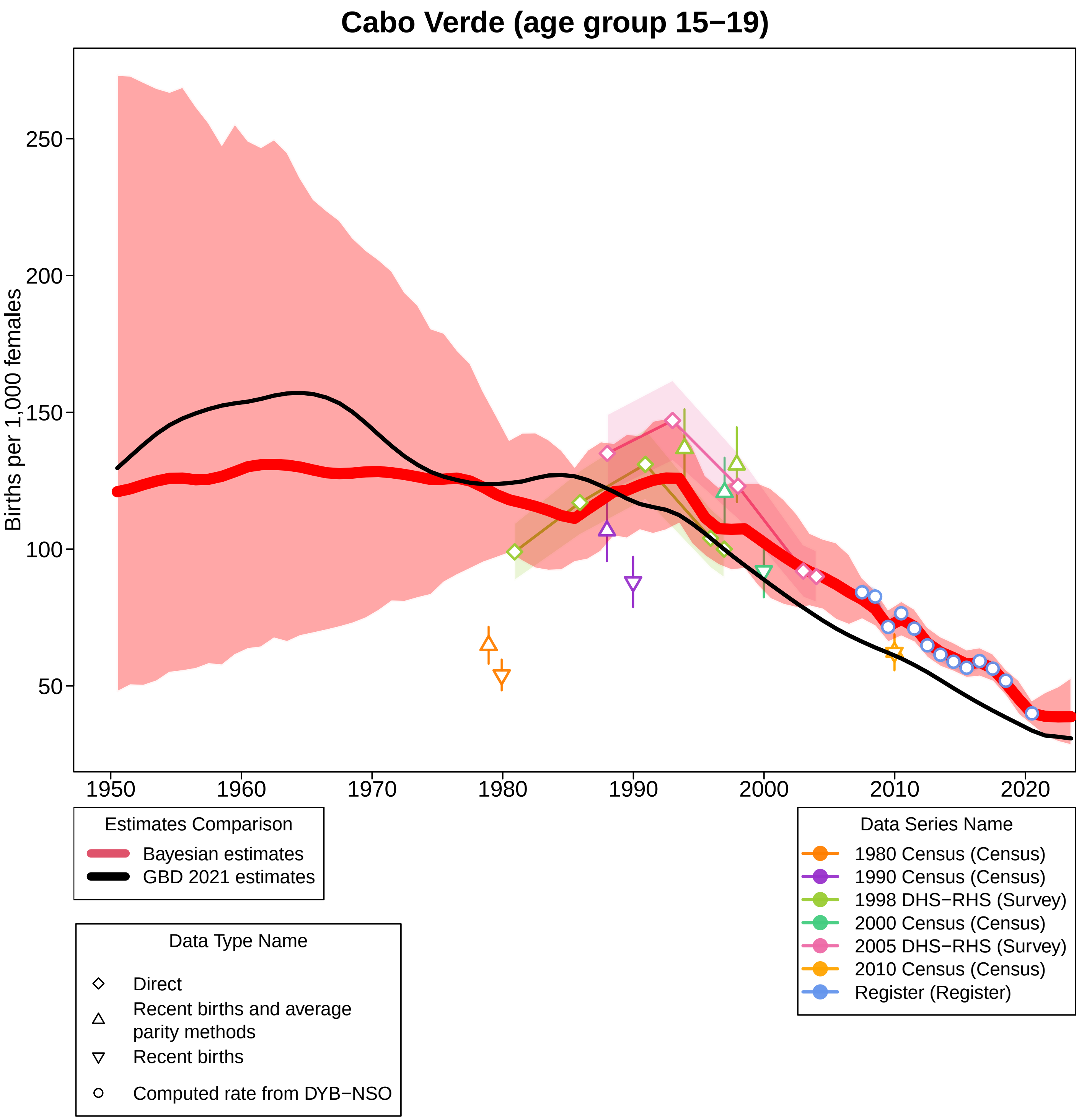
Cabo Verde (age group 15−19)
Births per 1,000 females
250
200
150
100
50
1950
1960
1970
1980
1990
2000
2010
2020
Estimates Comparison
Bayesian estimates
GBD 2021 estimates
Data Series Name
1980 Census (Census)
1990 Census (Census)
1998 DHS−RHS (Survey)
2000 Census (Census)
2005 DHS−RHS (Survey)
2010 Census (Census)
Register (Register)
Data Type Name
Direct
Recent births and average parity methods
Recent births
Computed rate from DYB−NSO

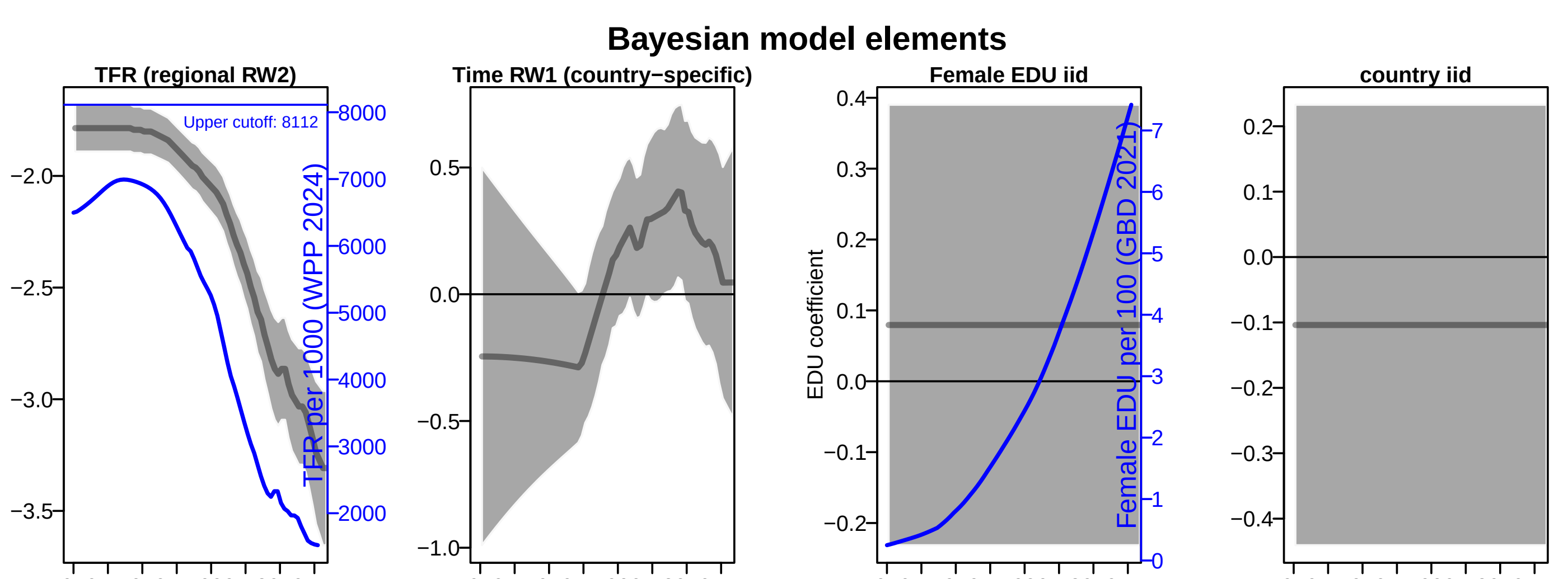
Bayesian model elements
TFR (regional RW2)
Upper cutoff: 8112
TFR per 1000 (WPP 2024)
Time RW1 (country−specific)
Female EDU iid
EDU coefficient
Female EDU per 100 (GBD 2021)
country iid
1950
1970
1990
2010

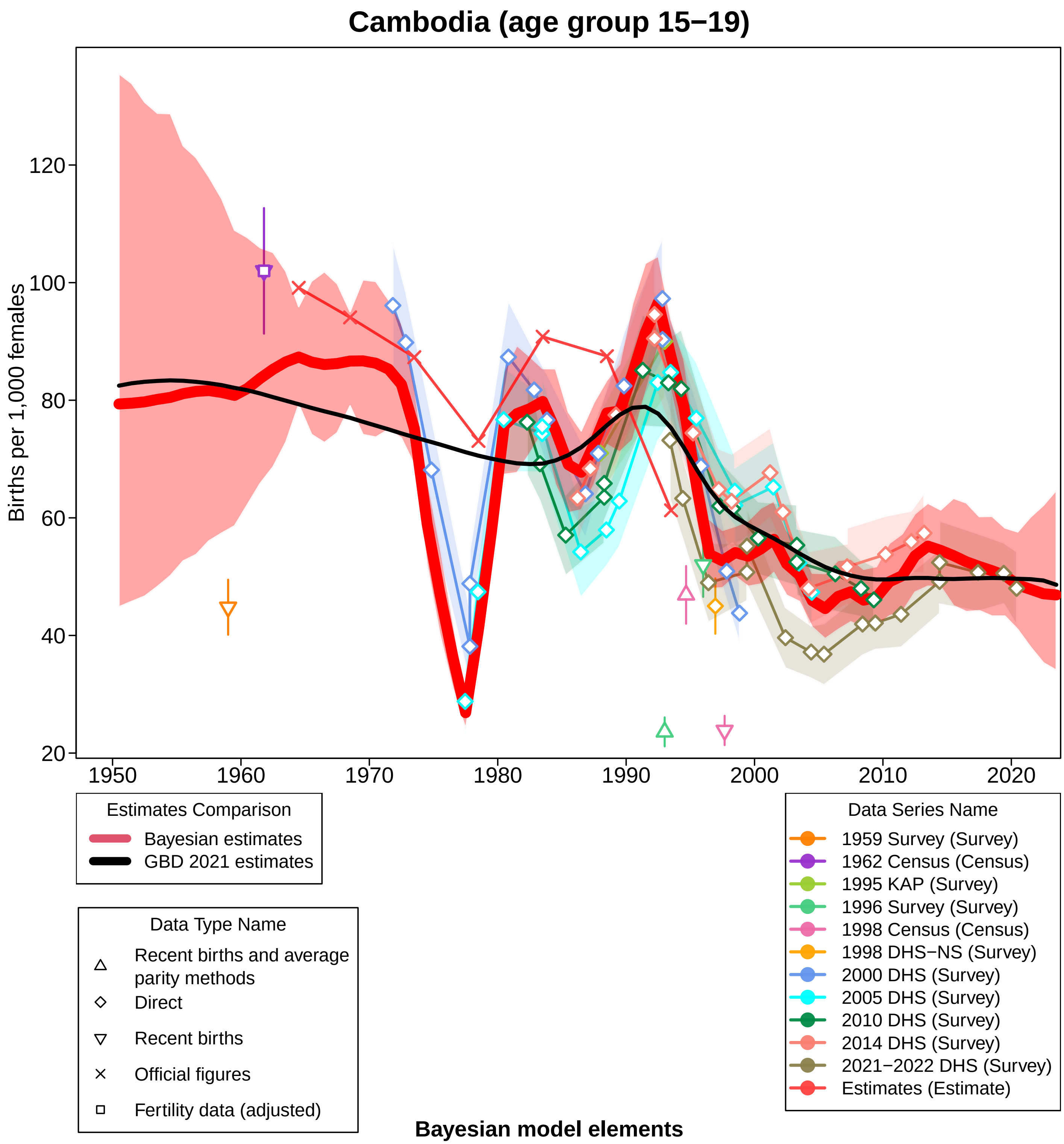

Cambodia (age group 15−19)
Births per 1,000 females
20
40
60
80
100
120
1950
1960
1970
1980
1990
2000
2010
2020
Estimates Comparison
Bayesian estimates
GBD 2021 estimates
Data Type Name
Recent births and average parity methods
Direct
Recent births
Official figures
Fertility data (adjusted)
Data Series Name
1959 Survey (Survey)
1962 Census (Census)
1995 KAP (Survey)
1996 Survey (Survey)
1998 Census (Census)
1998 DHS−NS (Survey)
2000 DHS (Survey)
2005 DHS (Survey)
2010 DHS (Survey)
2014 DHS (Survey)
2021−2022 DHS (Survey)
Estimates (Estimate)

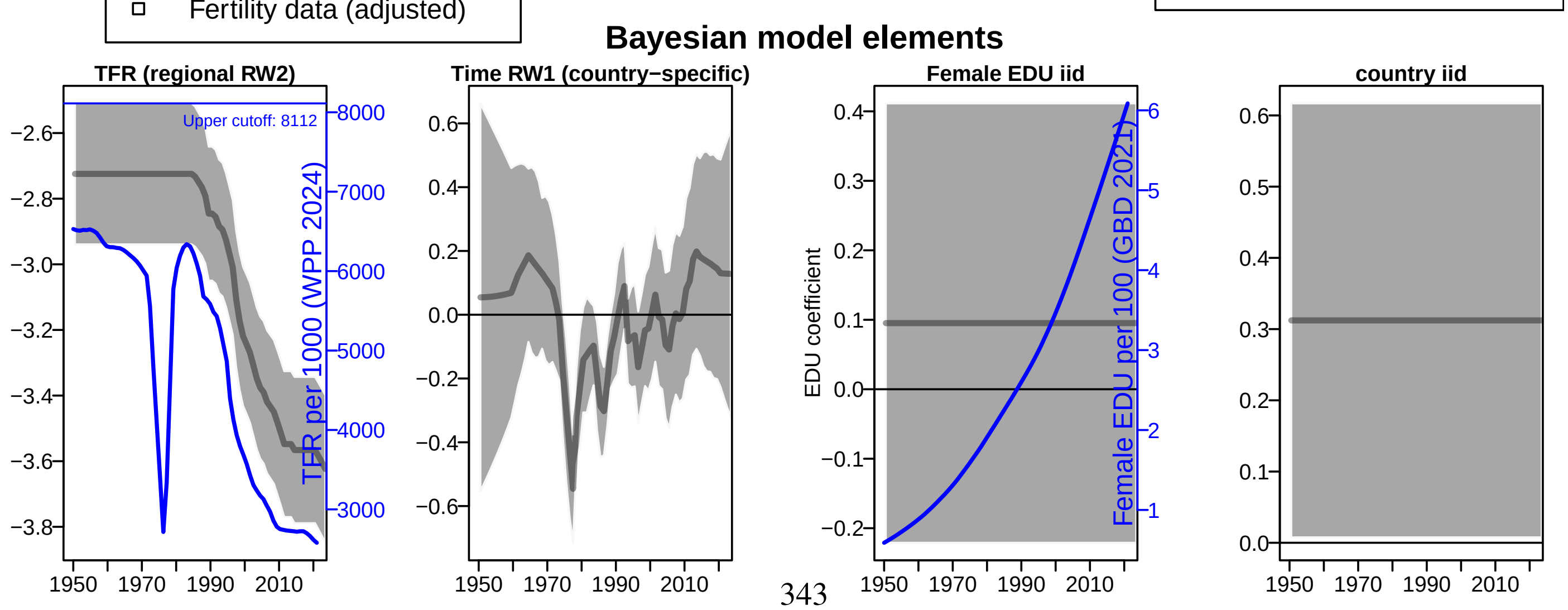

Bayesian model elements
TFR (regional RW2)
Upper cutoff: 8112
TFR per 1000 (WPP 2024)
Time RW1 (country−specific)
Female EDU iid
EDU coefficient
Female EDU per 100 (GBD 2021)
country iid

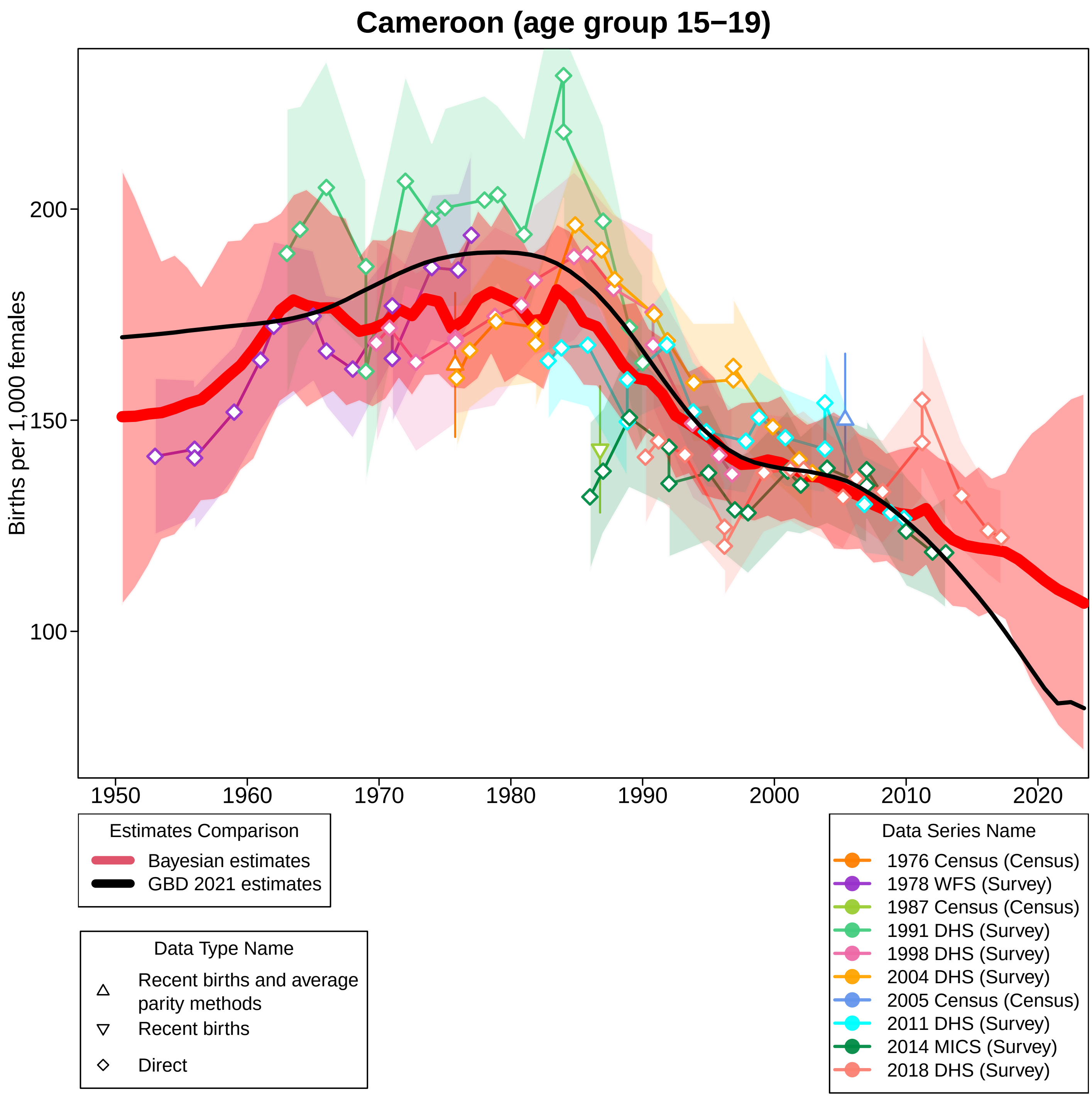
Cameroon (age group 15−19)
Births per 1,000 females
200
150
100
1950
1960
1970
1980
1990
2000
2010
2020
Estimates Comparison
Bayesian estimates
GBD 2021 estimates
Data Type Name
Recent births and average parity methods
Recent births
Direct
Data Series Name
1976 Census (Census)
1978 WFS (Survey)
1987 Census (Census)
1991 DHS (Survey)
1998 DHS (Survey)
2004 DHS (Survey)
2005 Census (Census)
2011 DHS (Survey)
2014 MICS (Survey)
2018 DHS (Survey)

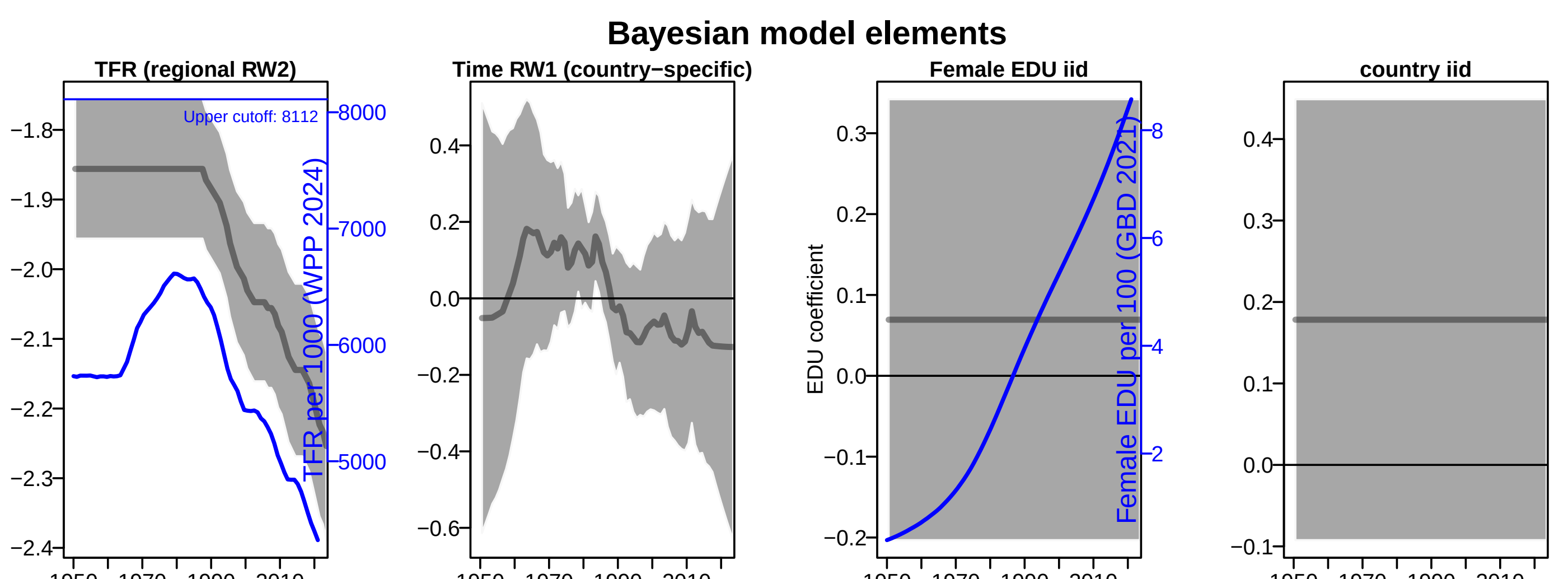
Bayesian model elements
TFR (regional RW2)
Upper cutoff: 8112
TFR per 1000 (WPP 2024)
Time RW1 (country−specific)
Female EDU iid
EDU coefficient
Female EDU per 100 (GBD 2021)
country iid

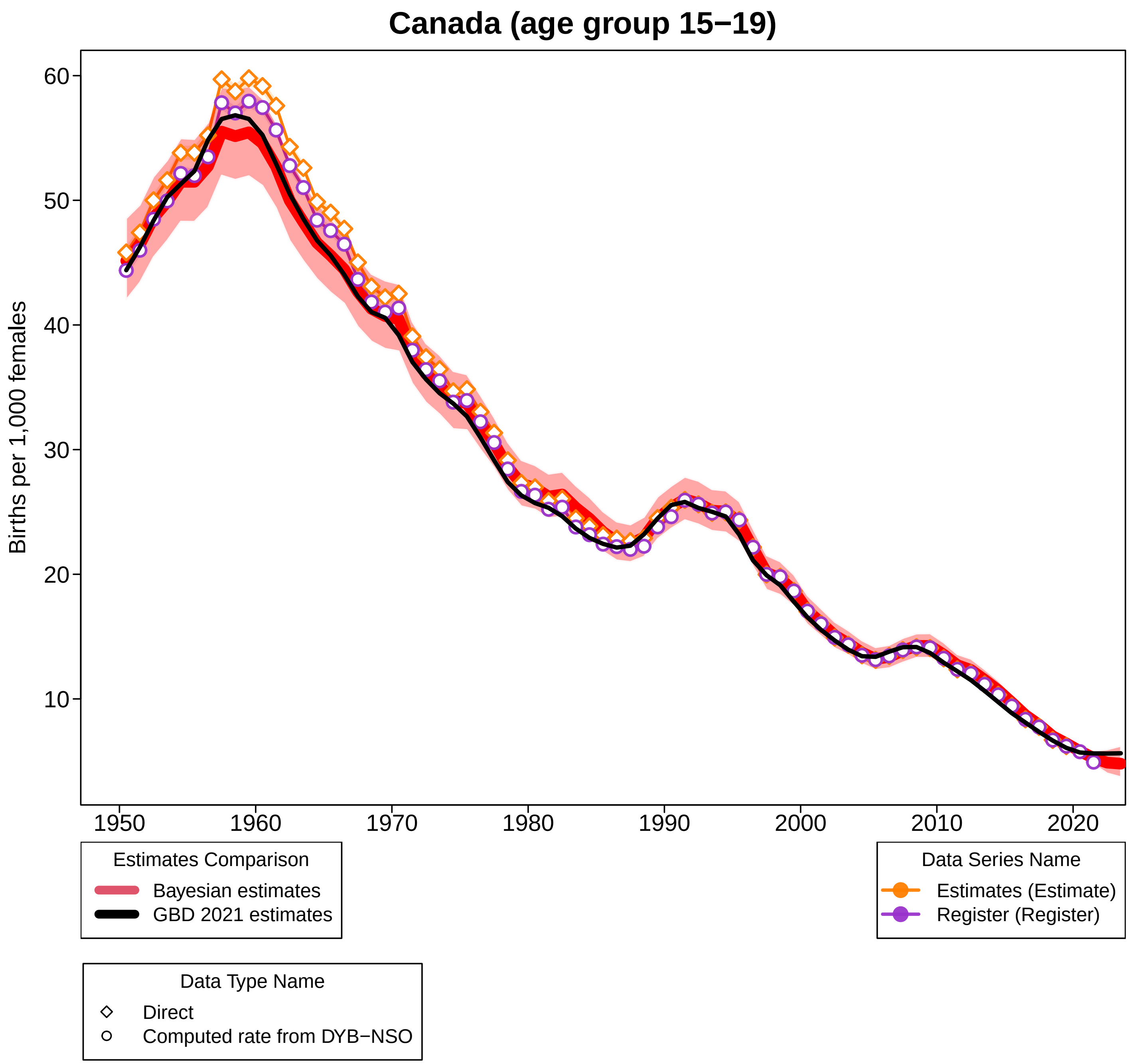
Canada (age group 15−19)
Births per 1,000 females
60
50
40
30
20
10
1950
1960
1970
1980
1990
2000
2010
2020
Estimates Comparison
Bayesian estimates
GBD 2021 estimates
Data Series Name
Estimates (Estimate)
Register (Register)
Data Type Name
Direct
Computed rate from DYB−NSO

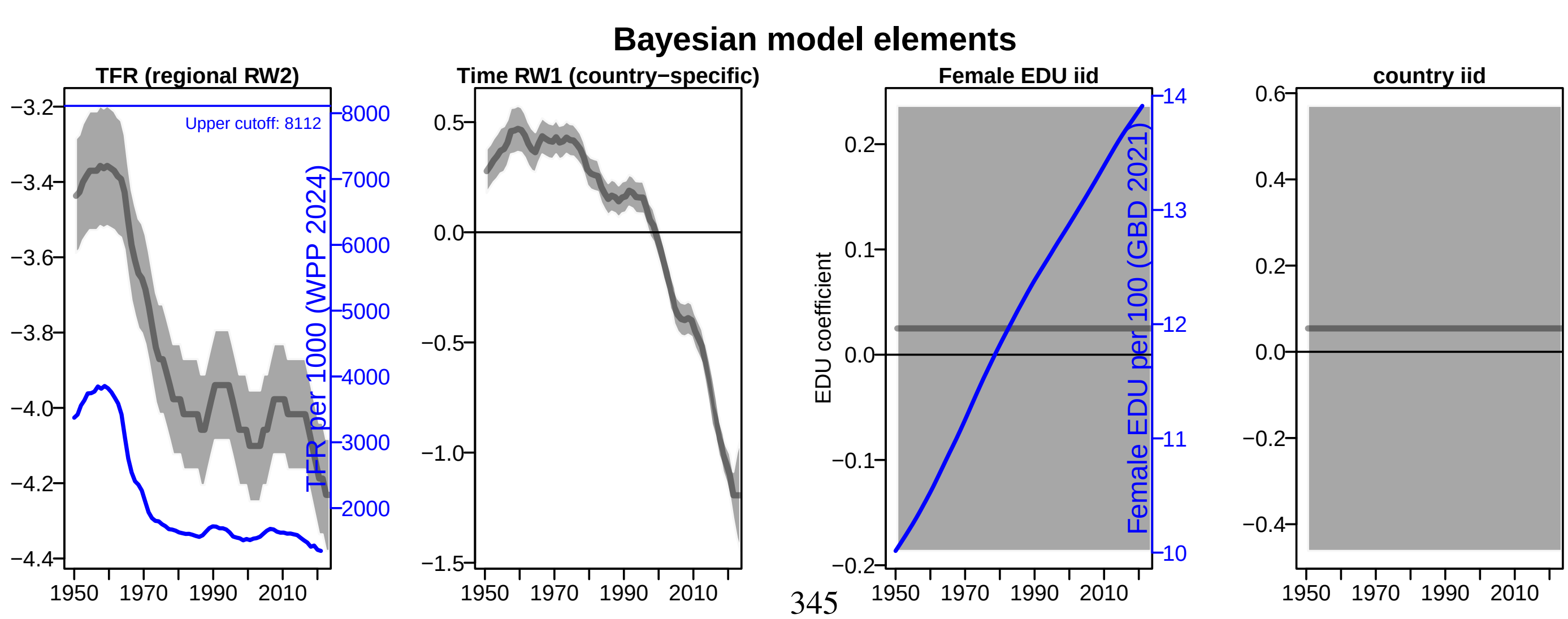
Bayesian model elements
TFR (regional RW2)
Upper cutoff: 8112
TFR per 1000 (WPP 2024)
Time RW1 (country−specific)
Female EDU iid
EDU coefficient
Female EDU per 100 (GBD 2021)
country iid

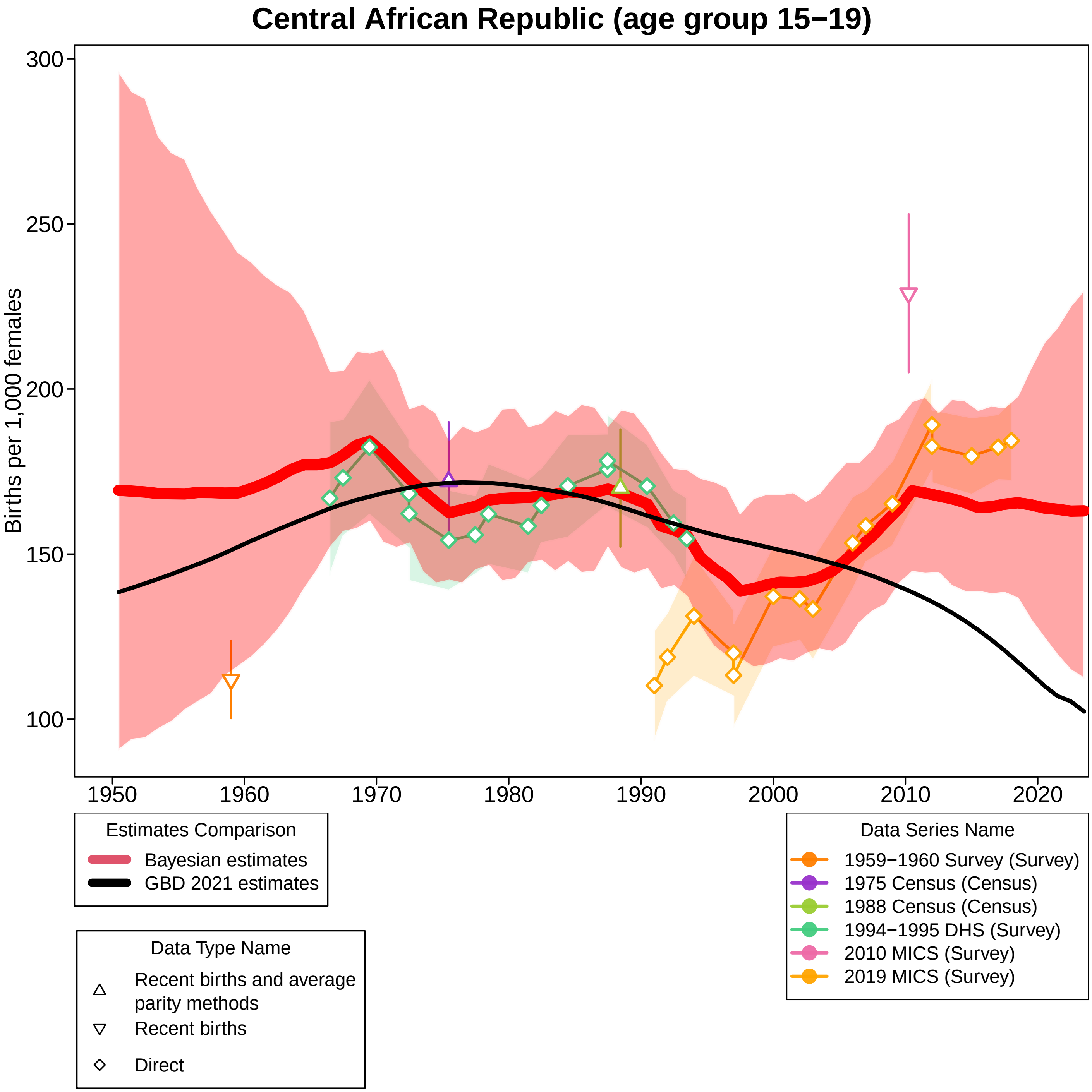
Central African Republic (age group 15−19)
Births per 1,000 females
300
250
200
150
100
1950
1960
1970
1980
1990
2000
2010
2020
Estimates Comparison
Bayesian estimates
GBD 2021 estimates
Data Series Name
1959−1960 Survey (Survey)
1975 Census (Census)
1988 Census (Census)
1994−1995 DHS (Survey)
2010 MICS (Survey)
2019 MICS (Survey)
Data Type Name
Recent births and average parity methods
Recent births
Direct

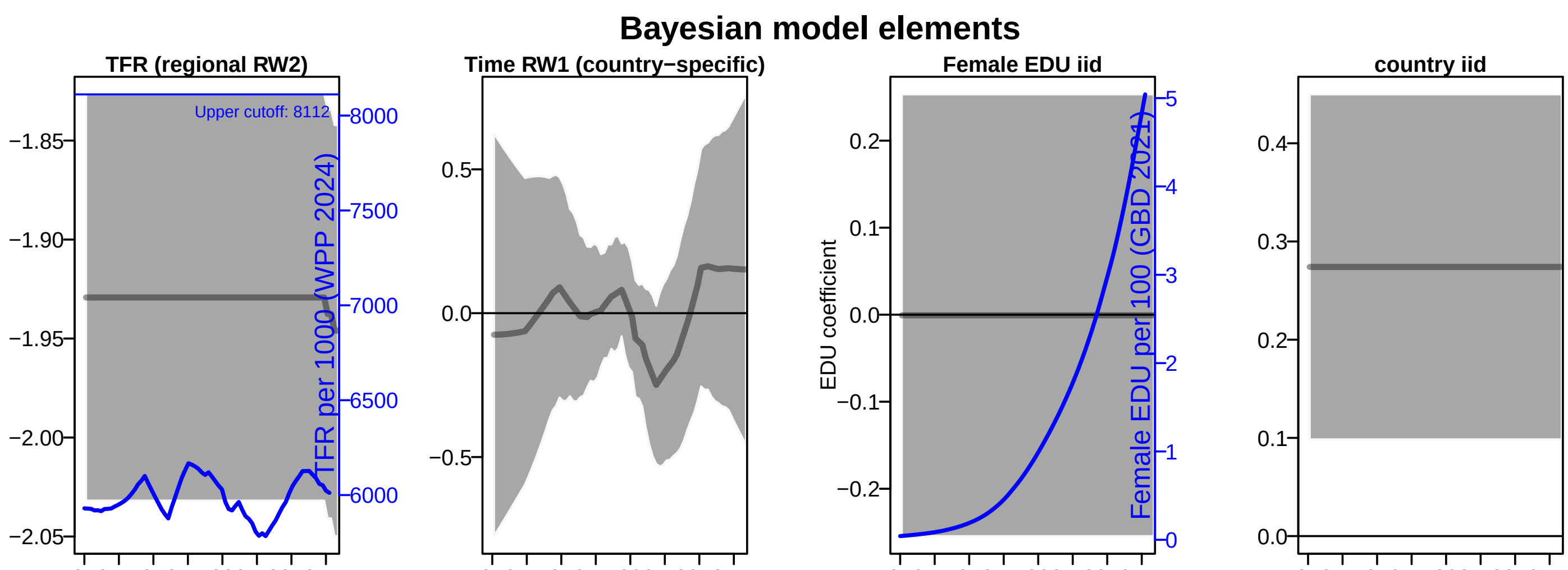
Bayesian model elements
TFR (regional RW2)
Upper cutoff: 8112
TFR per 1000 (WPP 2024)
Time RW1 (country−specific)
Female EDU iid
EDU coefficient
Female EDU per 100 (GBD 2021)
country iid

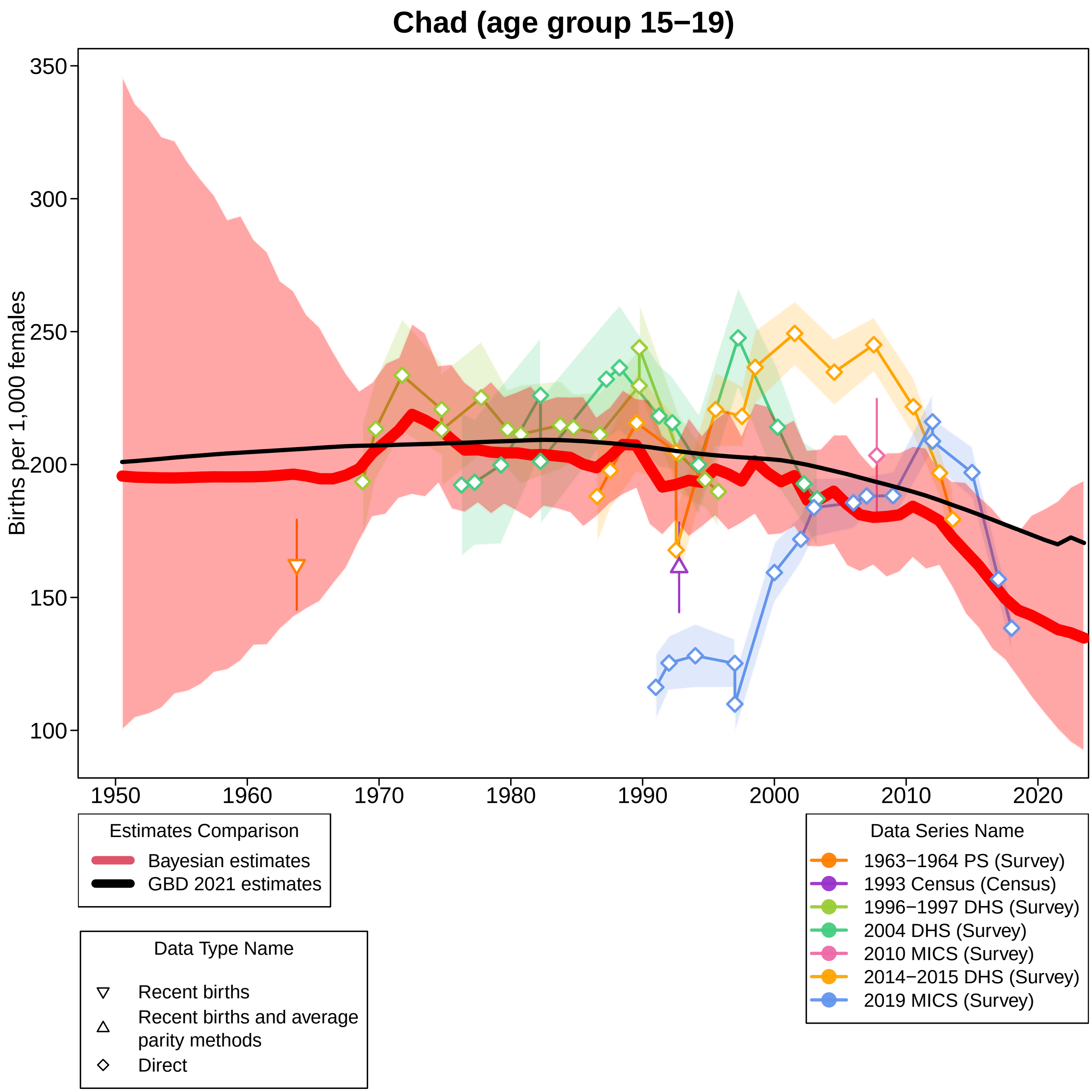
Chad (age group 15−19)
Births per 1,000 females
350
300
250
200
150
100
1950
1960
1970
1980
1990
2000
2010
2020
Estimates Comparison
Bayesian estimates
GBD 2021 estimates
Data Type Name
Recent births
Recent births and average parity methods
Direct
Data Series Name
1963−1964 PS (Survey)
1993 Census (Census)
1996−1997 DHS (Survey)
2004 DHS (Survey)
2010 MICS (Survey)
2014−2015 DHS (Survey)
2019 MICS (Survey)

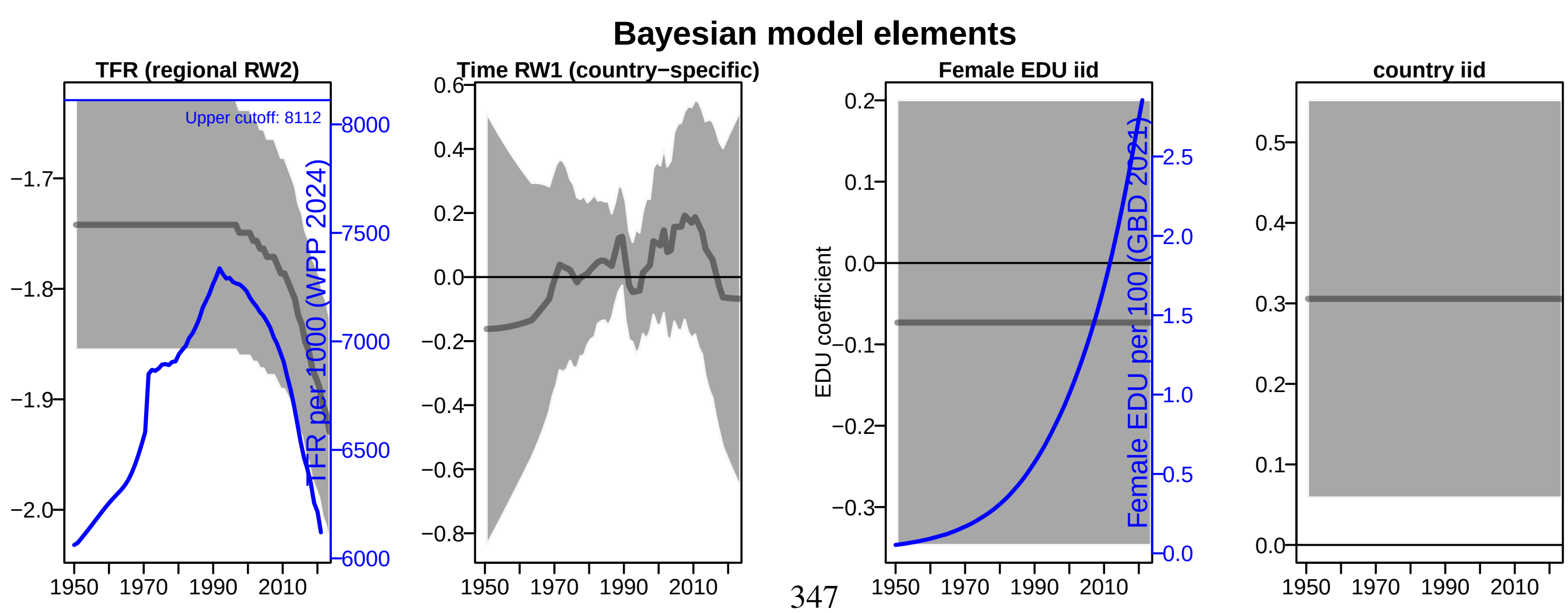
Bayesian model elements
TFR (regional RW2)
Upper cutoff: 8112
TFR per 1000 (WPP 2024)
Time RW1 (country−specific)
Female EDU iid
EDU coefficient
Female EDU per 100 (GBD 2021)
country iid

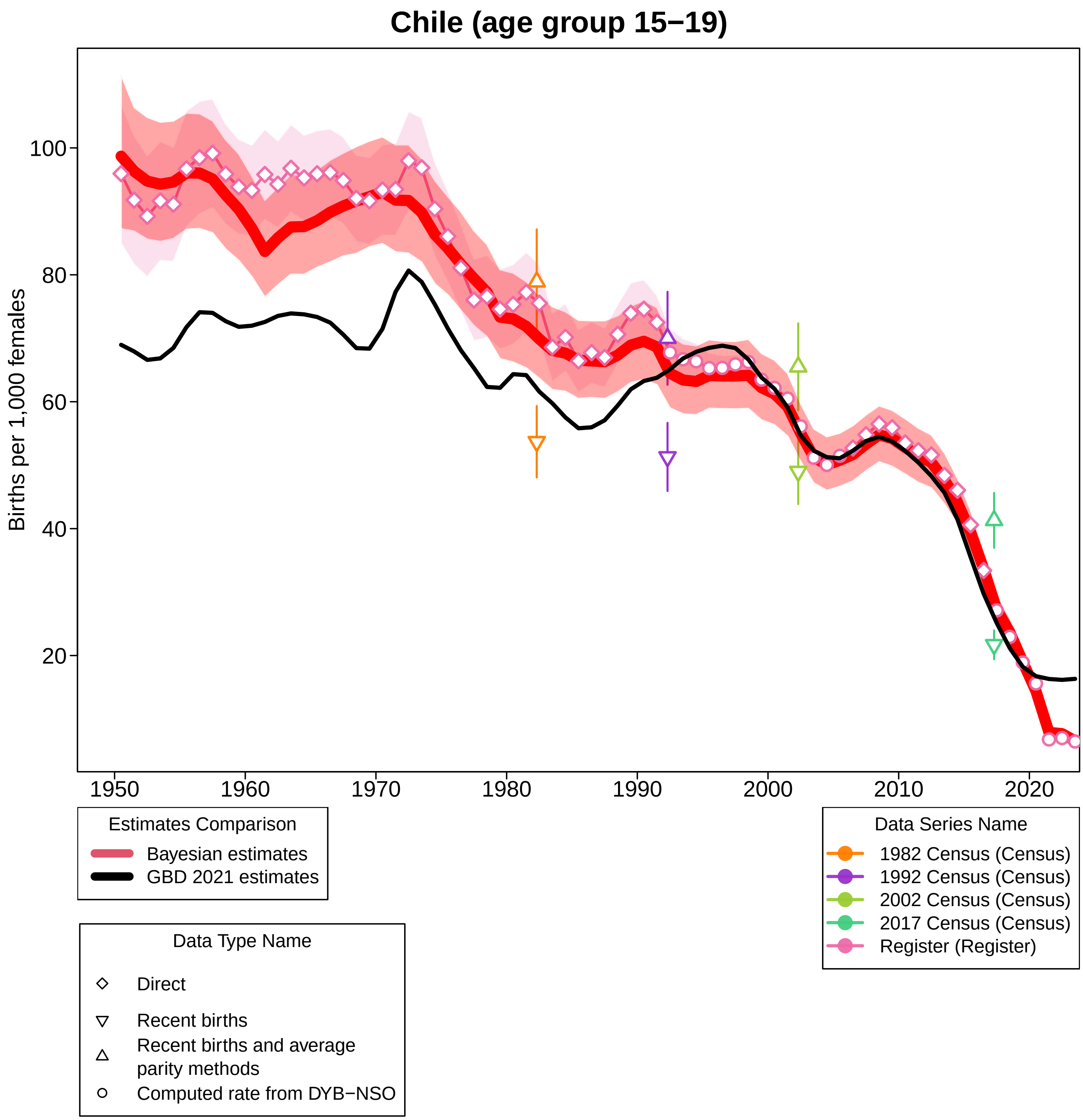

Chile (age group 15−19)
Births per 1,000 females
100
80
60
40
20
1950
1960
1970
1980
1990
2000
2010
2020
Estimates Comparison
Bayesian estimates
GBD 2021 estimates
Data Series Name
1982 Census (Census)
1992 Census (Census)
2002 Census (Census)
2017 Census (Census)
Register (Register)
Data Type Name
Direct
Recent births
Recent births and average parity methods
Computed rate from DYB−NSO

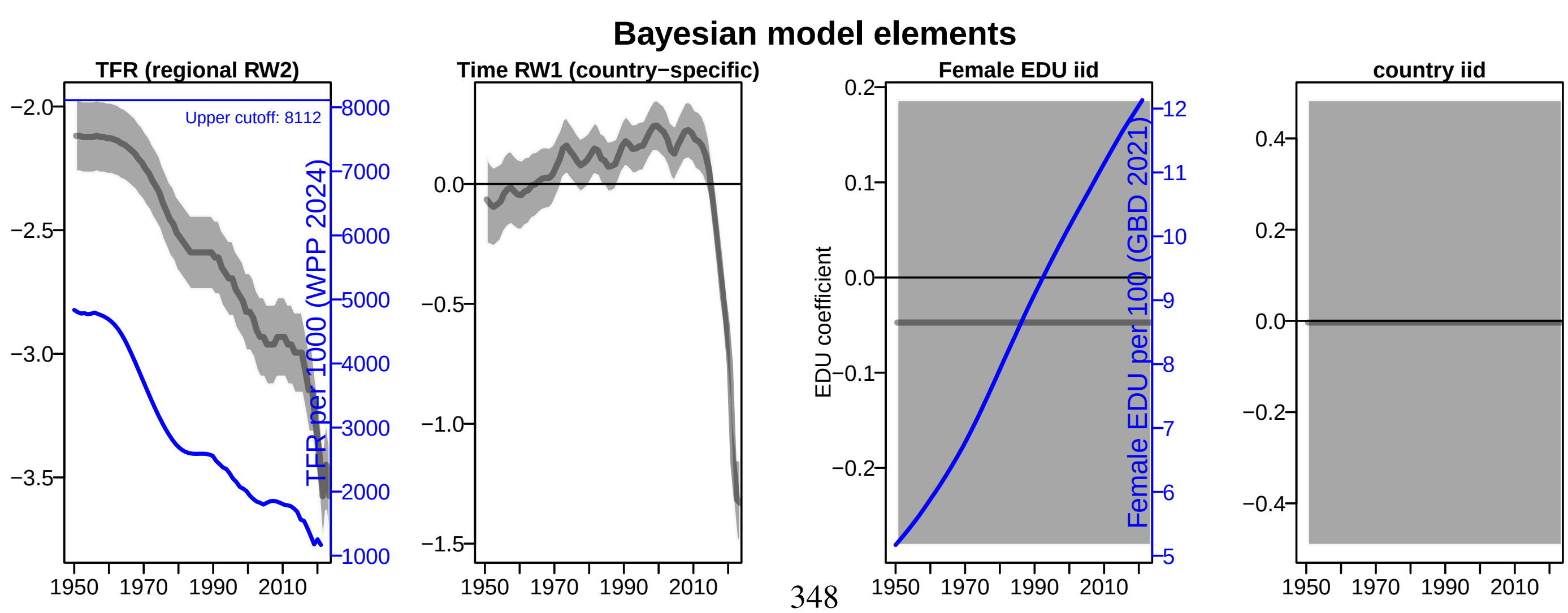

Bayesian model elements
TFR (regional RW2)
Upper cutoff: 8112
TFR per 1000 (WPP 2024)
Time RW1 (country−specific)
Female EDU iid
EDU coefficient
Female EDU per 100 (GBD 2021)
country iid

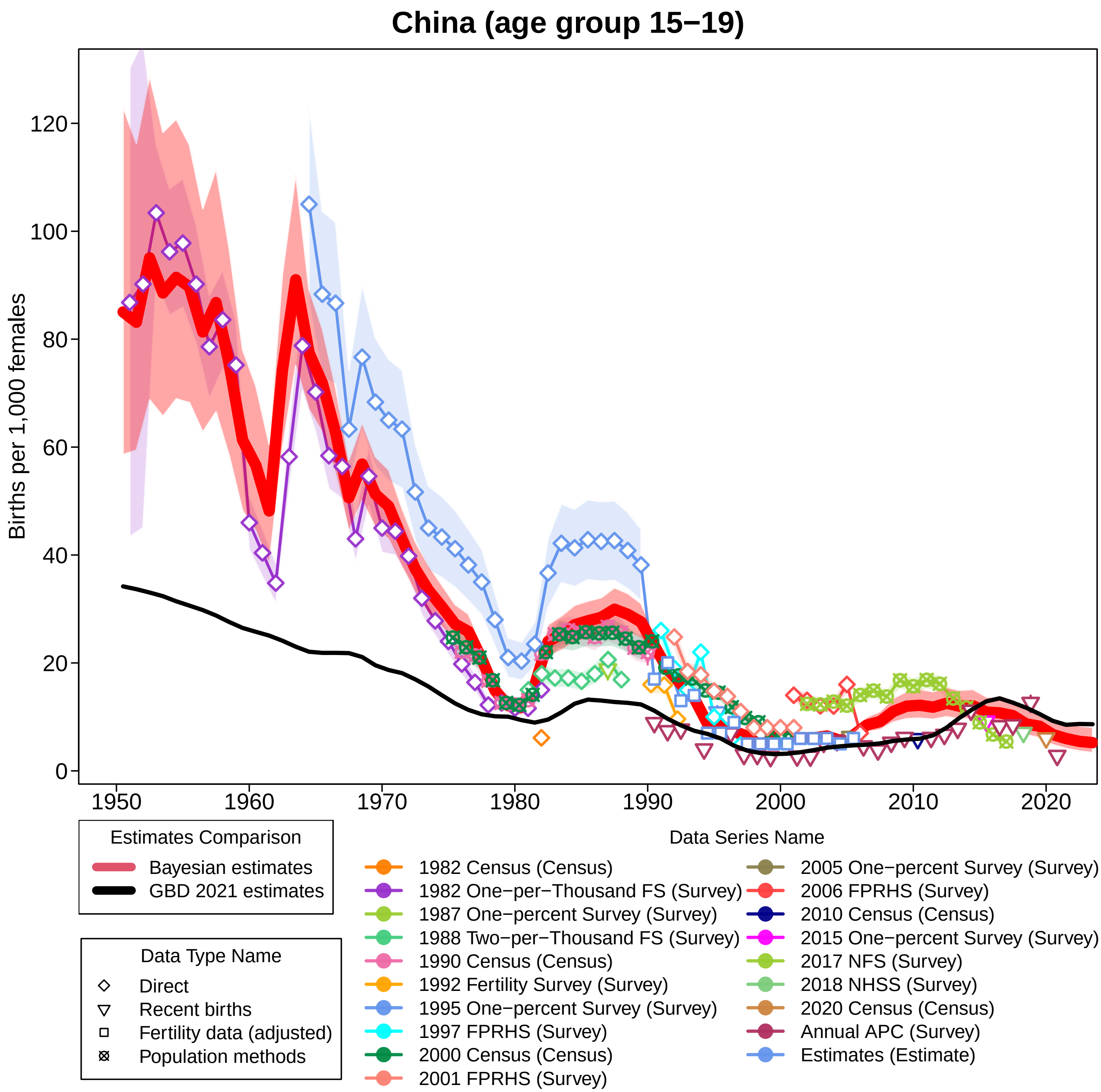
China (age group 15−19)
Births per 1,000 females
0
20
40
60
80
100
120
1950
1960
1970
1980
1990
2000
2010
2020
Estimates Comparison
Bayesian estimates
GBD 2021 estimates
Data Type Name
Direct
Recent births
Fertility data (adjusted)
Population methods
Data Series Name
1982 Census (Census)
1982 One−per−Thousand FS (Survey)
1987 One−percent Survey (Survey)
1988 Two−per−Thousand FS (Survey)
1990 Census (Census)
1992 Fertility Survey (Survey)
1995 One−percent Survey (Survey)
1997 FPRHS (Survey)
2000 Census (Census)
2001 FPRHS (Survey)
2005 One−percent Survey (Survey)
2006 FPRHS (Survey)
2010 Census (Census)
2015 One−percent Survey (Survey)
2017 NFS (Survey)
2018 NHSS (Survey)
2020 Census (Census)
Annual APC (Survey)
Estimates (Estimate)

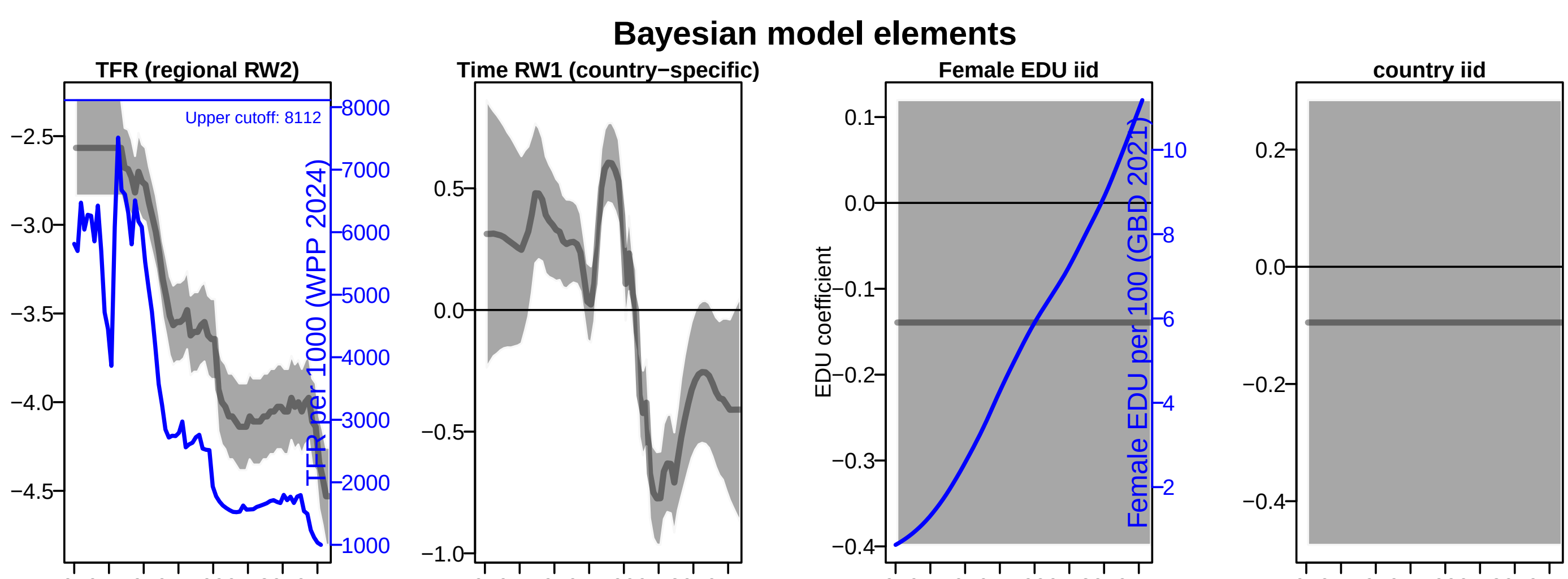
Bayesian model elements
TFR (regional RW2)
Upper cutoff: 8112
TFR per 1000 (WPP 2024)
Time RW1 (country−specific)
Female EDU iid
EDU coefficient
Female EDU per 100 (GBD 2021)
country iid

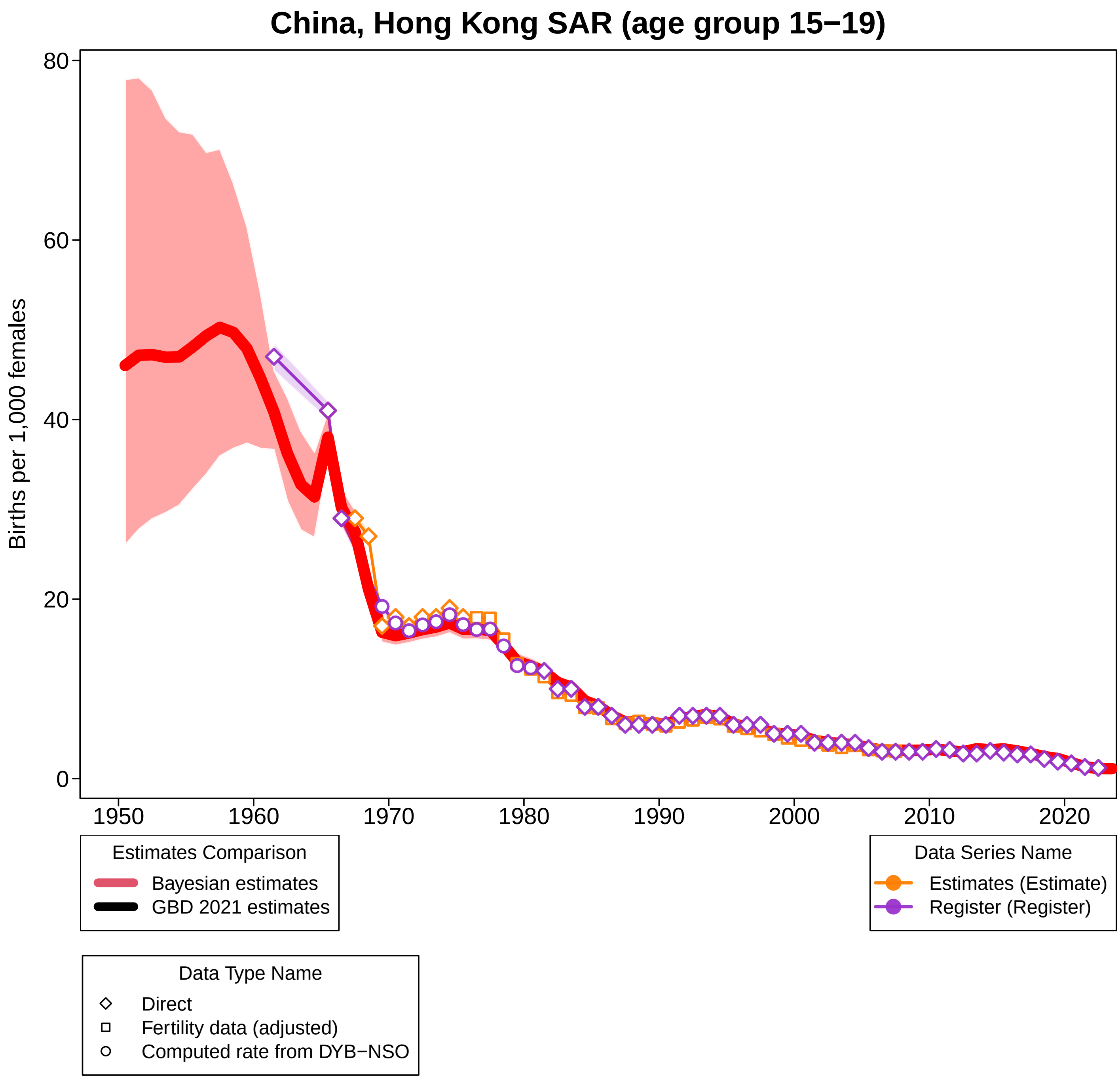
China, Hong Kong SAR (age group 15−19)
Births per 1,000 females
0
20
40
60
80
1950
1960
1970
1980
1990
2000
2010
2020
Estimates Comparison
Bayesian estimates
GBD 2021 estimates
Data Series Name
Estimates (Estimate)
Register (Register)
Data Type Name
Direct
Fertility data (adjusted)
Computed rate from DYB−NSO

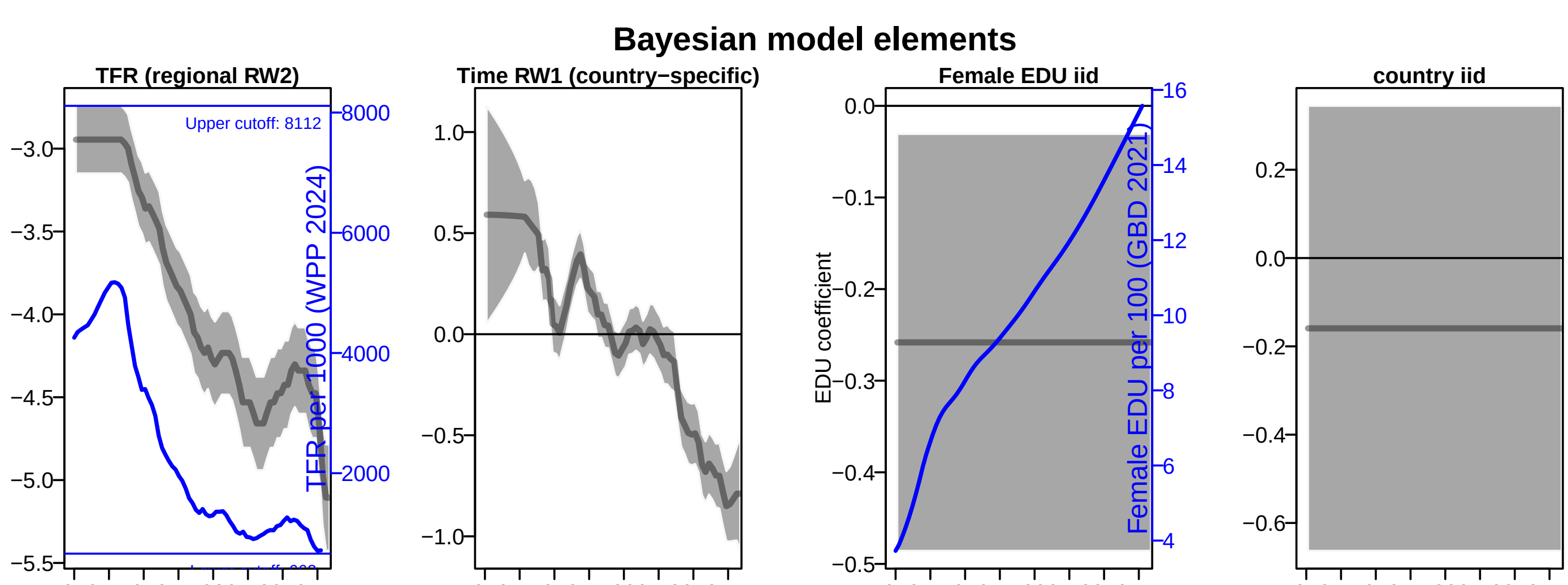
Bayesian model elements
TFR (regional RW2)
Upper cutoff: 8112
TFR per 1000 (WPP 2024)
Time RW1 (country−specific)
Female EDU iid
EDU coefficient
Female EDU per 100 (GBD 2021)
country iid

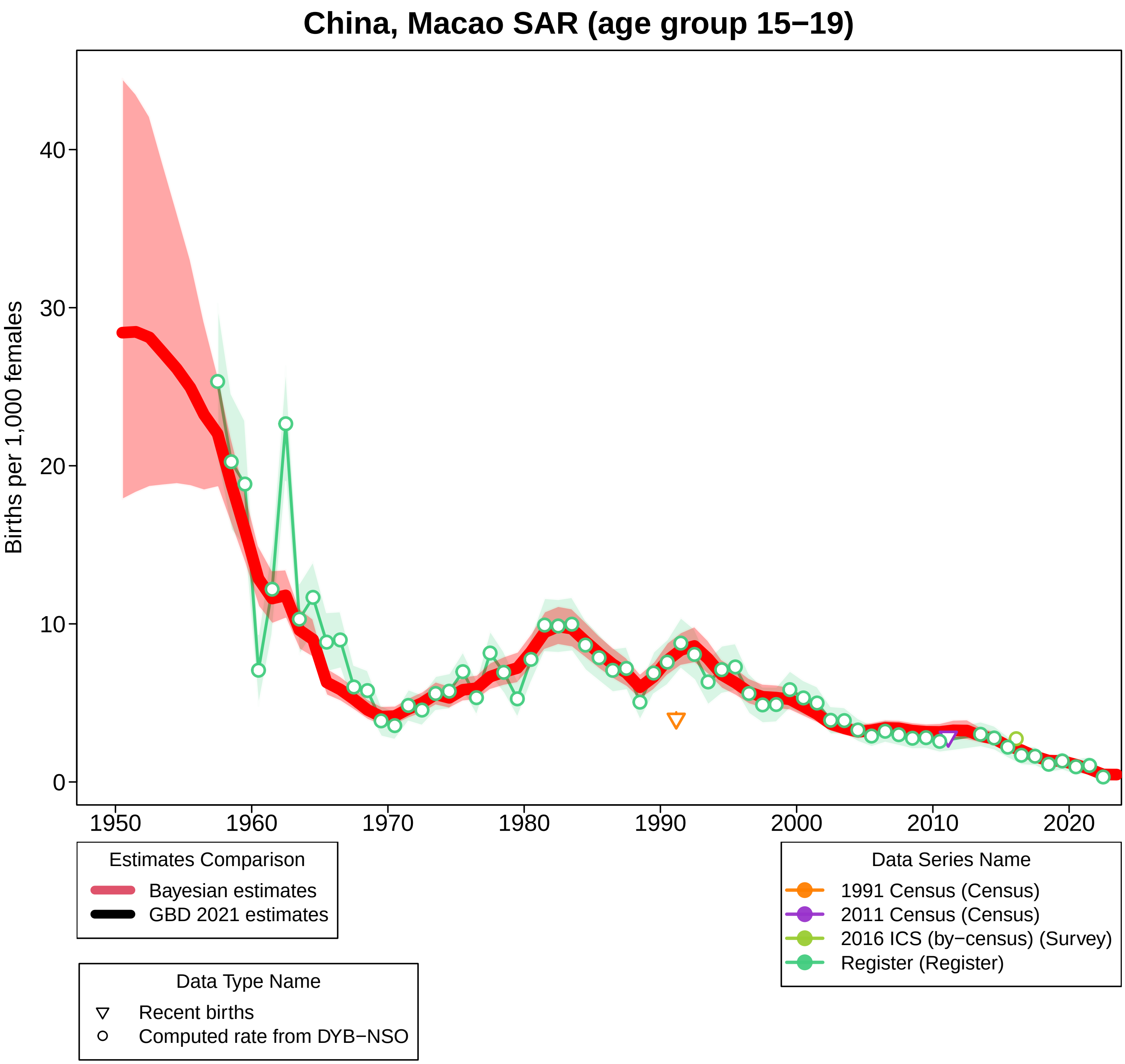
China, Macao SAR (age group 15−19)
Births per 1,000 females
Estimates Comparison
Bayesian estimates
GBD 2021 estimates
Data Series Name
1991 Census (Census)
2011 Census (Census)
2016 ICS (by−census) (Survey)
Register (Register)
Data Type Name
Recent births
Computed rate from DYB−NSO

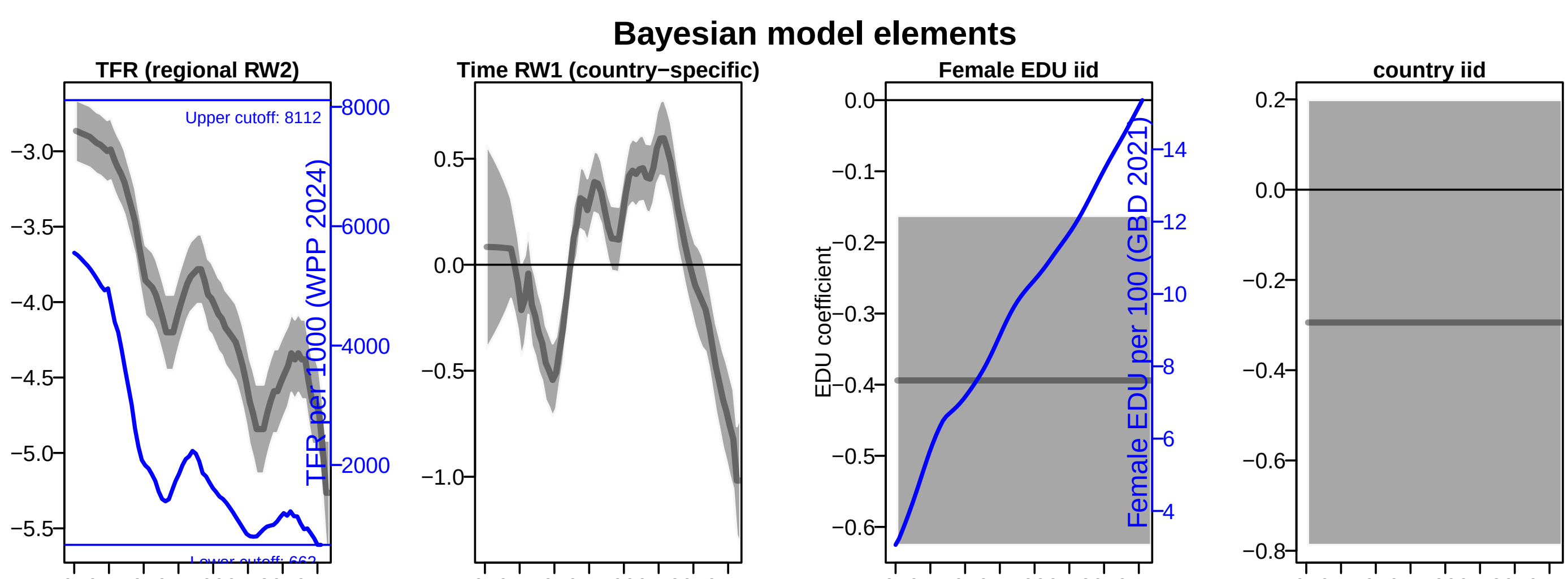
Bayesian model elements
TFR (regional RW2)
Upper cutoff: 8112
TFR per 1000 (WPP 2024)
Time RW1 (country−specific)
Female EDU iid
EDU coefficient
Female EDU per 100 (GBD 2021)
country iid

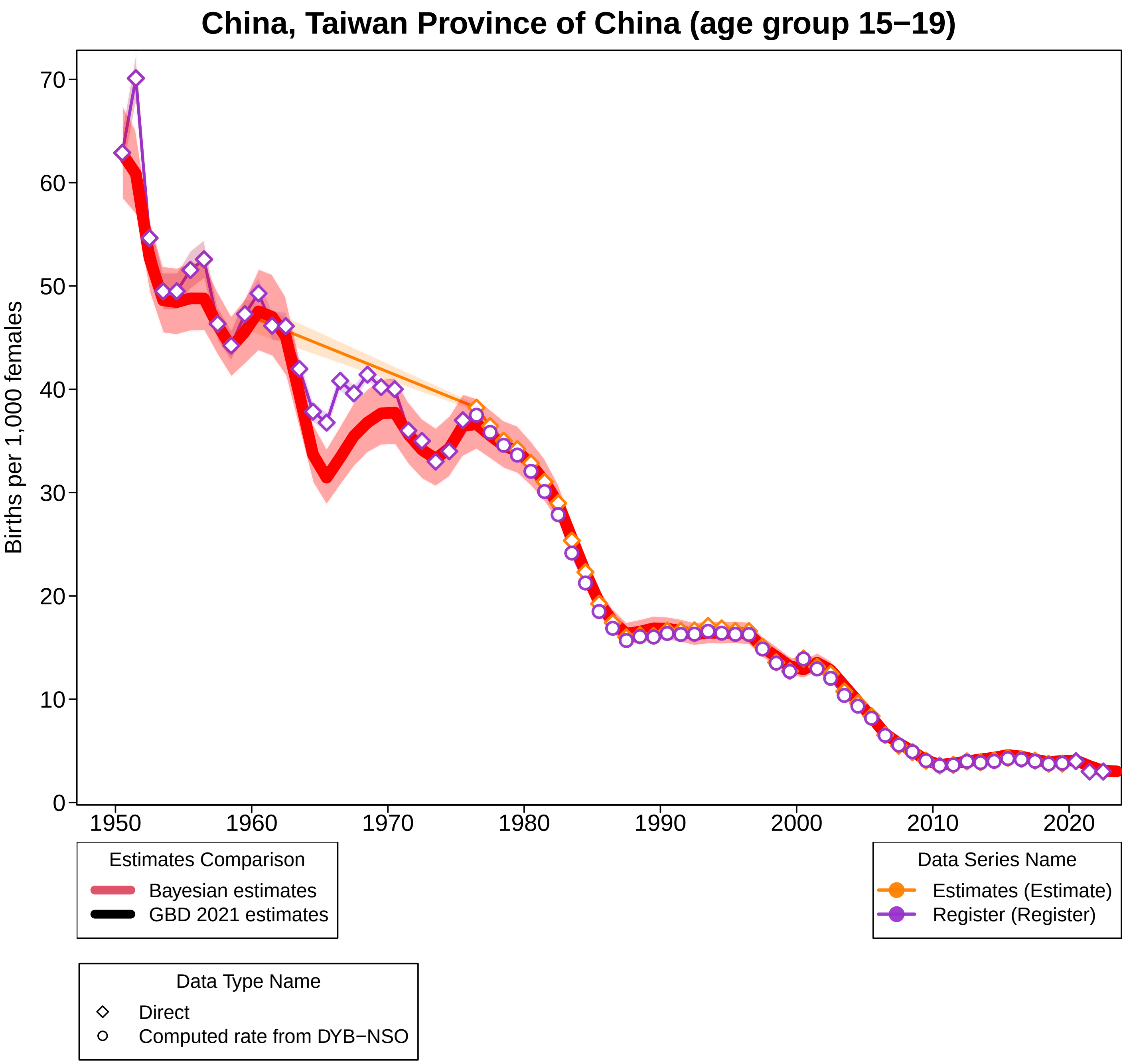
China, Taiwan Province of China (age group 15−19)
Births per 1,000 females
0
10
20
30
40
50
60
70
1950
1960
1970
1980
1990
2000
2010
2020
Estimates Comparison
Bayesian estimates
GBD 2021 estimates
Data Series Name
Estimates (Estimate)
Register (Register)
Data Type Name
Direct
Computed rate from DYB−NSO

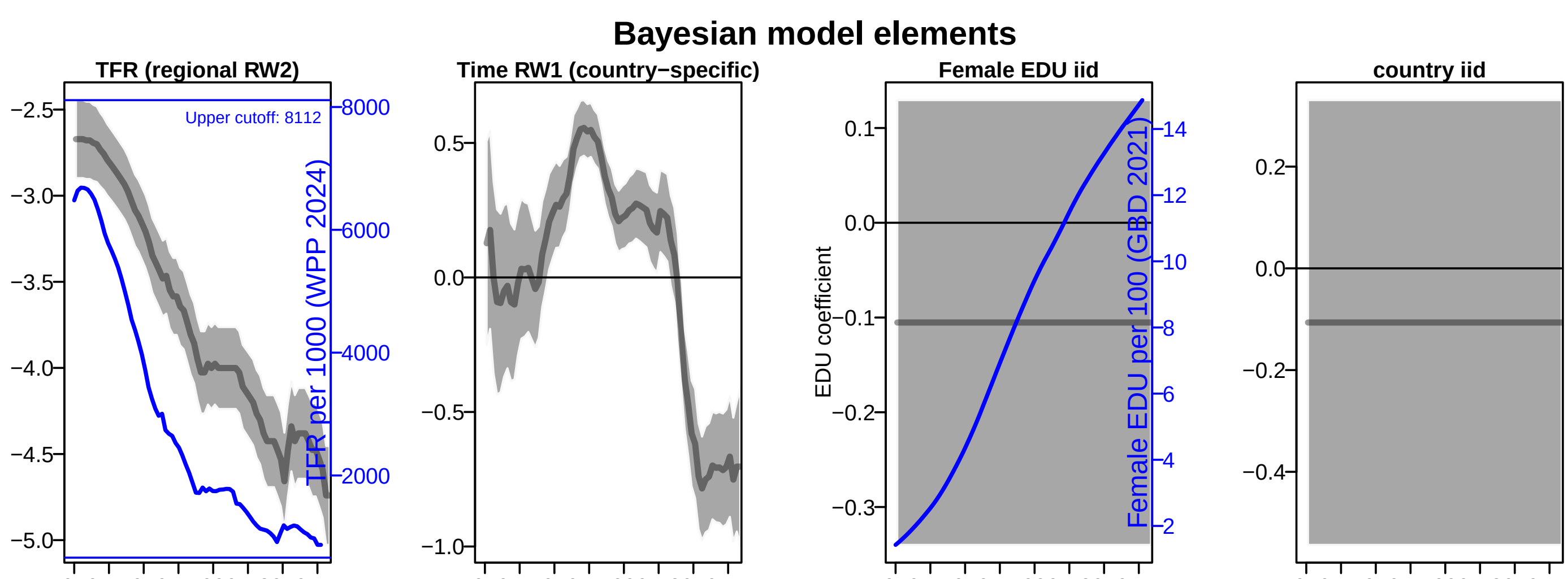
Bayesian model elements
TFR (regional RW2)
Upper cutoff: 8112
TFR per 1000 (WPP 2024)
Time RW1 (country−specific)
Female EDU iid
EDU coefficient
Female EDU per 100 (GBD 2021)
country iid
1950
1970
1990
2010

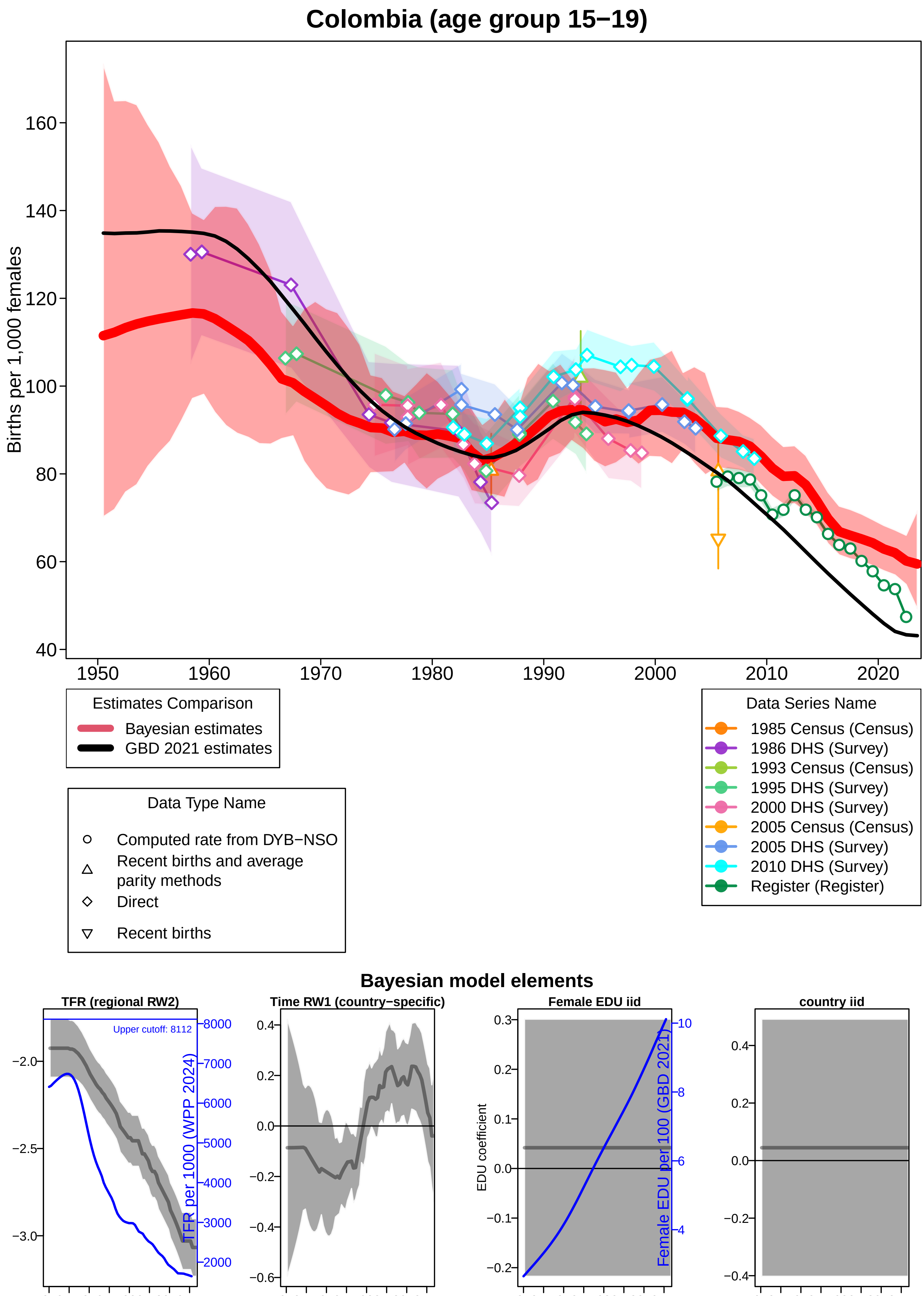
Colombia (age group 15−19)
Births per 1,000 females
160
140
120
100
80
60
40
1950
1960
1970
1980
1990
2000
2010
2020
Estimates Comparison
Bayesian estimates
GBD 2021 estimates
Data Series Name
1985 Census (Census)
1986 DHS (Survey)
1993 Census (Census)
1995 DHS (Survey)
2000 DHS (Survey)
2005 Census (Census)
2005 DHS (Survey)
2010 DHS (Survey)
Register (Register)
Data Type Name
Computed rate from DYB−NSO
Recent births and average parity methods
Direct
Recent births
Bayesian model elements
TFR (regional RW2)
Upper cutoff: 8112
TFR per 1000 (WPP 2024)
Time RW1 (country−specific)
Female EDU iid
EDU coefficient
Female EDU per 100 (GBD 2021)
country iid

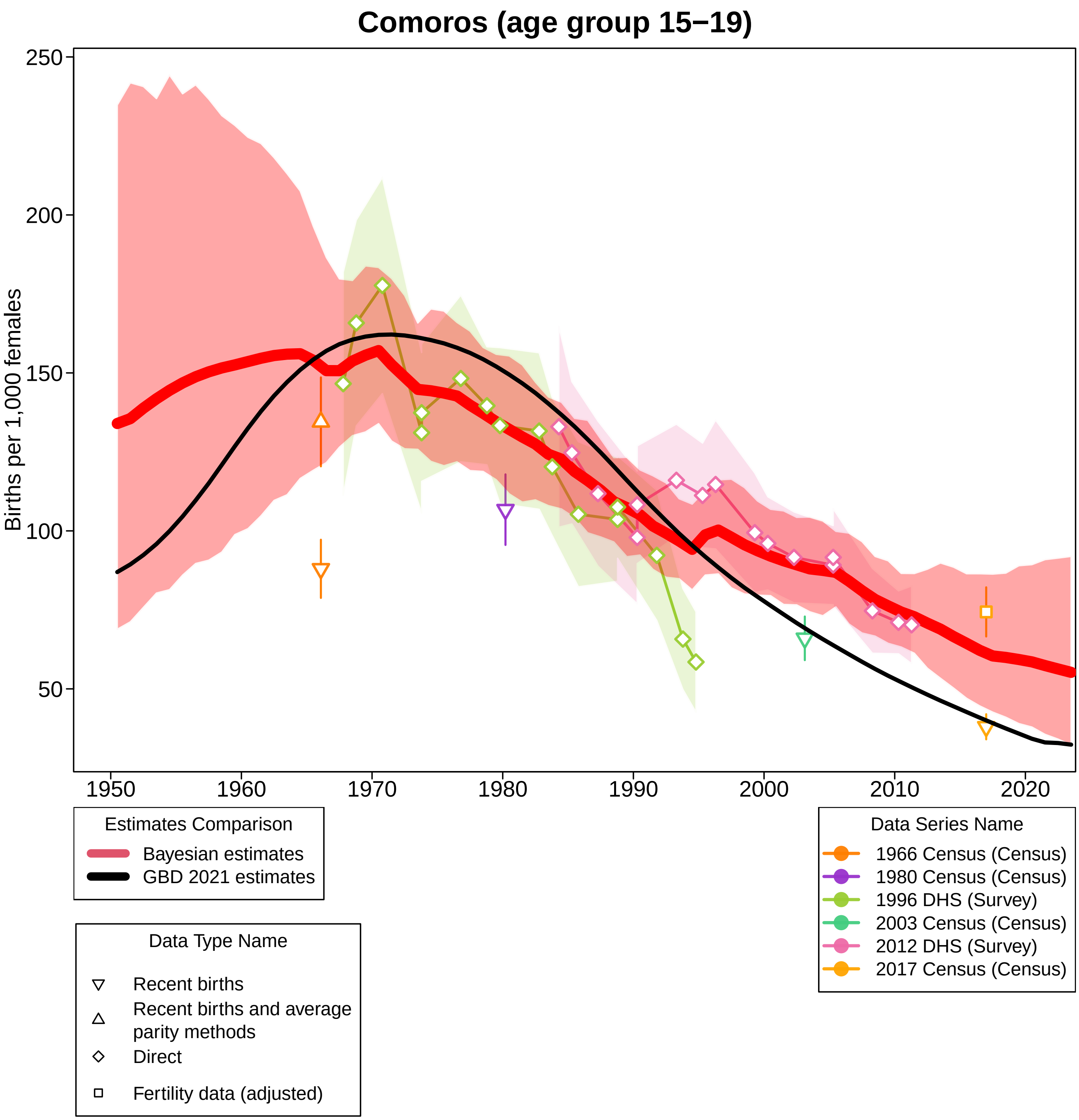
Comoros (age group 15−19)
Births per 1,000 females
250
200
150
100
50
1950
1960
1970
1980
1990
2000
2010
2020
Estimates Comparison
Bayesian estimates
GBD 2021 estimates
Data Series Name
1966 Census (Census)
1980 Census (Census)
1996 DHS (Survey)
2003 Census (Census)
2012 DHS (Survey)
2017 Census (Census)
Data Type Name
Recent births
Recent births and average parity methods
Direct
Fertility data (adjusted)

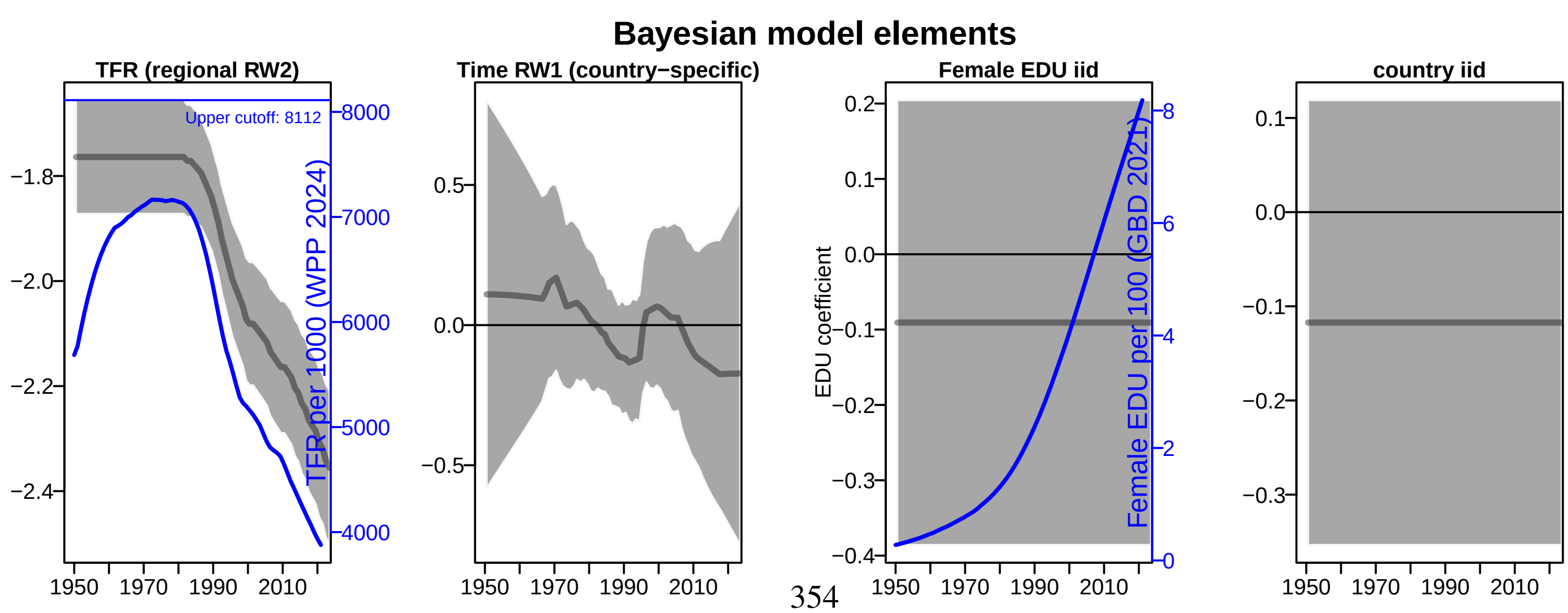
Bayesian model elements
TFR (regional RW2)
Upper cutoff: 8112
TFR per 1000 (WPP 2024)
−1.8
−2.0
−2.2
−2.4
8000
7000
6000
5000
4000
Time RW1 (country−specific)
0.5
0.0
−0.5
Female EDU iid
EDU coefficient
Female EDU per 100 (GBD 2021)
0.2
0.1
0.0
−0.1
−0.2
−0.3
−0.4
8
6
4
2
0
country iid
0.1
0.0
−0.1
−0.2
−0.3
1950
1970
1990
2010

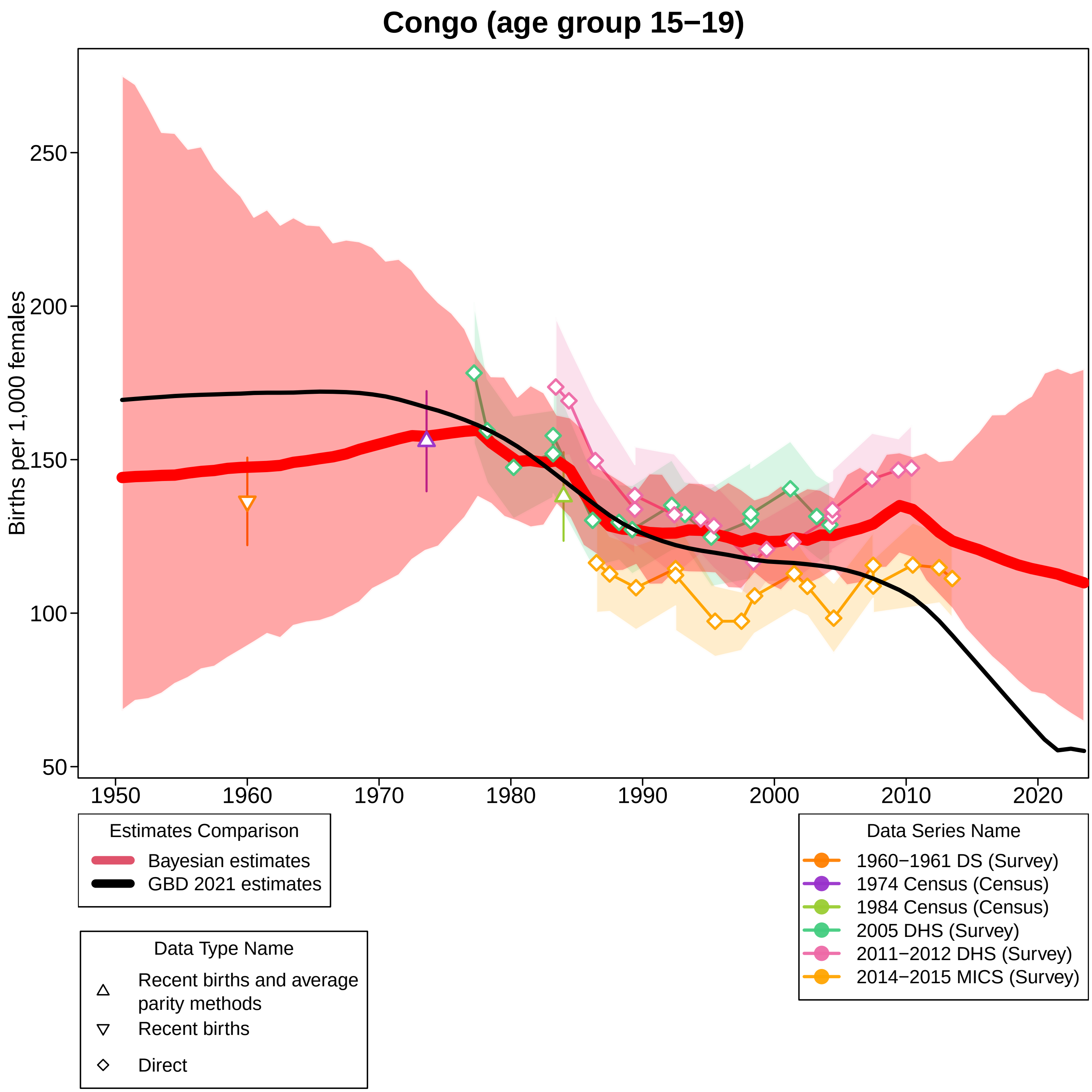
Congo (age group 15−19)
Births per 1,000 females
250
200
150
100
50
1950
1960
1970
1980
1990
2000
2010
2020
Estimates Comparison
Bayesian estimates
GBD 2021 estimates
Data Type Name
Recent births and average parity methods
Recent births
Direct
Data Series Name
1960−1961 DS (Survey)
1974 Census (Census)
1984 Census (Census)
2005 DHS (Survey)
2011−2012 DHS (Survey)
2014−2015 MICS (Survey)

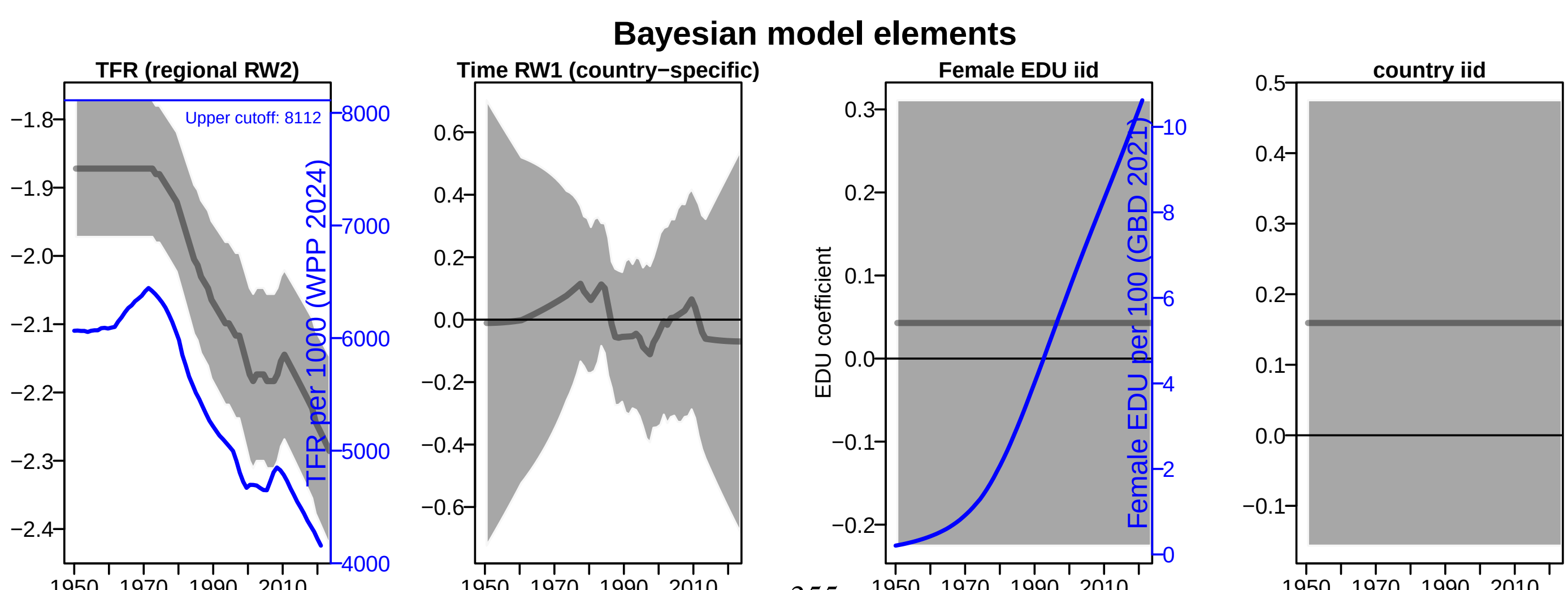
Bayesian model elements
TFR (regional RW2)
Upper cutoff: 8112
TFR per 1000 (WPP 2024)
Time RW1 (country−specific)
Female EDU iid
EDU coefficient
Female EDU per 100 (GBD 2021)
country iid

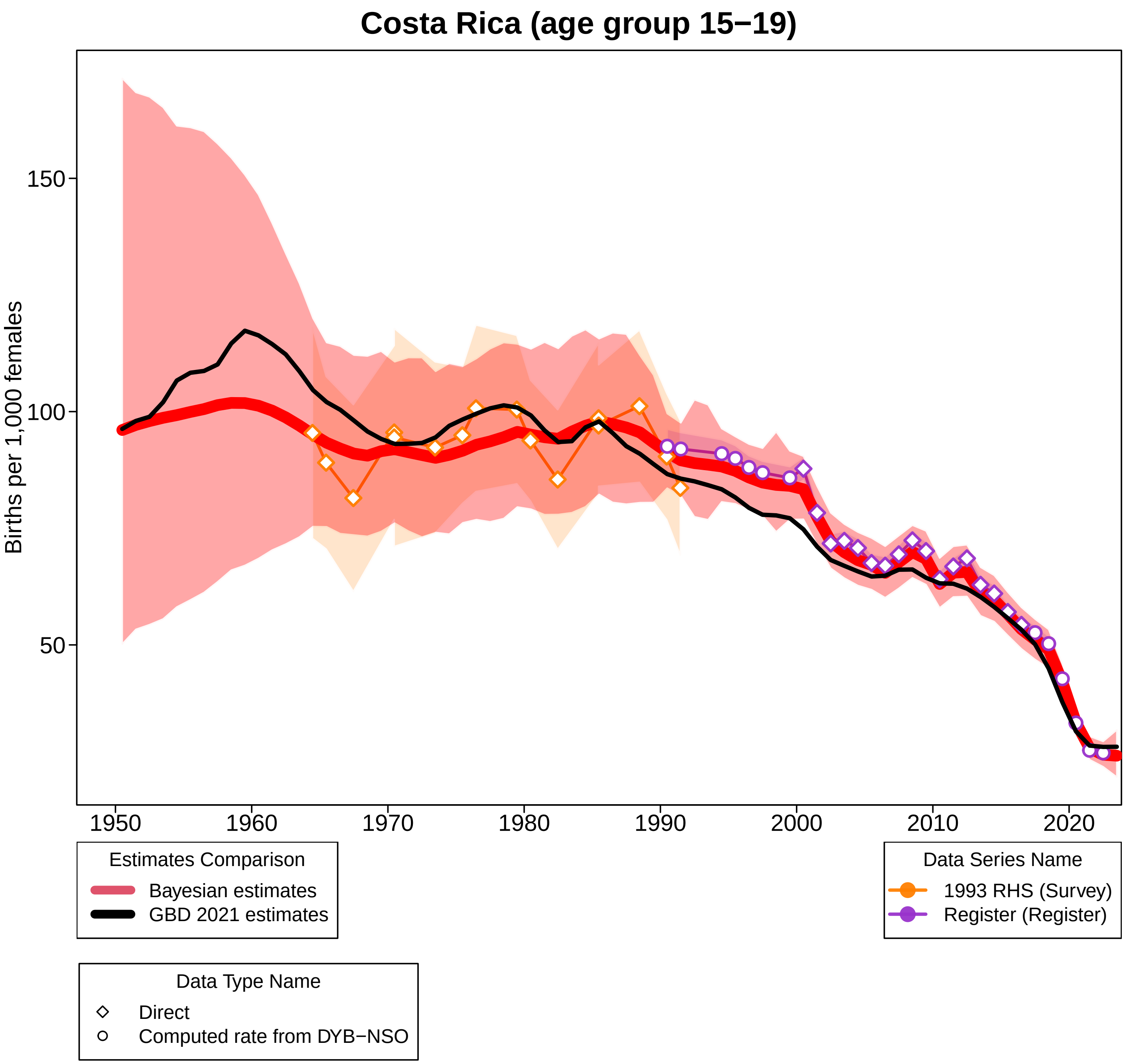

Costa Rica (age group 15−19)
Births per 1,000 females
150
100
50
1950
1960
1970
1980
1990
2000
2010
2020
Estimates Comparison
Bayesian estimates
GBD 2021 estimates
Data Series Name
1993 RHS (Survey)
Register (Register)
Data Type Name
Direct
Computed rate from DYB−NSO

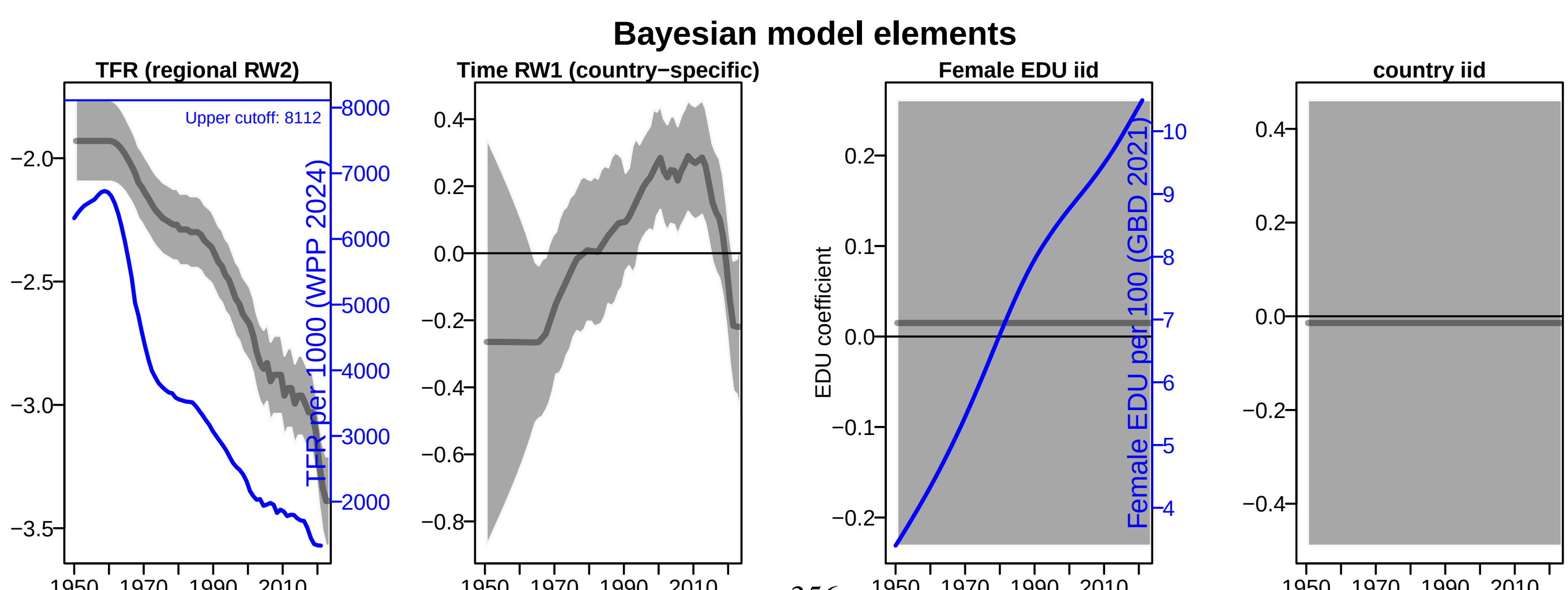

Bayesian model elements
TFR (regional RW2)
Upper cutoff: 8112
TFR per 1000 (WPP 2024)
Time RW1 (country−specific)
Female EDU iid
EDU coefficient
Female EDU per 100 (GBD 2021)
country iid

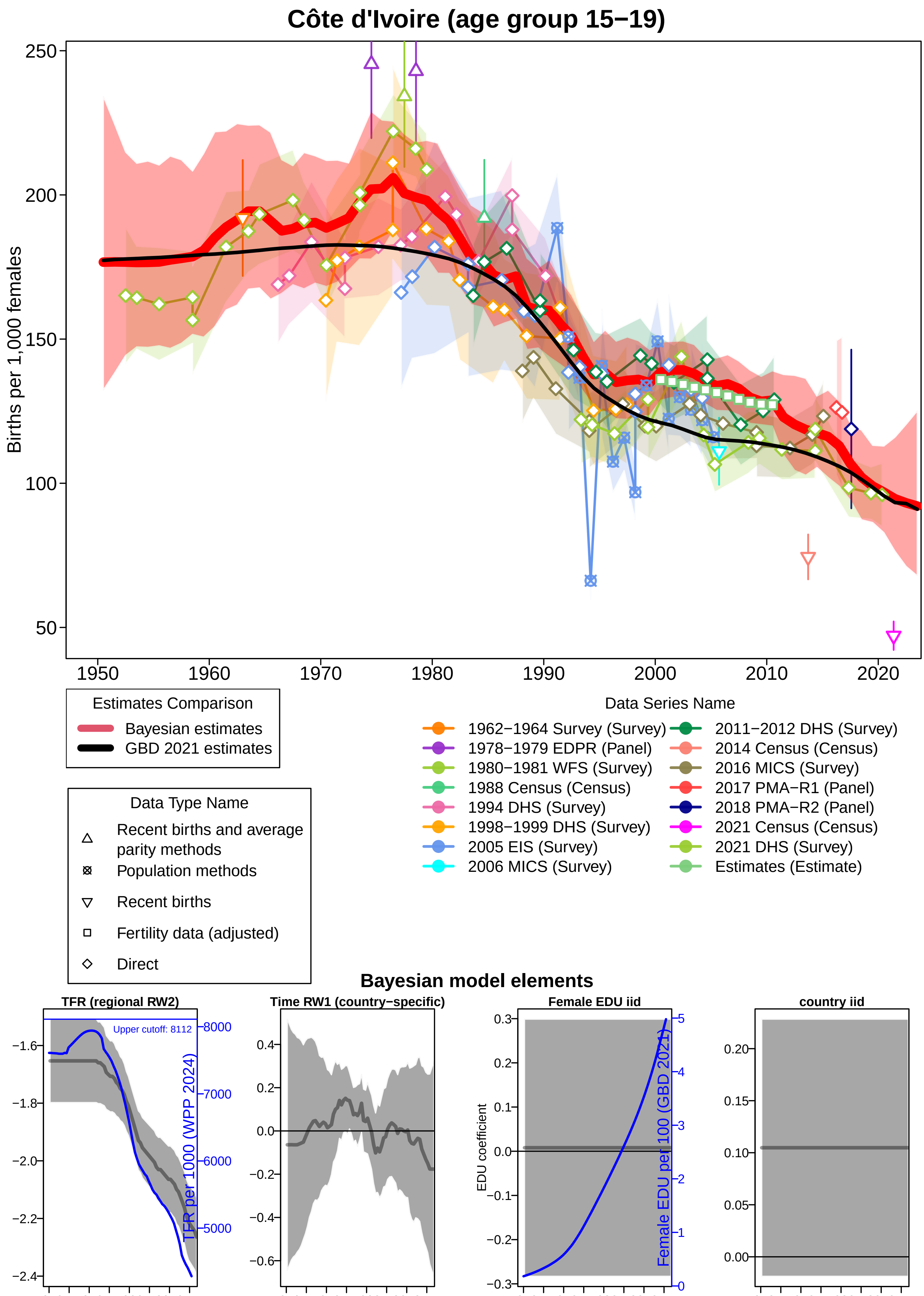

Côte d'Ivoire (age group 15−19)
Births per 1,000 females
Estimates Comparison
Bayesian estimates
GBD 2021 estimates
Data Type Name
Recent births and average parity methods
Population methods
Recent births
Fertility data (adjusted)
Direct
Data Series Name
1962−1964 Survey (Survey)
1978−1979 EDPR (Panel)
1980−1981 WFS (Survey)
1988 Census (Census)
1994 DHS (Survey)
1998−1999 DHS (Survey)
2005 EIS (Survey)
2006 MICS (Survey)
2011−2012 DHS (Survey)
2014 Census (Census)
2016 MICS (Survey)
2017 PMA−R1 (Panel)
2018 PMA−R2 (Panel)
2021 Census (Census)
2021 DHS (Survey)
Estimates (Estimate)
Bayesian model elements
TFR (regional RW2)
Upper cutoff: 8112
TFR per 1000 (WPP 2024)
Time RW1 (country−specific)
Female EDU iid
EDU coefficient
Female EDU per 100 (GBD 2021)
country iid

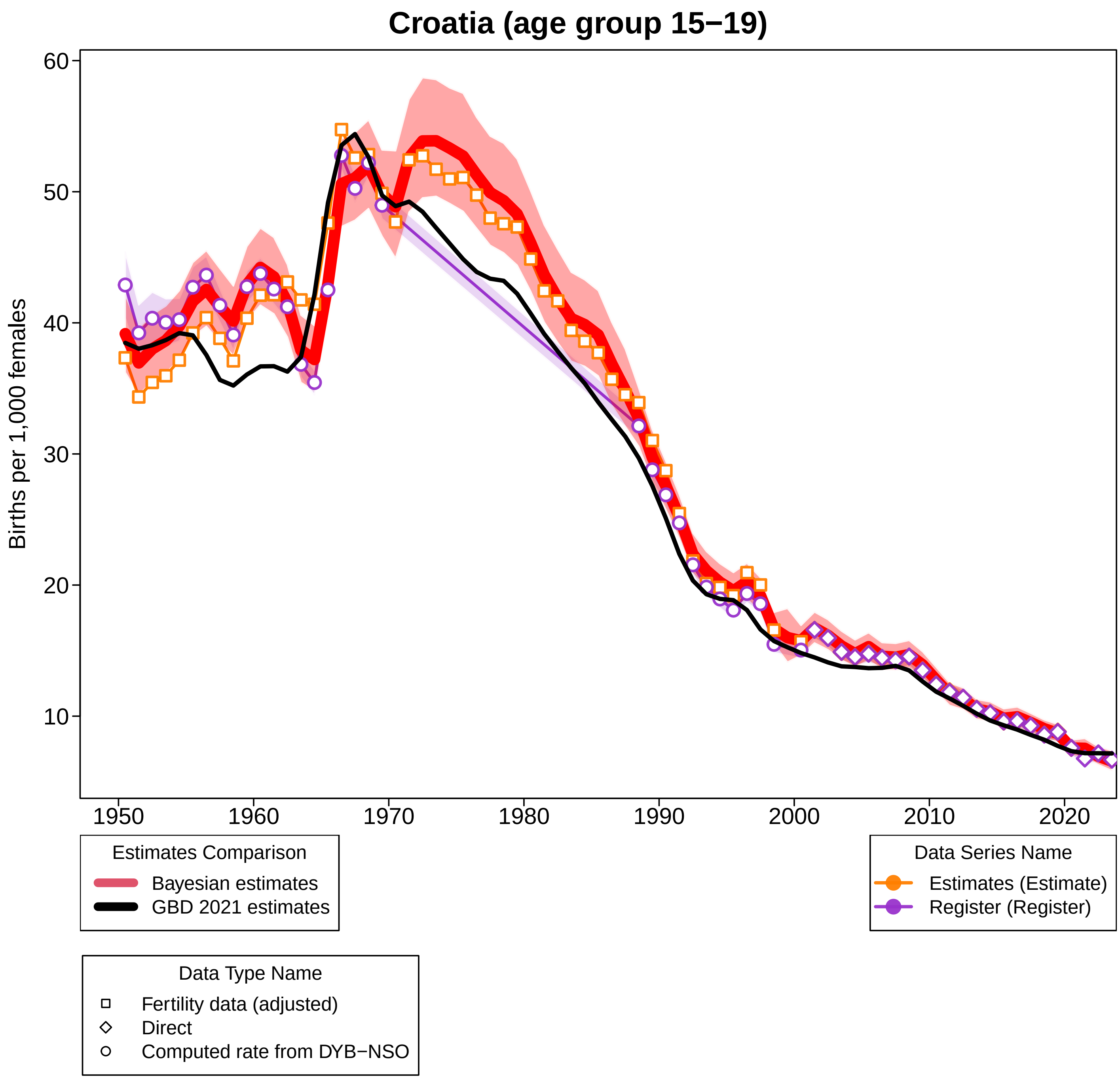

Croatia (age group 15−19)
Births per 1,000 females
60
50
40
30
20
10
1950
1960
1970
1980
1990
2000
2010
2020
Estimates Comparison
Bayesian estimates
GBD 2021 estimates
Data Series Name
Estimates (Estimate)
Register (Register)
Data Type Name
Fertility data (adjusted)
Direct
Computed rate from DYB−NSO

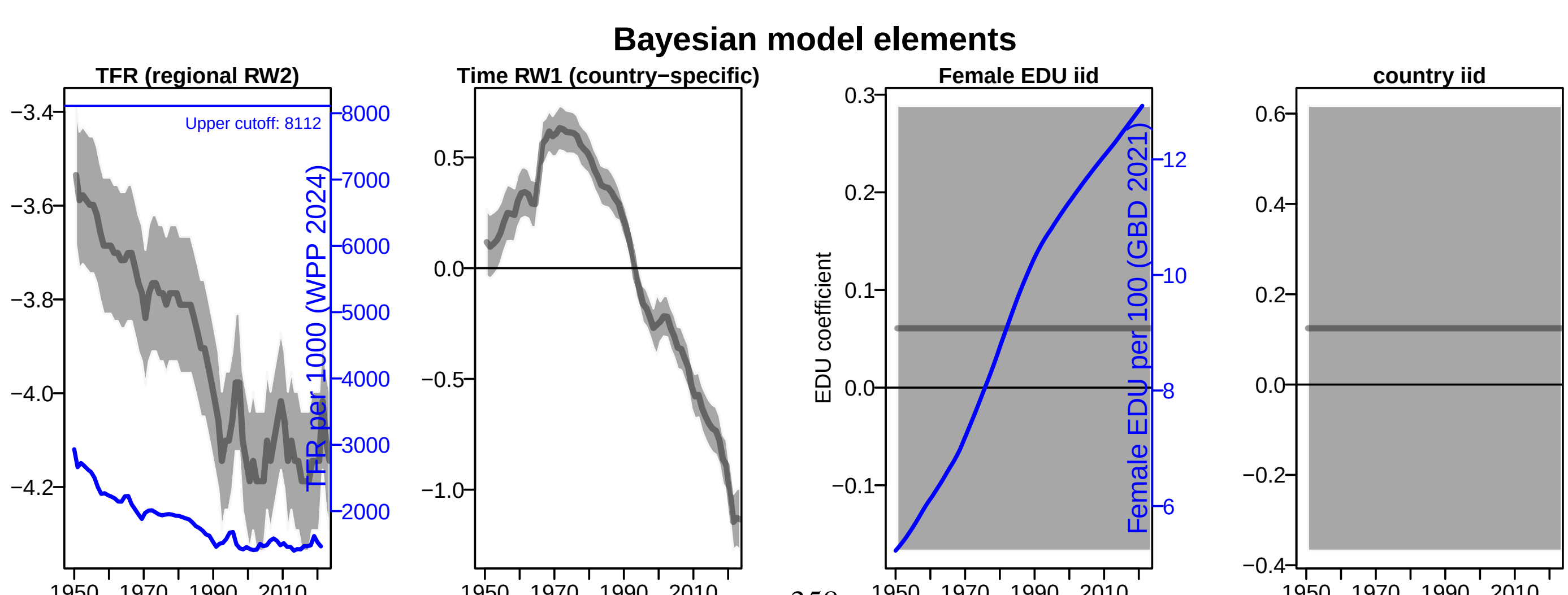

Bayesian model elements
TFR (regional RW2)
Upper cutoff: 8112
TFR per 1000 (WPP 2024)
Time RW1 (country−specific)
Female EDU iid
EDU coefficient
Female EDU per 100 (GBD 2021)
country iid

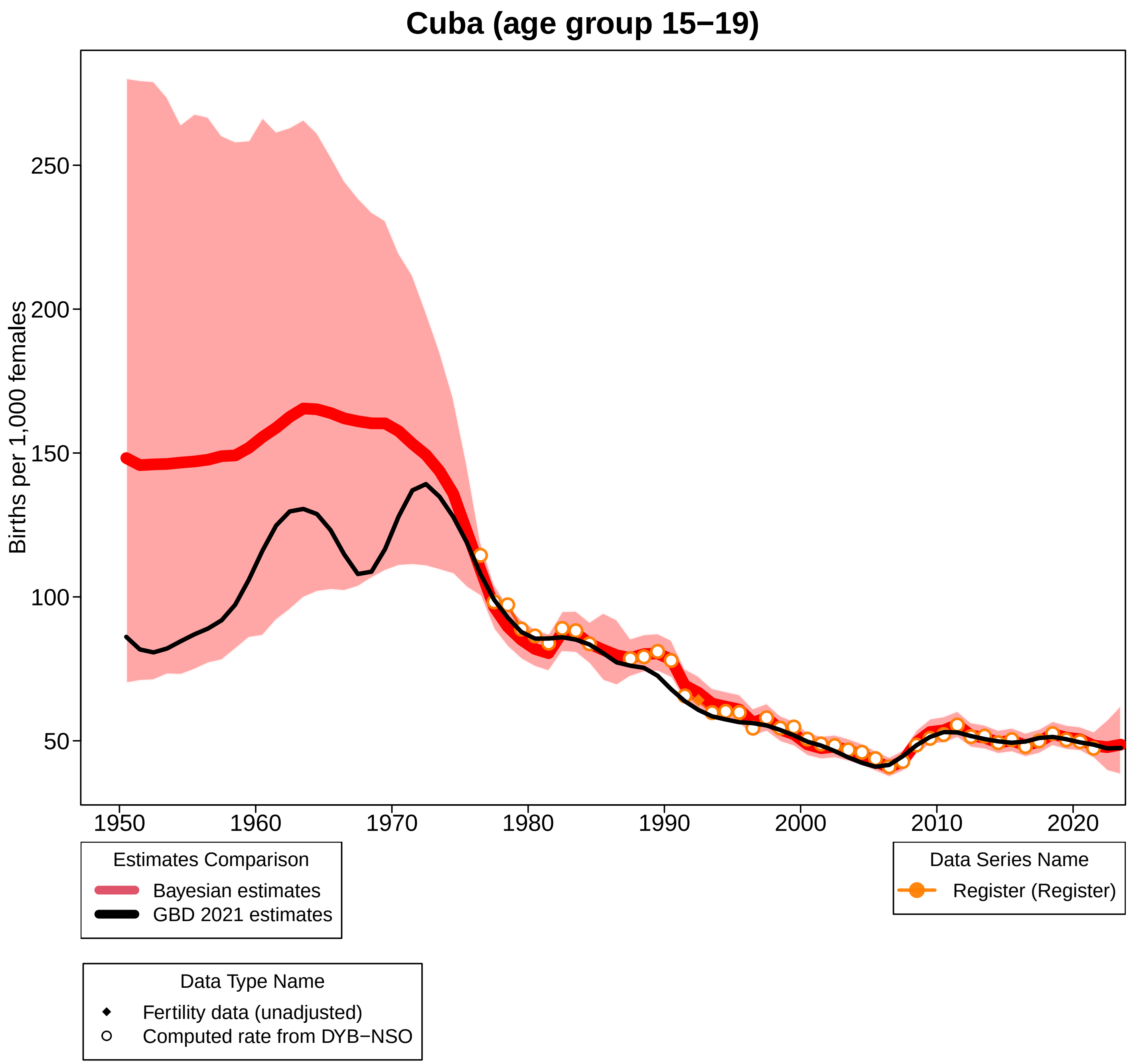

Cuba (age group 15−19)
Births per 1,000 females
250
200
150
100
50
1950
1960
1970
1980
1990
2000
2010
2020
Estimates Comparison
Bayesian estimates
GBD 2021 estimates
Data Series Name
Register (Register)
Data Type Name
Fertility data (unadjusted)
Computed rate from DYB−NSO

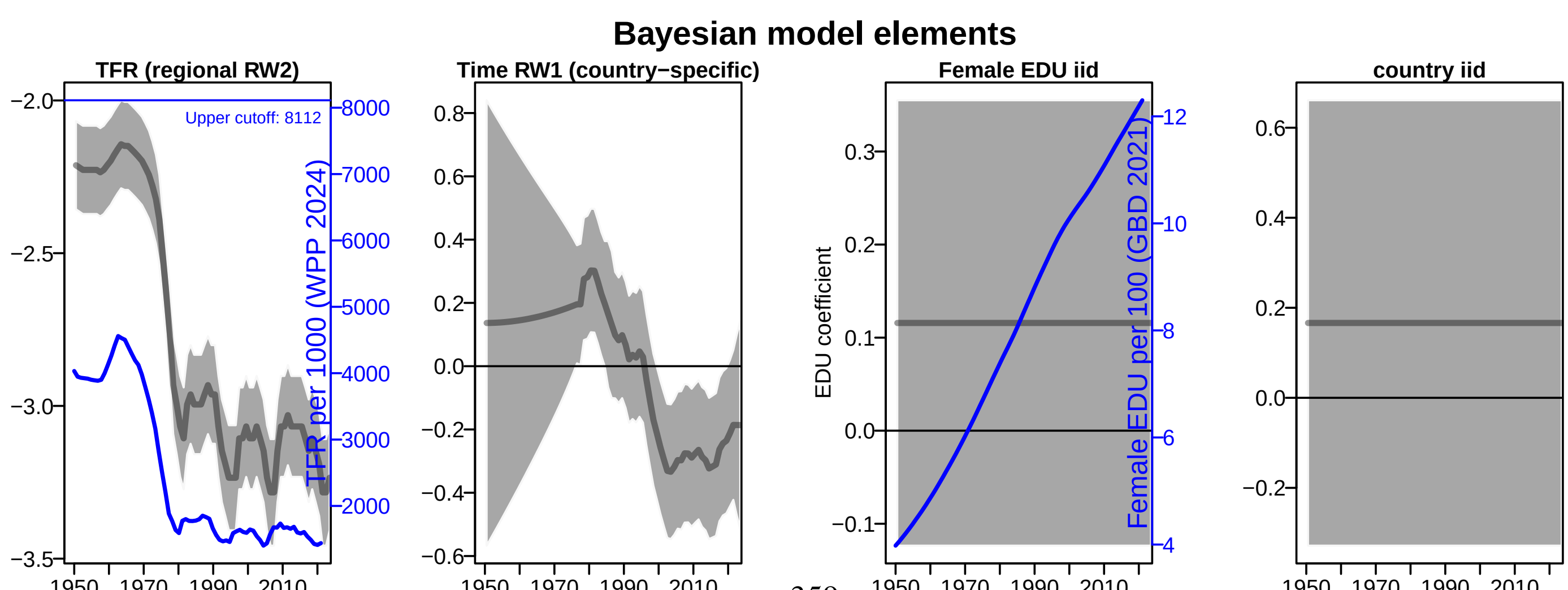

Bayesian model elements
TFR (regional RW2)
Upper cutoff: 8112
TFR per 1000 (WPP 2024)
Time RW1 (country−specific)
Female EDU iid
EDU coefficient
Female EDU per 100 (GBD 2021)
country iid

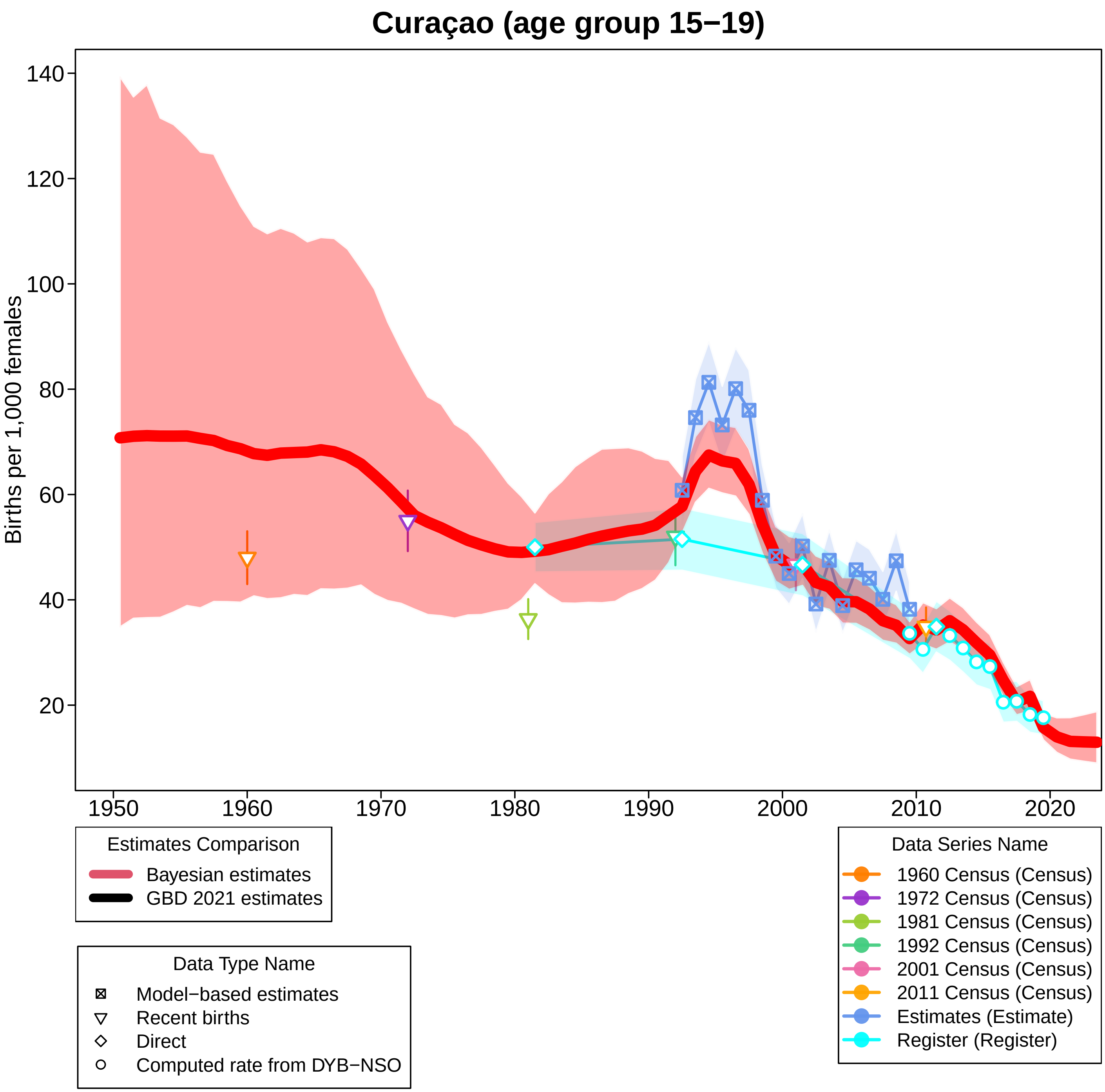

Curaçao (age group 15−19)
Births per 1,000 females
140
120
100
80
60
40
20
1950
1960
1970
1980
1990
2000
2010
2020
Estimates Comparison
Bayesian estimates
GBD 2021 estimates
Data Type Name
Model−based estimates
Recent births
Direct
Computed rate from DYB−NSO
Data Series Name
1960 Census (Census)
1972 Census (Census)
1981 Census (Census)
1992 Census (Census)
2001 Census (Census)
2011 Census (Census)
Estimates (Estimate)
Register (Register)

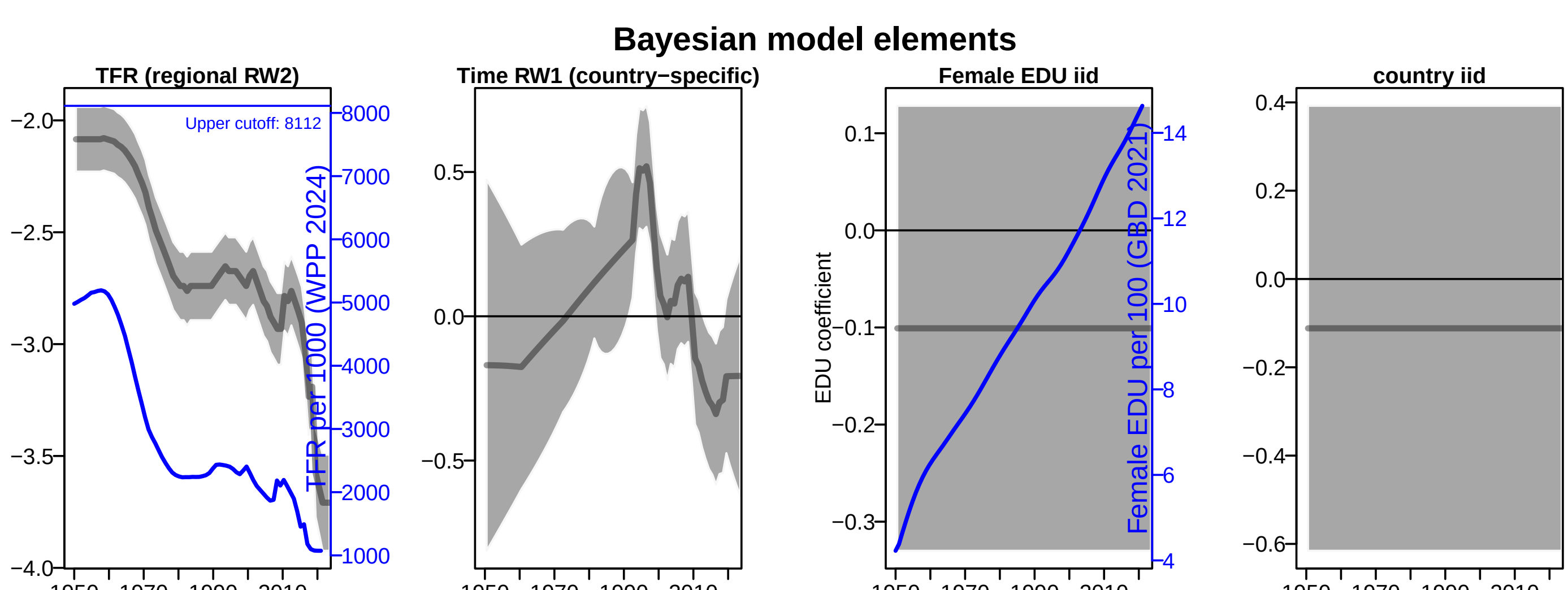

Bayesian model elements
TFR (regional RW2)
Upper cutoff: 8112
TFR per 1000 (WPP 2024)
Time RW1 (country−specific)
Female EDU iid
EDU coefficient
Female EDU per 100 (GBD 2021)
country iid

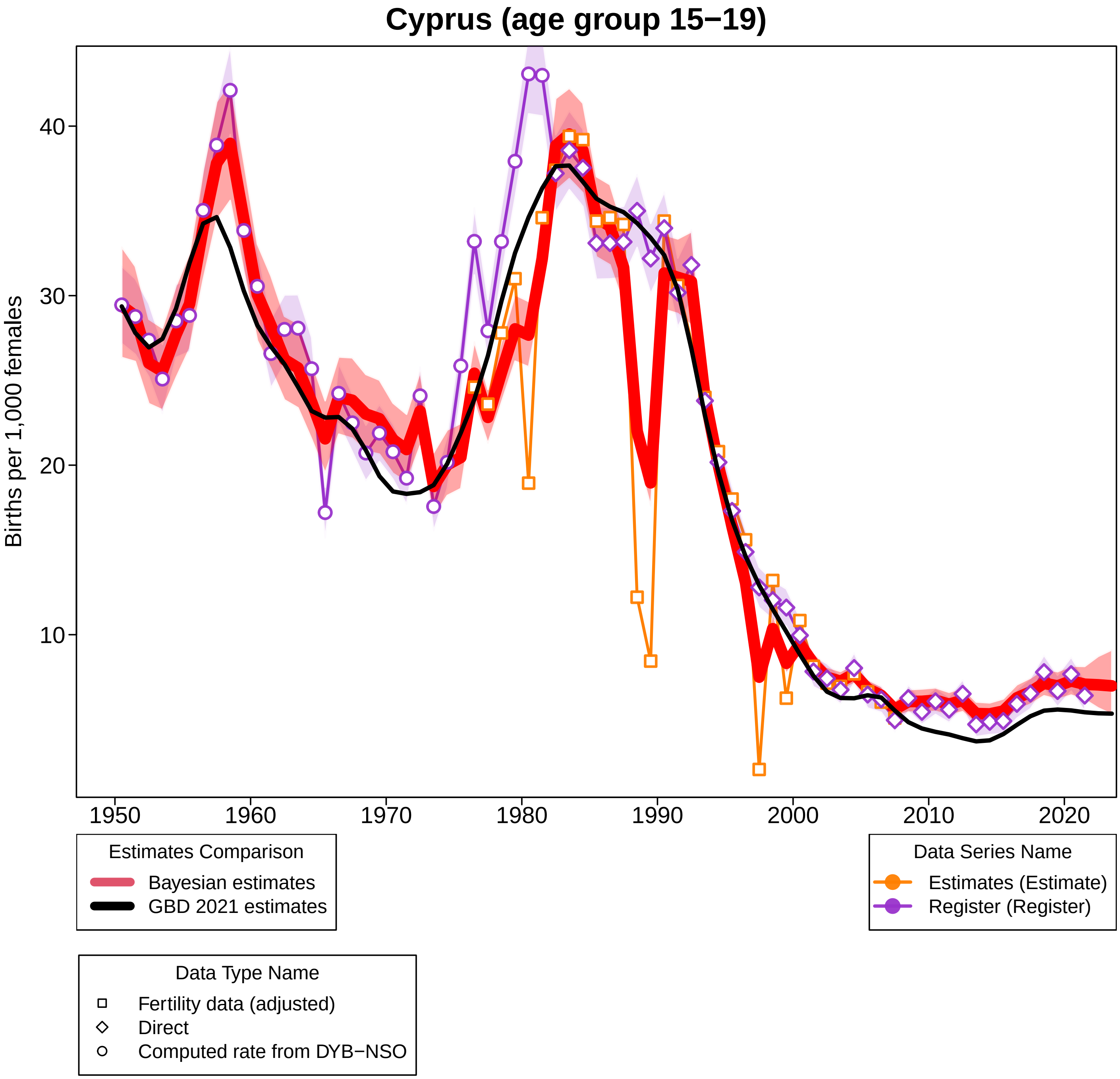
Cyprus (age group 15−19)
Births per 1,000 females
10
20
30
40
1950
1960
1970
1980
1990
2000
2010
2020
Estimates Comparison
Bayesian estimates
GBD 2021 estimates
Data Series Name
Estimates (Estimate)
Register (Register)
Data Type Name
Fertility data (adjusted)
Direct
Computed rate from DYB−NSO

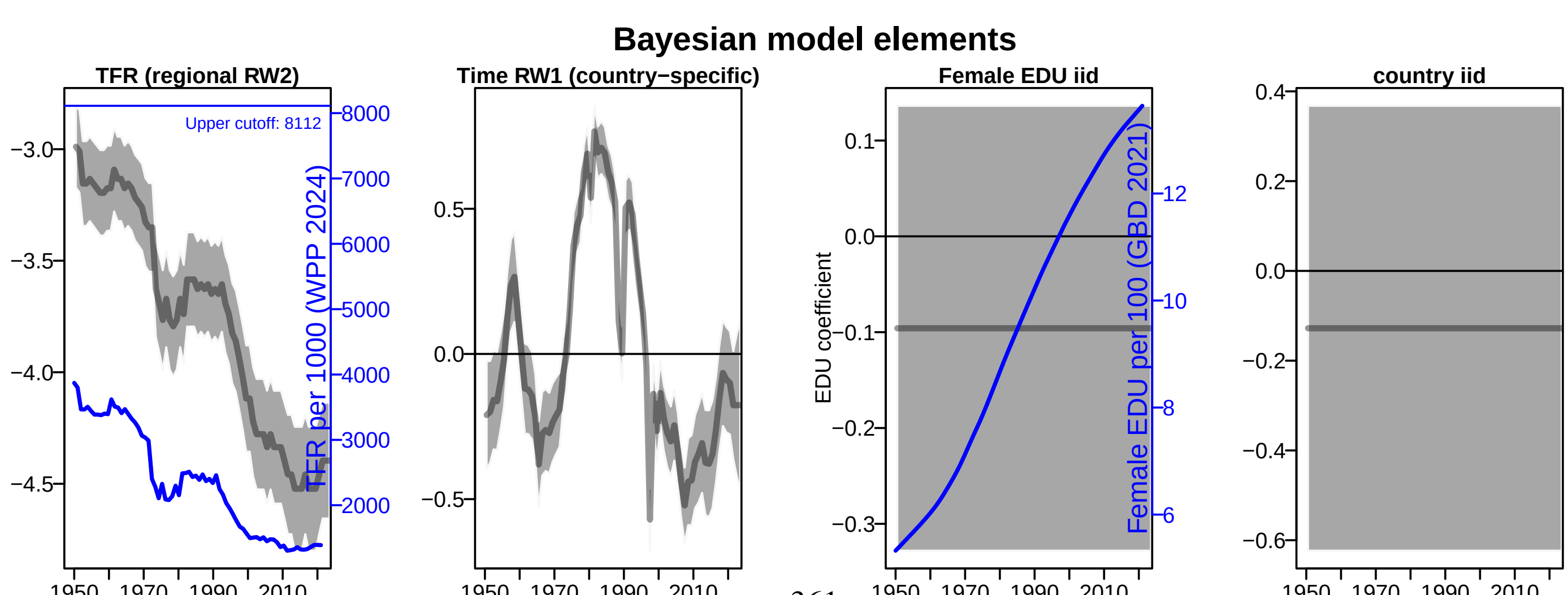
Bayesian model elements
TFR (regional RW2)
Upper cutoff: 8112
TFR per 1000 (WPP 2024)
Time RW1 (country−specific)
Female EDU iid
EDU coefficient
Female EDU per 100 (GBD 2021)
country iid
1950
1970
1990
2010

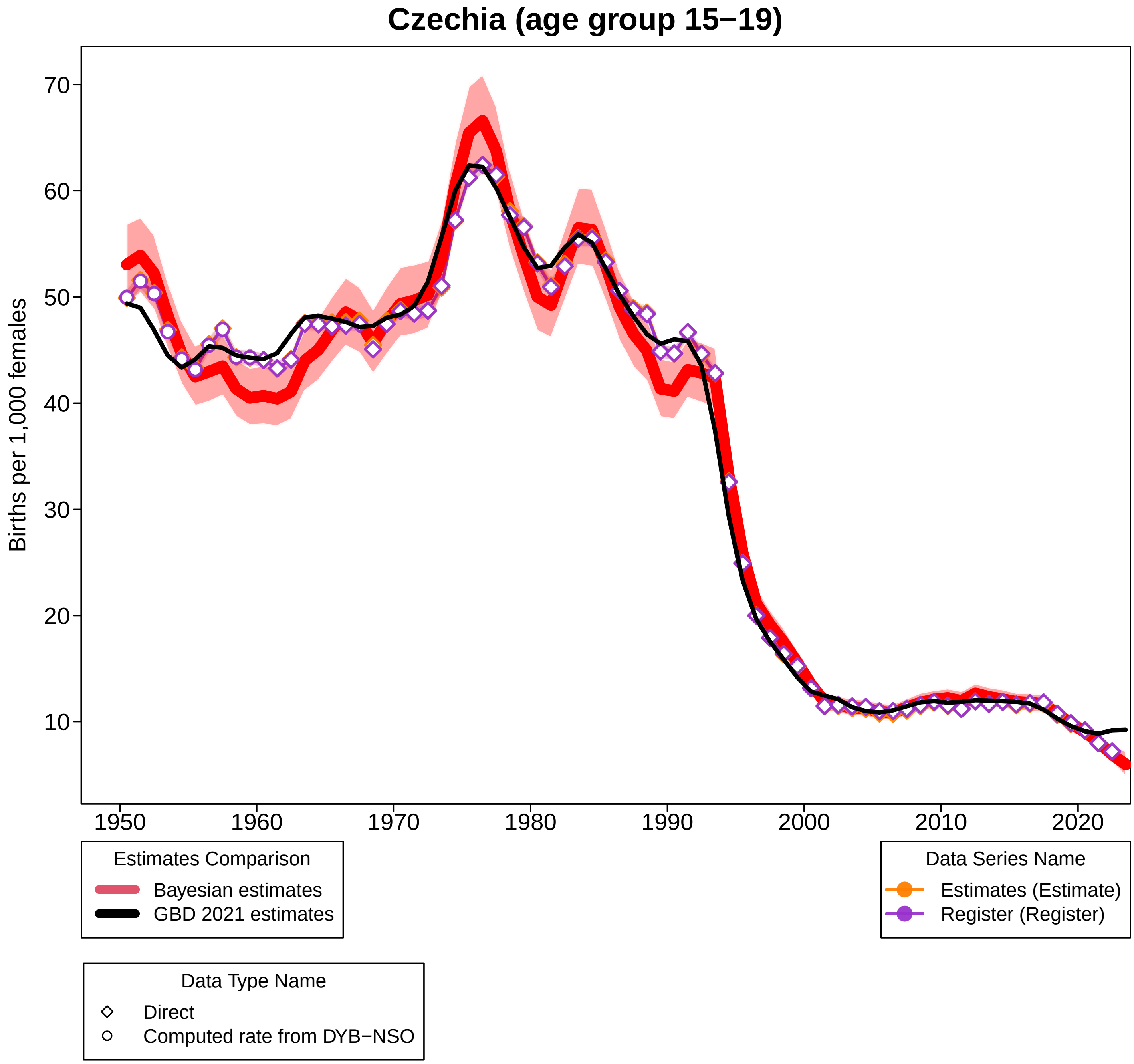
Czechia (age group 15−19)
Births per 1,000 females
70
60
50
40
30
20
10
1950
1960
1970
1980
1990
2000
2010
2020
Estimates Comparison
Bayesian estimates
GBD 2021 estimates
Data Series Name
Estimates (Estimate)
Register (Register)
Data Type Name
Direct
Computed rate from DYB−NSO

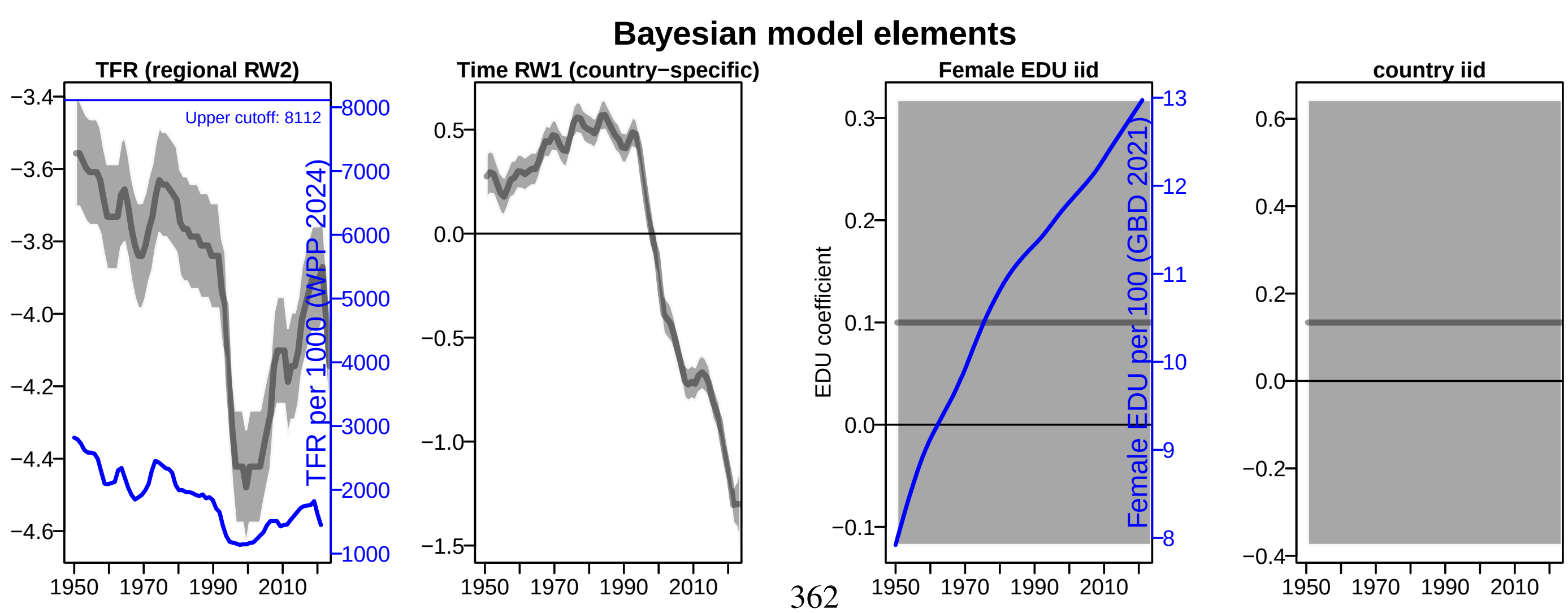
Bayesian model elements
TFR (regional RW2)
Upper cutoff: 8112
TFR per 1000 (WPP 2024)
Time RW1 (country−specific)
Female EDU iid
EDU coefficient
Female EDU per 100 (GBD 2021)
country iid

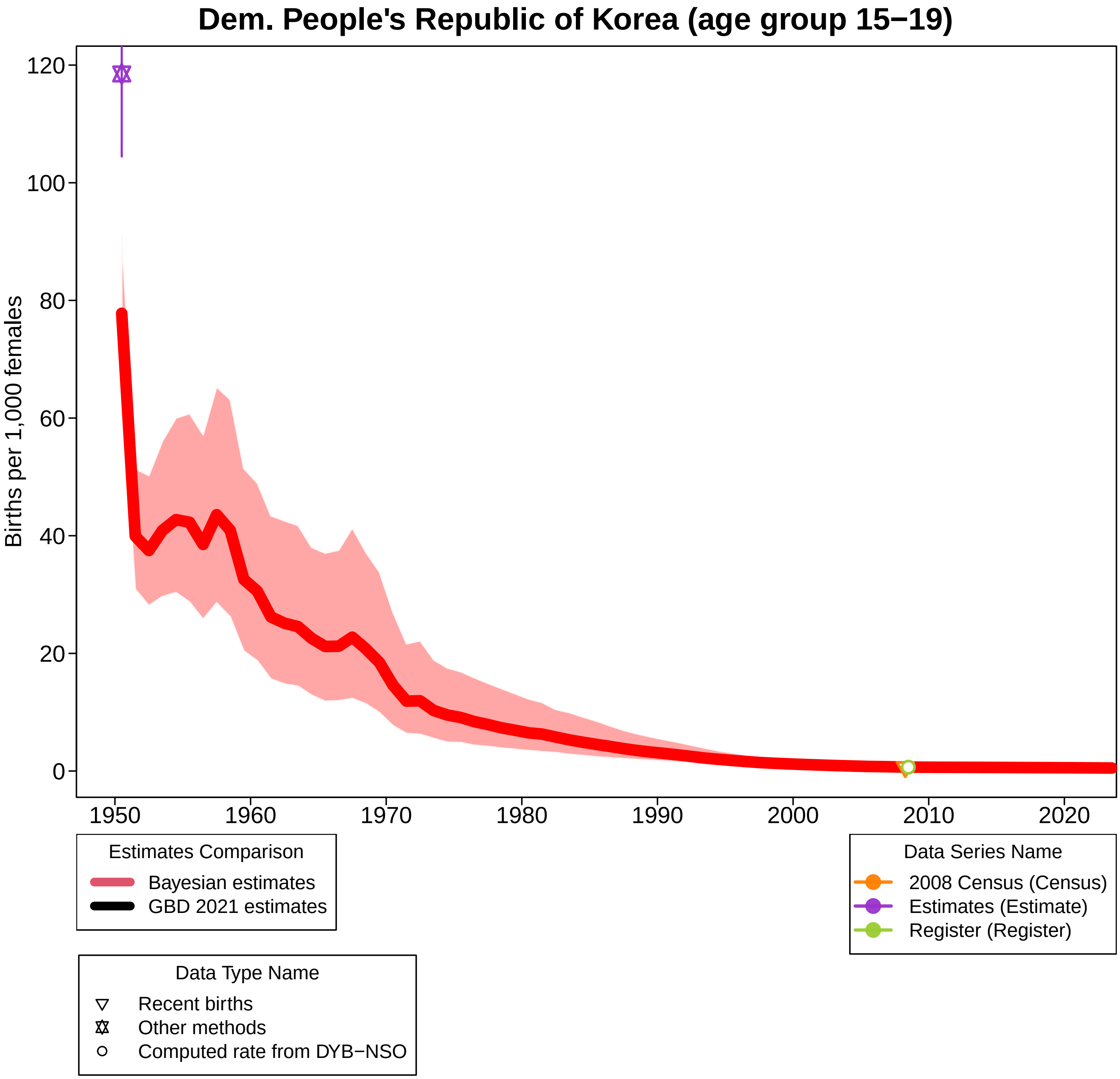
Dem. People's Republic of Korea (age group 15−19)
Births per 1,000 females
Estimates Comparison
Bayesian estimates
GBD 2021 estimates
Data Series Name
2008 Census (Census)
Estimates (Estimate)
Register (Register)
Data Type Name
Recent births
Other methods
Computed rate from DYB−NSO

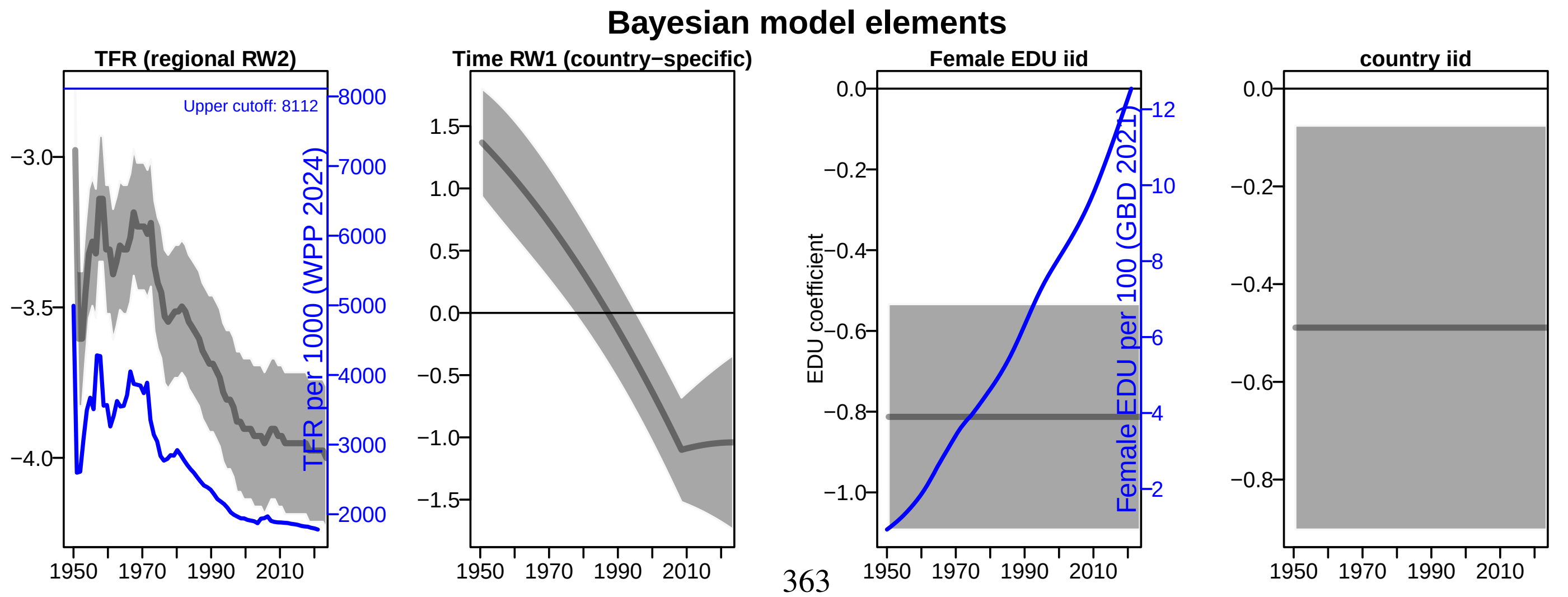
Bayesian model elements
TFR (regional RW2)
Upper cutoff: 8112
TFR per 1000 (WPP 2024)
Time RW1 (country−specific)
Female EDU iid
EDU coefficient
Female EDU per 100 (GBD 2021)
country iid

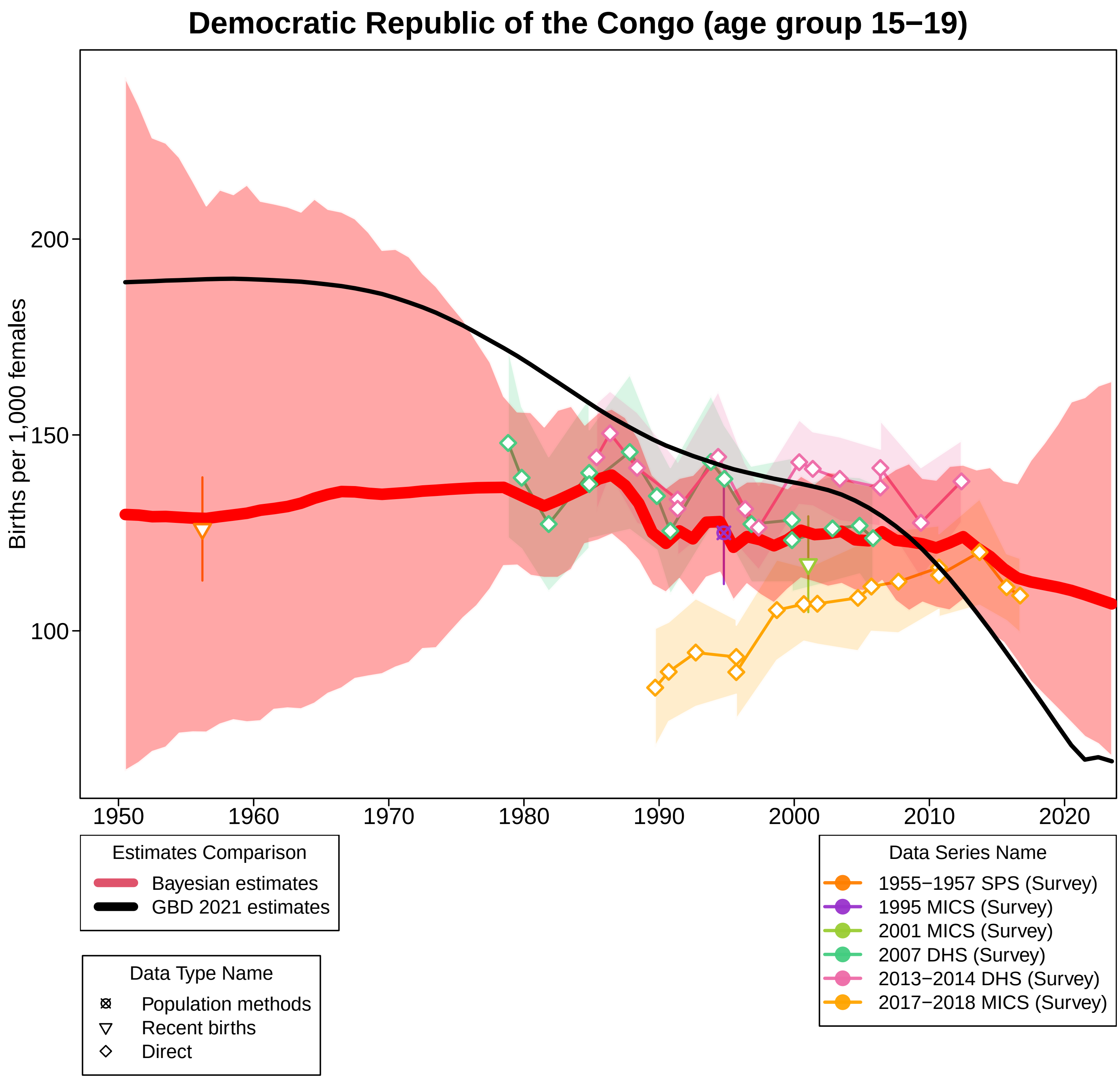
Democratic Republic of the Congo (age group 15−19)
Births per 1,000 females
200
150
100
1950
1960
1970
1980
1990
2000
2010
2020
Estimates Comparison
Bayesian estimates
GBD 2021 estimates
Data Type Name
Population methods
Recent births
Direct
Data Series Name
1955−1957 SPS (Survey)
1995 MICS (Survey)
2001 MICS (Survey)
2007 DHS (Survey)
2013−2014 DHS (Survey)
2017−2018 MICS (Survey)

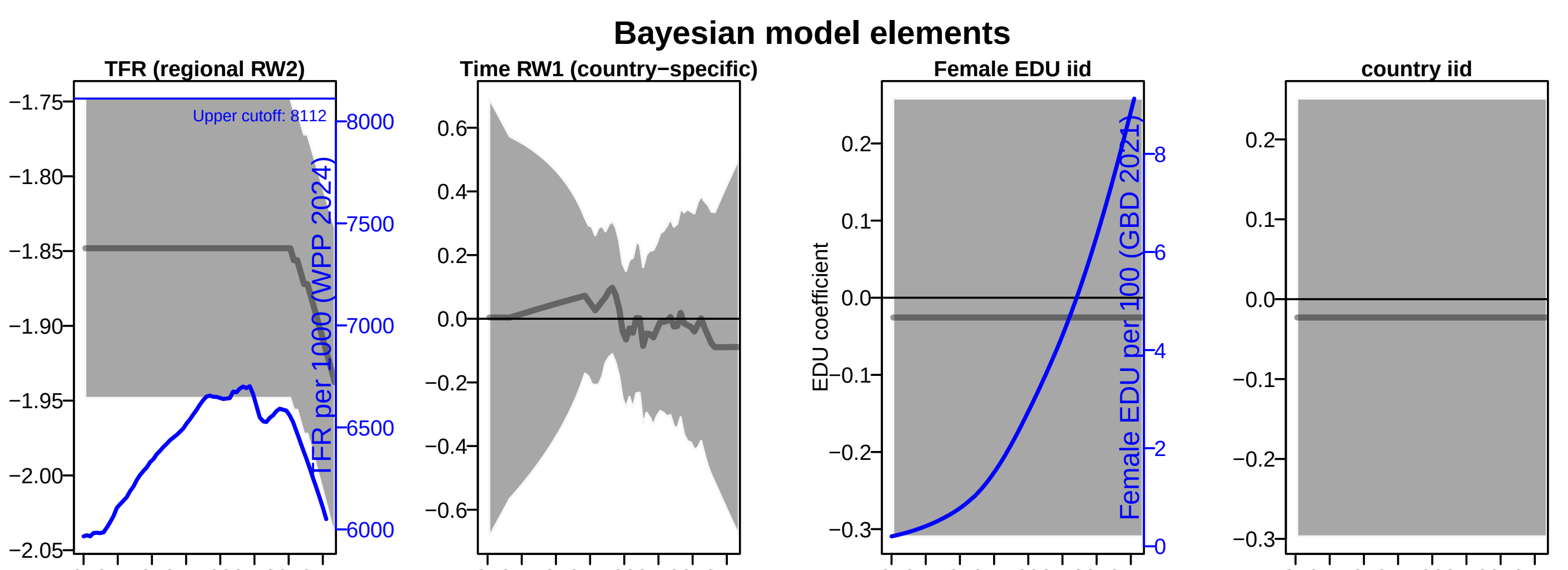
Bayesian model elements
TFR (regional RW2)
Upper cutoff: 8112
TFR per 1000 (WPP 2024)
Time RW1 (country−specific)
Female EDU iid
EDU coefficient
Female EDU per 100 (GBD 2021)
country iid
1950
1970
1990
2010

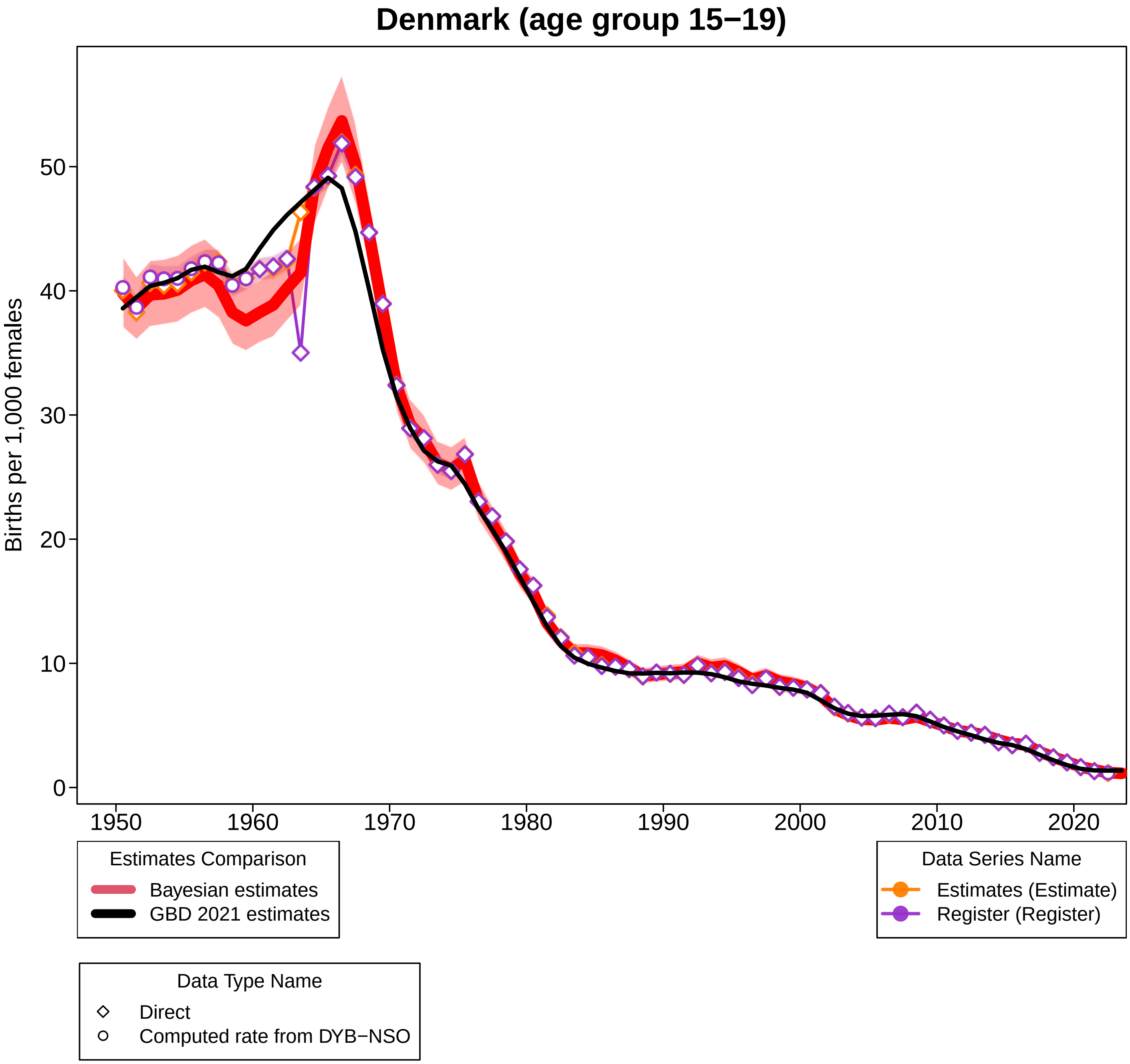
Denmark (age group 15−19)
Births per 1,000 females
0
10
20
30
40
50
1950
1960
1970
1980
1990
2000
2010
2020
Estimates Comparison
Bayesian estimates
GBD 2021 estimates
Data Series Name
Estimates (Estimate)
Register (Register)
Data Type Name
Direct
Computed rate from DYB−NSO

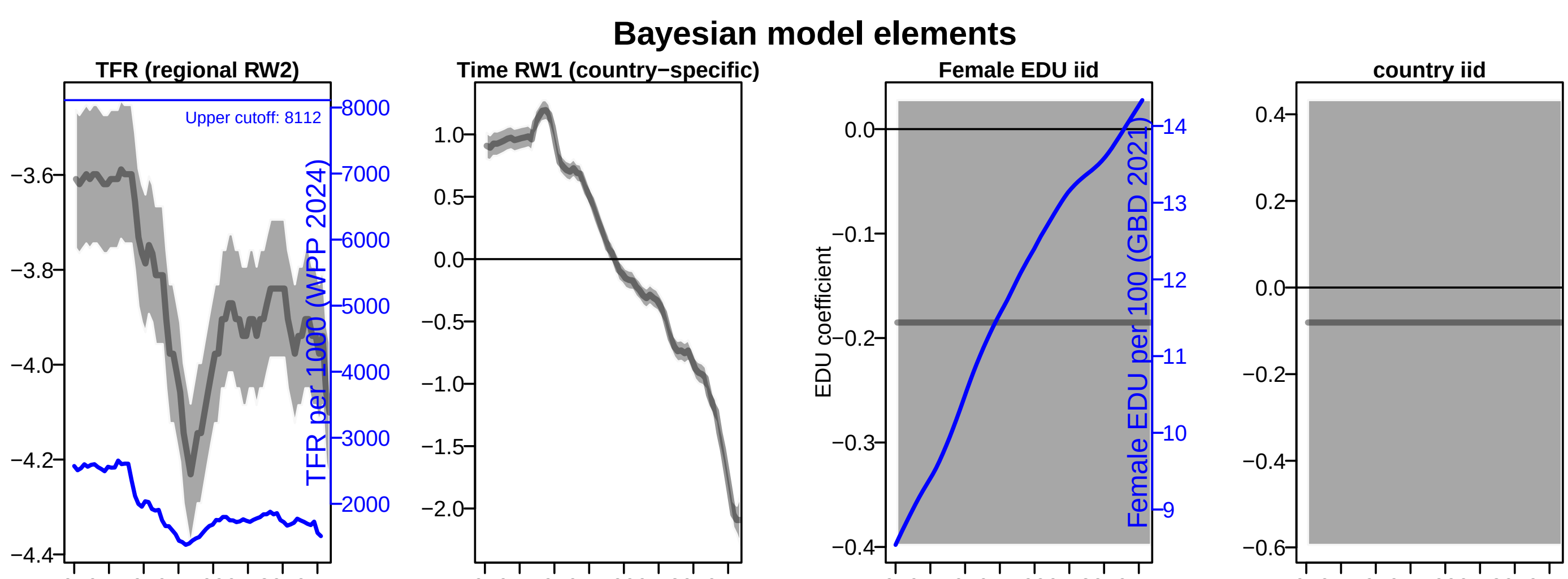
Bayesian model elements
TFR (regional RW2)
Upper cutoff: 8112
TFR per 1000 (WPP 2024)
Time RW1 (country−specific)
Female EDU iid
EDU coefficient
Female EDU per 100 (GBD 2021)
country iid

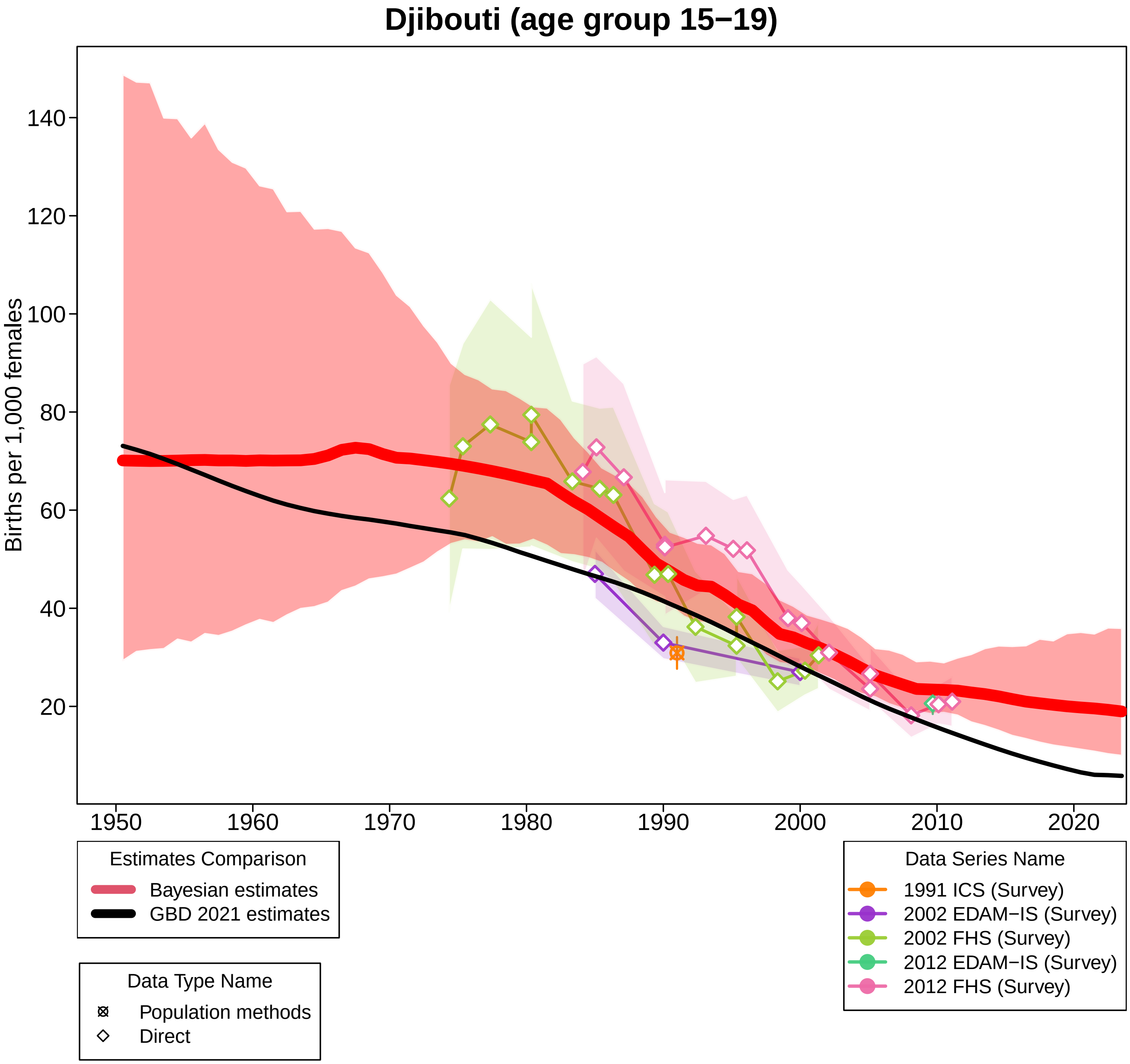
Djibouti (age group 15−19)
Births per 1,000 females
140
120
100
80
60
40
20
1950
1960
1970
1980
1990
2000
2010
2020
Estimates Comparison
Bayesian estimates
GBD 2021 estimates
Data Type Name
Population methods
Direct
Data Series Name
1991 ICS (Survey)
2002 EDAM−IS (Survey)
2002 FHS (Survey)
2012 EDAM−IS (Survey)
2012 FHS (Survey)

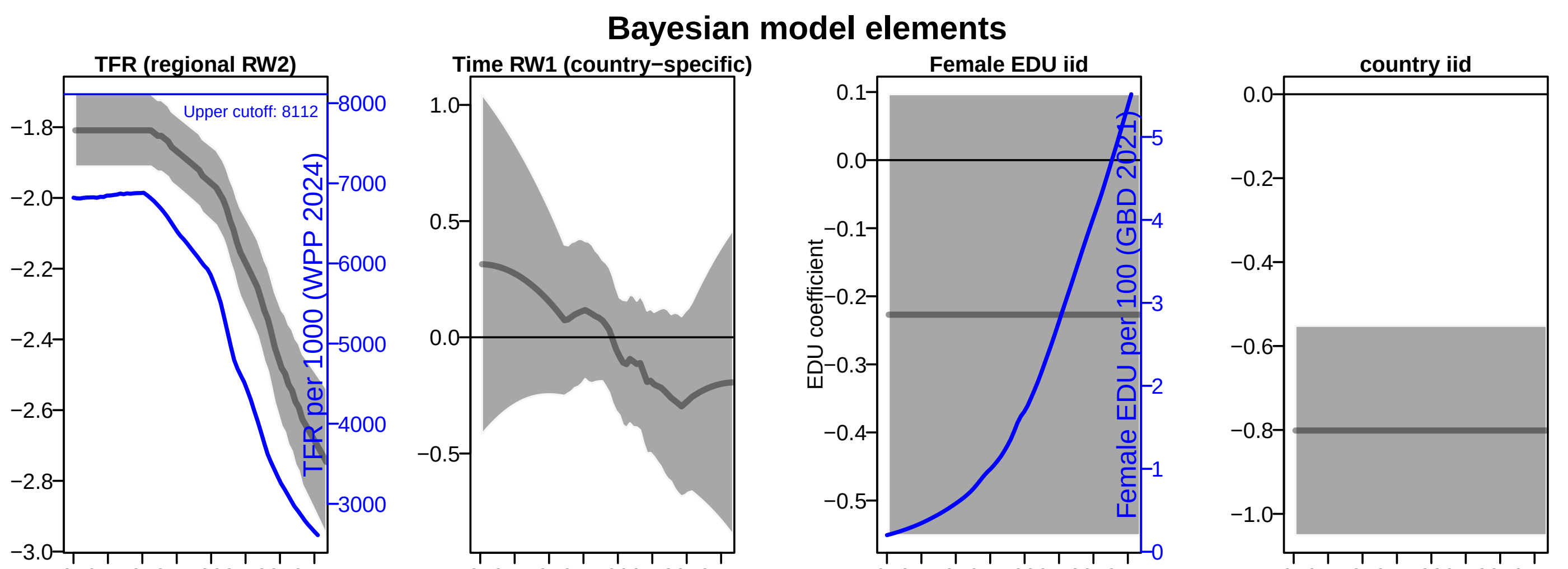
Bayesian model elements
TFR (regional RW2)
Upper cutoff: 8112
TFR per 1000 (WPP 2024)
Time RW1 (country−specific)
Female EDU iid
EDU coefficient
Female EDU per 100 (GBD 2021)
country iid

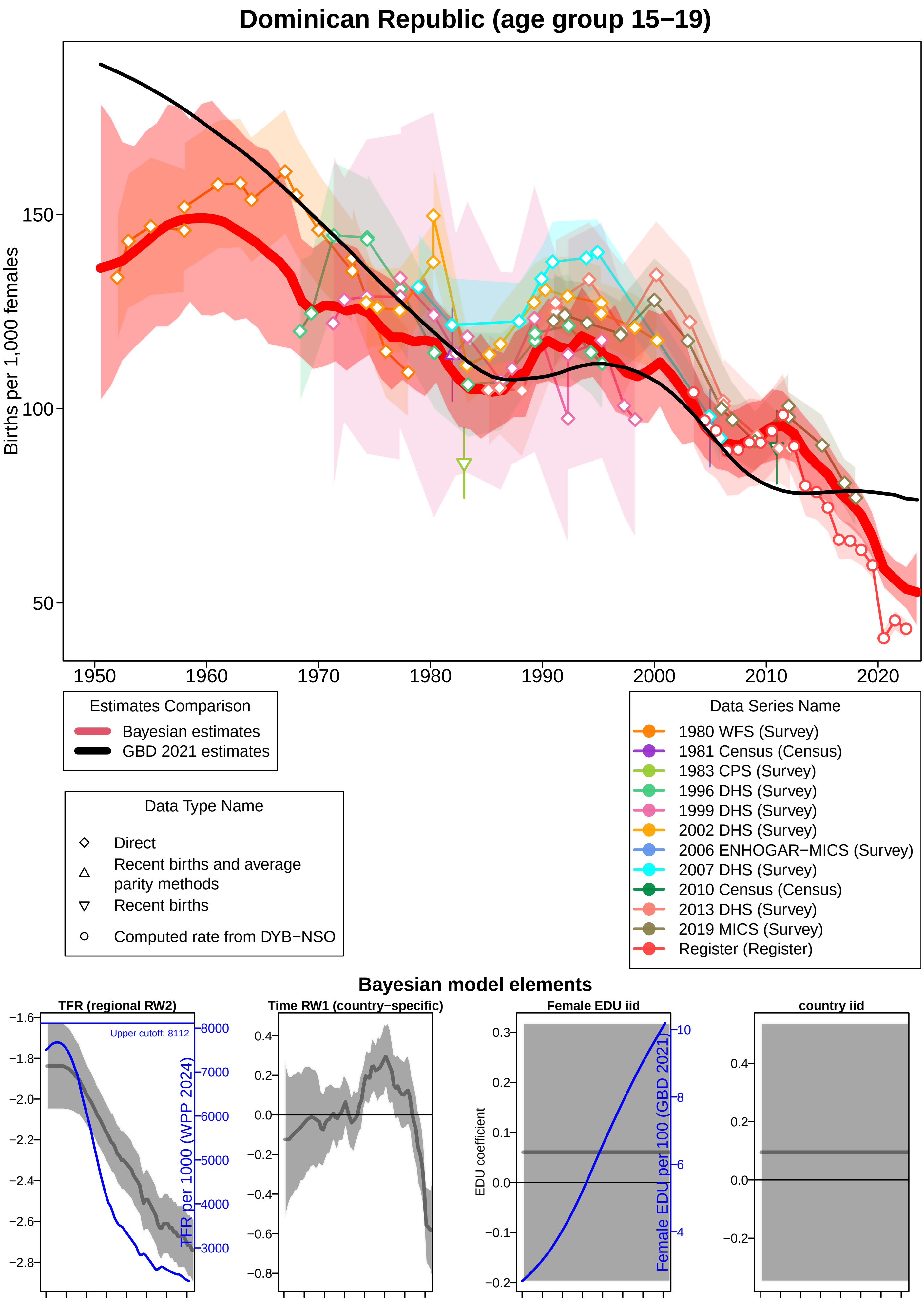
Dominican Republic (age group 15−19)
Births per 1,000 females
150
100
50
1950
1960
1970
1980
1990
2000
2010
2020
Estimates Comparison
Bayesian estimates
GBD 2021 estimates
Data Type Name
Direct
Recent births and average parity methods
Recent births
Computed rate from DYB−NSO
Data Series Name
1980 WFS (Survey)
1981 Census (Census)
1983 CPS (Survey)
1996 DHS (Survey)
1999 DHS (Survey)
2002 DHS (Survey)
2006 ENHOGAR−MICS (Survey)
2007 DHS (Survey)
2010 Census (Census)
2013 DHS (Survey)
2019 MICS (Survey)
Register (Register)
Bayesian model elements
TFR (regional RW2)
Upper cutoff: 8112
TFR per 1000 (WPP 2024)
Time RW1 (country−specific)
Female EDU iid
EDU coefficient
Female EDU per 100 (GBD 2021)
country iid

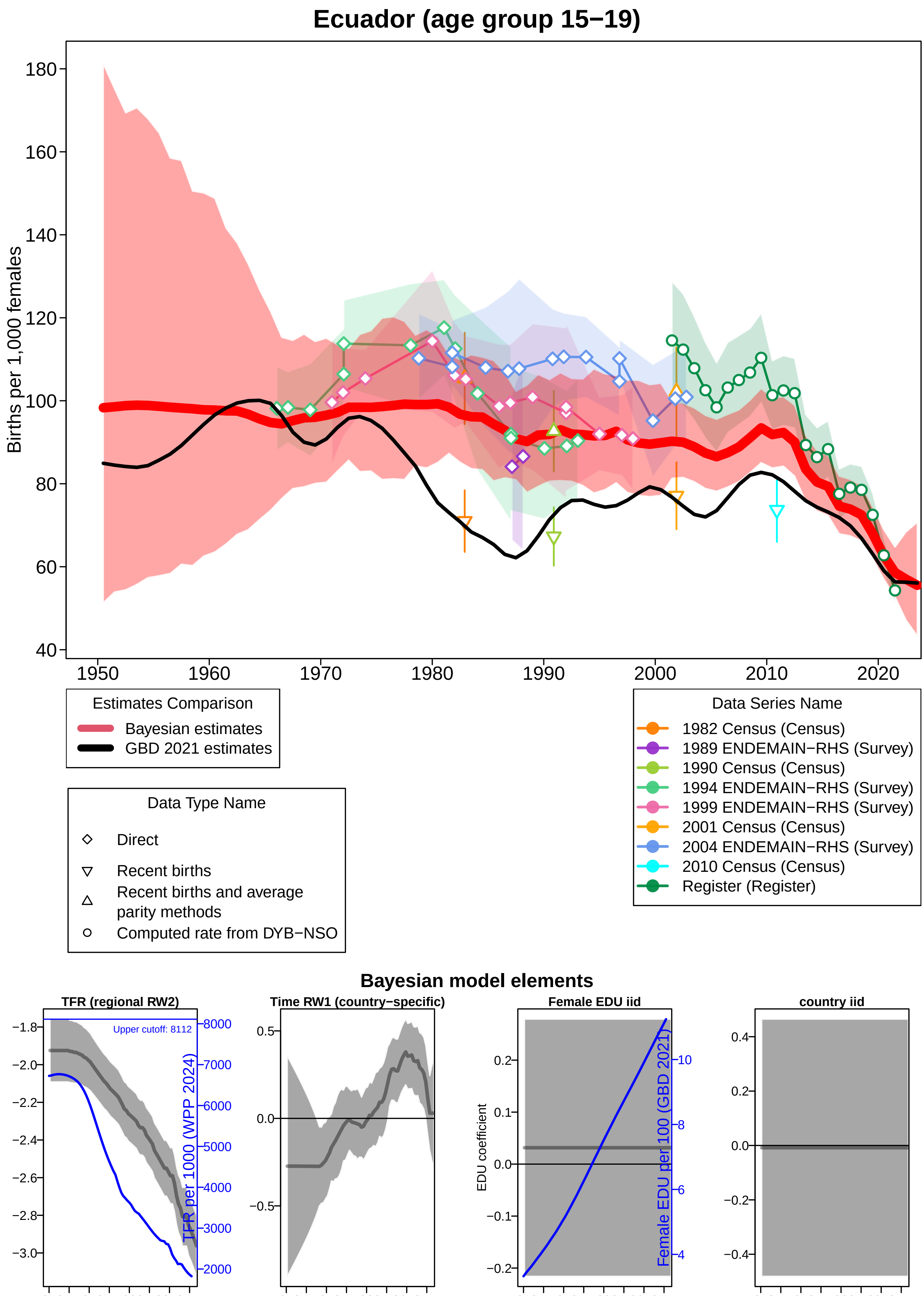
Ecuador (age group 15−19)
Births per 1,000 females
180
160
140
120
100
80
60
40
1950
1960
1970
1980
1990
2000
2010
2020
Estimates Comparison
Bayesian estimates
GBD 2021 estimates
Data Series Name
1982 Census (Census)
1989 ENDEMAIN−RHS (Survey)
1990 Census (Census)
1994 ENDEMAIN−RHS (Survey)
1999 ENDEMAIN−RHS (Survey)
2001 Census (Census)
2004 ENDEMAIN−RHS (Survey)
2010 Census (Census)
Register (Register)
Data Type Name
Direct
Recent births
Recent births and average parity methods
Computed rate from DYB−NSO
Bayesian model elements
TFR (regional RW2)
Upper cutoff: 8112
TFR per 1000 (WPP 2024)
Time RW1 (country−specific)
Female EDU iid
EDU coefficient
Female EDU per 100 (GBD 2021)
country iid

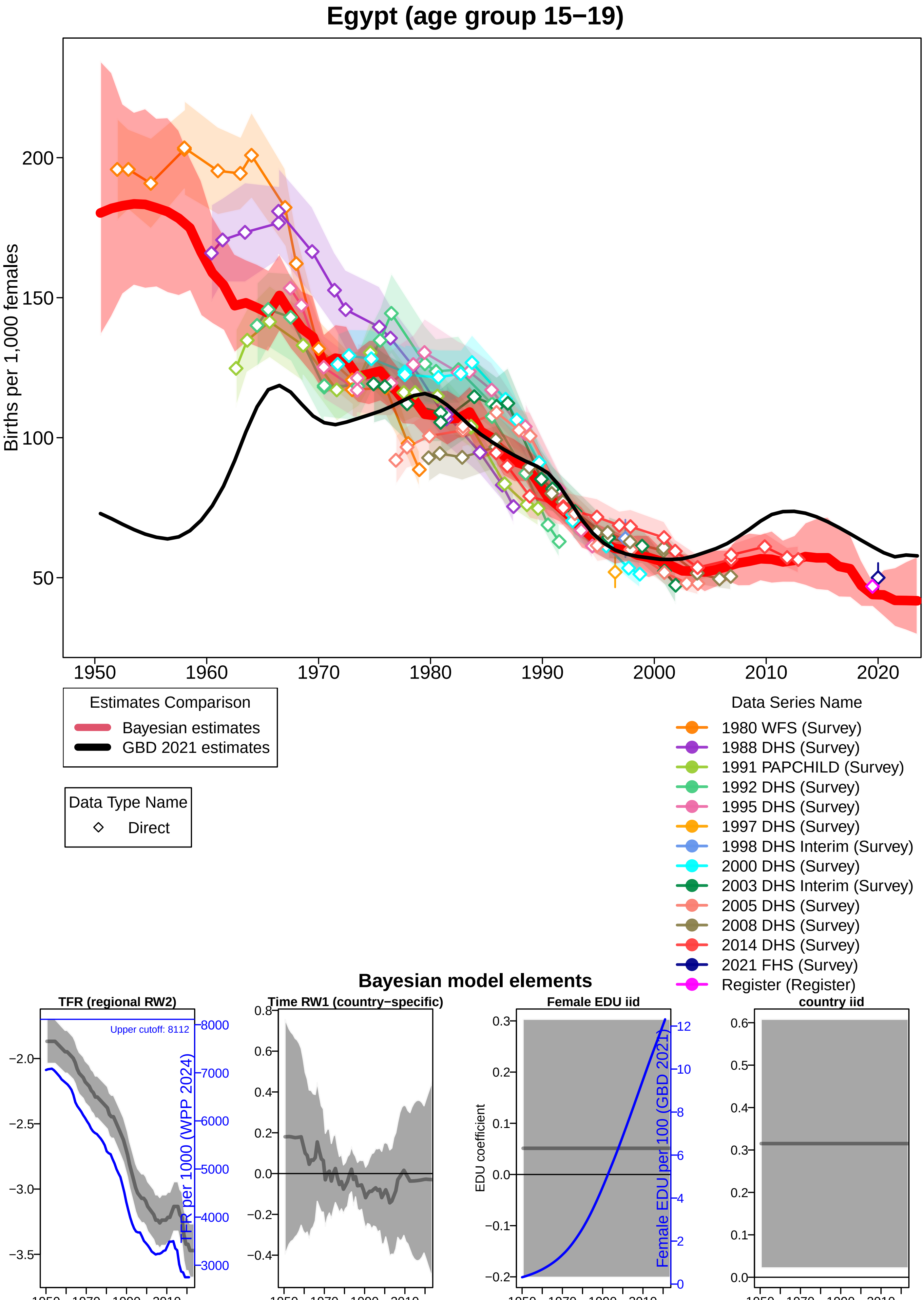

Egypt (age group 15−19)
Births per 1,000 females
200
150
100
50
1950
1960
1970
1980
1990
2000
2010
2020
Estimates Comparison
Bayesian estimates
GBD 2021 estimates
Data Type Name
Direct
Data Series Name
1980 WFS (Survey)
1988 DHS (Survey)
1991 PAPCHILD (Survey)
1992 DHS (Survey)
1995 DHS (Survey)
1997 DHS (Survey)
1998 DHS Interim (Survey)
2000 DHS (Survey)
2003 DHS Interim (Survey)
2005 DHS (Survey)
2008 DHS (Survey)
2014 DHS (Survey)
2021 FHS (Survey)
Register (Register)
Bayesian model elements
TFR (regional RW2)
Upper cutoff: 8112
TFR per 1000 (WPP 2024)
Time RW1 (country−specific)
Female EDU iid
EDU coefficient
Female EDU per 100 (GBD 2021)
country iid

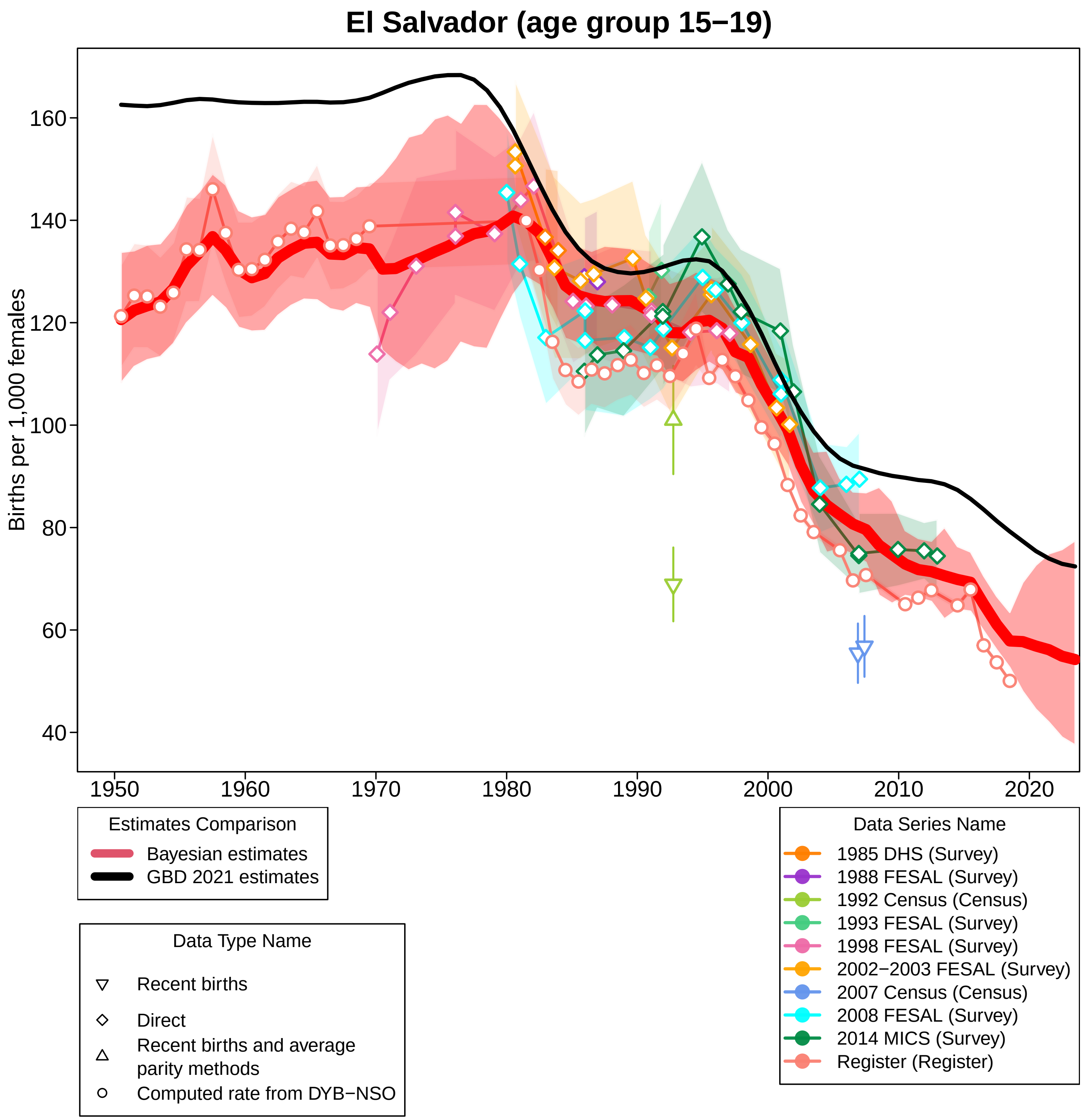
El Salvador (age group 15−19)
Births per 1,000 females
160
140
120
100
80
60
40
1950
1960
1970
1980
1990
2000
2010
2020
Estimates Comparison
Bayesian estimates
GBD 2021 estimates
Data Type Name
Recent births
Direct
Recent births and average parity methods
Computed rate from DYB−NSO
Data Series Name
1985 DHS (Survey)
1988 FESAL (Survey)
1992 Census (Census)
1993 FESAL (Survey)
1998 FESAL (Survey)
2002−2003 FESAL (Survey)
2007 Census (Census)
2008 FESAL (Survey)
2014 MICS (Survey)
Register (Register)

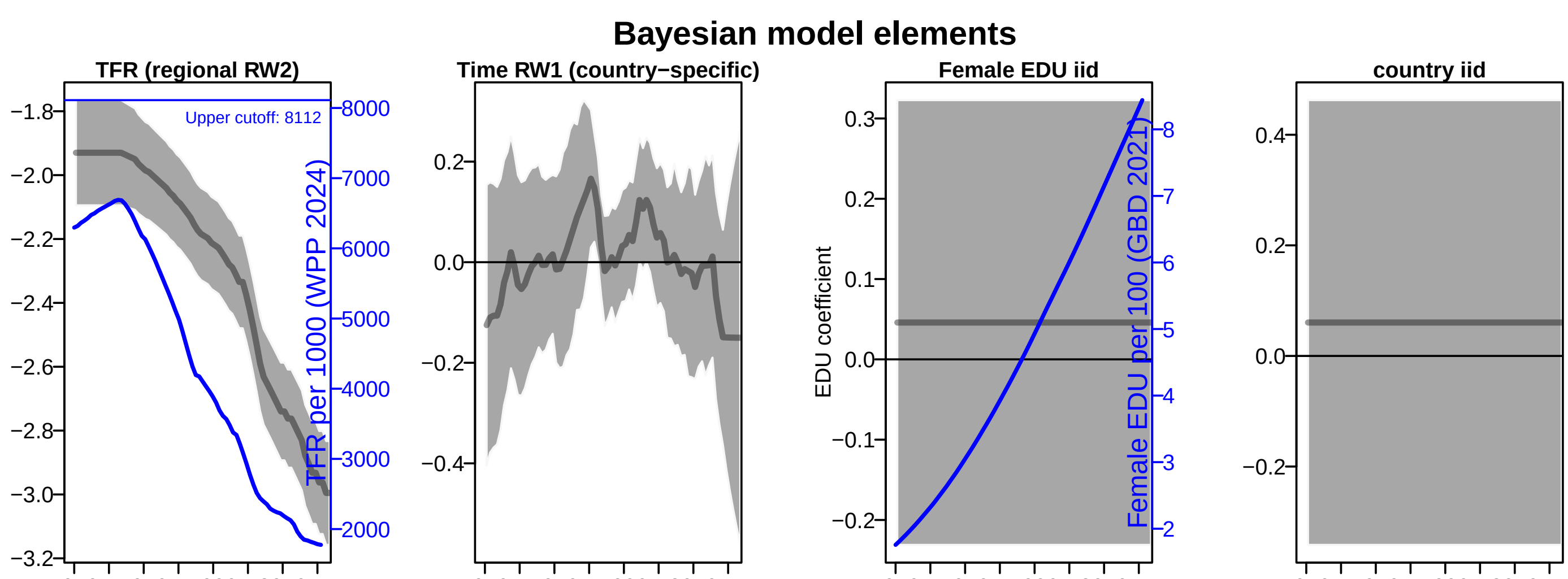
Bayesian model elements
TFR (regional RW2)
Upper cutoff: 8112
TFR per 1000 (WPP 2024)
Time RW1 (country−specific)
Female EDU iid
EDU coefficient
Female EDU per 100 (GBD 2021)
country iid
1950
1970
1990
2010

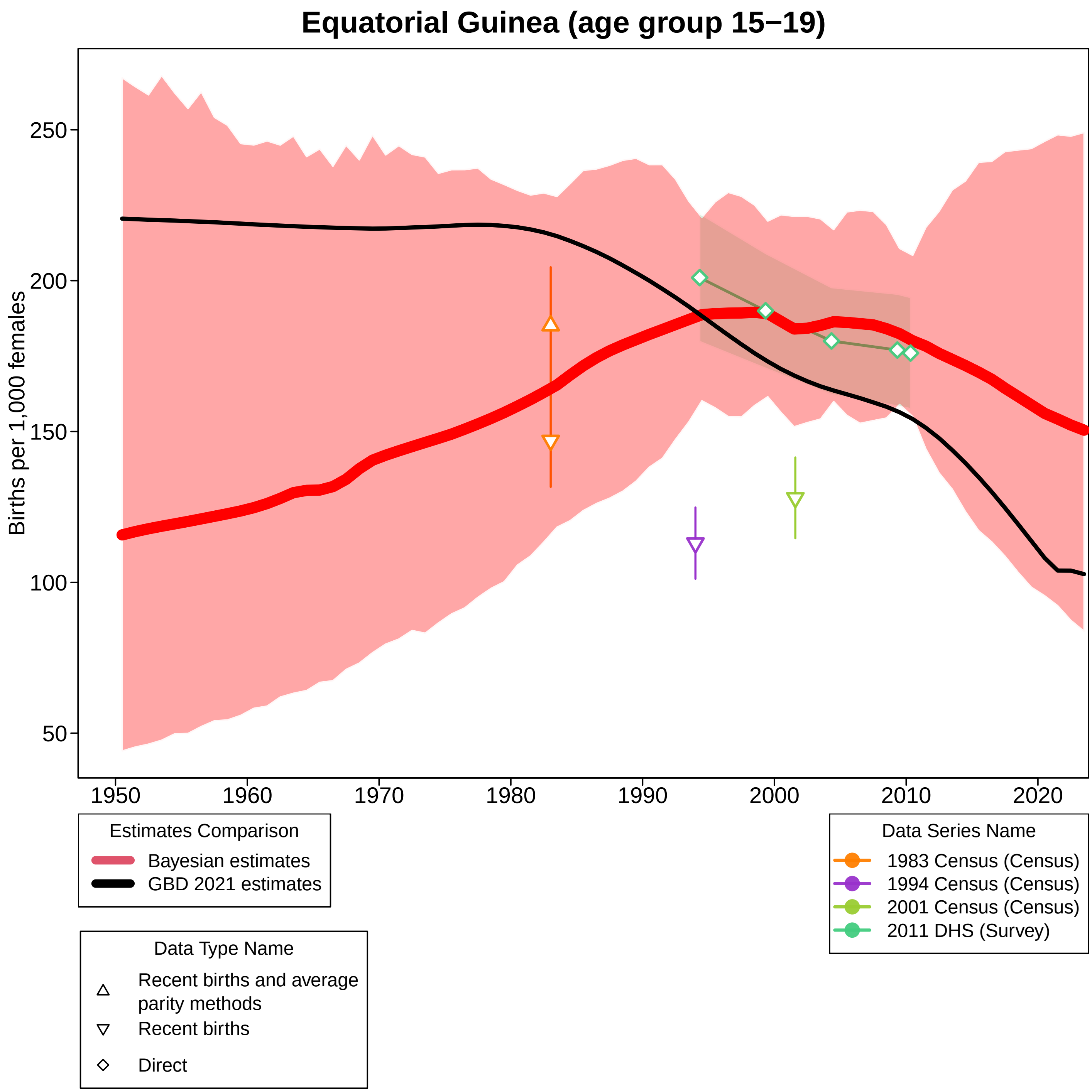
Equatorial Guinea (age group 15−19)
Births per 1,000 females
250
200
150
100
50
1950
1960
1970
1980
1990
2000
2010
2020
Estimates Comparison
Bayesian estimates
GBD 2021 estimates
Data Series Name
1983 Census (Census)
1994 Census (Census)
2001 Census (Census)
2011 DHS (Survey)
Data Type Name
Recent births and average parity methods
Recent births
Direct

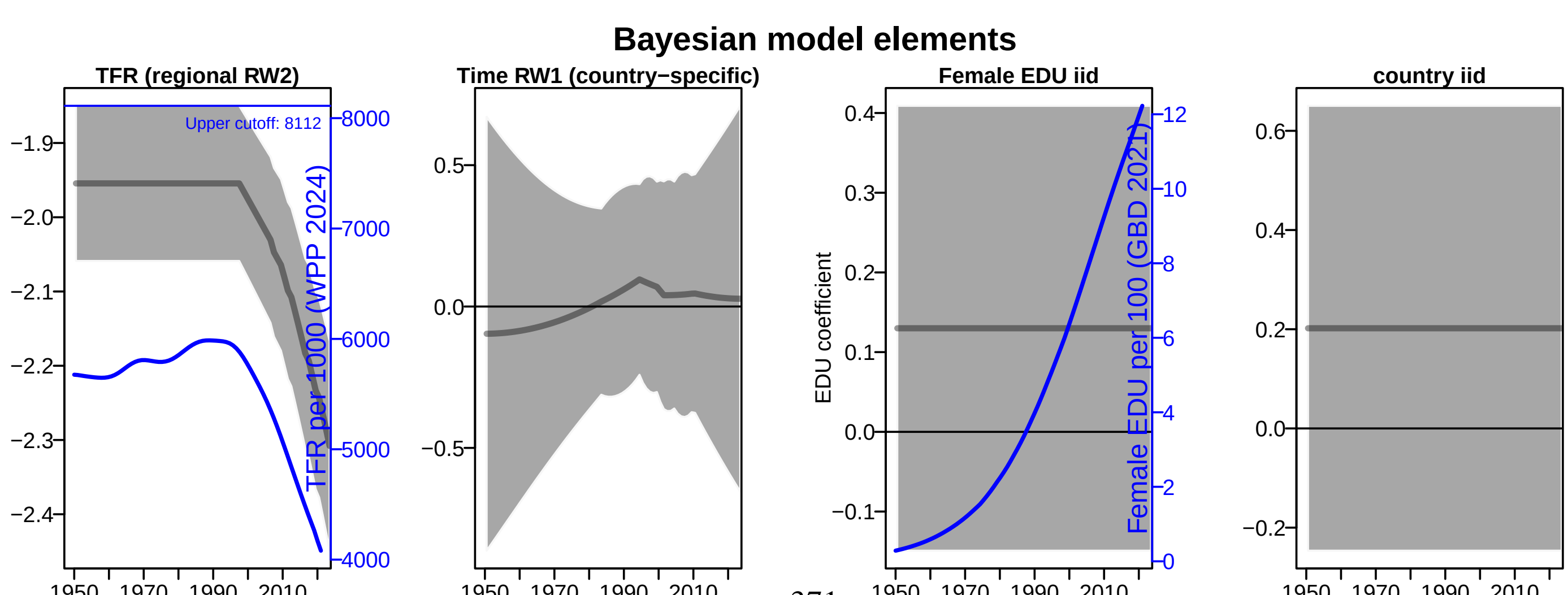
Bayesian model elements
TFR (regional RW2)
Upper cutoff: 8112
TFR per 1000 (WPP 2024)
Time RW1 (country−specific)
Female EDU iid
EDU coefficient
Female EDU per 100 (GBD 2021)
country iid

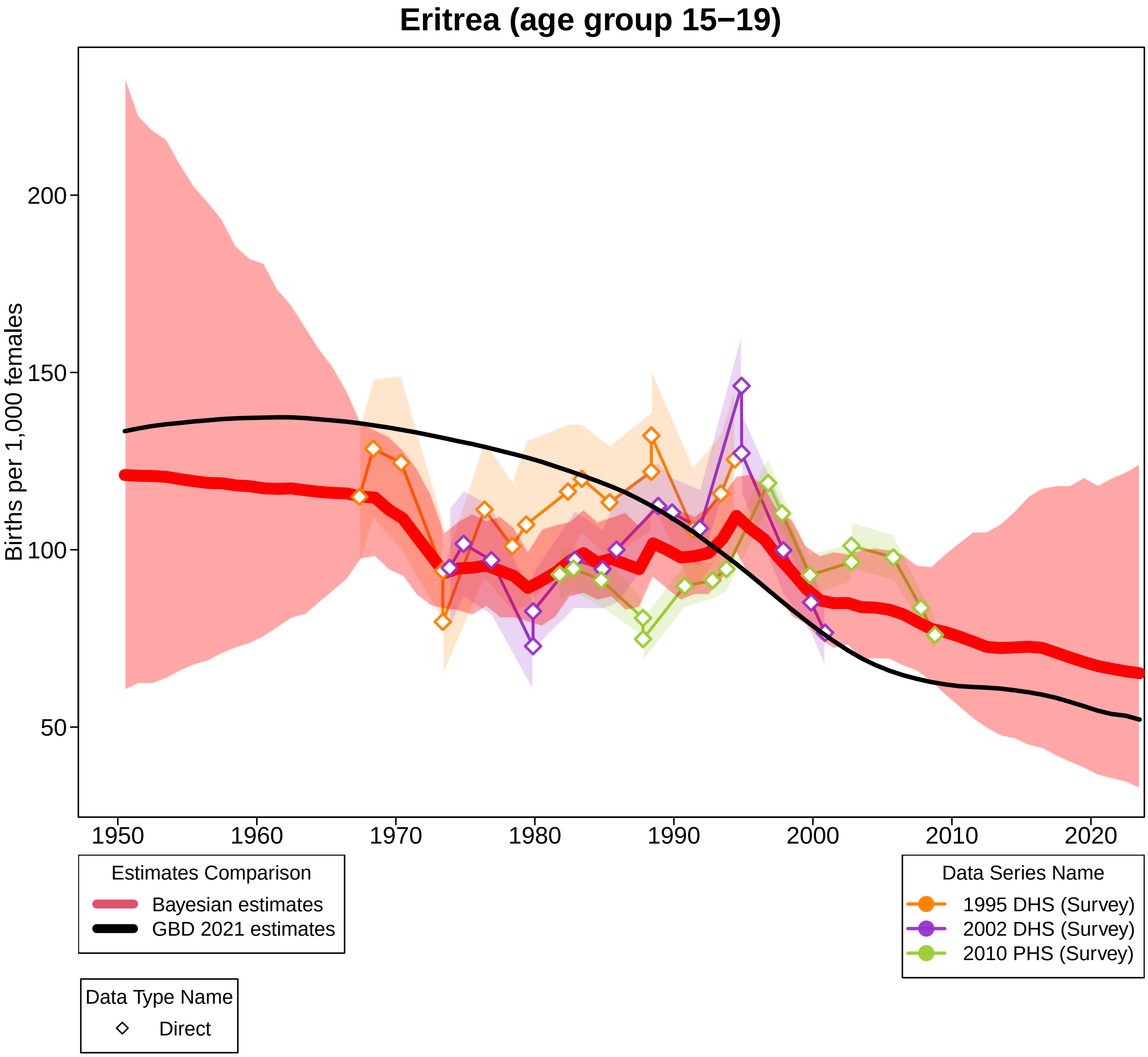

Eritrea (age group 15−19)
Births per 1,000 females
200
150
100
50
1950
1960
1970
1980
1990
2000
2010
2020
Estimates Comparison
Bayesian estimates
GBD 2021 estimates
Data Series Name
1995 DHS (Survey)
2002 DHS (Survey)
2010 PHS (Survey)
Data Type Name
Direct

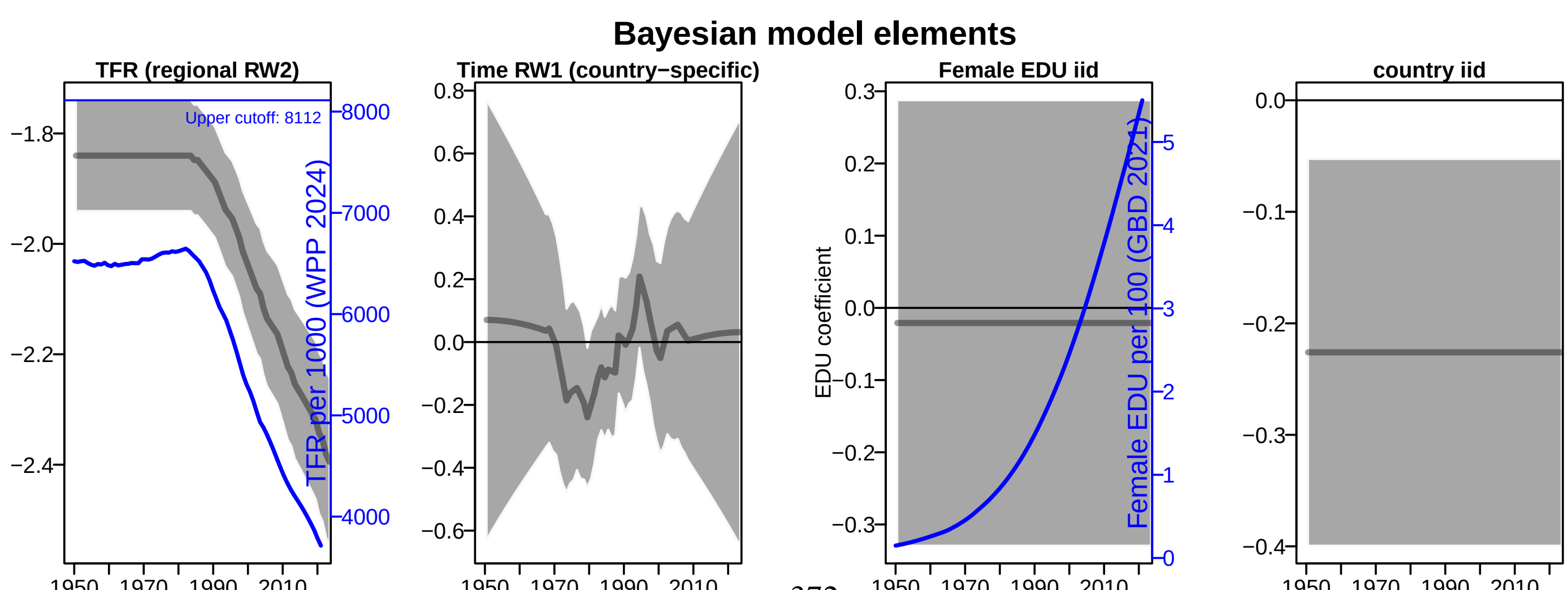

Bayesian model elements
TFR (regional RW2)
Upper cutoff: 8112
TFR per 1000 (WPP 2024)
Time RW1 (country−specific)
Female EDU iid
EDU coefficient
Female EDU per 100 (GBD 2021)
country iid

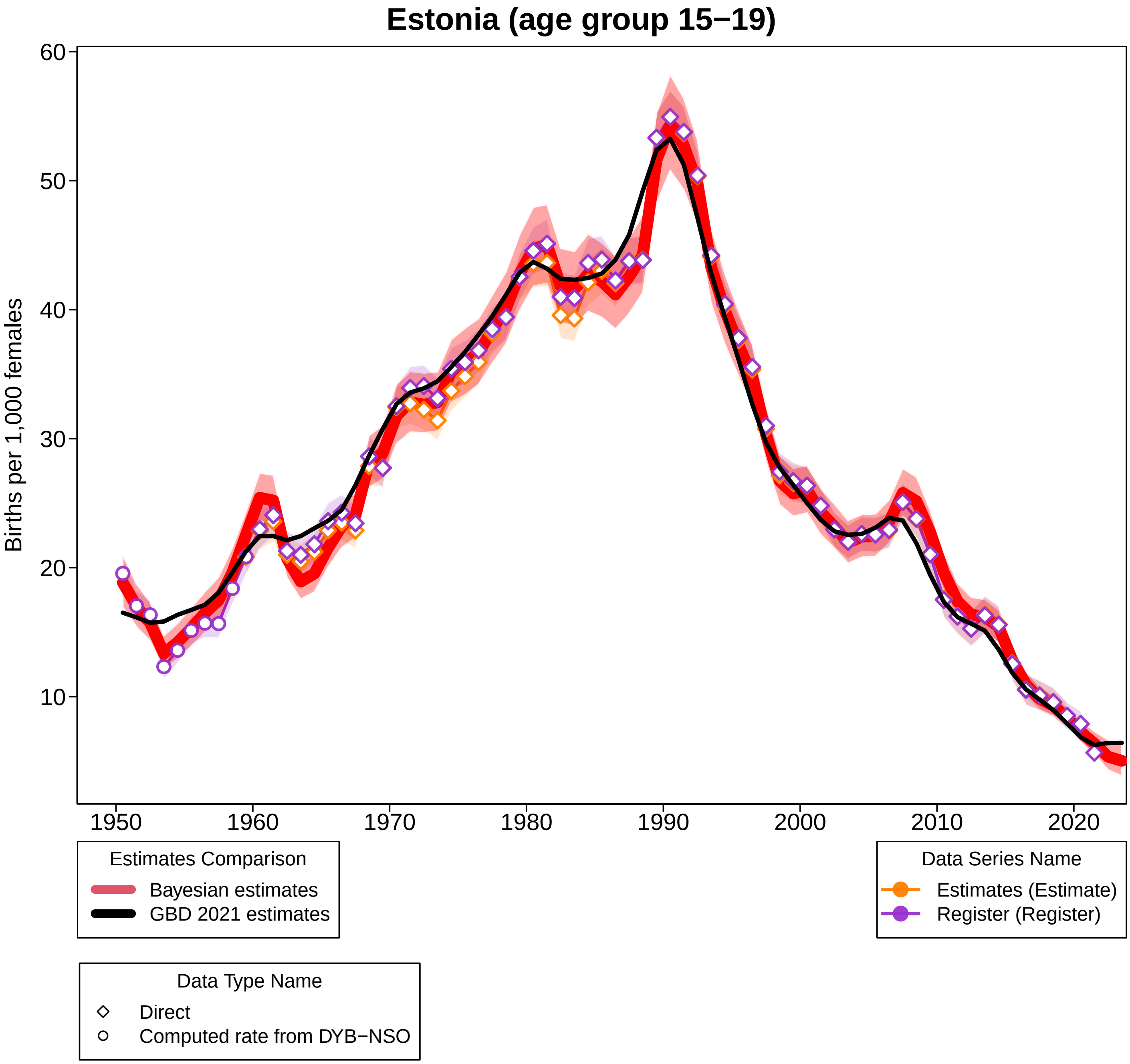
Estonia (age group 15−19)
Births per 1,000 females
60
50
40
30
20
10
1950
1960
1970
1980
1990
2000
2010
2020
Estimates Comparison
Bayesian estimates
GBD 2021 estimates
Data Series Name
Estimates (Estimate)
Register (Register)
Data Type Name
Direct
Computed rate from DYB−NSO

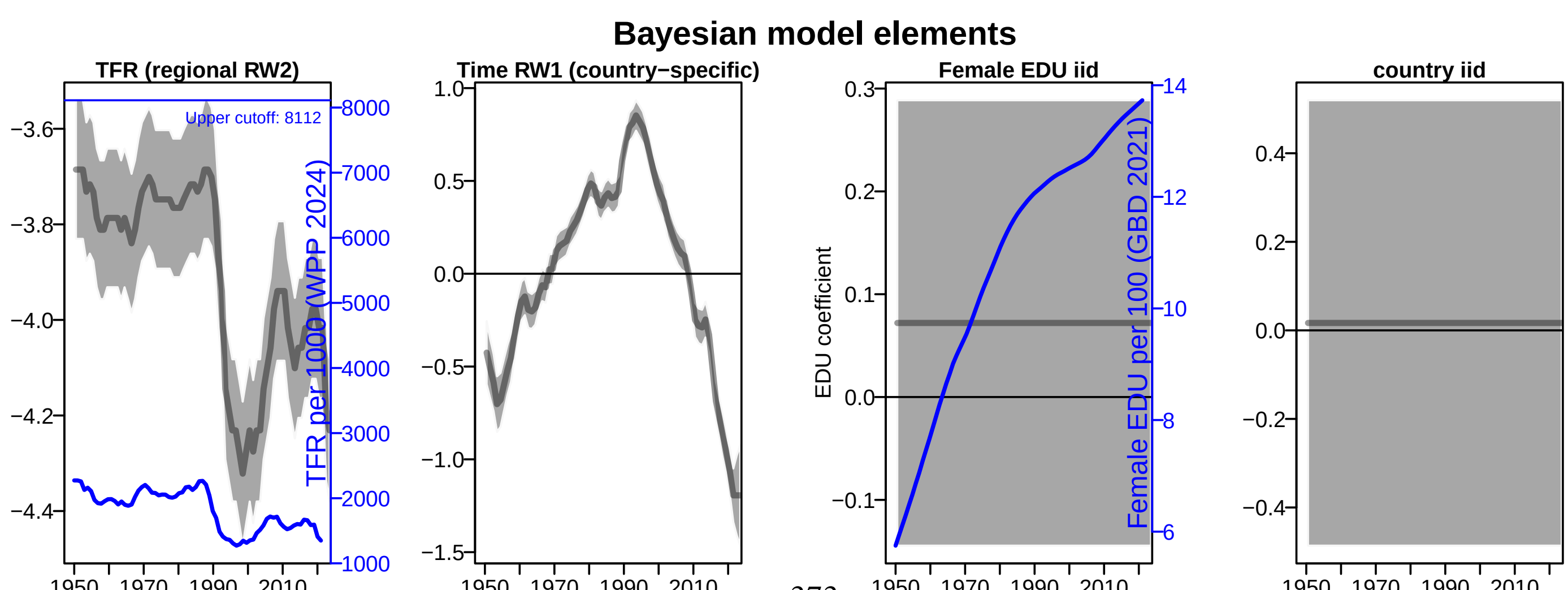
Bayesian model elements
TFR (regional RW2)
Upper cutoff: 8112
TFR per 1000 (WPP 2024)
Time RW1 (country−specific)
Female EDU iid
EDU coefficient
Female EDU per 100 (GBD 2021)
country iid

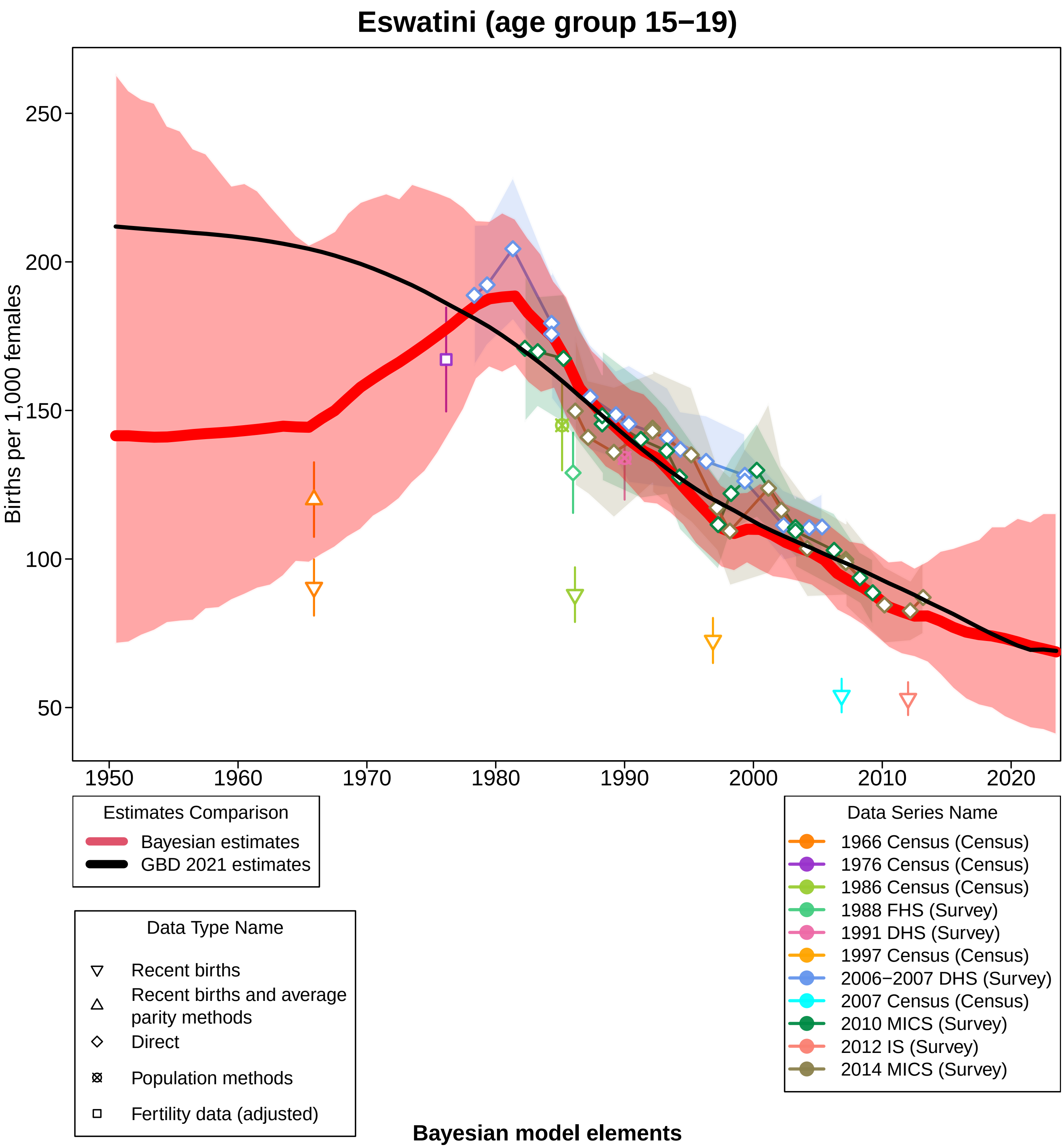
Eswatini (age group 15−19)
Births per 1,000 females
Estimates Comparison
Bayesian estimates
GBD 2021 estimates
Data Series Name
1966 Census (Census)
1976 Census (Census)
1986 Census (Census)
1988 FHS (Survey)
1991 DHS (Survey)
1997 Census (Census)
2006−2007 DHS (Survey)
2007 Census (Census)
2010 MICS (Survey)
2012 IS (Survey)
2014 MICS (Survey)
Data Type Name
Recent births
Recent births and average parity methods
Direct
Population methods
Fertility data (adjusted)

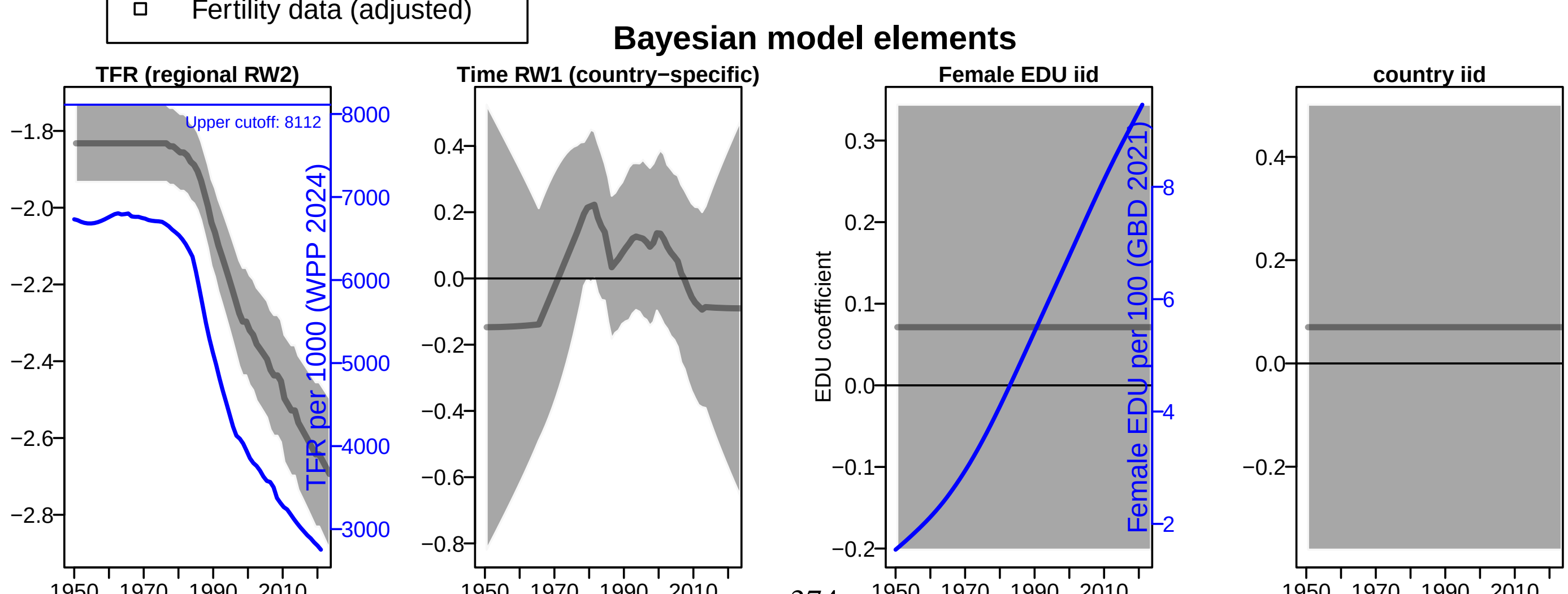
Bayesian model elements
TFR (regional RW2)
Upper cutoff: 8112
TFR per 1000 (WPP 2024)
Time RW1 (country−specific)
Female EDU iid
EDU coefficient
Female EDU per 100 (GBD 2021)
country iid

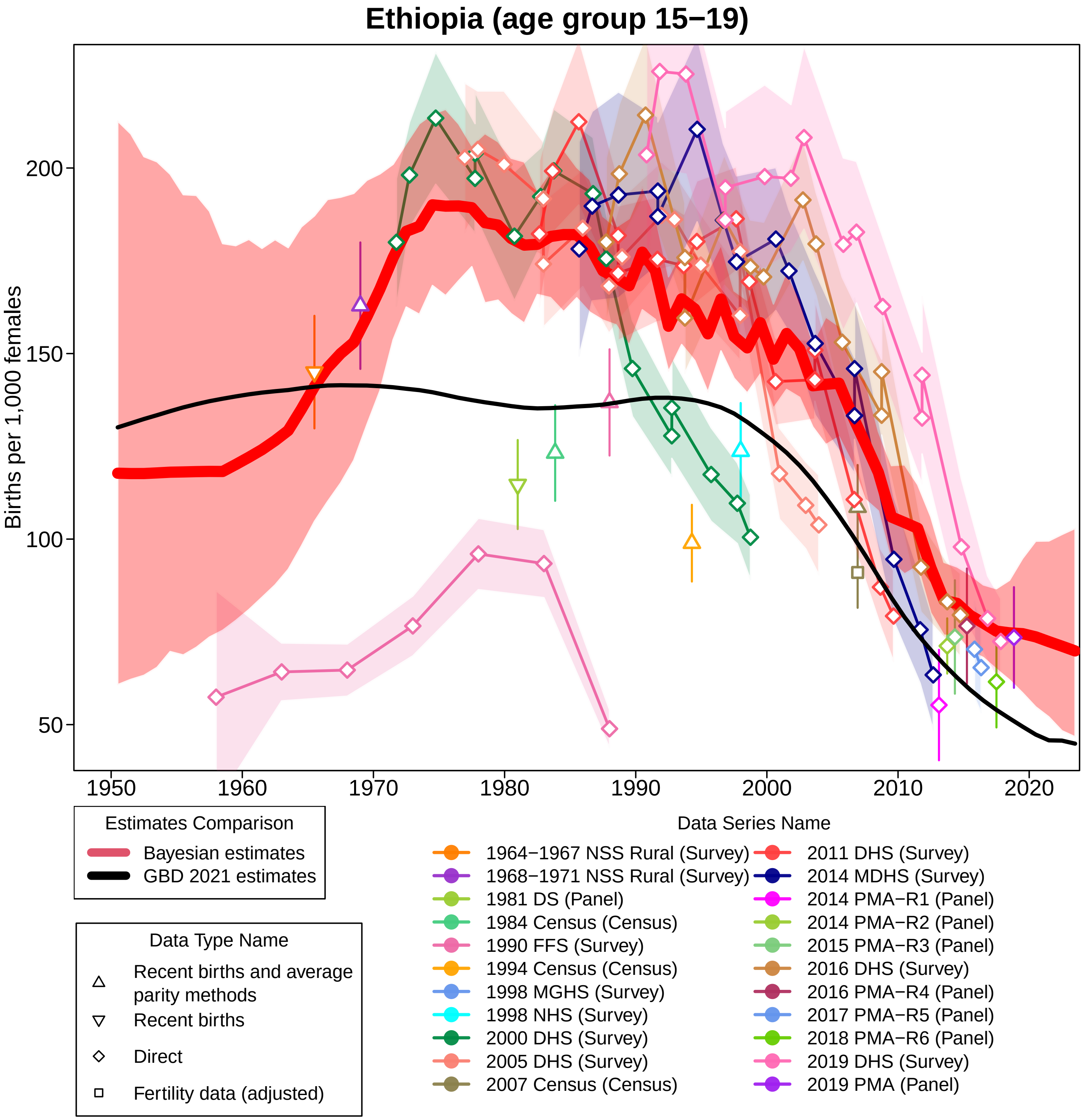
Ethiopia (age group 15−19)
Births per 1,000 females
200
150
100
50
1950
1960
1970
1980
1990
2000
2010
2020
Estimates Comparison
Bayesian estimates
GBD 2021 estimates
Data Type Name
Recent births and average parity methods
Recent births
Direct
Fertility data (adjusted)
Data Series Name
1964−1967 NSS Rural (Survey)
1968−1971 NSS Rural (Survey)
1981 DS (Panel)
1984 Census (Census)
1990 FFS (Survey)
1994 Census (Census)
1998 MGHS (Survey)
1998 NHS (Survey)
2000 DHS (Survey)
2005 DHS (Survey)
2007 Census (Census)
2011 DHS (Survey)
2014 MDHS (Survey)
2014 PMA−R1 (Panel)
2014 PMA−R2 (Panel)
2015 PMA−R3 (Panel)
2016 DHS (Survey)
2016 PMA−R4 (Panel)
2017 PMA−R5 (Panel)
2018 PMA−R6 (Panel)
2019 DHS (Survey)
2019 PMA (Panel)

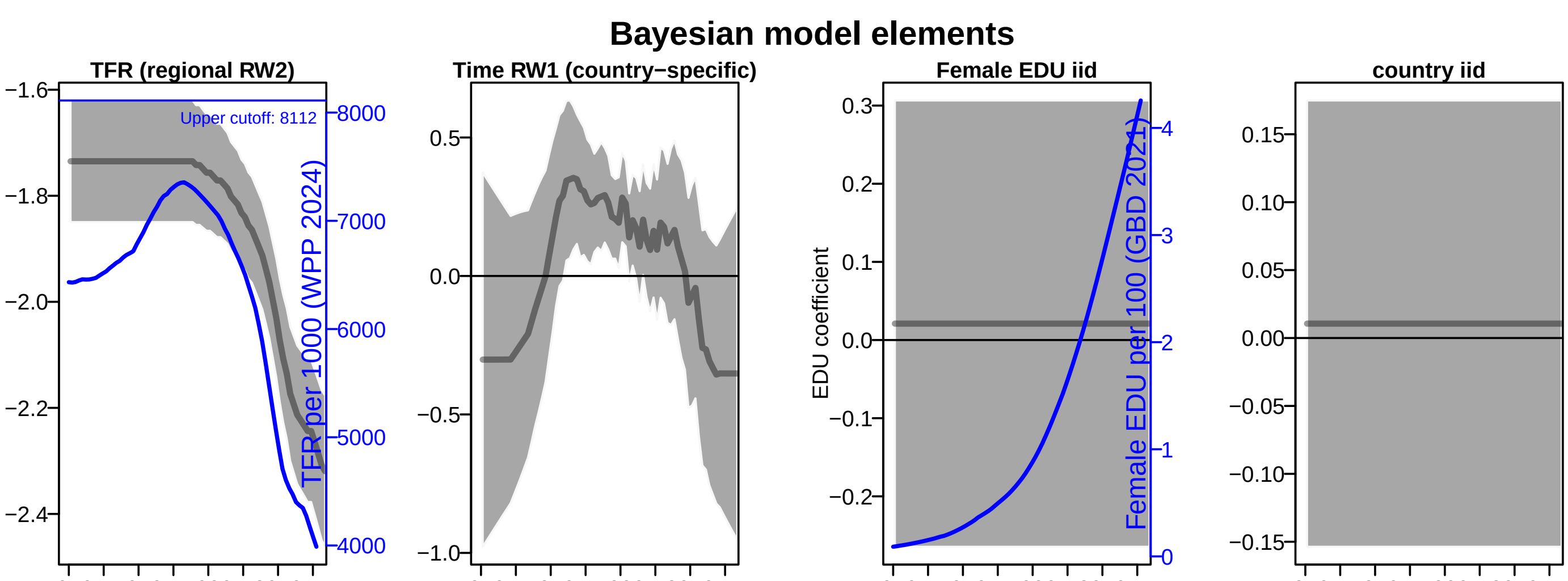
Bayesian model elements
TFR (regional RW2)
Upper cutoff: 8112
TFR per 1000 (WPP 2024)
Time RW1 (country−specific)
Female EDU iid
EDU coefficient
Female EDU per 100 (GBD 2021)
country iid

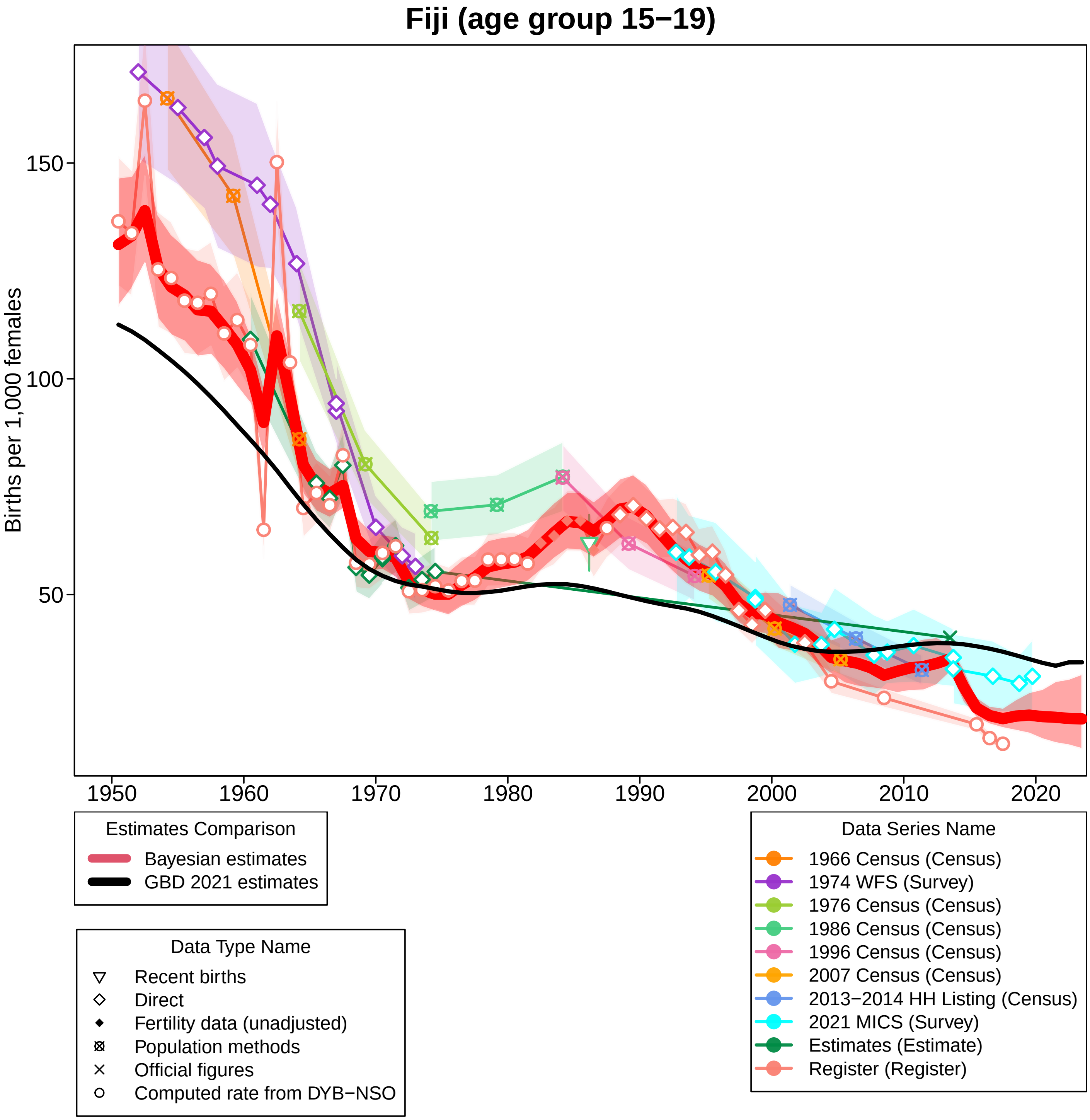

Fiji (age group 15−19)
Births per 1,000 females
150
100
50
1950
1960
1970
1980
1990
2000
2010
2020
Estimates Comparison
Bayesian estimates
GBD 2021 estimates
Data Type Name
Recent births
Direct
Fertility data (unadjusted)
Population methods
Official figures
Computed rate from DYB−NSO
Data Series Name
1966 Census (Census)
1974 WFS (Survey)
1976 Census (Census)
1986 Census (Census)
1996 Census (Census)
2007 Census (Census)
2013−2014 HH Listing (Census)
2021 MICS (Survey)
Estimates (Estimate)
Register (Register)

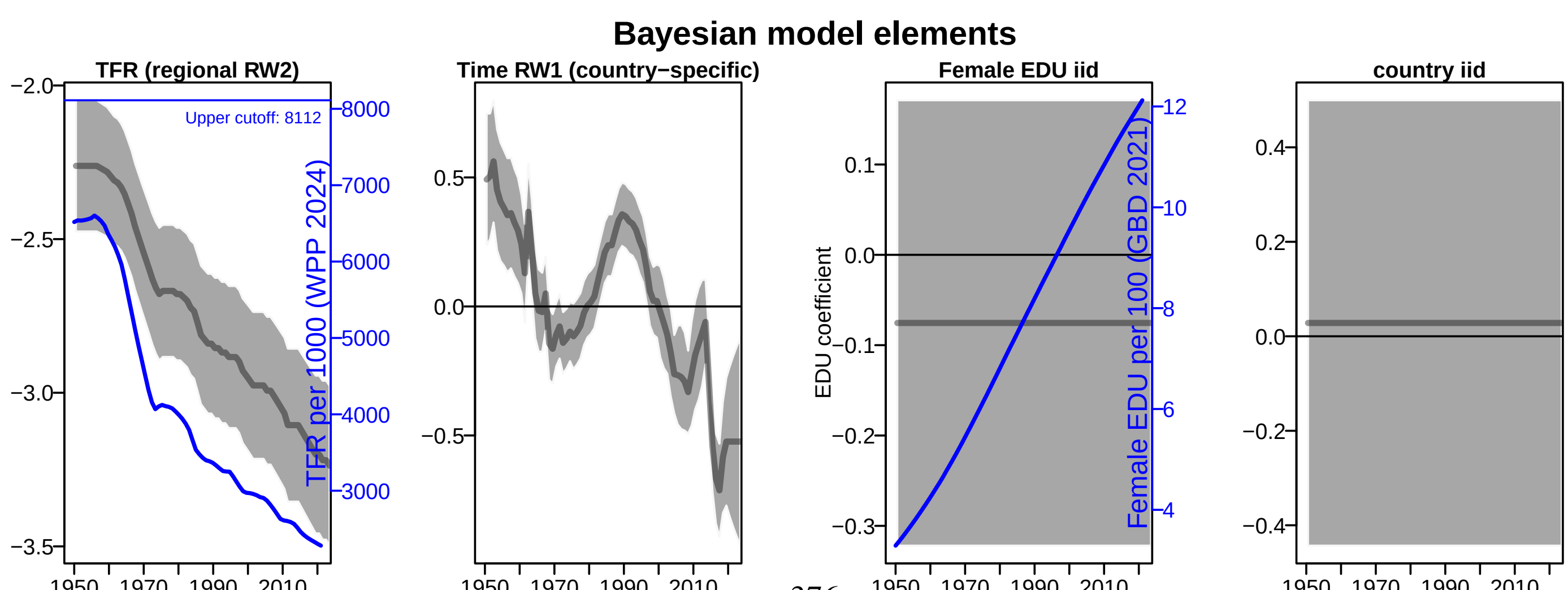

Bayesian model elements
TFR (regional RW2)
Upper cutoff: 8112
TFR per 1000 (WPP 2024)
−2.0
−2.5
−3.0
−3.5
8000
7000
6000
5000
4000
3000
Time RW1 (country−specific)
0.5
0.0
−0.5
Female EDU iid
EDU coefficient
Female EDU per 100 (GBD 2021)
0.1
0.0
−0.1
−0.2
−0.3
12
10
8
6
4
country iid
0.4
0.2
0.0
−0.2
−0.4
1950
1970
1990
2010

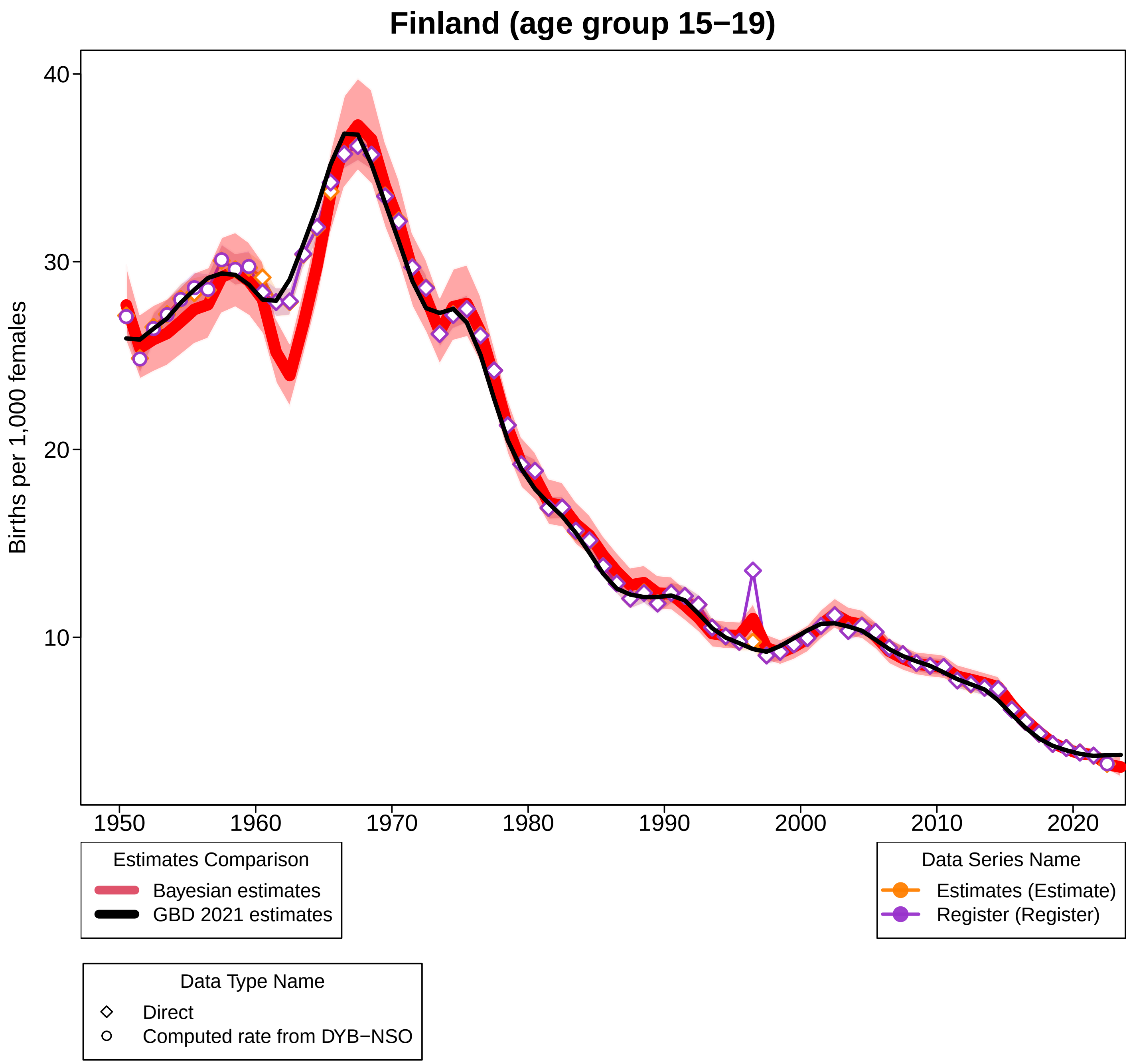
Finland (age group 15−19)
Births per 1,000 females
40
30
20
10
1950
1960
1970
1980
1990
2000
2010
2020
Estimates Comparison
Bayesian estimates
GBD 2021 estimates
Data Series Name
Estimates (Estimate)
Register (Register)
Data Type Name
Direct
Computed rate from DYB−NSO

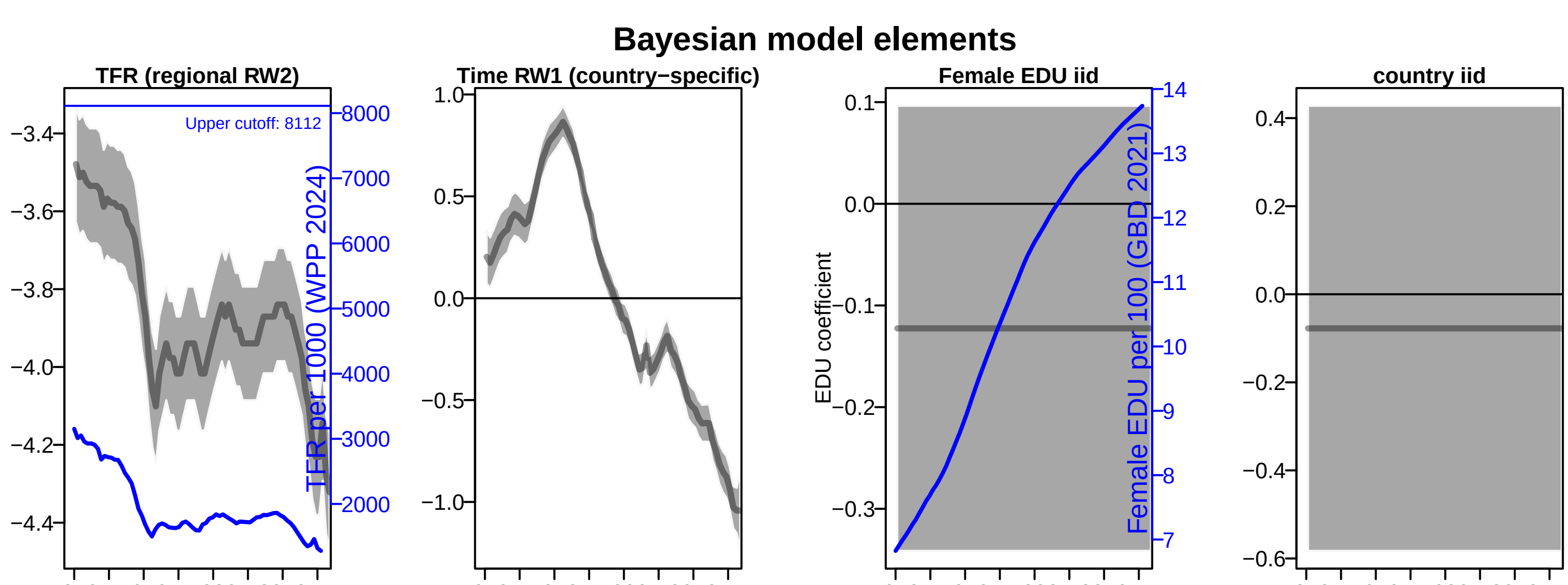
Bayesian model elements
TFR (regional RW2)
Upper cutoff: 8112
TFR per 1000 (WPP 2024)
Time RW1 (country−specific)
Female EDU iid
EDU coefficient
Female EDU per 100 (GBD 2021)
country iid

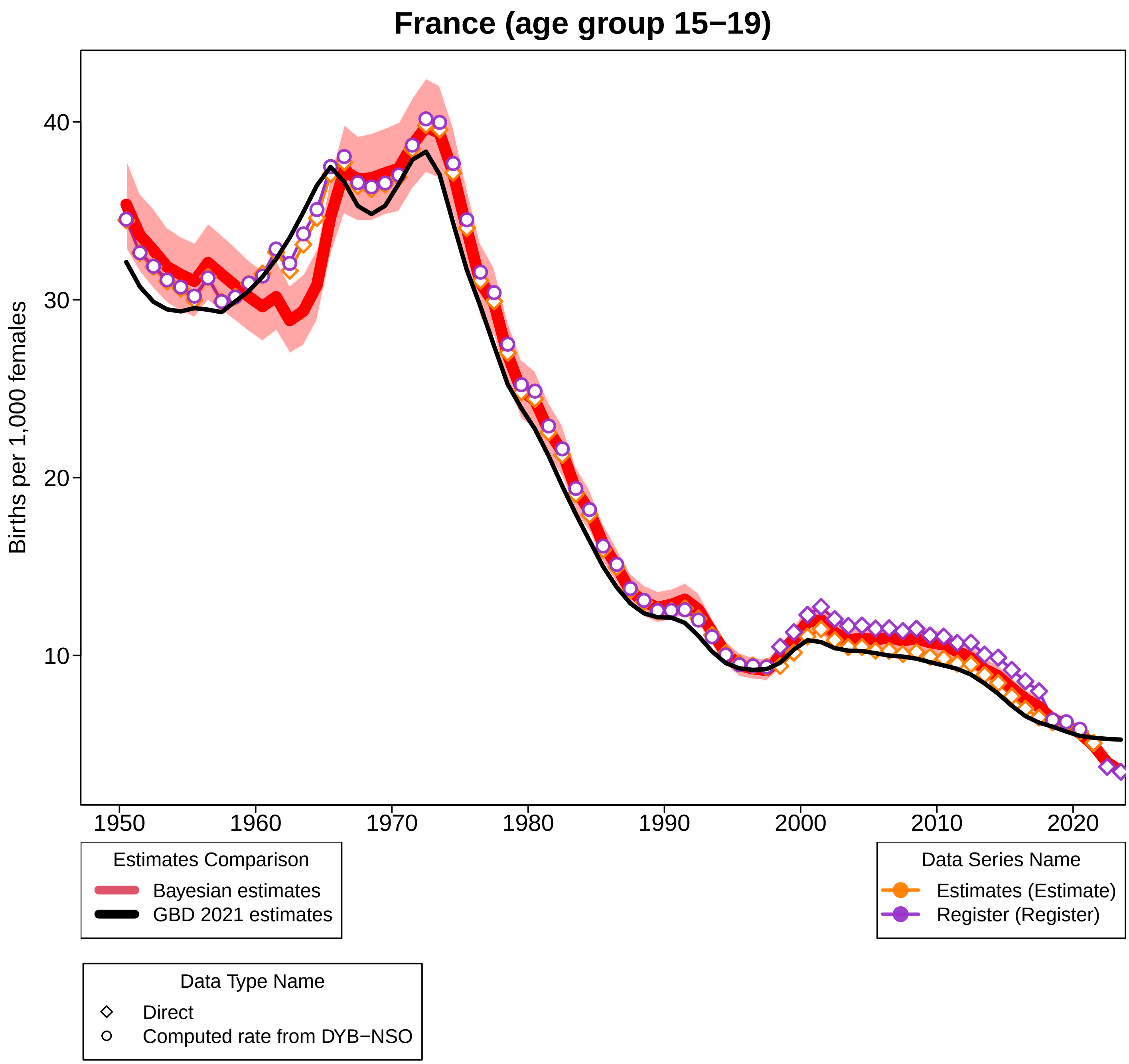
France (age group 15−19)
Births per 1,000 females
40
30
20
10
1950
1960
1970
1980
1990
2000
2010
2020
Estimates Comparison
Bayesian estimates
GBD 2021 estimates
Data Series Name
Estimates (Estimate)
Register (Register)
Data Type Name
Direct
Computed rate from DYB−NSO

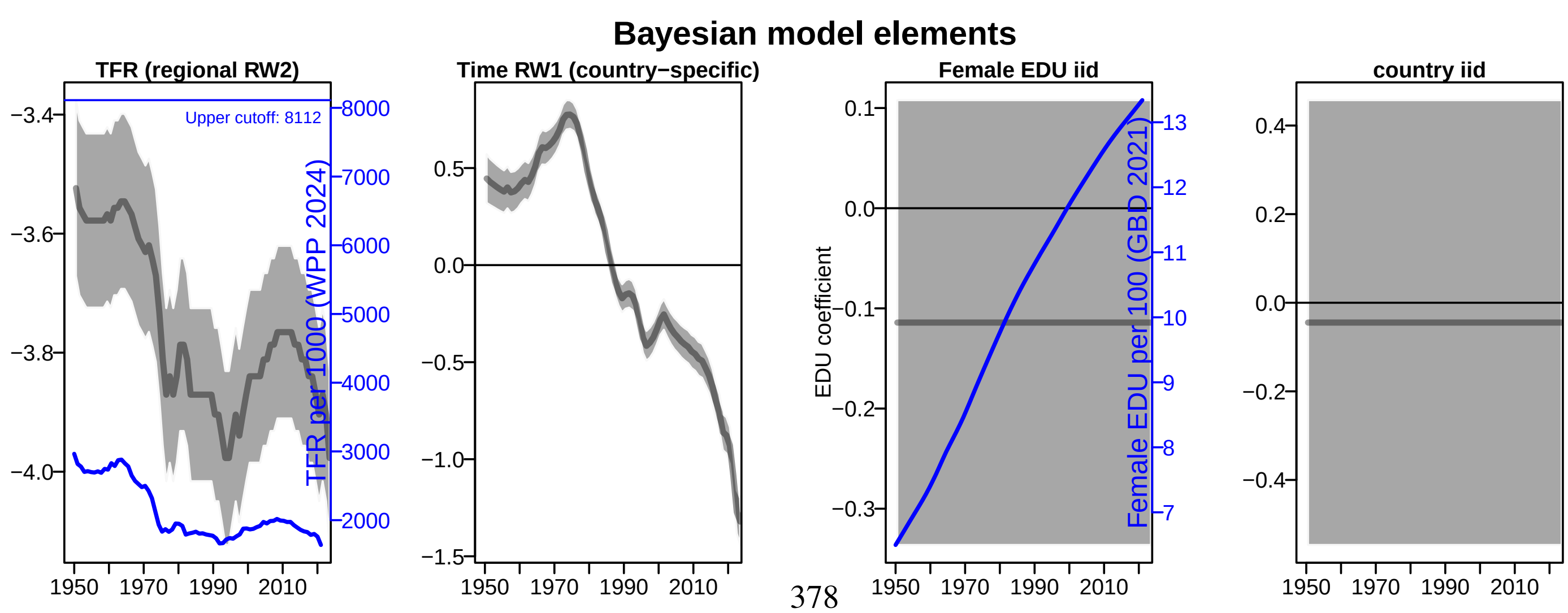
Bayesian model elements
TFR (regional RW2)
Upper cutoff: 8112
TFR per 1000 (WPP 2024)
Time RW1 (country−specific)
Female EDU iid
EDU coefficient
Female EDU per 100 (GBD 2021)
country iid

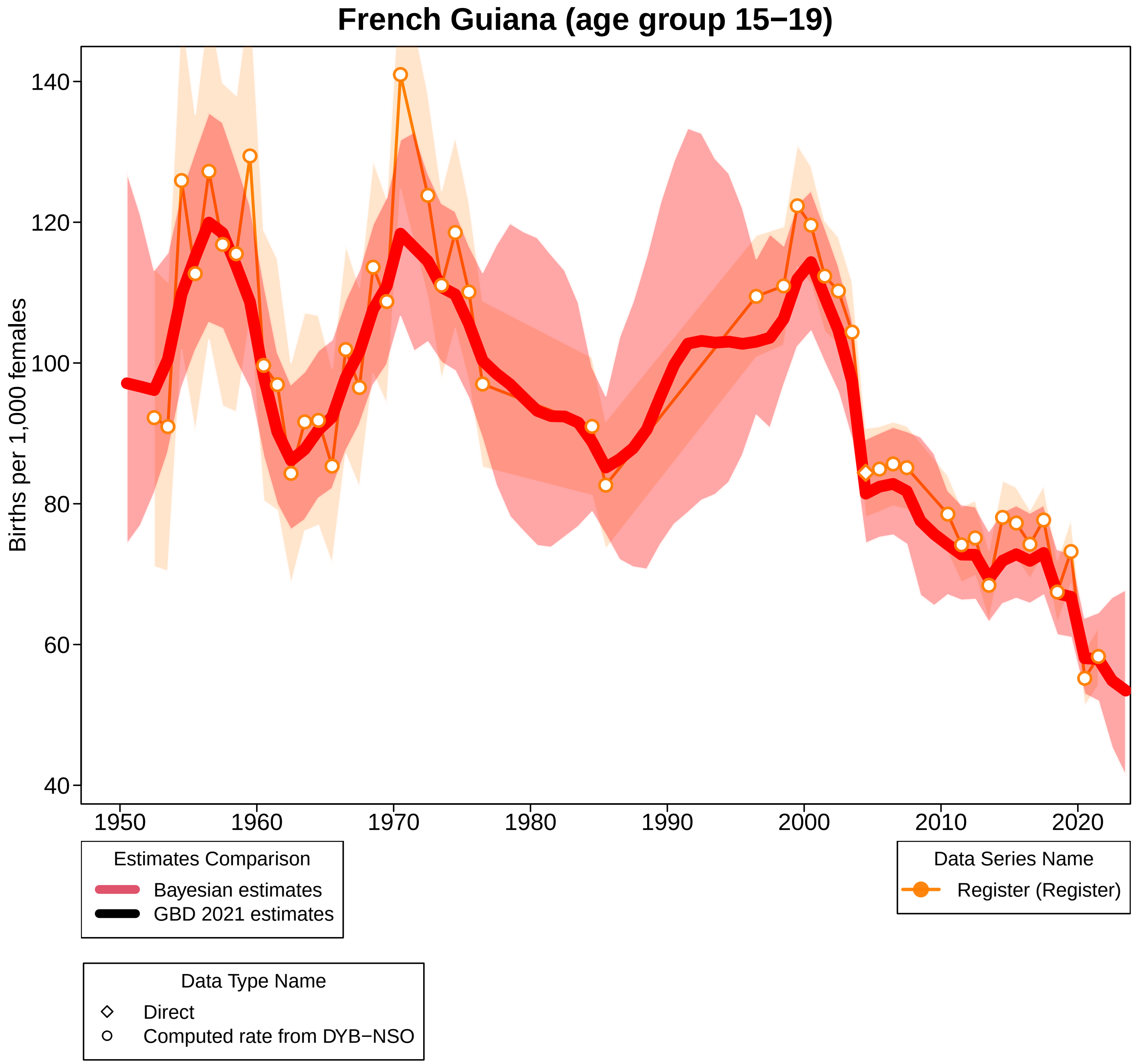
French Guiana (age group 15−19)
Births per 1,000 females
140
120
100
80
60
40
1950
1960
1970
1980
1990
2000
2010
2020
Estimates Comparison
Bayesian estimates
GBD 2021 estimates
Data Series Name
Register (Register)
Data Type Name
Direct
Computed rate from DYB−NSO

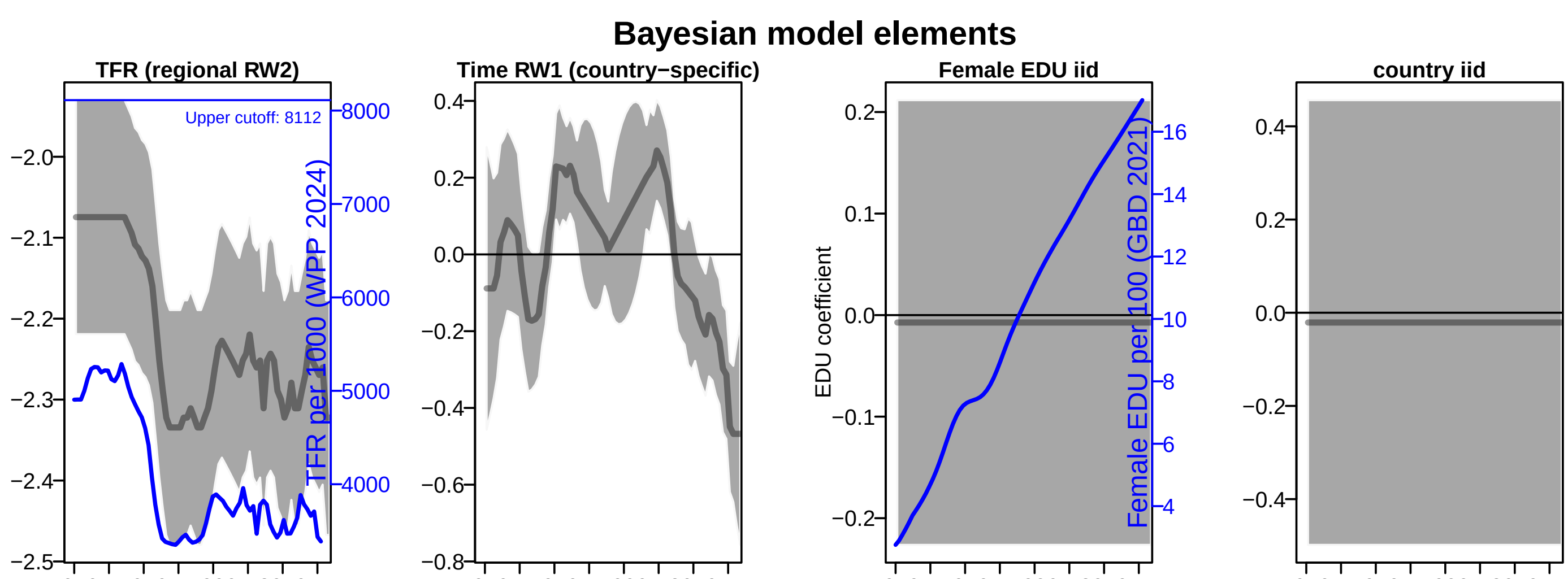
Bayesian model elements
TFR (regional RW2)
Upper cutoff: 8112
TFR per 1000 (WPP 2024)
Time RW1 (country−specific)
Female EDU iid
EDU coefficient
Female EDU per 100 (GBD 2021)
country iid
1950
1970
1990
2010

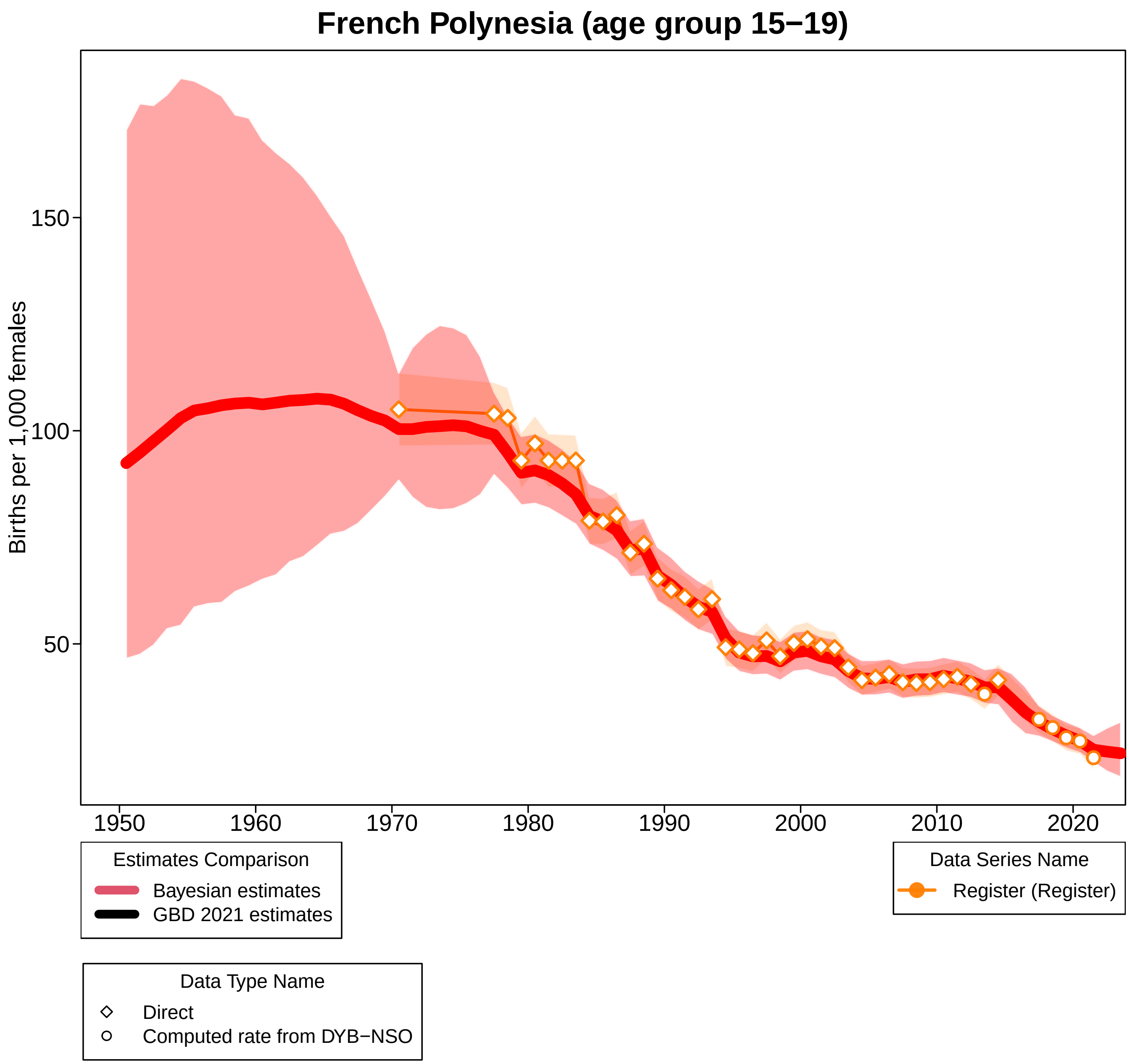
French Polynesia (age group 15−19)
Births per 1,000 females
150
100
50
1950
1960
1970
1980
1990
2000
2010
2020
Estimates Comparison
Bayesian estimates
GBD 2021 estimates
Data Series Name
Register (Register)
Data Type Name
Direct
Computed rate from DYB−NSO

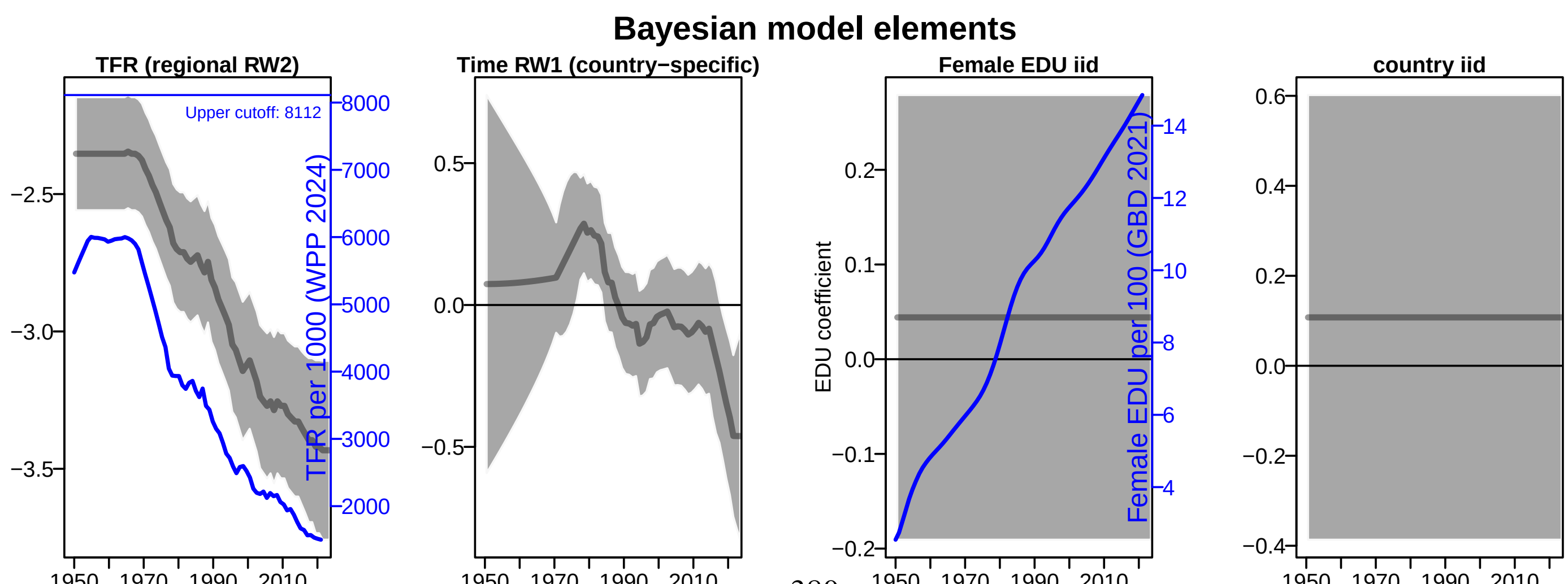
Bayesian model elements
TFR (regional RW2)
Upper cutoff: 8112
TFR per 1000 (WPP 2024)
Time RW1 (country−specific)
Female EDU iid
EDU coefficient
Female EDU per 100 (GBD 2021)
country iid

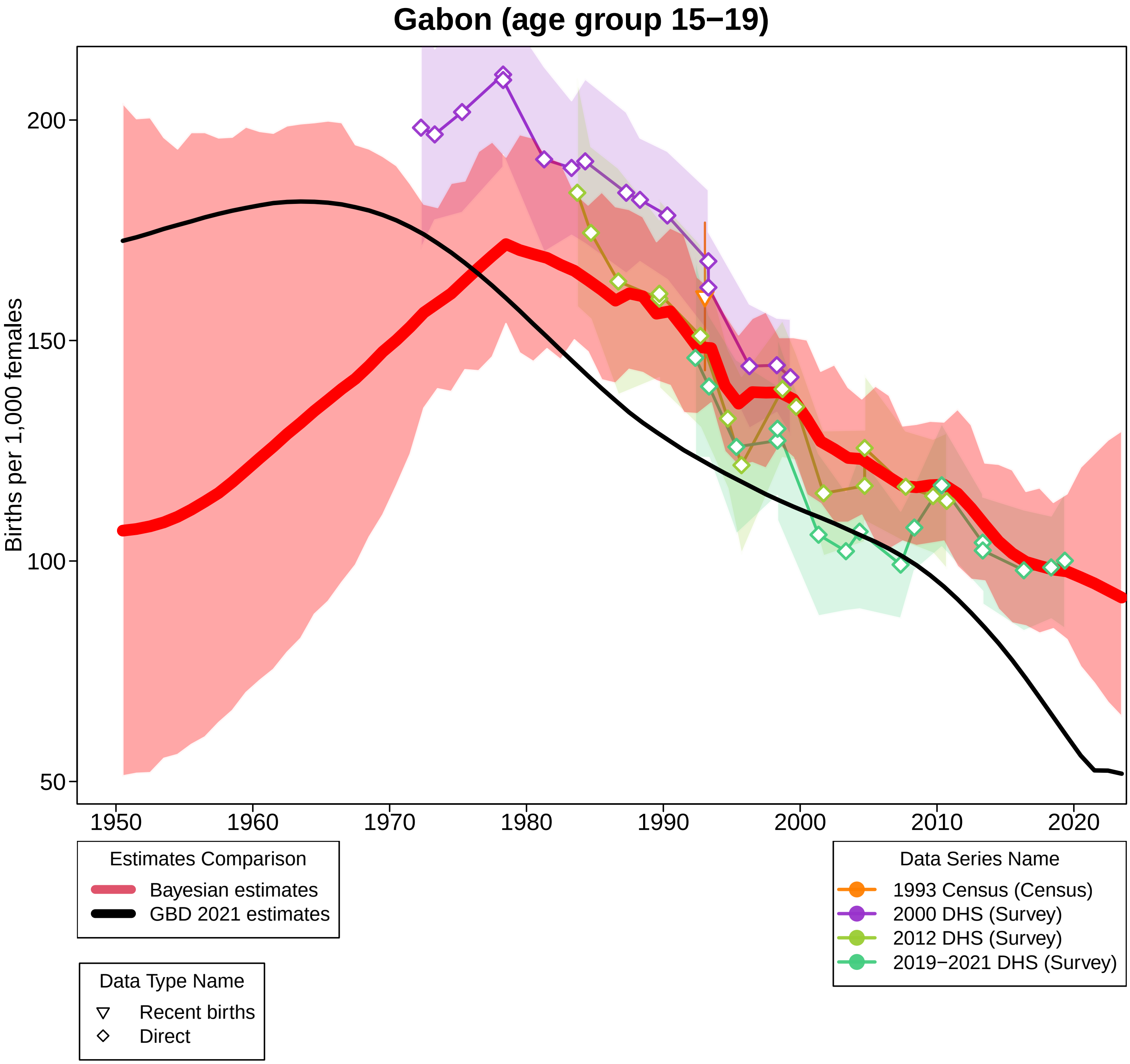

Gabon (age group 15−19)
Births per 1,000 females
200
150
100
50
1950
1960
1970
1980
1990
2000
2010
2020
Estimates Comparison
Bayesian estimates
GBD 2021 estimates
Data Series Name
1993 Census (Census)
2000 DHS (Survey)
2012 DHS (Survey)
2019−2021 DHS (Survey)
Data Type Name
Recent births
Direct

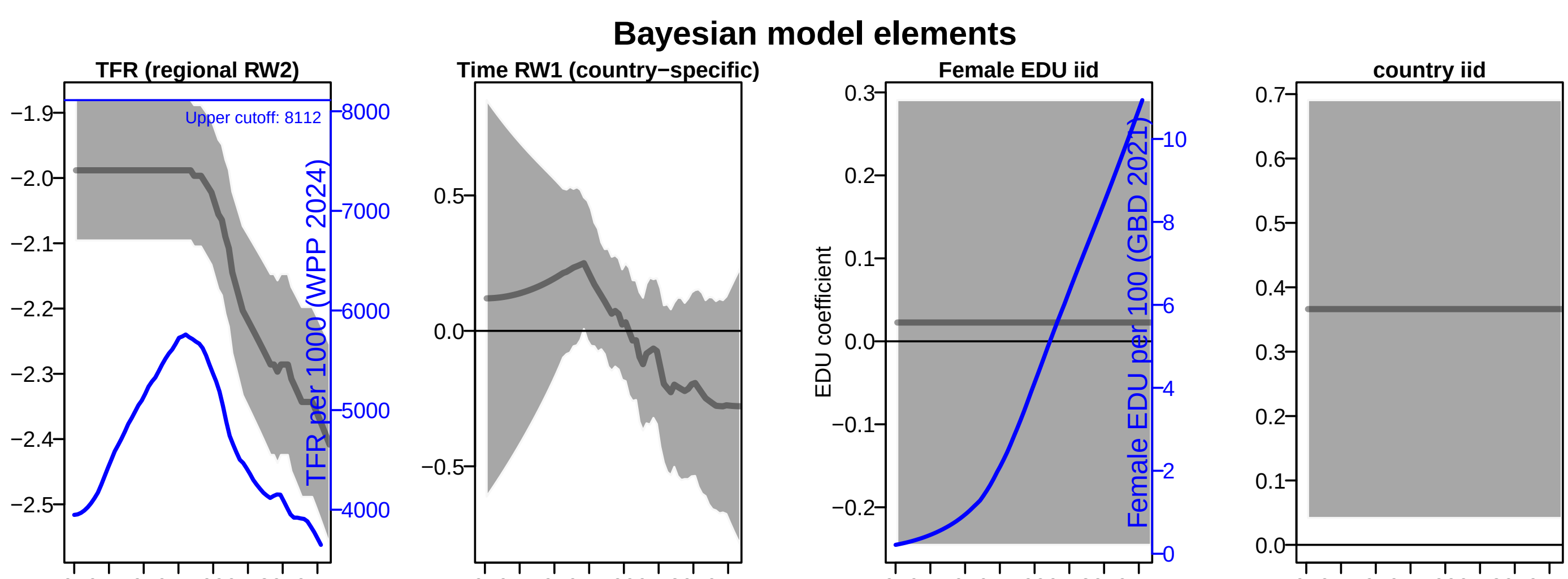

Bayesian model elements
TFR (regional RW2)
Upper cutoff: 8112
TFR per 1000 (WPP 2024)
Time RW1 (country−specific)
Female EDU iid
EDU coefficient
Female EDU per 100 (GBD 2021)
country iid

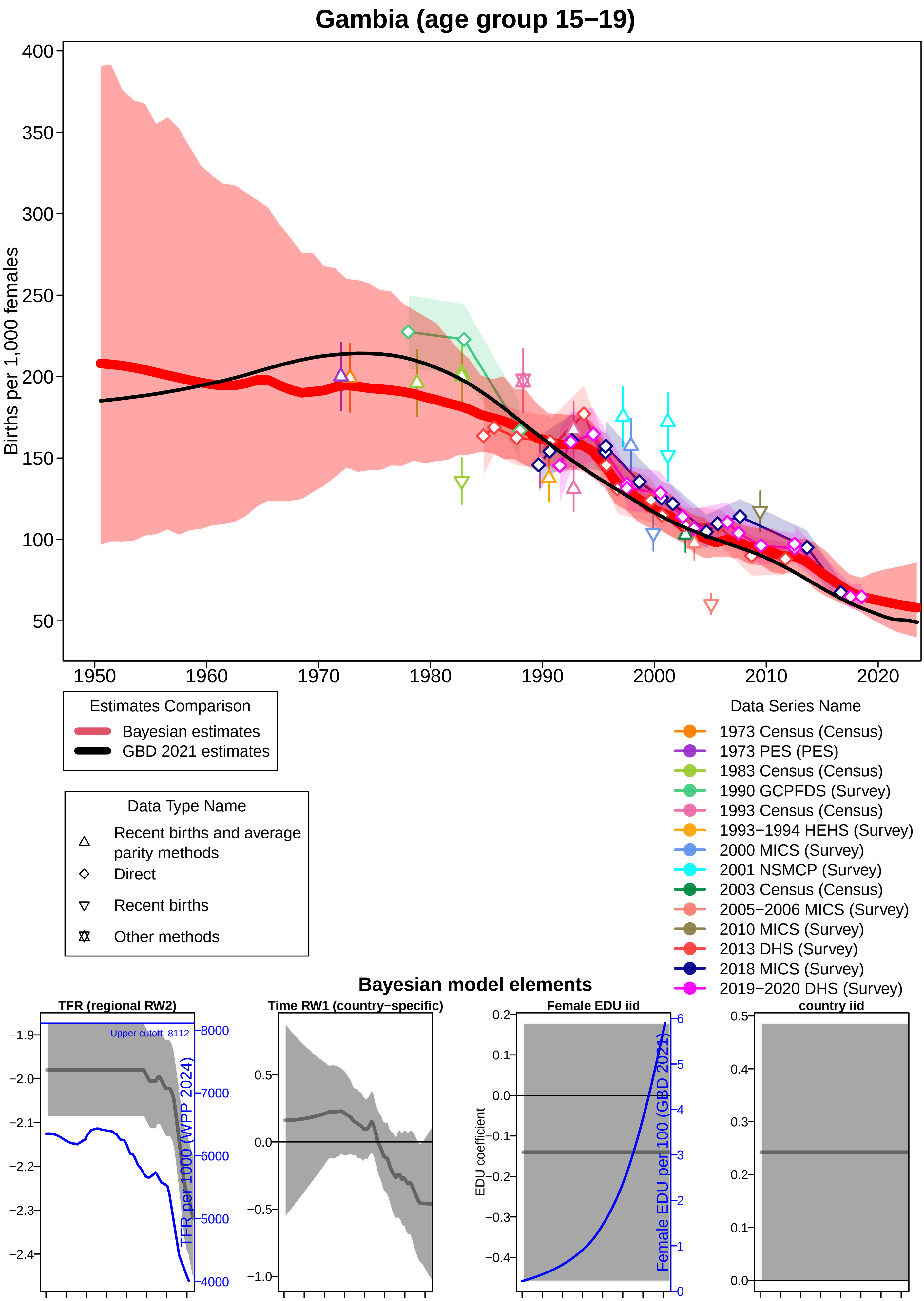

Gambia (age group 15−19)
Births per 1,000 females
400
350
300
250
200
150
100
50
1950
1960
1970
1980
1990
2000
2010
2020
Estimates Comparison
Bayesian estimates
GBD 2021 estimates
Data Type Name
Recent births and average parity methods
Direct
Recent births
Other methods
Data Series Name
1973 Census (Census)
1973 PES (PES)
1983 Census (Census)
1990 GCPFDS (Survey)
1993 Census (Census)
1993−1994 HEHS (Survey)
2000 MICS (Survey)
2001 NSMCP (Survey)
2003 Census (Census)
2005−2006 MICS (Survey)
2010 MICS (Survey)
2013 DHS (Survey)
2018 MICS (Survey)
2019−2020 DHS (Survey)
Bayesian model elements
TFR (regional RW2)
Upper cutoff: 8112
TFR per 1000 (WPP 2024)
Time RW1 (country−specific)
Female EDU iid
EDU coefficient
Female EDU per 100 (GBD 2021)
country iid

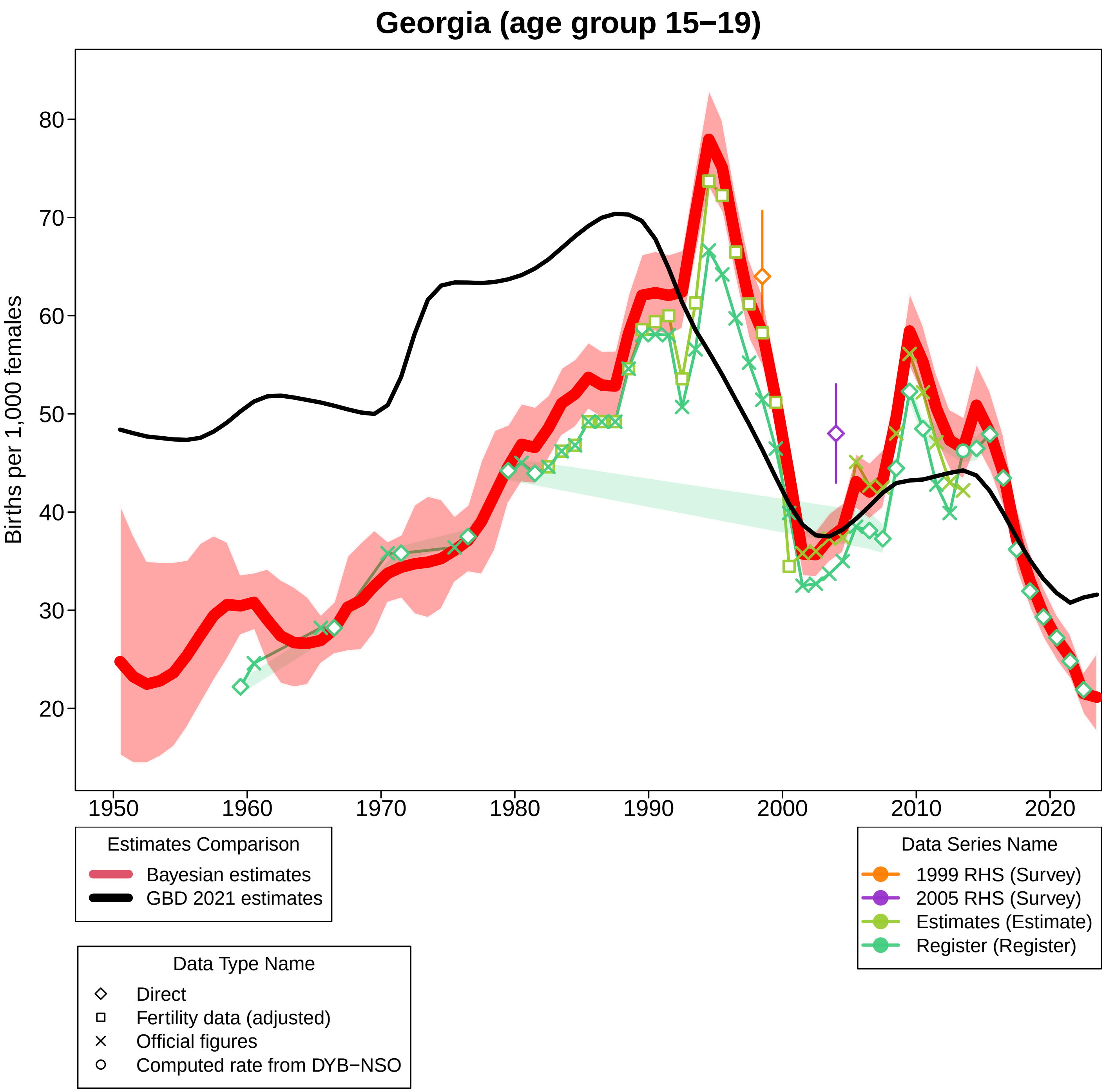
Georgia (age group 15−19)
Births per 1,000 females
80
70
60
50
40
30
20
1950
1960
1970
1980
1990
2000
2010
2020
Estimates Comparison
Bayesian estimates
GBD 2021 estimates
Data Series Name
1999 RHS (Survey)
2005 RHS (Survey)
Estimates (Estimate)
Register (Register)
Data Type Name
Direct
Fertility data (adjusted)
Official figures
Computed rate from DYB−NSO

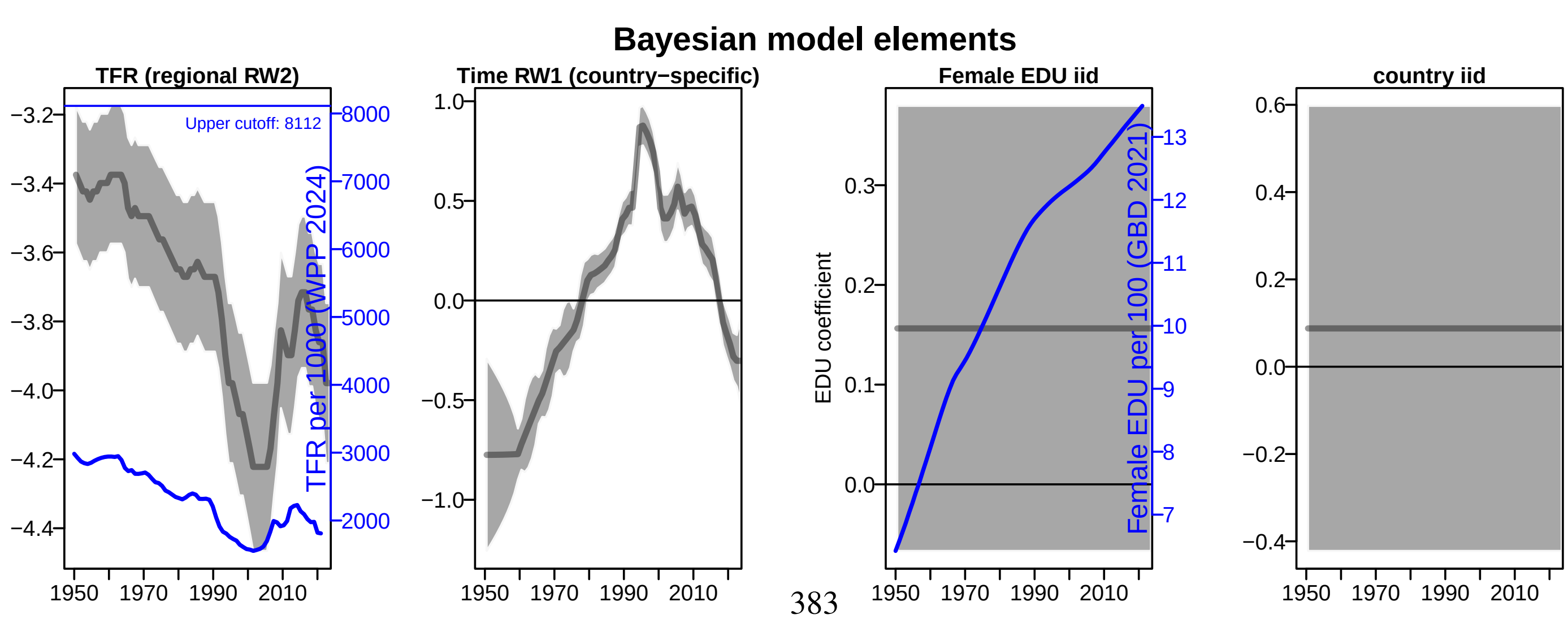
Bayesian model elements
TFR (regional RW2)
Upper cutoff: 8112
TFR per 1000 (WPP 2024)
Time RW1 (country−specific)
Female EDU iid
EDU coefficient
Female EDU per 100 (GBD 2021)
country iid

Germany (age group 15−19)

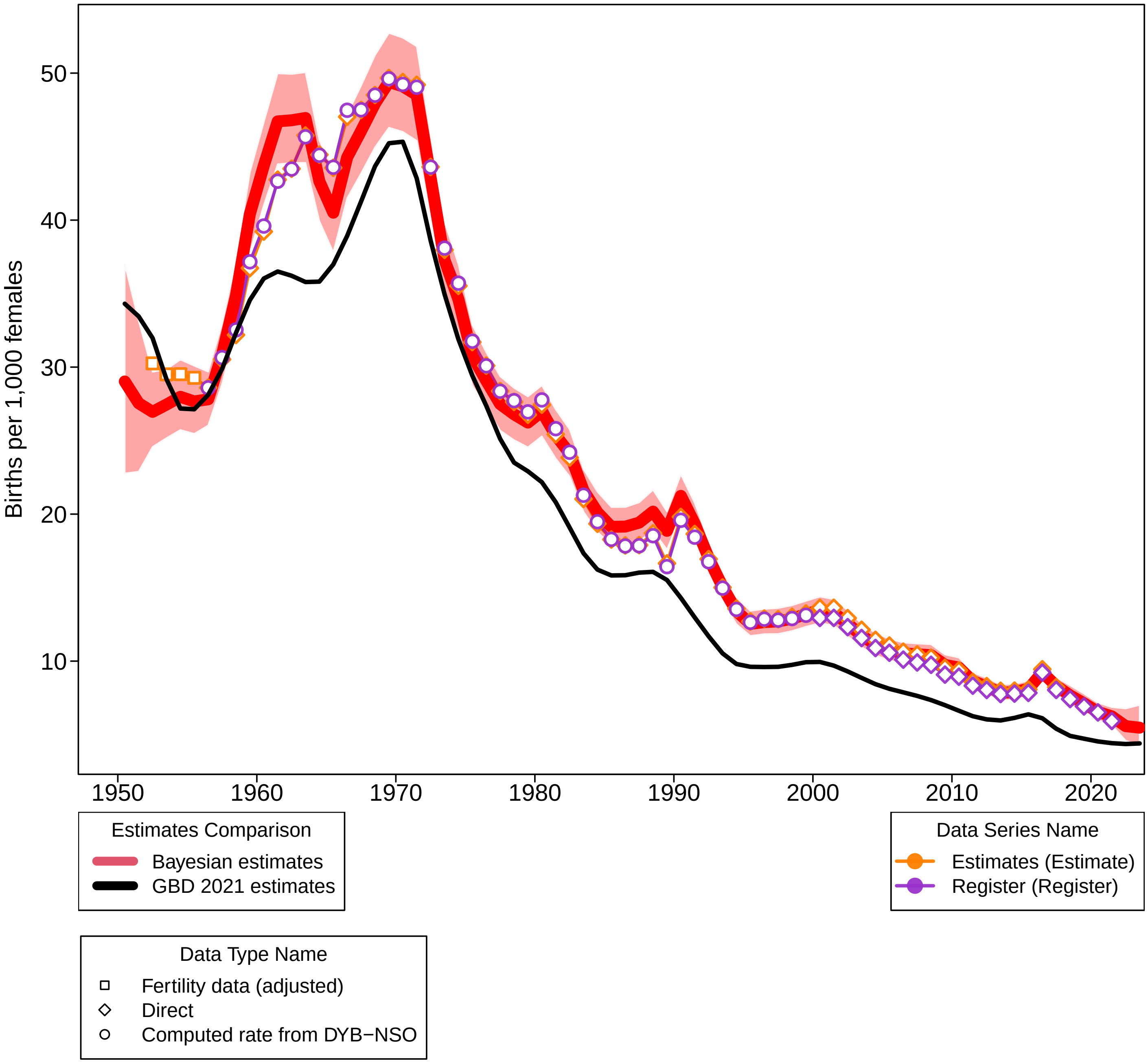

Bayesian model elements

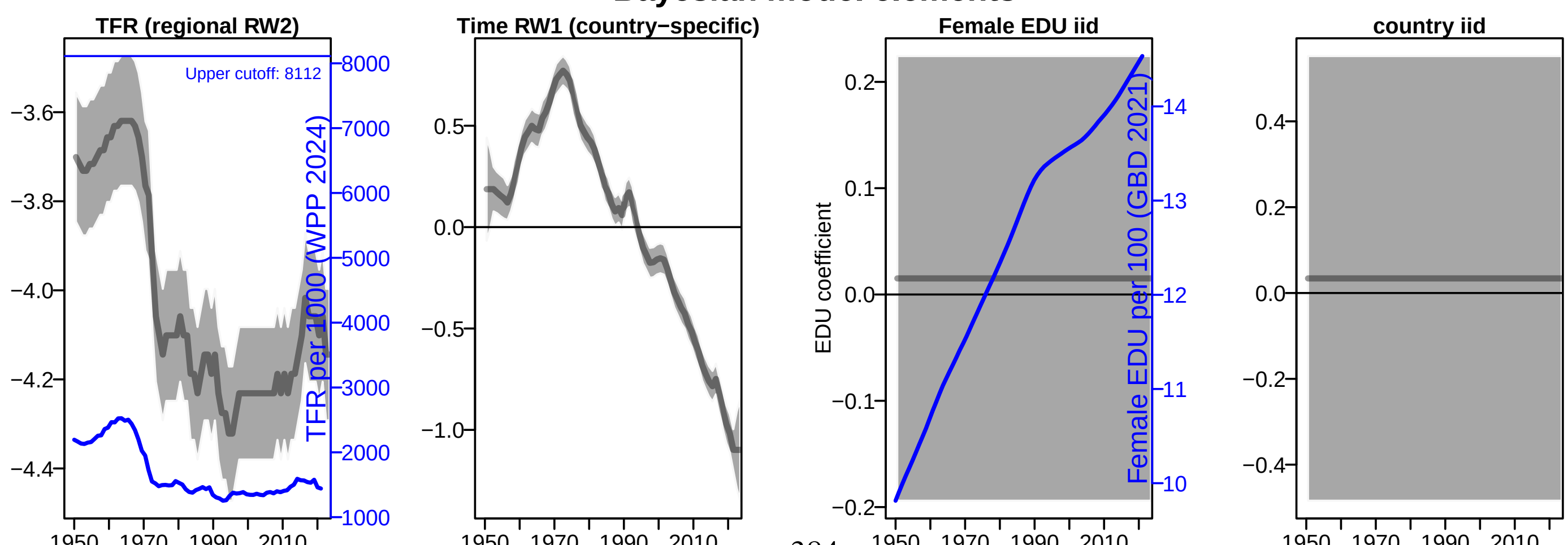

Ghana (age group 15−19)

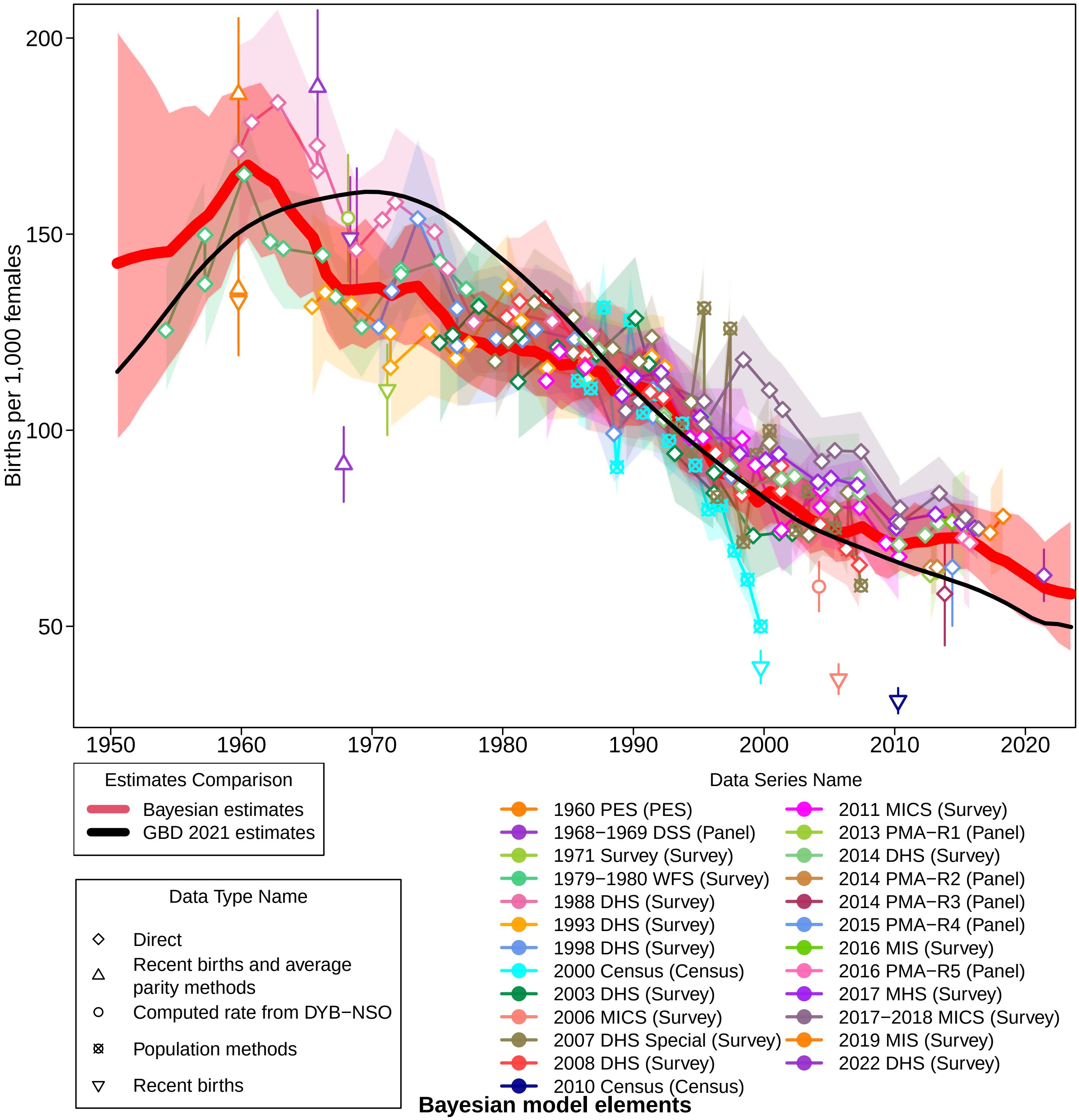

Bayesian model elements

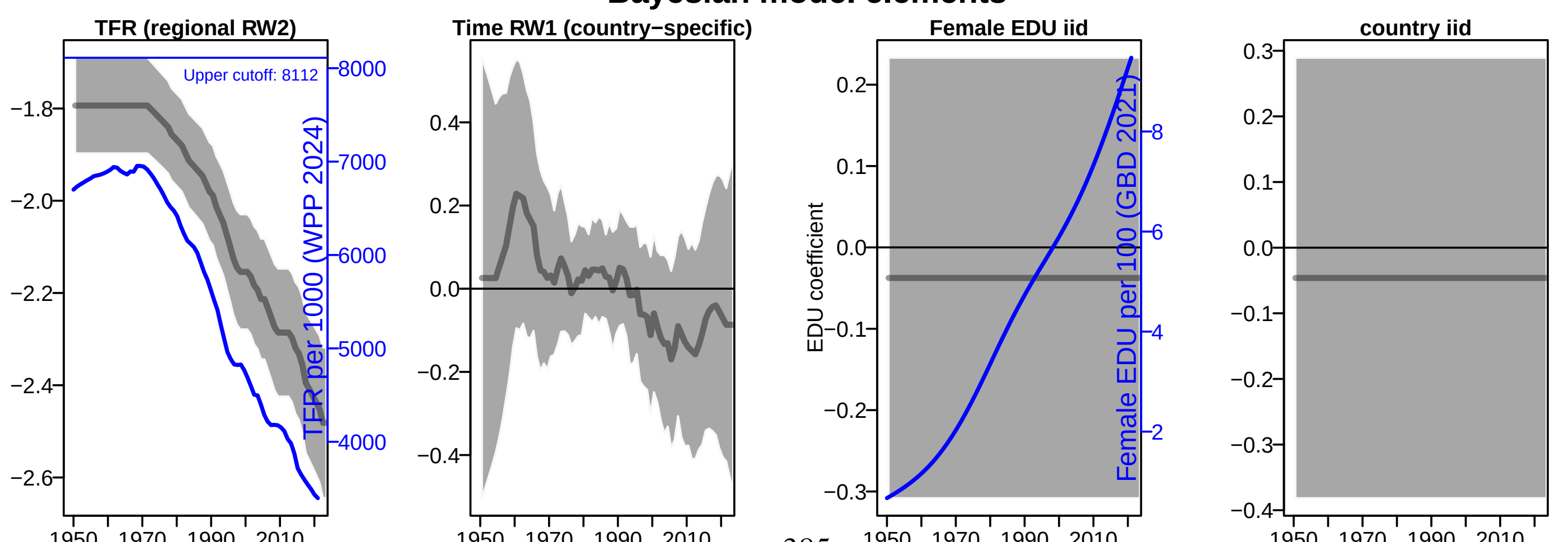

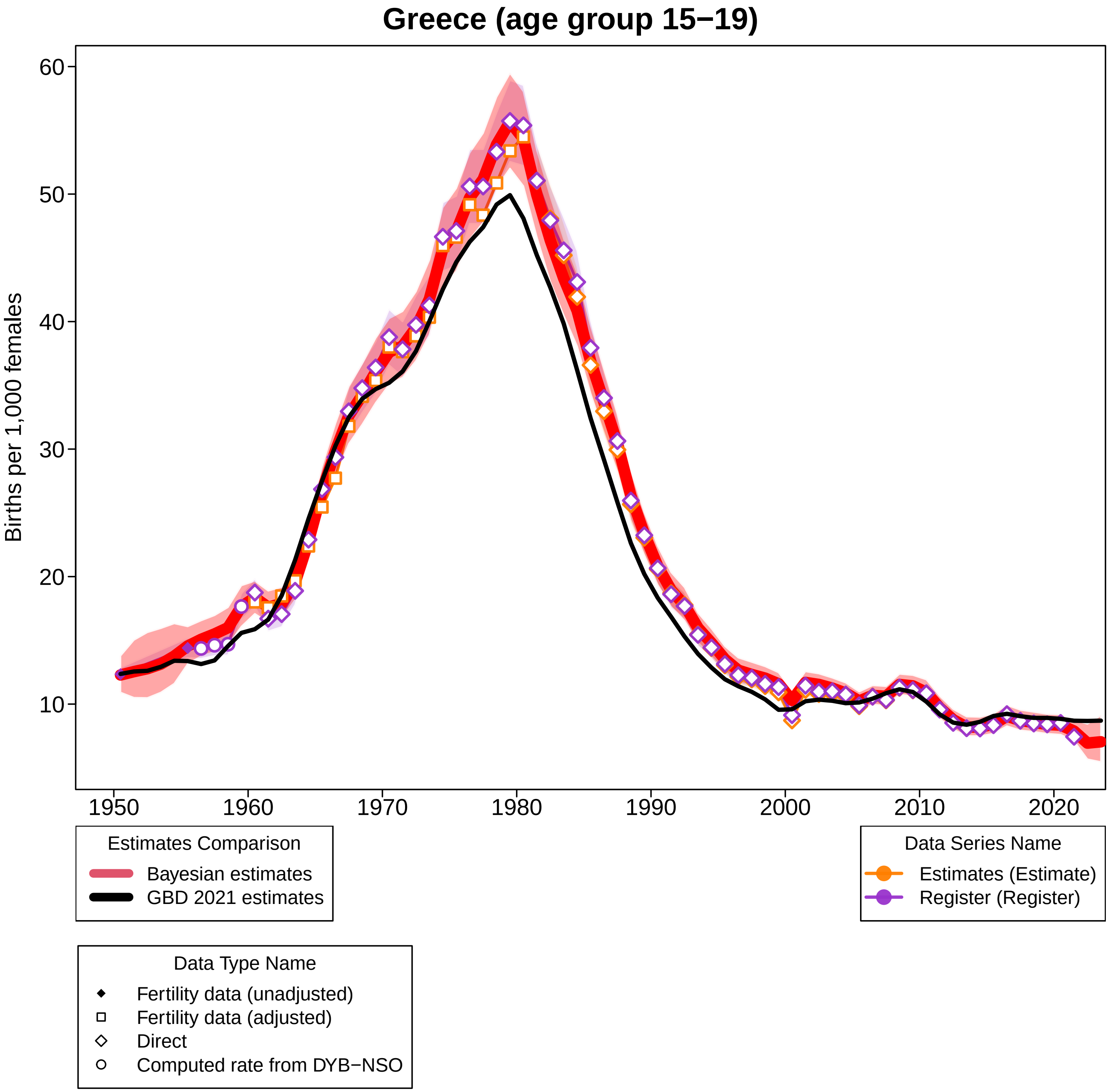
Greece (age group 15−19)
Births per 1,000 females
60
50
40
30
20
10
1950
1960
1970
1980
1990
2000
2010
2020
Estimates Comparison
Bayesian estimates
GBD 2021 estimates
Data Series Name
Estimates (Estimate)
Register (Register)
Data Type Name
Fertility data (unadjusted)
Fertility data (adjusted)
Direct
Computed rate from DYB−NSO

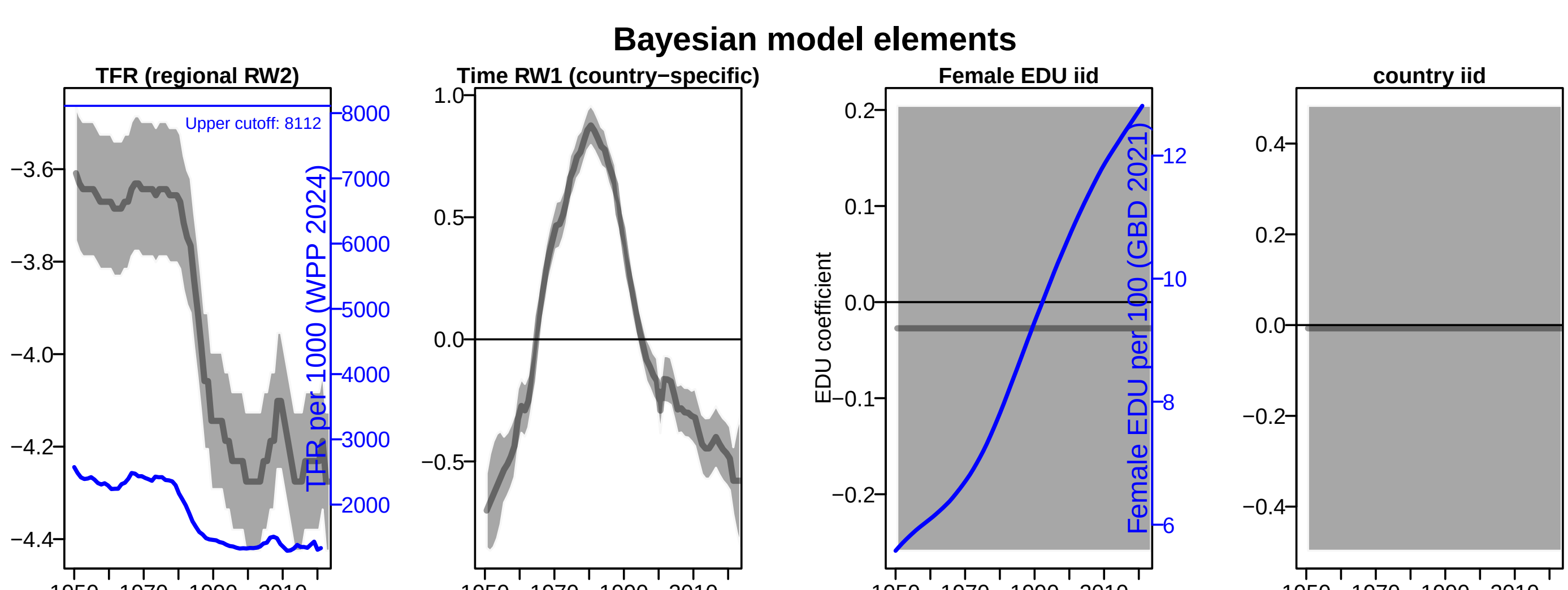
Bayesian model elements
TFR (regional RW2)
Upper cutoff: 8112
TFR per 1000 (WPP 2024)
Time RW1 (country−specific)
Female EDU iid
EDU coefficient
Female EDU per 100 (GBD 2021)
country iid

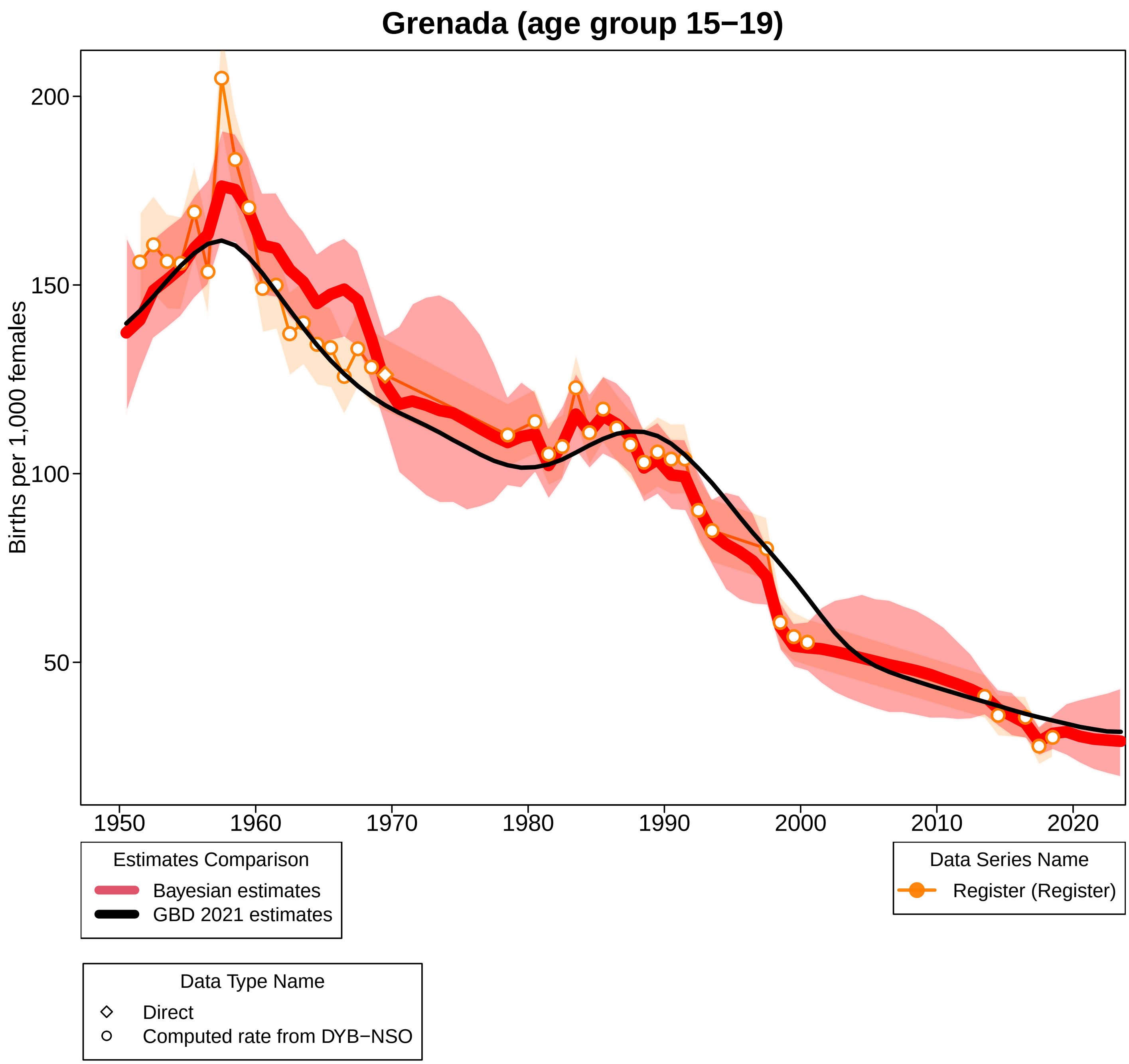

Grenada (age group 15−19)
Births per 1,000 females
200
150
100
50
1950
1960
1970
1980
1990
2000
2010
2020
Estimates Comparison
Bayesian estimates
GBD 2021 estimates
Data Series Name
Register (Register)
Data Type Name
Direct
Computed rate from DYB−NSO

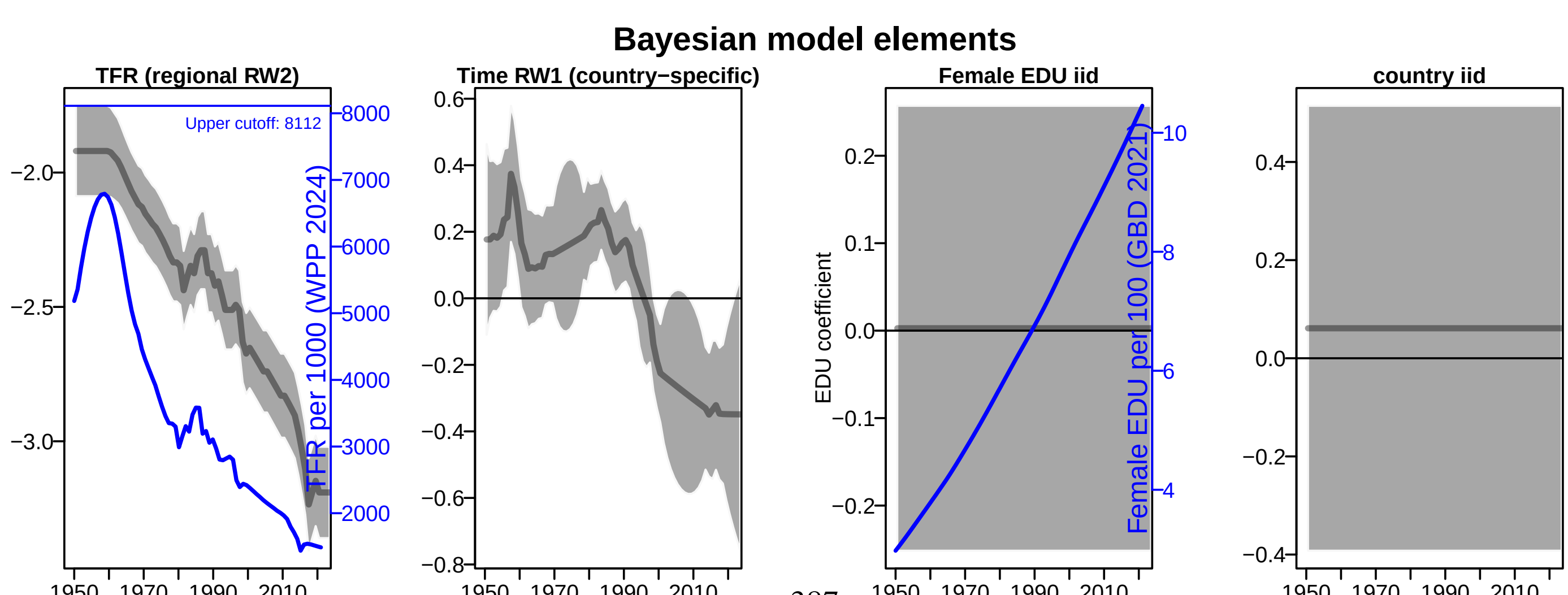

Bayesian model elements
TFR (regional RW2)
Upper cutoff: 8112
TFR per 1000 (WPP 2024)
Time RW1 (country−specific)
Female EDU iid
EDU coefficient
Female EDU per 100 (GBD 2021)
country iid

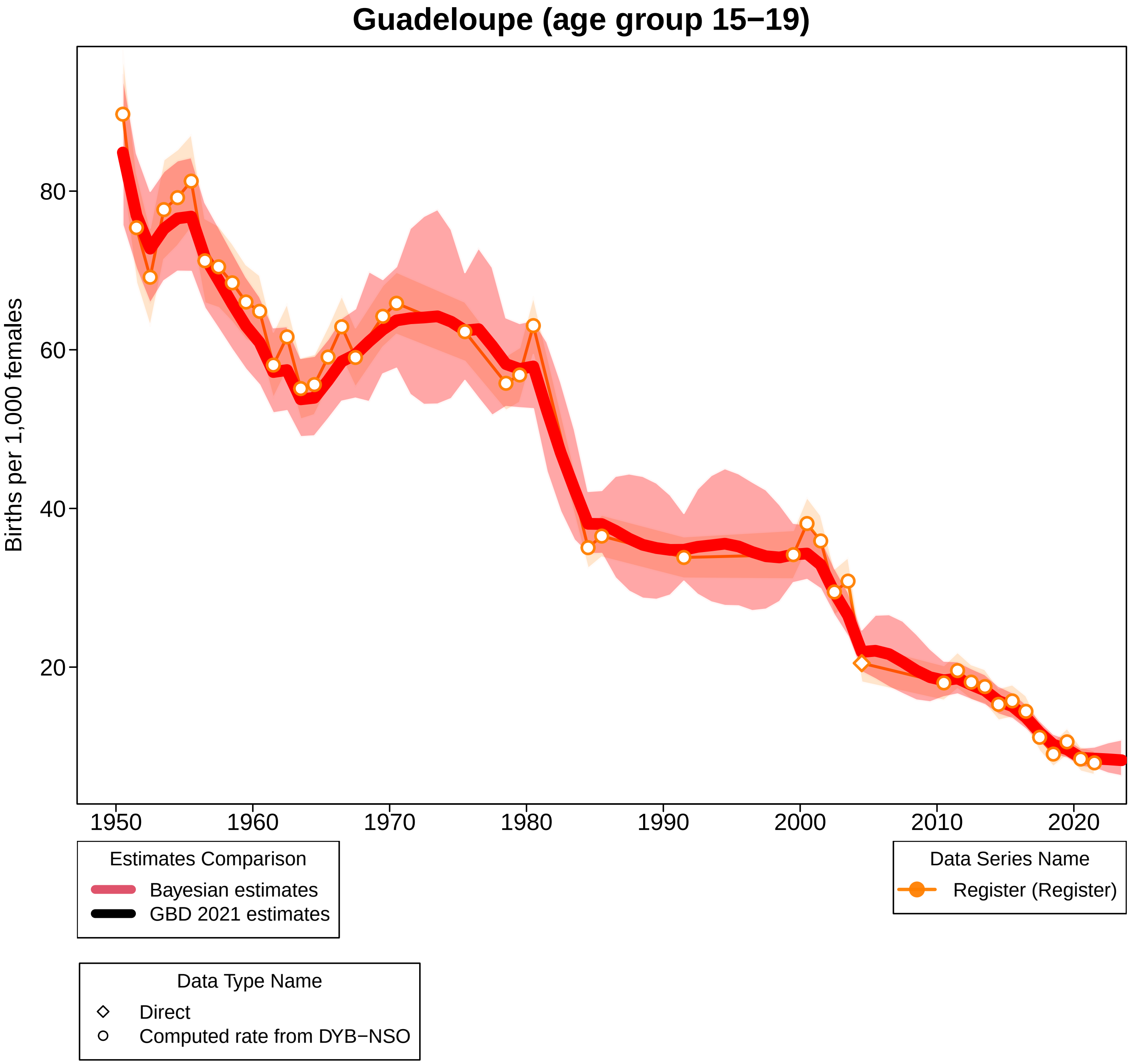
Guadeloupe (age group 15−19)
Births per 1,000 females
80
60
40
20
1950
1960
1970
1980
1990
2000
2010
2020
Estimates Comparison
Bayesian estimates
GBD 2021 estimates
Data Series Name
Register (Register)
Data Type Name
Direct
Computed rate from DYB−NSO

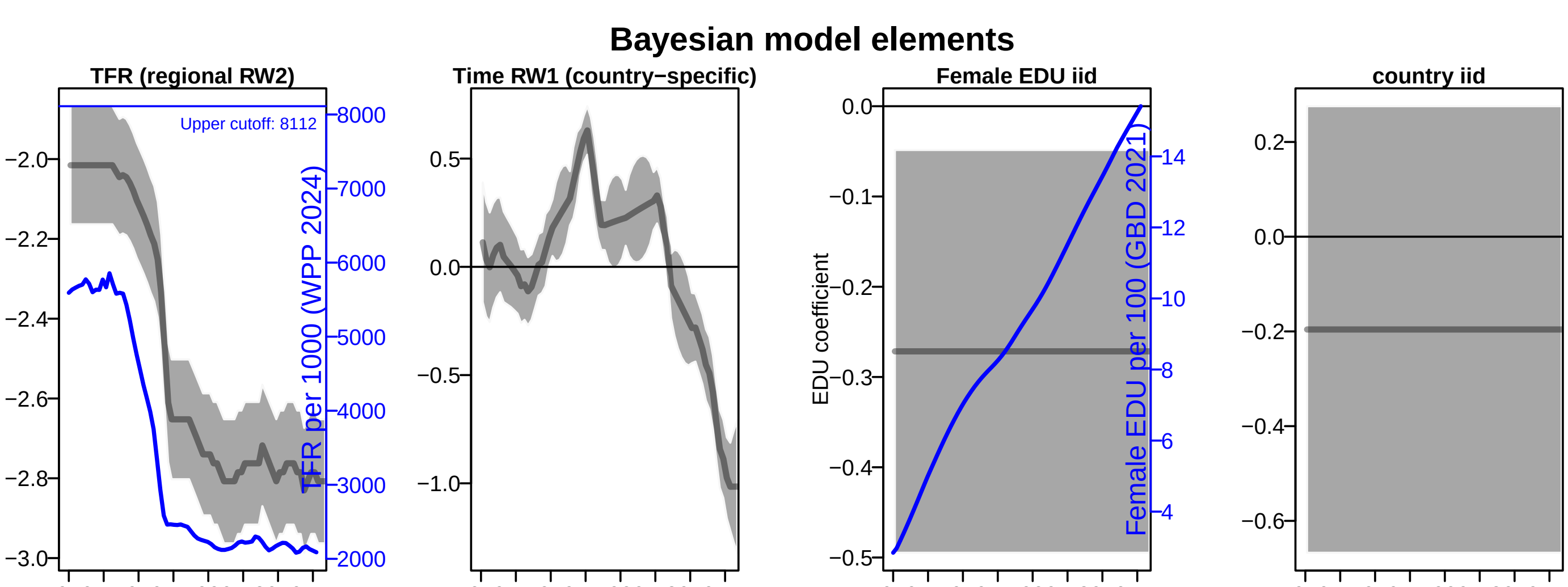
Bayesian model elements
TFR (regional RW2)
Upper cutoff: 8112
TFR per 1000 (WPP 2024)
Time RW1 (country−specific)
Female EDU iid
EDU coefficient
Female EDU per 100 (GBD 2021)
country iid

Guam (age group 15−19)

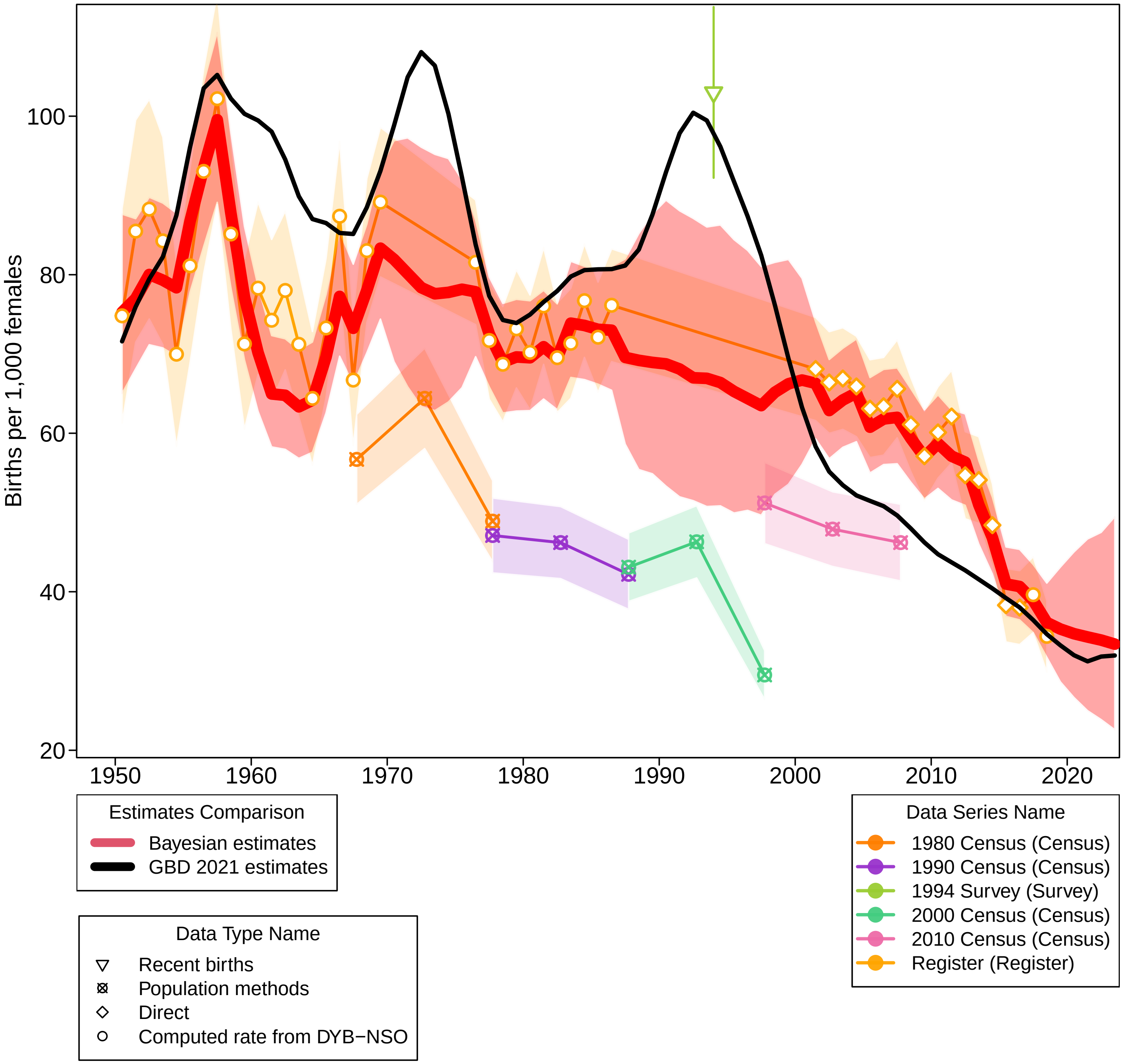

Bayesian model elements

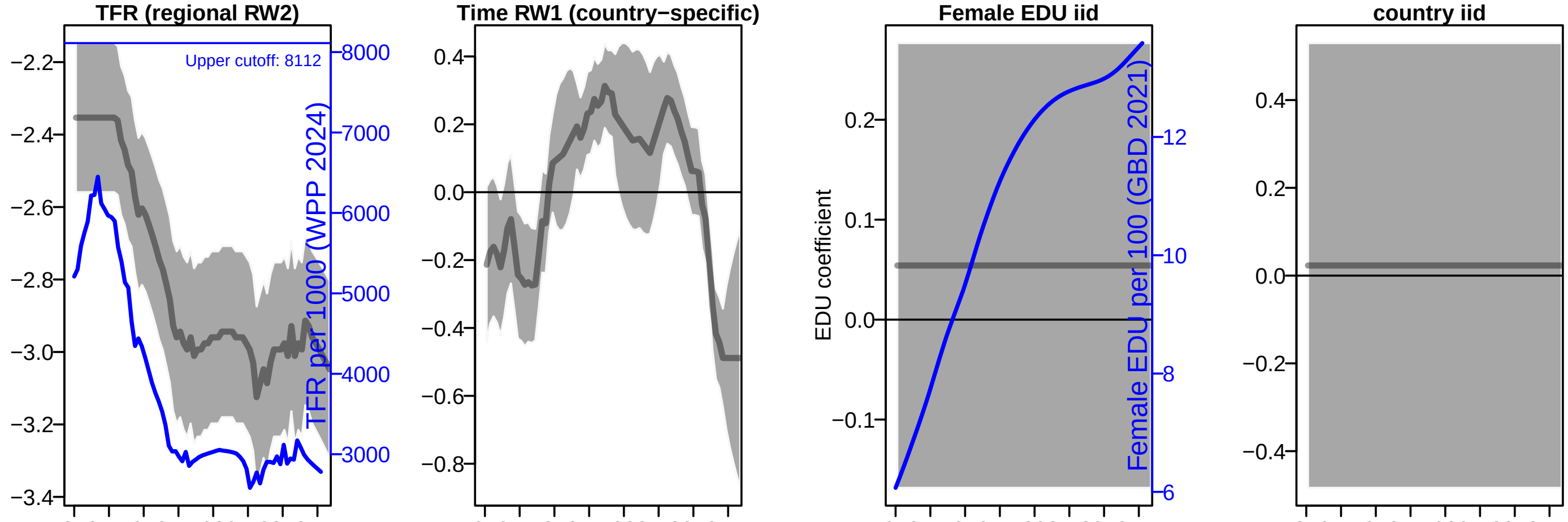

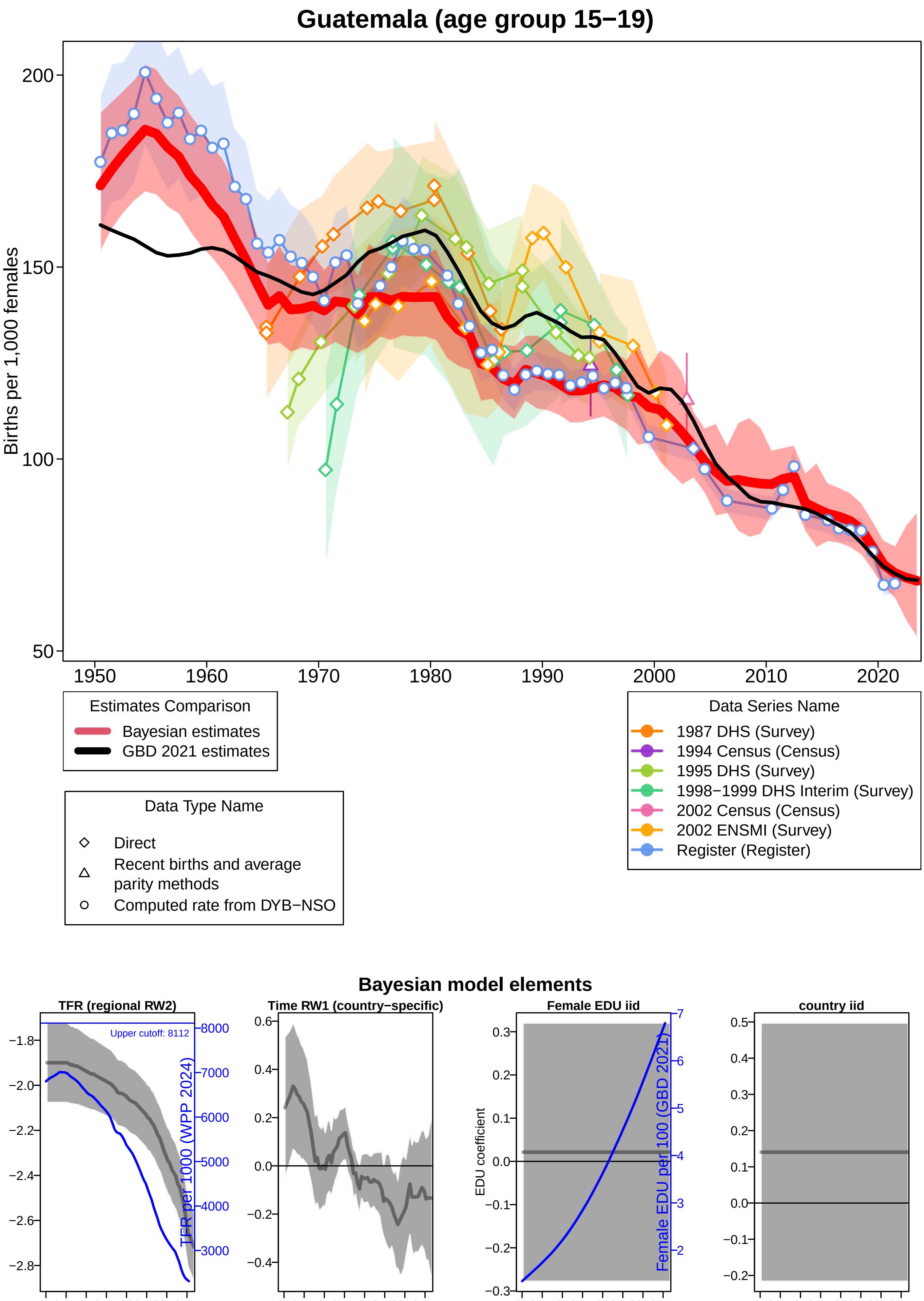
Guatemala (age group 15−19)
Births per 1,000 females
200
150
100
50
1950
1960
1970
1980
1990
2000
2010
2020
Estimates Comparison
Bayesian estimates
GBD 2021 estimates
Data Series Name
1987 DHS (Survey)
1994 Census (Census)
1995 DHS (Survey)
1998−1999 DHS Interim (Survey)
2002 Census (Census)
2002 ENSMI (Survey)
Register (Register)
Data Type Name
Direct
Recent births and average parity methods
Computed rate from DYB−NSO
Bayesian model elements
TFR (regional RW2)
Upper cutoff: 8112
TFR per 1000 (WPP 2024)
Time RW1 (country−specific)
Female EDU iid
EDU coefficient
Female EDU per 100 (GBD 2021)
country iid

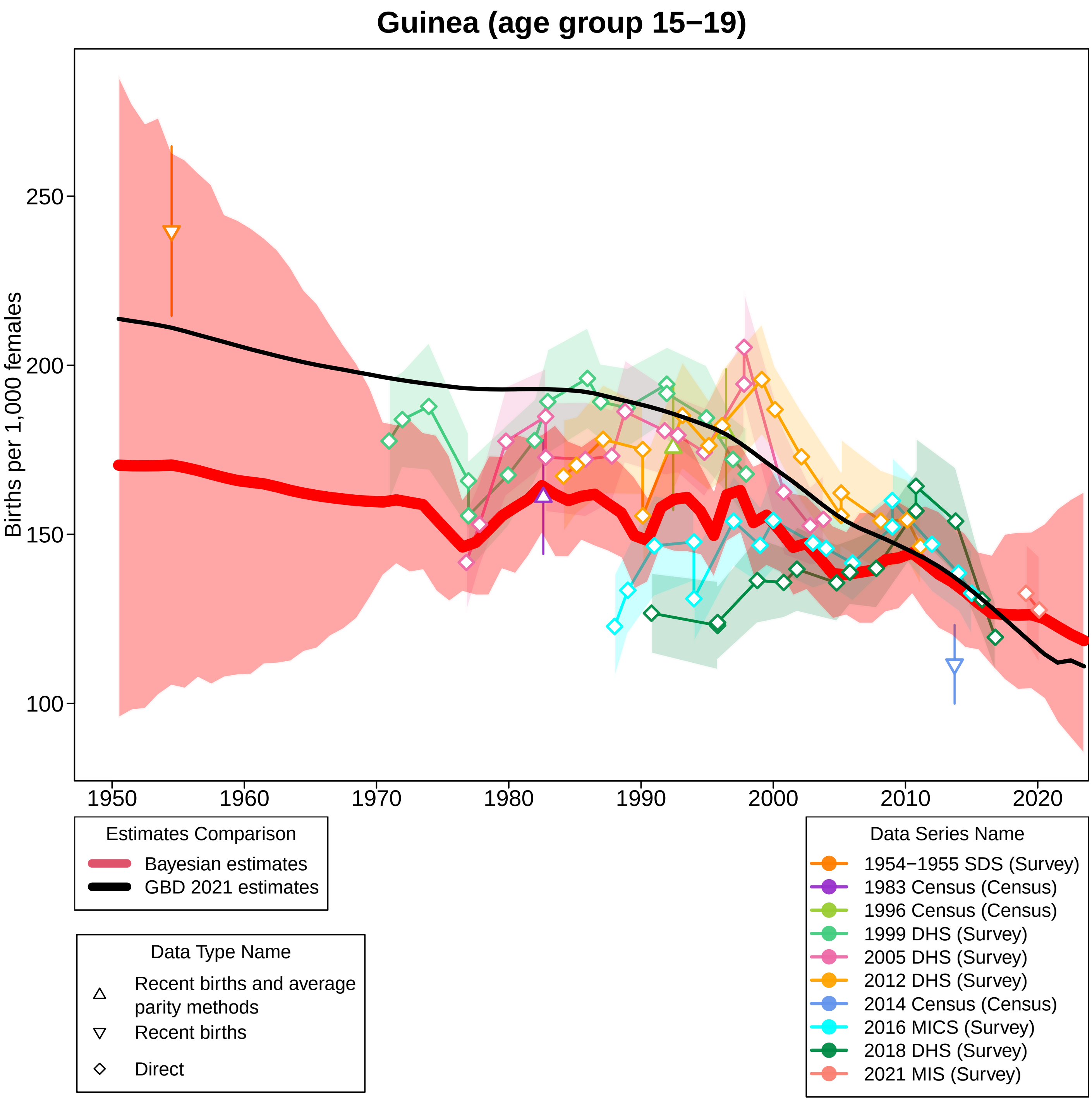
Guinea (age group 15−19)
Births per 1,000 females
250
200
150
100
1950
1960
1970
1980
1990
2000
2010
2020
Estimates Comparison
Bayesian estimates
GBD 2021 estimates
Data Type Name
Recent births and average parity methods
Recent births
Direct
Data Series Name
1954−1955 SDS (Survey)
1983 Census (Census)
1996 Census (Census)
1999 DHS (Survey)
2005 DHS (Survey)
2012 DHS (Survey)
2014 Census (Census)
2016 MICS (Survey)
2018 DHS (Survey)
2021 MIS (Survey)

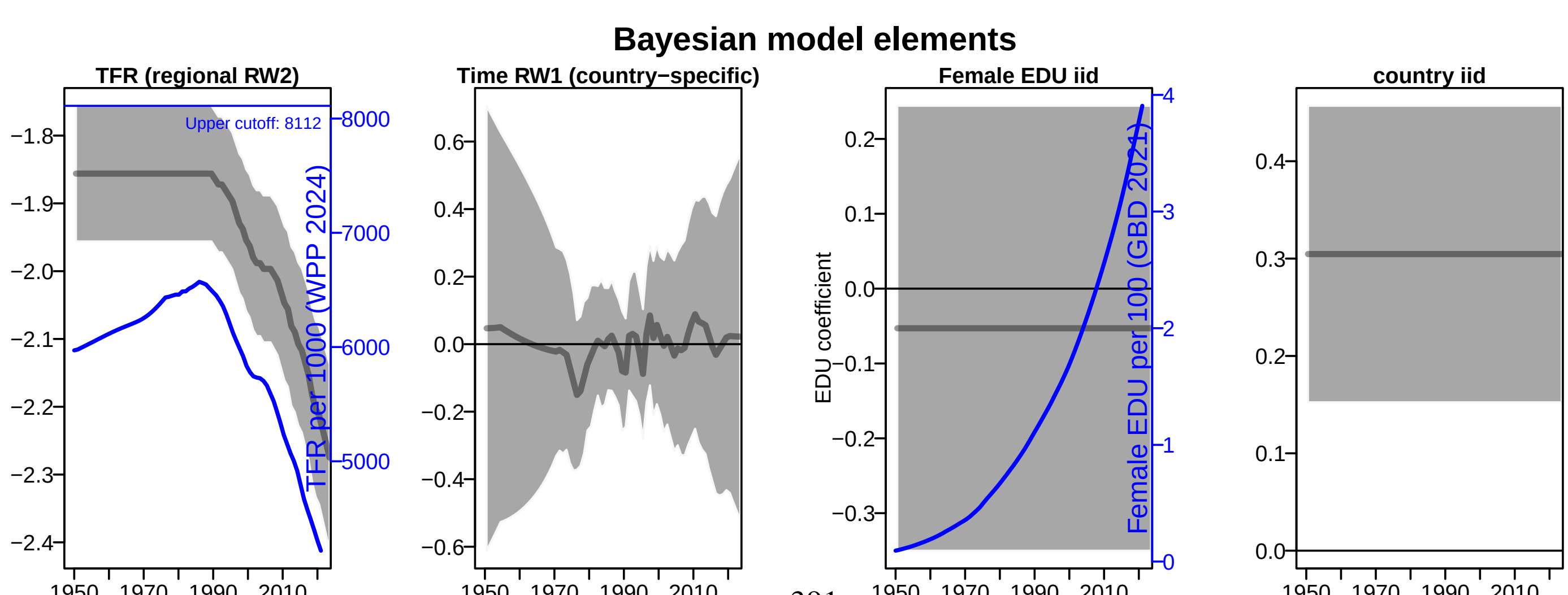
Bayesian model elements
TFR (regional RW2)
Upper cutoff: 8112
TFR per 1000 (WPP 2024)
Time RW1 (country−specific)
Female EDU iid
EDU coefficient
Female EDU per 100 (GBD 2021)
country iid

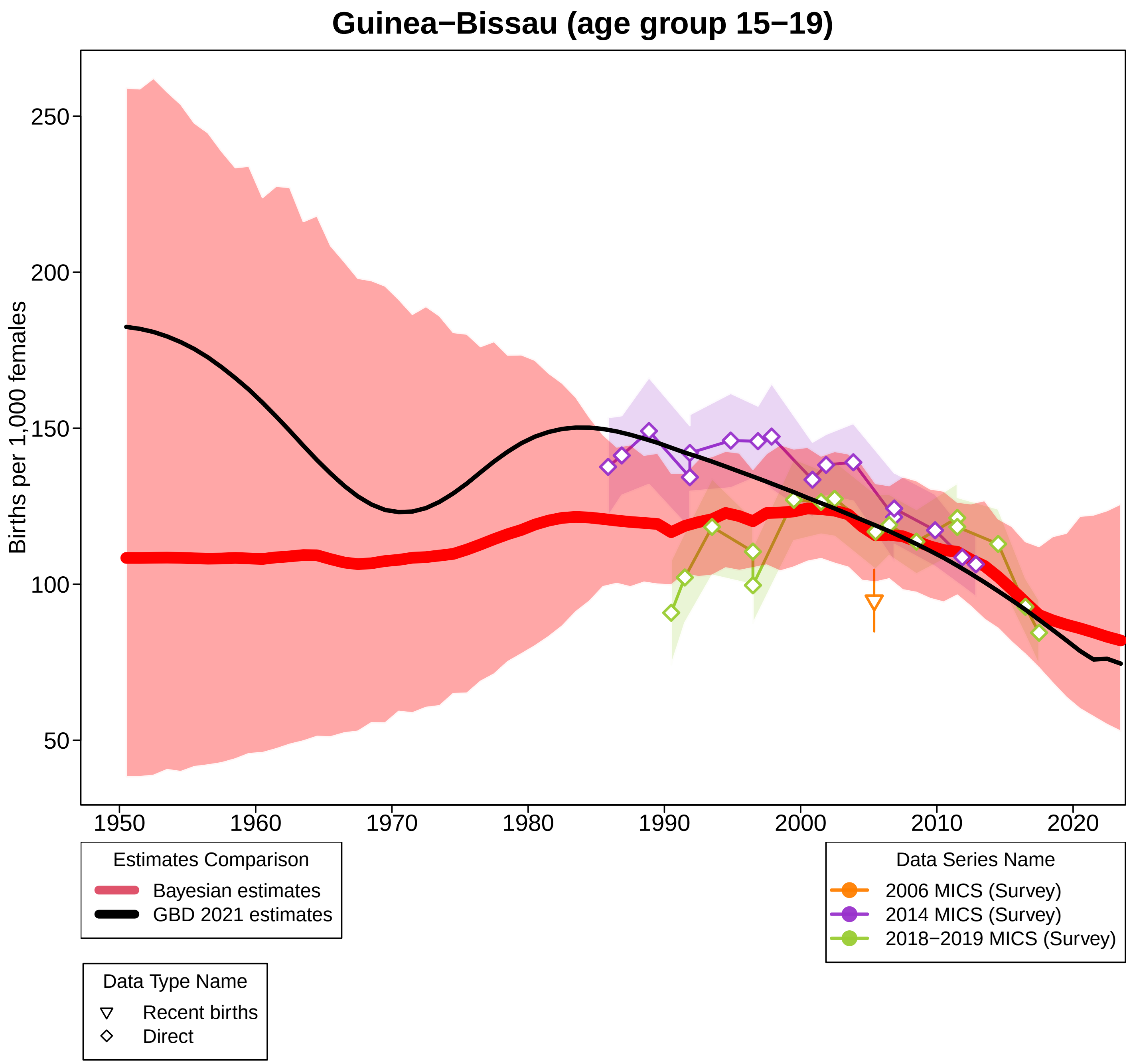
Guinea−Bissau (age group 15−19)
Births per 1,000 females
250
200
150
100
50
1950
1960
1970
1980
1990
2000
2010
2020
Estimates Comparison
Bayesian estimates
GBD 2021 estimates
Data Series Name
2006 MICS (Survey)
2014 MICS (Survey)
2018−2019 MICS (Survey)
Data Type Name
Recent births
Direct

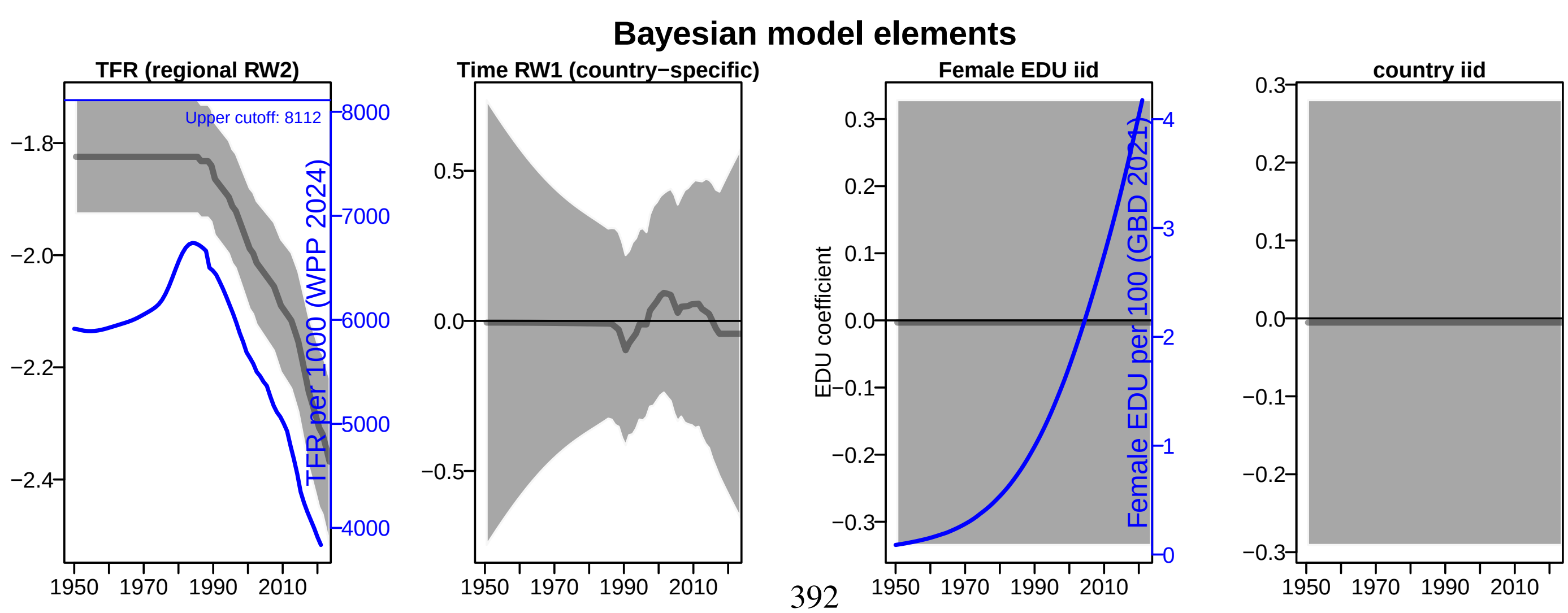
Bayesian model elements
TFR (regional RW2)
Upper cutoff: 8112
TFR per 1000 (WPP 2024)
Time RW1 (country−specific)
Female EDU iid
EDU coefficient
Female EDU per 100 (GBD 2021)
country iid

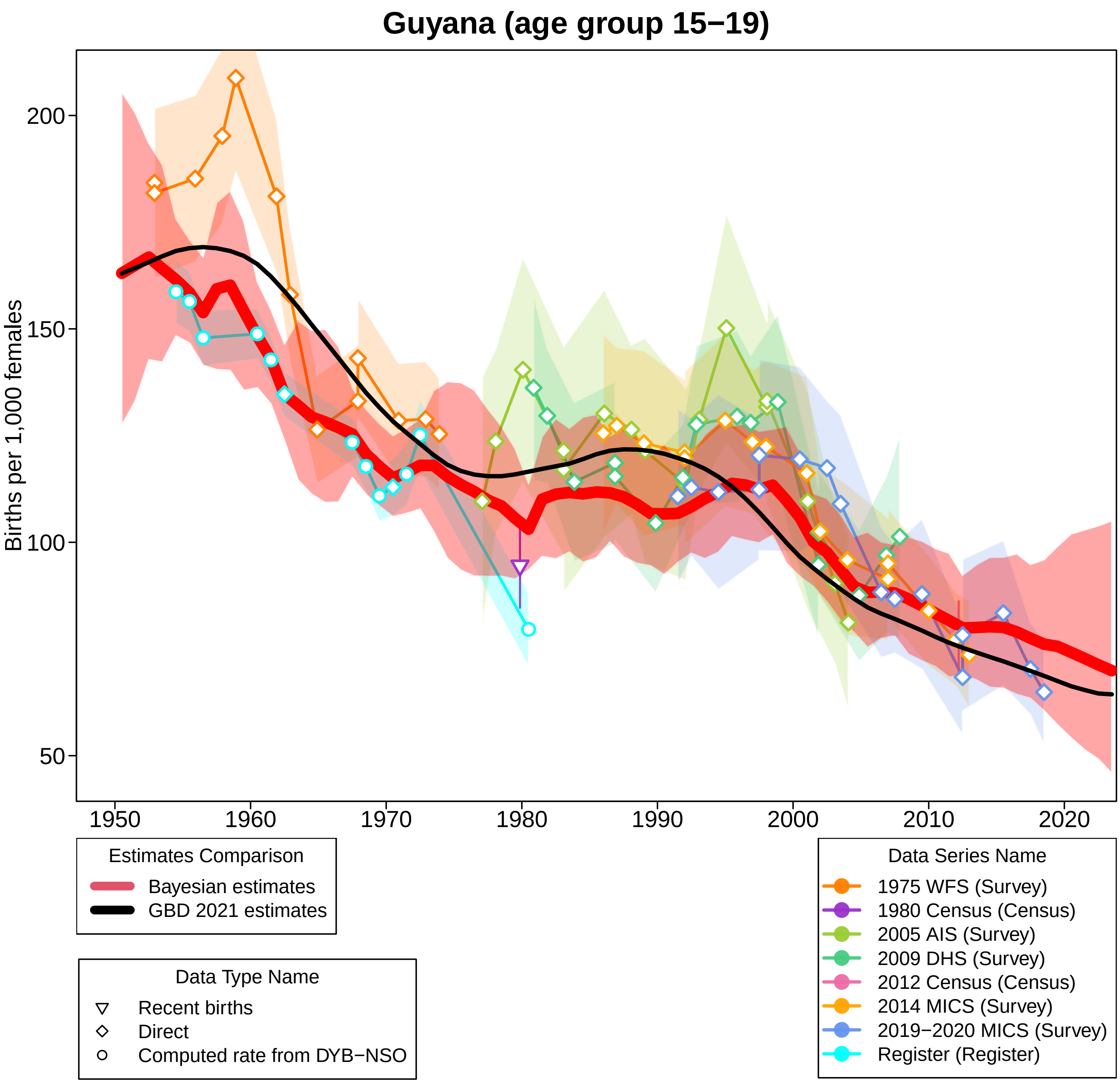
Guyana (age group 15−19)
Births per 1,000 females
200
150
100
50
1950
1960
1970
1980
1990
2000
2010
2020
Estimates Comparison
Bayesian estimates
GBD 2021 estimates
Data Type Name
Recent births
Direct
Computed rate from DYB−NSO
Data Series Name
1975 WFS (Survey)
1980 Census (Census)
2005 AIS (Survey)
2009 DHS (Survey)
2012 Census (Census)
2014 MICS (Survey)
2019−2020 MICS (Survey)
Register (Register)

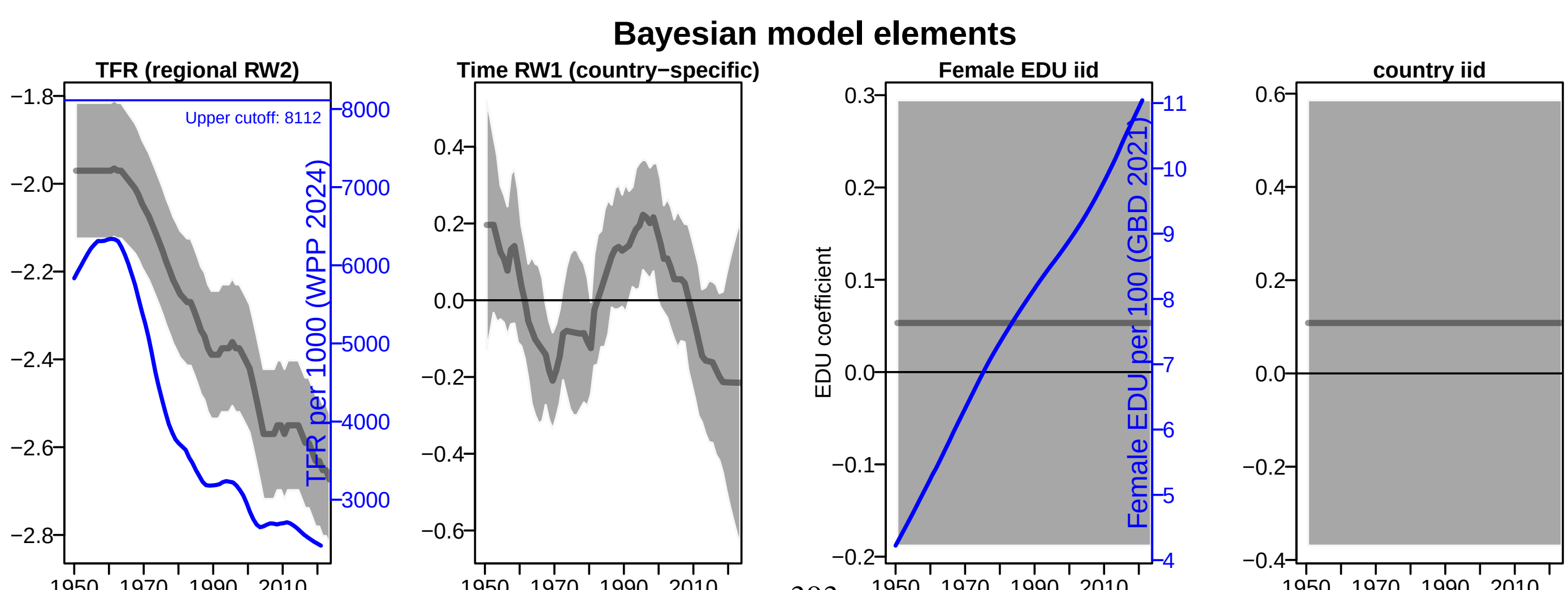
Bayesian model elements
TFR (regional RW2)
Upper cutoff: 8112
TFR per 1000 (WPP 2024)
Time RW1 (country−specific)
Female EDU iid
EDU coefficient
Female EDU per 100 (GBD 2021)
country iid

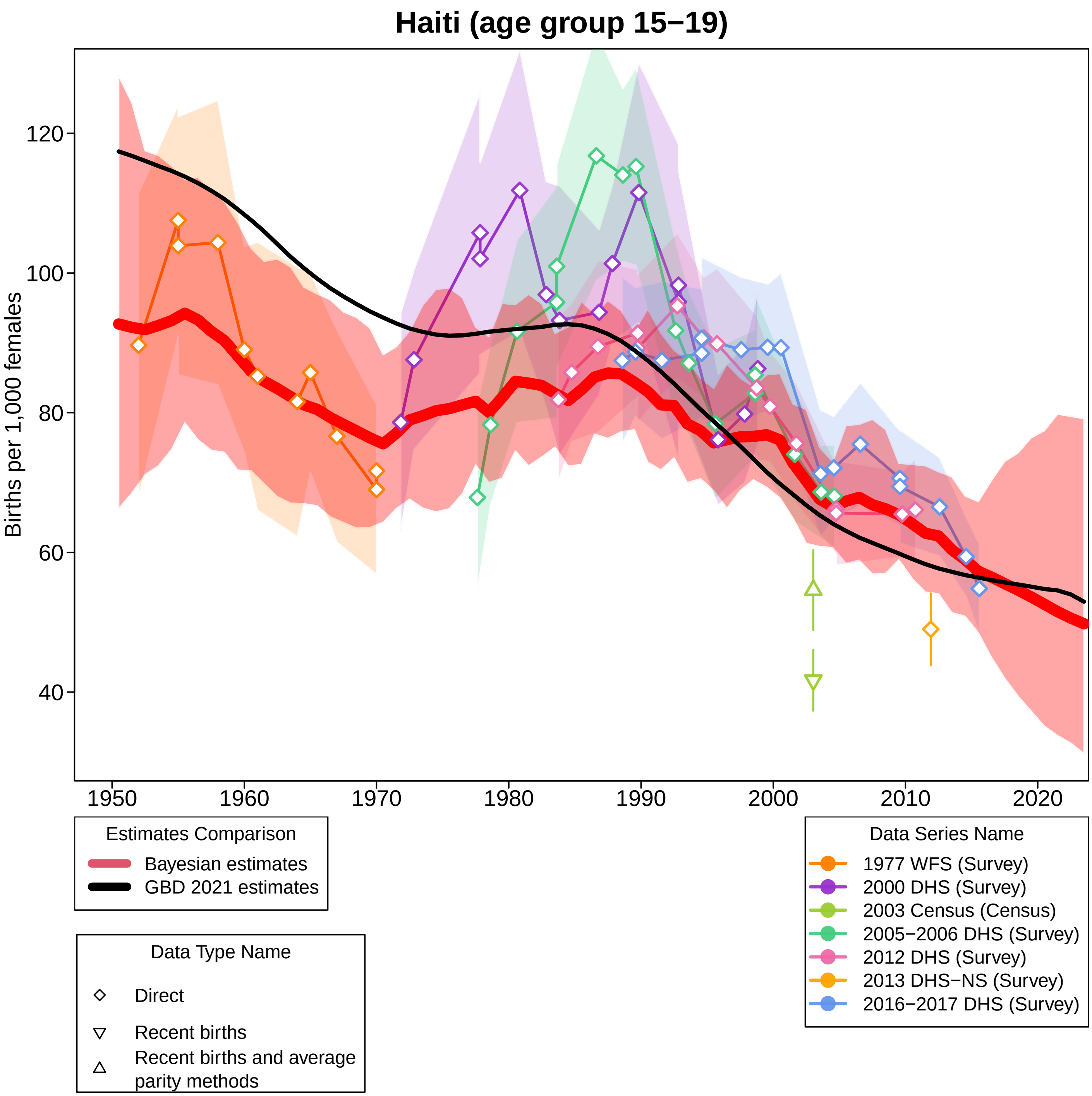
Haiti (age group 15−19)
Births per 1,000 females
120
100
80
60
40
1950
1960
1970
1980
1990
2000
2010
2020
Estimates Comparison
Bayesian estimates
GBD 2021 estimates
Data Series Name
1977 WFS (Survey)
2000 DHS (Survey)
2003 Census (Census)
2005−2006 DHS (Survey)
2012 DHS (Survey)
2013 DHS−NS (Survey)
2016−2017 DHS (Survey)
Data Type Name
Direct
Recent births
Recent births and average parity methods

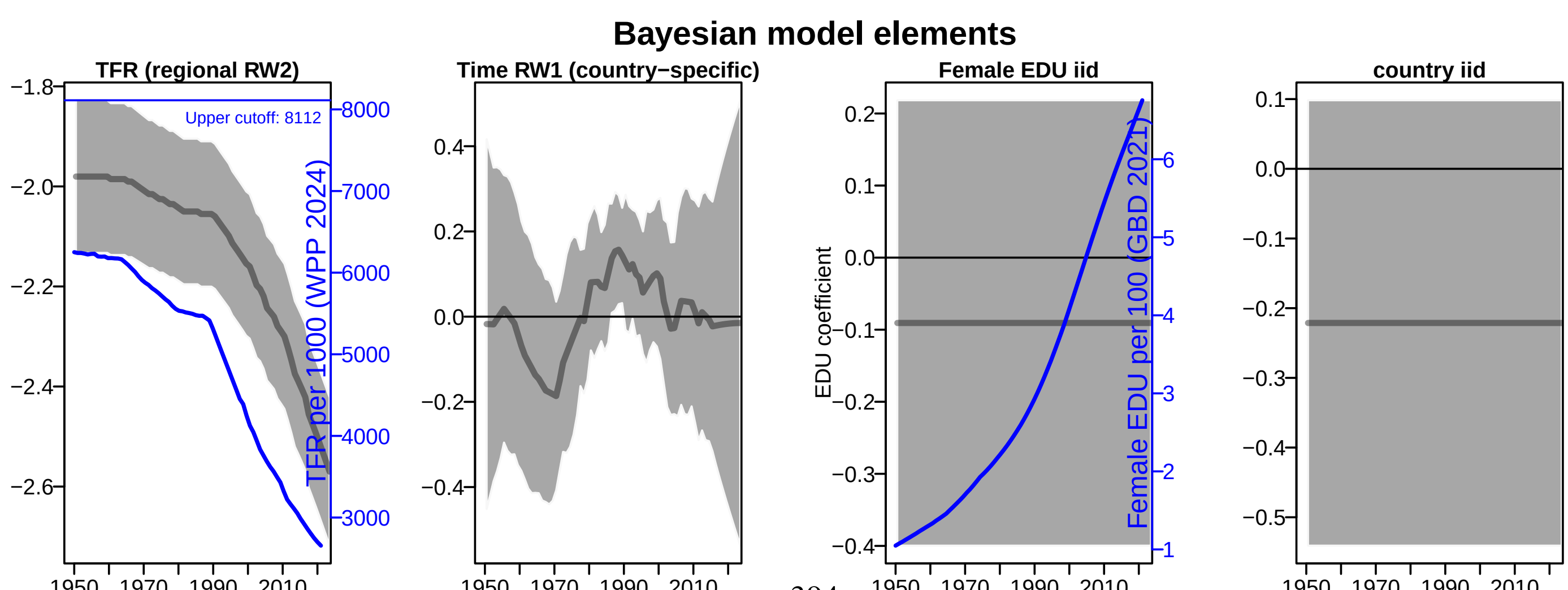
Bayesian model elements
TFR (regional RW2)
Upper cutoff: 8112
TFR per 1000 (WPP 2024)
Time RW1 (country−specific)
Female EDU iid
EDU coefficient
Female EDU per 100 (GBD 2021)
country iid

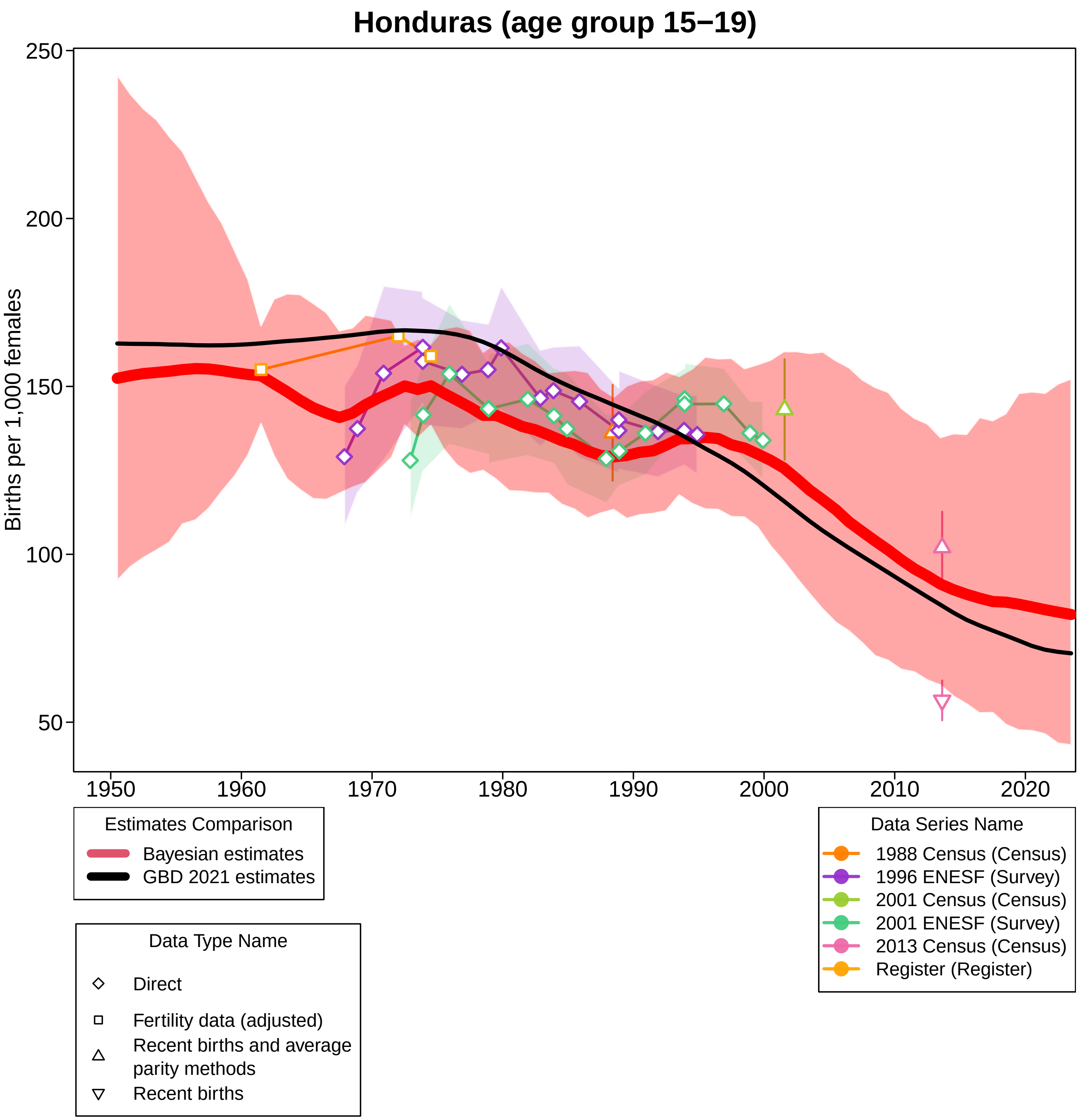

Honduras (age group 15−19)
Births per 1,000 females
250
200
150
100
50
1950
1960
1970
1980
1990
2000
2010
2020
Estimates Comparison
Bayesian estimates
GBD 2021 estimates
Data Series Name
1988 Census (Census)
1996 ENESF (Survey)
2001 Census (Census)
2001 ENESF (Survey)
2013 Census (Census)
Register (Register)
Data Type Name
Direct
Fertility data (adjusted)
Recent births and average parity methods
Recent births

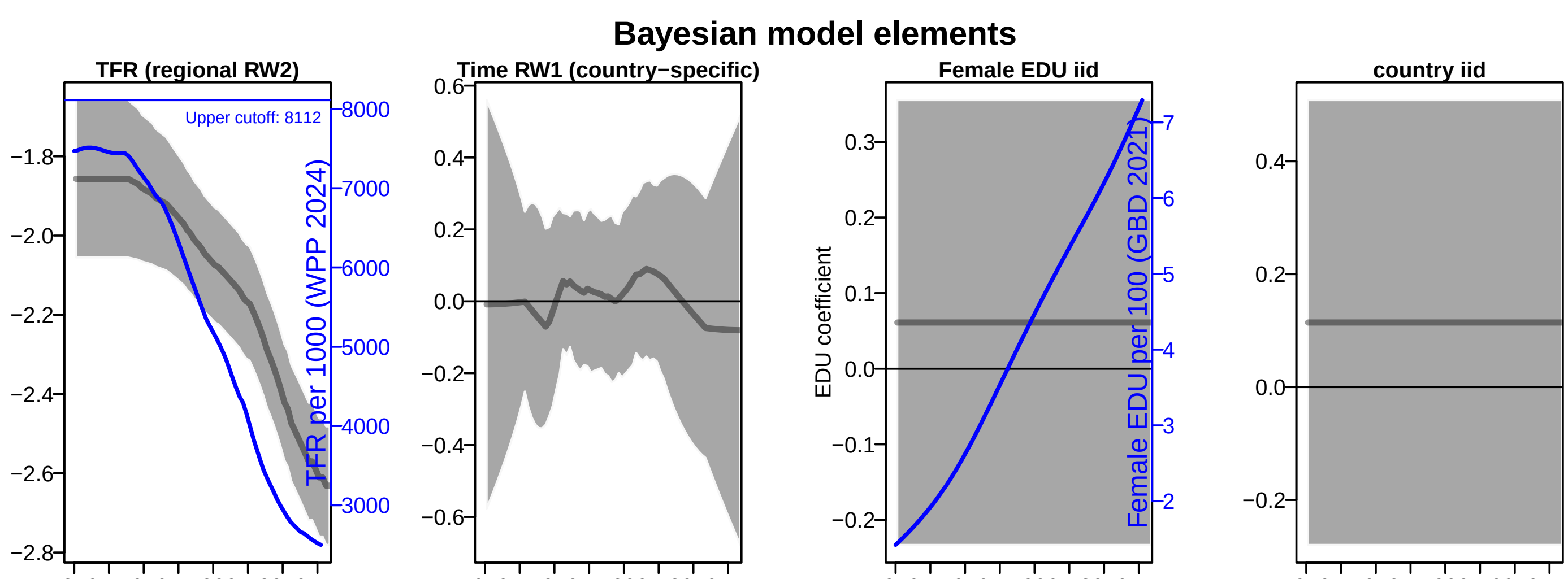

Bayesian model elements
TFR (regional RW2)
Upper cutoff: 8112
TFR per 1000 (WPP 2024)
Time RW1 (country−specific)
Female EDU iid
EDU coefficient
Female EDU per 100 (GBD 2021)
country iid
1950
1970
1990
2010

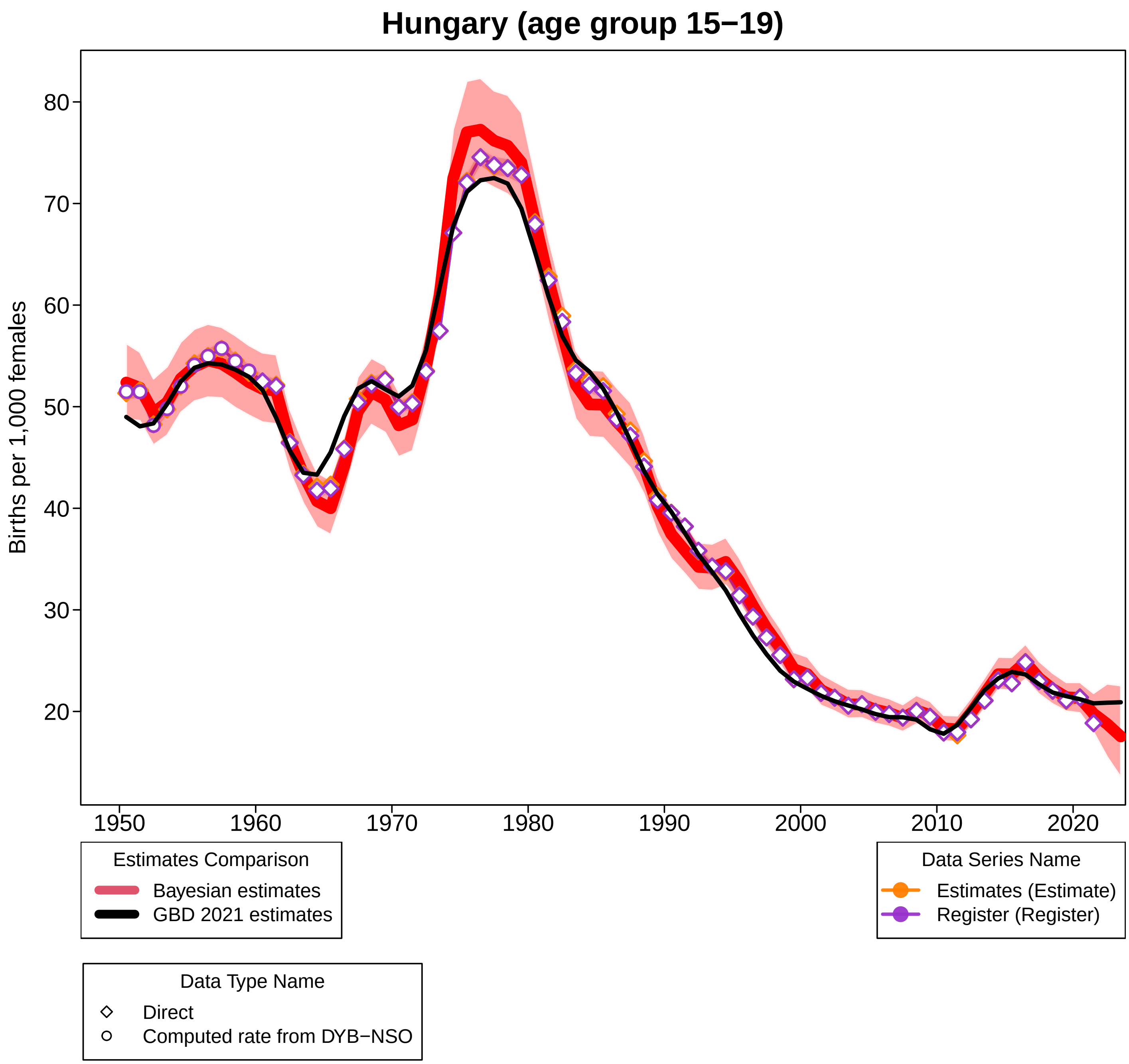
Hungary (age group 15−19)
Births per 1,000 females
80
70
60
50
40
30
20
1950
1960
1970
1980
1990
2000
2010
2020
Estimates Comparison
Bayesian estimates
GBD 2021 estimates
Data Series Name
Estimates (Estimate)
Register (Register)
Data Type Name
Direct
Computed rate from DYB−NSO

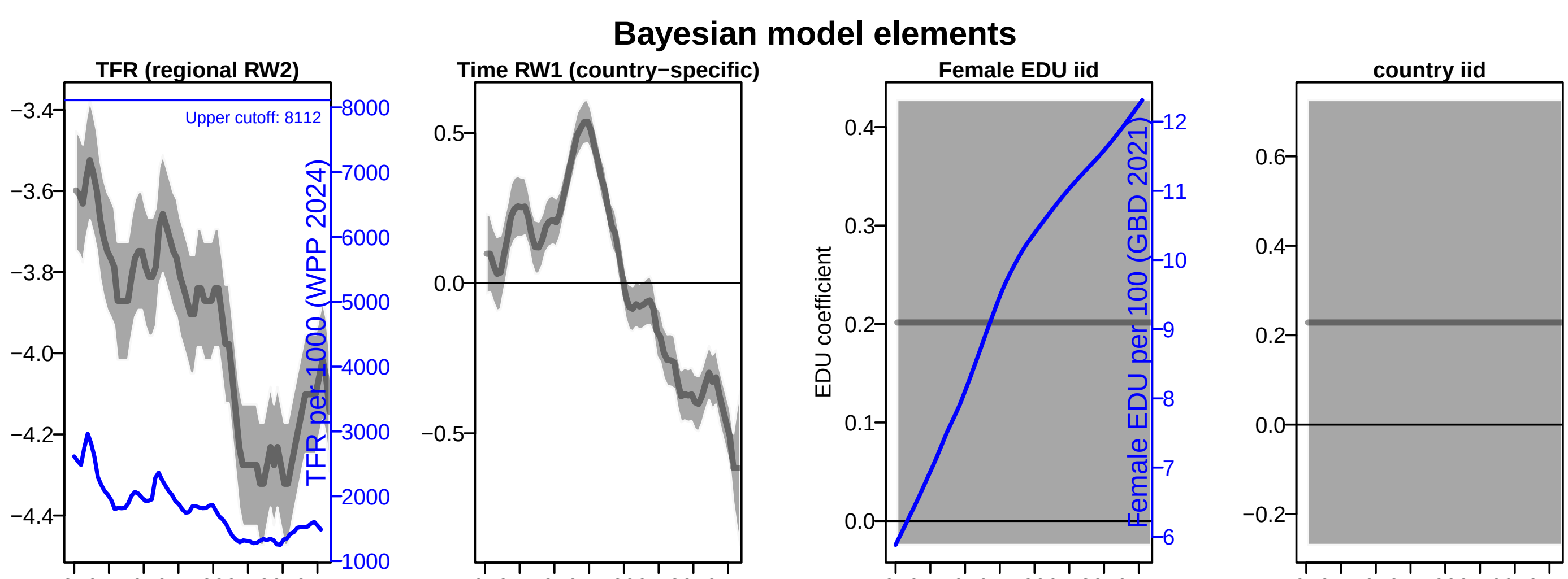
Bayesian model elements
TFR (regional RW2)
Upper cutoff: 8112
TFR per 1000 (WPP 2024)
−3.4
−3.6
−3.8
−4.0
−4.2
−4.4
8000
7000
6000
5000
4000
3000
2000
1000
Time RW1 (country−specific)
0.5
0.0
−0.5
Female EDU iid
EDU coefficient
Female EDU per 100 (GBD 2021)
0.4
0.3
0.2
0.1
0.0
12
11
10
9
8
7
6
country iid
0.6
0.4
0.2
0.0
−0.2
1950
1970
1990
2010

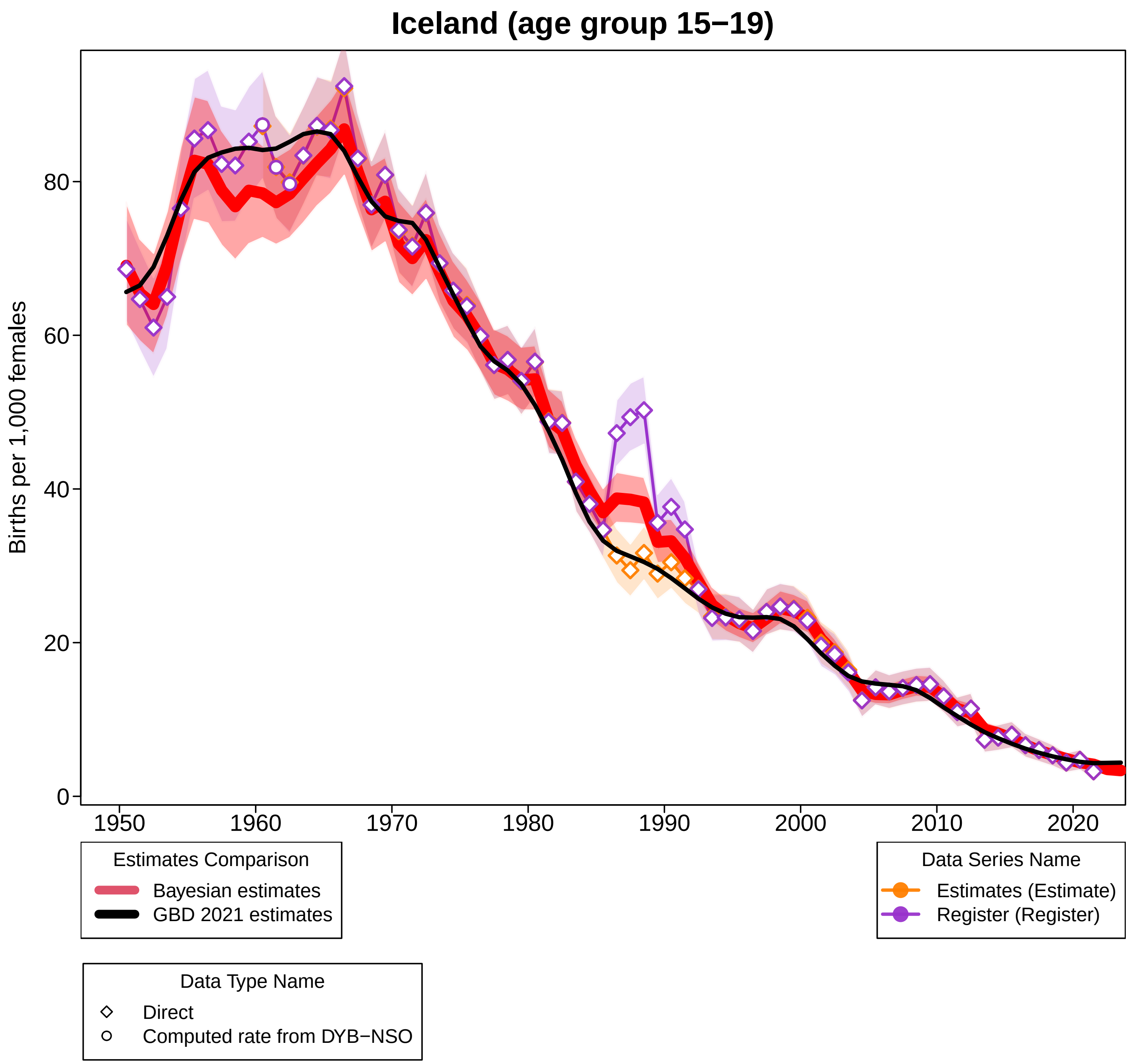
Iceland (age group 15−19)
Births per 1,000 females
0
20
40
60
80
1950
1960
1970
1980
1990
2000
2010
2020
Estimates Comparison
Bayesian estimates
GBD 2021 estimates
Data Series Name
Estimates (Estimate)
Register (Register)
Data Type Name
Direct
Computed rate from DYB−NSO

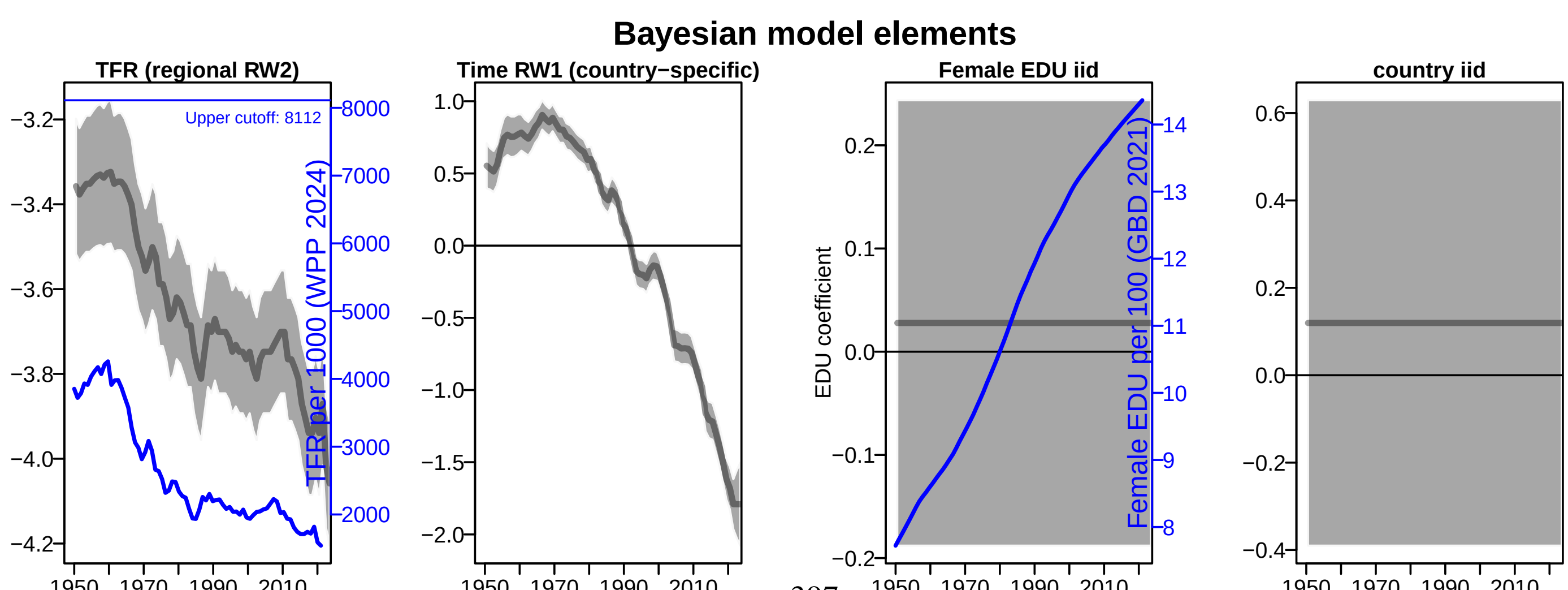
Bayesian model elements
TFR (regional RW2)
Upper cutoff: 8112
TFR per 1000 (WPP 2024)
Time RW1 (country−specific)
Female EDU iid
EDU coefficient
Female EDU per 100 (GBD 2021)
country iid

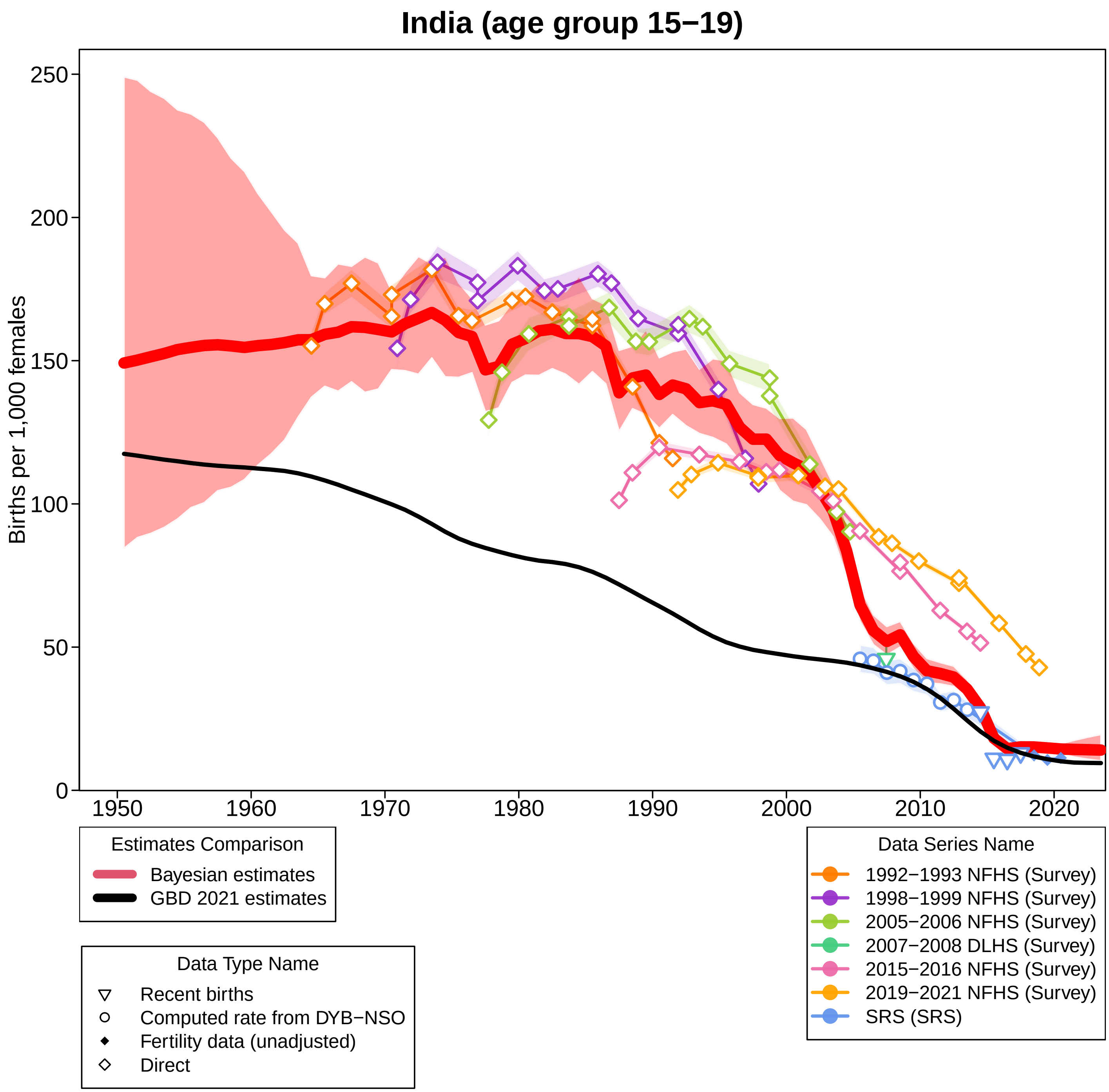
India (age group 15−19)
Births per 1,000 females
0
50
100
150
200
250
1950
1960
1970
1980
1990
2000
2010
2020
Estimates Comparison
Bayesian estimates
GBD 2021 estimates
Data Type Name
Recent births
Computed rate from DYB−NSO
Fertility data (unadjusted)
Direct
Data Series Name
1992−1993 NFHS (Survey)
1998−1999 NFHS (Survey)
2005−2006 NFHS (Survey)
2007−2008 DLHS (Survey)
2015−2016 NFHS (Survey)
2019−2021 NFHS (Survey)
SRS (SRS)

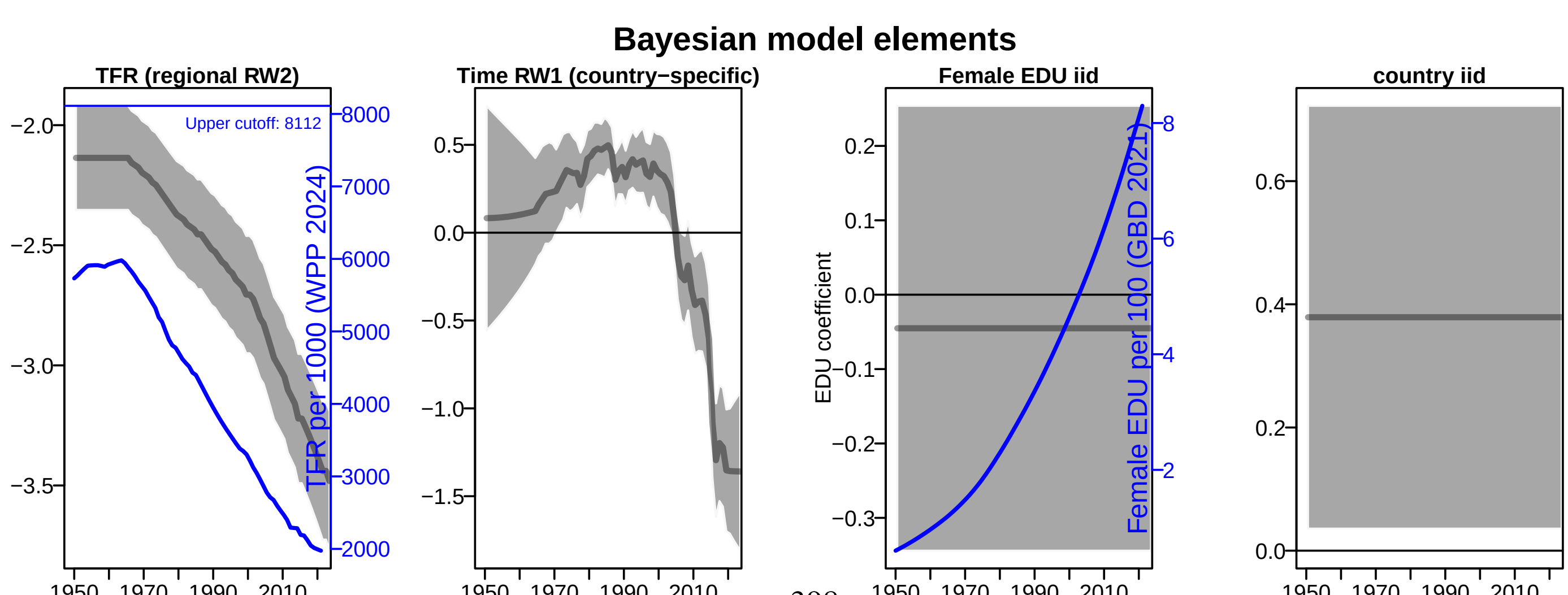
Bayesian model elements
TFR (regional RW2)
Upper cutoff: 8112
TFR per 1000 (WPP 2024)
Time RW1 (country−specific)
Female EDU iid
EDU coefficient
Female EDU per 100 (GBD 2021)
country iid
1950
1970
1990
2010

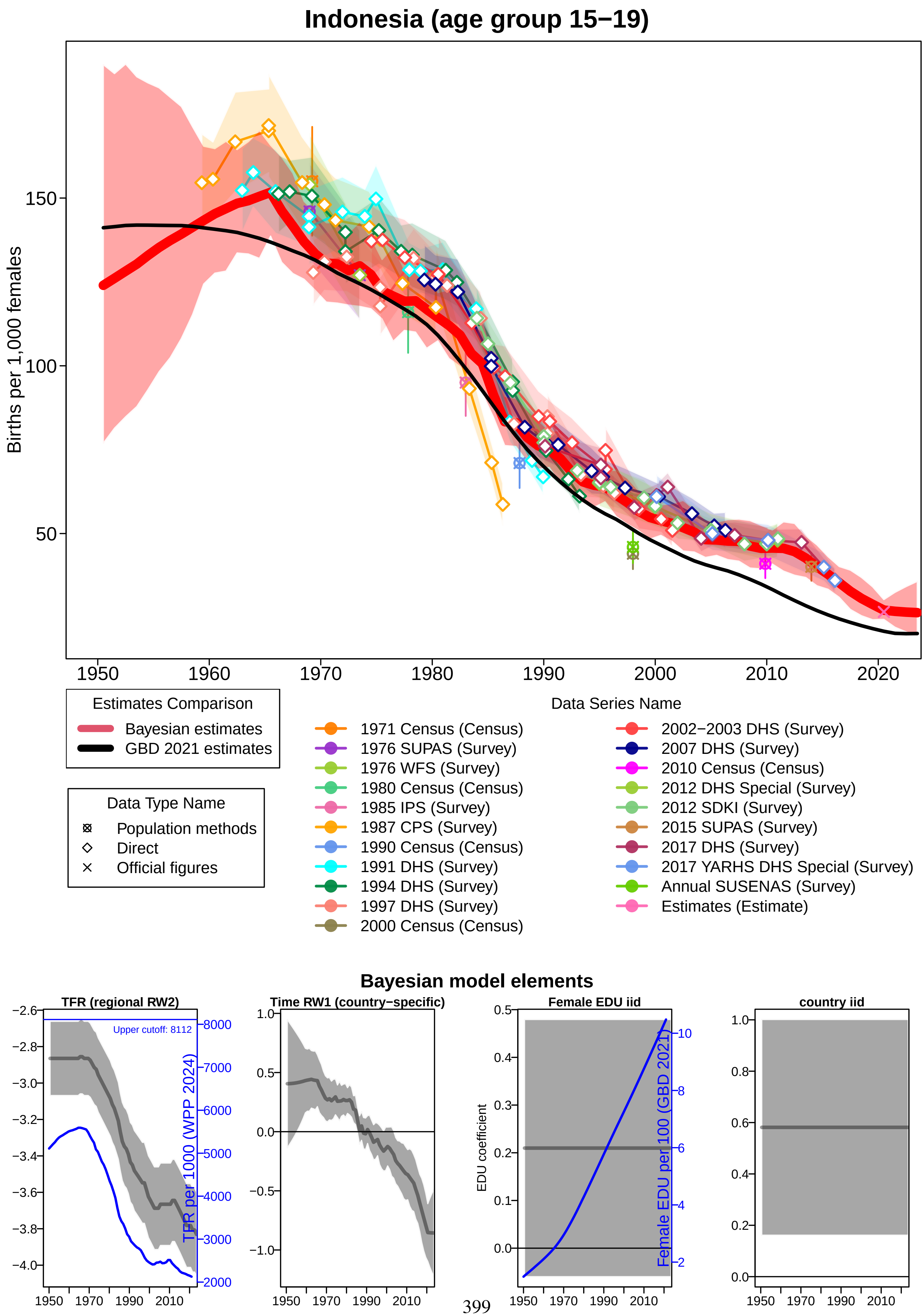
Indonesia (age group 15−19)
Births per 1,000 females
150
100
50
1950
1960
1970
1980
1990
2000
2010
2020
Estimates Comparison
Bayesian estimates
GBD 2021 estimates
Data Type Name
Population methods
Direct
Official figures
Data Series Name
1971 Census (Census)
1976 SUPAS (Survey)
1976 WFS (Survey)
1980 Census (Census)
1985 IPS (Survey)
1987 CPS (Survey)
1990 Census (Census)
1991 DHS (Survey)
1994 DHS (Survey)
1997 DHS (Survey)
2000 Census (Census)
2002−2003 DHS (Survey)
2007 DHS (Survey)
2010 Census (Census)
2012 DHS Special (Survey)
2012 SDKI (Survey)
2015 SUPAS (Survey)
2017 DHS (Survey)
2017 YARHS DHS Special (Survey)
Annual SUSENAS (Survey)
Estimates (Estimate)
Bayesian model elements
TFR (regional RW2)
Upper cutoff: 8112
TFR per 1000 (WPP 2024)
Time RW1 (country−specific)
Female EDU iid
EDU coefficient
Female EDU per 100 (GBD 2021)
country iid

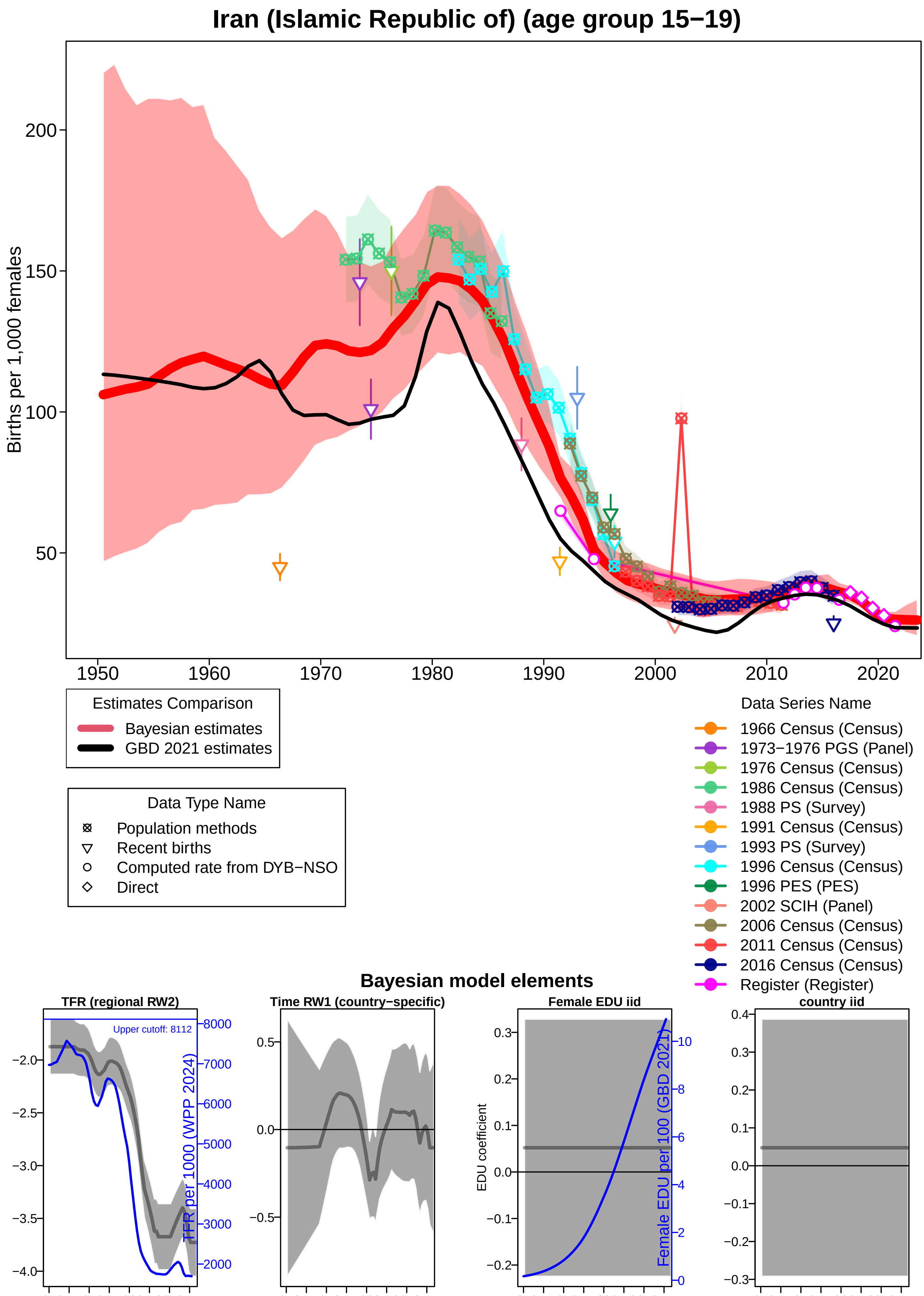
Iran (Islamic Republic of) (age group 15−19)
Births per 1,000 females
200
150
100
50
1950
1960
1970
1980
1990
2000
2010
2020
Estimates Comparison
Bayesian estimates
GBD 2021 estimates
Data Type Name
Population methods
Recent births
Computed rate from DYB−NSO
Direct
Data Series Name
1966 Census (Census)
1973−1976 PGS (Panel)
1976 Census (Census)
1986 Census (Census)
1988 PS (Survey)
1991 Census (Census)
1993 PS (Survey)
1996 Census (Census)
1996 PES (PES)
2002 SCIH (Panel)
2006 Census (Census)
2011 Census (Census)
2016 Census (Census)
Register (Register)
Bayesian model elements
TFR (regional RW2)
Upper cutoff: 8112
TFR per 1000 (WPP 2024)
Time RW1 (country−specific)
Female EDU iid
EDU coefficient
Female EDU per 100 (GBD 2021)
country iid

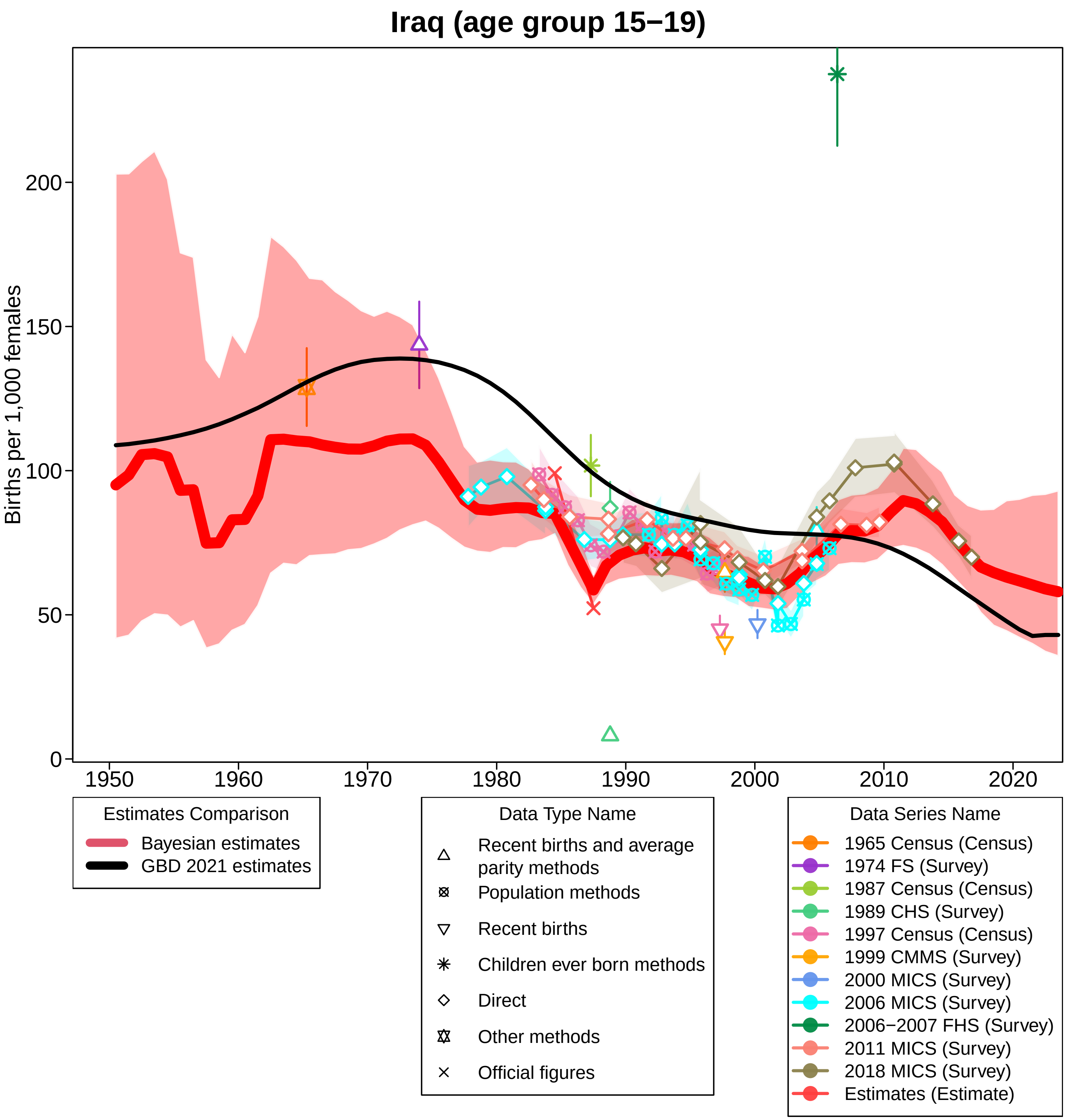
Iraq (age group 15−19)
Births per 1,000 females
0
50
100
150
200
1950
1960
1970
1980
1990
2000
2010
2020
Estimates Comparison
Bayesian estimates
GBD 2021 estimates
Data Type Name
Recent births and average parity methods
Population methods
Recent births
Children ever born methods
Direct
Other methods
Official figures
Data Series Name
1965 Census (Census)
1974 FS (Survey)
1987 Census (Census)
1989 CHS (Survey)
1997 Census (Census)
1999 CMMS (Survey)
2000 MICS (Survey)
2006 MICS (Survey)
2006−2007 FHS (Survey)
2011 MICS (Survey)
2018 MICS (Survey)
Estimates (Estimate)

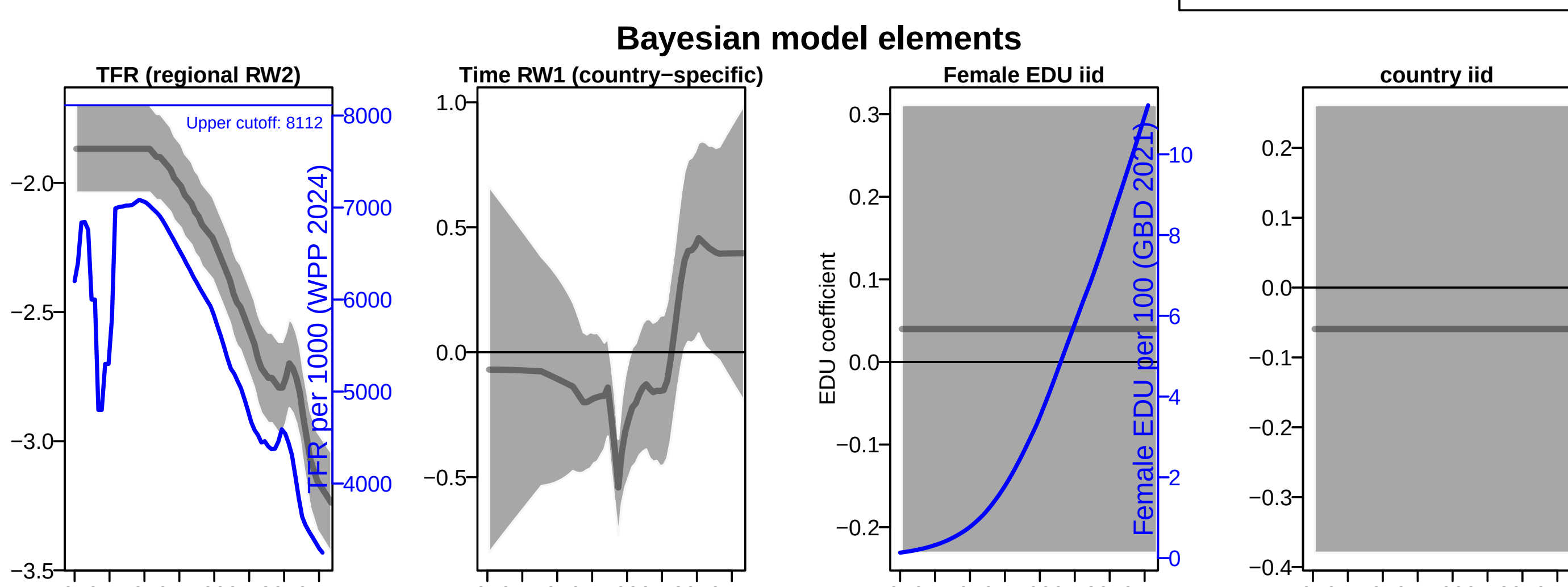
Bayesian model elements
TFR (regional RW2)
Upper cutoff: 8112
TFR per 1000 (WPP 2024)
Time RW1 (country−specific)
Female EDU iid
EDU coefficient
Female EDU per 100 (GBD 2021)
country iid

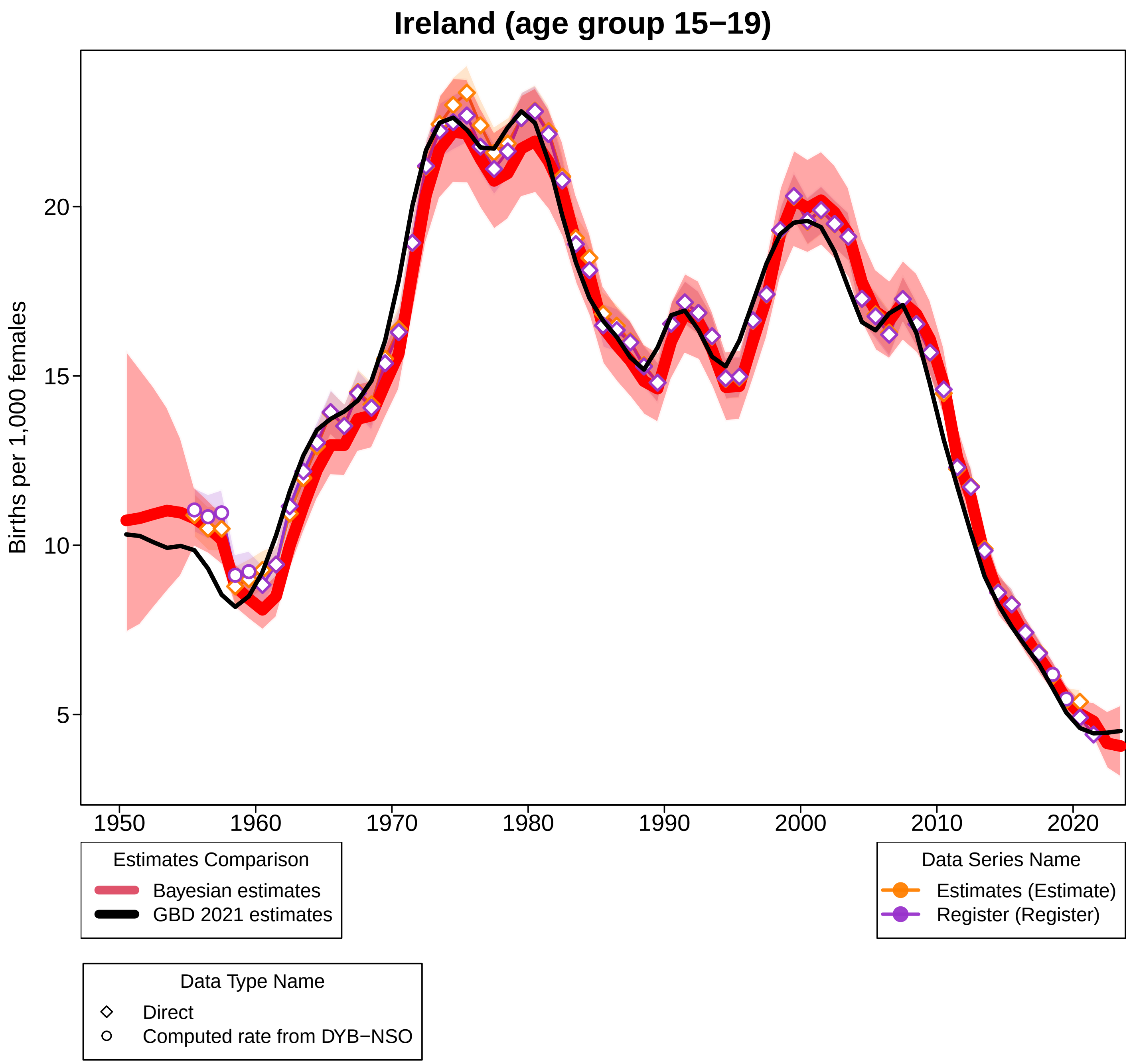
Ireland (age group 15−19)
Births per 1,000 females
5
10
15
20
1950
1960
1970
1980
1990
2000
2010
2020
Estimates Comparison
Bayesian estimates
GBD 2021 estimates
Data Series Name
Estimates (Estimate)
Register (Register)
Data Type Name
Direct
Computed rate from DYB−NSO

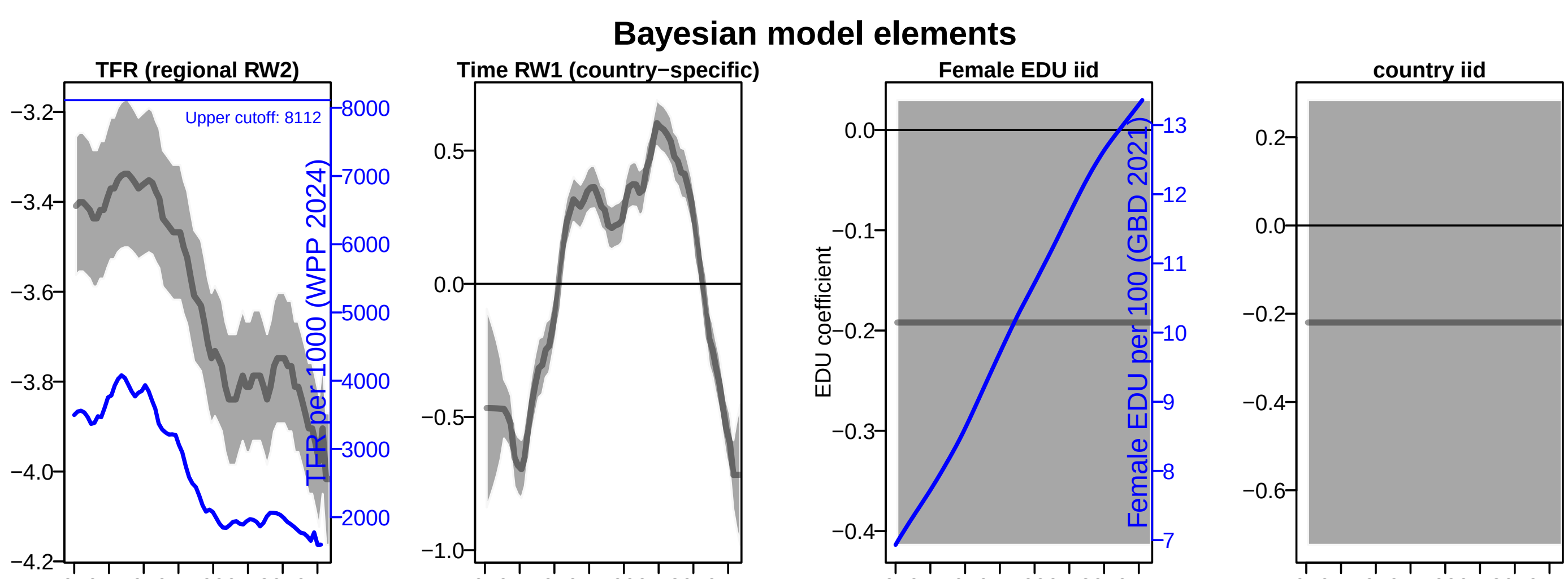
Bayesian model elements
TFR (regional RW2)
Upper cutoff: 8112
TFR per 1000 (WPP 2024)
Time RW1 (country−specific)
Female EDU iid
EDU coefficient
Female EDU per 100 (GBD 2021)
country iid

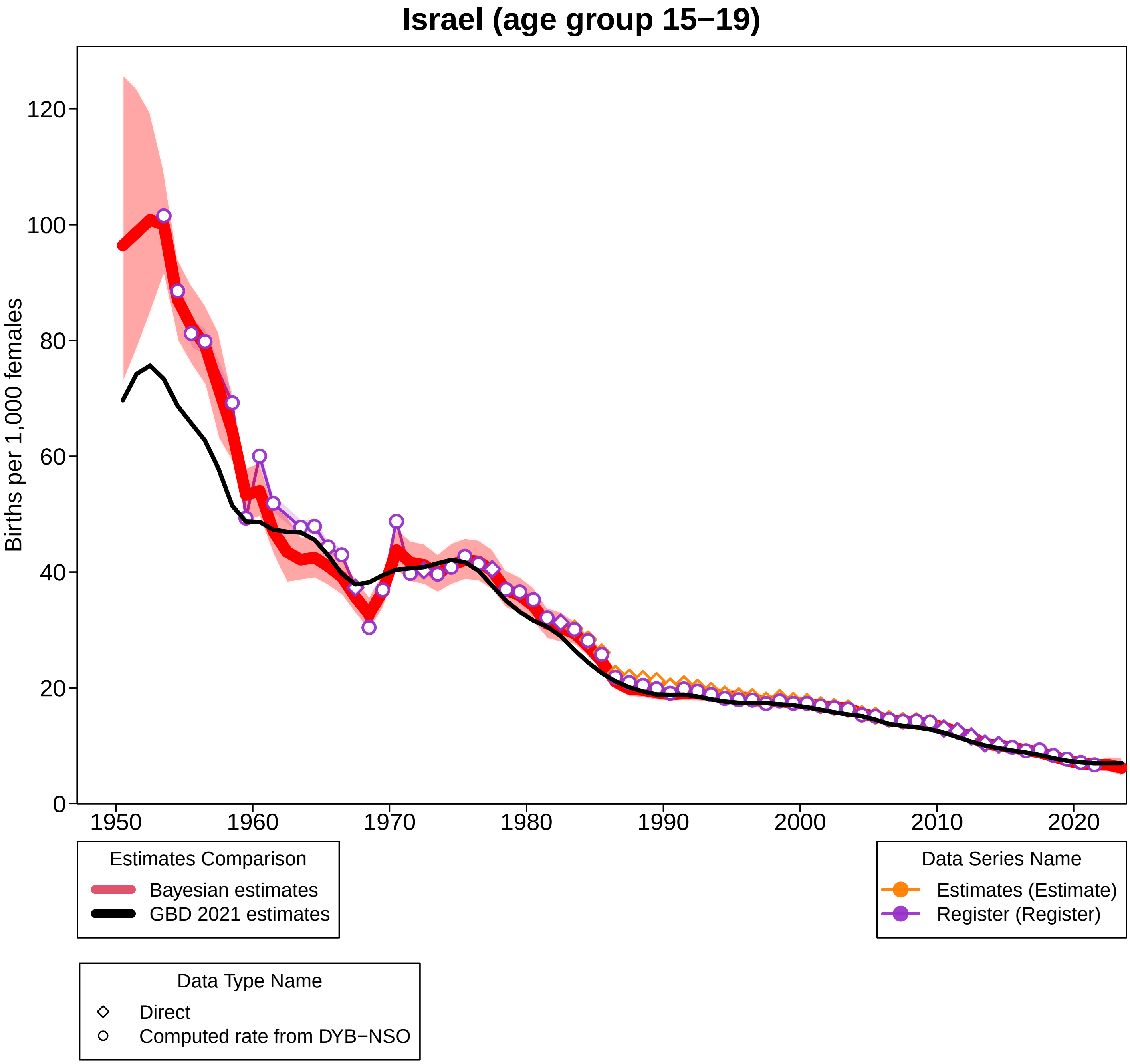
Israel (age group 15−19)
Births per 1,000 females
Estimates Comparison
Bayesian estimates
GBD 2021 estimates
Data Series Name
Estimates (Estimate)
Register (Register)
Data Type Name
Direct
Computed rate from DYB−NSO

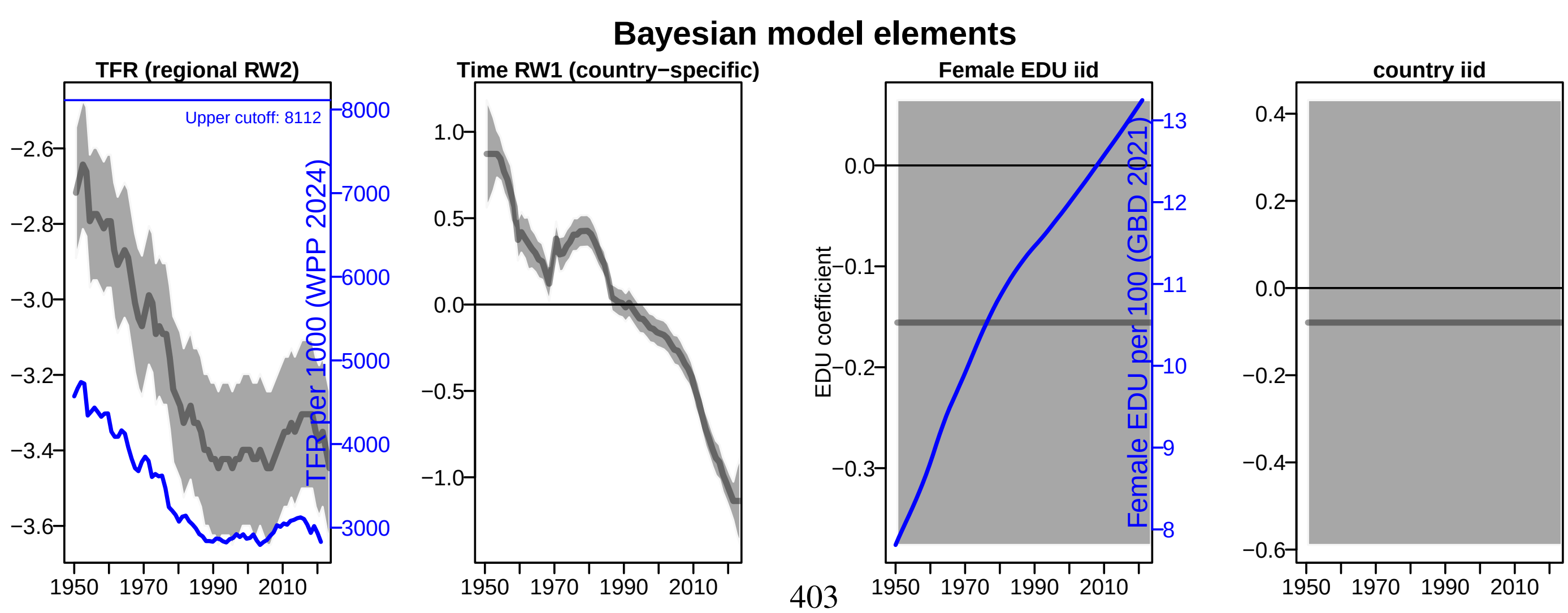
Bayesian model elements
TFR (regional RW2)
Upper cutoff: 8112
TFR per 1000 (WPP 2024)
Time RW1 (country−specific)
Female EDU iid
EDU coefficient
Female EDU per 100 (GBD 2021)
country iid

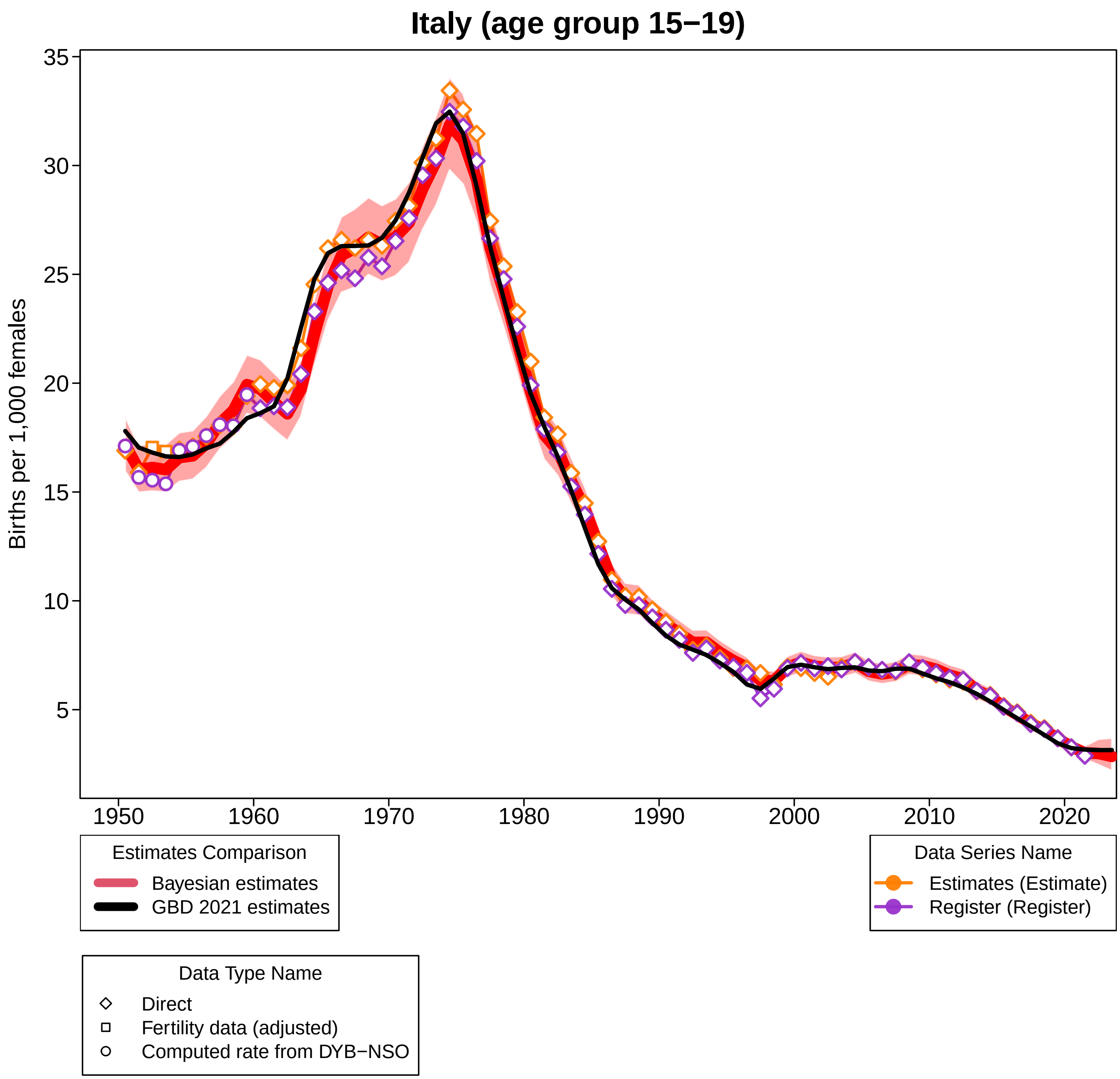
Italy (age group 15−19)
Births per 1,000 females
35
30
25
20
15
10
5
1950
1960
1970
1980
1990
2000
2010
2020
Estimates Comparison
Bayesian estimates
GBD 2021 estimates
Data Series Name
Estimates (Estimate)
Register (Register)
Data Type Name
Direct
Fertility data (adjusted)
Computed rate from DYB−NSO

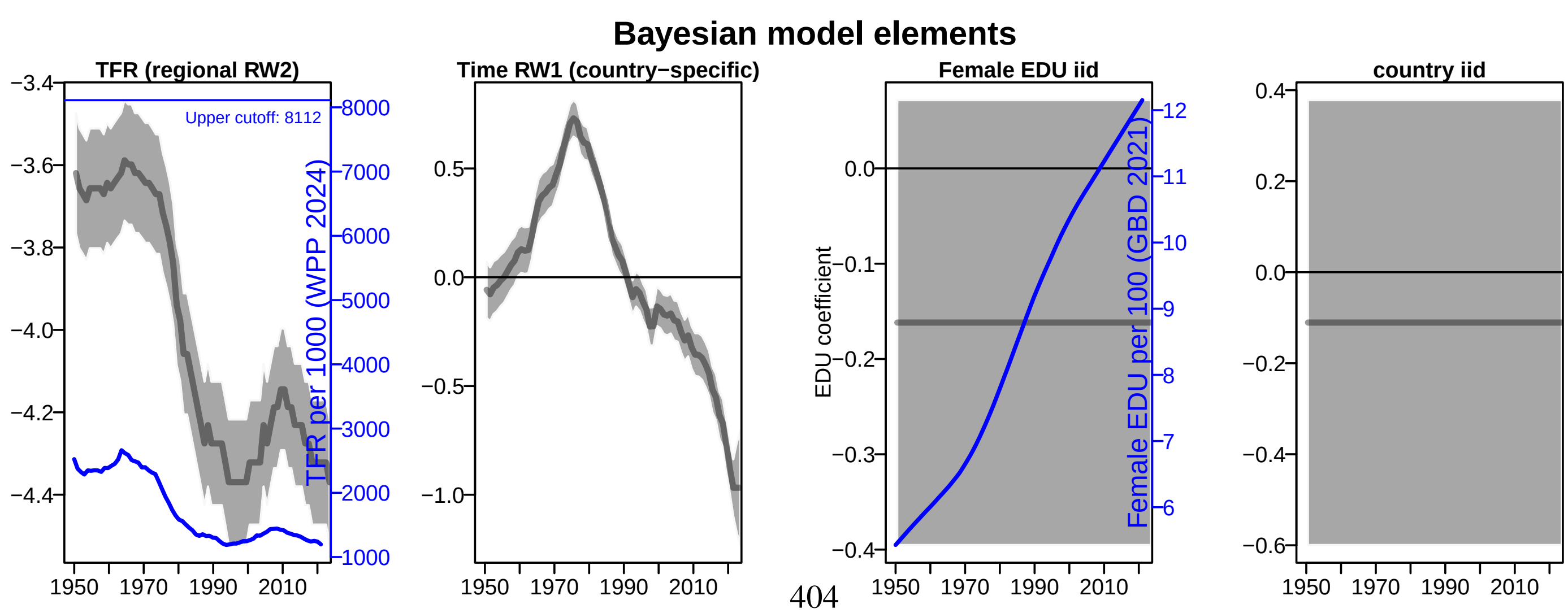
Bayesian model elements
TFR (regional RW2)
Upper cutoff: 8112
TFR per 1000 (WPP 2024)
Time RW1 (country−specific)
Female EDU iid
EDU coefficient
Female EDU per 100 (GBD 2021)
country iid
1950
1970
1990
2010

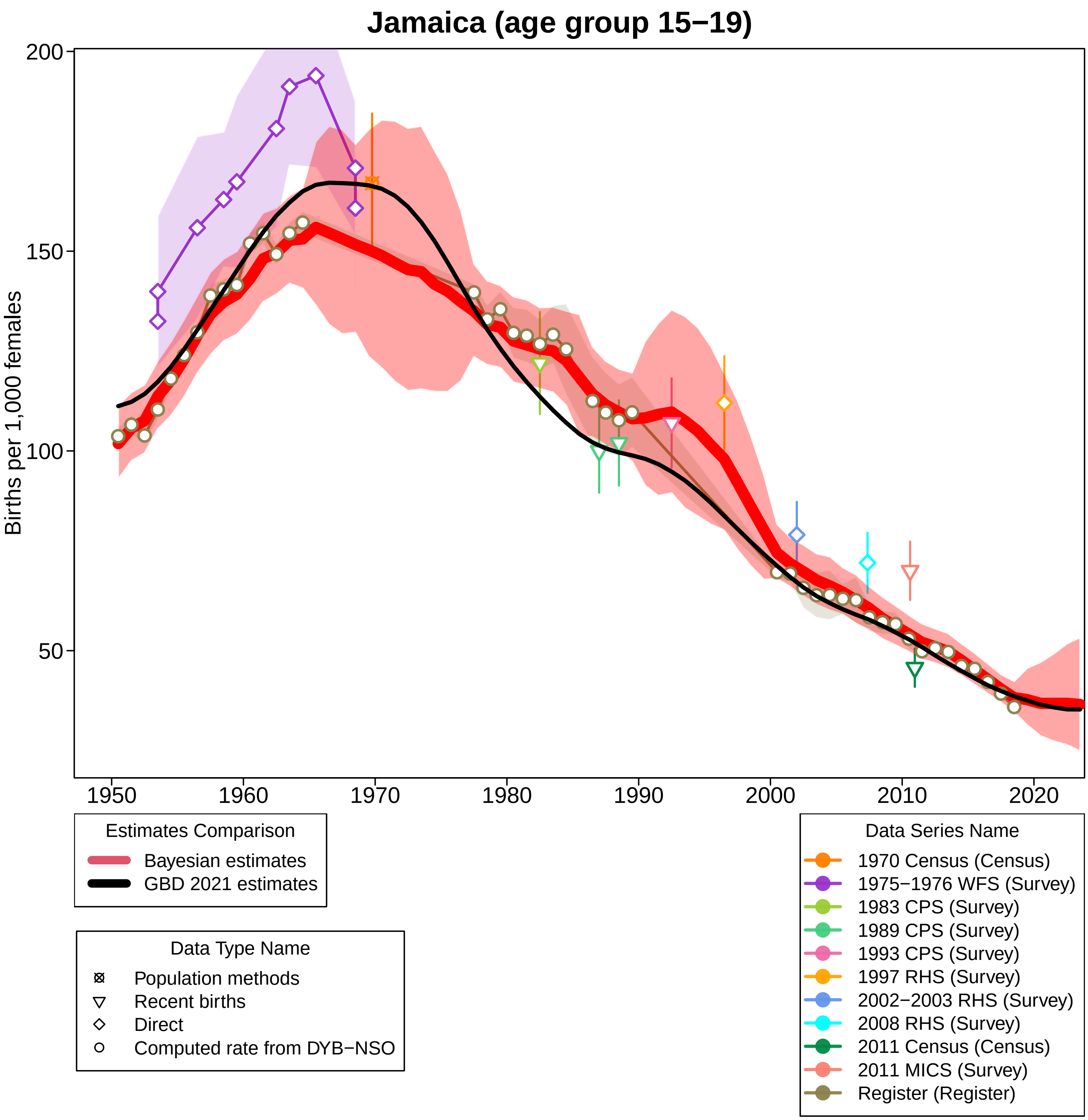

Jamaica (age group 15−19)
Births per 1,000 females
200
150
100
50
1950
1960
1970
1980
1990
2000
2010
2020
Estimates Comparison
Bayesian estimates
GBD 2021 estimates
Data Type Name
Population methods
Recent births
Direct
Computed rate from DYB−NSO
Data Series Name
1970 Census (Census)
1975−1976 WFS (Survey)
1983 CPS (Survey)
1989 CPS (Survey)
1993 CPS (Survey)
1997 RHS (Survey)
2002−2003 RHS (Survey)
2008 RHS (Survey)
2011 Census (Census)
2011 MICS (Survey)
Register (Register)

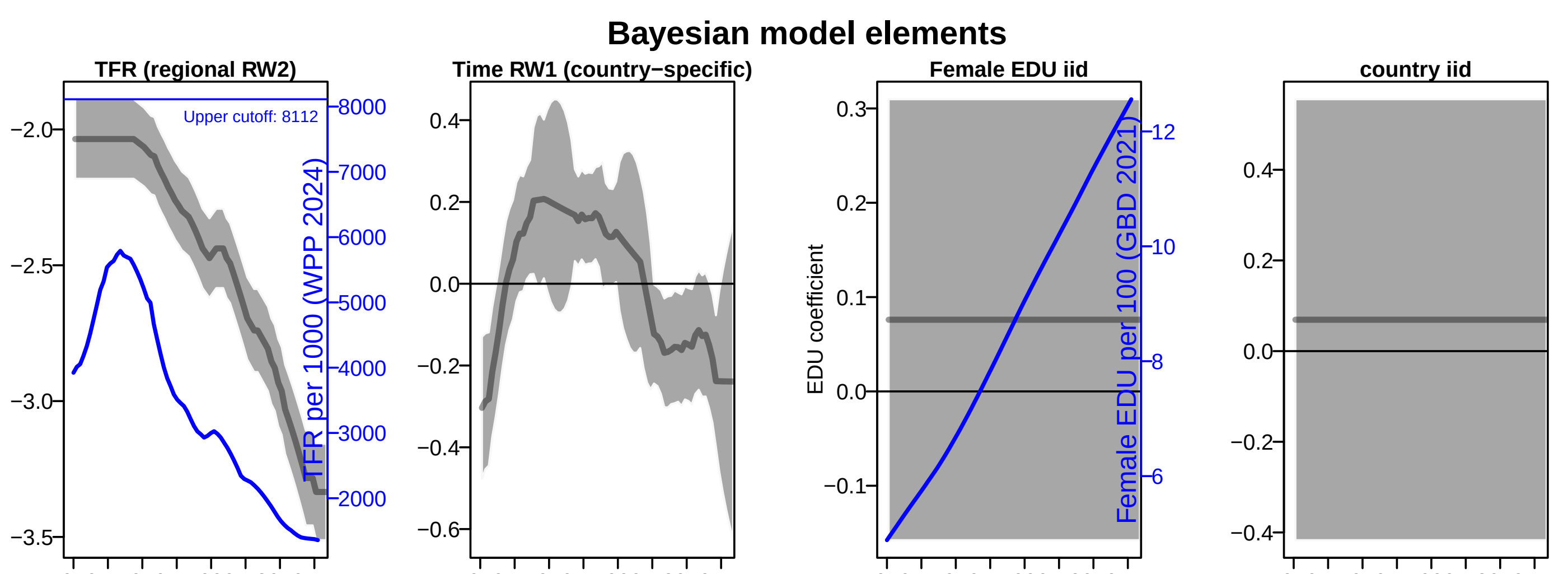

Bayesian model elements
TFR (regional RW2)
Upper cutoff: 8112
TFR per 1000 (WPP 2024)
Time RW1 (country−specific)
Female EDU iid
EDU coefficient
Female EDU per 100 (GBD 2021)
country iid
1950
1970
1990
2010

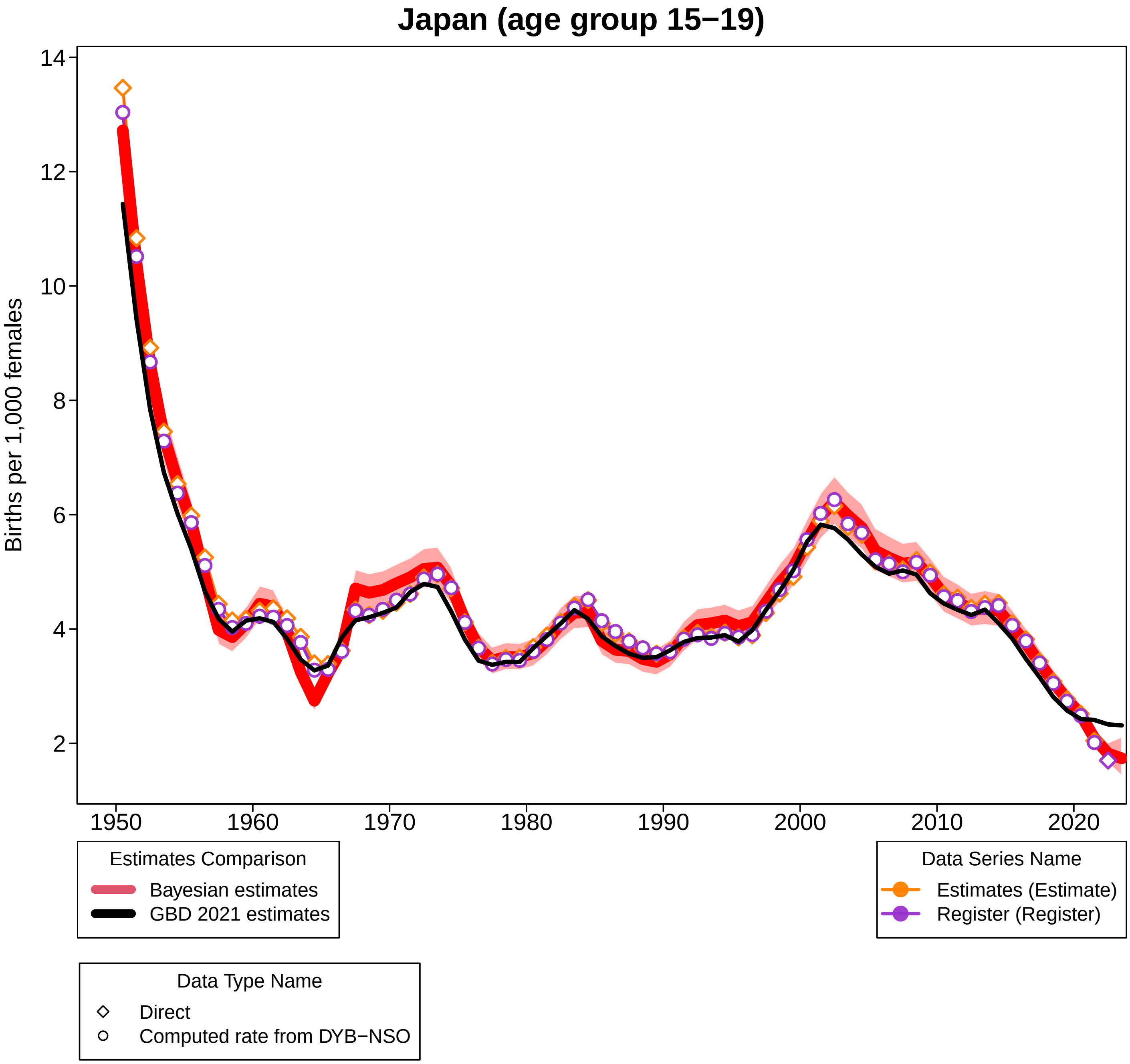
Japan (age group 15−19)
Births per 1,000 females
14
12
10
8
6
4
2
1950
1960
1970
1980
1990
2000
2010
2020
Estimates Comparison
Bayesian estimates
GBD 2021 estimates
Data Series Name
Estimates (Estimate)
Register (Register)
Data Type Name
Direct
Computed rate from DYB−NSO

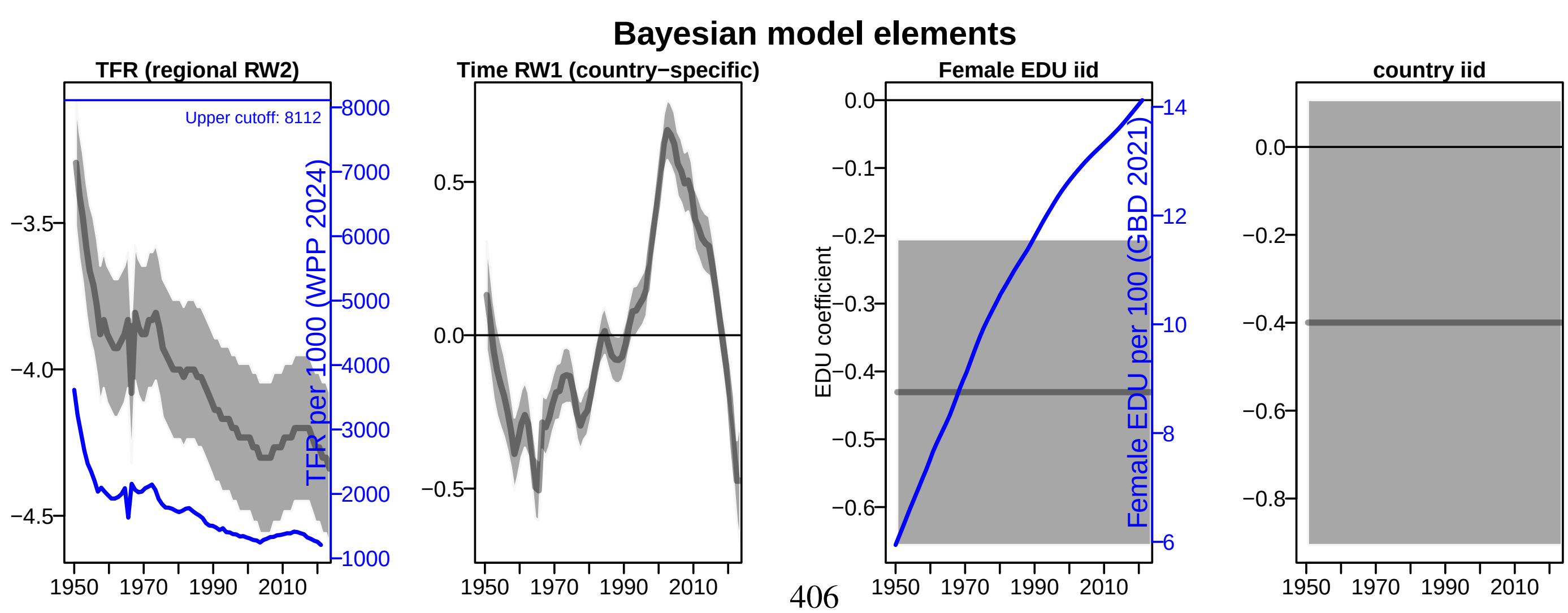
Bayesian model elements
TFR (regional RW2)
Upper cutoff: 8112
TFR per 1000 (WPP 2024)
Time RW1 (country−specific)
Female EDU iid
EDU coefficient
Female EDU per 100 (GBD 2021)
country iid

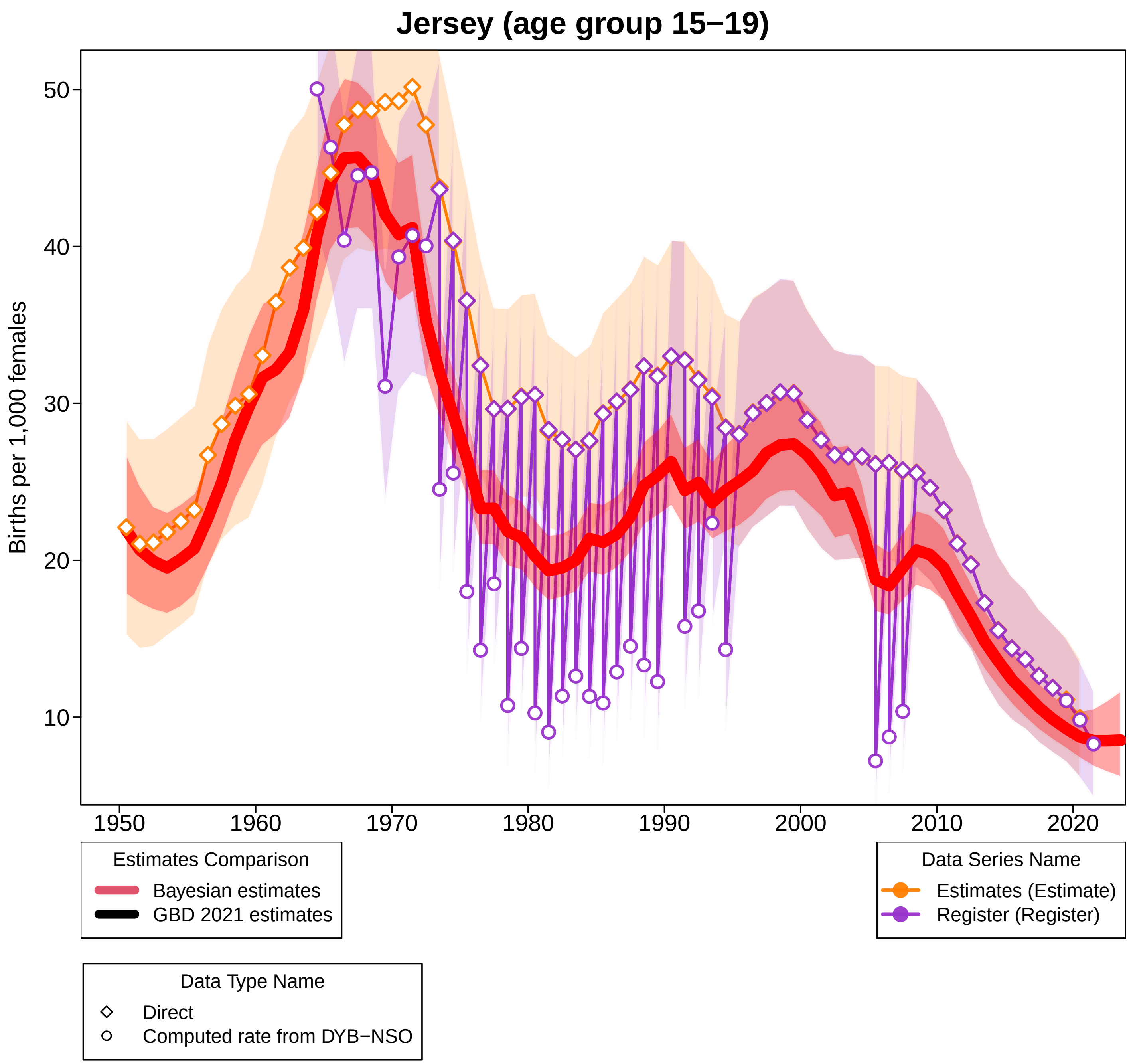
Jersey (age group 15−19)
Births per 1,000 females
Estimates Comparison
Bayesian estimates
GBD 2021 estimates
Data Series Name
Estimates (Estimate)
Register (Register)
Data Type Name
Direct
Computed rate from DYB−NSO

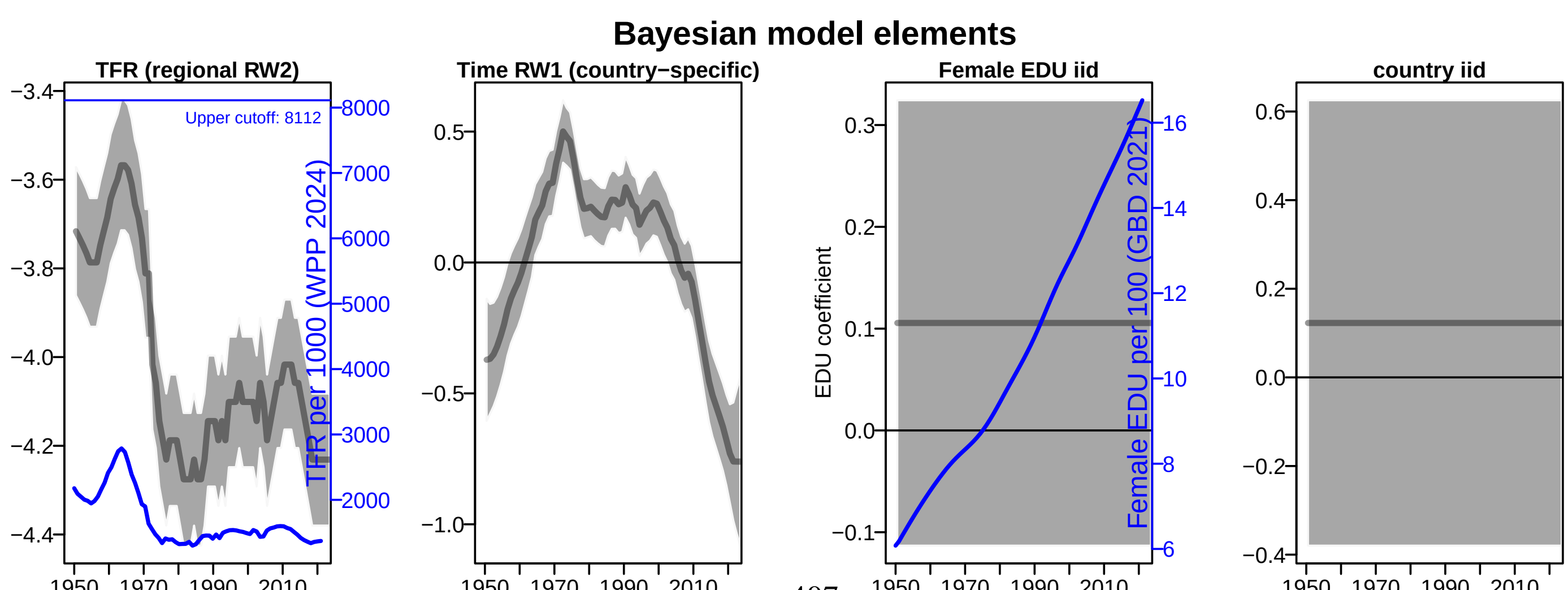
Bayesian model elements
TFR (regional RW2)
Upper cutoff: 8112
TFR per 1000 (WPP 2024)
Time RW1 (country−specific)
Female EDU iid
EDU coefficient
Female EDU per 100 (GBD 2021)
country iid

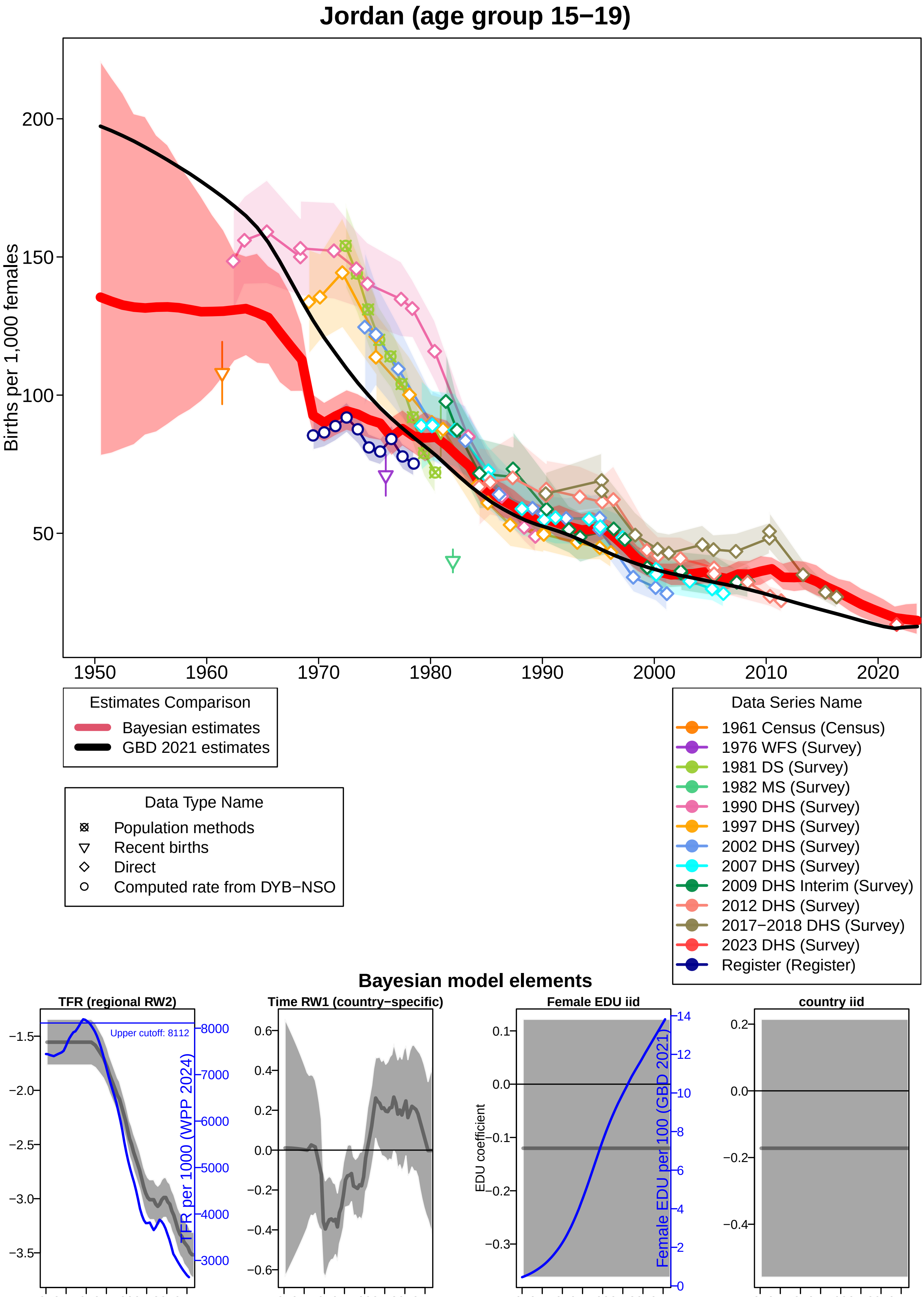
Jordan (age group 15−19)
Births per 1,000 females
200
150
100
50
1950
1960
1970
1980
1990
2000
2010
2020
Estimates Comparison
Bayesian estimates
GBD 2021 estimates
Data Type Name
Population methods
Recent births
Direct
Computed rate from DYB−NSO
Data Series Name
1961 Census (Census)
1976 WFS (Survey)
1981 DS (Survey)
1982 MS (Survey)
1990 DHS (Survey)
1997 DHS (Survey)
2002 DHS (Survey)
2007 DHS (Survey)
2009 DHS Interim (Survey)
2012 DHS (Survey)
2017−2018 DHS (Survey)
2023 DHS (Survey)
Register (Register)
Bayesian model elements
TFR (regional RW2)
Upper cutoff: 8112
TFR per 1000 (WPP 2024)
Time RW1 (country−specific)
Female EDU iid
EDU coefficient
Female EDU per 100 (GBD 2021)
country iid

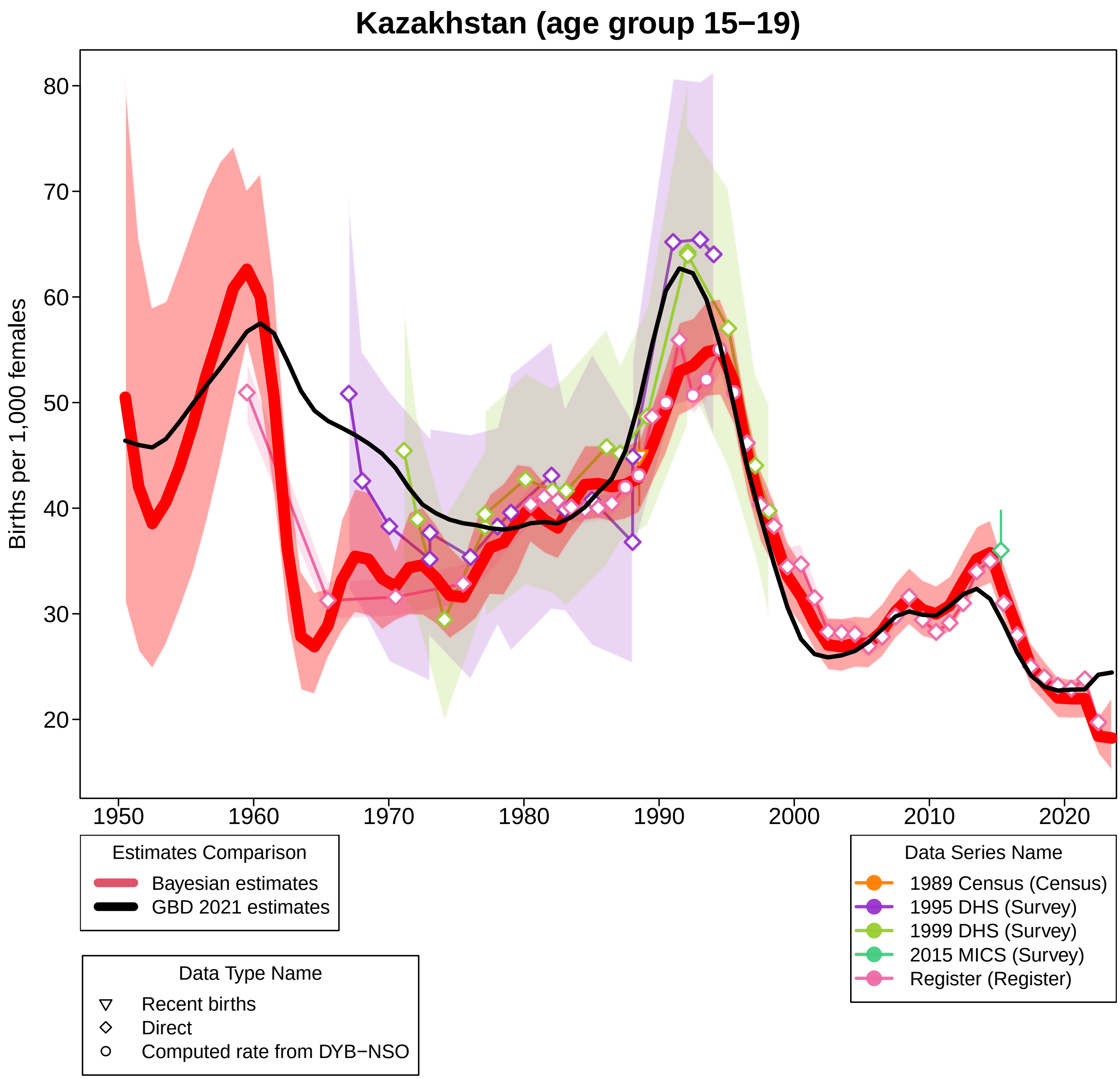
Kazakhstan (age group 15−19)
Births per 1,000 females
80
70
60
50
40
30
20
1950
1960
1970
1980
1990
2000
2010
2020
Estimates Comparison
Bayesian estimates
GBD 2021 estimates
Data Series Name
1989 Census (Census)
1995 DHS (Survey)
1999 DHS (Survey)
2015 MICS (Survey)
Register (Register)
Data Type Name
Recent births
Direct
Computed rate from DYB−NSO

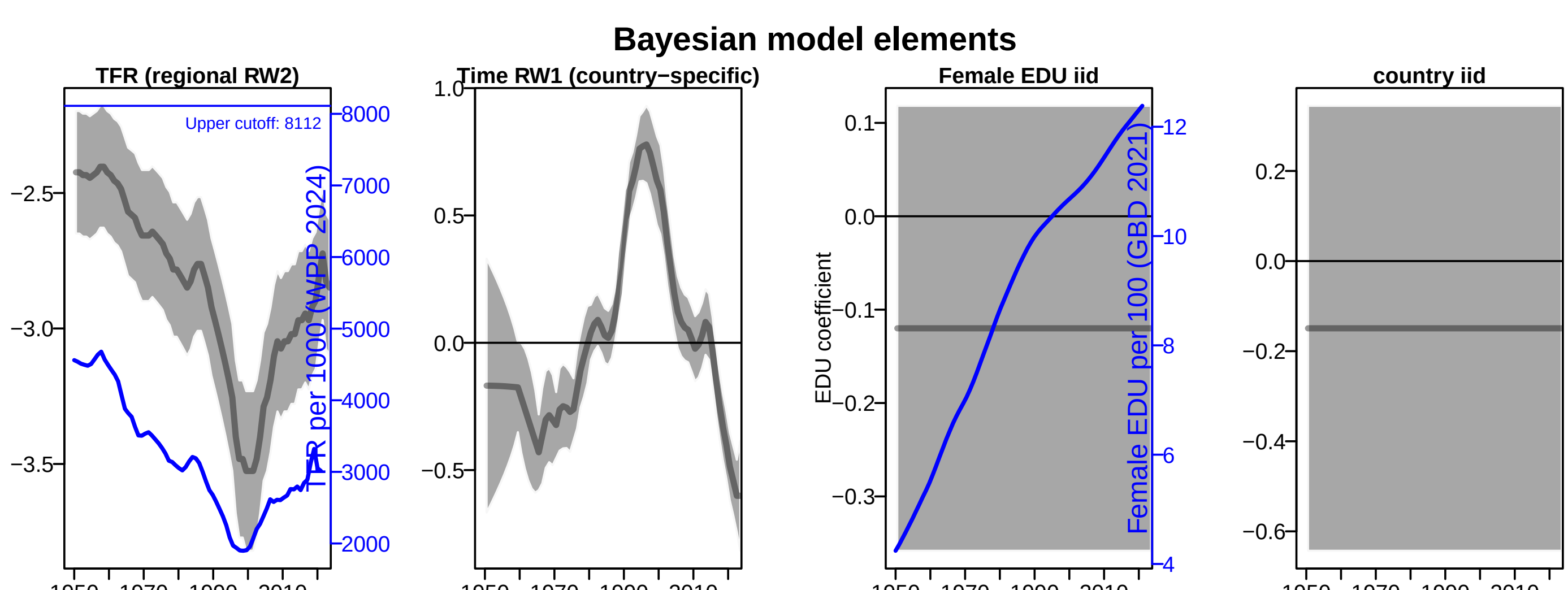
Bayesian model elements
TFR (regional RW2)
Upper cutoff: 8112
TFR per 1000 (WPP 2024)
Time RW1 (country−specific)
Female EDU iid
EDU coefficient
Female EDU per 100 (GBD 2021)
country iid

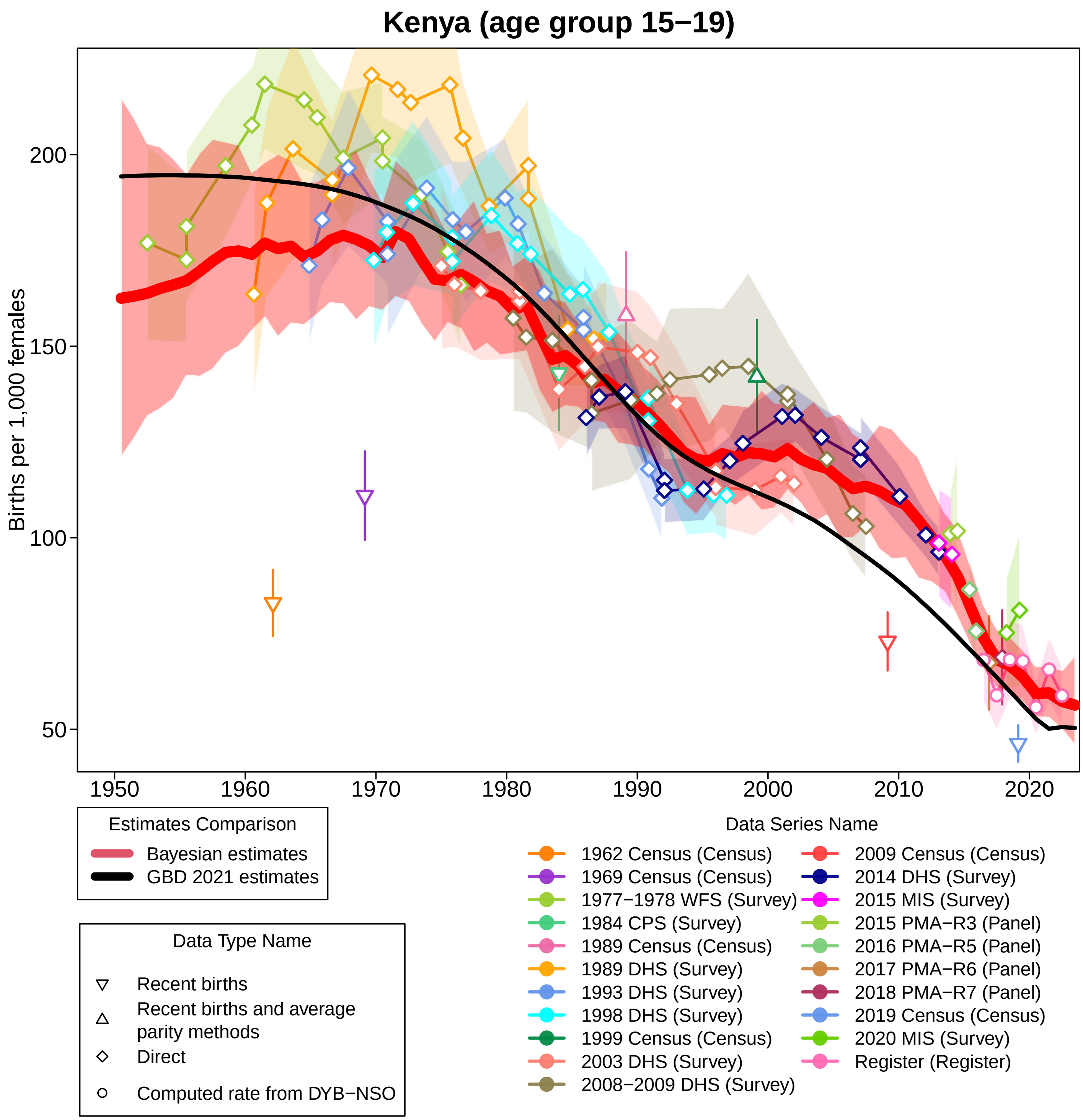
Kenya (age group 15−19)
Births per 1,000 females
200
150
100
50
1950
1960
1970
1980
1990
2000
2010
2020
Estimates Comparison
Bayesian estimates
GBD 2021 estimates
Data Type Name
Recent births
Recent births and average parity methods
Direct
Computed rate from DYB−NSO
Data Series Name
1962 Census (Census)
1969 Census (Census)
1977−1978 WFS (Survey)
1984 CPS (Survey)
1989 Census (Census)
1989 DHS (Survey)
1993 DHS (Survey)
1998 DHS (Survey)
1999 Census (Census)
2003 DHS (Survey)
2008−2009 DHS (Survey)
2009 Census (Census)
2014 DHS (Survey)
2015 MIS (Survey)
2015 PMA−R3 (Panel)
2016 PMA−R5 (Panel)
2017 PMA−R6 (Panel)
2018 PMA−R7 (Panel)
2019 Census (Census)
2020 MIS (Survey)
Register (Register)

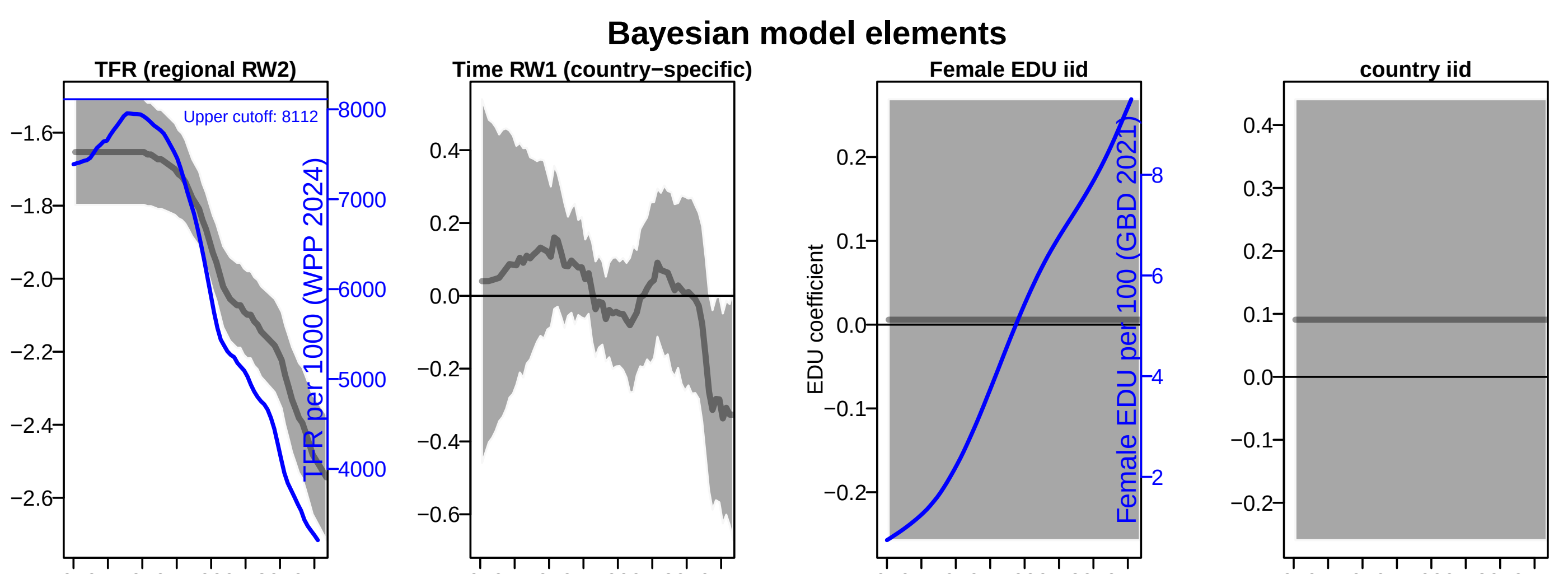
Bayesian model elements
TFR (regional RW2)
Upper cutoff: 8112
TFR per 1000 (WPP 2024)
Time RW1 (country−specific)
Female EDU iid
EDU coefficient
Female EDU per 100 (GBD 2021)
country iid

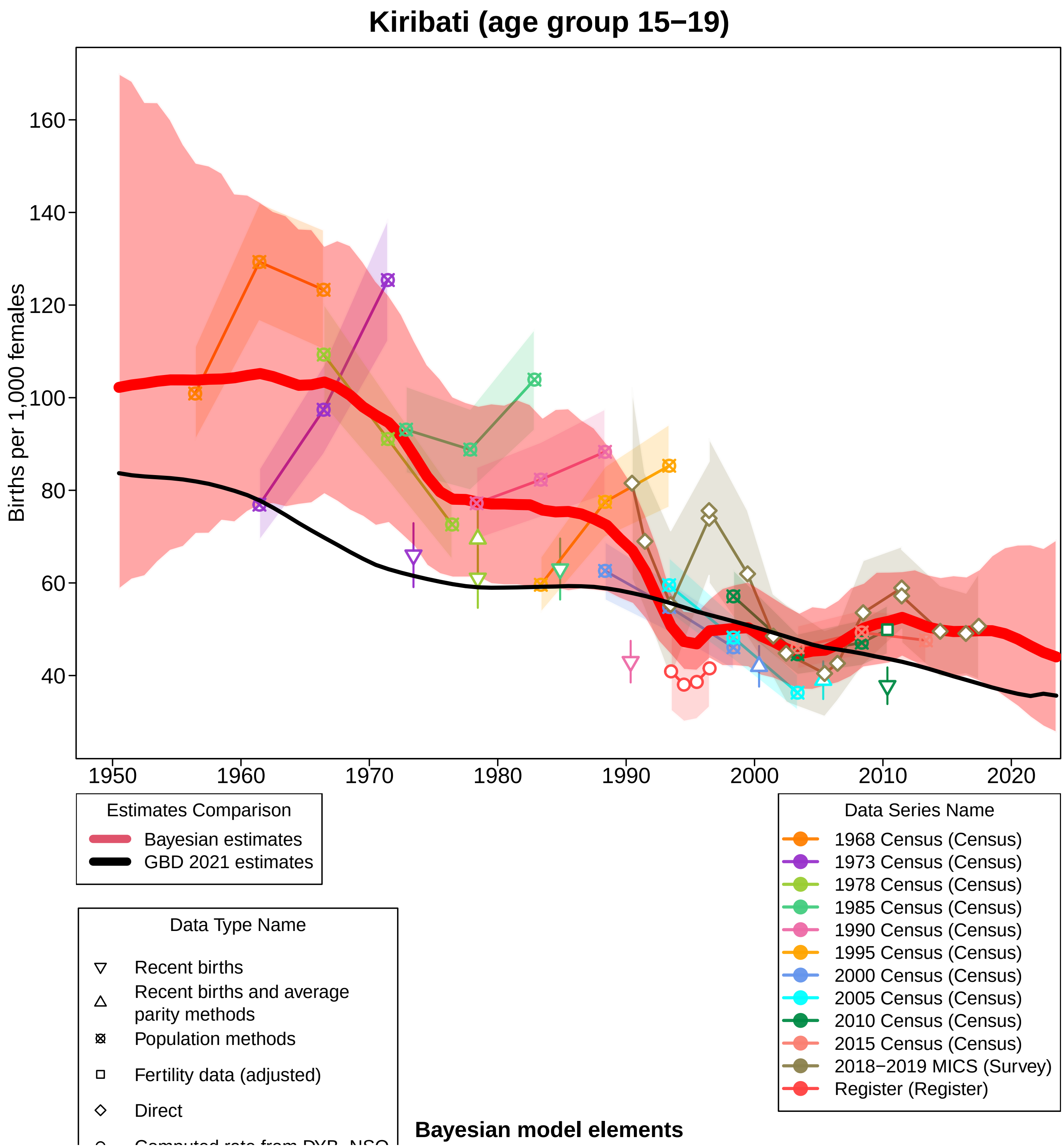

Kiribati (age group 15−19)
Births per 1,000 females
Estimates Comparison
Bayesian estimates
GBD 2021 estimates
Data Series Name
1968 Census (Census)
1973 Census (Census)
1978 Census (Census)
1985 Census (Census)
1990 Census (Census)
1995 Census (Census)
2000 Census (Census)
2005 Census (Census)
2010 Census (Census)
2015 Census (Census)
2018−2019 MICS (Survey)
Register (Register)
Data Type Name
Recent births
Recent births and average parity methods
Population methods
Fertility data (adjusted)
Direct

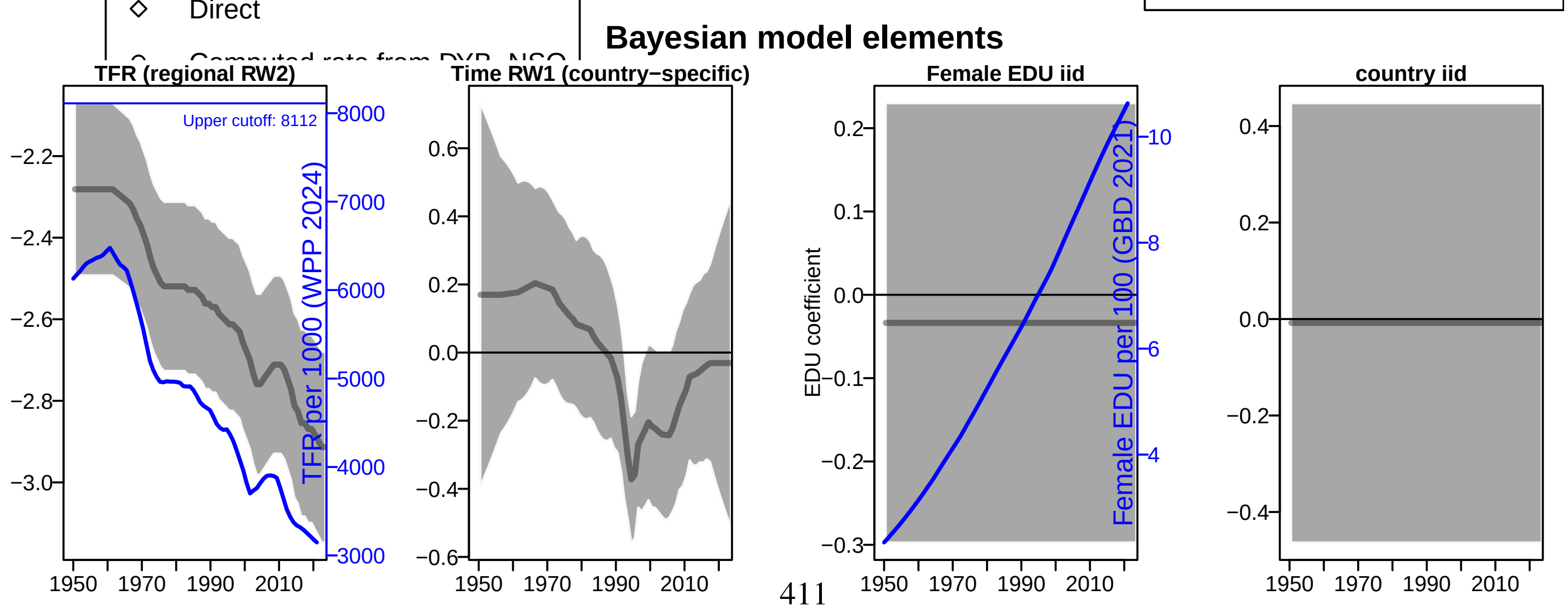

Bayesian model elements
TFR (regional RW2)
Upper cutoff: 8112
TFR per 1000 (WPP 2024)
Time RW1 (country−specific)
Female EDU iid
EDU coefficient
Female EDU per 100 (GBD 2021)
country iid

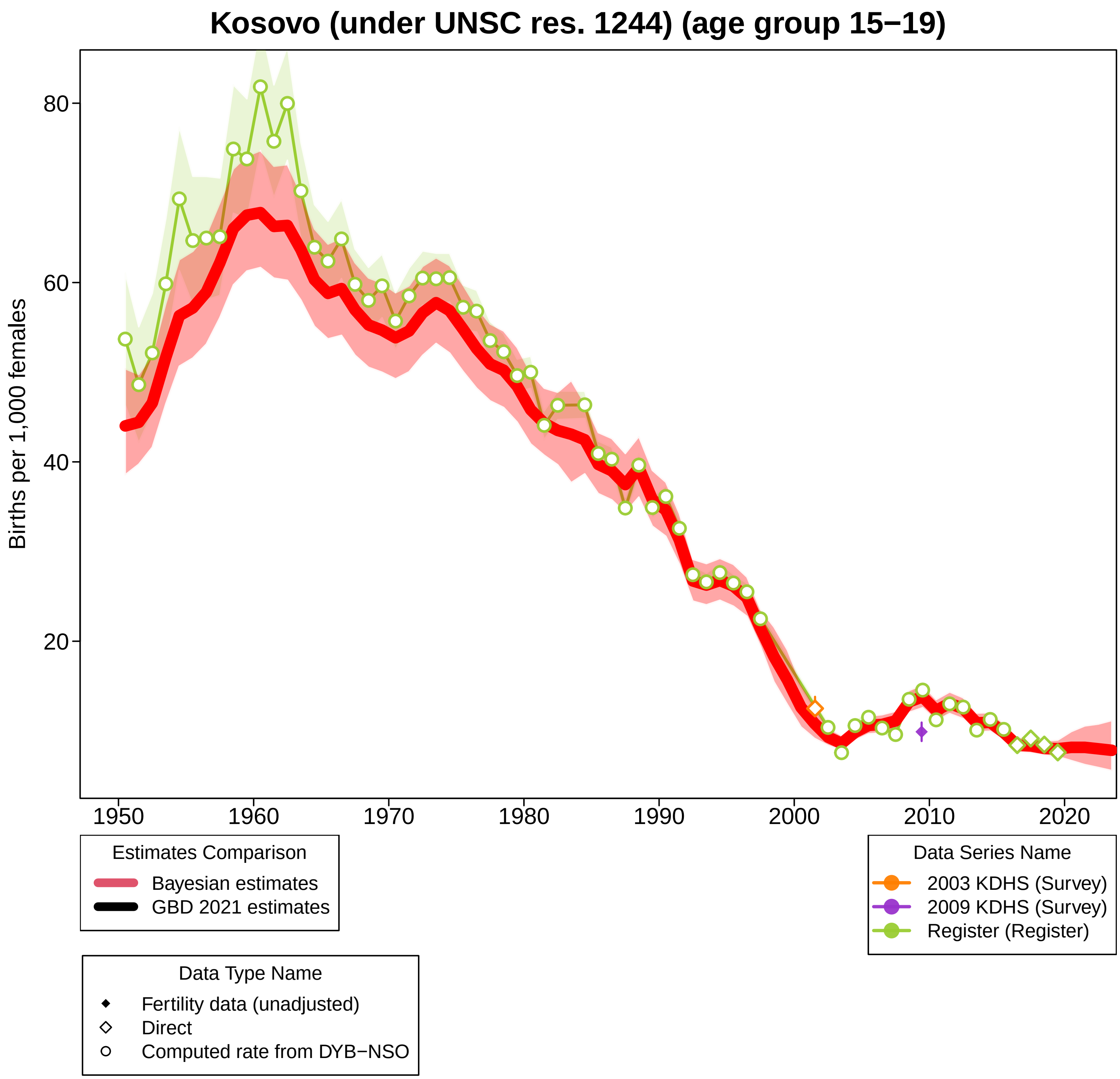
Kosovo (under UNSC res. 1244) (age group 15−19)
Births per 1,000 females
80
60
40
20
1950
1960
1970
1980
1990
2000
2010
2020
Estimates Comparison
Bayesian estimates
GBD 2021 estimates
Data Series Name
2003 KDHS (Survey)
2009 KDHS (Survey)
Register (Register)
Data Type Name
Fertility data (unadjusted)
Direct
Computed rate from DYB−NSO

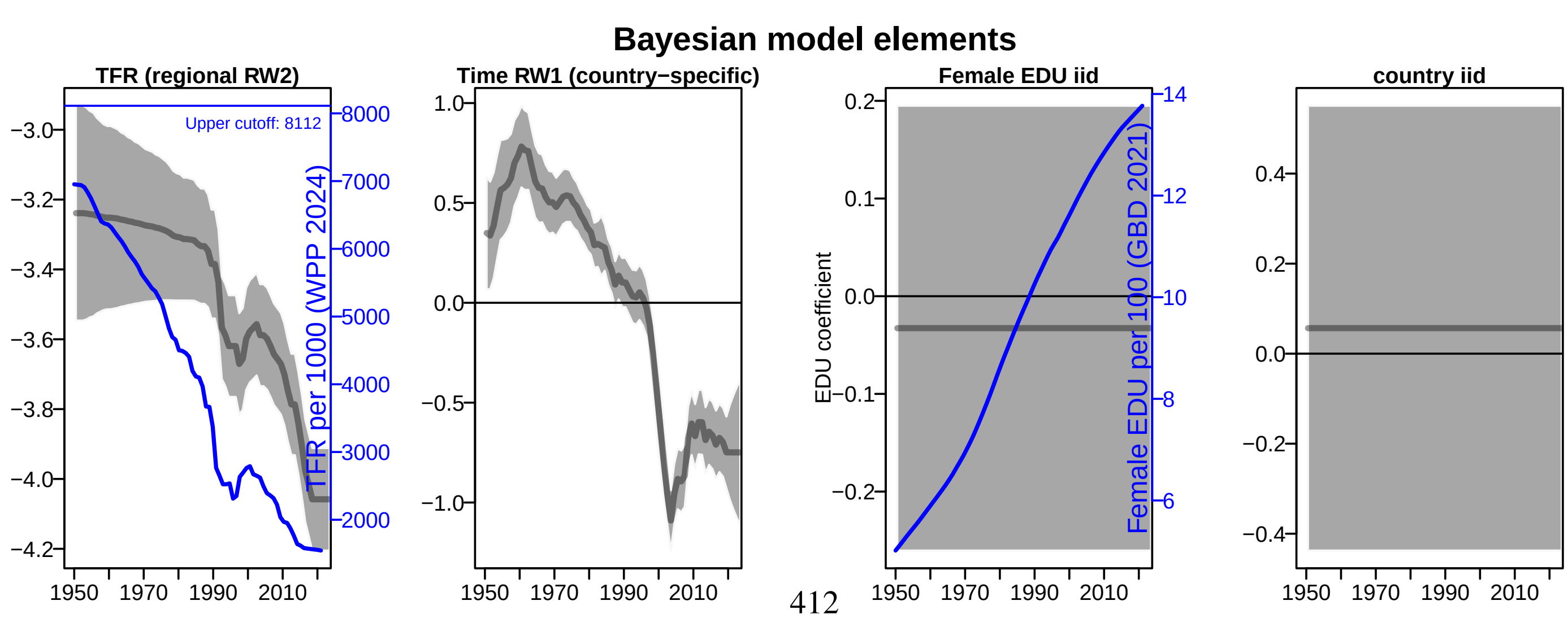
Bayesian model elements
TFR (regional RW2)
Upper cutoff: 8112
TFR per 1000 (WPP 2024)
Time RW1 (country−specific)
Female EDU iid
EDU coefficient
Female EDU per 100 (GBD 2021)
country iid

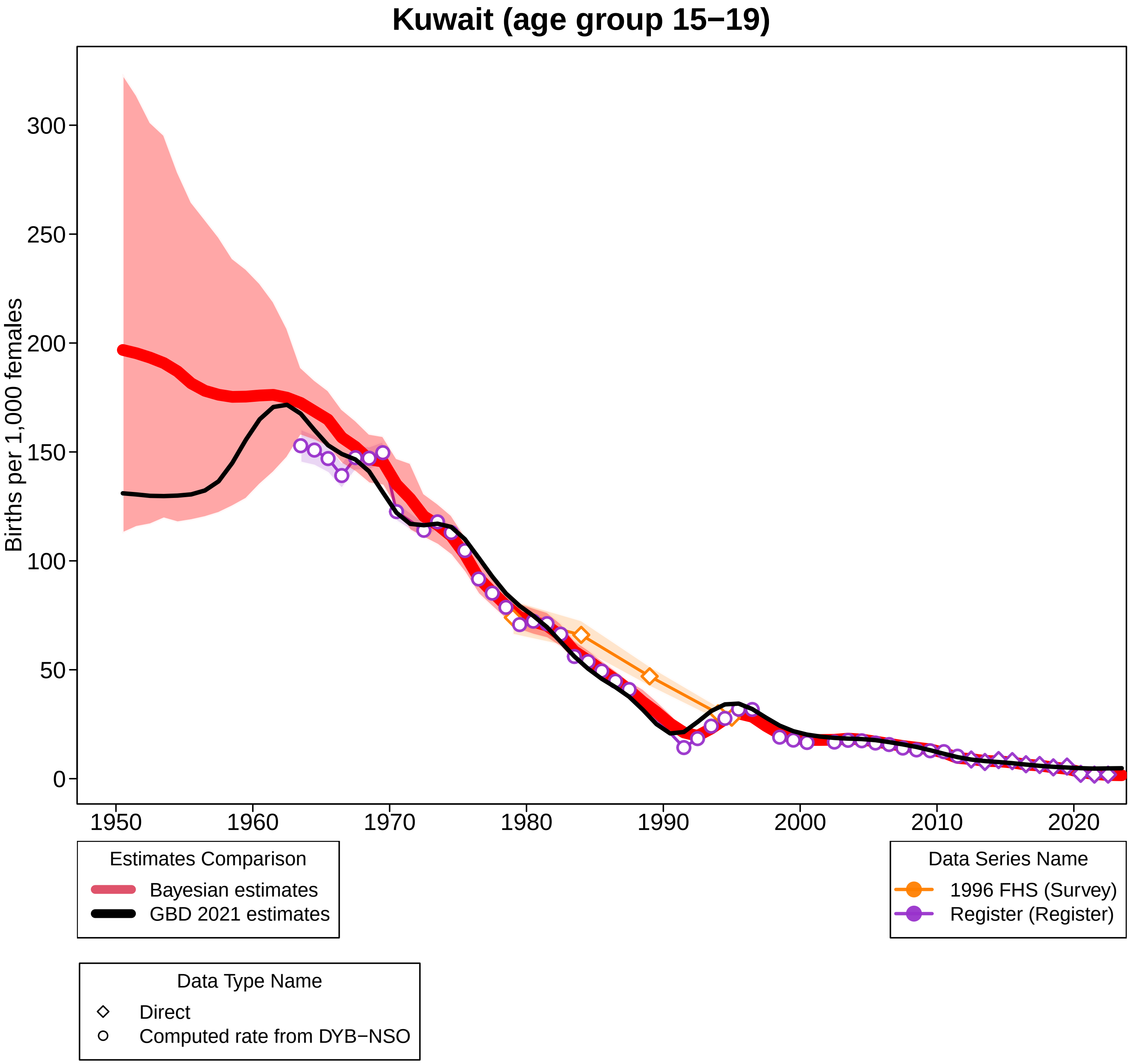

Kuwait (age group 15−19)
Births per 1,000 females
300
250
200
150
100
50
0
1950
1960
1970
1980
1990
2000
2010
2020
Estimates Comparison
Bayesian estimates
GBD 2021 estimates
Data Series Name
1996 FHS (Survey)
Register (Register)
Data Type Name
Direct
Computed rate from DYB−NSO

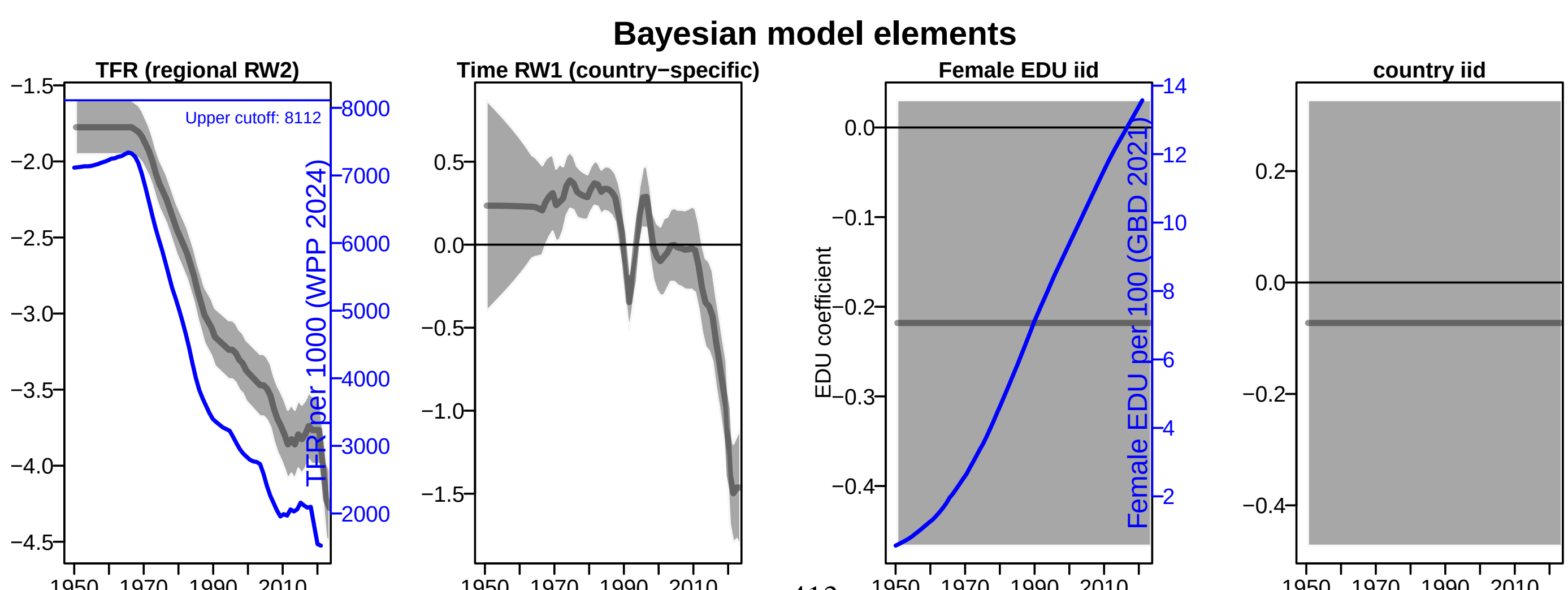

Bayesian model elements
TFR (regional RW2)
Upper cutoff: 8112
TFR per 1000 (WPP 2024)
Time RW1 (country−specific)
Female EDU iid
EDU coefficient
Female EDU per 100 (GBD 2021)
country iid

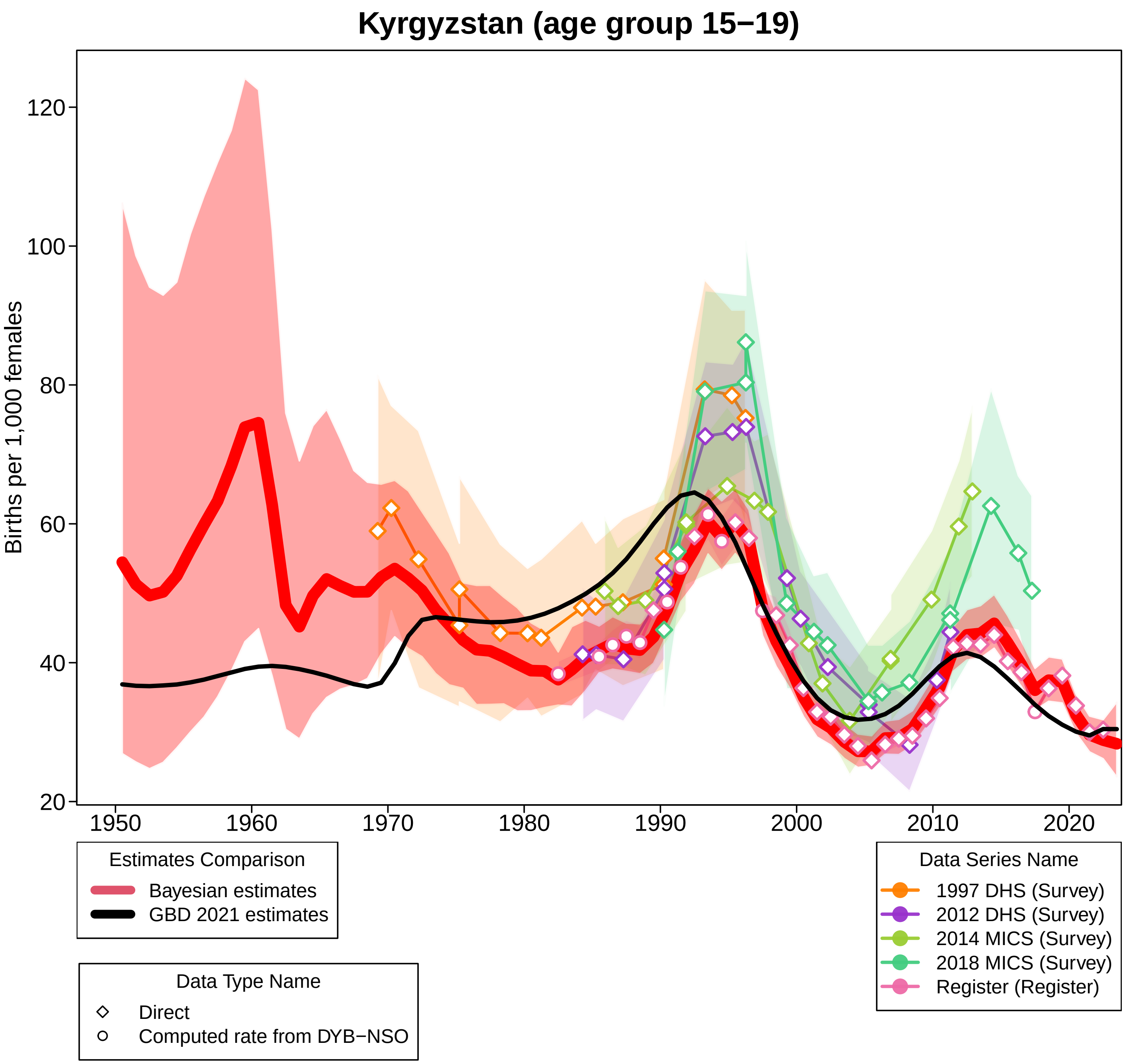
Kyrgyzstan (age group 15−19)
Births per 1,000 females
120
100
80
60
40
20
1950
1960
1970
1980
1990
2000
2010
2020
Estimates Comparison
Bayesian estimates
GBD 2021 estimates
Data Series Name
1997 DHS (Survey)
2012 DHS (Survey)
2014 MICS (Survey)
2018 MICS (Survey)
Register (Register)
Data Type Name
Direct
Computed rate from DYB−NSO

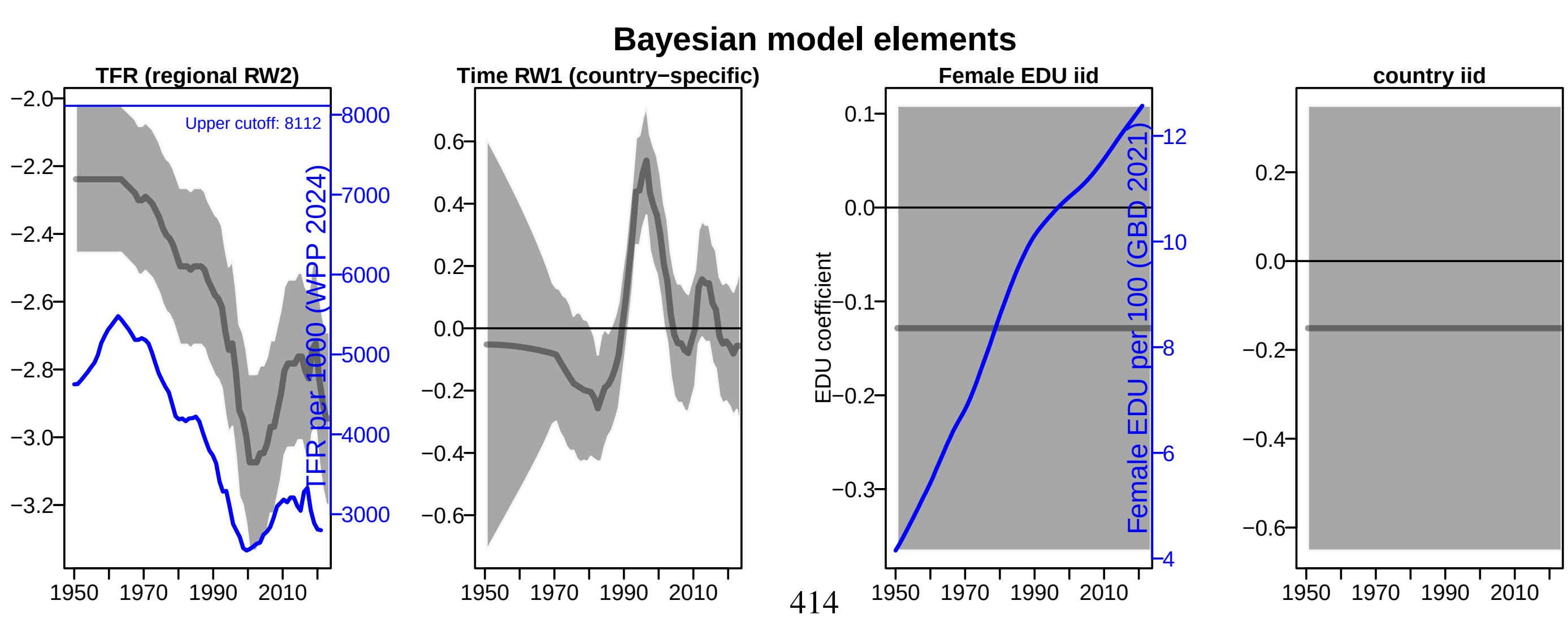
Bayesian model elements
TFR (regional RW2)
Upper cutoff: 8112
TFR per 1000 (WPP 2024)
Time RW1 (country−specific)
Female EDU iid
EDU coefficient
Female EDU per 100 (GBD 2021)
country iid

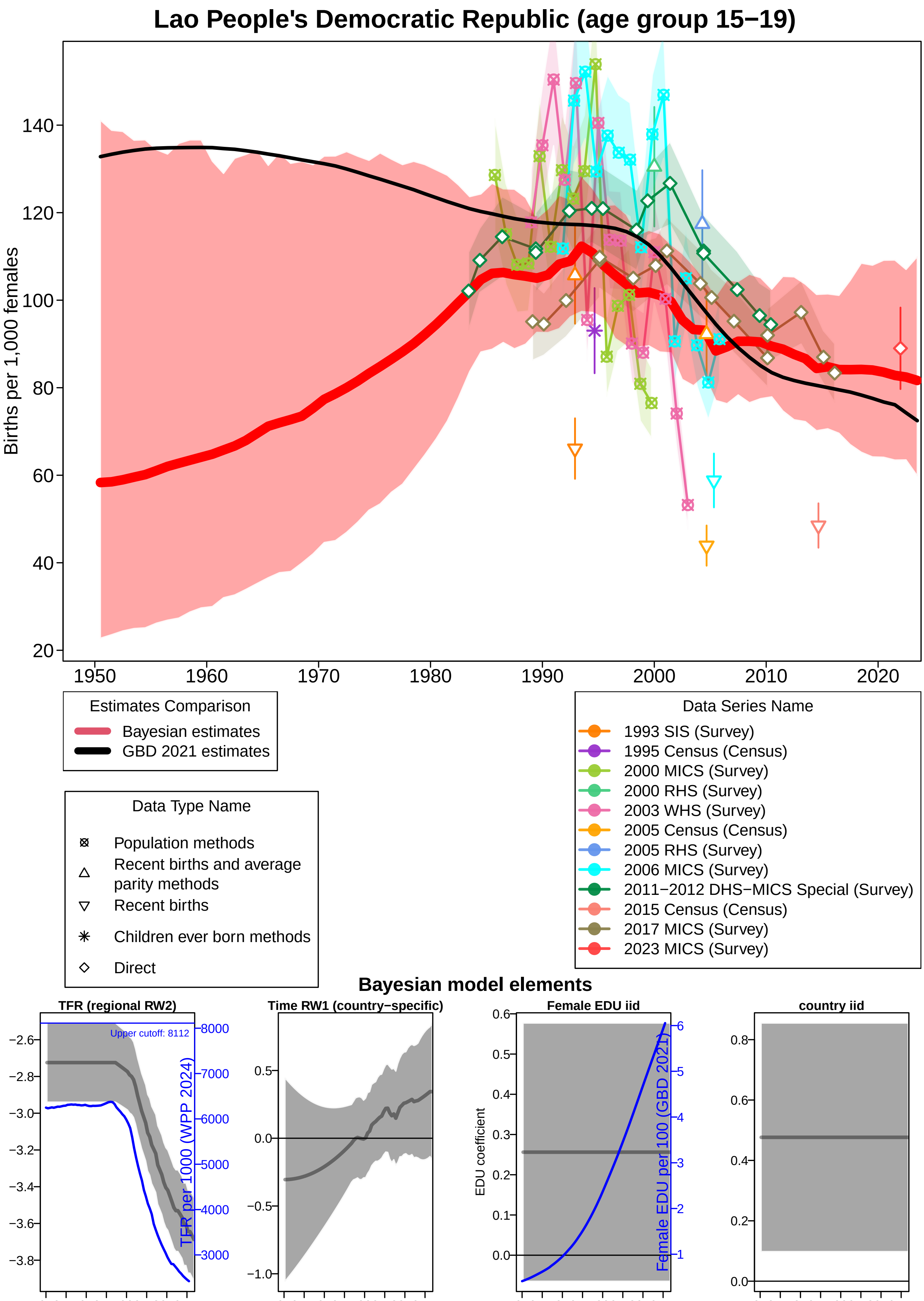
Lao People's Democratic Republic (age group 15−19)
Births per 1,000 females
Estimates Comparison
Bayesian estimates
GBD 2021 estimates
Data Type Name
Population methods
Recent births and average parity methods
Recent births
Children ever born methods
Direct
Data Series Name
1993 SIS (Survey)
1995 Census (Census)
2000 MICS (Survey)
2000 RHS (Survey)
2003 WHS (Survey)
2005 Census (Census)
2005 RHS (Survey)
2006 MICS (Survey)
2011−2012 DHS−MICS Special (Survey)
2015 Census (Census)
2017 MICS (Survey)
2023 MICS (Survey)
Bayesian model elements
TFR (regional RW2)
Upper cutoff: 8112
TFR per 1000 (WPP 2024)
Time RW1 (country−specific)
Female EDU iid
EDU coefficient
Female EDU per 100 (GBD 2021)
country iid

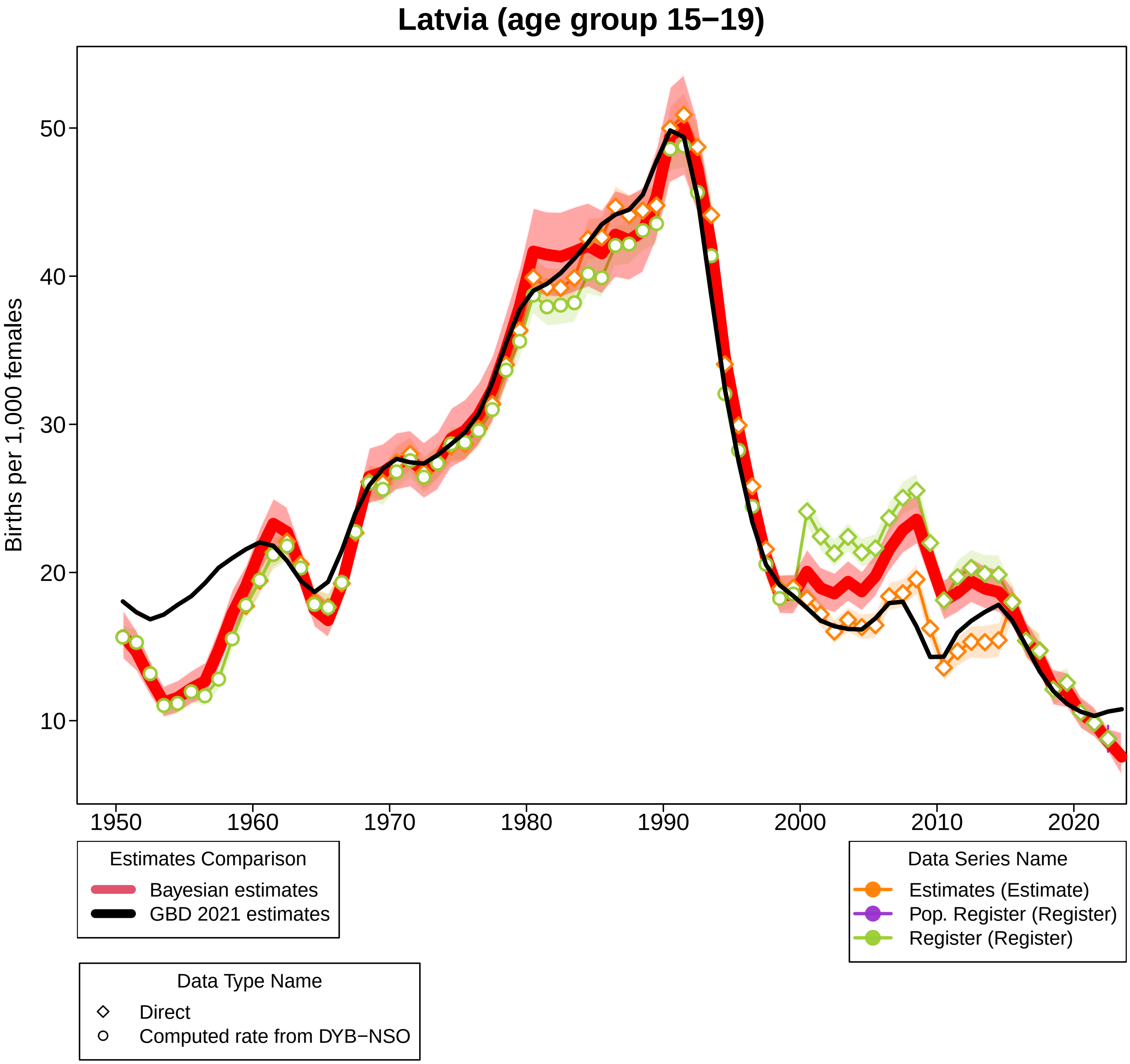
Latvia (age group 15−19)
Births per 1,000 females
50
40
30
20
10
1950
1960
1970
1980
1990
2000
2010
2020
Estimates Comparison
Bayesian estimates
GBD 2021 estimates
Data Series Name
Estimates (Estimate)
Pop. Register (Register)
Register (Register)
Data Type Name
Direct
Computed rate from DYB−NSO

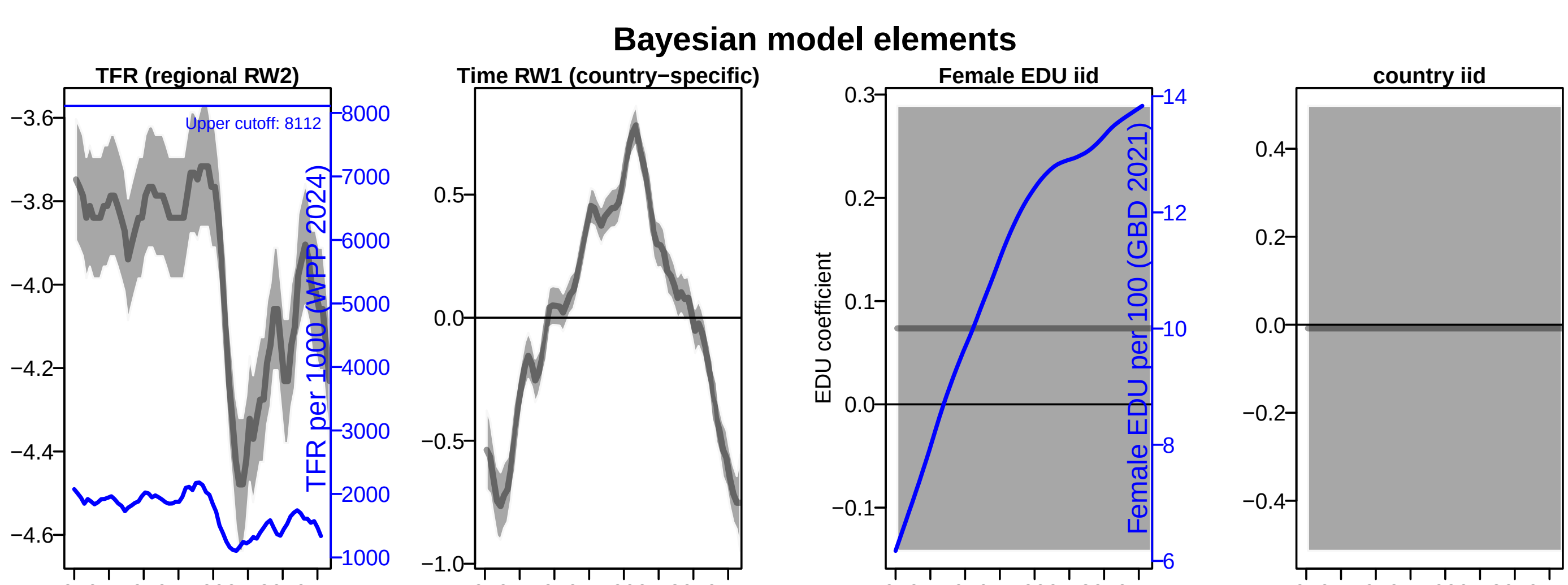
Bayesian model elements
TFR (regional RW2)
Upper cutoff: 8112
TFR per 1000 (WPP 2024)
Time RW1 (country−specific)
Female EDU iid
EDU coefficient
Female EDU per 100 (GBD 2021)
country iid
1950
1970
1990
2010

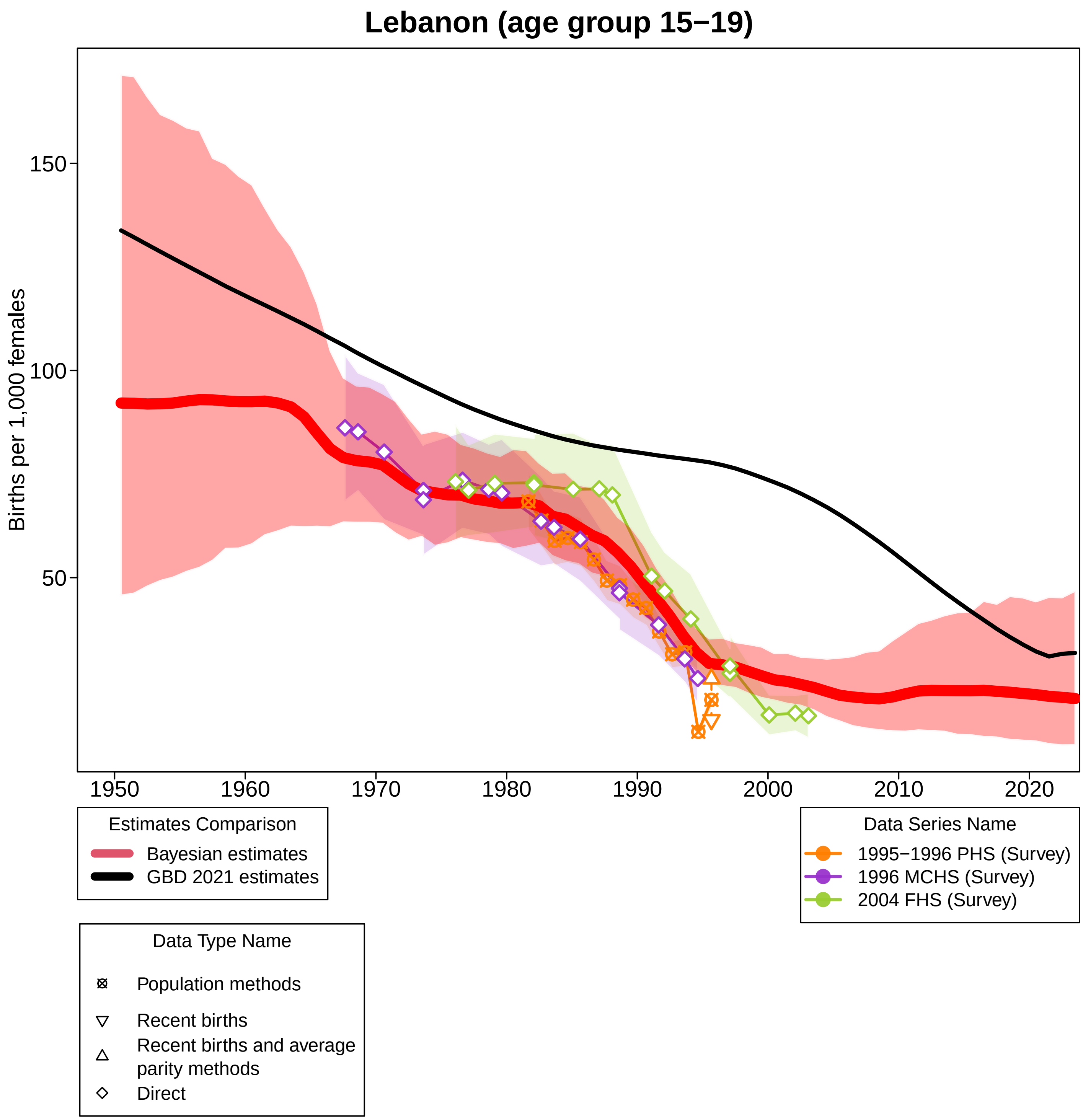
Lebanon (age group 15−19)
Births per 1,000 females
150
100
50
1950
1960
1970
1980
1990
2000
2010
2020
Estimates Comparison
Bayesian estimates
GBD 2021 estimates
Data Series Name
1995−1996 PHS (Survey)
1996 MCHS (Survey)
2004 FHS (Survey)
Data Type Name
Population methods
Recent births
Recent births and average parity methods
Direct

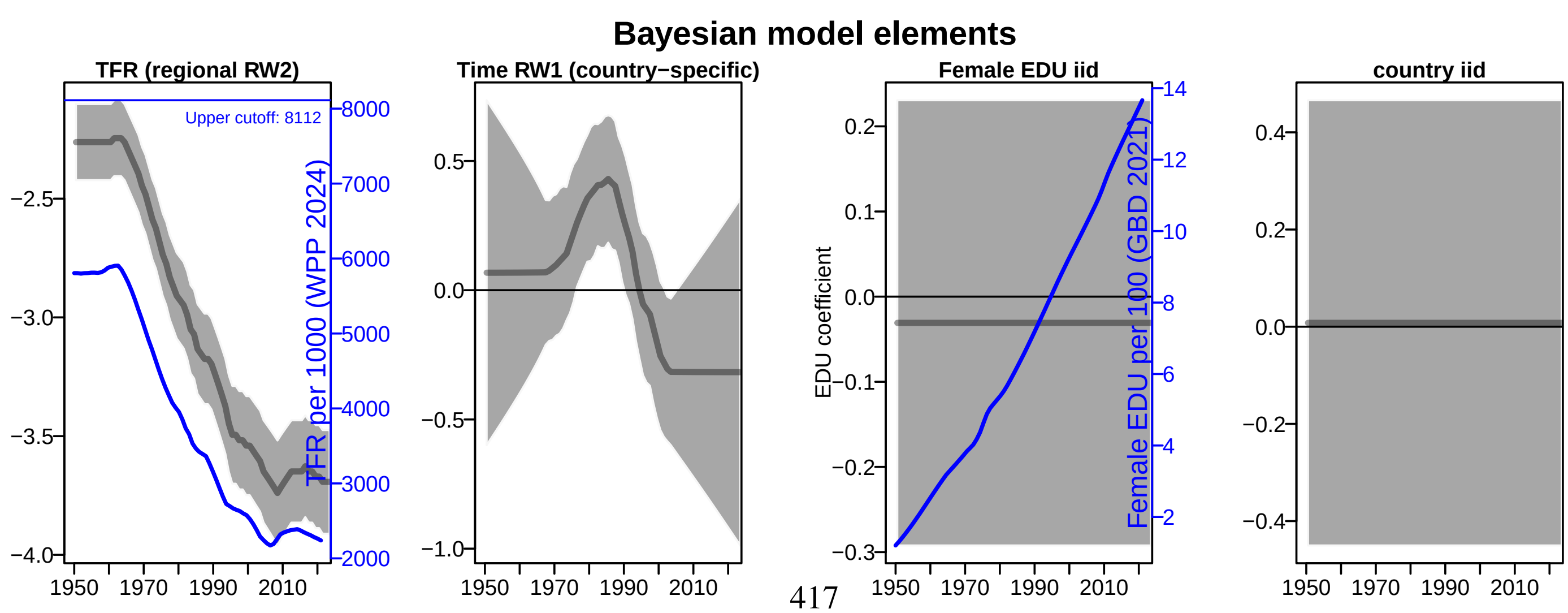
Bayesian model elements
TFR (regional RW2)
Upper cutoff: 8112
TFR per 1000 (WPP 2024)
Time RW1 (country−specific)
Female EDU iid
EDU coefficient
Female EDU per 100 (GBD 2021)
country iid

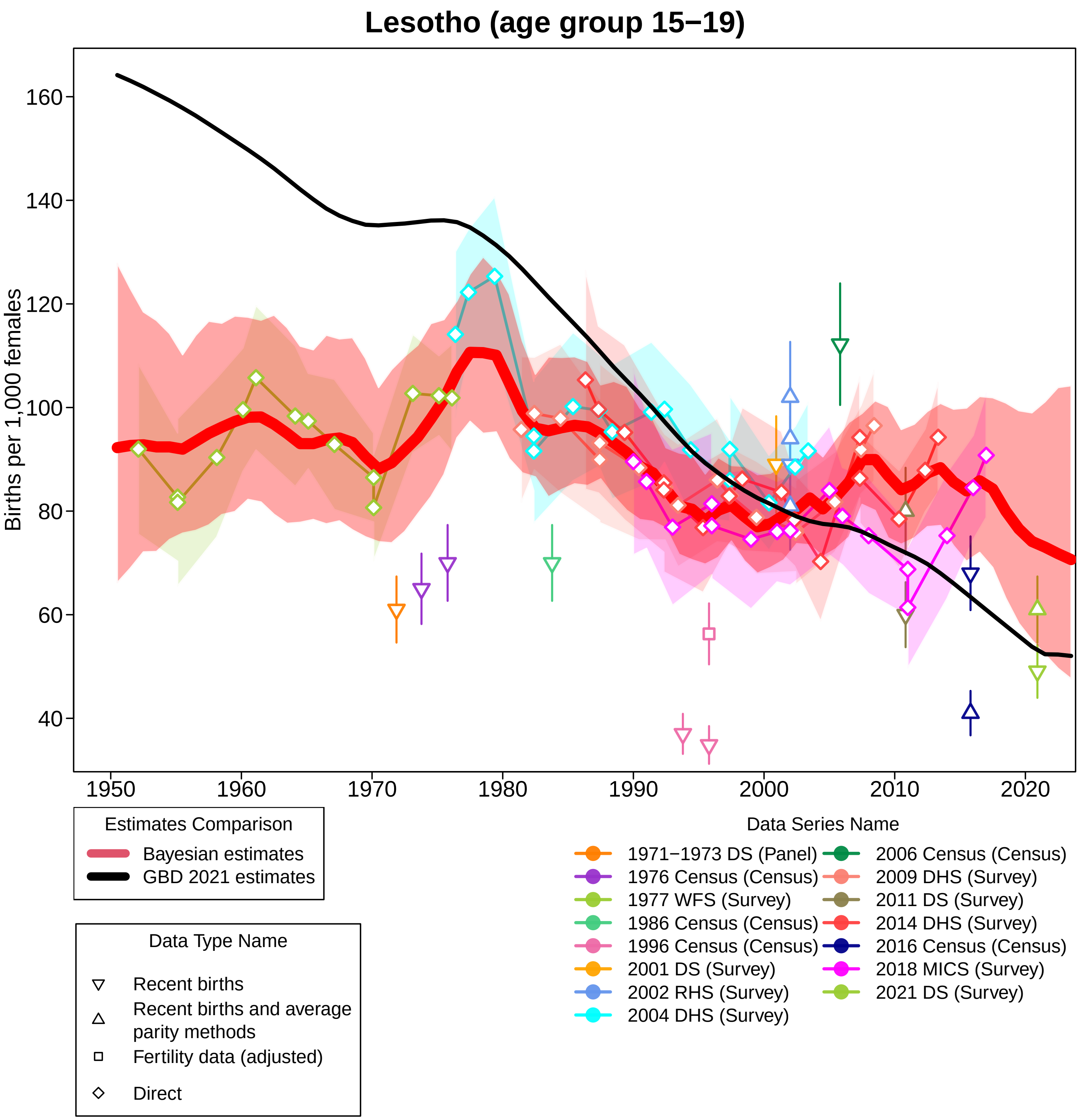
Lesotho (age group 15−19)
Births per 1,000 females
160
140
120
100
80
60
40
1950
1960
1970
1980
1990
2000
2010
2020
Estimates Comparison
Bayesian estimates
GBD 2021 estimates
Data Type Name
Recent births
Recent births and average parity methods
Fertility data (adjusted)
Direct
Data Series Name
1971−1973 DS (Panel)
1976 Census (Census)
1977 WFS (Survey)
1986 Census (Census)
1996 Census (Census)
2001 DS (Survey)
2002 RHS (Survey)
2004 DHS (Survey)
2006 Census (Census)
2009 DHS (Survey)
2011 DS (Survey)
2014 DHS (Survey)
2016 Census (Census)
2018 MICS (Survey)
2021 DS (Survey)

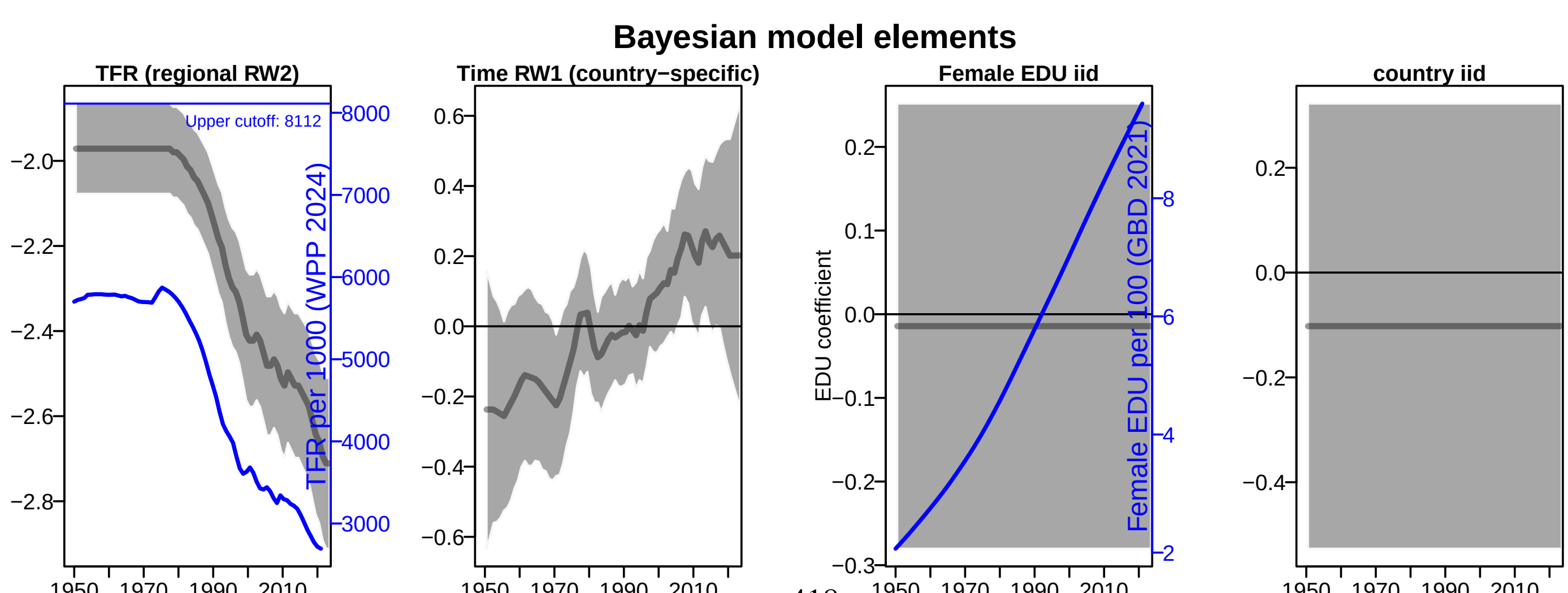
Bayesian model elements
TFR (regional RW2)
Upper cutoff: 8112
TFR per 1000 (WPP 2024)
Time RW1 (country−specific)
Female EDU iid
EDU coefficient
Female EDU per 100 (GBD 2021)
country iid

Liberia (age group 15−19)

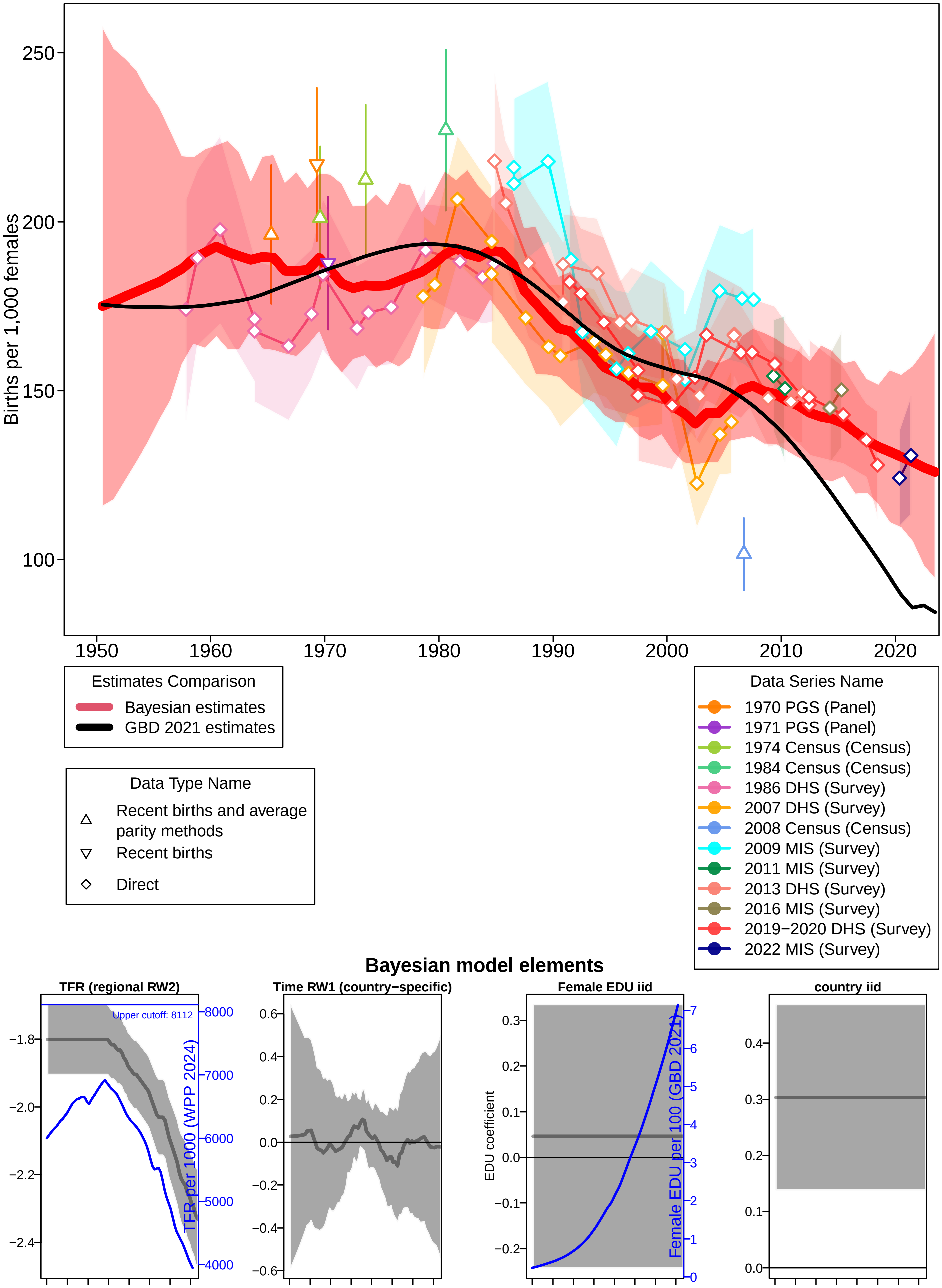

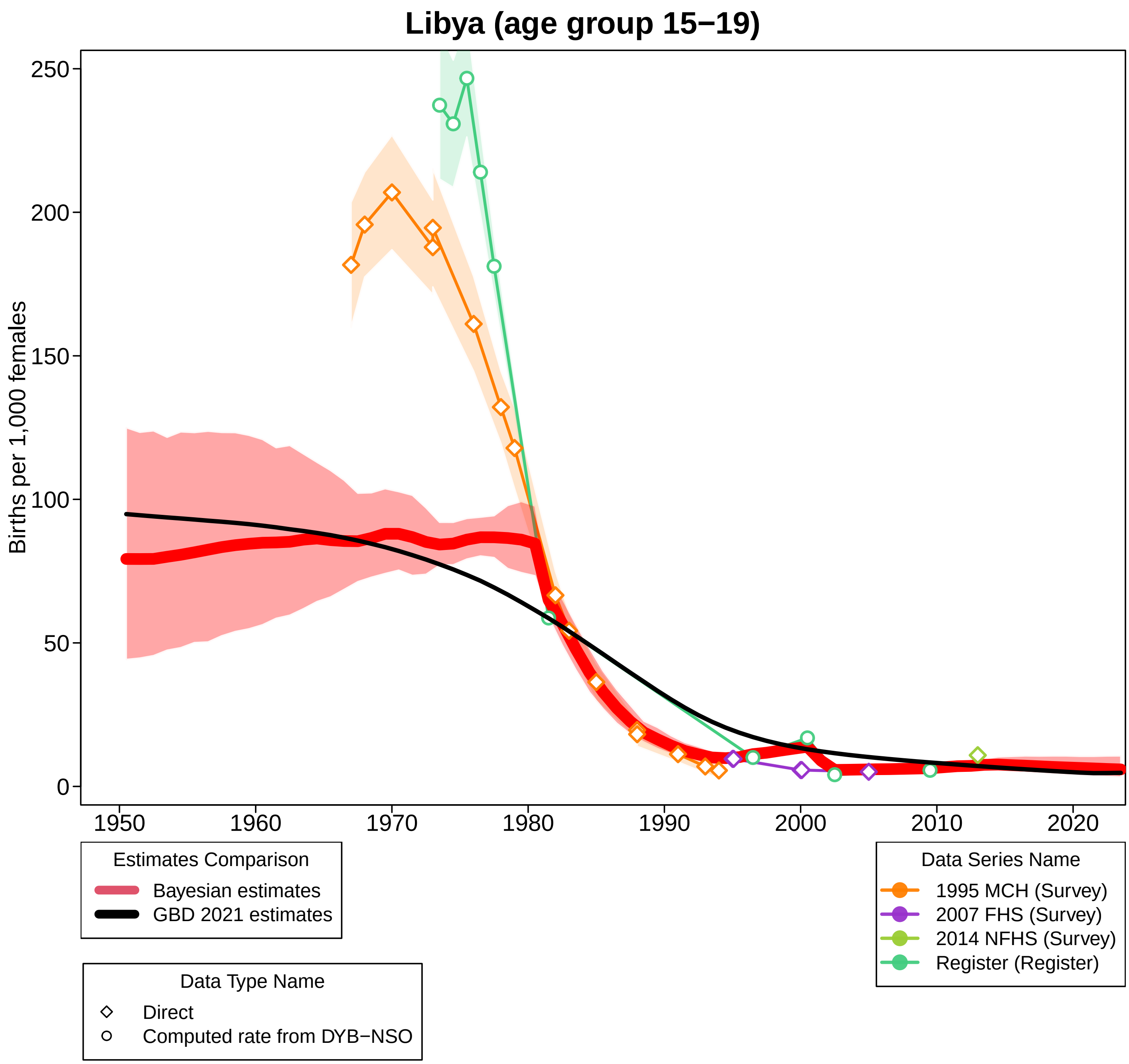
Libya (age group 15−19)
Births per 1,000 females
250
200
150
100
50
0
1950
1960
1970
1980
1990
2000
2010
2020
Estimates Comparison
Bayesian estimates
GBD 2021 estimates
Data Series Name
1995 MCH (Survey)
2007 FHS (Survey)
2014 NFHS (Survey)
Register (Register)
Data Type Name
Direct
Computed rate from DYB−NSO

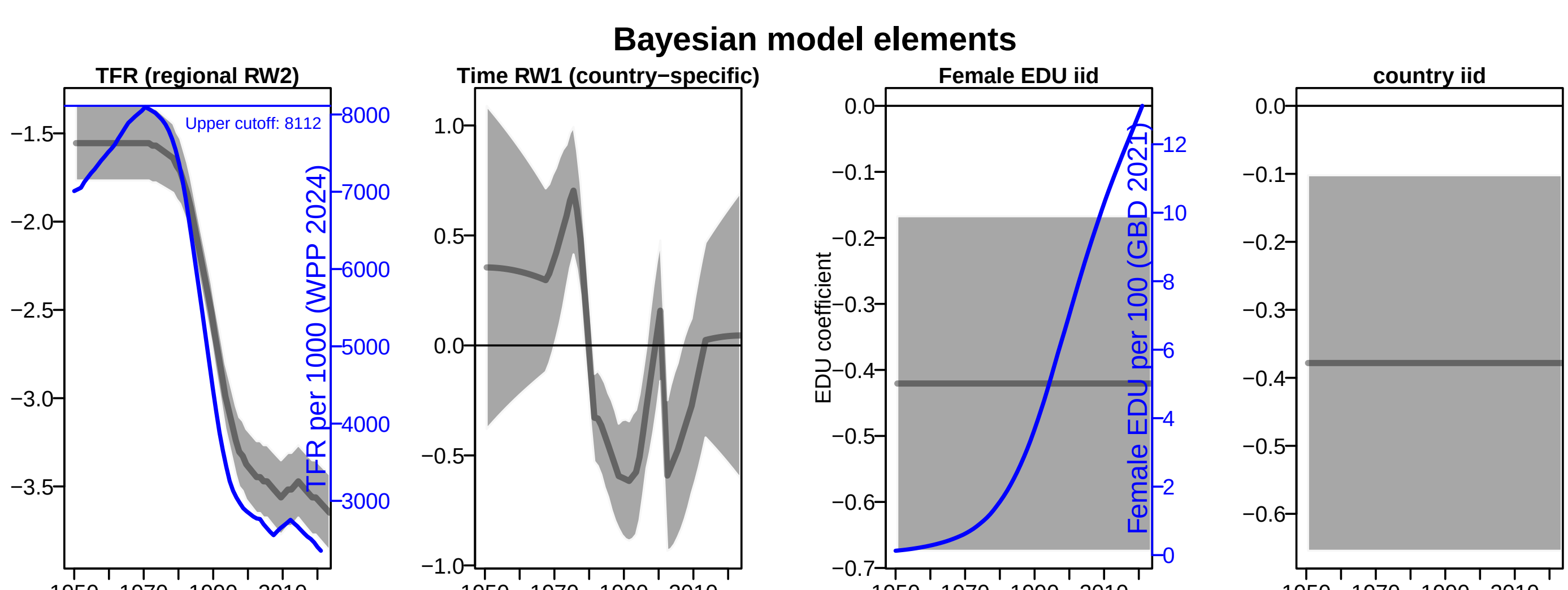
Bayesian model elements
TFR (regional RW2)
Upper cutoff: 8112
TFR per 1000 (WPP 2024)
Time RW1 (country−specific)
Female EDU iid
EDU coefficient
Female EDU per 100 (GBD 2021)
country iid

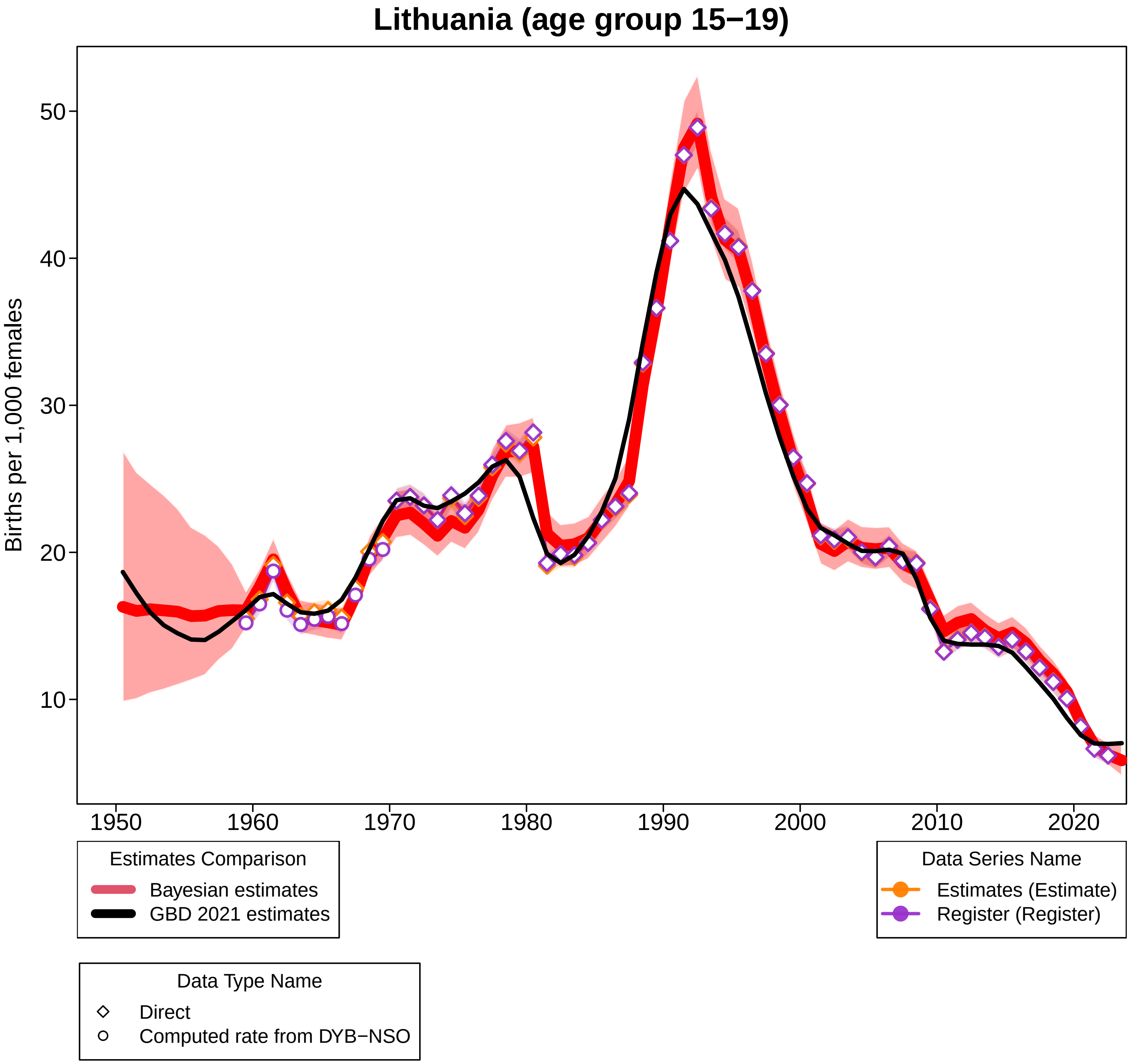
Lithuania (age group 15−19)
Births per 1,000 females
50
40
30
20
10
1950
1960
1970
1980
1990
2000
2010
2020
Estimates Comparison
Bayesian estimates
GBD 2021 estimates
Data Series Name
Estimates (Estimate)
Register (Register)
Data Type Name
Direct
Computed rate from DYB−NSO

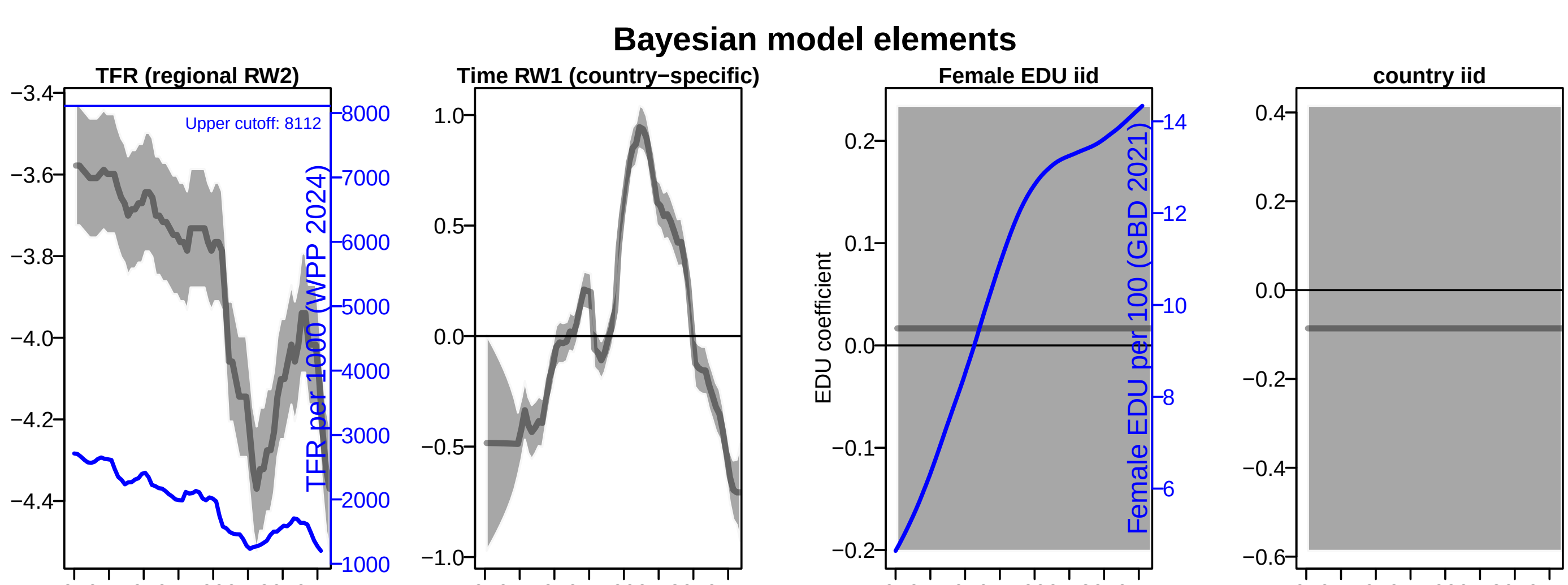
Bayesian model elements
TFR (regional RW2)
Upper cutoff: 8112
TFR per 1000 (WPP 2024)
Time RW1 (country−specific)
Female EDU iid
EDU coefficient
Female EDU per 100 (GBD 2021)
country iid

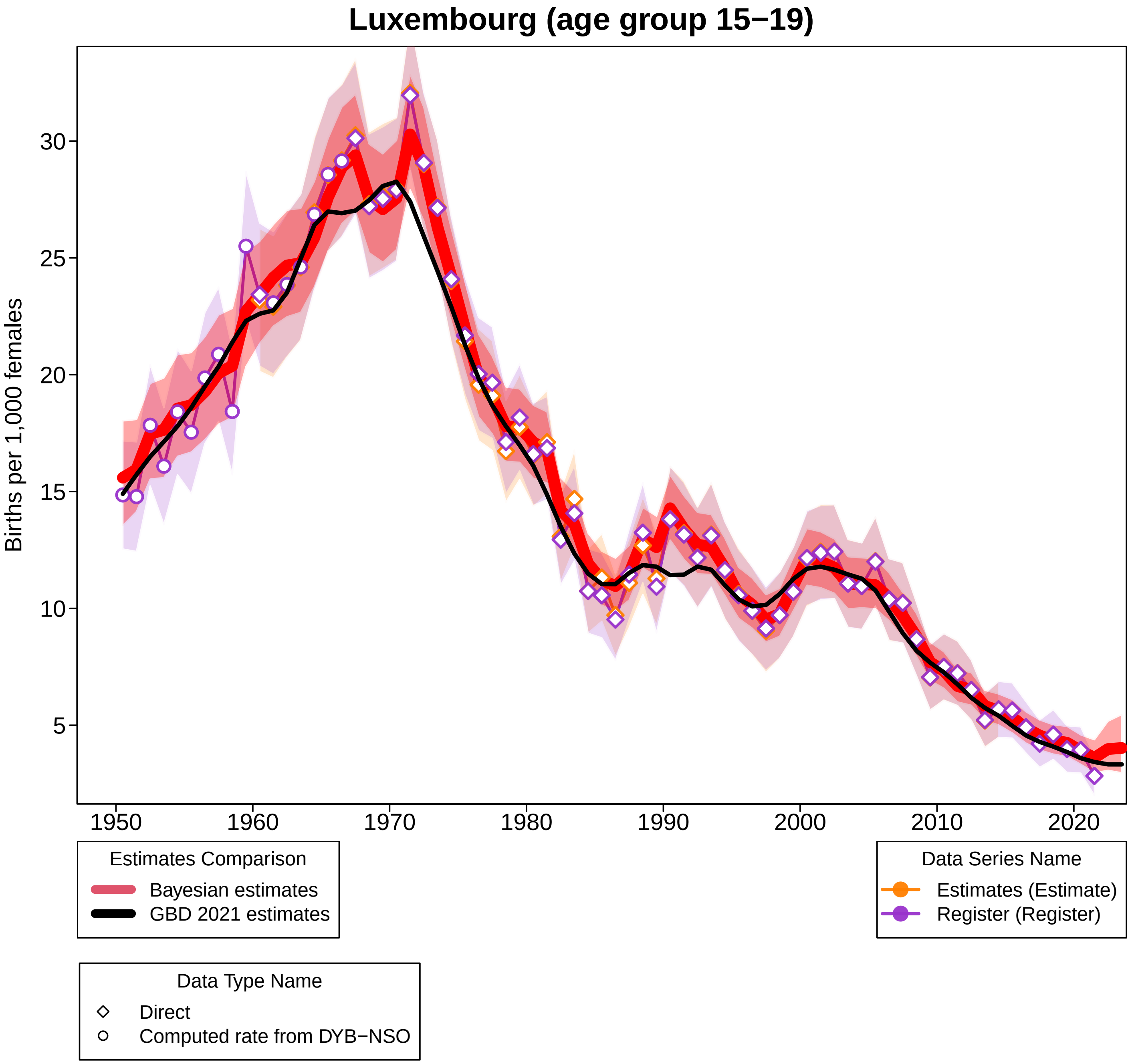
Luxembourg (age group 15−19)
Births per 1,000 females
Estimates Comparison
Bayesian estimates
GBD 2021 estimates
Data Series Name
Estimates (Estimate)
Register (Register)
Data Type Name
Direct
Computed rate from DYB−NSO

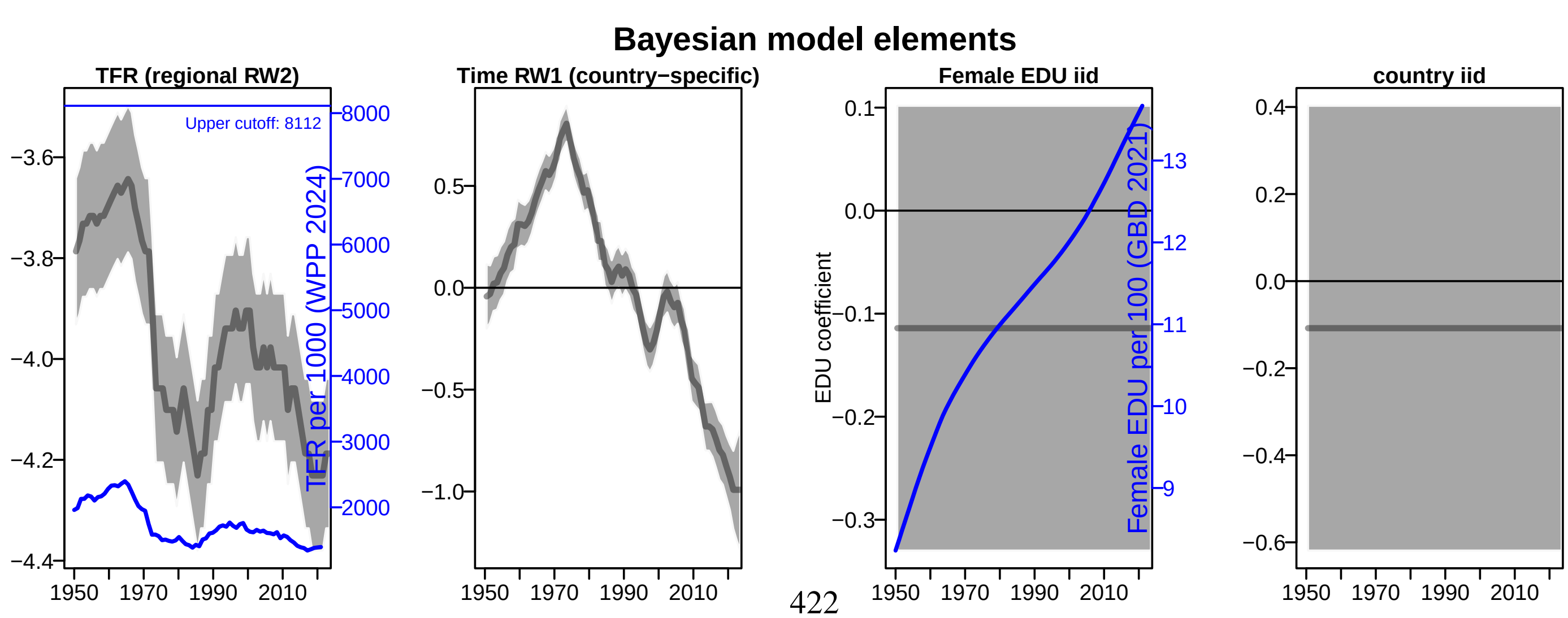
Bayesian model elements
TFR (regional RW2)
Upper cutoff: 8112
TFR per 1000 (WPP 2024)
Time RW1 (country−specific)
Female EDU iid
EDU coefficient
Female EDU per 100 (GBD 2021)
country iid

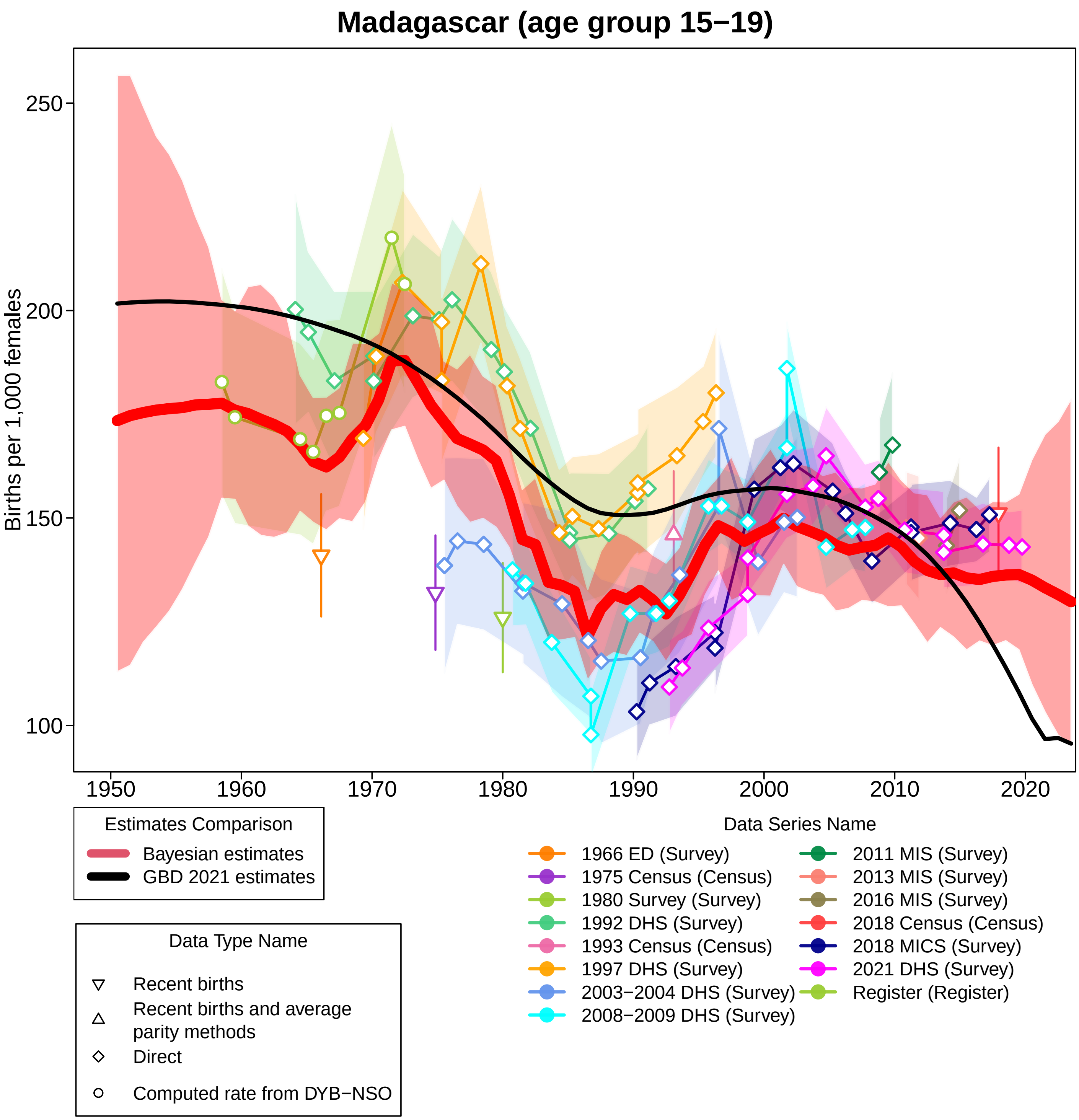
Madagascar (age group 15−19)
Births per 1,000 females
250
200
150
100
1950
1960
1970
1980
1990
2000
2010
2020
Estimates Comparison
Bayesian estimates
GBD 2021 estimates
Data Type Name
Recent births
Recent births and average parity methods
Direct
Computed rate from DYB−NSO
Data Series Name
1966 ED (Survey)
1975 Census (Census)
1980 Survey (Survey)
1992 DHS (Survey)
1993 Census (Census)
1997 DHS (Survey)
2003−2004 DHS (Survey)
2008−2009 DHS (Survey)
2011 MIS (Survey)
2013 MIS (Survey)
2016 MIS (Survey)
2018 Census (Census)
2018 MICS (Survey)
2021 DHS (Survey)
Register (Register)

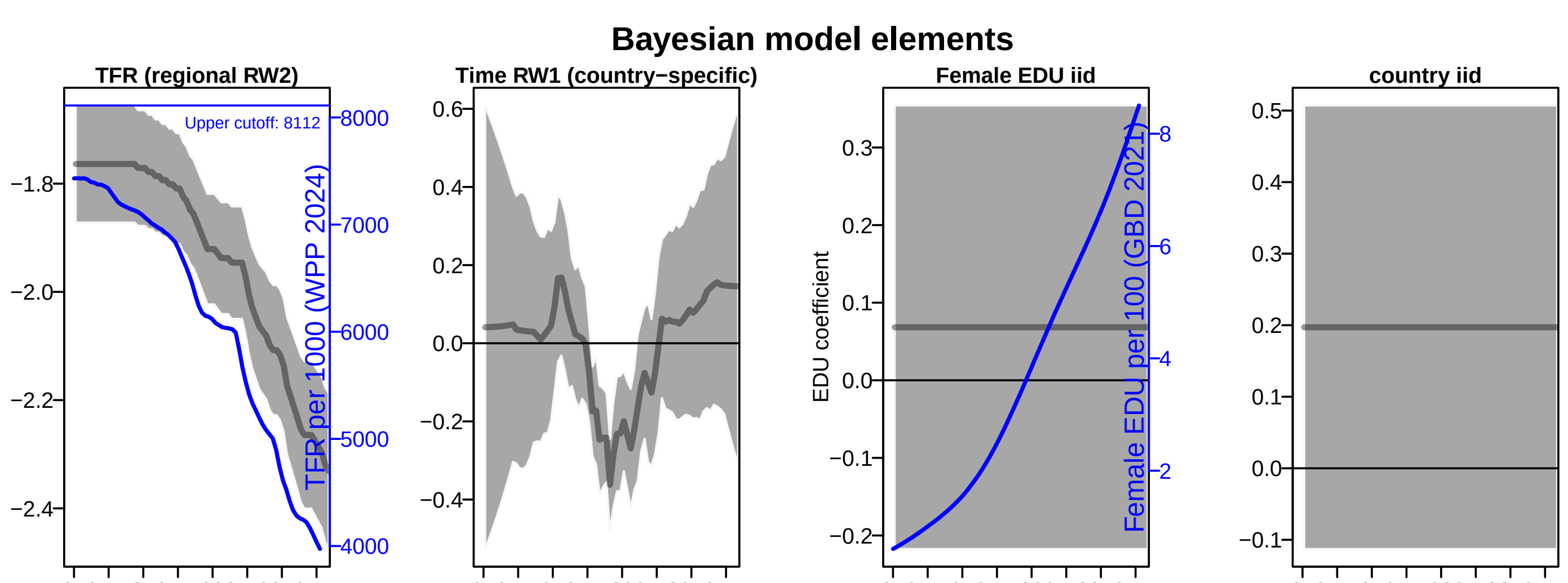
Bayesian model elements
TFR (regional RW2)
Upper cutoff: 8112
TFR per 1000 (WPP 2024)
Time RW1 (country−specific)
Female EDU iid
EDU coefficient
Female EDU per 100 (GBD 2021)
country iid

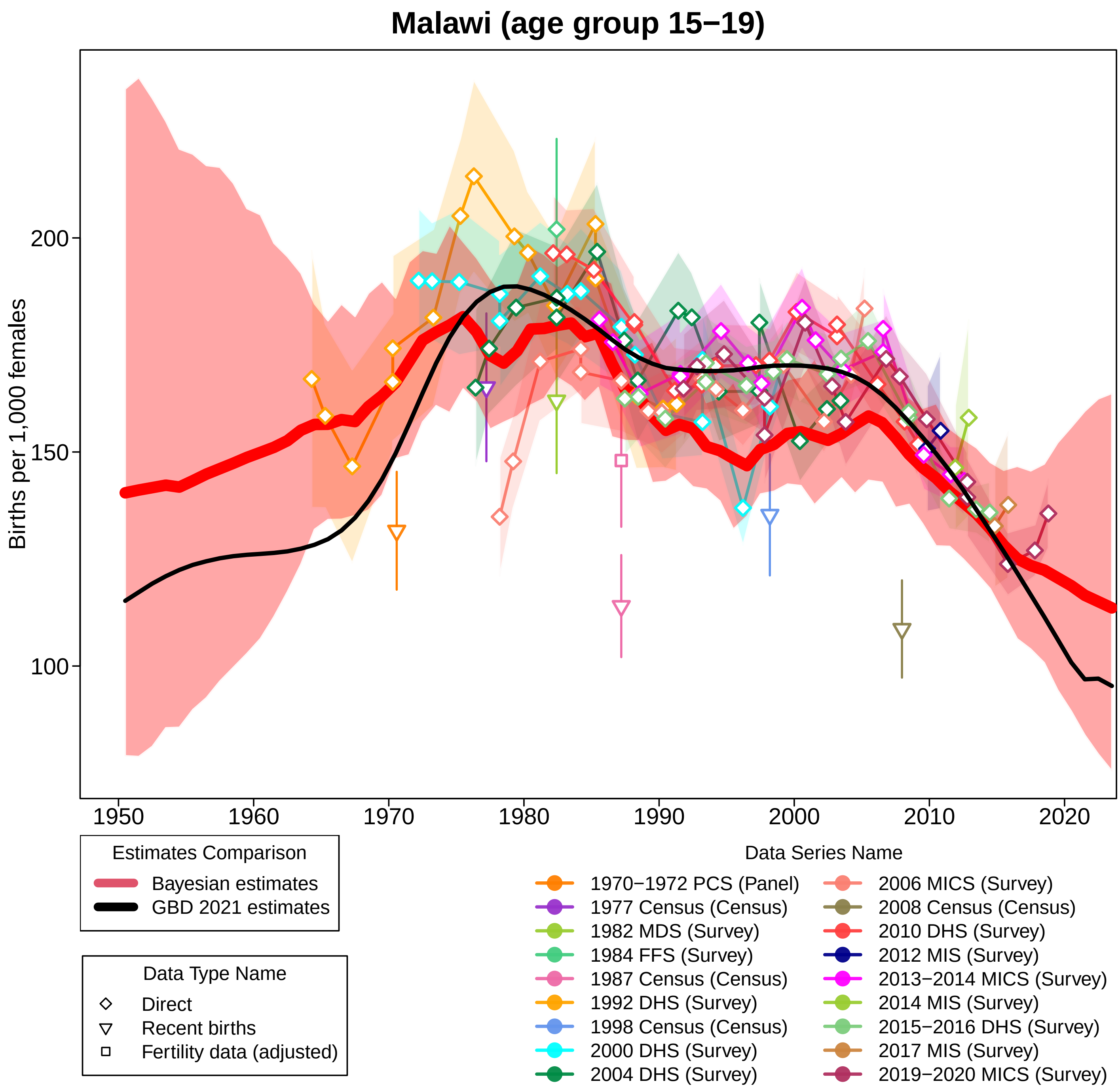
Malawi (age group 15−19)
Births per 1,000 females
200
150
100
1950
1960
1970
1980
1990
2000
2010
2020
Estimates Comparison
Bayesian estimates
GBD 2021 estimates
Data Type Name
Direct
Recent births
Fertility data (adjusted)
Data Series Name
1970−1972 PCS (Panel)
1977 Census (Census)
1982 MDS (Survey)
1984 FFS (Survey)
1987 Census (Census)
1992 DHS (Survey)
1998 Census (Census)
2000 DHS (Survey)
2004 DHS (Survey)
2006 MICS (Survey)
2008 Census (Census)
2010 DHS (Survey)
2012 MIS (Survey)
2013−2014 MICS (Survey)
2014 MIS (Survey)
2015−2016 DHS (Survey)
2017 MIS (Survey)
2019−2020 MICS (Survey)

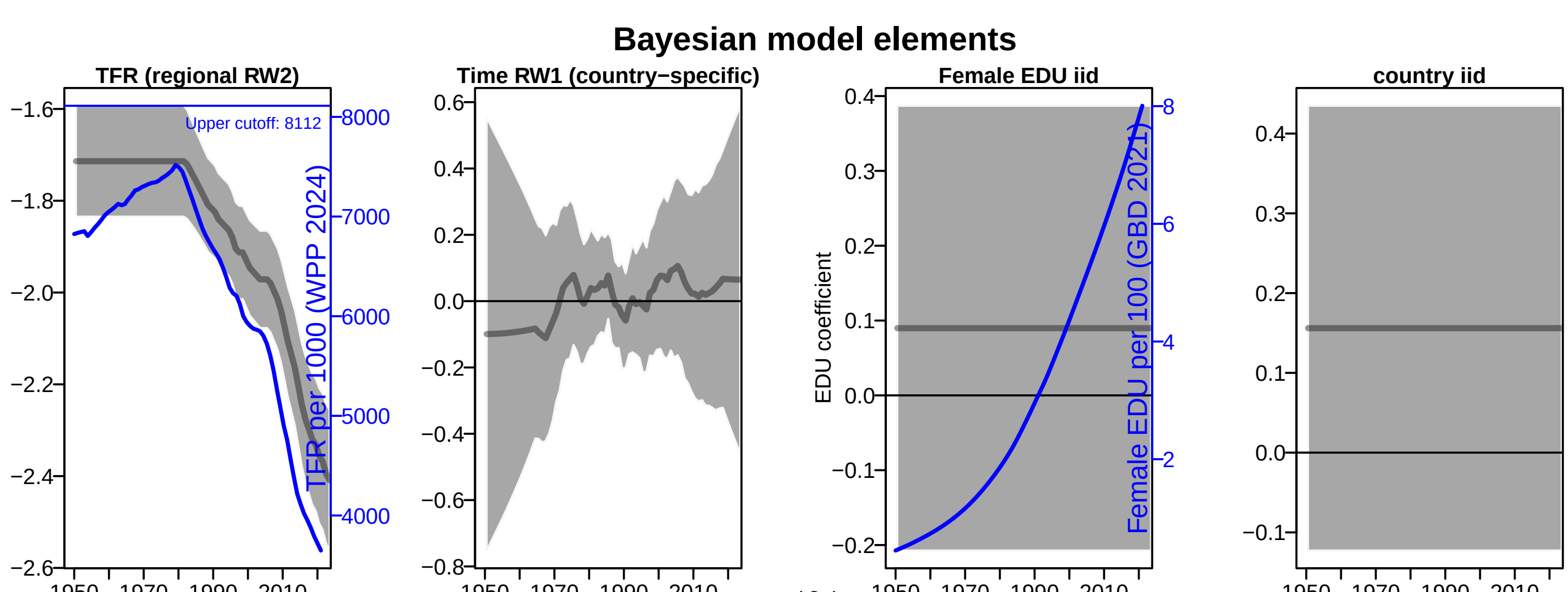
Bayesian model elements
TFR (regional RW2)
Upper cutoff: 8112
TFR per 1000 (WPP 2024)
Time RW1 (country−specific)
Female EDU iid
EDU coefficient
Female EDU per 100 (GBD 2021)
country iid

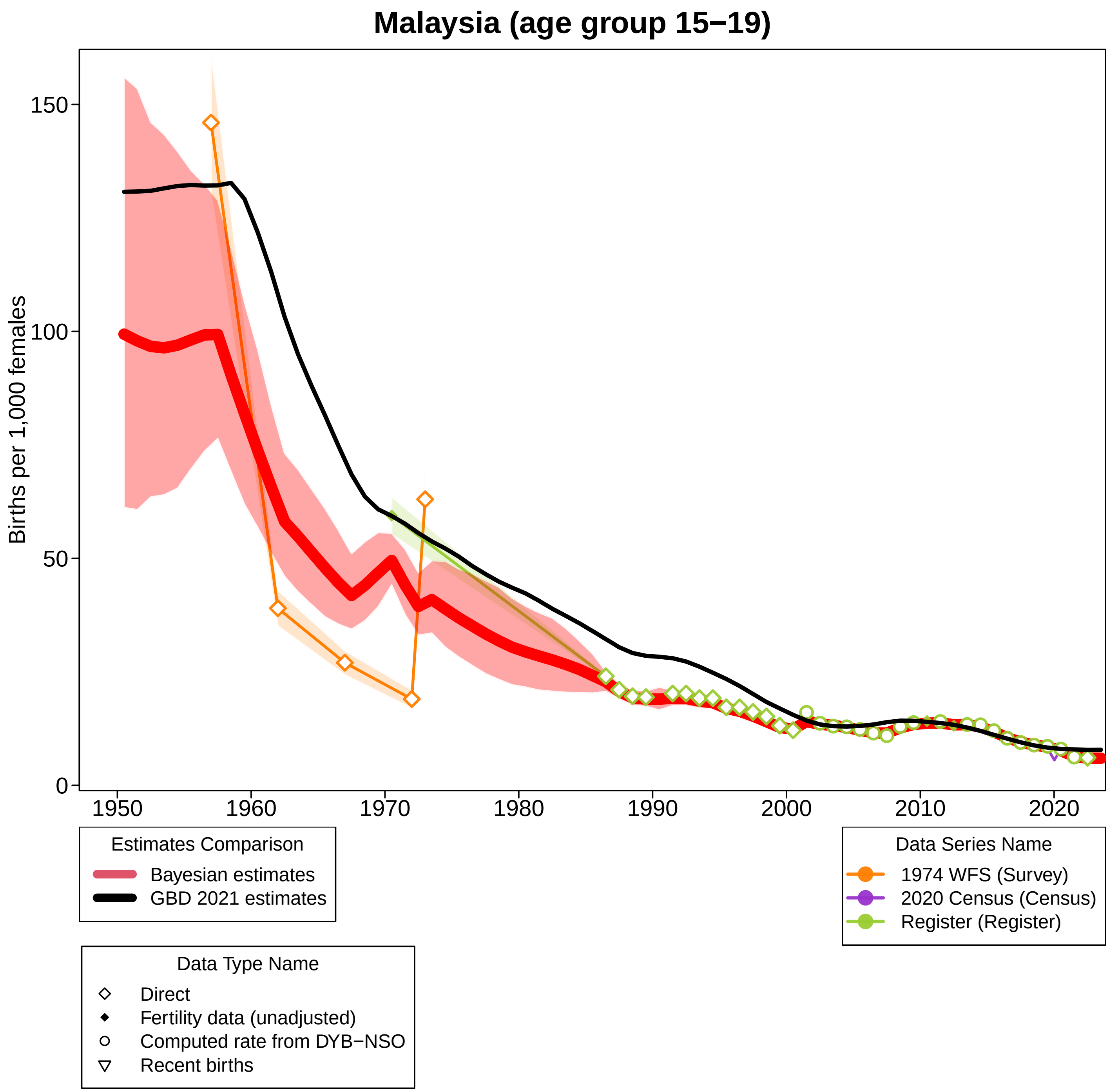
Malaysia (age group 15−19)
Births per 1,000 females
150
100
50
0
1950
1960
1970
1980
1990
2000
2010
2020
Estimates Comparison
Bayesian estimates
GBD 2021 estimates
Data Series Name
1974 WFS (Survey)
2020 Census (Census)
Register (Register)
Data Type Name
Direct
Fertility data (unadjusted)
Computed rate from DYB−NSO
Recent births

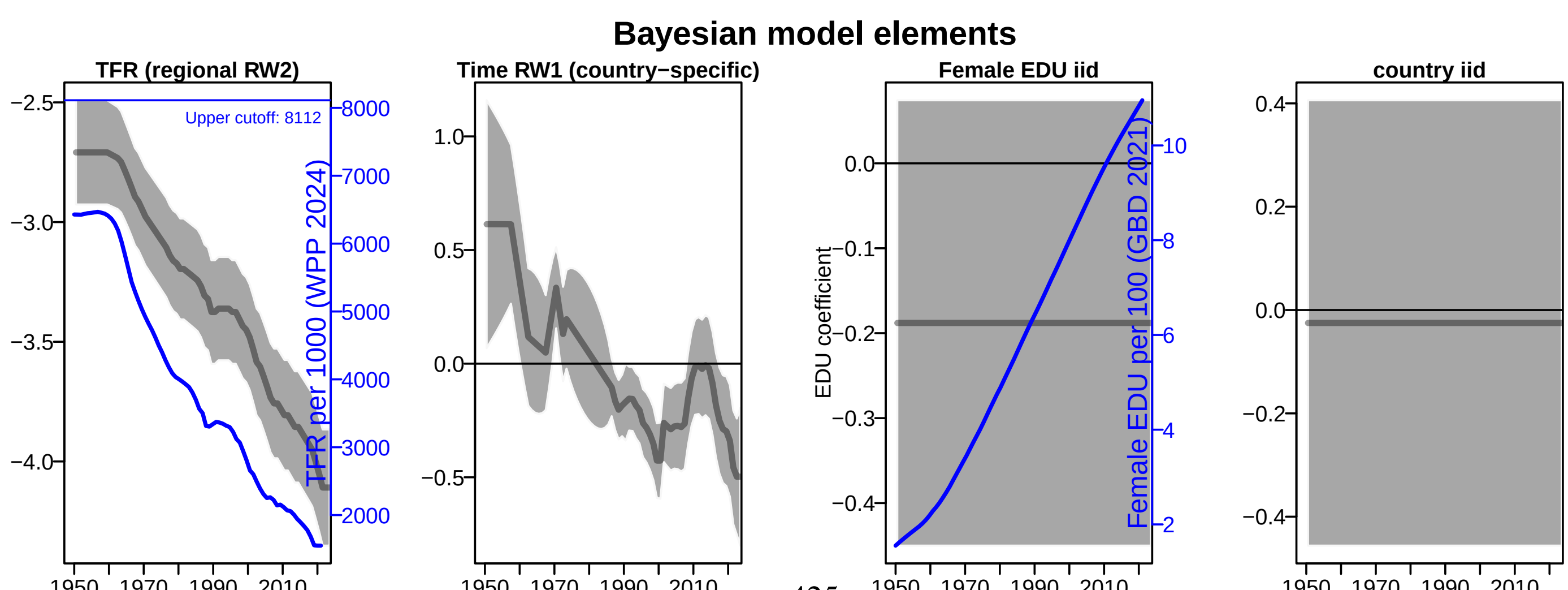
Bayesian model elements
TFR (regional RW2)
Upper cutoff: 8112
TFR per 1000 (WPP 2024)
Time RW1 (country−specific)
Female EDU iid
EDU coefficient
Female EDU per 100 (GBD 2021)
country iid

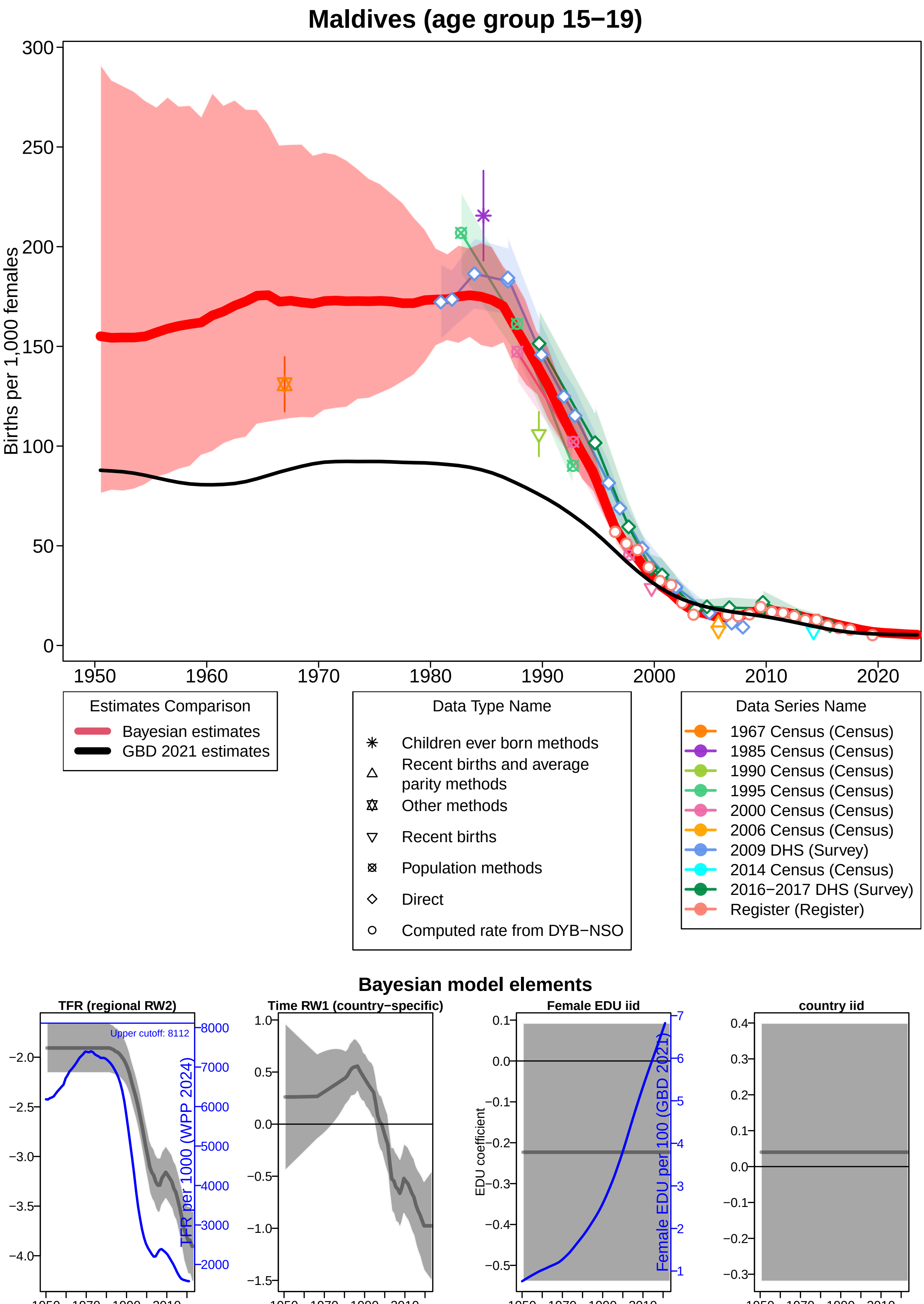
Maldives (age group 15−19)
Births per 1,000 females
Estimates Comparison
Bayesian estimates
GBD 2021 estimates
Data Type Name
Children ever born methods
Recent births and average parity methods
Other methods
Recent births
Population methods
Direct
Computed rate from DYB−NSO
Data Series Name
1967 Census (Census)
1985 Census (Census)
1990 Census (Census)
1995 Census (Census)
2000 Census (Census)
2006 Census (Census)
2009 DHS (Survey)
2014 Census (Census)
2016−2017 DHS (Survey)
Register (Register)
Bayesian model elements
TFR (regional RW2)
Upper cutoff: 8112
TFR per 1000 (WPP 2024)
Time RW1 (country−specific)
Female EDU iid
EDU coefficient
Female EDU per 100 (GBD 2021)
country iid

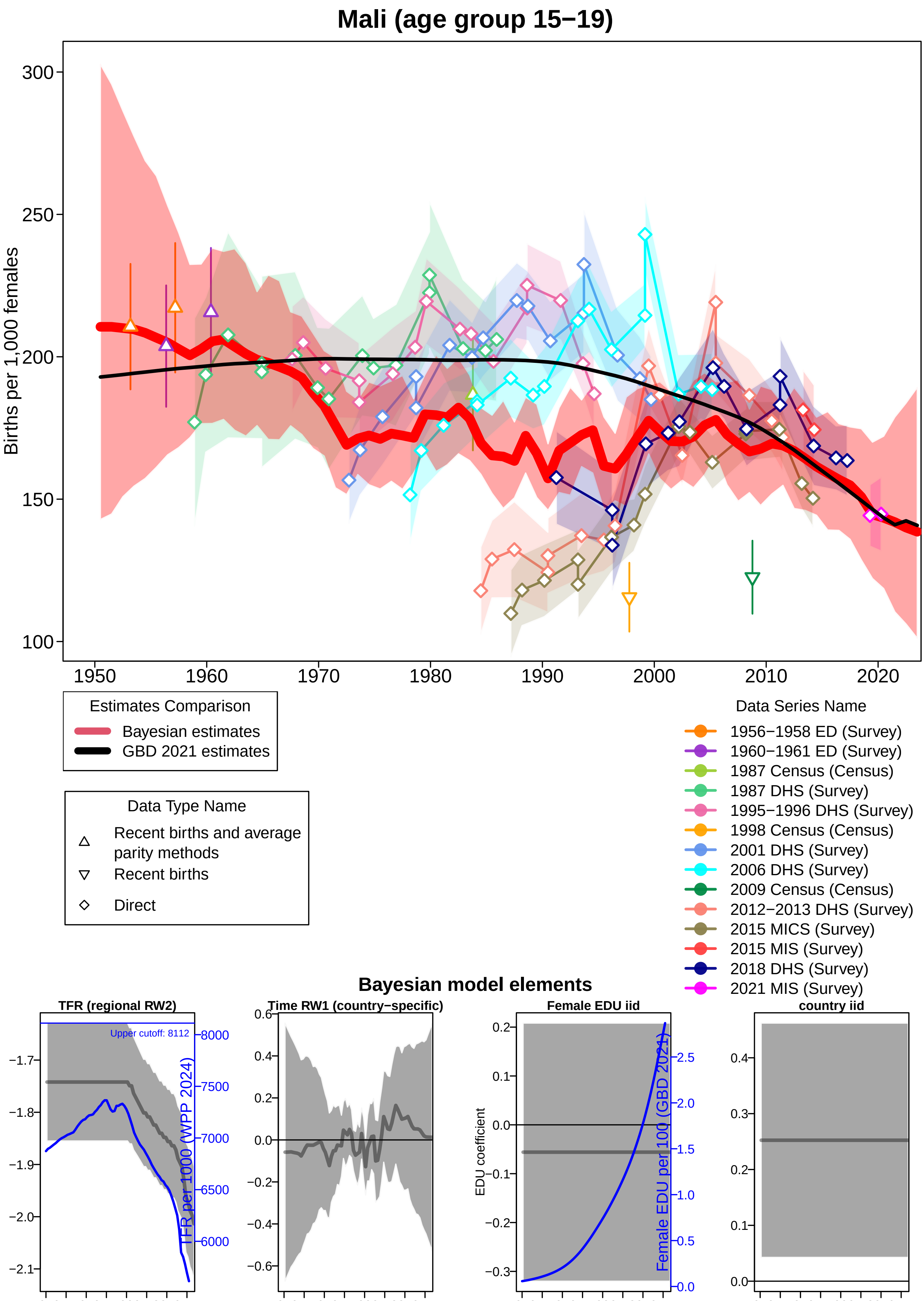
Mali (age group 15−19)
Births per 1,000 females
300
250
200
150
100
1950
1960
1970
1980
1990
2000
2010
2020
Estimates Comparison
Bayesian estimates
GBD 2021 estimates
Data Type Name
Recent births and average parity methods
Recent births
Direct
Data Series Name
1956−1958 ED (Survey)
1960−1961 ED (Survey)
1987 Census (Census)
1987 DHS (Survey)
1995−1996 DHS (Survey)
1998 Census (Census)
2001 DHS (Survey)
2006 DHS (Survey)
2009 Census (Census)
2012−2013 DHS (Survey)
2015 MICS (Survey)
2015 MIS (Survey)
2018 DHS (Survey)
2021 MIS (Survey)
Bayesian model elements
TFR (regional RW2)
Upper cutoff: 8112
TFR per 1000 (WPP 2024)
Time RW1 (country−specific)
Female EDU iid
EDU coefficient
Female EDU per 100 (GBD 2021)
country iid

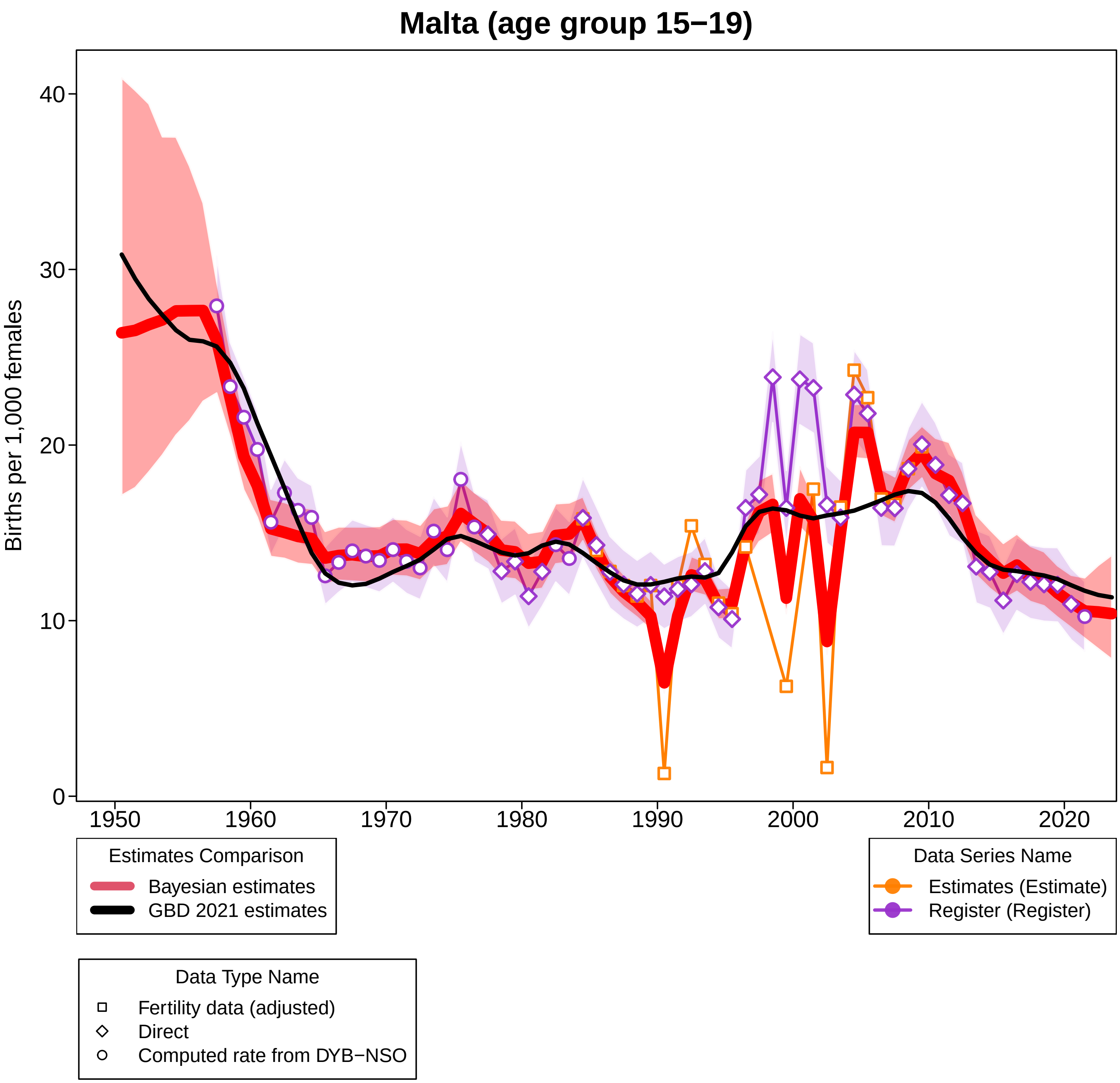
Malta (age group 15−19)
Births per 1,000 females
0
10
20
30
40
1950
1960
1970
1980
1990
2000
2010
2020
Estimates Comparison
Bayesian estimates
GBD 2021 estimates
Data Series Name
Estimates (Estimate)
Register (Register)
Data Type Name
Fertility data (adjusted)
Direct
Computed rate from DYB−NSO

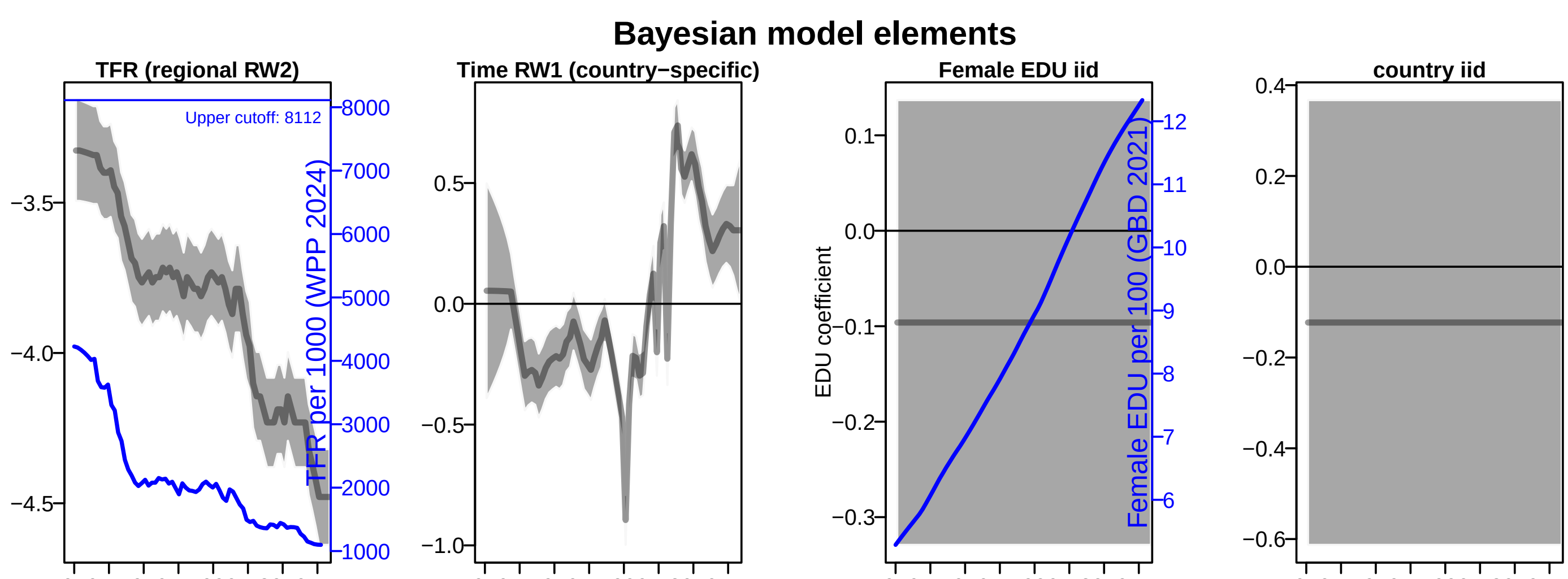
Bayesian model elements
TFR (regional RW2)
Upper cutoff: 8112
TFR per 1000 (WPP 2024)
Time RW1 (country−specific)
Female EDU iid
EDU coefficient
Female EDU per 100 (GBD 2021)
country iid
1950
1970
1990
2010

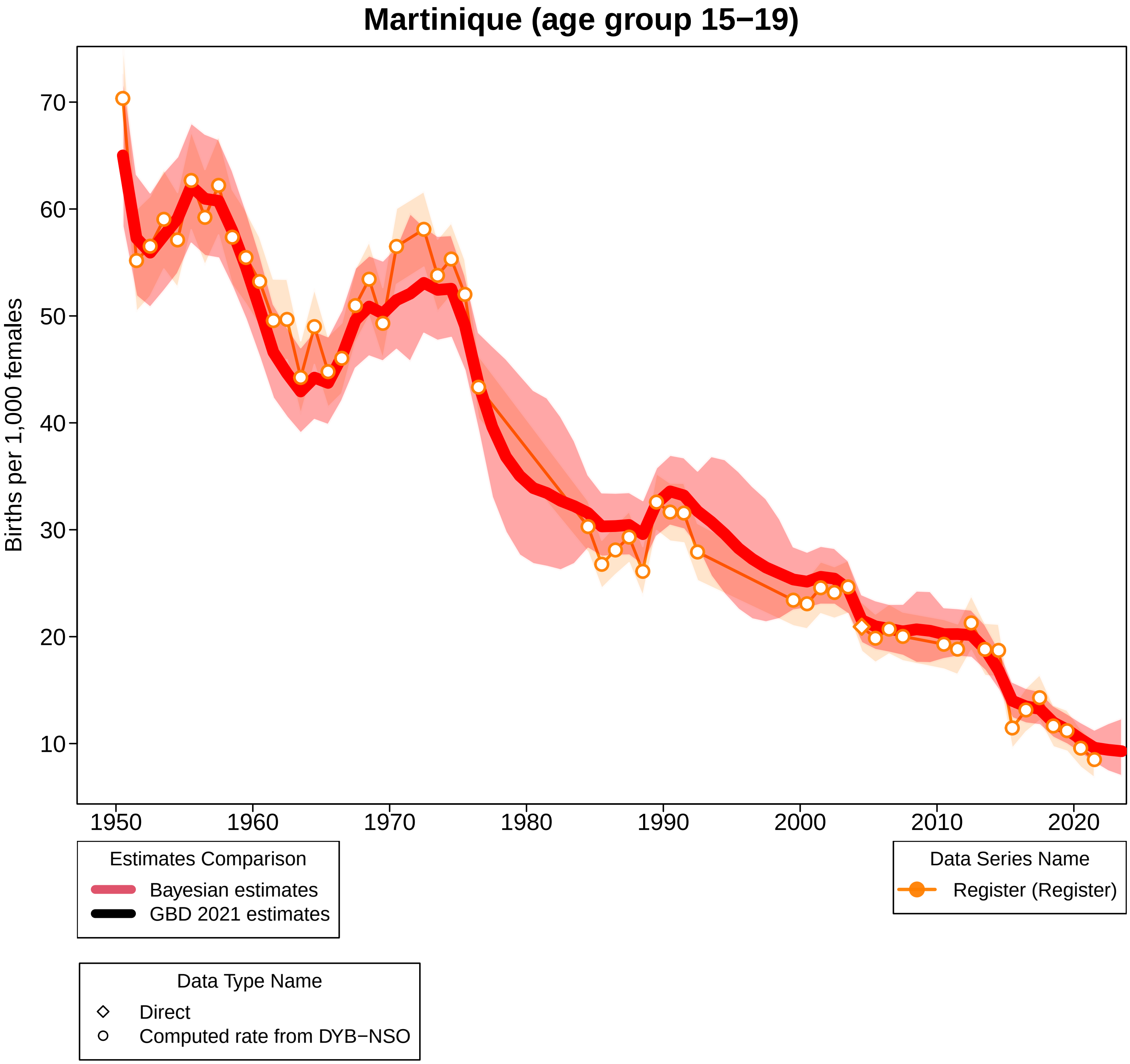
Martinique (age group 15−19)
Births per 1,000 females
70
60
50
40
30
20
10
1950
1960
1970
1980
1990
2000
2010
2020
Estimates Comparison
Bayesian estimates
GBD 2021 estimates
Data Series Name
Register (Register)
Data Type Name
Direct
Computed rate from DYB−NSO

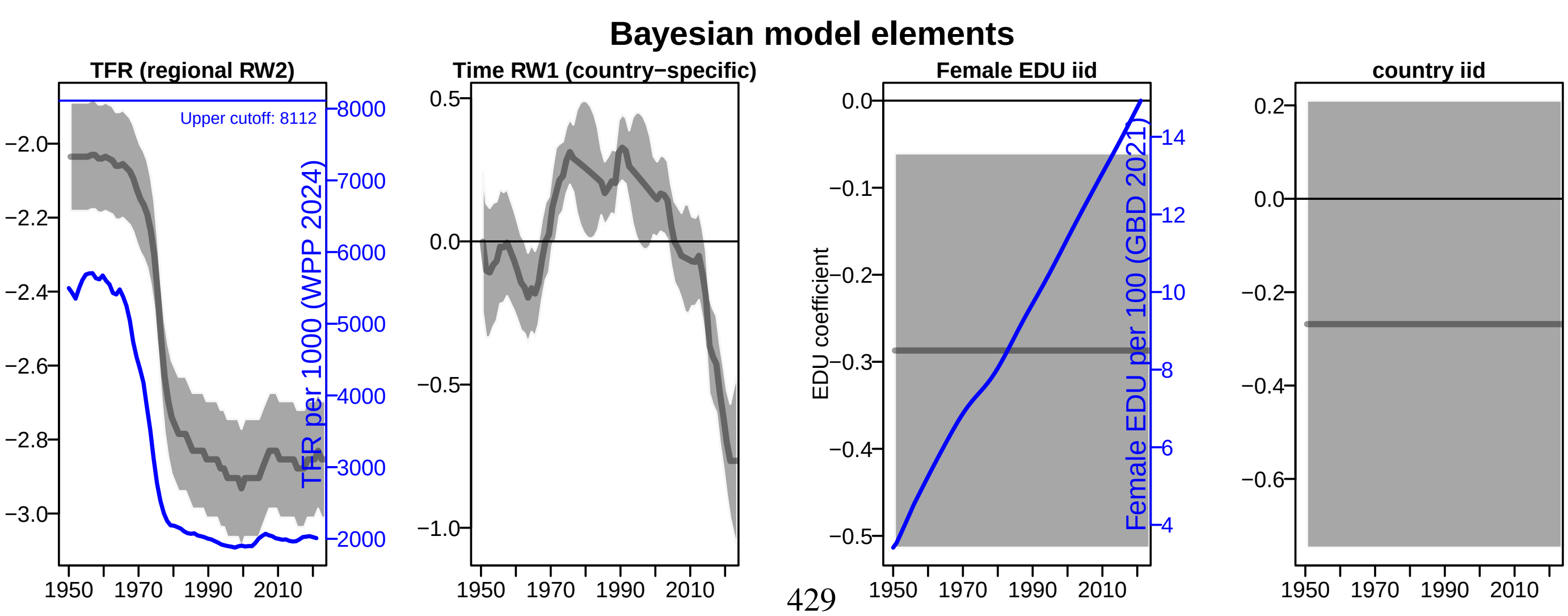
Bayesian model elements
TFR (regional RW2)
Upper cutoff: 8112
TFR per 1000 (WPP 2024)
Time RW1 (country−specific)
Female EDU iid
EDU coefficient
Female EDU per 100 (GBD 2021)
country iid

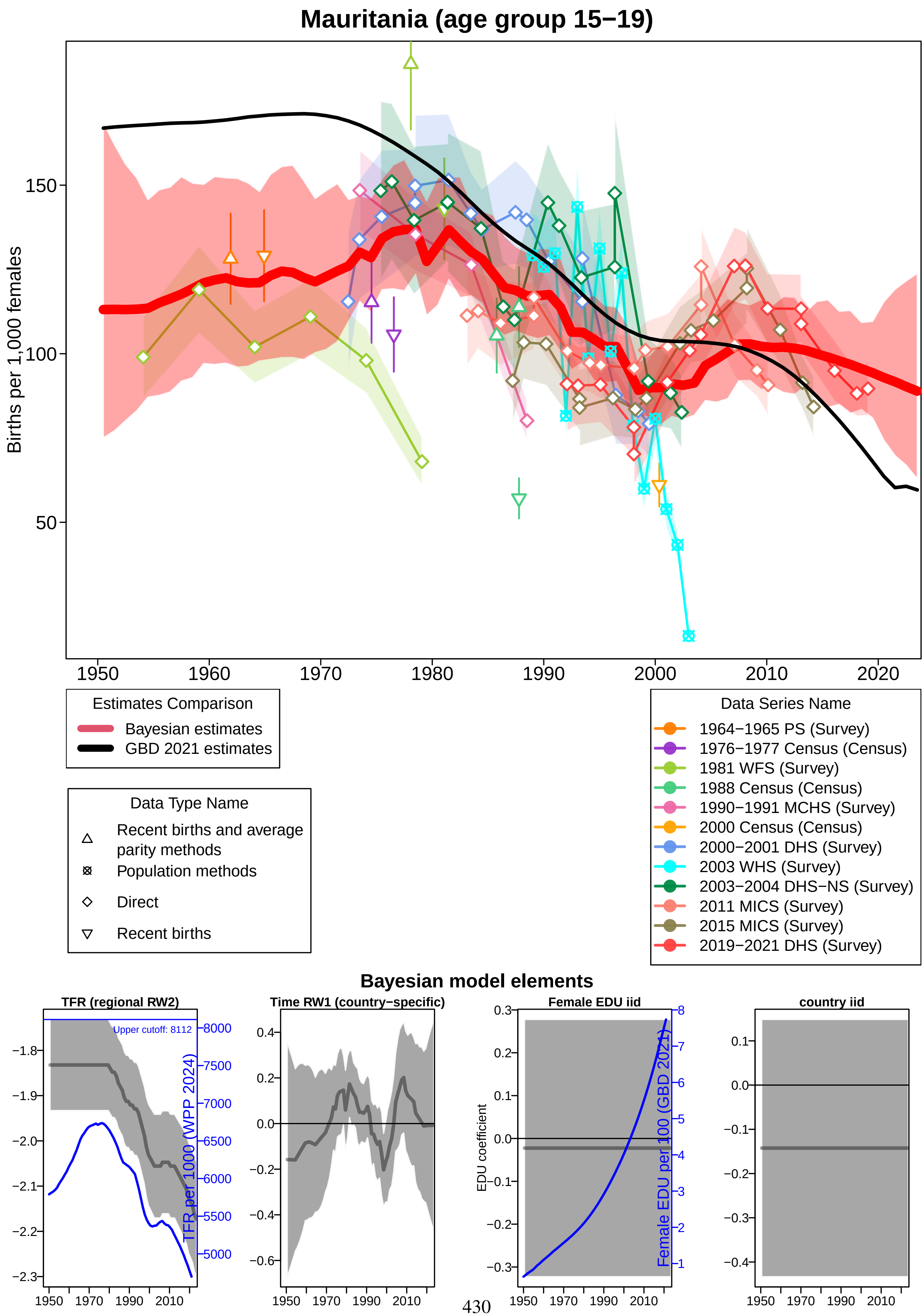

Mauritania (age group 15−19)
Births per 1,000 females
150
100
50
1950
1960
1970
1980
1990
2000
2010
2020
Estimates Comparison
Bayesian estimates
GBD 2021 estimates
Data Type Name
Recent births and average parity methods
Population methods
Direct
Recent births
Data Series Name
1964−1965 PS (Survey)
1976−1977 Census (Census)
1981 WFS (Survey)
1988 Census (Census)
1990−1991 MCHS (Survey)
2000 Census (Census)
2000−2001 DHS (Survey)
2003 WHS (Survey)
2003−2004 DHS−NS (Survey)
2011 MICS (Survey)
2015 MICS (Survey)
2019−2021 DHS (Survey)
Bayesian model elements
TFR (regional RW2)
Upper cutoff: 8112
TFR per 1000 (WPP 2024)
Time RW1 (country−specific)
Female EDU iid
EDU coefficient
Female EDU per 100 (GBD 2021)
country iid

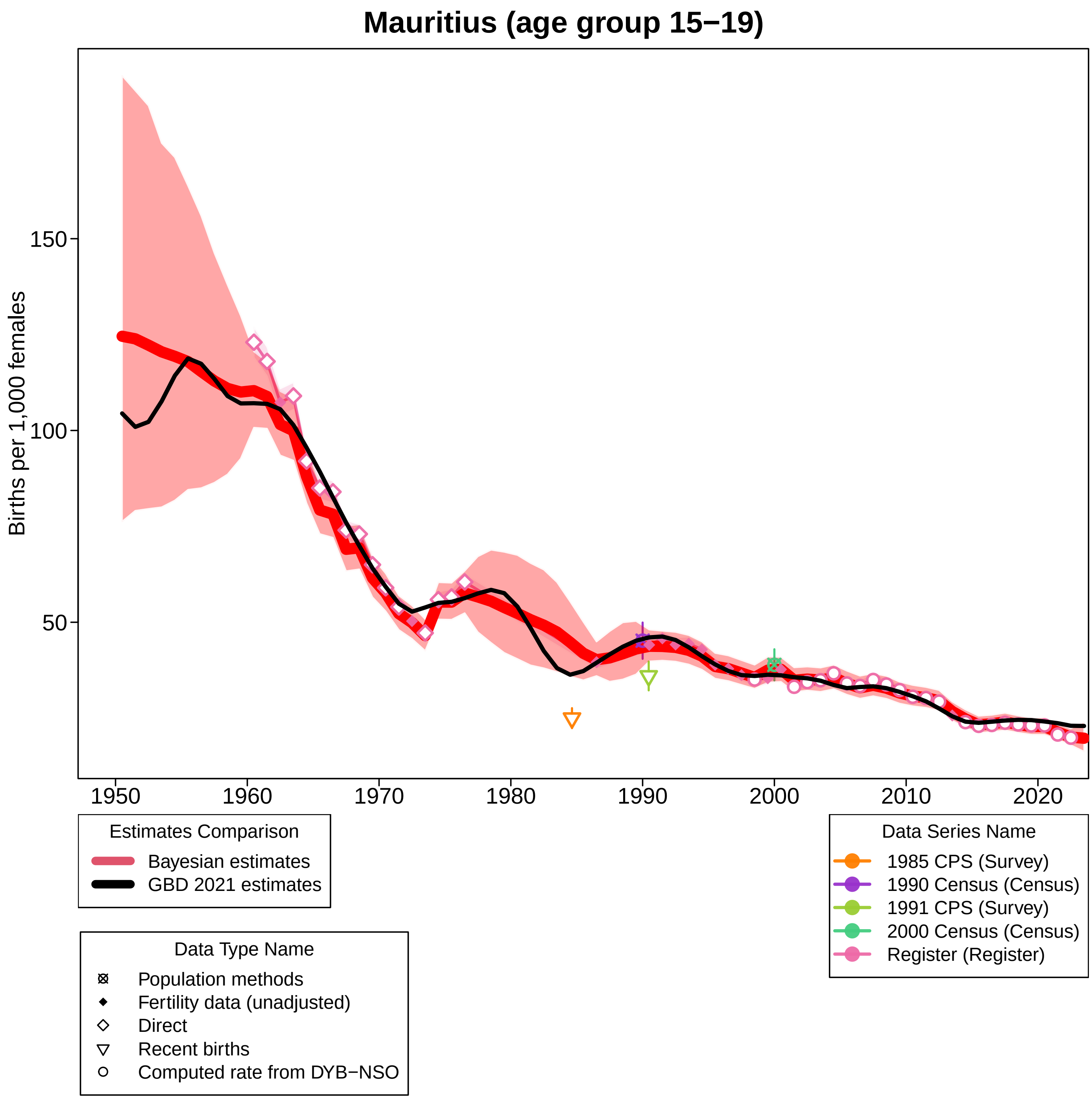
Mauritius (age group 15−19)
Births per 1,000 females
150
100
50
1950
1960
1970
1980
1990
2000
2010
2020
Estimates Comparison
Bayesian estimates
GBD 2021 estimates
Data Series Name
1985 CPS (Survey)
1990 Census (Census)
1991 CPS (Survey)
2000 Census (Census)
Register (Register)
Data Type Name
Population methods
Fertility data (unadjusted)
Direct
Recent births
Computed rate from DYB−NSO

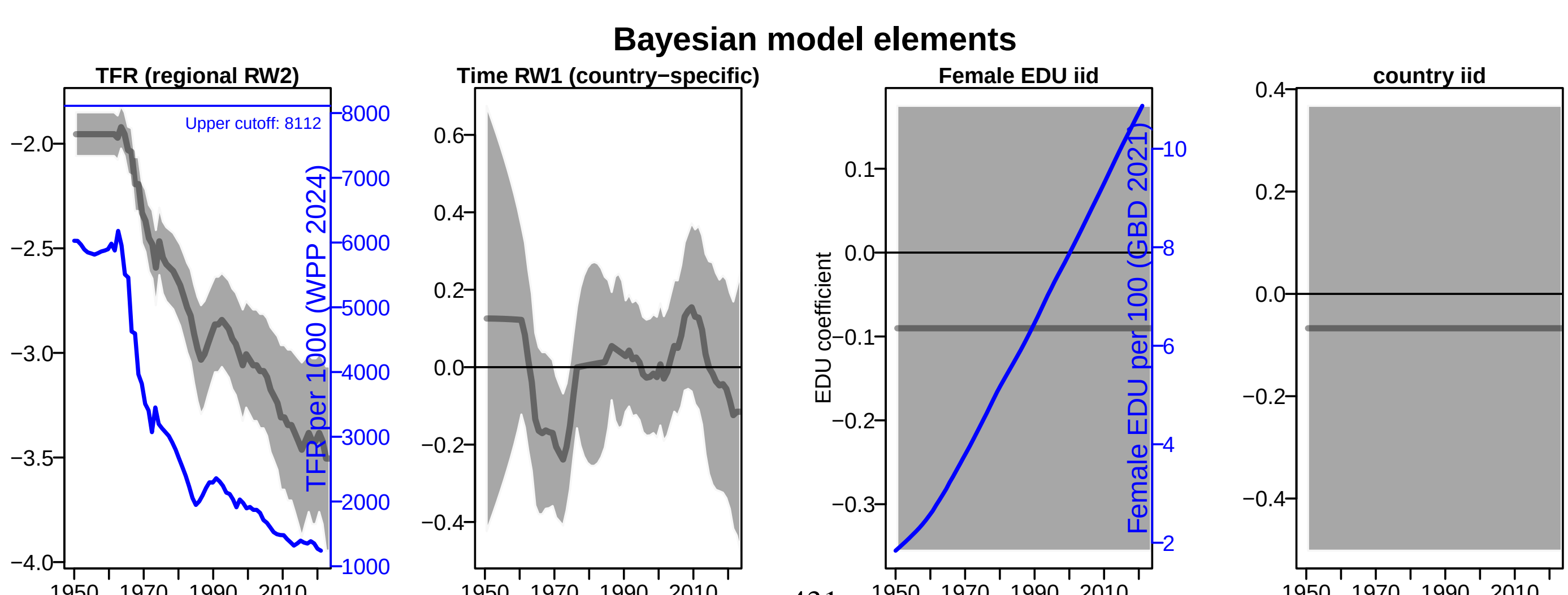
Bayesian model elements
TFR (regional RW2)
Upper cutoff: 8112
TFR per 1000 (WPP 2024)
Time RW1 (country−specific)
Female EDU iid
EDU coefficient
Female EDU per 100 (GBD 2021)
country iid

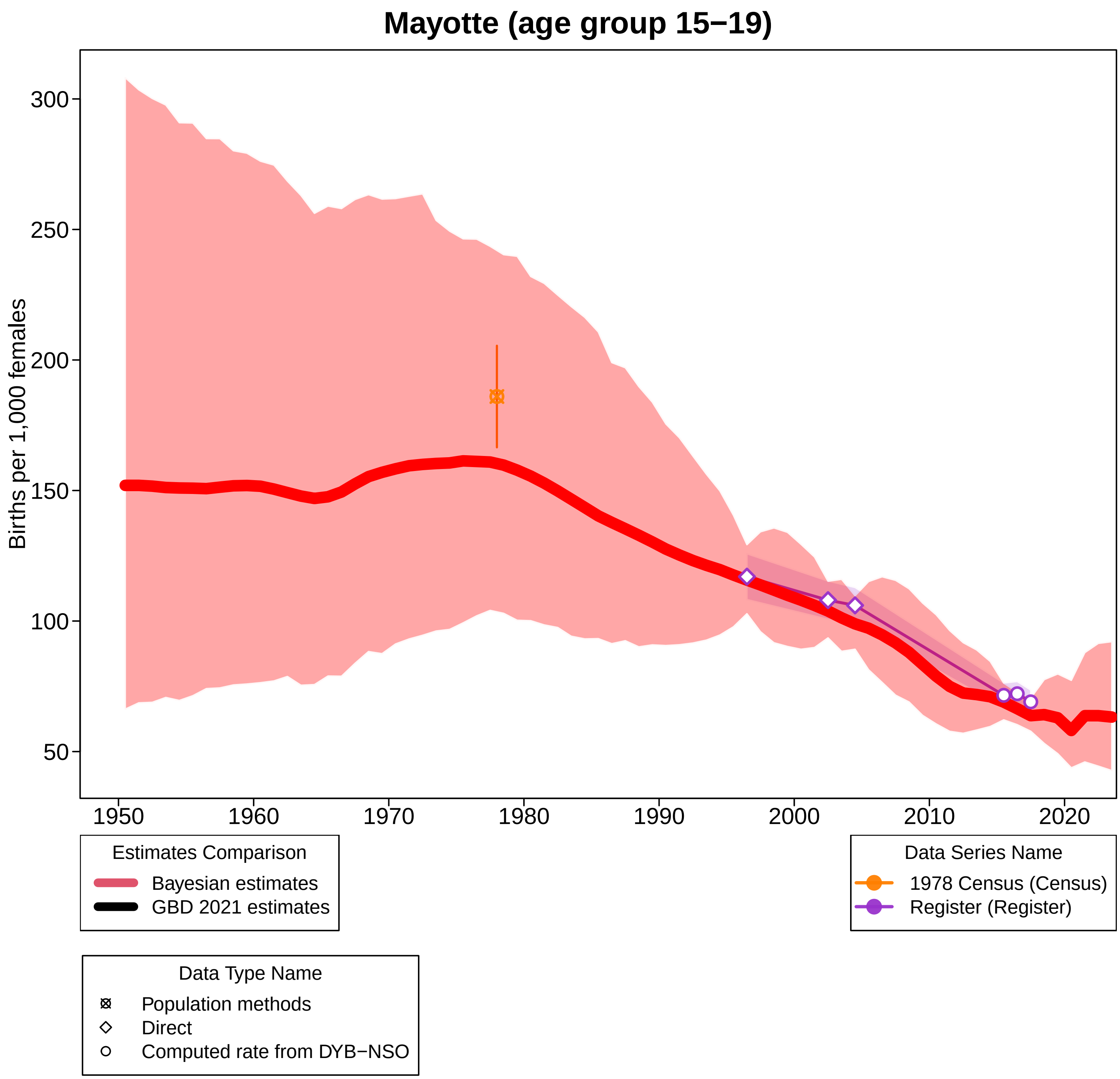
Mayotte (age group 15−19)
Births per 1,000 females
300
250
200
150
100
50
1950
1960
1970
1980
1990
2000
2010
2020
Estimates Comparison
Bayesian estimates
GBD 2021 estimates
Data Series Name
1978 Census (Census)
Register (Register)
Data Type Name
Population methods
Direct
Computed rate from DYB−NSO

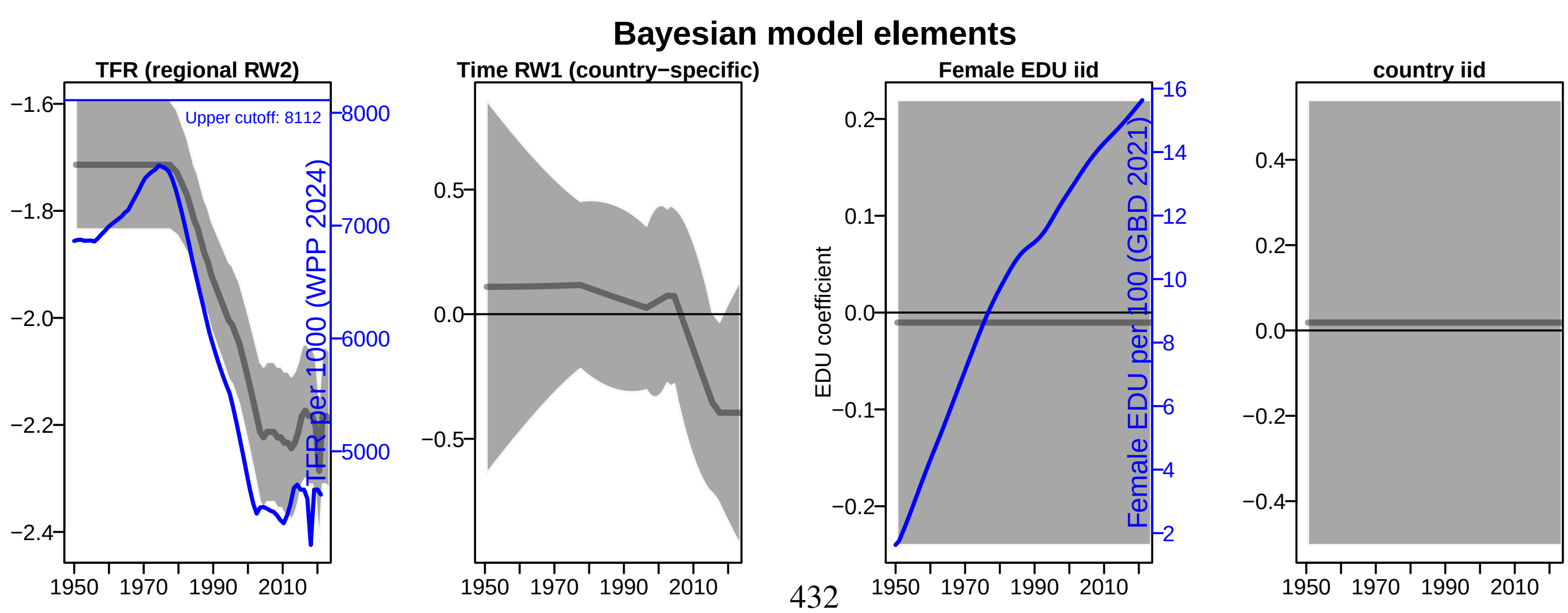
Bayesian model elements
TFR (regional RW2)
Upper cutoff: 8112
TFR per 1000 (WPP 2024)
Time RW1 (country−specific)
Female EDU iid
EDU coefficient
Female EDU per 100 (GBD 2021)
country iid

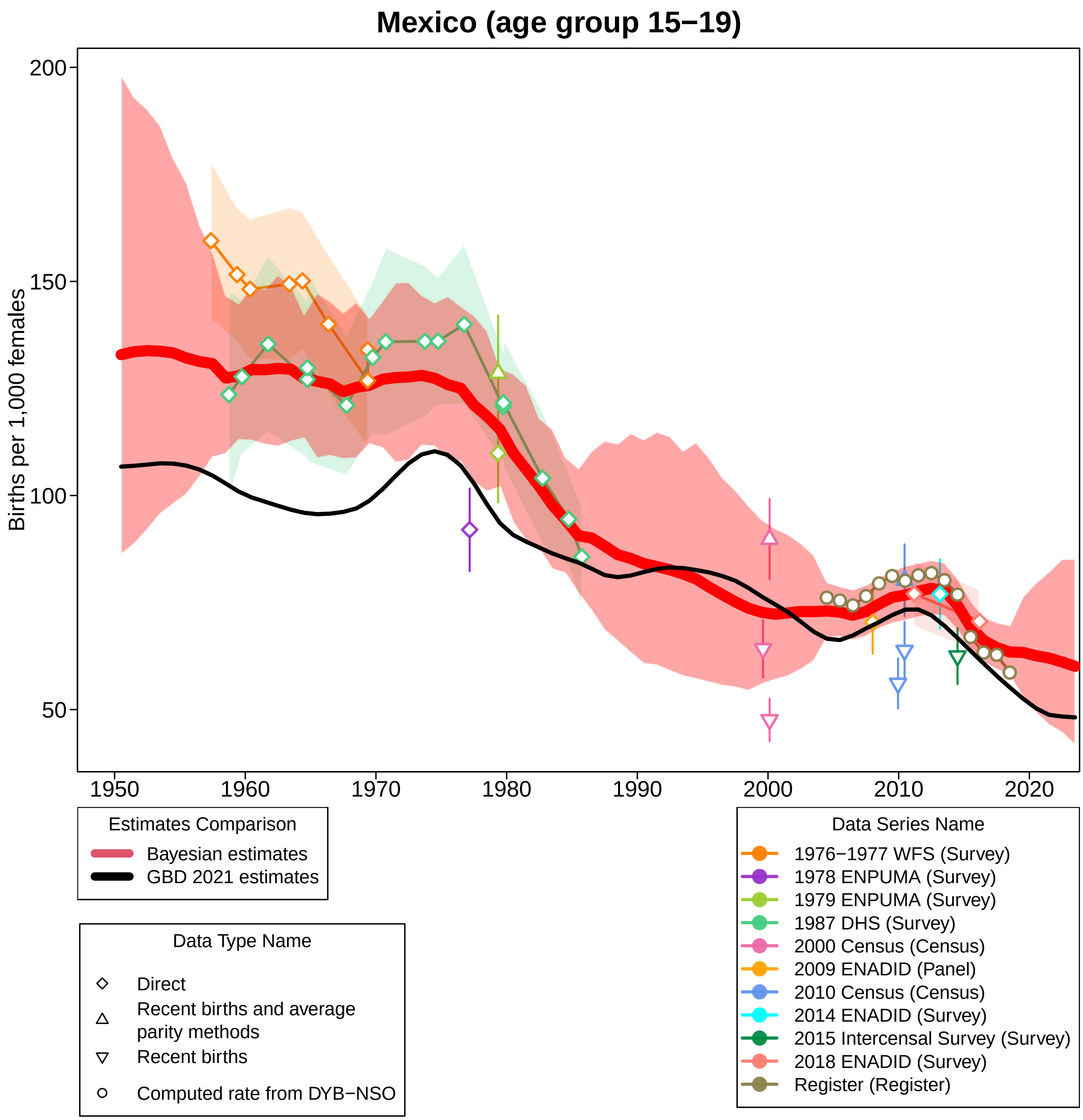
Mexico (age group 15−19)
Births per 1,000 females
200
150
100
50
1950
1960
1970
1980
1990
2000
2010
2020
Estimates Comparison
Bayesian estimates
GBD 2021 estimates
Data Type Name
Direct
Recent births and average parity methods
Recent births
Computed rate from DYB−NSO
Data Series Name
1976−1977 WFS (Survey)
1978 ENPUMA (Survey)
1979 ENPUMA (Survey)
1987 DHS (Survey)
2000 Census (Census)
2009 ENADID (Panel)
2010 Census (Census)
2014 ENADID (Survey)
2015 Intercensal Survey (Survey)
2018 ENADID (Survey)
Register (Register)

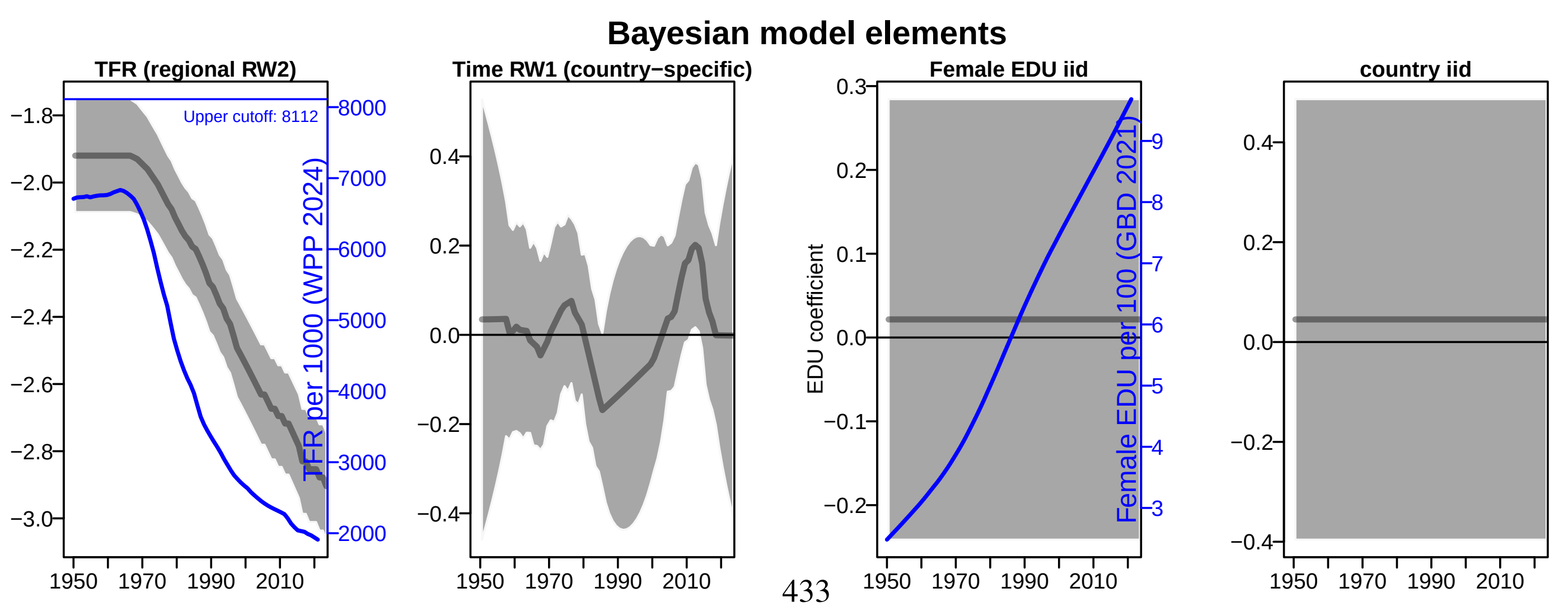
Bayesian model elements
TFR (regional RW2)
Upper cutoff: 8112
TFR per 1000 (WPP 2024)
Time RW1 (country−specific)
Female EDU iid
EDU coefficient
Female EDU per 100 (GBD 2021)
country iid
1950
1970
1990
2010

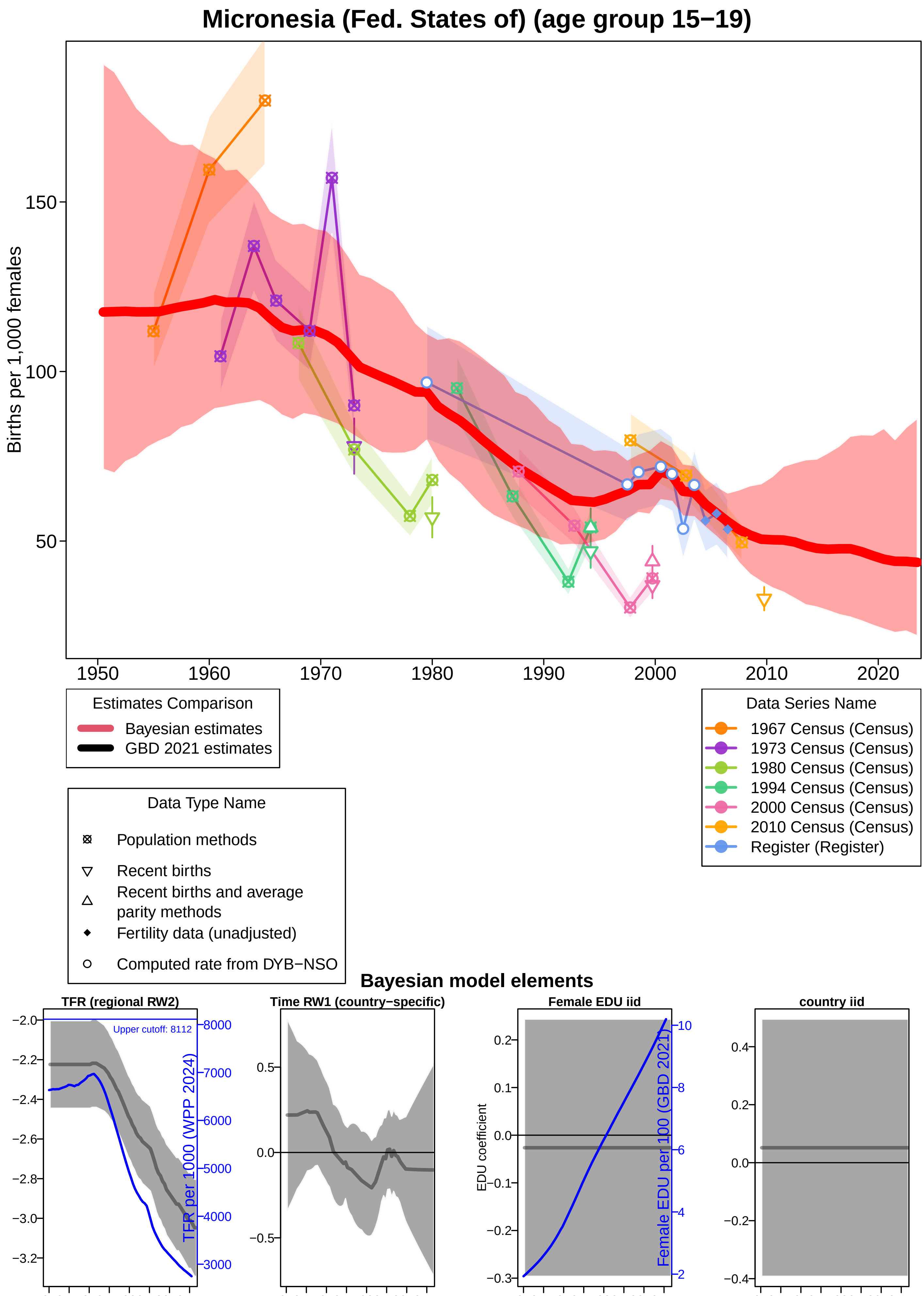

Micronesia (Fed. States of) (age group 15−19)
Births per 1,000 females
150
100
50
1950
1960
1970
1980
1990
2000
2010
2020
Estimates Comparison
Bayesian estimates
GBD 2021 estimates
Data Series Name
1967 Census (Census)
1973 Census (Census)
1980 Census (Census)
1994 Census (Census)
2000 Census (Census)
2010 Census (Census)
Register (Register)
Data Type Name
Population methods
Recent births
Recent births and average parity methods
Fertility data (unadjusted)
Computed rate from DYB−NSO
Bayesian model elements
TFR (regional RW2)
Upper cutoff: 8112
TFR per 1000 (WPP 2024)
Time RW1 (country−specific)
Female EDU iid
EDU coefficient
Female EDU per 100 (GBD 2021)
country iid

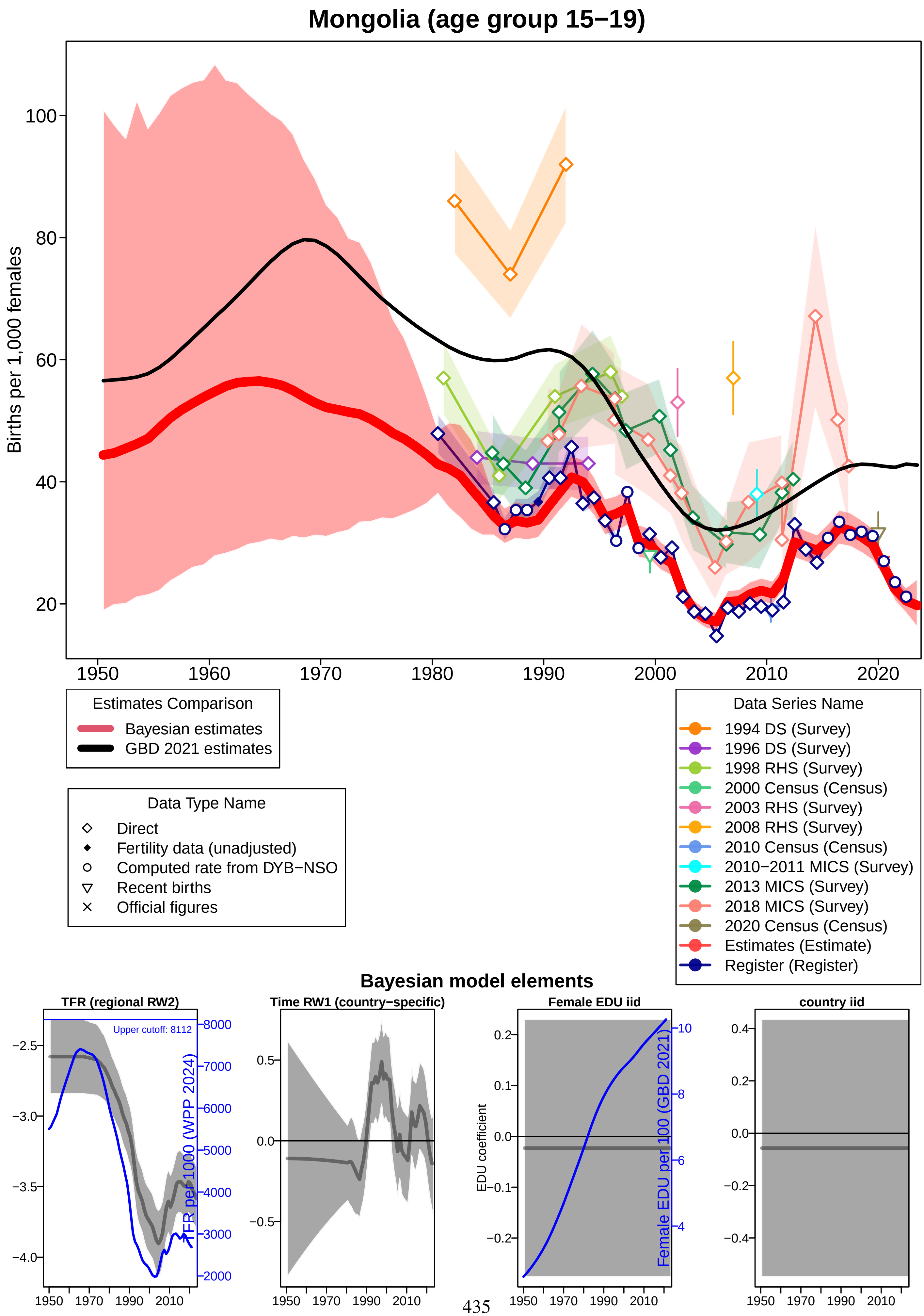
Mongolia (age group 15−19)
Births per 1,000 females
100
80
60
40
20
1950
1960
1970
1980
1990
2000
2010
2020
Estimates Comparison
Bayesian estimates
GBD 2021 estimates
Data Type Name
Direct
Fertility data (unadjusted)
Computed rate from DYB−NSO
Recent births
Official figures
Data Series Name
1994 DS (Survey)
1996 DS (Survey)
1998 RHS (Survey)
2000 Census (Census)
2003 RHS (Survey)
2008 RHS (Survey)
2010 Census (Census)
2010−2011 MICS (Survey)
2013 MICS (Survey)
2018 MICS (Survey)
2020 Census (Census)
Estimates (Estimate)
Register (Register)
Bayesian model elements
TFR (regional RW2)
Upper cutoff: 8112
TFR per 1000 (WPP 2024)
Time RW1 (country−specific)
Female EDU iid
EDU coefficient
Female EDU per 100 (GBD 2021)
country iid

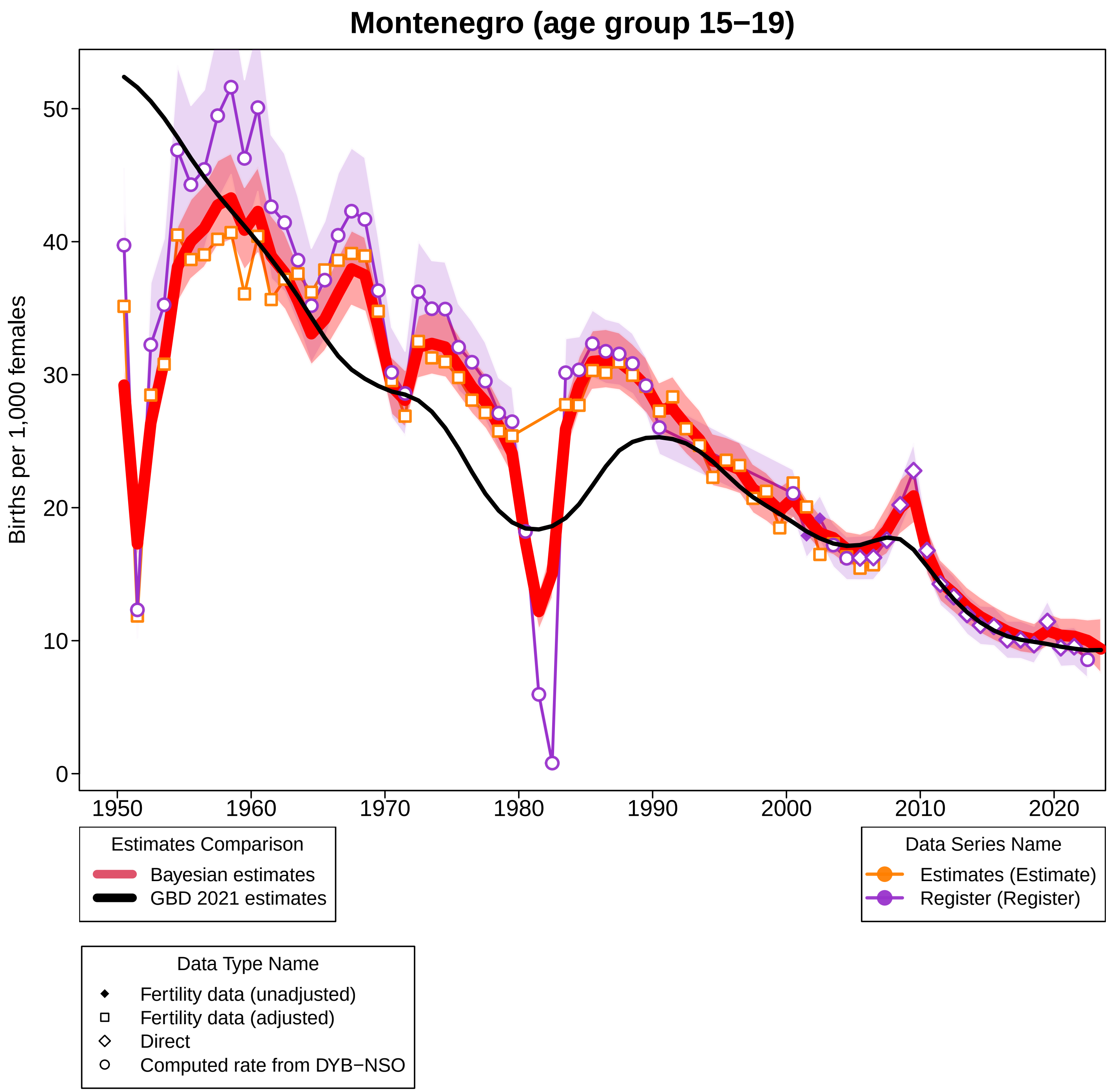
Montenegro (age group 15−19)
Births per 1,000 females
0
10
20
30
40
50
1950
1960
1970
1980
1990
2000
2010
2020
Estimates Comparison
Bayesian estimates
GBD 2021 estimates
Data Series Name
Estimates (Estimate)
Register (Register)
Data Type Name
Fertility data (unadjusted)
Fertility data (adjusted)
Direct
Computed rate from DYB−NSO

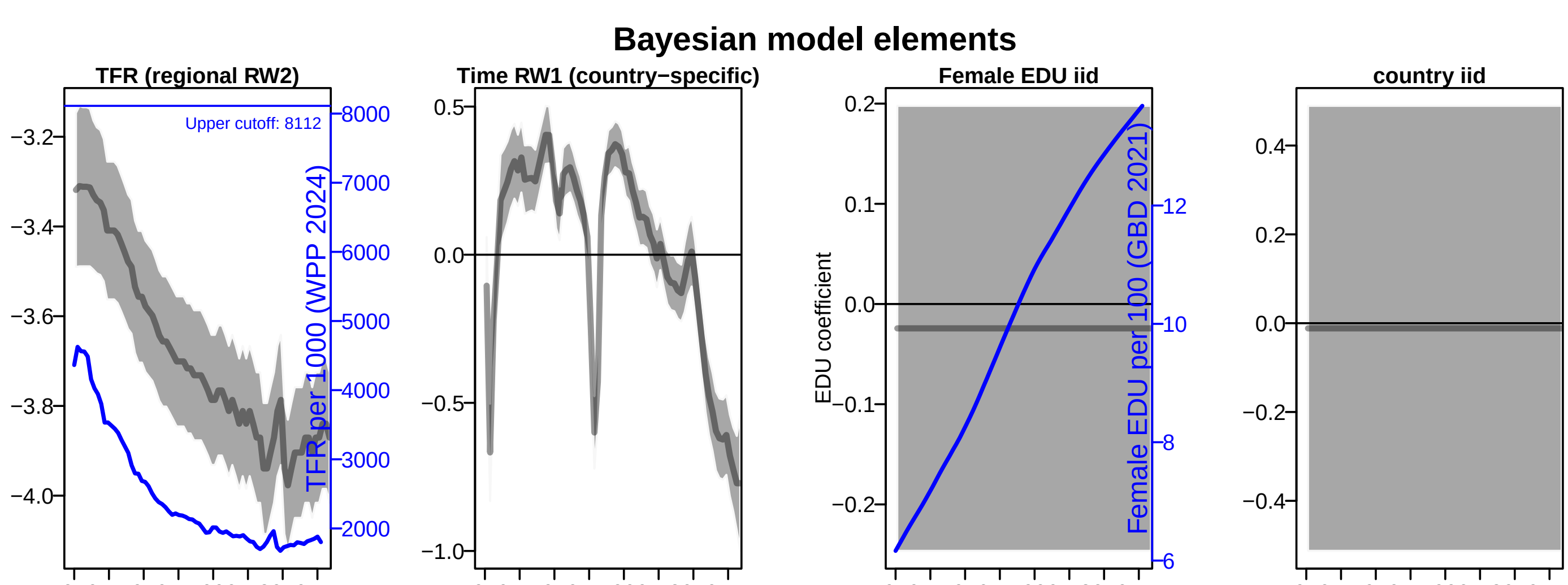
Bayesian model elements
TFR (regional RW2)
Upper cutoff: 8112
TFR per 1000 (WPP 2024)
Time RW1 (country−specific)
Female EDU iid
EDU coefficient
Female EDU per 100 (GBD 2021)
country iid
1950
1970
1990
2010

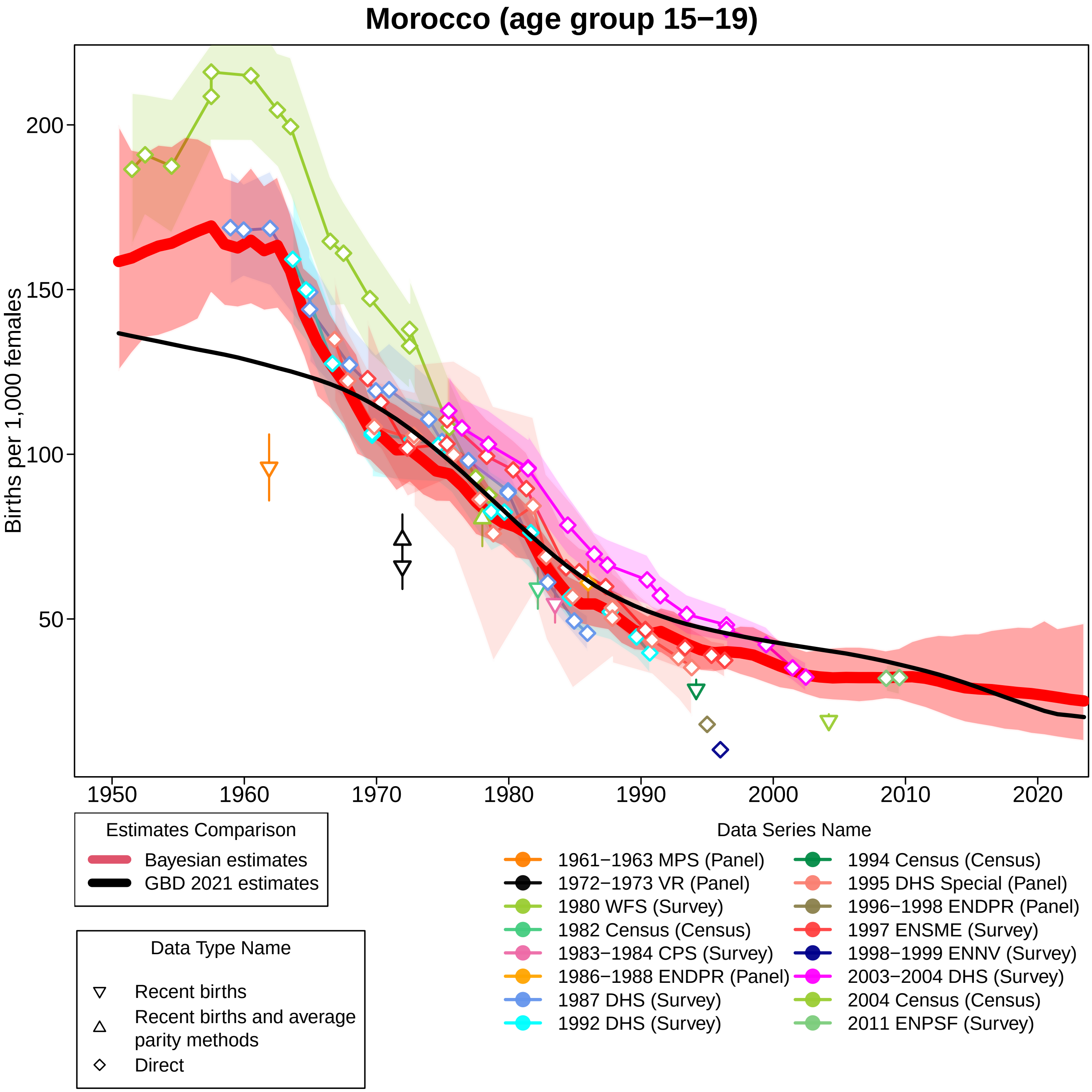
Morocco (age group 15−19)
Births per 1,000 females
200
150
100
50
1950
1960
1970
1980
1990
2000
2010
2020
Estimates Comparison
Bayesian estimates
GBD 2021 estimates
Data Type Name
Recent births
Recent births and average parity methods
Direct
Data Series Name
1961−1963 MPS (Panel)
1972−1973 VR (Panel)
1980 WFS (Survey)
1982 Census (Census)
1983−1984 CPS (Survey)
1986−1988 ENDPR (Panel)
1987 DHS (Survey)
1992 DHS (Survey)
1994 Census (Census)
1995 DHS Special (Panel)
1996−1998 ENDPR (Panel)
1997 ENSME (Survey)
1998−1999 ENNV (Survey)
2003−2004 DHS (Survey)
2004 Census (Census)
2011 ENPSF (Survey)

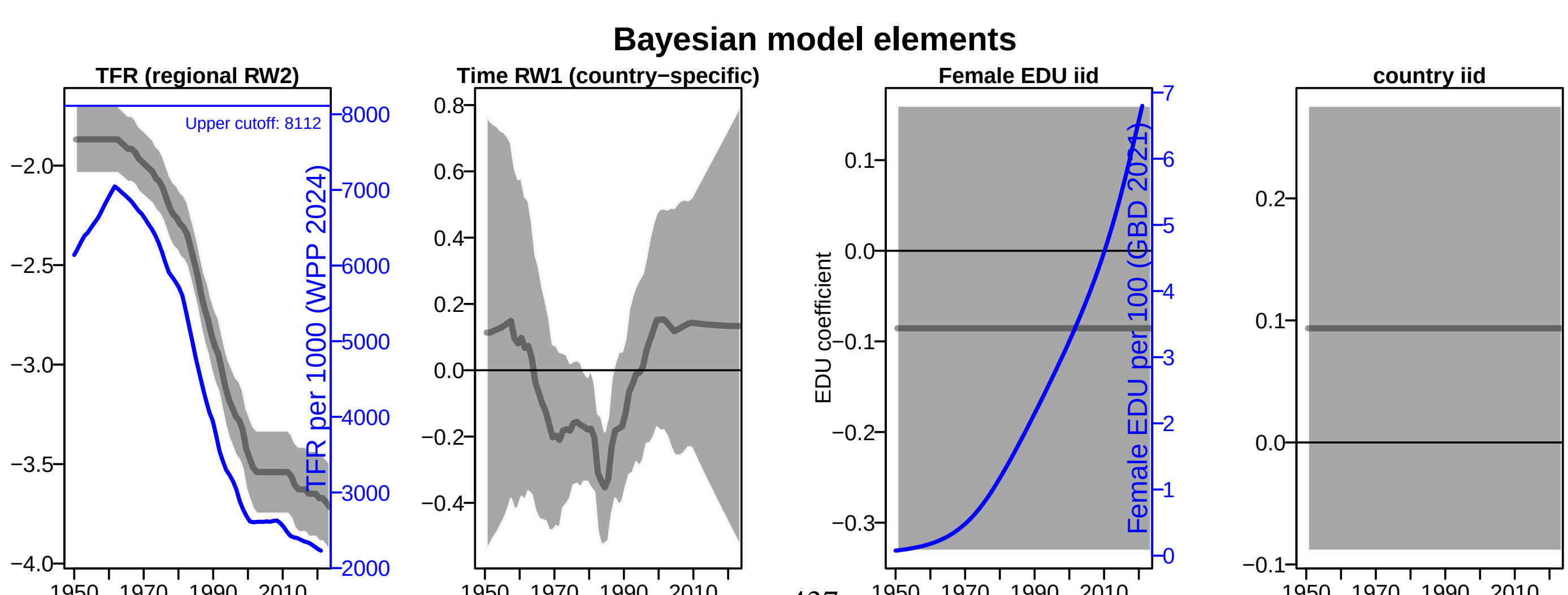
Bayesian model elements
TFR (regional RW2)
Upper cutoff: 8112
TFR per 1000 (WPP 2024)
Time RW1 (country−specific)
Female EDU iid
EDU coefficient
Female EDU per 100 (GBD 2021)
country iid

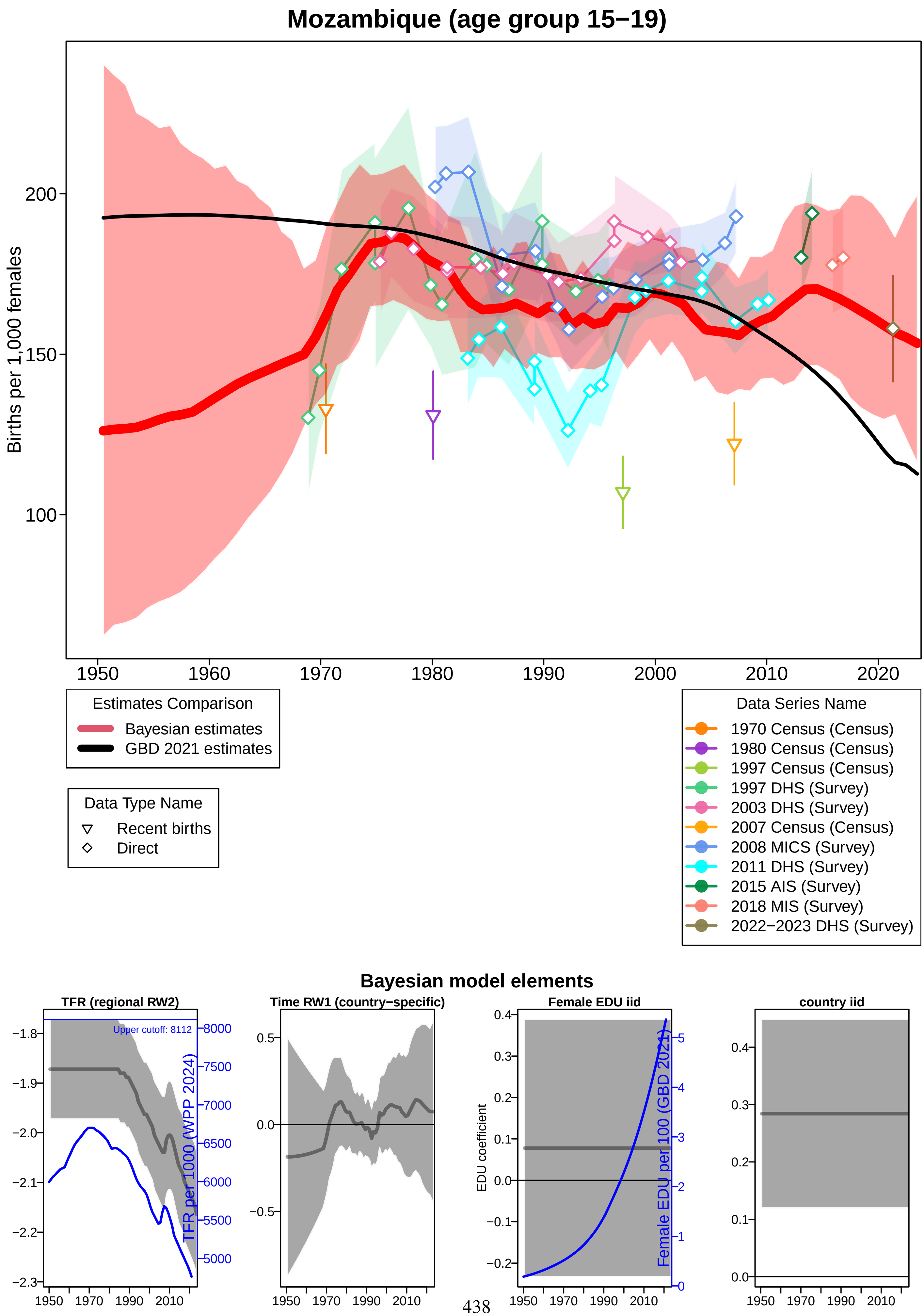

Mozambique (age group 15−19)
Births per 1,000 females
200
150
100
1950
1960
1970
1980
1990
2000
2010
2020
Estimates Comparison
Bayesian estimates
GBD 2021 estimates
Data Type Name
Recent births
Direct
Data Series Name
1970 Census (Census)
1980 Census (Census)
1997 Census (Census)
1997 DHS (Survey)
2003 DHS (Survey)
2007 Census (Census)
2008 MICS (Survey)
2011 DHS (Survey)
2015 AIS (Survey)
2018 MIS (Survey)
2022−2023 DHS (Survey)
Bayesian model elements
TFR (regional RW2)
Upper cutoff: 8112
TFR per 1000 (WPP 2024)
Time RW1 (country−specific)
Female EDU iid
EDU coefficient
Female EDU per 100 (GBD 2021)
country iid

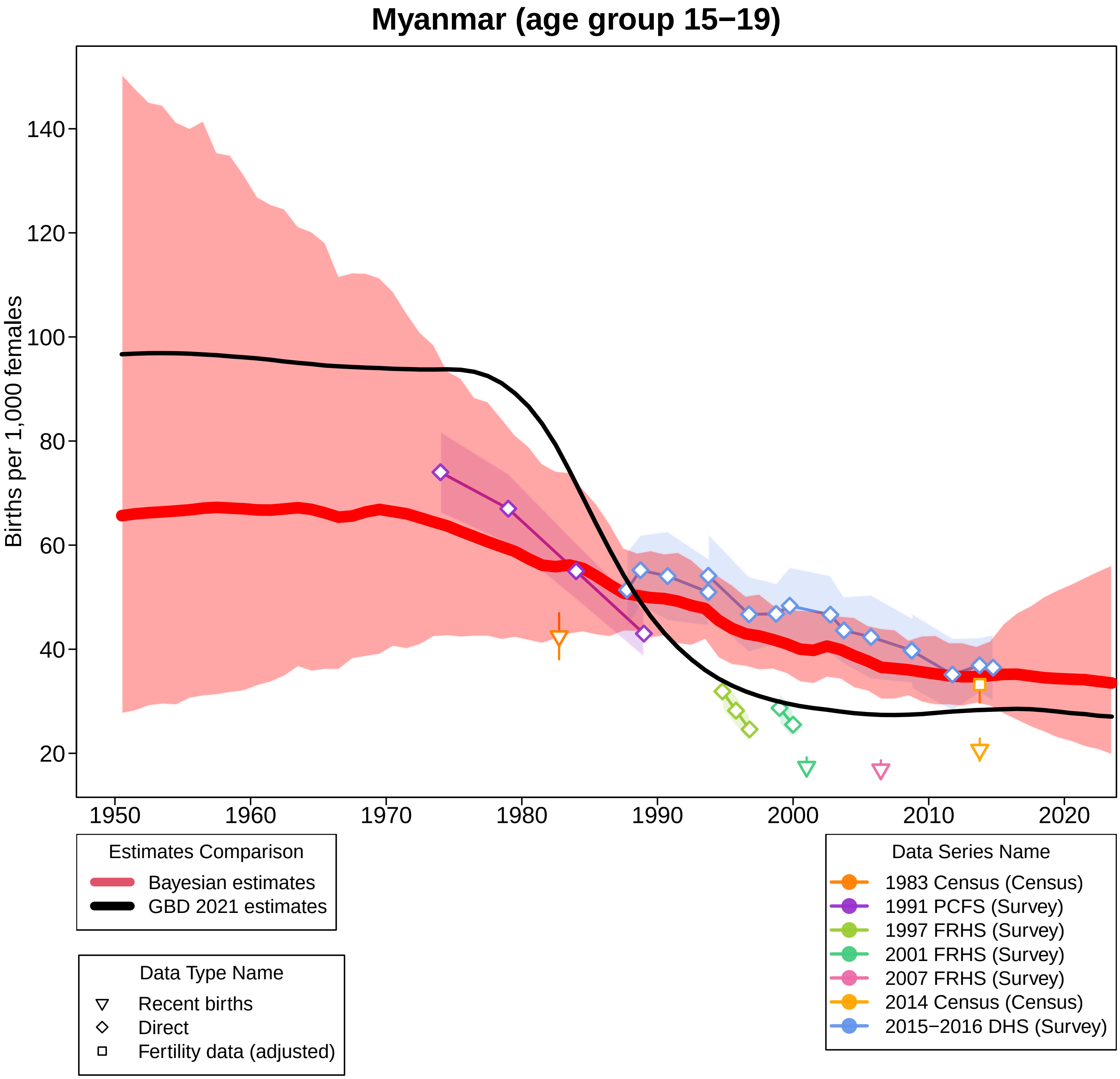
Myanmar (age group 15−19)
Births per 1,000 females
140
120
100
80
60
40
20
1950
1960
1970
1980
1990
2000
2010
2020
Estimates Comparison
Bayesian estimates
GBD 2021 estimates
Data Type Name
Recent births
Direct
Fertility data (adjusted)
Data Series Name
1983 Census (Census)
1991 PCFS (Survey)
1997 FRHS (Survey)
2001 FRHS (Survey)
2007 FRHS (Survey)
2014 Census (Census)
2015−2016 DHS (Survey)

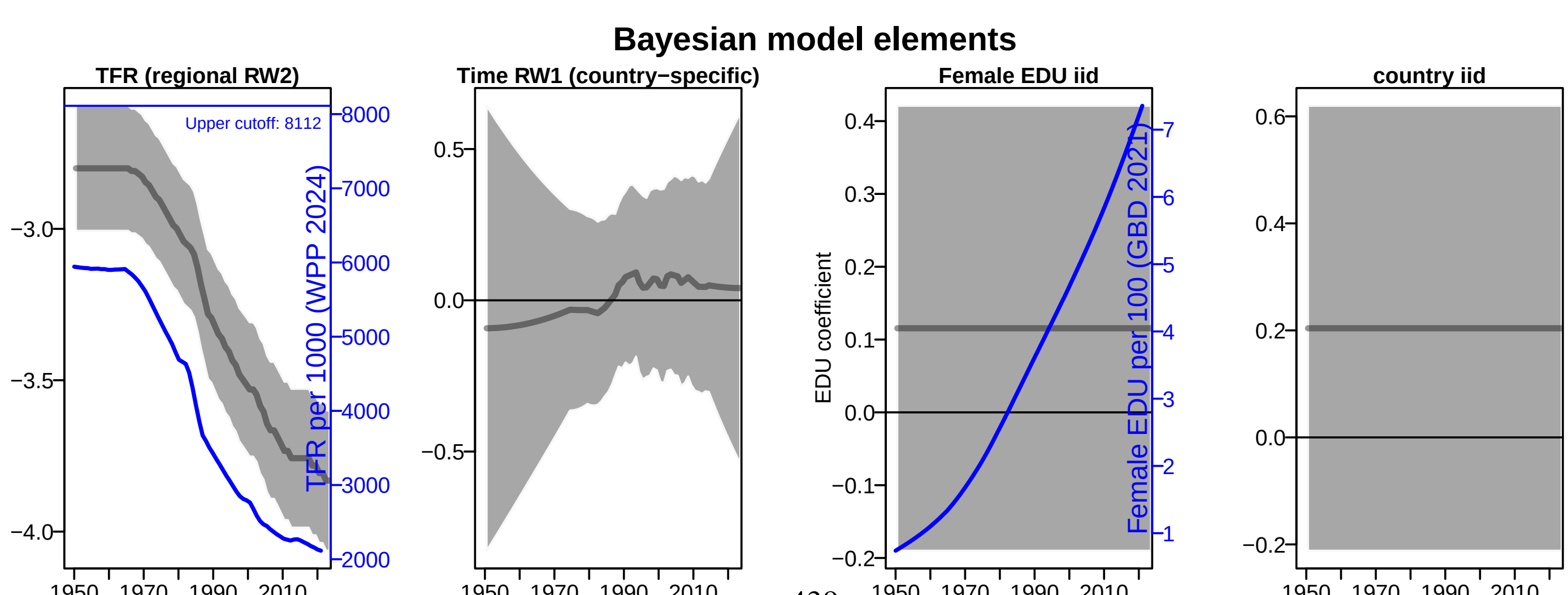
Bayesian model elements
TFR (regional RW2)
Upper cutoff: 8112
TFR per 1000 (WPP 2024)
Time RW1 (country−specific)
Female EDU iid
EDU coefficient
Female EDU per 100 (GBD 2021)
country iid

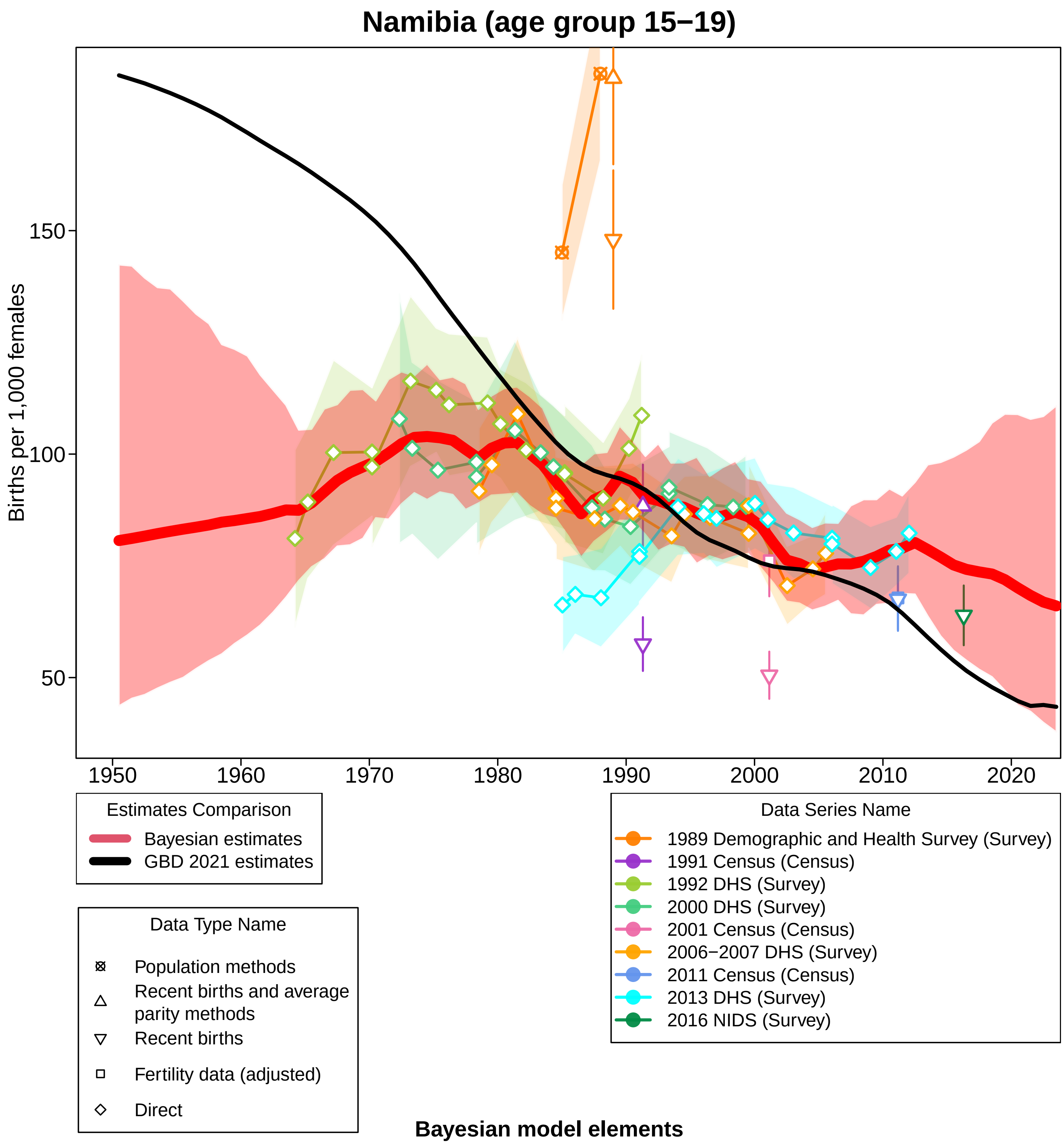
Namibia (age group 15−19)
Births per 1,000 females
150
100
50
1950
1960
1970
1980
1990
2000
2010
2020
Estimates Comparison
Bayesian estimates
GBD 2021 estimates
Data Series Name
1989 Demographic and Health Survey (Survey)
1991 Census (Census)
1992 DHS (Survey)
2000 DHS (Survey)
2001 Census (Census)
2006−2007 DHS (Survey)
2011 Census (Census)
2013 DHS (Survey)
2016 NIDS (Survey)
Data Type Name
Population methods
Recent births and average parity methods
Recent births
Fertility data (adjusted)
Direct

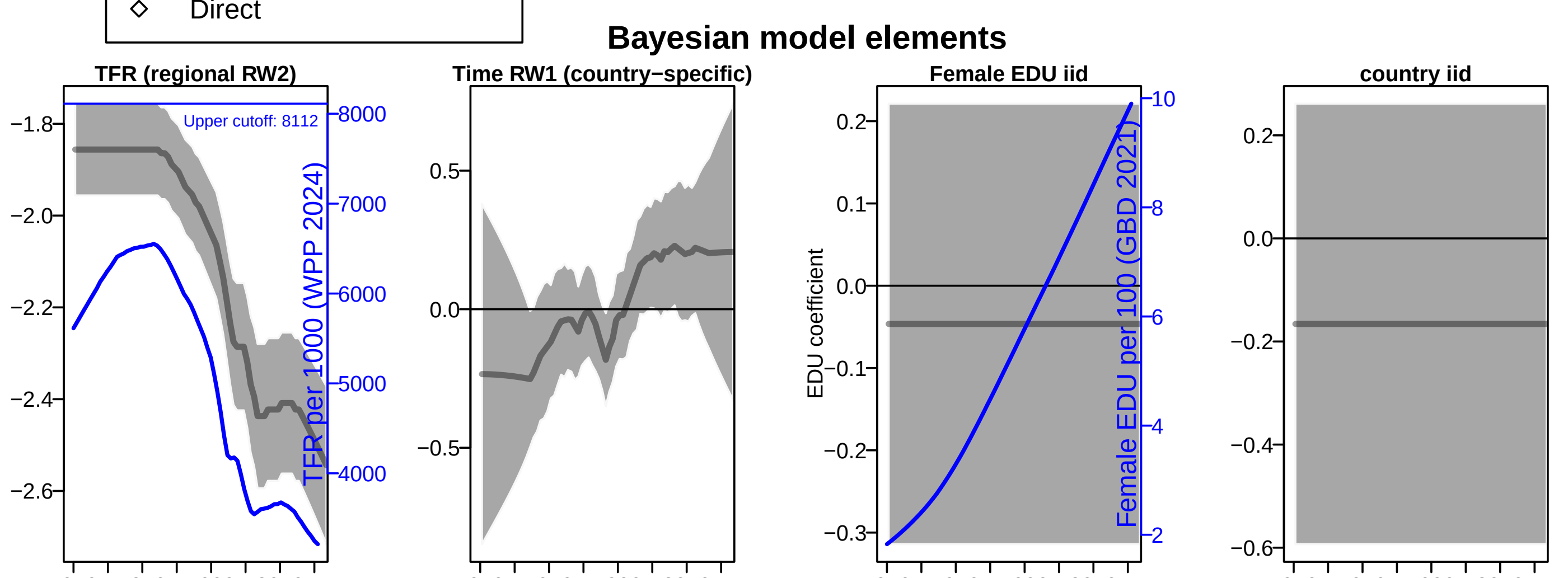
Bayesian model elements
TFR (regional RW2)
Upper cutoff: 8112
TFR per 1000 (WPP 2024)
Time RW1 (country−specific)
Female EDU iid
EDU coefficient
Female EDU per 100 (GBD 2021)
country iid

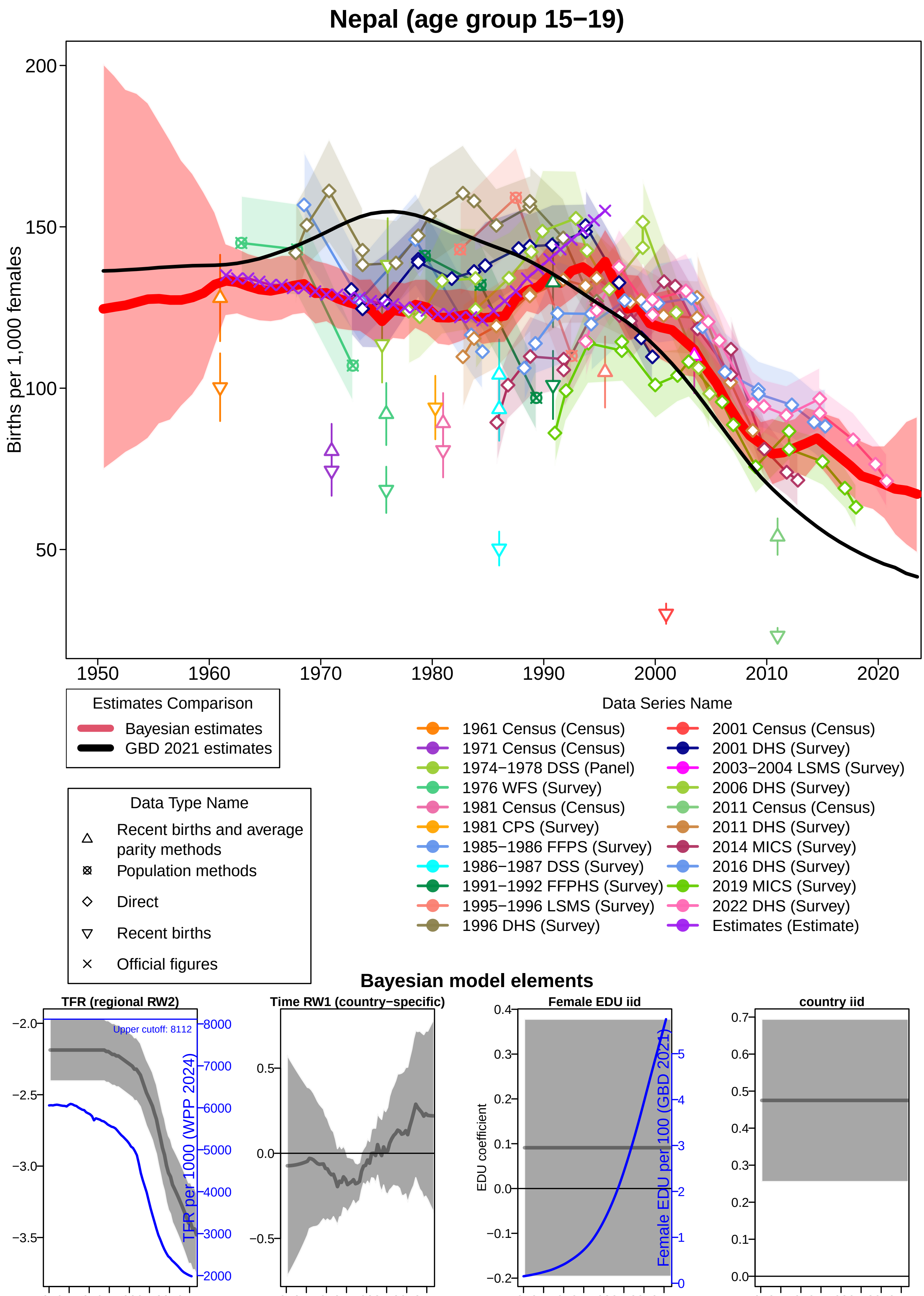

Nepal (age group 15−19)
Births per 1,000 females
200
150
100
50
1950
1960
1970
1980
1990
2000
2010
2020
Estimates Comparison
Bayesian estimates
GBD 2021 estimates
Data Type Name
Recent births and average parity methods
Population methods
Direct
Recent births
Official figures
Data Series Name
1961 Census (Census)
1971 Census (Census)
1974−1978 DSS (Panel)
1976 WFS (Survey)
1981 Census (Census)
1981 CPS (Survey)
1985−1986 FFPS (Survey)
1986−1987 DSS (Survey)
1991−1992 FFPHS (Survey)
1995−1996 LSMS (Survey)
1996 DHS (Survey)
2001 Census (Census)
2001 DHS (Survey)
2003−2004 LSMS (Survey)
2006 DHS (Survey)
2011 Census (Census)
2011 DHS (Survey)
2014 MICS (Survey)
2016 DHS (Survey)
2019 MICS (Survey)
2022 DHS (Survey)
Estimates (Estimate)
Bayesian model elements
TFR (regional RW2)
Upper cutoff: 8112
TFR per 1000 (WPP 2024)
Time RW1 (country−specific)
Female EDU iid
EDU coefficient
Female EDU per 100 (GBD 2021)
country iid

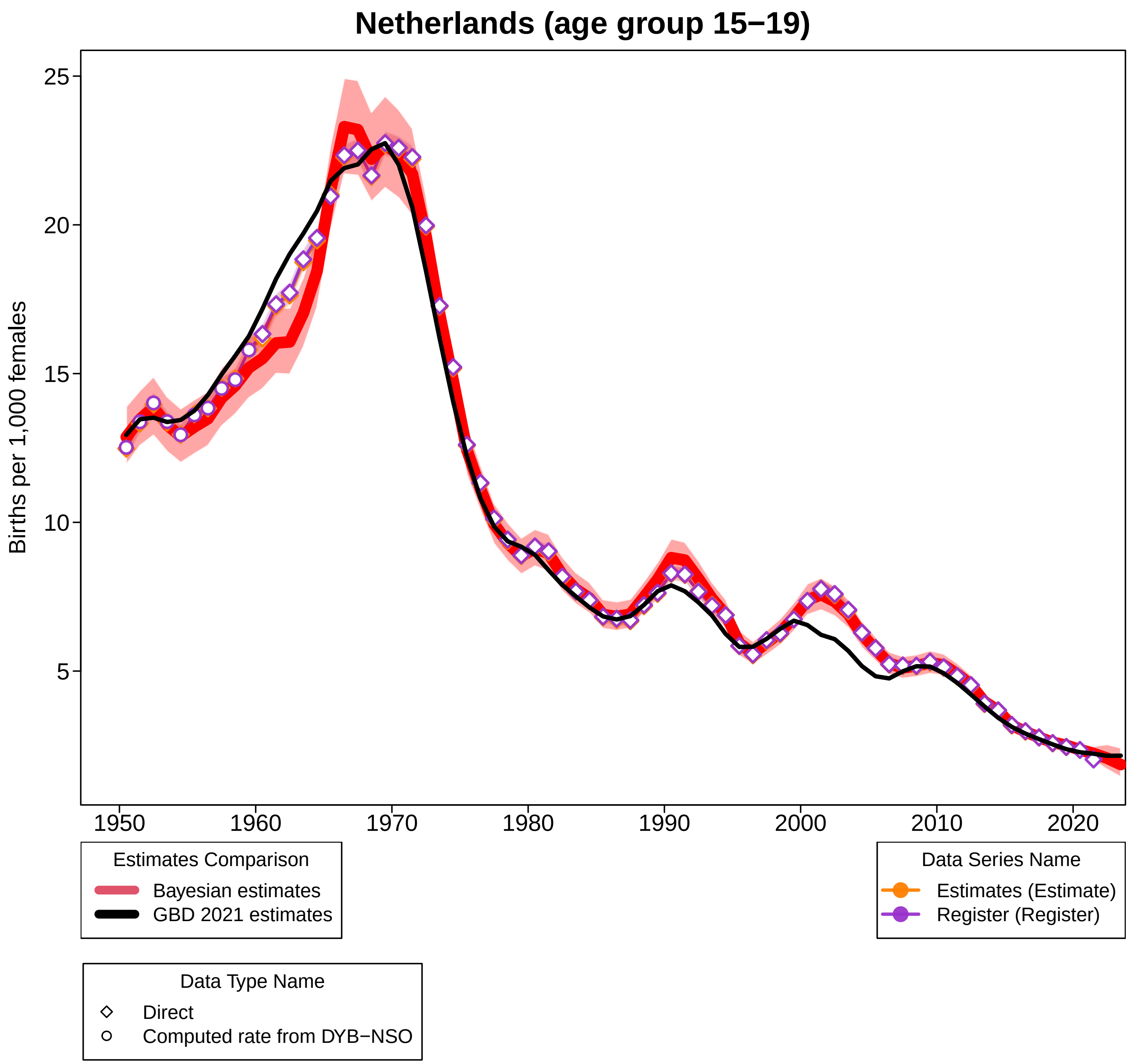
Netherlands (age group 15−19)
Births per 1,000 females
25
20
15
10
5
1950
1960
1970
1980
1990
2000
2010
2020
Estimates Comparison
Bayesian estimates
GBD 2021 estimates
Data Series Name
Estimates (Estimate)
Register (Register)
Data Type Name
Direct
Computed rate from DYB−NSO

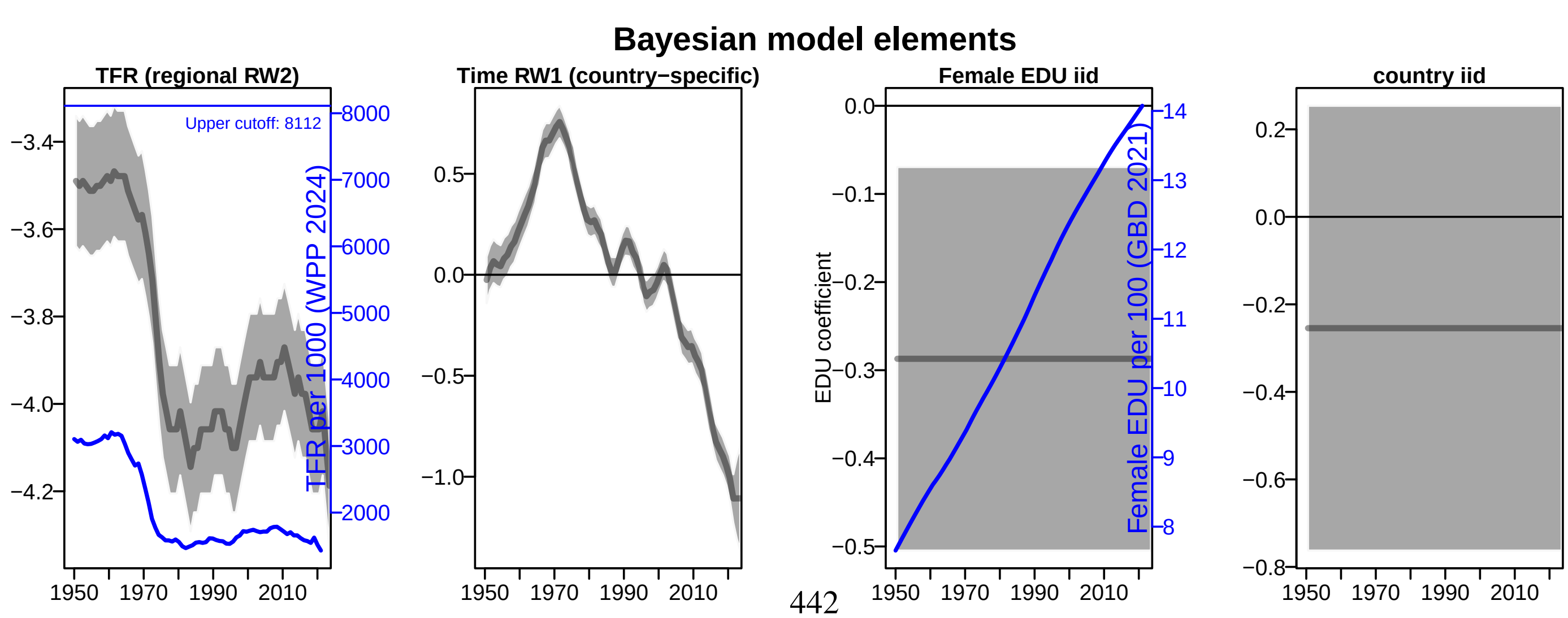
Bayesian model elements
TFR (regional RW2)
Upper cutoff: 8112
TFR per 1000 (WPP 2024)
Time RW1 (country−specific)
Female EDU iid
EDU coefficient
Female EDU per 100 (GBD 2021)
country iid

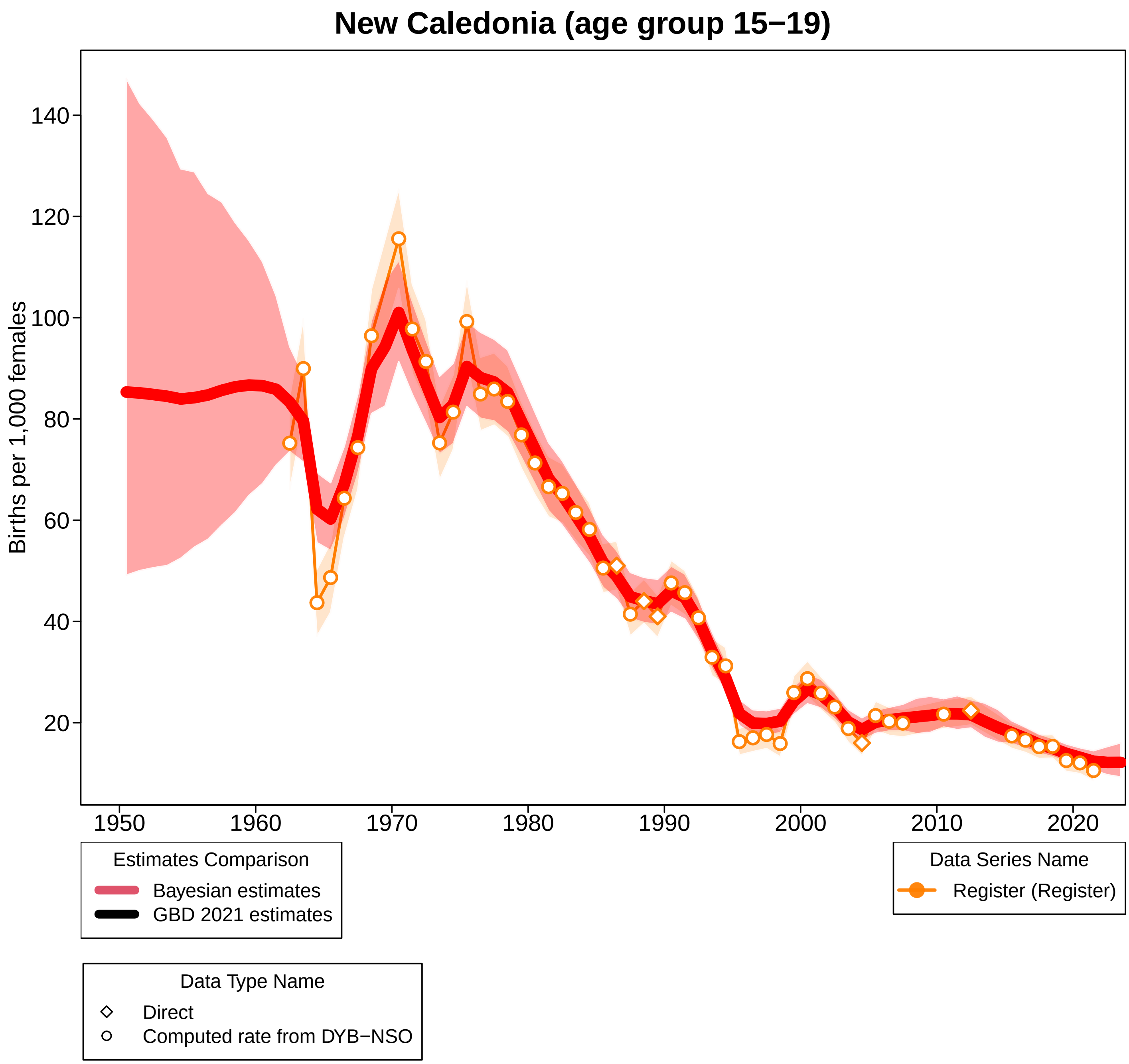
New Caledonia (age group 15−19)
Births per 1,000 females
140
120
100
80
60
40
20
1950
1960
1970
1980
1990
2000
2010
2020
Estimates Comparison
Bayesian estimates
GBD 2021 estimates
Data Series Name
Register (Register)
Data Type Name
Direct
Computed rate from DYB−NSO

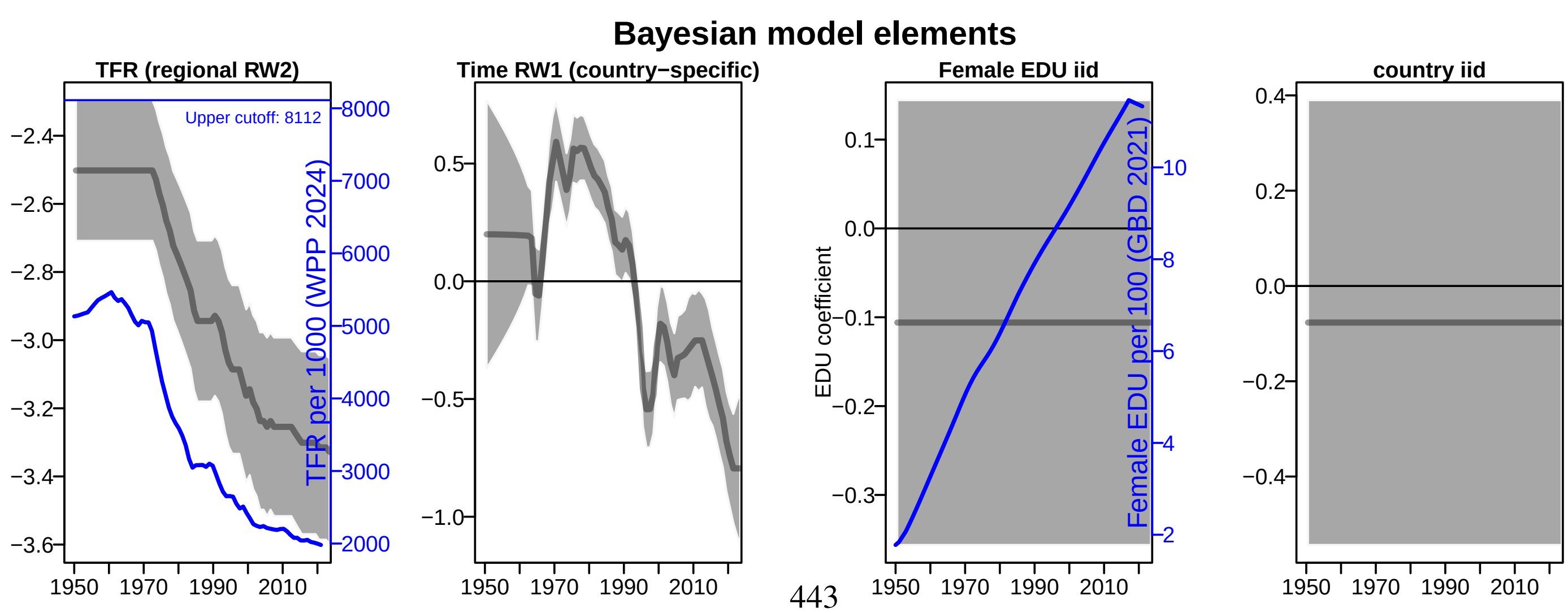
Bayesian model elements
TFR (regional RW2)
Upper cutoff: 8112
TFR per 1000 (WPP 2024)
Time RW1 (country−specific)
Female EDU iid
EDU coefficient
Female EDU per 100 (GBD 2021)
country iid

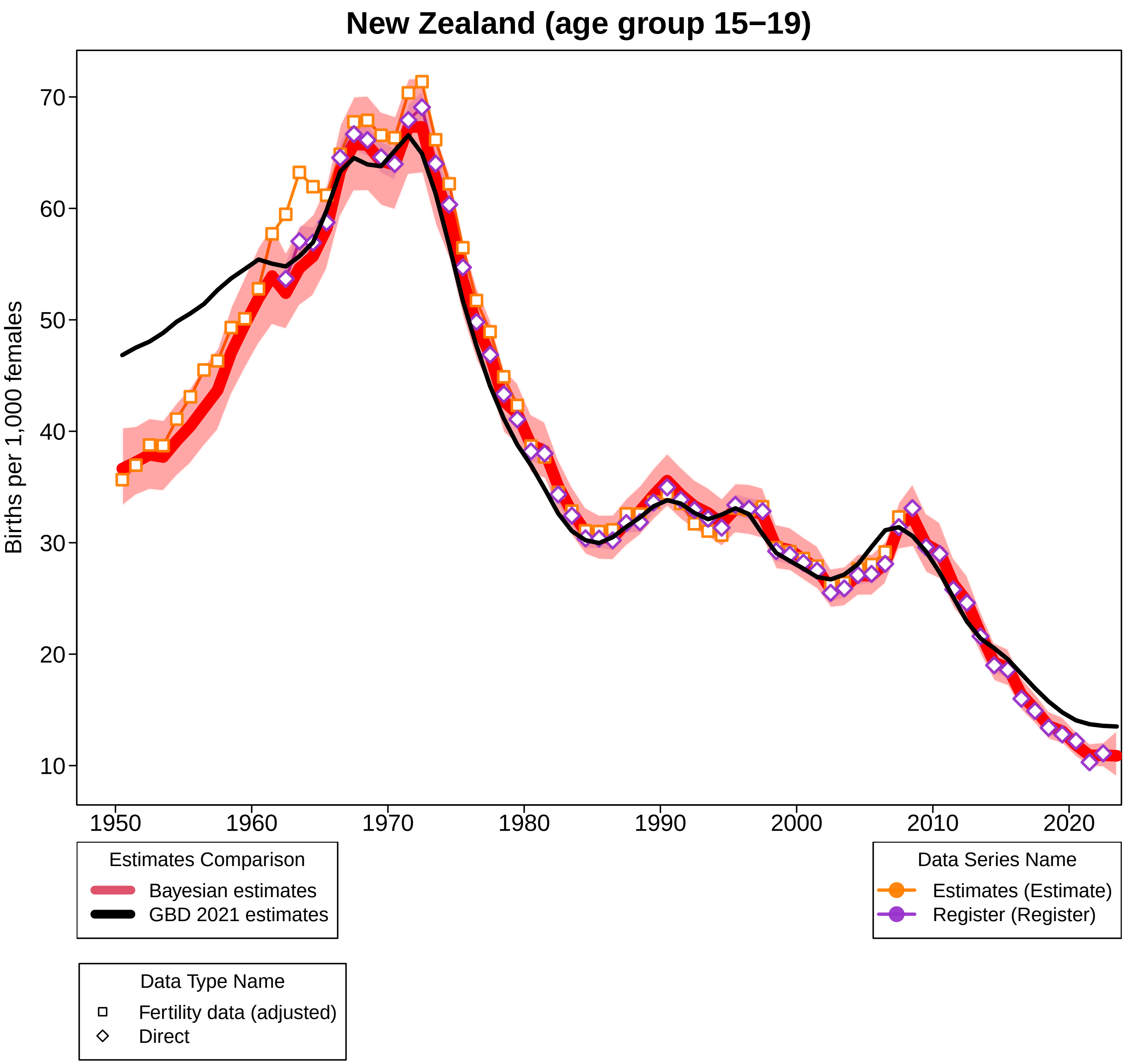
New Zealand (age group 15−19)
Births per 1,000 females
70
60
50
40
30
20
10
1950
1960
1970
1980
1990
2000
2010
2020
Estimates Comparison
Bayesian estimates
GBD 2021 estimates
Data Series Name
Estimates (Estimate)
Register (Register)
Data Type Name
Fertility data (adjusted)
Direct

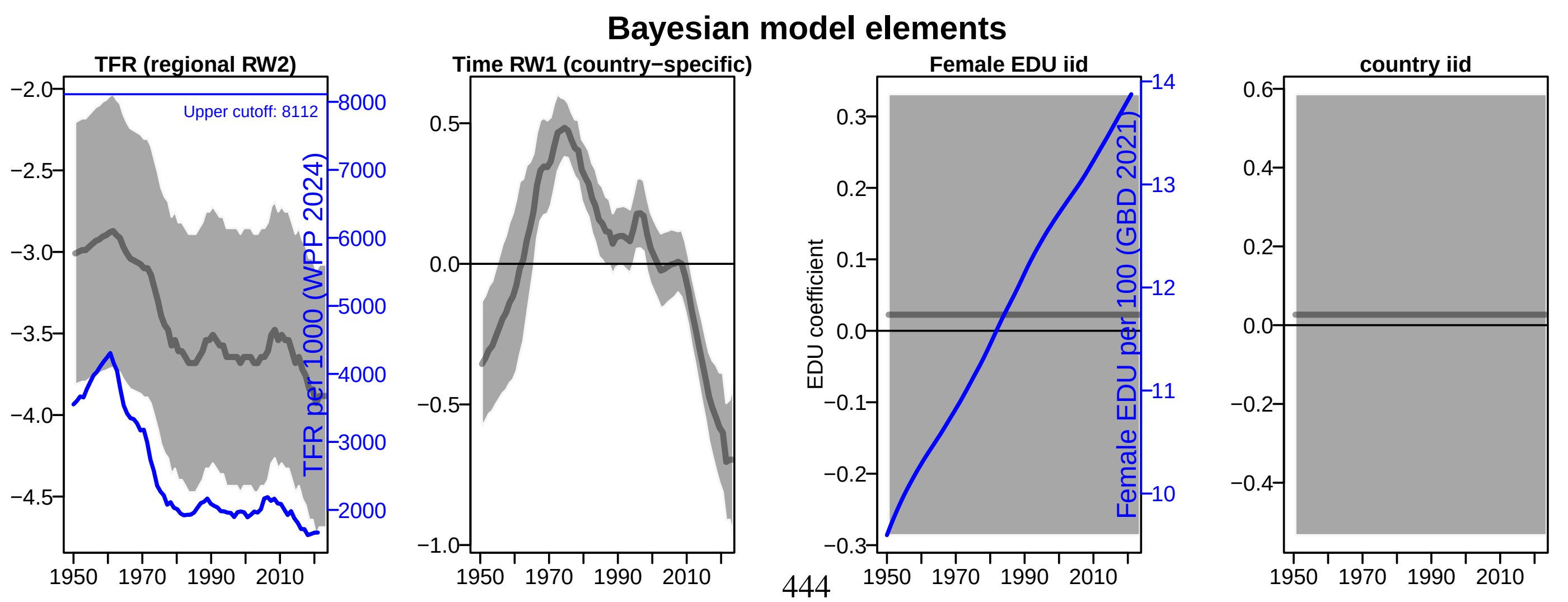
Bayesian model elements
TFR (regional RW2)
Upper cutoff: 8112
TFR per 1000 (WPP 2024)
Time RW1 (country−specific)
Female EDU iid
EDU coefficient
Female EDU per 100 (GBD 2021)
country iid

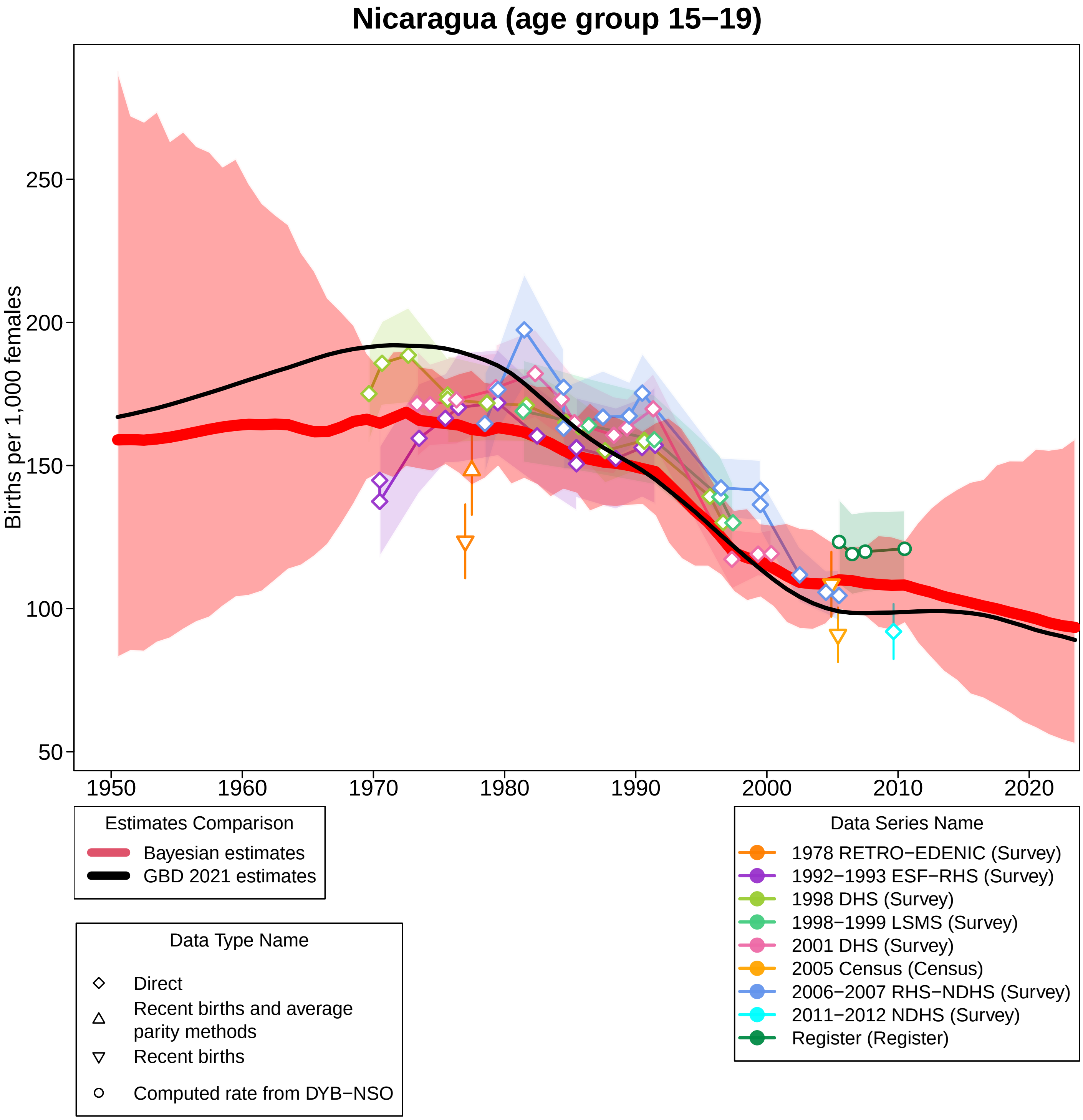
Nicaragua (age group 15−19)
Births per 1,000 females
250
200
150
100
50
1950
1960
1970
1980
1990
2000
2010
2020
Estimates Comparison
Bayesian estimates
GBD 2021 estimates
Data Series Name
1978 RETRO−EDENIC (Survey)
1992−1993 ESF−RHS (Survey)
1998 DHS (Survey)
1998−1999 LSMS (Survey)
2001 DHS (Survey)
2005 Census (Census)
2006−2007 RHS−NDHS (Survey)
2011−2012 NDHS (Survey)
Register (Register)
Data Type Name
Direct
Recent births and average parity methods
Recent births
Computed rate from DYB−NSO

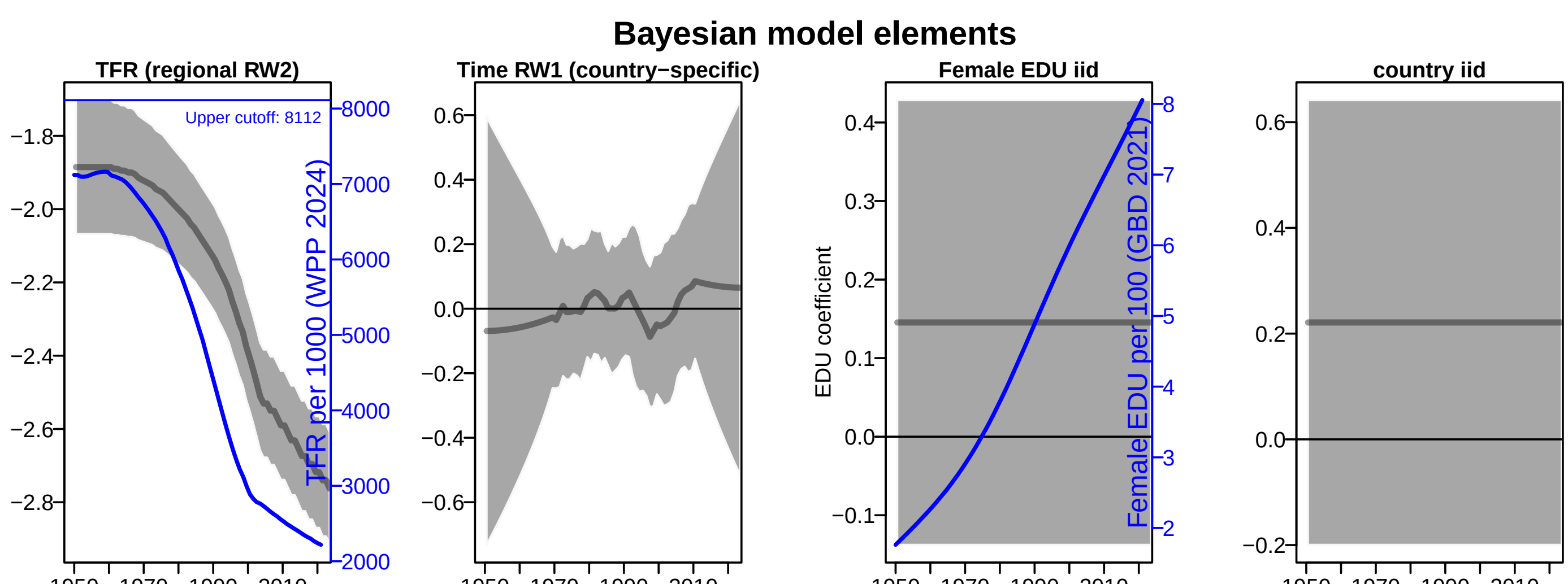
Bayesian model elements
TFR (regional RW2)
Upper cutoff: 8112
TFR per 1000 (WPP 2024)
Time RW1 (country−specific)
Female EDU iid
EDU coefficient
Female EDU per 100 (GBD 2021)
country iid
1950
1970
1990
2010

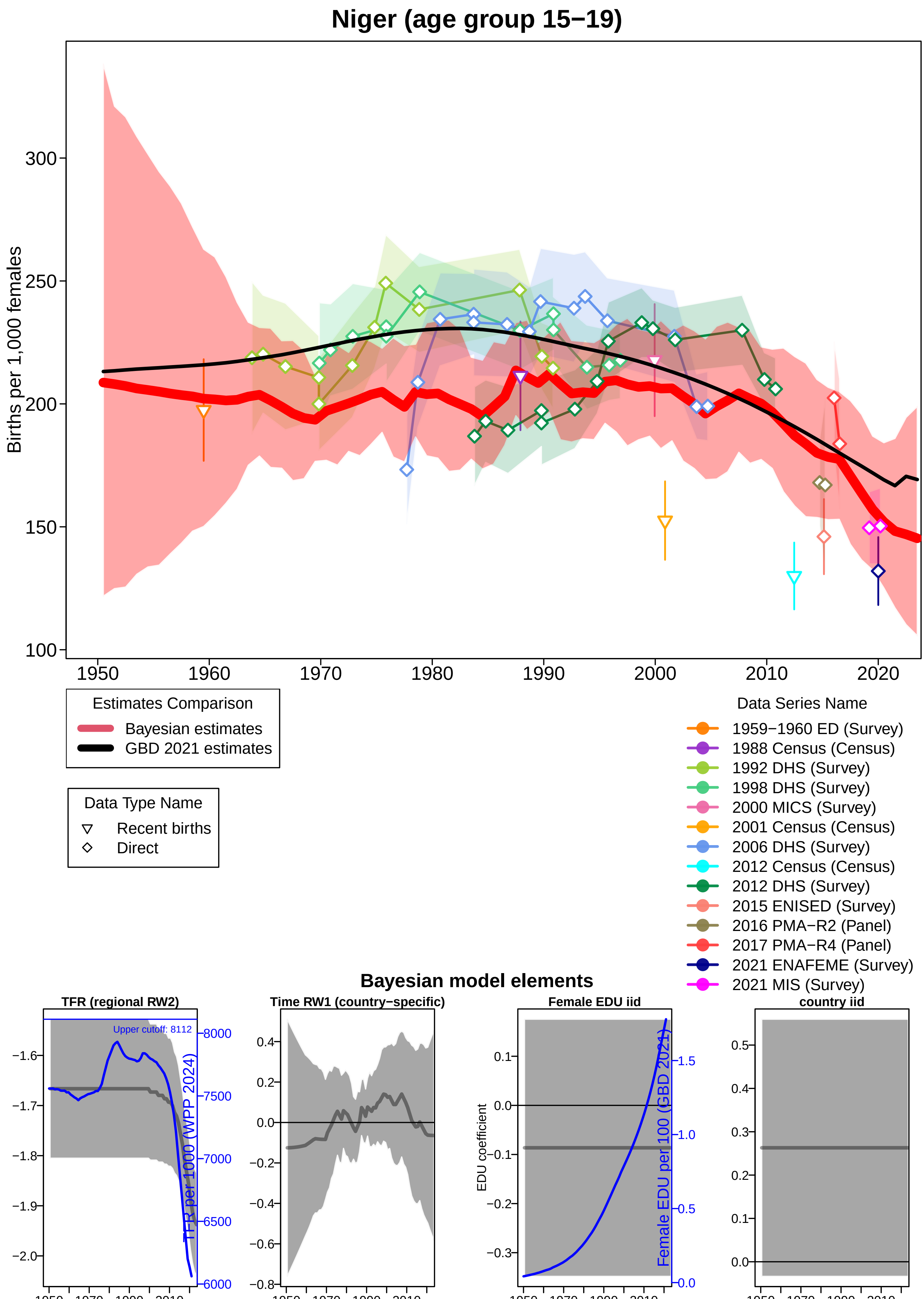

Niger (age group 15−19)
Births per 1,000 females
300
250
200
150
100
1950
1960
1970
1980
1990
2000
2010
2020
Estimates Comparison
Bayesian estimates
GBD 2021 estimates
Data Type Name
Recent births
Direct
Data Series Name
1959−1960 ED (Survey)
1988 Census (Census)
1992 DHS (Survey)
1998 DHS (Survey)
2000 MICS (Survey)
2001 Census (Census)
2006 DHS (Survey)
2012 Census (Census)
2012 DHS (Survey)
2015 ENISED (Survey)
2016 PMA−R2 (Panel)
2017 PMA−R4 (Panel)
2021 ENAFEME (Survey)
2021 MIS (Survey)
Bayesian model elements
TFR (regional RW2)
Upper cutoff: 8112
TFR per 1000 (WPP 2024)
−1.6
−1.7
−1.8
−1.9
−2.0
8000
7500
7000
6500
6000
Time RW1 (country−specific)
0.4
0.2
0.0
−0.2
−0.4
−0.6
−0.8
Female EDU iid
EDU coefficient
Female EDU per 100 (GBD 2021)
0.1
0.0
−0.1
−0.2
−0.3
1.5
1.0
0.5
0.0
country iid
0.5
0.4
0.3
0.2
0.1
0.0
1950
1970
1990
2010

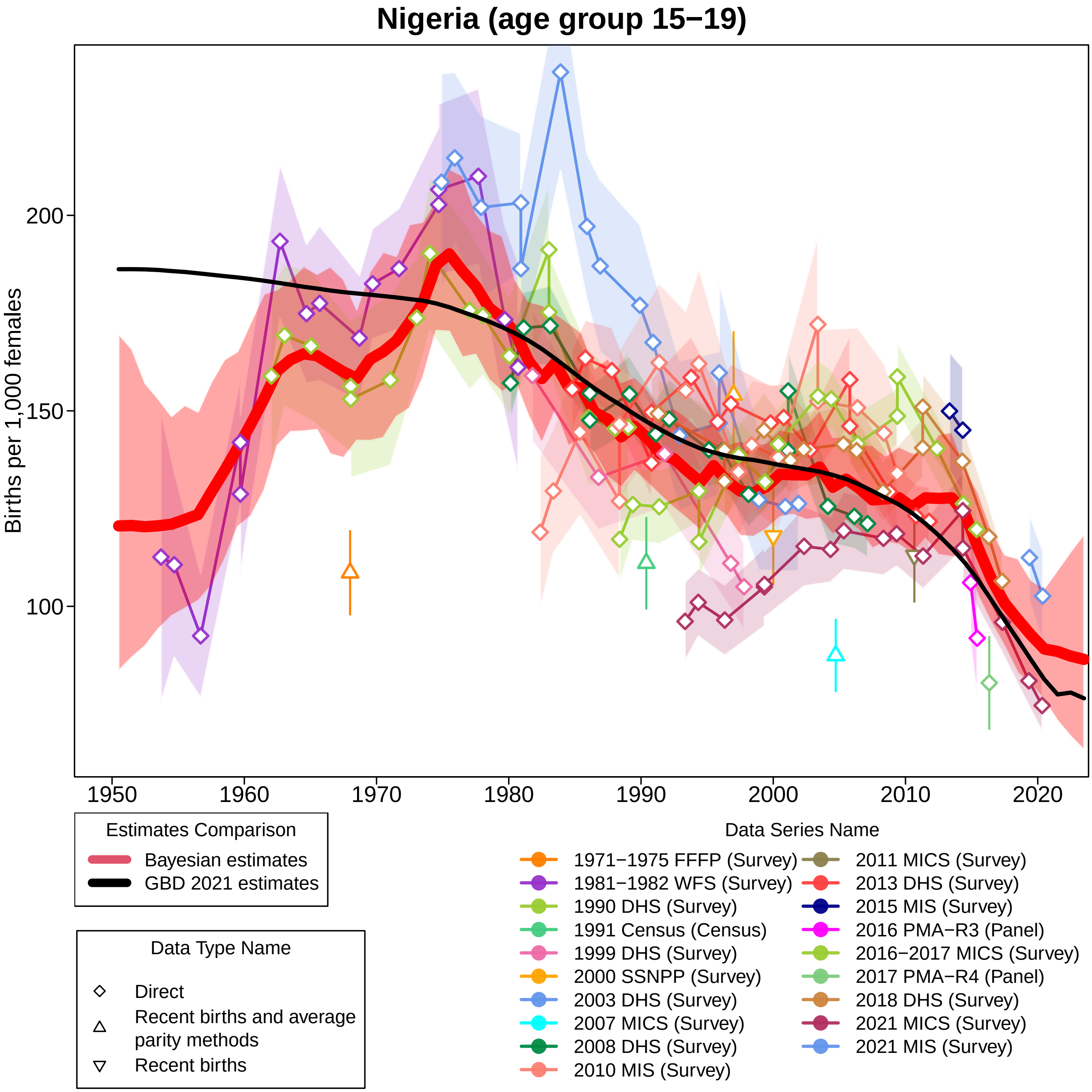
Nigeria (age group 15−19)
Births per 1,000 females
100
150
200
1950
1960
1970
1980
1990
2000
2010
2020
Estimates Comparison
Bayesian estimates
GBD 2021 estimates
Data Type Name
Direct
Recent births and average parity methods
Recent births
Data Series Name
1971−1975 FFFP (Survey)
1981−1982 WFS (Survey)
1990 DHS (Survey)
1991 Census (Census)
1999 DHS (Survey)
2000 SSNPP (Survey)
2003 DHS (Survey)
2007 MICS (Survey)
2008 DHS (Survey)
2010 MIS (Survey)
2011 MICS (Survey)
2013 DHS (Survey)
2015 MIS (Survey)
2016 PMA−R3 (Panel)
2016−2017 MICS (Survey)
2017 PMA−R4 (Panel)
2018 DHS (Survey)
2021 MICS (Survey)
2021 MIS (Survey)

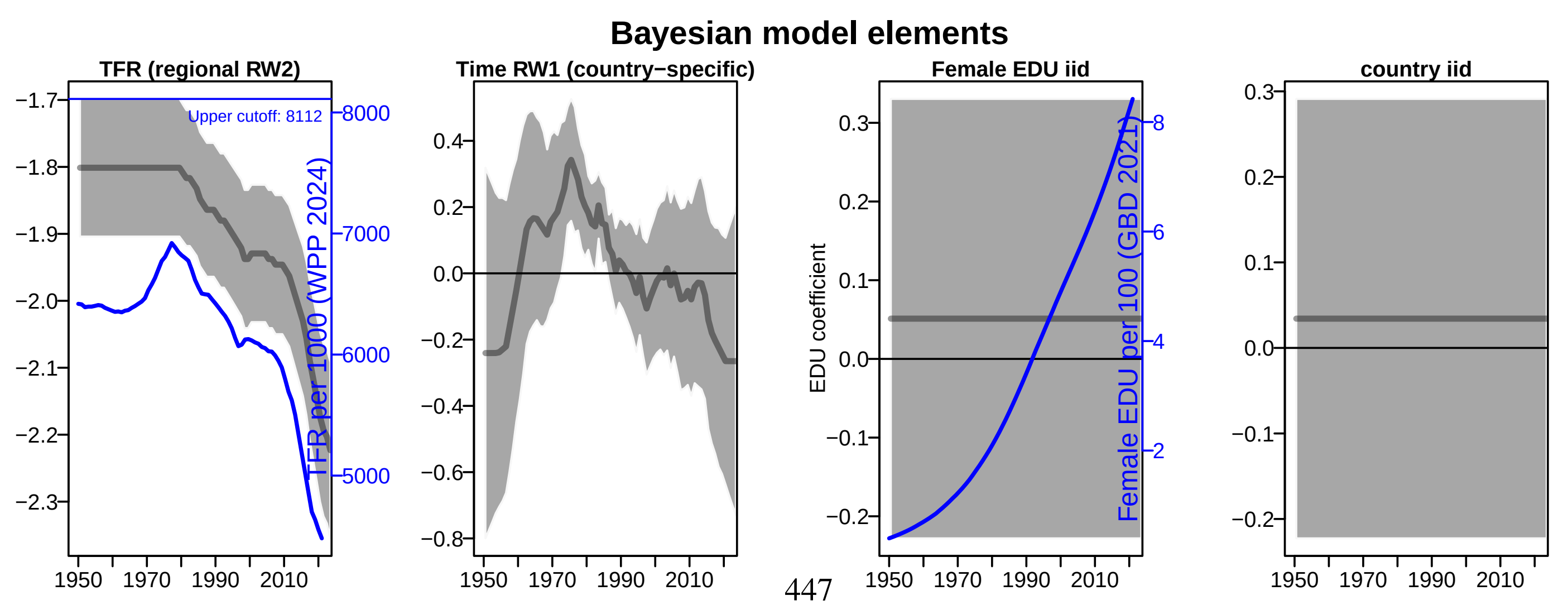
Bayesian model elements
TFR (regional RW2)
Upper cutoff: 8112
TFR per 1000 (WPP 2024)
Time RW1 (country−specific)
Female EDU iid
EDU coefficient
Female EDU per 100 (GBD 2021)
country iid
1950
1970
1990
2010

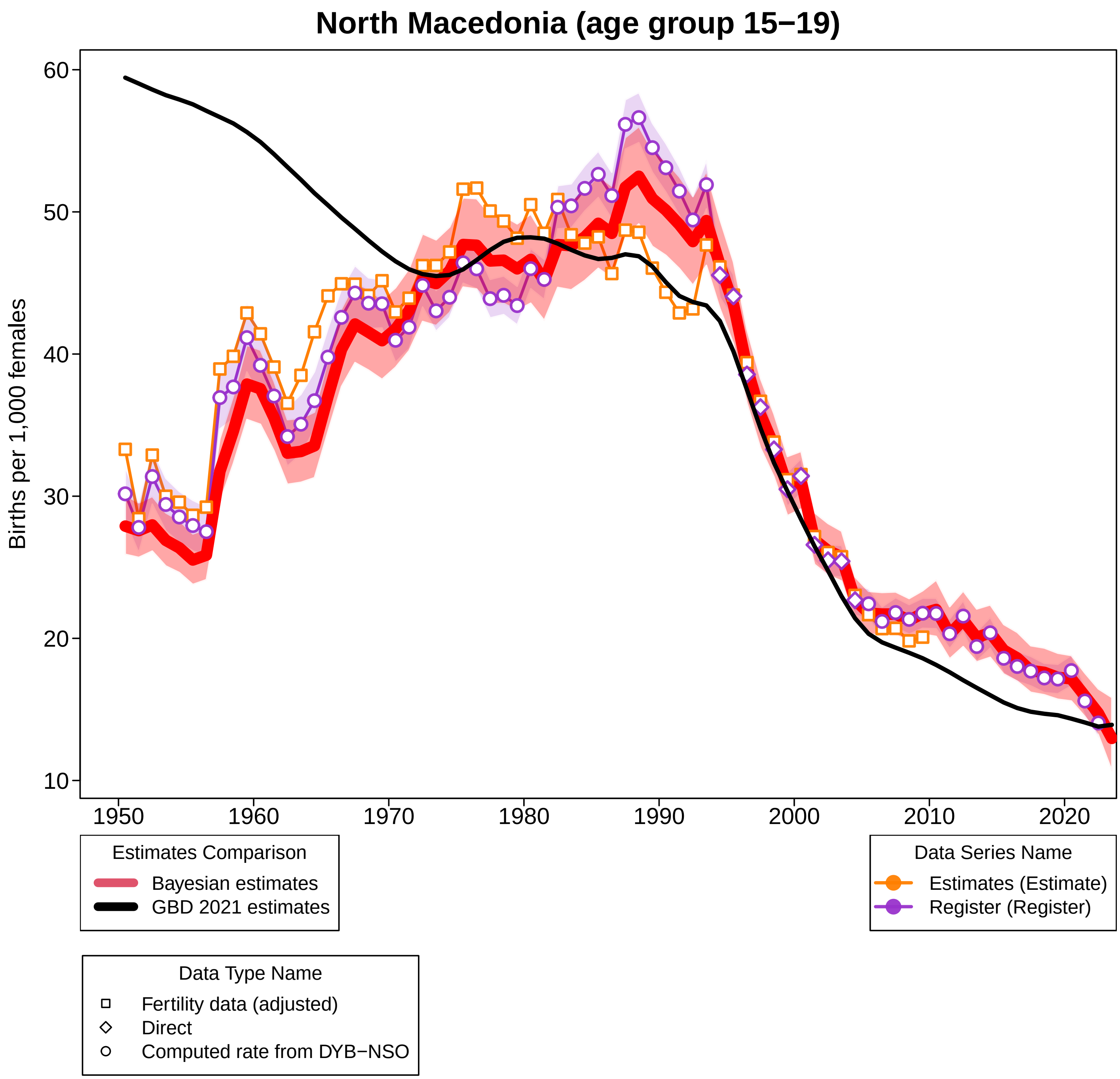
North Macedonia (age group 15−19)
Births per 1,000 females
60
50
40
30
20
10
1950
1960
1970
1980
1990
2000
2010
2020
Estimates Comparison
Bayesian estimates
GBD 2021 estimates
Data Series Name
Estimates (Estimate)
Register (Register)
Data Type Name
Fertility data (adjusted)
Direct
Computed rate from DYB−NSO

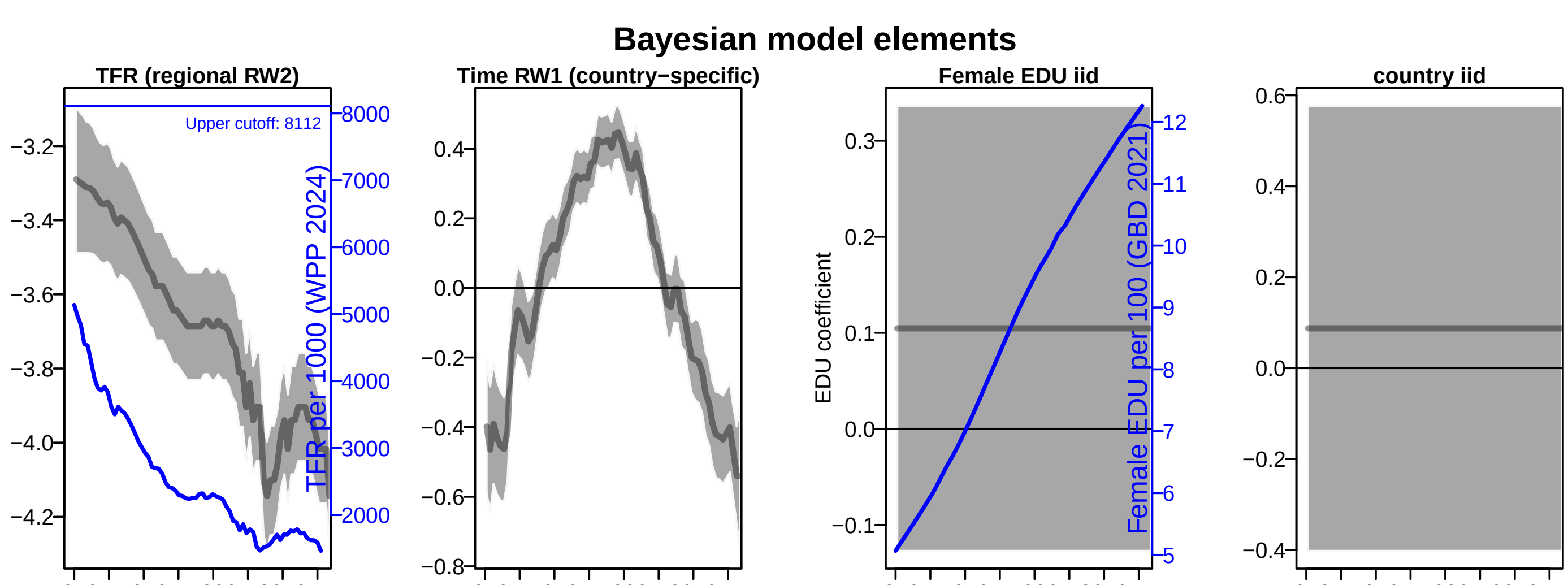
Bayesian model elements
TFR (regional RW2)
Upper cutoff: 8112
TFR per 1000 (WPP 2024)
Time RW1 (country−specific)
Female EDU iid
EDU coefficient
Female EDU per 100 (GBD 2021)
country iid
1950
1970
1990
2010

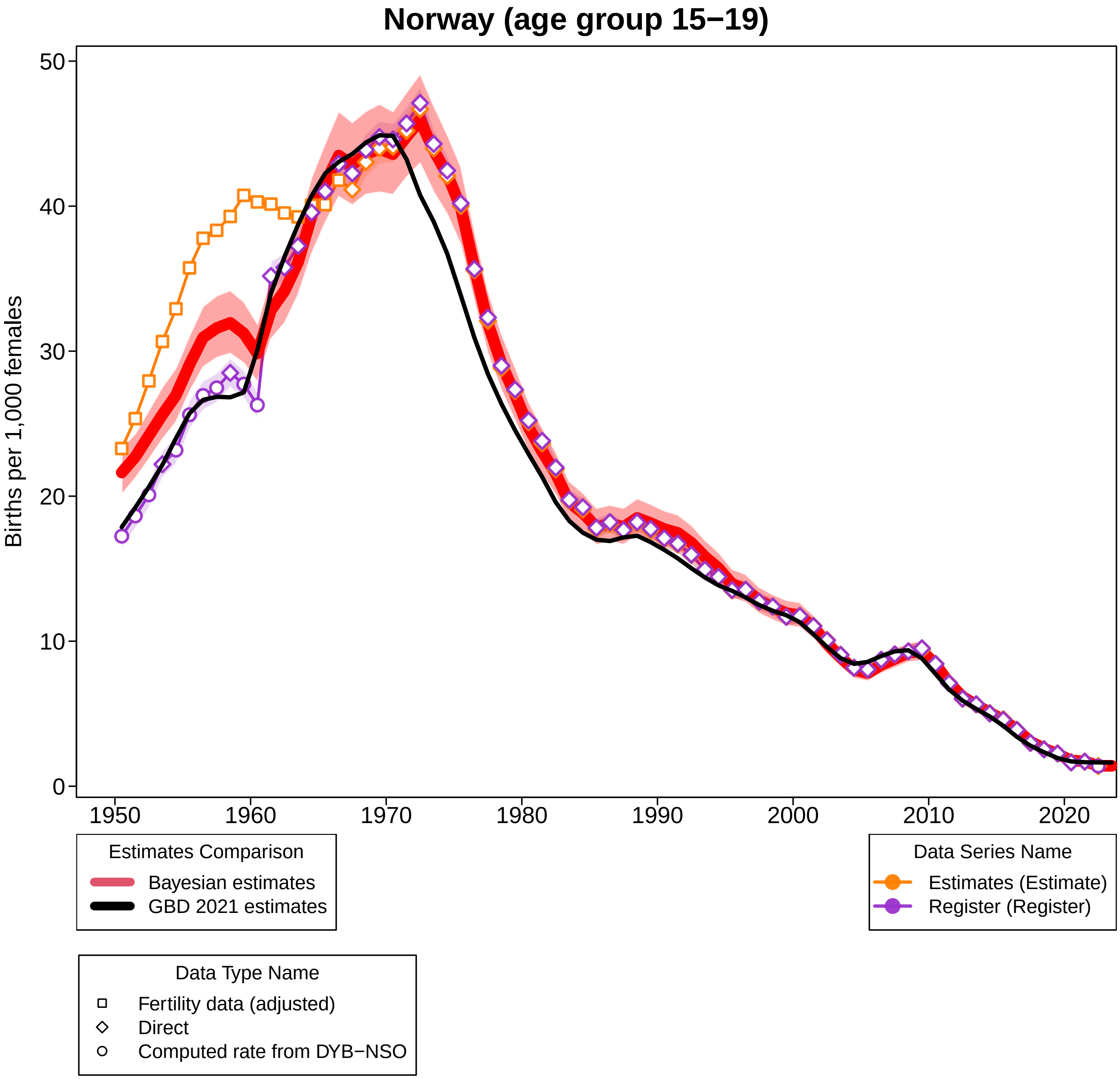

Norway (age group 15−19)
Births per 1,000 females
Estimates Comparison
Bayesian estimates
GBD 2021 estimates
Data Series Name
Estimates (Estimate)
Register (Register)
Data Type Name
Fertility data (adjusted)
Direct
Computed rate from DYB−NSO

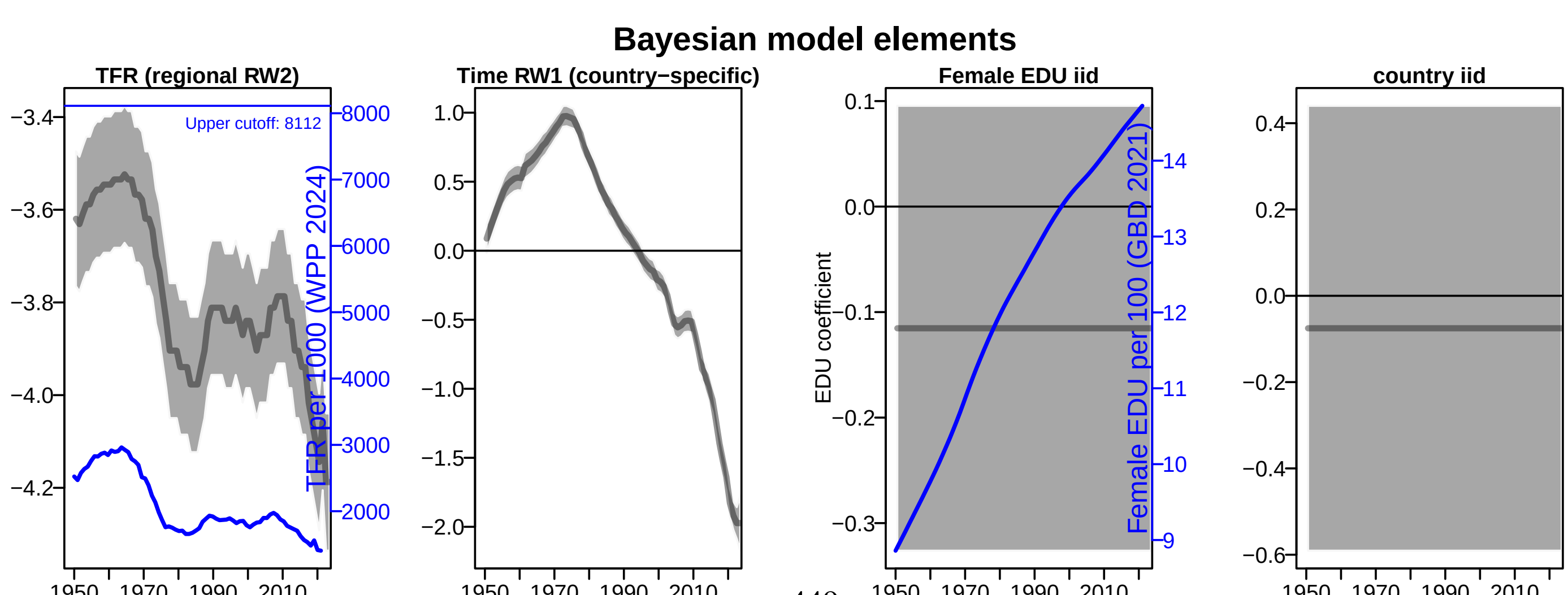

Bayesian model elements
TFR (regional RW2)
Upper cutoff: 8112
TFR per 1000 (WPP 2024)
Time RW1 (country−specific)
Female EDU iid
EDU coefficient
Female EDU per 100 (GBD 2021)
country iid

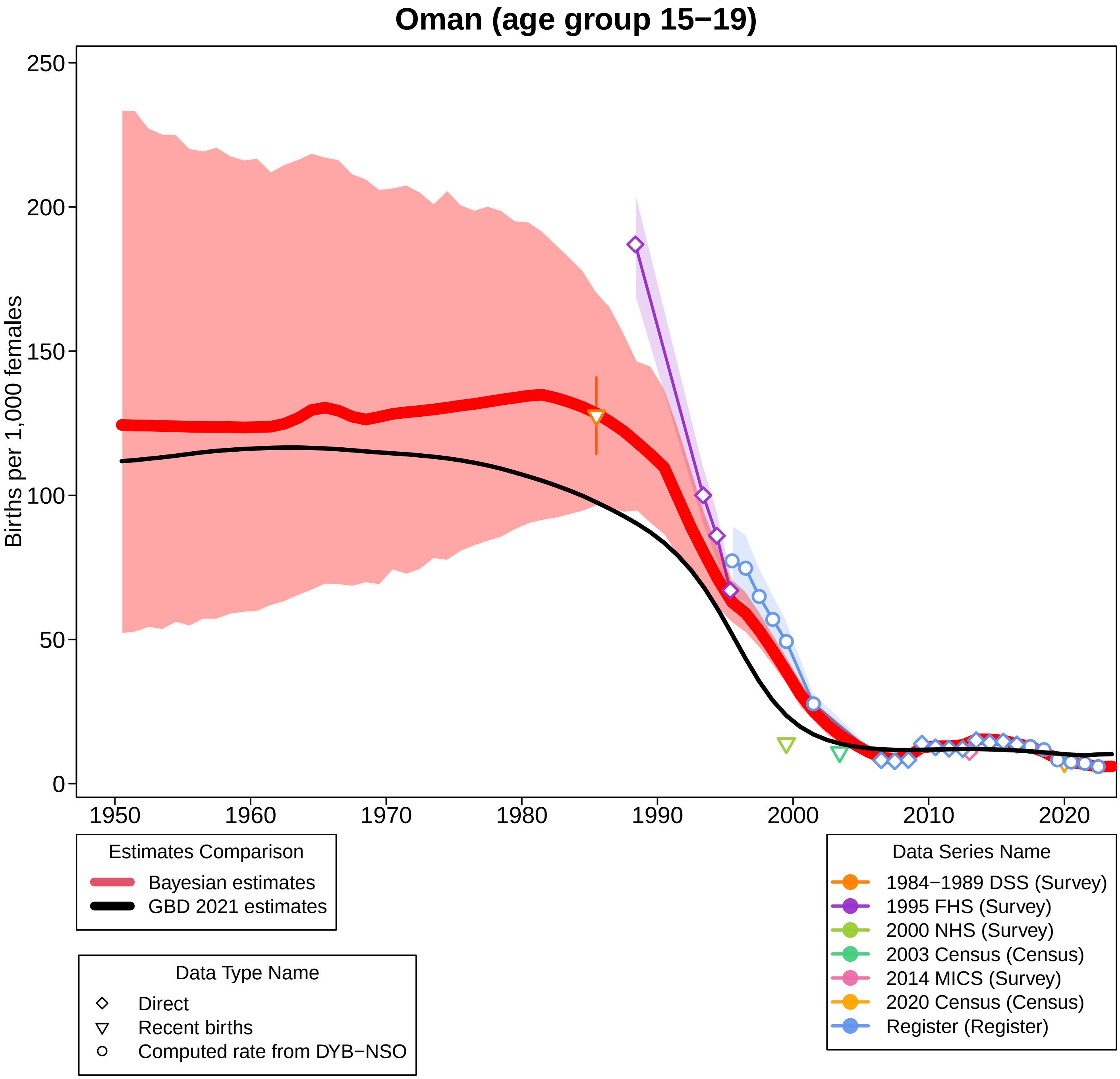
Oman (age group 15−19)
Births per 1,000 females
250
200
150
100
50
0
1950
1960
1970
1980
1990
2000
2010
2020
Estimates Comparison
Bayesian estimates
GBD 2021 estimates
Data Type Name
Direct
Recent births
Computed rate from DYB−NSO
Data Series Name
1984−1989 DSS (Survey)
1995 FHS (Survey)
2000 NHS (Survey)
2003 Census (Census)
2014 MICS (Survey)
2020 Census (Census)
Register (Register)

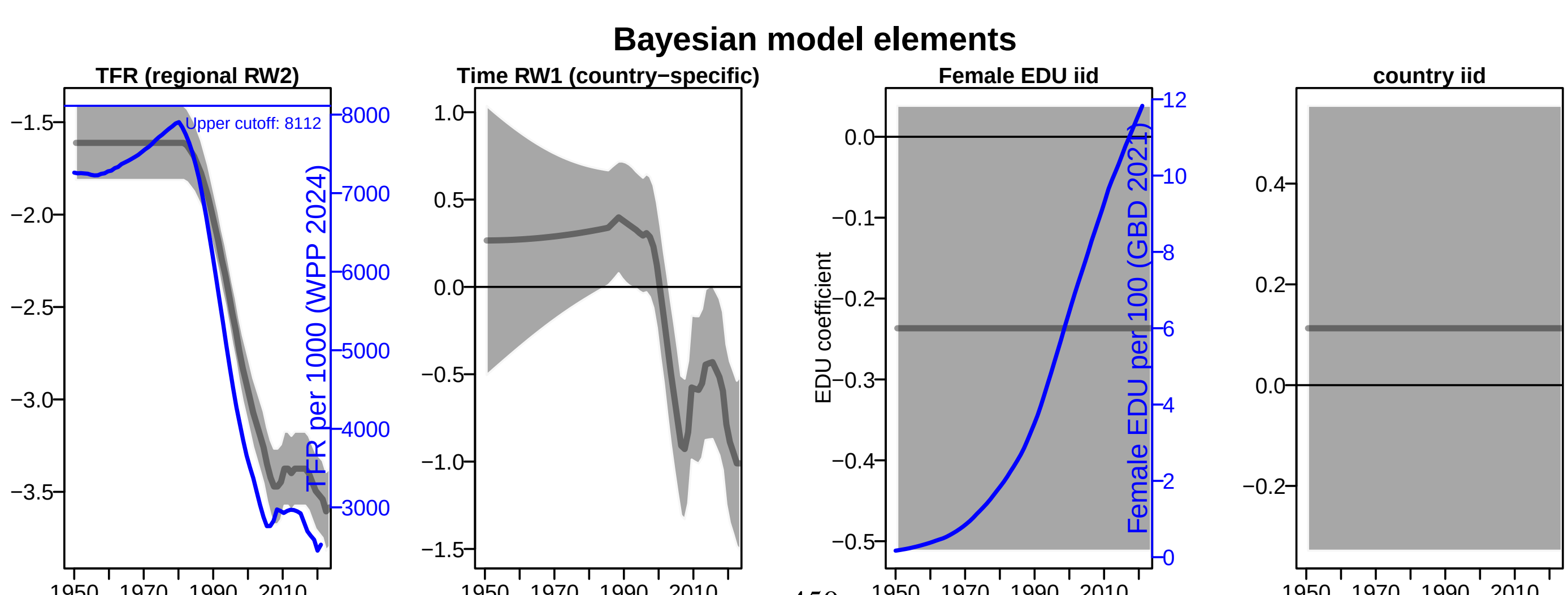
Bayesian model elements
TFR (regional RW2)
Upper cutoff: 8112
TFR per 1000 (WPP 2024)
Time RW1 (country−specific)
Female EDU iid
EDU coefficient
Female EDU per 100 (GBD 2021)
country iid

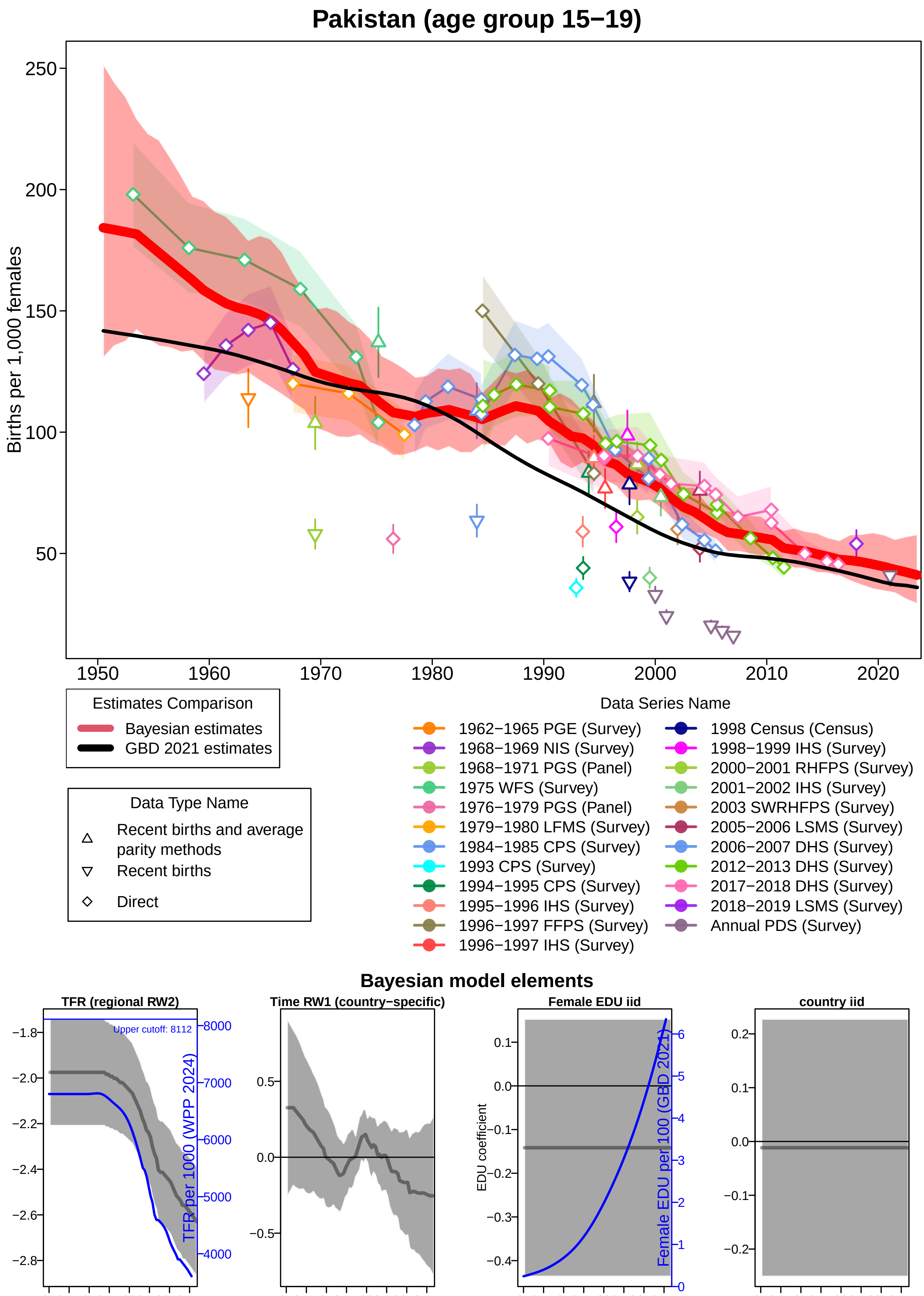
Pakistan (age group 15−19)
Births per 1,000 females
250
200
150
100
50
1950
1960
1970
1980
1990
2000
2010
2020
Estimates Comparison
Bayesian estimates
GBD 2021 estimates
Data Type Name
Recent births and average parity methods
Recent births
Direct
Data Series Name
1962−1965 PGE (Survey)
1968−1969 NIS (Survey)
1968−1971 PGS (Panel)
1975 WFS (Survey)
1976−1979 PGS (Panel)
1979−1980 LFMS (Survey)
1984−1985 CPS (Survey)
1993 CPS (Survey)
1994−1995 CPS (Survey)
1995−1996 IHS (Survey)
1996−1997 FFPS (Survey)
1996−1997 IHS (Survey)
1998 Census (Census)
1998−1999 IHS (Survey)
2000−2001 RHFPS (Survey)
2001−2002 IHS (Survey)
2003 SWRHFPS (Survey)
2005−2006 LSMS (Survey)
2006−2007 DHS (Survey)
2012−2013 DHS (Survey)
2017−2018 DHS (Survey)
2018−2019 LSMS (Survey)
Annual PDS (Survey)
Bayesian model elements
TFR (regional RW2)
Upper cutoff: 8112
TFR per 1000 (WPP 2024)
Time RW1 (country−specific)
Female EDU iid
EDU coefficient
Female EDU per 100 (GBD 2021)
country iid

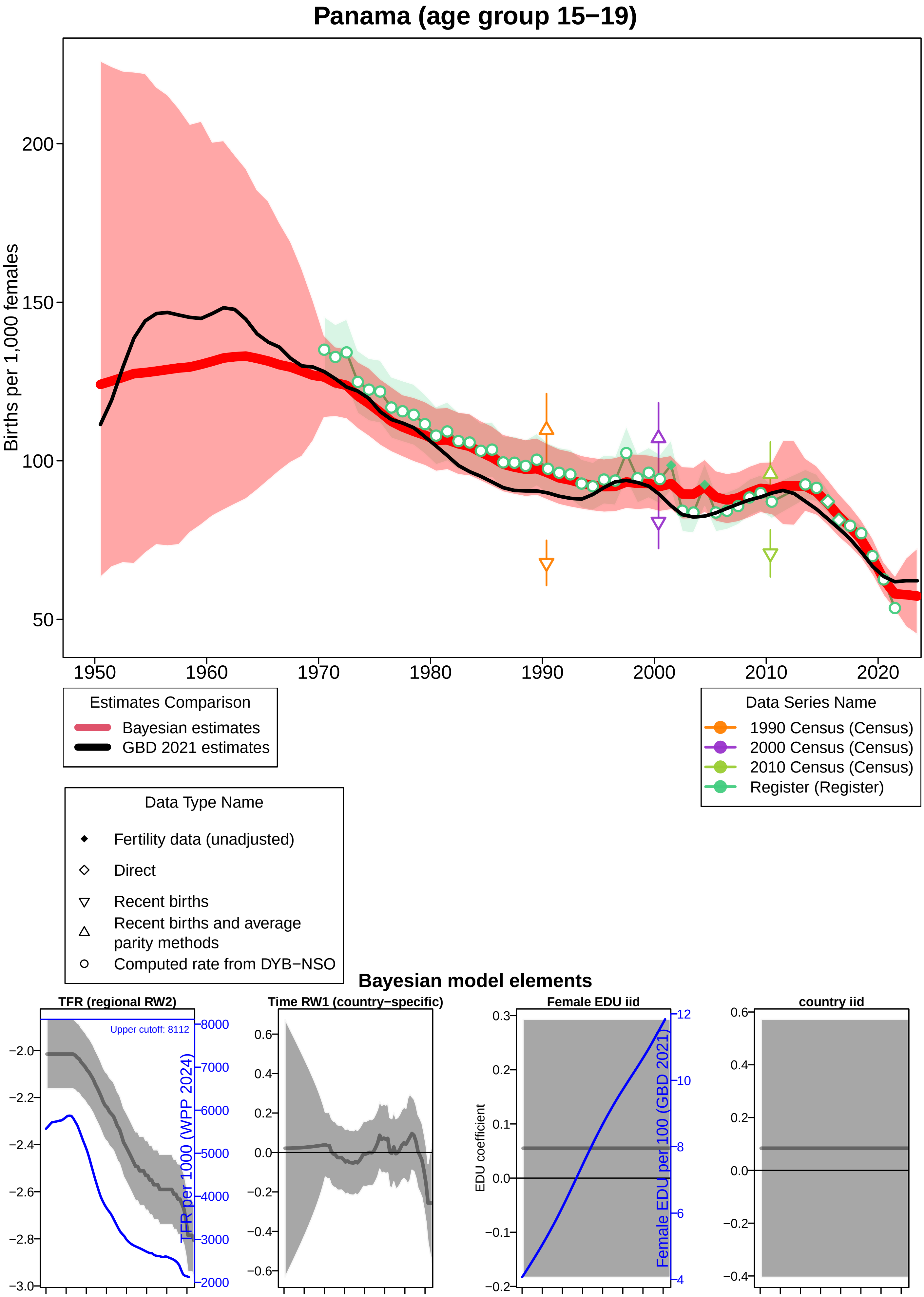
Panama (age group 15−19)
Births per 1,000 females
200
150
100
50
1950
1960
1970
1980
1990
2000
2010
2020
Estimates Comparison
Bayesian estimates
GBD 2021 estimates
Data Series Name
1990 Census (Census)
2000 Census (Census)
2010 Census (Census)
Register (Register)
Data Type Name
Fertility data (unadjusted)
Direct
Recent births
Recent births and average parity methods
Computed rate from DYB−NSO
Bayesian model elements
TFR (regional RW2)
Upper cutoff: 8112
TFR per 1000 (WPP 2024)
Time RW1 (country−specific)
Female EDU iid
EDU coefficient
Female EDU per 100 (GBD 2021)
country iid

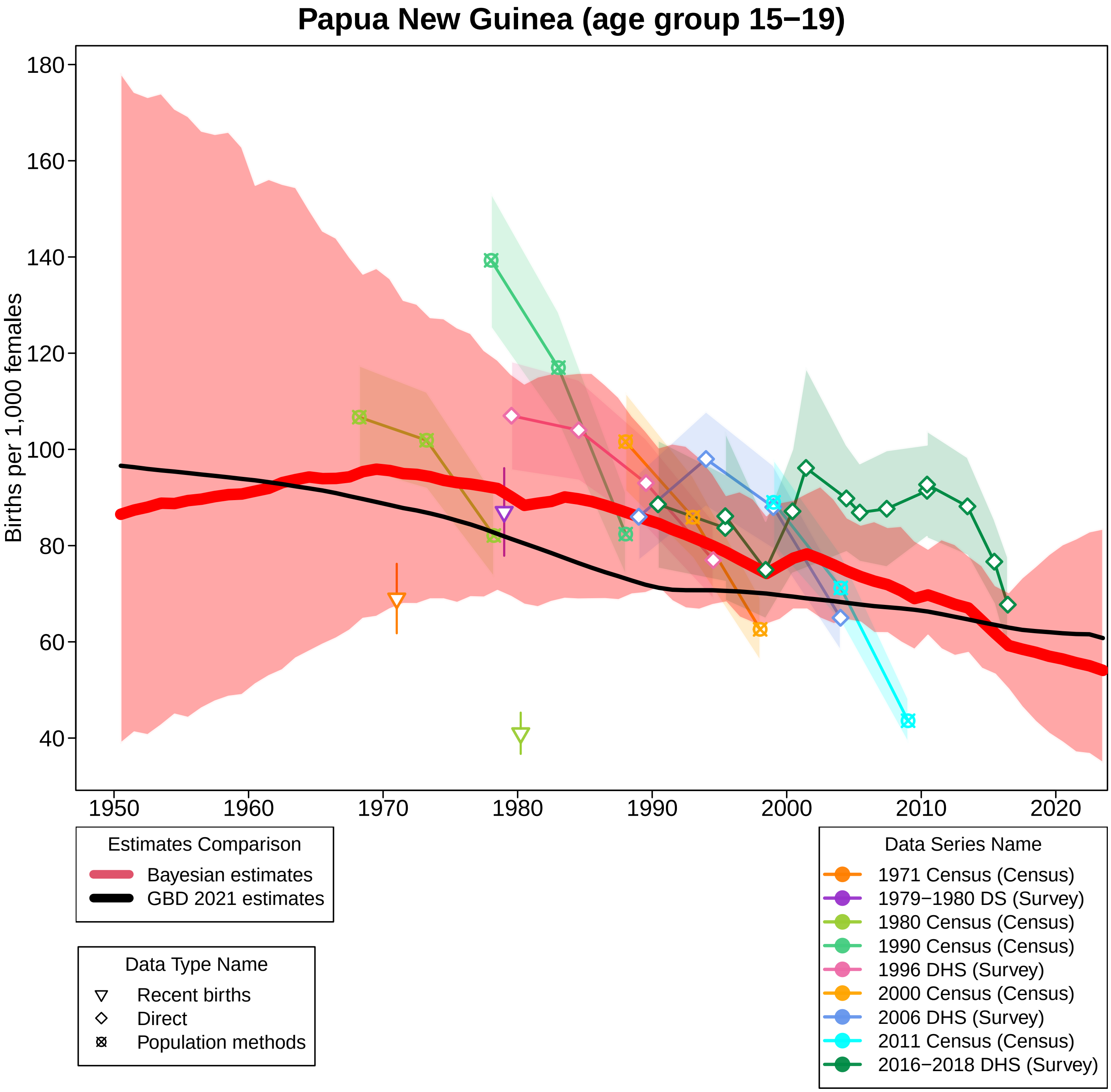
Papua New Guinea (age group 15−19)
Births per 1,000 females
180
160
140
120
100
80
60
40
1950
1960
1970
1980
1990
2000
2010
2020
Estimates Comparison
Bayesian estimates
GBD 2021 estimates
Data Type Name
Recent births
Direct
Population methods
Data Series Name
1971 Census (Census)
1979−1980 DS (Survey)
1980 Census (Census)
1990 Census (Census)
1996 DHS (Survey)
2000 Census (Census)
2006 DHS (Survey)
2011 Census (Census)
2016−2018 DHS (Survey)

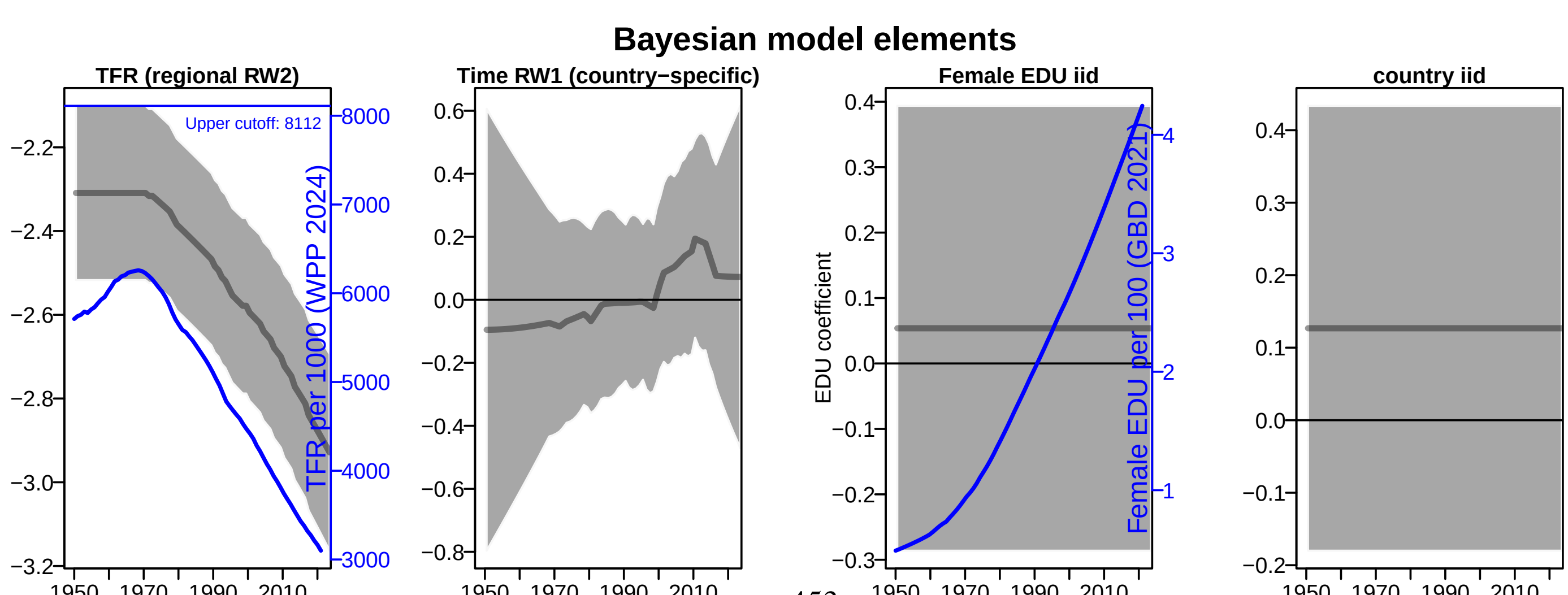
Bayesian model elements
TFR (regional RW2)
Upper cutoff: 8112
TFR per 1000 (WPP 2024)
−2.2
−2.4
−2.6
−2.8
−3.0
−3.2
8000
7000
6000
5000
4000
3000
Time RW1 (country−specific)
0.6
0.4
0.2
0.0
−0.2
−0.4
−0.6
−0.8
Female EDU iid
EDU coefficient
Female EDU per 100 (GBD 2021)
0.4
0.3
0.2
0.1
0.0
−0.1
−0.2
−0.3
4
3
2
1
country iid
0.4
0.3
0.2
0.1
0.0
−0.1
−0.2
1950
1970
1990
2010

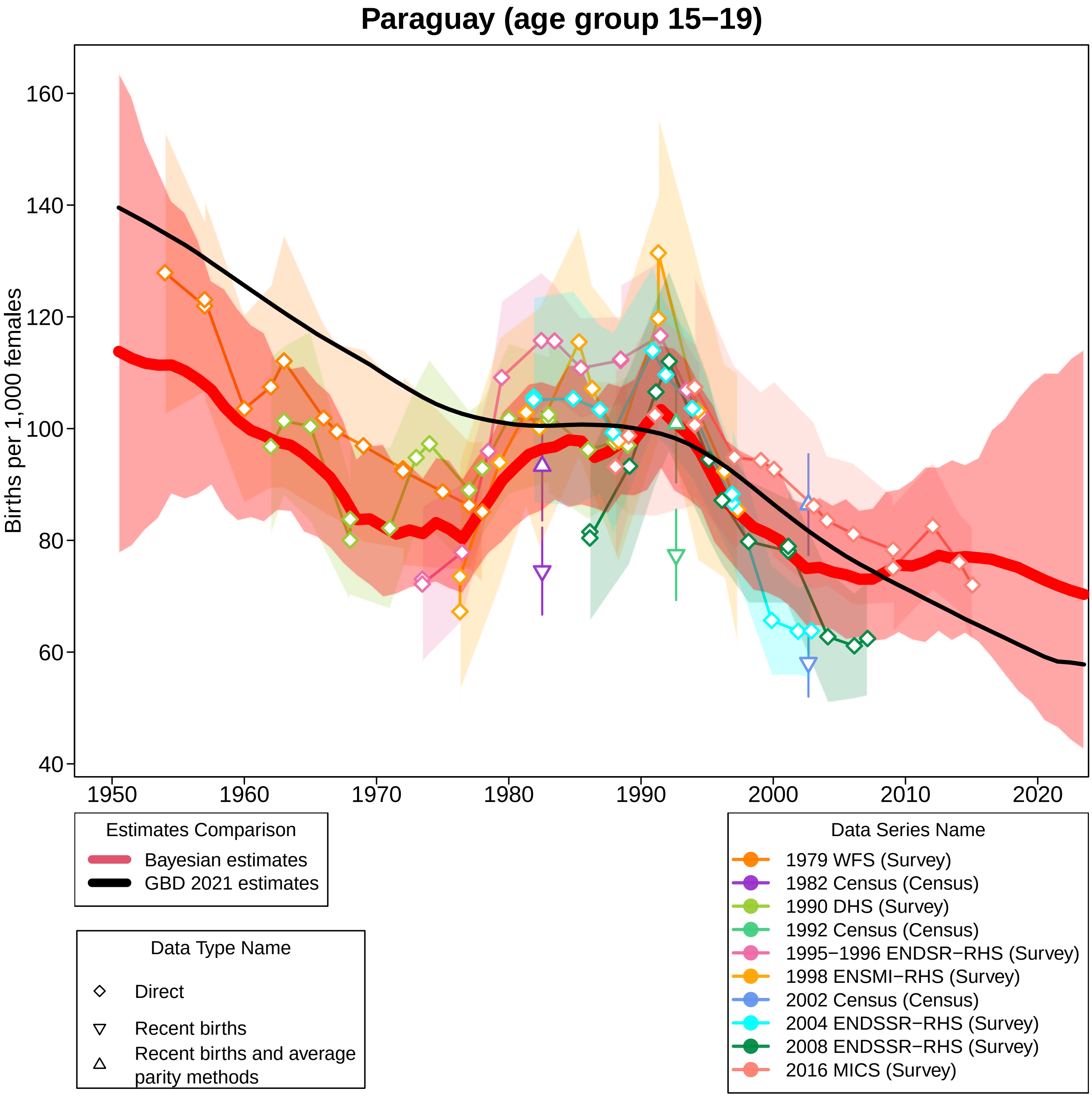
Paraguay (age group 15−19)
Births per 1,000 females
Estimates Comparison
Bayesian estimates
GBD 2021 estimates
Data Type Name
Direct
Recent births
Recent births and average parity methods
Data Series Name
1979 WFS (Survey)
1982 Census (Census)
1990 DHS (Survey)
1992 Census (Census)
1995−1996 ENDSR−RHS (Survey)
1998 ENSMI−RHS (Survey)
2002 Census (Census)
2004 ENDSSR−RHS (Survey)
2008 ENDSSR−RHS (Survey)
2016 MICS (Survey)

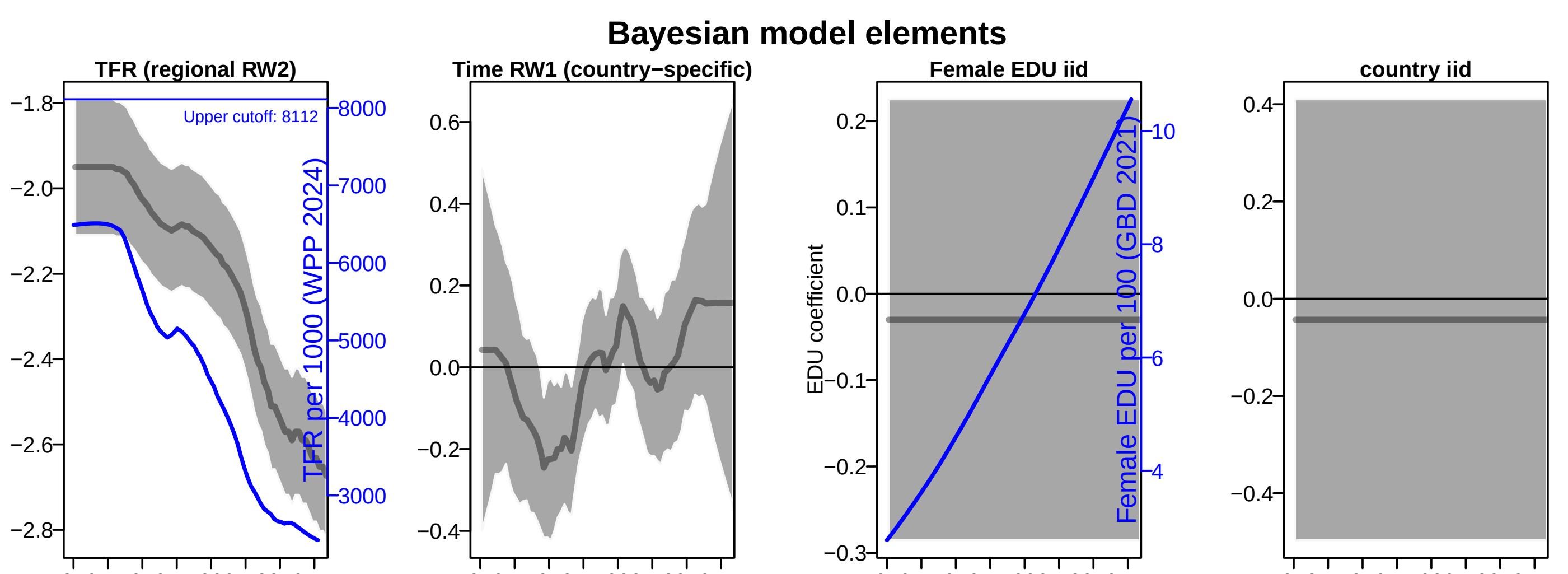
Bayesian model elements
TFR (regional RW2)
Upper cutoff: 8112
TFR per 1000 (WPP 2024)
Time RW1 (country−specific)
Female EDU iid
EDU coefficient
Female EDU per 100 (GBD 2021)
country iid

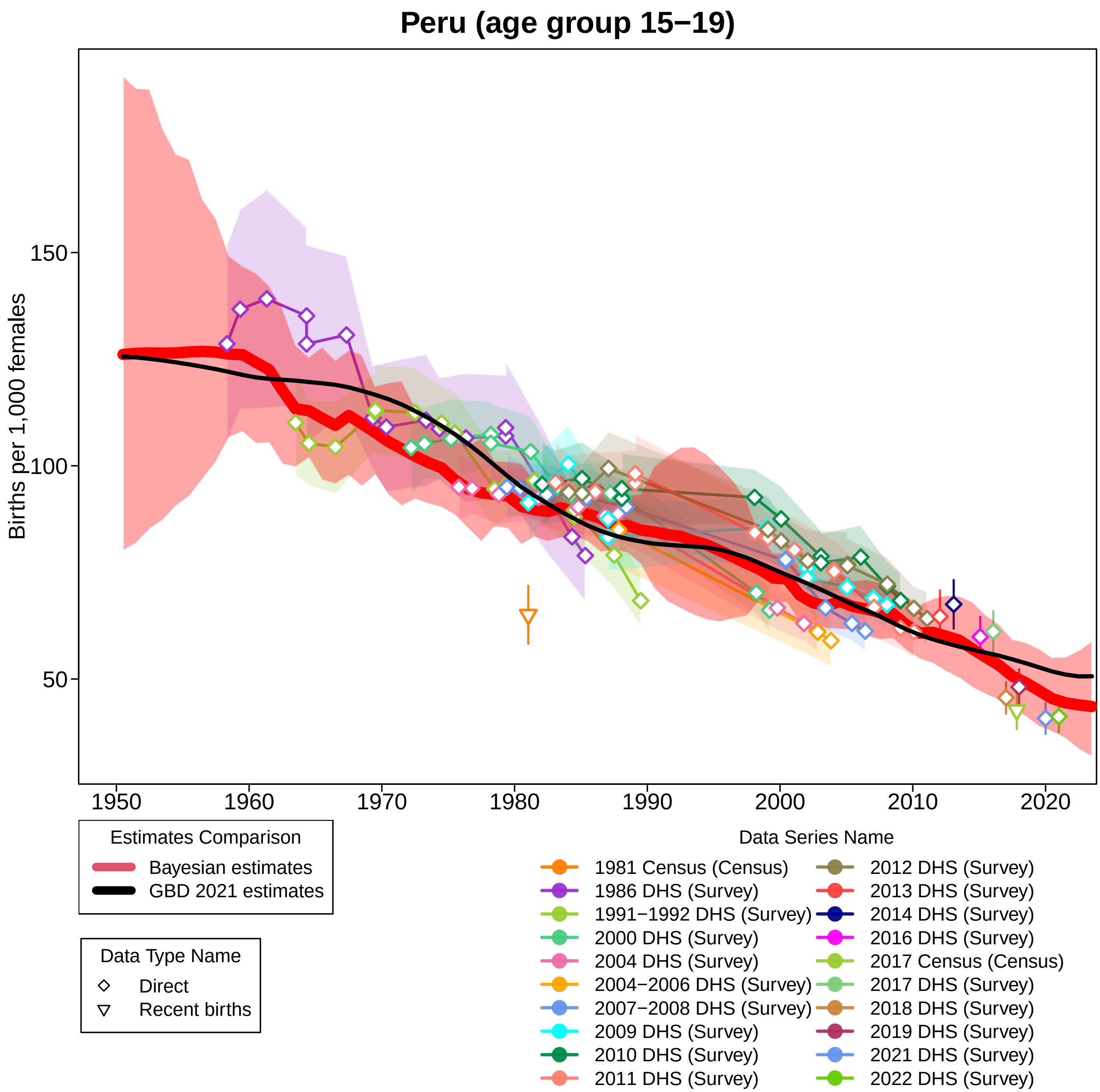
Peru (age group 15−19)
Births per 1,000 females
150
100
50
1950
1960
1970
1980
1990
2000
2010
2020
Estimates Comparison
Bayesian estimates
GBD 2021 estimates
Data Type Name
Direct
Recent births
Data Series Name
1981 Census (Census)
1986 DHS (Survey)
1991−1992 DHS (Survey)
2000 DHS (Survey)
2004 DHS (Survey)
2004−2006 DHS (Survey)
2007−2008 DHS (Survey)
2009 DHS (Survey)
2010 DHS (Survey)
2011 DHS (Survey)
2012 DHS (Survey)
2013 DHS (Survey)
2014 DHS (Survey)
2016 DHS (Survey)
2017 Census (Census)
2017 DHS (Survey)
2018 DHS (Survey)
2019 DHS (Survey)
2021 DHS (Survey)
2022 DHS (Survey)

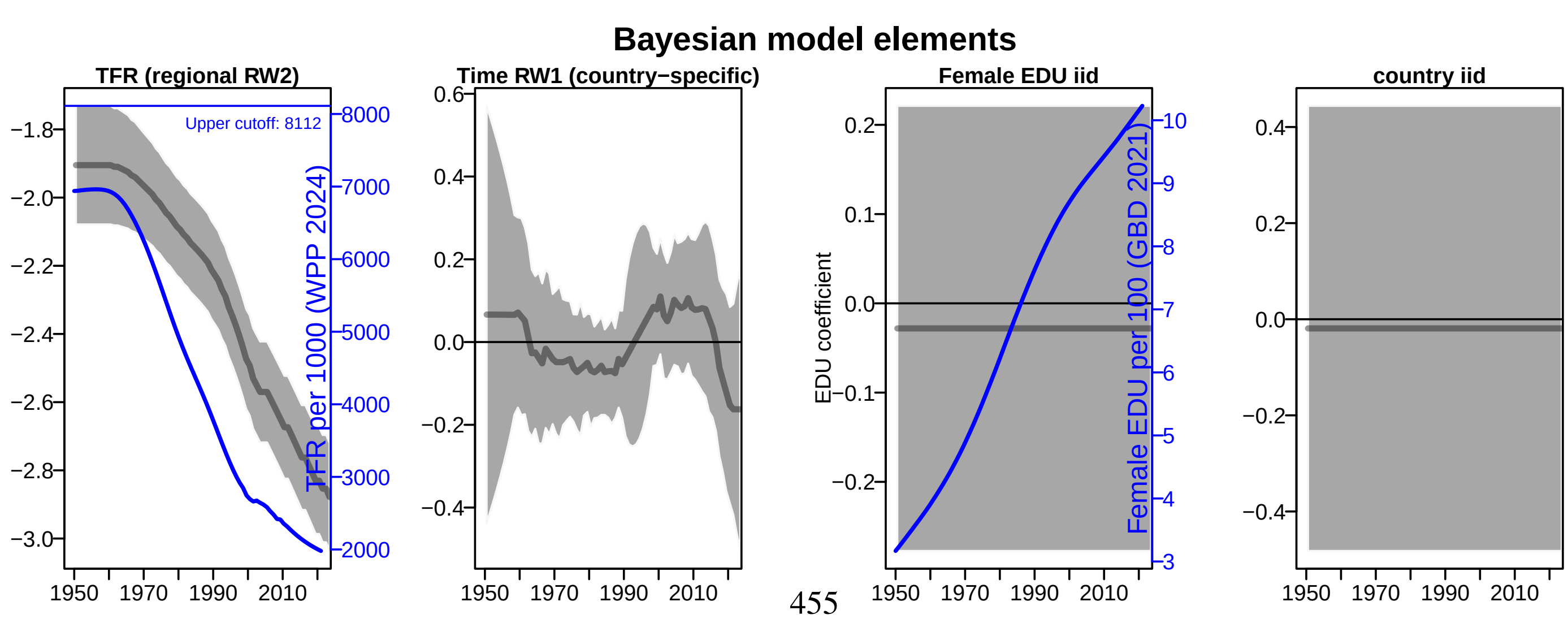
Bayesian model elements
TFR (regional RW2)
Upper cutoff: 8112
TFR per 1000 (WPP 2024)
Time RW1 (country−specific)
Female EDU iid
EDU coefficient
Female EDU per 100 (GBD 2021)
country iid

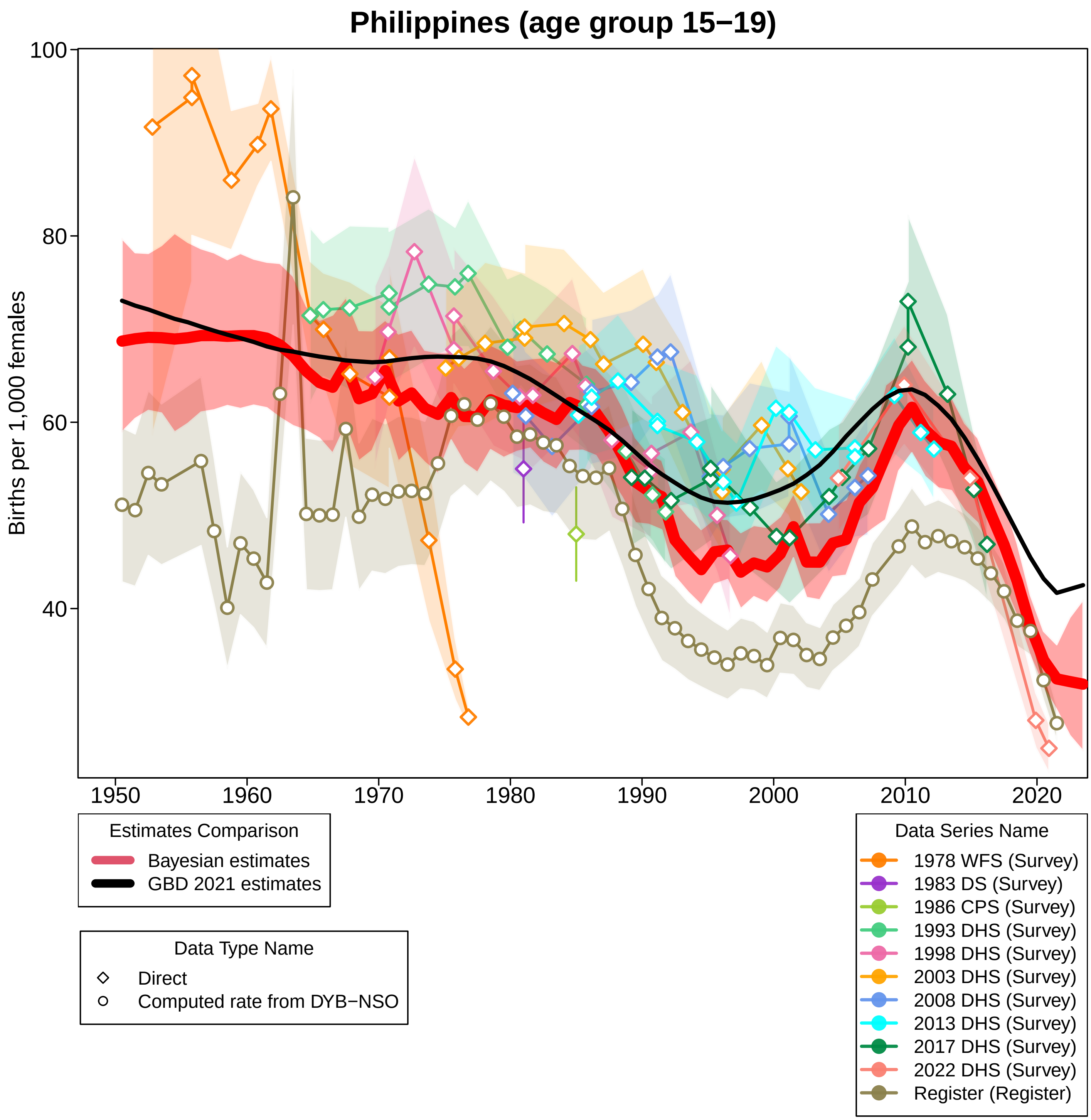
Philippines (age group 15−19)
Births per 1,000 females
100
80
60
40
1950
1960
1970
1980
1990
2000
2010
2020
Estimates Comparison
Bayesian estimates
GBD 2021 estimates
Data Type Name
Direct
Computed rate from DYB−NSO
Data Series Name
1978 WFS (Survey)
1983 DS (Survey)
1986 CPS (Survey)
1993 DHS (Survey)
1998 DHS (Survey)
2003 DHS (Survey)
2008 DHS (Survey)
2013 DHS (Survey)
2017 DHS (Survey)
2022 DHS (Survey)
Register (Register)

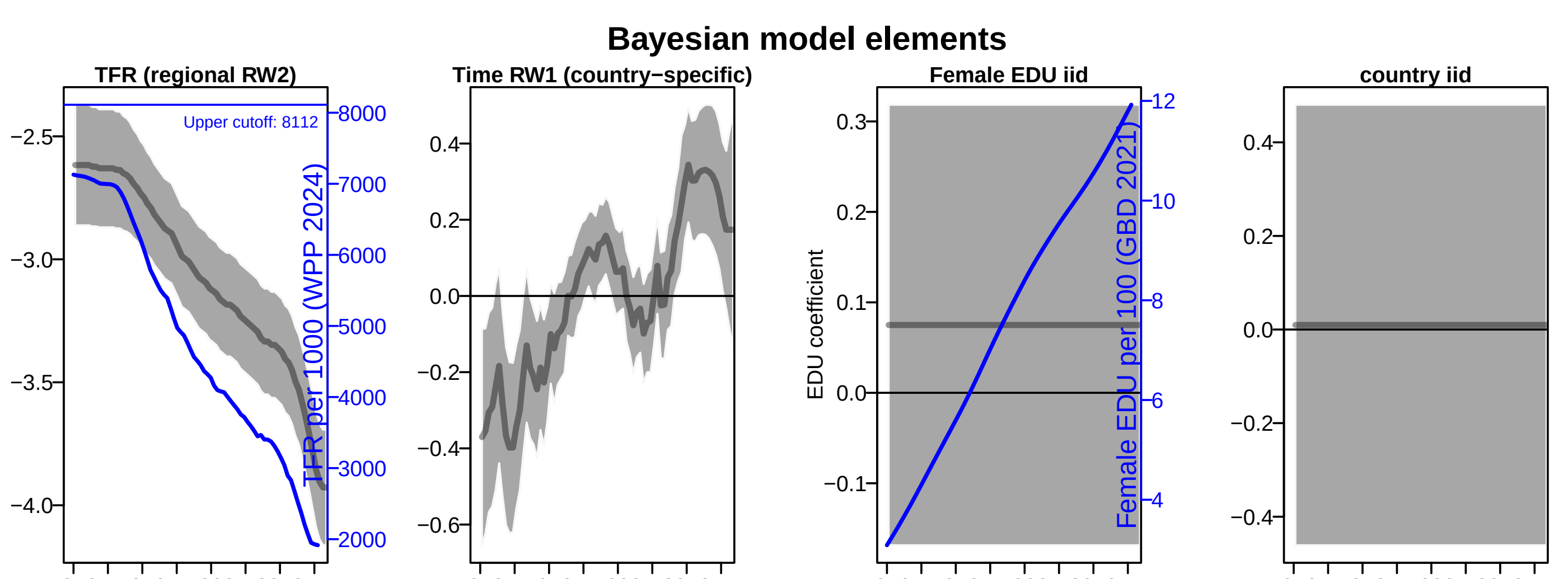
Bayesian model elements
TFR (regional RW2)
Upper cutoff: 8112
TFR per 1000 (WPP 2024)
Time RW1 (country−specific)
Female EDU iid
EDU coefficient
Female EDU per 100 (GBD 2021)
country iid
1950
1970
1990
2010

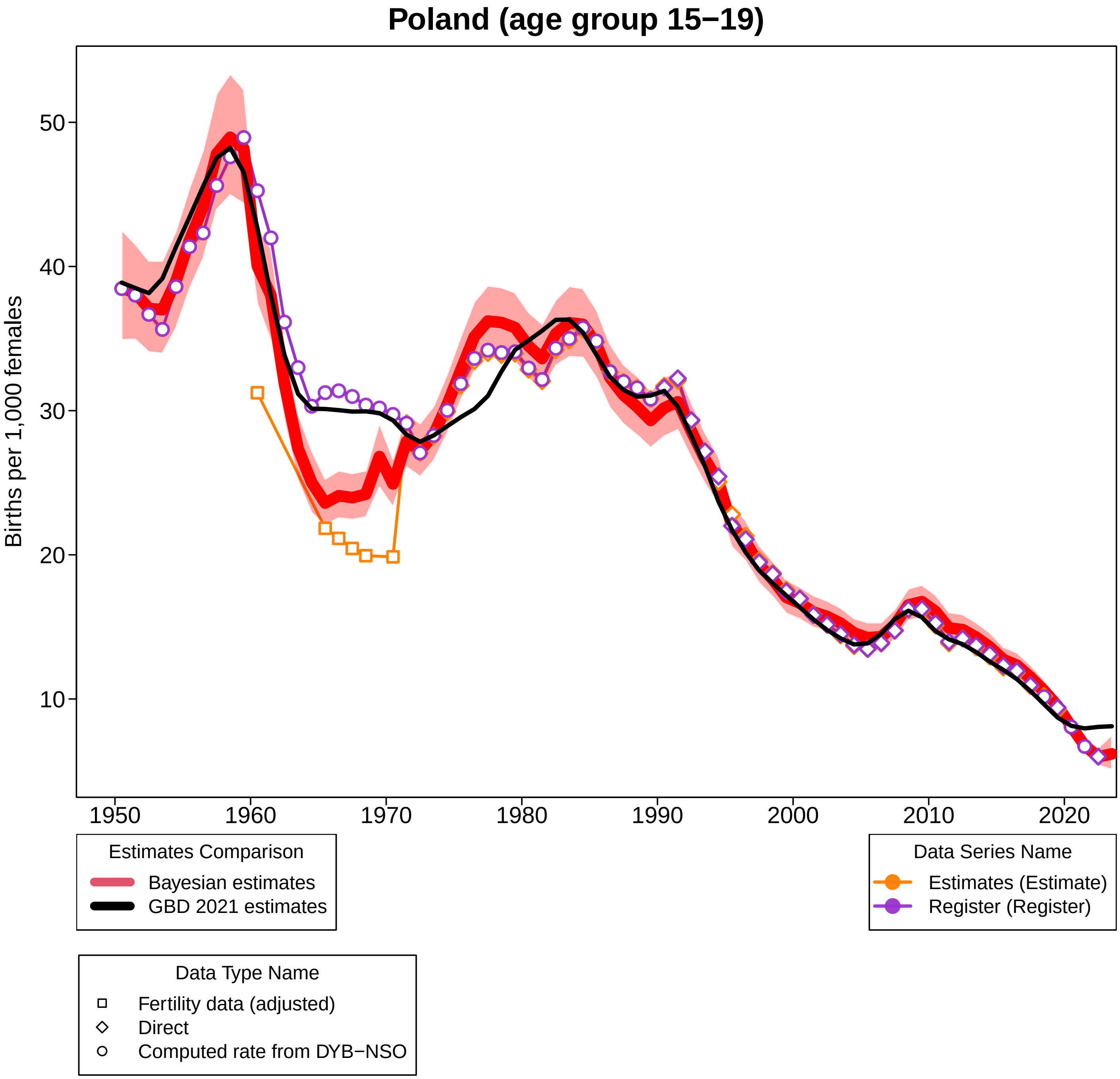
Poland (age group 15−19)
Births per 1,000 females
50
40
30
20
10
1950
1960
1970
1980
1990
2000
2010
2020
Estimates Comparison
Bayesian estimates
GBD 2021 estimates
Data Series Name
Estimates (Estimate)
Register (Register)
Data Type Name
Fertility data (adjusted)
Direct
Computed rate from DYB−NSO

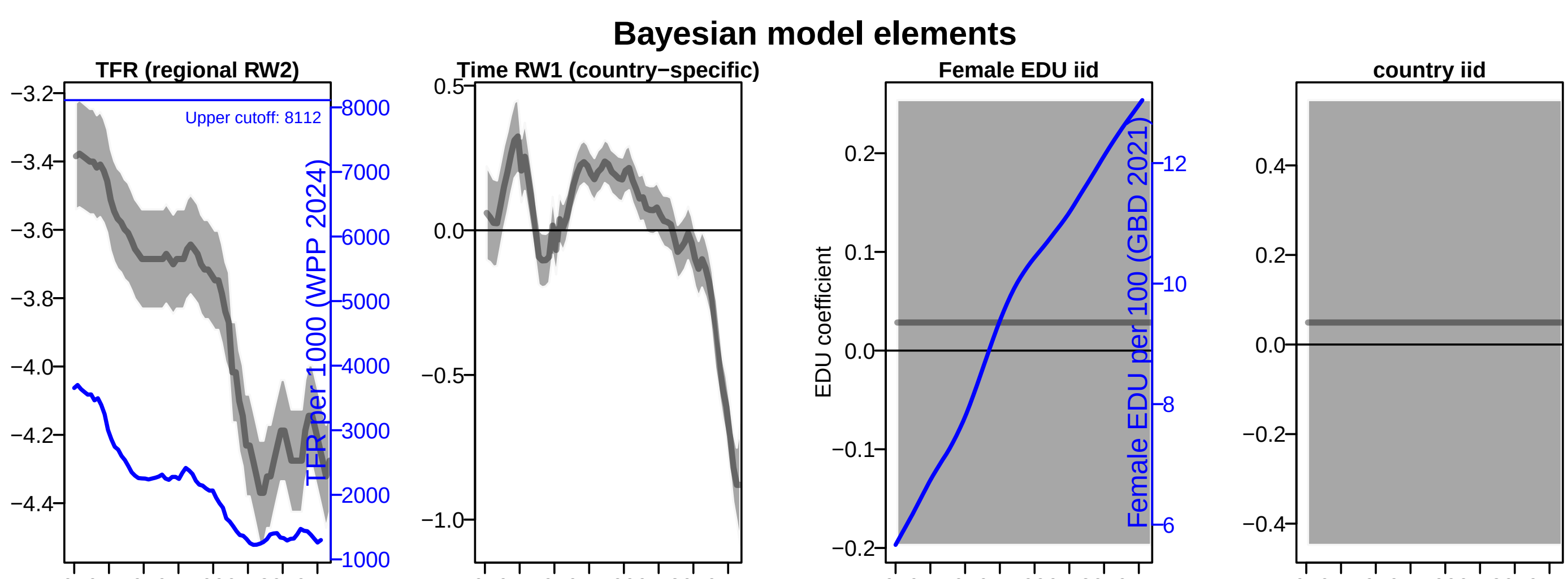
Bayesian model elements
TFR (regional RW2)
Upper cutoff: 8112
TFR per 1000 (WPP 2024)
Time RW1 (country−specific)
Female EDU iid
EDU coefficient
Female EDU per 100 (GBD 2021)
country iid

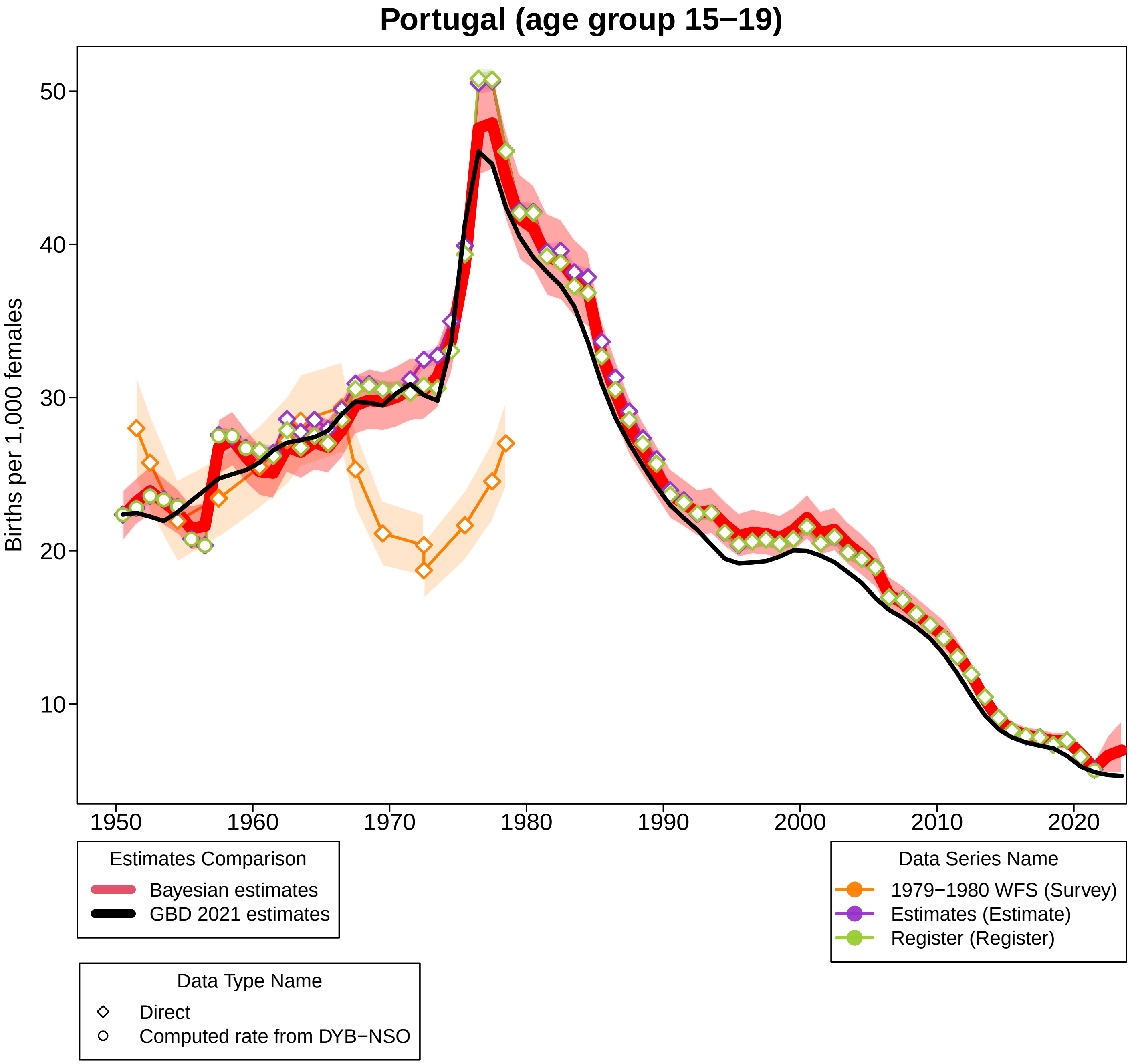
Portugal (age group 15−19)
Births per 1,000 females
50
40
30
20
10
1950
1960
1970
1980
1990
2000
2010
2020
Estimates Comparison
Bayesian estimates
GBD 2021 estimates
Data Series Name
1979−1980 WFS (Survey)
Estimates (Estimate)
Register (Register)
Data Type Name
Direct
Computed rate from DYB−NSO

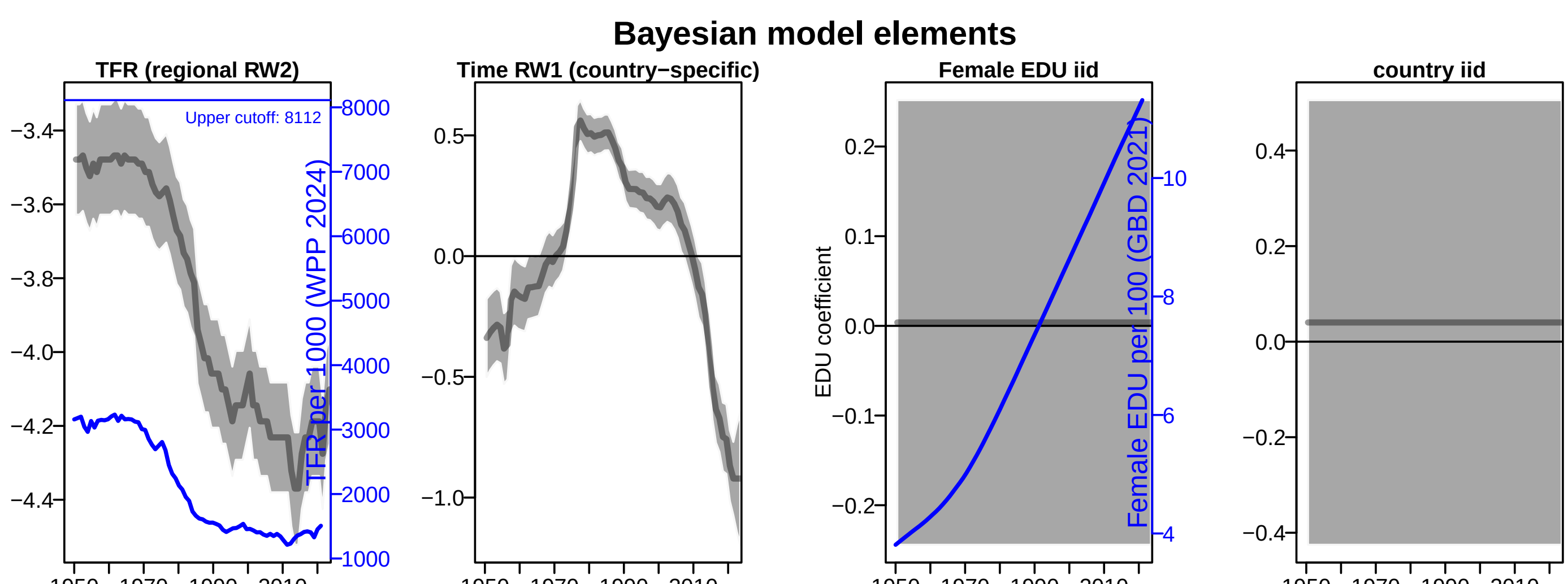
Bayesian model elements
TFR (regional RW2)
Upper cutoff: 8112
TFR per 1000 (WPP 2024)
Time RW1 (country−specific)
Female EDU iid
EDU coefficient
Female EDU per 100 (GBD 2021)
country iid

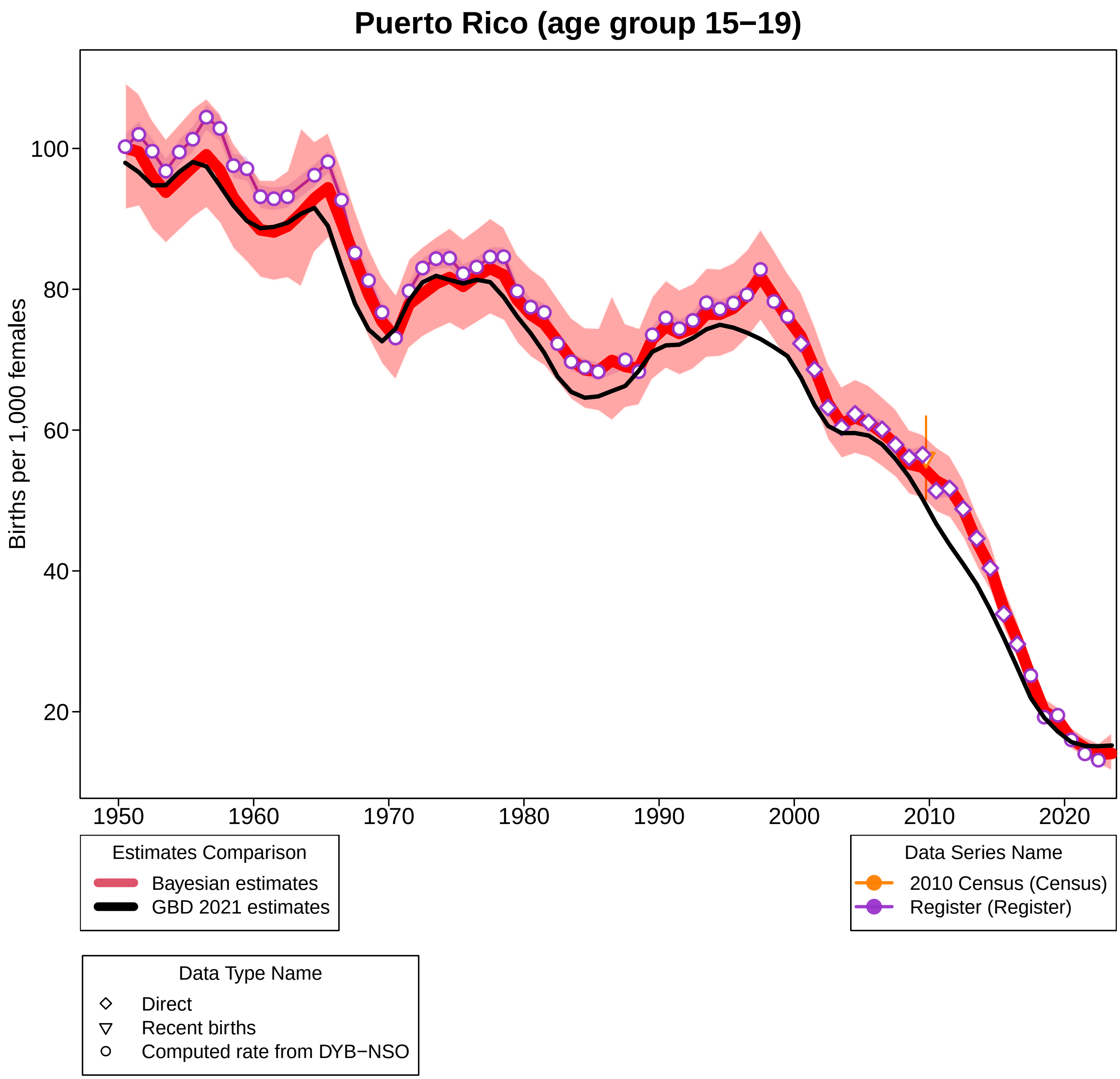

Puerto Rico (age group 15−19)
Births per 1,000 females
100
80
60
40
20
1950
1960
1970
1980
1990
2000
2010
2020
Estimates Comparison
Bayesian estimates
GBD 2021 estimates
Data Series Name
2010 Census (Census)
Register (Register)
Data Type Name
Direct
Recent births
Computed rate from DYB−NSO

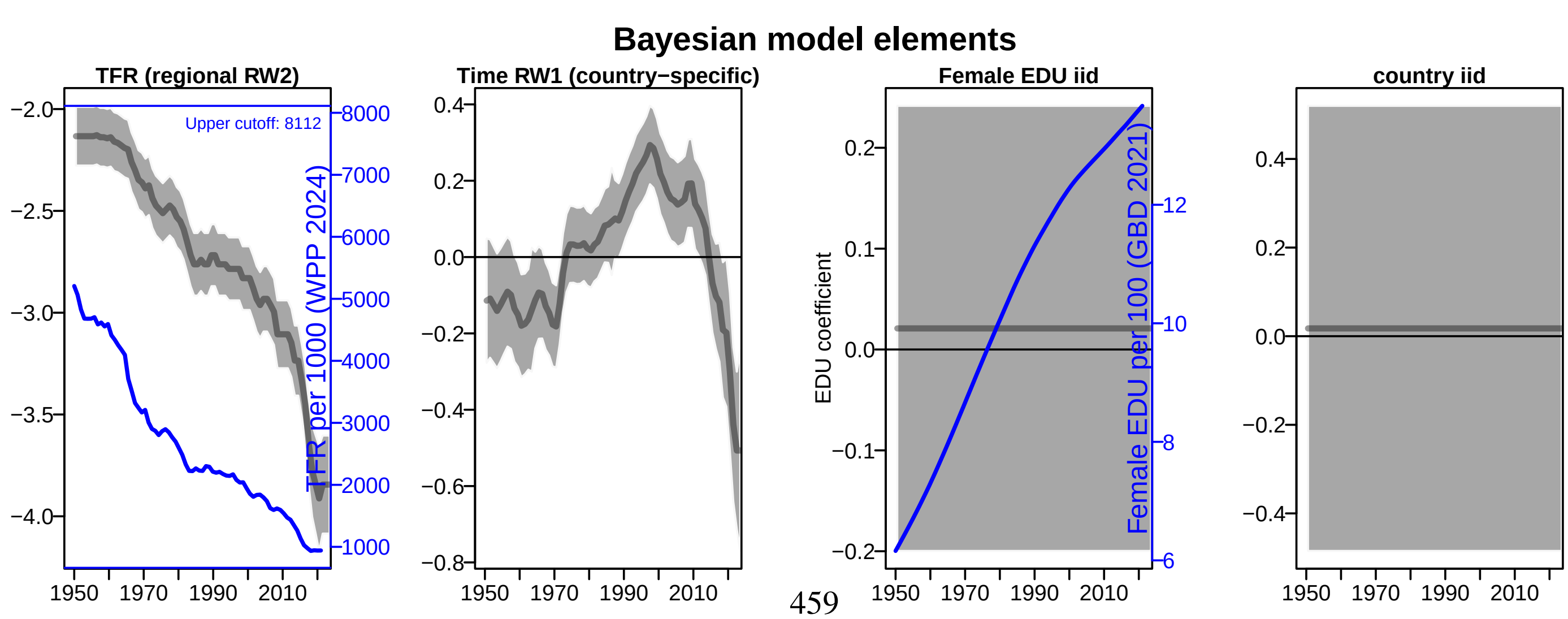

Bayesian model elements
TFR (regional RW2)
Upper cutoff: 8112
TFR per 1000 (WPP 2024)
Time RW1 (country−specific)
Female EDU iid
EDU coefficient
Female EDU per 100 (GBD 2021)
country iid

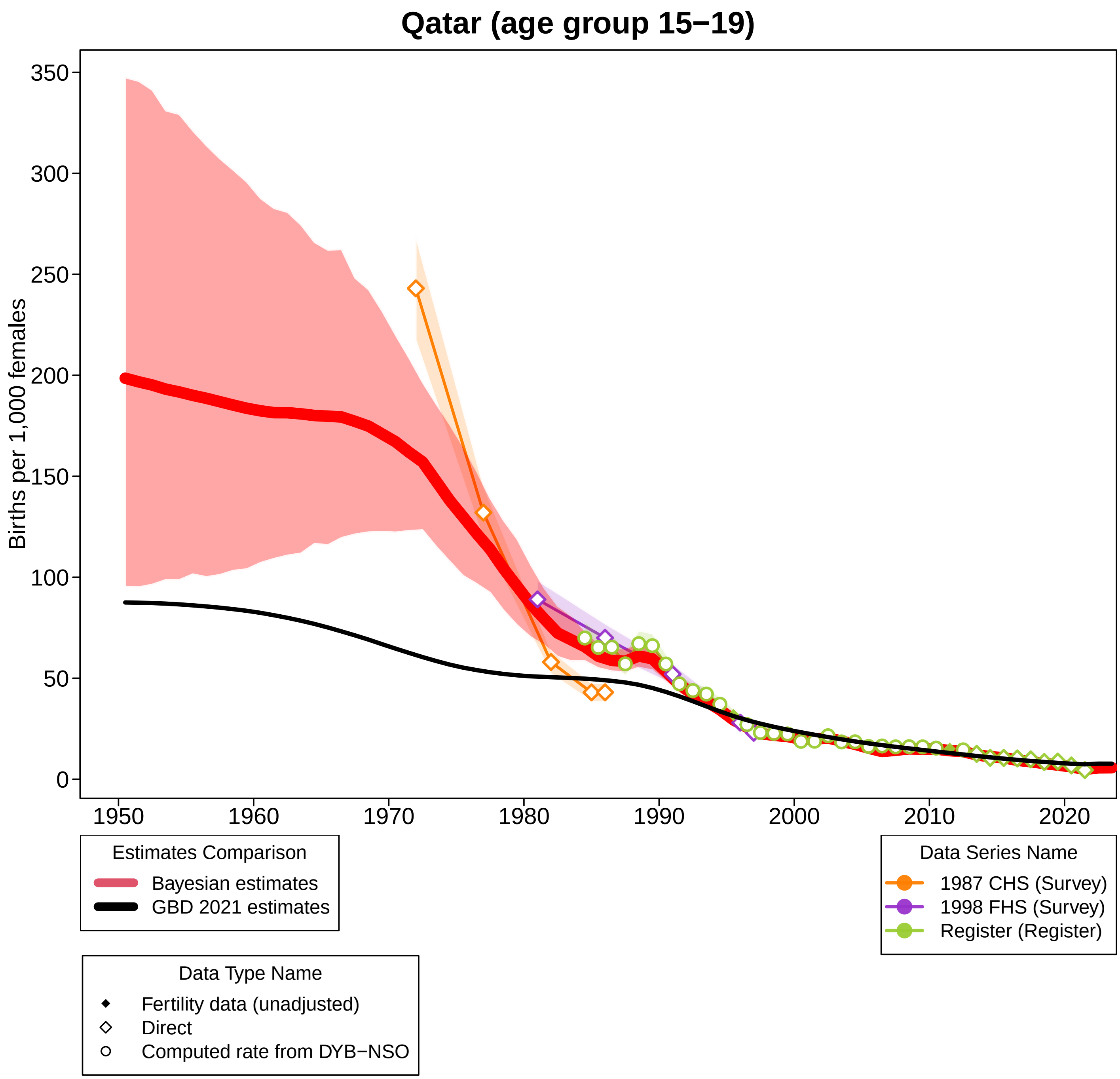

Qatar (age group 15−19)
Births per 1,000 females
350
300
250
200
150
100
50
0
1950
1960
1970
1980
1990
2000
2010
2020
Estimates Comparison
Bayesian estimates
GBD 2021 estimates
Data Series Name
1987 CHS (Survey)
1998 FHS (Survey)
Register (Register)
Data Type Name
Fertility data (unadjusted)
Direct
Computed rate from DYB−NSO

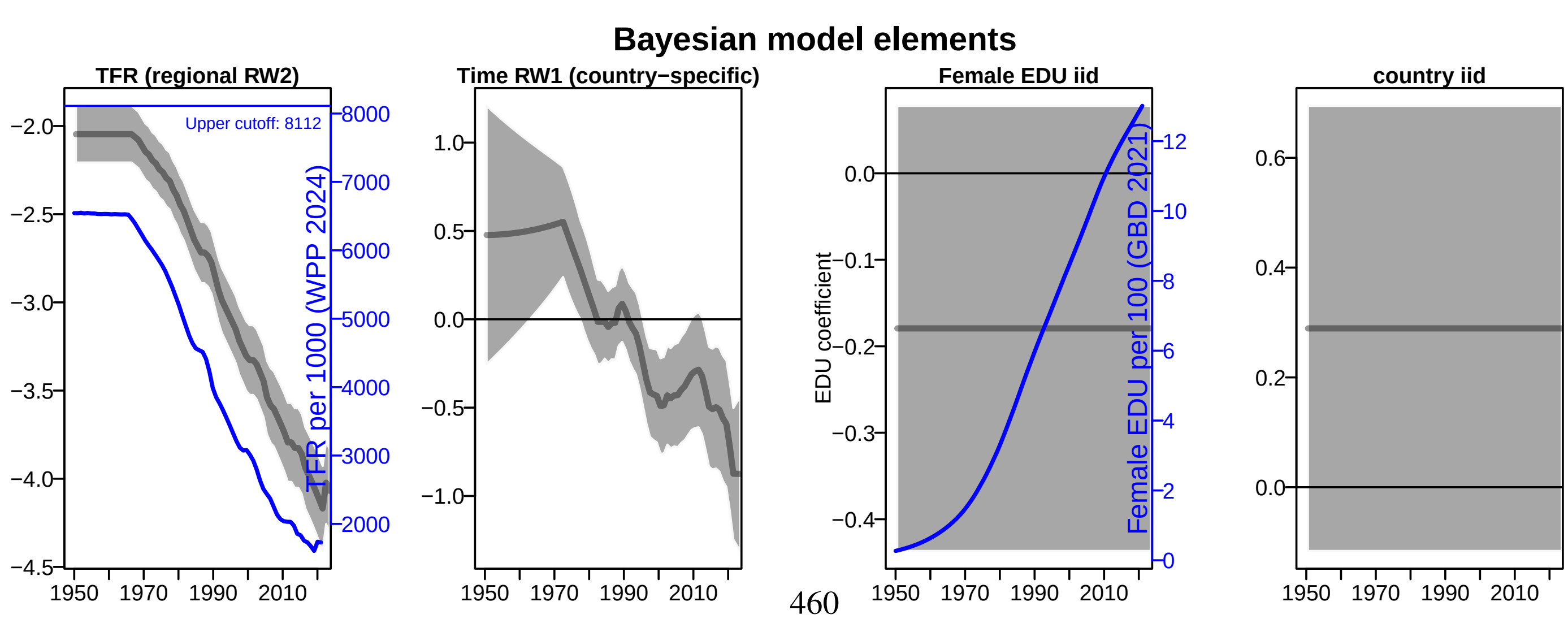

Bayesian model elements
TFR (regional RW2)
Upper cutoff: 8112
TFR per 1000 (WPP 2024)
Time RW1 (country−specific)
Female EDU iid
EDU coefficient
Female EDU per 100 (GBD 2021)
country iid

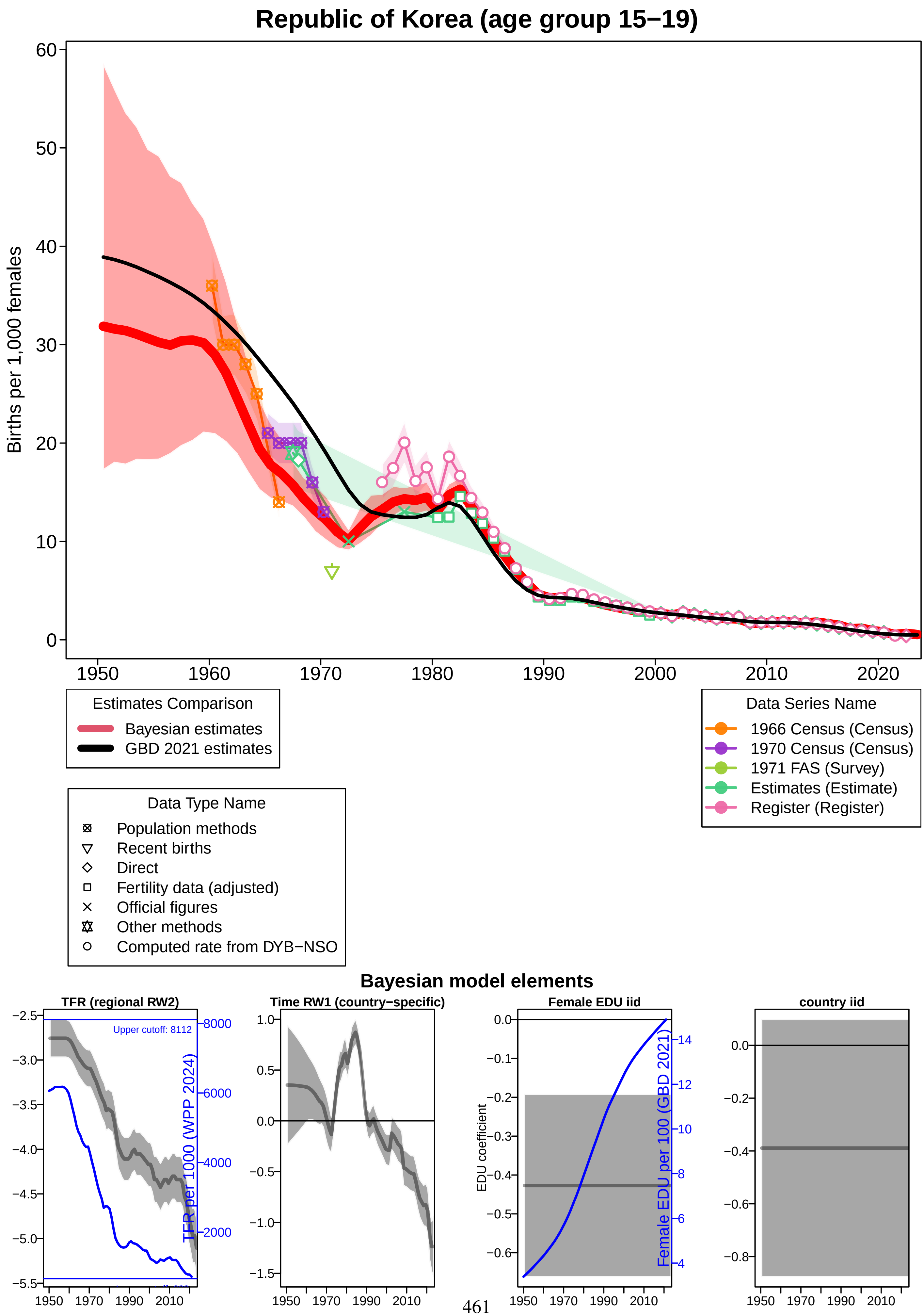
Republic of Korea (age group 15−19)
Births per 1,000 females
60
50
40
30
20
10
0
1950
1960
1970
1980
1990
2000
2010
2020
Estimates Comparison
Bayesian estimates
GBD 2021 estimates
Data Series Name
1966 Census (Census)
1970 Census (Census)
1971 FAS (Survey)
Estimates (Estimate)
Register (Register)
Data Type Name
Population methods
Recent births
Direct
Fertility data (adjusted)
Official figures
Other methods
Computed rate from DYB−NSO
Bayesian model elements
TFR (regional RW2)
Upper cutoff: 8112
TFR per 1000 (WPP 2024)
Time RW1 (country−specific)
Female EDU iid
EDU coefficient
Female EDU per 100 (GBD 2021)
country iid

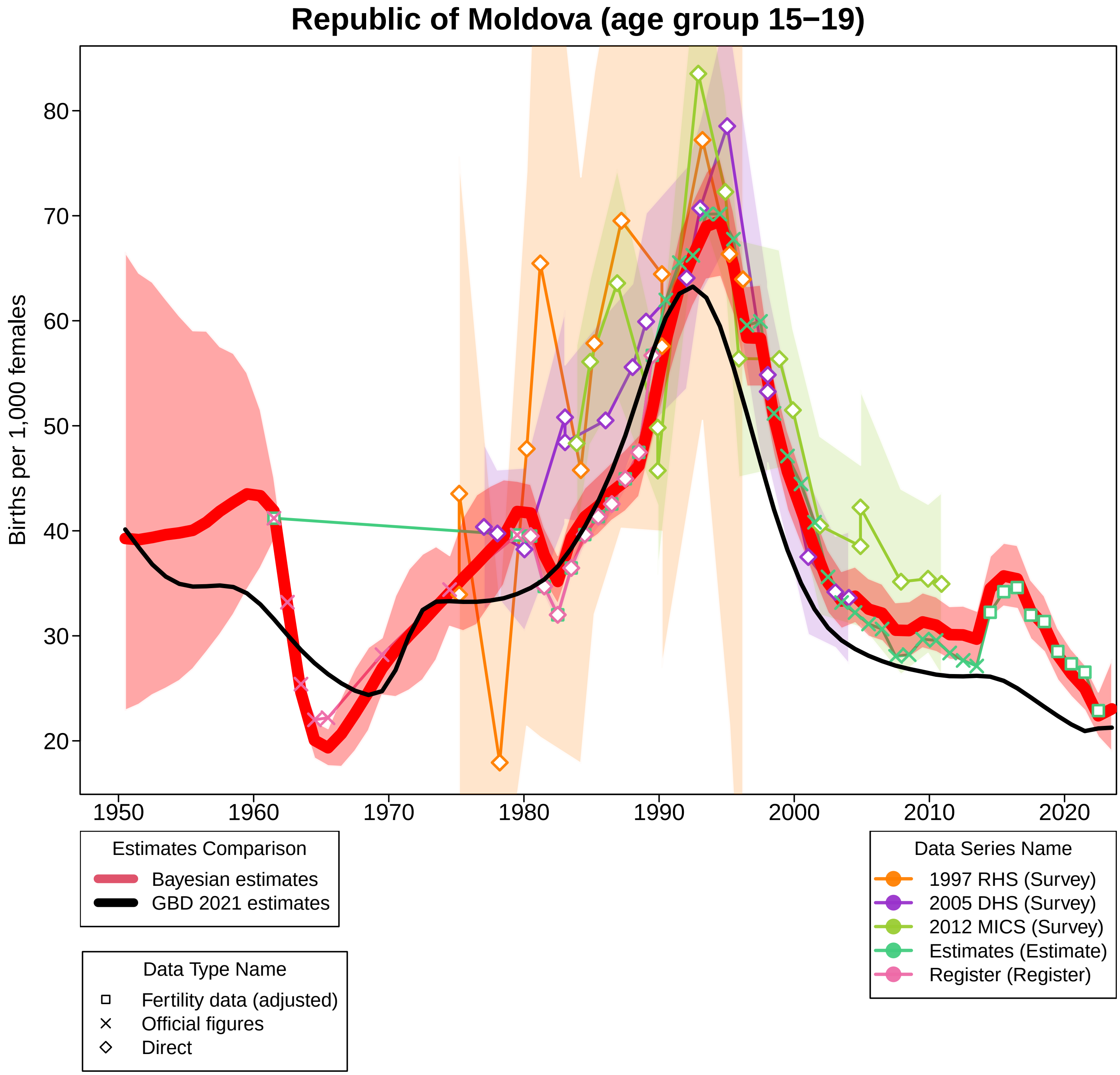
Republic of Moldova (age group 15−19)
Births per 1,000 females
80
70
60
50
40
30
20
1950
1960
1970
1980
1990
2000
2010
2020
Estimates Comparison
Bayesian estimates
GBD 2021 estimates
Data Series Name
1997 RHS (Survey)
2005 DHS (Survey)
2012 MICS (Survey)
Estimates (Estimate)
Register (Register)
Data Type Name
Fertility data (adjusted)
Official figures
Direct

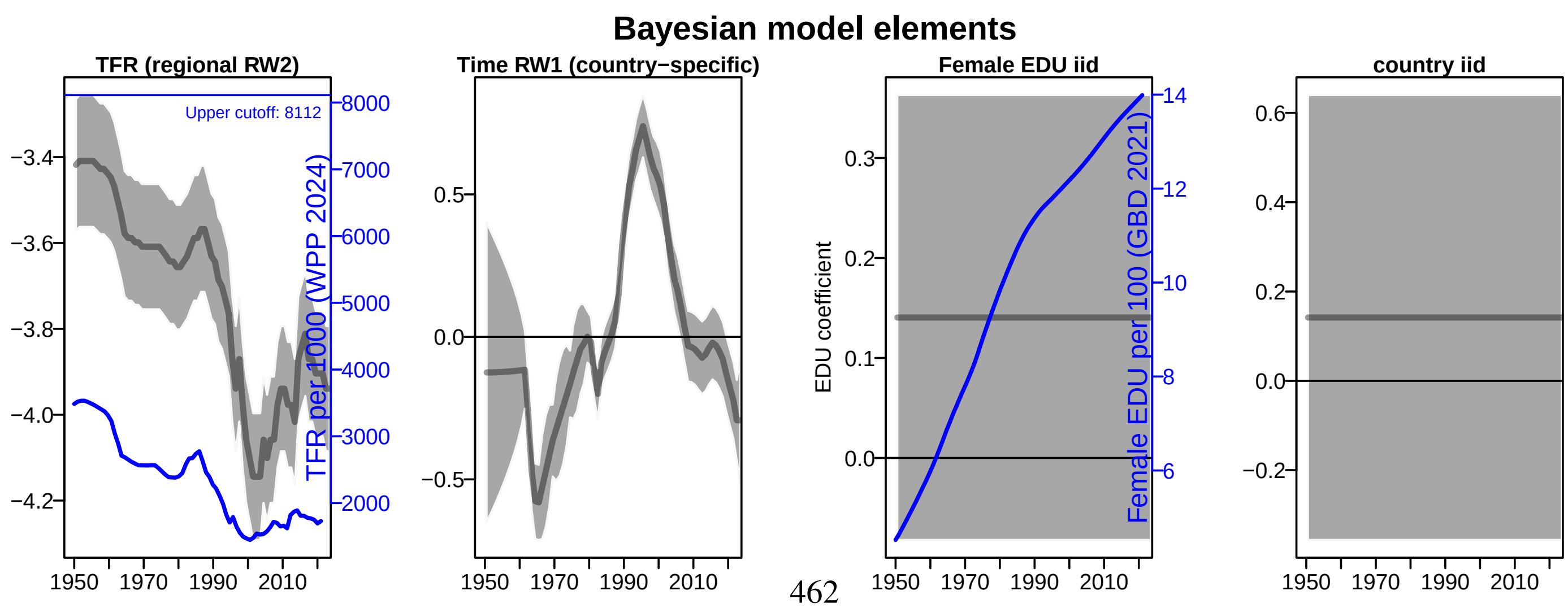
Bayesian model elements
TFR (regional RW2)
Upper cutoff: 8112
TFR per 1000 (WPP 2024)
Time RW1 (country−specific)
Female EDU iid
EDU coefficient
Female EDU per 100 (GBD 2021)
country iid
1950
1970
1990
2010

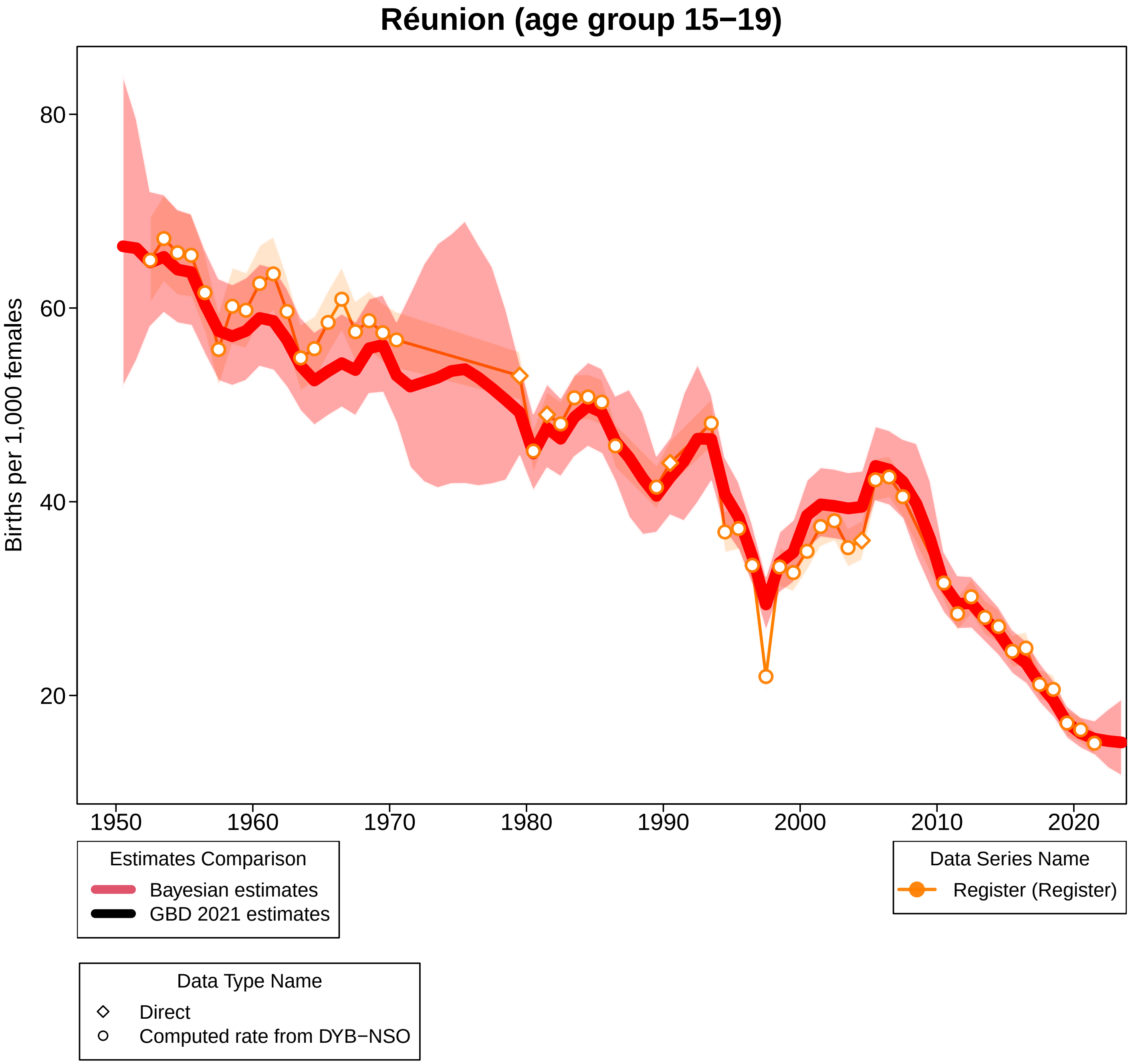

Réunion (age group 15−19)
Births per 1,000 females
80
60
40
20
1950
1960
1970
1980
1990
2000
2010
2020
Estimates Comparison
Bayesian estimates
GBD 2021 estimates
Data Series Name
Register (Register)
Data Type Name
Direct
Computed rate from DYB−NSO

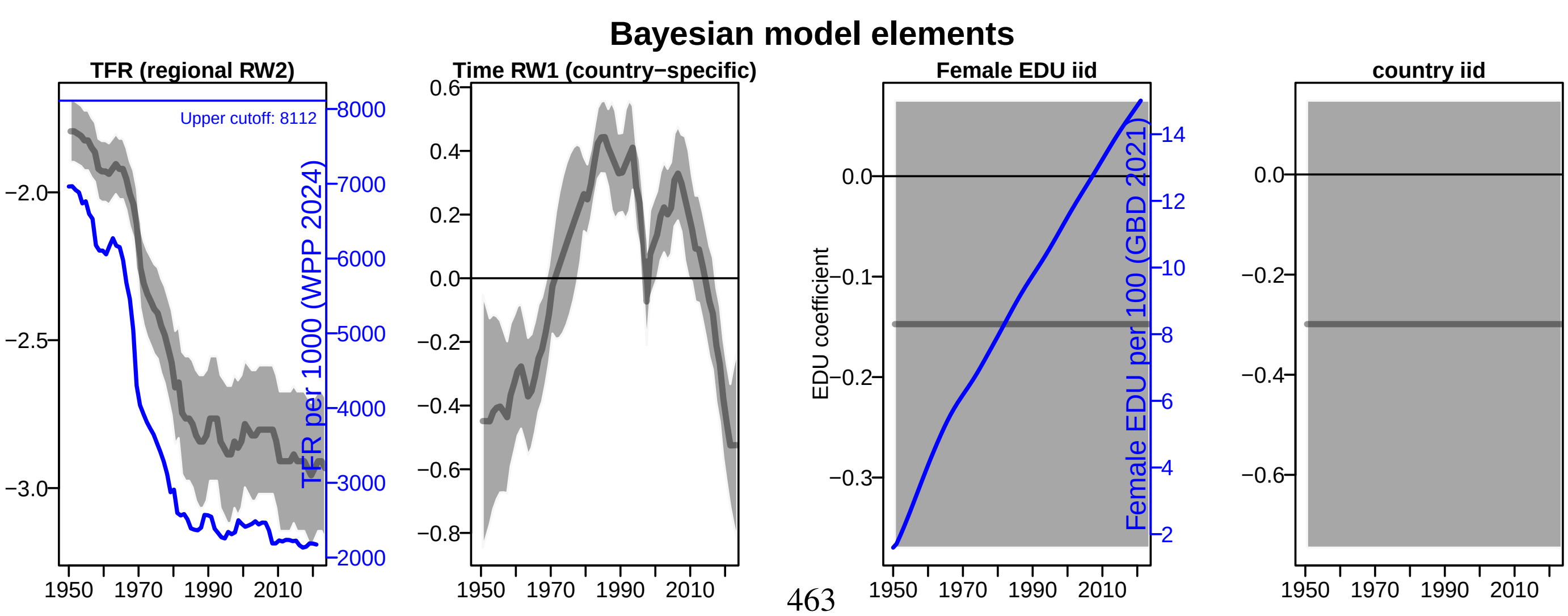

Bayesian model elements
TFR (regional RW2)
Upper cutoff: 8112
TFR per 1000 (WPP 2024)
Time RW1 (country−specific)
Female EDU iid
EDU coefficient
Female EDU per 100 (GBD 2021)
country iid

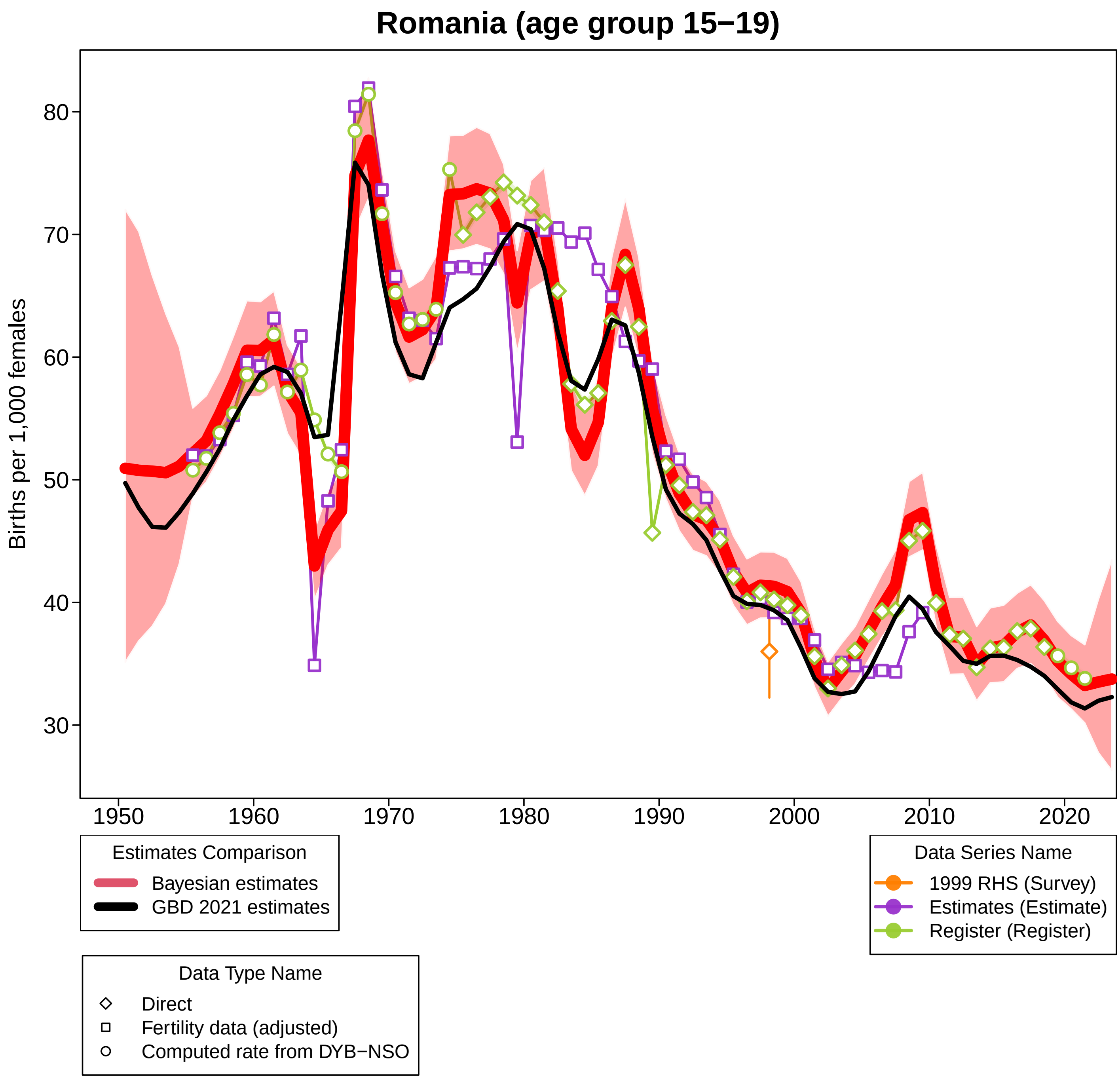
Romania (age group 15−19)
Births per 1,000 females
80
70
60
50
40
30
1950
1960
1970
1980
1990
2000
2010
2020
Estimates Comparison
Bayesian estimates
GBD 2021 estimates
Data Series Name
1999 RHS (Survey)
Estimates (Estimate)
Register (Register)
Data Type Name
Direct
Fertility data (adjusted)
Computed rate from DYB−NSO

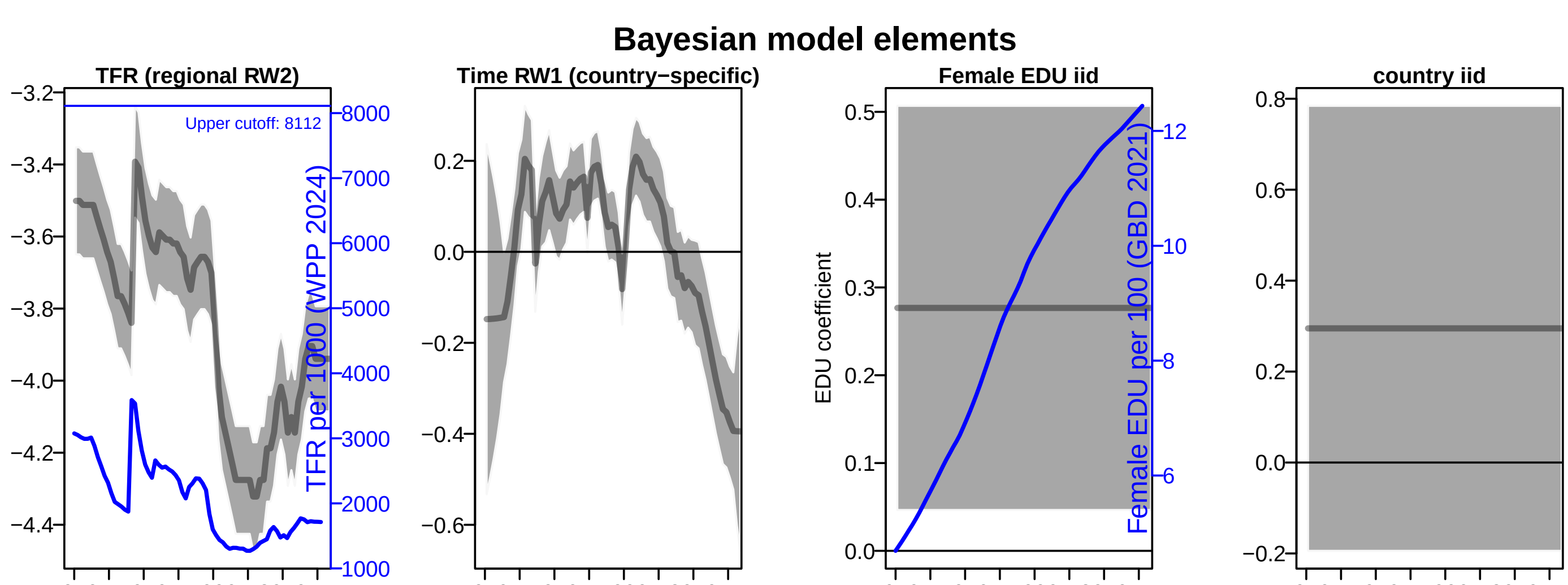
Bayesian model elements
TFR (regional RW2)
Upper cutoff: 8112
TFR per 1000 (WPP 2024)
Time RW1 (country−specific)
Female EDU iid
EDU coefficient
Female EDU per 100 (GBD 2021)
country iid
1950
1970
1990
2010

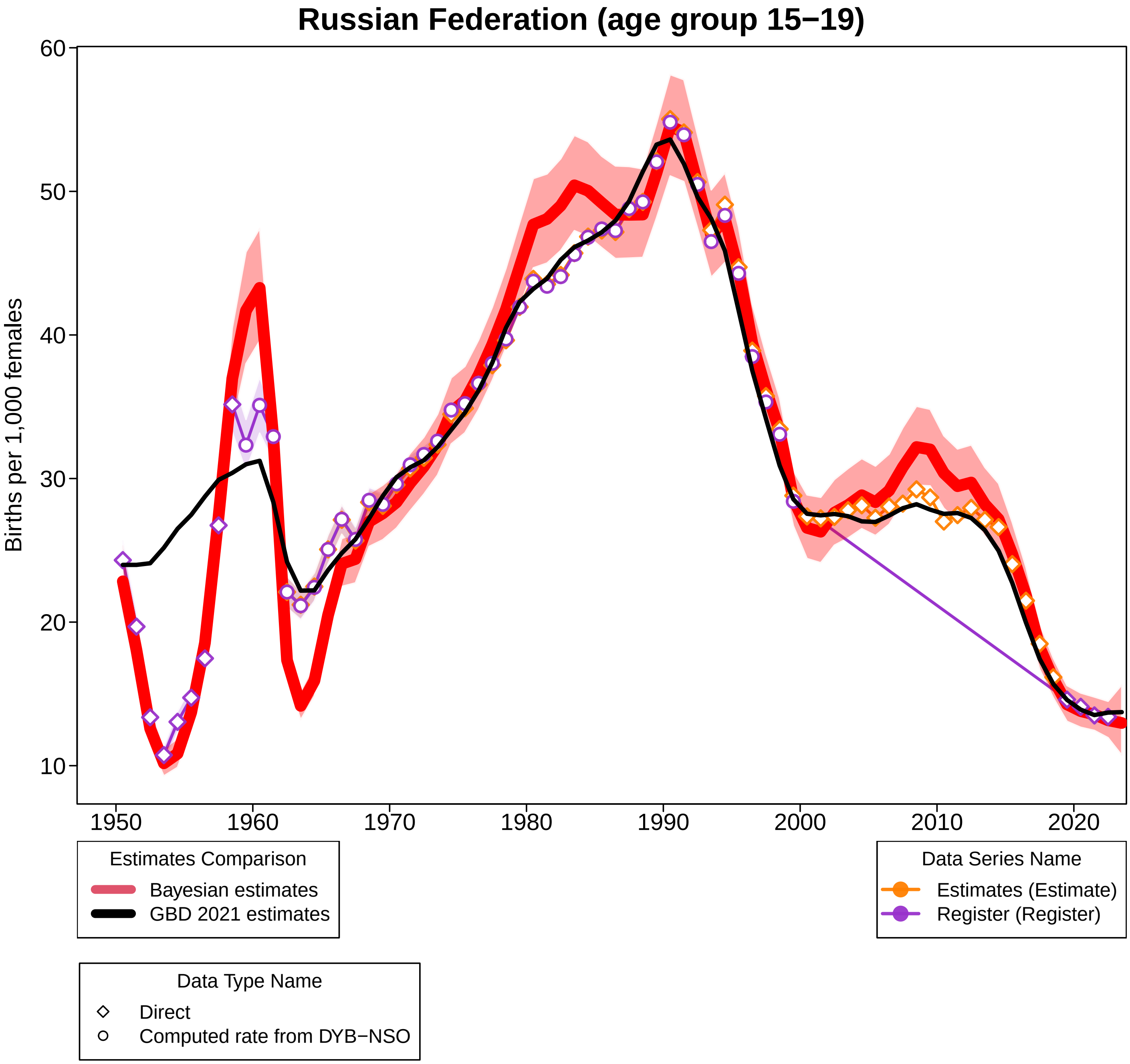

Russian Federation (age group 15−19)
Births per 1,000 females
60
50
40
30
20
10
1950
1960
1970
1980
1990
2000
2010
2020
Estimates Comparison
Bayesian estimates
GBD 2021 estimates
Data Series Name
Estimates (Estimate)
Register (Register)
Data Type Name
Direct
Computed rate from DYB−NSO

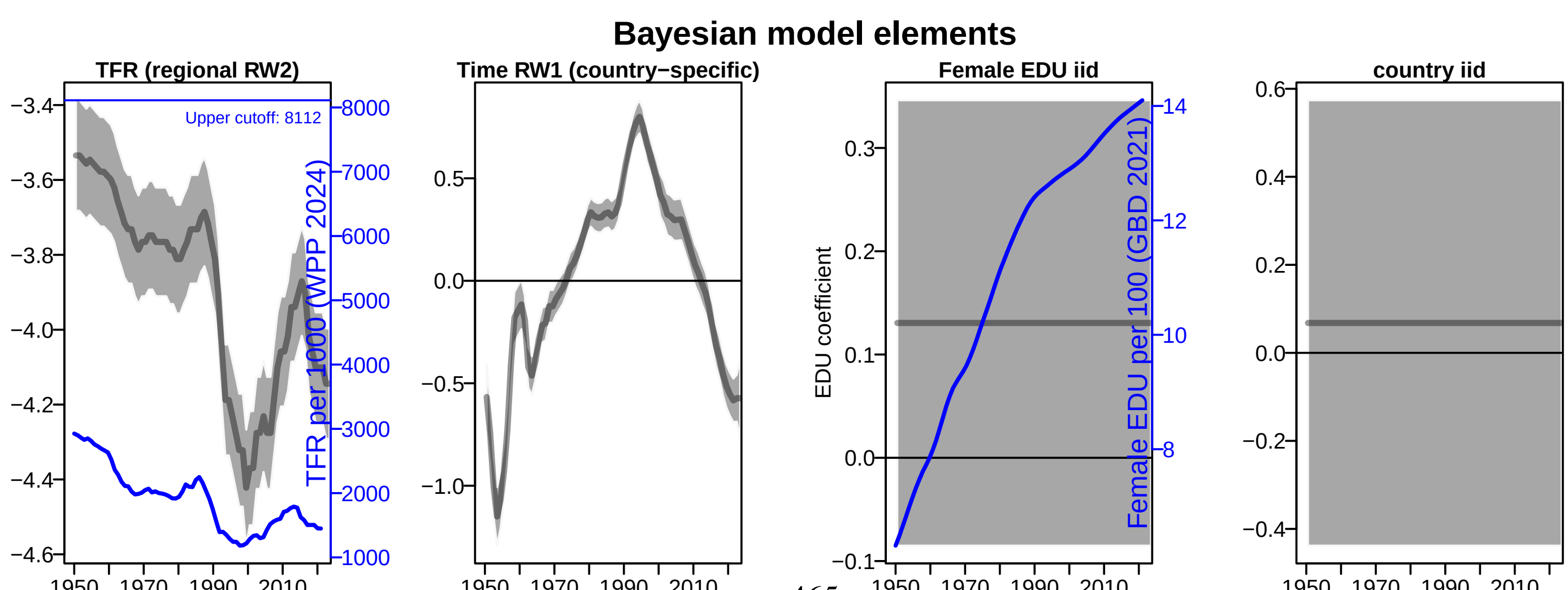

Bayesian model elements
TFR (regional RW2)
Upper cutoff: 8112
TFR per 1000 (WPP 2024)
Time RW1 (country−specific)
Female EDU iid
EDU coefficient
Female EDU per 100 (GBD 2021)
country iid

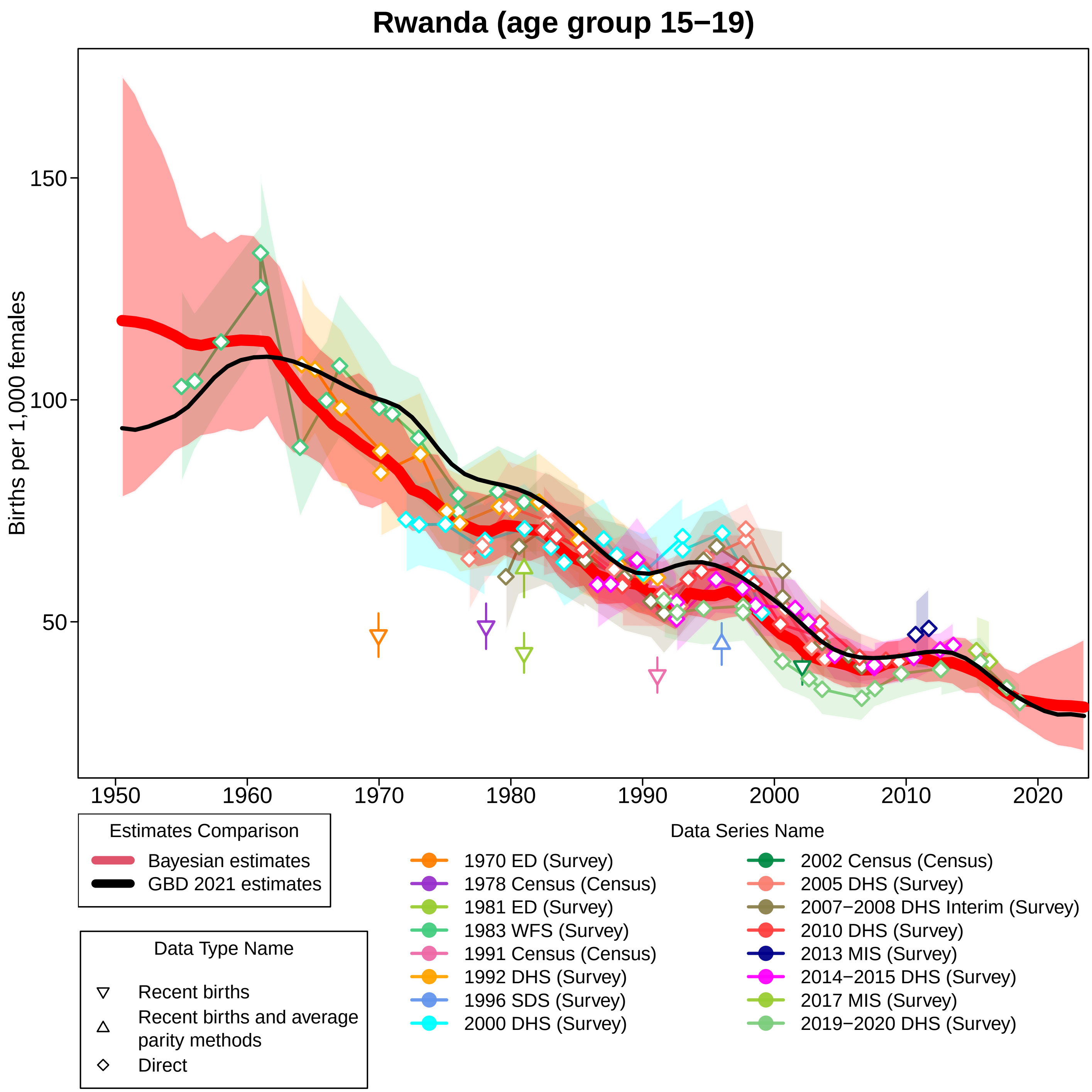
Rwanda (age group 15−19)
Births per 1,000 females
150
100
50
1950
1960
1970
1980
1990
2000
2010
2020
Estimates Comparison
Bayesian estimates
GBD 2021 estimates
Data Type Name
Recent births
Recent births and average parity methods
Direct
Data Series Name
1970 ED (Survey)
1978 Census (Census)
1981 ED (Survey)
1983 WFS (Survey)
1991 Census (Census)
1992 DHS (Survey)
1996 SDS (Survey)
2000 DHS (Survey)
2002 Census (Census)
2005 DHS (Survey)
2007−2008 DHS Interim (Survey)
2010 DHS (Survey)
2013 MIS (Survey)
2014−2015 DHS (Survey)
2017 MIS (Survey)
2019−2020 DHS (Survey)

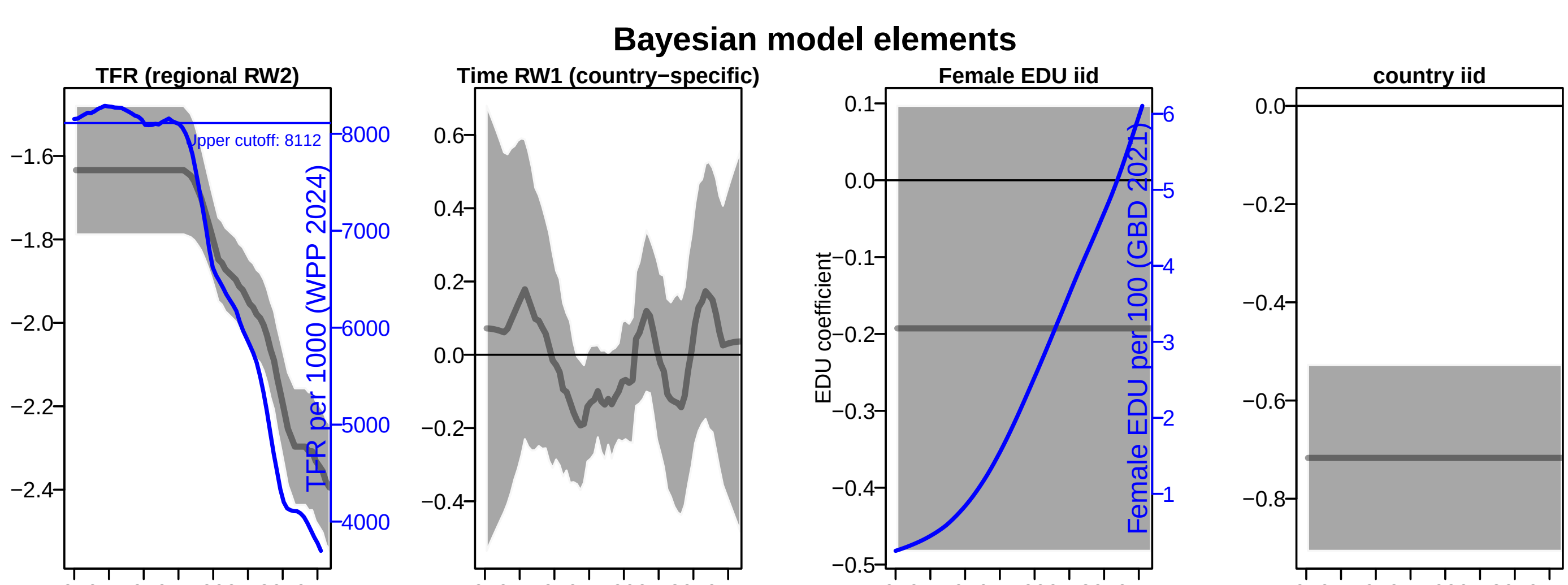
Bayesian model elements
TFR (regional RW2)
Upper cutoff: 8112
TFR per 1000 (WPP 2024)
Time RW1 (country−specific)
Female EDU iid
EDU coefficient
Female EDU per 100 (GBD 2021)
country iid
1950
1970
1990
2010

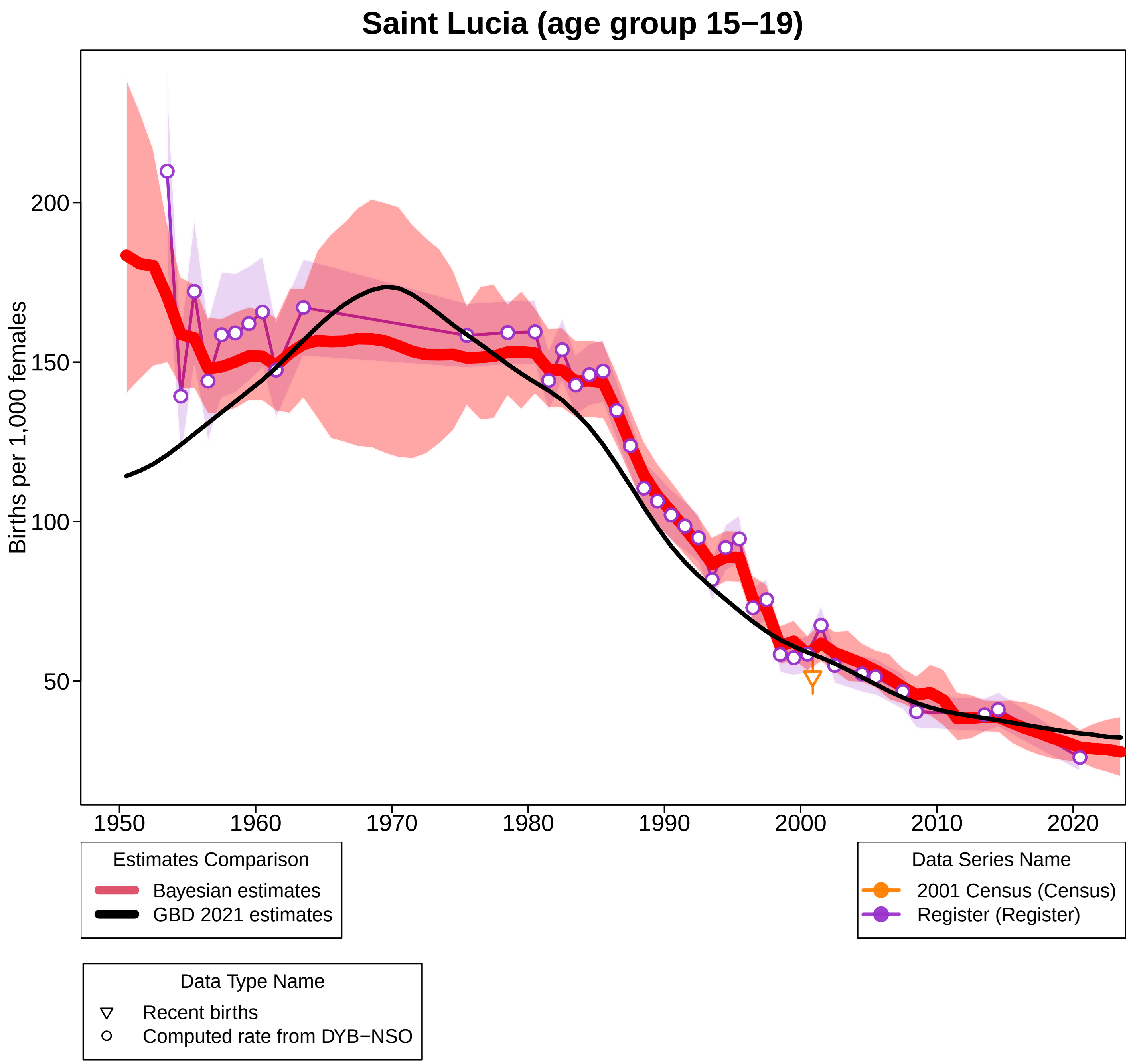
Saint Lucia (age group 15−19)
Births per 1,000 females
200
150
100
50
1950
1960
1970
1980
1990
2000
2010
2020
Estimates Comparison
Bayesian estimates
GBD 2021 estimates
Data Series Name
2001 Census (Census)
Register (Register)
Data Type Name
Recent births
Computed rate from DYB−NSO

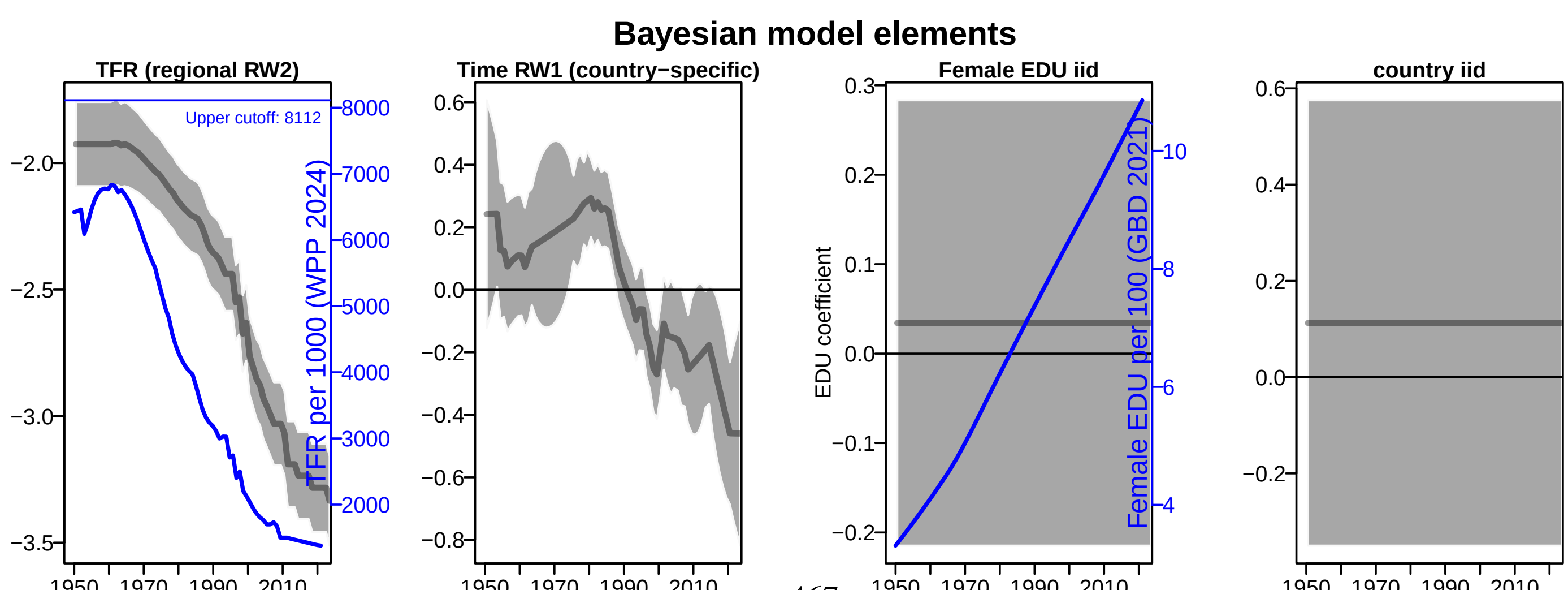
Bayesian model elements
TFR (regional RW2)
Upper cutoff: 8112
TFR per 1000 (WPP 2024)
Time RW1 (country−specific)
Female EDU iid
EDU coefficient
Female EDU per 100 (GBD 2021)
country iid

Saint Vincent and the Grenadines (age group 15−19)

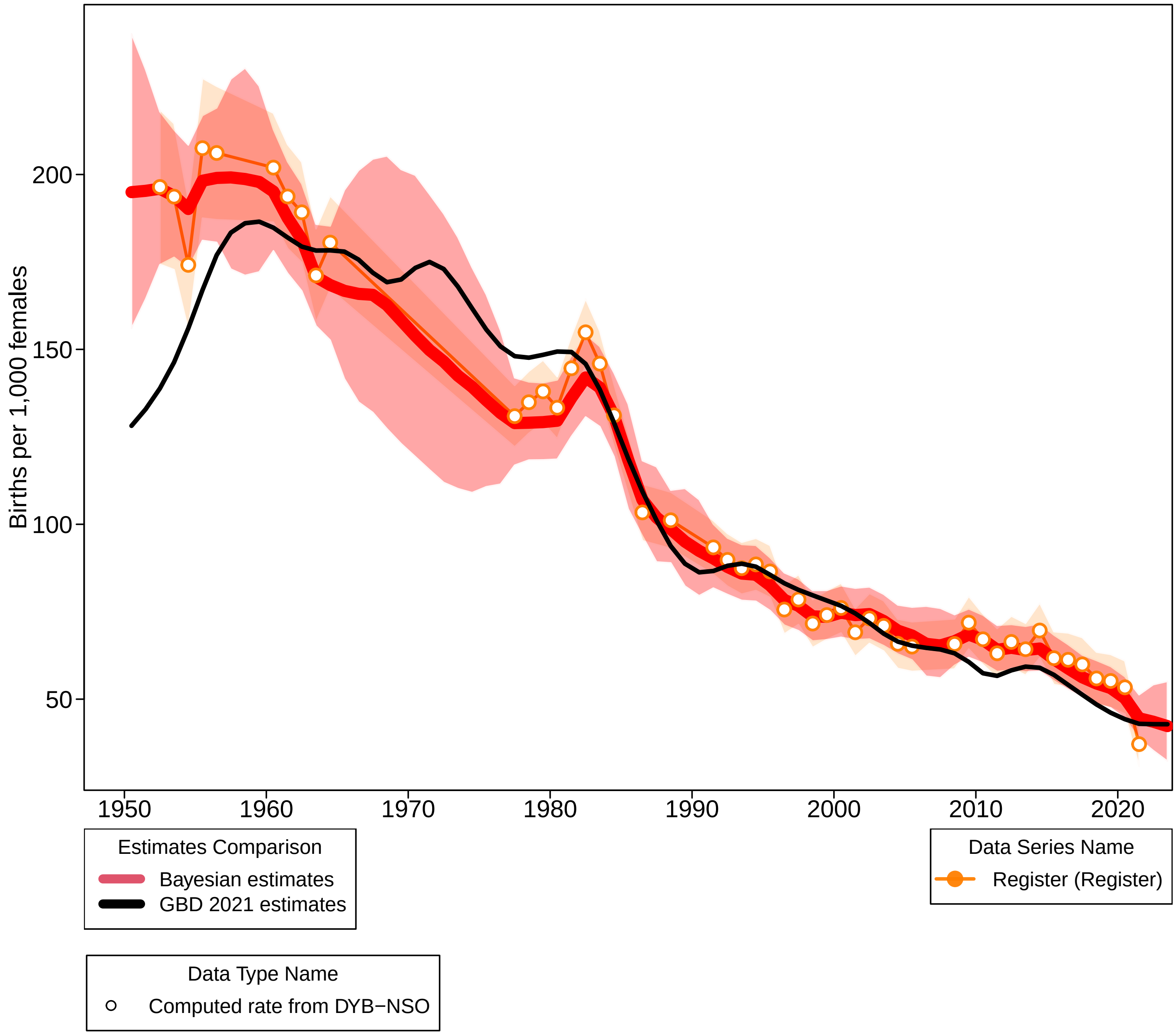

Bayesian model elements

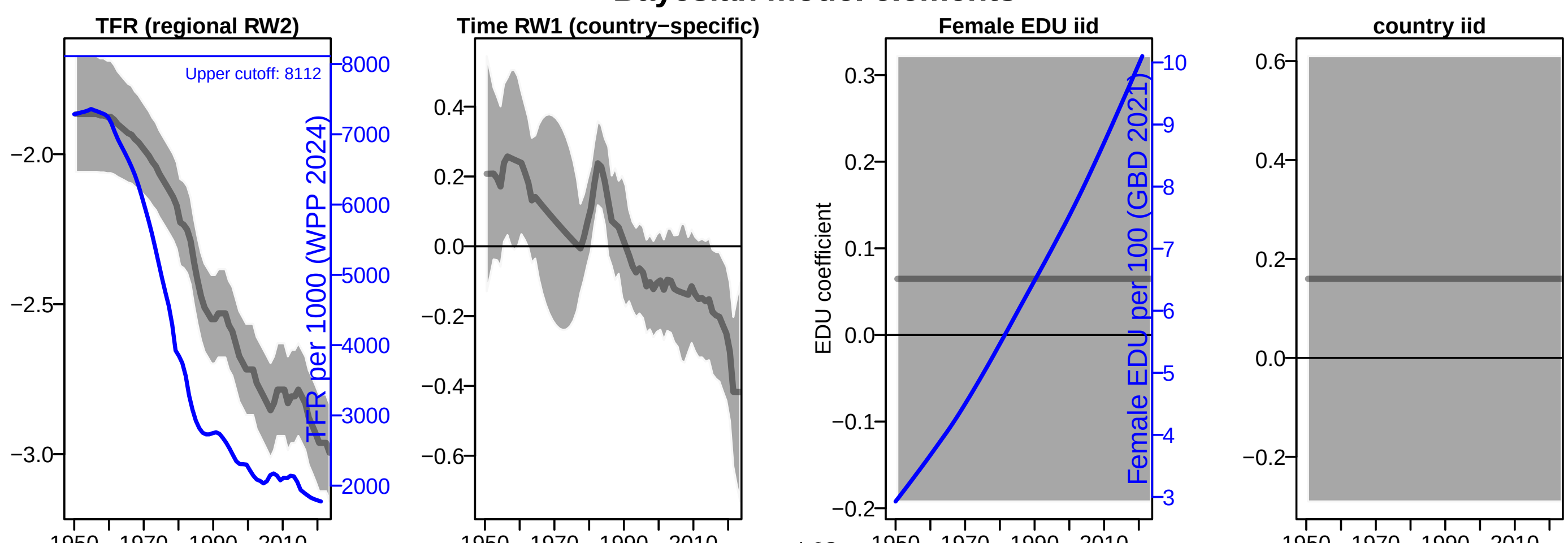

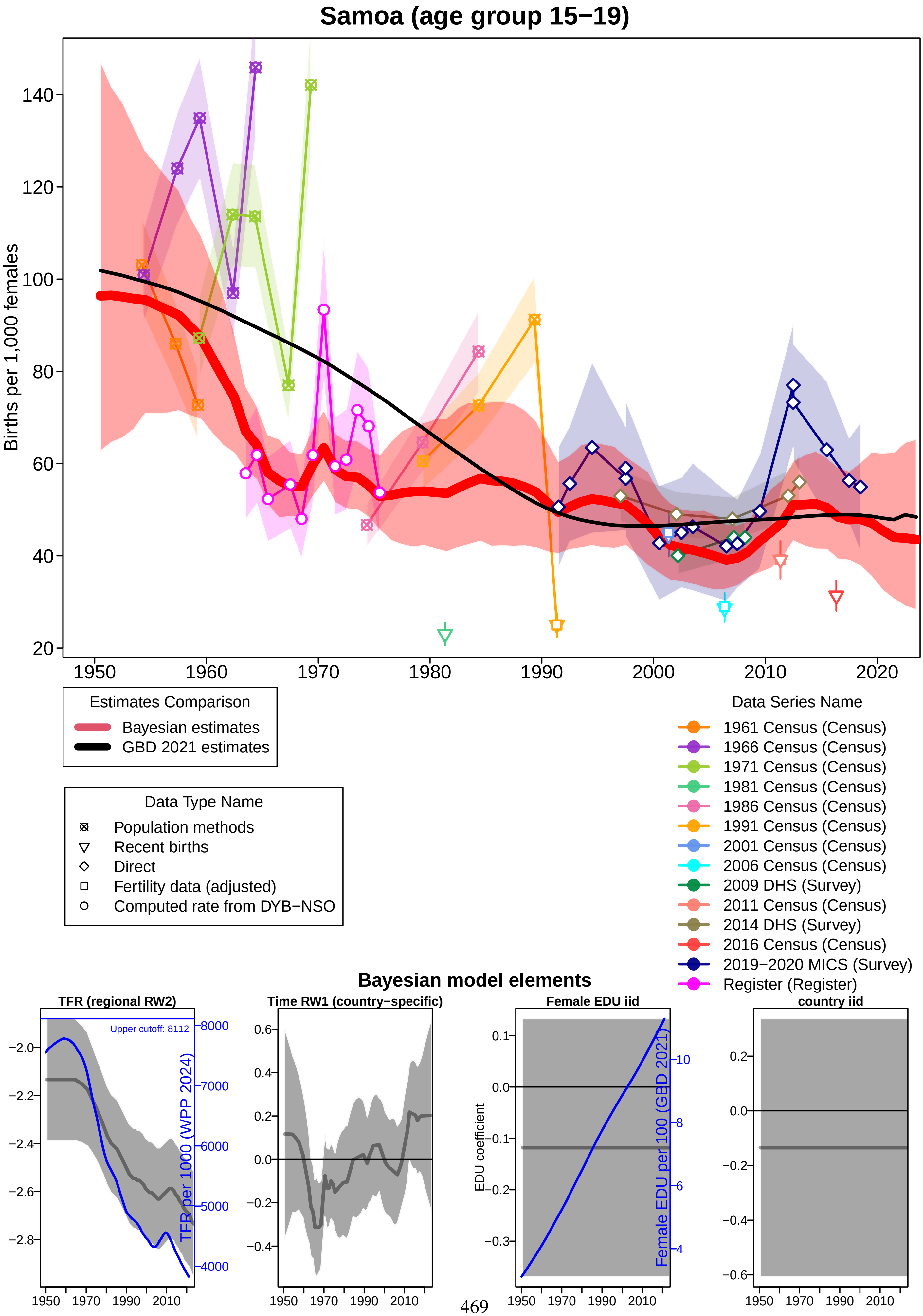
Samoa (age group 15−19)
Births per 1,000 females
140
120
100
80
60
40
20
1950
1960
1970
1980
1990
2000
2010
2020
Estimates Comparison
Bayesian estimates
GBD 2021 estimates
Data Type Name
Population methods
Recent births
Direct
Fertility data (adjusted)
Computed rate from DYB−NSO
Data Series Name
1961 Census (Census)
1966 Census (Census)
1971 Census (Census)
1981 Census (Census)
1986 Census (Census)
1991 Census (Census)
2001 Census (Census)
2006 Census (Census)
2009 DHS (Survey)
2011 Census (Census)
2014 DHS (Survey)
2016 Census (Census)
2019−2020 MICS (Survey)
Register (Register)
Bayesian model elements
TFR (regional RW2)
Upper cutoff: 8112
TFR per 1000 (WPP 2024)
Time RW1 (country−specific)
Female EDU iid
EDU coefficient
Female EDU per 100 (GBD 2021)
country iid

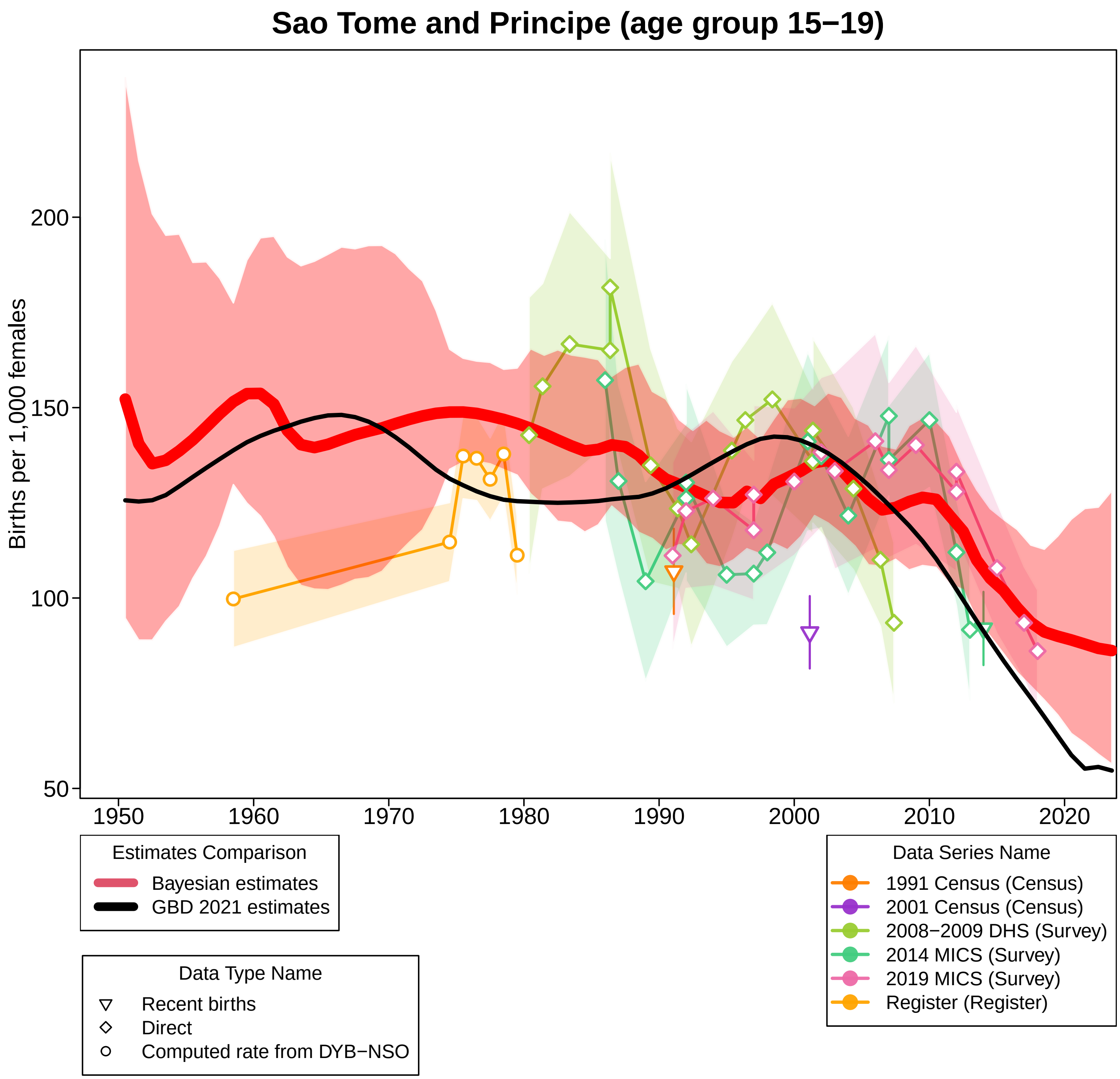

Sao Tome and Principe (age group 15−19)
Births per 1,000 females
200
150
100
50
1950
1960
1970
1980
1990
2000
2010
2020
Estimates Comparison
Bayesian estimates
GBD 2021 estimates
Data Type Name
Recent births
Direct
Computed rate from DYB−NSO
Data Series Name
1991 Census (Census)
2001 Census (Census)
2008−2009 DHS (Survey)
2014 MICS (Survey)
2019 MICS (Survey)
Register (Register)

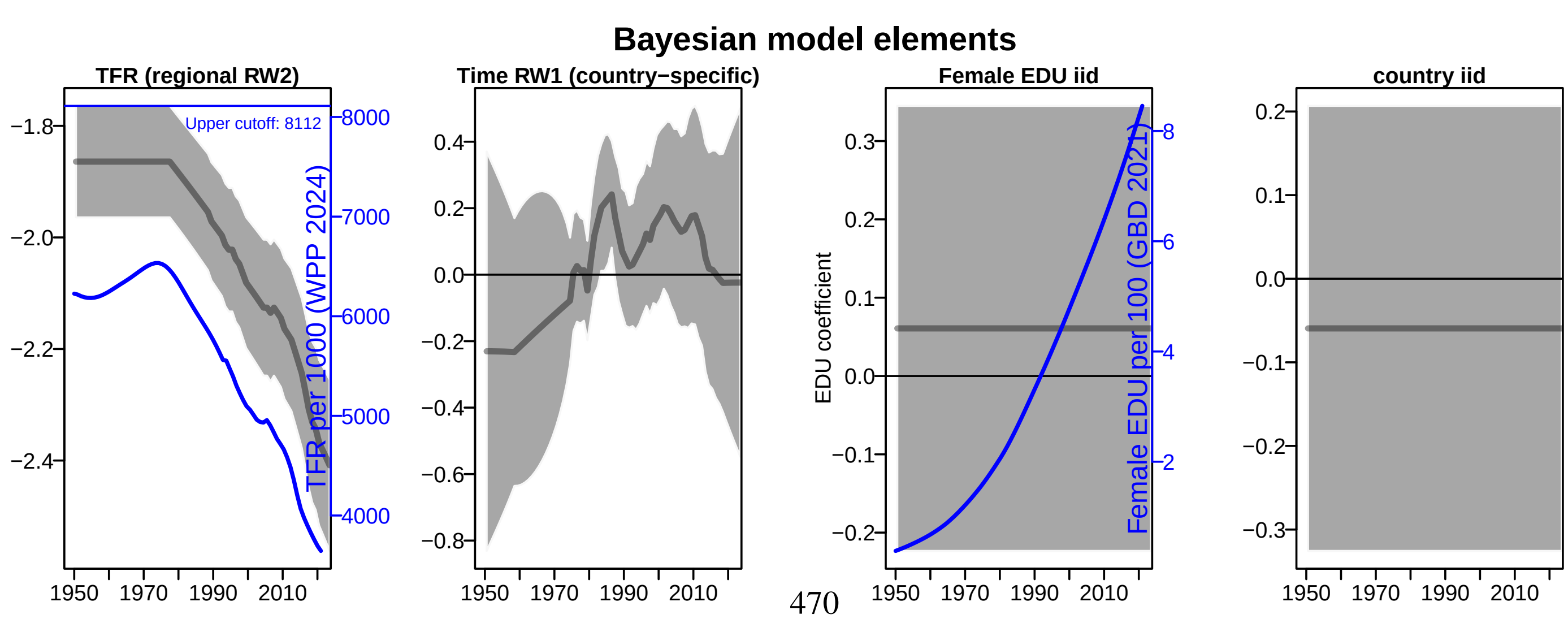

Bayesian model elements
TFR (regional RW2)
Upper cutoff: 8112
TFR per 1000 (WPP 2024)
Time RW1 (country−specific)
Female EDU iid
EDU coefficient
Female EDU per 100 (GBD 2021)
country iid

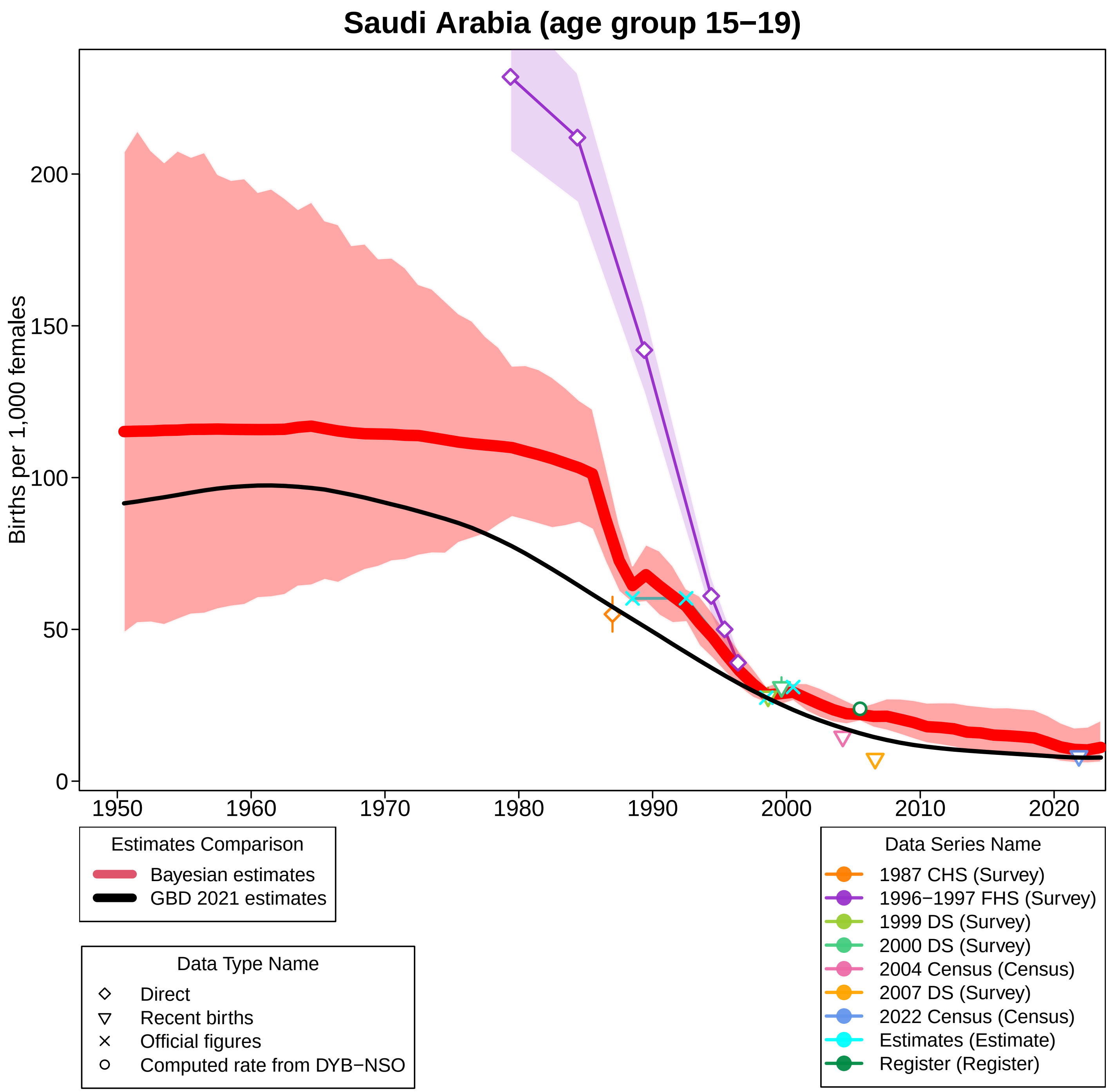
Saudi Arabia (age group 15−19)
Births per 1,000 females
0
50
100
150
200
1950
1960
1970
1980
1990
2000
2010
2020
Estimates Comparison
Bayesian estimates
GBD 2021 estimates
Data Type Name
Direct
Recent births
Official figures
Computed rate from DYB−NSO
Data Series Name
1987 CHS (Survey)
1996−1997 FHS (Survey)
1999 DS (Survey)
2000 DS (Survey)
2004 Census (Census)
2007 DS (Survey)
2022 Census (Census)
Estimates (Estimate)
Register (Register)

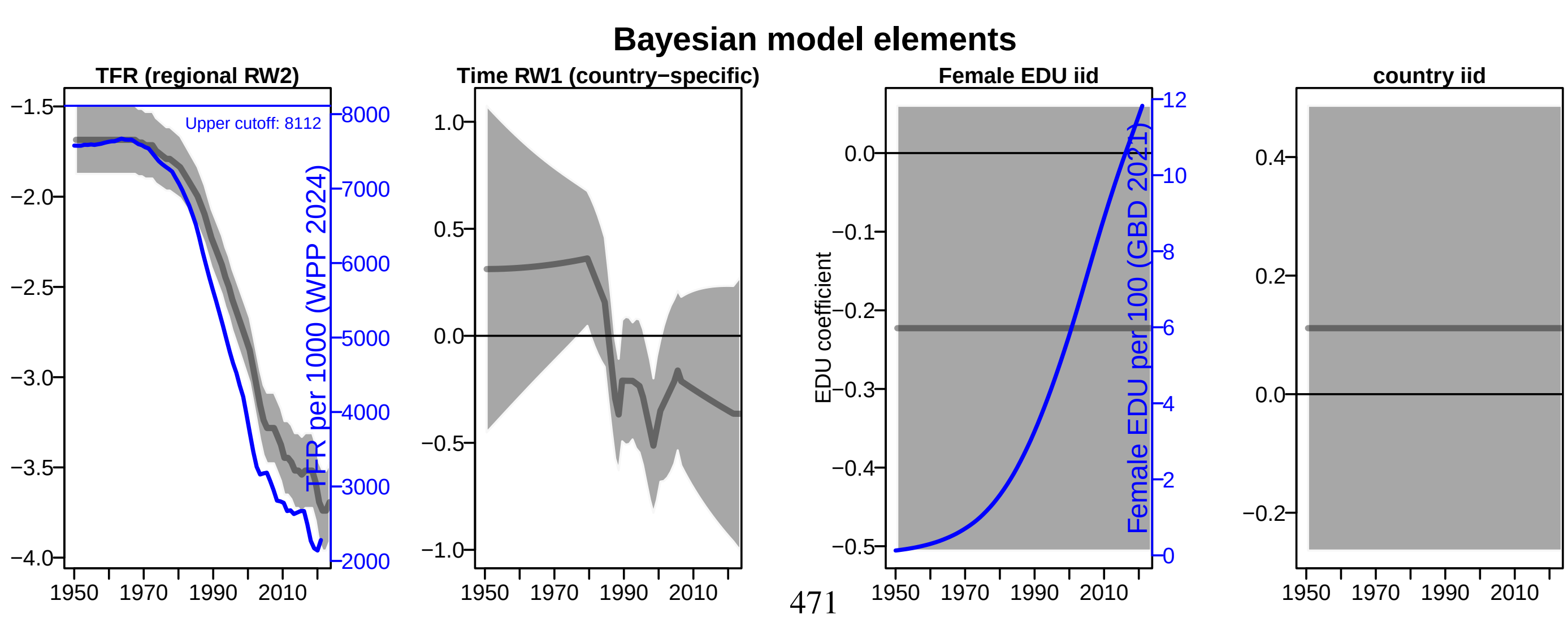
Bayesian model elements
TFR (regional RW2)
Upper cutoff: 8112
TFR per 1000 (WPP 2024)
Time RW1 (country−specific)
Female EDU iid
EDU coefficient
Female EDU per 100 (GBD 2021)
country iid

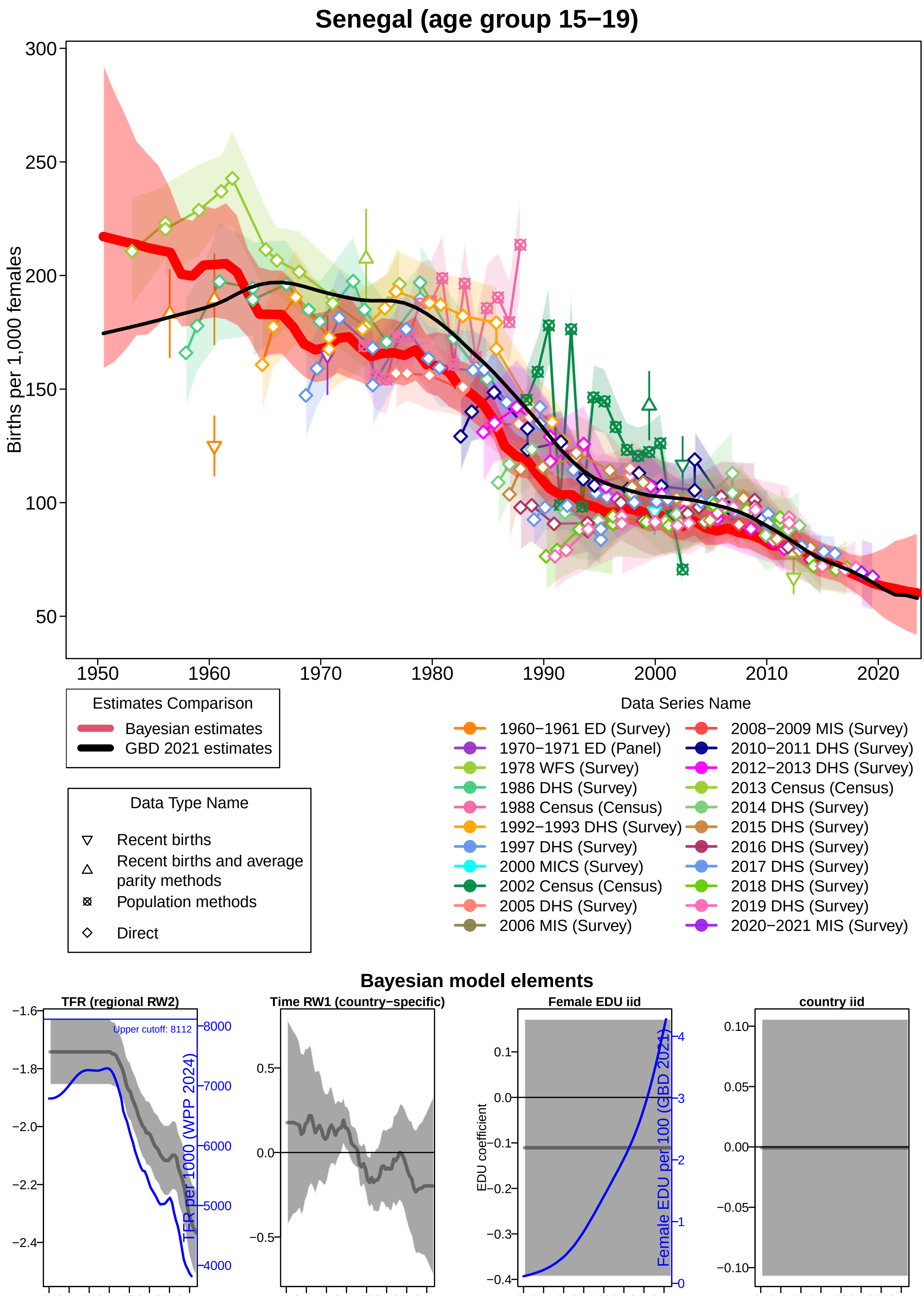

Senegal (age group 15−19)
Births per 1,000 females
300
250
200
150
100
50
1950
1960
1970
1980
1990
2000
2010
2020
Estimates Comparison
Bayesian estimates
GBD 2021 estimates
Data Type Name
Recent births
Recent births and average parity methods
Population methods
Direct
Data Series Name
1960−1961 ED (Survey)
1970−1971 ED (Panel)
1978 WFS (Survey)
1986 DHS (Survey)
1988 Census (Census)
1992−1993 DHS (Survey)
1997 DHS (Survey)
2000 MICS (Survey)
2002 Census (Census)
2005 DHS (Survey)
2006 MIS (Survey)
2008−2009 MIS (Survey)
2010−2011 DHS (Survey)
2012−2013 DHS (Survey)
2013 Census (Census)
2014 DHS (Survey)
2015 DHS (Survey)
2016 DHS (Survey)
2017 DHS (Survey)
2018 DHS (Survey)
2019 DHS (Survey)
2020−2021 MIS (Survey)
Bayesian model elements
TFR (regional RW2)
Upper cutoff: 8112
TFR per 1000 (WPP 2024)
Time RW1 (country−specific)
Female EDU iid
EDU coefficient
Female EDU per 100 (GBD 2021)
country iid

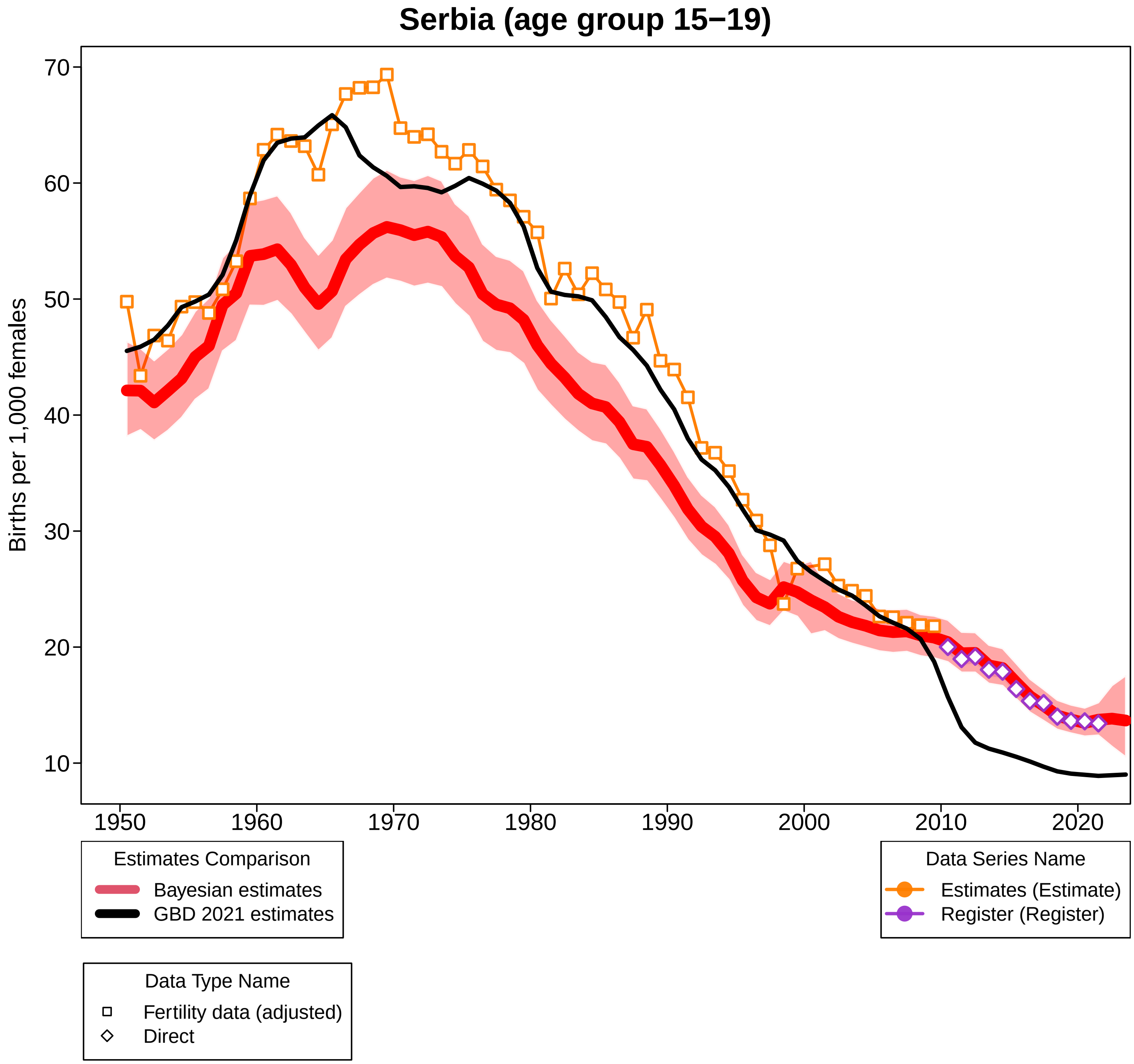
Serbia (age group 15−19)
Births per 1,000 females
70
60
50
40
30
20
10
1950
1960
1970
1980
1990
2000
2010
2020
Estimates Comparison
Bayesian estimates
GBD 2021 estimates
Data Series Name
Estimates (Estimate)
Register (Register)
Data Type Name
Fertility data (adjusted)
Direct

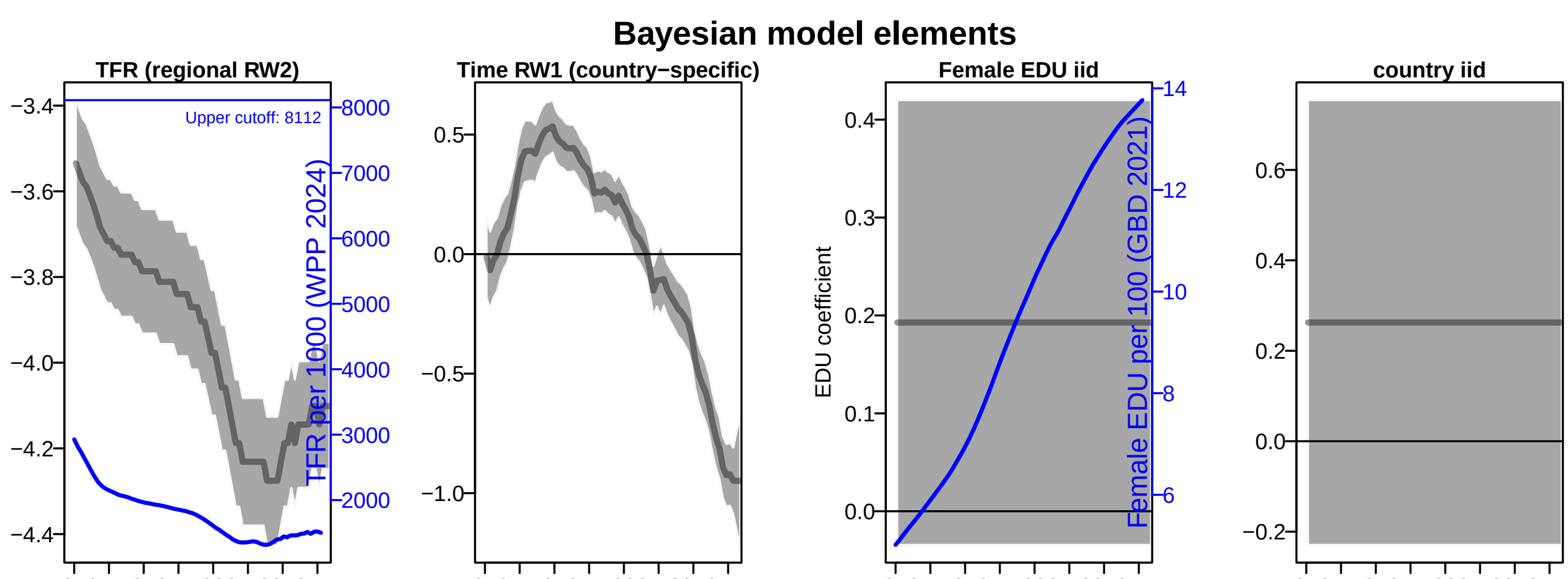
Bayesian model elements
TFR (regional RW2)
Upper cutoff: 8112
TFR per 1000 (WPP 2024)
−3.4
−3.6
−3.8
−4.0
−4.2
−4.4
8000
7000
6000
5000
4000
3000
2000
Time RW1 (country−specific)
0.5
0.0
−0.5
−1.0
Female EDU iid
EDU coefficient
Female EDU per 100 (GBD 2021)
0.4
0.3
0.2
0.1
0.0
14
12
10
8
6
country iid
0.6
0.4
0.2
0.0
−0.2
1950
1970
1990
2010

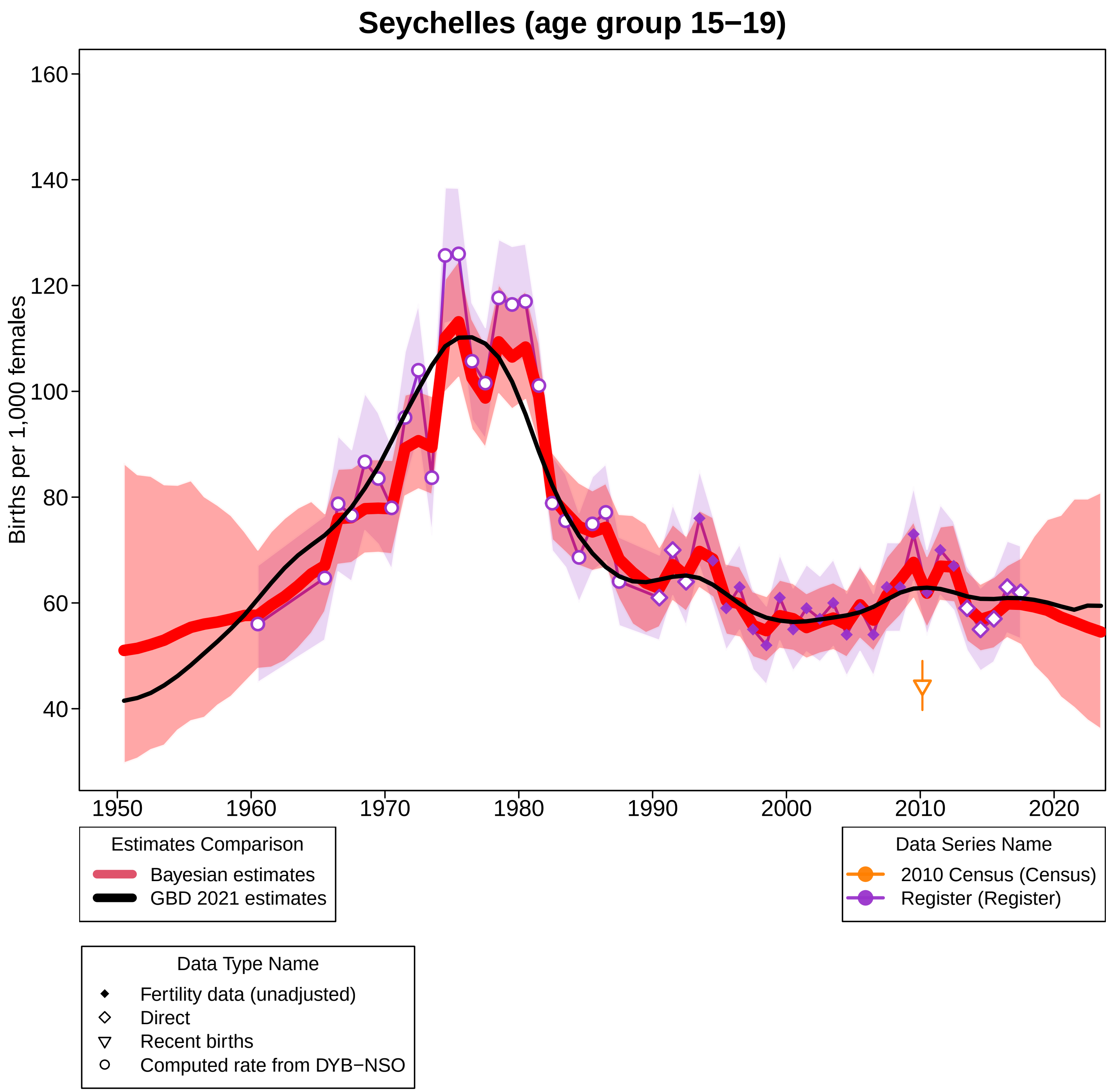

Seychelles (age group 15−19)
Births per 1,000 females
160
140
120
100
80
60
40
1950
1960
1970
1980
1990
2000
2010
2020
Estimates Comparison
Bayesian estimates
GBD 2021 estimates
Data Series Name
2010 Census (Census)
Register (Register)
Data Type Name
Fertility data (unadjusted)
Direct
Recent births
Computed rate from DYB−NSO

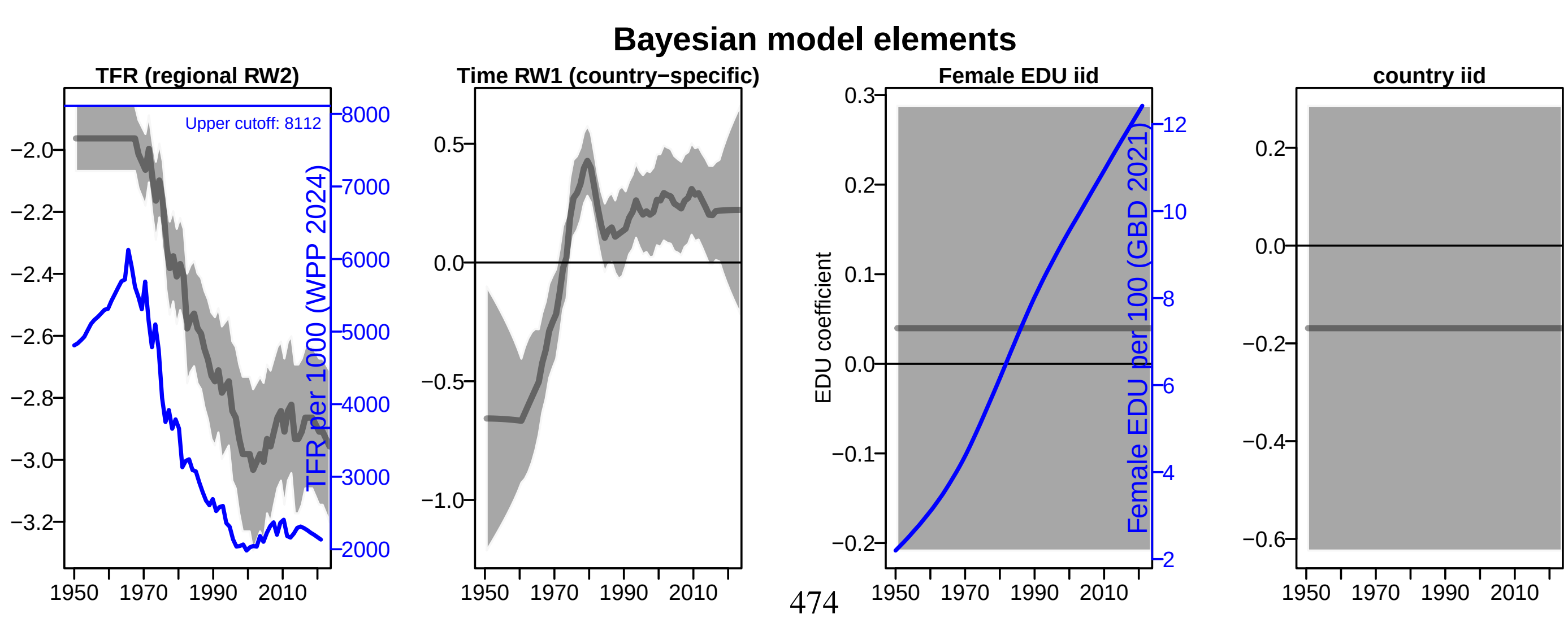

Bayesian model elements
TFR (regional RW2)
Upper cutoff: 8112
TFR per 1000 (WPP 2024)
Time RW1 (country−specific)
Female EDU iid
EDU coefficient
Female EDU per 100 (GBD 2021)
country iid

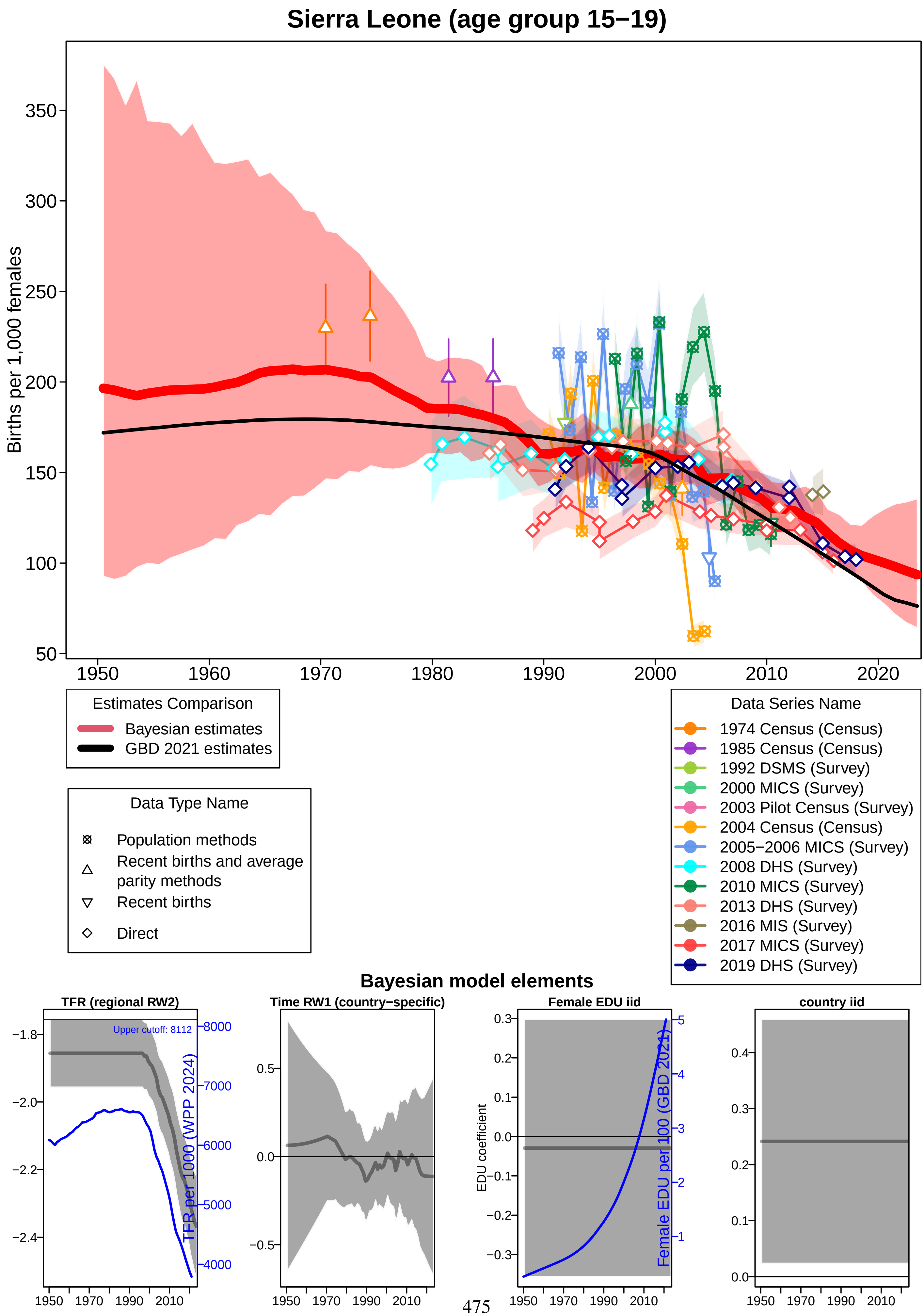

Sierra Leone (age group 15−19)
Births per 1,000 females
350
300
250
200
150
100
50
1950
1960
1970
1980
1990
2000
2010
2020
Estimates Comparison
Bayesian estimates
GBD 2021 estimates
Data Type Name
Population methods
Recent births and average parity methods
Recent births
Direct
Data Series Name
1974 Census (Census)
1985 Census (Census)
1992 DSMS (Survey)
2000 MICS (Survey)
2003 Pilot Census (Survey)
2004 Census (Census)
2005−2006 MICS (Survey)
2008 DHS (Survey)
2010 MICS (Survey)
2013 DHS (Survey)
2016 MIS (Survey)
2017 MICS (Survey)
2019 DHS (Survey)
Bayesian model elements
TFR (regional RW2)
Upper cutoff: 8112
TFR per 1000 (WPP 2024)
Time RW1 (country−specific)
Female EDU iid
EDU coefficient
Female EDU per 100 (GBD 2021)
country iid

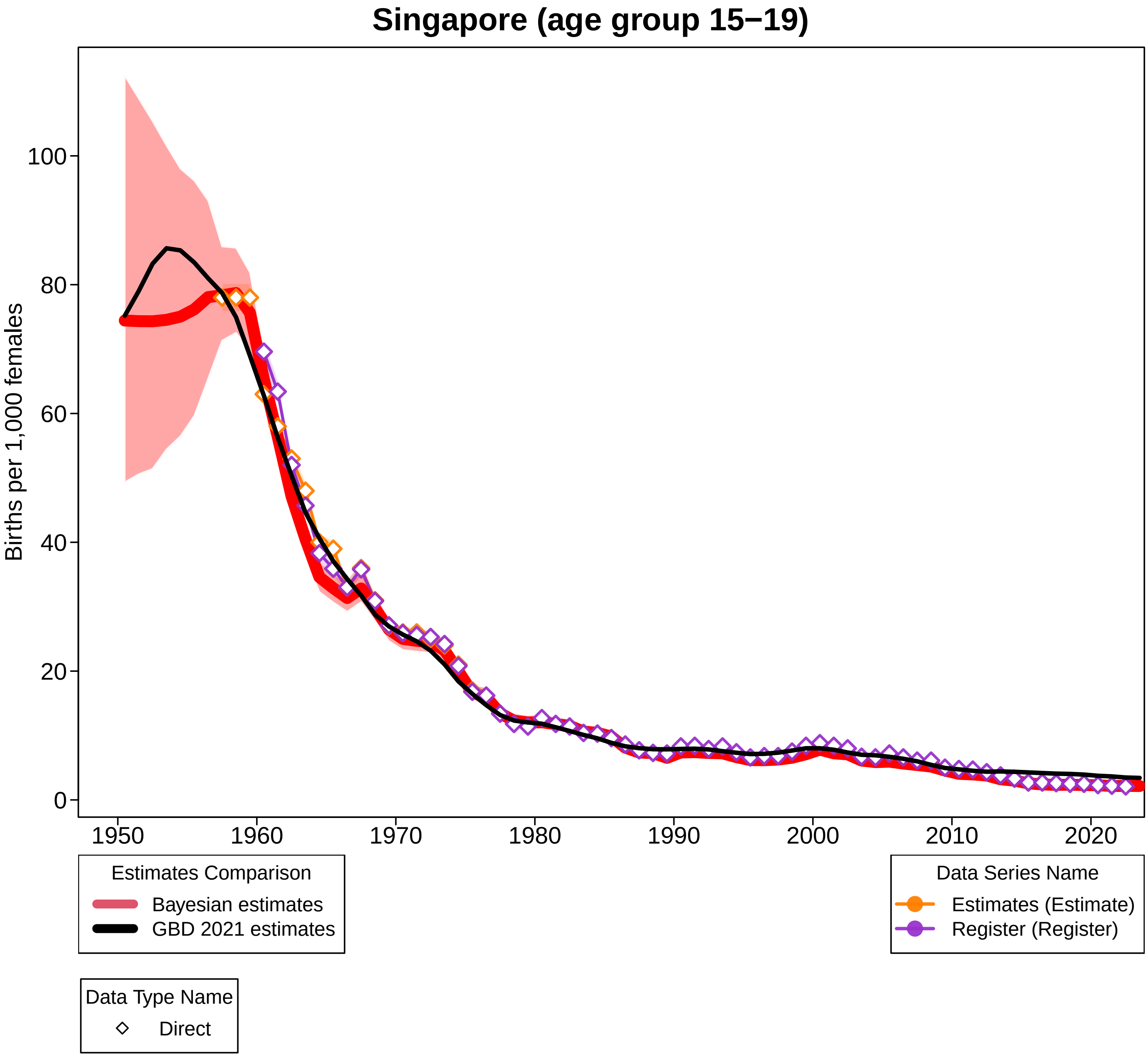

Singapore (age group 15−19)
Births per 1,000 females
100
80
60
40
20
0
1950
1960
1970
1980
1990
2000
2010
2020
Estimates Comparison
Bayesian estimates
GBD 2021 estimates
Data Series Name
Estimates (Estimate)
Register (Register)
Data Type Name
Direct

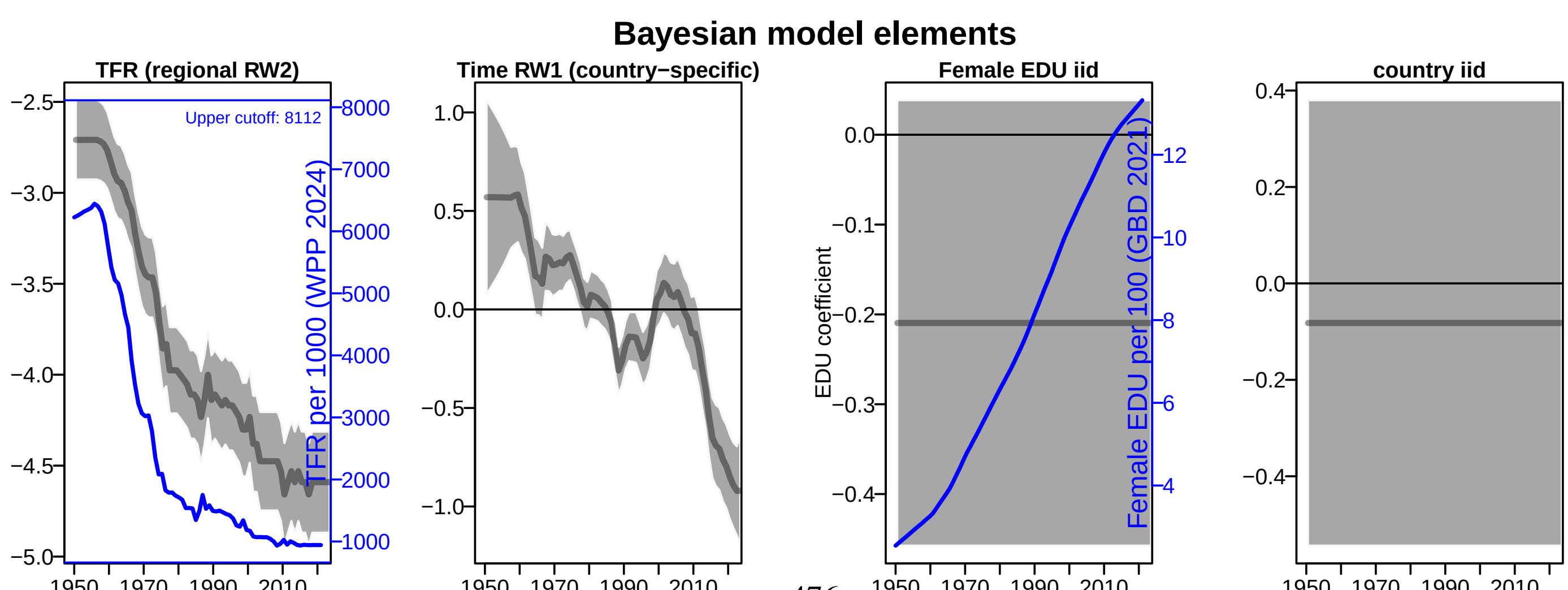

Bayesian model elements
TFR (regional RW2)
Upper cutoff: 8112
TFR per 1000 (WPP 2024)
Time RW1 (country−specific)
Female EDU iid
EDU coefficient
Female EDU per 100 (GBD 2021)
country iid

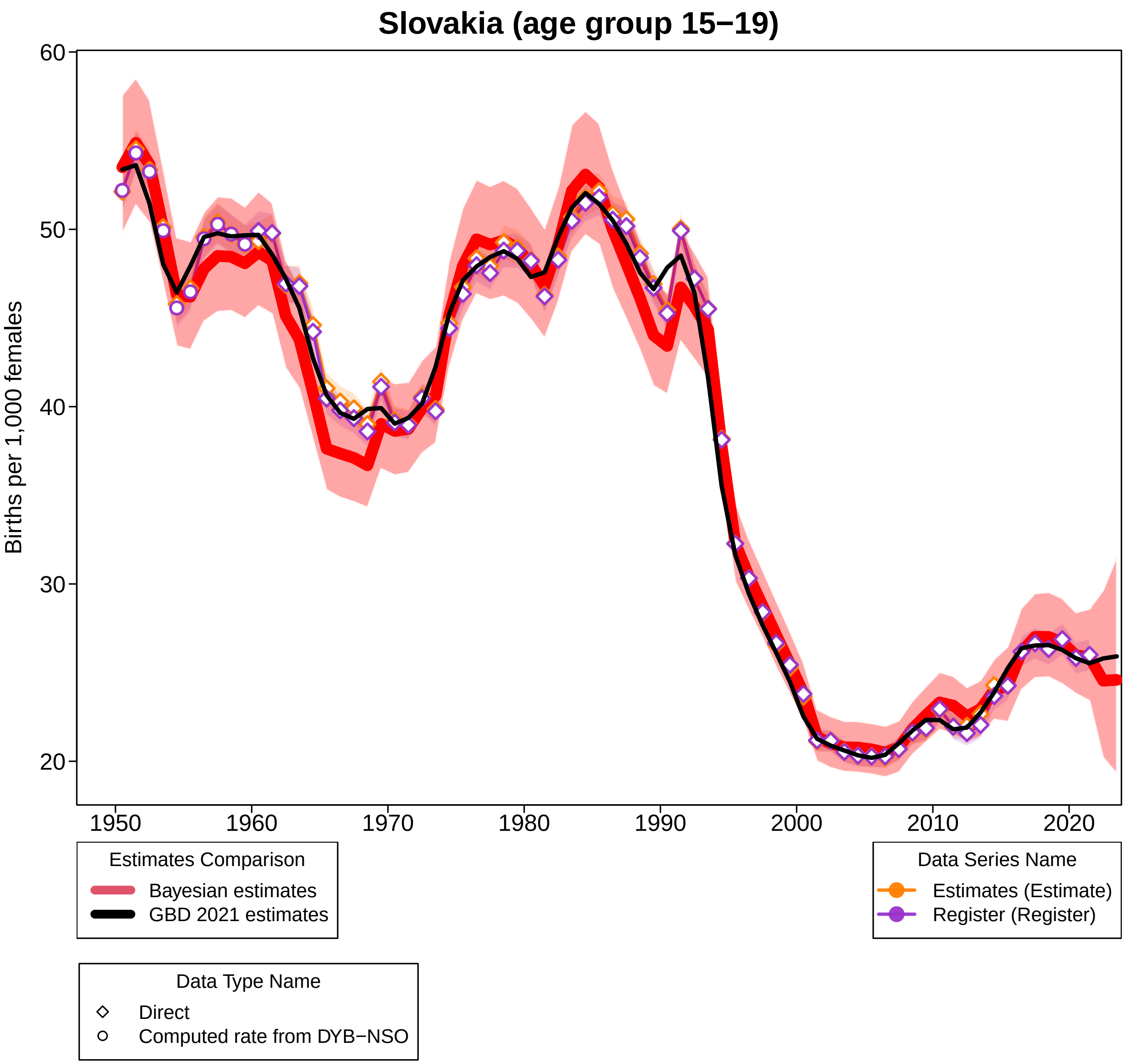
Slovakia (age group 15−19)
Births per 1,000 females
60
50
40
30
20
1950
1960
1970
1980
1990
2000
2010
2020
Estimates Comparison
Bayesian estimates
GBD 2021 estimates
Data Series Name
Estimates (Estimate)
Register (Register)
Data Type Name
Direct
Computed rate from DYB−NSO

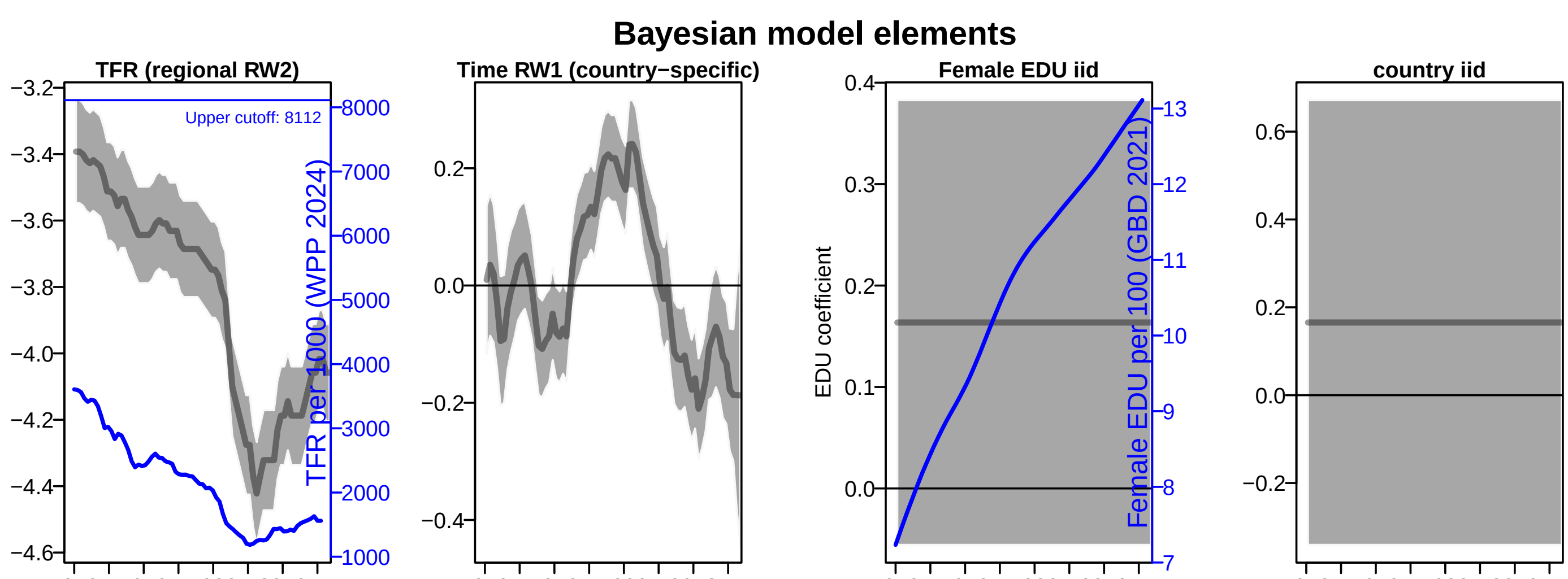
Bayesian model elements
TFR (regional RW2)
Upper cutoff: 8112
TFR per 1000 (WPP 2024)
Time RW1 (country−specific)
Female EDU iid
EDU coefficient
Female EDU per 100 (GBD 2021)
country iid
1950
1970
1990
2010

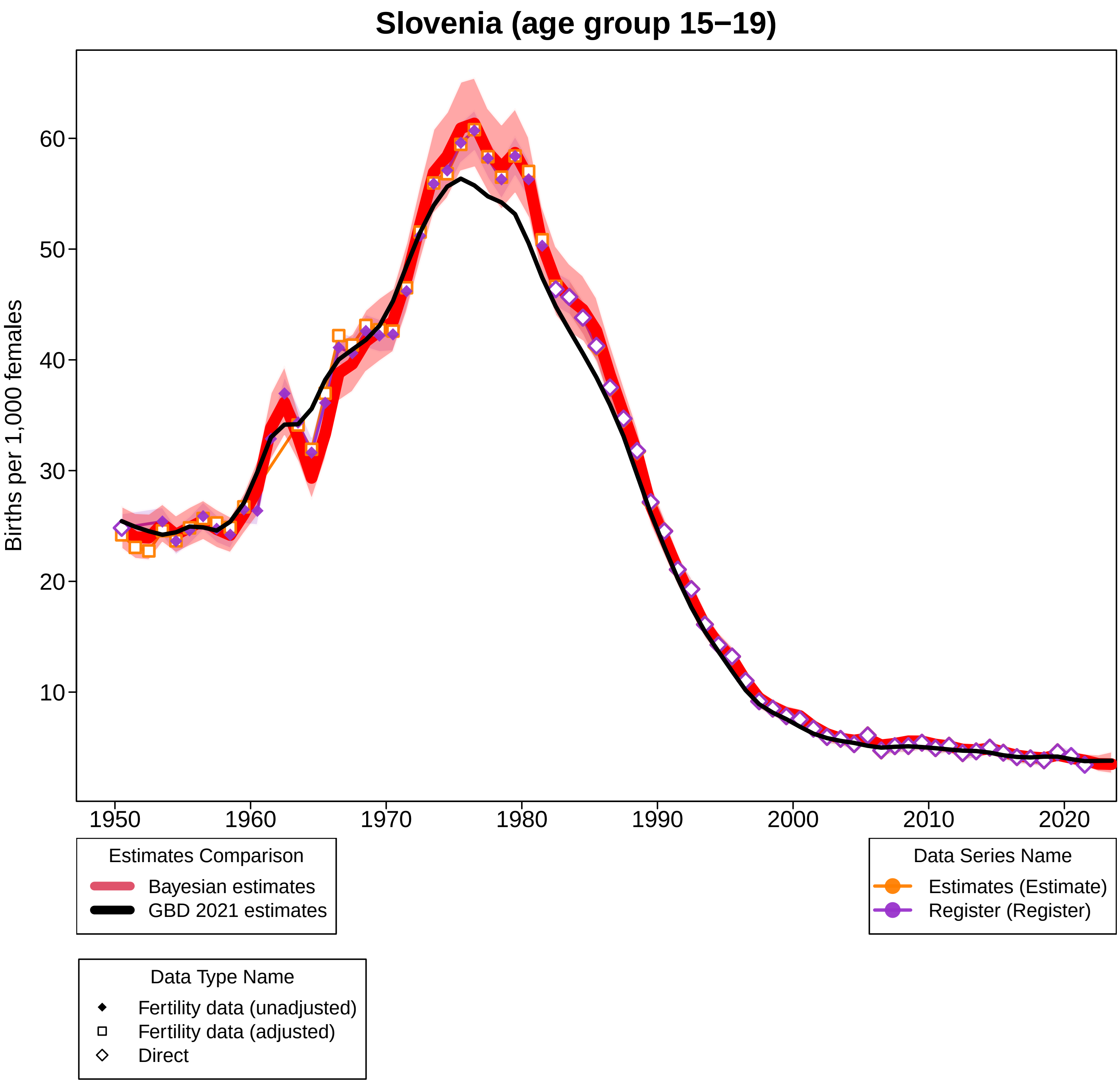

Slovenia (age group 15−19)
Births per 1,000 females
60
50
40
30
20
10
1950
1960
1970
1980
1990
2000
2010
2020
Estimates Comparison
Bayesian estimates
GBD 2021 estimates
Data Series Name
Estimates (Estimate)
Register (Register)
Data Type Name
Fertility data (unadjusted)
Fertility data (adjusted)
Direct

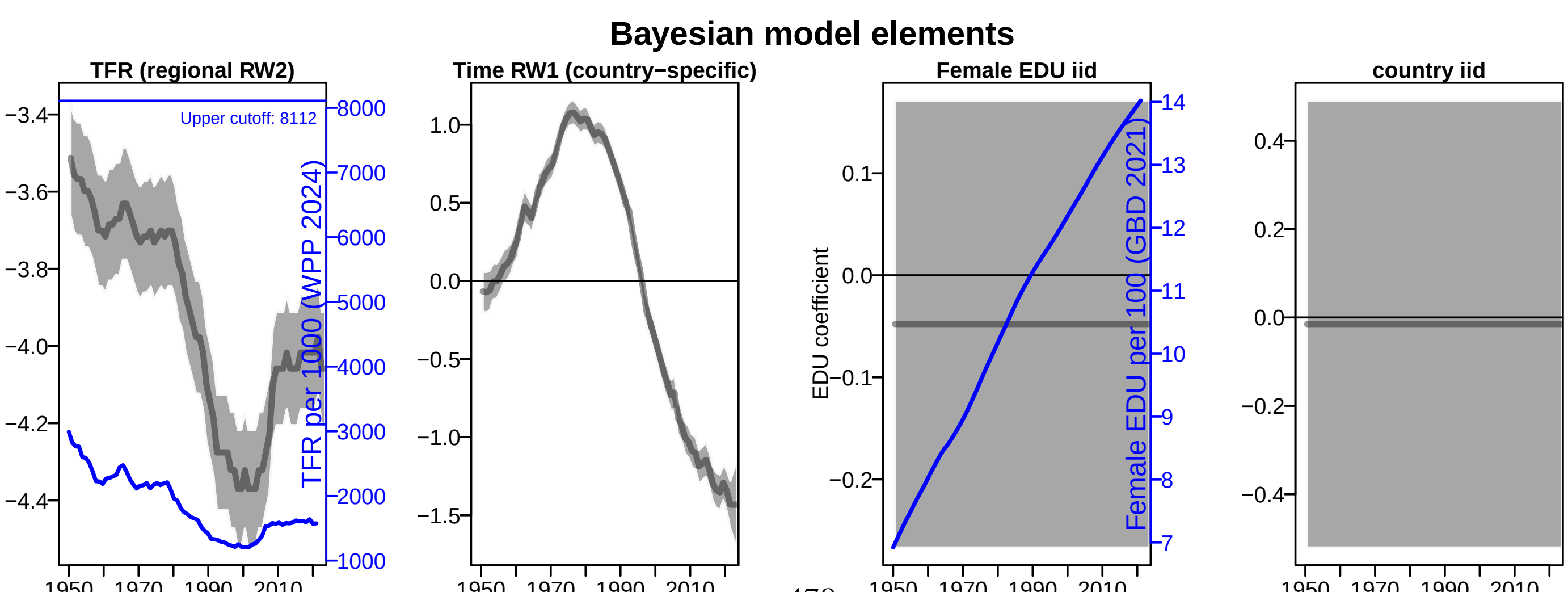

Bayesian model elements
TFR (regional RW2)
Upper cutoff: 8112
TFR per 1000 (WPP 2024)
Time RW1 (country−specific)
Female EDU iid
EDU coefficient
Female EDU per 100 (GBD 2021)
country iid

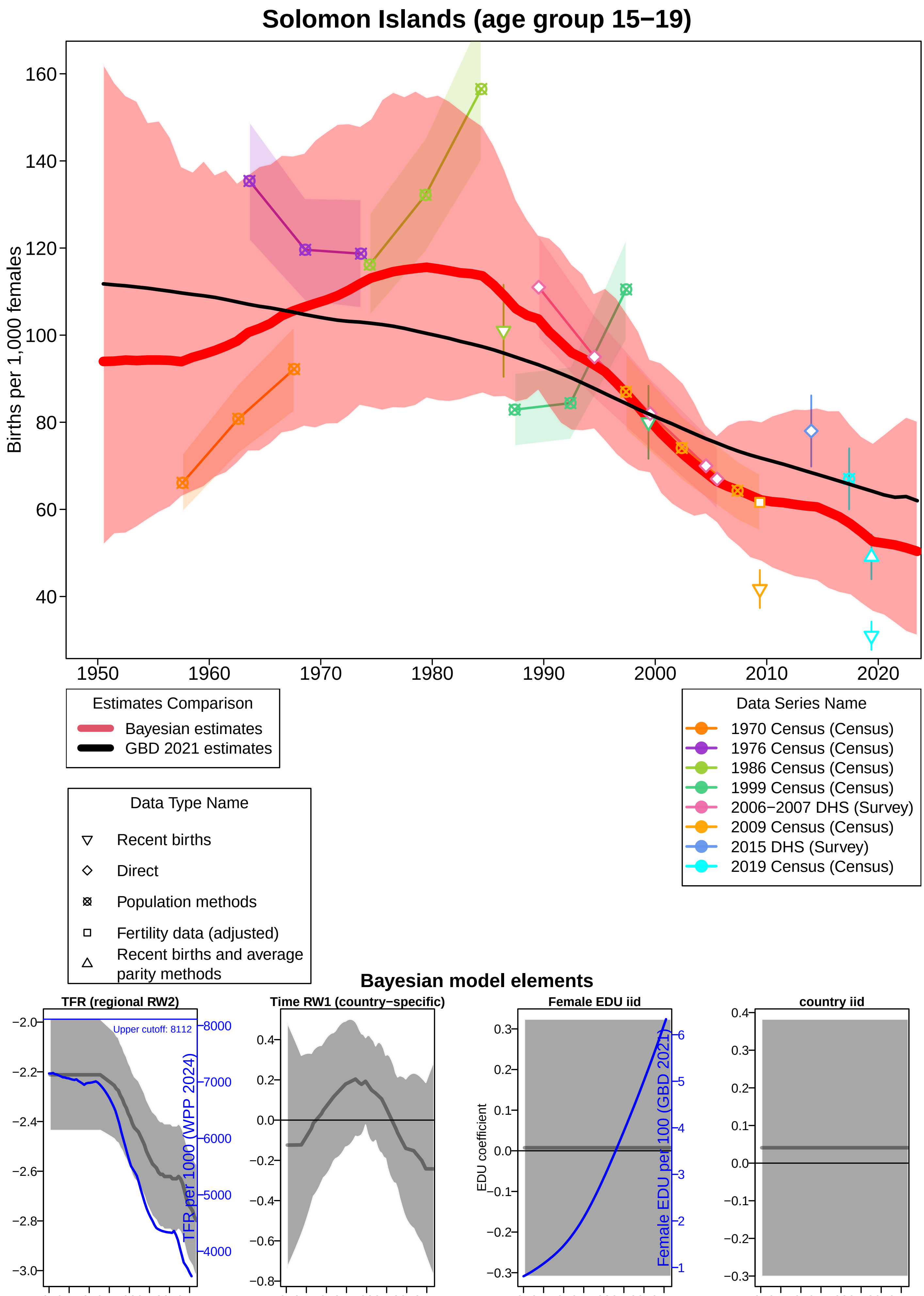
Solomon Islands (age group 15−19)
Births per 1,000 females
Estimates Comparison
Bayesian estimates
GBD 2021 estimates
Data Series Name
1970 Census (Census)
1976 Census (Census)
1986 Census (Census)
1999 Census (Census)
2006−2007 DHS (Survey)
2009 Census (Census)
2015 DHS (Survey)
2019 Census (Census)
Data Type Name
Recent births
Direct
Population methods
Fertility data (adjusted)
Recent births and average parity methods
Bayesian model elements
TFR (regional RW2)
Upper cutoff: 8112
TFR per 1000 (WPP 2024)
Time RW1 (country−specific)
Female EDU iid
EDU coefficient
Female EDU per 100 (GBD 2021)
country iid

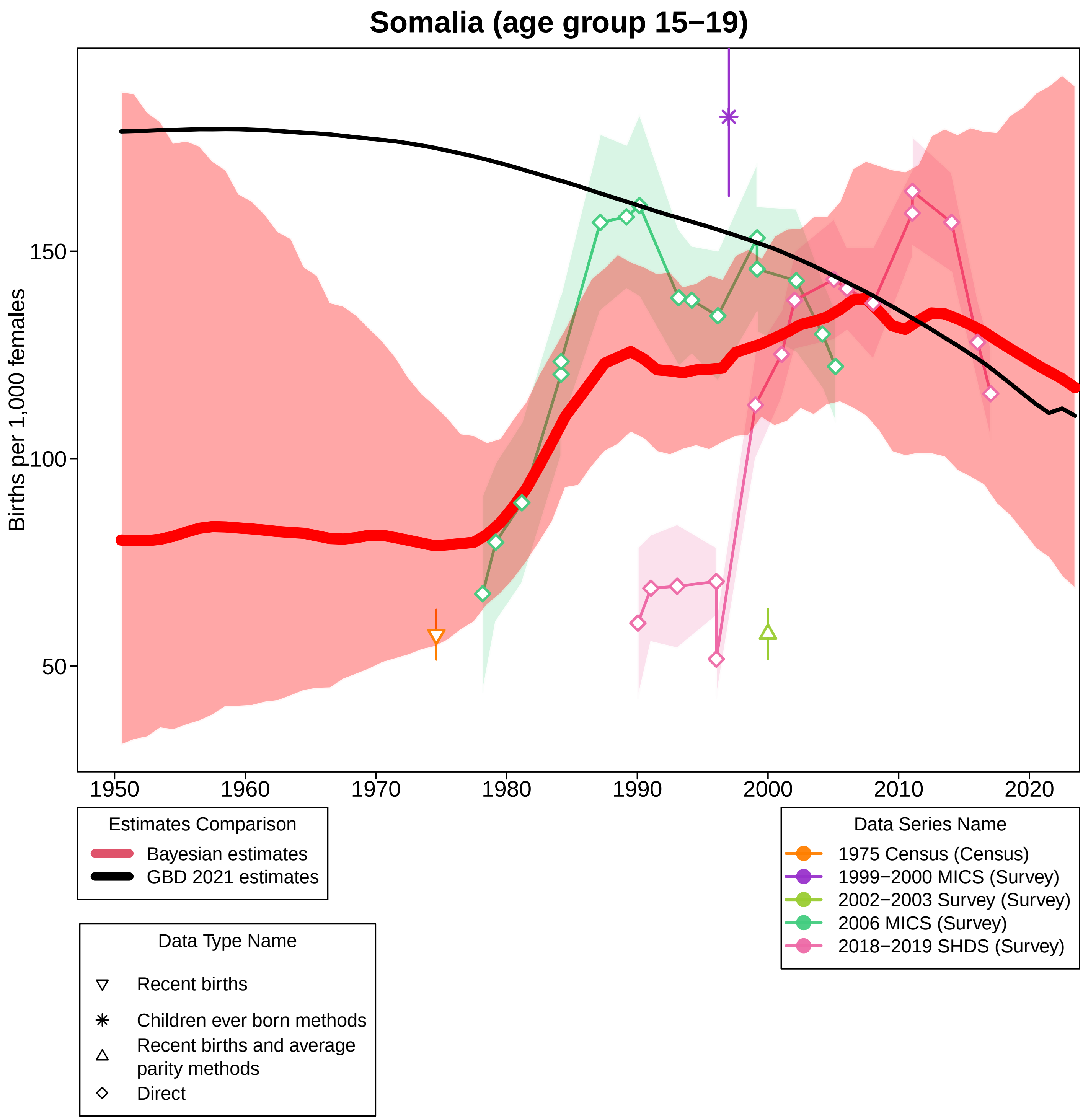
Somalia (age group 15−19)
Births per 1,000 females
150
100
50
1950
1960
1970
1980
1990
2000
2010
2020
Estimates Comparison
Bayesian estimates
GBD 2021 estimates
Data Series Name
1975 Census (Census)
1999−2000 MICS (Survey)
2002−2003 Survey (Survey)
2006 MICS (Survey)
2018−2019 SHDS (Survey)
Data Type Name
Recent births
Children ever born methods
Recent births and average parity methods
Direct

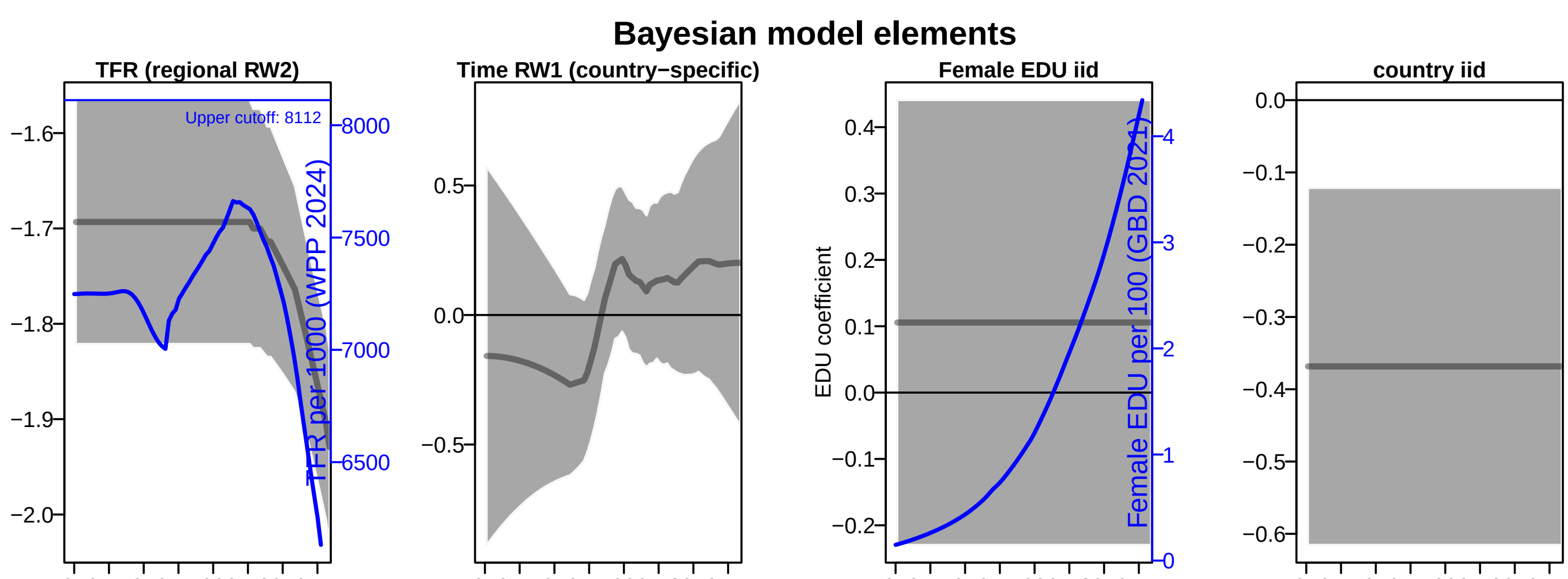
Bayesian model elements
TFR (regional RW2)
Upper cutoff: 8112
TFR per 1000 (WPP 2024)
Time RW1 (country−specific)
Female EDU iid
EDU coefficient
Female EDU per 100 (GBD 2021)
country iid

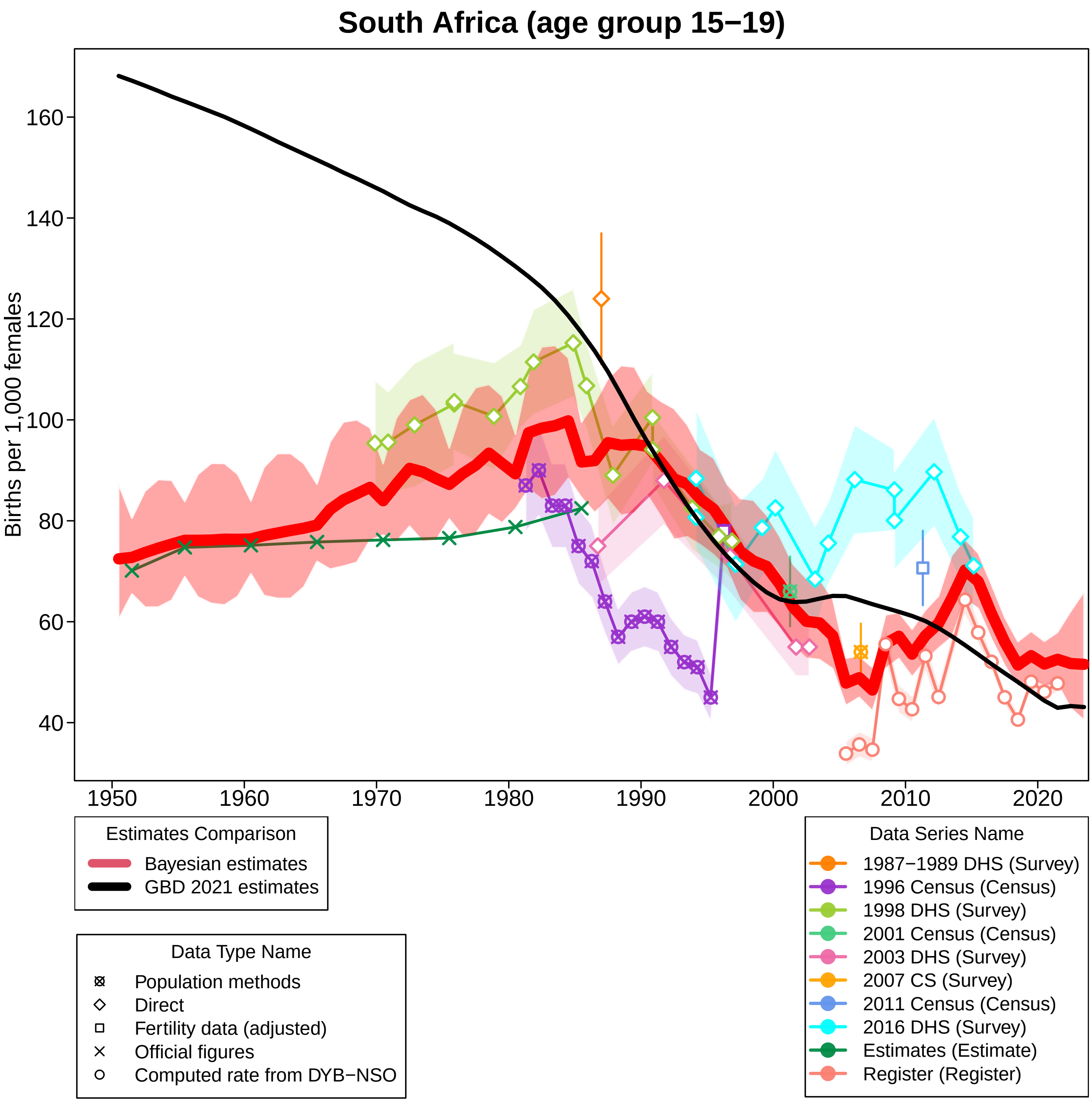
South Africa (age group 15−19)
Births per 1,000 females
160
140
120
100
80
60
40
1950
1960
1970
1980
1990
2000
2010
2020
Estimates Comparison
Bayesian estimates
GBD 2021 estimates
Data Type Name
Population methods
Direct
Fertility data (adjusted)
Official figures
Computed rate from DYB−NSO
Data Series Name
1987−1989 DHS (Survey)
1996 Census (Census)
1998 DHS (Survey)
2001 Census (Census)
2003 DHS (Survey)
2007 CS (Survey)
2011 Census (Census)
2016 DHS (Survey)
Estimates (Estimate)
Register (Register)

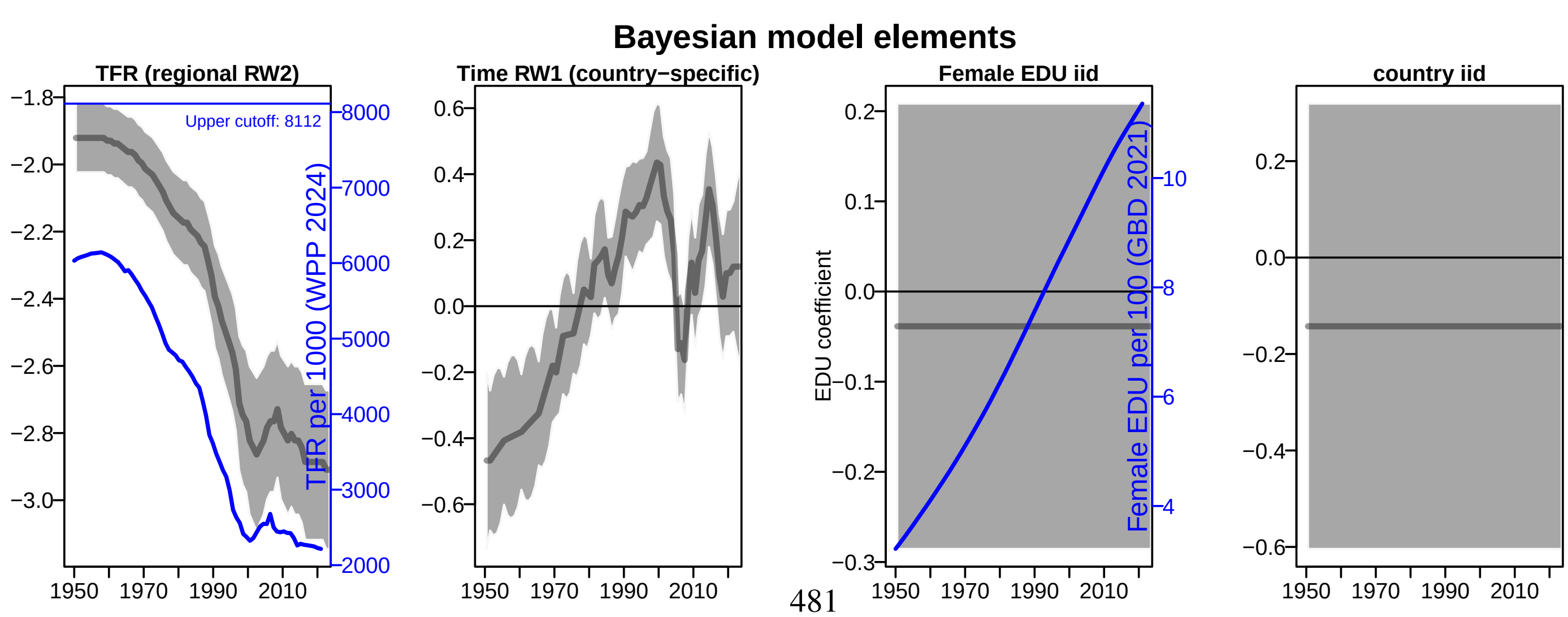
Bayesian model elements
TFR (regional RW2)
Upper cutoff: 8112
TFR per 1000 (WPP 2024)
Time RW1 (country−specific)
Female EDU iid
EDU coefficient
Female EDU per 100 (GBD 2021)
country iid
1950
1970
1990
2010

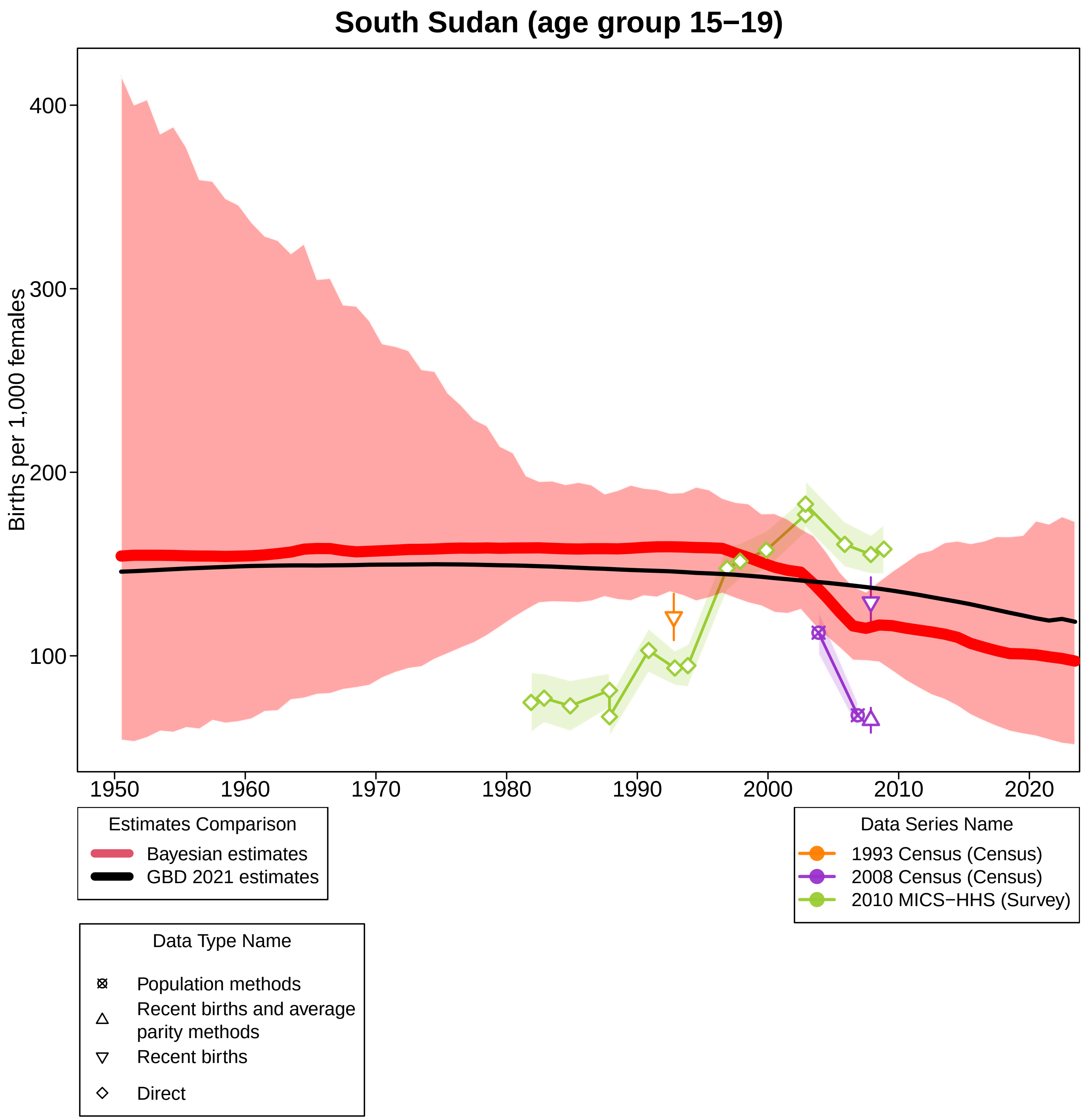
South Sudan (age group 15−19)
Births per 1,000 females
400
300
200
100
1950
1960
1970
1980
1990
2000
2010
2020
Estimates Comparison
Bayesian estimates
GBD 2021 estimates
Data Series Name
1993 Census (Census)
2008 Census (Census)
2010 MICS−HHS (Survey)
Data Type Name
Population methods
Recent births and average parity methods
Recent births
Direct

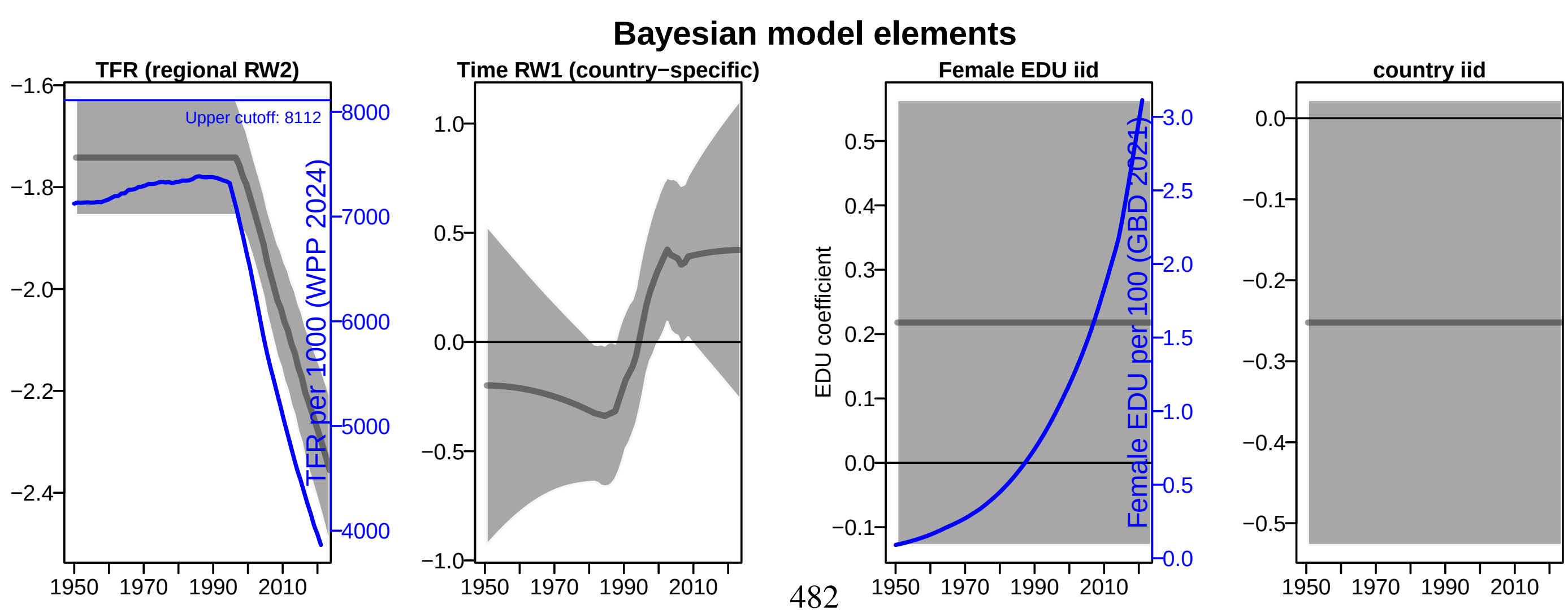
Bayesian model elements
TFR (regional RW2)
Upper cutoff: 8112
TFR per 1000 (WPP 2024)
Time RW1 (country−specific)
Female EDU iid
EDU coefficient
Female EDU per 100 (GBD 2021)
country iid

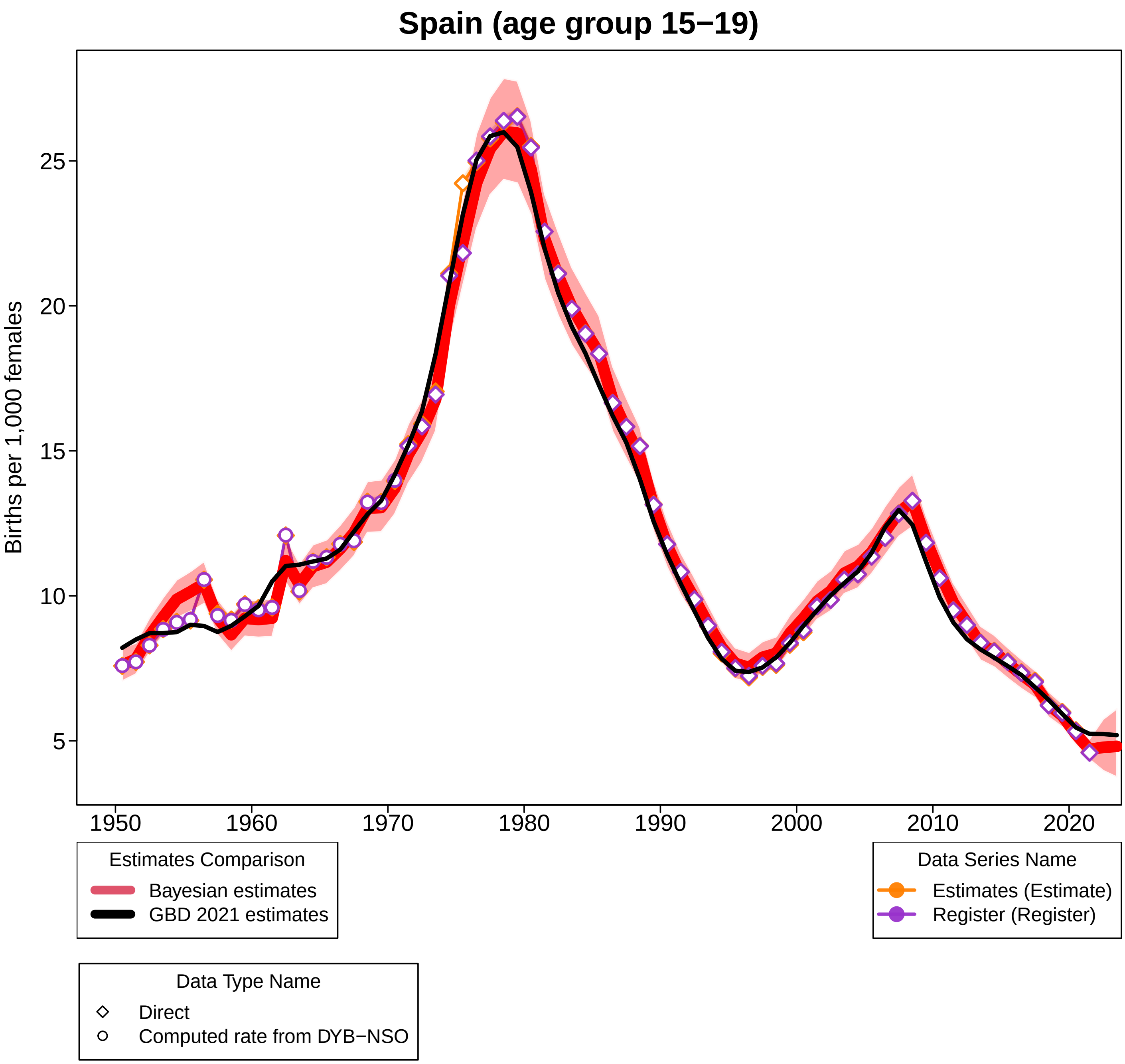
Spain (age group 15−19)
Births per 1,000 females
5
10
15
20
25
1950
1960
1970
1980
1990
2000
2010
2020
Estimates Comparison
Bayesian estimates
GBD 2021 estimates
Data Series Name
Estimates (Estimate)
Register (Register)
Data Type Name
Direct
Computed rate from DYB−NSO

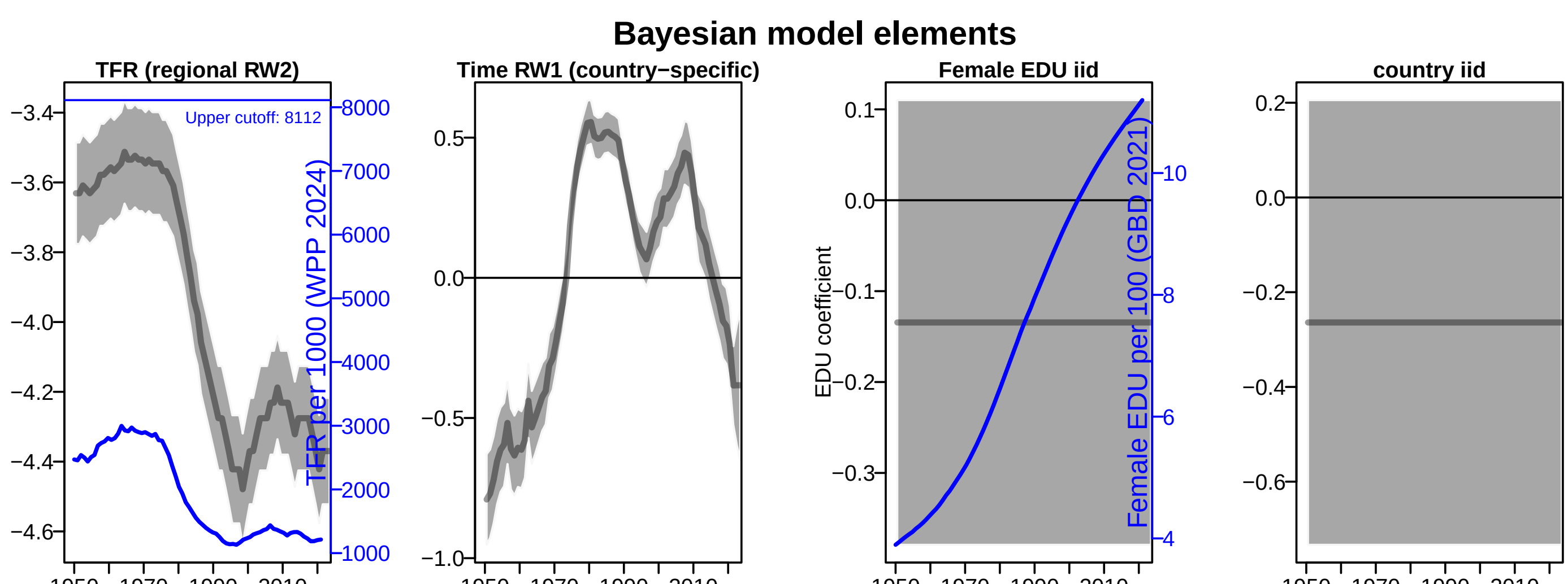
Bayesian model elements
TFR (regional RW2)
Upper cutoff: 8112
TFR per 1000 (WPP 2024)
Time RW1 (country−specific)
Female EDU iid
EDU coefficient
Female EDU per 100 (GBD 2021)
country iid

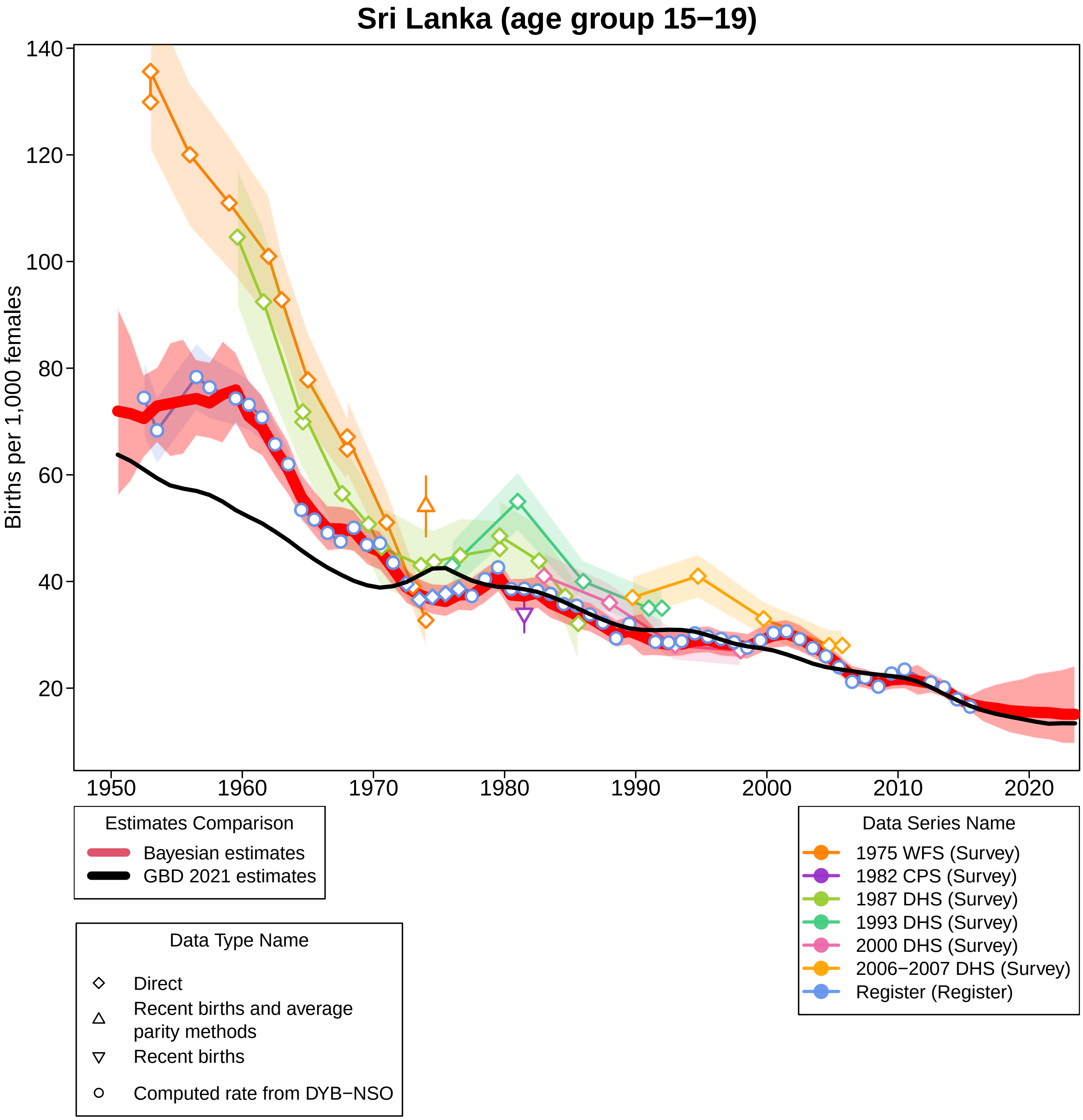
Sri Lanka (age group 15−19)
Births per 1,000 females
Estimates Comparison
Bayesian estimates
GBD 2021 estimates
Data Type Name
Direct
Recent births and average parity methods
Recent births
Computed rate from DYB−NSO
Data Series Name
1975 WFS (Survey)
1982 CPS (Survey)
1987 DHS (Survey)
1993 DHS (Survey)
2000 DHS (Survey)
2006−2007 DHS (Survey)
Register (Register)

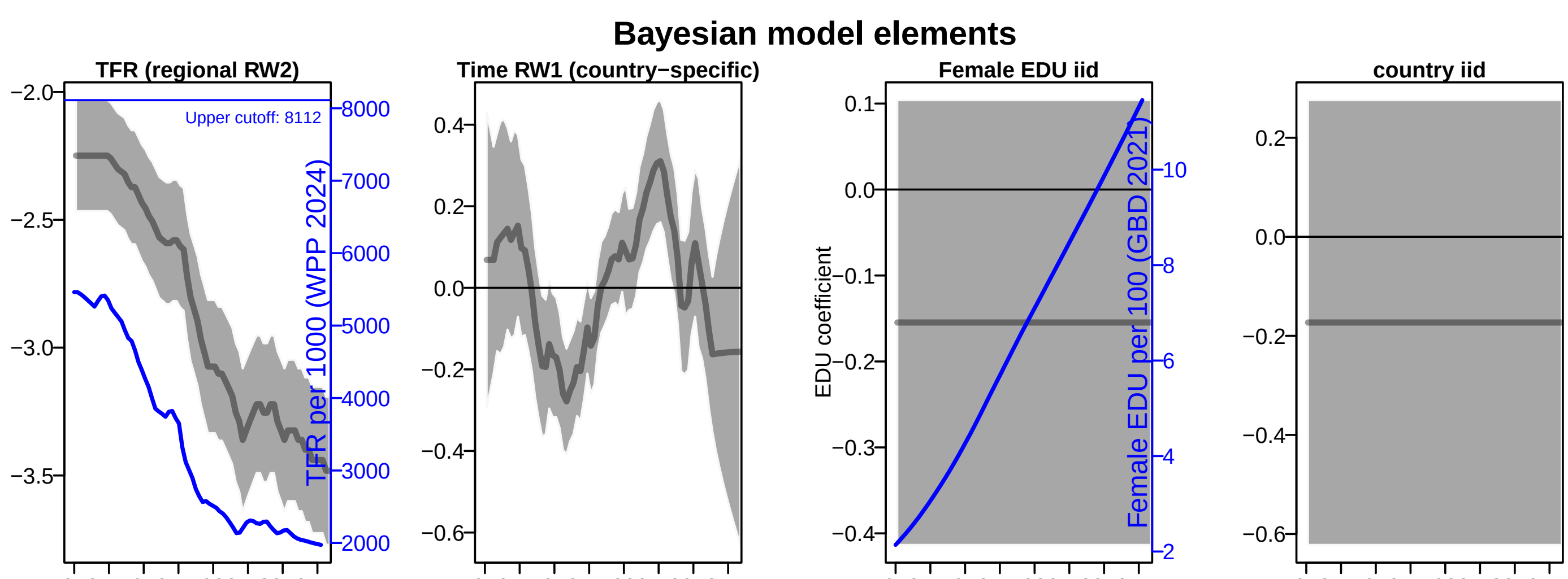
Bayesian model elements
TFR (regional RW2)
Upper cutoff: 8112
TFR per 1000 (WPP 2024)
Time RW1 (country−specific)
Female EDU iid
EDU coefficient
Female EDU per 100 (GBD 2021)
country iid

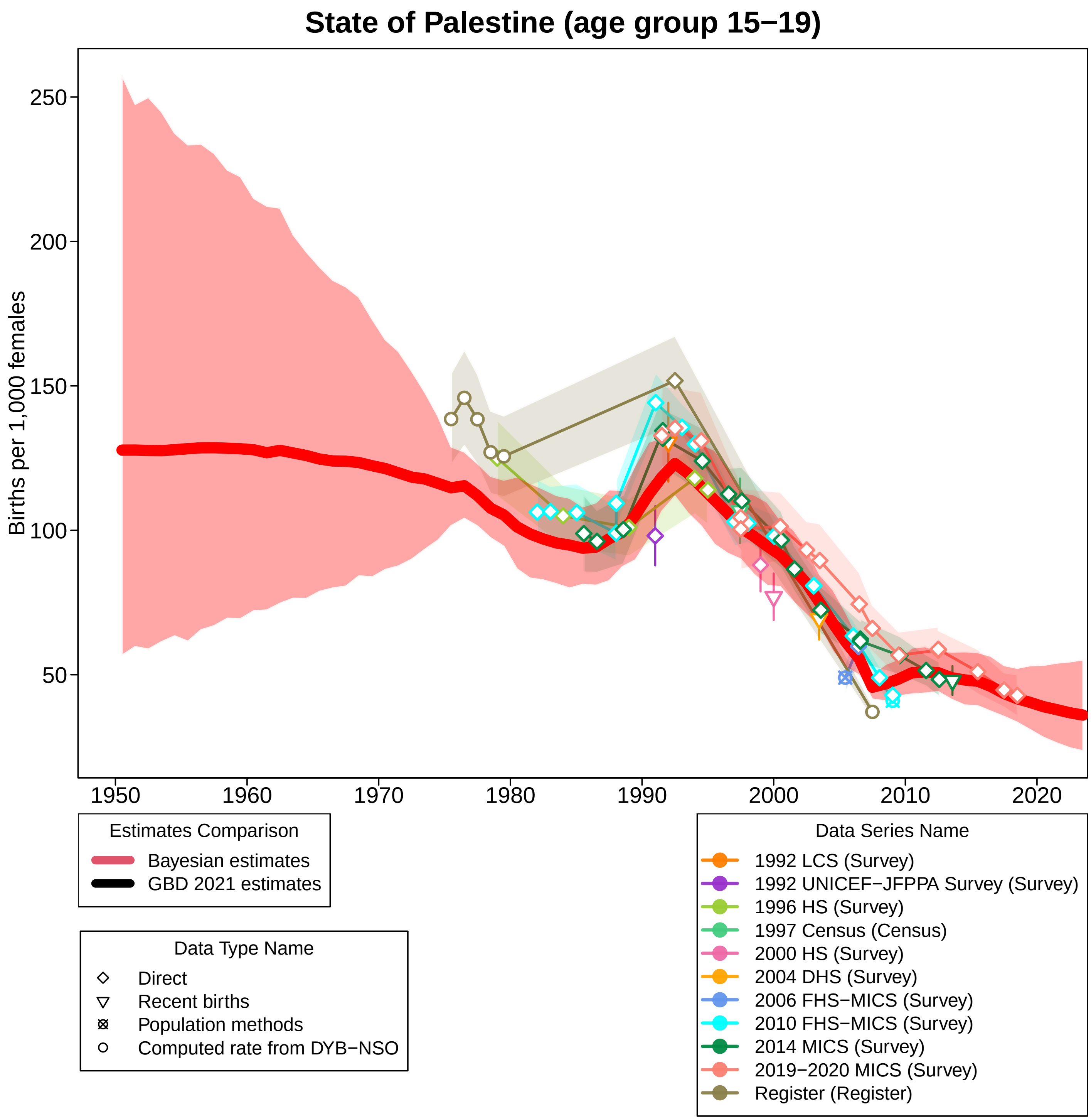

State of Palestine (age group 15−19)
Births per 1,000 females
250
200
150
100
50
1950
1960
1970
1980
1990
2000
2010
2020
Estimates Comparison
Bayesian estimates
GBD 2021 estimates
Data Type Name
Direct
Recent births
Population methods
Computed rate from DYB−NSO
Data Series Name
1992 LCS (Survey)
1992 UNICEF−JFPPA Survey (Survey)
1996 HS (Survey)
1997 Census (Census)
2000 HS (Survey)
2004 DHS (Survey)
2006 FHS−MICS (Survey)
2010 FHS−MICS (Survey)
2014 MICS (Survey)
2019−2020 MICS (Survey)
Register (Register)

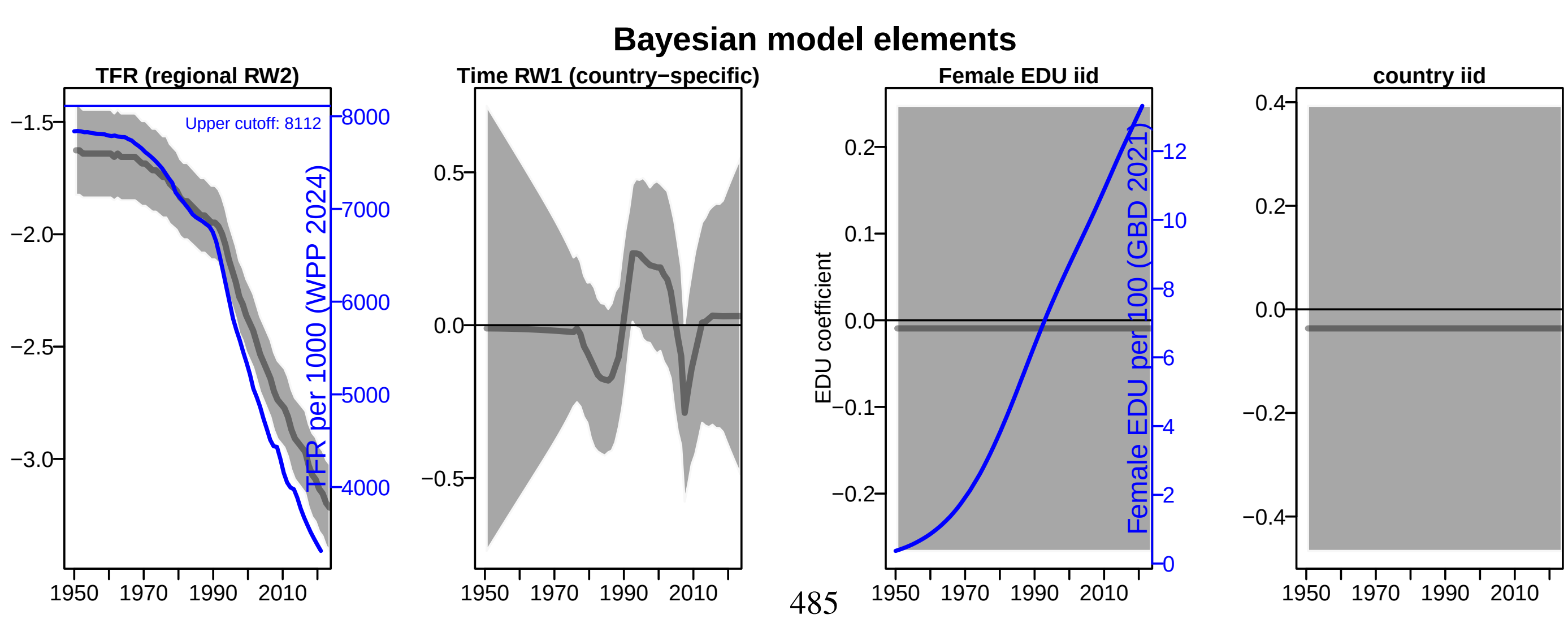

Bayesian model elements
TFR (regional RW2)
Upper cutoff: 8112
TFR per 1000 (WPP 2024)
Time RW1 (country−specific)
Female EDU iid
EDU coefficient
Female EDU per 100 (GBD 2021)
country iid

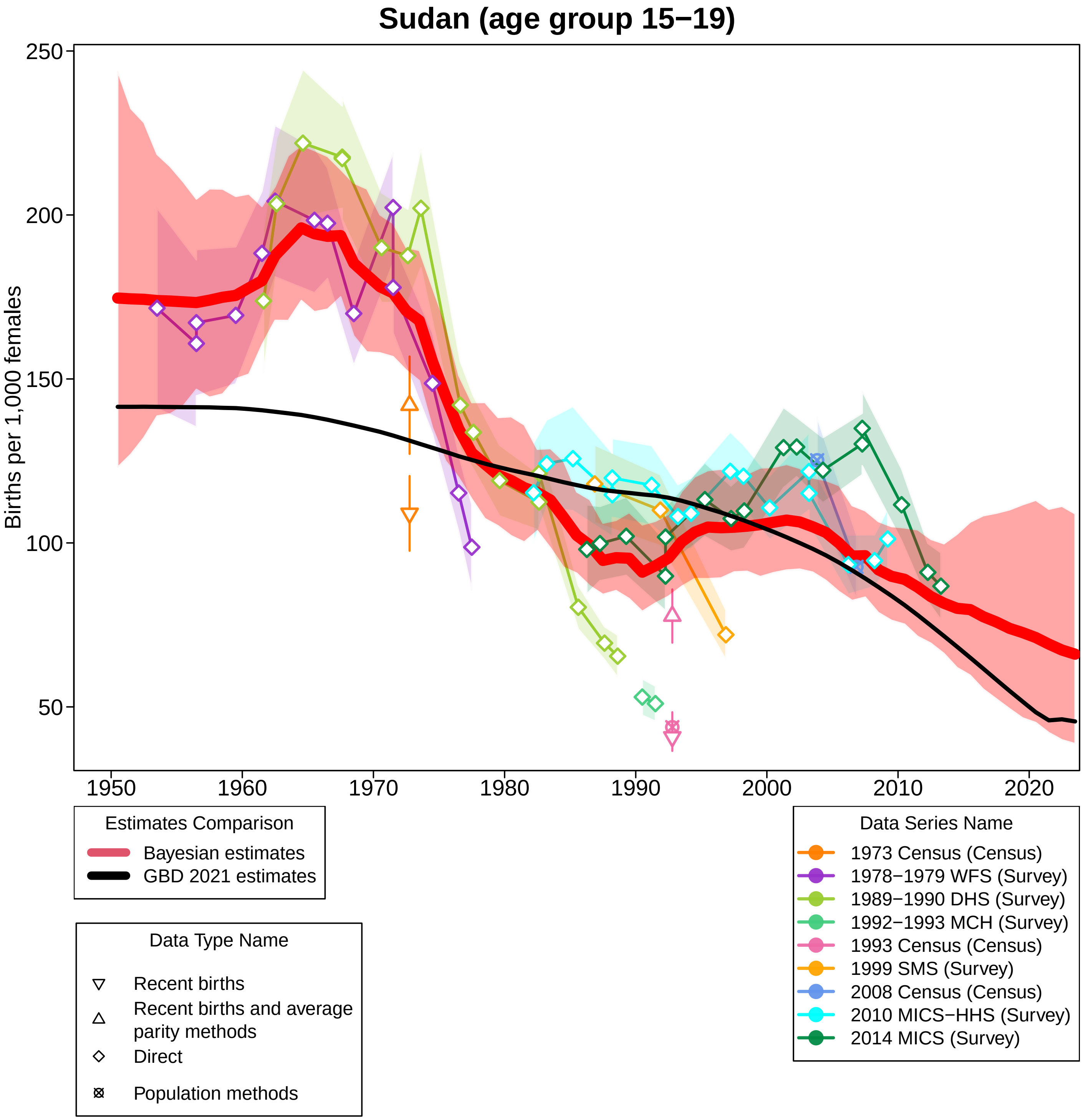
Sudan (age group 15−19)
Births per 1,000 females
250
200
150
100
50
1950
1960
1970
1980
1990
2000
2010
2020
Estimates Comparison
Bayesian estimates
GBD 2021 estimates
Data Type Name
Recent births
Recent births and average parity methods
Direct
Population methods
Data Series Name
1973 Census (Census)
1978−1979 WFS (Survey)
1989−1990 DHS (Survey)
1992−1993 MCH (Survey)
1993 Census (Census)
1999 SMS (Survey)
2008 Census (Census)
2010 MICS−HHS (Survey)
2014 MICS (Survey)

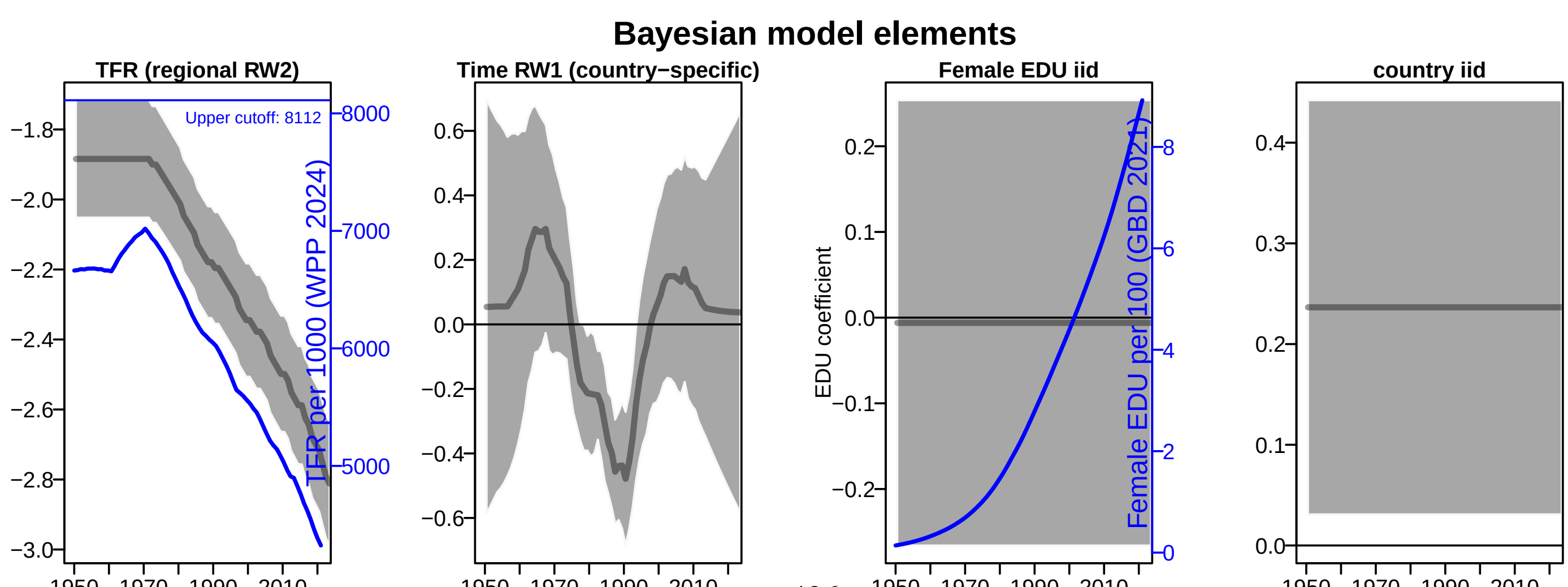
Bayesian model elements
TFR (regional RW2)
Upper cutoff: 8112
TFR per 1000 (WPP 2024)
Time RW1 (country−specific)
Female EDU iid
EDU coefficient
Female EDU per 100 (GBD 2021)
country iid
1950
1970
1990
2010

Suriname (age group 15−19)

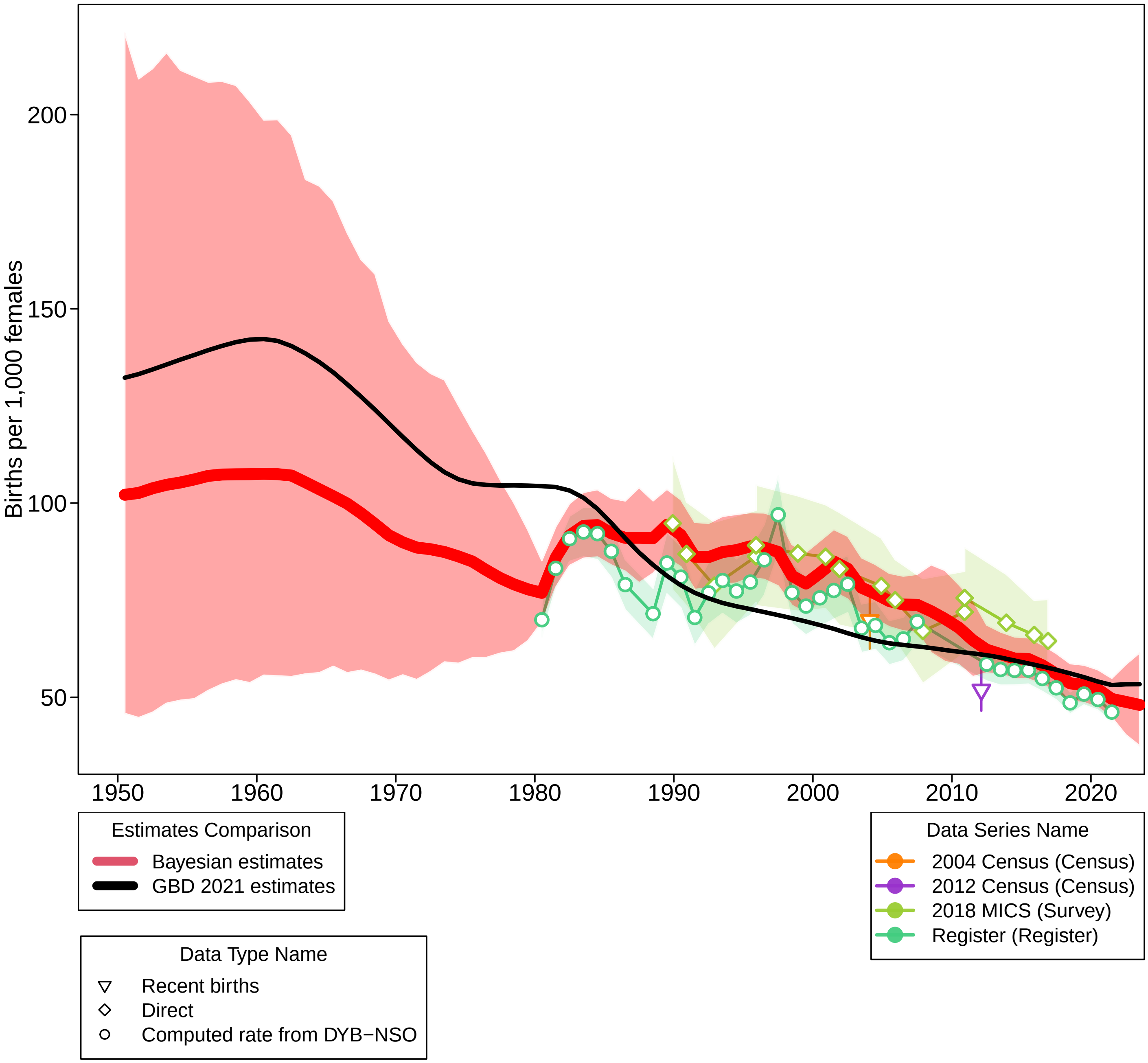

Bayesian model elements

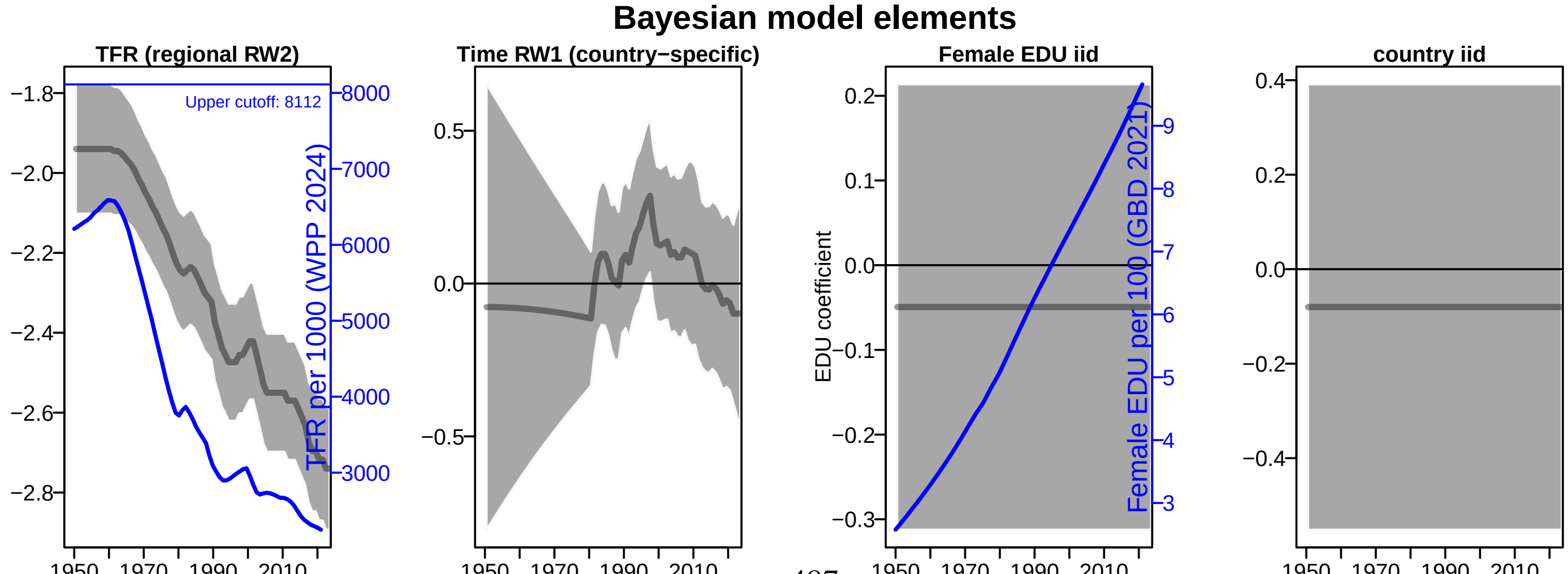

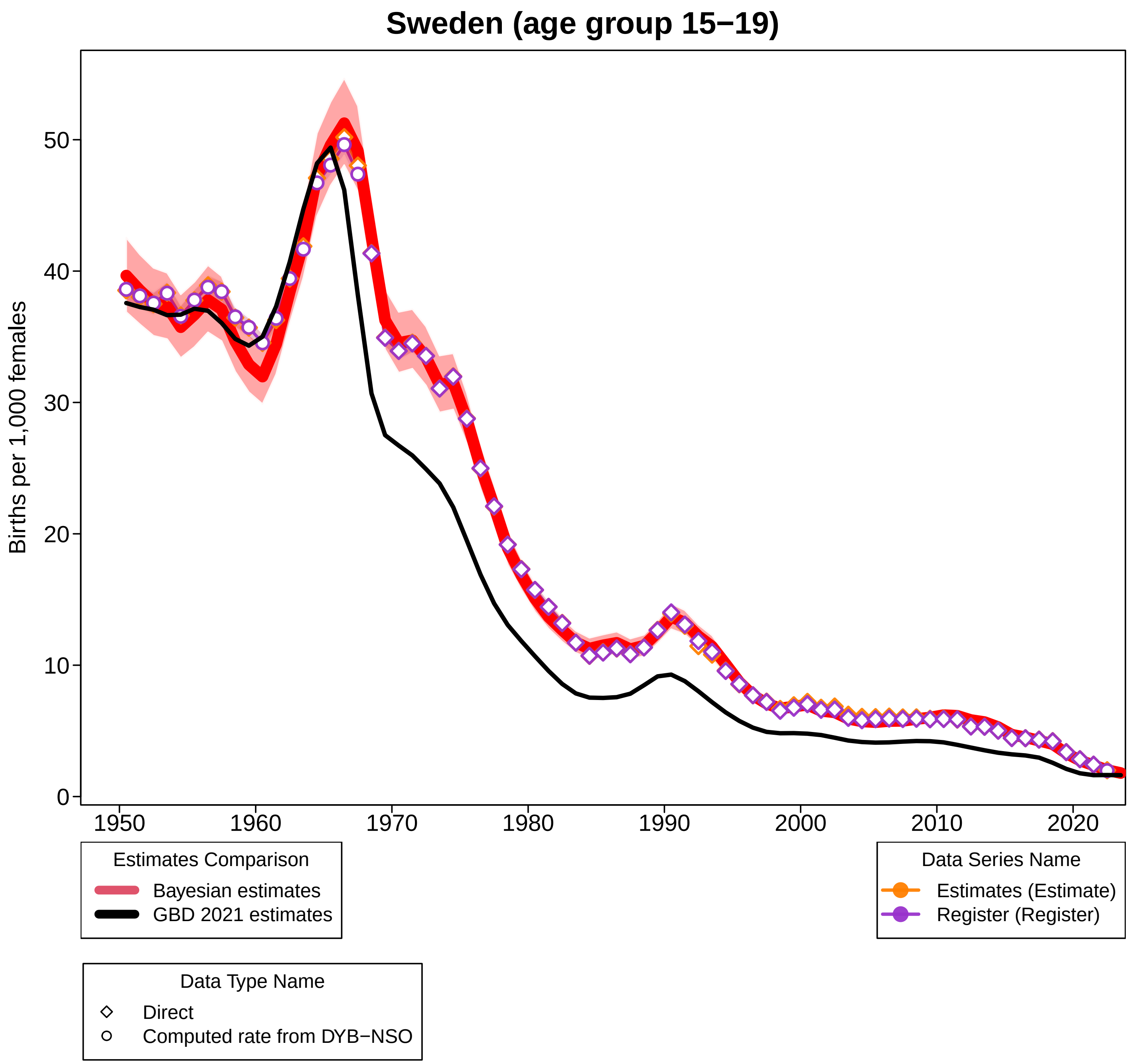
Sweden (age group 15−19)
Births per 1,000 females
0
10
20
30
40
50
1950
1960
1970
1980
1990
2000
2010
2020
Estimates Comparison
Bayesian estimates
GBD 2021 estimates
Data Series Name
Estimates (Estimate)
Register (Register)
Data Type Name
Direct
Computed rate from DYB−NSO

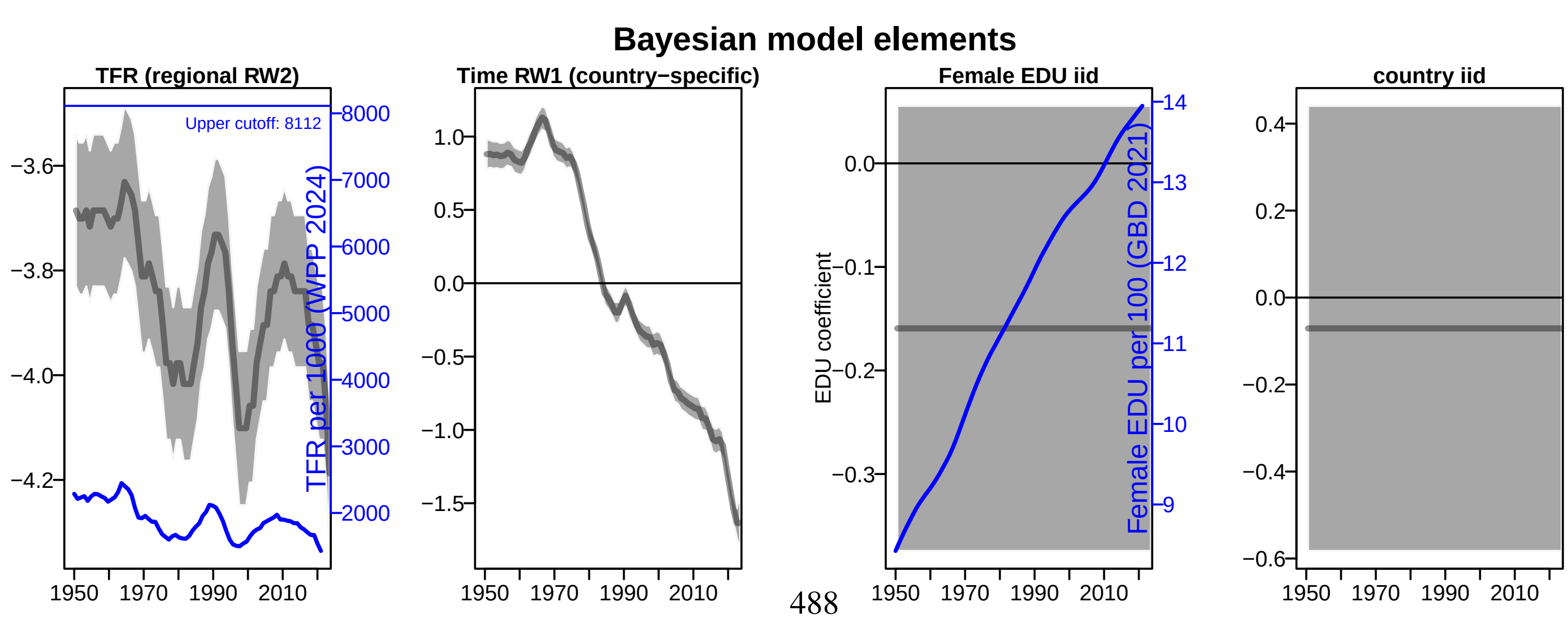
Bayesian model elements
TFR (regional RW2)
Upper cutoff: 8112
TFR per 1000 (WPP 2024)
Time RW1 (country−specific)
Female EDU iid
EDU coefficient
Female EDU per 100 (GBD 2021)
country iid

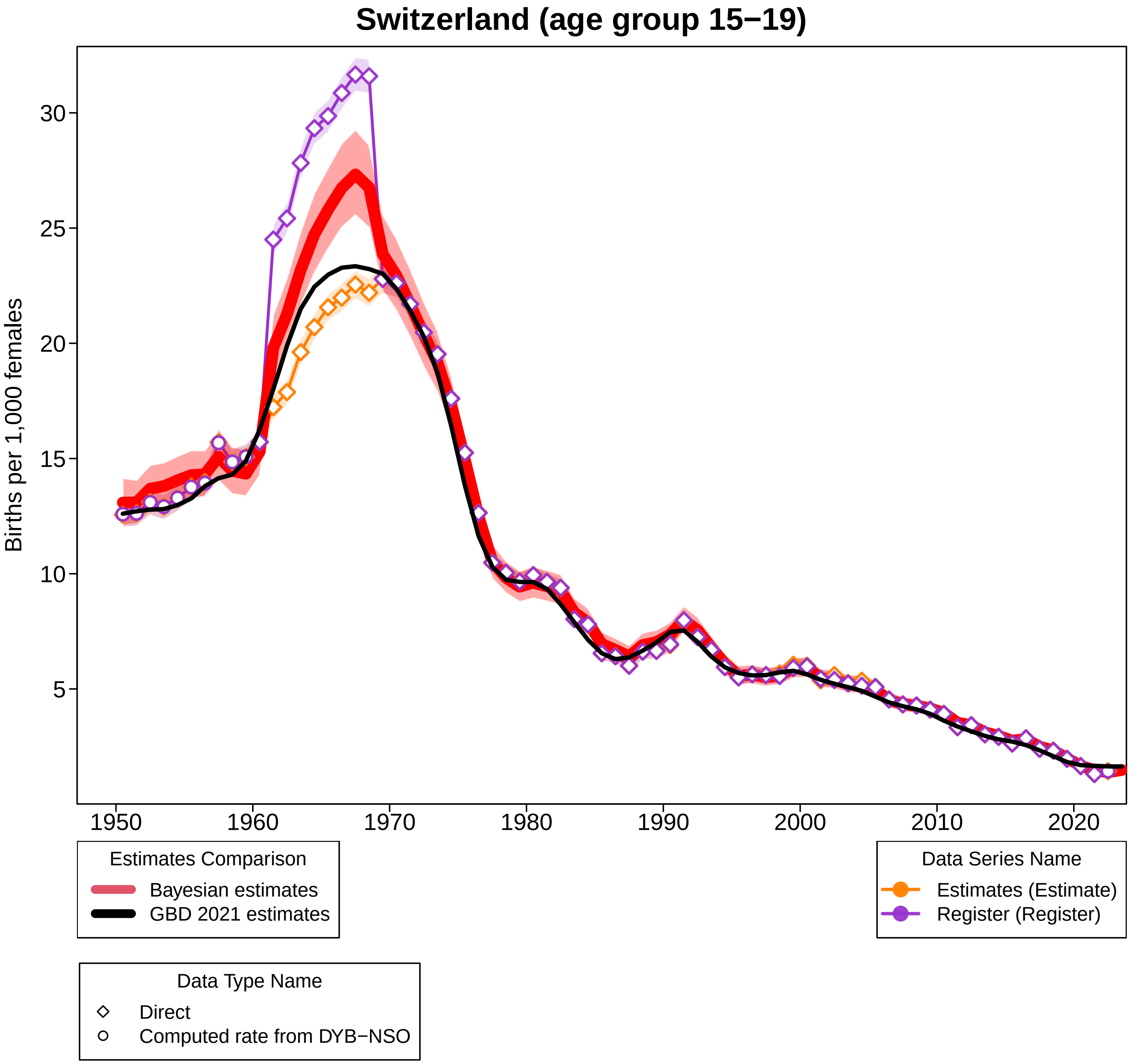
Switzerland (age group 15−19)
Births per 1,000 females
30
25
20
15
10
5
1950
1960
1970
1980
1990
2000
2010
2020
Estimates Comparison
Bayesian estimates
GBD 2021 estimates
Data Series Name
Estimates (Estimate)
Register (Register)
Data Type Name
Direct
Computed rate from DYB−NSO

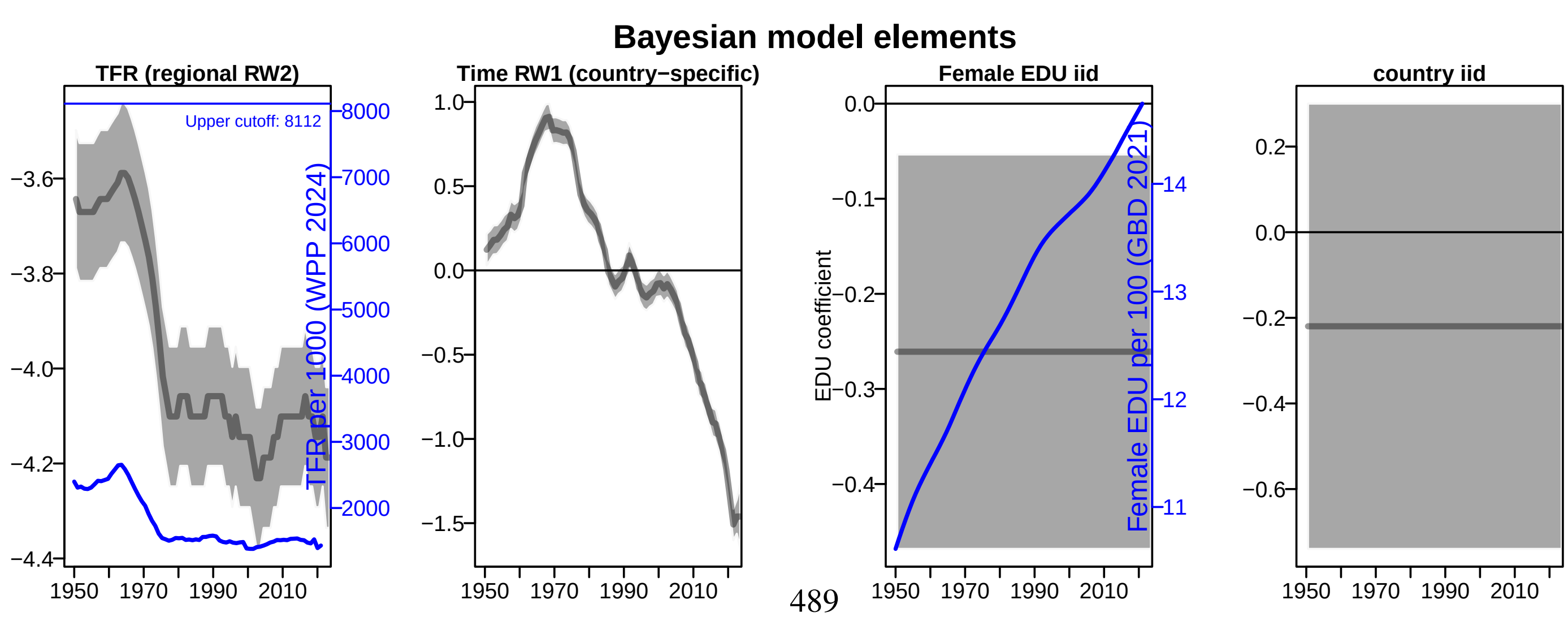
Bayesian model elements
TFR (regional RW2)
Upper cutoff: 8112
TFR per 1000 (WPP 2024)
Time RW1 (country−specific)
Female EDU iid
EDU coefficient
Female EDU per 100 (GBD 2021)
country iid

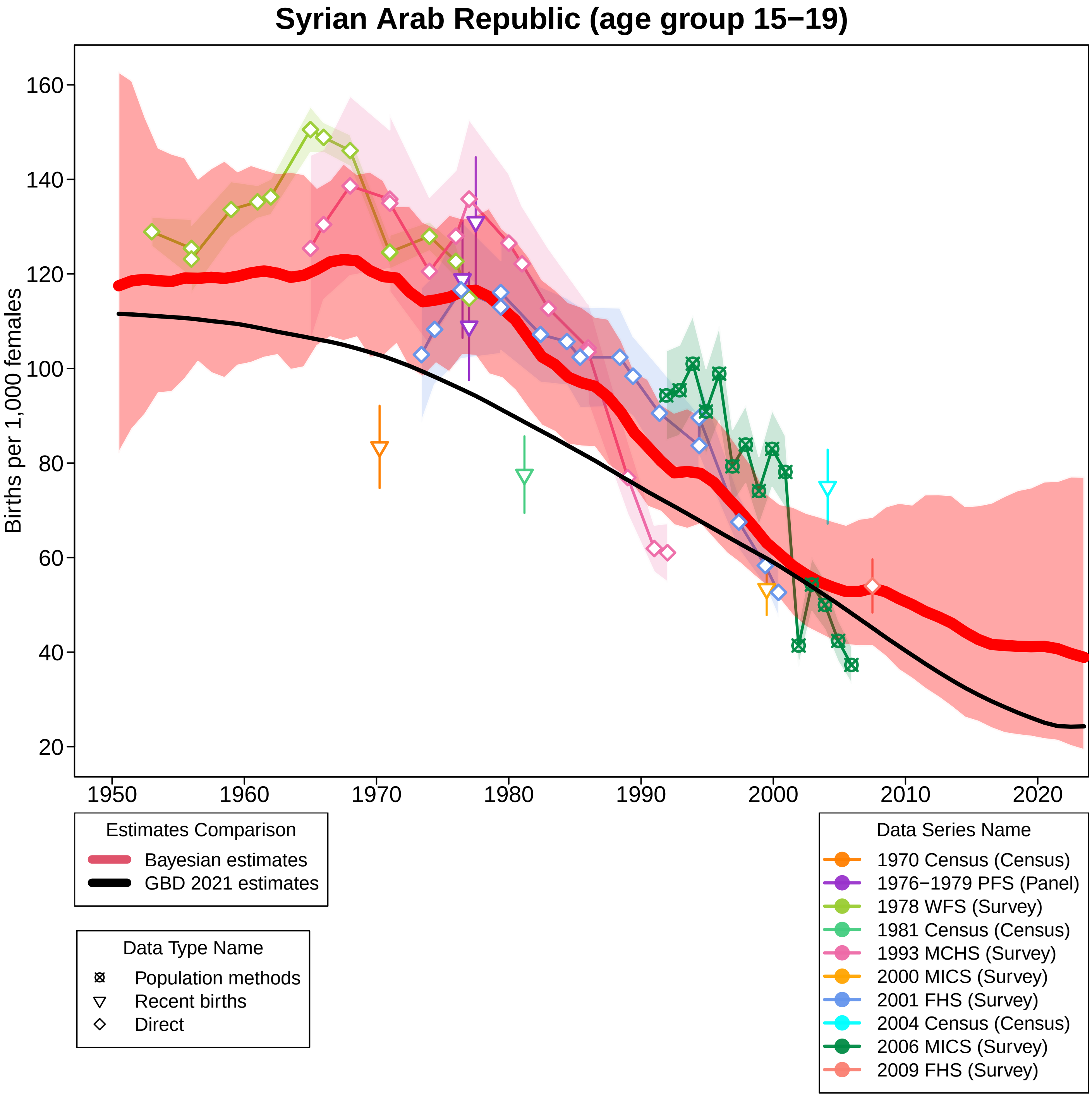
Syrian Arab Republic (age group 15−19)
Births per 1,000 females
160
140
120
100
80
60
40
20
1950
1960
1970
1980
1990
2000
2010
2020
Estimates Comparison
Bayesian estimates
GBD 2021 estimates
Data Type Name
Population methods
Recent births
Direct
Data Series Name
1970 Census (Census)
1976−1979 PFS (Panel)
1978 WFS (Survey)
1981 Census (Census)
1993 MCHS (Survey)
2000 MICS (Survey)
2001 FHS (Survey)
2004 Census (Census)
2006 MICS (Survey)
2009 FHS (Survey)

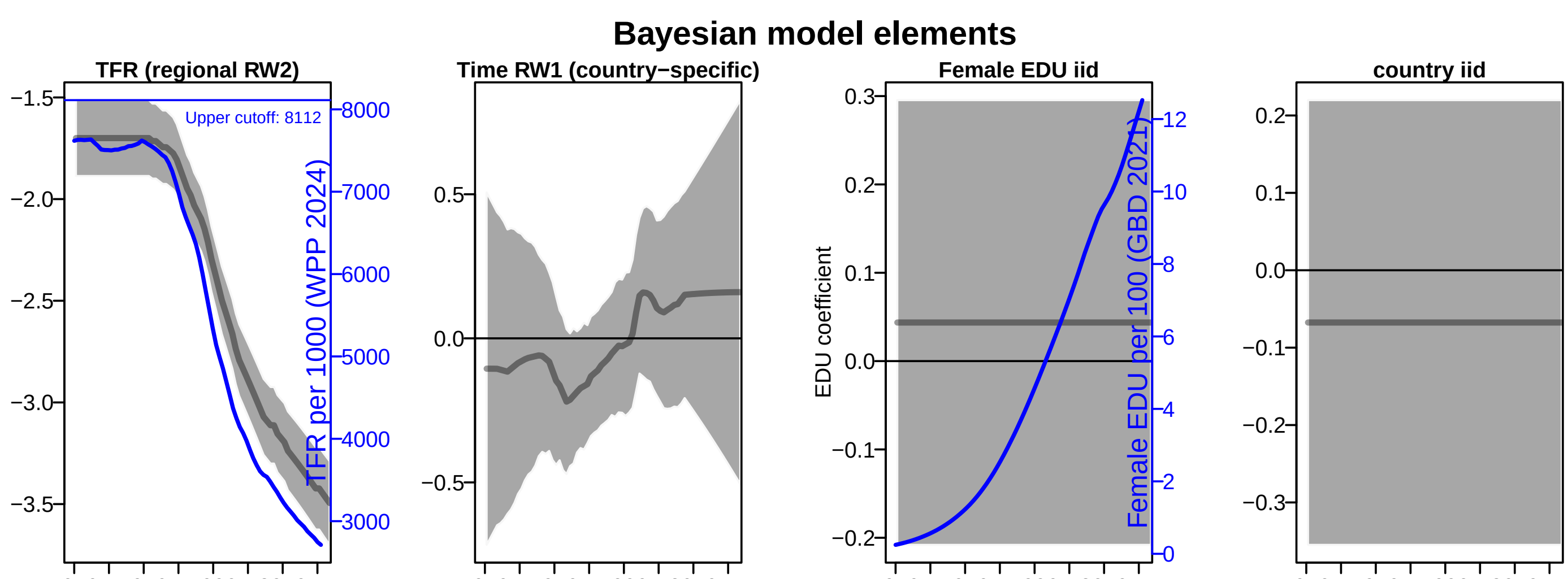
Bayesian model elements
TFR (regional RW2)
Upper cutoff: 8112
TFR per 1000 (WPP 2024)
Time RW1 (country−specific)
Female EDU iid
EDU coefficient
Female EDU per 100 (GBD 2021)
country iid
1950
1970
1990
2010

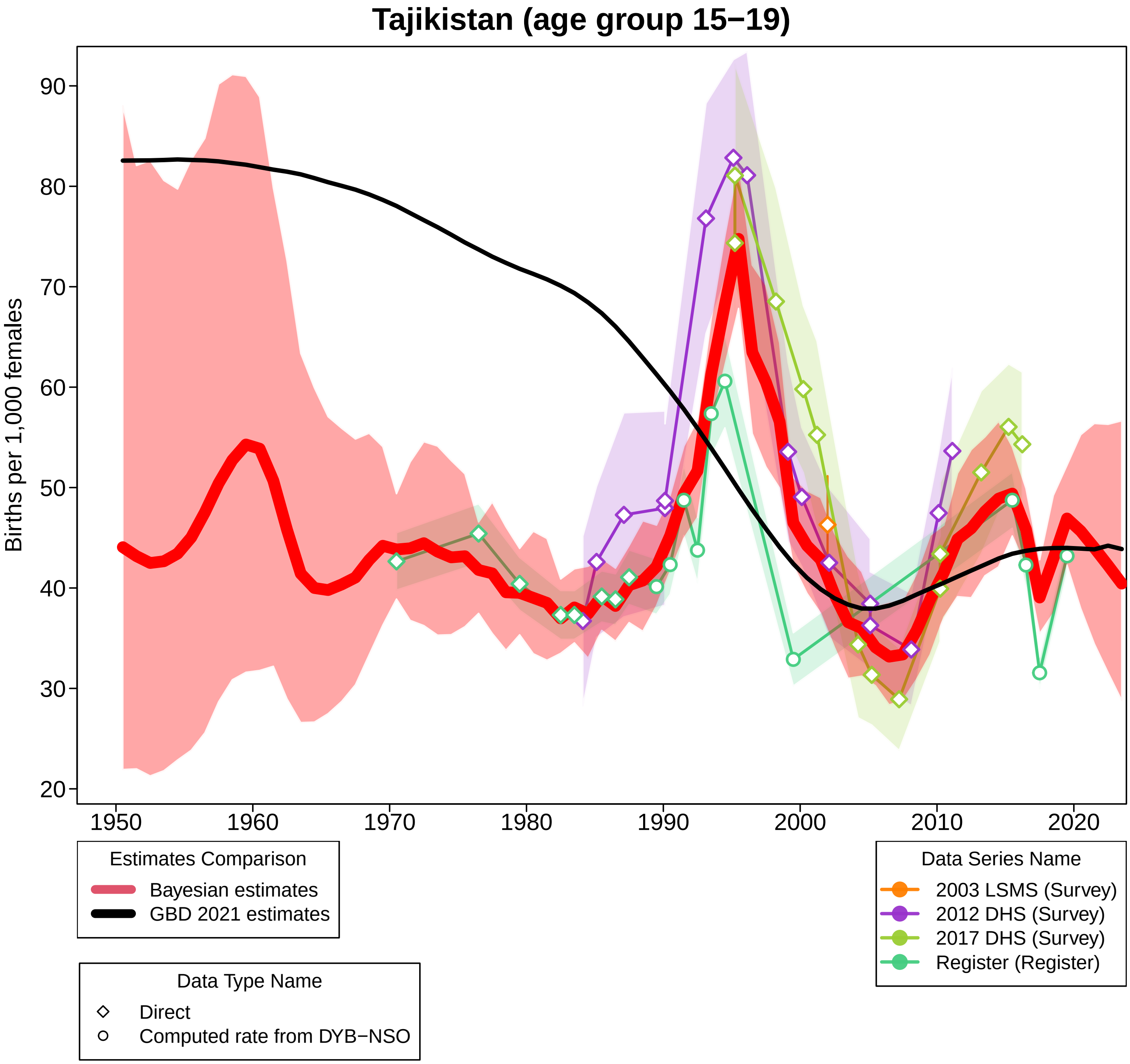
Tajikistan (age group 15−19)
Births per 1,000 females
90
80
70
60
50
40
30
20
1950
1960
1970
1980
1990
2000
2010
2020
Estimates Comparison
Bayesian estimates
GBD 2021 estimates
Data Series Name
2003 LSMS (Survey)
2012 DHS (Survey)
2017 DHS (Survey)
Register (Register)
Data Type Name
Direct
Computed rate from DYB−NSO

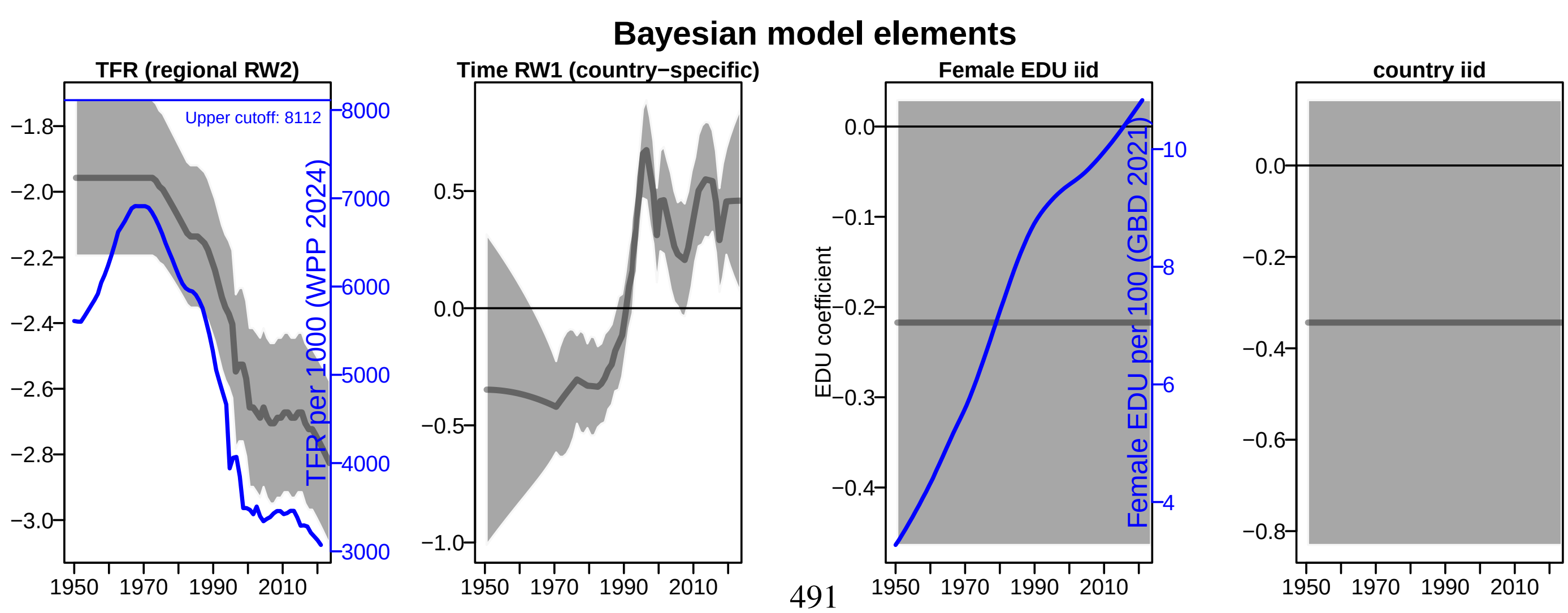
Bayesian model elements
TFR (regional RW2)
Upper cutoff: 8112
TFR per 1000 (WPP 2024)
Time RW1 (country−specific)
Female EDU iid
EDU coefficient
Female EDU per 100 (GBD 2021)
country iid

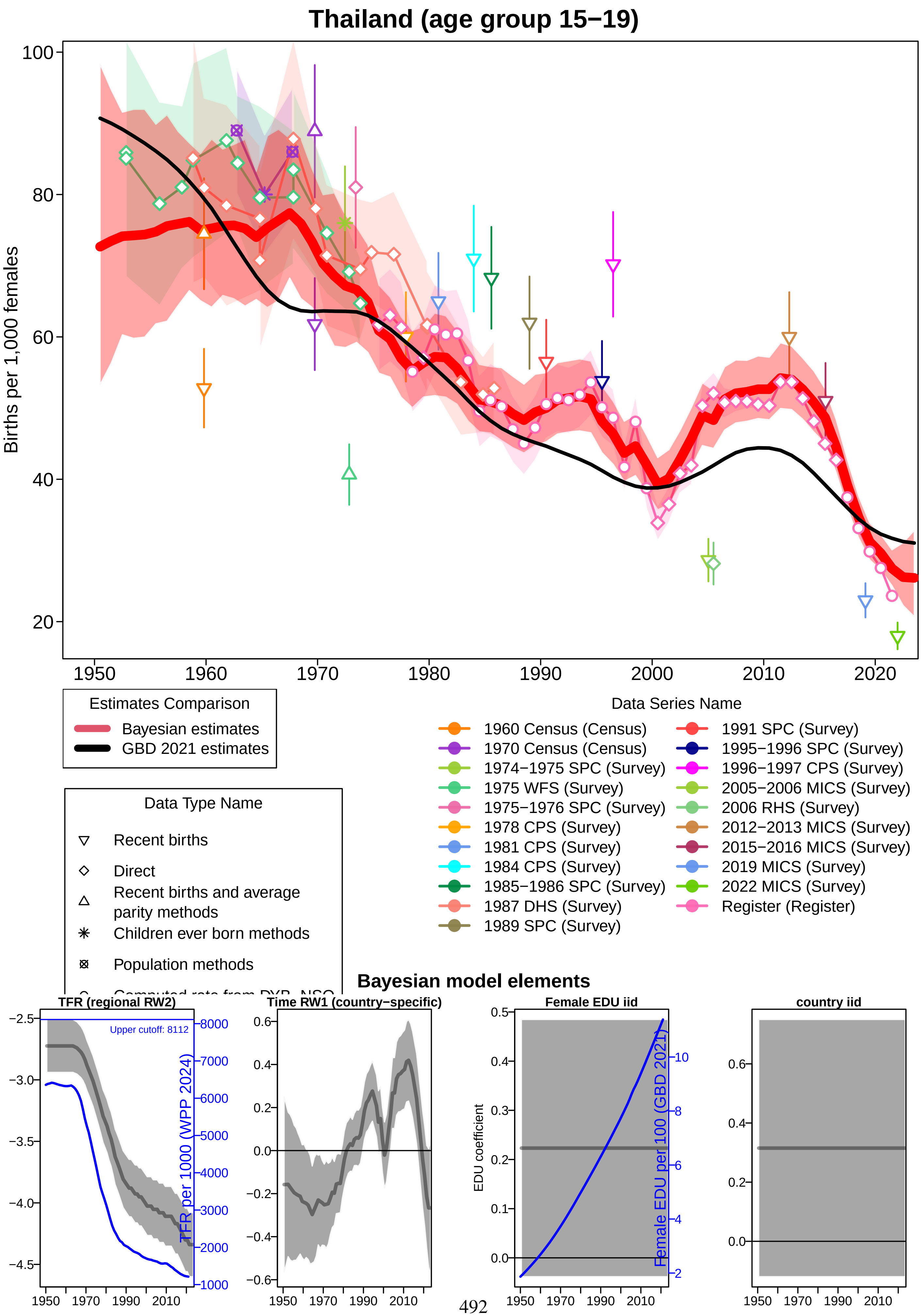
Thailand (age group 15−19)
Births per 1,000 females
100
80
60
40
20
1950
1960
1970
1980
1990
2000
2010
2020
Estimates Comparison
Bayesian estimates
GBD 2021 estimates
Data Series Name
1960 Census (Census)
1970 Census (Census)
1974−1975 SPC (Survey)
1975 WFS (Survey)
1975−1976 SPC (Survey)
1978 CPS (Survey)
1981 CPS (Survey)
1984 CPS (Survey)
1985−1986 SPC (Survey)
1987 DHS (Survey)
1989 SPC (Survey)
1991 SPC (Survey)
1995−1996 SPC (Survey)
1996−1997 CPS (Survey)
2005−2006 MICS (Survey)
2006 RHS (Survey)
2012−2013 MICS (Survey)
2015−2016 MICS (Survey)
2019 MICS (Survey)
2022 MICS (Survey)
Register (Register)
Data Type Name
Recent births
Direct
Recent births and average parity methods
Children ever born methods
Population methods
Bayesian model elements
TFR (regional RW2)
Upper cutoff: 8112
TFR per 1000 (WPP 2024)
Time RW1 (country−specific)
Female EDU iid
EDU coefficient
Female EDU per 100 (GBD 2021)
country iid

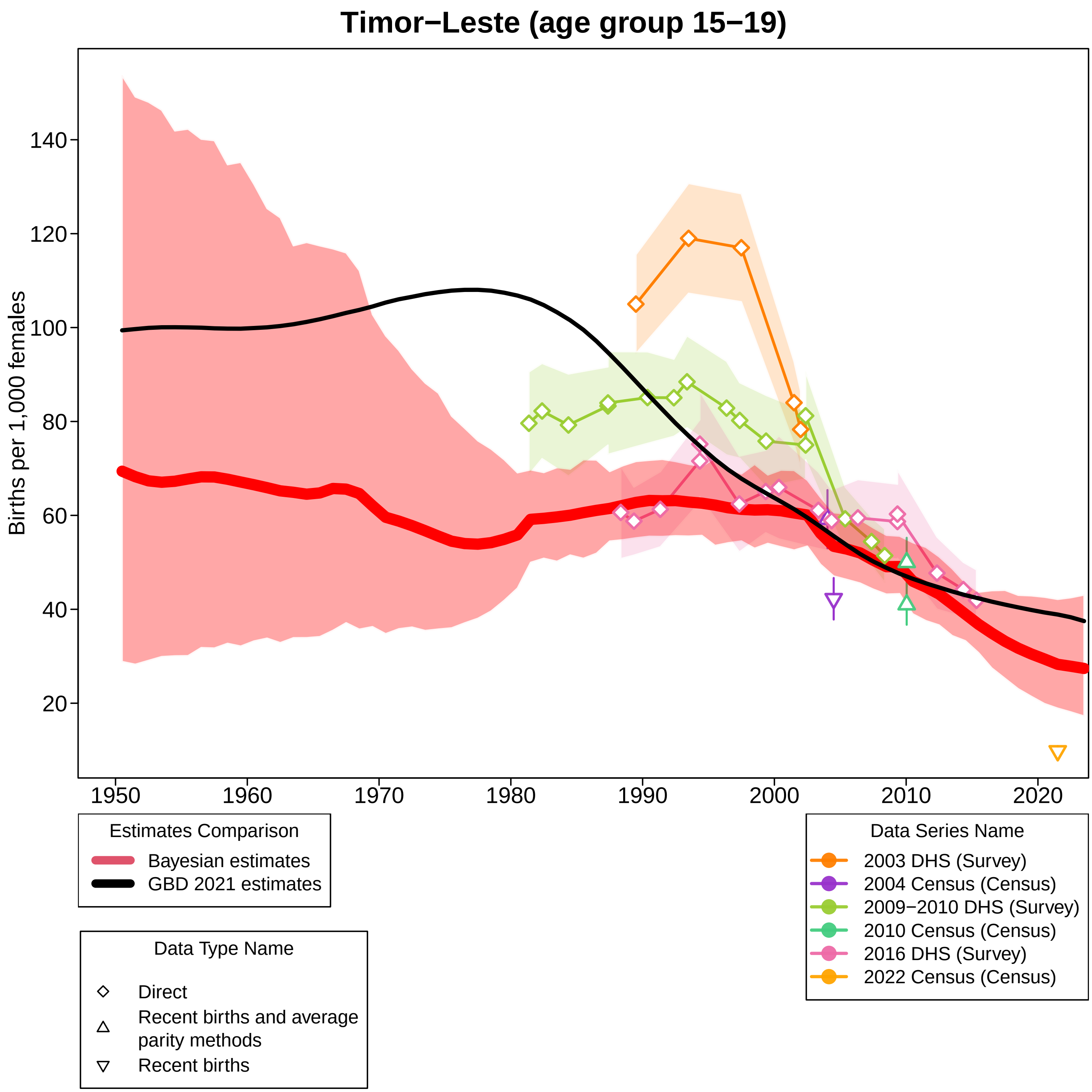
Timor−Leste (age group 15−19)
Births per 1,000 females
140
120
100
80
60
40
20
1950
1960
1970
1980
1990
2000
2010
2020
Estimates Comparison
Bayesian estimates
GBD 2021 estimates
Data Series Name
2003 DHS (Survey)
2004 Census (Census)
2009−2010 DHS (Survey)
2010 Census (Census)
2016 DHS (Survey)
2022 Census (Census)
Data Type Name
Direct
Recent births and average parity methods
Recent births

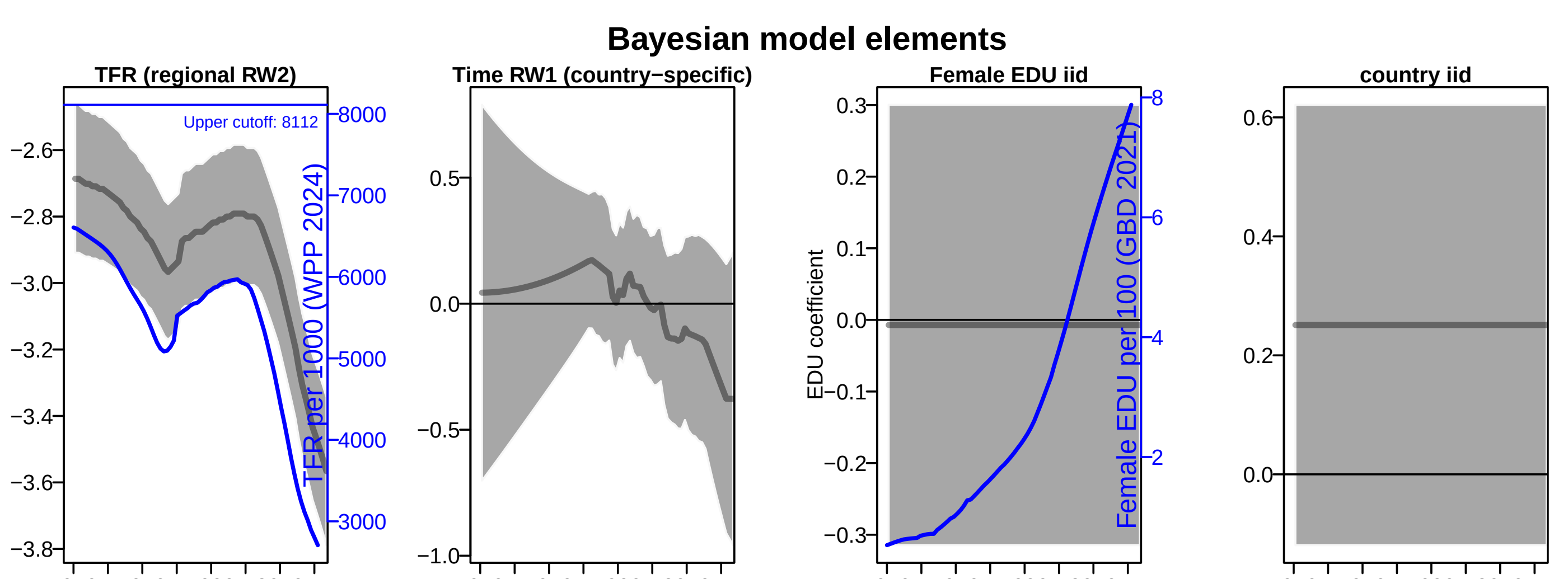
Bayesian model elements
TFR (regional RW2)
Upper cutoff: 8112
TFR per 1000 (WPP 2024)
Time RW1 (country−specific)
Female EDU iid
EDU coefficient
Female EDU per 100 (GBD 2021)
country iid

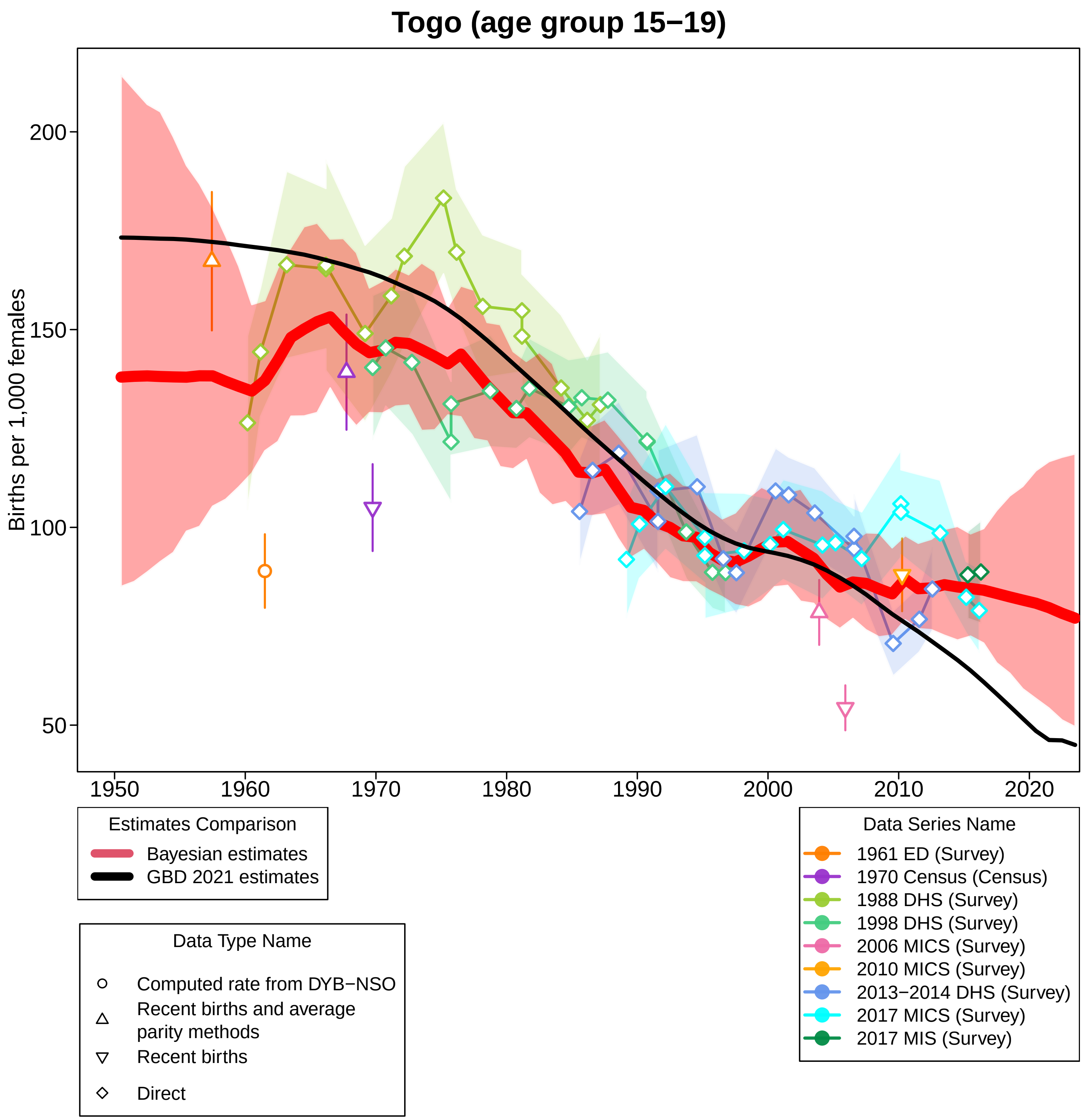
Togo (age group 15−19)
Births per 1,000 females
200
150
100
50
1950
1960
1970
1980
1990
2000
2010
2020
Estimates Comparison
Bayesian estimates
GBD 2021 estimates
Data Type Name
Computed rate from DYB−NSO
Recent births and average parity methods
Recent births
Direct
Data Series Name
1961 ED (Survey)
1970 Census (Census)
1988 DHS (Survey)
1998 DHS (Survey)
2006 MICS (Survey)
2010 MICS (Survey)
2013−2014 DHS (Survey)
2017 MICS (Survey)
2017 MIS (Survey)

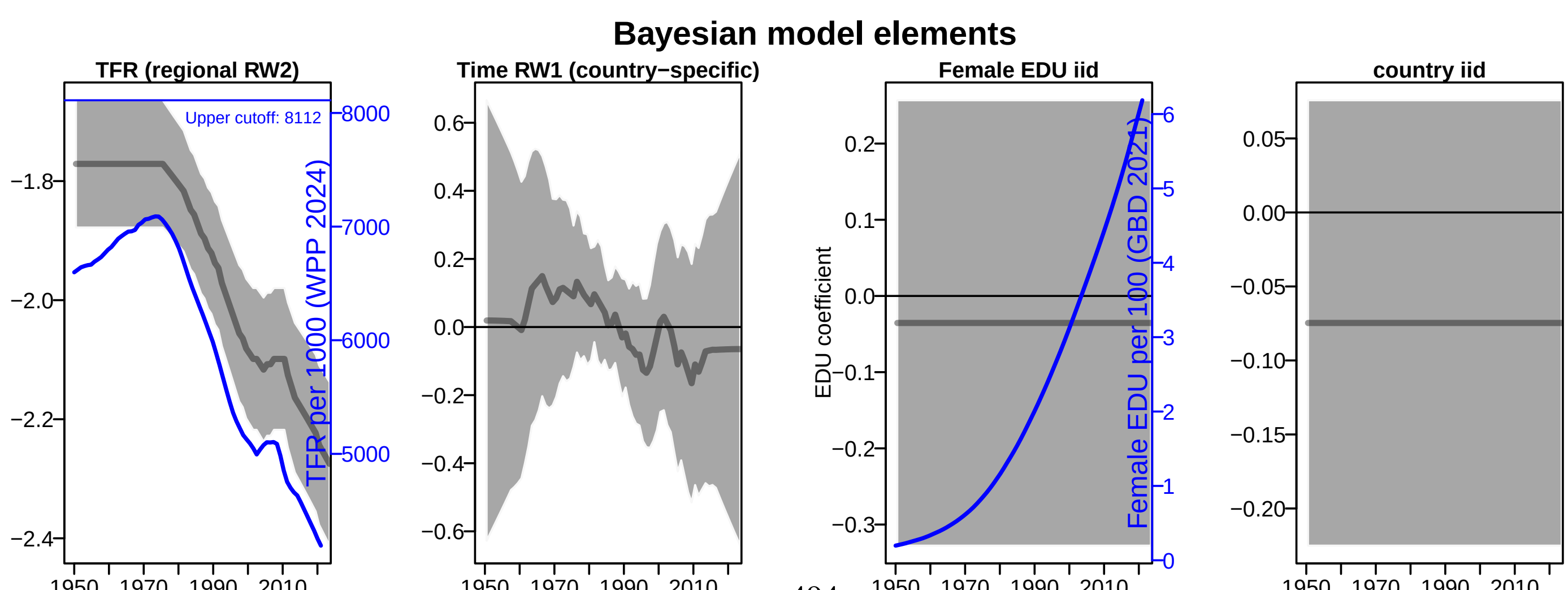
Bayesian model elements
TFR (regional RW2)
Upper cutoff: 8112
TFR per 1000 (WPP 2024)
Time RW1 (country−specific)
Female EDU iid
EDU coefficient
Female EDU per 100 (GBD 2021)
country iid
1950
1970
1990
2010

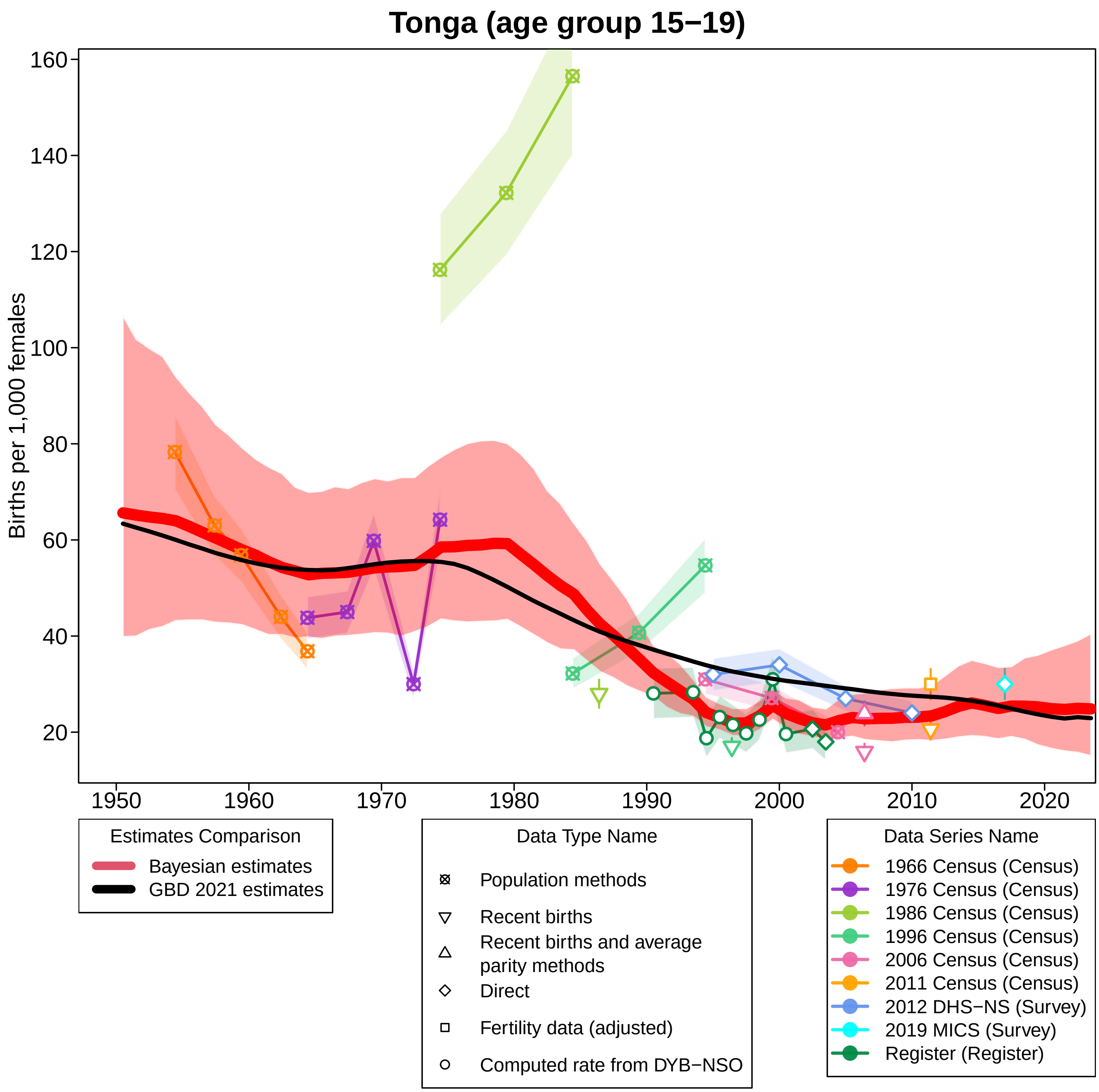
Tonga (age group 15−19)
Births per 1,000 females
160
140
120
100
80
60
40
20
1950
1960
1970
1980
1990
2000
2010
2020
Estimates Comparison
Bayesian estimates
GBD 2021 estimates
Data Type Name
Population methods
Recent births
Recent births and average parity methods
Direct
Fertility data (adjusted)
Computed rate from DYB−NSO
Data Series Name
1966 Census (Census)
1976 Census (Census)
1986 Census (Census)
1996 Census (Census)
2006 Census (Census)
2011 Census (Census)
2012 DHS−NS (Survey)
2019 MICS (Survey)
Register (Register)

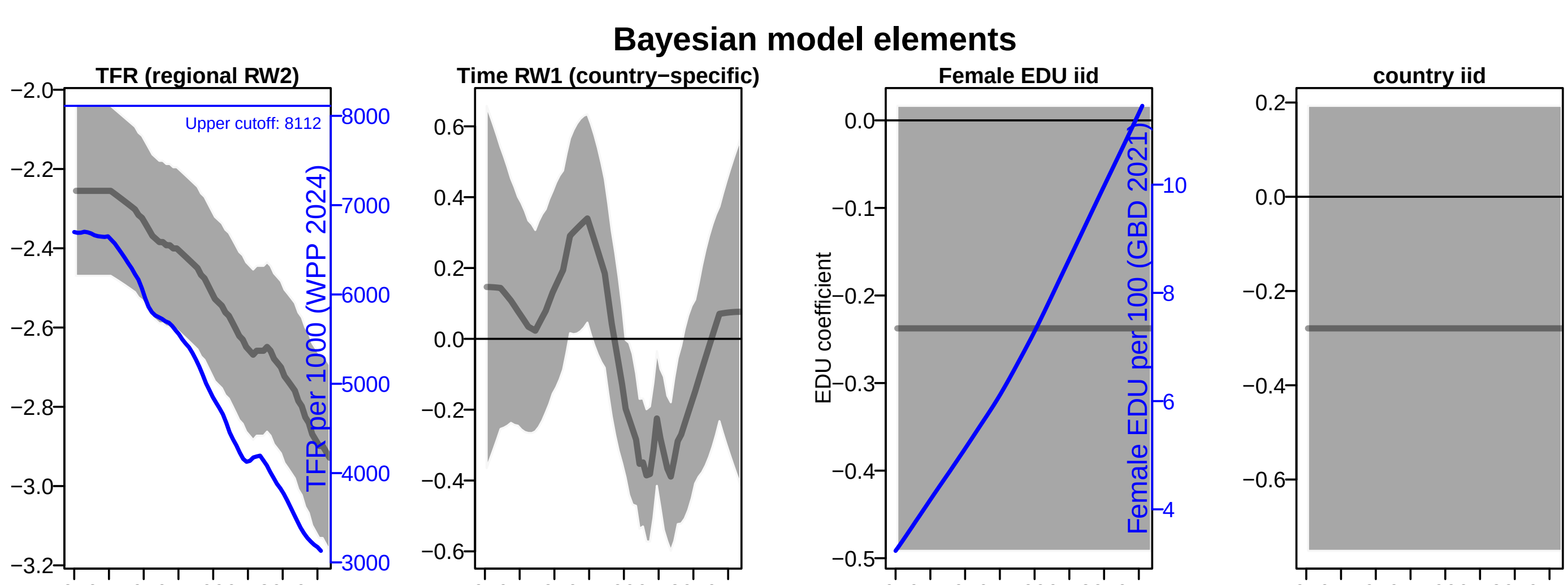
Bayesian model elements
TFR (regional RW2)
Upper cutoff: 8112
TFR per 1000 (WPP 2024)
Time RW1 (country−specific)
Female EDU iid
EDU coefficient
Female EDU per 100 (GBD 2021)
country iid
1950
1970
1990
2010

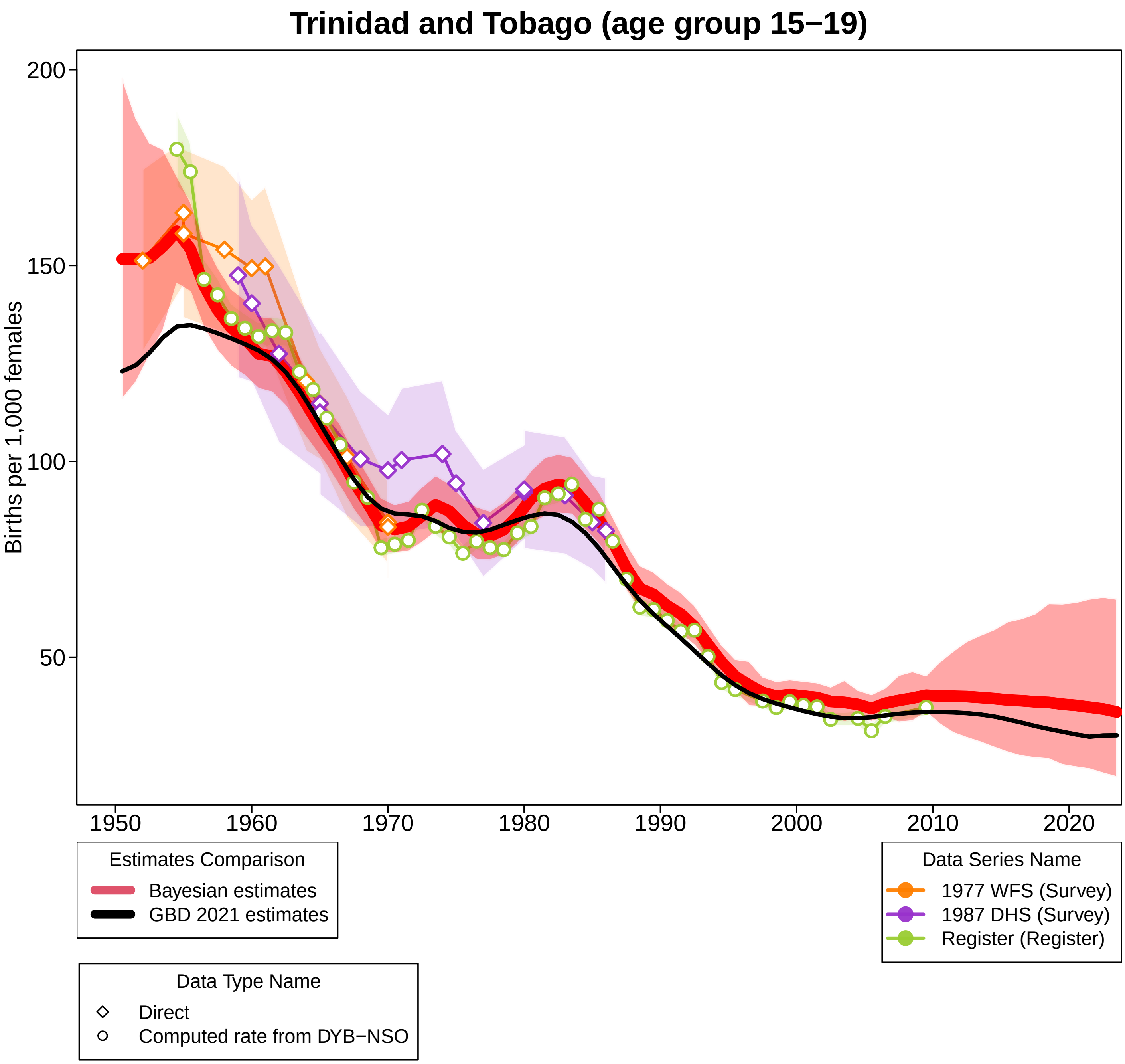
Trinidad and Tobago (age group 15−19)
Births per 1,000 females
200
150
100
50
1950
1960
1970
1980
1990
2000
2010
2020
Estimates Comparison
Bayesian estimates
GBD 2021 estimates
Data Series Name
1977 WFS (Survey)
1987 DHS (Survey)
Register (Register)
Data Type Name
Direct
Computed rate from DYB−NSO

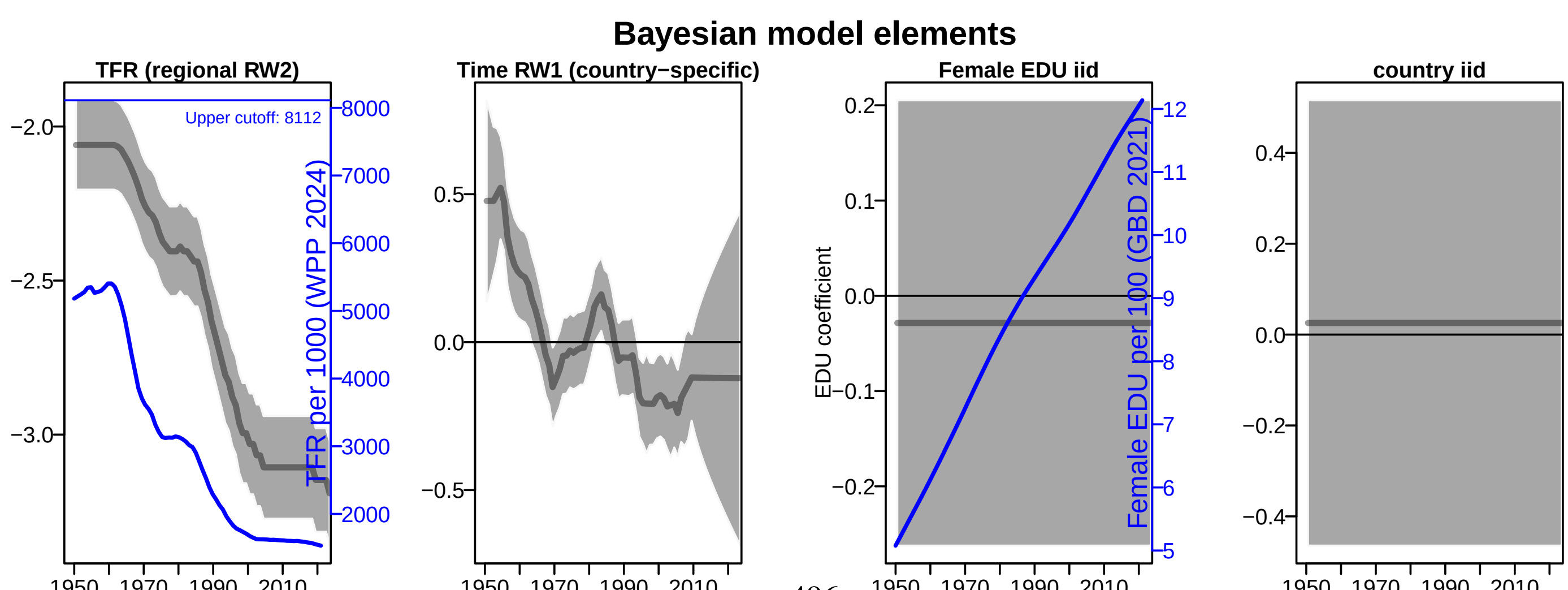
Bayesian model elements
TFR (regional RW2)
Upper cutoff: 8112
TFR per 1000 (WPP 2024)
Time RW1 (country−specific)
Female EDU iid
EDU coefficient
Female EDU per 100 (GBD 2021)
country iid
1950
1970
1990
2010

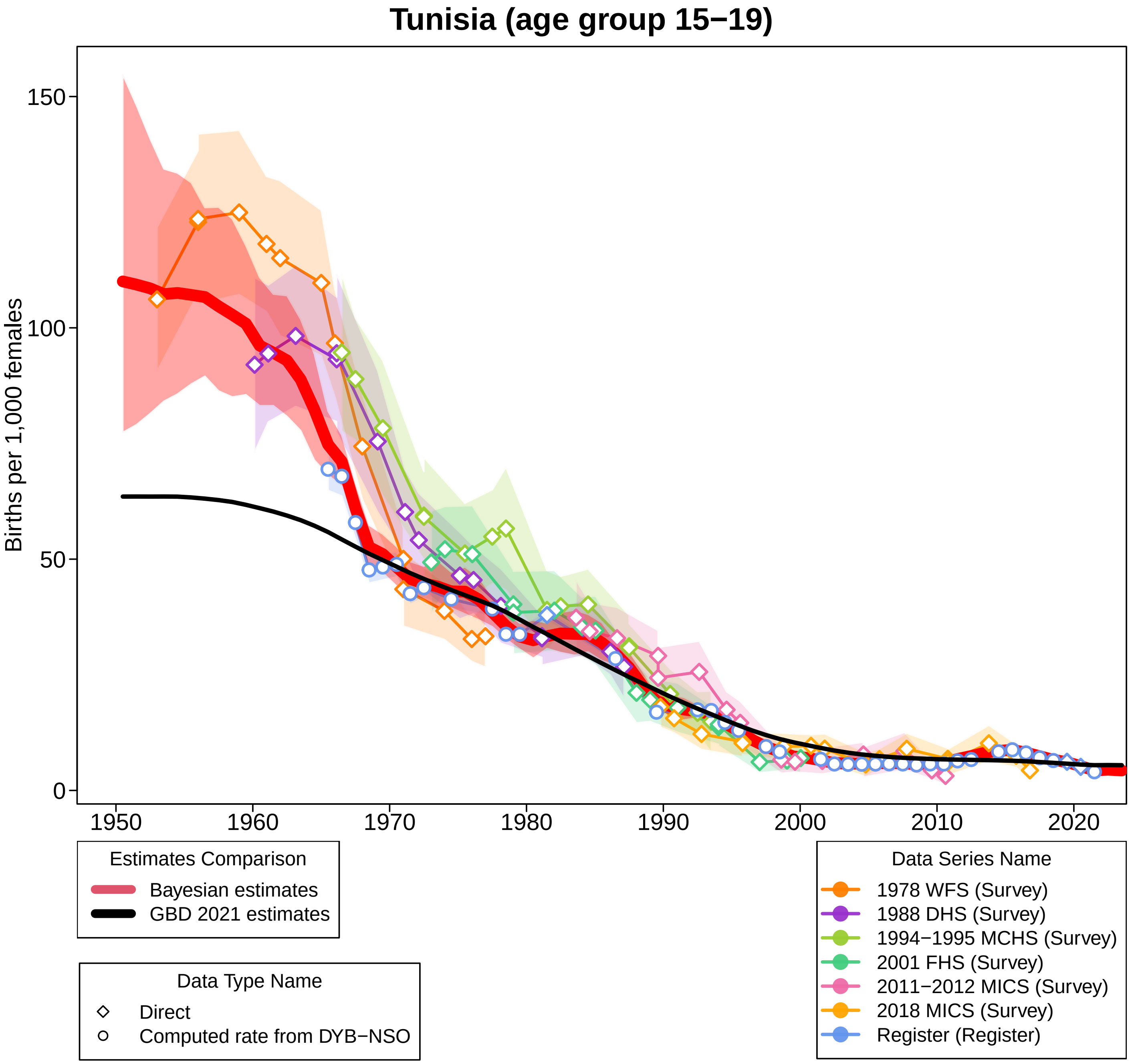
Tunisia (age group 15−19)
Births per 1,000 females
150
100
50
0
1950
1960
1970
1980
1990
2000
2010
2020
Estimates Comparison
Bayesian estimates
GBD 2021 estimates
Data Type Name
Direct
Computed rate from DYB−NSO
Data Series Name
1978 WFS (Survey)
1988 DHS (Survey)
1994−1995 MCHS (Survey)
2001 FHS (Survey)
2011−2012 MICS (Survey)
2018 MICS (Survey)
Register (Register)

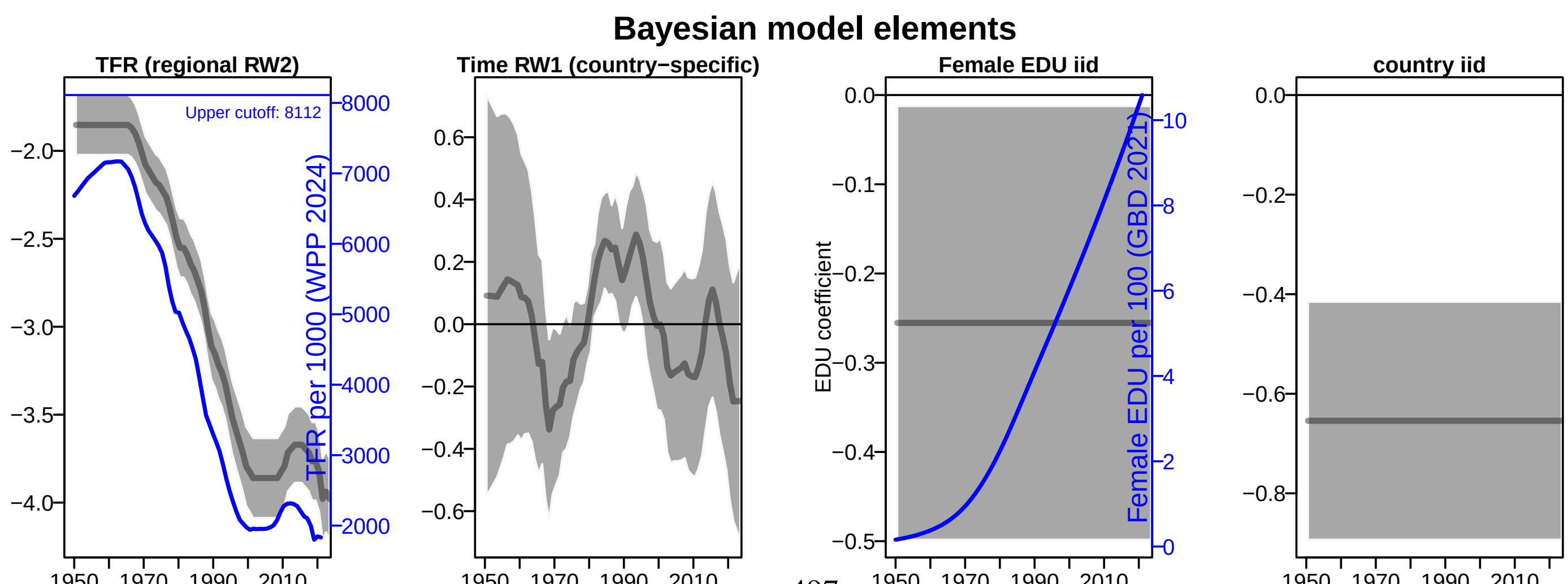
Bayesian model elements
TFR (regional RW2)
Upper cutoff: 8112
TFR per 1000 (WPP 2024)
Time RW1 (country−specific)
Female EDU iid
EDU coefficient
Female EDU per 100 (GBD 2021)
country iid

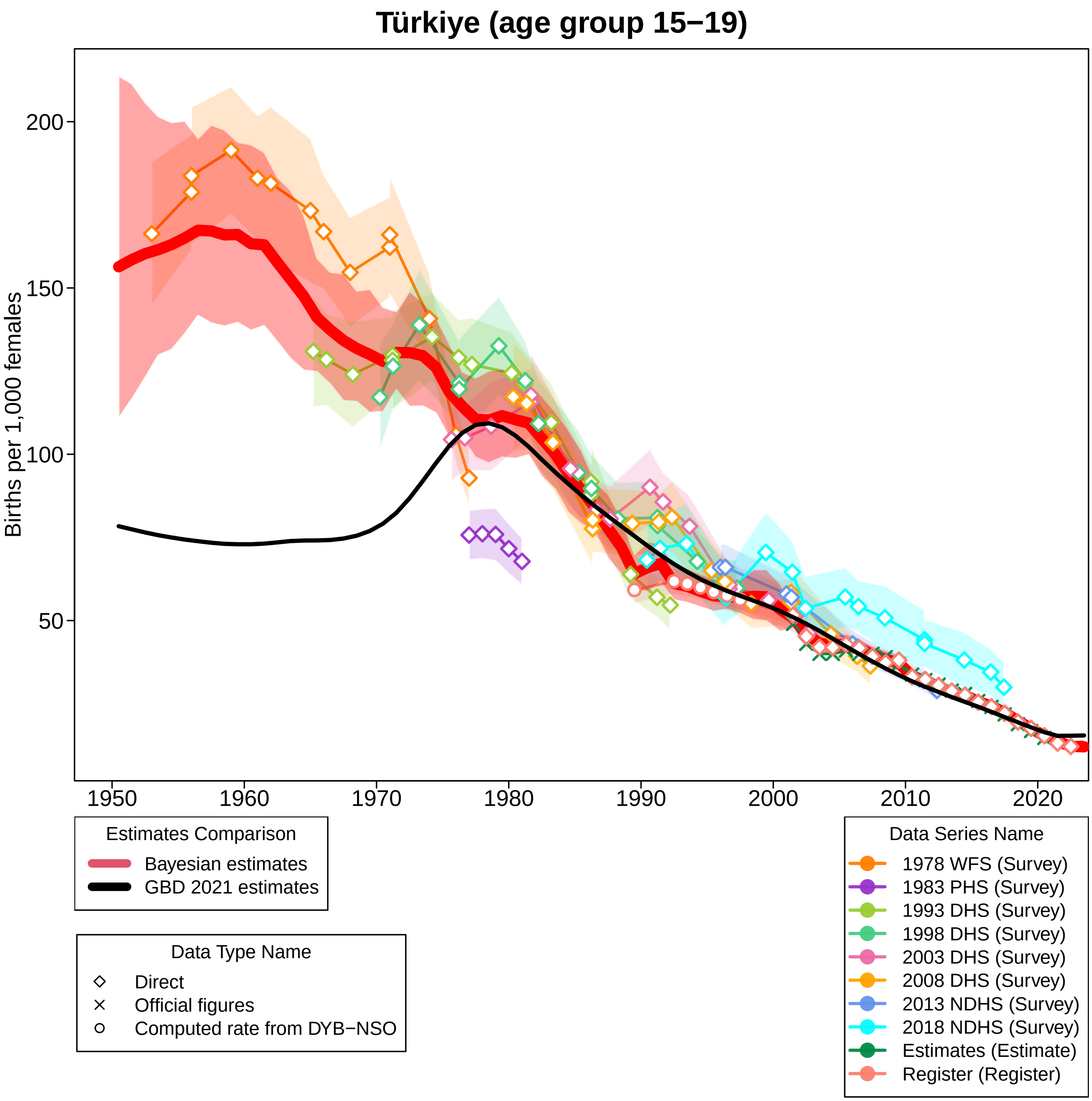

Türkiye (age group 15−19)
Births per 1,000 females
Estimates Comparison
Bayesian estimates
GBD 2021 estimates
Data Type Name
Direct
Official figures
Computed rate from DYB−NSO
Data Series Name
1978 WFS (Survey)
1983 PHS (Survey)
1993 DHS (Survey)
1998 DHS (Survey)
2003 DHS (Survey)
2008 DHS (Survey)
2013 NDHS (Survey)
2018 NDHS (Survey)
Estimates (Estimate)
Register (Register)

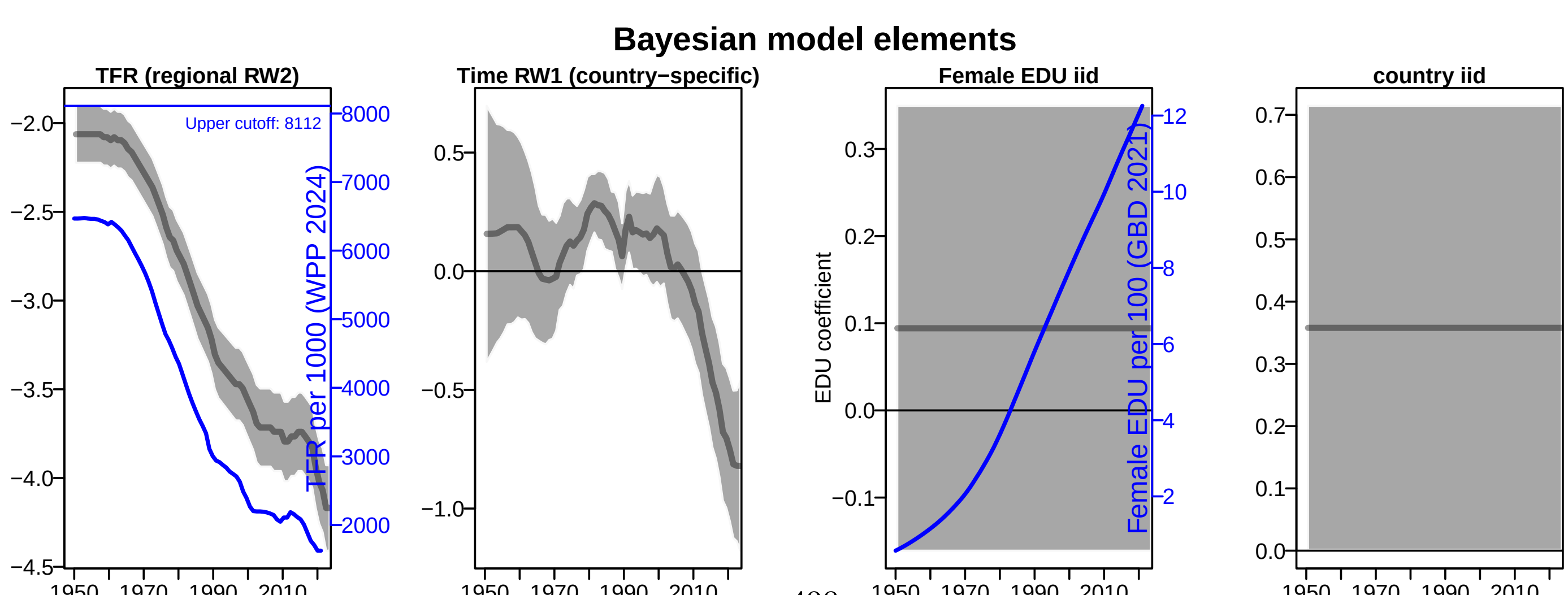

Bayesian model elements
TFR (regional RW2)
Upper cutoff: 8112
TFR per 1000 (WPP 2024)
Time RW1 (country−specific)
Female EDU iid
EDU coefficient
Female EDU per 100 (GBD 2021)
country iid

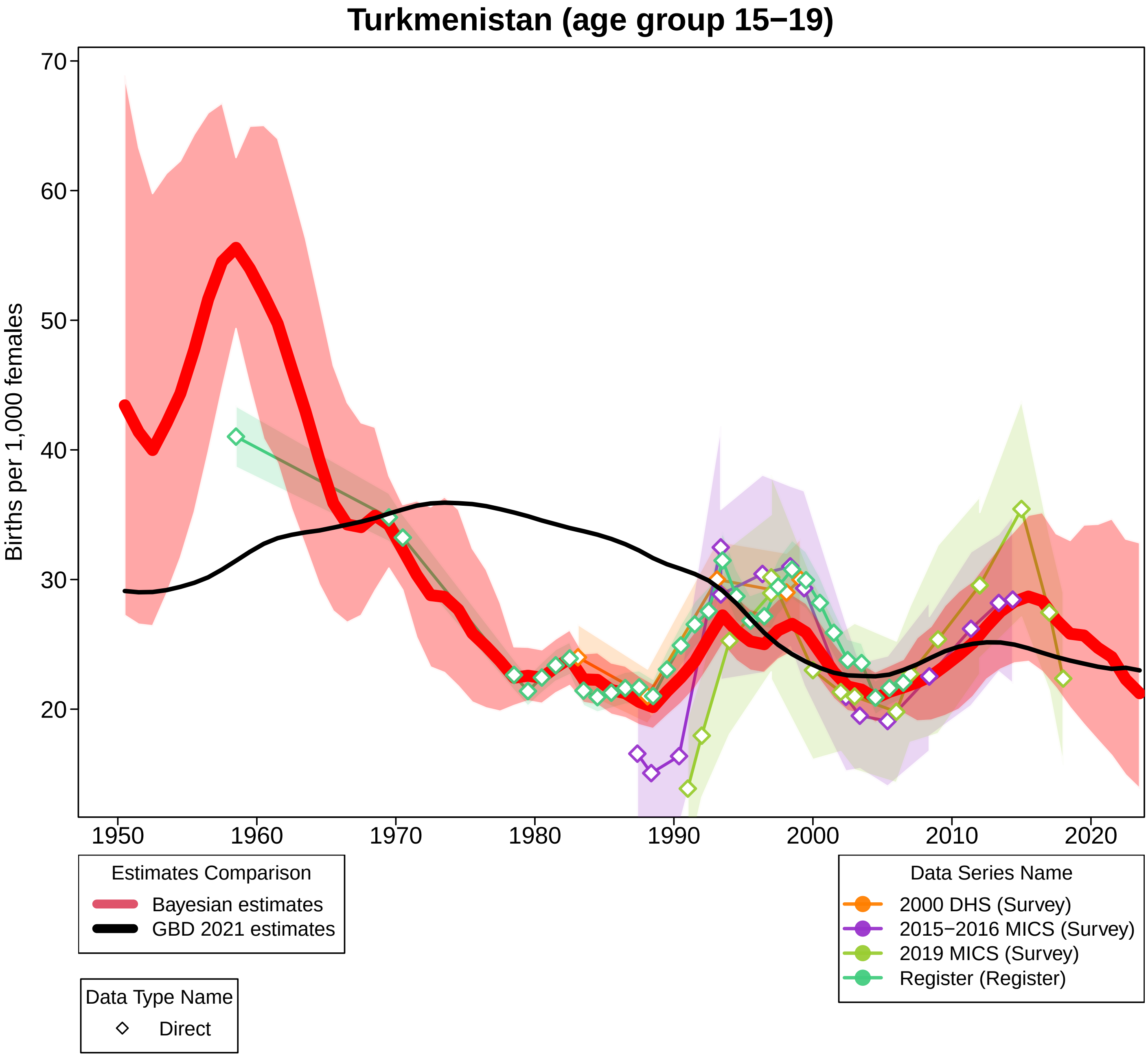
Turkmenistan (age group 15−19)
Births per 1,000 females
70
60
50
40
30
20
1950
1960
1970
1980
1990
2000
2010
2020
Estimates Comparison
Bayesian estimates
GBD 2021 estimates
Data Type Name
Direct
Data Series Name
2000 DHS (Survey)
2015−2016 MICS (Survey)
2019 MICS (Survey)
Register (Register)

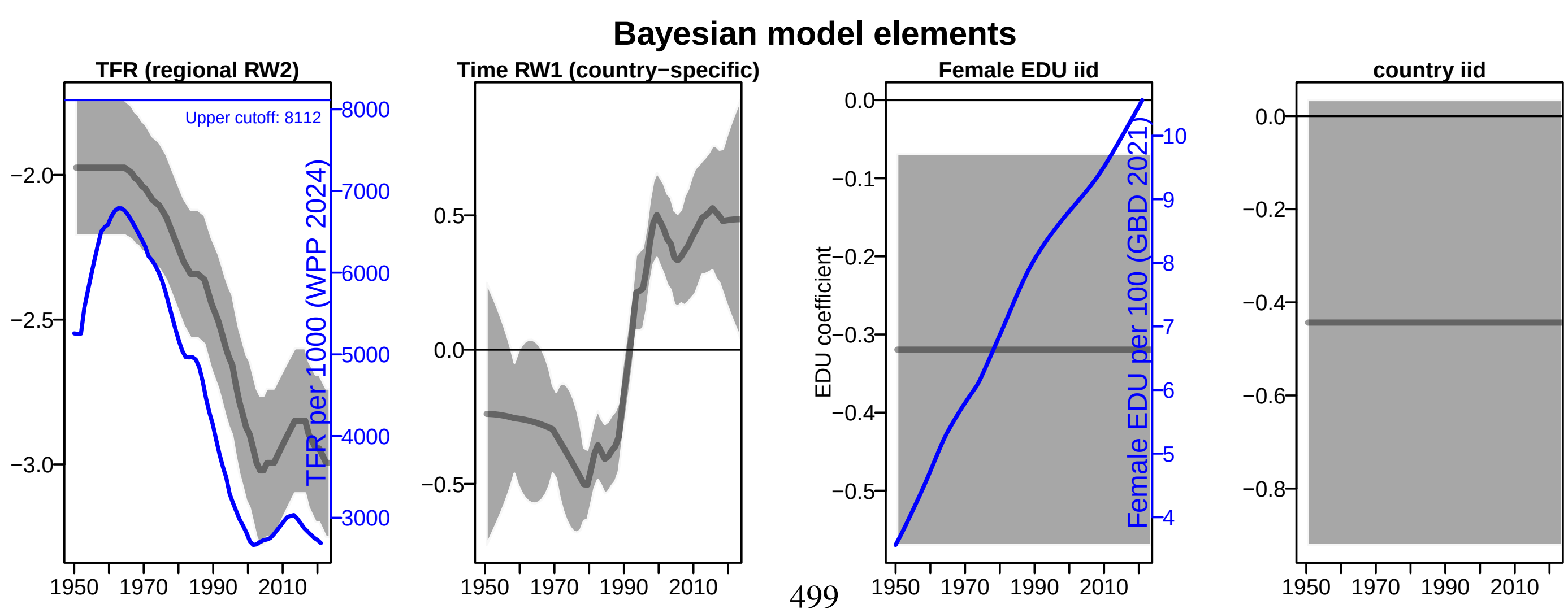
Bayesian model elements
TFR (regional RW2)
Upper cutoff: 8112
TFR per 1000 (WPP 2024)
Time RW1 (country−specific)
Female EDU iid
EDU coefficient
Female EDU per 100 (GBD 2021)
country iid

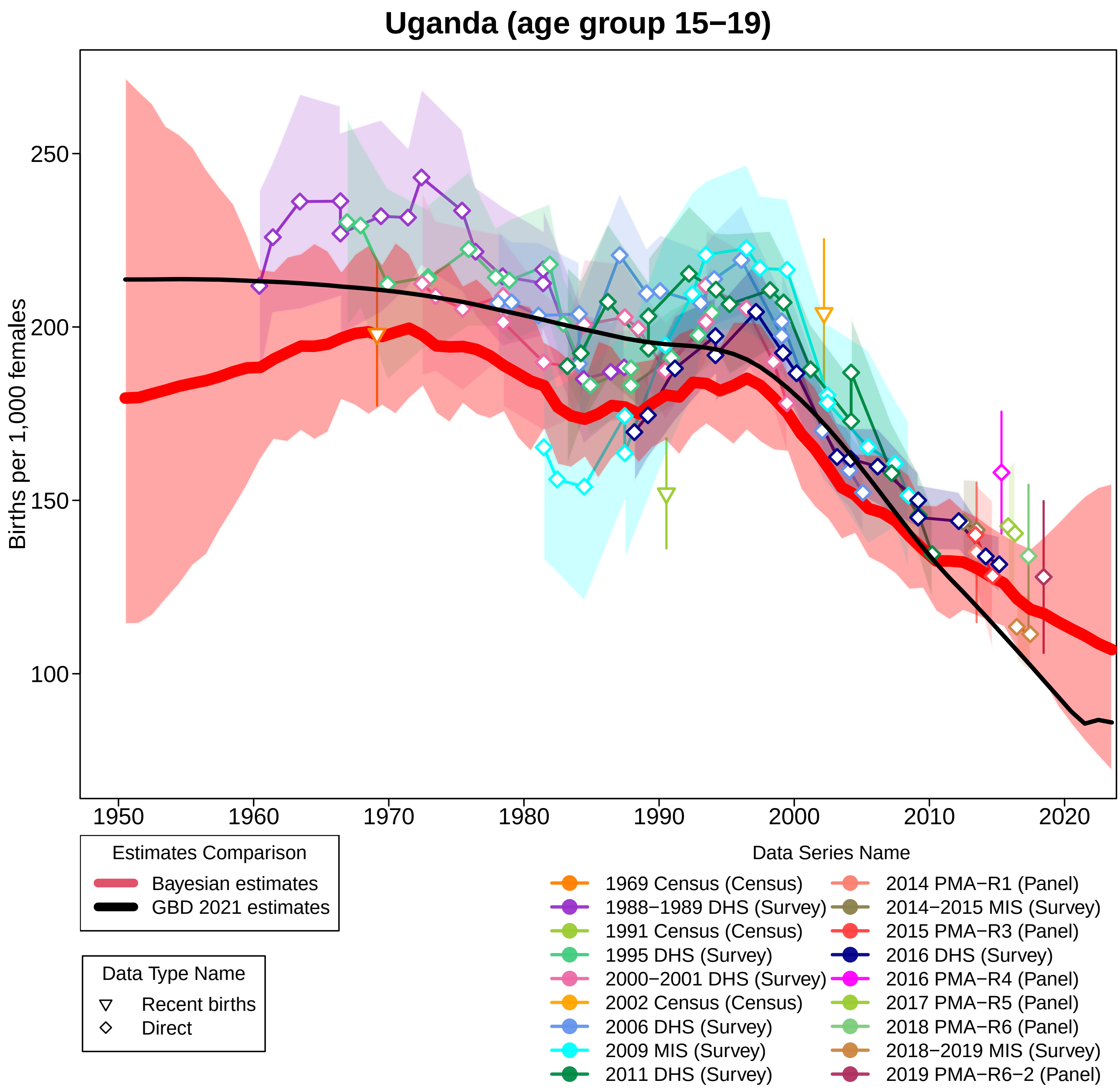
Uganda (age group 15−19)
Births per 1,000 females
250
200
150
100
1950
1960
1970
1980
1990
2000
2010
2020
Estimates Comparison
Bayesian estimates
GBD 2021 estimates
Data Type Name
Recent births
Direct
Data Series Name
1969 Census (Census)
1988−1989 DHS (Survey)
1991 Census (Census)
1995 DHS (Survey)
2000−2001 DHS (Survey)
2002 Census (Census)
2006 DHS (Survey)
2009 MIS (Survey)
2011 DHS (Survey)
2014 PMA−R1 (Panel)
2014−2015 MIS (Survey)
2015 PMA−R3 (Panel)
2016 DHS (Survey)
2016 PMA−R4 (Panel)
2017 PMA−R5 (Panel)
2018 PMA−R6 (Panel)
2018−2019 MIS (Survey)
2019 PMA−R6−2 (Panel)

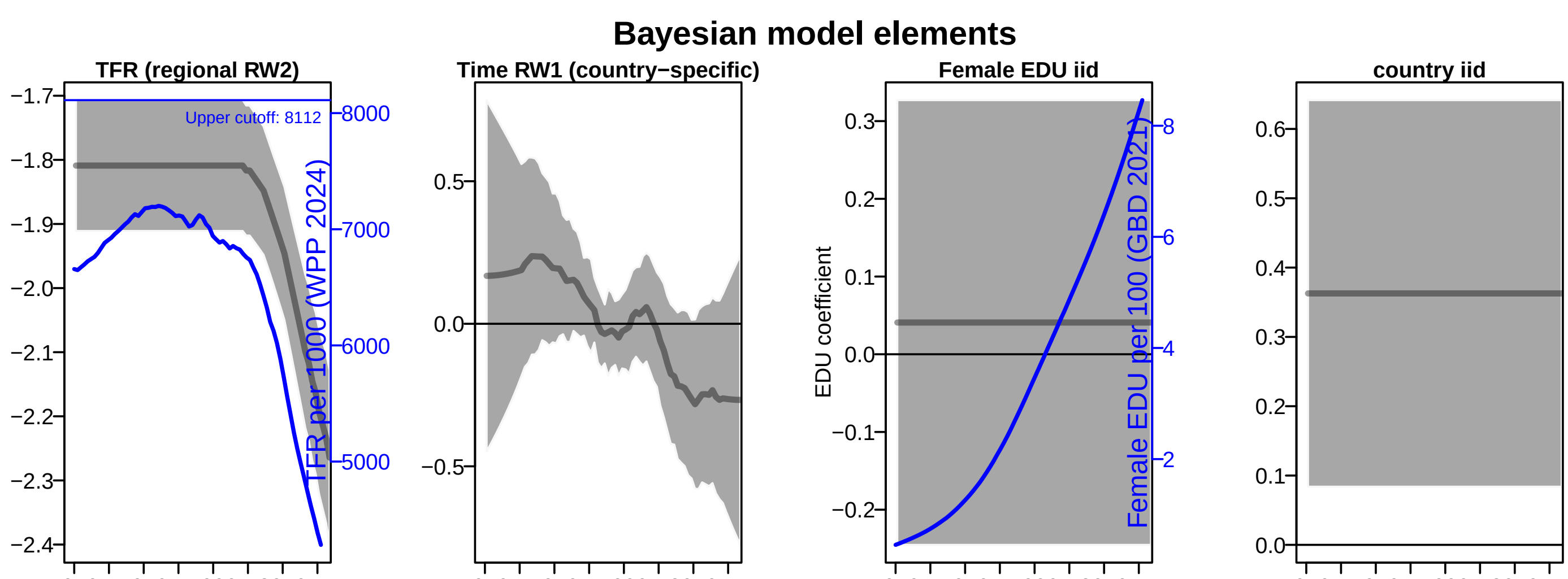
Bayesian model elements
TFR (regional RW2)
Upper cutoff: 8112
TFR per 1000 (WPP 2024)
Time RW1 (country−specific)
Female EDU iid
EDU coefficient
Female EDU per 100 (GBD 2021)
country iid
1950
1970
1990
2010

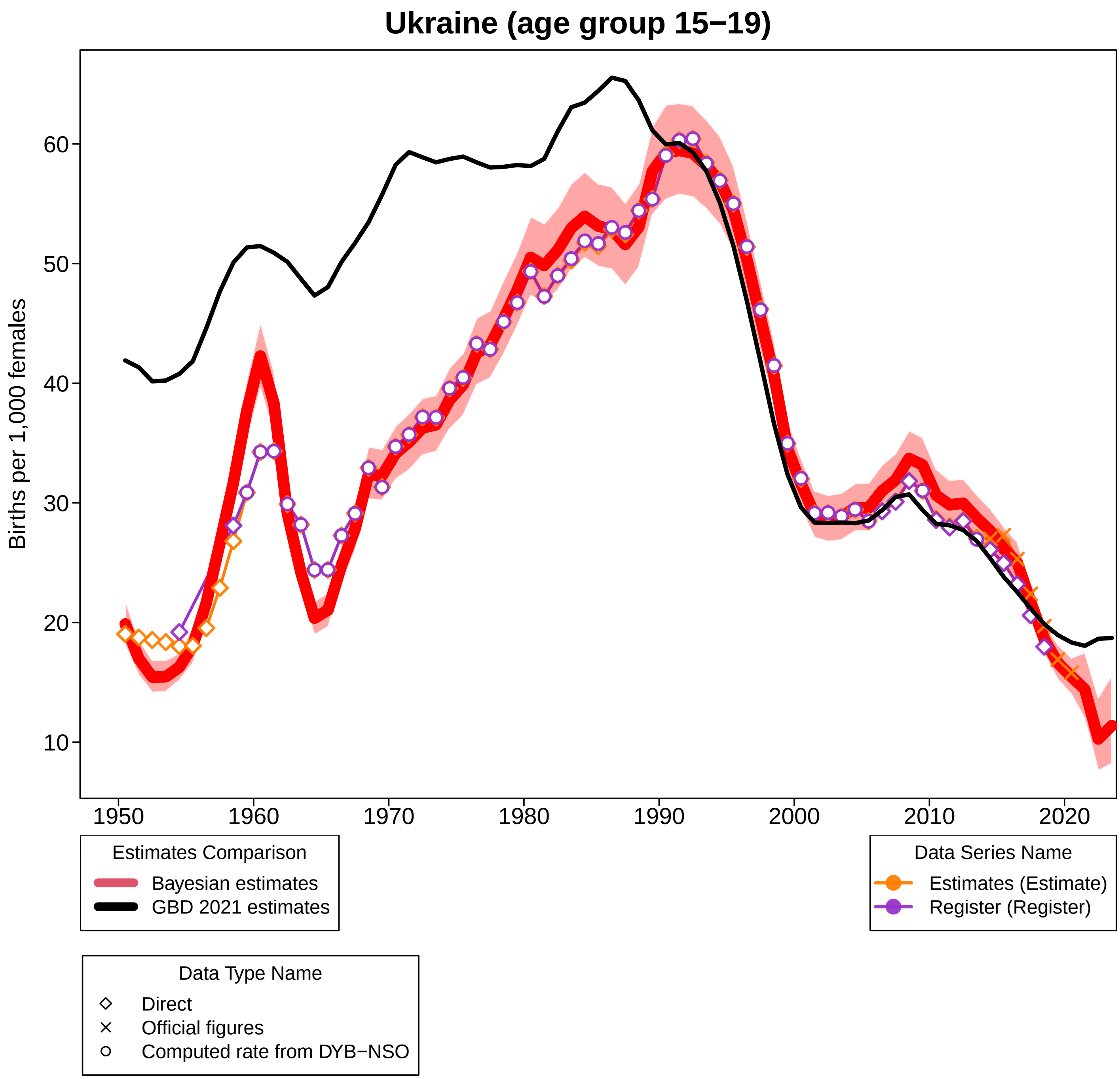

Ukraine (age group 15−19)
Births per 1,000 females
10
20
30
40
50
60
1950
1960
1970
1980
1990
2000
2010
2020
Estimates Comparison
Bayesian estimates
GBD 2021 estimates
Data Series Name
Estimates (Estimate)
Register (Register)
Data Type Name
Direct
Official figures
Computed rate from DYB−NSO

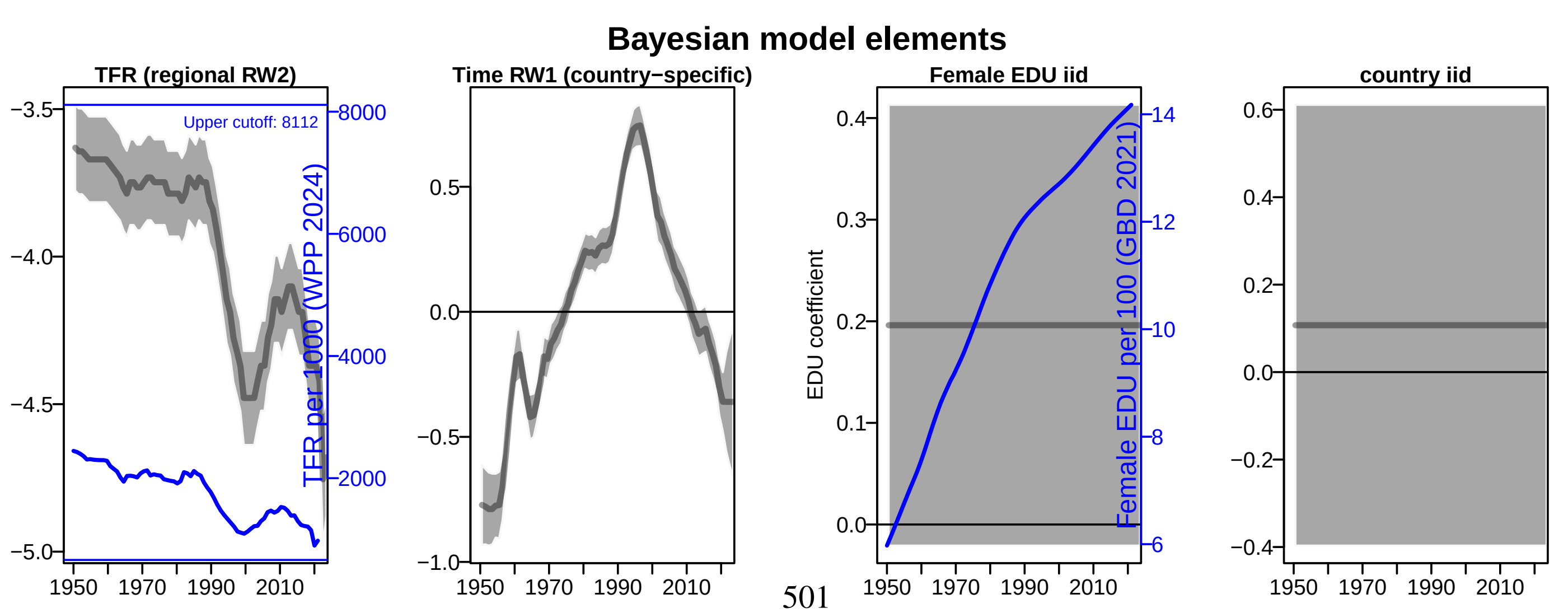

Bayesian model elements
TFR (regional RW2)
Upper cutoff: 8112
TFR per 1000 (WPP 2024)
Time RW1 (country−specific)
Female EDU iid
EDU coefficient
Female EDU per 100 (GBD 2021)
country iid

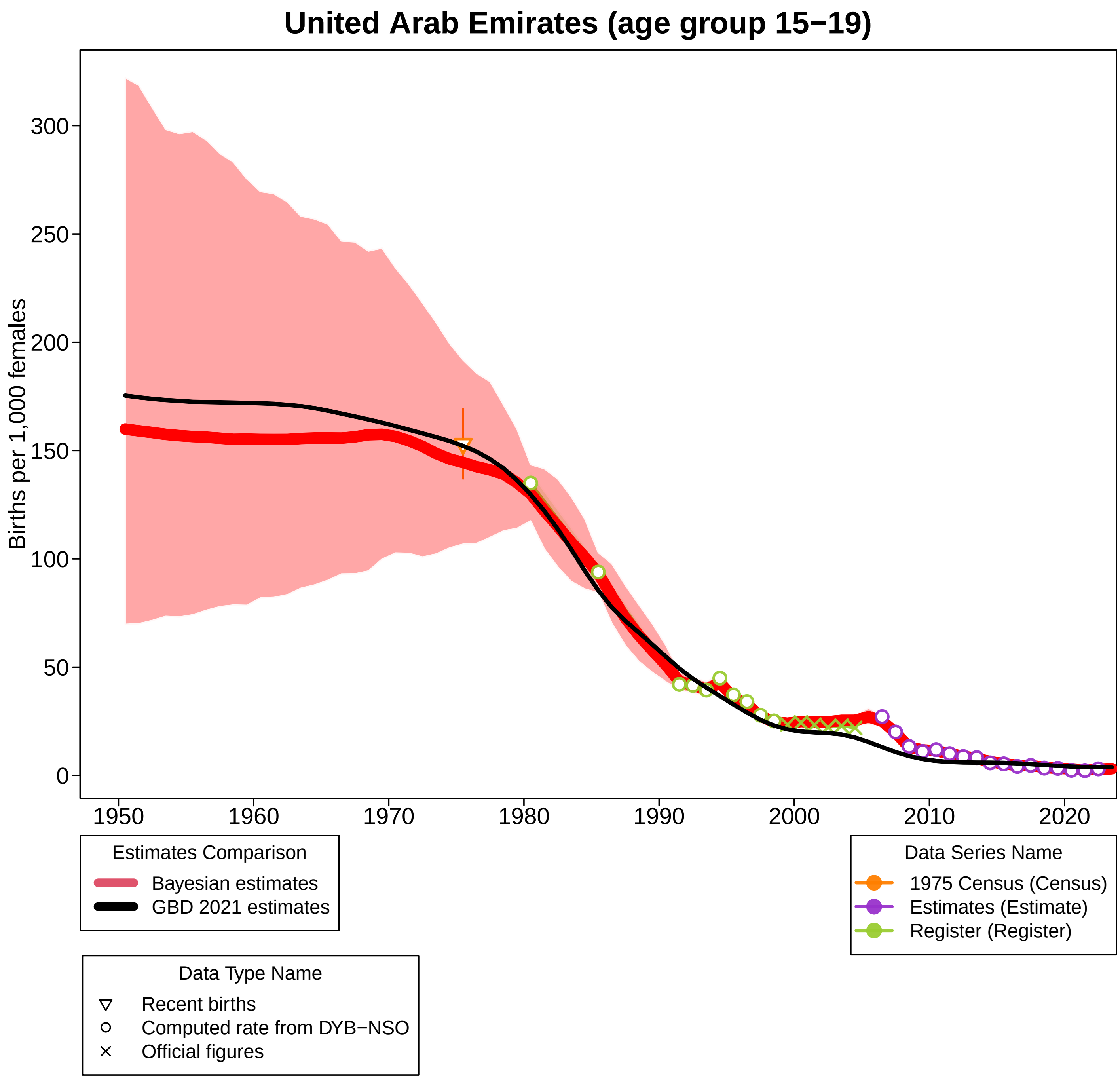
United Arab Emirates (age group 15−19)
Births per 1,000 females
0
50
100
150
200
250
300
1950
1960
1970
1980
1990
2000
2010
2020
Estimates Comparison
Bayesian estimates
GBD 2021 estimates
Data Series Name
1975 Census (Census)
Estimates (Estimate)
Register (Register)
Data Type Name
Recent births
Computed rate from DYB−NSO
Official figures

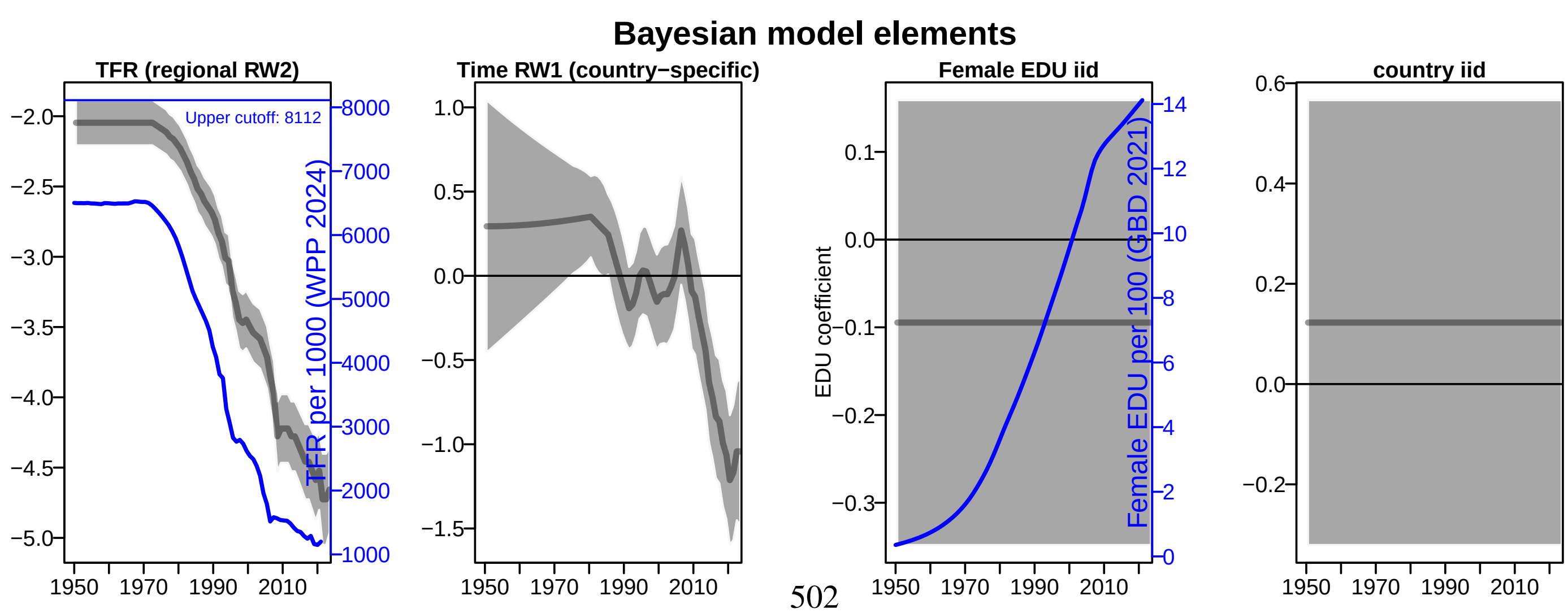
Bayesian model elements
TFR (regional RW2)
Upper cutoff: 8112
TFR per 1000 (WPP 2024)
Time RW1 (country−specific)
Female EDU iid
EDU coefficient
Female EDU per 100 (GBD 2021)
country iid

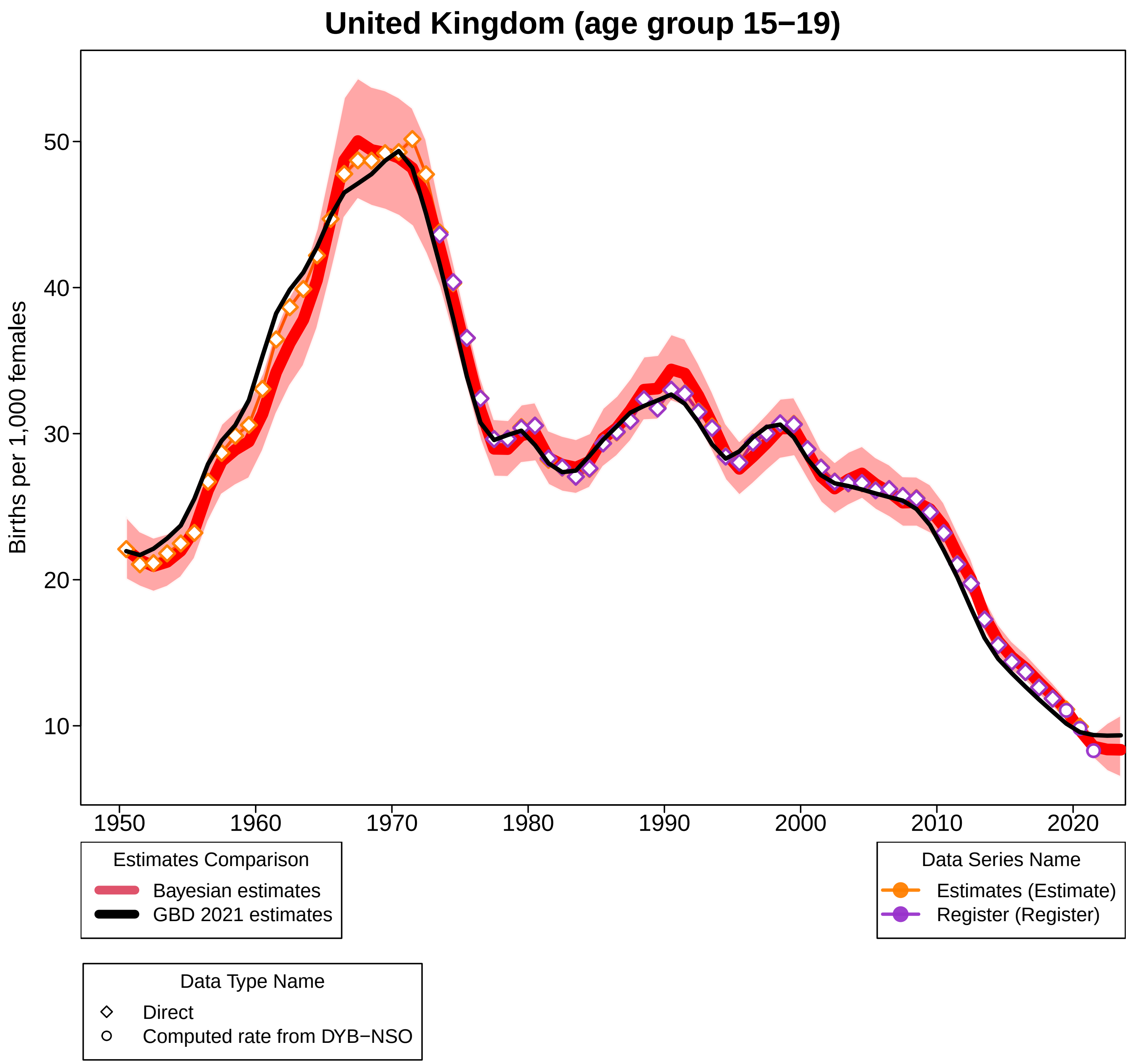
United Kingdom (age group 15−19)
Births per 1,000 females
50
40
30
20
10
1950
1960
1970
1980
1990
2000
2010
2020
Estimates Comparison
Bayesian estimates
GBD 2021 estimates
Data Series Name
Estimates (Estimate)
Register (Register)
Data Type Name
Direct
Computed rate from DYB−NSO

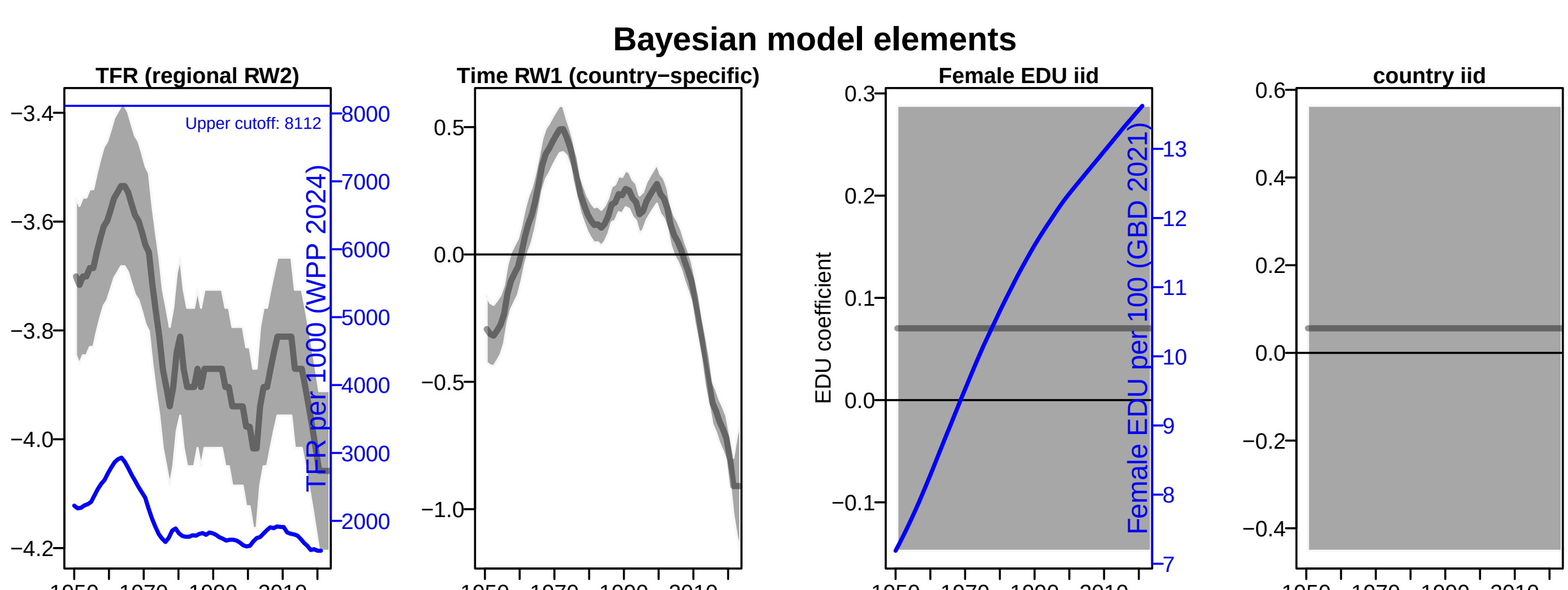
Bayesian model elements
TFR (regional RW2)
Upper cutoff: 8112
TFR per 1000 (WPP 2024)
Time RW1 (country−specific)
Female EDU iid
EDU coefficient
Female EDU per 100 (GBD 2021)
country iid
1950
1970
1990
2010

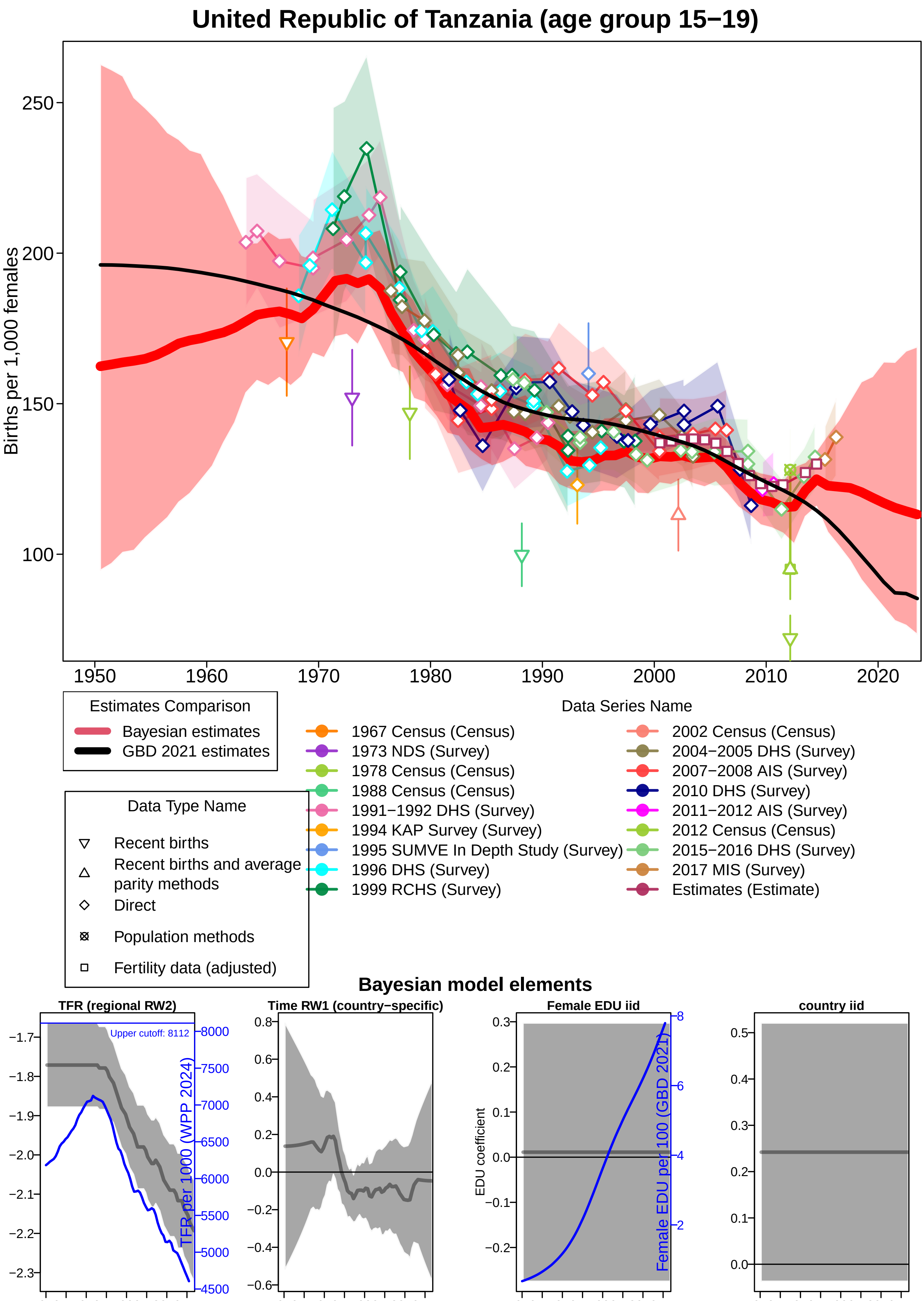
United Republic of Tanzania (age group 15−19)
Births per 1,000 females
250
200
150
100
1950
1960
1970
1980
1990
2000
2010
2020
Estimates Comparison
Bayesian estimates
GBD 2021 estimates
Data Series Name
1967 Census (Census)
1973 NDS (Survey)
1978 Census (Census)
1988 Census (Census)
1991−1992 DHS (Survey)
1994 KAP Survey (Survey)
1995 SUMVE In Depth Study (Survey)
1996 DHS (Survey)
1999 RCHS (Survey)
2002 Census (Census)
2004−2005 DHS (Survey)
2007−2008 AIS (Survey)
2010 DHS (Survey)
2011−2012 AIS (Survey)
2012 Census (Census)
2015−2016 DHS (Survey)
2017 MIS (Survey)
Estimates (Estimate)
Data Type Name
Recent births
Recent births and average parity methods
Direct
Population methods
Fertility data (adjusted)
Bayesian model elements
TFR (regional RW2)
Upper cutoff: 8112
TFR per 1000 (WPP 2024)
Time RW1 (country−specific)
Female EDU iid
EDU coefficient
Female EDU per 100 (GBD 2021)
country iid

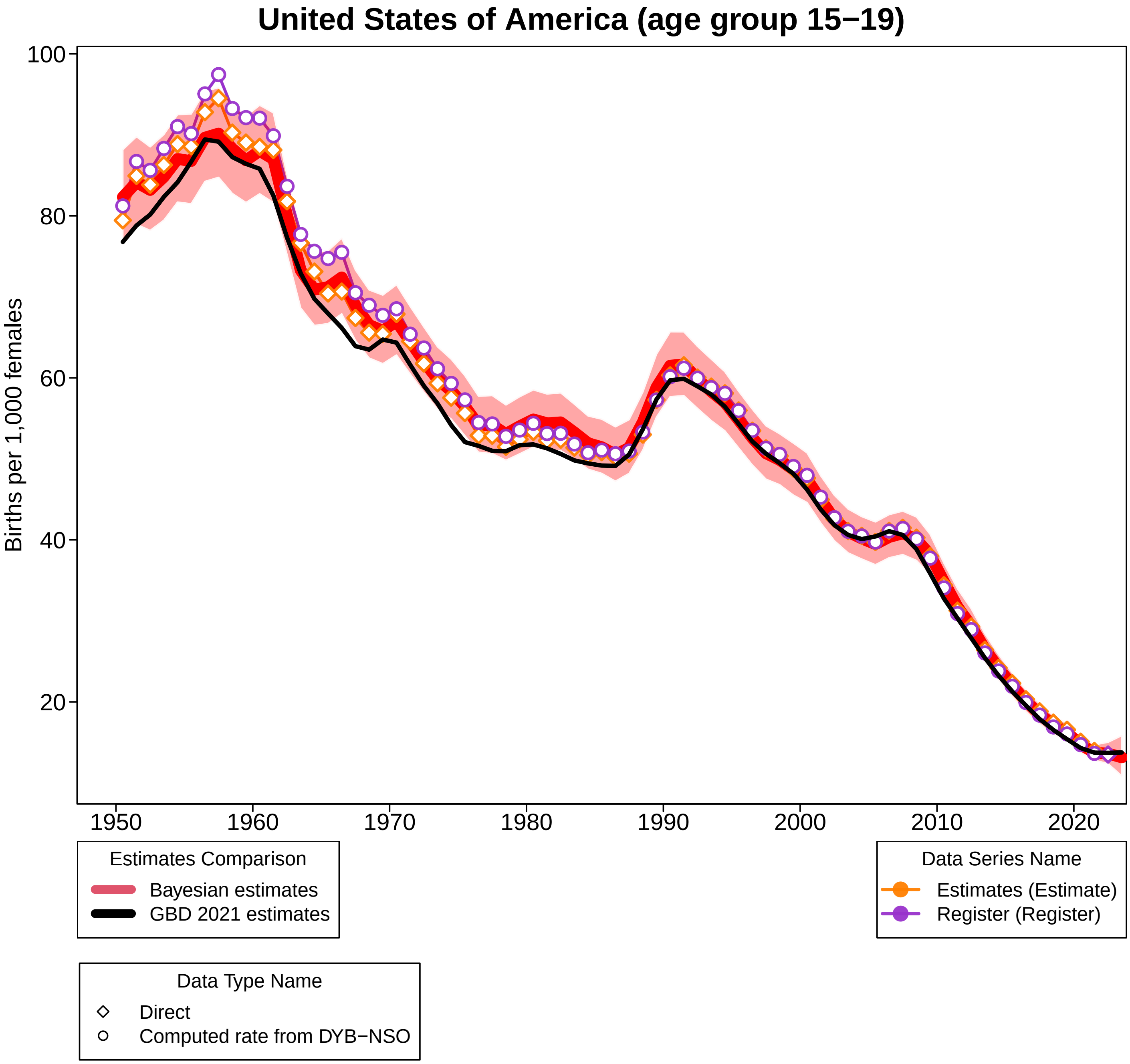

United States of America (age group 15−19)
Births per 1,000 females
Estimates Comparison
Bayesian estimates
GBD 2021 estimates
Data Series Name
Estimates (Estimate)
Register (Register)
Data Type Name
Direct
Computed rate from DYB−NSO

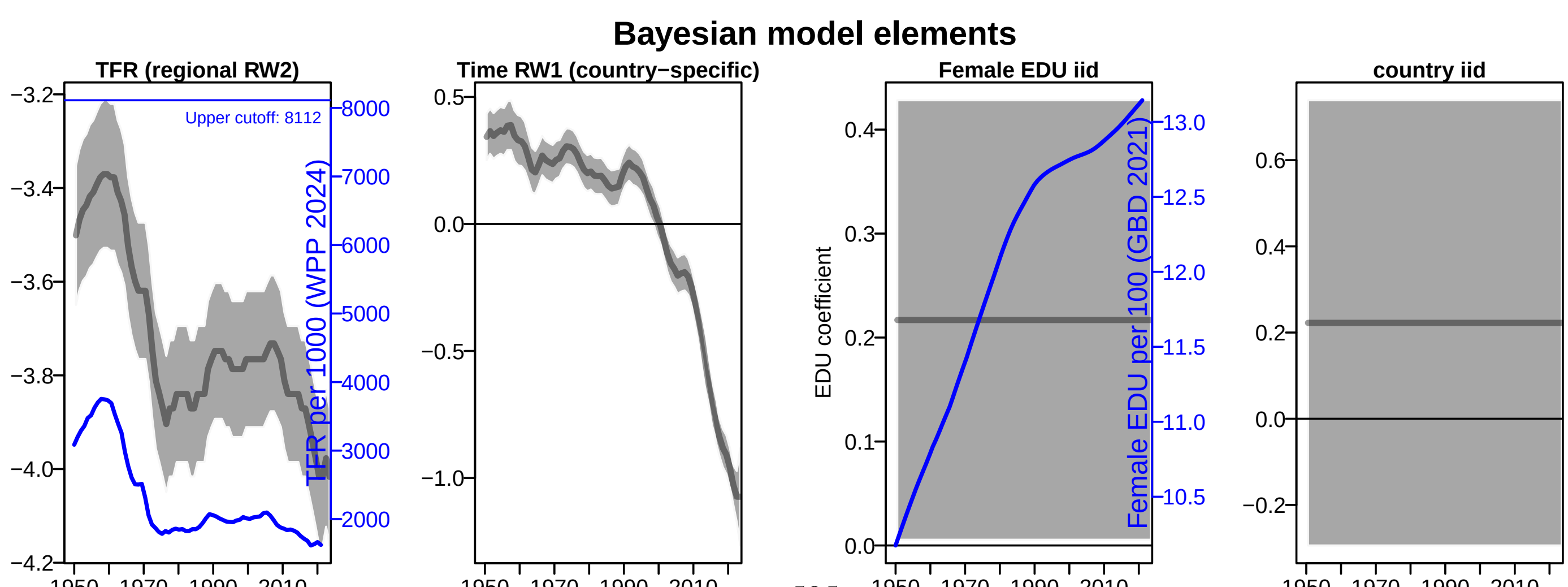

Bayesian model elements
TFR (regional RW2)
Upper cutoff: 8112
TFR per 1000 (WPP 2024)
Time RW1 (country−specific)
Female EDU iid
EDU coefficient
Female EDU per 100 (GBD 2021)
country iid

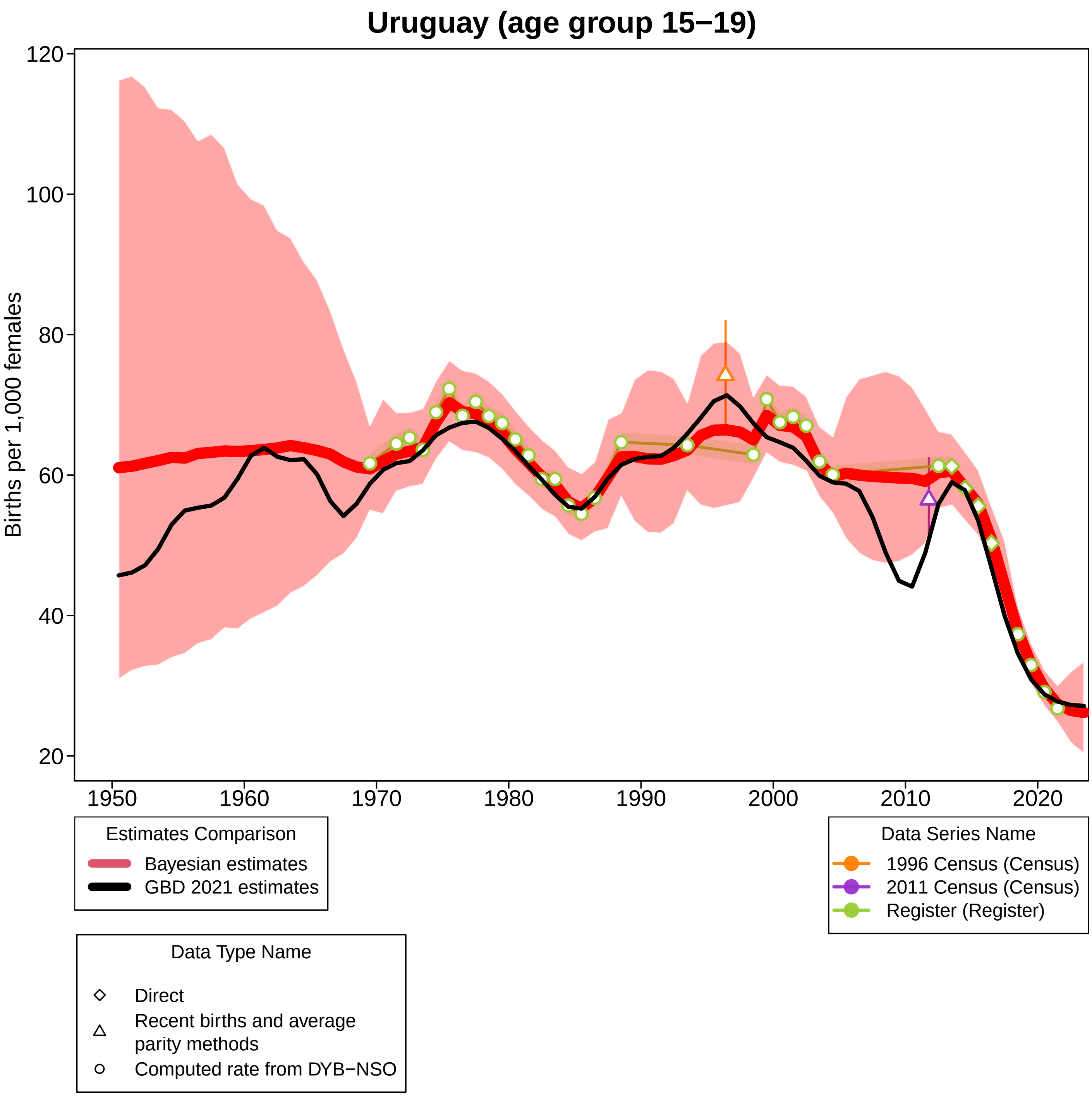
Uruguay (age group 15−19)
Births per 1,000 females
120
100
80
60
40
20
1950
1960
1970
1980
1990
2000
2010
2020
Estimates Comparison
Bayesian estimates
GBD 2021 estimates
Data Series Name
1996 Census (Census)
2011 Census (Census)
Register (Register)
Data Type Name
Direct
Recent births and average parity methods
Computed rate from DYB−NSO

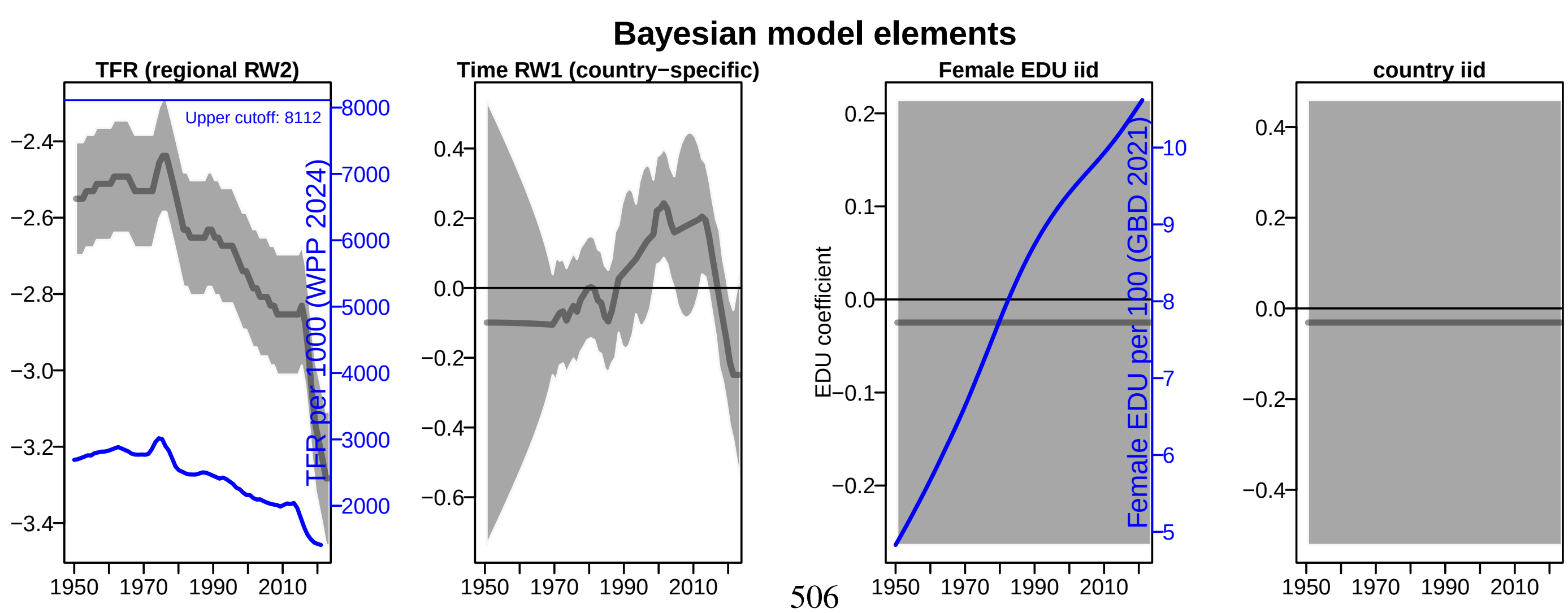
Bayesian model elements
TFR (regional RW2)
Upper cutoff: 8112
TFR per 1000 (WPP 2024)
Time RW1 (country−specific)
Female EDU iid
EDU coefficient
Female EDU per 100 (GBD 2021)
country iid

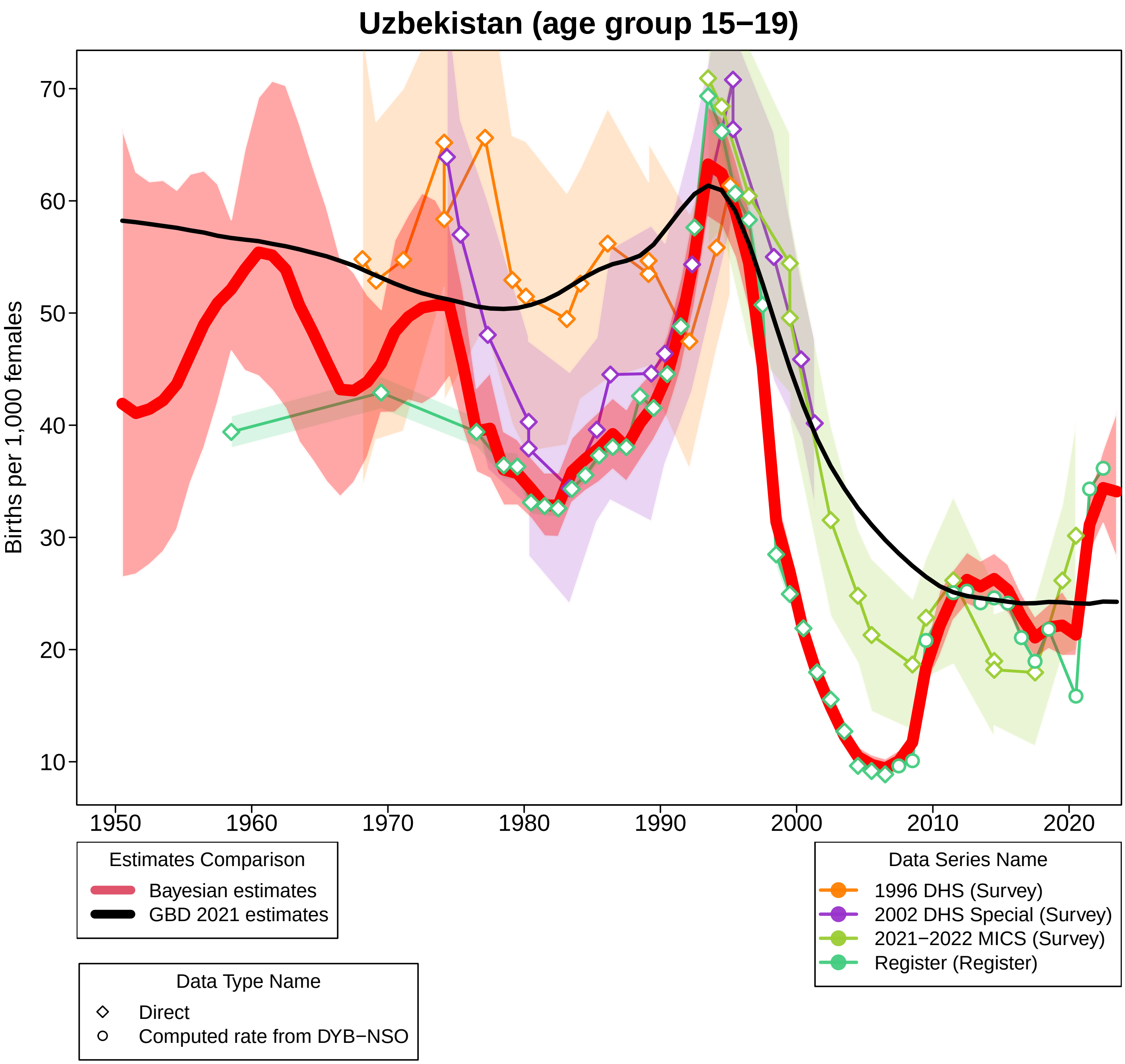
Uzbekistan (age group 15−19)
Births per 1,000 females
70
60
50
40
30
20
10
1950
1960
1970
1980
1990
2000
2010
2020
Estimates Comparison
Bayesian estimates
GBD 2021 estimates
Data Series Name
1996 DHS (Survey)
2002 DHS Special (Survey)
2021−2022 MICS (Survey)
Register (Register)
Data Type Name
Direct
Computed rate from DYB−NSO

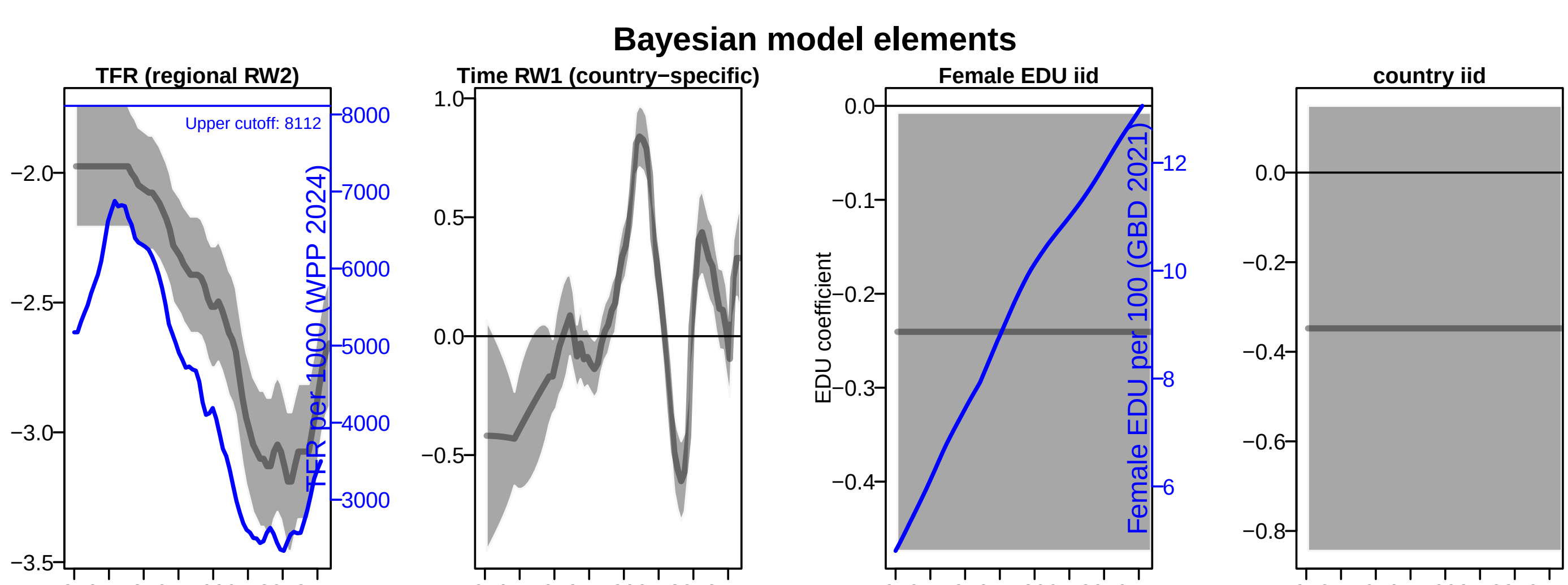
Bayesian model elements
TFR (regional RW2)
Upper cutoff: 8112
TFR per 1000 (WPP 2024)
Time RW1 (country−specific)
Female EDU iid
EDU coefficient
Female EDU per 100 (GBD 2021)
country iid

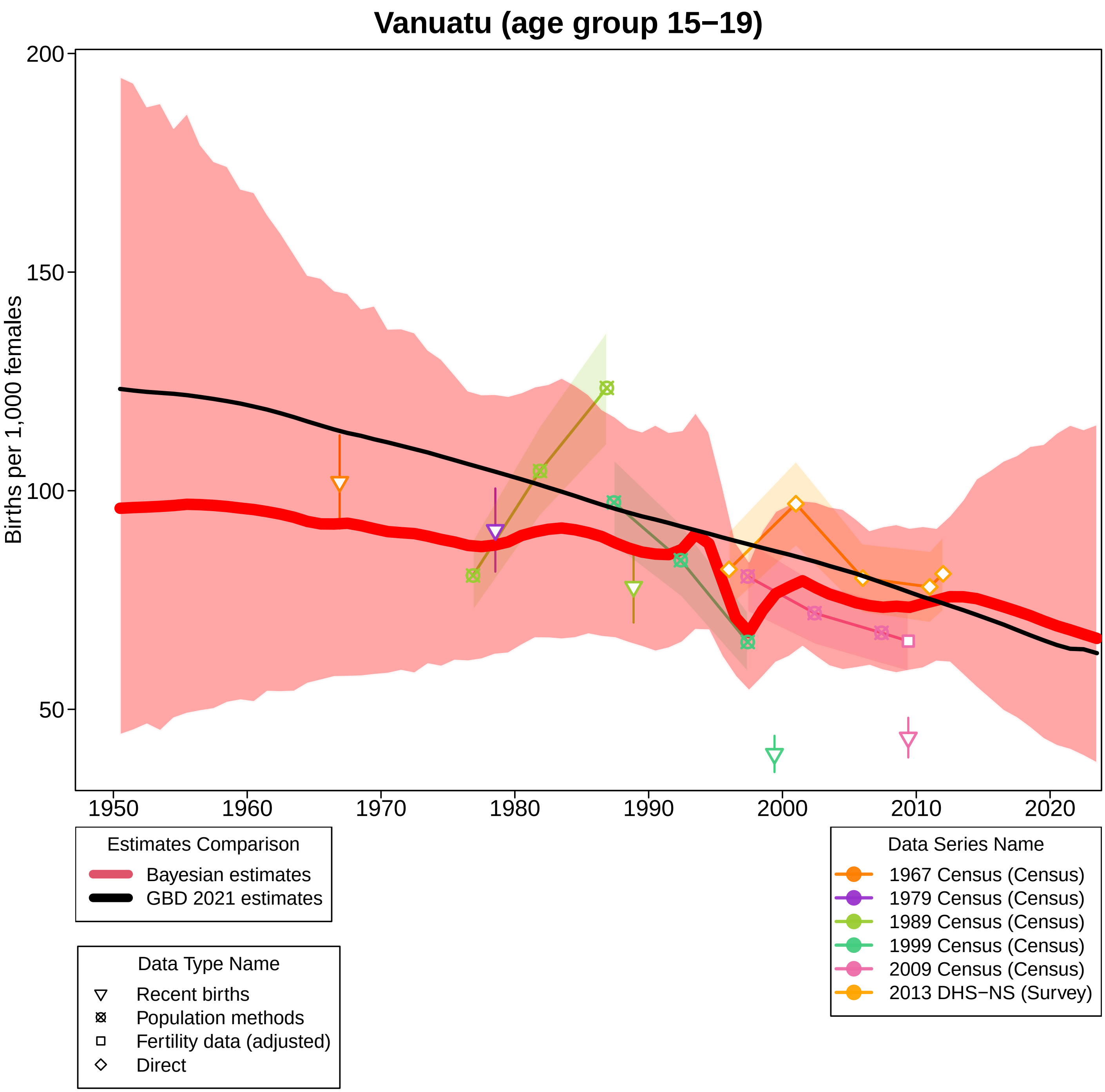
Vanuatu (age group 15−19)
Births per 1,000 females
200
150
100
50
1950
1960
1970
1980
1990
2000
2010
2020
Estimates Comparison
Bayesian estimates
GBD 2021 estimates
Data Series Name
1967 Census (Census)
1979 Census (Census)
1989 Census (Census)
1999 Census (Census)
2009 Census (Census)
2013 DHS−NS (Survey)
Data Type Name
Recent births
Population methods
Fertility data (adjusted)
Direct

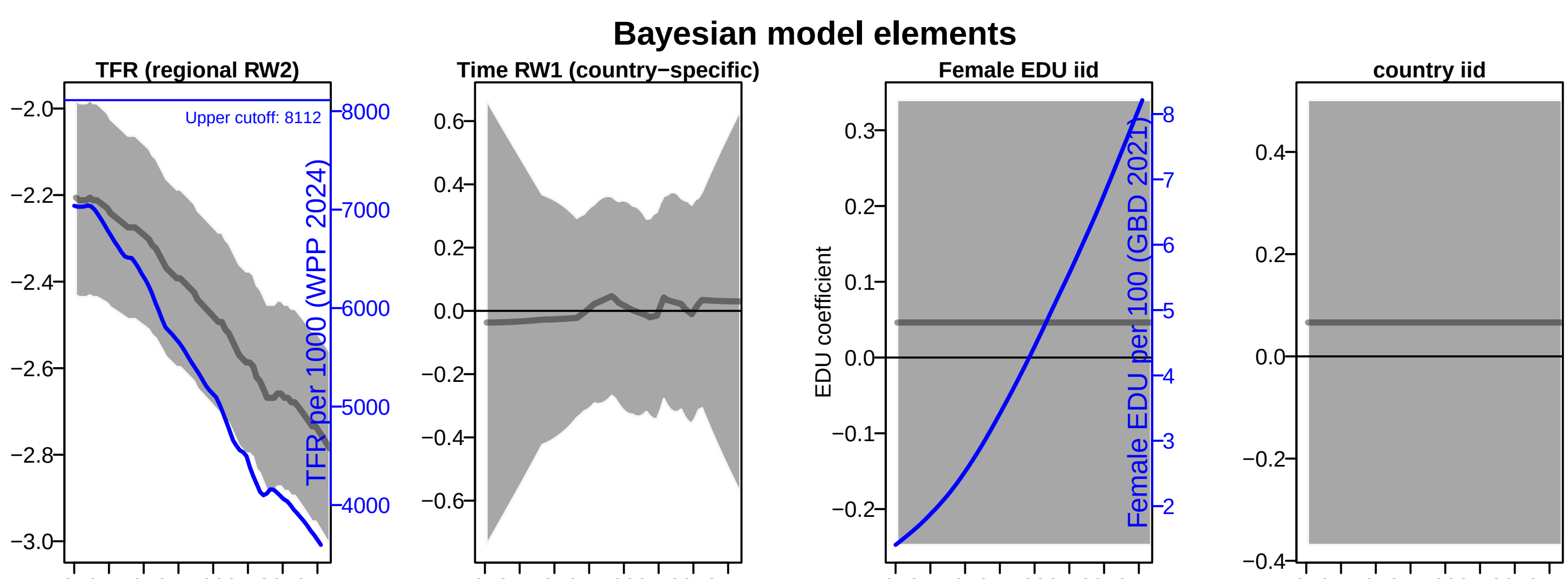
Bayesian model elements
TFR (regional RW2)
Upper cutoff: 8112
TFR per 1000 (WPP 2024)
−2.0
−2.2
−2.4
−2.6
−2.8
−3.0
8000
7000
6000
5000
4000
Time RW1 (country−specific)
0.6
0.4
0.2
0.0
−0.2
−0.4
−0.6
Female EDU iid
EDU coefficient
Female EDU per 100 (GBD 2021)
0.3
0.2
0.1
0.0
−0.1
−0.2
8
7
6
5
4
3
2
country iid
0.4
0.2
0.0
−0.2
−0.4
1950
1970
1990
2010

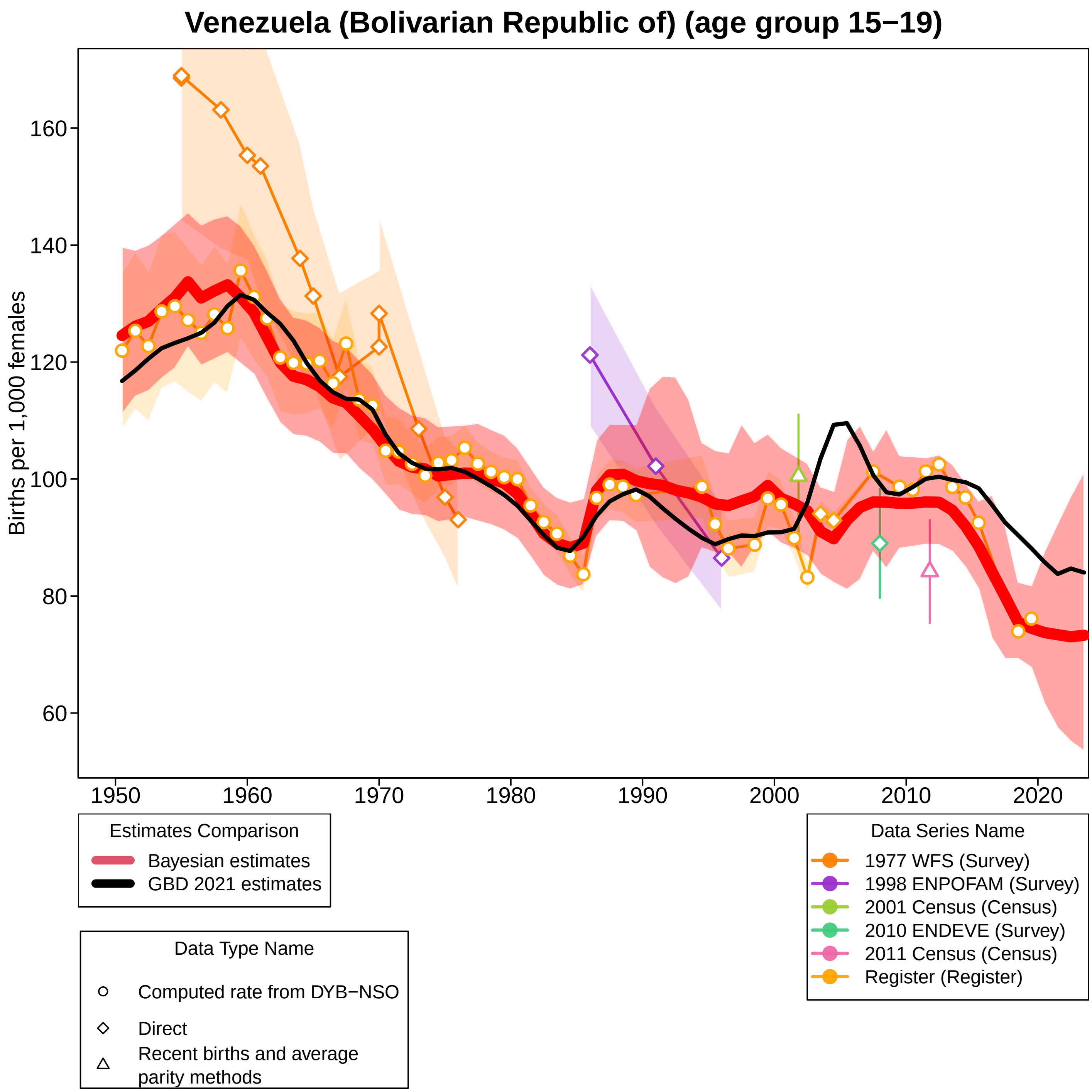

Venezuela (Bolivarian Republic of) (age group 15−19)
Births per 1,000 females
160
140
120
100
80
60
1950
1960
1970
1980
1990
2000
2010
2020
Estimates Comparison
Bayesian estimates
GBD 2021 estimates
Data Series Name
1977 WFS (Survey)
1998 ENPOFAM (Survey)
2001 Census (Census)
2010 ENDEVE (Survey)
2011 Census (Census)
Register (Register)
Data Type Name
Computed rate from DYB−NSO
Direct
Recent births and average parity methods

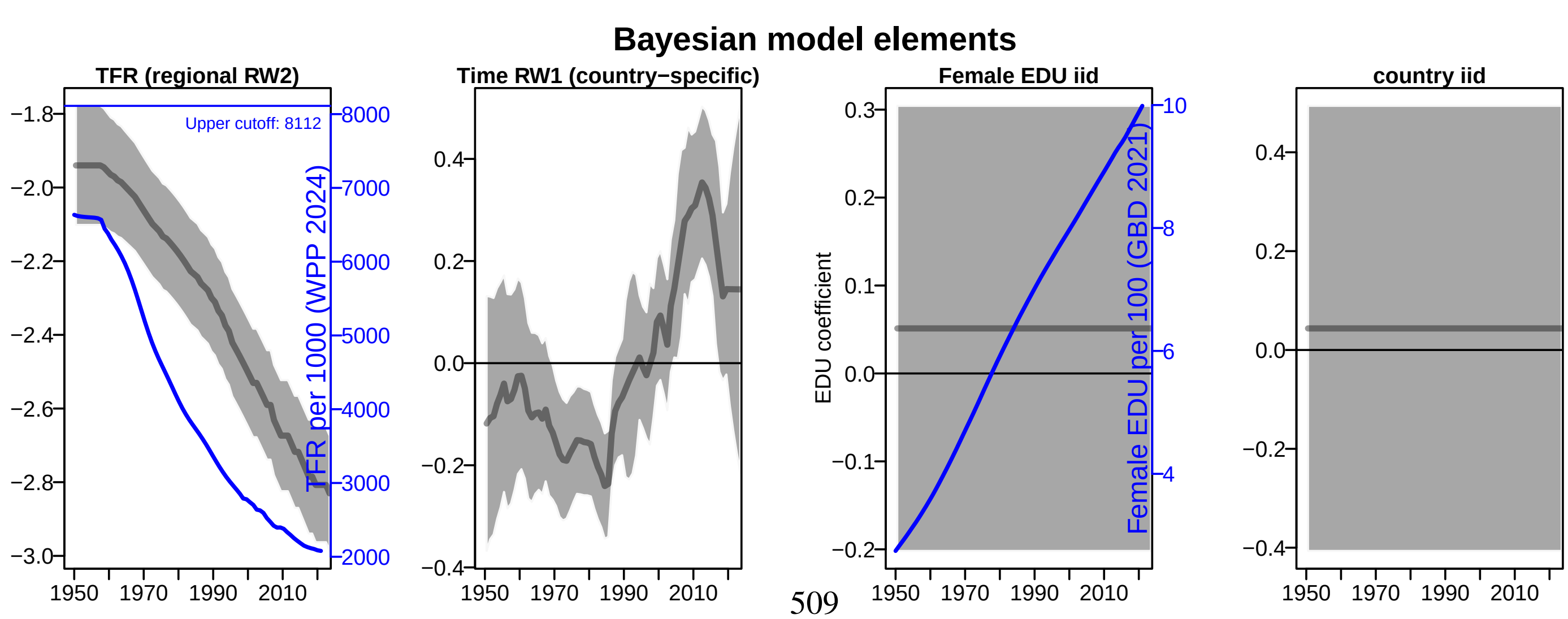

Bayesian model elements
TFR (regional RW2)
Upper cutoff: 8112
TFR per 1000 (WPP 2024)
Time RW1 (country−specific)
Female EDU iid
EDU coefficient
Female EDU per 100 (GBD 2021)
country iid

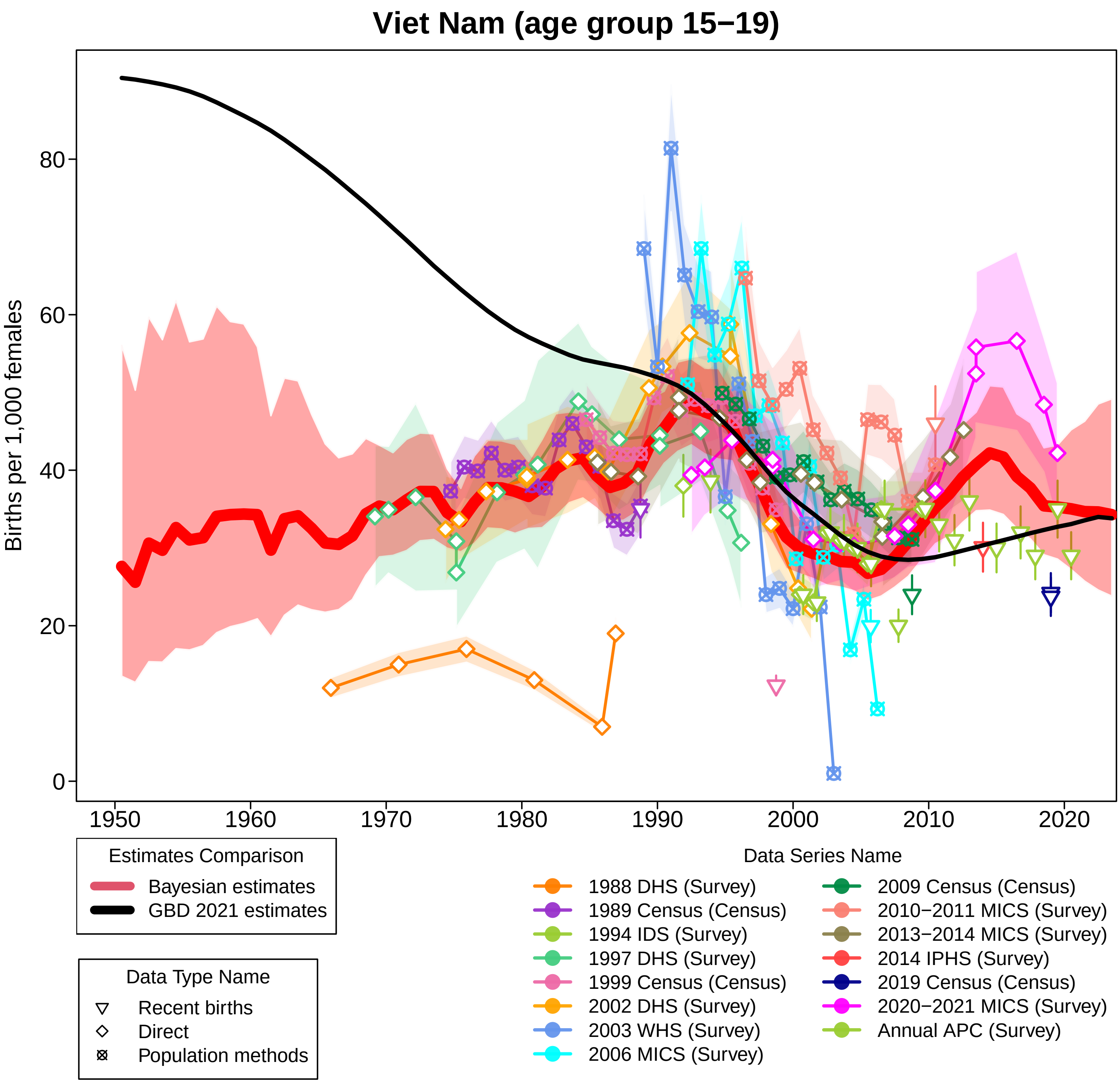
Viet Nam (age group 15−19)
Births per 1,000 females
0
20
40
60
80
1950
1960
1970
1980
1990
2000
2010
2020
Estimates Comparison
Bayesian estimates
GBD 2021 estimates
Data Type Name
Recent births
Direct
Population methods
Data Series Name
1988 DHS (Survey)
1989 Census (Census)
1994 IDS (Survey)
1997 DHS (Survey)
1999 Census (Census)
2002 DHS (Survey)
2003 WHS (Survey)
2006 MICS (Survey)
2009 Census (Census)
2010−2011 MICS (Survey)
2013−2014 MICS (Survey)
2014 IPHS (Survey)
2019 Census (Census)
2020−2021 MICS (Survey)
Annual APC (Survey)

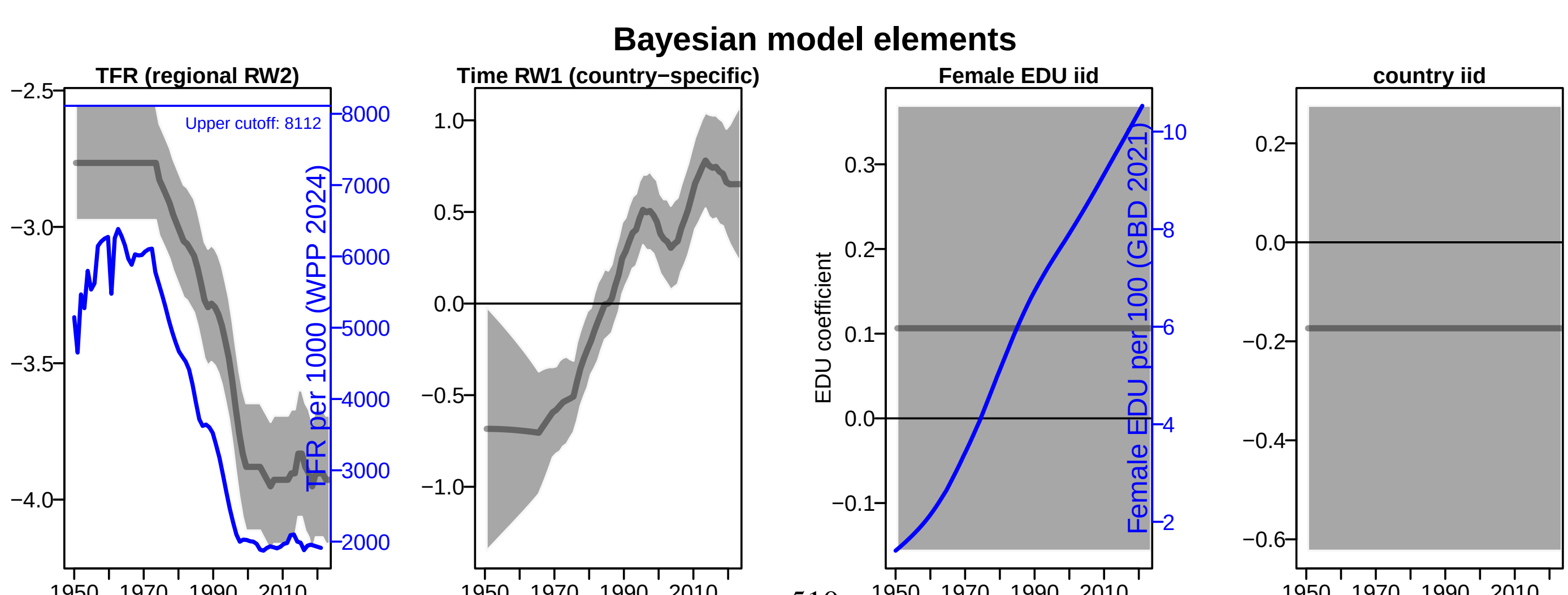
Bayesian model elements
TFR (regional RW2)
Upper cutoff: 8112
TFR per 1000 (WPP 2024)
Time RW1 (country−specific)
Female EDU iid
EDU coefficient
Female EDU per 100 (GBD 2021)
country iid

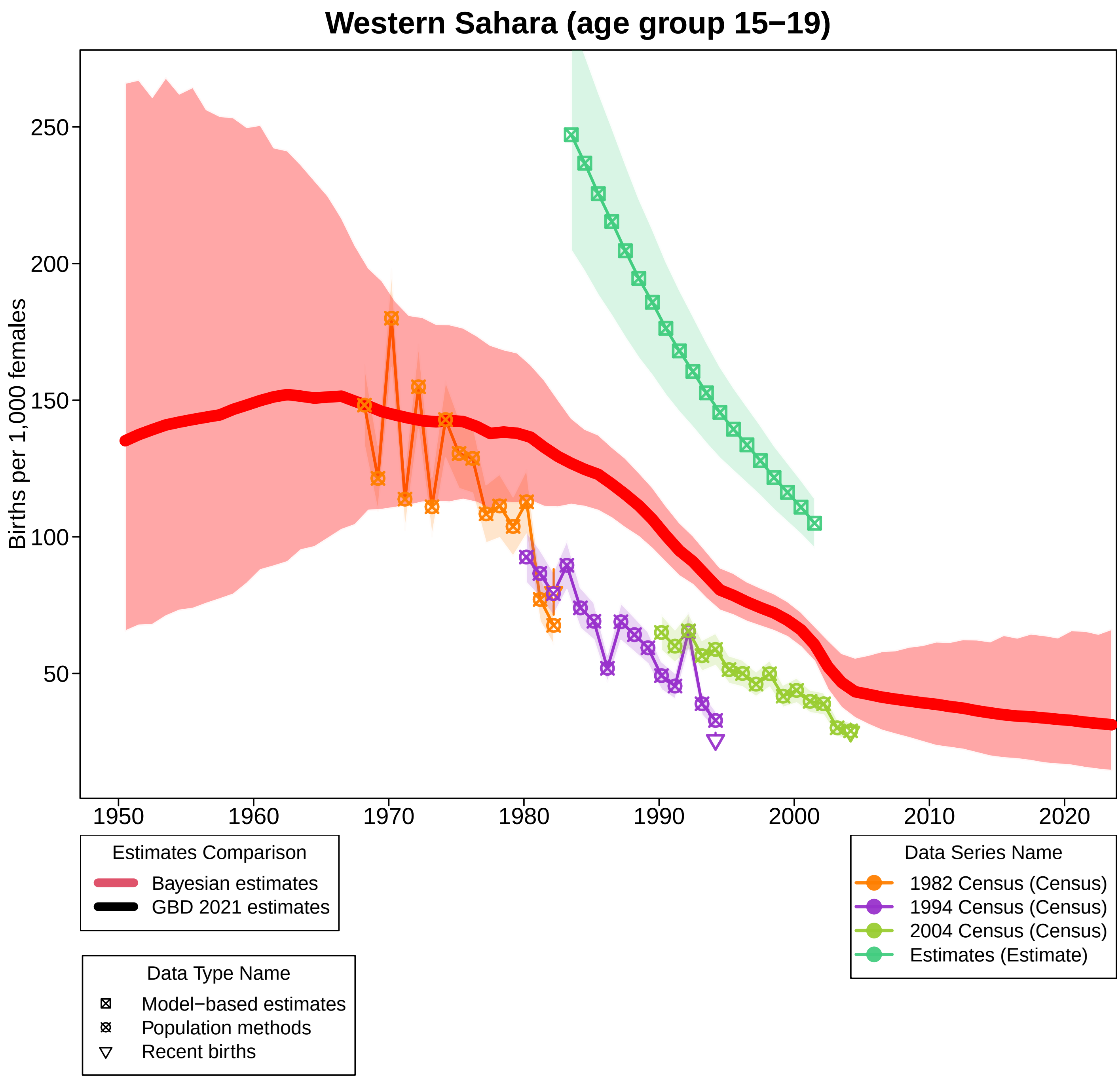
Western Sahara (age group 15−19)
Births per 1,000 females
250
200
150
100
50
1950
1960
1970
1980
1990
2000
2010
2020
Estimates Comparison
Bayesian estimates
GBD 2021 estimates
Data Series Name
1982 Census (Census)
1994 Census (Census)
2004 Census (Census)
Estimates (Estimate)
Data Type Name
Model−based estimates
Population methods
Recent births

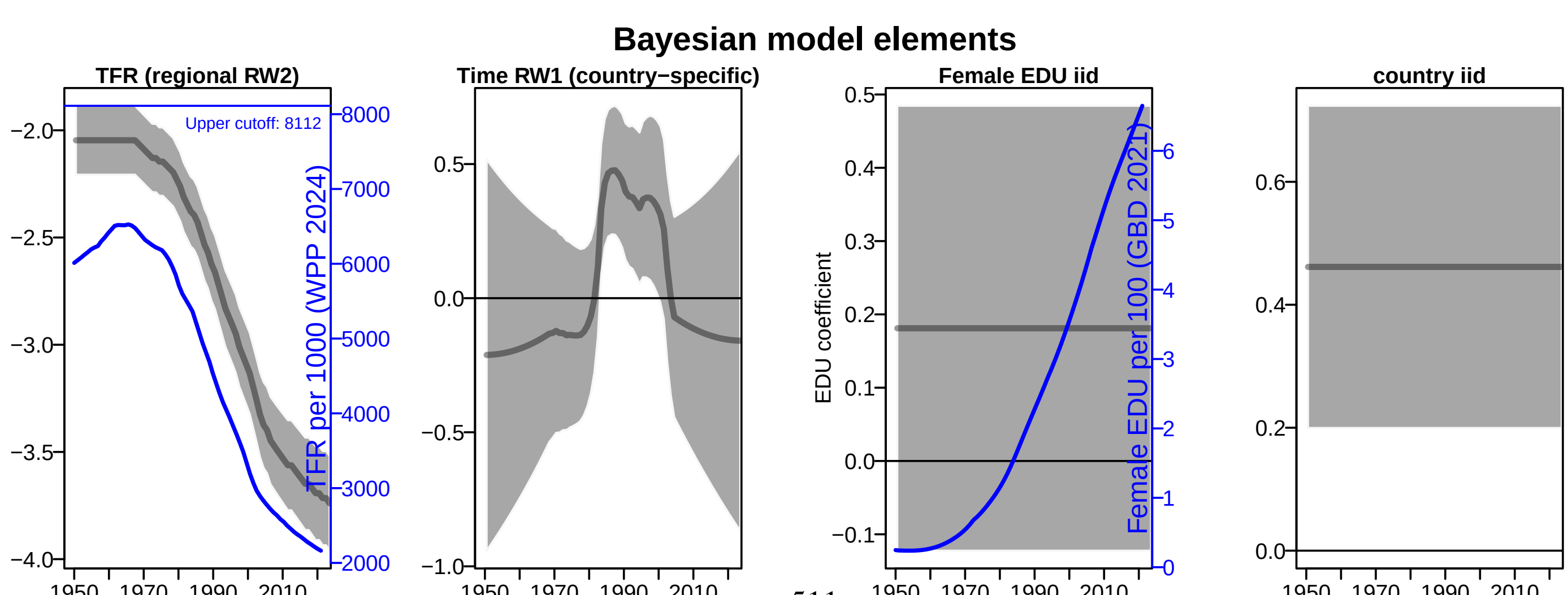
Bayesian model elements
TFR (regional RW2)
Upper cutoff: 8112
TFR per 1000 (WPP 2024)
Time RW1 (country−specific)
Female EDU iid
EDU coefficient
Female EDU per 100 (GBD 2021)
country iid

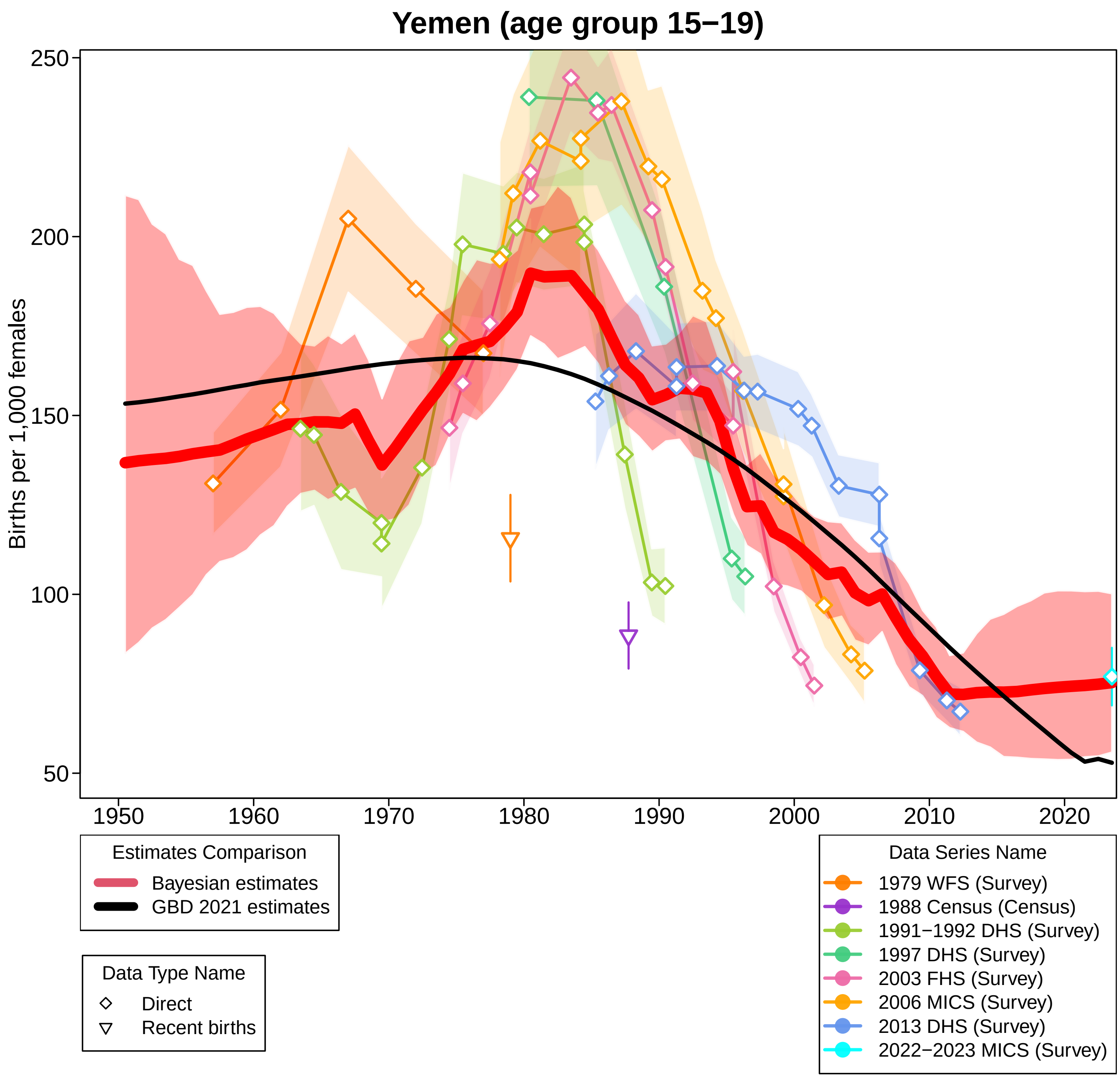
Yemen (age group 15−19)
Births per 1,000 females
250
200
150
100
50
1950
1960
1970
1980
1990
2000
2010
2020
Estimates Comparison
Bayesian estimates
GBD 2021 estimates
Data Type Name
Direct
Recent births
Data Series Name
1979 WFS (Survey)
1988 Census (Census)
1991−1992 DHS (Survey)
1997 DHS (Survey)
2003 FHS (Survey)
2006 MICS (Survey)
2013 DHS (Survey)
2022−2023 MICS (Survey)

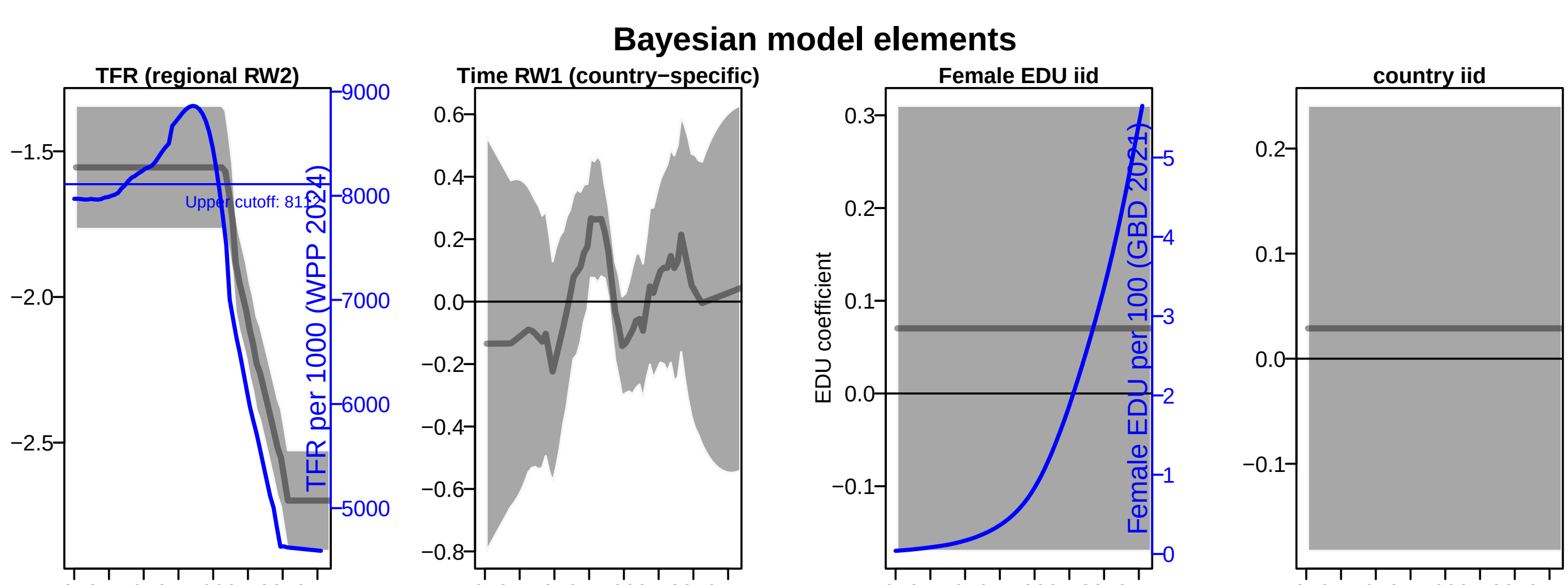
Bayesian model elements
TFR (regional RW2)
Upper cutoff: 8124
TFR per 1000 (WPP 2024)
Time RW1 (country−specific)
Female EDU iid
EDU coefficient
Female EDU per 100 (GBD 2021)
country iid
1950
1970
1990
2010

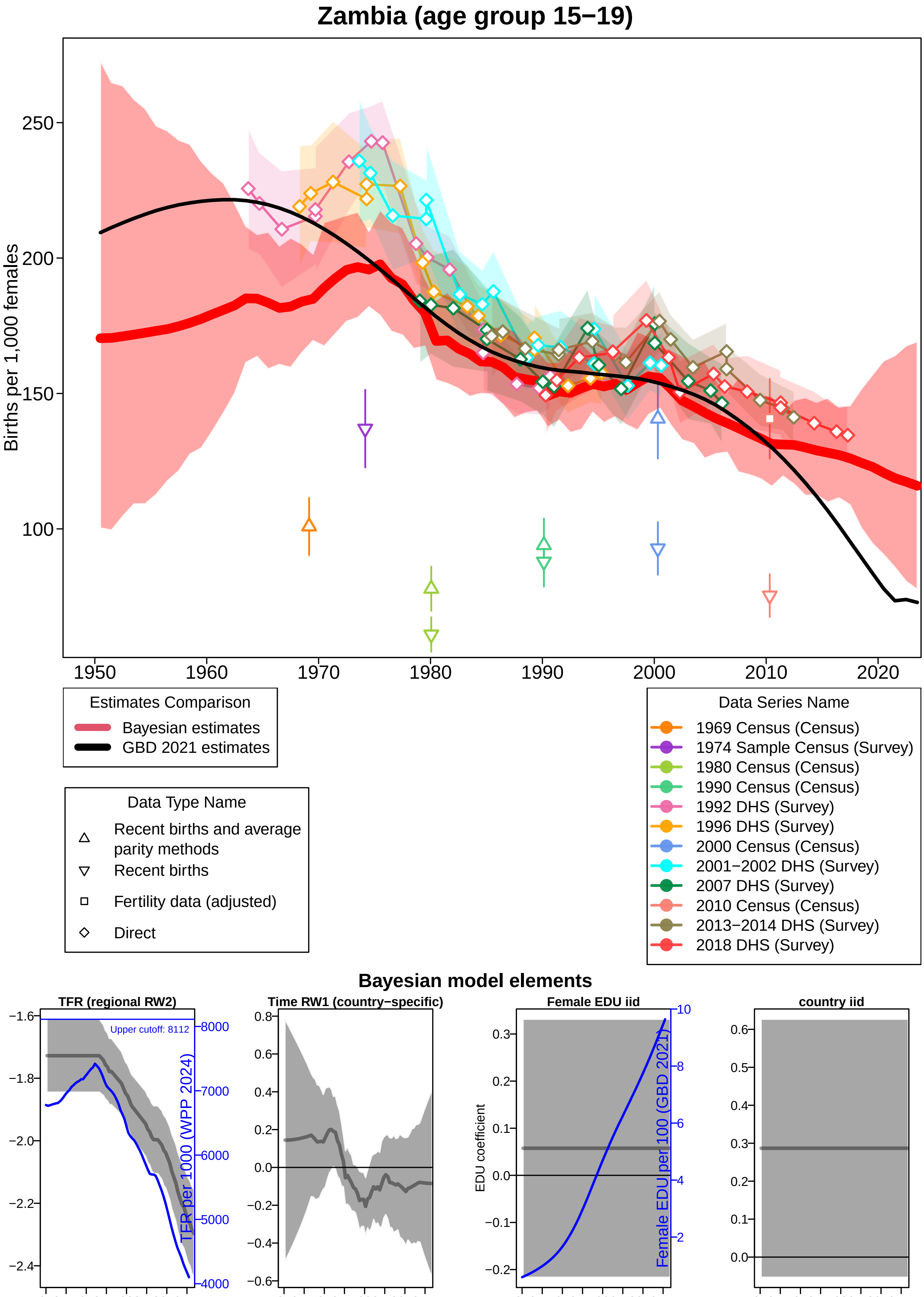
Zambia (age group 15−19)
Births per 1,000 females
250
200
150
100
1950
1960
1970
1980
1990
2000
2010
2020
Estimates Comparison
Bayesian estimates
GBD 2021 estimates
Data Type Name
Recent births and average parity methods
Recent births
Fertility data (adjusted)
Direct
Data Series Name
1969 Census (Census)
1974 Sample Census (Survey)
1980 Census (Census)
1990 Census (Census)
1992 DHS (Survey)
1996 DHS (Survey)
2000 Census (Census)
2001−2002 DHS (Survey)
2007 DHS (Survey)
2010 Census (Census)
2013−2014 DHS (Survey)
2018 DHS (Survey)
Bayesian model elements
TFR (regional RW2)
Upper cutoff: 8112
TFR per 1000 (WPP 2024)
Time RW1 (country−specific)
Female EDU iid
EDU coefficient
Female EDU per 100 (GBD 2021)
country iid

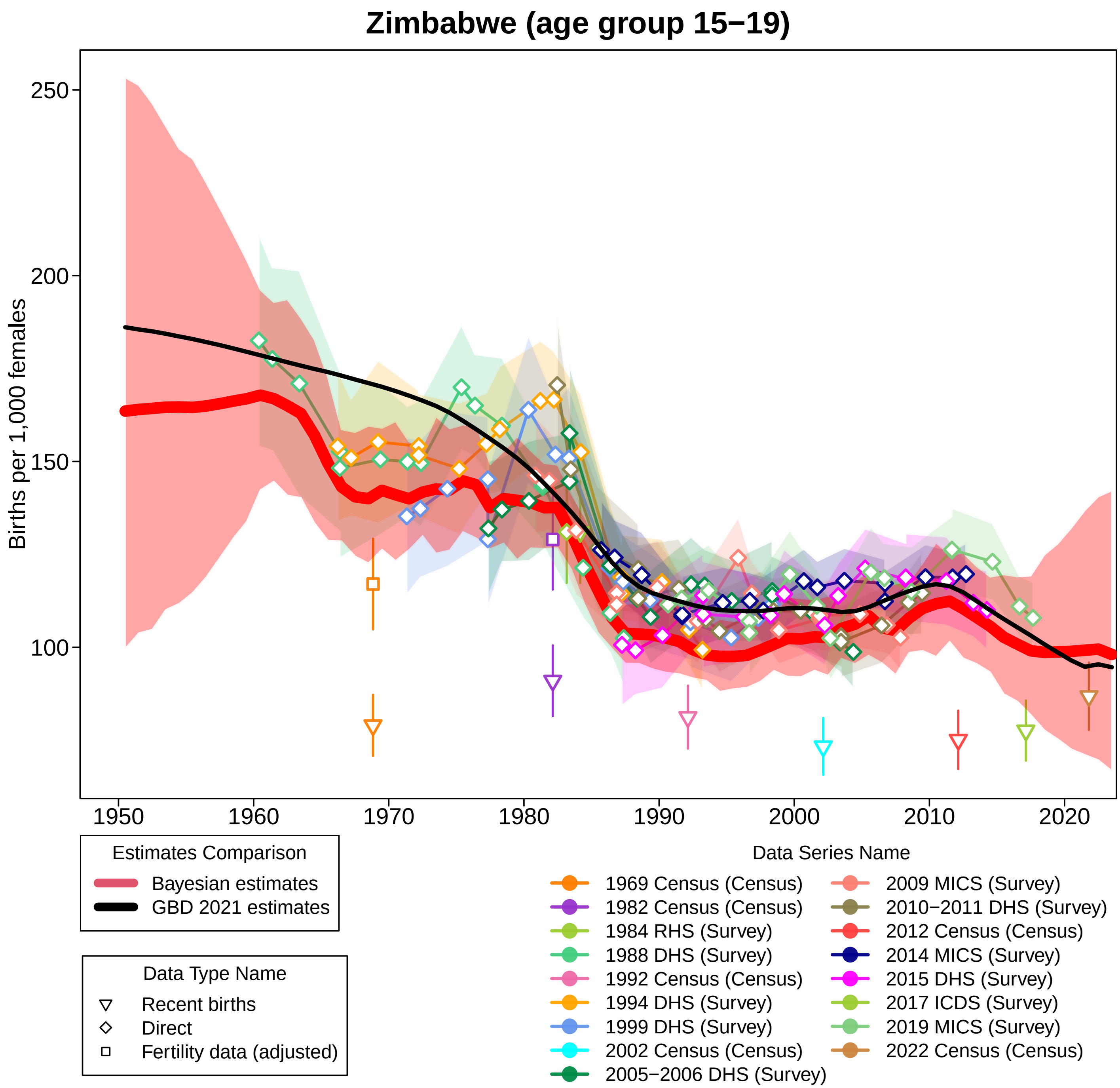
Zimbabwe (age group 15−19)
Births per 1,000 females
250
200
150
100
1950
1960
1970
1980
1990
2000
2010
2020
Estimates Comparison
Bayesian estimates
GBD 2021 estimates
Data Type Name
Recent births
Direct
Fertility data (adjusted)
Data Series Name
1969 Census (Census)
1982 Census (Census)
1984 RHS (Survey)
1988 DHS (Survey)
1992 Census (Census)
1994 DHS (Survey)
1999 DHS (Survey)
2002 Census (Census)
2005−2006 DHS (Survey)
2009 MICS (Survey)
2010−2011 DHS (Survey)
2012 Census (Census)
2014 MICS (Survey)
2015 DHS (Survey)
2017 ICDS (Survey)
2019 MICS (Survey)
2022 Census (Census)

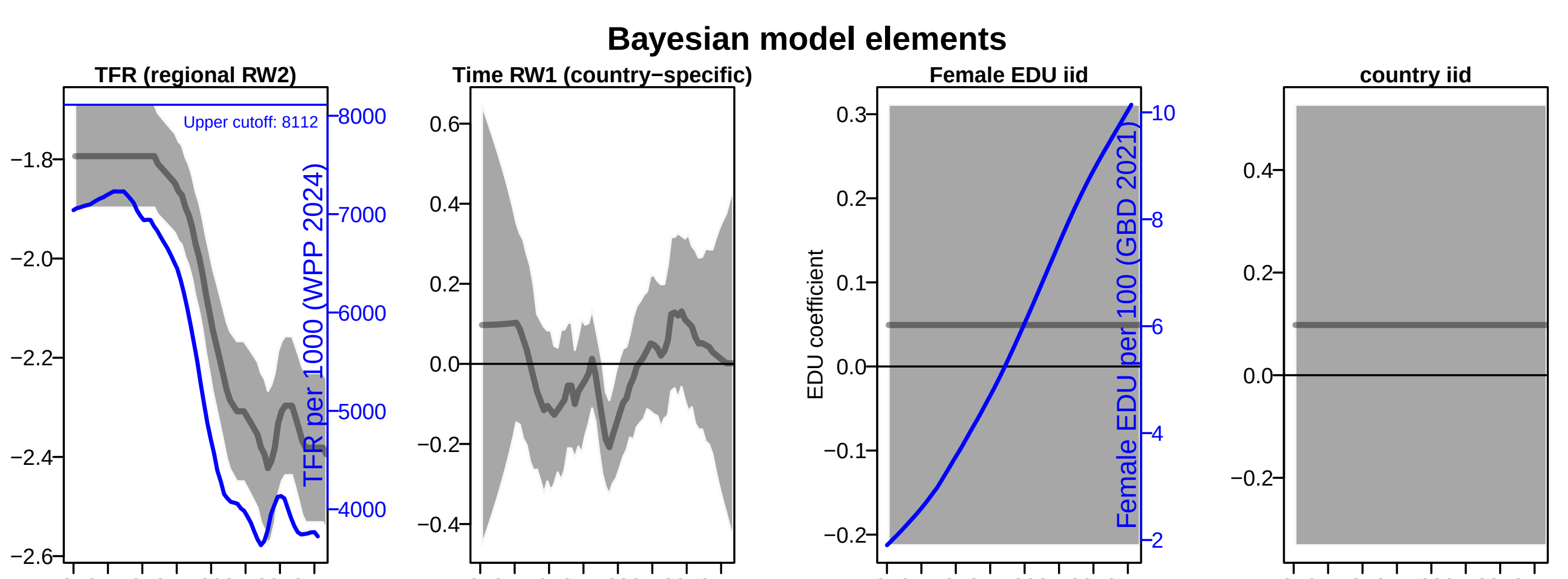
Bayesian model elements
TFR (regional RW2)
Upper cutoff: 8112
TFR per 1000 (WPP 2024)
Time RW1 (country−specific)
Female EDU iid
EDU coefficient
Female EDU per 100 (GBD 2021)
country iid

— The End —
